# Supplementary material for: Mechanochemical synthesis of organoselenium compounds
Source: Nat Commun. 2024 Jan 26;15:769. doi: 10.1038/s41467-024-44891-2 (PMC10817960; doi:10.1038/s41467-024-44891-2)
Supplement: Supplementary file 1 — Supplementary Information [file 41467_2024_44891_MOESM1_ESM.pdf]

Supplementary Information for

Mechanochemical Synthesis of Organoselenium Compounds

Shanshan Chen,[1] Chunying Fan,[1] Zijian Xu,[2] Mengyao Pei,[1] Jiemin Wang,[1] Jiye Zhang,[1] Yilei Zhang,[3] Jiyu Li,[4] Junliang Lu,[4] Cheng Peng, *,[1] Xiaofeng Wei*,[1]

[1]School of Pharmacy, Xi'an Jiaotong University, No.76, Yanta West Road, Xi'an 710061, China

[2]Shanghai Synchrotron Radiation Facility, Shanghai Advanced Research Institute, Chinese Academy of Sciences, Shanghai 201204, China

[3]Department of Biochemistry and Molecular Biology, School of Basic Medical Sciences, Xi'an Jiaotong University Health Science Center, Yanta District, Xi'an, China

[4]Xi'an Aisiyi Health Industry Co., Ltd, Xi'an 710075, China

Email: pcheng@xjtu.edu.cn, xiaofeng.wei@xjtu.edu.cn

Content

General methods and materials	2
Synthesis of substrate	5
Optimization of the reaction conditions	8
Methods' synthesis of symmetrical dichalcogenides	11
Methods' synthesis of unsymmetrical monochalcogenides	12
Methods' synthesis of unsymmetrical monochalcogenides in solution	17
Methods' synthesis of unsymmetrical monochalcogenides with gram-scale reaction	17
Mechanistic insights	18
X-Ray Absorption Fine Structure (XAFS) Analysis	20
Characterization of Obtained Products	21
Supplementary Spectra: (NMR, IR, HR-MS)	58
References	294

General methods and materials

Unless otherwise stated, reactions were carried out using dry solvents under nitrogen or argon atmosphere. Starting materials were purchased from Energy, Bide, and Leyan. Conversion was monitored by thin layer chromatography (TLC) using Merck TLC silica gel 60 F254 and visualized by UV-light at 254 nm. Magnesium turnings (99.5%, product no. 13706041) and Mg powder (99%, product no. 13500062) were purchased from Inopharm Chemical Reagent Shaanxi Co. LTD. Se powder (99.99%) were purchased from Adamas in China. All reagents purchased are used directly without treatment. All reactions were performed using grinding vessels in the Retsch MM 400 (Supplementary Fig. 1) and Retsch MM 500 (Supplementary Fig. 1). Both jars and balls were made of stainless steel (Supplementary Fig. 2). Temperature-controllable heat gun used in this study (Supplementary Fig. 3). NMR spectra were recorded on JEOL JNM-ECZ400S spectrometers (^{1}H: 400 MHz or 600 MHz, ^{13}C: 101 MHz or 150 MHz, ^{77}Se: 76 Hz, ^{19}F: 376 Hz). Tetramethylsilane (^{1}H) and (^{13}C) were employed as external standards, respectively. Multiplicity was recorded as follows: s = singlet, brs = broad singlet, d = doublet, t = triplet, q = quartet, quint = quintet, sex = sextet, sept = septet, and m = multiplet. Infrared spectra were recorded on a Bruker VERTEX70 spectrometer. Absorptions are reported in wavenumber (cm^{-1}). High-resolution electrospray ionization and electronic impact mass spectrometry were performed on a WATERS I-Class VION IMS Qt of double focusing magnetic sector mass spectrometer. *N, N*-dimethylformamide and Mesitylene was used as an internal standard to determine the NMR yields. The above tests were conducted in the the Instrument Analysis Center of XJTU.

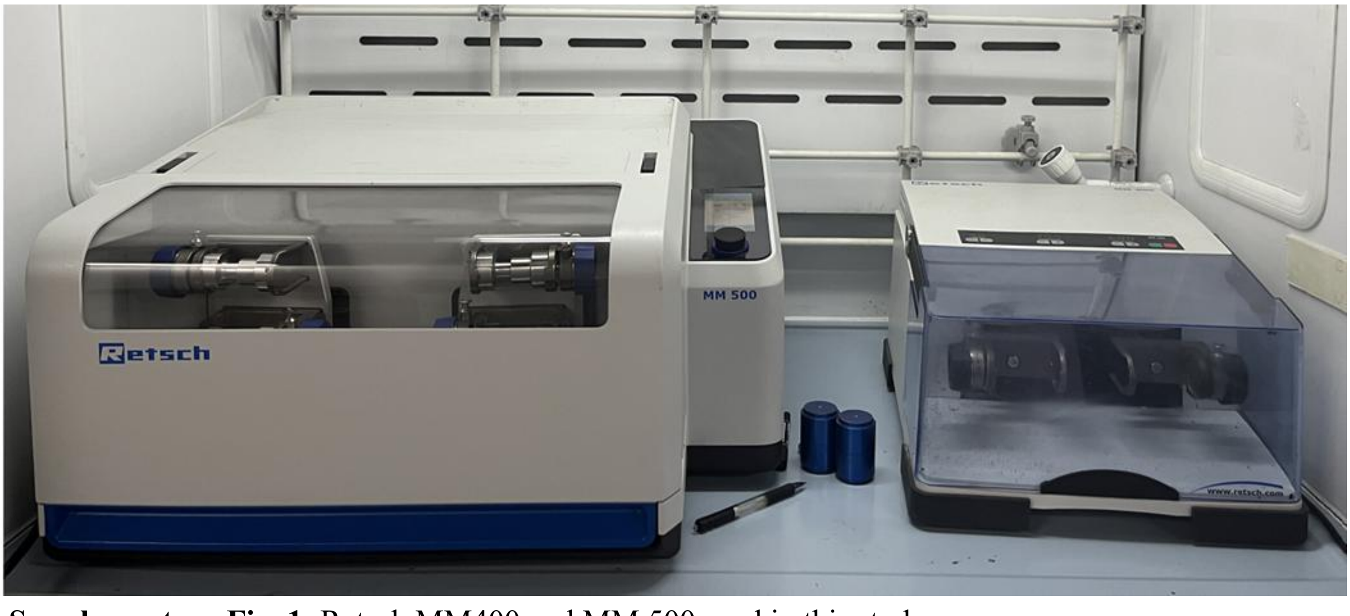

Supplementary Fig. 1. Retsch MM400 and MM 500 used in this study.

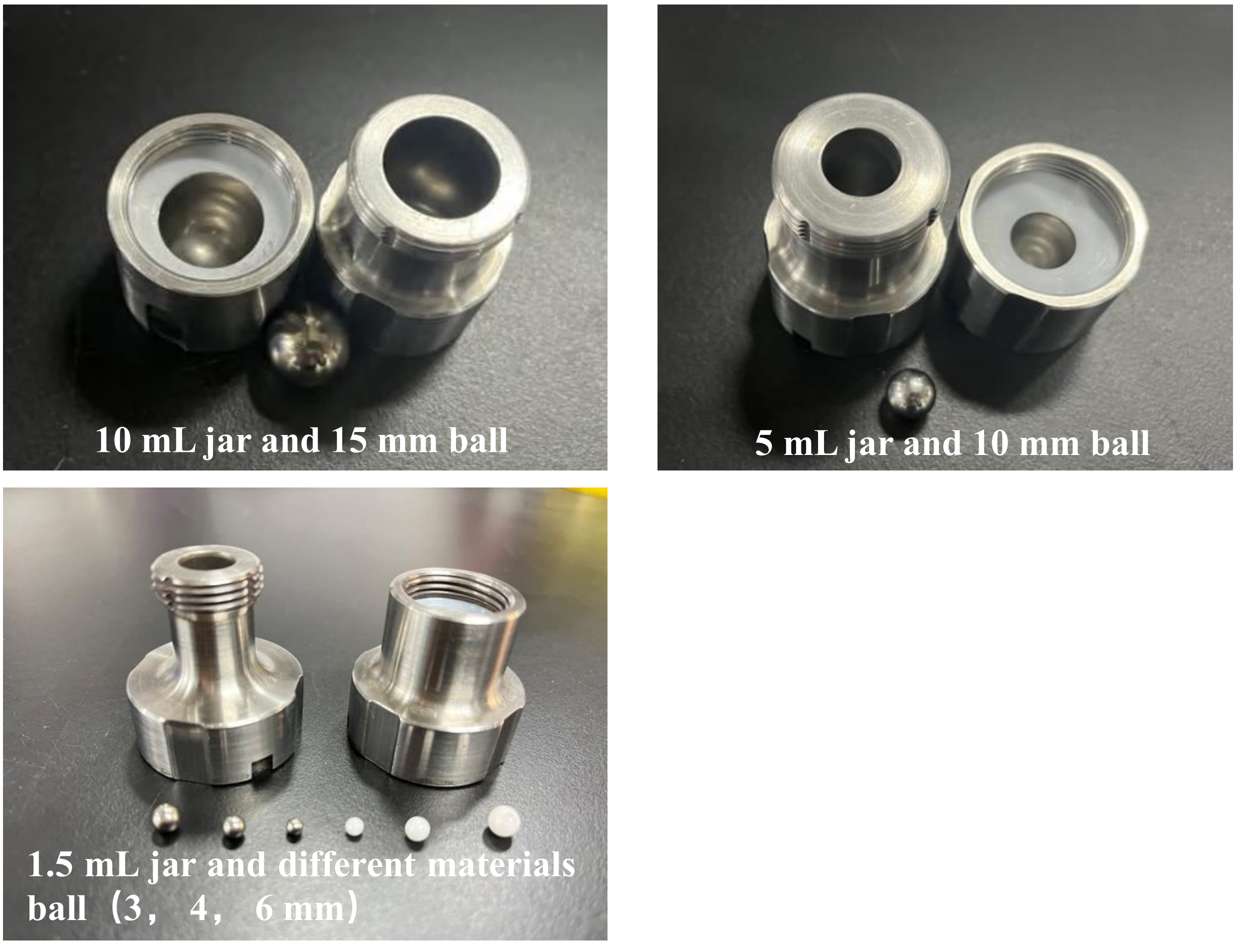

Supplementary Fig. 2. Stainless jars and balls used in this study

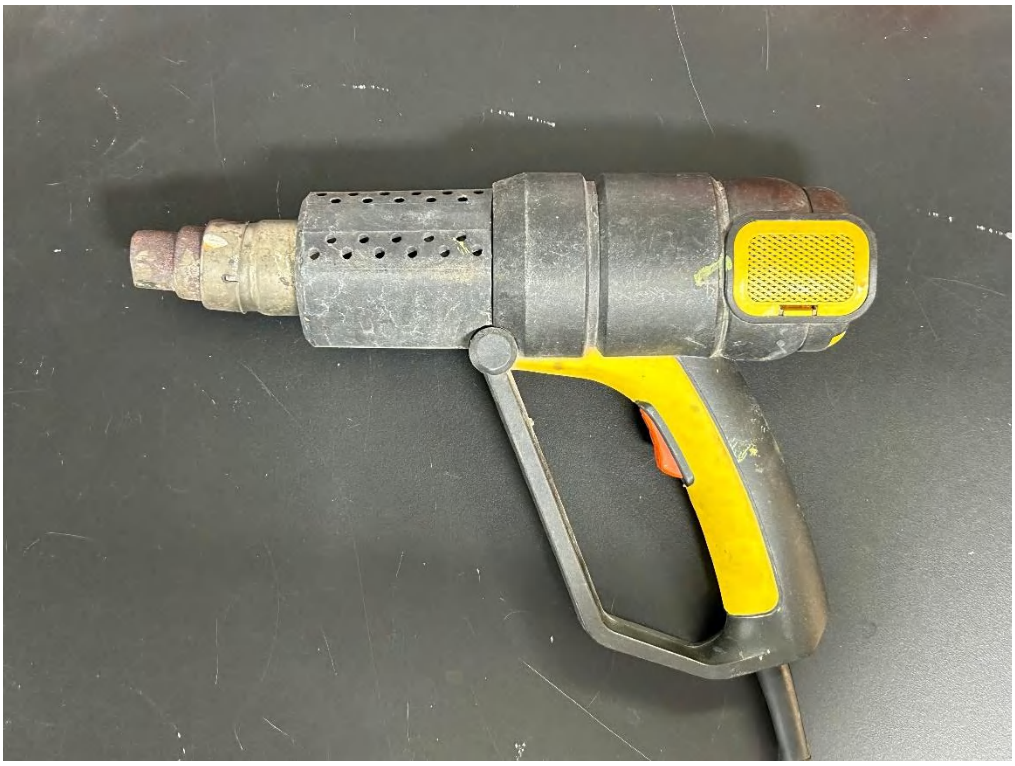

Supplementary Fig. 3. Temperature-controllable heat gun used in this study

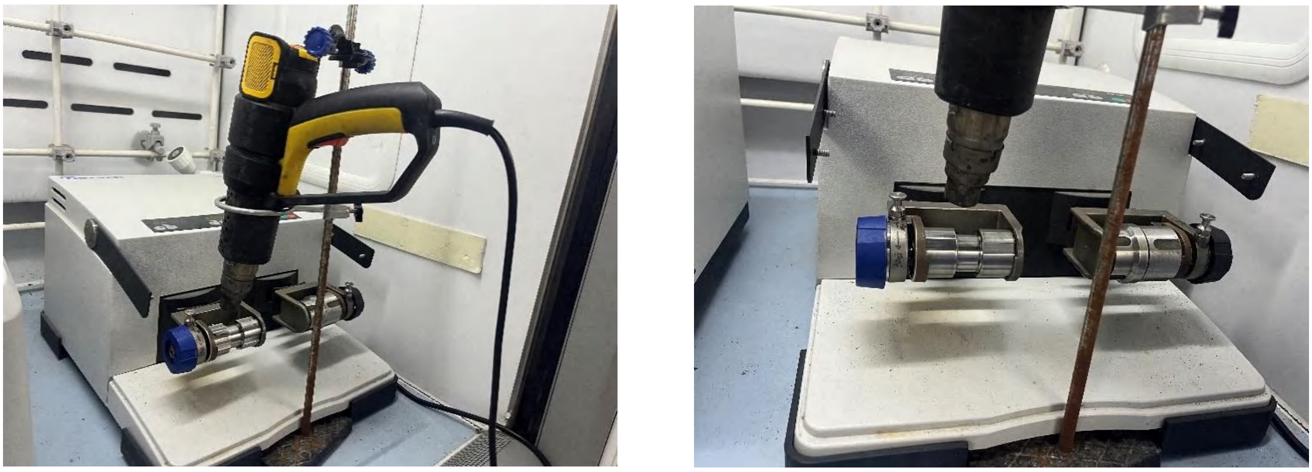

Supplementary Fig. 4. Reaction set up with a heat gun

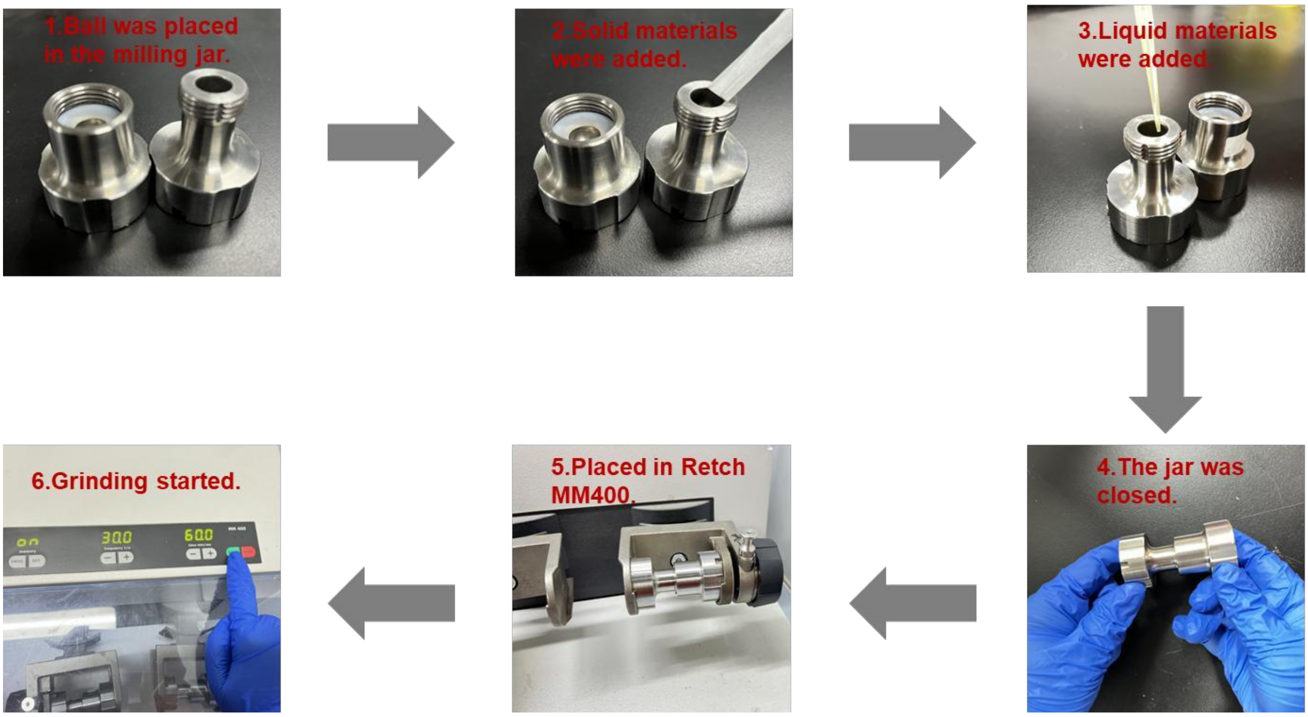

Supplementary Fig. 5. Procedure of setting up the mechanochemical reactions.

Synthesis of substrate

Methods' preparation of *N*-phenylacrylamide derivatives[1]

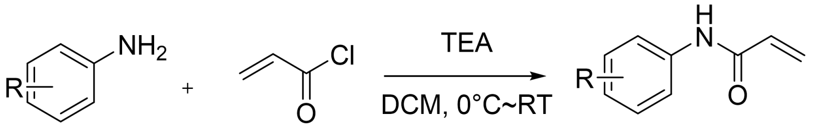

To a solution of dry DCM (15 mL), aniline (5 mol, 1.0 equiv, 535.1 mg-966.2 mg) and TEA (10 mol, 2.0 equiv, 1.39 mL) were added in a round-bottomed flask equipped with a magnetic stirring bar. The reaction mixture was cooled to 0°C and stirred for 5 minutes under an argon atmosphere. Acryloyl chloride (6 mmol, 1.2 equiv, 0.49 mL) was added dropwise and the resulting mixture was stirred for 15 minutes and warmed to room temperature. The reaction was monitored by TLC analysis and once the reaction was completed, the suspension was brought to 0°C and diluted with H_2O. The mixture was extracted with DCM (10 mL×3). The combined organic layers were washed with brine, dried over Na_2SO_4 and concentrated under vacuum. The crude product was purified by rapid column chromatography (SiO_2, petroleum ether/ethyl acetate, 5:1-3:1).

Methods' preparation of (iodomethyl)benzene[2]

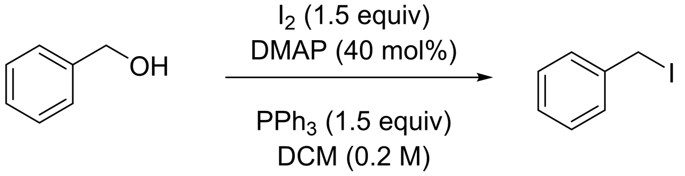

Add iodine (30 mmol, 1.5 equiv, 7.614 g) and polymer supported 4-DMAP (8 mmol, 0.4 equiv, 977.4 mg) to a stirred solution of triphenylphosphine (30 mmol, 1.5 equiv, 7.869 g) in dry DCM. Continue the stirring for 2 minutes. Add benzyl alcohol (20 mmol, 1.0 equiv, 2.07 mL) to the reaction mixture. Allow the mixture to react for 60 min. After the reaction was judged to be complete (via TLC), the reaction was quenched by the addition of H_2O; the aqueous layer was extracted twice with CH_2Cl_2 and the combined organic layers were washed with an aqueous solution of sodium thiosulphate, dried over Na_2SO_4, filtered and concentrated under reduced pressure. The crude product was purified by flash column chromatography (SiO_2, Hexane)

Methods' preparation of (2-iodoethyl)benzene[2]

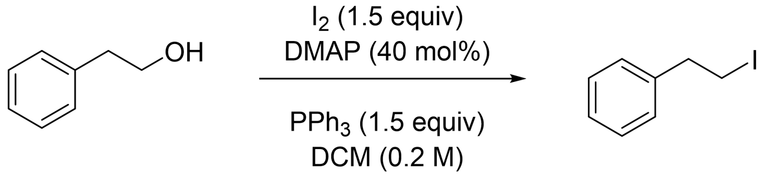

Add iodine (30 mmol, 1.5 equiv, 7.614 g) and polymer supported 4-DMAP (8 mmol, 0.4 equiv, 977.4 mg) to a stirred solution of triphenylphosphine (30 mmol, 1.5 equiv, 7.869 g) in dry DCM.

Continue the stirring for 2 minutes. Add benzyl alcohol (20 mmol, 1.0 equiv, 2.4 mL) to the reaction mixture. Allow the mixture to react for 60 min. After the reaction was judged to be complete (via TLC), the reaction was quenched by the addition of H_2O; the aqueous layer was extracted twice with CH_2Cl_2 and the combined organic layers were washed with an aqueous solution of sodium thiosulphate, dried over Na_2SO_4, filtered and concentrated under reduced pressure. The crude product was purified by flash column chromatography (SiO_2, Hexane)

Methods' preparation of 1-(tert-butyl)-2-iodobenzene[3]

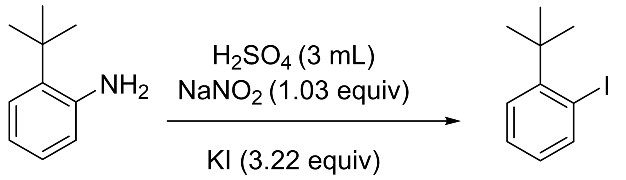

The compound 2-*tert*-butylaniline (2.5 mL, 2.4 g, 16 mmol) was added to a solution of concentrated H_2SO_4 (3 mL) diluted with water (12 mL), forming a pale pink suspension, and cooled to – 40°C. Sodium nitrite (1.15 g, 16.5 mmol) dissolved in water (10 mL) was added and the solution stirred for 10 min. The temperature was adjusted to – 15°C and a pre-cooled solution of KI (8.55 g, 51.5 mmol) in water (20 mL) was added at which point the solution turned red. The solution was stirred for a further hour whilst warming to 0°C. The solution was cautiously neutralised with dilute aqueous NaOH solution (1 M, 50 mL) before being extracted with hexanes (3×30 mL). The hexanes layers were combined and dried over Na_2SO_4 and the solvent removed in vacuo. The crude product was purified by flash column chromatography (SiO_2, Hexane)

Methods' preparation of 2-iodo-1-methyl-1*H*-indole[4]

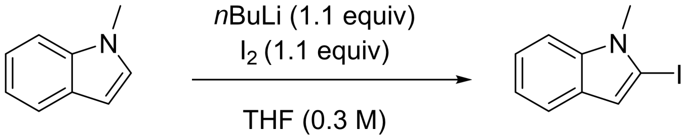

In a 50 mL flame dried Schlenk flask 1-methyl-1*H*-indole (5.54 mmol, 1.0 equiv, 618 mg,) was dissolved in 20 mL dry THF and cooled to 0°C. 1.4 mL *n*BuLi (6.10 mmol, 1.1 equiv, 2.4 mL, 2.5 M in hexane) were added dropwise. The reaction mixture was allowed to warm up to room temperature and stirred overnight. I_2 (6.10 mmol, 1.1 equiv, 1.79 g in 45 mL THF) were added at 0°C and the mixture was stirred for two more hours. After completion, the reaction was quenched with a saturated NH_4Cl solution (20 mL). The mixture was extracted with ethyl acetate (40 mL). The organic phase was washed with $Na_2S_2O_3$ (20 mL), brine (20 mL) and dried over Na_2SO_4. The solvent was removed under reduced pressure and the crude product was purified by flash column chromatography (SiO_2, Hexane)

Methods' preparation of 2-iodobenzofuran[5]

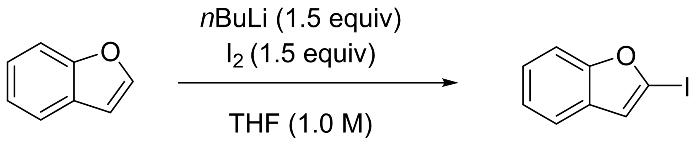

In a 50 mL flame dried Schlenk flask benzofuran (5.0 mmol, 1.0 equiv, 550 µL) was dissolved in 5 mL dry THF and cooled to 0°C. 3.0 mL *n*BuLi (7.5 mmol, 1.5 equiv, 3.0 mL, 2.5 M in hexane) were added dropwise. Keep the reaction mixture at 0°C for 60 min. The reaction mixture was allowed to warm up to room temperature. I_2 (7.5 mmol, 1.5 equiv, 1.90 g in 5 mL THF) were added at 0°C and the mixture was stirred for two more hours. After completion, wash the reaction mixture twice with saturated aqueous $Na_2S_2O_3$ solution. The mixture was extracted with ethyl acetate. The organic phase was washed with brine and dried over Na_2SO_4. The solvent was removed under reduced pressure and the crude product was purified by flash column chromatography (SiO_2, Hexane)

Methods' preparation of 2, 3-dihydro-1*H*-inden-2-ol[6]

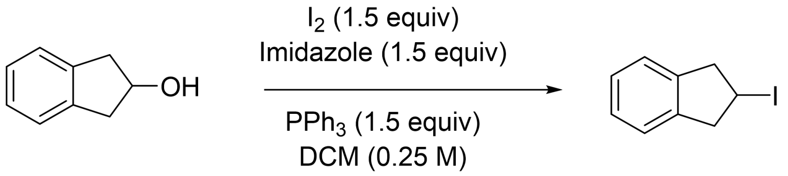

Under inert atmosphere (argon), PPh_3 (15 mmol, 1.5 equiv, 3.93 g) and imidazole (15 mmol, 1.5 equiv, 1.02 g) were dissolved in dry DCM (40 mL) and the mixture was cooled at 0°C by means of an ice bath. Next, I_2 (15 mmol, 1.5 equiv, 3.80 g) was added and the resulting suspension was stirred for 30 min. 2-Hydroxyindan (9.9 mmol, 1.0 equiv, 1.30 g) was added dropwise to the reaction mixture and the reaction crude was left stirring for 60 min. After the reaction was judged to be complete (via TLC), the reaction was quenched by the addition of H_2O; the aqueous layer was extracted twice with CH_2Cl_2 and the combined organic layers were washed with an aqueous solution of sodium thiosulphate, dried over Na_2SO_4, filtered and concentrated under reduced pressure. The crude product was purified by flash column chromatography (SiO_2, Hexane)

Methods' preparation of (3-iodopropoxy)benzene[6]

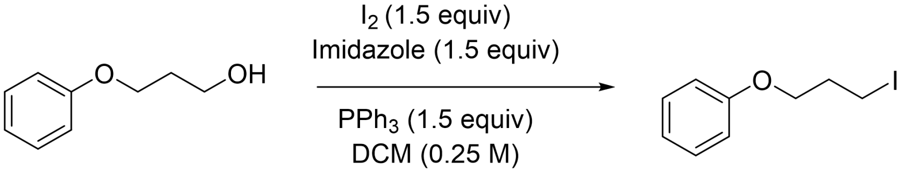

Under inert atmosphere (argon), PPh_3 (7.5 mmol, 1.5 equiv, 1.97 g) and imidazole (7.5 mmol, 1.5 equiv, 0.50 g) were dissolved in dry DCM (20 mL) and the mixture was cooled at 0°C by means of an ice bath. Next, I_2 (7.5 mmol, 1.5 equiv, 1.90 g) was added and the resulting suspension was stirred for 30 min. 3-Phenoxy-1-propanol (5.0 mmol, 1.0 equiv, 710 µL) was added dropwise to the reaction mixture and the reaction crude was left stirring for 60 min. After the reaction was judged to be complete

(via TLC), the reaction was quenched by the addition of H_2O; the aqueous layer was extracted twice with CH_2Cl_2 and the combined organic layers were washed with an aqueous solution of sodium thiosulphate, dried over Na_2SO_4, filtered and concentrated under reduced pressure. The crude product was purified by flash column chromatography (Hexanes)

Methods' preparation of 1-(allyloxy)-2-bromobenzene[7]

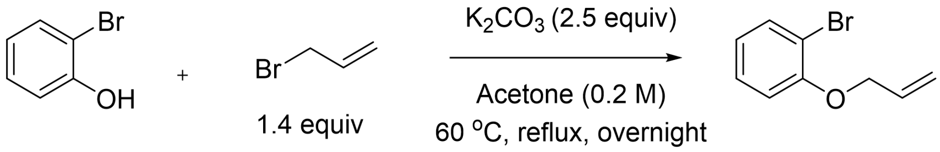

In a 100 mL flame dried Schlenk flask 2-Bromophenol (10.0 mmol, 1.0 equiv, 1.16 mL) was dissolved in 54 mL Acetone and rised to 60°C. Allyl bromide (14 mmol, 1.4 equiv, 1.2 mL) and potassium carbonate (25 mmol, 2.5 equiv, 3.455 g) were added. The mixture was refluxed overnight. The reaction was monitored by TLC analysis and once the reaction was completed, the reaction mixture was allowed to warm up to room temperature and Acetone was removed under reduced pressure. The mixture was extracted with ethyl acetate. The organic phase was washed with brine and dried over Na_2SO_4. The solvent was removed under reduced pressure and the crude product was purified by flash column chromatography (Hexanes)

Optimization of the reaction conditions

Supplementary Table 1. Optimization of the reaction conditions using aryl halides

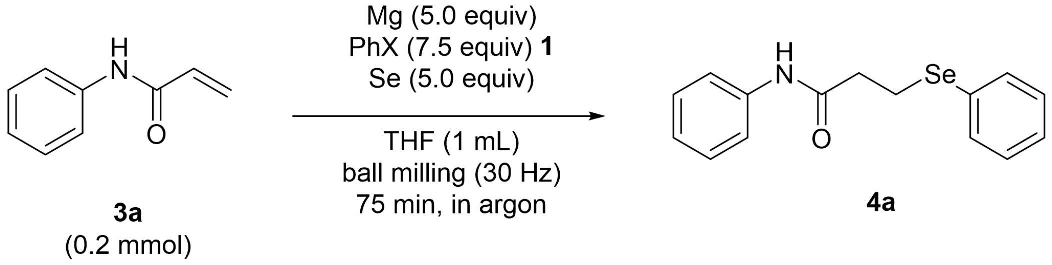

Entry	PhX	Yield(%)^b
1	PhBr	80
2	PhI	89

^{a}Reactions performed using Retsch MM 400, stainless-steel milling jar (10 mL) and a stainless-steel ball (diameter: 10 mm). Conditions: **3a** (0.20 mmol), Mg (1.0 mmol), Se (1.0 mmol), **1** (1.5 mol), THF (1 mL); ^{b}isolated yield

Supplementary Table 2. Optimization of the reaction conditions

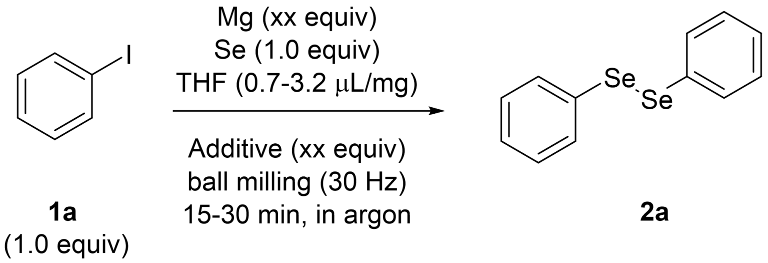

Entry	Mg (eq.)	Additives (eq.)	Conditions (air or argon)	THF (μL/mg)	Time (min)	Yield (%)[a]
1	0.5	NaCl / 0.5	air	3.1	30	36
2	0.5	NaCl / 1.0	air	2.8	30	38
3	1.0	—	air	3.2	15	51
4	1.0	—	air	3.2	60	47
5	1.0	—	argon	3.2	60	62
6	1.5	—	argon	3.1	60	75
7	1.5	—	argon	3.1	15	65
8	1.5	NaCl / 1.0	argon	2.6	60	80
9	1.5	NaCl / 1.5	argon	2.4	15	59
10	1.5	LiCl / 1.5	argon	2.6	15	95
11	1.0	LiCl / 1.5	argon	2.7	15	90
12	0.5	LiCl / 1.0	argon	3.0	15	47
13	1.0	LiBr / 1.5	argon	2.2	15	81
14	1.0	$LiBF_4$ / 1.5	argon	2.1	15	15
**15[b]**	**1.0**	**LiCl / 1.5**	**argon**	**1.4**	**15**	**89**
16[c]	1.0	LiCl / 1.5	argon	0.7	15	37
17[d]	1.0	LiCl / 1.5	argon	2.7	15	90
18[e]	1.0	LiCl / 1.5	argon	2.7	15	83
19[f]	1.0	LiCl / 1.5	argon	2.7	15	90
20[g]	1.0	LiCl / 1.5	argon	2.7	15	88
21[h]	1.0	LiCl / 1.5	argon	2.7	15	20
22[i]	1.0	LiCl / 1.5	argon	2.7	15	44
23[j]	1.0	LiCl / 1.5	argon	2.7	15	61

[a]Reactions performed using Retsch MM 400, stainless-steel milling jar (1.5 mL) and a stainless-steel ball (diameter: 6 mm). Conditions: **1a** (0.20 mmol), Mg (0.2 mmol), Se (0.2 mmol), THF (2.1-3.2 μL/mg); η = v (liquid; μL)/m (reagents; mg). Yield were determined by ^{1}H NMR analysis of the crude mixture using DMF as an internal standard; [b]Conditions: THF (1.4 μL/mg); [c]Conditions: THF (0.7 μL/mg); [d]A zirconia ball (diameter: 6 mm); [e]Two zirconia balls (diameter: 4 mm); [f] Four zirconia balls (diameter: 3 mm); [g]Four stainless-steel balls; [h]Solution-based reaction conditions: THF (200μL) in a vial (5 mL), rt, 15 min; [i]Solution-based reaction conditions: THF (200μL) in a Schlenk (10 mL), 40°C, 15 min;[j] Solution-based reaction conditions: THF (200μL) in a vial (5 mL), rt, 12h

Supplementary Table 3. Optimization of the reaction conditions

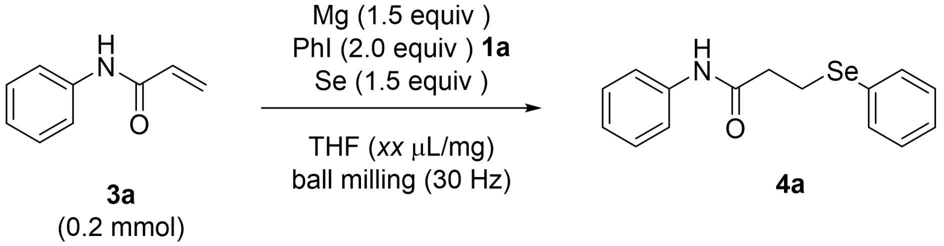

Entry	Ball (mm)	Ball number	Additives (eq.)	THF (µL/mg)	Time (min)	Yield (%)
1	6.0	1	—	0.4	120	19
2	6.0	1	—	0.7	120	54
3	6.0	1	—	0.7	120	49
4	6.0	1	NaCl/ 3.0 eq.	0.6	120	49
5	6.0	1	NaCl/ 3.0 eq.	0.6	120	37
6	6.0	1	NaCl/ 1.5 eq.	0.3	120	14
7	6.0	1	—	1.4	120	92
**8**	**6.0**	**1**	**—**	**1.4**	**90**	**93(94)[b]**
9	6.0	1	—	1.4	75	62
10	6.0	1	—	1.4	60	69
11	6.0	1	—	1.4	45	60
12	3.0	6	—	1.4	120	76
13	3.0	4	—	1.4	120	85
14	3.0	2	—	1.4	120	60
15	3.0	6	—	0.7	120	34
16	3.0	4	—	0.7	120	22
17	3.0	2	—	0.7	120	33
18[c]	6.0	1	—	0.7	90	57
19[c]	6.0	1	Mg/ 0.5 eq.	0.7	90	71
20[c]	6.0	1	Mg/ 1.0 eq.	0.7	90	76
21[c]	6.0	1	Mg/ 0.5 eq.	0.7	120	69
22[d]	3.0	4	—	1.4	90	93
23[d]	4.0	2	—	1.4	90	78
24[d]	6.0	1	—	1.4	90	87
25[e]	6.0	1	—	1.4	90	14
26[f]	6.0	1	—	1.4	90	20

[a]Reactions performed using Retsch MM 400, stainless-steel milling jar (1.5 mL) and a stainless-steel ball (diameter: 6 mm). Conditions: **3a** (0.2 mmol), Mg (0.3 mmol), Se (0.3 mmol), **1a** (0.4 mmol), THF (1.4 µL/mg); η = v (liquid; µL)/m (reagents; mg). Yield was determined by ^{1}H NMR analysis of the crude mixture using DMF as an internal standard; [b]isolated yields; [c]Additive: Mg (0.2 mmol), THF (0.7 µL/mg); [d]Zirconia ball; [e]Solution-based reaction conditions: THF (200 µL) vial (5 mL), rt; [f]Solution-based reaction conditions: THF (200 µL) in a Schlenk (10 mL), 40°C.

Supplementary Table 4. Optimization of the reaction frequency[a]

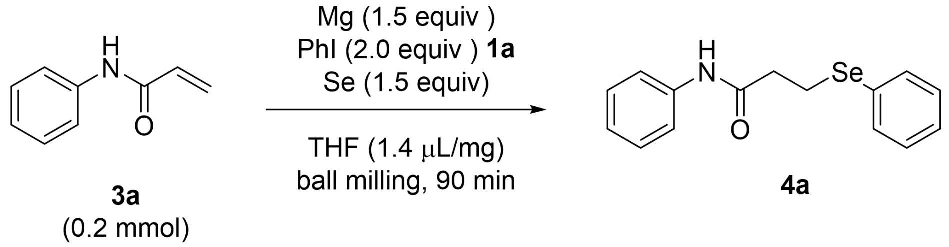

Entry	Frequency(Hz)	Yield(%)[a]
1	10	29
2	20	82
3	30	93(94)[b]

[a]Reactions performed using Retsch MM 400, stainless-steel milling jar (1.5 mL) and a stainless-steel ball (diameter: 6 mm). Conditions: **3a** (0.2 mmol), Mg (0.3 mmol), Se (0.3 mmol), **1a** (0.4 mmol), THF (1.4 μL/mg); η = V (liquid; μL)/m (reagents; mg). Yields were determined by ^{1}H NMR analysis of the crude mixture using DMF as an internal standard; [b]isolated yields.

Methods' synthesis of symmetrical dichalcogenides

Conditions A: Reactions using organic halides

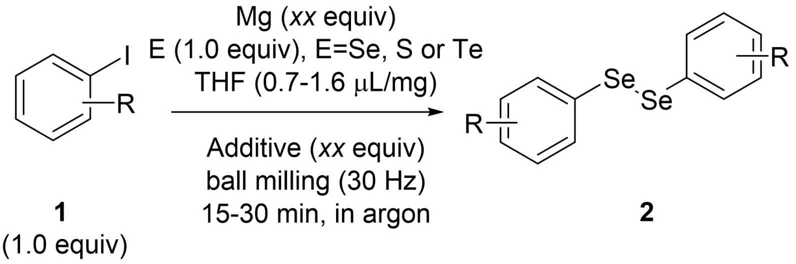

Mg turnings (0.2 mmol, 1.0 equiv, 4.8 mg) were placed in a milling jar (1.5 mL) with a ball (6 mm, diameter) in argon. The organic halides (**1**, 0.2 mmol, 1.0 equiv), Se/S/Te (0.2 mmol, 1.0 equiv, 16.0 mg/6.4 mg/25.5 mg), LiCl (0.3 mmol, 1.5 equiv, 12.7 mg) and dry THF (1.1-1.6 μL/mg) were added to the jar using a syringe. After the jar was closed in argon, the jar was placed in the ball milling (Retsch MM 400, 15-30 min, 30 Hz). After grinding for 15-30 min, the mixture was eluted from silica gel with EA (ethyl acetate), the solvent was removed by vacuum distillation, and the pure product was obtained by rapid column chromatography (SiO_2, Hexane)

Conditions B: Reactions using Benzylnitrile iodide

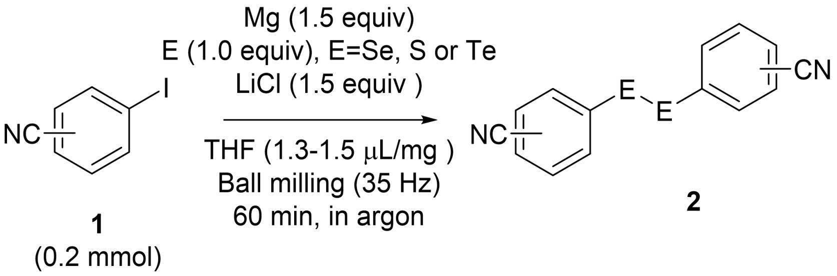

Mg turnings (0.3 mmol, 1.5 equiv, 7.2 mg) were placed in a milling jar (1.5 mL) with a ball (6 mm, diameter) in argon. The benzonitrile iodide (**1**, 0.2 mmol, 1.0 equiv, 45.8 mg), Se (0.2 mmol, 1.0 equiv, 16 mg), LiCl (0.3 mmol, 1.5 equiv, 12.7 mg) and dry THF (1.3-1.5 μL/mg) were added to the jar using a syringe. After the jar was closed in argon, the jar was placed in the ball milling (Retsch MM 500, 60 min, 35 Hz). After grinding for 60 min, the mixture was eluted from silica gel with EA (ethyl acetate), the solvent was removed by vacuum distillation, and the pure product was obtained by rapid column chromatography (SiO_2, petroleum ether/ethyl acetate, 10: 1 to 5:1).

Conditions C: Reactions using alkyl iodide

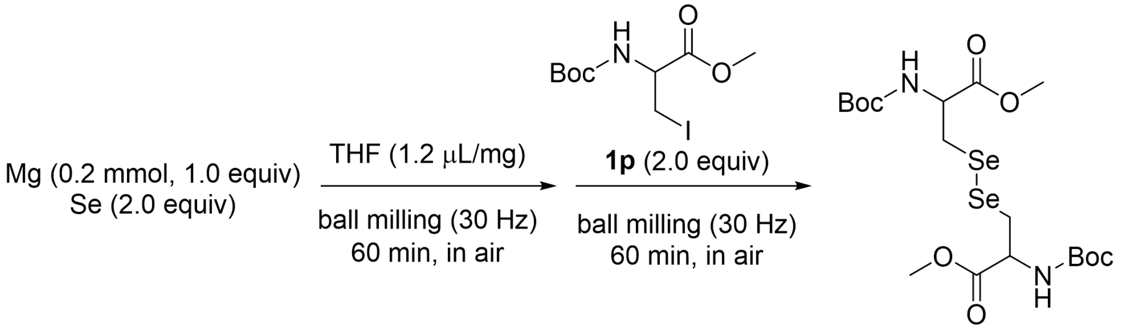

2p

Mg turnings (0.2 mmol, 1.0 equiv, 4.8 mg) were placed in a milling jar (1.5 mL) with a ball (6 mm, diameter) in argon. Se (0.4 mmol, 2.0 equiv, 32.0 mg) and dry THF (1.2 μL/mg) were added to the jar using a syringe. After the jar was closed in air, the jar was placed in the ball milling (Retsch MM 400, 60 min, 30 Hz). After grinding for 60 min, the jar was opened in air and charged with alkyl iodide (**1p**, 0.4 mmol, 2.0 equiv, 131.6 mg). The jar was then closed without purging with inert gas, and was placed in the ball milling (Retsch MM 400, 60 min, 30 Hz). After grinding for 60 min, the mixture was eluted from silica gel with EA (ethyl acetate), the solvent was removed by vacuum distillation, and the pure product was obtained by rapid column chromatography (SiO_2, petroleum ether/ethyl acetate, 10:1 to 5:1).

Methods' synthesis of unsymmetrical monochalcogenides

Conditions D: Reactions using organic halides

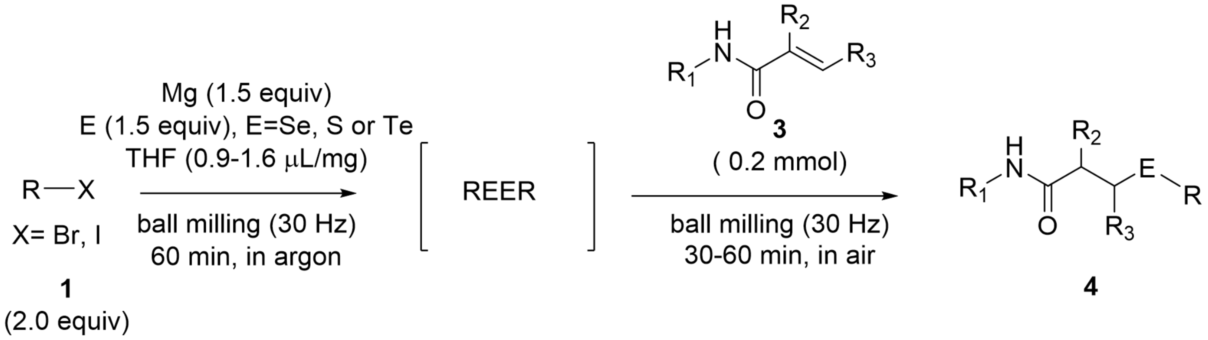

Mg turnings (0.3 mmol, 1.5 equiv, 7.2 mg) were placed in a milling jar (1.5 mL) with a ball (6 mm, diameter) in argon. An organic halides (**1**, 0.4 mmol, 2.0 equiv, 81.6 -149.0 mg), Se/S/Te (0.3 mmol, 1.5 equiv, 16.0 mg/6.4 mg/25.5 mg) and dry THF (0.9-1.6 μL/mg) were added to the jar using a syringe. After the jar was closed in argon, the jar was placed in the ball milling (Retsch MM 400, 60 min, 30 Hz). After grinding for 60 min, the jar was opened in air and charged with electrophilic reagent (**3**, 0.2 mmol, 1.0 equiv, 29.4 mg-49.4 mg). The jar was then closed without purging with inert gas, and was placed in the ball milling (Retsch MM 400, 30-60 min, 30 Hz). After grinding for 30-60 min, the mixture was eluted from silica gel with EA (ethyl acetate), the solvent was removed by vacuum distillation, and the pure product was obtained by rapid column chromatography (SiO_2, petroleum ether/ethyl acetate, 10:1 to 5:1).

Conditions E: Reactions using organic halides

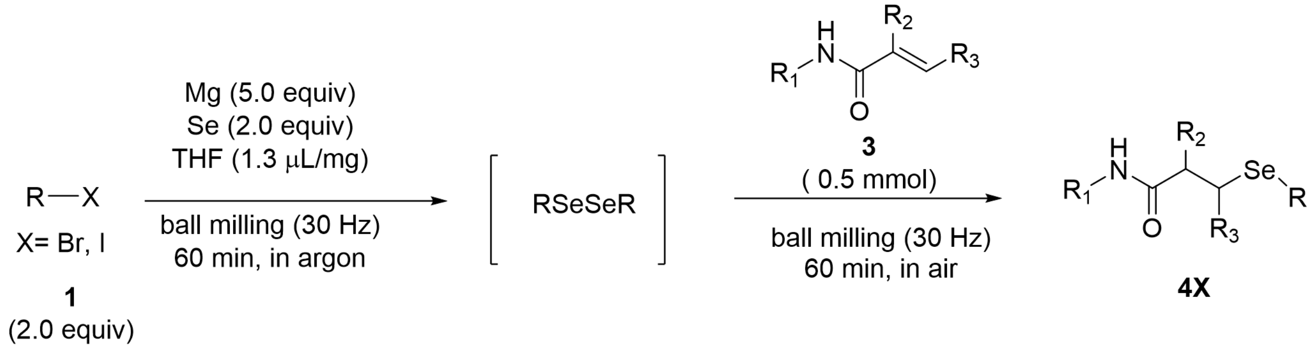

Mg turnings (2.5 mmol, 5.0 equiv, 60.0 mg) were placed in a milling jar (5.0 mL) with a ball (10 mm, diameter) in argon. An organic halides (**1**, 1.0 mmol, 2.0 equiv, 114 μL), Se (1.0 mmol, 2.0 equiv, 80.0 mg) and dry THF (1.3 μL/mg) were added to the jar using a syringe. After the jar was closed in argon, the jar was placed in the ball milling (Retsch MM 400, 60 min, 30 Hz). After grinding for 60 min, the jar was opened in air and charged with electrophilic reagent (**3**, 0.5 mmol, 1.0 equiv, 73.5 mg). The jar was then closed without purging with inert gas, and was placed in the ball milling (Retsch MM 400, 30 min, 30 Hz). After grinding for 30 min, the mixture was eluted from silica gel with EA (ethyl acetate), the solvent was removed by vacuum distillation, and the pure product was obtained by rapid column chromatography (SiO_2, petroleum ether/ethyl acetate, 10:1 to 5:1)

Conditions F: Reactions using organic halides

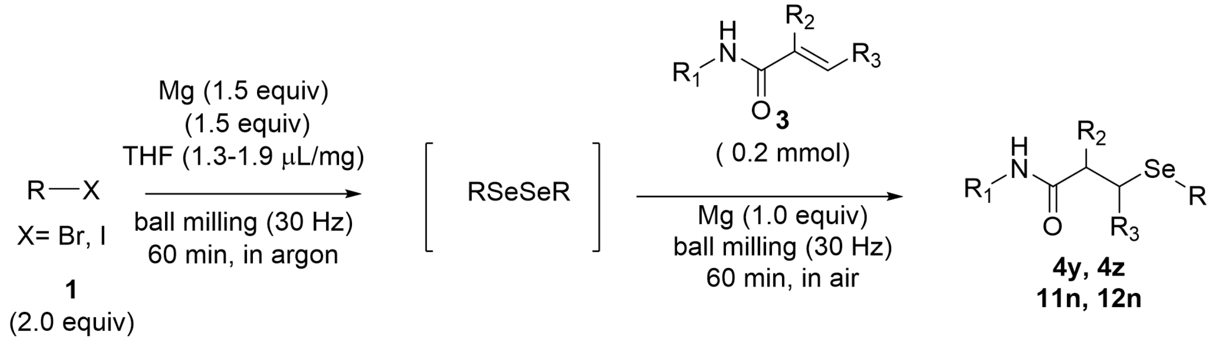

Mg turnings (0.3 mmol, 1.5 equiv, 7.2 mg) were placed in a milling jar (1.5 mL) with a ball (6 mm, diameter) in argon. An organic halides (**1**, 0.4 mmol, 2.0 equiv, 64.8-92.8 mg), Se (0.3 mmol, 1.5 equiv, 24.0 mg) and dry THF (1.3-1.9 μL/mg) were added to the jar using a syringe. After the jar was closed in argon, the jar was placed in the ball milling (Retsch MM 400, 60 min, 30 Hz). After grinding for 60 min, the jar was opened in air and charged with electrophilic reagent (**3**, 0.2 mmol, 1.0 equiv, 29.4 mg) and Mg (0.2 mmol, 1.0 equiv, 4.8 mg). The jar was then closed without purging with inert gas, and was placed in the ball milling (Retsch MM 400, 60 min, 30 Hz). After grinding for 60 min, the mixture was eluted from silica gel with EA (ethyl acetate), the solvent was removed by vacuum distillation, and the pure product was obtained by rapid column chromatography (SiO_2, petroleum ether/ethyl acetate, 10:1 to 5:1).

Conditions G: Reactions using polycyclic aromatic halides

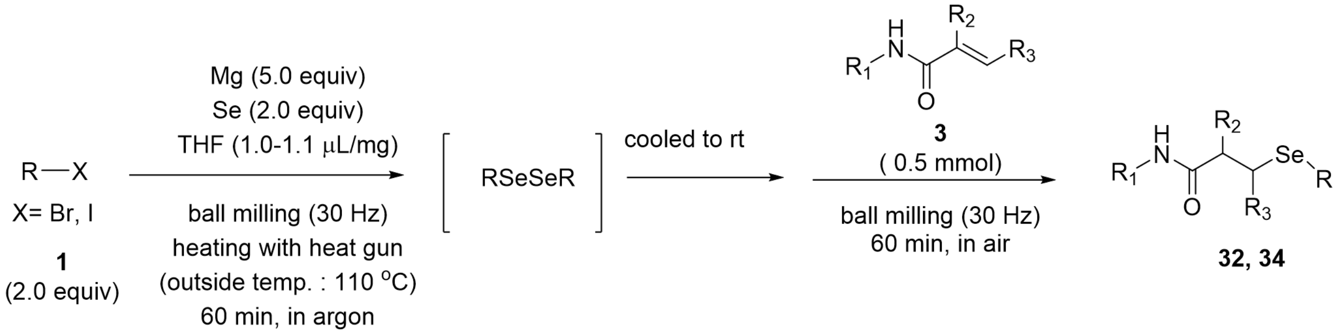

Mg turnings (2.5 mmol, 5.0 equiv, 60.0 mg) were placed in a milling jar (5.0 mL) with a ball (10 mm, diameter) in argon. An organic halides (**1**, 1.0 mmol, 2.0 equiv, 257.1-281.2 mg), Se (1.0 mmol, 2.0 equiv, 80.0 mg) and dry THF (1.0-1.1 μL/mg) were added to the jar using a syringe. After the jar was closed in argon, the jar was placed in the ball milling (Retsch MM 400, 60 min, 30 Hz). A heat gun was set approximately 110 °C above the jar and was turned on (outside temperature: 110°C)[8]. After grinding for 60 min, the jar was cooled to room temperature, opened in air and charged with electrophilic reagent (**3**, 0.5 mmol, 1.0 equiv, 73.5 mg). The jar was then closed without purging with inert gas, and was placed in the ball milling (Retsch MM 400, 60 min, 30 Hz). After grinding for 60 min, the mixture was eluted from silica gel with EA (ethyl acetate), the solvent was removed by vacuum distillation, and the pure product was obtained by rapid column chromatography (SiO_2, petroleum ether/ethyl acetate, 10:1 to 5:1).

Conditions H: Nucleophilic substitution to various electrophiles

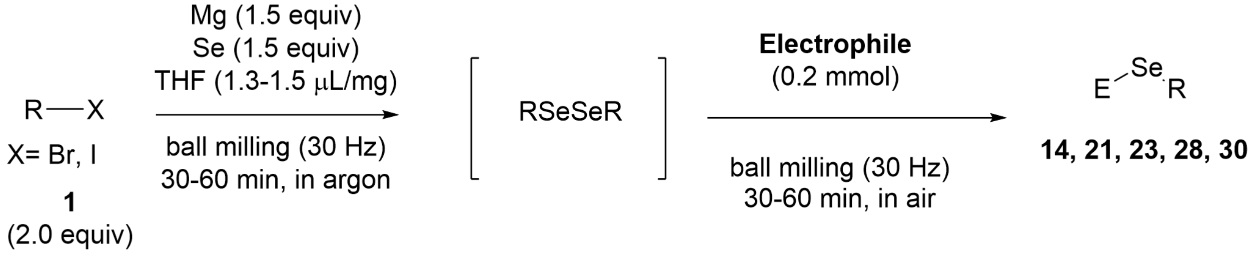

Mg turnings (0.3 mmol, 1.5 equiv, 7.2 mg) were placed in a milling jar (1.5 mL) with a ball (6 mm, diameter) in argon. An organic halides (**1**, 0.4 mmol, 2.0 equiv, 81.6 mg), Se (0.3 mmol, 1.5 equiv, 24.0 mg) and dry THF (1.3-1.5 μL/mg) were added to the jar using a syringe. After the jar was closed in argon, the jar was placed in the ball milling (Retsch MM 400, 30-60 min, 30 Hz). After grinding for 30-60 min, the jar was opened in air and charged with electrophilic reagent (**Electrophile**, 0.2 mmol, 1.0 equiv, 22.9 -45.2 mg). The jar was then closed without purging with inert gas, and was placed in the ball milling (Retsch MM 400, 30-60 min, 30 Hz). After grinding for 30-60 min, the mixture was eluted from silica gel with EA (ethyl acetate), the solvent was removed by vacuum distillation, and the pure product was obtained by rapid column chromatography (SiO_2, petroleum ether/ethyl acetate, 100:0 to 5:1).

Conditions I: Nucleophilic substitution reactions

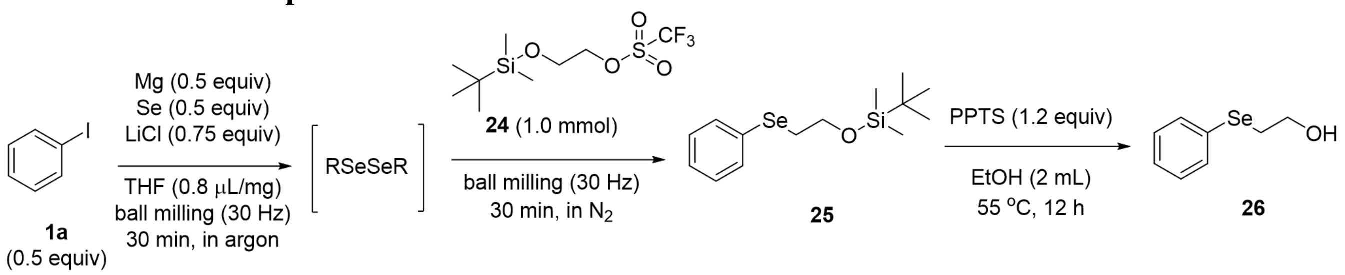

Mg turnings (0.5 mmol, 0.5 equiv, 12.0 mg) were placed in a milling jar (5.0 mL) with a ball (10 mm, diameter) in argon. An organic halides (**1**, 0.5 mmol, 0.5 equiv, 102.0 mg, 56μL), Se (0.5 mmol, 0.5 equiv, 40.0 mg), LiCl (0.75 mol, 0.75 equiv, 31.8 mg) and dry THF (0.8 μL/mg) were added to the jar using a syringe. After the jar was closed in argon, the jar was placed in the ball milling (Retsch MM 400, 30 min, 30 Hz). After grinding for 30 min, the jar was opened in argon and charged with reagent (**24**, 1.0 mmol, 1.0 equiv, 308.4 mg). The jar was then closed without purging with inert gas, and was placed in the ball milling (Retsch MM 400, 30 min, 30 Hz). After grinding for 60 min, the mixture was eluted from silica gel with EA (ethyl acetate), the solvent was removed by vacuum distillation, and the pure product **25** was obtained by rapid column chromatography (SiO_2, Hexane).

To a solution of EtOH (2 mL), **25** (0.4 mmol, 1.0 equiv, 126.1 mg) and PPTS (0.4 mmol, 1.2 equiv, 120.6 mg) were added in a Shrek tube equipped with a magnetic stirring bar at 50°C and stirred for 12 hours. The progress of the reaction was monitored by TLC and upon completion of reaction; the solvent was removed by vacuum distillation, and the pure product **26** was obtained by rapid column chromatography (SiO_2, petroleum ether/ethyl acetate, 5:1 to 3:1)

Conditions J: Nucleophilic substitution to 2-chloropyrimidine

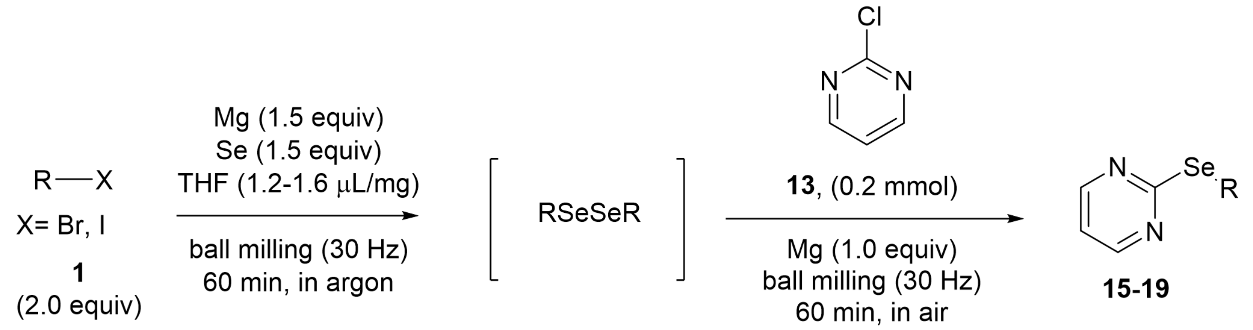

Mg turnings (0.3 mmol, 1.5 equiv, 7.2 mg) were placed in a milling jar (1.5 mL) with a ball (6 mm, diameter) in argon. An organic halides (**1**, 0.4 mmol, 2.0 equiv, 87.2 mg-113.2 mg), Se (0.3 mmol, 1.5 equiv, 24.0 mg) and dry THF (1.2-1.6 μL/mg) were added to the jar using a syringe. After the jar was closed in argon, the jar was placed in the ball milling (Retsch MM 400, 60 min, 30 Hz). After grinding for 60 min, the jar was opened in air and charged with electrophilic reagent (**13**, 0.2 mmol, 1.0 equiv, 22.9 mg) and Mg (0.2 mmol, 1.0 equiv, 4.8 mg). The jar was then closed without purging with inert gas, and was placed in the ball milling (Retsch MM 400, 60 min, 30 Hz). After grinding for 60 min, the mixture was eluted from silica gel with EA (ethyl acetate), the solvent was removed by vacuum distillation, and the pure product was obtained by rapid column chromatography (SiO_2, petroleum ether/ethyl acetate, 10:1 to 5:1).

Conditions K: Reactions in air

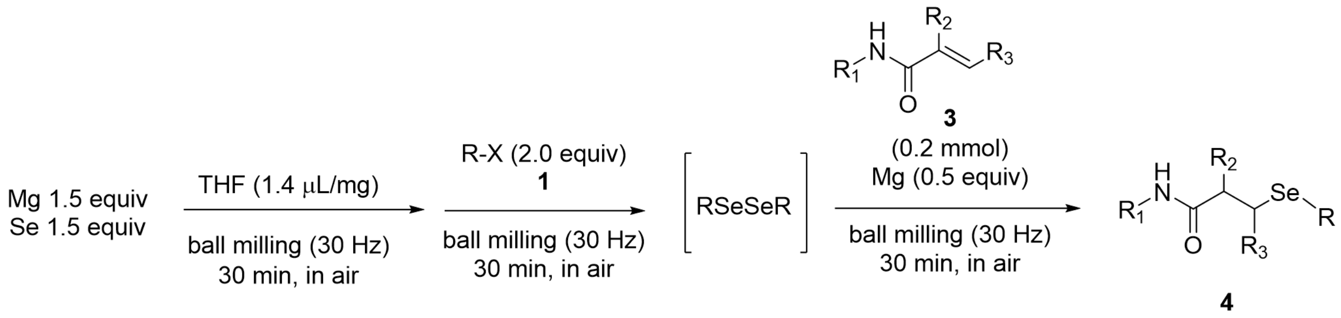

Mg turnings (0.3 mmol, 1.5 equiv, 7.2 mg) were placed in a milling jar (1.5 mL) with a ball (6 mm, diameter) in air. Se (0.3 mmol, 1.5 equiv, 24.0 mg) and THF (1.4 μL/mg) were added to the jar using a syringe. After the jar was closed in air, the jar was placed in the ball milling (Retsch MM 400, 30 min, 30 Hz). After grinding for 30 min, the jar was opened in air and charged with organic halides (**1**, 0.4 mmol, 2.0 equiv, 81.6 mg, 45 μL). The jar was then closed without purging with inert gas, and was placed in the ball milling (Retsch MM 400, 30 min, 30 Hz). After grinding for 30 min, the jar was opened in air and charged with electrophilic reagent (**3**, 0.2 mmol, 1.0 equiv, 29.4 mg), Mg turnings(0.1 mmol, 0.5 equiv, 2.4 mg) and was placed in the ball milling (Retsch MM 400, 30 min, 30 Hz). After grinding for 30 min, the mixture was eluted from silica gel with EA (ethyl acetate), the solvent was

removed by vacuum distillation, and the pure product was obtained by rapid column chromatography (SiO_2, petroleum ether/ethyl acetate, 10:1 to 5:1)

Methods' synthesis of unsymmetrical monochalcogenides in solution

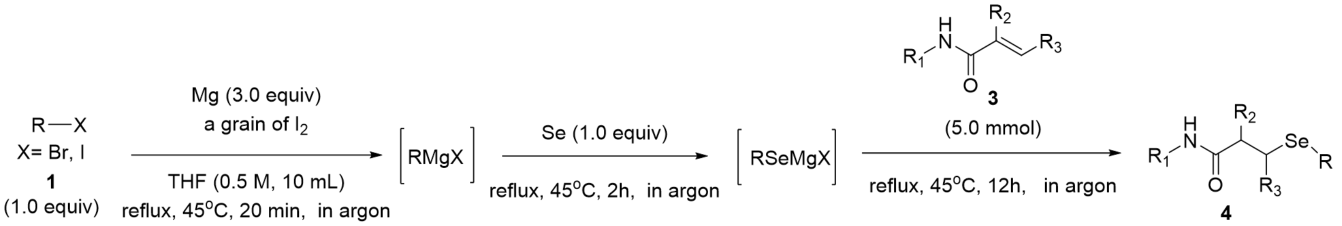

Wash the magnesium strip with 1 N HCl, wash with water and ethanol in turn, and dry the magnesium strip polishing, cutting into smaller magnesium chips, vacuum drying for a period of time for standby; under the protection of nitrogen, put the treated magnesium scraps into a dry three necked bottle (50 mL) with magneton, constant pressure drop funnel and reflux condenser, add a small piece of iodine, and heat it slightly in a constant temperature magnetic stirrer. The iodine in the bottle is heated and sublimated, increasing the contact surface with magnesium. Dissolve the organic halide in 10 mL of ultra dry THF, and iodine fades. After about 20 minutes of dripping, reflux for 20 minutes to obtain a clear liquid; Add selenium powder, reflow for about 2 hours to obtain selenium formate reagent, add electrophilic reagent, after the reaction is completed, quench with 1N HCl, extract with ethyl acetate, dry, and pass the column to obtain the product by rapid column chromatography (SiO_2, petroleum ether/ethyl acetate, 10:1 to 5:1)

Methods' synthesis of unsymmetrical monochalcogenides with gram-scale reaction

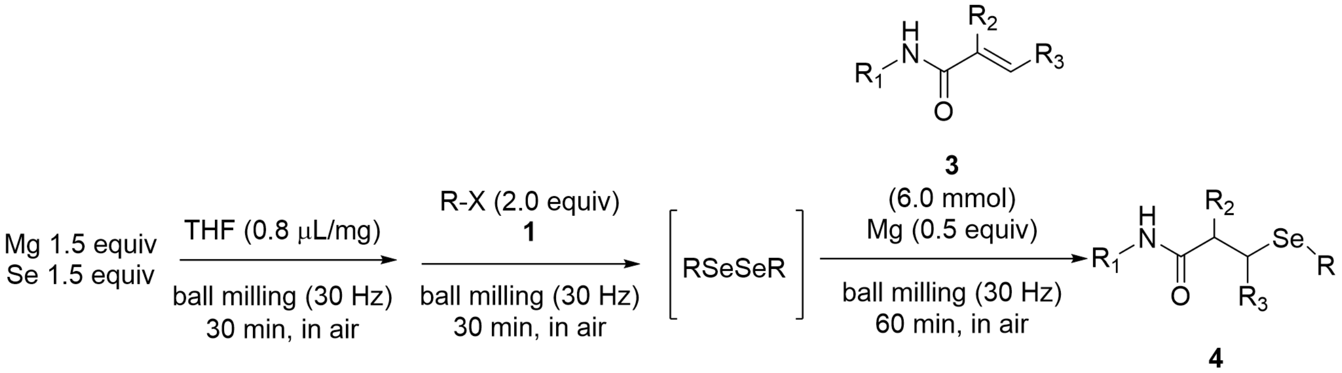

Mg turnings (9.0 mmol, 1.5 equiv, 216.0 mg) were placed in a milling jar (10 mL) with a ball (15 mm, diameter) in air. Se (9.0 mmol, 1.5 equiv, 720.0 mg) and THF (0.8 μL/mg) were added to the jar using a syringe. After the jar was closed in air, the jar was placed in the ball milling (Retsch MM 400, 30 min, 30 Hz). After grinding for 30 min, the jar was opened in air and charged with organic halides (**1**,

12 mmol, 2.0 equiv, 2448.0 mg, 1.35 mL). The jar was then closed without purging with inert gas, and was placed in the ball milling (Retsch MM 400, 30 min, 30 Hz). After grinding for 30 min, the jar was opened in air and charged with electrophilic reagent (**3**, 6.0 mmol, 1.0 equiv, 882.0 mg), Mg turnings (3.0 mmol, 0.5 equiv, 72.0 mg) and was placed in the ball milling (Retsch MM 400, 60 min, 30 Hz). After grinding for 60 min, the mixture was eluted from silica gel with EA (ethyl acetate), the solvent was removed by vacuum distillation, and the pure product was obtained by rapid column chromatography (SiO_2, petroleum ether/ethyl acetate, 10:1 to 5:1).

Mechanistic insights

a)

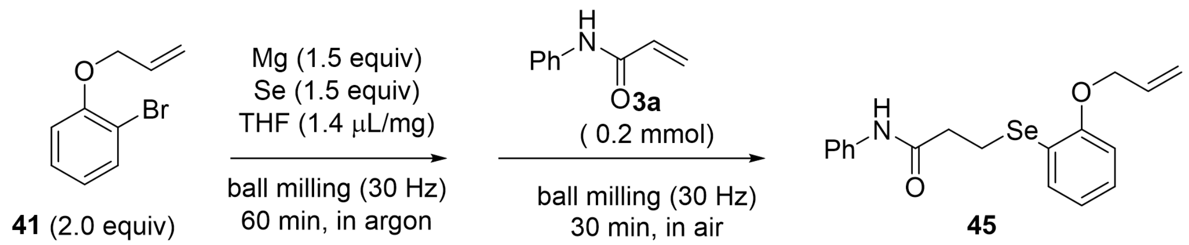

Mg turnings (0.3 mmol, 1.5 equiv, 7.2 mg) were placed in a milling jar (1.5 mL) with a ball (6 mm, diameter) in argon. An organic halides (**41**, 0.4 mmol, 2.0 equiv, 85.2 mg), Se (0.3 mmol, 1.5 equiv, 24.0 mg) and dry THF (1.4 μL/mg) were added to the jar using a syringe. After the jar was closed in argon, the jar was placed in the ball milling (Retsch MM 400, 60 min, 30 Hz). After grinding for 60 min, the jar was opened in air and charged with electrophilic reagent (**3a**, 0.2 mmol, 1.0 equiv, 29.4 mg). The jar was then closed without purging with inert gas, and was placed in the ball milling (Retsch MM 400, 30 min, 30 Hz). After grinding for 30 min, the mixture was eluted from silica gel with EA (ethyl acetate), the solvent was removed by vacuum distillation, and the pure product **45** was obtained by rapid column chromatography (SiO_2, petroleum ether/ethyl acetate, 10:1 to 5:1).

b)

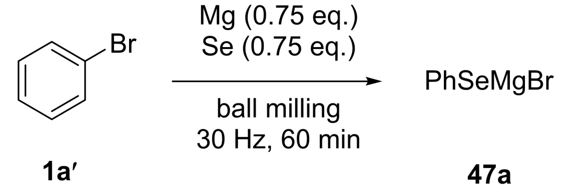

Mg turnings (0.3 mmol, 0.75 equiv, 7.2 mg) were placed in milling jar (1.5 mL) with a ball (6 mm, diameter) in argon. **1a'** (0.4 mmol, 1.0 equiv, 81.6 mg. 45 μL), Se (0.3 mmol, 0.75 equiv, 24.0 mg) and dry THF (2.1 μL/mg) were added to the jar using a syringe. After the jar was closed in argon, the jar was placed in the ball milling (Retsch MM 400, 60 min, 30 Hz). After grinding for 60 min, in the glovebox with argon protected, a portion of the mixture was performing the workup via short column

filtration with ^{77}Se (76 Hz, $CDCl_3$, rt) were tested at JEOL JNM-ECZ400S spectrometers. And the residual mixture was directly used for NEXAFS testing.

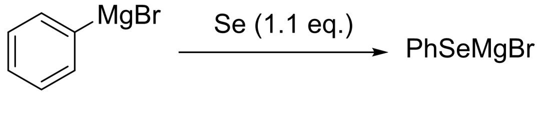

46 (1.0 M) **47b**

In a 50 mL bottle, under argon, add PhMgBr (1.0 M) 5 mL, the reaction mixture was cooled to 0°C and stirred for 10 minutes under an argon atmosphere and Selenium powder is then added. Argon purge three times and stir at room temperature 2h. Under argon, the supernatant without $MgBr_2$ was used for NEXAFS testing.

c)

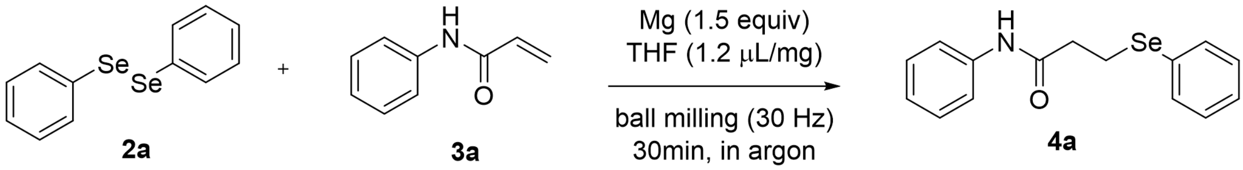

2a (1.5 equiv) **3a** (0.2 mmol) **4a**

Mg turnings (0.15 mmol, 1.5 equiv, 3.6 mg) were placed in milling jar (1.5 mL) with a ball (6 mm, diameter) in argon. **2a** (0.3 mmol, 1.5 equiv, 93.6 mg), **3** (0.2 mmol, 1.0 equiv, 29.4 mg) and dry THF (1.2 μL/mg) were added to the jar using a syringe. After the jar was closed in argon, the jar was placed in the ball milling (Retsch MM 400, 30 min, 30 Hz). After grinding for 30 min, the mixture was eluted from silica gel with EA (ethyl acetate), the solvent was removed by vacuum distillation, and the curde product yield were determined by ^1H NMR analysis of the crude mixture using DMF as an internal standard and the pure product was obtained by rapid column chromatography (SiO_2, petroleum ether/ethyl acetate, 10:1 to 5:1)

X-Ray Absorption Fine Structure (XAFS) Analysis

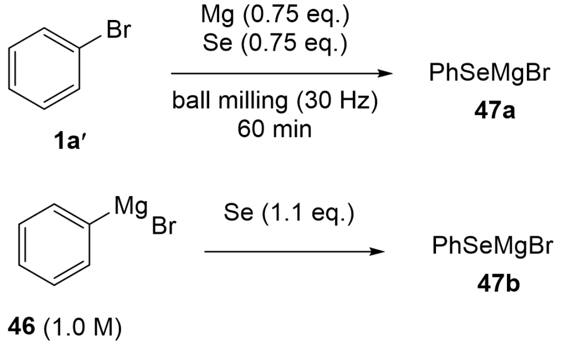

sampling in glovebox(Ar)
then C-K, Mg-K and Se-L_3 NEXAFS analysis
under reduced pressure at 3.4 X 10^{-6} Pa

Mg K-edge[8, 9], C K-edge NEXAFS[8, 10], Se L_3-edge[11] NEXAFS measurements were performed at the soft X-ray beamline BL08U1A of SSRF. The mechanochemically-prepared gummy organoselemium sample of **47a** was pasted on a high purity gold foil and fixed onto a copper sample holder. For the sample preparation of solution-phase **47b**[12], 1.0 M of THF solution of **47b** without $MgBr_2$ precipitation was dropped and dried on a high purity gold foil. The sample holder was transferred into a vacuum chamber and fixed on a linear and rotatable manipulator. The chamber was evacuated to a pressure of less than 3.4×10^{-6} Pa. The sample preparation and install processes were carefully performed under argon atmospheres. All the NEXAFS spectra were taken in total electron yield (TEY) mode by measuring the sample drain current.

The Mg K-edge NEXAFS spectra were measured in the X-ray energy range of 1250-1400 eV with the energy step set to 0.2 eV in the range of 1290-1320 eV and 1.0 eV in the ranges of 1250-1290 eV and 1320-1400 eV.

The C K-edge NEXASF spectra were taken in the X-ray energy range of 280-300 eV. The energy step size for C spectra was set to 0.05 eV in the range of 283-289 eV and 0.2 eV in the ranges of 280-283 eV and 289-300 eV.

The Se L_3-edge NEXAFS spectra were taken in the X-ray energy range of 1330-1500 eV. The energy step for Se spectra was set to 0.2 eV in the range of 1400-1500 eV and 1.0 eV in the range of 1330-1400 eV.

The data processing for the removal of background of incident X-ray, baseline subtraction, and normalization of the resulting spectra were performed by using Origin software.

Characterization of Obtained Products

2-diphenyldiselane(2a)

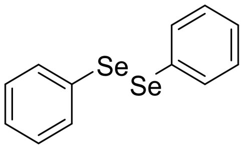

Prepared by **Conditions A.** Yellow solid, 27.5 mg, 88% yield, η = 1.4 μL/mg; ^{1}H and ^{13}C NMR of the product **2a** were in agreement with the literature[13]; ^{77}Se NMR of the product **2a** were in agreement with the literature[14]. **^{1}H NMR (400 MHz, CDCl$_3$)** δ 7.61-7.63 (m, 4H, Ph-H), 7.23-7.30 (m, 6H, Ph-H); **^{13}C NMR (101 MHz, CDCl$_3$)** δ 131.6 (CH), 131.0 (C-Se), 129.3 (CH), 127.8 (CH); **^{77}Se NMR (76 MHz, CDCl$_3$)** δ 463.0.

1,2-bis(4-(trimethylsilyl)phenyl)diselane(2b)

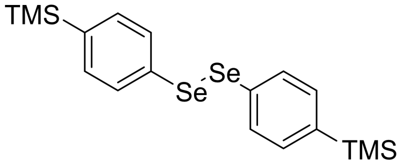

Prepared by **Conditions A.** Yellow grease, 37.8 mg, 83% yield, η = 1.2 μL/mg; ^{1}H and ^{13}C NMR of the product **2b** were in agreement with the literature[15]. **^{1}H NMR (400 MHz, CDCl$_3$)** δ 7.61 (d, J = 7.6 Hz, 4H, Ph -H), 7.42 (d, J = 8.0 Hz, 4H, Ph -H), 1.04 (s, 18H, CH$_3$); **^{13}C NMR (101 MHz, CDCl$_3$)** δ 140.0 (C), 134.2 (CH), 131.9 (C-Se), 130.3 (CH), -1.0 (CH$_3$); **^{77}Se NMR (76 MHz, CDCl$_3$)** δ 450.4.

1,2-bis(4-chlorophenyl)diselane(2c)

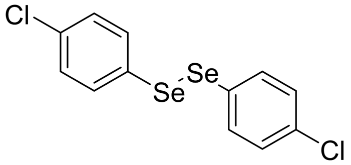

Prepared by **Conditions A.**Yellow grease, 36.8 mg, 97% yield, η = 1.2 μL/mg; ^{1}H and ^{13}C NMR of the product **2c** were in agreement with the literature[13]. **^{1}H NMR (400 MHz, CDCl$_3$)** δ 7.51 (d, J = 8.8 Hz, 4H, Ph-H), 7.24 (d, J = 8.4 Hz, 4H, Ph-H); **^{13}C NMR (101 MHz, CDCl$_3$)** δ 134.4 (C), 133.4 (CH), 129.5 (CH), 128.9 (C-Se); **^{77}Se NMR (76 MHz, CDCl$_3$)** δ 477.2.

1,2-bis(4-(*tert*-butyl)phenyl)diselane(2d)

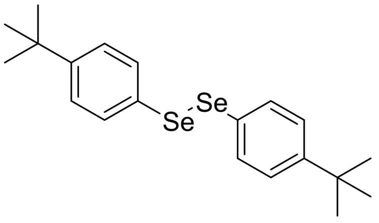

Prepared by **Conditions A.**Yellow grease, 38.9 mg, 91% yield, η = 1.2 μL/mg; ^{1}H and ^{13}C NMR of the product **2d** were in agreement with the literature[16]. **^{1}H NMR (400 MHz, CDCl$_3$)** δ 7.57 (d, J = 8.0 Hz, 4H, Ph-H), 7.31 (d, J = 8.0 Hz, 4H, Ph-H), 1.32 (s, 18H, CH$_3$); **^{13}C NMR**

(101 MHz, $CDCl_3$) δ 151.2 (C), 131.7 (CH), 127.8 (C-Se), 126.4 (CH), 34.7 (C), 31.4 (CH_3); **^{77}Se NMR (76 MHz, $CDCl_3$)** δ 461.9.

1,2-bis(4-ethylphenyl)diselane(2e)

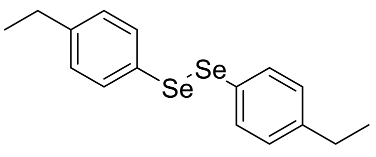

Prepared by **Conditions A.** Yellow grease, 32.4 mg, 88% yield, η = 1.3 μL/mg; ^{1}H and ^{13}C NMR of the product **2e** were in agreement with the literature[17]. **^{1}H NMR (400 MHz, $CDCl_3$)** δ 7.54 (d, J = 8.0 Hz, 4H, Ph-H), 7.11 (d, J = 8.0 Hz, 4H, Ph-H), 2.64 (q, J = 7.6 Hz, 4H, CH_2), 1.23 (t, J = 7.6 Hz, 6H, CH_3); **^{13}C NMR (101 MHz, $CDCl_3$)** δ 144.4 (C), 132.3 (CH), 128.9 (CH), 128.0 (C-Se), 28.6 (CH_2), 15.6 (CH_3); **^{77}Se NMR (76 MHz, $CDCl_3$)** δ 471.4.

1,2-bis(4-fluorophenyl)diselane (2f)

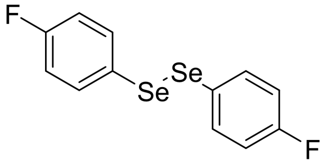

Prepared by **Conditions A.**Yellow grease, 30.1 mg, 86% yield, η = 1.3 μL/mg; ^{1}H, ^{13}C and ^{19}F NMR of the product **2f** were in agreement with the literature[13]. **^{1}H NMR (400 MHz, $CDCl_3$)** δ 7.55 (dd, J = 8.8, 5.2 Hz, 4H, Ph-H), 6.97 (t, J = 8.4 Hz, 4H, Ph-H); **^{13}C NMR (101 MHz, $CDCl_3$)** δ 163.1 (d, J =248.4 Hz, C), 134.9 (d, J = 8.2 Hz, CH), 125.7 (d, J = 3.0 Hz, C-Se), 116.5 (d, J = 21.9 Hz, CH); **^{77}Se NMR (76 MHz, $CDCl_3$)** δ 492.5; **^{19}F NMR (376 MHz, $CDCl_3$)** δ -112.9.

1,2-di-p-tolyldiselane(2g)

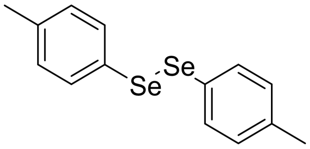

Prepared by **Conditions A.** Yellow grease, 30.1 mg, 88% yield, η = 1.3 μL/mg; ^{1}H and ^{13}C NMR of the product **2g** were in agreement with the literature[13]. **^{1}H NMR (400 MHz, $CDCl_3$)** δ 7.50 (d, J = 8.0 Hz, 4H, Ph-H), 7.08 (d, J = 8.0 Hz, 4H, Ph-H), 2.35 (s, 6H, CH_3); **^{13}C NMR (101 MHz, $CDCl_3$)** δ 138.1 (C), 132.4 (CH), 130.0 (CH), 127.7 (C-Se), 21.2 (CH_3); **^{77}Se NMR (76 MHz, $CDCl_3$)** δ 474.8.

1,2-bis(4-methoxyphenyl)diselane (2h)

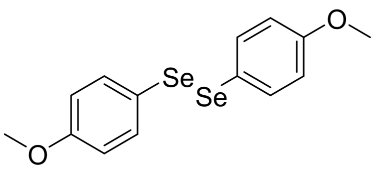

Prepared by **Conditions A.** Yellow grease, 27.6 mg, 74% yield, η = 1.3 μL/mg; ^{1}H and ^{13}C NMR of the product **2h** were in agreement with the literature[13]. **^{1}H NMR (400 MHz, $CDCl_3$)** δ 7.51 (d, J = 8.8 Hz, 4H, Ph-H), 6.81 (d, J = 8.8 Hz, 4H, Ph-H), 3.81 (s, 6H, OCH_3); **^{13}C NMR**

(101 MHz, $CDCl_3$) δ 160.1 (C), 135.6 (CH), 122.0 (C-Se), 114.8 (CH), 55.4 (OCH_3); **^{77}Se NMR (76 MHz, $CDCl_3$)** δ 505.3.

4,4'-diselanediyldibenzonitrile(2i)

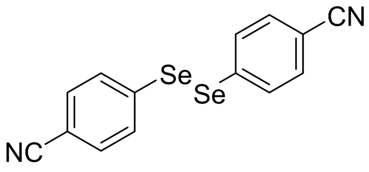

Prepared by **Conditions B.** Yellow solid, 26.9mg, 74% yield, η = 1.3 μL/mg; ^{1}H and ^{13}C NMR of the product **2i** were in agreement with the literature[18]. **^{1}H NMR (400 MHz, $CDCl_3$)** δ 7.68 (d, J = 8.4 Hz, 4H, Ph-H), 7.55 (d, J = 8.4 Hz, 4H, Ph-H); **^{13}C NMR (101 MHz, $CDCl_3$)** δ 136.5 (C-Se), 135.3 (CH), 130.6 (CH), 118.3 (CN), 111.5 (C); **^{77}Se NMR (76 MHz, $CDCl_3$)** δ 455.3.

1,2-bis(2-fluorophenyl)diselane(2j)

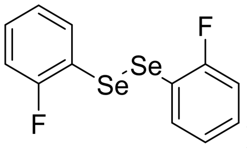

Prepared by **Conditions A.** Yellow grease, 32.8 mg, 94% yield, η = 1.3 μL/mg; ^{1}H and ^{13}C NMR of the product **2j** were in agreement with the literature[19]. **^{1}H NMR (400 MHz, $CDCl_3$)** δ 7.64 (t, J = 8.0 Hz, 2H, Ph-H), 7.24-7.30 (m, 2H, Ph-H), 7.02-7.10 (m, 4H, Ph-H); **^{13}C NMR (101 MHz, $CDCl_3$)** δ 160.9 (d, J = 243.3 Hz, C), 134.1 (d, J = 1.4 Hz, CH), 130.2 (d, J = 7.6 Hz, CH), 125.2 (d, J = 3.4 Hz, CH), 116.9 (d, J = 21.9 Hz, C-Se), 115.5 (d, J = 22.9 Hz, CH); **^{77}Se NMR (76 MHz, $CDCl_3$)** δ 376.3; **^{19}F NMR (376 MHz, $CDCl_3$)** δ -103.1.

1,2-di-*o*-tolyldiselane(2k)

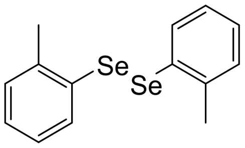

Prepared by **Conditions A.** Yellow grease, 30.1 mg, 89% yield, η = 1.3 μL/mg; ^{1}H and ^{13}C NMR of the product **2k** were in agreement with the literature[13]. **^{1}H NMR (400 MHz, $CDCl_3$)** δ 7.65 (d, J = 8.2 Hz, 2H, Ph-H), 7.17-7.18 (m, 4H, Ph-H), 7.07-7.12 (m, 2H, Ph-H), 2.45 (s, 6H, CH_3); **^{13}C NMR (101 MHz, $CDCl_3$)** δ 138.9 (C), 132.7 (CH), 131.0 (C-Se), 130.1 (CH), 128.1 (CH), 127.5 (CH), 22.3 (CH_3); **^{77}Se NMR (76 MHz, $CDCl_3$)** δ 401.5.

2,2'-diselanediyldibenzonitrile(2l)

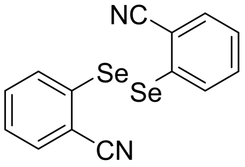

Prepared by **Conditions B.** Yellow solid, 24.6 mg, 68% yield, η = 1.3 μL/mg; ^{1}H and ^{13}C NMR of the product **2l** were in agreement with the literature[20]. **^{1}H NMR (400 MHz, $CDCl_3$)** δ 7.84 (d, J = 8.0 Hz, 2H, Ph-H), 7.63 (dd, J = 7.6, 1.2 Hz, 2H, Ph-H), 7.52 (td, J = 7.8, 1.3 Hz, 2H, Ph-H),

7.39 (t, J = 7.6 Hz, 2H, Ph-H); **^{13}C NMR (101 MHz, CDCl$_3$)** δ 134.1 (CH), 134.0 (C-Se), 133.8 (CH), 133.6 (CH), 128.9 (CH), 117.3 (CN), 115.7 (C); **^{77}Se NMR (76 MHz, CDCl$_3$)** δ 484.9.

1,2-bis(3-methoxyphenyl)diselane(2m)

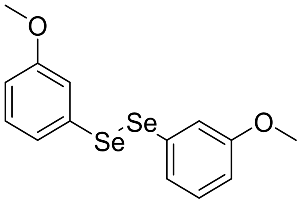

Prepared by **Conditions A.** Yellow grease, 36.7 mg, 98% yield, η = 1.3 μL/mg; ^{1}H and ^{13}C NMR of the product **2m** were in agreement with the literature[13]. **^{1}H NMR (400 MHz, CDCl$_3$)** δ 7.16-7.21 (m, 6H, Ph-H), 6.77-6.80 (m, 2H, Ph-H), 3.77 (s, 6H, OCH$_3$); **^{13}C NMR (101 MHz, CDCl$_3$)** δ 160.0 (C), 131.9 (C-Se), 130.0 (CH), 123.6 (CH), 116.6 (CH), 113.9 (CH), 55.4 (OCH$_3$); **^{77}Se NMR (76 MHz, CDCl$_3$)** δ 465.8.

1,2-di(pyridin-2-yl)diselane(2n)

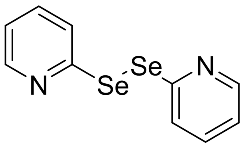

Prepared by **Conditions A.** Yellow grease, 27.6 mg, 88% yield, η = 1.3 μL/mg; ^{1}H and ^{13}C NMR of the product **2n** were in agreement with the literature[13]. **^{1}H NMR (400 MHz, CDCl$_3$)** δ 8.45 (d, J = 4.6 Hz, 2H, Ar-H), 7.79 (d, J = 8.0 Hz, 2H, Ar-H), 7.54 (td, J = 8.0, 1.6 Hz, 2H, Ar-H), 7.08 (dd, J = 6.8, 5.2 Hz, 2H, Ar-H); **^{13}C NMR (101 MHz, CDCl$_3$)** δ 154.5 (C-Se), 149.7 (CH), 137.7 (CH), 123.7 (CH), 121.4 (CH); **^{77}Se NMR (76 MHz, CDCl$_3$)** δ 447.6.

1,2-di(thiophen-2-yl)diselane(2o)

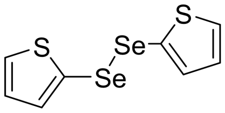

Prepared by **Conditions A.** Yellow grease, 23.8 mg, 73% yield, η = 1.3 μL/mg; ^{1}H and ^{13}C NMR of the product **2o** were in agreement with the literature[13]. **^{1}H NMR (400 MHz, CDCl$_3$)** δ 7.49 (dd, J = 5.2, 0.8 Hz, 2H, Ar-H), 7.23 (dd, J = 3.6, 0.8 Hz, 2H, Ar-H), 7.01 (dd, J = 5.2, 3.6 Hz, 2H, Ar-H); **^{13}C NMR (101 MHz, CDCl$_3$)** δ 137.1 (CH), 133.1 (CH), 128.3 (CH), 125.7 (C-Se); **^{77}Se NMR (76 MHz, CDCl$_3$)** δ 494.3.

Dimethyl 3,3'-diselanediylbis(2-((*tert*-butoxycarbonyl)-amino)-propanoate) (2p)

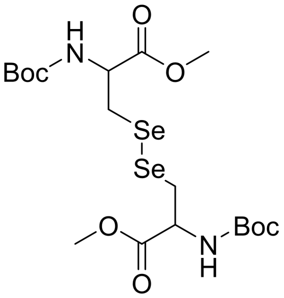
Prepared by **Conditions C.** Light yellow Solid, 55% NMR yield, η = 1.2 µL/mg; ^{1}H and ^{13}C NMR of the product **2p** were in agreement with the literature[21]. **^{1}H NMR (400 MHz, $CDCl_3$)** δ 5.37 (d, J = 7.6 Hz, 2H, N-H), 4.55 (d, J = 6.8 Hz, 2H, CH), 3.73 (s, 6H, CH_3), 2.96-2.99 (m, 4H, CH_2-Se), 1.41 (s, 18H, CH_3); **^{13}C NMR (101 MHz, $CDCl_3$)** δ 171.4 (C=O), 155.2 (C=O), 80.3 (C), 53.6 (CH), 52.7 (CH_3), 28.3 (CH_3), 27.1 (CH_2-Se); **^{77}Se NMR (76 MHz, $CDCl_3$)** δ 101.8; **HR-MS (ESI)** m/z calcd for $C_{18}H_{32}N_2O_8Se_2Na$, $[M+Na]^+$: 587.03813, found: 587.03815.

Di-tert-butyl 3,3'-diselanediylbis(azetidine-1-carboxylate) (2q)

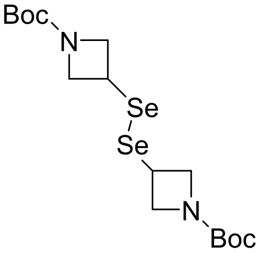
Prepared by **Conditions A.** Yellow grease, 35.8 mg, 76% yield, η = 1.1 µL/mg, melting point 87 – 90°C; **^{1}H NMR (400 MHz, $CDCl_3$)** δ 4.31-4.42 (m, 4H, CH_2), 4.08-4.14 (m, 2H, CH), 3.87-3.99 (m, 4H, CH_2), 1.42 (s, 18H, CH_3); **^{13}C NMR (101 MHz, $CDCl_3$)** δ 156.1 (C=O), 80.1 (C), 29.4 (CH_2), 28.5 (CH_3), 27.6 (CH-Se); **^{77}Se NMR (76 MHz, $CDCl_3$)** δ 375.4; **IR (film)**: ν (cm^{-1}) 2973, 2928, 2876, 1697, 1388, 1152, 771, 573; **HR-MS (ESI)** m/z calcd for $C_{16}H_{28}N_2O_4Se_2K$, $[M+K]^+$: 519.06371, found: 519.06510.

1,2-diphenyldisulfane(2ra)

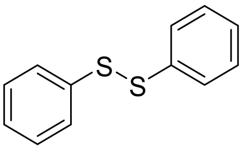
Prepared by **Conditions A.** White solid, 21.2 mg , 97% yield, η = 1.6 µL/mg; ^{1}H and ^{13}C NMR of the product **2ra** were in agreement with the literature[22]. **^{1}H NMR (400 MHz, $CDCl_3$)** δ 7.49-7.52 (m, 4H, Ph-H), 7.28-7.33 (m, 4H, Ph-H), 7.21-7.25 (m, 4H, Ph-H); **^{13}C NMR (101 MHz, $CDCl_3$)** δ 137.1 (C-S), 129.2 (CH), 127.6 (CH), 127.3 (C).

1,2-di-*p*-tolyldisulfane(2rb)

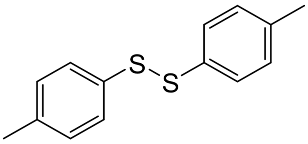
Prepared by **Conditions A.** Colorless grease, 17.1 mg, 69% yield, η = 1.6 µL/mg; ^{1}H and ^{13}C NMR of the product **2rb** were in agreement with the literature[22]. **^{1}H NMR (400**

MHz, $CDCl_3$) δ 7.39 (d, *J* = 8.0 Hz, 4H, Ph-H), 7.10 (d, *J* = 8.0 Hz, 4H, Ph-H), 2.33 (s. 6H, CH_3); **^{13}C NMR (101 MHz, $CDCl_3$)** δ 136.9 (C), 133.1 (C-S), 129.4 (CH), 122.8 (CH), 21.2 (CH_3).

1,2-bis(4-chlorophenyl)disulfane(2rc)

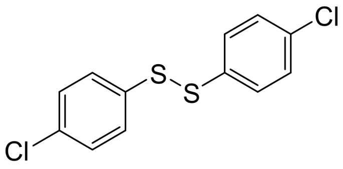

Prepared by **Conditions A.** Colorless grease, 24.0 mg, 84% yield, η = 1.5 μL/mg; ^{1}H and ^{13}C NMR of the product **2rc** were in agreement with the literature[23]. **^{1}H NMR (400 MHz, $CDCl_3$)** δ 7.40 (d, *J* = 8.0 Hz, 4H, Ph-H), 7.28 (d, *J* = 8.0 Hz, 4H, Ph-H); **^{13}C NMR (101 MHz, $CDCl_3$)** δ 135.3 (C-S), 133.8 (C), 132.1 (CH), 128.9 (CH).

4,4'-disulfanediyldibenzonitrile(2rd)

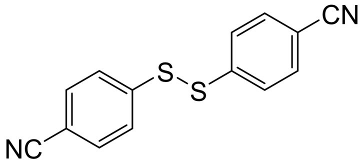

Prepared by Conditions **B**. White soild, 20.3 mg, 76% yield, η = 1.5 μL/mg; ^{1}H and ^{13}C NMR of the product **2rd** were in agreement with the literature[24]. **^{1}H NMR (400 MHz, $CDCl_3$)** δ 7.54-7.61 (m, 8H, Ph-H); **^{13}C NMR (101 MHz, $CDCl_3$)** δ 142.2 (C-S), 133.8 (CH), 126.6 (CH), 120.4 (C), 113.8 (CN).

1,2-di-*o*-tolyldisulfane(2re)

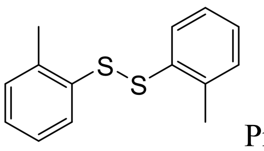

Prepared by **Conditions A.** Colorless grease, 17.7 mg, 72% yield, η = 1.6 μL/mg; ^{1}H and ^{13}C NMR of the product **2re** were in agreement with the literature[25]. **^{1}H NMR (400 MHz, $CDCl_3$)** δ 7.50-7.53 (m, 2H, Ph-H), 7.04-7.19 (m, 6H, Ph-H), 2.43 (S, 6H, CH_3); **^{13}C NMR (101 MHz, $CDCl_3$)** δ 131.3 (C), 130.6 (C-S), 130.4 (CH), 128.7 (CH), 127.4 (CH), 126.8 (CH), 20.1 (CH_3).

1,2-bis(3-methoxyphenyl)disulfane(2rf)

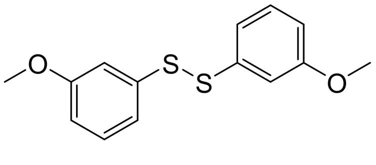

Prepared by Conditions **A**. Colorless grease, 22.7 mg, 82% yield, η = 1.5 μL/mg; ^{1}H and ^{13}C NMR of the product **2rf** were in agreement with the literature[26]. **^{1}H NMR (400 MHz, $CDCl_3$)** δ 7.21 (t, *J* = 8.0 Hz, 2H, Ph-H), 7.06-7.09 (m, 4H, Ph-H), 6.75-6.78 (m, 2H, Ph-H), 3.77 (s, 6H, CH_3); **^{13}C NMR (101 MHz, $CDCl_3$)** δ 160.2 (C), 138.4 (C-S), 129.1 (CH), 119.7 (CH), 113.3 (CH), 112.7 (CH), 54.4 (CH_3).

1,2-di(pyridin-2-yl)disulfane(2rg)

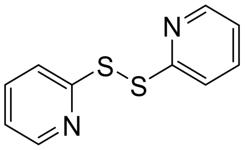

Prepared by Conditions **A**. Yellow soild, 19.2 mg, 87% yield, η = 1.6 μL/mg; ^{1}H and ^{13}C NMR of the product **2rg** were in agreement with the literature[27]. **^{1}H NMR (400 MHz, CDCl$_3$)** δ 7.58 (t, J = 8.2 Hz, 4H, Ar-H), 7.37-7.42 (m, 2H, Ar-H), 6.76-6.80 (m, 2H, Ar-H); **^{13}C NMR (101 MHz, CDCl$_3$)** δ 176.9 (C-S), 138.0 (CH), 136.9 (CH), 134.0 (CH), 114.1 (CH).

1,2-di(thiophen-2-yl)disulfane(2rh)

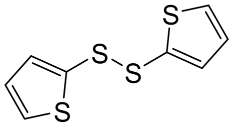

Prepared by Conditions **A**. Yellow soild, 13.6 mg, 59% yield, η = 1.6 μL/mg; ^{1}H and ^{13}C NMR of the product **2rh** were in agreement with the literature[27]. **^{1}H NMR (400 MHz, CDCl$_3$)** δ 7.58 (t, J = 8.2 Hz, 4H, Ar-H), 7.37-7.42 (m, 2H, Ar-H), 6.76-6.80 (m, 2H, Ar-H); **^{13}C NMR (101 MHz, CDCl$_3$)** δ 124.1 (CH), 121.4 (CH), 121.2 (C-S), 117.7 (CH).

1,2-diphenylditellane(2sa)

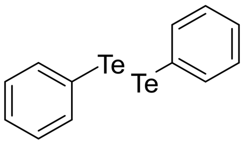

Prepared by **Conditions A.** Red solid, 32.6 mg, 80% yield, η = 1.4 μL/mg; ^{1}H and ^{13}C NMR of the product **2sa** were in agreement with the literature[13]. **^{1}H NMR (400 MHz, CDCl$_3$)** δ 7.80 (d, J = 8.4 Hz, 4H, Ph-H), 7.17-7.26 (m, 6H, Ph-H); **^{13}C NMR (101 MHz, CDCl$_3$)** δ 137.7 (CH), 129.4 (CH), 128.2 (C), 108.0 (C-Te).

1,2-di-p-tolylditellane(2sb)

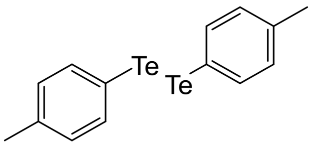

Prepared by **Conditions A.** Red grease, 36.1 mg, 83% yield, η = 1.3 μL/mg; ^{1}H and ^{13}C NMR of the product **2sb** were in agreement with the literature[13]. **^{1}H NMR (400 MHz, CDCl$_3$)** δ 7.69 (d, J = 8.0 Hz, 4H, Ph-H), 7.01 (d, J = 8.0 Hz, 4H, Ph-H), 2.38 (s, 6H, CH$_3$); **^{13}C NMR (101 MHz, CDCl$_3$)** δ 138.4 (C), 138.2 (CH), 128.8 (CH), 104.2 (C-Te), 20.4 (CH$_3$).

1,2-bis(4-chlorophenyl)ditellane(2sc)

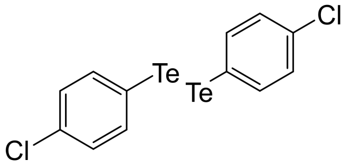

Prepared by **Conditions A.** Red soild, 26.7 mg, 56% yield, η = 1.3 μL/mg; ^{1}H and ^{13}C NMR of the product **2sc** were in agreement with the literature[13]. **^{1}H NMR (400 MHz, CDCl$_3$)** δ 7.69 (d, J = 8.0 Hz, 4H, Ph-H), 7.15 (d, J = 8.0 Hz, 4H, Ph-H); **^{13}C NMR (101 MHz, CDCl$_3$)** δ 139.8 (CH), 136.0 (C), 130.4 (CH), 104.2 (C-Te).

4,4'-ditellanediyldibenzonitrile(2sd)

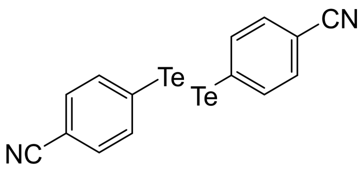

Prepared by **Conditions B.** Red soild, 23.1 mg, 50% yield, η = 1.3 μL/mg; **^{1}H NMR (400 MHz, $CDCl_3$)** δ 7.67 (d, J = 8.0, 4H, Ph-H), 7.44 (d, J = 8.0, 4H, Ph-H); **^{13}C NMR (101 MHz, $CDCl_3$)** δ 136.7 (CH), 132.6 (CH), 120.2 (C-Te), 114.5 (CN), 112.8 (C); **IR (film)**: ν (cm^{-1}) 2923, 2851, 2229, 1724, 1584, 1479, 1011, 819, 542; **HR-MS (ESI)** m/z calcd for $C_{14}H_9N_2Te_2$, $[M+H]^+$: 464.88847, found: 464.88931.

1,2-di-o-tolylditellane(2se)

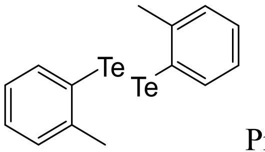

Prepared by **Conditions A.** Red grease, 24.2 mg, 55% yield, η = 1.3 μL/mg; ^{1}H and ^{13}C NMR of the product **2se** were in agreement with the literature[28]. **^{1}H NMR (400 MHz, $CDCl_3$)** δ 7.90 (d, J = 8.0 Hz, 4H, Ph-H), 7.14-7.20 (m, 4H, Ph-H), 6.96-7.01 (m, 2H, Ph-H), 2.50 (s, 6H, CH_3); **^{13}C NMR (101 MHz, $CDCl_3$)** δ 141.8 (C), 139.6 (CH), 129.0 (CH), 128.4 (CH), 126.7 (CH), 111.0 (C-Te), 20.1 (CH_3).

1,2-bis(3-methoxyphenyl)ditellane (2sf)

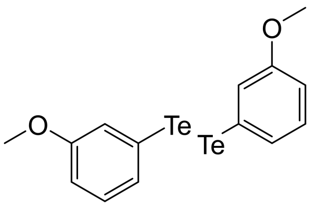

Prepared by **Conditions A.** Red grease, 39.2 mg, 83% yield, η = 1.3 μL/mg; ^{1}H and ^{13}C NMR of the product **2sf** were in agreement with the literature[29]. **^{1}H NMR (400 MHz, $CDCl_3$)** δ 7.32-7.36 (m, 4H, Ph-H), 7.06 (t, J = 8.0 Hz, 2H, Ph-H), 6.73-6.75 (m, 2H, Ph-H), 3.74 (s, 6H, CH_3); **^{13}C NMR (101 MHz, $CDCl_3$)** δ 159.7 (C), 130.0 (CH), 129.8 (CH), 121.9 (CH), 113.0 (CH), 108.0 (C-Te), 53.7 (CH_3).

1,2-di(pyridin-2-yl)ditellane(2sg)

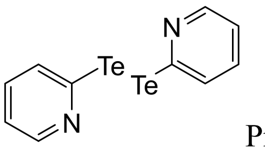

Prepared by **Conditions A.** Red grease, 32.6 mg, 79% yield, η = 1.4 μL/mg; ^{1}H and ^{13}C NMR of the product **2sg** were in agreement with the literature[13]. **^{1}H NMR (400 MHz, $CDCl_3$)** δ 8.44-8.46 (m, 2H, Ar-H), 8.04 (d, J = 8.0 Hz, 2H, Ar-H), 7.36-7.41 (m, 2H, Ar-H), 7.01-7.04 (m, 2H, Ar-H); **^{13}C NMR (101 MHz, $CDCl_3$)** δ 150.1 (C), 137.1 (CH), 135.2 (C-Te), 131.3 (CH), 120.8 (CH).

1,2-di(thiophen-2-yl)ditellane(2sh)

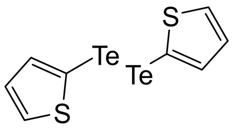

Prepared by **Conditions A.** Red soild, 23.3 mg, 55% yield, η = 1.4 µL/mg; ^{1}H and ^{13}C NMR of the product **2sg** were in agreement with the literature[30]. **^{1}H NMR (400 MHz, $CDCl_3$)** δ 7.39-7.47 (m, 4H, Ar-H), 6.91-6.97 (m, 4H, Ar-H); **^{13}C NMR (101 MHz, $CDCl_3$)** δ 141.7 (CH), 135.1 (CH), 128.4 (CH), 94.0 (C-Te).

***N*-phenyl-3-(phenylselanyl)propanamide (4a)**

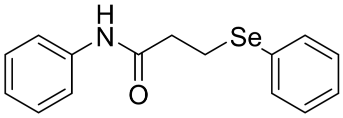

Prepared by **Conditions D**. White solid, 57.4 mg, 94% yield, η = 1.4 µL/mg, melting point 90 – 92°C; **^{1}H NMR (400 MHz, $CDCl_3$)** δ 7.52-7.54 (m, 2H, Ph-H), 7.48 (d, *J* = 7.8 Hz , 2H, Ph-H), 7.27-7.33 (m, 6H, Ph-H), 7.11 (t, *J* = 7.4 Hz, 1H, Ph-H), 3.23 (t, *J* = 7.2 Hz, 2H, CH_2), 2.73 (t, *J* = 7.2 Hz, 2H, CH_2); **^{13}C NMR (101 MHz, $CDCl_3$)** δ 169.7 (C=O), 137.7 (C), 133.1 (CH), 129.4 (CH), 129.3 (C-Se), 129.1 (CH), 127.4 (CH), 124.6 (CH), 120.0 (CH), 38.2 (CH_2-Se), 22.4 (CH_2); **^{77}Se NMR (76 MHz, $CDCl_3$)** δ 308.5; **IR (film)**: ν (cm^{-1}) 3302, 2955, 2924, 2852, 1661, 1545, 1443, 1377, 737, 691, 470; **HR-MS (ESI)** m/z calcd for $C_{15}H_{16}NOSe$, $[M+H]^+$: 306.03916, found 306.03908.

3-((4-chlorophenyl)selanyl)-*N*-phenylpropanamide(4b)

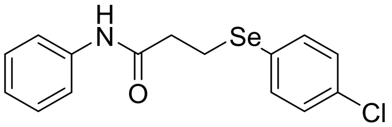

Prepared by **Conditions D.** Light yellow Solid, 64.2 mg, 95% yield, η = 1.3 µL/mg, melting point 95 – 96°C; **^{1}H NMR (400 MHz, $CDCl_3$)** δ 7.45 (t, *J* = 8.8 Hz, 4H, Ph-H), 7.31 (t, *J* = 7.8 Hz, 2H, Ph-H), 7.35 (s, 1H, NH), 7.23 (d, *J* = 8.4 Hz, 2H, Ph-H), 7.11 (t, *J* = 7.4 Hz, 1H, Ph-H), 3.20 (t, *J* = 7.2 Hz, 2H, CH_2), 2.72 (t, *J* = 7.2 Hz, 2H, CH_2); **^{13}C NMR (101 MHz, $CDCl_3$)** δ 169.5 (C=O), 137.6 (C), 134.4 (CH), 133.6 (C), 129.5 (CH), 129.1 (CH), 127.6 (C-Se), 124.6 (CH), 120.0 (CH), 38.1 (CH_2-Se), 22.7 (CH_2); **^{77}Se NMR (76 MHz, $CDCl_3$)** δ 308.7; **IR (film)**: ν (cm^{-1}) 3304, 2923, 1662, 1544, 1444, 1090, 1011, 813, 755, 693, 492; **HR-MS (ESI)** m/z calcd for $C_{15}H_{15}NOSeCl$, $[M+H]^+$: 340.00019, found 340.00059.

3-((4-(tert-butyl)phenyl)selanyl)-*N*-phenylpropanamide(4c)

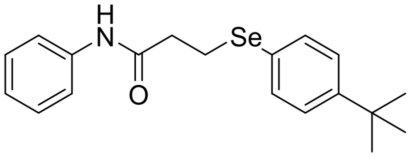

Prepared by **Conditions D**. Light yellow grease, 36.2 mg, 50 % yield, η = 1.2 µL/mg; **^{1}H NMR (400 MHz, $CDCl_3$)** δ 7.46-7.48 (m, 4H, Ph-H), 7.29-7.32 (m, 5H, Ph-H), 7.10 (t, *J* = 7.4 Hz, 1H, Ph-H), 3.20 (t, *J* = 7.2 Hz, 2H, CH_2), 2.73 (t, *J* = 7.2 Hz, 2H, CH_2), 1.31 (s, 9H, CH_3); **^{13}C NMR (101 MHz, $CDCl_3$)** δ 169.8 (C=O), 150.8 (C), 137.7 (C), 133.2 (CH), 129.1 (CH), 126.5

(CH), 125.5 (C-Se), 124.5 (CH), 120.0 (CH), 38.4 (CH_2), 34.7 (C), 31.4 (CH_2-Se), 22.5 (CH_2); **^{77}Se NMR (76 MHz, $CDCl_3$)** δ 298.0; **IR (film)**: ν (cm^{-1}) 3299, 2959, 2867, 1600, 1443, 1143, 1011, 754, 692; **HR-MS (ESI)** m/z calcd for $C_{19}H_{24}NOSe$, $[M+H]^+$: 362.10176, found: 362.10216.

3-((4-ethylphenyl)selanyl)-*N*-phenylpropanamide(4d)

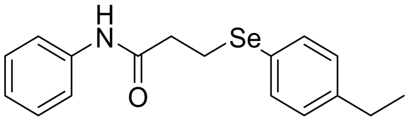

Prepared by **Conditions D**. Light yellow grease, 40.8mg, 61% yield, η = 1.3 μL/mg; **^{1}H NMR (400 MHz, $CDCl_3$)** δ 7.44-7.50 (m, 4H, Ph-H), 7.30 (t, J = 7.8 Hz, 3H, Ph-H), 7.10 (d, J = 8.0 Hz, 3H, Ph-H), 3.17 (t, J = 7.2 Hz, 2H, CH_2), 2.71 (t, J = 7.2 Hz, 2H, CH_2), 2.62 (q, J = 7.6 Hz, 2H, CH_2), 1.22 (t, J = 7.6 Hz, 3H, CH_3); **^{13}C NMR (101 MHz, $CDCl_3$)** δ 169.7 (C=O), 144.0 (C), 137.7 (C), 133.7 (CH), 129.1 (CH), 129.0 (CH), 125.6 (C-Se), 124.5 (CH), 119.9 (CH), 38.3 (CH_2-Se), 28.6 (CH_2), 22.6 (CH_2), 15.6 (CH_3); **^{77}Se NMR (76 MHz, $CDCl_3$)** δ 301.0; **IR (film): ν (cm^{-1})** 3302, 2963, 2928, 1661, 1545, 1443, 754, 693, 506; **HR-MS (ESI)** m/z calcd for $C_{17}H_{20}NOSe$, $[M+H]^+$: 334.07046, found 334.07045.

***N*-phenyl-3-((4-(trifluoromethoxy)phenyl)selanyl)propanamide(4e)**

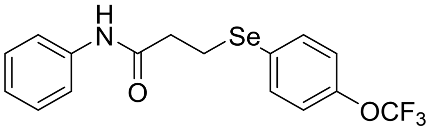

Prepared by **Conditions D.** Light yellow solid, 54.8 mg, 71 % yield, η = 1.1 μL/mg, melting point 88 – 94°C; **^{1}H NMR (400 MHz, $CDCl_3$)** δ 7.54 (d, J = 8.6 Hz, 2H, Ph-H), 7.47 (d, J = 7.9 Hz, 2H, Ph-H), 7.31 (t, J =7.6 Hz, 2H, Ph-H), 7.24 (s, 1H, NH), 7.11 (d, J = 7.8 Hz, 2H, Ph-H), 3.24 (t, J = 7.2 Hz, 2H, CH_2), 2.75 (t, J = 7.2 Hz, 2H, CH_2); **^{13}C NMR (101 MHz, $CDCl_3$)** δ 169.3 (C=O), 148.7 (C), 137.6 (C), 134.5 (CH), 129.2 (CH), 127.8 (C-Se), 124.7 (CH), 121.9 (CH), 120.0 (CH), 119.2 (OCF_3), 38.2 (CH_2-Se), 22.8 (CH_2); **^{77}Se NMR (76 MHz, $CDCl_3$)** δ 309.5; **^{19}F NMR (376 MHz, $CDCl_3$)** δ -57.8; **IR (film)**: ν (cm^{-1}) 3328, 2926, 2253, 1660, 1258, 904, 727, 650, 499; **HR-MS (ESI)** m/z calcd for $C_{16}H_{15}NO_2Se\ F_3$, $[M+H]^+$: 390.02146, found 390.02165.

***N*-phenyl-3-((4-(trimethylsilyl)phenyl)selanyl)propanamide(4f)**

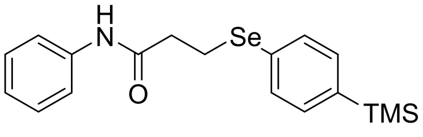

Prepared by **Conditions D.** Light yellow grease, 54.5 mg, 72% yield, η = 1.3 μL/mg; **^{1}H NMR (400 MHz, $CDCl_3$)** δ 7.41-7.51 (m, 6H, Ph-H), 7.29 - 7.33 (m, 3H, Ph-H), 7.11 (t, J = 7.4 Hz, 1H, Ph-H), 3.24 (t, J = 7.2 Hz, 2H, CH_2), 2.75 (t, J = 7.2 Hz, 2H, CH_2), 0.26 (s, 9H, CH_3); **^{13}C NMR (101 MHz, $CDCl_3$)** δ 169.6 (C=O), 139.5 (C), 137.7 (C), 134.3 (CH), 132.0 (CH), 130.4 (C), 129.1 (CH), 124.5 (CH), 120.0 (CH), 38.3 (CH_2-Se), 22.0 (CH_2), -1.1 (CH_3); **^{77}Se NMR (76 MHz, $CDCl_3$)** δ 306.8; **IR (film)**: ν (cm^{-1}) 3301, 3058, 2925, 1545 , 1249, 841, 754, 692, 495; **HR-MS (ESI)** m/z calcd for $C_{18}H_{24}NOSeSi$, $[M+H]^+$: 378.07869, found 378.07697.

tert-butyl-4-((3-oxo-3-(phenylamino)propyl)selanyl)benzoate(4g)

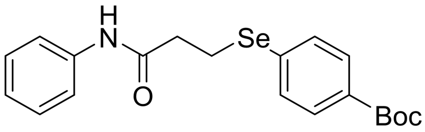

Prepared by **Conditions D.** Light yellow grease, 55.2 mg, 68% yield, η = 1.1 μL/mg; **^{1}H NMR (400 MHz, $CDCl_3$)** δ 7.83 (d, J = 8.4 Hz, 2H, Ph-H), 7.47(t, J = 7.9 Hz, 4H, Ph-H), 7.37 (s, 1H, NH), 7.29 (t, J = 8.4 Hz, 2H, Ph-H), 7.09 (t, J = 7.4 Hz, 1H, Ph-H), 3.27 (t, J = 7.2 Hz, 2H, CH_2), 2.75 (t, J = 7.2 Hz, 2H, CH_2), 1.57 (s, 9H, CH_3); **^{13}C NMR (101 MHz, $CDCl_3$)** δ 169.4 (C=O), 165.6 (C=O), 137.6 (C), 136.2 (C), 131.0 (CH), 130.4 (C-Se), 130.1 (CH), 129.1 (CH), 124.6 (CH), 120.0 (CH), 81.3 (C), 37.9 (CH_2-Se), 28.3 (CH_3), 21.7 (CH_2); **^{77}Se NMR (76 MHz, $CDCl_3$)** δ 315.4; **IR (film)**: ν (cm^{-1}) 3317, 2926, 1709, 1295, 1164, 1200, 756; **HR-MS (ESI)** m/z calcd for $C_{20}H_{23}NO_3SeNa$, $[M+Na]^+$: 428.07354 , found 428.07388.

_N_-phenyl-3-(p-tolylselanyl)propanamide(4h)

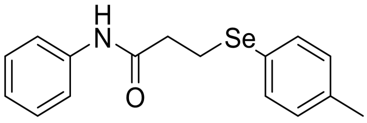

Prepared by **Conditions D.** White solid, 50.0 mg, 79% yield, η = 1.4 μL/mg, melting point 68 – 70°C; **^{1}H NMR (400 MHz, $CDCl_3$)** δ 7.45 (dd, J = 14.8, 7.6 Hz, 4H, Ph-H), 7.31 (t, J = 8.0 Hz, 3H, Ph-H), 7.10 (t, J = 7.6 Hz, 3H, Ph-H), 3.18 (t, J = 7.0 Hz, 2H, CH_2), 2.70 (t, J = 7.2 Hz, 2H, CH_2), 2.33 (s, 3H, CH_3); **^{13}C NMR (101 MHz, $CDCl_3$)** δ 169.7 (C=O), 137.7 (C), 137.6 (C), 133.7 (CH), 130.2 (CH), 129.1 (CH), 125.3 (C-Se), 124.5 (CH), 120.0 (CH), 38.2 (CH_2-Se), 22.6 (CH_3), 21.2 (CH_2); **^{77}Se NMR (76 MHz, $CDCl_3$)** δ 300.8; **IR (film)**: ν (cm^{-1}) 3305, 2956, 2924, 1663, 1544, 1444, 754, 693; **HR-MS (ESI)** m/z calcd for $C_{16}H_{18}NOSe$, $[M+H]^+$: 320.05481, found: 320.05539.

3-((2-fluorophenyl)selanyl)-_N_-phenylpropanamide(4i)

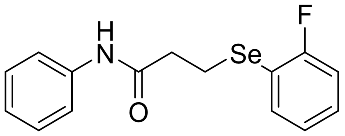

Prepared by **Conditions D.** Light yellow grease, 53.6 mg, 83% yield, η = 1.3 μL/mg; **^{1}H NMR (400 MHz, $CDCl_3$)** δ 7.47-7.56 (m, 4H, Ph-H), 7.24-7.32 (m, 3H, Ph-H), 7.04-7.12 (m, 3H, Ph-H), 3.21 (t, J = 7.2 Hz, 2H, CH_2), 2.73 (t, J = 7.2 Hz, 2H, CH_2); **^{13}C NMR (101 MHz, $CDCl_3$)** δ 169.7 (C=O), 162.2 (d, J = 242.8 Hz, C), 137.7 (C), 135.0 (d, J = 2.0 Hz, C-Se), 129.7 (d, J = 7.7 Hz, CH), 129.1 (CH), 125.0 (d, J = 3.4 Hz, CH), 124.5 (CH), 120.1 (CH), 116.0 (d, J = 22.7 Hz, CH), 115.7 (d, J = 23.8 Hz, CH), 38.2 (CH_2-Se), 21.9 (CH_2); **^{77}Se NMR (76 MHz, $CDCl_3$)** δ 234.9; **^{19}F NMR (376 MHz, $CDCl_3$)** δ -114.0; **IR (film)**: ν (cm^{-1}) 3303, 2920, 1665, 1499, 1444, 1227, 825, 756, 506; **HR-MS (ESI)** m/z calcd for $C_{15}H_{14}NOSeFK$, $[M+K]^+$: 361.98562, found: 361.98437

3-((2-chlorophenyl)selanyl)-_N_-phenylpropanamide(4j)

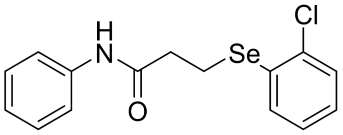 Prepared by **Conditions D**. Light yellow solid, 57.3 mg, 85% yield, η = 1.3 µL/mg, melting point 88 – 91°C; **^{1}H NMR (400 MHz, $CDCl_3$)** δ 7.64 (s, 1H, NH), 7.48 (d, J = 8.0 Hz, 2H, Ph-H), 7.41-7.43 (m, 1H, Ph-H), 7.34-7.37 (m, 1H, Ph-H), 7.29 (t, J = 8.0 Hz, 2H, Ph-H), 7.15-7.17 (m, 2H, Ph-H), 7.10 (t, J = 7.4 Hz, 1H, Ph-H), 3.25 (t, J = 7.2 Hz, 2H, CH_2), 2.77 (t, J = 7.2 Hz, 2H, CH_2); **^{13}C NMR (101 MHz, $CDCl_3$)** δ 169.7 (C=O), 137.6 (C), 135.6 (C), 131.5 (CH), 130.5 (C-Se), 129.8 (CH), 129.1 (CH), 127.8 (CH), 127.5 (CH), 124.6 (CH), 120.2 (CH), 37.6 (CH_2-Se), 21.2 (CH_2); **^{77}Se NMR (76 MHz, $CDCl_3$)** δ 299.5; **IR (film)**: ν (cm^{-1}) 3301, 3138, 2925, 2854, 1660, 1444, 1251, 1025, 903, 749, 539, 504; **HR-MS (ESI)** m/z calcd for $C_{15}H_{16}NOSeCl$, $[M+H]^+$: 340.00019, found: 340.00020.

3-((2-(tert-butyl)phenyl)selanyl)-*N*-phenylpropanamide(4k)

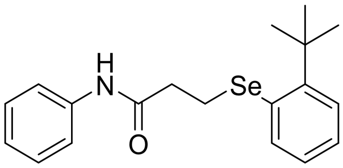 Prepared by **Conditions D**. Light yellow grease, 39.9 mg, 55% yield, η = 1.2 µL/mg; **^{1}H NMR (400 MHz, $CDCl_3$)** δ 7.56 (dd, J = 8.0, 1.2 Hz, 1H, Ph-H), 7.48 (d, J = 7.6 Hz, 3H, Ph-H), 7.41 (dd, J = 8.0, 1.6 Hz, 1H, Ph-H), 7.30 (t, J = 7.8 Hz, 2H, Ph-H), 7.19 (td, J = 7.2, 1.6 Hz, 1H, Ph-H), 7.08-7.13 (m, 2H, Ph-H), 3.27 (t, J = 7.6 Hz, 2H, CH_2), 2.75 (t, J = 7.6 Hz, 2H, CH_2), 1.50 (s, 9H, CH_3); **^{13}C NMR (101 MHz, $CDCl_3$)** δ 169.8 (C=O), 151.1 (C), 137.7 (C), 134.7 (CH), 129.4 (C-Se), 129.1(CH), 127.0 (CH), 126.8 (CH), 126.7 (CH), 124.5 (CH), 120.1 (CH), 37.6 (C), 37.0 (CH_2-Se), 30.8 (CH_3), 24.1 (CH_2); **^{77}Se NMR (76 MHz, $CDCl_3$)** δ 299.2; **IR (film)**: ν (cm^{-1}) 3296, 3139, 2954, 2868, 1657, 1442, 1247, 752, 692, 504; **HR-MS (ESI)** m/z calcd for $C_{19}H_{23}NOSeNa$, $[M+Na]^+$: 384.08371, found: 384.08538.

3-((3-methoxyphenyl)selanyl)-*N*-phenylpropanamide(4l)

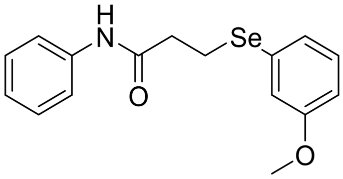 Prepared by **Conditions D.** Light yellow solid, 58.9 mg, 88% yield, η = 1.3 µL/mg; **^{1}H NMR (400 MHz, $CDCl_3$)** δ 7.53 (s, 1H, Ph-H), 7.48 (d, J = 8.0 Hz, 2H, Ph-H), 7.29 (t, J = 8.0 Hz, 2H, Ph-H), 7.18 (t, J = 8.0 Hz, 1H, Ph-H), 7.06-7.11(m, 3H, Ph-H), 6.79 (dd, J = 8.4, 2.4 Hz, 2H, Ph-H), 3.77 (s, 3H, CH_3), 3.21 (t, J = 7.2 Hz, 2H, CH_2), 2.73 (t, J = 7.2 Hz, 2H, CH_2); **^{13}C NMR (101 MHz, $CDCl_3$)** δ 169.8 (C=O), 159.9 (C), 137.7 (C), 130.5 (C-Se), 130.1 (CH), 129.0 (CH), 124.9 (CH), 124.5 (CH), 120.1 (CH), 118.2 (CH), 113.0 (CH), 55.4 (CH_3), 38.1 (CH_2-Se), 22.3 (CH_2); **^{77}Se**

NMR (76 MHz, CDCl₃) δ 314.3; **IR (film)**: ν (cm^{-1}) 3302, 2922, 1666, 1588, 1463, 1377, 1246, 1031, 756, 691; **HR-MS (ESI)** m/z calcd for $C_{16}H_{18}NO_2Se$, $[M+H]^+$: 336.04973, found 336.04983.

***N*-phenyl-3-(m-tolylselanyl)propanamide(4m)**

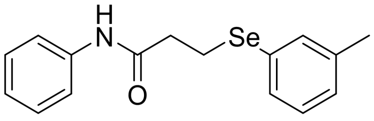

Prepared by **Conditions D.** Light yellow solid, 50.2 mg, 79% yield, η = 1.4 µL/mg, melting point 63 – 68°C; **^{1}H NMR (400 MHz, $CDCl_3$)** δ 7.48 (d, *J* = 7.8 Hz, 2H, Ph-H), 7.26-7.38 (m, 5H, Ph-H), 7.17 (t, *J* = 7.6 Hz, 1H, Ph-H), 7.09 (q, *J* = 7.6 Hz, 2H, Ph-H), 3.21 (t, *J* = 7.2 Hz, 2H, CH_2), 2.73 (t, *J* = 7.2 Hz, 2H, CH_2), 2.32 (s, 3H, CH_3); **^{13}C NMR (101 MHz, $CDCl_3$)** δ 169.8 (C=O), 139.2 (C), 137.7 (C), 133.7 (CH), 130.0 (CH), 129.1 (C-Se), 129.2 (CH), 129.1 (CH), 128.3 (CH), 124.5 (CH), 120.0 (CH), 38.3 (CH_2-Se), 22.3 (CH_2), 21.4 (CH_3); **^{77}Se NMR (76 MHz, $CDCl_3$)** δ 304.8; **IR (film)**: ν (cm^{-1}) 3301, 2922, 1661, 1545, 1443, 1358, 1252, 755, 690, 505; **HR-MS (ESI)** m/z calcd for $C_{16}H_{18}NOSe$, $[M+H]^+$: 320.05481, found: 320.05560.

3-((2,4-difluorophenyl)selanyl)-*N*-phenylpropanamide(4n)

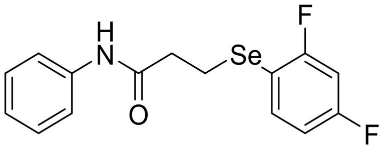

Prepared by **Conditions D**. White solid, 50.9 mg, 75% yield, η = 1.3 µL/mg, melting point 68 – 70°C; **^{1}H NMR (400 MHz, $CDCl_3$)** δ 7.46 - 7.55 (m, 4H, Ph-H), 7.30 (t, *J* = 7.6 Hz, 2H, Ph-H), 7.10 (t, *J* = 7.4 Hz, 1H, Ph-H), 6.79 - 6.85 (m, 2H) , Ph-H, 3.16 (t, *J* = 7.2 Hz, 2H, CH_2), 2.72 (t, *J* = 7.2 Hz, 2H, CH_2); **^{13}C NMR (101 MHz, $CDCl_3$)** δ 169.6 (C=O), 164.3 (dd, *J* = 53.7, 11.8 Hz, C), 161.8 (dd, *J* = 49.3, 11.5 Hz, C), 137.6 (C), 136.9 (dd, *J* = 9.1, 3.3 Hz, CH), 129.1 (CH), 124.6 (CH), 120.1 (CH), 112.3 (dd, *J* = 21.2, 3.1 Hz, CH), 110.8 (d, *J* = 23.2 Hz, C-Se), 101.4 (t, *J* = 26.8 Hz, CH), 38.3 (CH_2-Se), 22.6 (CH_2); **^{77}Se NMR (76 MHz, $CDCl_3$)** δ 235.7; **^{19}F NMR (376 MHz, $CDCl_3$)** δ -97.5 (q, *J* = 8.4 Hz), -109.2 (t, *J*= 7.6 Hz).; **IR (film)**: ν (cm^{-1}) 3302, 2925, 1662, 1597, 1481, 1263, 1138, 964, 755, 693, 594; **HR-MS (ESI)** m/z calcd for $C_{15}H_{14}NOSeF_2$, $[M+H]^+$: 342.02032, found: 342.02050.

3-((2,4-dichlorophenyl)selanyl)-*N*-phenylpropanamide(4o)

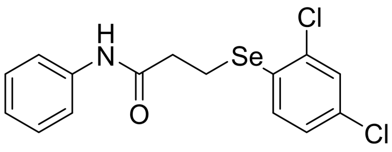

Prepared by **Conditions D**. Light yellow solid, 51.6 mg, 69% yield, η = 1.2 µL/mg, melting point 85 – 87°C; **^{1}H NMR (400 MHz, $CDCl_3$)** δ 7.49 (d, *J* = 8.0 Hz, 2H, Ph-H), 7.25-7.39 (m, 6H, Ph-H), 7.10-7.15 (m, 2H, Ph-H), 3.30 (t, *J* = 7.2 Hz, 2H, CH_2), 2.82 (t, *J* = 7.2 Hz, 2H, CH_2); **^{13}C NMR (101 MHz, $CDCl_3$)** δ 169.1 (C=O), 137.6 (C), 133.7 (C), 133.3 (C), 132.7 (C-Se), 130.6 (CH), 129.2 (CH), 127.7 (CH), 124.7 (CH), 120.0 (CH), 108.4 (CH), 37.5 (CH_2-Se), 21.4 (CH_2);

^{77}Se NMR (76 MHz, CDCl$_3$) δ 314.4; **IR (film)**: ν (cm^{-1}) 3300, 3061, 2920, 1660, 1544, 1443, 1027, 806, 753, 692, 565; **HR-MS (ESI)** m/z calcd for $C_{15}H_{14}NOSeCl_2$, [M+H]$^+$: 373.96122, found: 373.96068.

3-((2,5-dichlorophenyl)selanyl)-*N*-phenylpropanamide(4p)

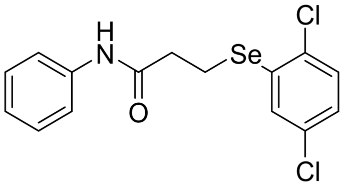

Prepared by **Conditions D**. Light yellow solid, 47.5 mg, 64% yield, η = 1.2 µL/mg, melting point 83 – 86°C; **^{1}H NMR (400 MHz, CDCl$_3$)** δ 7.47 (d, *J* = 7.6 Hz, 2H, Ph-H), 7.38-7.40 (m, 2H, Ph-H), 7.32 (t, *J*= 7.6 Hz, 2H, Ph-H), 7.25 (s, 1H, Ph-H), 7.17 (dd, *J* = 8.4, 2.4 Hz, 1H, Ph-H), 7.12 (t, *J* = 7.4 Hz, 1H, Ph-H), 3.28 (t, *J* = 7.2 Hz, 2H, CH$_2$), 2.78 (t, *J* = 7.2 Hz, 2H, CH$_2$); **^{13}C NMR (101 MHz, CDCl$_3$)** δ 169.2 (C=O), 137.6 (C), 136.7 (C), 133.4 (C), 132.8 (CH), 129.6 (CH), 129.2 (CH), 128.9 (C-Se), 127.8 (CH), 124.7 (CH), 120.0 (CH), 37.8 (CH$_2$-Se), 21.6 (CH$_2$); **^{77}Se NMR (76 MHz, CDCl$_3$)** δ 302.7; **IR (film)**: ν (cm^{-1}) 3300, 3061, 2923, 2852, 1659, 1544, 1444, 1249, 1096, 1024, 906, 732, 692, 505, 432; **HR-MS (ESI)** m/z calcd for $C_{15}H_{14}NOSeCl_2$, [M+H]$^+$: 373.96122, found: 373.96063.

3-((3,4-dichlorophenyl)selanyl)-*N*-phenylpropanamide(4q)

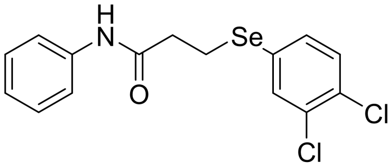

Prepared by Conditions **D**. Light yellow solid, 65.8 mg, 88% yield, η = 1.2 µL/mg, melting point 74 – 77°C; **^{1}H NMR (400 MHz, CDCl$_3$)** δ 7.59 (s, 1H, NH), 7.47 (d, *J* = 7.8 Hz, 2H, Ph-H), 7.29-7.35 (m, 5H, Ph-H), 7.11 (t, *J* = 7.8 Hz, 1H, Ph-H), 3.23 (t, *J* = 7.2 Hz, 2H, CH$_2$), 2.75 (t, *J* = 7.2 Hz, 2H, CH$_2$); **^{13}C NMR (101 MHz, CDCl$_3$)** δ 167.9 (C=O), 137.6 (C), 134.2 (CH), 133.1 (C), 132.1 (CH), 131.7 (C), 131.0 (CH), 129.3 (C-Se), 129.2 (CH), 125.1 (CH), 119.0 (CH), 37.6 (CH$_2$-Se), 22.2 (CH$_2$); **^{77}Se NMR (76 MHz, CDCl$_3$)** δ 320.6; **IR (film)**: ν (cm-1) 3301, 3060, 2924, 2851, 1661, 1546, 1444, 1360, 1252, 1136, 1029, 754, 693, 504; **HR-MS (ESI)** m/z calcd for $C_{15}H_{14}NOSeCl_2$, [M+H]+: 373.96122, found: 373.95691.

3-((4-chloro-3-(4-ethoxybenzyl)phenyl)selanyl)-*N*-phenylpropanamide(4r)

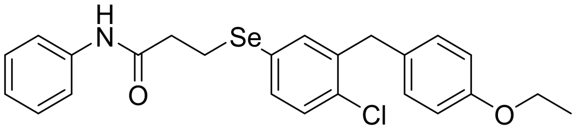

Prepared by **Conditions D.** White solid, 73.2 mg, 77% yield, η = 1.0 µL/mg, melting point 88 – 91°C; **^{1}H NMR (400 MHz, CDCl$_3$)** δ 7.45 (d, *J* = 7.8 Hz, 2H, Ph-H), 7.24 -7.33 (m, 5H, Ph-H), 7.17 (s, 1H, Ph-H), 7.07-7.13 (m, 3H, Ph-H), 6.82 (d, *J* = 8.8 Hz, 2H, Ph-H), 3.94-4.00 (m, 4H, CH$_2$), 3.15 (t, *J* = 7.2 Hz, 2H, CH$_2$), 2.66 (t, *J* = 7.2 Hz, 2H, CH$_2$), 1.38 (t, *J* =

7.0 Hz, 3H, CH_3); **^{13}C NMR (101 MHz, $CDCl_3$)** δ 169.4 (C=O), 157.6 (C), 140.3 (C), 137.6 (C), 135.4 (CH), 133.6 (C), 132.1 (CH), 131.0 (C), 130.3 (CH), 130.0 (CH), 129.1 (CH), 127.8 (C-Se), 124.6 (CH), 120.0 (CH), 114.6 (CH), 63.5 (CH_2), 38.3 (CH_2), 38.1 (CH_2-Se), 22.6 (CH_2), 15.0 (CH_3); **^{77}Se NMR (76 MHz, $CDCl_3$)** δ 309.2; **IR (film)**: ν (cm^{-1}) 3303, 2923, 1663, 1510, 1443, 1245, 1043, 755, 507; **HR-MS (ESI)** m/z calcd for $C_{24}H_{25}NO_2SeCl$, $[M+H]^+$: 474.07336, found: 474.07337.

***N*-phenyl-3-(thiophen-2-ylselanyl)propanamide(4s)**

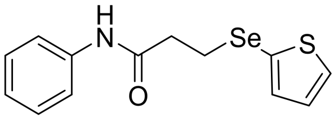

Prepared by **Conditions D.** White solid, 55.4 mg, 89% yield, η = 1.4 μL/mg, melting point 71 – 74°C; **^{1}H NMR (400 MHz, $CDCl_3$)** δ 7.50 (d, *J* = 7.8 Hz, 2H, Ph-H), 7.40 (d, *J* = 6.0 Hz, 1H, Ar-H), 7.32 (t, *J* = 7.8 Hz, 3H, Ph-H), 7.23 (d, *J* = 3.2 Hz, 1H, Ar-H), 7.11 (t, *J* = 7.4 Hz, 1H, Ph-H), 7.00 (dd, *J* = 5.2, 3.2 Hz, 1H, Ar-H), 3.10 (t, *J* = 7.2 Hz, 2H, CH_2), 2.74 (t, *J* = 7.2 Hz, 2H, CH_2); **^{13}C NMR (101 MHz, $CDCl_3$)** δ 169.5 (C=O), 137.7 (C), 136.4 (CH), 131.4 (CH), 129.2 (CH), 128.4 (CH), 124.6 (CH), 122.9 (C-Se), 120.0 (CH), 38.1 (CH_2-Se), 25.9 (CH_2); **^{77}Se NMR (76 MHz, $CDCl_3$)** δ 224.8 ; **IR (film)**: ν (cm^{-1}) 3299, 3064, 2924, 1660, 1544, 1443, 1309, 962, 754, 693, 504; **HR-MS (ESI)** m/z calcd for $C_{13}H_{14}NOSeS$, $[M+H]^+$: 311.99558, found: 311.99569.

***N*-phenyl-3-(thiophen-3-ylselanyl)propanamide(4t)**

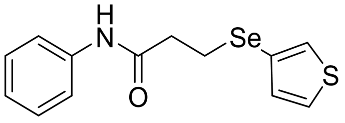

Prepared by **Conditions D.** White solid, 44.8 mg, 72% yield, η = 1.4 μL/mg, melting point 76 – 78°C; **^{1}H NMR (400 MHz, $CDCl_3$)** δ 7.49 (d, *J* = 7.8 Hz, 2H, Ph-H), 7.29-7.36 (m, 5H, Ph-H), 7.09-7.13 (m, 2H, Ph-H), 3.14 (t, *J* = 7.2 Hz, 2H, CH_2), 2.71 (t, *J* = 7.2 Hz, 2H, CH_2); **^{13}C NMR (101 MHz, $CDCl_3$)** δ 169.3 (C=O), 138.5 (C), 132.4 (CH), 129.2 (CH), 128.0 (CH), 126.9 (CH), 124.6 (CH), 122.1 (C-Se), 119.9 (CH), 38.4 (CH_2-Se), 23.1 (CH_2); **^{77}Se NMR (76 MHz, $CDCl_3$)** δ 233.8 ; **IR (film)**: ν (cm^{-1}) 3300, 3098, 2923, 1661, 1545, 1443, 1252, 755, 692, 603, 504; **HR-MS (ESI)** m/z calcd for $C_{13}H_{13}NOSeSNa$, $[M+Na]^+$: 333.97753, found: 333.97757.

3-(naphthalen-2-ylselanyl)-*N*-phenylpropanamide(4u)

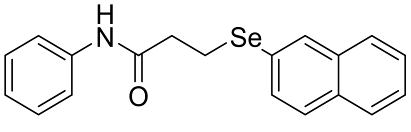

Prepared by **Conditions D.** White solid, 55.7 mg, 79% yield, η = 1.2 μL/mg, melting point 76 – 79°C; **^{1}H NMR (400 MHz, $CDCl_3$)** δ 8.01 (s, 1H, Ph-H), 7.80-7.82 (m, 1H, Ph-H), 7.75 (d, *J* = 8.0 Hz, 2H, Ph-H), 7.59 (dd, *J* = 8.8, 1.6 Hz, 2H, Ph-H), 7.44 - 7.52 (m, 4H, Ph-H), 7.29 (t, *J* = 7.8 Hz, 2H, Ph-H), 7.16 (s, 1H, NH), 7.10 (t, *J* = 7.4 Hz, 1H, Ph-H), 3.32 (t, *J* = 7.2 Hz, 2H, CH_2), 2.75 (t, *J* = 7.2 Hz, 2H); **^{13}C NMR (101 MHz, $CDCl_3$)** δ 169.6 (C=O), 137.7 (C), 134.0 (C), 132.5 (C), 131.9 (CH), 130.4 (CH), 129.1 (CH), 128.8 (CH), 127.9 (CH), 127.3 (CH), 126.8 (CH),

126.7 (CH), 126.3 (CH), 124.5 (C-Se), 119.9 (CH), 38.2 (CH_2-Se), 22.3 (CH_2); **^{77}Se NMR (76 MHz, $CDCl_3$)** δ 310.5; **IR (film)**: ν (cm^{-1}) 3302, 3054, 2921, 1665, 1499, 1444, 1377, 754, 474; **HR-MS (ESI)** m/z calcd for $C_{19}H_{17}NOSeNa$, $[M+Na]^+$: 378.03676, found: 378.03696.

3-(benzofuran-2-ylselanyl)-*N*-phenylpropanamide(4v)

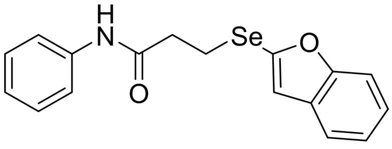

Prepared by **Conditions D.** White solid, 43.5 mg, 63% yield, η = 1.3 μL/mg, melting point 63 – 67°C; **^{1}H NMR (400 MHz, $CDCl_3$)** δ 7.45-7.52 (m, 5H, Ph-H), 7.20-7.32 (m, 4H, Ph-H), 7.11 (t, *J* = 7.4 Hz, 1H, Ph-H), 6.92 (s, 1H, Ph-H), 3.24 (t, *J* = 7.0 Hz, 2H, CH_2), 2.81 (t, *J* = 7.0 Hz, 2H, CH_2); **^{13}C NMR (101 MHz, $CDCl_3$)** δ 169.4 (C=O), 157.4 (C), 143.4 (C-Se), 137.6 (C), 129.1 (CH), 128.7 (C), 124.6 (CH), 124.5 (CH), 123.1 (CH), 120.5 (CH), 120.0 (CH), 114.8 (CH), 111.1 (CH), 38.5 (CH_2-Se), 23.2 (CH_2); **^{77}Se NMR** (76 MHz, $CDCl_3$) δ 242.2; **IR (film)**: ν (cm^{-1}) 3298, 3136, 2924, 1658, 1441, 1249, 748, 692, 504; **HR-MS (ESI)** m/z calcd for $C_{17}H_{15}NO_2SeNa$, $[M+Na]^+$: 368.01602, found: 368.01723.

3-((1-methyl-1*H*-indol-2-yl)selanyl)-*N*-phenylpropanamide(4w)

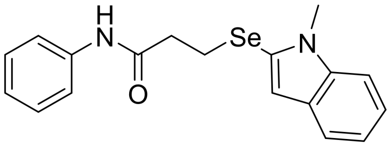

Prepared by **Conditions D.** Yellow solid, 39.9 mg, 56% yield, η = 1.2 μL/mg, melting point 65 – 70°C; **^{1}H NMR (400 MHz, $CDCl_3$)** δ 7.58 (d, *J* = 8.0 Hz, 1H, Ph-H), 7.42 (d, *J* = 8.0 Hz, 2H, Ph-H), 7.22-7.32 (m, 5H, Ph-H), 7.08-7.16 (m, 3H, Ph-H), 6.78 (s, 1H, Ar-H), 3.84 (s, 3H, CH_3), 3.05 (t, *J* = 7.2 Hz, 2H, CH_2), 2.66 (t, *J* = 7.2 Hz, 2H, CH_2); **^{13}C NMR (101 MHz, $CDCl_3$)** δ 169.4 (C=O), 138.7 (C), 137.6 (C), 129.1 (CH), 127.9 (C-Se), 124.5 (CH), 122.5 (CH), 124.4 (C), 120.3 (CH), 119.9 (CH), 119.8 (CH), 111.4 (CH), 109.9 (CH), 37.7 (CH_2-Se), 31.5 (CH_3), 23.2 (CH_2); **^{77}Se NMR (76 MHz, $CDCl_3$)** δ 175.7; **IR (film)**: ν (cm^{-1}) 3302, 3056, 2922, 2852, 1662, 1443, 1251, 750, 693; **HR-MS (ESI)** m/z calcd for $C_{18}H_{19}N_2OSe$, $[M+H]^+$: 359.06571, found: 359.06511.

3-(butylselanyl)-*N*-phenylpropanamide(4x)

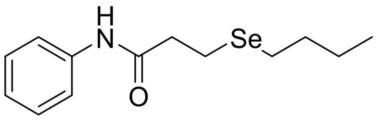

Prepared by **Conditions E.** Light yellow solid, 69.2 mg, 49% yield, η = 1.3 μL/mg, melting point 80 – 83°C; **^{1}H NMR (400 MHz, $CDCl_3$)** δ 7.61 (s, 1H, NH), 7.52 (d, *J* = 7.6 Hz, 2H, Ph-H), 7.31 (t, *J* = 8.0 Hz, 2H, Ph-H), 7.10 (t, *J* = 7.4 Hz, 1H, Ph-H), 2.89 (t, *J* = 7.0 Hz, 2H, CH_2), 2.74 (t, *J* = 7.1 Hz, 2H, CH_2), 2.62 (t, *J* = 7.0 Hz, 2H, CH_2), 1.61-1.69 (m, 2H, CH_2), 1.34-1.44 (m, 2H, CH_2), 0.90 (t, *J* = 7.4 Hz, 3H, CH_3); **^{13}C NMR (101 MHz, $CDCl_3$)** δ 170.0 (C=O), 137.8 (C), 129.1 (CH), 124.5 (CH), 120.0 (CH), 38.9 (CH_2), 32.7 (CH_2-Se), 24.6 (CH_2-Se), 23.1 (CH_2), 18.4 (CH_2), 13.7

(CH_3); **^{77}Se NMR (76 MHz, $CDCl_3$)** δ 175.2; **IR (film)**: ν (cm^{-1}) 3298, 2957, 2927, 1660, 1544, 1442, 1252, 754, 693, 504; **HR-MS (ESI)** m/z calcd for $C_{13}H_{20}NOSe$, $[M+H]^+$: 286.07046, found: 286.07122.

3-(hex-5-en-1-ylselanyl)-*N*-phenylpropanamide(4y)

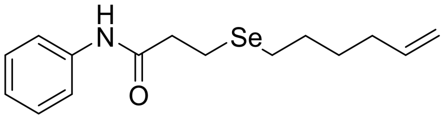

Prepared by **Conditions F.** Light yellow grease, 32.1 mg, 52% yield, η = 1.6 μL/mg; **^{1}H NMR (400 MHz, $CDCl_3$)** δ 7.51-7.55 (m, 3H, Ph-H), 7.32 (t, *J* = 8.0 Hz, 2H, Ph-H), 7.11 (t, *J* = 7.4 Hz, 1H, Ph-H), 5.73-5.83 (m,1H, CH), 4.93-5.02 (m, 2H, CH_2), 2.89 (t, *J* = 7.2 Hz, 2H, CH_2), 2.74 (t, *J* = 7.2 Hz, 2H, CH_2), 2.62 (t, *J* = 7.6 Hz, 2H, CH_2), 2.05 (q, *J* = 7.8 Hz, 2H, CH_2), 1.64-1.72 (m, 2H, CH_2), 1.43-1.51 (m, 2H, CH_2); **^{13}C NMR (101 MHz, $CDCl_3$)** δ 170.0 (C=O), 139.6 (CH), 137.8 (C), 129.1 (CH), 124.5 (CH), 120.0 (CH), 114.8 (CH_2), 39.0 (CH_2), 33.3 (CH_2), 30.1 (CH_2-Se), 29.2 (CH_2-Se), 24.7 (CH_2), 18.5 (CH_2); **^{77}Se NMR (76 MHz, $CDCl_3$)** δ 176.2; **IR (film)**: ν (cm^{-1}) 3300, 3075, 2925, 2854, 1661, 1545, 1443, 1252, 754, 693; **HR-MS (ESI)** m/z calcd for $C_{15}H_{22}NOSe$, $[M+H]^+$: 312.08611, found: 312.08572.

3-(benzylselanyl)-*N*-phenylpropanamide(4z)

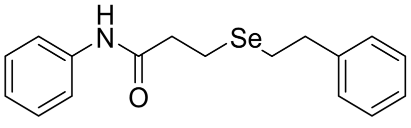

Prepared by **Conditions F.** Light yellow grease, 17.5 mg, 26% yield, η = 1.3 μL/mg; **^{1}H NMR (400 MHz, $CDCl_3$)** δ 7.50 (d, *J* = 8.0 Hz, 2H, Ph-H), 7.28-7.34 (m, 5H, Ph-H), 7.19-7.24 (m, 3H, Ph-H), 7.11 (t, *J* = 7.4 Hz, 1H, Ph-H), 2.99 (t, *J* = 7.4 Hz, 2H, CH_2), 2.86-2.90 (m, 4H, CH_2), 2.68 (t, *J* = 7.2 Hz, 2H, CH_2); **^{13}C NMR (101 MHz, $CDCl_3$)** δ 169.8 (C=O), 141.1 (C), 137.8 (C-Se), 129.1 (CH), 128.7 (CH), 128.6 (CH), 126.5 (CH), 124.5 (CH), 120.0 (CH), 39.0 (CH_2), 37.2 (CH_2-Se), 25.9 (CH_2-Se), 18.8 (CH_2); **^{77}Se NMR (76 MHz, $CDCl_3$)** δ 186.2; **IR (film)**: ν (cm^{-1}) 3300, 3027, 2921, 2850, 1660, 1443, 1252, 753, 696; **HR-MS (ESI)** m/z calcd for $C_{17}H_{20}NOSe$, $[M+H]^+$: 334.07046, found: 334.07107.

***N*-(4-methoxyphenyl)-3-(phenylselanyl)propanamide(5)**

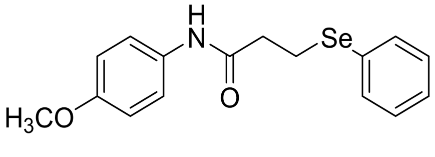

Prepared by **Conditions D.** Brown solid, 48.8 mg, 73% yield, η = 1.4 μL/mg, melting point 63 – 69°C; **^{1}H NMR (400 MHz, $CDCl_3$)** δ 7.51-7.54 (m, 2H, Ph-H), 7.37 (d, *J* = 9.0 Hz, 2H, Ph-H), 7.27-7.30 (m, 3H, Ph-H), 7.15 (s, 1H, NH), 6.84 (d, *J* = 9.0 Hz, 2H, Ph-H), 3.78 (s, 3H, CH_3), 3.23 (t, *J* = 7.2 Hz, 2H, CH_2), 2.71 (t, *J* = 7.2 Hz, 2H, CH_2); **^{13}C NMR (101 MHz, $CDCl_3$)** δ 169.5 (C=O), 165.1 (C), 156.6 (C), 133.1 (CH), 130.8 (C-Se), 129.4 (CH), 127.4 (CH), 121.9 (CH), 144.3 (CH), 55.6 (CH_3), 38.1 (CH_2-Se), 22.5 (CH_2); **^{77}Se NMR (76 MHz, $CDCl_3$)** δ 307.3; **IR (film)**: ν

(cm-1) 3325, 2916, 2838, 1770, 1510, 1236, 1168, 1033, 820, 735, 514; **HR-MS (ESI)** Calcd for $C_{16}H_{18}NOSe$, $[M+H]^+$: 336.04973, found: 336.04945.

***tert*-butyl 4-(3-(phenylselanyl)propanamido)benzoate(6)**

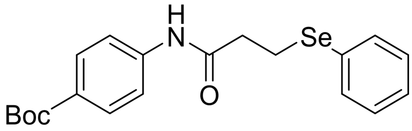

Prepared by **Conditions D.** Light yellow grease, 58.3 mg, 72 % yield, η = 1.2 μL/mg; **^{1}H NMR (400 MHz, $CDCl_3$)** δ 7.92 (d, *J* = 8.8 Hz, 2H, Ph-H), 7.50-7.53 (m, 4H, Ph-H), 7.44 (s, 1H, NH), 7.25-7.27 (m, 3H, Ph-H), 3.21 (t, *J* = 7.2 Hz, 2H, CH_2), 2.75 (t, *J* = 7.2 Hz, 2H, CH_2), 1.57 (s, 9H, CH_3); **^{13}C NMR (101 MHz, $CDCl_3$)** δ 169.6 (C=O), 139.1 (C), 137.6 (C), 133.1 (CH), 129.4 (CH), 128.9 (C-Se), 127.4 (CH), 125.4 (CH), 120.7 (CH), 117.0 (CH), 51.7 (C), 38.2 (CH_2-Se), 22.4 (CH_3), 21.6 (CH_2); **^{77}Se NMR (76 MHz, $CDCl_3$)** δ 308.4 ; **IR (film)**: ν (cm-1) 3317, 2921, 1707, 1599, 1294, 1161, 736, 465; **HR-MS (ESI)** m/z calcd for $C_{20}H_{24}NO_3Se$, $[M+H]^+$: 406.09159, found: 406.09147.

3-(phenylselanyl)-*N*-(m-tolyl)propanamide(7)

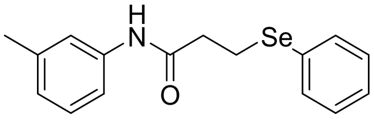

Prepared by **Conditions D.** Light yellow grease, 57.5mg. 90 % yield, η = 1.4 μL/mg, melting point 66 – 69°C; **^{1}H NMR (400 MHz, $CDCl_3$)** δ 7.52-7.54 (m, 2H, Ph-H), 7.35 (s, 1H, NH), 7.23-7.28 (m, 5H, Ph-H), 7.18 (t, *J* = 7.6 Hz, 1H, Ph-H), 6.92 (d, *J* = 7.6 Hz, 1H, Ph-H), 3.22 (t, *J* = 7.2 Hz, 2H, CH_2), 2.72 (t, *J* = 7.2 Hz, 2H, CH_2), 2.32 (s, 3H, CH_3); **^{13}C NMR (101 MHz, $CDCl_3$)** δ 169.6 (C=O), 139.1 (C), 137.6 (C), 133.1 (CH), 129.4 (CH), 128.9 (C-Se), 127.4 (CH), 125.3 (CH), 120.7 (CH), 117.1 (CH), 38.2 (CH_2-Se), 22.4 (CH_3), 21.6 (CH_2); **^{77}Se NMR (76 MHz, $CDCl_3$)** δ 308.4; **IR (film)**: ν (cm-1) 3299, 2922, 1659, 1552, 1437, 735, 690, 441; **HR-MS (ESI)** m/z calcd for $C_{16}H_{18}NOSe$, $[M+H]^+$: 320.05481, found: 320.05460.

***N,N*-dimethyl-3-(phenylselanyl)propanamide(8)**

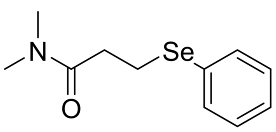

Prepared by **Conditions D.** Light yellow grease, 28.8 mg, 56% yield, η = 1.4 μL/mg; **^{1}H NMR (400 MHz, $CDCl_3$)** δ 7.49-7.52 (m, 2H, Ph-H), 7.24-7.28 (m, 3H, Ph-H), 3.17 (t, *J* = 7.4 Hz, 2H, CH_2), 2.93 (d, *J* = 4.8 Hz, 6H, CH_3), 2.72 (t, *J* = 7.4 Hz, 2H, CH_2); **^{13}C NMR (101 MHz, $CDCl_3$)** δ 171.6 (C=O), 132.7 (CH), 130.2 (C-Se), 129.2 (CH), 127.0 (CH), 37.2 (CH_3), 35.6 (CH_3), 34.4 (CH_2-Se), 22.5 (CH_2); **^{77}Se NMR (76 MHz, $CDCl_3$)** δ 309.0; **IR (film)**: ν (cm-1) 3449, 2924, 2853, 1641, 1400, 738, 692; **HR-MS (ESI)** m/z calcd for $C_{11}H_{16}NOSe$, $[M+H]^+$: 258.03916, found: 258.03840.

***N*-benzyl-3-(phenylselanyl)propanamide(9)**

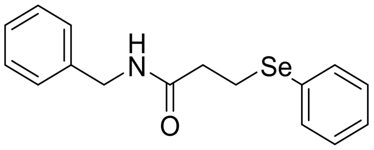 Prepared by **Conditions D**. White solid, 40.0 mg, 63% yield, η = 1.4 μL/mg, melting point 98 – 102°C; **^{1}H NMR (400 MHz, $CDCl_3$)** δ 7.48-7.50 (m, 2H, Ph-H), 7.24 -7.36 (m, 8H, Ph-H), 5.75 (s, 1H, NH), 4.43 (d, J = 5.6 Hz, 2H, CH_2), 3.19 (t, J = 7.2 Hz, 2H, CH_2), 2.59 (t, J = 7.2 Hz, 2H, CH_2); **^{13}C NMR (101 MHz, $CDCl_3$)** δ 171.2 (C=O), 138.1 (C), 138.0 (CH), 129.5 (C-Se), 129.3 (CH), 128.9 (CH), 128.0 (CH), 127.7 (CH), 127.3 (CH), 43.9 (CH_2), 37.3 (CH_2-Se), 22.6 (CH_2); **^{77}Se NMR (76 MHz, $CDCl_3$)** δ 308.4; **IR (film)**: ν (cm^{-1}) 3280, 2923, 1638, 1555, 1436, 1250, 729, 694, 464; **HR-MS (ESI)** m/z calcd for $C_{16}H_{18}NOSe$, $[M+H]^+$: 320.05481, found: 320.05458.

2-methyl-*N*-phenyl-3-(phenylselanyl)propanamide(10)

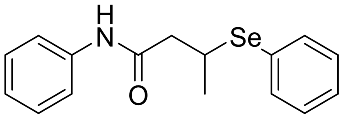 Prepared by **Conditions D.** White solid, 22.1 mg, 35% yield, η = 1.4 μL/mg, melting point 81 – 85°C; **^{1}H NMR (400 MHz, $CDCl_3$)** δ 7.58-7.60 (m, 2H, Ph-H), 7.49 (d, J = 7.6 Hz, 2H, Ph-H), 7.28-7.37 (m, 6H, Ph-H), 7.11 (t, J = 7.4 Hz, 1H, Ph-H), 3.75-3.83 (m, 1H, CH), 2.72 (dd, J = 14.8, 7.6 Hz, 1H, CH_2), 2.57 (dd, J = 14.8, 7.6 Hz, 1H, CH_2), 1.52 (d, J = 6.8 Hz, 3H, CH_3); **^{13}C NMR (101 MHz, $CDCl_3$)** δ 169.2 (C=O), 137.7 (C), 135.3 (CH), 129.3 (CH), 129.1 (CH), 128.3 (C-Se), 128.1 (CH), 124.6 (CH), 120.0 (CH), 45.7 (CH-Se), 34.7 (CH_2), 22.1 (CH_3) ; **^{77}Se NMR (76 MHz, $CDCl_3$)** δ 413.0; **IR (film)**: ν (cm^{-1}) 3300, 2923, 1658, 1544, 1443, 740, 692; **HR-MS (ESI)** m/z calcd for $C_{16}H_{17}NOSeNa$, $[M+Na]^+$: 342.03676 , found: 342.03694.

***N*-phenyl-3-(phenylthio)propanamide(11a)**

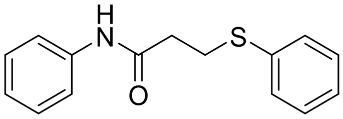 Prepared by **Conditions D.** White soild, 41.7 mg, 81% yield, η = 1.6 μL/mg; ^{1}H and ^{13}C NMR of the product **11a** were in agreement with the literature[31]. **^{1}H NMR (400 MHz, $CDCl_3$)** δ 7.69 (s, 1H, NH), 7.48 (d, J = 8.4 Hz, 2H, Ph-H), 7.36 (d, J = 8.0 Hz, 2H, Ph-H), 7.29 (t, J = 7.6 Hz, 4H, Ph-H), 7.20 (t, J = 7.4 Hz, 1H, Ph-H), 7.10 (t, J = 7.2 Hz, 1H, Ph-H), 3.26 (t, J = 7.2 Hz, 2H, CH_2), 2.63 (t, J = 7.2 Hz, 2H, CH_2); **^{13}C NMR (101 MHz, $CDCl_3$)** δ 169.5 (C=O), 137.7 (C), 135.2 (C-S), 129.7 (CH), 129.2 (CH), 129.1 (CH), 126.6 (CH), 124.5 (CH), 120.1 (CH), 37.1 (CH_2), 29.3 (CH_2-S).

***N*-phenyl-3-(p-tolylthio)propanamide(11b)**

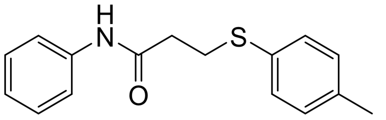 Prepared by **Conditions D.** Light yellow soild, 35.6 mg, 66% yield, η =1.6 μL/mg, melting point 63 – 65°C; **^{1}H NMR (400 MHz, $CDCl_3$)** δ 7.48 (d, J = 8.0 Hz, 3H, Ph-H),

7.28-7.32 (m, 4H, Ph-H), 7.09-7.13 (m, 3H, Ph-H), 3.24 (t, *J* = 7.2 Hz, 2H, CH_2), 2.61 (t, *J* = 7.2 Hz, 2H, CH_2), 2.32 (s, 3H, CH_3); **^{13}C NMR (101 MHz, $CDCl_3$)** δ 168.9 (C=O), 138.6 (C), 137.0 (C), 131.2 (C-S), 130.8 (CH), 130.0 (CH), 129.1 (CH), 124.5 (CH), 118.4 (CH), 37.3 (CH_2), 30.2 (CH_2-S), 21.1 (CH_3); **IR (film)**: ν (cm-1) 3301, 3139, 2923, 1661, 1545, 1433, 805, 755, 693, 503; **HR-MS (ESI)** m/z calcd for $C_{16}H_{18}NOS$, $[M+H]^+$: 272.10736, found: 272.10607.

3-((4-chlorophenyl)thio)-*N*-phenylpropanamide(11c)

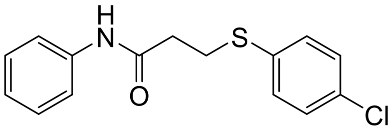

Prepared by **Conditions D.** Light yellow soild, 35.5 mg, 61% yield, η = 1.5 μL/mg, melting point 65 – 66°C; **^{1}H NMR (600 MHz, $CDCl_3$)** δ 7.41 (d, *J* = 8.0 Hz, 2H, Ph-H), 7.36 (s, 1H, NH), 7.23-7.25 (m, 3H, Ph-H), 7.19-7.20 (m, 2H, Ph-H), 7.05 (t, *J* = 7.4 Hz, 1H, Ph-H), 3.20 (t, *J* = 7.2 Hz, 2H, CH_2), 2.57 (t, *J* = 7.2 Hz, 2H, CH_2); **^{13}C NMR (150 MHz, $CDCl_3$)** δ 169.0 (C=O), 137.6 (C), 134.5 (C-S), 132.7 (C), 131.2 (CH), 129.3 (CH), 129.1 (CH), 124.2 (CH), 120.0 (CH), 37.1 (CH_2), 29.6 (CH_2-S); **IR (film)**: ν (cm^{-1}) 3323, 2947, 1653, 1542, 1446, 1088, 1048, 880, 750; **HR-MS (ESI)** m/z calcd for $C_{15}H_{15}NOSCl$, $[M+H]^+$: 292.05174, found: 292.05145.

***N*-phenyl-3-(*o*-tolylthio)propanamide (11d)**

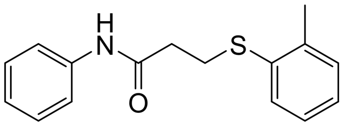

Prepared by **Conditions D.** Light yellow soild, 34.7 mg, 64% yield, η = 1.6 μL/mg, melting point 70 – 74°C; **^{1}H NMR (600 MHz, $CDCl_3$)** δ 7.49 (d, *J* = 7.8 Hz, 2H, Ph-H), 7.38 (s, 1H, NH), 7.30-7.38 (m, 3H, Ph-H), 7.17-7.20 (m, 2H, Ph-H), 7.10-7.14 (m, 2H, Ph-H), 3.27 (t, *J* = 7.2 Hz, 2H, CH_2), 2.66 (t, *J* = 7.2 Hz, 2H, CH_2), 2.38 (s, 3H, CH_3); **^{13}C NMR (150 MHz, $CDCl_3$)** δ 169.3 (C=O), 138.4 (C), 137.7 (C), 134.5 (C-S), 130.5 (CH), 129.1 (CH), 128.8 (CH), 126.7 (CH), 126.4 (CH), 124.6 (CH), 120.0 (CH), 37.1 (CH_2), 27.6 (CH_2-S), 21.3 (CH_3); **IR (film)**: ν (cm^{-1}) 3300, 3060, 2920, 1661, 1543, 1443, 1254, 1066, 750, 693, 505, 437; **HR-MS (ESI)** m/z calcd for $C_{16}H_{17}NOSNa$, $[M+Na]^+$: 294.09031, found: 294.09051.

3-((2-chlorophenyl)thio)-*N*-phenylpropanamide(11e)

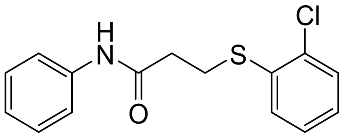

Prepared by **Conditions D.** Whie soild, 37.8 mg, 65% yield, η = 1.5 μL/mg, melting point 95 – 101°C; **^{1}H NMR (400 MHz, $CDCl_3$)** δ 7.50 (d, *J* = 8.0 Hz, 1H, Ph-H), 7.38 (d, *J* = 8.0 Hz, 3H, Ph-H), 7.32 (t, *J* = 7.8 Hz, 2H, Ph-H), 7.23 (d, *J* = 8.0 Hz, 1H, Ph-H), 7.10-7.17 (m, 2H, Ph-H), 3.33 (t, *J* = 7.2 Hz, 2H, CH_2), 2.69 (t, *J* = 7.2 Hz, 2H, CH_2); **^{13}C NMR (101 MHz, $CDCl_3$)** δ 169.0 (C=O), 138.4 (C), 135.1 (C-S), 134.3 (C), 130.0 (CH), 129.5 (CH), 129.1 (CH), 127.5 (CH), 127.3 (CH), 124.7 (CH), 120.1 (CH), 36.8 (CH_2), 27.7 (CH_2-S); **IR (film)**: ν (cm^{-1}) 3300, 3060, 2923, 1662,

1544, 1443, 1253, 1035, 749, 693, 505; **HR-MS (ESI)** m/z calcd for $C_{15}H_{14}ClNOSNa$, $[M+Na]^+$: 314.03668, found: 314.03561.

***N*-phenyl-3-(m-tolylthio)propanamide(11f)**

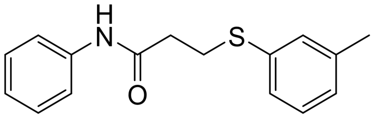

Prepared by **Conditions D.** Light yellow grease, 35.4 mg, 65% yield, η = 1.6 μL/mg, melting point 69 – 73℃; **^{1}H NMR (400 MHz, CDCl$_3$)** δ 7.48 (d, *J* = 8.0 Hz, 2H, Ph-H), 7.40 (s, 1H, NH), 7.31 (t, *J* = 8.0 Hz, 2H, Ph-H), 7.19 (d, *J* = 6.0 Hz, 3H, Ph-H), 7.11 (t, *J* = 7.4 Hz, 1H, Ph-H), 7.03 (d, *J* = 8.4 Hz, 1H, Ph-H), 3.28 (t, *J* = 7.2 Hz, 2H, CH_2), 2.64 (t, *J* = 7.2 Hz, 2H, CH_2), 2.32 (s, 3H, CH_3); **^{13}C NMR (101 MHz, CDCl$_3$)** δ 169.4 (C=O), 139.1 (C), 137.2 (C), 134.9 (C-S), 130.6 (CH), 129.1 (CH), 127.6 (CH), 126.9 (CH), 124.0 (CH), 121.4 (CH), 37.3 (CH_2), 29.4 (CH_2-S), 21.4 (CH_3); **IR (film)**: ν (cm^{-1}) 3300, 3056, 2923, 1661, 1543, 1442, 1309, 1254, 754, 690, 505; **HR-MS (ESI)** m/z calcd for $C_{16}H_{17}NOSNa$, $[M+Na]^+$: 294.09031, found: 294.09004.

3-((2,4-difluorophenyl)thio)-*N*-phenylpropanamide(11g)

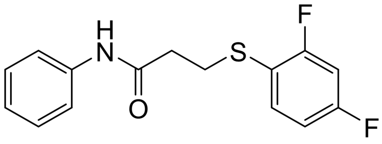

Prepared by **Conditions D.** Light yellow soild, 56.0 mg, 96% yield, η = 1.5 μL/mg, melting point 66 – 68℃; **^{1}H NMR (400 MHz, CDCl$_3$)** δ 7.42-7.49 (m, 4H, Ph-H), 7.31 (t, *J* = 8.0 Hz, 2H, Ph-H), 7.11 (t, *J* = 7.4 Hz, 1H, Ph-H), 6.85 (t, *J* = 8.2 Hz, 2H, Ph-H), 3.20 (t, *J* = 7.0 Hz, 2H, CH_2), 2.60 (t, *J* = 7.0 Hz, 2H, CH_2); **^{13}C NMR (CDCl$_3$, rt 101 MHz)** ^{13}C NMR (CDCl3, rt 101 MHz) δ 150.6 (C=O), 146.7 (dd, *J* = 30.0, 9.0 Hz, C), 144.7 (dd, *J* = 28.0, 9.0 Hz, C), 125.6 (C), 120.5 (dd, *J* = 18.0, 1.0 Hz, CH), 118.7 (CH), 115.1 (CH), 114.5 (CH), 109.2 (dd, *J* = 18.0, 3.0 Hz, C-S), 105.2 (dd, *J* = 15.0, 3.0 Hz, CH), 99.2 (t, *J* = 22.0 Hz, CH), 45.5 (CH_2), 30.5 (CH_2-S); **^{19}F NMR (376 MHz, CDCl$_3$)** δ -103.0, -108.9; **IR (film)**: ν (cm^{-1}) 3303, 2927, 1598, 1484, 1418, 1263, 1140, 965, 755, 693, 505; **HR-MS (ESI)** m/z calcd for $C_{15}H_{16}NOSF_2$, $[M+H]^+$: 294.07387, found: 294.07326.

3-((2,5-dichlorophenyl)thio)-*N*-phenylpropanamide(11h)

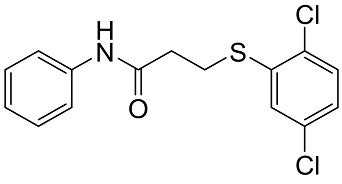

Prepared by **Conditions D.** White soild, 58.5 mg, 90% yield, η = 1.3 μL/mg, melting point 93 – 94℃; **^{1}H NMR (400 MHz, CDCl$_3$)** δ 7.47 (d, *J* = 9.0 Hz, 2H, Ph-H), 7.41 (s, 1H, NH), 7.24-7.31 (m, 4H, Ph-H), 7.05-7.11 (m, 2H, Ph-H), 3.29 (t, *J* = 7.2 Hz, 2H, CH_2), 2.69 (t, *J* = 7.2 Hz, 2H, CH_2); **^{13}C NMR (CDCl$_3$, rt 101 MHz)** δ 169.2 (C=O), 138.3 (C), 137.3 (C-S), 133.3 (C), 131.8 (C), 130.7 (CH), 129.1 (CH), 127.9 (CH), 126.9 (CH), 124.7 (CH), 120.8 (CH), 36.4 (CH_2), 28.1

(CH_2-S); **IR (film)**: ν (cm^{-1}) 3300, 2923, 2852, 1661, 1546, 1445, 1094, 1035, 806, 1035, 754, 574; **HR-MS (ESI)** m/z calcd for $C_{15}H_{16}NOSCl_2$, $[M+H]^+$: 326.01377, found: 326.01337.

3-((3,4-dichlorophenyl)thio)-*N*-phenylpropanamide(11i)

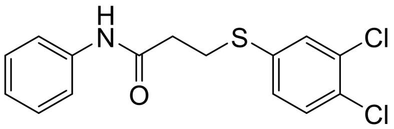

Prepared by **Conditions D.** Light yellow soild, 48.0 mg, 74% yield, η = 1.3 μL/mg, melting point 81 – 83°C; **^{1}H NMR (400 MHz, $CDCl_3$)** δ 7.47 (d, J = 7.6 Hz, 2H, Ph-H), 7.42 (d, J = 2.2 Hz, 2H, Ph-H), 7.29-7.35 (m, 3H, Ph-H), 7.17 (dd, J = 8.4, 2.2 Hz, 1H, Ph-H), 7.11 (t, J = 7.4 Hz, 1H, Ph-H), 3.28 (t, J = 7.2 Hz, 2H, CH_2), 2.65 (t, J = 7.2 Hz, 2H, CH_2); **^{13}C NMR (101 MHz, $CDCl_3$)** δ 168.8 (C=O), 137.5 (C), 136.0 (C-S), 133.1 (C), 130.9 (CH), 130.8 (CH), 130.6 (C), 129.2 (CH), 128.6 (CH), 124.7 (CH), 120.1 (CH), 37.0 (CH_2), 29.3 (CH_2-S); **IR (film):** ν (cm^{-1}) 3303, 2954, 2922, 2851, 1665, 1546, 1460, 1377, 1027, 812, 756, 694; **HR-MS (ESI)** m/z calcd for $C_{15}H_{16}NOSCl_2$, $[M+H]^+$: 326.01377, found: 326.01319.

3-((4-chloro-3-(4-ethoxybenzyl)phenyl)thio)-*N*-phenylpropanamide(11j)

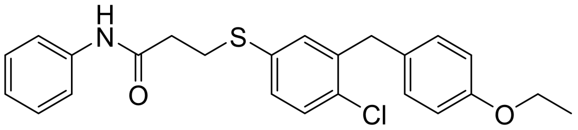

Prepared by **Conditions D.** White soild, 78.0 mg, 92% yield, η = 1.1 μL/mg, melting point 116 – 118°C; **^{1}H NMR (400 MHz, $CDCl_3$)** δ 7.45 (d, J = 7.8 Hz, 2H, Ph-H), 7.28-7.33 (m, 5H, Ph-H), 7.06-7.17 (m, 5H, Ph-H), 6.81 (d, J = 8.6 Hz, 2H, Ph-H), 3.95-4.00 (m, 4H, CH_2), 3.21 (t, J = 7.2 Hz, 2H, CH_2), 2.57 (t, J = 7.2 Hz, 2H, CH_2), 1.38 (t, J = 7.0 Hz, 3H, CH_3); **^{13}C NMR (150 MHz, $CDCl_3$)** ^{13}C NMR (150 MHz, CDCl3) δ 169.0 (C=O), 157.6 (C), 140.2 (C), 137.6 (C), 134.0 (C-S), 132.6 (C), 132.1 (CH), 131.0 (CH), 130.2 (CH), 130.0 (CH), 129.1 (CH), 128.9 (CH), 128.8 (CH), 124.6 (CH), 120.1 (CH), 114.7 (CH), 63.5 (CH_2), 38.3 (CH_2), 37.1 (CH_2), 29.5 (CH_2-S), 15.0 (CH_3); **IR (film)**: ν (cm^{-1}) 3303, 2924, 1600, 1510, 1442, 1243, 1042, 810, 755, 693, 536; **HR-MS (ESI)** m/z calcd for $C_{24}H_{25}ClNO_2S$, $[M+H]^+$: 426.12890, found: 426.12687.

***N*-phenyl-3-(thiophen-2-ylthio)propanamide(11k)**

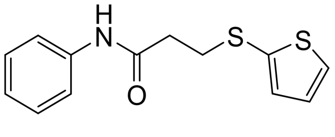

Prepared by **Conditions D.** Light yellow soild, 29.8 mg, 57% yield, η = 1.6 μL/mg, melting point 86 – 88°C; **^{1}H NMR (600 MHz, $CDCl_3$)** δ 7.50 (d, J = 8.0 Hz, 2H, Ph-H), 7.38 (d, J = 6.4 Hz, 1H, Ph-H), 7.32 (t, J = 8.0 Hz, 3H, Ph-H), 7.17 (d, J = 3.6 Hz, 1H, Ar-H), 7.11 (t, J = 7.4 Hz, 1H, Ph-H), 6.99-7.01 (m, 1H, Ar-H), 3.15 (t, J = 7.2 Hz, 2H, CH_2), 2.64 (t, J = 7.2 Hz, 2H, CH_2); **^{13}C NMR (150 MHz, $CDCl_3$)** δ 169.1 (C=O), 137.7 (C), 134.5 (CH), 133.3 (C-S), 130.0 (CH), 129.2 (CH), 127.9 (CH), 124.6 (CH), 120.0 (CH), 37.4 (CH_2), 33.7 (CH_2-S); **IR (film)**: ν (cm^{-1}) 3300, 3083, 2923,

1662, 1600, 1544, 1443, 755, 694, 505; **HR-MS (ESI)** m/z calcd for $C_{13}H_{13}NOS_2Na$, $[M+Na]^+$: 286.03308, found: 286.03227.

***N*-phenyl-3-(thiophen-3-ylthio)propanamide(11l)**

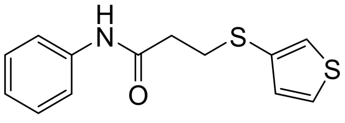

Prepared by **Conditions D.** Light yellow soild, 52.1 mg, 99% yield, η = 1.6 μL/mg, melting point 82 – 85°C; **^{1}H NMR (400 CDCl$_3$)** δ 7.49 (d, *J* = 8.0 Hz, 2H, Ph-H), 7.41 (s, 1H, Ph-H), 7.29-7.36 (m, 3H, Ph-H), 7.23 (d, *J* = 3.0 Hz, 1H, Ar-H), 7.06-7.13 (m, 2H, Ph-H), 3.20 (t, *J* = 7.0 Hz, 2H, CH_2), 2.63 (t, J = 7.2 Hz, 2H, CH_2); **^{13}C NMR (101 MHz, CDCl$_3$)** δ 169.3 (C=O), 136.0 (C), 131.3 (C-S), 130.1 (CH), 129.1 (CH), 127.3 (CH), 124.9 (CH), 124.6 (CH), 120.0 (CH), 37.4 (CH_2), 30.0 (CH_2-S); **IR (film)**: ν (cm^{-1}) 3300, 2955, 2920, 1663, 1444, 1096, 1012, 814, 755, 693; **HR-MS (ESI)** m/z calcd for $C_{13}H_{13}NOS_2Na$, $[M+Na]^+$: 286.03308, found: 286.02927.

3-(naphthalen-2-ylthio)-*N*-phenylpropanamide(11m)

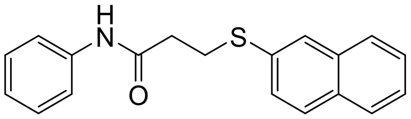

Prepared by **Conditions D.** White soild, 53.1mg, 86% yield, η = 1.2 μL/mg, melting point 97 – 100°C; **^{1}H NMR (400 MHz, CDCl$_3$)** δ 7.73-7.80 (m, 4H, Ph-H), 7.43-7.49 (m, 6H, Ph-H), 7.29 (t, *J* = 7.8 Hz, 2H, Ph-H), 7.10 (t, *J* = 7.4 Hz, 1H, Ph-H), 3.37 (t, *J* = 7.2 Hz, 2H, CH_2), 2.66 (d, *J* = 7.2 Hz, 2H, CH_2-S); **^{13}C NMR (101 MHz, CDCl3)** δ 169.3 (C=O), 137.7 (C), 134.2 (C), 132.7 (C-S), 132.0 (C), 129.1 (CH), 128.8 (CH), 127.8 (CH), 127.7 (CH), 127.2 (CH), 126.8 (CH), 126.1 (CH), 124.6 (CH), 120.0 (CH), 37.6 (CH_2), 29.2 (CH_2-S); **IR (film)**: ν (cm^{-1}) 3300, 3053, 2925, 1660, 1498, 1442, 1256, 810, 744, 692, 472; **HR-MS (ESI)** m/z calcd for $C_{19}H_{18}NOS$, $[M+H]^+$: 308.10736, found: 308.10561.

3-(hex-5-en-1-ylthio)-*N*-phenylpropanamide(11n)

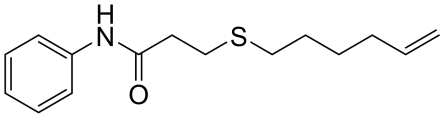

Prepared by **Conditions F.** Light yellow grease, 22.3 mg, 42% yield, η = 1.9 μL/mg; **^{1}H NMR (400 MHz, CDCl$_3$)** δ 7.60 (s, 1H, NH), 7.52 (d, *J* = 8.0 Hz, 2H, Ph-H), 7.32 (t, *J* = 7.8 Hz, 2H, Ph-H), 7.11 (t, *J* = 7.4 Hz, 1H, Ph-H), 5.73-5.83 (m, 1H, CH), 4.94-5.02 (m, 2H, CH_2), 2.90 (t, *J* = 7.0 Hz, 2H, CH_2), 2.56-2.65 (m, 4H, CH_2), 2.06 (q, *J* = 7.2 Hz, 2H, CH_2), 1.59-1.69 (m, 4H, CH_2), 1.45-1.52 (m, 2H, CH_2); **^{13}C NMR (101 MHz, CDCl$_3$)** δ 168.9 (C=O), 137.8 (CH), 137.7 (C), 128.7 (CH), 124.9 (CH), 121.1 (CH), 115.3 (CH_2), 37.7 (CH_2), 33.4 (CH_2), 32.4 (CH_2), 29.8 (CH_2-S), 28.1 (CH_2), 27.7 (CH_2-S); **IR (film)**: ν (cm^{-1}) 3304, 2954, 2923, 1665, 1546, 1444, 1256, 910, 756, 694, 506; **HR-MS (ESI)** m/z calcd for $C_{15}H_{21}NOSNa$, $[M+Na]^+$: 286.12161, found: 286.12132.

***N*-phenyl-3-(phenyltellanyl)propanamide(12a)**

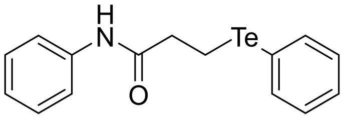 Prepared by **Conditions D.** Whie soild, 50.0 mg, 71% yield, η = 1.3 µL/mg, melting point 74 – 78°C; **^{1}H NMR (400 MHz, $CDCl_3$)** δ 7.75 (d, J = 8.0 Hz, 2H, Ph-H), 7.47 (d, J = 7.8 Hz, 2H, Ph-H), 7.27-7.37 (m, 4H, Ph-H), 7.21 (t, J = 7.4 Hz, 2H, Ph-H), 7.10 (t, J = 7.4 Hz, 1H, Ph-H), 3.13 (t, J = 7.2 Hz, 2H, CH_2), 2.94 (t, J = 7.2 Hz, 2H, CH_2-Te); **^{13}C NMR (101 MHz, $CDCl_3$)** δ 170.7 (C=O), 138.7 (CH), 137.7 (C), 129.4 (CH), 129.1 (CH), 128.0 (CH), 124.5 (CH), 120.0 (CH), 112.1 (C-Te), 39.7 (CH_2), 1.3 (CH_2-Te); **IR (film)**: ν (cm^{-1}) 3299, 3135, 2922, 2851, 1599, 1546, 1443, 734, 692; **HR-MS (ESI)** m/z calcd for $C_{15}H_{15}NOTeNa$, $[M+Na]^+$: 378.01081, found: 378.01248.

***N*-phenyl-3-(p-tolyltellanyl)propanamide(12b)**

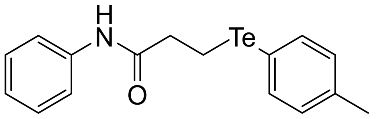 Prepared by **Conditions D.** Light yellow soild, 66.9 mg, 91% yield, η = 1.2 µL/mg, melting point 73 – 75°C; **^{1}H NMR (400 MHz, $CDCl_3$)** δ 7.66 (d, J = 7.6 Hz, 2H, Ph-H), 7.47 (d, J = 7.6 Hz, 2H, Ph-H), 7.30 (t, J = 7.8 Hz, 2H, Ph-H), 7.19 (s, 1H, NH), 7.10 (t, J = 7.4 Hz, 1H, Ph-H), 7.03 (d, J = 7.6 Hz, 2H, Ph-H), 3.10 (t, J = 7.2 Hz, 2H, CH_2), 2.90 (t, J = 7.2 Hz, 2H, CH_2-Te), 2.34 (s, 3H, CH_3); **^{13}C NMR (150 MHz, $CDCl_3$)** δ 170.7 (C=O), 139.2 (CH), 138.2 (C), 137.7 (C), 130.4 (CH), 129.1 (CH), 124.5 (CH), 120.0 (CH), 107.7 (C-Te), 40.5 (CH_2), 21.3 (CH_3), 1.1 (CH_2-Te); **IR (film)**: ν (cm^{-1}) 3284, 3136, 2923, 1662, 1543, 1442, 1249, 1200, 797, 754, 692, 480; **HR-MS (ESI)** m/z calcd for $C_{16}H_{17}NOTeNa$, $[M+Na]^+$: 392. 02346, found: 392.02431.

3-((4-chlorophenyl)tellanyl)-*N*-phenylpropanamide(12c)

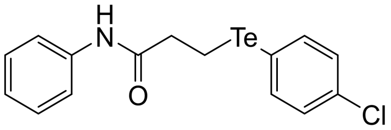 Prepared by **Conditions D.** Light yellow soild, 43.9 mg, 57% yield, η = 1.2 µL/mg, melting point 69 – 72°C; **^{1}H NMR (400 MHz, $CDCl_3$)** δ 7.67 (d, J = 6.4 Hz, 1H, Ph-H), 7.47 (d, J = 7.6 Hz, 2H, Ph-H), 7.31 (t, J = 8.0 Hz, 2H, Ph-H), 7.17 (d, J = 8.4 Hz, 2H, Ph-H), 7.11 (t, J = 7.4 Hz, 1H, Ph-H), 3.12 (t, J = 7.2 Hz, 2H, CH_2), 2.96 (t, J = 7.2 Hz, 2H, CH_2-Te); **^{13}C NMR (101 MHz, $CDCl_3$)** δ 170.5 (C=O), 140.1 (CH), 139.3 (C), 137.6 (CH), 134.5 (C), 129.6 (CH), 129.1 (CH), 124.6 (CH), 120.0 (CH), 109.9 (C-Te), 39.5 (CH_2), 1.7 (CH_2-Te); **IR (film)**: ν (cm^{-1}) 3291, 2921, 2852, 1667, 1547, 1444, 1249, 1089, 1018, 809, 756, 693, 485; **HR-MS (ESI)** m/z calcd for $C_{15}H_{14}NOTeClNa$, $[M+Na]^+$: 411.96984, found: 411.96888.

***N*-phenyl-3-(o-tolyltellanyl)propanamide(12d)**

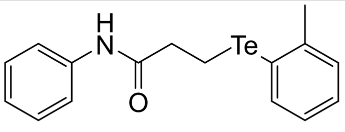 Prepared by **Conditions D.** White soild, 56.9 mg, 78% yield, η = 1.2 µL/mg, melting point 71 – 75°C; **^{1}H NMR (400 MHz, $CDCl_3$)** δ 7.68 (d, J = 8.2 Hz, 1H, Ph-H), 7.48 (d, J = 8.8 Hz, 2H, Ph-H), 7.30 (t, J = 8.0 Hz, 3H, Ph-H), 7.15-7.24 (m, 2H, Ph-H), 7.10 (t, J = 7.4 Hz, 1H, Ph-H), 7.02 (t, J = 6.2 Hz, 1H, Ph-H), 3.11 (t, J = 7.2 Hz, 2H, CH_2), 2.92 (t, J = 7.2 Hz, 2H, CH_2-Te), 2.44 (s, 3H, CH_3); **^{13}C NMR (101 MHz, $CDCl_3$)** δ 170.7 (C=O), 142.8 (C), 137.7 (C), 137.5 (CH), 129.3 (CH), 129.2 (CH), 129.1 (CH), 128.8 (CH), 128.1 (CH), 126.7 (CH), 124.5 (CH), 120.0 (CH), 116.5 (C-Te), 39.4 (CH_2), 26.7 (CH_3), 0.3 (CH_2-Te); **IR (film):** ν (cm^{-1}) 3286, 2955, 2922, 2852, 1668, 1444, 755, 693; **HR-MS (ESI)** m/z calcd for $C_{16}H_{17}NOTeNa$, $[M+Na]^+$: 392.02346, found: 392.02398.

3-((2-chlorophenyl)tellanyl)-*N*-phenylpropanamide(12e)

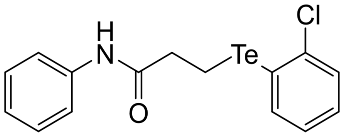 Prepared by **Conditions D.** Yellow soild, 62.6 mg, 81% yield, η = 1.2 µL/mg, melting point 64 – 69°C; **^{1}H NMR (400 MHz, $CDCl_3$)** δ 7.67 (d, J = 8.4 Hz, 2H, Ph-H), 7.47 (d, J = 7.4 Hz, 2H, Ph-H), 7.32 (t, J = 8.0 Hz, 2H, Ph-H), 7.09-7.18 (m, 4H, Ph-H), 3.12 (t, J = 7.2 Hz, 2H, CH_2), 2.96 (t, J = 7.2 Hz, 2H, CH_2-Te); **^{13}C NMR (101 MHz, $CDCl_3$)** δ 170.7 (C=O), 138.9 (C), 137.6 (C), 136.0 (CH), 129.1 (CH), 129.0 (CH), 128.7 (CH), 127.5 (CH), 124.6 (CH), 120.1 (CH), 117.0 (C-Te), 39.0 (CH_2), 1.2 (CH_2-Te); **IR (film)**: ν (cm^{-1}) 3282, 2955, 2923, 2852, 1545, 1444, 1250, 754, 693, 498; **HR-MS (ESI)** m/z calcd for $C_{15}H_{14}NOTeClH$, $[M+H]^+$: 389.98289, found: 389.98129.

***N*-phenyl-3-(*m*-tolyltellanyl)propanamide(12f)**

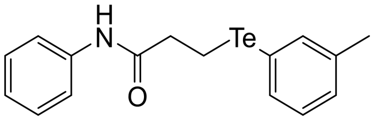 Prepared by **Conditions D.** Light yellow grease, 45.0 mg, 61% yield, η = 1.2 µL/mg, melting point 77 – 81°C;; **^{1}H NMR (400 MHz, $CDCl_3$)** δ 7.59 (s, 1H, Ph-H), 7.55 (t, J = 7.2 Hz, 1H, Ph-H), 7.47 (d, J = 7.8 Hz, 2H, Ph-H), 7.30 (t, J = 8.0 Hz, 3H, Ph-H), 7.08-7.12 (m, 3H, Ph-H), 3.12 (t, J = 7.2 Hz, 2H, CH_2), 2.93 (t, J = 7.2 Hz, 2H, CH_2-Te), 2.30 (s, 3H, CH_3); **^{13}C NMR (101 MHz, $CDCl_3$)** δ 170.8 (C=O), 141.3 (CH), 138.3 (C), 137.7 (C), 135.7 (CH), 129.2 (CH), 129.1 (CH), 128.5 (CH), 124.5 (CH), 120.0 (CH), 111.9 (C-Te), 39.7 (CH_2), 21.3 (CH_3), 1.1 (CH_2-Te); **IR (film)**: ν (cm^{-1}) 3290, 2927, 2852, 1545, 1443, 1249, 755, 692; **HR-MS (ESI)** m/z calcd for $C_{16}H_{17}NOTeNa$, $[M+Na]^+$: 392.02346, found: 392.02630.

3-((2,4-difluorophenyl)tellanyl)-*N*-phenylpropanamide(12g)

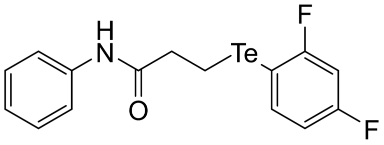 Prepared by **Conditions D.** Yellow soild, 46.0 mg, 59% yield, *η* = 1.2 μL/mg, melting point 75 – 78°C; **^{1}H NMR (400 MHz, $CDCl_3$)** δ 7.69 (q, *J* = 7.8 Hz, 1H, Ph-H), 7.48 (d, *J* = 7.2 Hz, 2H, Ph-H), 7.31 (t, *J* = 8.0 Hz, 3H, Ph-H), 7.11 (t, *J* = 8.0 Hz, 1H, Ph-H), 6.75-6.84 (m, 2H, Ph-H), 2.99-3.13 (m, 4H, CH_2); **^{13}C NMR (101 MHz, $CDCl_3$)** δ 170.6 (C=O), 165.4 (dd, *J* = 49.0, 12.0 Hz, C), 162.8 (d, *J* = 3.0 Hz, C), 140.9 (t, *J* = 7.5 Hz, CH), 139.7 (C), 137.5(CH), 129.1(CH), 124.6(CH), 120.0 (CH), 112.7 (dd, *J* = 16.0, 2.0 Hz (CH), 103.6 (dd, *J* = 14.0, 4.0 Hz, C-Te), 39.4 (CH_2), 1.2 (CH_2-Te); **^{19}F NMR (376 MHz, $CDCl_3$)** δ -86.6 (t, *J* = 7.5 Hz), -109.3 (q, *J* = 7.5 Hz); **IR (film)**: ν (cm^{-1}) 3310, 2954, 2919, 2851, 1459, 1377, 1024, 962, 757; **HR-MS (ESI)** m/z calcd for $C_{15}H_{13}NOTeF_2K$, $[M+K]^+$: 429.98590, found: 429.98649.

3-((2,5-dichlorophenyl)tellanyl)-*N*-phenylpropanamide(12h)

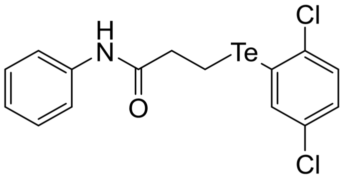 Prepared by **Conditions D.** Yellow soild, 61.2 mg, 73% yield, *η* = 1.1 μL/mg, melting point 78 – 82°C; **^{1}H NMR (400 MHz, $CDCl_3$)** δ 7.46-7.50 (m, 3H, Ph-H), 7.32 (t, *J* = 7.8 Hz, 3H, Ph-H), 7.24 (d, *J* = 7.8 Hz, 1H, Ph-H), 7.10-7.14 (m, 2H, Ph-H), 3.07-3.17 (m, 4H, CH_2); **^{13}C NMR (101 MHz, $CDCl_3$)** δ170.5 (C=O), 137.5 (C), 137.0 (C), 135.0 (CH), 133.2 (C), 129.7 (CH), 129.2 (CH), 128.4 (CH), 124.8 (CH), 120.1 (CH), 119.7 (C-Te), 38.6 (CH_2), 1.8 (CH_2-Te); **IR (film)**: ν (cm^{-1}) 3301, 2919, 2851, 1664, 1544, 1441, 1246, 1095, 1020, 755, 693, 559; **HR-MS (ESI)** m/z calcd for $C_{15}H_{13}NOTeCl_2Na$, $[M+H]^+$: 423.94592, found: 423.94497.

3-((3,4-dichlorophenyl)tellanyl)-*N*-phenylpropanamide(12i)

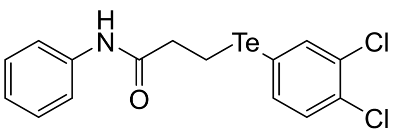 Prepared by **Conditions D.** Yellow soild, 61.8 mg, 73% yield, *η* = 1.1 μL/mg, melting point 79 – 85°C; **^{1}H NMR (400 MHz, $CDCl_3$)** δ 7.80-7.82 (m, 1H, Ph-H), 7.52-7.56 (m, 1H, Ph-H), 7.46-7.49 (m, 2H, Ph-H), 7.29-7.35 (m, 2H, Ph-H), 7.23-7.27 (m, 2H, Ph-H), 7.10-7.15 (m, 1H, Ph-H), 3.12-3.16(m, 2H, CH_2), 2.99-3.04 (m, 2H, CH_2); **^{13}C NMR (101 MHz, $CDCl_3$)** δ 170.3 (C=O), 139.8 (CH), 137.7 (CH), 137.5 (C), 133.1 (C), 132.5 (C), 131.1 (CH), 129.2 (CH), 124.7 (CH), 120.0 (CH), 111.7 (C-Te), 39.3 (CH_2), 2.2 (CH_2-Te); **IR (film)**: ν (cm^{-1}) 3300, 2922, 2851, 1657, 1542, 1443, 1130, 1027, 753, 692, 670; **HR-MS (ESI)** m/z calcd for $C_{15}H_{14}NOTeF_2$, $[M+H]^+$: 423.94592, found: 423.94424.

3-((4-chloro-3-(4-ethoxybenzyl)phenyl)tellanyl)-*N*-phenylpropanamide(12j)

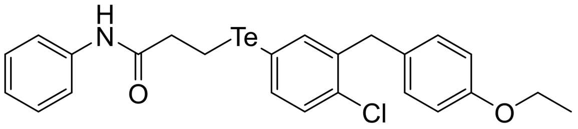 Prepared by **Conditions D.** Light yellow soild, 92.7 mg, 89% yield, η = 0.9 μL/mg, melting point 80 – 83°C; **^{1}H NMR (400 MHz, $CDCl_3$)** δ 7.51 (d, J = 8.0 Hz, 2H, Ph-H), 7.45 (d, J = 7.6 Hz, 2H, Ph-H), 7.31 (t, J = 8.0 Hz, 2H, Ph-H), 7.18 (d, J = 7.8 Hz, 1H, Ph-H), 7.10 (dd, J = 14.0, 9.6 Hz, 4H, Ph-H), 6.82 (d, J = 8.8 Hz, 2H, Ph-H), 3.95-4.00 (m, 4H, CH_2), 3.06 (t, J = 7.2 Hz, 2H, CH_2), 2.88 (t, J = 7.2 Hz, 2H, CH_2-Te), 1.38 (d, J = 14.0 Hz, 3H, CH_3); **^{13}C NMR (101 MHz, $CDCl_3$)** δ 170.5 (C=O), 157.6 (C) , 141.0 (CH), 140.3 (C), 138.3 (CH), 131.1 (C), 130.4 (CH), 130.0 (CH), 129.2 (C), 129.1 (CH), 128.6 (CH), 124.6 (C), 120.0 (CH), 114.6 (CH), 110.3 (C-Te), 63.5 (CH_2), 40.3 (CH_2), 38.2 (CH_2), 15.0 (CH_3), 1.6 (CH_2-Te); **IR (film)**: ν (cm^{-1}) 3300, 2955, 2923, 1498, 1441, 1242, 1039, 754, 692, 527; **HR-MS (ESI)** m/z calcd for $C_{24}H_{24}ClNO_2TeNa$, $[M+Na]^+$: 546.04100, found: 546.04129.

***N*-phenyl-3-(thiophen-2-yltellanyl)propanamide(12k)**

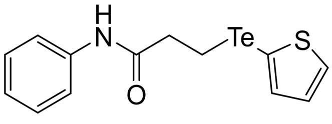 Prepared by **Conditions D.** White soild, 28.2 mg, 39% yield, η = 1.3 μL/mg, melting point 63 – 65°C; **^{1}H NMR (400 MHz, $CDCl_3$)** δ 7.49 (d, J = 8.0 Hz, 2H, Ph-H), 7.44 (d, J = 5.2 Hz, 1H, Ar-H), 7.40 (d, J = 3.6 Hz, 1H, Ar-H), 7.31 (t, J = 8.0 Hz, 3H, Ph-H), 7.11 (t, J = 7.4 Hz, 1H, Ph-H), 6.95 (dd, J = 5.2, 3.4 Hz, 1H, Ar-H), 2.93-3.02 (m, 4H); **^{13}C NMR (101 MHz, $CDCl_3$)** δ 170.7 (C=O), 141.6 (CH), 137.6 (C), 134.5 (CH), 129.2 (CH), 124.6 (CH), 120.1 (CH), 99.0 (C-Te), 39.1 (CH_2), 4.2 (CH_2-Te); **IR (film)**: ν (cm^{-1}) 3292, 2922, 1542, 1442, 1247, 842, 754, 639, 505; **HR-MS (ESI)** m/z calcd for $C_{13}H_{14}NOSTe$, $[M+H]^+$: 361.98228, found: 361.98133.

***N*-phenyl-3-(thiophen-2-yltellanyl)propanamide(12l)**

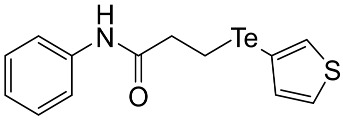 Prepared by **Conditions D.** Yellow soild, 20.2 mg, 28% yield, η = 1.3 μL/mg, melting point 61 – 63°C; **^{1}H NMR (400 MHz, $CDCl_3$)** δ 7.54 (d, J = 3.6 Hz, 1H, Ph-H), 7.48 (d, J = 8.0 Hz, 2H, Ph-H), 7.27-7.34 (m, 4H, Ph-H), 7.09-7.13 (m, 2H, Ph-H), 3.03 (t, J = 7.2 Hz, 2H, CH_2), 2.91 (t, J = 7.0 Hz, 2H, CH_2-Te); **^{13}C NMR (101 MHz, $CDCl_3$)** δ 170.6 (C=O), 138.4 (C), 137.2 (CH), 135.6 (CH), 130.5 (CH), 127.2 (CH), 123.3 (CH), 118.9 (CH), 101.6 (C-Te), 39.6 (CH_2), -2.7 (CH_2-Te); **IR (film)**: ν (cm^{-1}) 3290, 2924, 2852, 1600, 1545, 1443, 1249, 1076, 755, 692, 591; **HR-MS (ESI)** m/z calcd for $C_{13}H_{13}NOSTeNa$, $[M+Na]^+$: 383.96323, found: 383.96151.

3-(naphthalen-2-yltellanyl)-*N*-phenylpropanamide(12m)

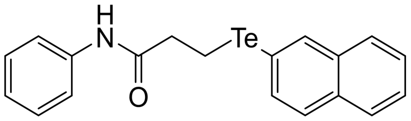 Prepared by **Conditions D.** Light yellow soild, 67.0 mg, 83% yield, η = 1.1 μL/mg, melting point 94 – 96°C; **^{1}H NMR (400 MHz, CDCl$_3$)** δ 8.23 (s, 1H, Ph-H), 7.67-7.82 (m, 4H, Ph-H), 7.44-7.51 (m, 4H, Ph-H), 7.29 (t, J = 8.0 Hz, 3H, Ph-H), 7.06-7.15 (m, 2H, Ph-H), 3.21 (t, J = 7.2 Hz, 2H, CH$_2$), 2.95 (t, J = 7.2 Hz, 2H, CH$_2$-Te); **^{13}C NMR (101 MHz, CDCl$_3$)** δ 170.6 (C=O), 138.4 (CH), 137.3 (C), 135.3 (CH), 134.2 (C), 132.7 (C), 129.2 (CH), 129.1 (CH), 128.6 (CH), 127.9 (CH), 127.4 (CH), 126.6 (CH), 124.5 (CH), 120.0 (CH), 109.4 (C-Te), 37.6 (CH$_2$), 1.3 (CH$_2$-Te); **IR (film)**: ν (cm^{-1}) 3299, 3049, 2920, 2851, 1543, 1442, 1248, 813, 754, 693, 474; **HR-MS (ESI)** m/z calcd for $C_{19}H_{18}NOTe$, $[M+H]^+$: 406.03151, found: 406.03049.

3-(hex-5-en-1-yltellanyl)-*N*-phenylpropanamide(12n)

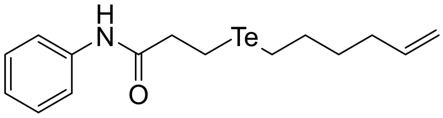 Prepared by **Conditions F.** Light yellow grease, 19.2 mg, 27% yield, η = 1.4 μL/mg; **^{1}H NMR (400 MHz, CDCl$_3$)** δ 7.50 (d, J = 8.0 Hz, 2H, Ph-H), 7.44 (s,1H, NH), 7.31 (t, J = 8.0 Hz, 2H, Ph-H), 7.10 (t, J = 8.0 Hz, 1H, Ph-H), 5.73-5.83 (m, 1H, CH), 4.93-5.02 (m, 2H, CH$_2$), 2.89-2.93 (m, 4H, CH$_2$), 2.68 (t, J = 8.0 Hz, 2H, CH$_2$), 2.05 (q, J = 8.0 Hz, 2H, CH$_2$), 1.72-1.80 (m, 2H, CH$_2$), 1.41-1.48 (m, 2H, CH$_2$);**^{13}C NMR (101 MHz, CDCl$_3$)** δ 170.7 (C=O), 138.6 (CH$_2$), 137.8 (C), 129.1 (CH), 124.5 (CH), 120.0 (CH), 114.8 (CH), 40.6 (CH$_2$), 33.1 (CH$_2$), 31.8 (CH$_2$), 31.3 (CH$_2$), 3.9 (CH$_2$-Te), -4.6 (CH$_2$-Te); **IR (film)**: ν (cm^{-1}) 3270, 3143, 3078, 2923, 1666, 1442, 1332, 1254, 747, 687, 596; **HR-MS (ESI)** m/z calcd for $C_{13}H_{14}NOTe$, $[M+H]^+$: 362.07281, found: 362.07082.

2-(phenylselanyl)pyrimidine(14)

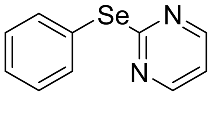 Prepared by **Conditions H.** Light yellow grease, 35.2 mg, 75% yield, η = 1.5 μL/mg; ^{1}H and ^{13}C NMR of the product **14** were in agreement with the literature[32]. **^{1}H NMR (400 MHz, CDCl$_3$)** δ 8.47 (d, J = 4.8 Hz 2H, Ar-H), 7.72-7.75 (m, 2H, Ph-H), 7.38-7.43 (m, 3H, Ph-H), 7.00 (t, J = 4.8 Hz, 1H, Ar-H); **^{13}C NMR (101 MHz, CDCl$_3$)** δ 171.6 (C-Se), 157.7 (CH), 136.3 (CH), 129.5 (CH), 129.1 (CH), 127.4 (C-Se), 117.8 (CH); **^{77}Se NMR (76 MHz, CDCl$_3$)** δ 494.5 ; **IR (film)**: ν (cm^{-1}) 2955, 2924, 1546, 1374, 1162, 738, 689; **HR-MS (ESI)** m/z calcd for $C_{10}H_9N_2Se$, $[M+H]^+$: 236.99255, found: 236.99239.

2-(benzylselanyl)pyrimidine(15) [33]

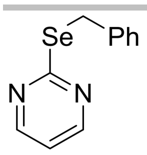

Prepared by **Conditions J.** Light yellow grease, 33.1 mg, 66% yield, η = 1.3 μL/mg; **^{1}H NMR (400 MHz, CDCl$_3$)** δ 8.52 (d, J = 5.2 Hz, 2H, Ar-H), 7.42 (d, J = 7.2 Hz, 2H, Ph-H), 7.22 (t, J = 7.4 Hz, 2H, Ph-H), 7.23 (t, J = 7.2 Hz, 1H, Ph-H), 7.02 (t, J = 4.8 Hz, 1H, Ar-H), 4.47 (s, 2H, CH$_2$); **^{13}C NMR (101 MHz, CDCl$_3$)** δ 170.9 (C-Se), 157.4 (CH), 138.7 (C), 129.2 (CH), 128.6 (CH), 127.1 (CH), 117.4 (CH), 30.5 (CH$_2$-Se); **^{77}Se NMR (76 MHz, CDCl$_3$)** δ 438.6; **IR (film)**: ν (cm^{-1}) 2957, 2920, 1645, 1459, 1378, 1264, 740; **HR-MS (ESI)** m/z calcd for $C_{11}H_{11}N_2Se$, [M+H]$^+$: 251.00820, found: 251.00831.

2-((3-phenoxypropyl)selanyl)pyrimidine(16)

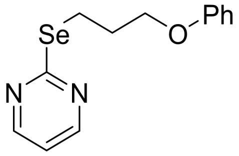

Prepared by **Conditions J.** Light yellow grease, 31.5 mg, 54% yield, η = 1.2 μL/mg; **^{1}H NMR (400 MHz, CDCl$_3$)** δ 8.47 (d, J = 4.8 Hz, 2H, Ar-H), 7.26-7.31 (m, 2H, Ph-H), 6.96-6.99 (m, 1H, Ph-H), 6.90-6.94 (m, 3H, Ph-H), 4.11 (t, J = 6.0 Hz, 2H, CH$_2$), 3.35 (t, J = 7.2 Hz, 2H, CH$_2$), 2.30-2.37 (m, 2H, CH$_2$); **^{13}C NMR (101 MHz, CDCl$_3$)** δ 170.4 (C-Se), 158.9 (C), 157.4 (CH), 129.5 (CH), 120.8 (CH), 117.3 (CH), 114.6 (CH), 67.1 (CH$_2$), 29.8 (CH$_2$-Se), 23.4 (CH$_2$); **^{77}Se NMR (76 MHz, CDCl$_3$)** δ 370.3; **IR (film)**: ν (cm^{-1}) 2923, 1586, 1376, 1242, 1171, 754, 692; **HR-MS (ESI)** m/z calcd for $C_{13}H_{15}N_2OSe$, [M+H]$^+$: 295.03441, found: 295.03488.

2-(phenethylselanyl)pyrimidine(17)

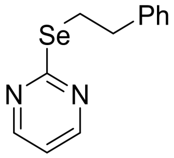

Prepared by **Conditions J.** Light yellow grease, 26.5 mg, 50% yield, η = 1.3 μL/mg; **^{1}H NMR (400 MHz, CDCl$_3$)** δ 8.51 (d, J = 4.8 Hz, 2H, Ar-H), 7.20-7.34 (m, 5H, Ph-H), 7.00 (t, J = 4.8 Hz, 1H, Ar-H), 3.41 (t, J = 8.0 Hz, 2H, CH$_2$), 3.14 (t, J = 8.0 Hz, 2H, CH$_2$); **^{13}C NMR (101 MHz, CDCl$_3$)** δ 170.6 (C-Se), 157.4 (CH), 141.4 (C), 128.7 (CH), 128.6 (CH), 126.5 (CH), 117.3 (CH), 36.7 (CH$_2$), 27.9 (CH$_2$-Se); **^{77}Se NMR (76 MHz, CDCl$_3$)** δ 381.6; **IR (film):** ν (cm^{-1}) 2921, 1558, 1546, 1376, 1171, 699; **HR-MS (ESI)** m/z calcd for $C_{12}H_{13}N_2Se$, [M+H]$^+$: 265.02385, found: 265.02428.

2-((2,3-dihydro-1*H*-inden-2-yl)selanyl)pyrimidine(18)

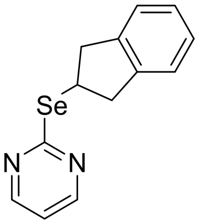

Prepared by **Conditions J.** Light yellow grease, 23.8 mg, 43% yield, η = 1.2 μL/mg; **^{1}H NMR (400 MHz, CDCl$_3$)** δ 8.58 (d, J = 4.8 Hz, 2H, Ar-H), 7.14-7.21 (m, 4H, Ph-H), 7.08 (t, J = 4.8 Hz, 1H, Ar-H), 4.23-4.31 (m, 1H, CH-Se), 3.39 (dd, J = 16.8, 7.2 Hz, 2H, CH$_2$), 3.19 (dd, J = 16.8, 5.2 Hz, 2H, CH$_2$); **^{13}C NMR (101 MHz, CDCl$_3$)** δ 167.0 (C-Se), 157.9 (CH), 141.8 (CH), 126.8 (CH), 124.6 (CH), 118.4 (CH), 41.6(CH-Se), 41.2 (CH$_2$); **^{77}Se NMR (76 MHz, CDCl$_3$)** δ 469.6; **IR (film)**: ν (cm^{-1}) 2920, 1558, 1546, 1375, 1171, 628; **HR-MS (ESI)** m/z calcd for $C_{13}H_{13}N_2Se$, [M+H]$^+$: 277.02385, found: 277.02379.

tert-butyl 3-(pyrimidin-2-ylselanyl)azetidine-1-carboxylate(19)

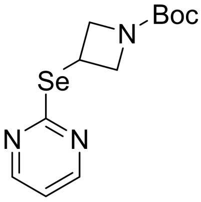

Prepared by **Conditions J.** Light yellow grease, 17.6 mg, 28% yield, η = 1.2 μL/mg; **^{1}H NMR (400 MHz, CDCl$_3$)** δ 8.58 (d, J = 4.8 Hz, 2H, Ar-H), 7.10 (t, J = 4.8 Hz, 1H, Ar-H), 4.31-4.35 (m, 2H, CH$_2$), 4.11-4.19 (m, 1H, CH), 4.02-4.06 (m, 2H, CH$_2$), 1.40 (s, 9H, CH$_3$); **^{13}C NMR (101 MHz, CDCl$_3$)** δ 166.4 (C-Se), 158.1 (CH), 156.1 (CH), 141.8 (C-Se), 118.7 (CH), 79.9 (CH), 28.5 (C), 28.4 (CH$_3$); **^{77}Se NMR (76 MHz, CDCl$_3$)** δ 453.6; **IR (film)**: ν (cm^{-1}) 3358, 2922, 1698, 1552, 1373, 1157, 765; **HR-MS (ESI)** m/z calcd for $C_{12}H_{18}N_3OSe$, [M+H]$^+$: 316.05588, found: 316.05390.

1-(2-(phenylselanyl)phenyl)ethan-1-one(21)

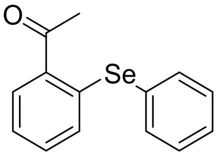

Prepared by **Conditions H.** Light yellow grease, 33.8 mg, 61% yield, η = 1.4 μL/mg; ^{1}H and ^{13}C NMR of the product **21** were in agreement with the literature[34]. **^{1}H NMR (400 MHz, CDCl$_3$)** δ 7.94-7.96 (m, 1H, Ph-H), 7.68-7.71 (m, 2H, Ph-H), 7.39-7.47 (m, 3H, Ph-H), 7.18-7.23 (m, 2H, Ph-H), 6.96-7.01 (m, 1H, Ph-H), 2.68 (s, 3H, CH$_3$); **^{13}C NMR (101 MHz, CDCl$_3$)** δ 198.9 (C=O), 140.6 (C), 137.5 (CH), 134.0 (C-Se), 132.6 (CH), 131.7 (CH), 129.8 (CH), 129.7 (C-Se), 129.6 (CH), 129.2 (CH), 124.7 (CH), 27.4 (CH$_3$); **^{77}Se NMR (76 MHz, CDCl$_3$)** δ 488.5; **HR-MS (ESI)** m/z calcd for $C_{14}H_{12}OSeNa$, [M+Na]$^+$: 298.99456, found 298.99465.

(phenylselanyl)propanoate(23)

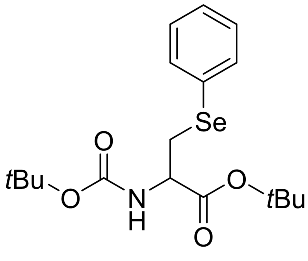

Prepared by **Conditions D.** Light yellow grease, 42.7 mg, 53% yield, η = 1.3 μL/mg; **^{1}H NMR (400 MHz, $CDCl_3$)** δ 7.50-7.53 (m, 2H, Ph-H), 7.22-7.25 (m, 3H, Ph-H), 5.28 (d, J = 7.2 Hz, 1H, NH), 4.52-4.56 (m, 1H, CH), 3.26-3.39 (m, 2H, CH_2), 1.42 (s, 9H, CH_3), 1.39 (s, 9H, CH_3); **^{13}C NMR (101 MHz, $CDCl_3$)** δ 169.8 (C=O), 155.0 (C=O), 133.1 (CH), 129.9 (CH), 129.2 (C-Se), 127.3 (CH), 82.7 (C), 79.8 (C), 54.3 (CH), 30.8 (CH_2-Se), 28.3 (CH_3), 28.0 (CH_3); **^{77}Se NMR (76 MHz, $CDCl_3$)** δ 253.4 ; **IR (film)**: ν (cm^{-1}) 3302, 3138, 3053, 2927, 1661, 1499, 1443, 1251, 1156, 755, 693; **HR-MS (ESI)** m/z calcd for $C_{18}H_{27}NOSeK$, $[M+K]^+$: 440.07369, found: 440.07446.

tert-butyldimethyl(2-(phenylselanyl)ethoxy)silane(25)

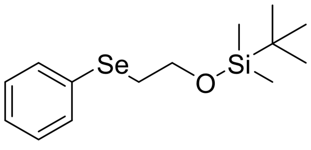

Prepared by **Conditions I.** Light yellow grease, 126.4 mg, 80% yield, η = 0.8 μL/mg; **^{1}H NMR (400 MHz, $CDCl_3$)** δ 7.49-7.52 (m, 2H, Ph-H), 7.22-7.27 (m, 3H, Ph-H), 3.84 (t, J = 7.6 Hz, 2H, CH_2), 3.03 (t, J = 7.6 Hz, 2H, CH_2), 0.88 (s, 9H, CH_3), 0.03 (s, 6H, CH_3) ; **^{13}C NMR (101 MHz, $CDCl_3$)** δ 132.6 (CH), 130.2 (C-Se), 129.1 (CH), 126.9 (CH), 63.2 (CH_2), 30.1 (CH_2-Se), 26.0 (C), 18.5 (CH_3), -5.1 (CH_3); **^{77}Se NMR (76 MHz, $CDCl_3$)** δ 298.1; **IR (film)**: ν (cm^{-1}) 2922, 1735, 1460, 1377, 1158, 1025, 954, 734.

2-(phenylselanyl)ethan-1-ol(26)

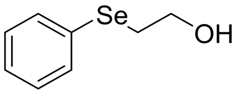

Prepared by **Conditions I.** Yellow grease, 56.8 mg, 70% yield; ^{1}H and ^{13}C NMR of the product **26** were in agreement with the literature[35]. **^{1}H NMR (400 MHz, $CDCl_3$)** δ 7.52-7.54 (m, 2H, Ph-H), 7.25-7.28 (m, 3H, Ph-H), 3.76 (t, J = 6.4 Hz, 2H, CH_2), 3.08 (t, J = 6.4 Hz, 2H, CH_2), 2.29 (s, 1H, OH); **^{13}C NMR (101 MHz, $CDCl_3$)** δ 133.2 (CH), 129.3 (CH), 128.8 (C-Se), 127.4 (CH), 61.0 (CH_2), 31.7 (CH_2-Se); **^{77}Se NMR (76 MHz, $CDCl_3$)** δ 237.5; **HR-MS (ESI)** m/z calcd for $C_8H_{11}OSe$, $[M+H]^+$:202.99696, found: 202.99687.

butyl(phenyl)selane(28)

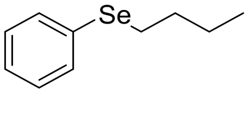

Prepared by **Conditions H**. Yellow grease, 35.7 mg, 84% yield, η = 1.3 μL/mg; ^{1}H and ^{13}C NMR of the product **28** were in agreement with the literature[36]. **^{1}H NMR (400 MHz, $CDCl_3$)** δ 7.47-7.52 (m, 2H, Ph-H), 7.22-7.30 (m, 3H, Ph-H), 2.94 (t, J = 7.4 Hz, 2H, CH_2), 1.68-1.75 (m, 2H, CH_2), 1.41-1.50 (m, 2H, CH_2), 0.93 (t, J = 7.4 Hz, 3H, CH_3); **^{13}C NMR (101 MHz, $CDCl_3$)** δ 132.4

(CH), 130.8 (CH), 129.1 (C-Se), 126.7 (CH), 32.3 (CH_2-Se), 27.7 (CH_2), 23.1 (CH_2), 13.7 (CH_3); **^{77}Se NMR (76 MHz, $CDCl_3$)** δ 291.2.

Se-phenyl benzoselenoate(30)

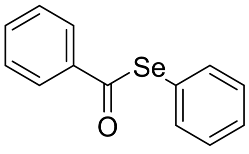

Prepared by **Conditions H.** yellow grease, 36.1 mg, 69% yield, η = 1.3 μL/mg; ^{1}H and ^{13}C NMR of the product **30** were in agreement with the literature[37]. **^{1}H NMR (400 MHz, $CDCl_3$)** δ 7.93-7.96 (m, 2H, Ph-H), 7.60-7.65 (m, 3H, Ph-H), 7.50 (t, J = 7.8 Hz, 2H, Ph-H), 7.41-7.45 (m, 3H, Ph-H); **^{13}C NMR (101 MHz, $CDCl_3$)** δ 193.5 (C=O), 138.6 (C-Se), 136.5 (CH), 134.0 (CH), 129.5 (CH), 129.2 (CH), 129.1 (CH), 127.5 (CH), 125.9 (C-Se); **^{77}Se NMR (76 MHz, $CDCl_3$)** δ 416.9.

3-(phenanthren-9-ylselanyl)-*N*-phenylpropanamide(32)

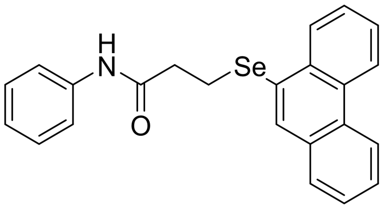

Prepared by **Conditions G.** White solid, 176.6 mg, 87% yield, η = 1.1 μL/mg, melting point 73 – 78°C; **^{1}H NMR (400 MHz, $CDCl_3$)** δ 8.66-8.72 (m, 4H, Ph-H), 8.49-8.52 (m, 1H, Ph-H), 8.14 (s, 1H, Ph-H), 7.82 (d, J = 7.8 Hz, 1H, Ph-H), 7.59 - 7.72 (m, 4H, Ph-H), 7.42 (d, J = 8.0 Hz, 2H, Ph-H), 7.26-7.30 (m, 2H, Ph-H), 7.07-7.12 (m, 2H, Ph-H), 3.31 (t, J = 7.2 Hz, 2H, CH_2), 2.68 (t, J = 7.2 Hz, 2H, CH_2); **^{13}C NMR (101 MHz, $CDCl_3$)** δ 169.6 (C=O), 167.2 (CH), 137.7 (C), 134.3 (CH), 132.7(C), 131.8(C), 130.8(C), 130.4 (C-Se), 129.1 (CH), 128.6 (CH), 128.3 (CH), 127.4 (CH), 127.3 (CH), 127.2 (CH), 124.5 (CH), 123.2 (CH), 122.8 (CH), 119.8 (CH), 114.0 (C), 38.1 (CH_2-Se), 22.4 (CH_2); **^{77}Se NMR (76 MHz, $CDCl_3$)** δ 254.5; **IR (film)**: ν (cm^{-1}) 3300, 2923, 1662, 1600, 1545, 1443, 748; **HR-MS (ESI)** m/z calcd for $C_{23}H_{20}NOSe$, $[M+H]^+$: 406.07046, found: 406.07035.

***N*-phenyl-3-(pyren-4-ylselanyl)propanamide(34)**

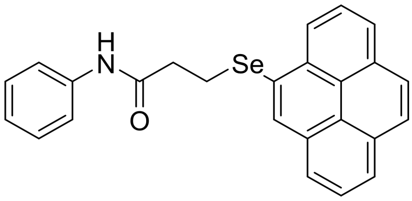

Prepared by **Conditions G.** Light yellow solid, 167.3 mg, 78% yield, η = 1.0 μL/mg; **^{1}H NMR (400 MHz, DMSO-D_6)** δ 9.89 (s, 1H, Ph-H), 8.48-8.52 (m, 1H, Ph-H), 8.05-8.37 (m, 9H, Ph-H), 7.45-7.54 (m, 1H, Ph-H), 7.24 - 7.28 (m, 2H, Ph-H), 6.94-7.03 (m, 1H, Ph-H), 3.39 (t, J = 7.2 Hz, 2H, CH_2), 2.79 (t, J = 7.2 Hz, 2H, CH_2); **^{13}C NMR (101 MHz, DMSO-D_6)** δ 191.0 (C=O), 167.5 (CH), 139.1 (C), 131.2 (CH), 130.9 (C), 130.5 (C), 130.2 (C-Se), 128.7 (CH), 128.2 (C), 127.5 (CH), 127.4 (CH), 126.6 (C), 126.1 (CH), 125.6 (C), 125.5 (CH), 125.4 (CH), 123.1 (C), 119.1 (CH), 37.1 (CH_2-Se), 22.8 (CH_2); **^{77}Se NMR (76 MHz, DMSO-D_6)** δ 248.8; **IR (film)**: ν (cm^{-1}) 3302,

3042, 2955, 2919, 2851, 1664, 1544, 1443, 1250, 845, 755; **HR-MS (ESI)** m/z calcd for $C_{25}H_{19}NOSeNa$, $[M+Na]^+$: 452.05241, found: 452.05165.

3-((4-iodophenyl)selanyl)-*N*-phenylpropanamide(36)

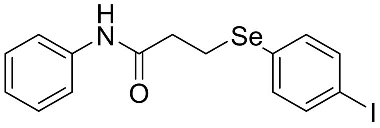

Prepared by **Conditions D.** Light yellow solid, 65.0 mg, 76% yield, η = 1.0 μL/mg, melting point 73 – 77°C; **^{1}H NMR (400 MHz, $CDCl_3$)** δ 7.57 (d, *J* = 8.2 Hz, 2H, Ph-H), 7.46 (d, *J* = 7.6 Hz, 2H, Ph-H), 7.31 (t, *J* = 7.8 Hz, 3H, Ph-H), 7.24 (d, *J* = 8.2 Hz, 1H, Ph-H), 7.11 (t, *J* = 7.4 Hz, 1H, Ph-H), 3.21 (t, *J* = 7.2 Hz, 2H, CH_2), 2.73 (t, *J* = 7.2 Hz, 2H, CH_2); **^{13}C NMR (101 MHz, $CDCl_3$)** δ 169.4 (C=O), 138.3 (CH), 137.6 (C), 134.7 (CH), 133.1 (C-Se), 129.2 (CH), 124.6 (CH), 120.0 (CH), 92.9 (C), 38.1 (CH_2-Se), 22.4 (CH_2); **^{77}Se NMR (76 MHz, $CDCl_3$)** δ 309.7; **IR (film)**: ν (cm^{-1}) 3284, 3133, 2922, 1650, 1537, 1442, 999, 690, 472; **HR-MS (ESI)** m/z calcd for $C_{15}H_{15}NOSeI$, $[M+H]^+$: 431.93581, found: 431.93654.

3-((4-iodophenyl)thio)-*N*-phenylpropanamide(37)

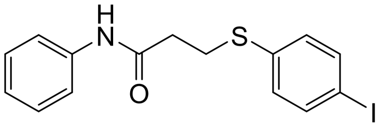

Prepared by **Conditions D.** White soild, 58.4 mg, 76% yield, η = 1.2 μL/mg, melting point 84 – 86°C; **^{1}H NMR (400 MHz, $CDCl_3$)** δ 7.60 (d, *J* = 8.1 Hz, 2H, Ph-H), 7.47 (d, *J* = 8.0 Hz, 2H, Ph-H), 7.27-7.41 (m, 3H, Ph-H), 7.09-7.13 (m, 3H, Ph-H), 3.28 (t, *J* = 7.2 Hz, 2H, CH_2), 2.64 (t, *J* = 7.2 Hz, 2H, CH_2); **^{13}C NMR (101 MHz, $CDCl_3$)** δ 168.2 (C=O), 139.3 (CH), 137.6 (C), 136.1 (C-S), 132.2 (CH), 128.6 (CH), 123.5 (CH), 119.0 (CH), 87.7 (C), 35.5 (CH_2), 29.1 (CH_2-S); **IR (film)**: ν (cm^{-1}) 3287, 2955, 2922, 2851, 1651, 1543, 1444, 1092, 1012, 754, 503; **HR-MS (ESI)** m/z calcd for $C_{15}H_{15}NOSI$, $[M+H]^+$: 383.98735, found: 383.98692.

3-((4-iodophenyl)tellanyl)-*N*-phenylpropanamide(38)

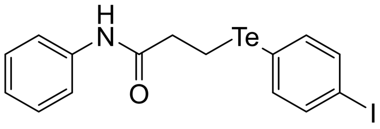

Prepared by **Conditions D.** Yellow soild, 70.1 mg, 73% yield, η = 1.0 μL/mg, melting point 85 – 88°C; **^{1}H NMR (400 MHz, $CDCl_3$)** δ 7.51-7.43 (m, 6H, Ph-H), 7.31 (t, *J* = 7.8 Hz, 3H, Ph-H), 7.11 (t, *J* = 7.8 Hz, 1H, Ph-H), 3.11 (t, *J* = 7.8 Hz, 2H, CH_2), 2.95 (t, *J* = 7.8 Hz, 2H, CH_2); **^{13}C NMR (101 MHz, $CDCl_3$)** δ 170.5 (C=O), 140.3 (CH), 138.4 (CH), 137.6 (C), 129.1 (CH), 124.6 (CH), 120.0 (CH), 111.8 (C-Te), 94.2 (C), 39.5 (CH_2), 1.7 (CH_2-Te); **IR (film)**: ν (cm^{-1}) 3300, 2954, 2921, 2851, 1661, 1544, 1443, 998, 755, 693, 471; **HR-MS (ESI)** m/z calcd for $C_{15}H_{15}NOTeI$, $[M+H]^+$: 481.91551, found: 481.91660.

7-chloro-4-(phenylselanyl)quinoline(40)

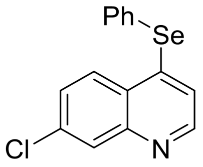 Prepared by **Conditions H.** Light yellow Solid, 47.9 mg, 75% yield, η = 1.3 μL/mg; ^{1}H and ^{13}C NMR of the product **40** were in agreement with the literature[38]. **^{1}H NMR (400 MHz, CDCl$_3$)** δ 8.52 (d, *J* = 4.8 Hz, 1H), 8.08 (d, *J* = 2.0 Hz, 1H, Ar-H), 8.01 (d, *J* = 8.8 Hz, 1H, Ph-H), 7.64-7.67 (m, 2H, Ph-H), 7.53 (dd, *J* = 8.8, 2.0 Hz, 1H, Ph-H), 7.42-7.49 (m, 3H, Ph-H), 6.99 (d, *J* = 4.8 Hz, 1H, Ar-H); **^{13}C NMR (101 MHz, CDCl$_3$)** δ 150.5 (CH), 148.2 (C-Se), 146.4 (C), 136.5 (CH), 135.9 (C), 130.3 (CH), 129.7 (CH), 129.0 (CH), 127.8 (CH), 126.8 (CH), 126.4 (C-Se), 126.1 (C), 122.1 (CH); **^{77}Se NMR (76 MHz, CDCl$_3$)** δ 387.7.

1-(allyloxy)-2-bromobenzene(41)

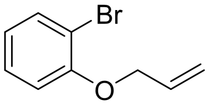 Colorless grease, 1921.0 mg, 91% yield; ^{1}H and ^{13}C NMR of the product **41** were in agreement with the literature[7]. **^{1}H NMR (400 MHz, CDCl$_3$)** δ 7.55 (dd, *J* = 8.0, 1.6 Hz, 1H, Ph-H), 7.23-7.27 (m, 1H, Ph-H), 6.90 (dd, *J* = 8.0, 1.2 Hz, 1H, Ph-H), 6.84 (td, *J* = 7.6, 1.6 Hz, 1H, Ph-H), 6.03-6.12 (m, 1H, CH), 5.50 (dq, *J* = 17.2, 1.6 Hz, 1H, CH$_2$), 5.32 (dq, *J* = 10.4, 1.6 Hz, 1H, CH$_2$), 4.61 (dt, *J* = 5.2, 1.6 Hz, 2H, CH$_2$); **^{13}C NMR (101 MHz, CDCl$_3$)** δ 155.0 (C), 133.5 (CH), 132.7 (CH), 128.5 (CH), 122.1 (CH), 117.8 (CH$_2$), 113.6 (CH), 112.3 (C), 69.7 (CH).

3-((2-(allyloxy)phenyl)selanyl)-*N*-phenylpropanamide(45)

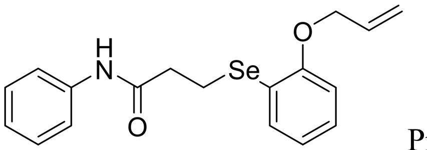 Prepared by **Mechanistic insights a).** Light yellow Solid, 30.1 mg, 42% yield, η = 1.4 μL/mg; **^{1}H NMR (400 MHz, CDCl$_3$)** δ 7.49 (d, *J* = 7.8 Hz, 2H, Ph-H), 7.44 (dd, *J* = 8.0, 1.2 Hz, 2H, Ph-H), 7.29-7.33 (m, 2H, Ph-H), 7.23 (td, *J* = 8.2, 1.6 Hz, 1H, Ph-H), 7.10 (t, *J* = 7.4 Hz, 1H, Ph-H), 6.92 (t, *J* = 7.6 Hz, 1H, Ph-H), 6.86 (d, *J* = 8.0 Hz, 1H, Ph-H), 6.01-6.10 (m, 1H, CH), 5.45 (dq, *J* = 17.2, 1.6 Hz, 1H, CH$_2$), 5.28 (dq, *J* =10.8, 1.2 Hz, 1H, CH$_2$), 4.60 (dt, *J* = 5.2, 1.2 Hz, 2H, CH$_2$), 3.23 (t, *J* = 7.2 Hz, 2H, CH$_2$), 2.74 (t, *J* = 7.2 Hz, 2H, CH$_2$); **^{13}C NMR (101 MHz, CDCl$_3$)** δ 169.9 (C=O), 157.4 (C), 137.8 (C), 132.9 (CH), 132.5 (CH), 129.1 (CH), 128.4 (CH), 124.5 (CH), 121.9 (CH), 120.0 (CH), 118.9 (C-Se), 117.9 (CH), 112.3 (CH$_2$), 69.5 (CH$_2$), 38.1 (CH$_2$-Se), 20.3 (CH$_2$); **^{77}Se NMR (76 MHz, CDCl$_3$)** δ 245.0; **IR (film)**: ν (cm^{-1}) 3301, 3137, 2923, 1622, 1422, 1239, 751; **HR-MS (ESI)** m/z calcd for $C_{18}H_{20}NO_2Se$, $[M+H]^+$: 362.06538, found 362.06585.

***tert*-butyl 4-acrylamidobenzoate (3b)**

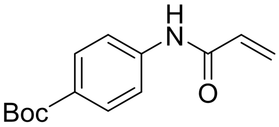 Light yellow Solid, 935.0 mg, 76% yield; ^{1}H and ^{13}C NMR of the product **3b** were in agreement with the literature[1]. **^{1}H NMR (400 MHz, CDCl$_3$)** δ 8.46 (s, 1H, NH), 7.92 (d, *J* = 8.4 Hz, 2H, Ph-H), 7.68 (d, *J* = 8.8 Hz, 3H, Ph-H), 6.44 (dd, *J* = 16.8, 1.6 Hz, 1H, CH), 6.33 (dd, *J* = 17.0, 9.4 Hz, 1H, CH_2), 5.75 (dd, *J* = 9.8, 1.6 Hz, 1H, CH_2), 1.57 (s, 9H, CH_3); **^{13}C NMR (101 MHz, CDCl$_3$)** δ 165.6 (C=O), 164.2 (C=O), 141.9 (C), 131.0 (CH), 130.7 (CH), 128.6 (C), 127.6 (CH_2), 119.2 (CH), 81.2 (C), 28.3 (CH_3).

***N*-(4-methoxyphenyl)acrylamide (3c)**

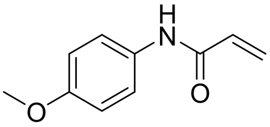 Brown Solid, 540.0 mg, 61% yield; ^{1}H and ^{13}C NMR of the product **3c** were in agreement with the literature[1]. **^{1}H NMR (400 MHz, CDCl$_3$)** δ 8.17 (s, 1H, NH), 7.48 (d, *J* = 9.2 Hz, 2H, Ph-H), 6.8 (d, *J* = 8.8 Hz, 2H, Ph-H), 6.38 (dd, *J* = 17.0, 2.2 Hz, CH), 6.28 (dd, *J* = 16.8, 9.6 Hz, CH_2), 5.67 (dd, *J* = 9.6, 2.2 Hz, 1H, CH_2), 3.76 (s, 3H, CH_3); **^{13}C NMR (101 MHz, CDCl$_3$)** δ 163.9 (C=O), 156.6 (C), 131.4 (CH), 131.1 (C), 127.3 (CH_2), 122.1 (CH), 114.1 (CH), 55.5 (CH_3).

***N*-(m-tolyl)acrylamide (3d)**

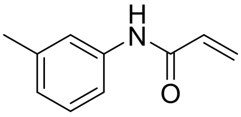 Light yellow solid, 555.0 mg, 69% yield; ^{1}H and ^{13}C NMR of the product **3d** were in agreement with the literature[1]. **^{1}H NMR (400 MHz, CDCl$_3$)** δ 8.31 (s, 1H, NH), 7.46 (s, 1H, NH), 7.39 (d, *J* = 8.0 Hz, 1H, Ph-H), 7.17 (t, *J* = 7.8 Hz, 1H, Ph-H), 6.92 (d, *J* = 7.6 Hz, 1H, Ph-H), 6.41 (dd, *J* = 16.8, 2.0 Hz, 1H, CH=), 6.33 (dd, *J* = 16.8, 9.6 Hz, 1H, =CH_2), 5.70 (dd, *J* = 9.6, 2.0 Hz, 1H, CH_2), 2.28 (s, 3H, CH_3); **^{13}C NMR (101 MHz, CDCl$_3$)** 164.1(C=O), 138.9 (C), 137.8 (C), 131.5 (CH), 128.8 (CH), 127.6 (CH_2), 125.4 (CH), 121.0 (CH), 117.5 (CH), 21.5 (CH_3).

(iodomethyl)benzene(1s)

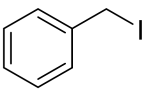 Yellow grease, 3507.1 mg, 80% yield; ^{1}H and ^{13}C NMR of the product **1s** were in agreement with the literature[2]. **^{1}H NMR (400 MHz, CDCl$_3$)** δ 7.37-7.40 (m, 2H, Ph-H), 7.23-7.33 (m, 3H, Ph-H), 4.47 (s, 2H, CH_2); **^{13}C NMR (101 MHz, CDCl$_3$)** δ 139.4 (C), 128.9 (CH), 128.8 (CH), 128.0 (CH), 5.9 (CH_2).

(2-iodoethyl)benzene(1t)

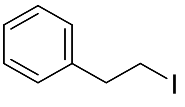 Purple grease, 1274.0 mg, 28% yield; ^{1}H and ^{13}C NMR of the product **1t** were in agreement with the literature[2]. **^{1}H NMR (400 MHz, CDCl$_3$)** δ 7.26 - 7.36 (m, 3H, Ph-H), 7.20-7.22 (m,

3H, Ph-H), 3.37 (t, *J* = 7.8 Hz, 2H, CH_2), 3.20 (t, *J* = 8.0 Hz, 2H, CH_2); **^{13}C NMR (101 MHz, $CDCl_3$)** δ 140.7 (C), 128.7 (CH), 128.4 (CH), 127.0 (CH), 40.5 (CH_2), 5.8 (CH_2).

1-(tert-butyl)-2-iodobenzene (1u)

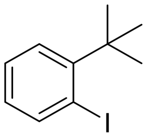

Brown grease, 400.0 mg, 10% yield; ^{1}H and ^{13}C NMR of the product **1u** were in agreement with the literature[3]. **^{1}H NMR (400 MHz, $CDCl_3$)** δ 8.01 (dd, *J* = 7.8, 1.4 Hz, 1H, Ph-H), 7.45 (dd, *J* = 8.0, 1.6 Hz, 1H, Ph-H), 7.27-7.32 (m, 1H, Ph-H), 6.82-6.86 (m, 1H, Ph-H), 1.55 (s, 9H, CH_3); **^{13}C NMR (101 MHz, $CDCl_3$)** 150.3 (C), 143.7 (CH), 128.0 (CH), 127.7 (CH), 127.6 (CH), 95.3 (C), 36.3 (C), 30.0 (CH_3).

2-iodo-1-methyl-1*H*-indole (1v)

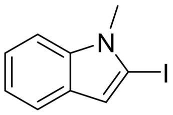

White solid, 630.0 mg, 44% yield; ^{1}H and ^{13}C NMR of the product **1v** were in agreement with the literature[4]. **^{1}H NMR (400 MHz, $CDCl_3$)** δ 7.55 (d, *J* = 8.0 Hz, 1H, Ph-H), 7.32 (d, *J* = 8.4 Hz, 1H, Ph-H), 7.16-7.20 (m, 1H, Ph-H), 7.10 (td, *J* = 7.6, 1.0 Hz, 1H, Ph-H), 6.82 (s, 1H, Ar-H), 3.77 (s, 3H, CH_3); **^{13}C NMR (101 MHz, $CDCl_3$)** 138.1 (C), 129.6 (C), 121.9 (C), 119.8 (CH), 119.5 (CH), 111.8 (CH), 109.7 (CH), 84.0 (CH), 34.1 (CH_3).

2-iodobenzofuran (1w)

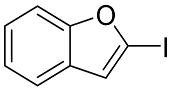

Yellow grease, 956.0 mg, 78% yield; ^{1}H and ^{13}C NMR of the product **1w** were in agreement with the literature[5]. **^{1}H NMR (400 MHz, $CDCl_3$)** δ 7.49-7.54 (m, 2H, Ph-H), 7.22-7.27 (m, 2H, Ph-H), 6.97 (s, 1H, Ar-H); **^{13}C NMR (101 MHz, $CDCl_3$)** 158.2 (C), 129.2 (C), 124.3 (CH), 123.2 (CH), 119.8 (CH), 117.3 (CH), 110.9 (CH), 96.0 (C).

2,3-dihydro-1*H*-inden-2-ol (1x)

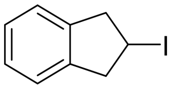

White solid, 1513.7 mg, 62% yield; ^{1}H and ^{13}C NMR of the product **1t** were in agreement with the literature[6]. **^{1}H NMR (400 MHz, $CDCl_3$)** δ 7.19-7.27 (m, 4H, Ph-H), 4.67-4.73 (m, 1H, CH), 3.47 (dd, *J* = 16.8, 6.4 Hz, CH_2), 3.38 (dd, *J* = 16.8, 5.0 Hz, CH_2); **^{13}C NMR (101 MHz, $CDCl_3$)** 141.5 (C), 127.0 (CH), 124.4 (CH), 46.6 (CH_2), 24.1 (CH).

(3-iodopropoxy)benzene (1y)[6]

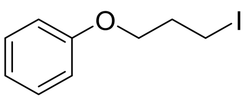

Yellow grease, 1010.0 mg, 76% yield; ^{1}H and ^{13}C NMR of the product **1y** were in agreement with the literature[6]. **^{1}H NMR (400 MHz, $CDCl_3$)** δ 7.28-7.32 (m, 2H, Ph-H), 6.97 (t, *J* = 7.4

Hz, 1H, Ph-H), 6.92 (d, *J* = 8.4 Hz, 1H, Ph-H), 4.05 (t, *J* = 5.8 Hz, 2H, CH_2), 3.39 (t, *J* = 6.8 Hz, 2H, CH_2), 2.26-2.32 (m, 2H, CH_2); **^{13}C NMR (101 MHz, $CDCl_3$)** 158.7 (C), 129.6 (CH), 121.0 (CH), 114.6 (CH), 67.2 (CH_2), 33.1 (CH_2), 2.8 (CH_2)

Supplementary Spectra: (NMR, IR, HR-MS)

^{1}H NMR (400 MHz, $CDCl_3$, 25°C) of compound **2a**

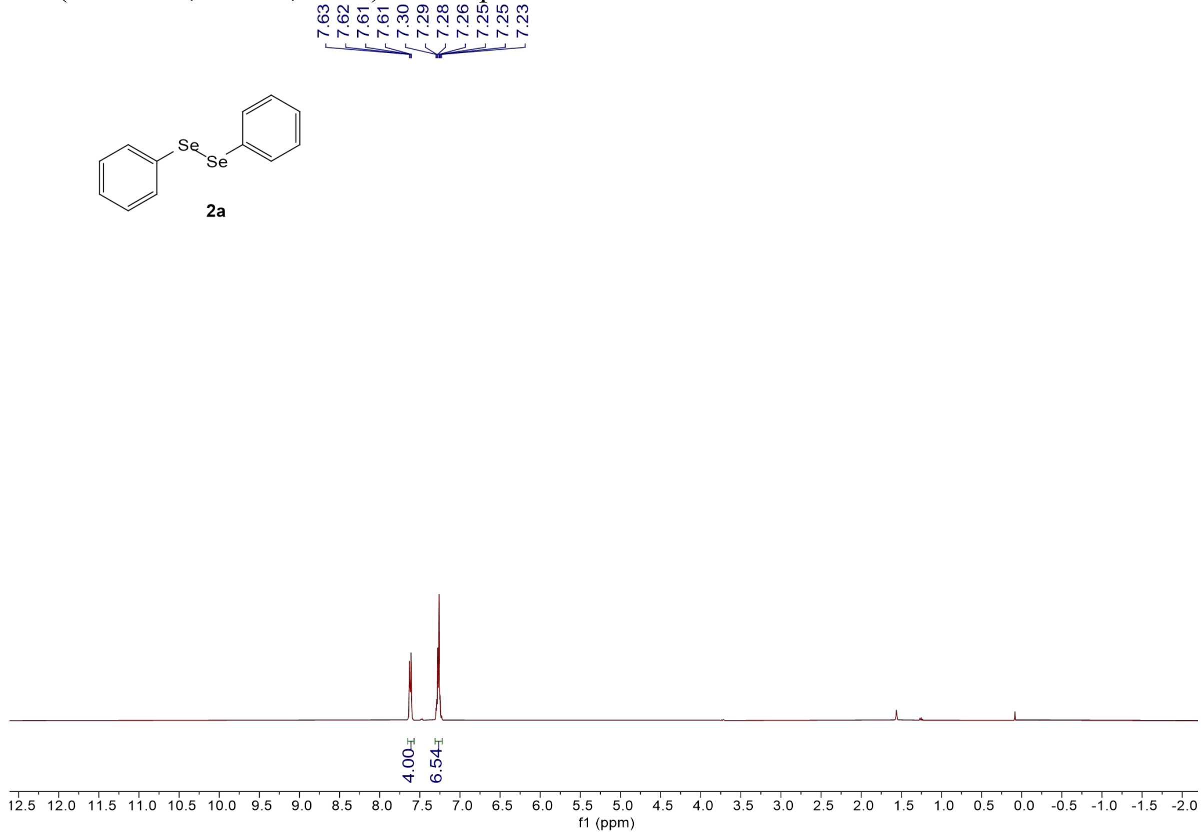

^{13}C NMR (101 MHz, $CDCl_3$, 25°C) of compound **2a**

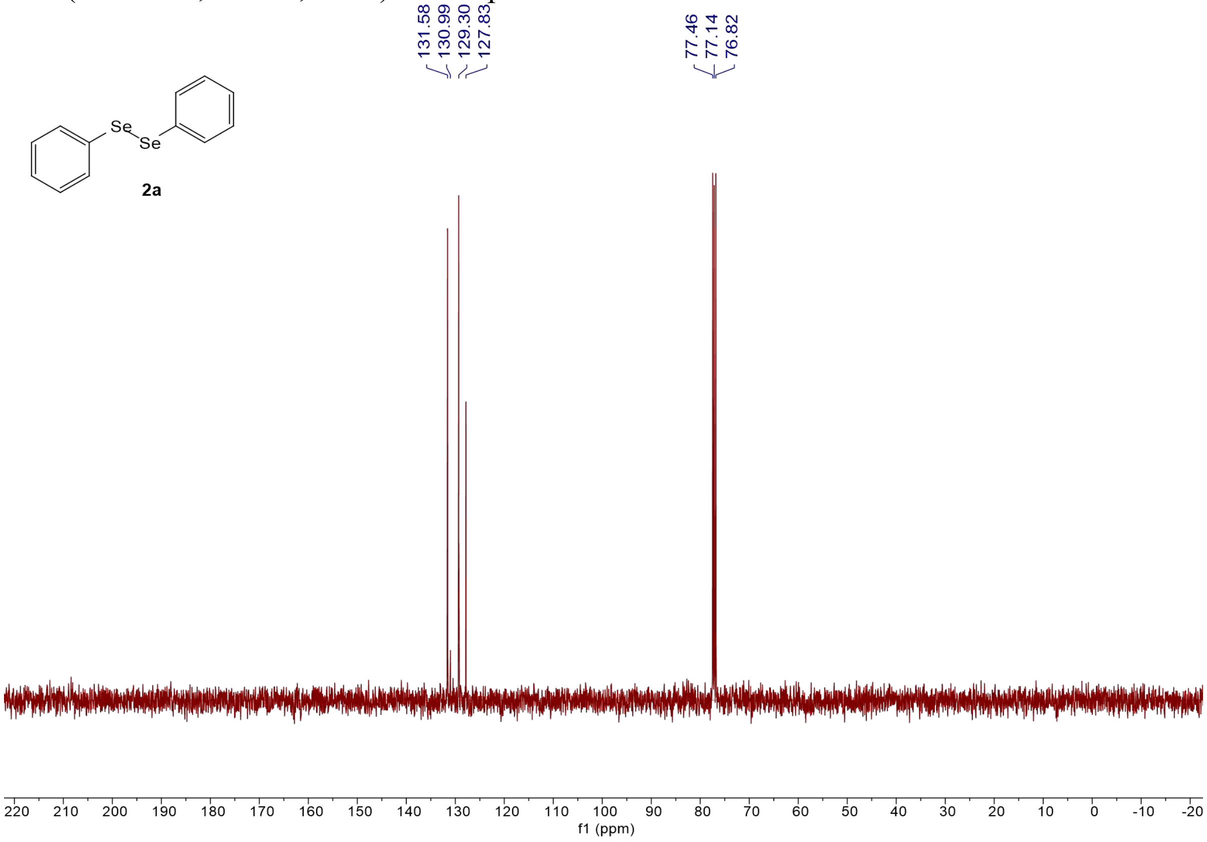

^{77}Se NMR (76 MHz, $CDCl_3$, 25°C) of compound **2a**

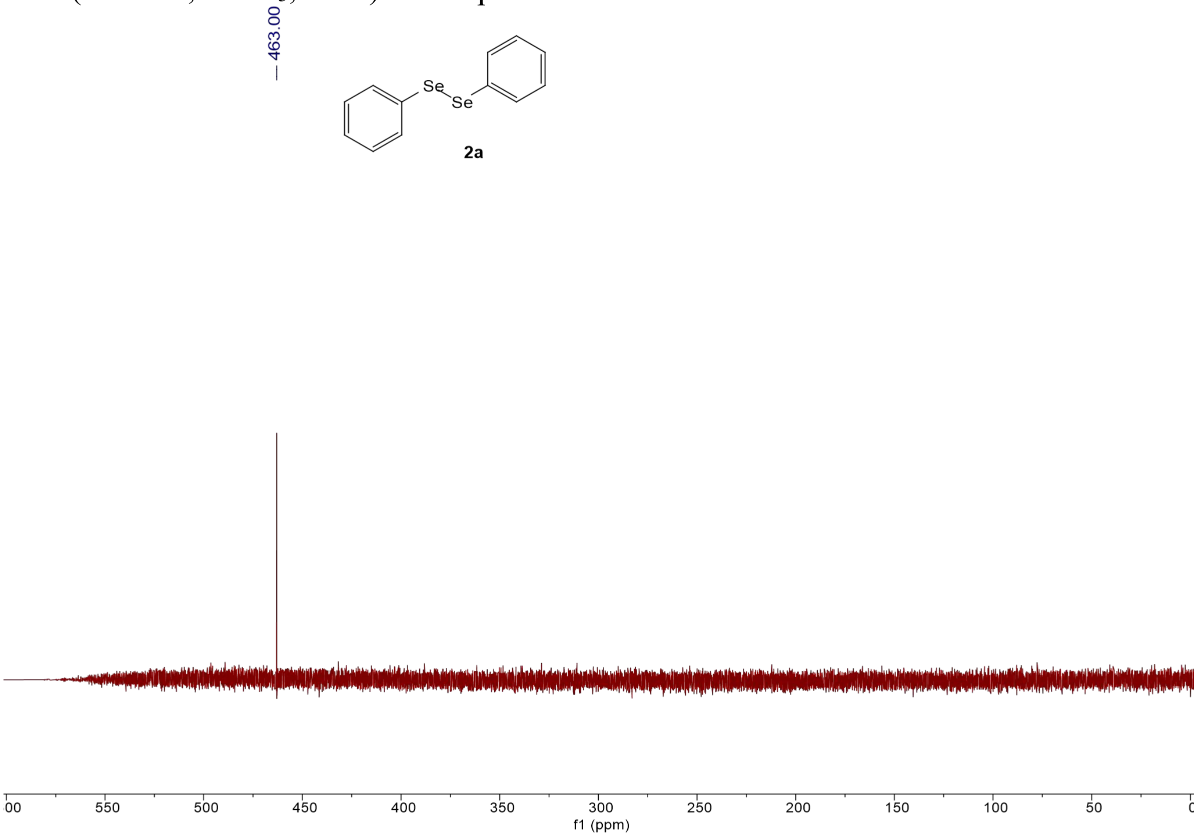

Supplementary Fig. 6. NMR spectra of compound **2a**

^{1}H NMR (400 MHz, $CDCl_3$, 25°C) of compound **2b**

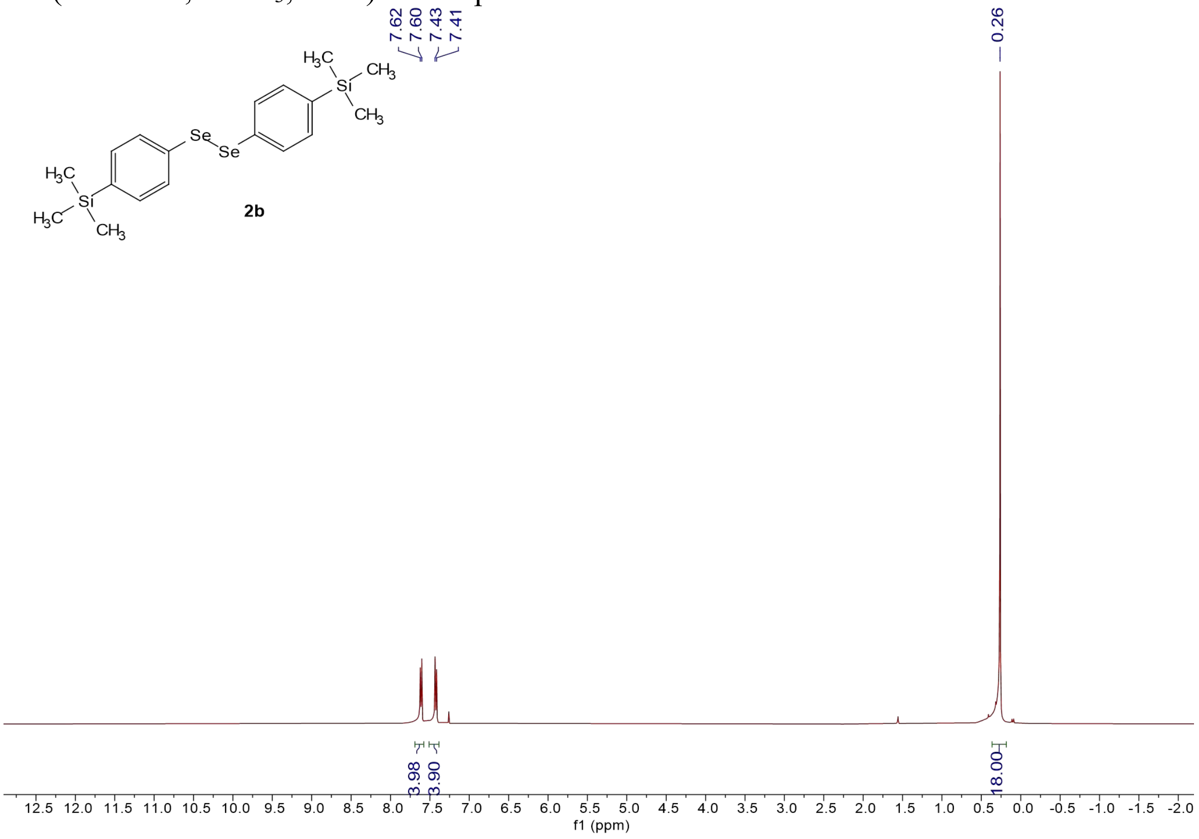

^{13}C NMR (101 MHz, $CDCl_3$, 25°C) of compound **2b**

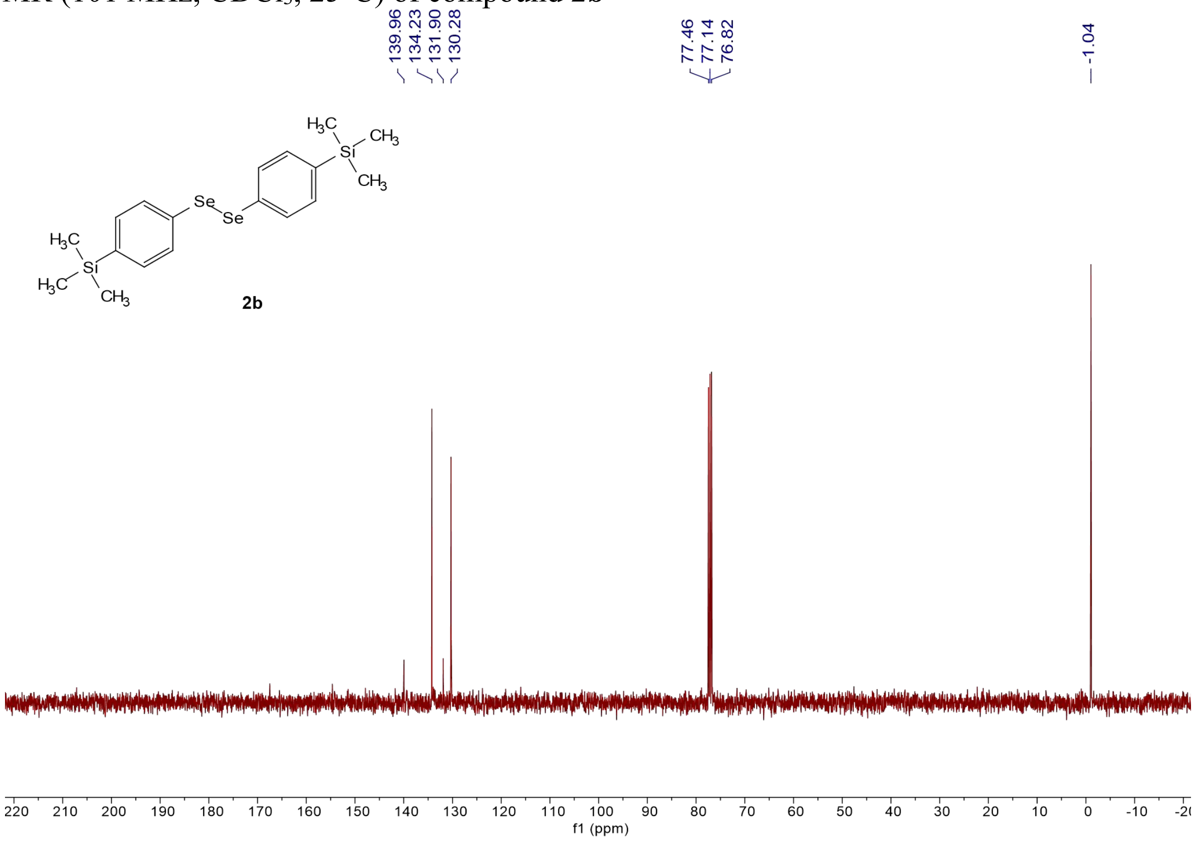

^{77}Se NMR (76 MHz, $CDCl_3$, 25°C) of compound **2b**

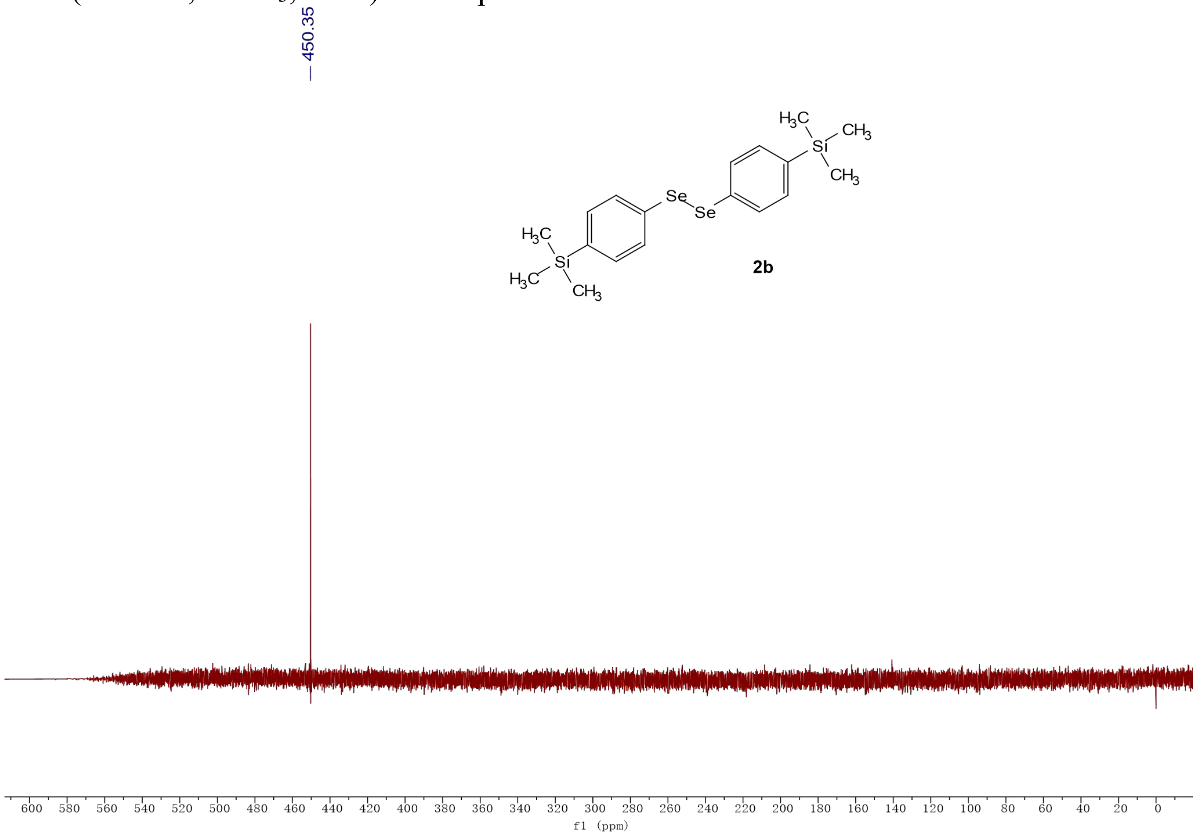

Supplementary Fig. 7. NMR spectra of compound **2b**

^{1}H NMR (400 MHz, $CDCl_3$, 25°C) of compound **2c**

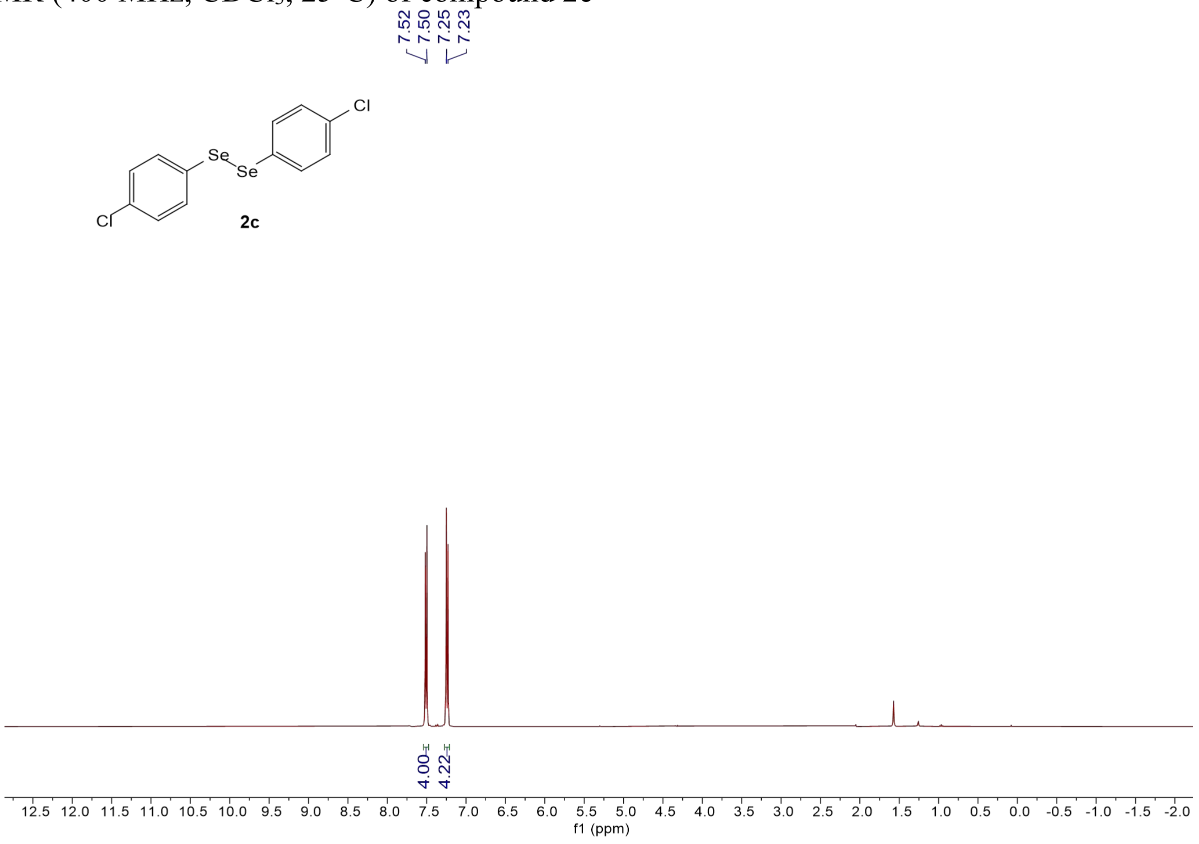

^{13}C NMR (101 MHz, $CDCl_3$, 25°C) of compound **2c**

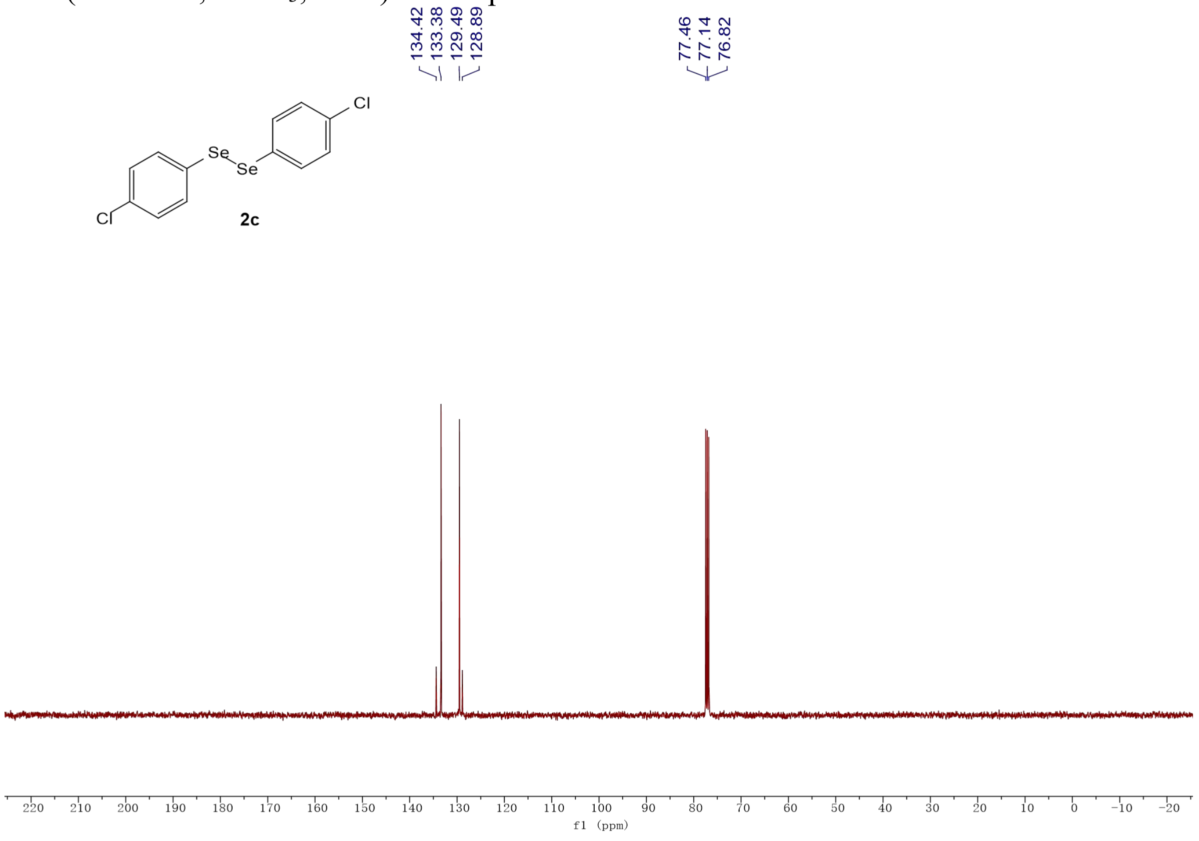

^{77}Se NMR (76 MHz, $CDCl_3$, 25°C) of compound **2c**

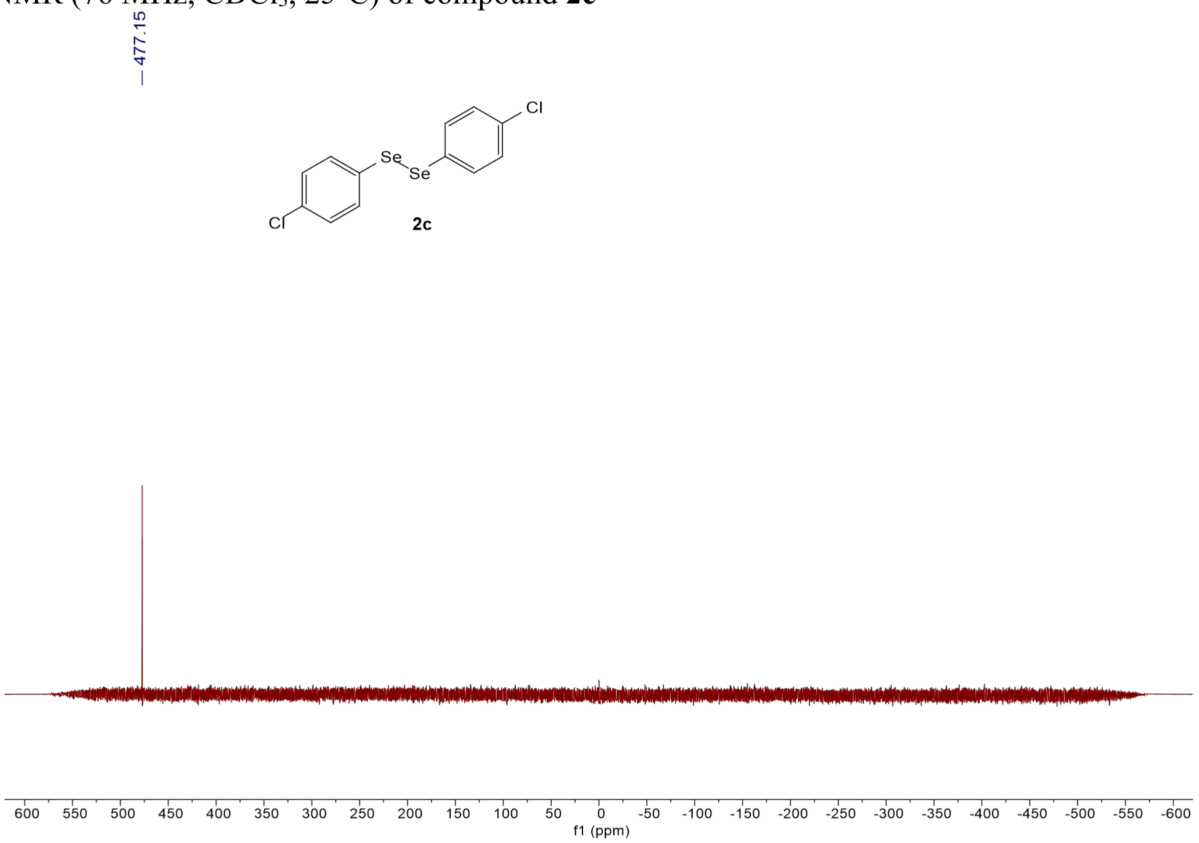

Supplementary Fig. 8. NMR spectra of compound **2c**

^{1}H NMR (400 MHz, $CDCl_3$, 25°C) of compound **2d**

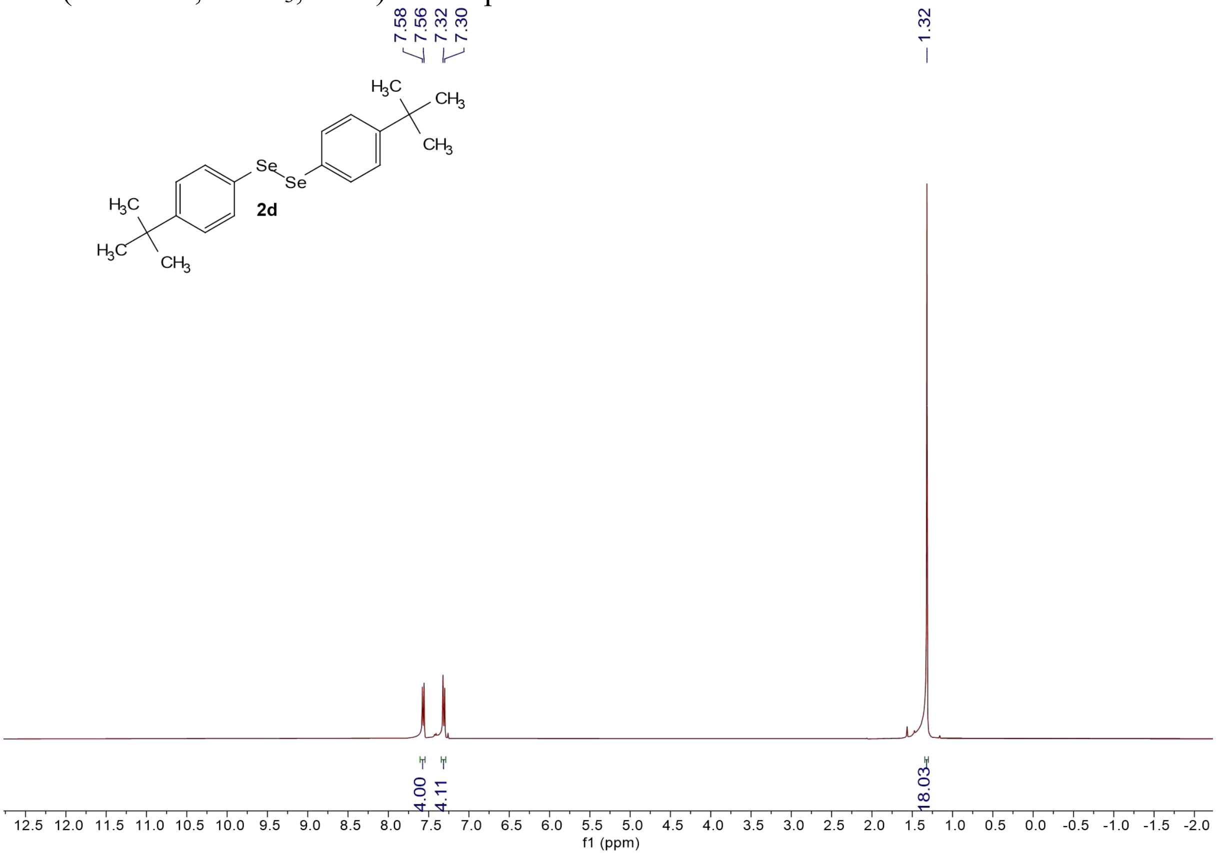

^{13}C NMR (101 MHz, $CDCl_3$, 25°C) of compound **2d**

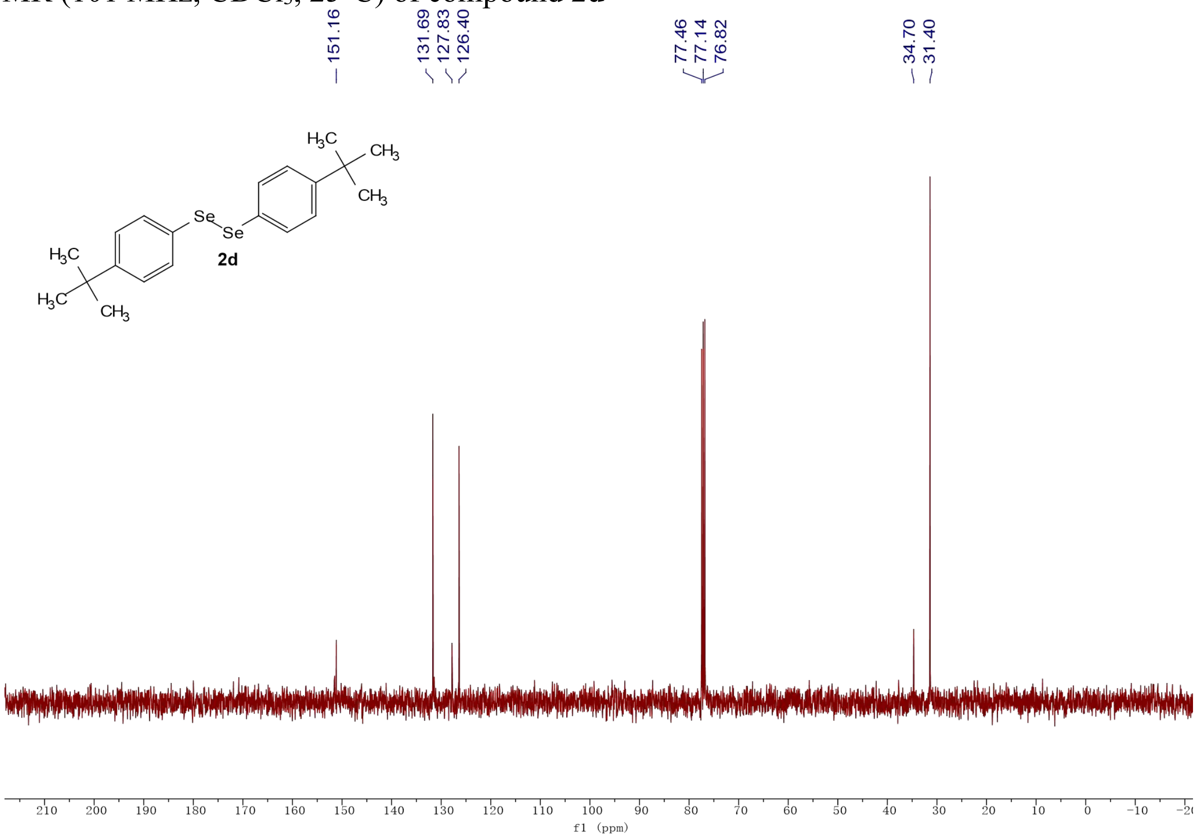

^{77}Se NMR (76 MHz, $CDCl_3$, 25°C) of compound **2d**

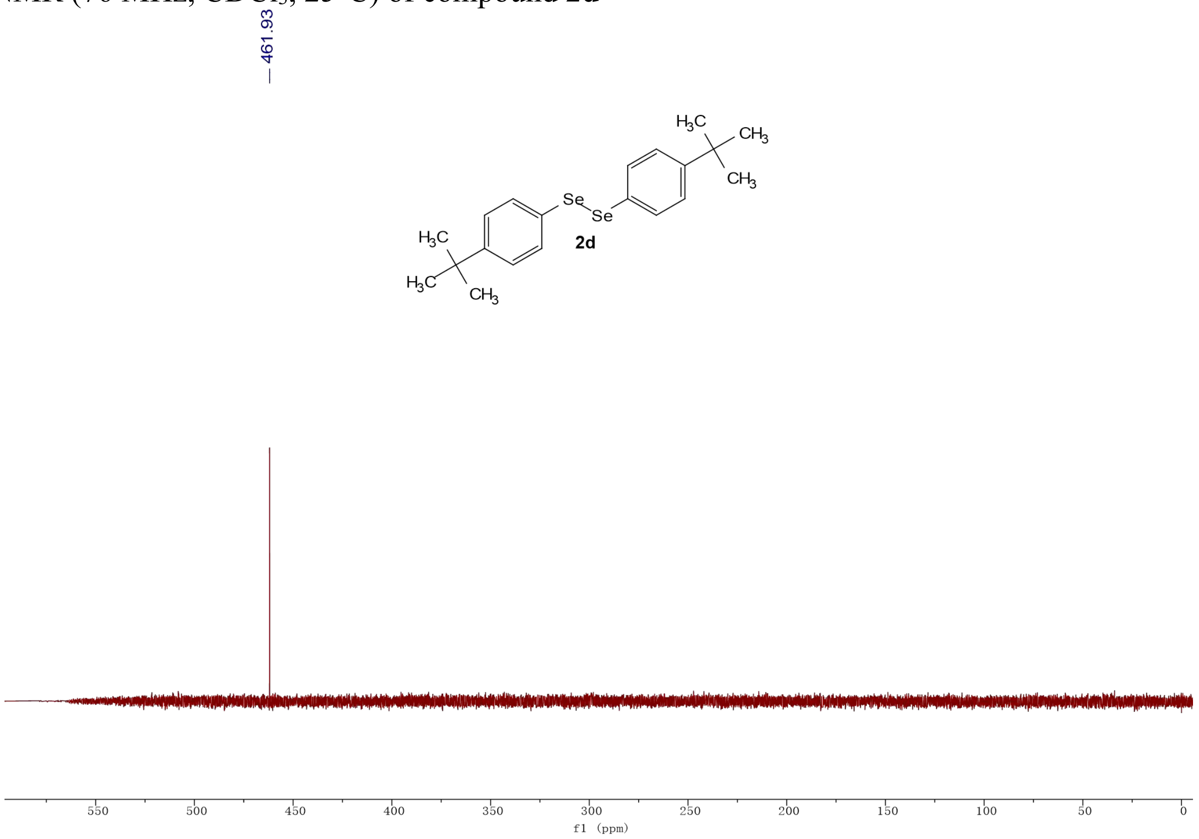

Supplementary Fig. 9. NMR spectra of compound **2d**

^{1}H NMR (400 MHz, $CDCl_3$, 25°C) of compound **2e**

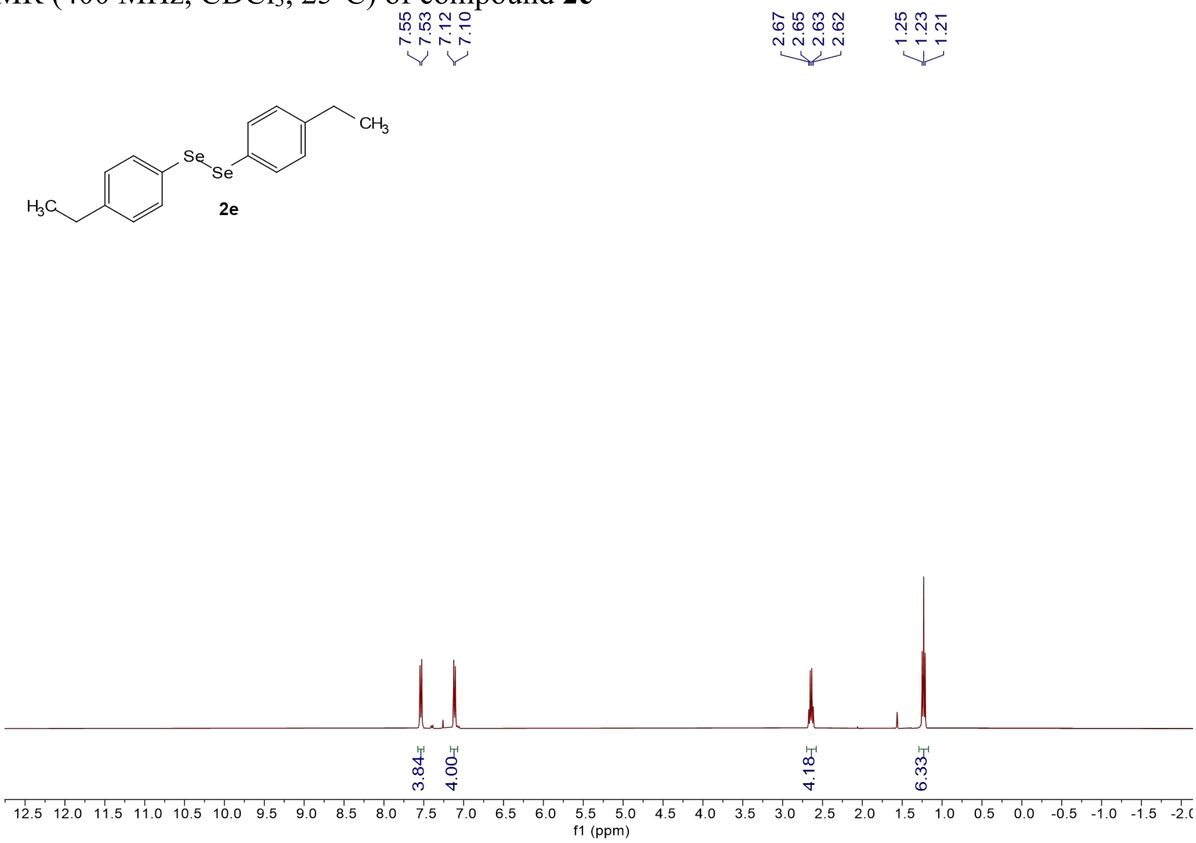

^{13}C NMR (101 MHz, CDCl3, 25°C) of compound **2e**

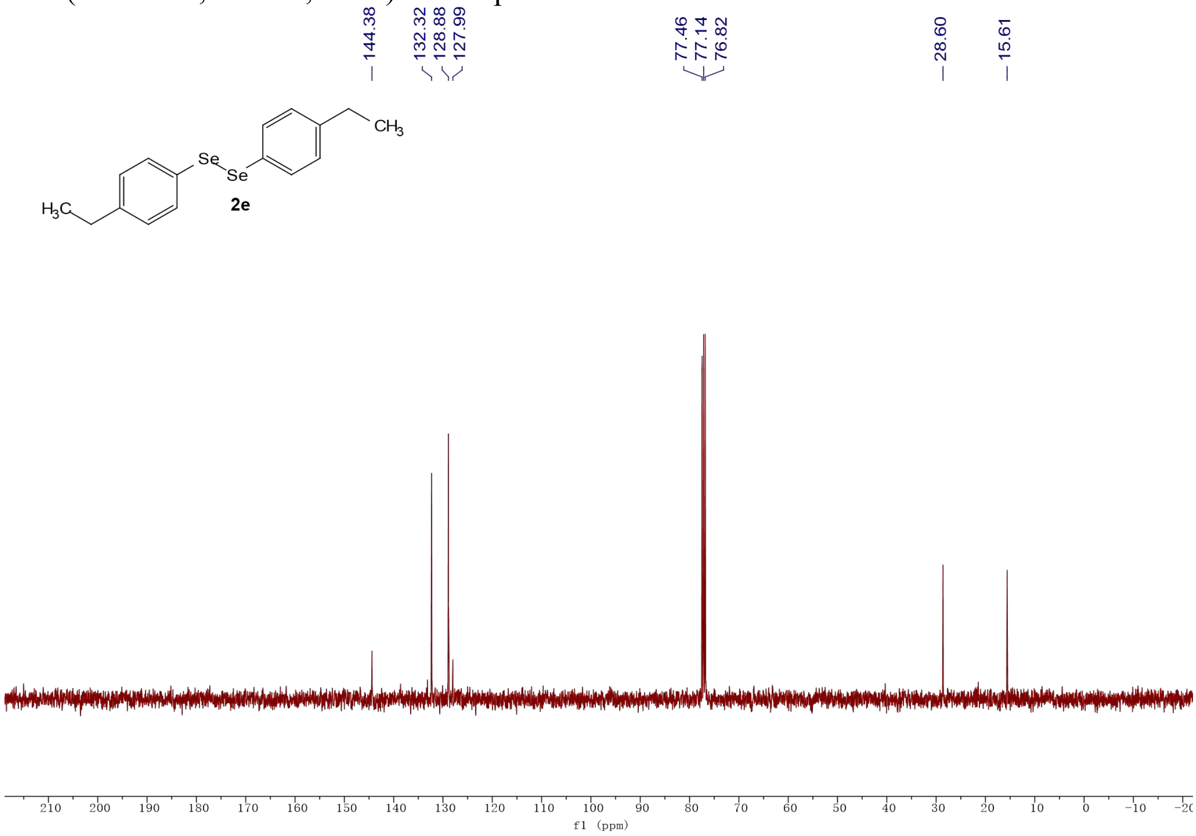

^{77}Se NMR (76 MHz, $CDCl_3$, 25°C) of compound **2e**

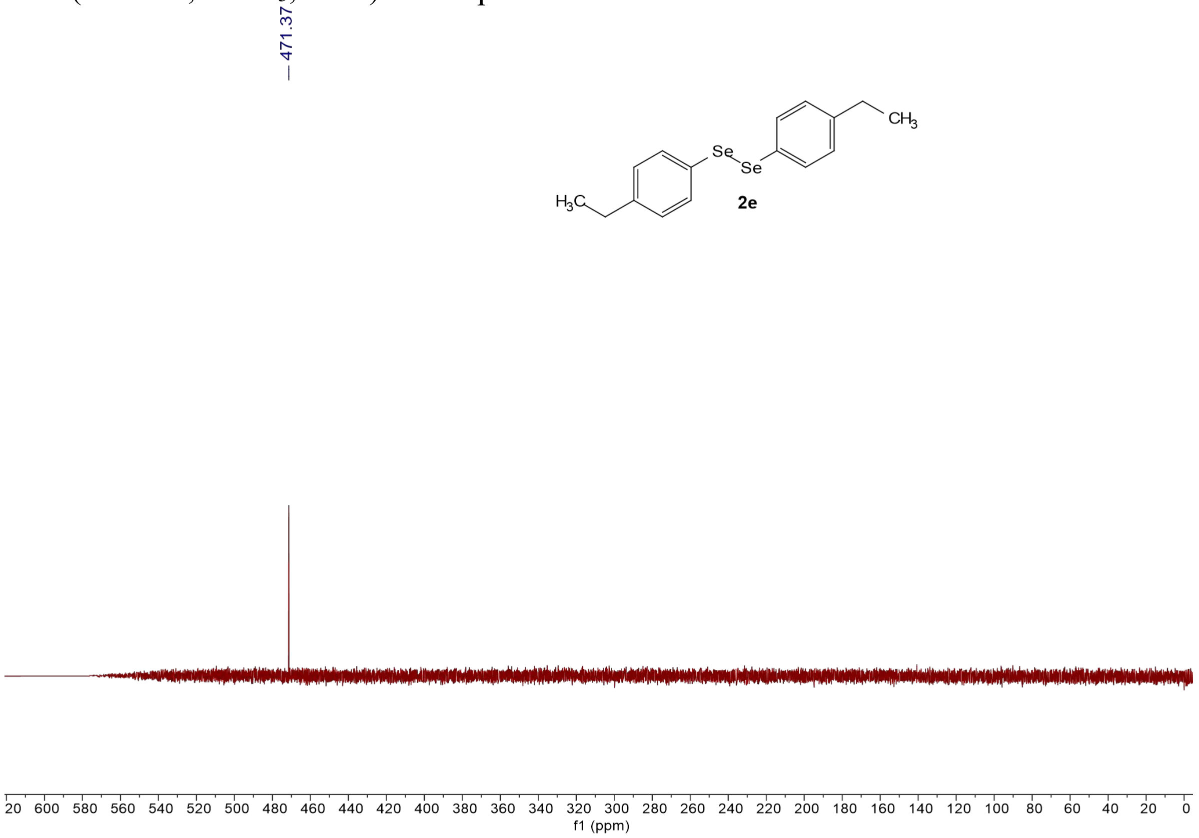

Supplementary Fig. 10. NMR spectra of compound **2e**

^{1}H NMR (400 MHz, $CDCl_3$, 25°C) of compound **2f**

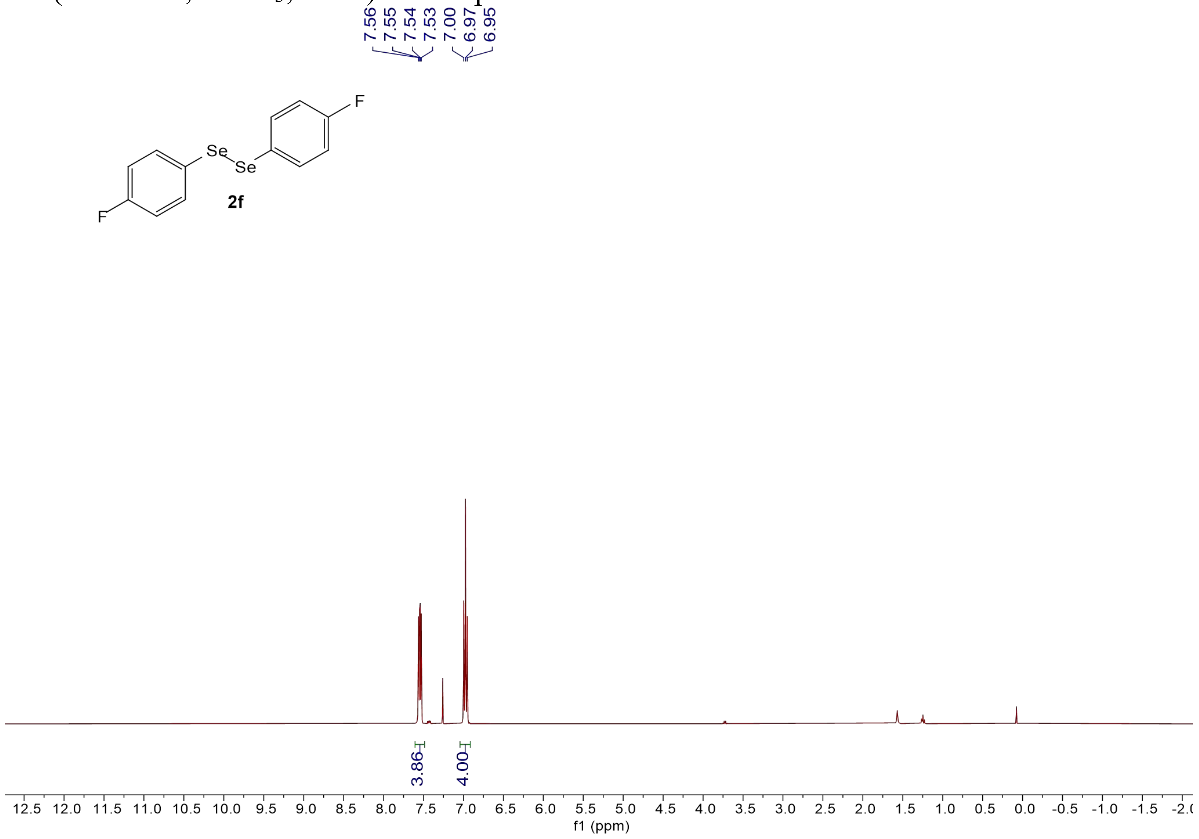

^{13}C NMR (101 MHz, $CDCl_3$, 25°C) of compound **2f**

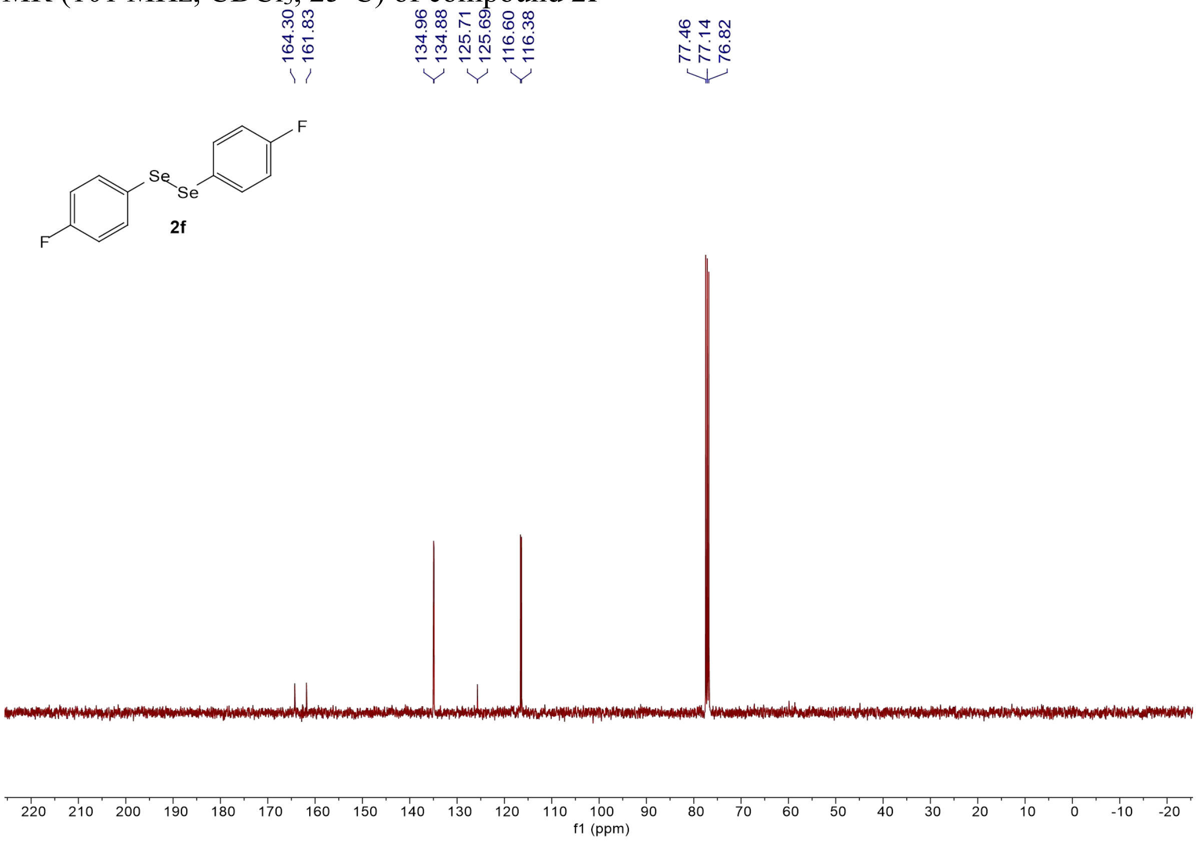

^{77}Se NMR (76 MHz, $CDCl_3$, 25°C) of compound **2f**

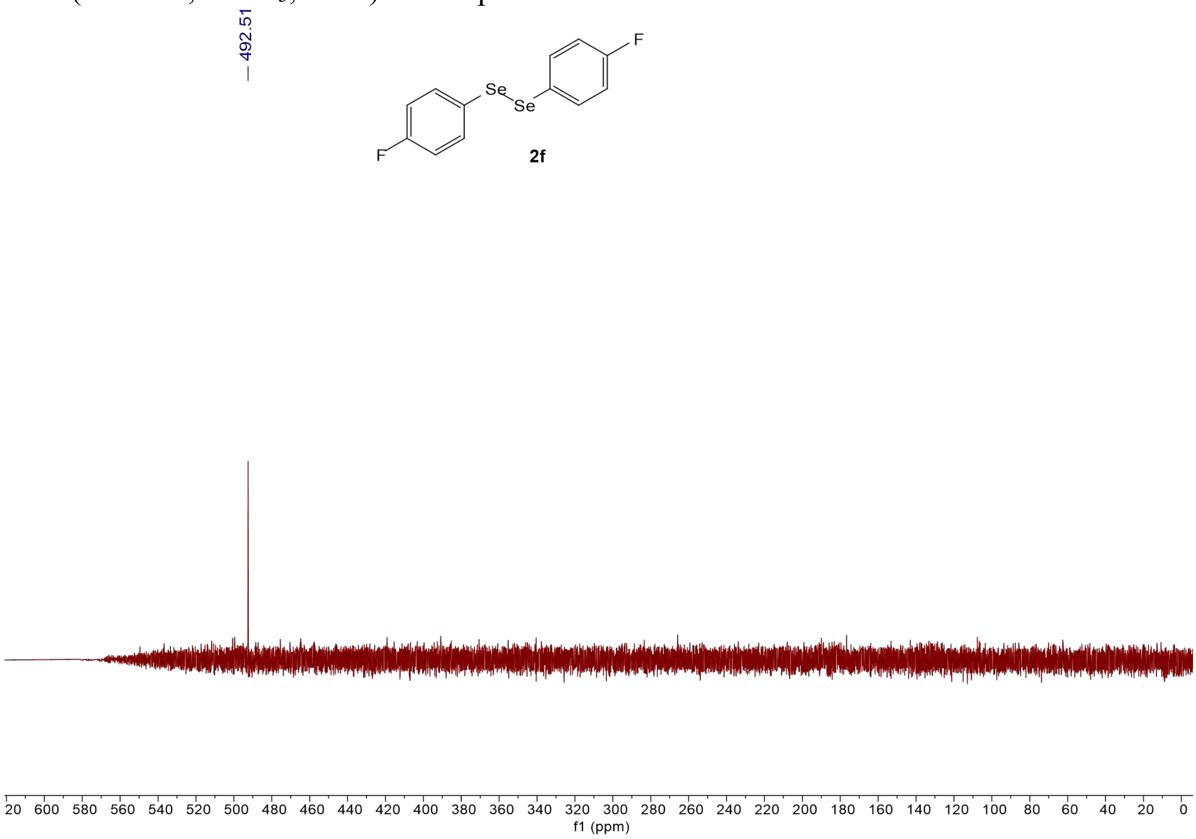

^{19}**F NMR** (376 MHz, $CDCl_3$, 25°C) of compound **2f**

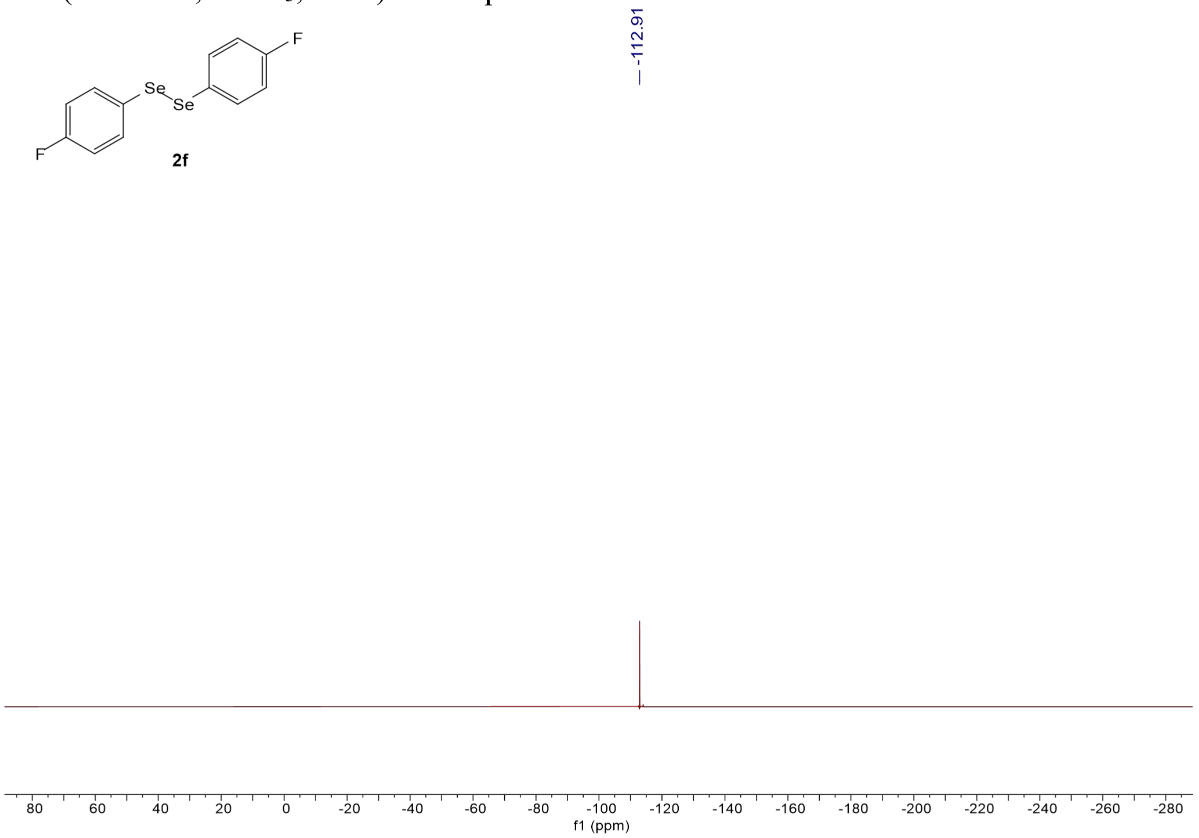

Supplementary Fig. 11. NMR spectra of compound **2f**

^{1}**H NMR** (400 MHz, $CDCl_3$, 25°C) of compound **2g**

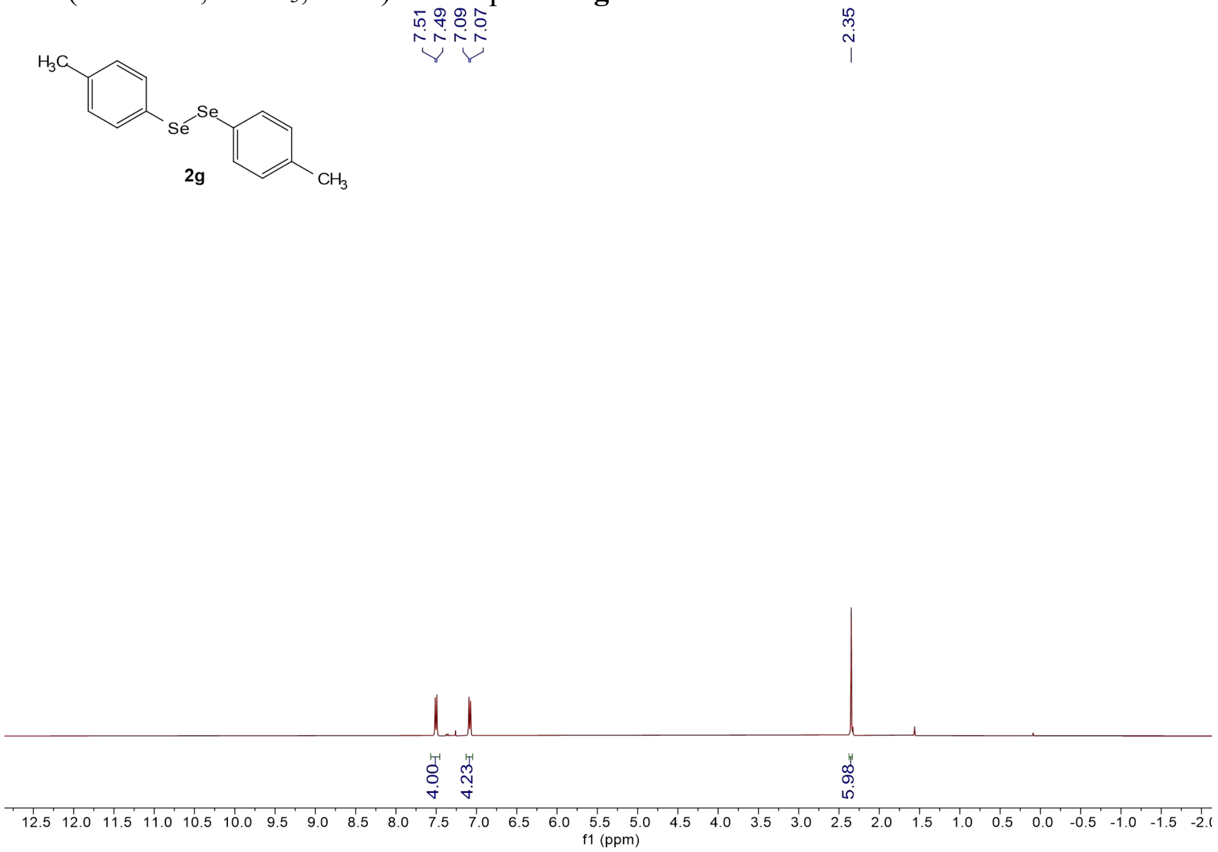

^{13}C NMR (101 MHz, $CDCl_3$, 25°C) of compound **2g**

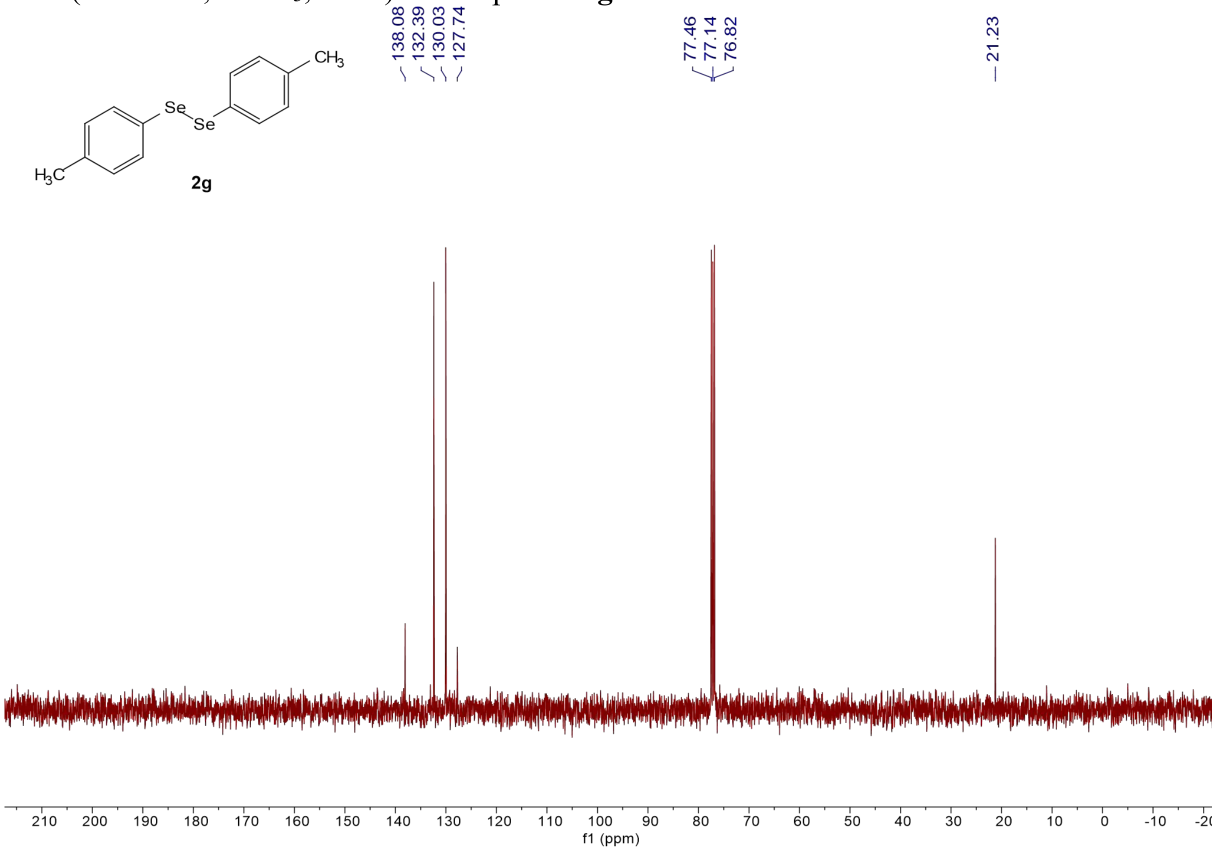

^{77}Se NMR (76 MHz, $CDCl_3$, 25°C) of compound **2g**

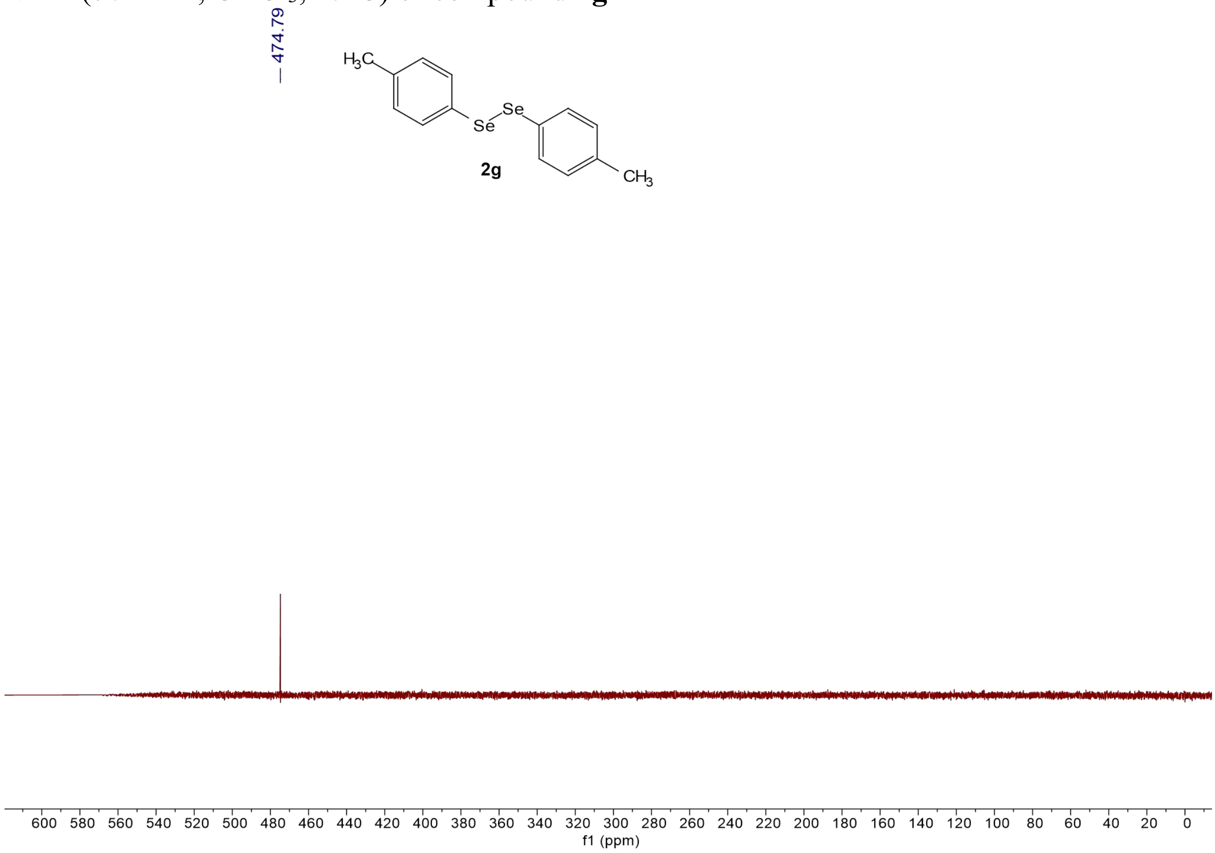

Supplementary Fig. 12. NMR spectra of compound **2g**

^{1}H NMR (400 MHz, $CDCl_3$, 25℃) of compound **2h**

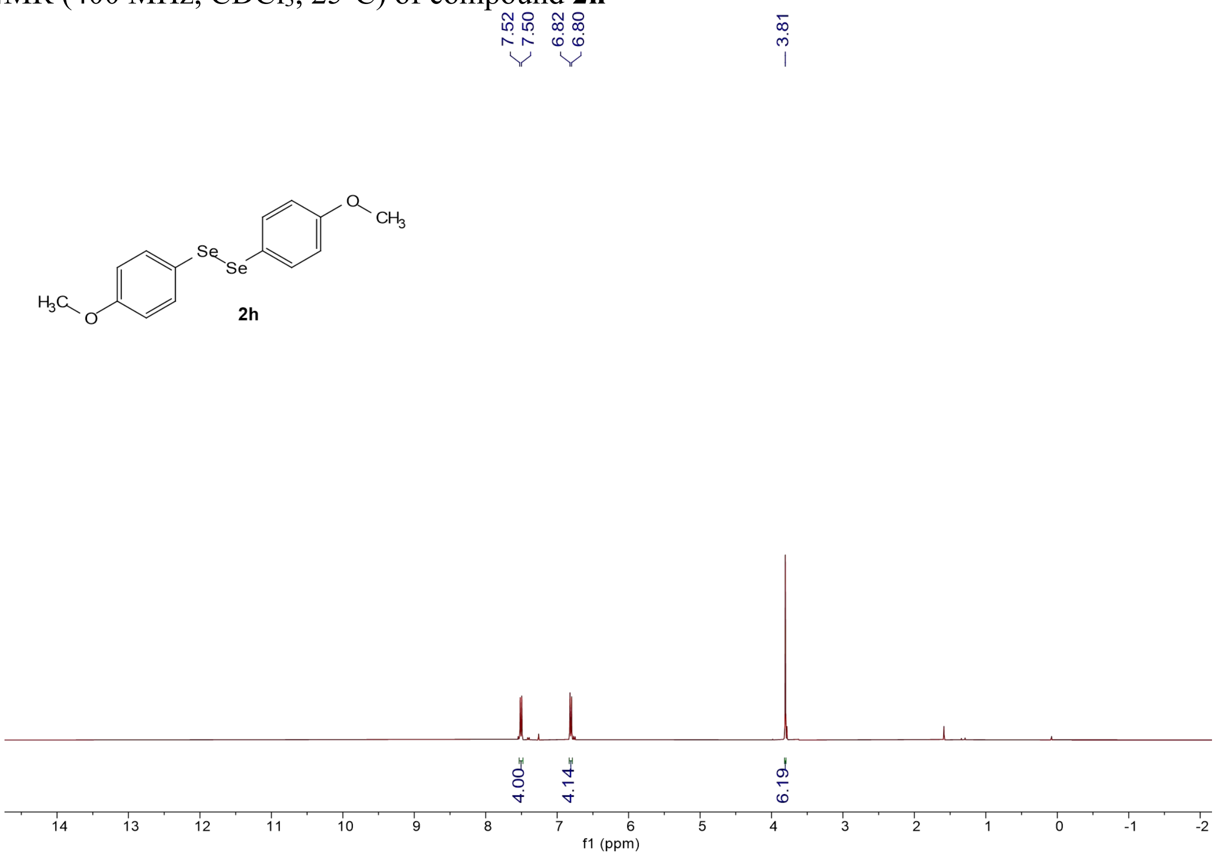

^{13}C NMR (101 MHz, $CDCl_3$, 25℃) of compound **2h**

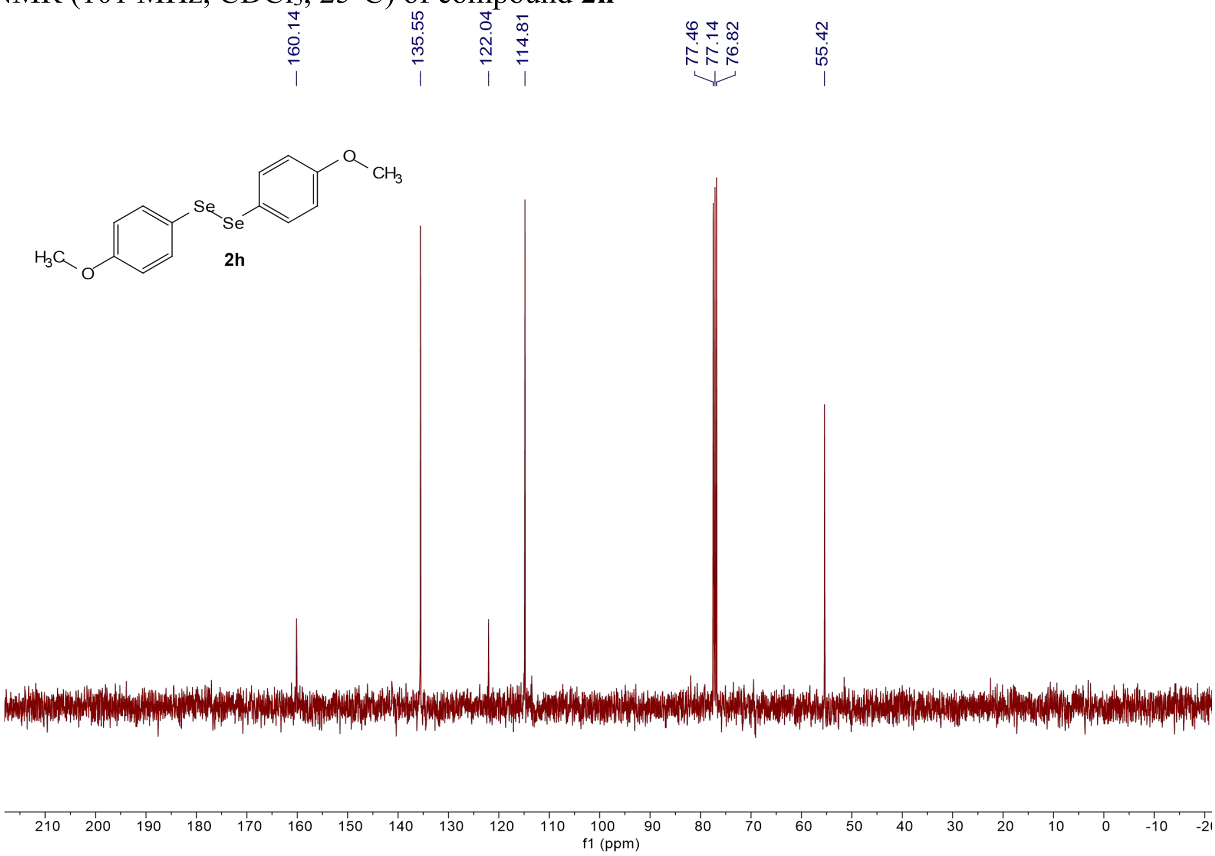

^{77}Se NMR (76 MHz, $CDCl_3$, 25°C) of compound **2h**

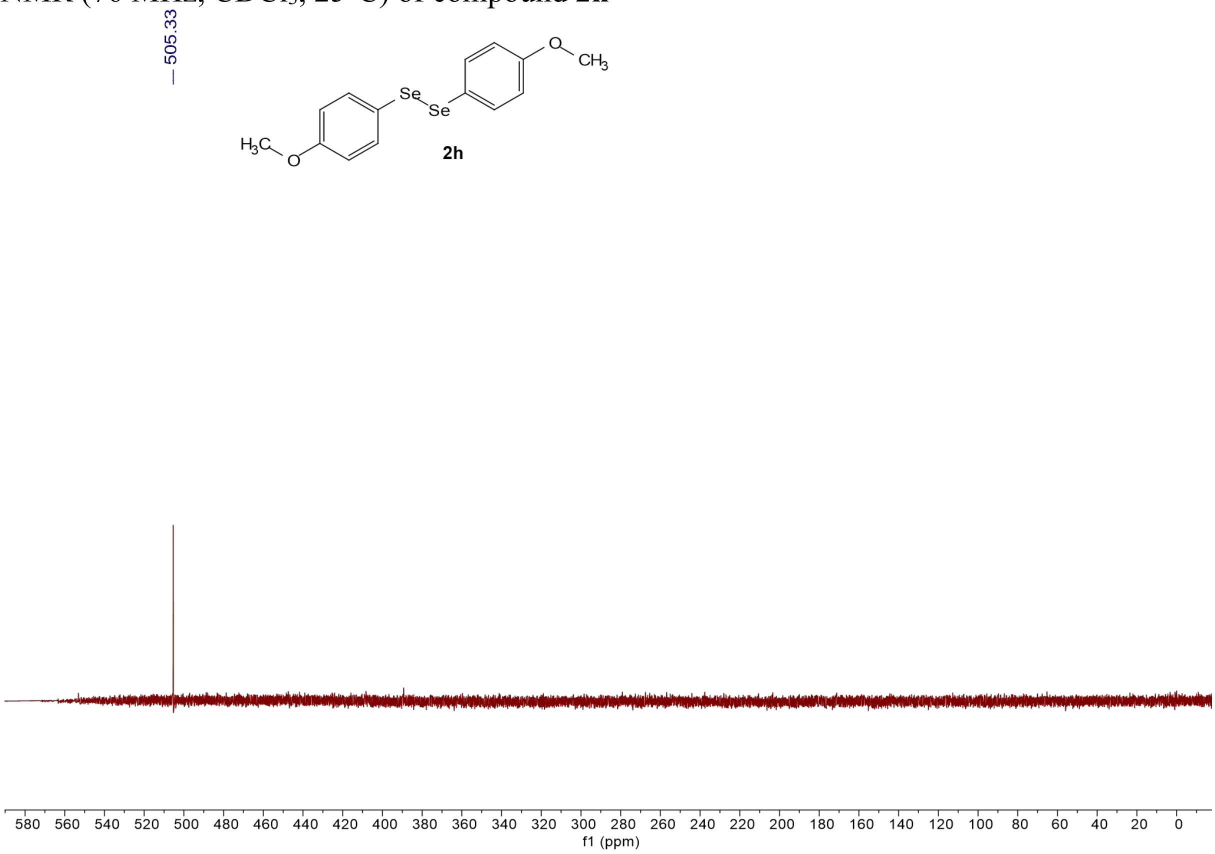

Supplementary Fig. 13. NMR spectra of compound **2h**

^{1}H NMR (400 MHz, $CDCl_3$, 25°C) of compound **2i**

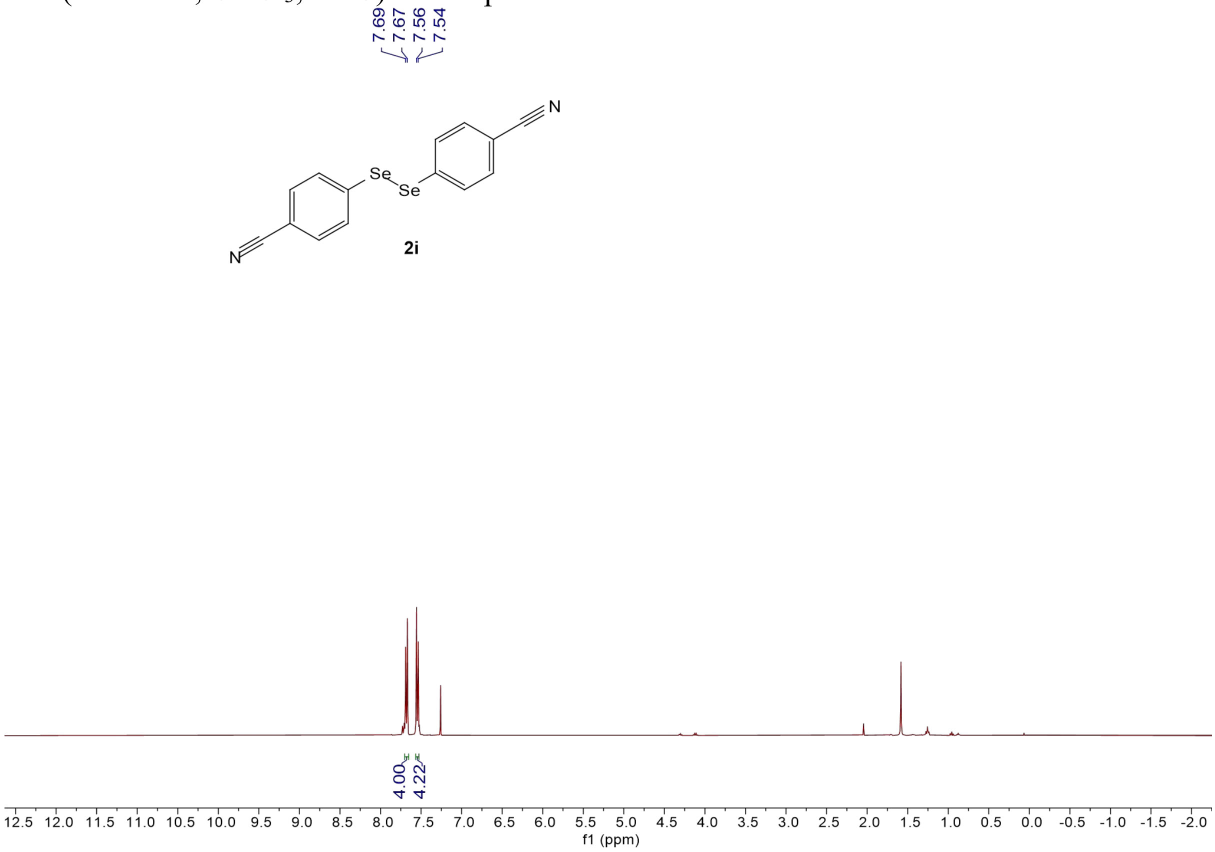

^{13}C NMR (101 MHz, $CDCl_3$, 25°C) of compound **2i**

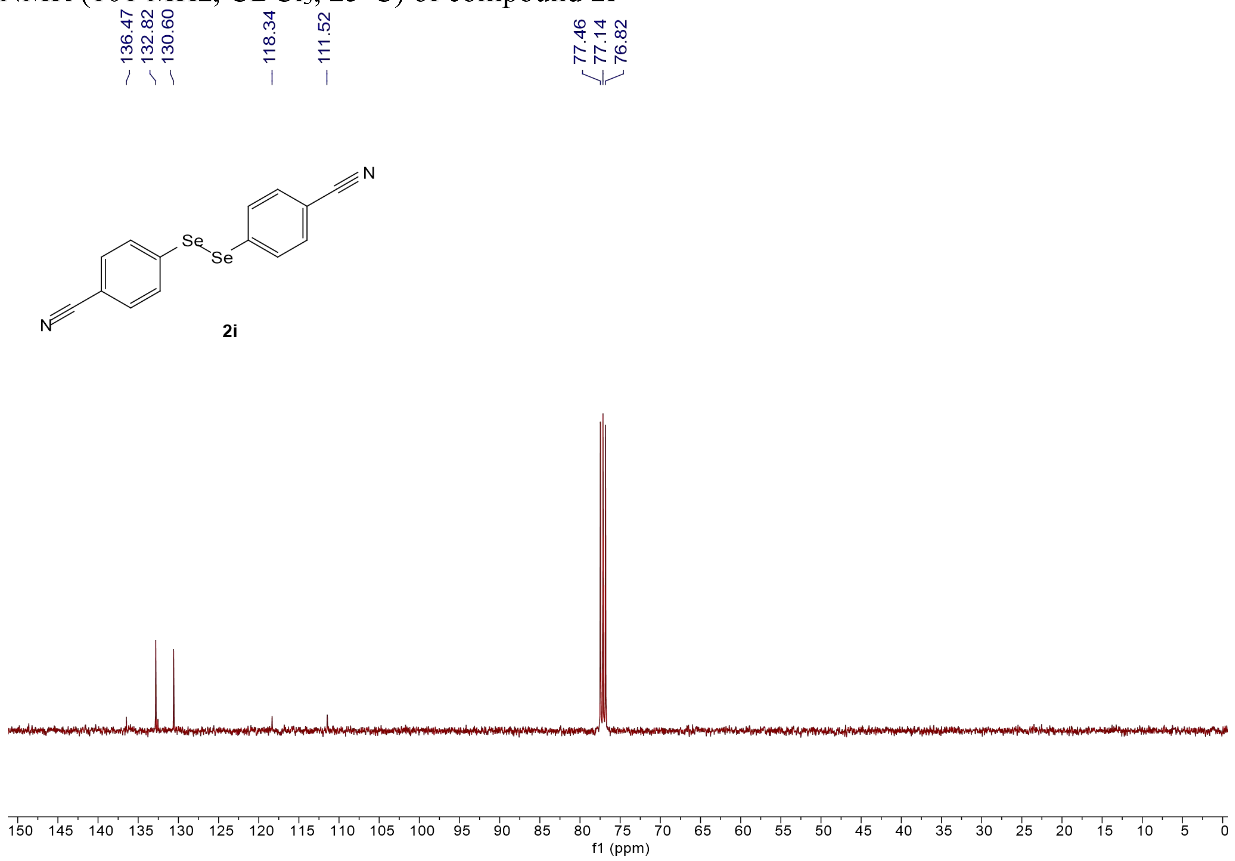

^{77}Se NMR (76 MHz, $CDCl_3$, 25°C) of compound **2i**

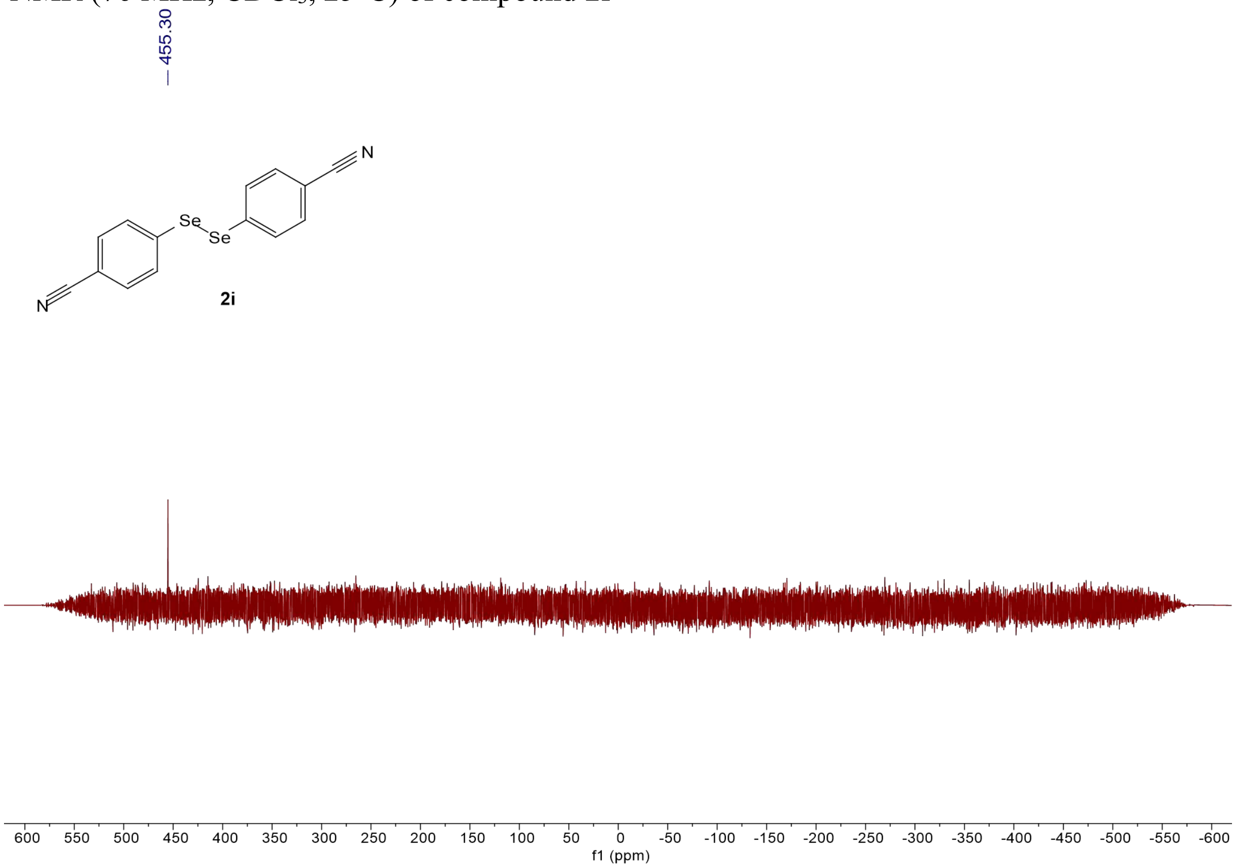

Supplementary Fig. 14. NMR spectra of compound **2i**

^{1}H NMR (400 MHz, $CDCl_3$, 25°C) of compound **2j**

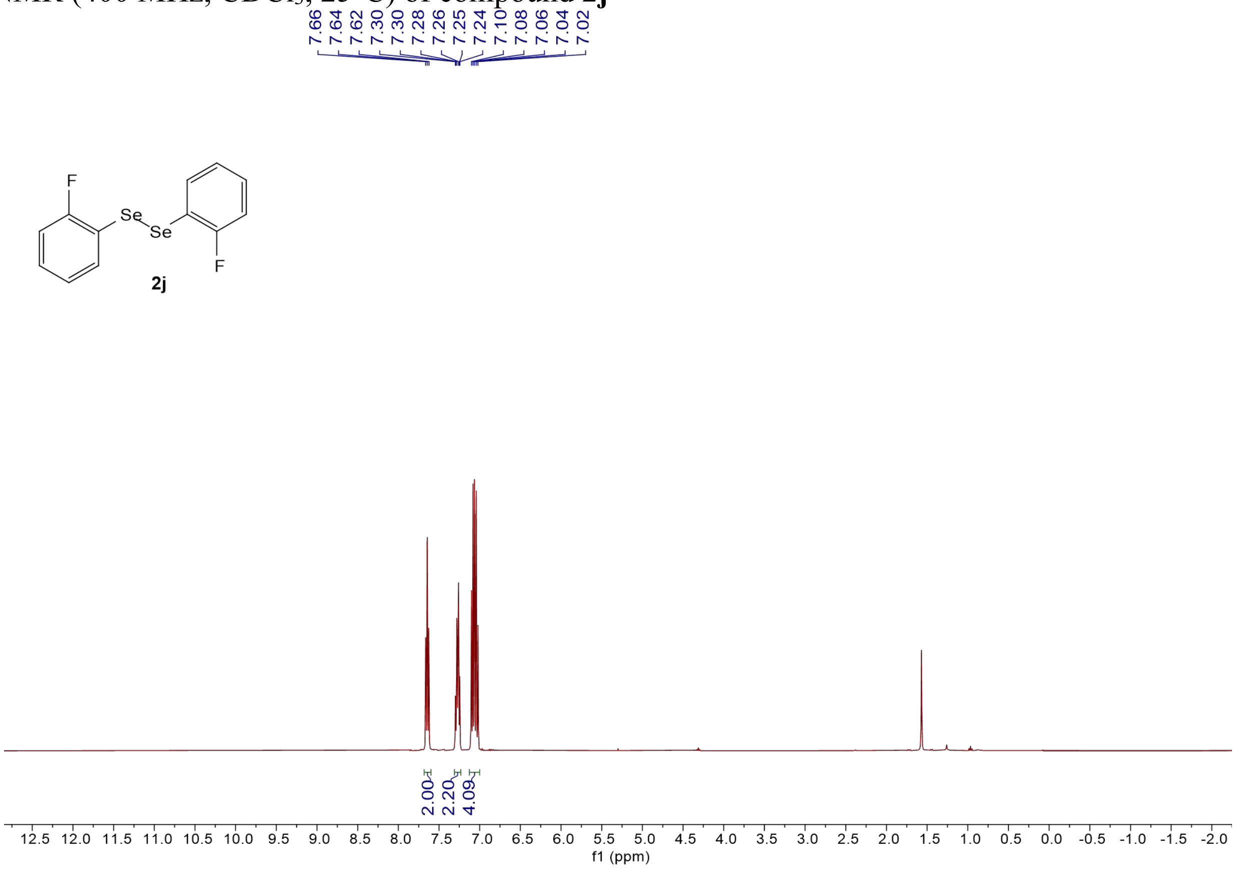

^{13}C NMR (101 MHz, $CDCl_3$, 25°C) of compound **2j**

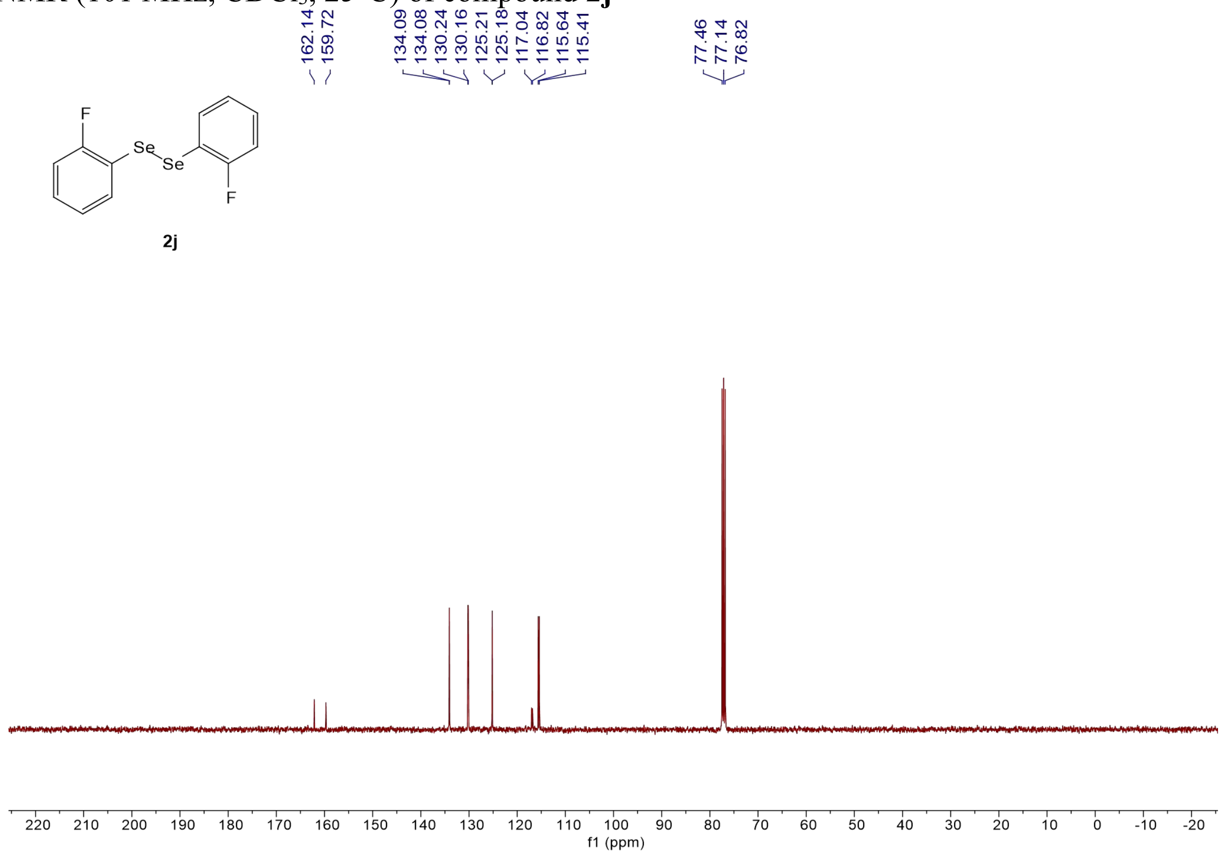

^{77}Se NMR (76 MHz, $CDCl_3$, 25°C) of compound **2j**

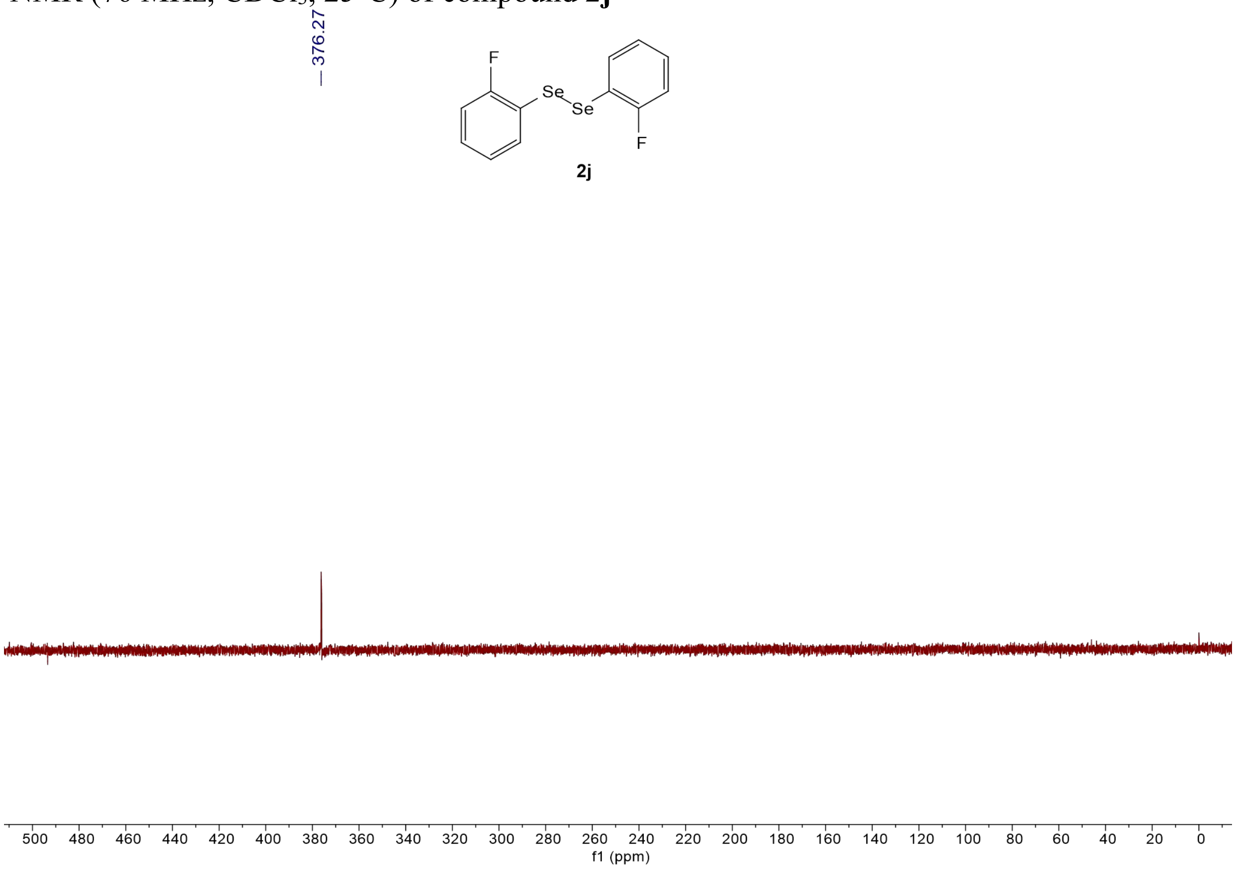

^{19}F NMR (376 MHz, $CDCl_3$, 25°C) of compound **2j**

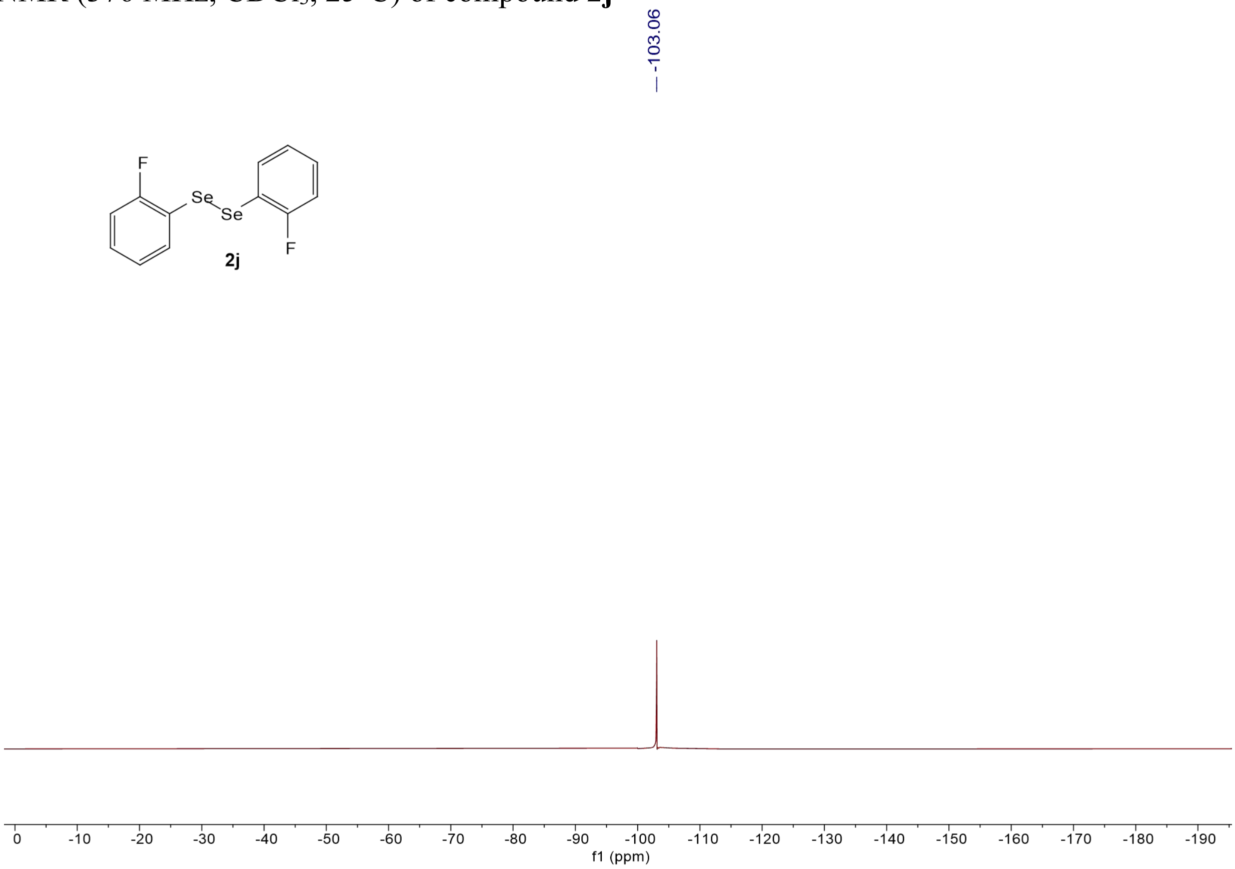

Supplementary Fig. 15. NMR spectra of compound **2j**

^{1}H NMR (400 MHz, $CDCl_3$, 25°C) of compound **2k**

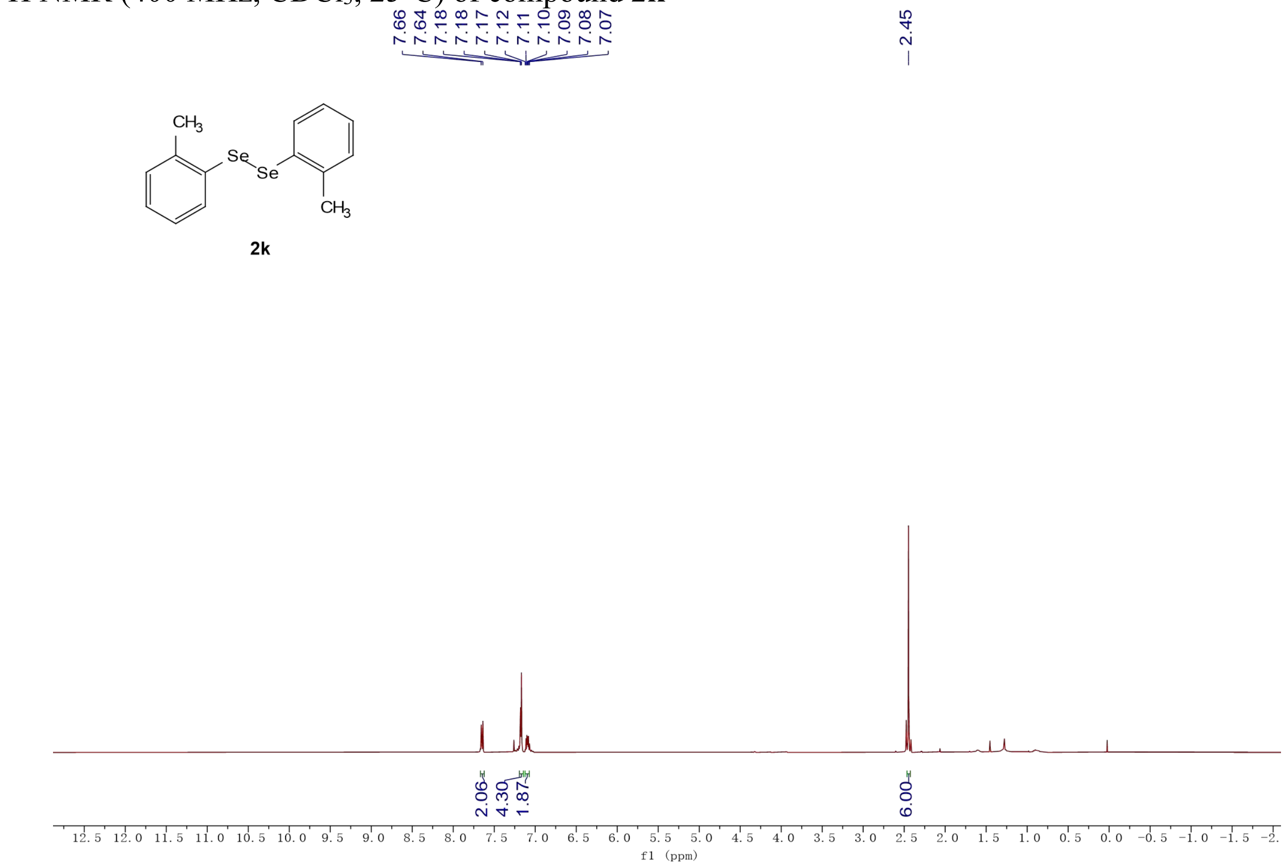

^{13}C NMR (101 MHz, $CDCl_3$, 25°C) of compound **2k**

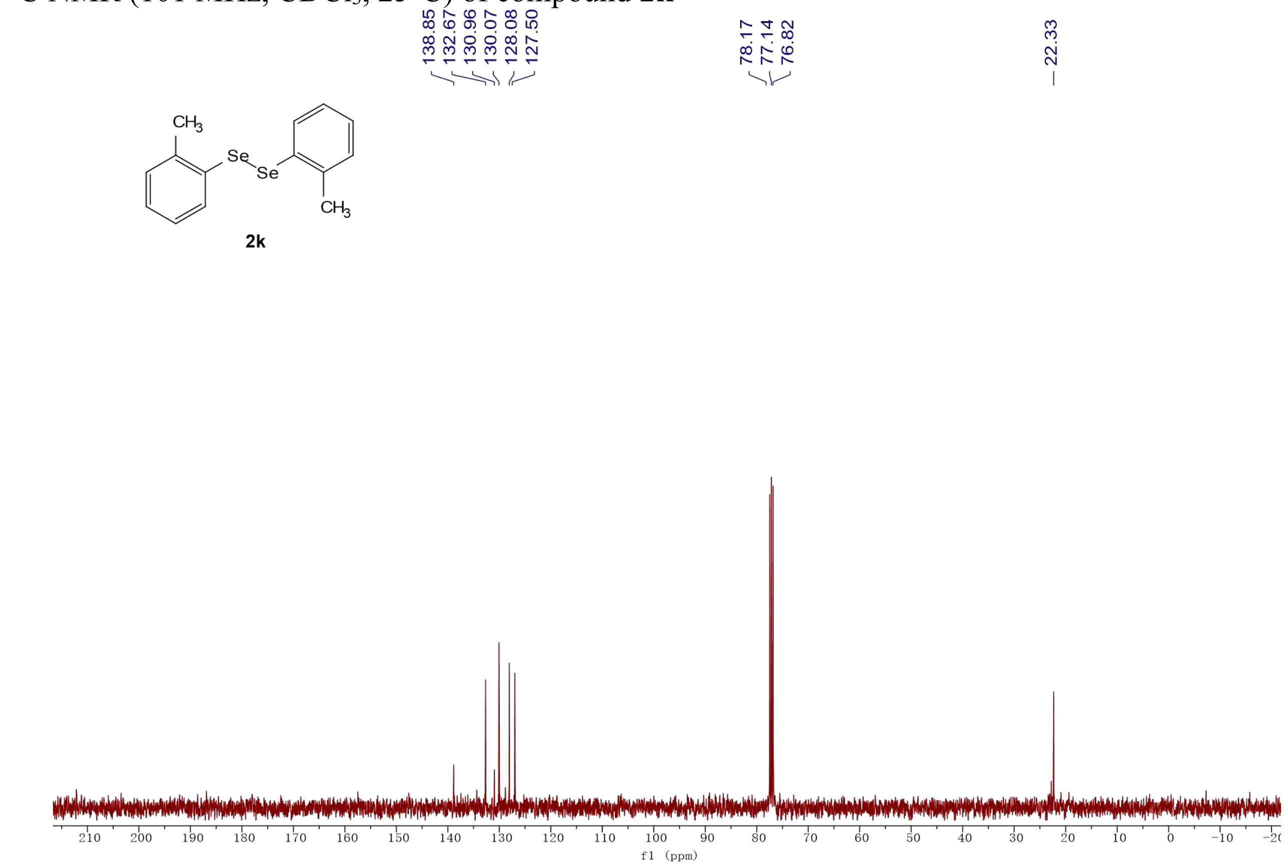

^{77}Se NMR (76 MHz, $CDCl_3$, 25°C) of compound **2k**

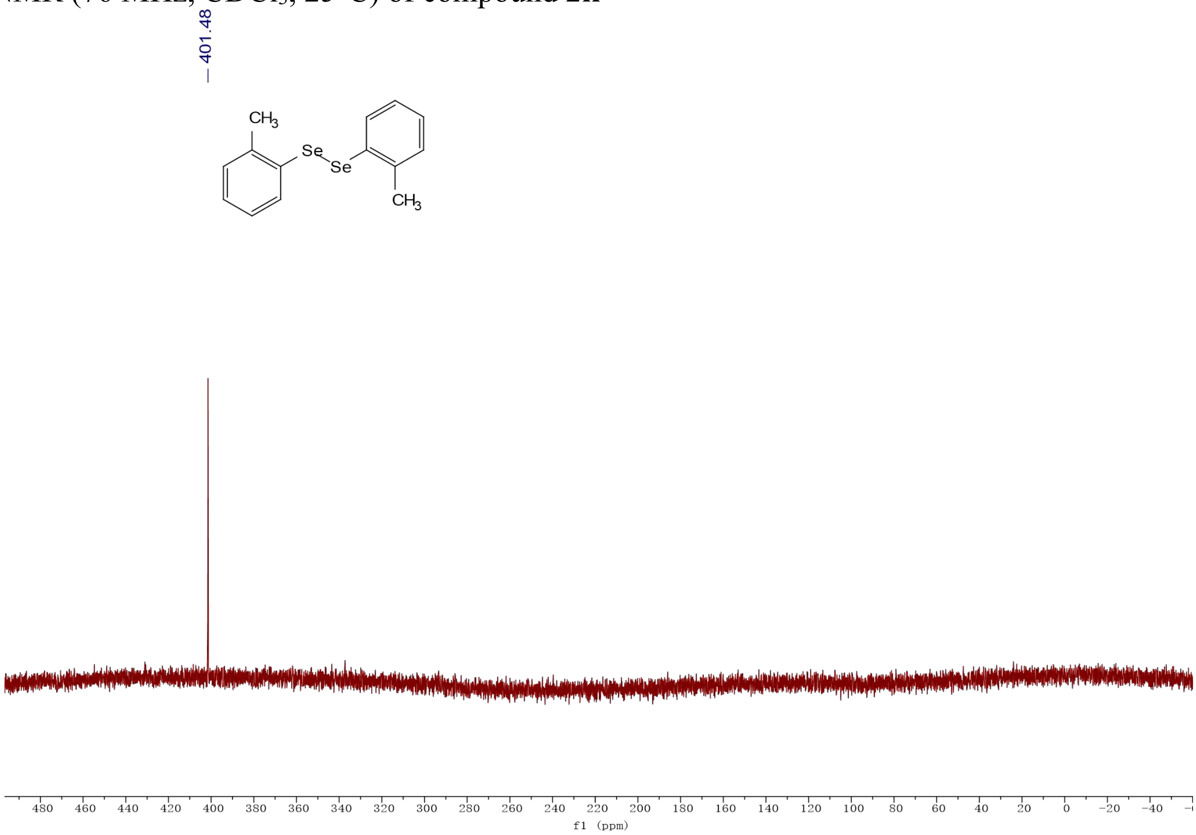

Supplementary Fig. 16. NMR spectra of compound **2k**

^{1}H NMR (400 MHz, $CDCl_3$, 25°C) of compound **2l**

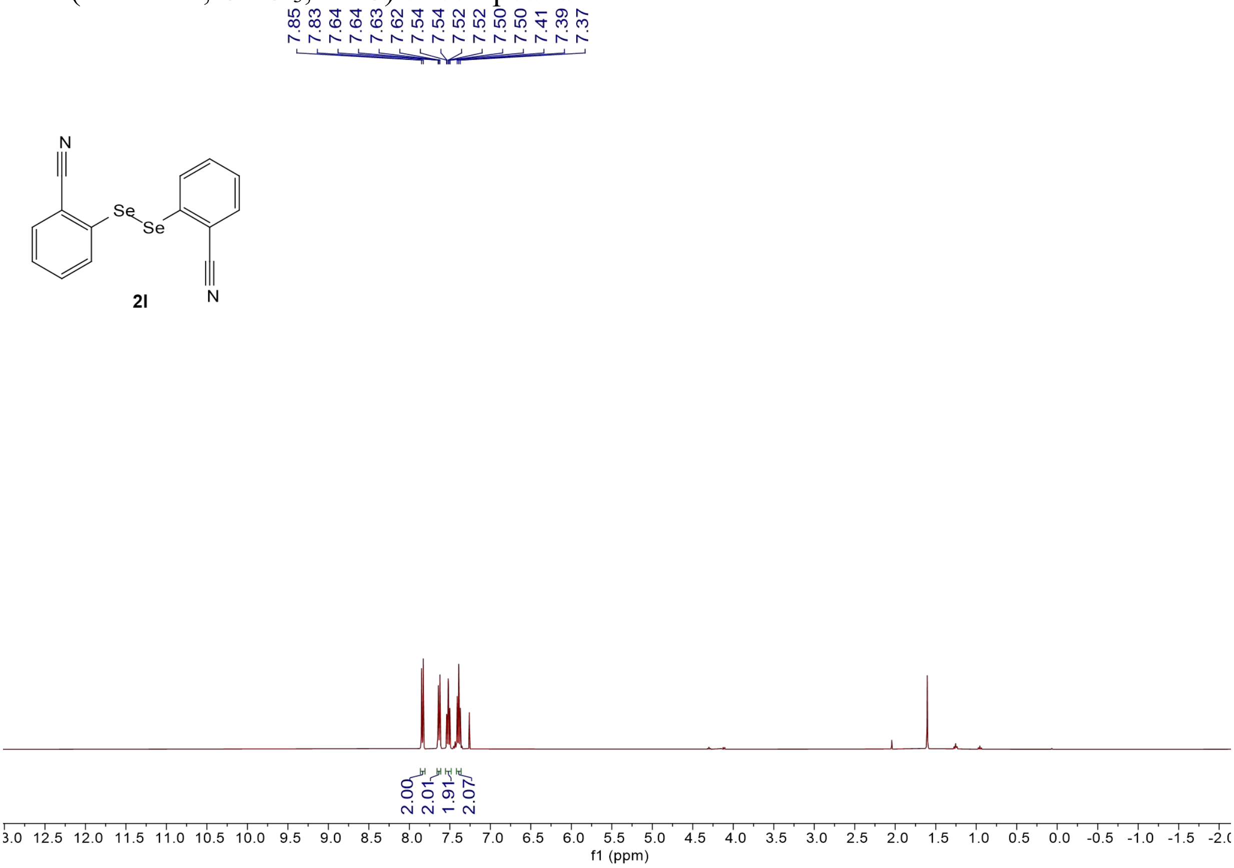

^{13}C NMR (101 MHz, $CDCl_3$, 25°C) of compound **2l**

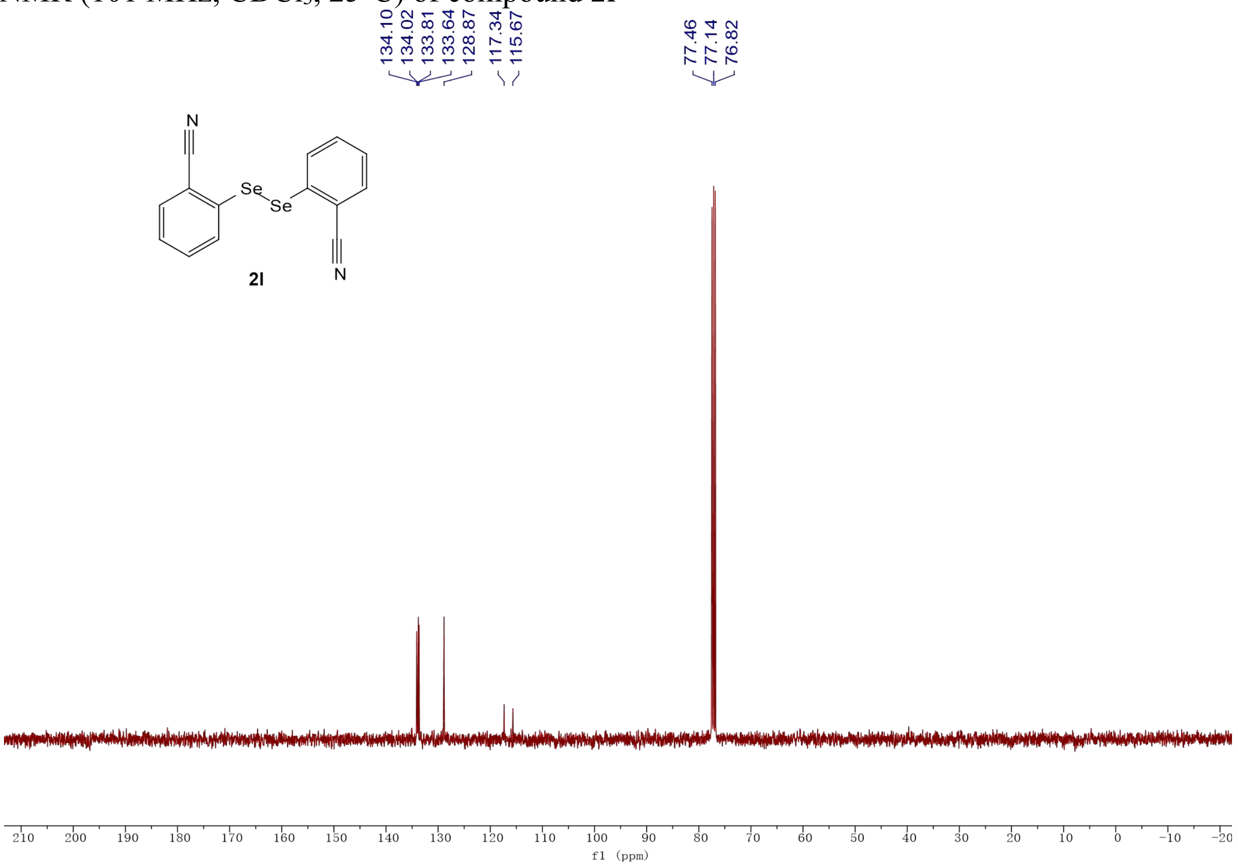

^{77}Se NMR (76 MHz, $CDCl_3$, 25°C) of compound **2l**

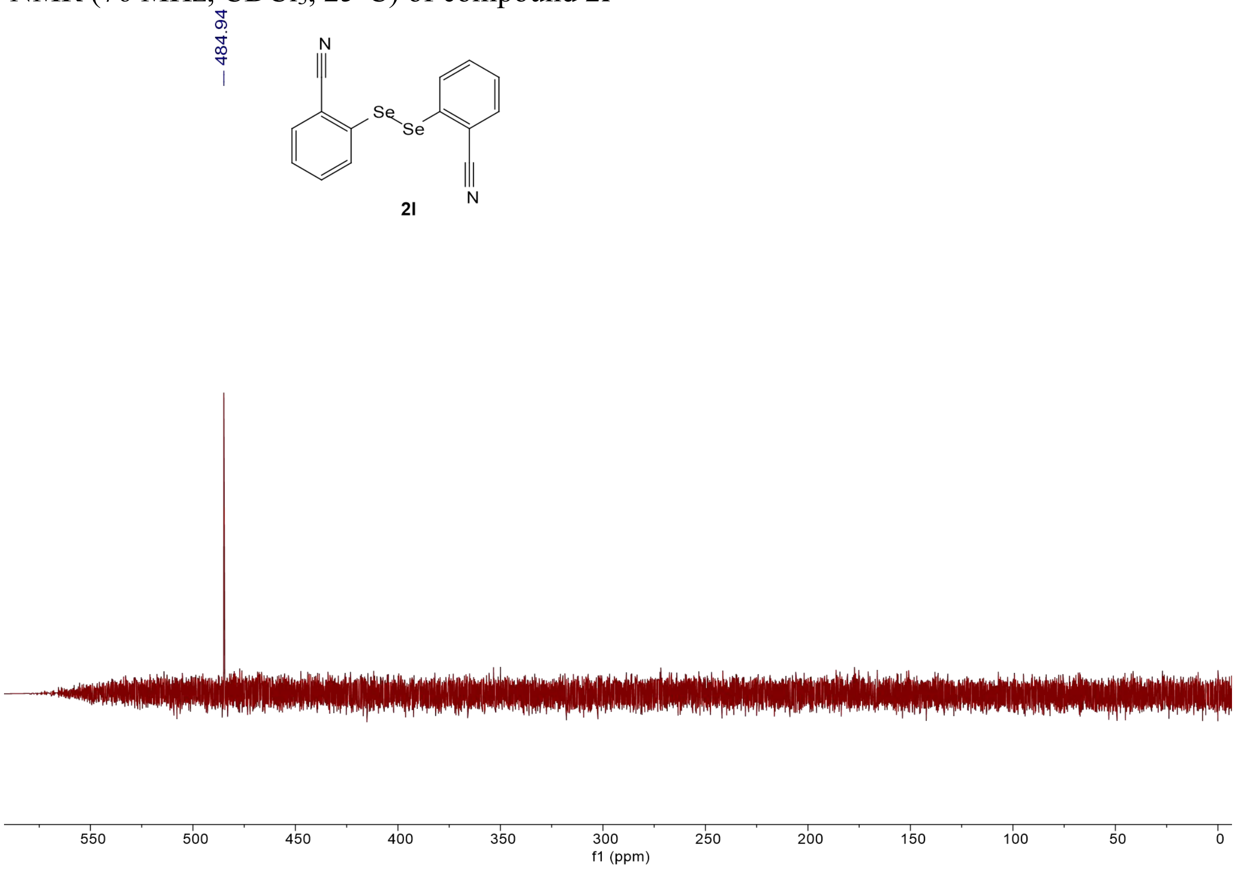

Supplementary Fig. 17. NMR spectra of compound **2l**

^{1}H NMR (400 MHz, $CDCl_3$, 25°C) of compound **2m**

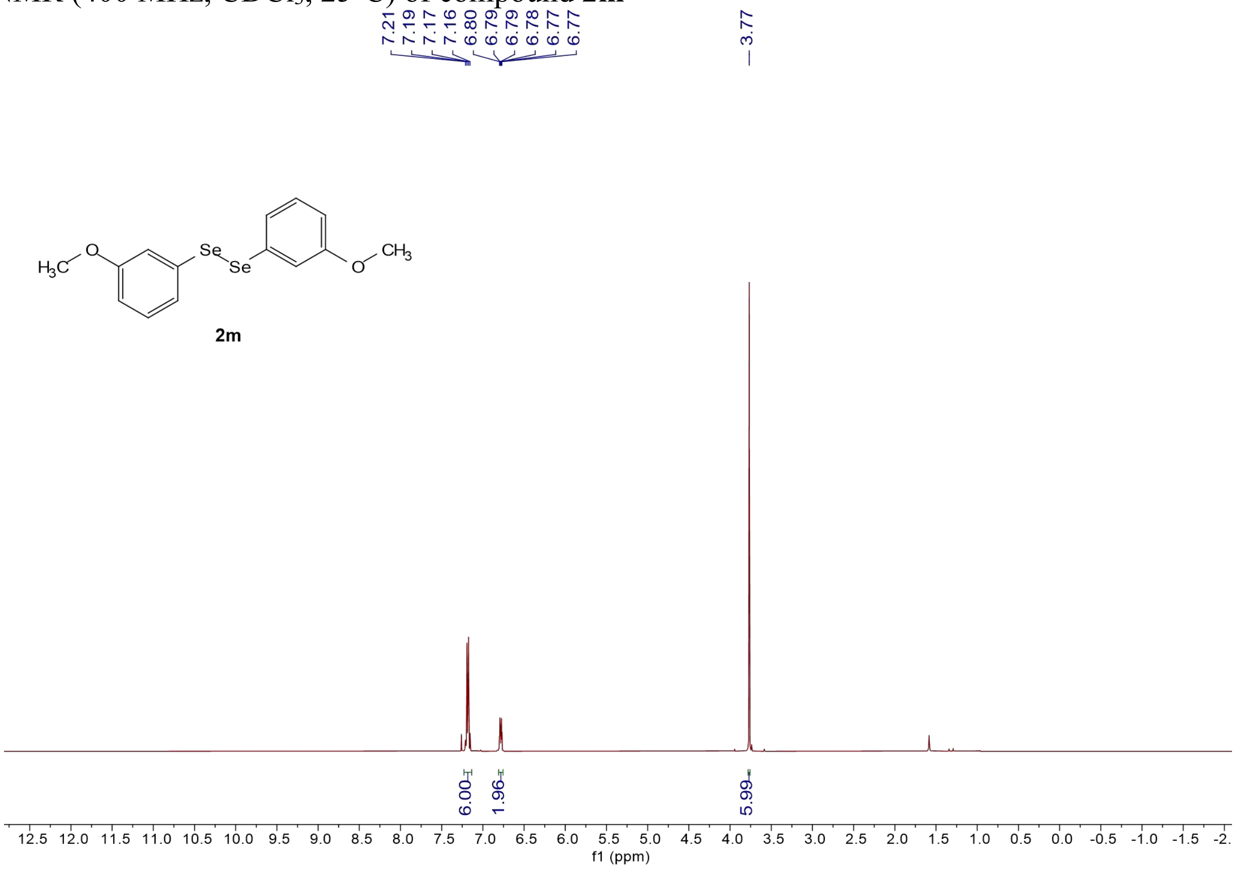

^{13}C NMR (101 MHz, $CDCl_3$, 25°C) of compound **2m**

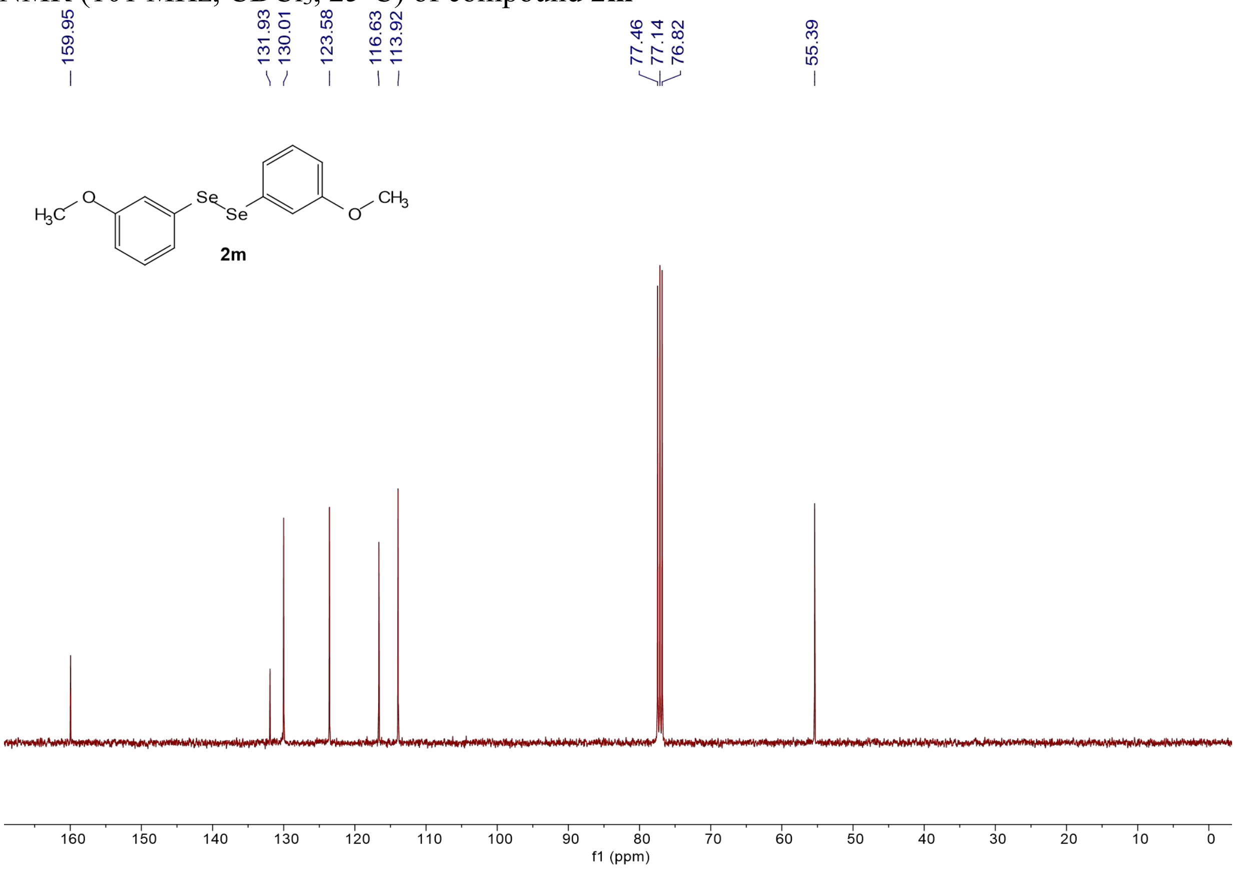

^{77}Se NMR (76 MHz, $CDCl_3$, 25°C) of compound **2m**

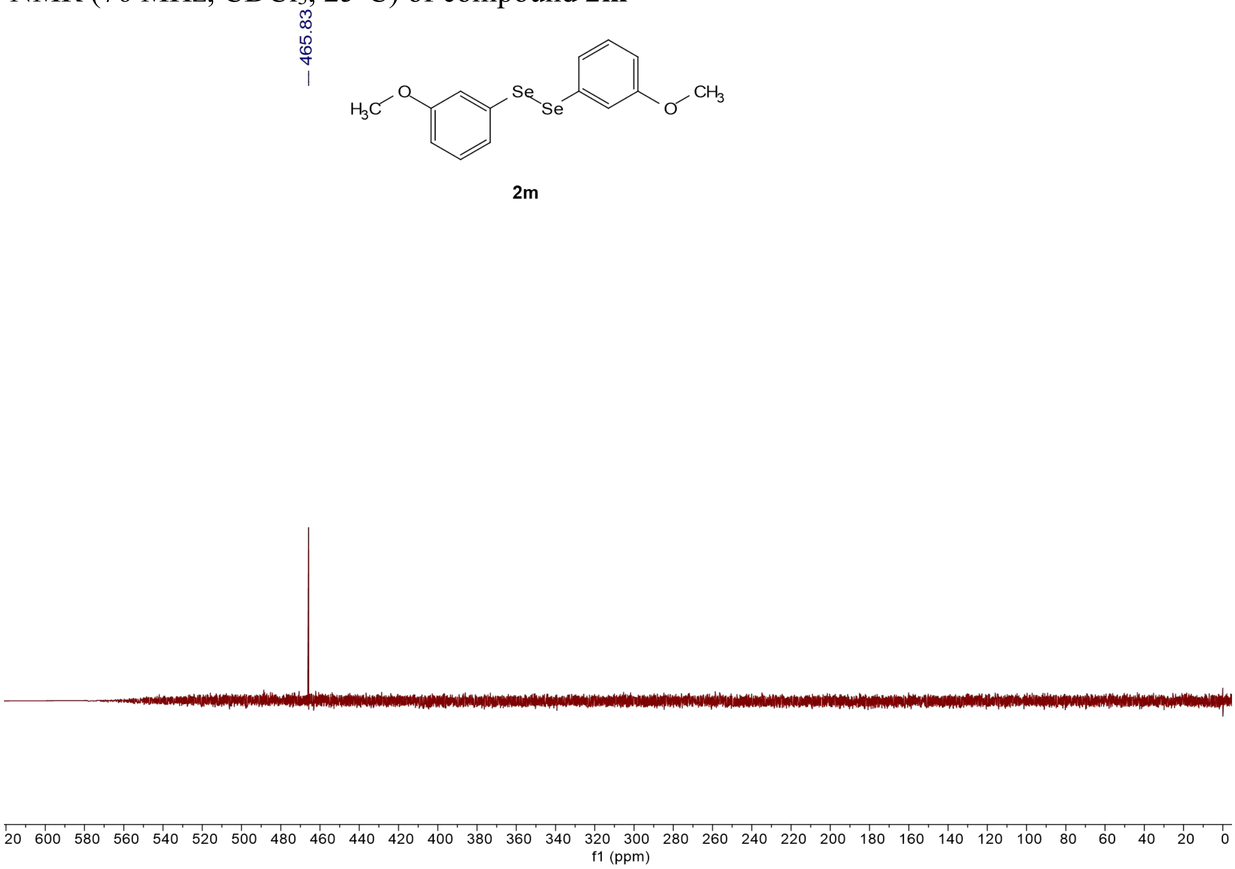

Supplementary Fig. 18. NMR spectra of compound **2m**

^{1}H NMR (400 MHz, $CDCl_3$, 25°C) of compound **2n**

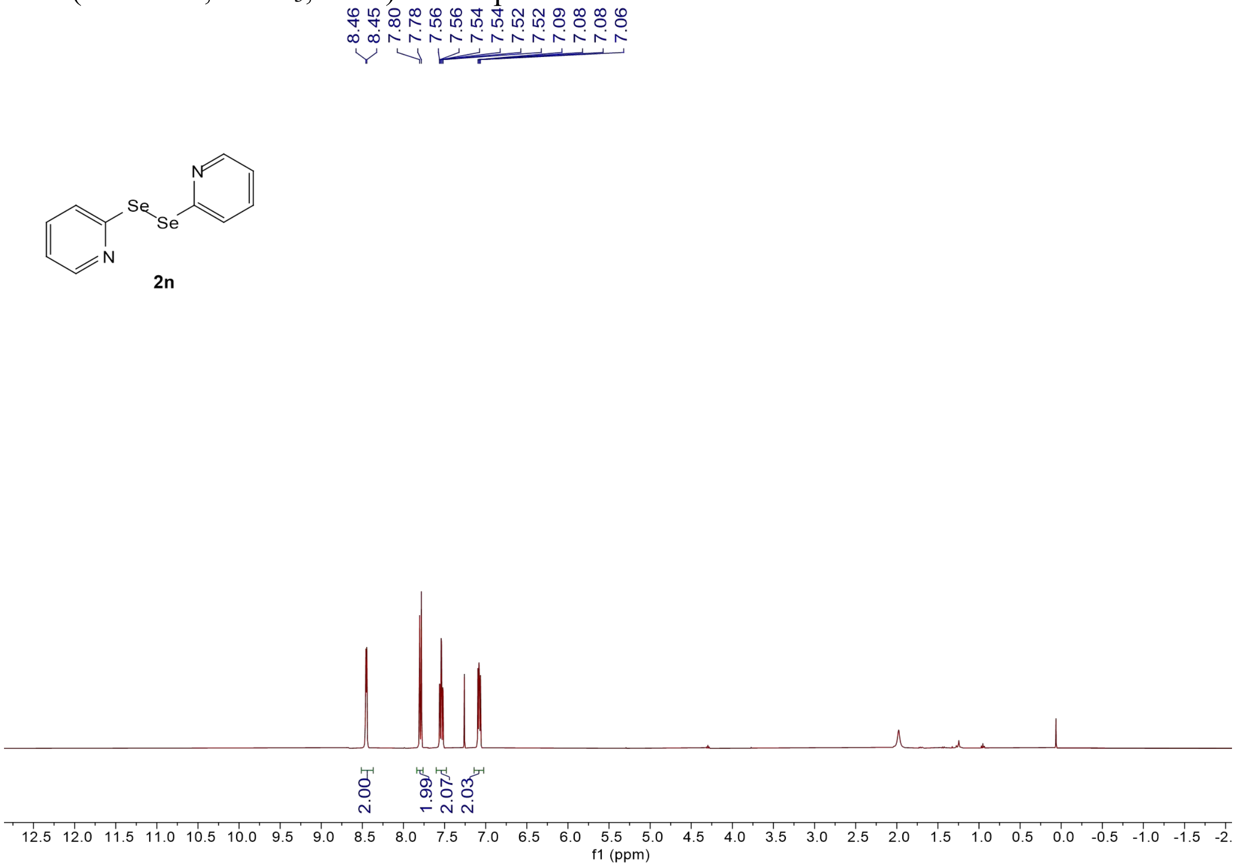

^{13}C NMR (101 MHz, $CDCl_3$, 25°C) of compound **2n**

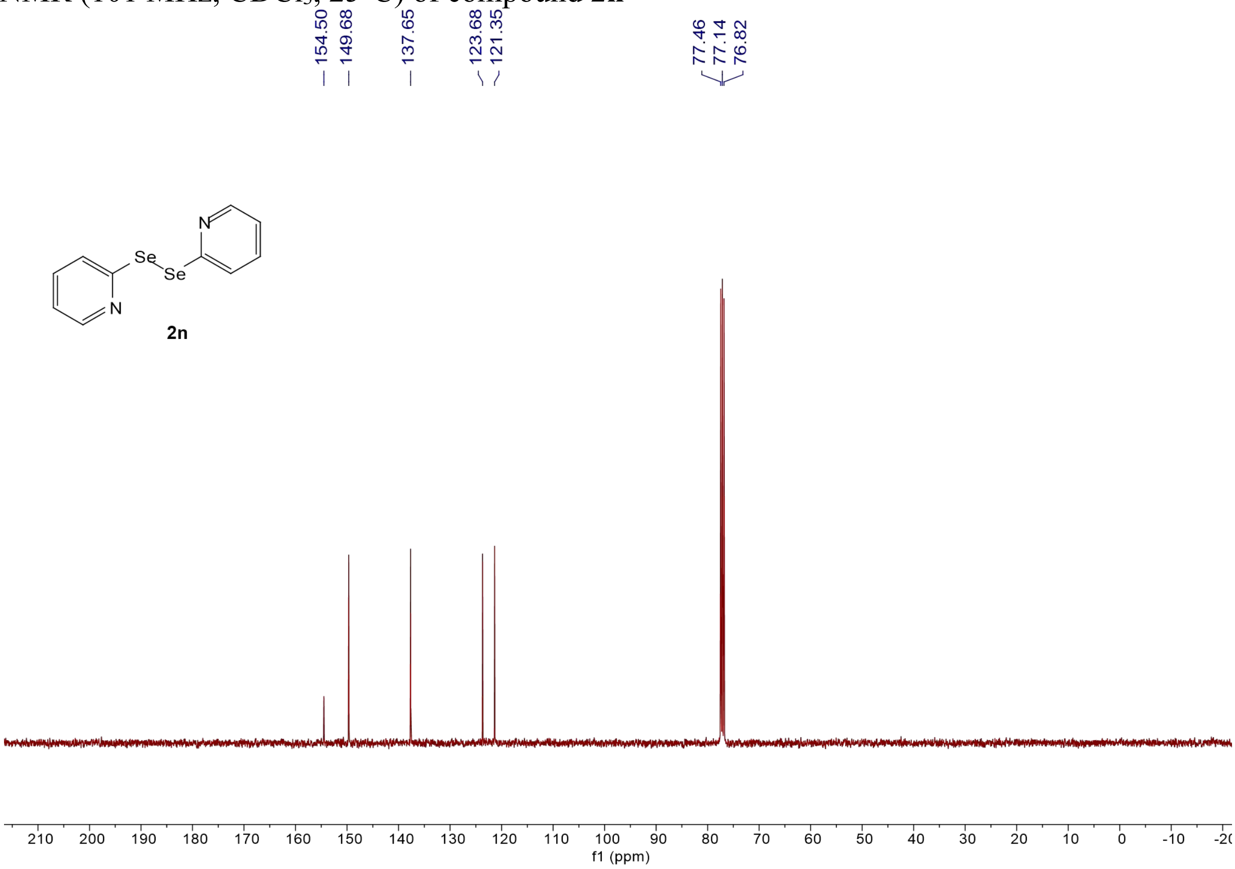

^{77}Se NMR (76 MHz, $CDCl_3$, 25°C) of compound **2n**

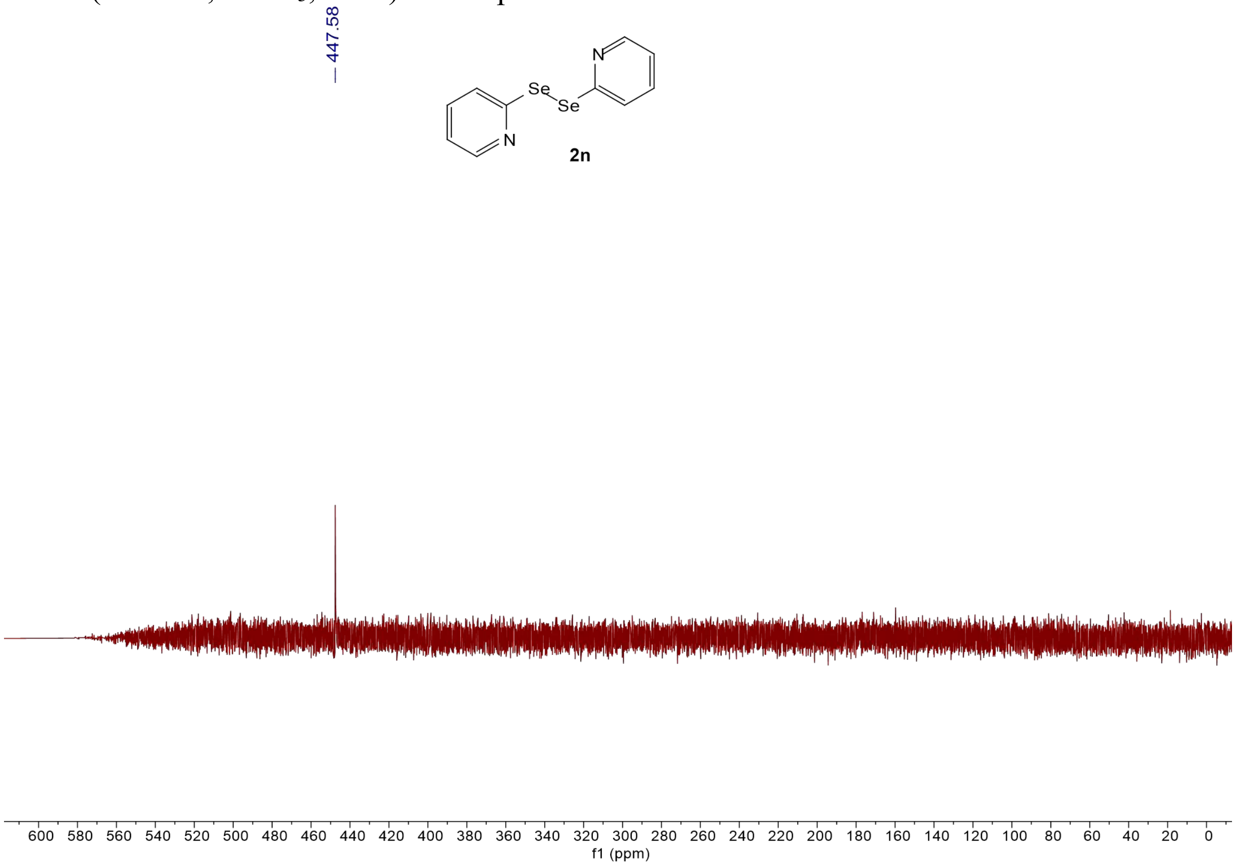

Supplementary Fig. 19. NMR spectra of compound **2n**

^{1}H NMR (400 MHz, $CDCl_3$, 25°C) of compound **2o**

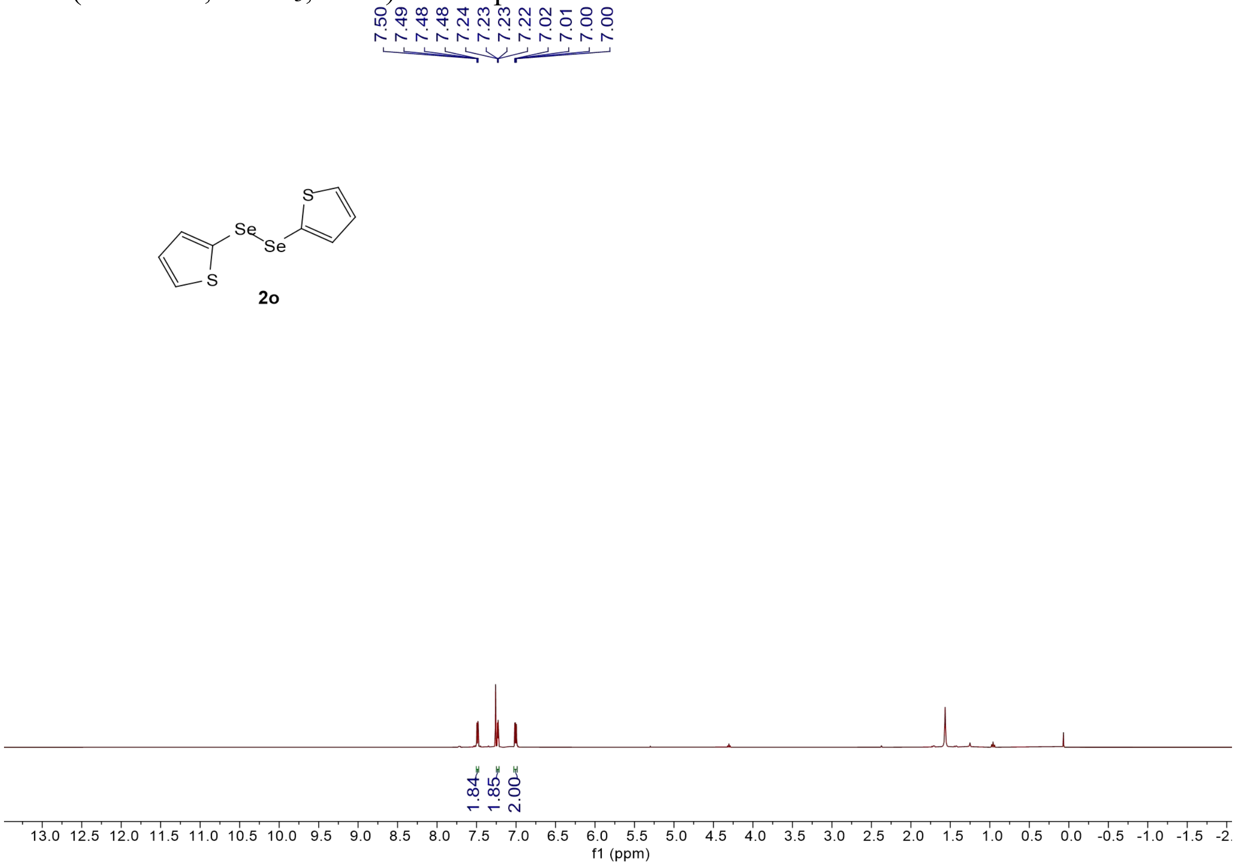

^{13}C NMR (101 MHz, $CDCl_3$, 25°C) of compound **2o**

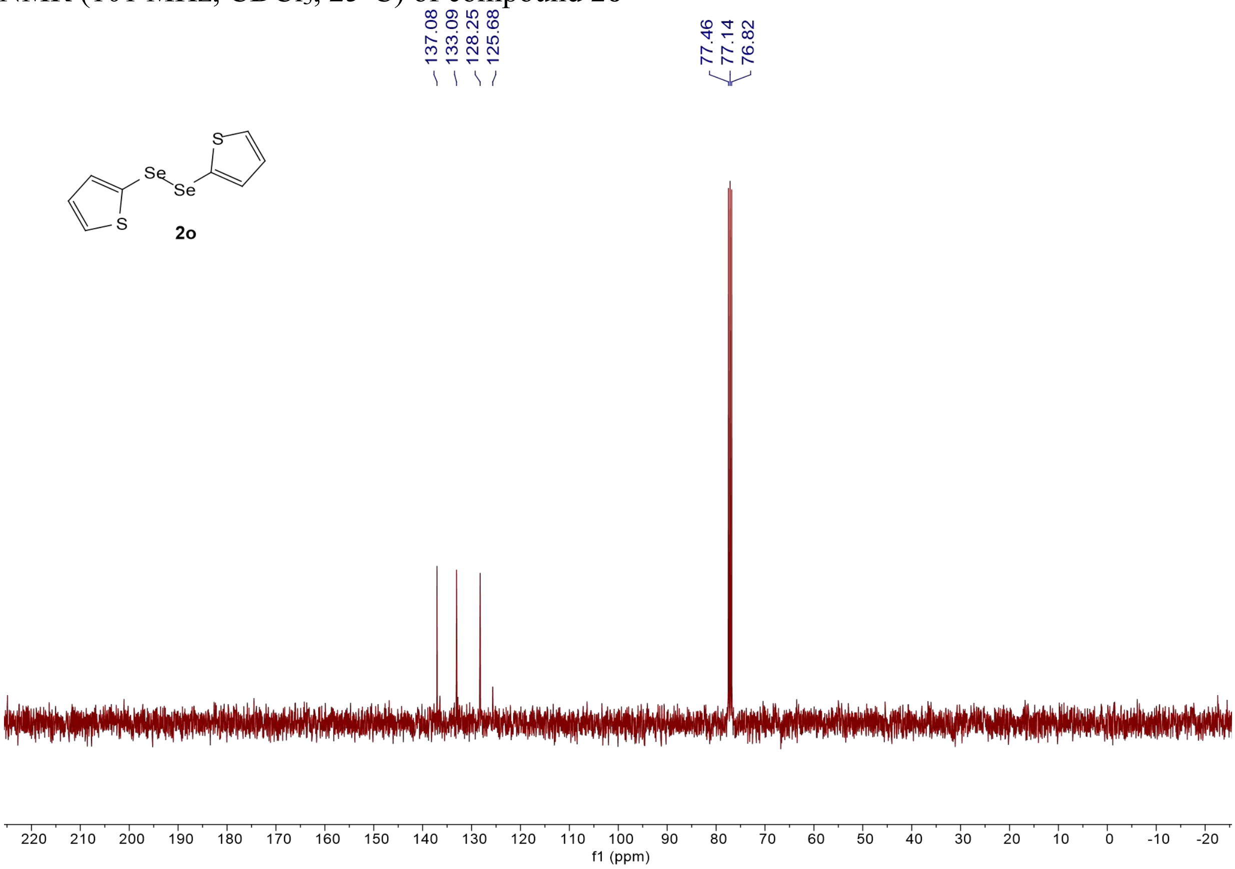

^{77}Se NMR (76 MHz, $CDCl_3$, 25°C) of compound **2o**

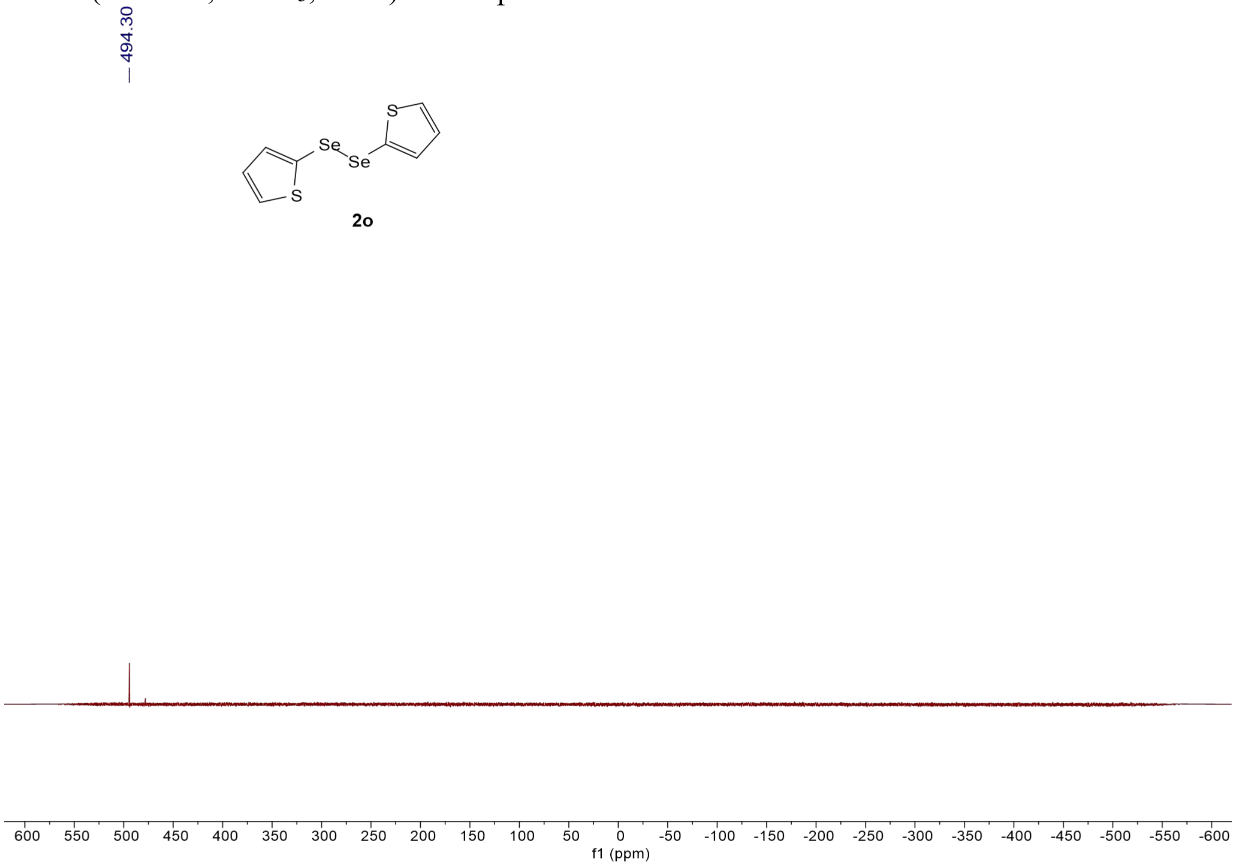

Supplementary Fig. 20. NMR spectra of compound **2o**

^{1}H NMR (400 MHz, $CDCl_3$, 25°C) of compound **2p**

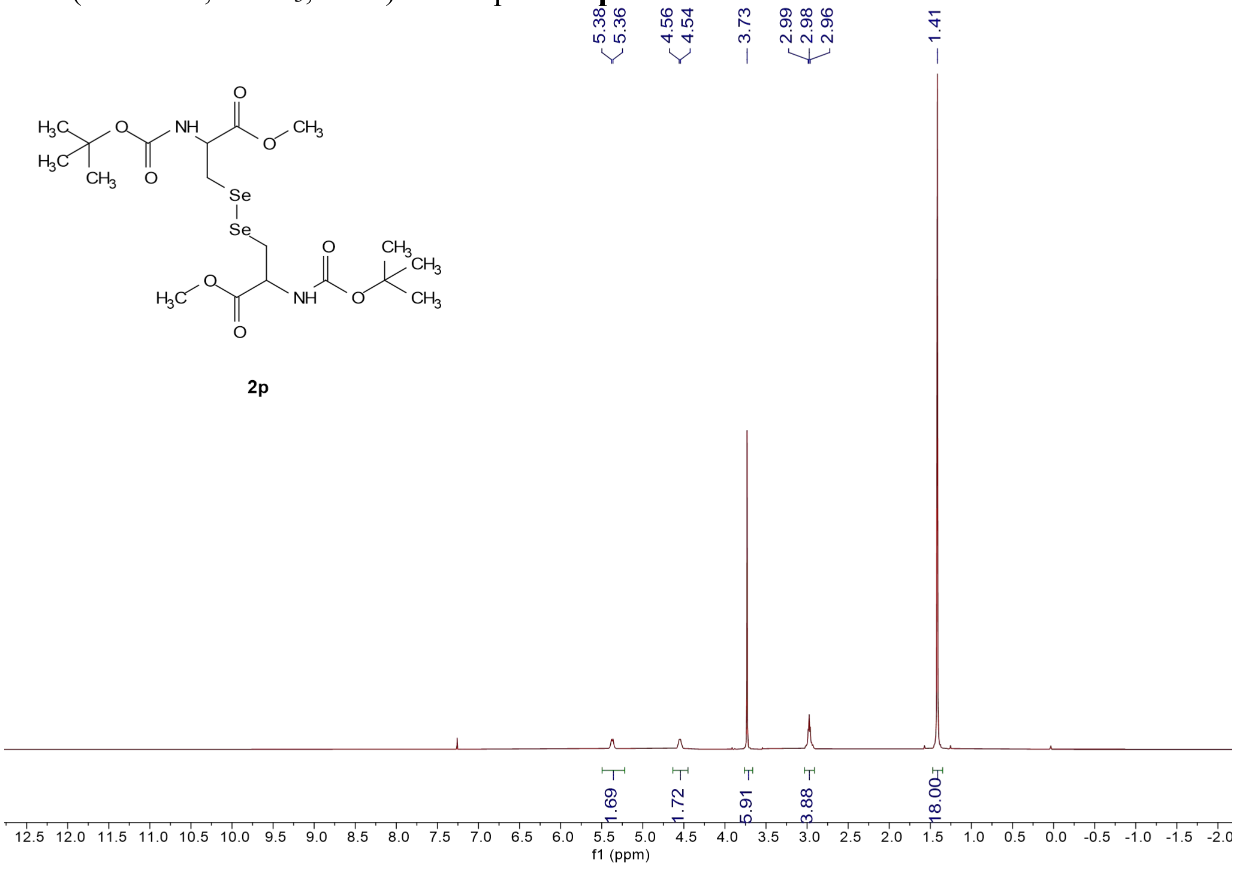

^{13}C NMR (101 MHz, $CDCl_3$, 25°C) of compound **2p**

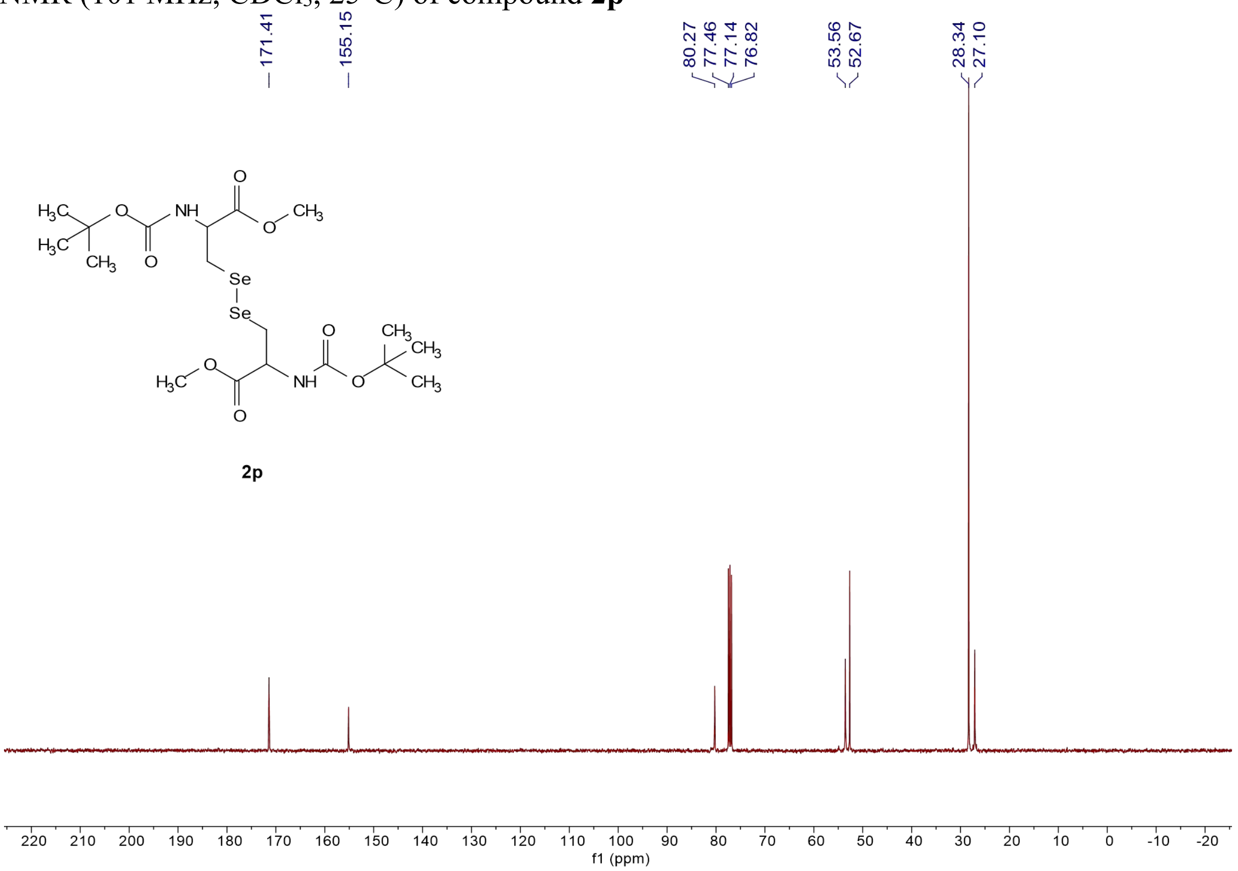

^{77}Se NMR (76 MHz, $CDCl_3$, 25°C) of compound **2p**

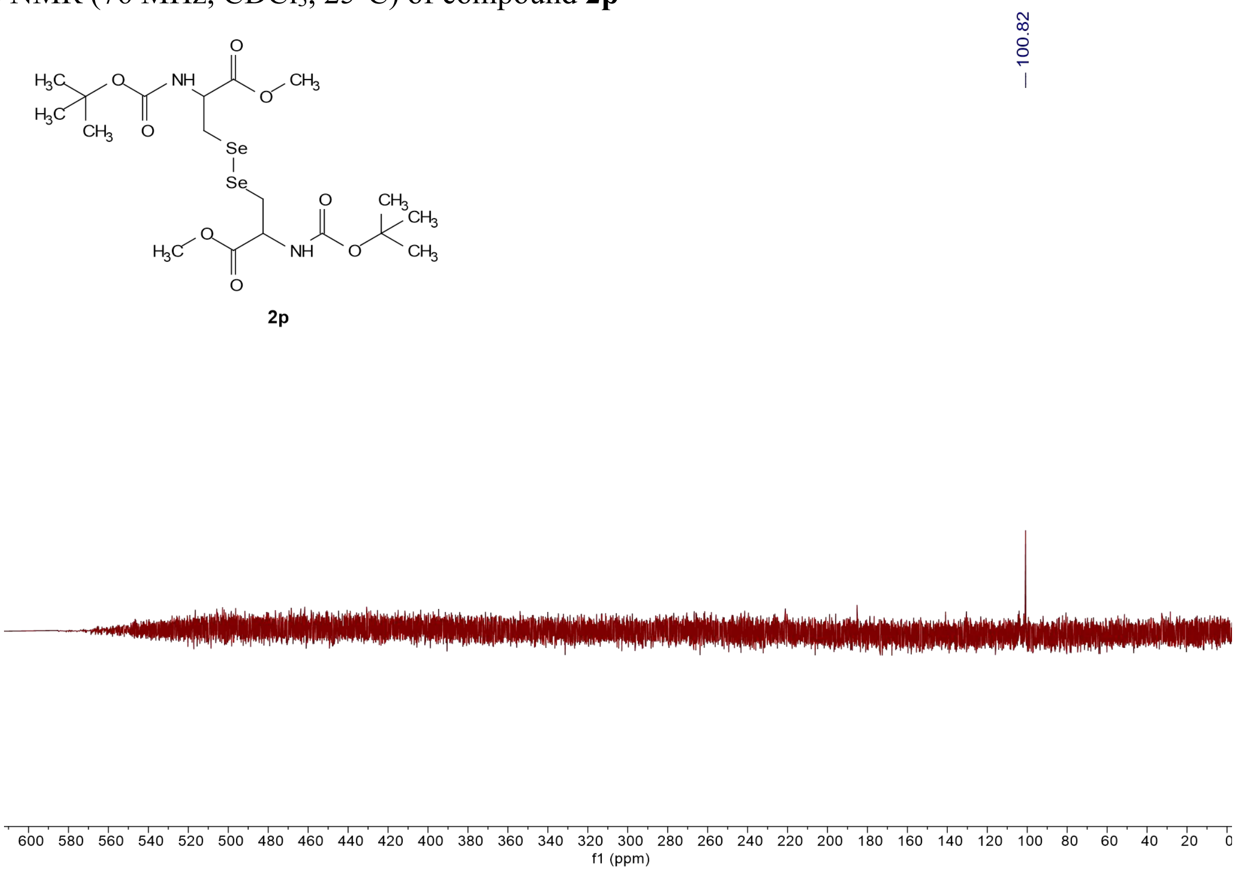

Supplementary Fig. 21. NMR spectra of compound **2p**

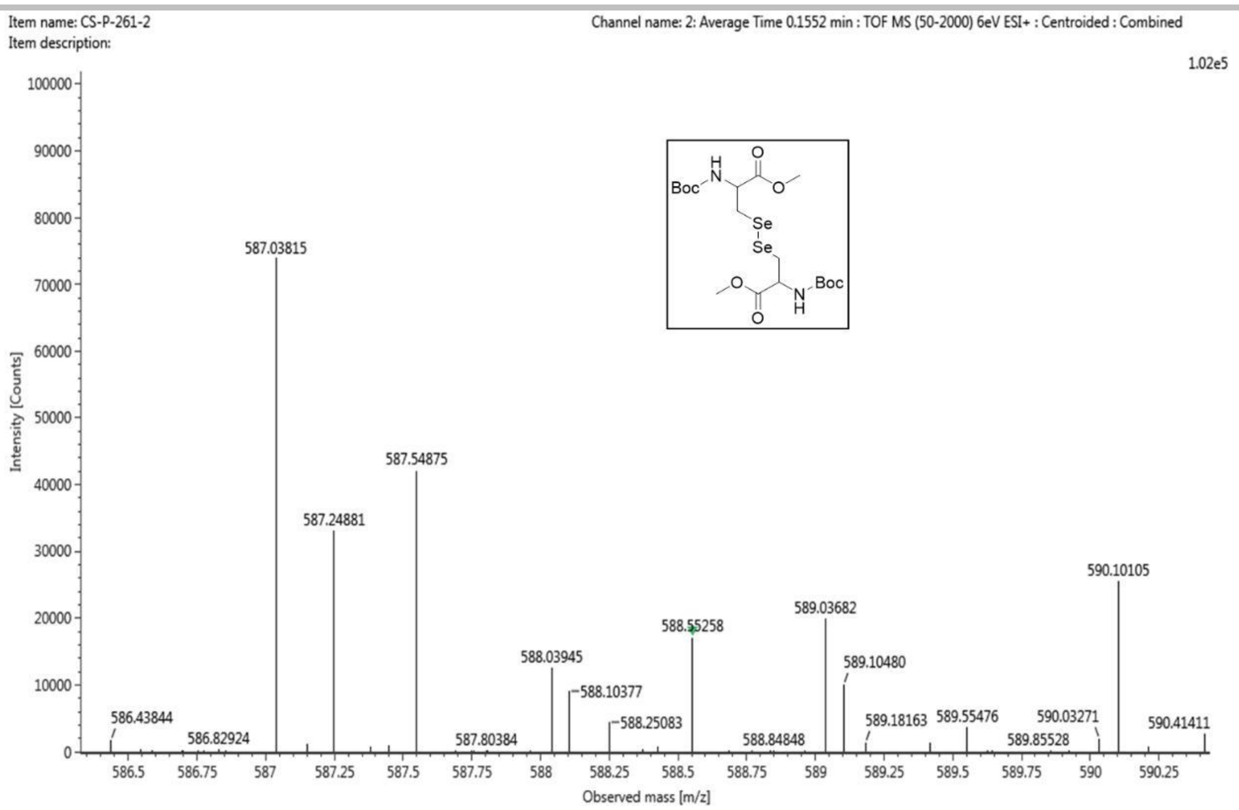

Supplementary Fig. 22. HR-MS of compound **2p**

^{1}H NMR (400 MHz, $CDCl_3$, 25°C) of compound **2q**

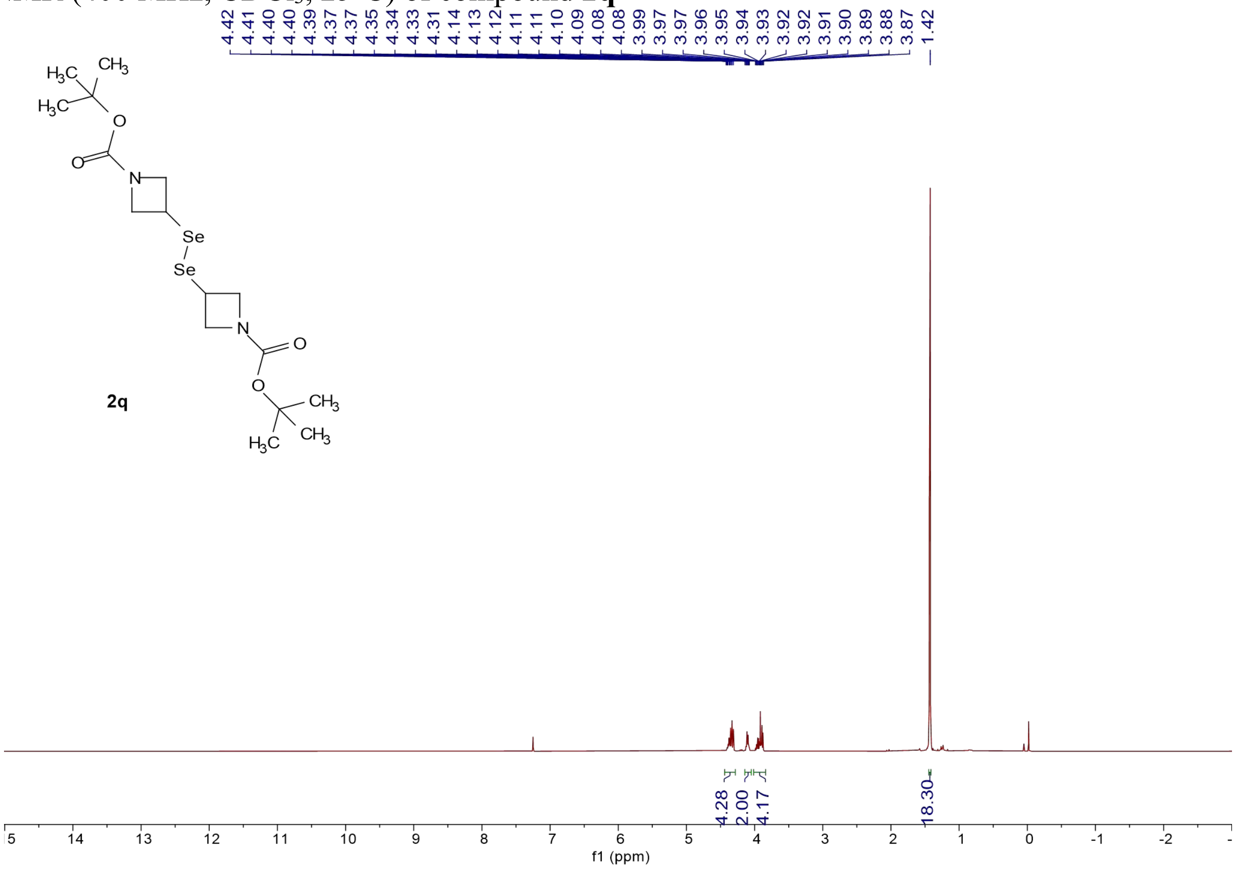

^{13}C NMR (101 MHz, $CDCl_3$, 25°C) of compound **2q**

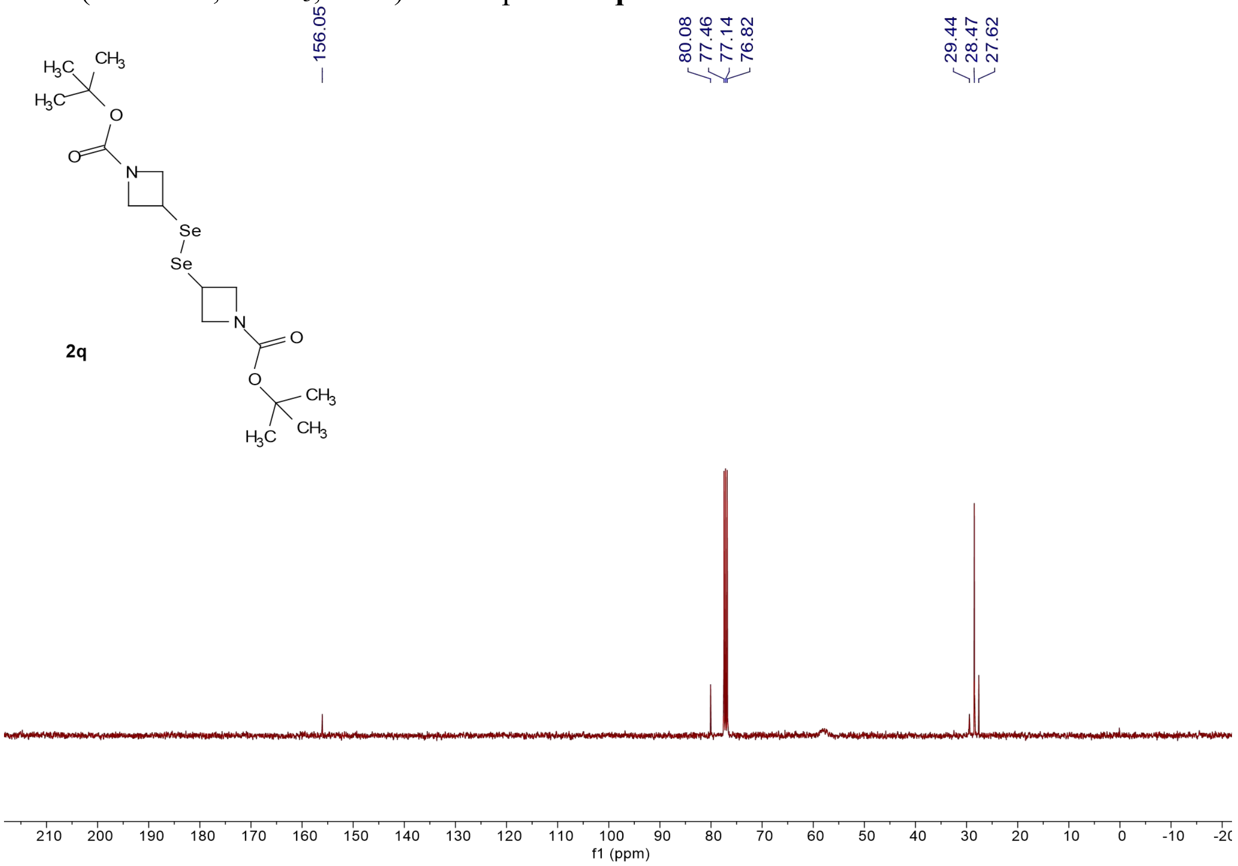

^{77}Se NMR (76 MHz, $CDCl_3$, 25°C) of compound **2q**

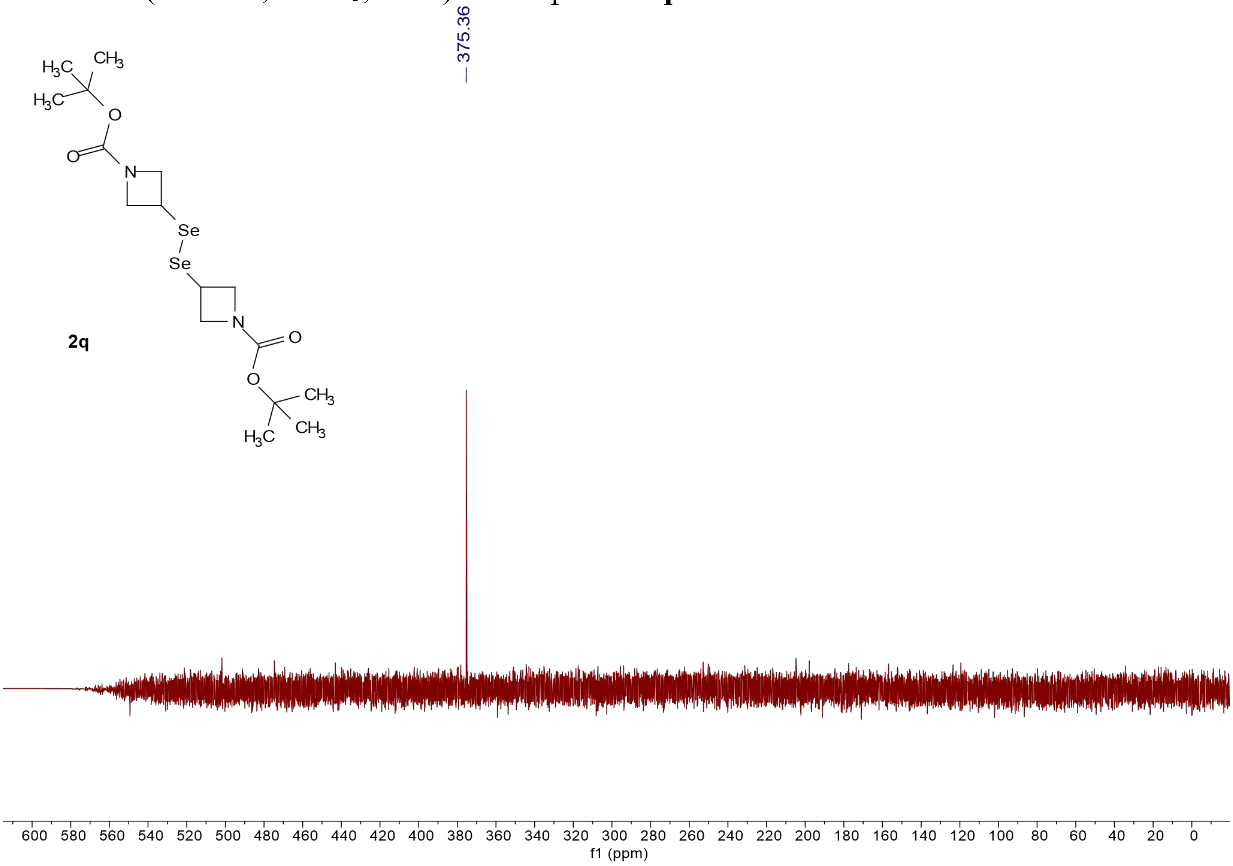

Supplementary Fig. 23. NMR spectra of compound **2q**

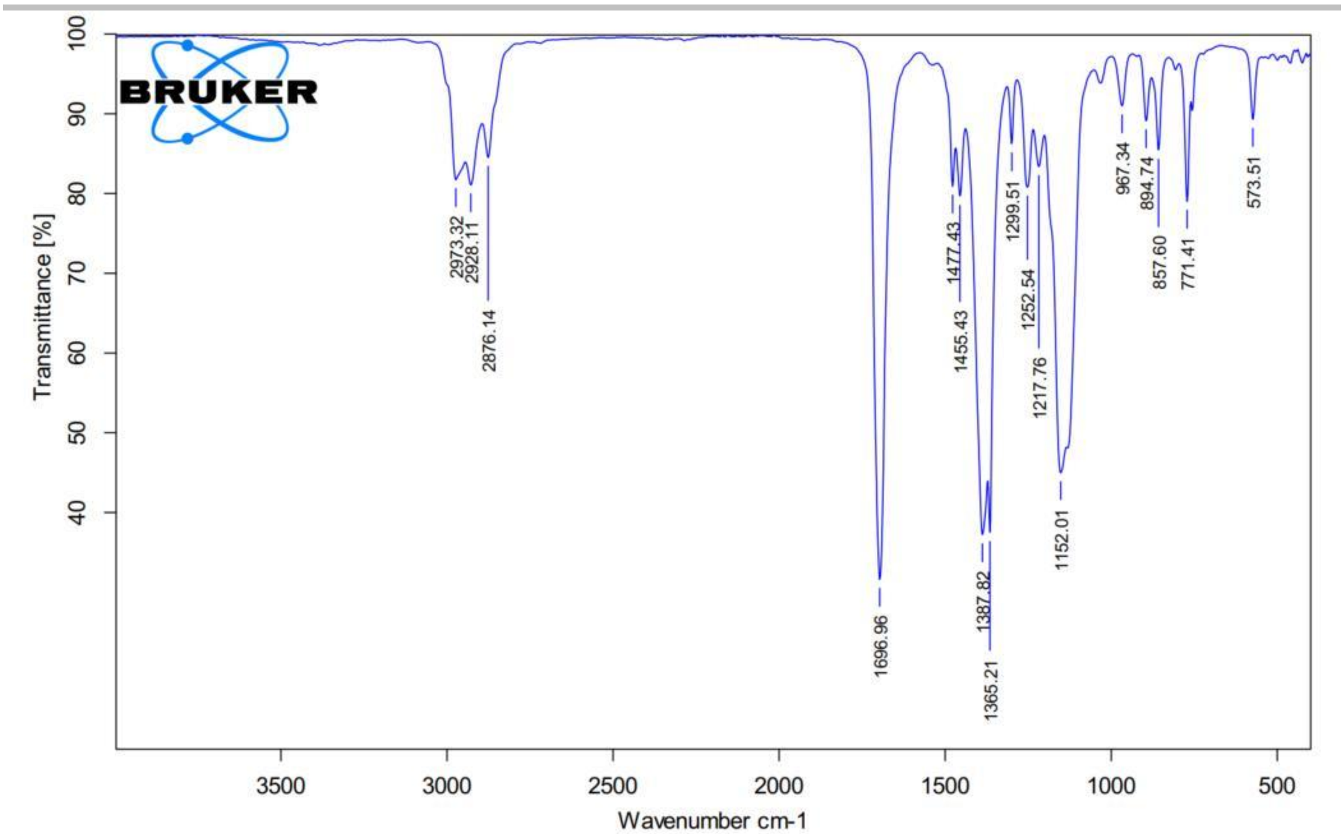

Supplementary Fig. 24. IR of compound **2q**

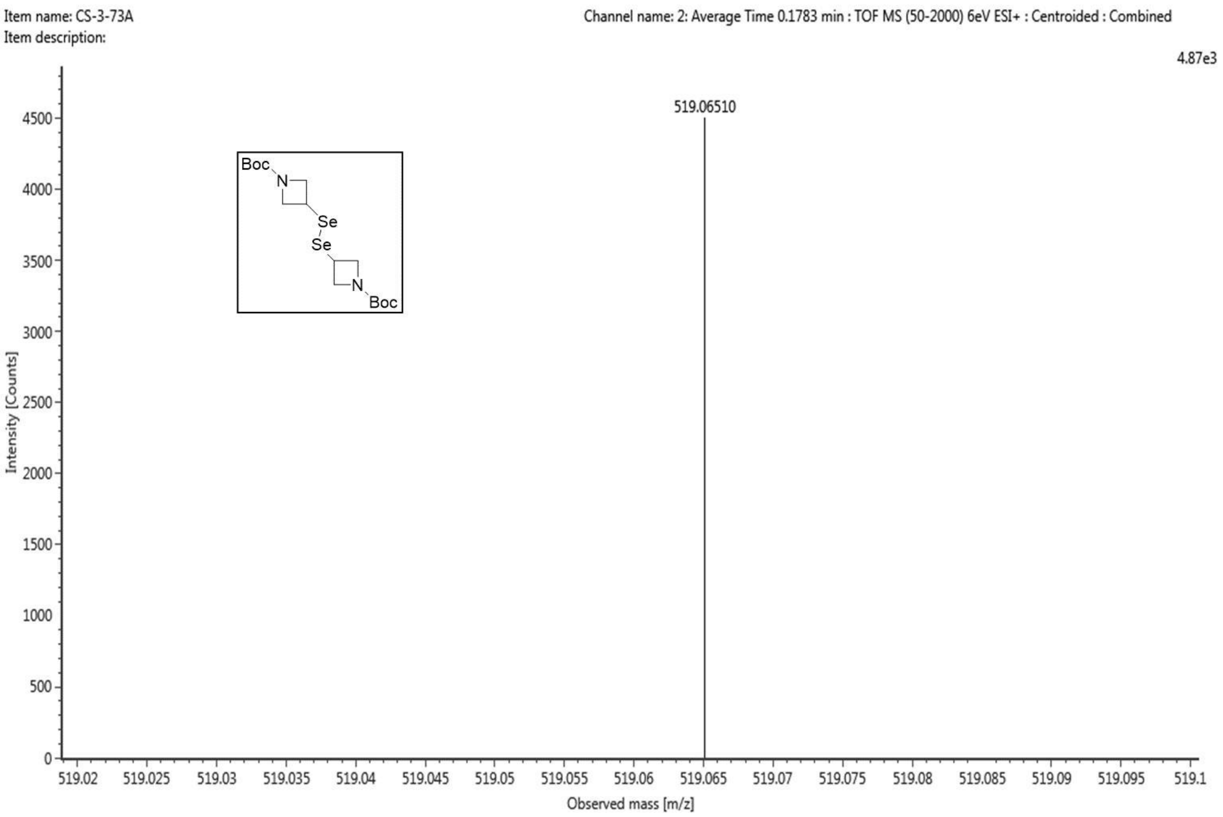

Supplementary Fig. 25. HR-MS of compound **2q**

^{1}H NMR (400 MHz, $CDCl_3$, 25°C) of compound **2ra**

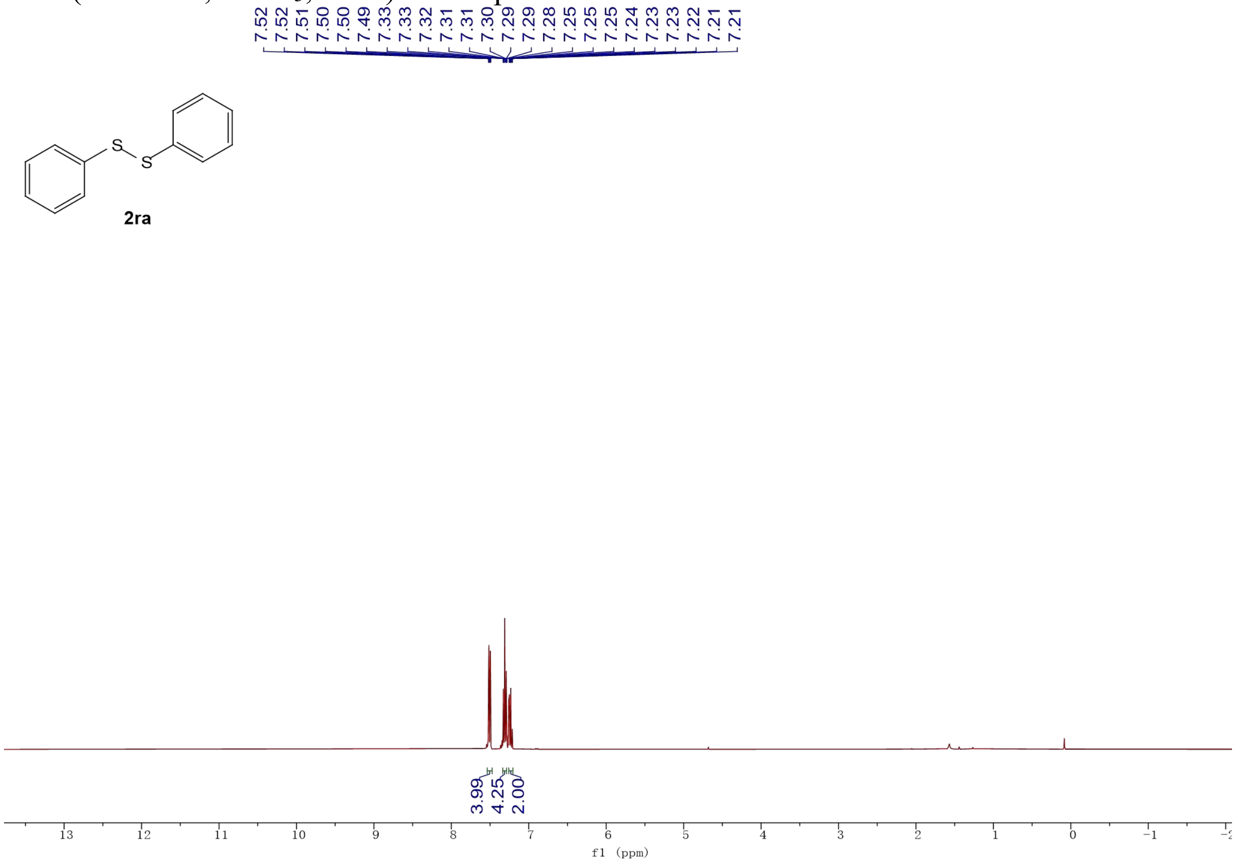

^{13}C NMR (101 MHz, $CDCl_3$, 25°C) of compound **2ra**

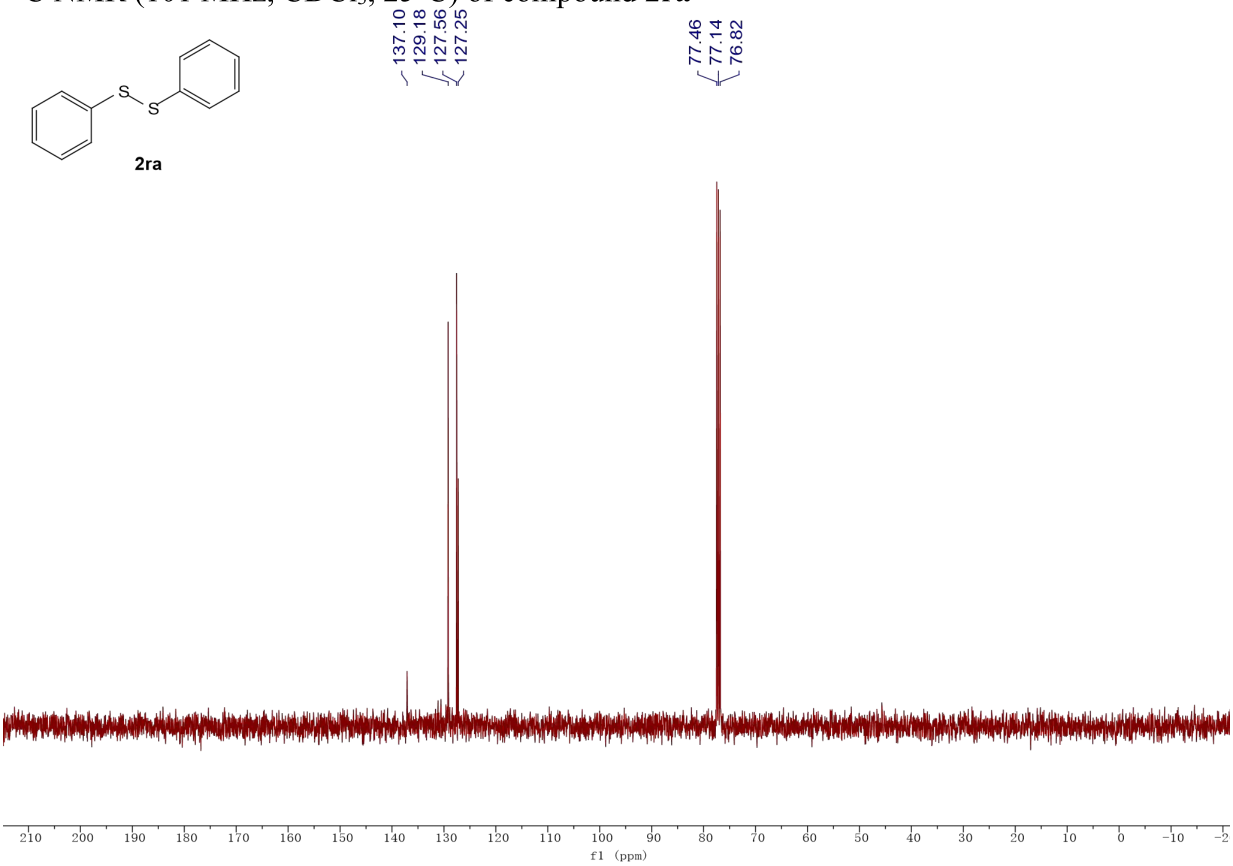

Supplementary Fig. 26. NMR spectra of compound **2ra**

^1H NMR (400 MHz, $CDCl_3$, 25°C) of compound **2rb**

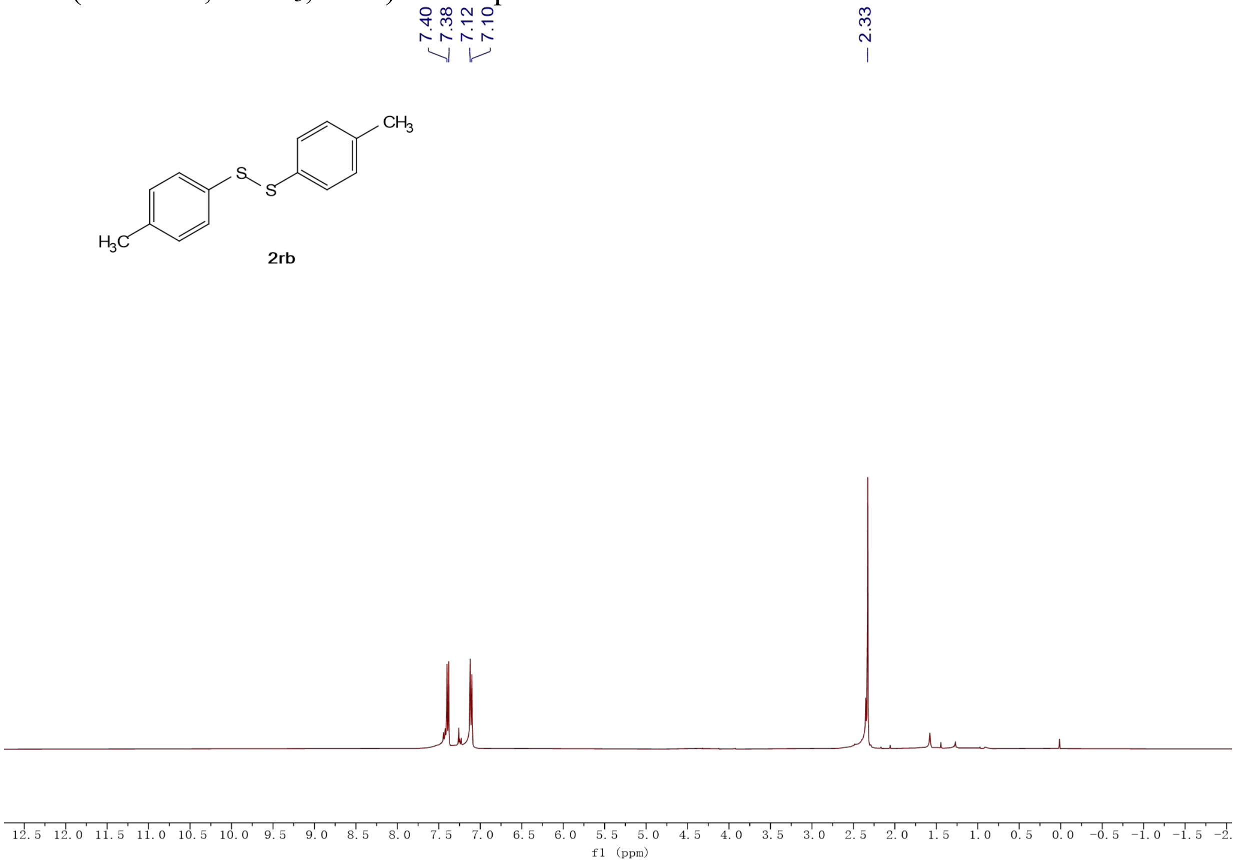

^{13}C NMR (101 MHz, $CDCl_3$, 25°C) of compound **2rb**

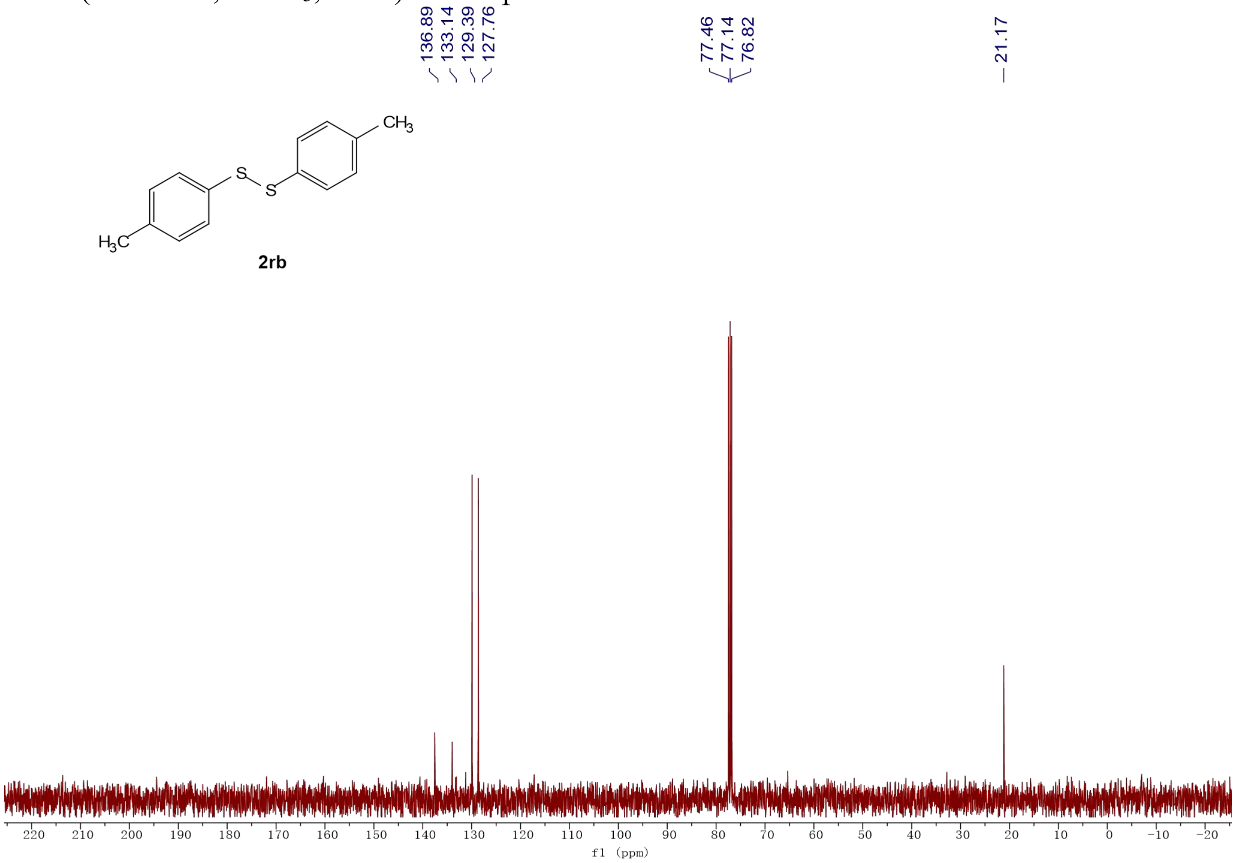

Supplementary Fig. 27. NMR spectra of compound **2rb**

^{1}H NMR (400 MHz, $CDCl_3$, 25℃) of compound **2rc**

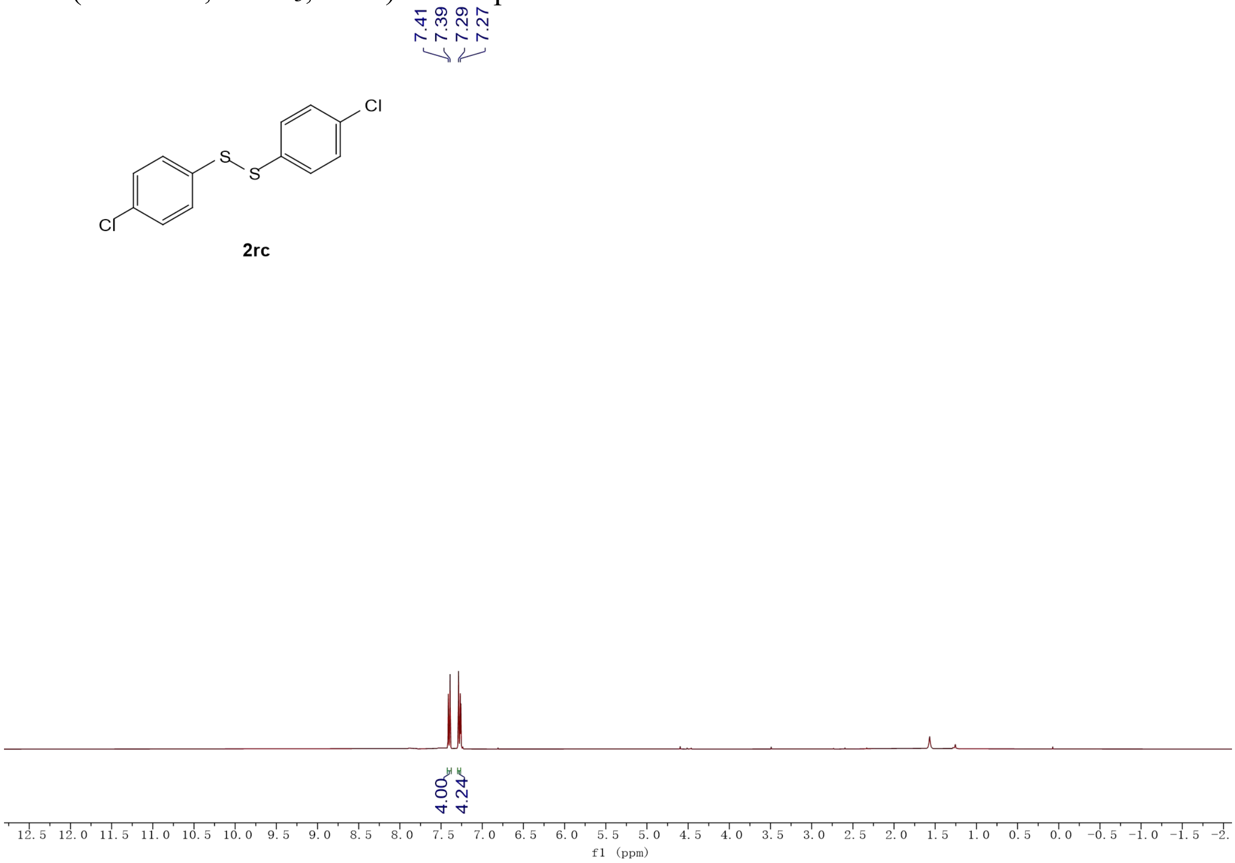

^{13}C NMR (101 MHz, $CDCl_3$, 25℃) of compound **2rc**

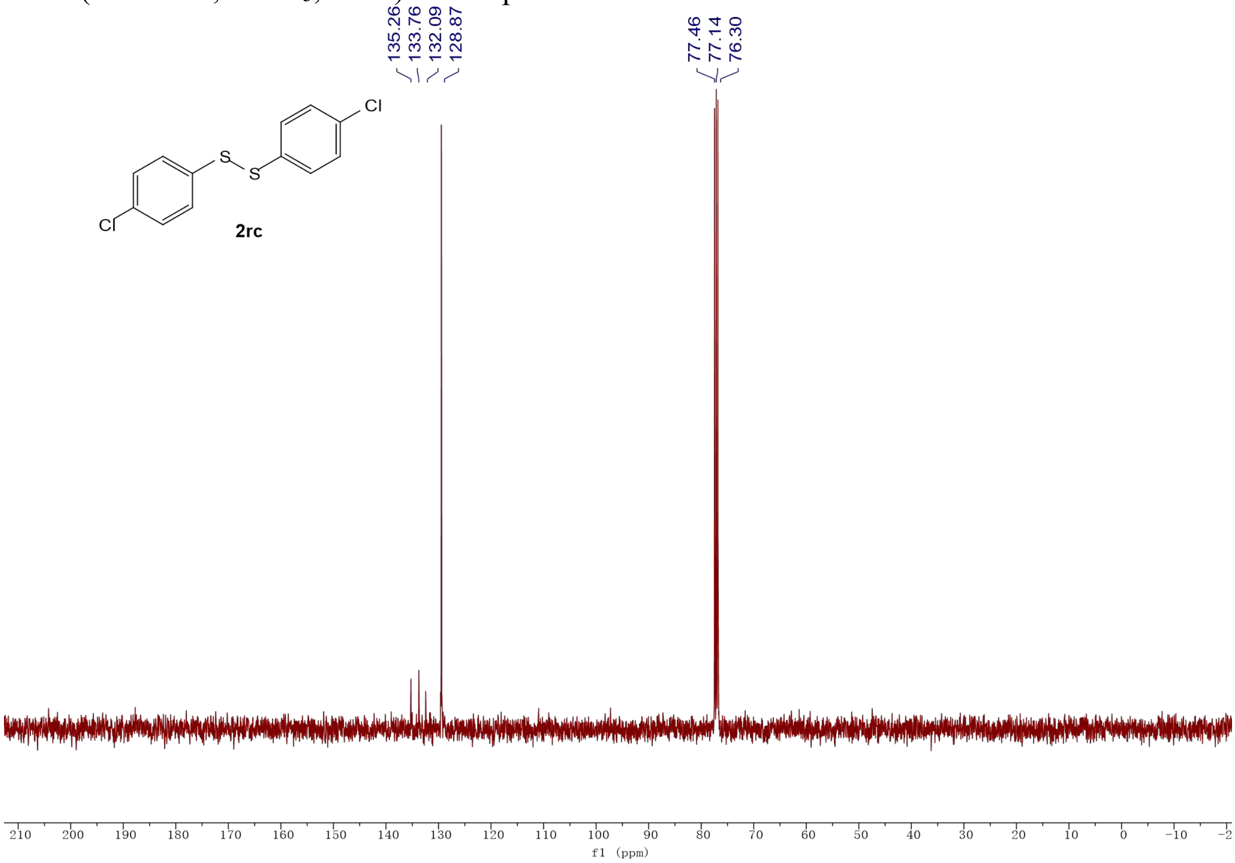

Supplementary Fig. 28. NMR spectra of compound **2rc**

^{1}H NMR (400 MHz, $CDCl_3$, 25°C) of compound **2rd**

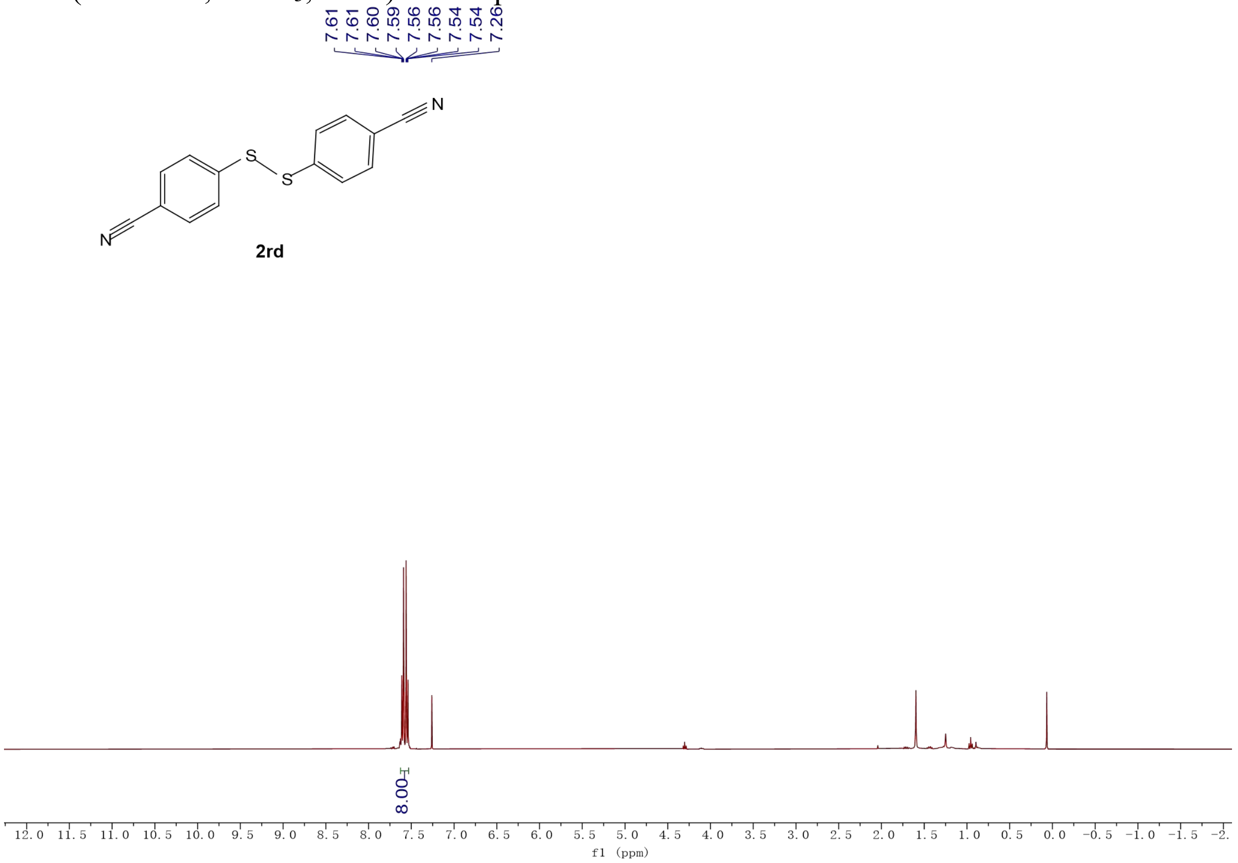

^{13}C NMR (101 MHz, $CDCl_3$, 25°C) of compound **2rd**

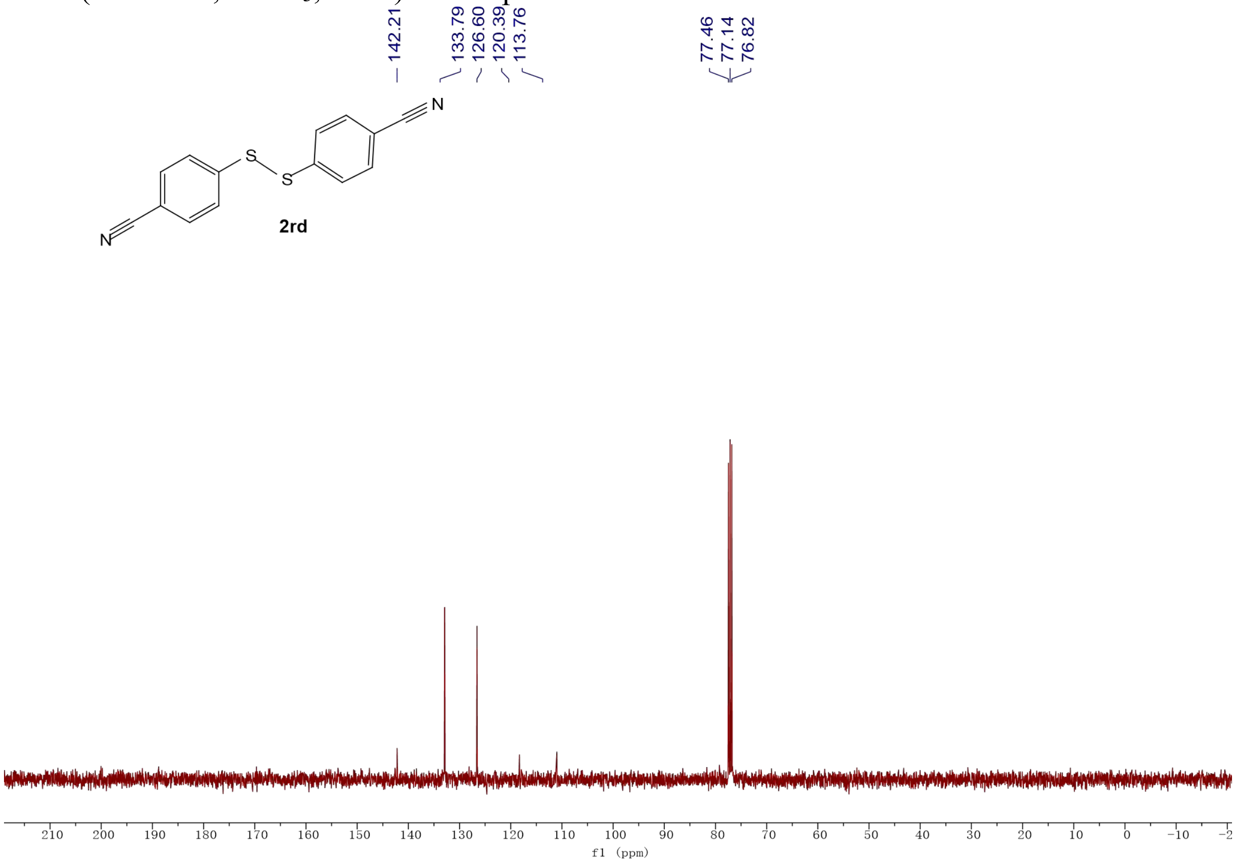

Supplementary Fig. 29. NMR spectra of compound **2rd**

^{1}H NMR (400 MHz, $CDCl_3$, 25°C) of compound **2re**

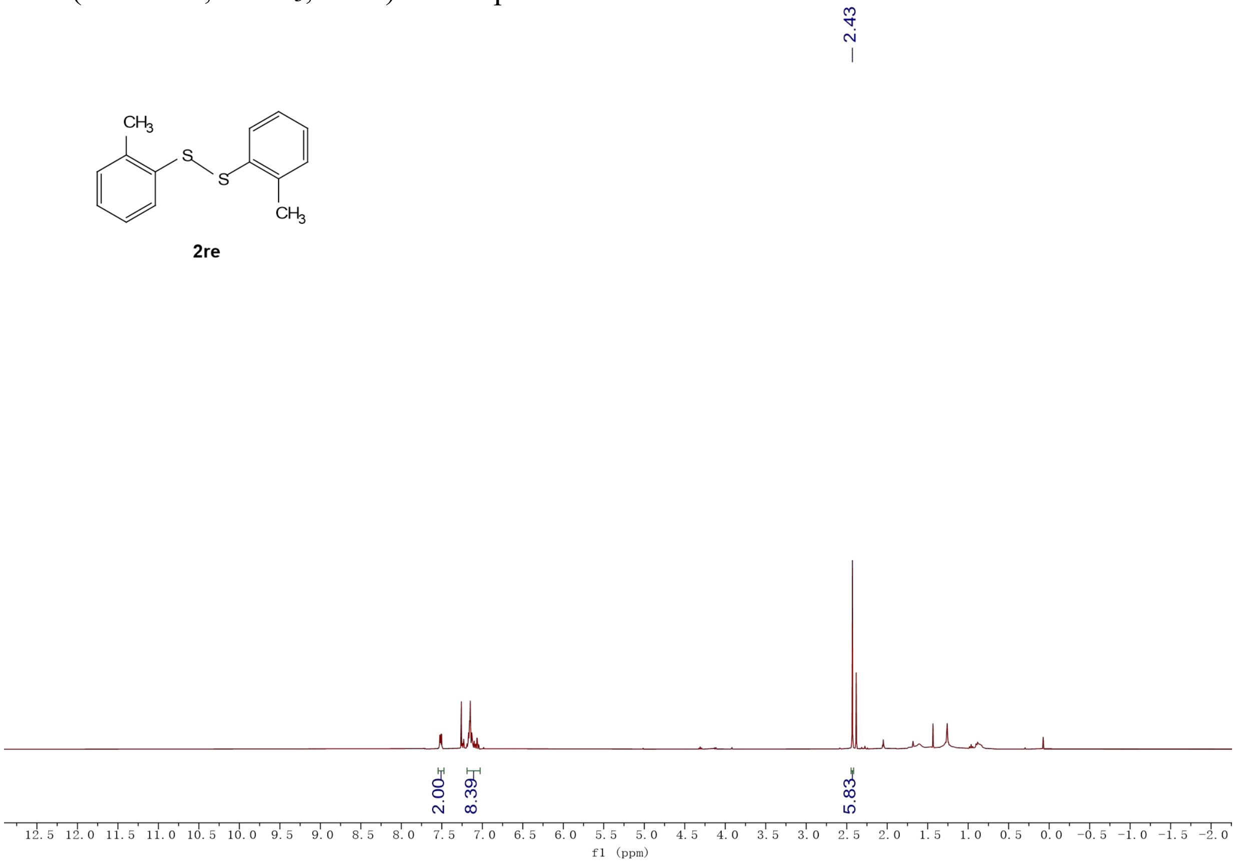

^{13}C NMR (101 MHz, $CDCl_3$, 25°C) of compound **2re**

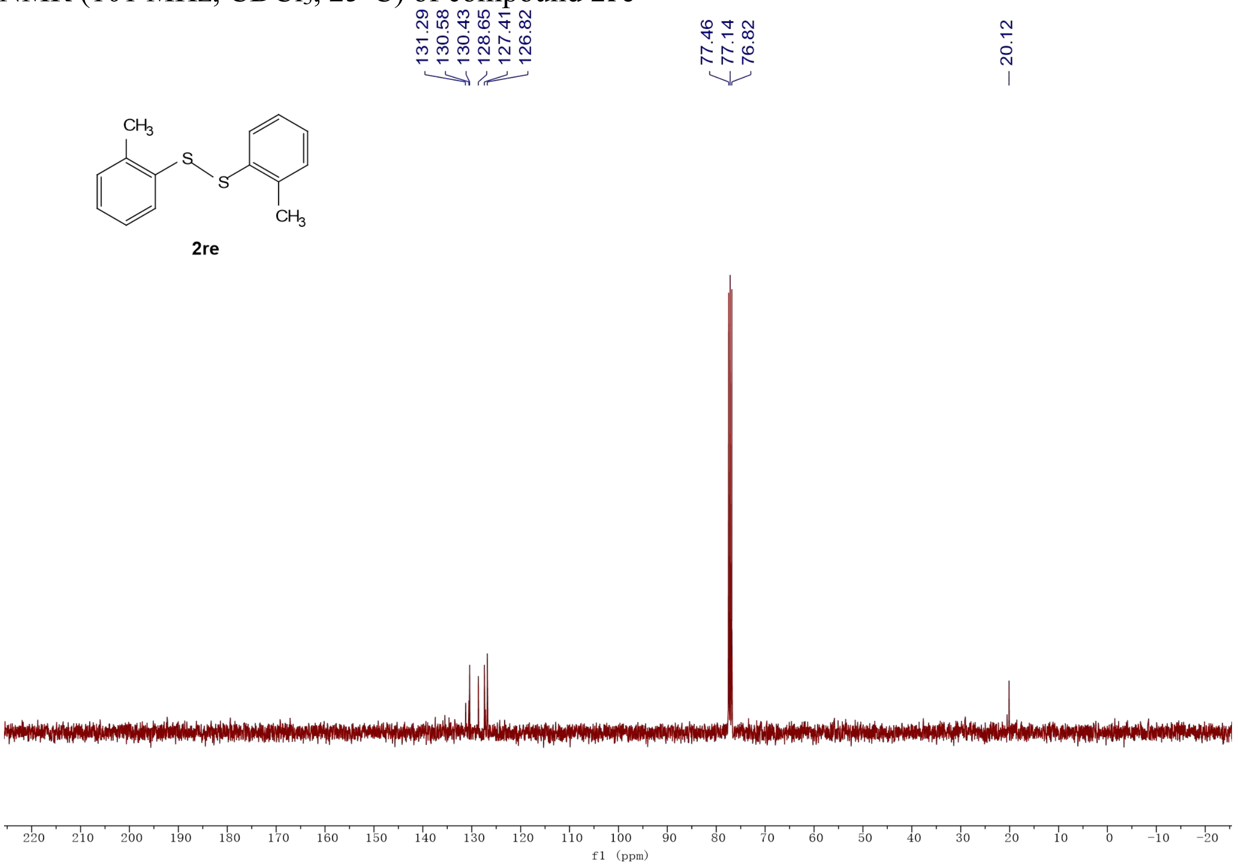

Supplementary Fig. 30. NMR spectra of compound **2re**

^{1}H NMR (400 MHz, $CDCl_3$, 25°C) of compound **2rf**

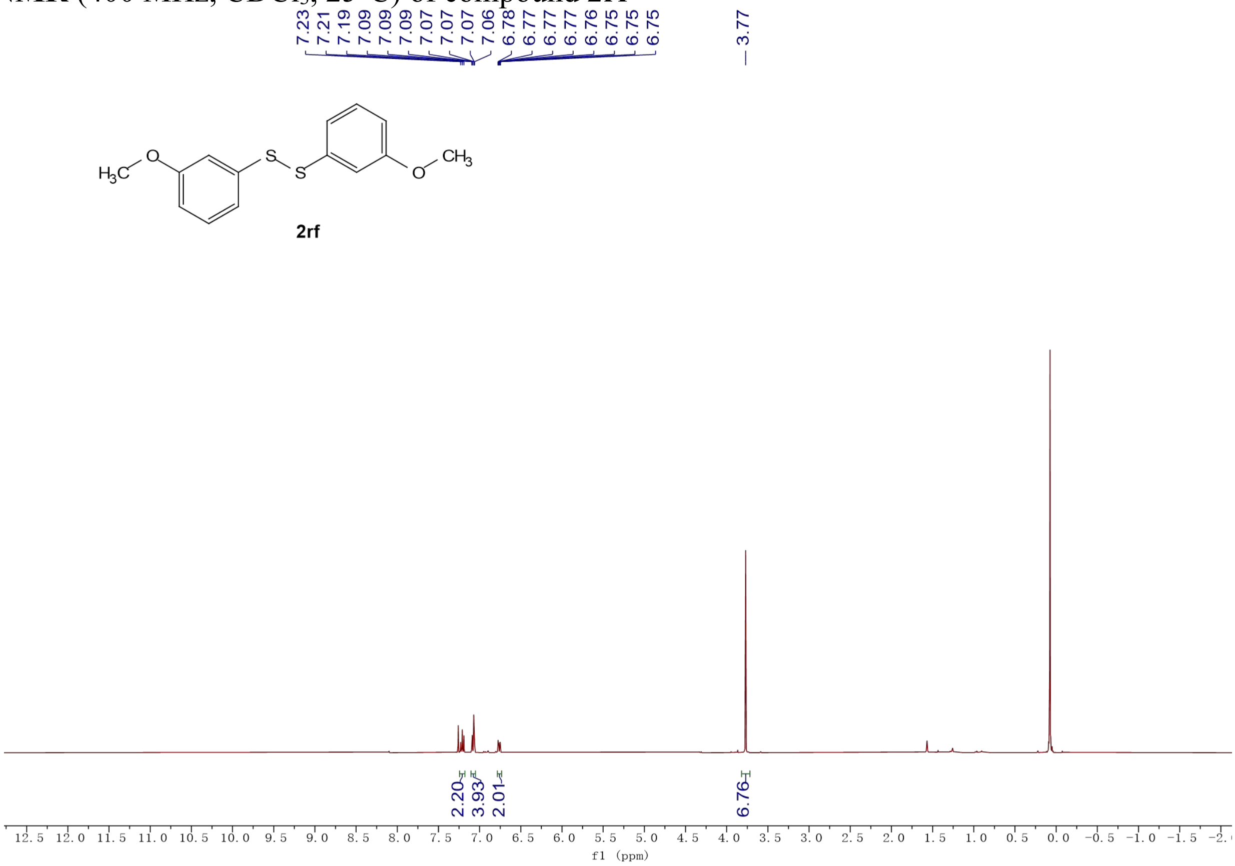

^{13}C NMR (101 MHz, $CDCl_3$, 25°C) of compound **2rf**

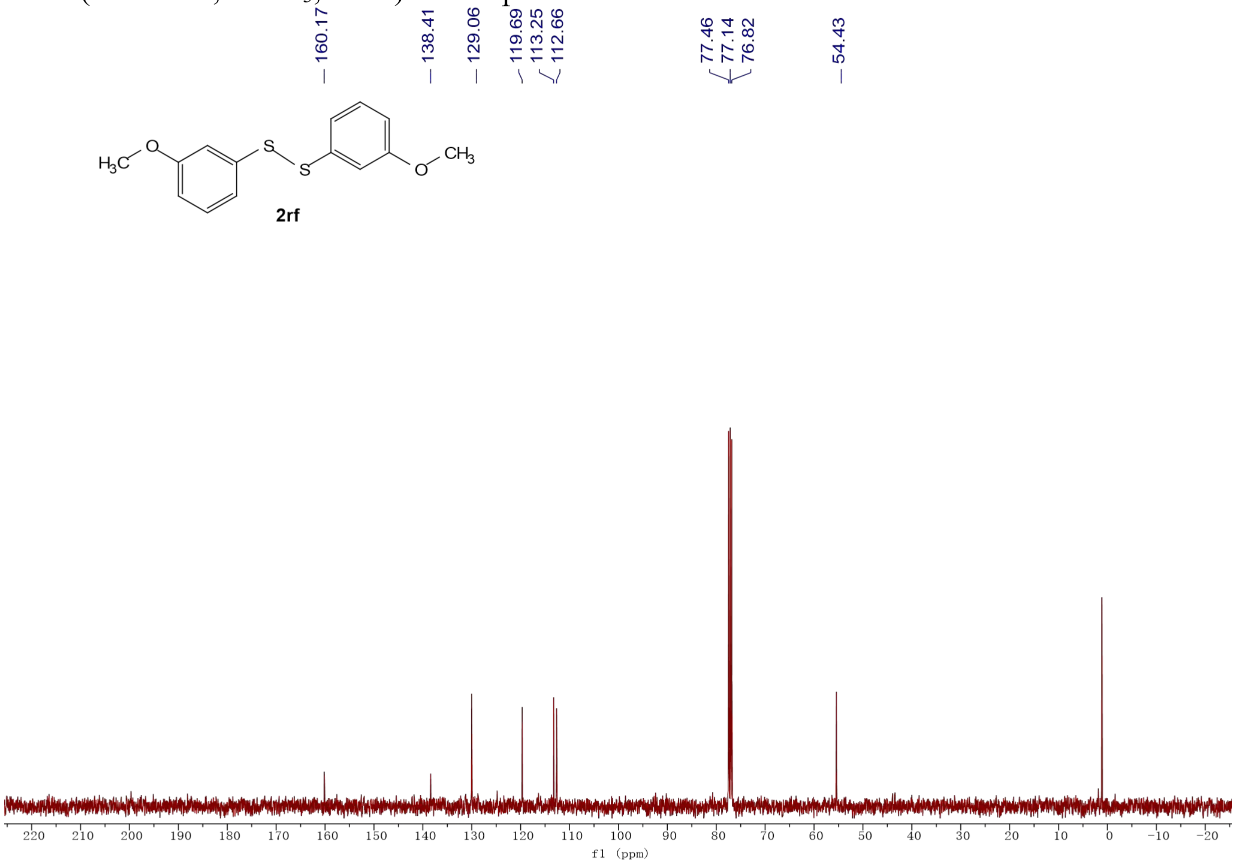

Supplementary Fig. 31. NMR spectra of compound **2rf**

^{1}H NMR (400 MHz, $CDCl_3$, 25°C) of compound **2rg**

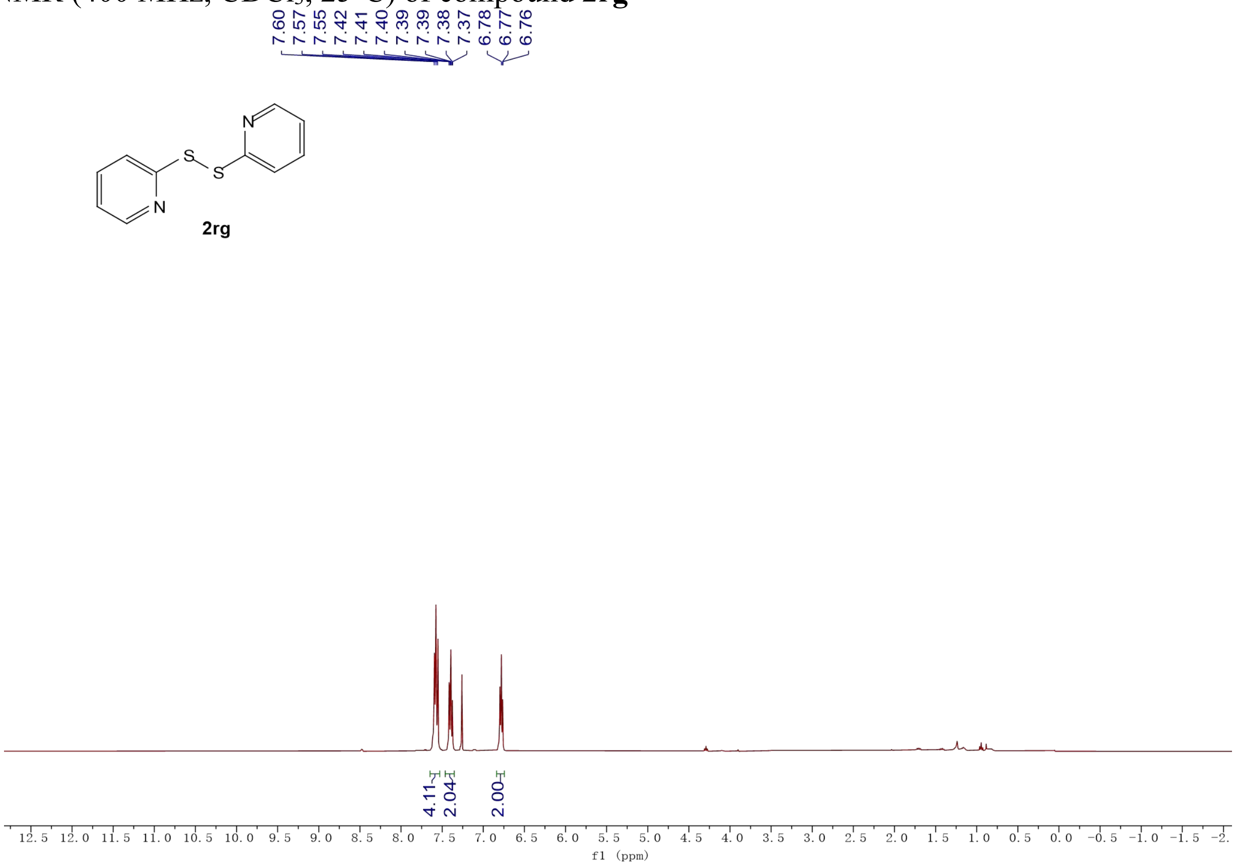

^{13}C NMR (101 MHz, $CDCl_3$, 25°C) of compound **2rg**

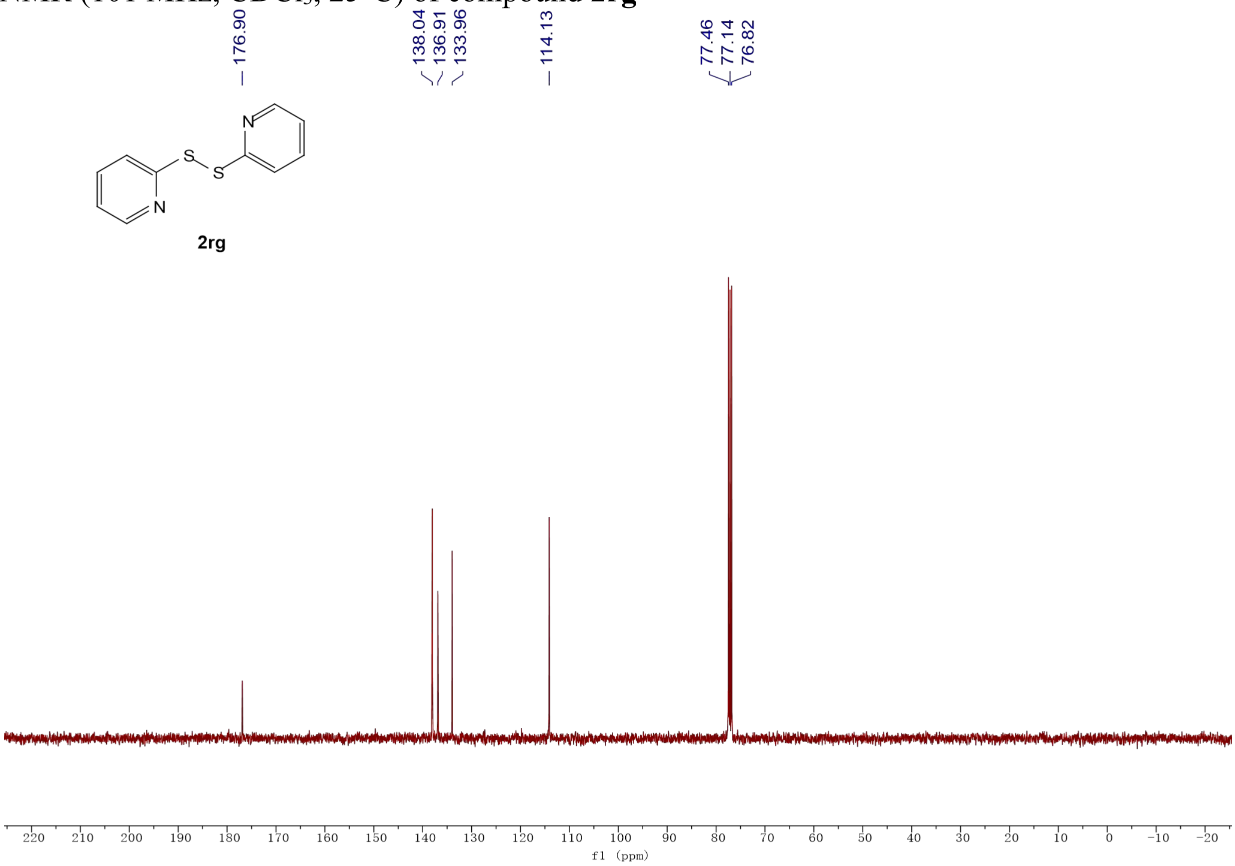

Supplementary Fig. 32. NMR spectra of compound **2rg**

^{1}H NMR (400 MHz, $CDCl_3$, 25°C) of compound **2rh**

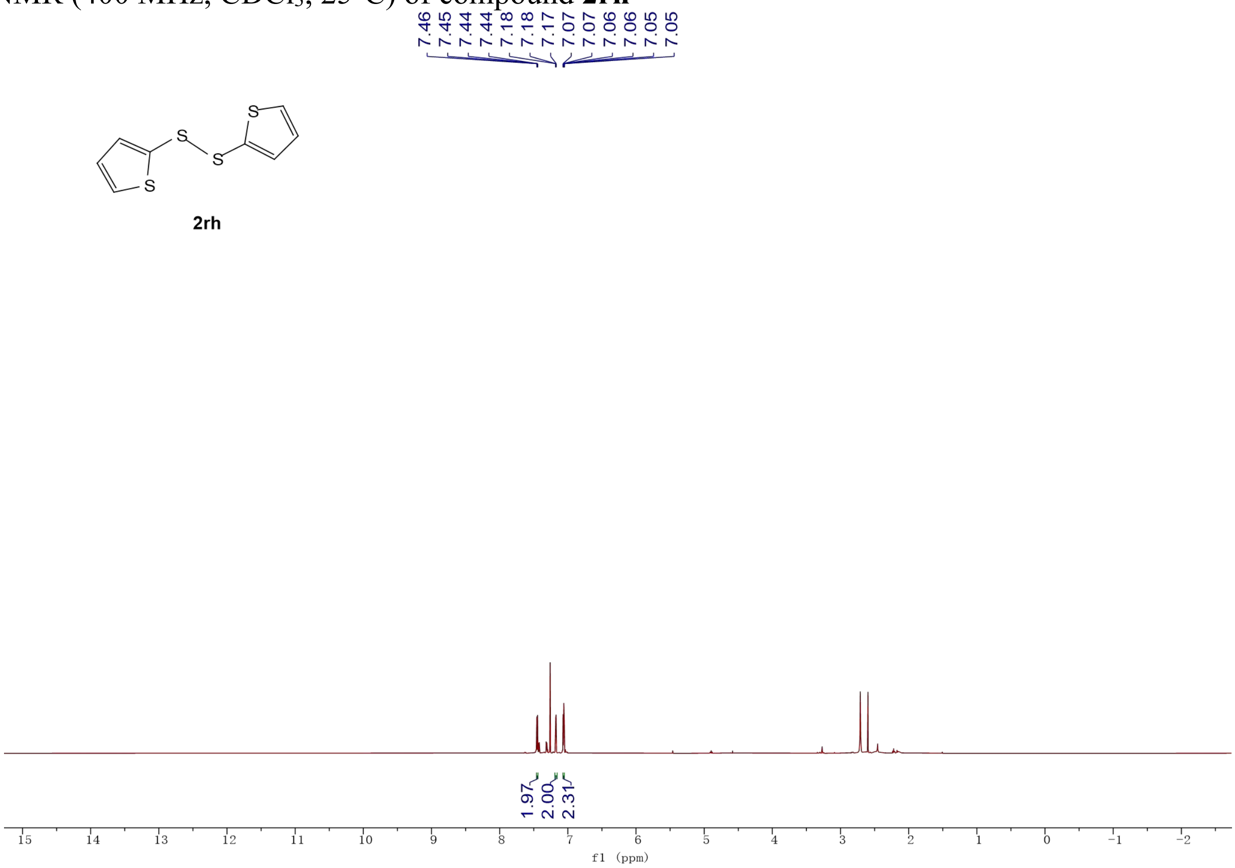

^{13}C NMR (101 MHz, $CDCl_3$, 25°C) of compound **2rh**

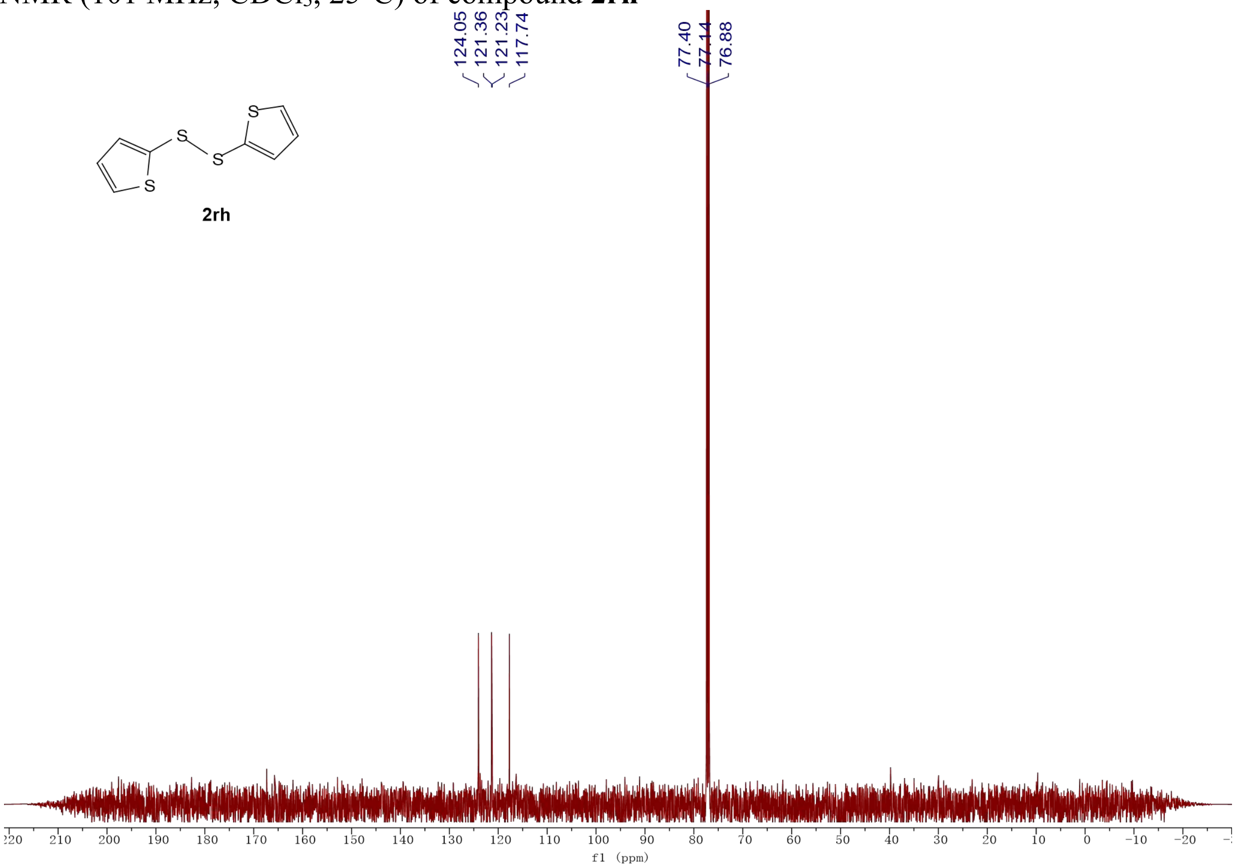

Supplementary Fig. 33. NMR spectra of compound **2rh**

^{1}H NMR (400 MHz, $CDCl_3$, 25°C) of compound **2sa**

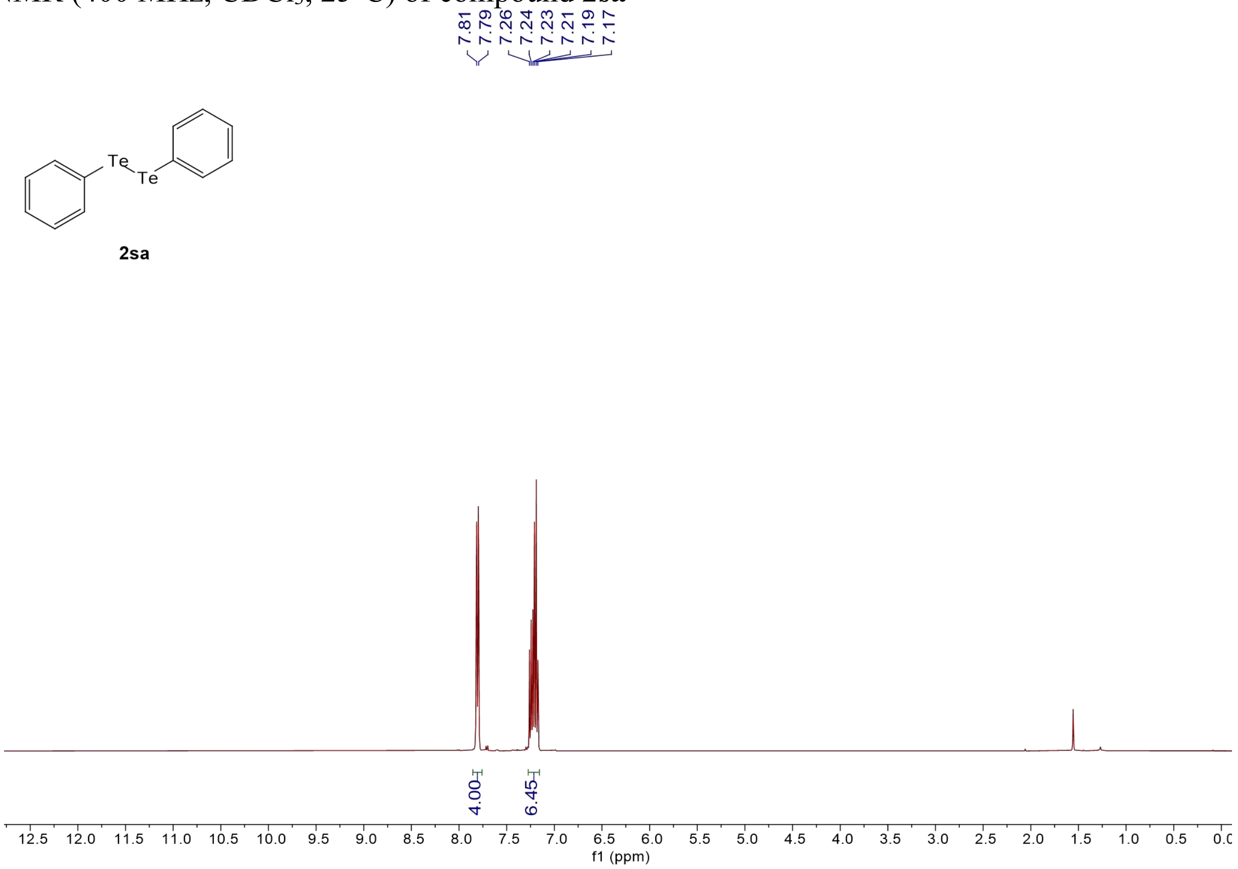

^{13}C NMR (101 MHz, $CDCl_3$, 25°C) of compound **2sa**

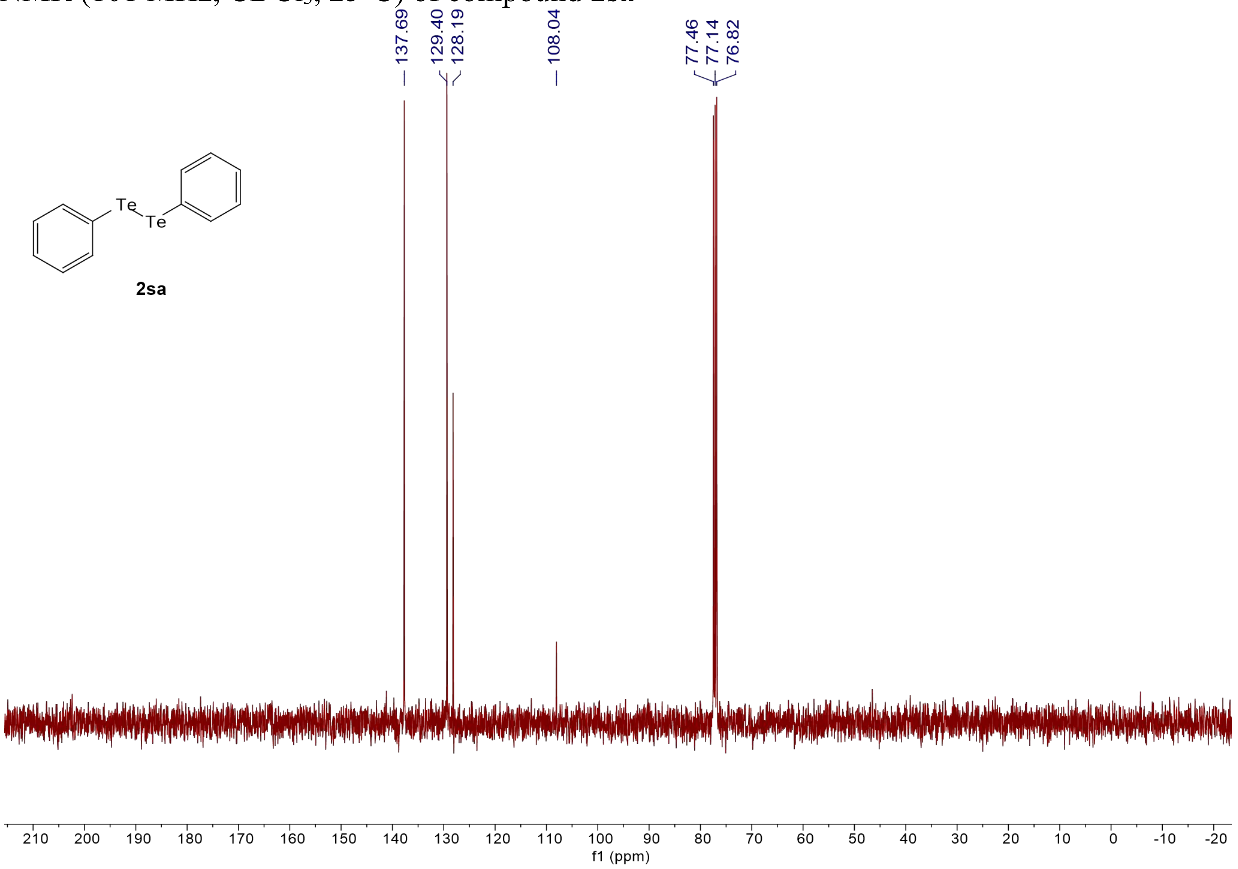

Supplementary Fig. 34. NMR spectra of compound **2sa**

^{1}H NMR (400 MHz, $CDCl_3$, 25°C) of compound **2sb**

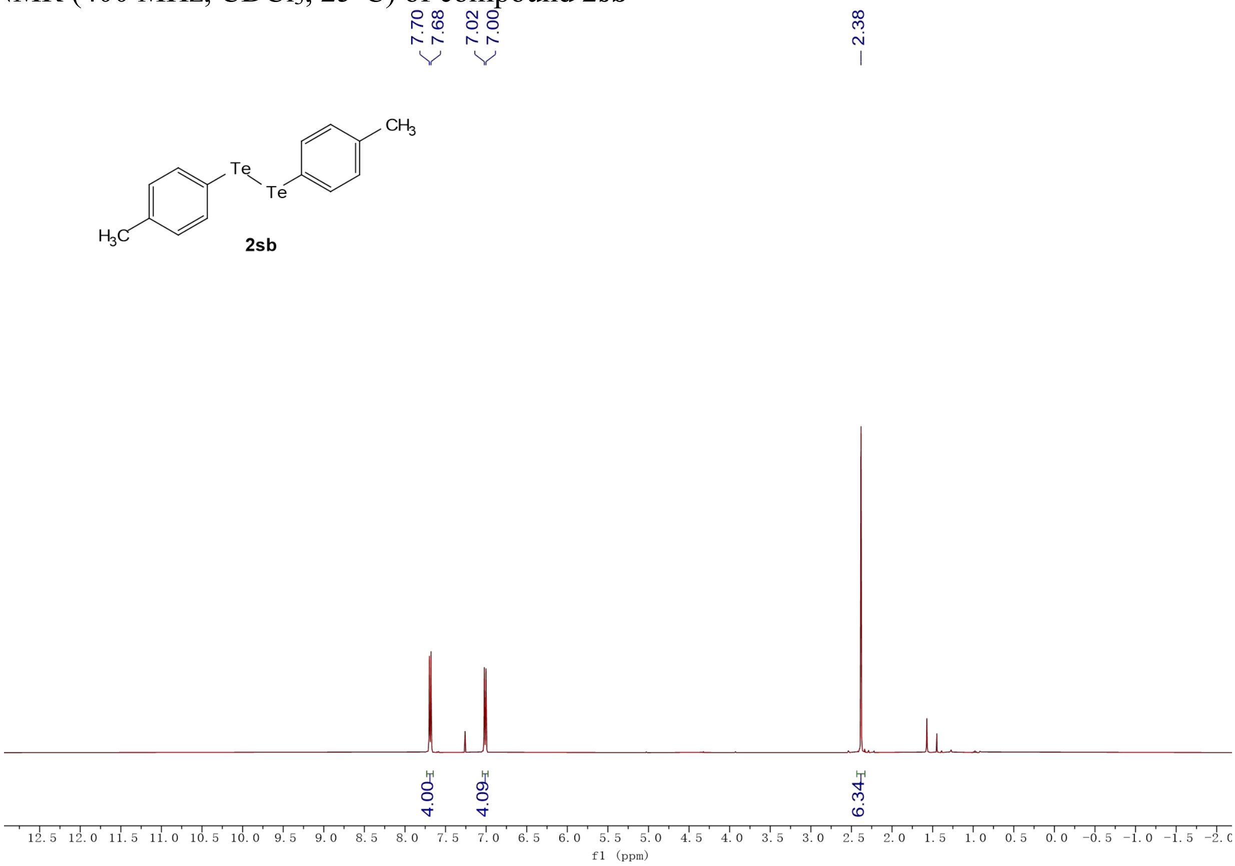

^{13}C NMR (101 MHz, $CDCl_3$, 25°C) of compound **2sb**

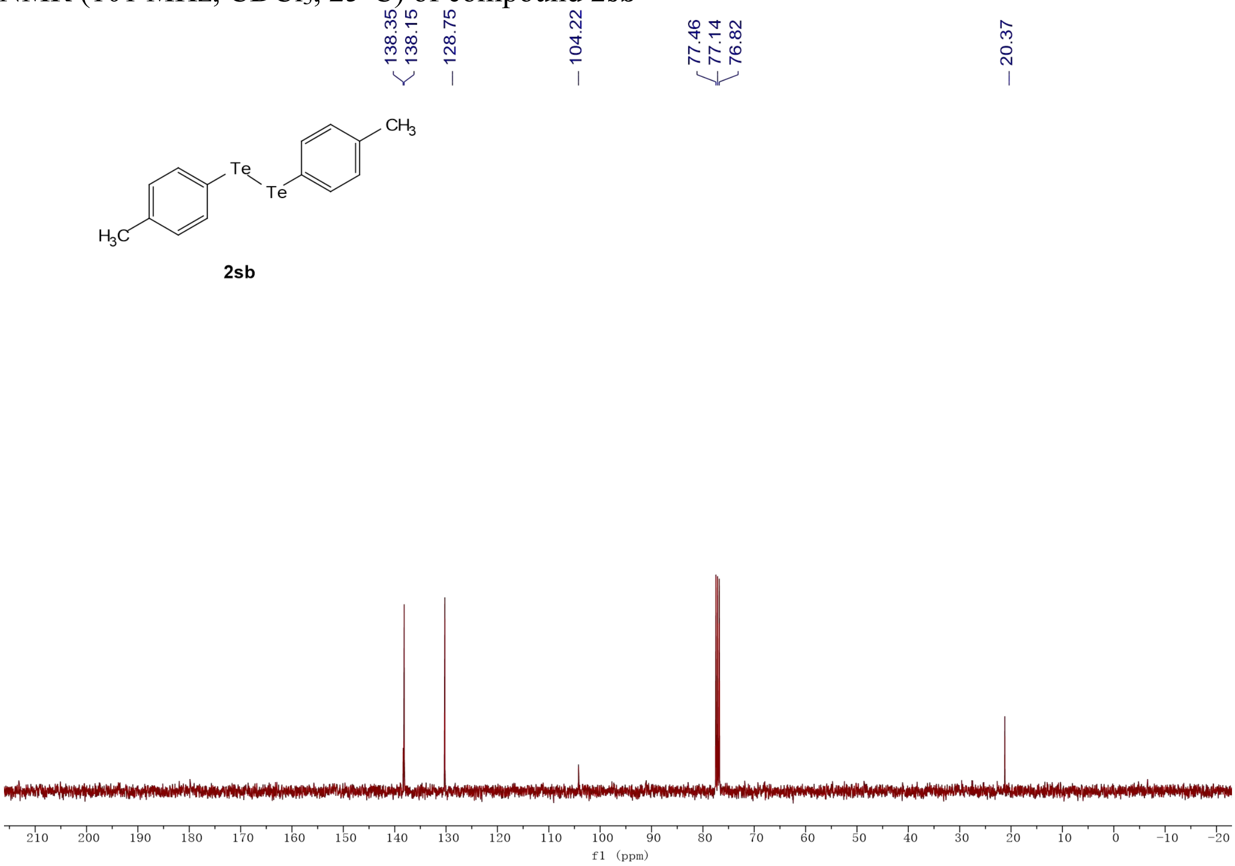

Supplementary Fig. 35. NMR spectra of compound **2sb**

^1H NMR (400 MHz, $CDCl_3$, 25°C) of compound **2sc**

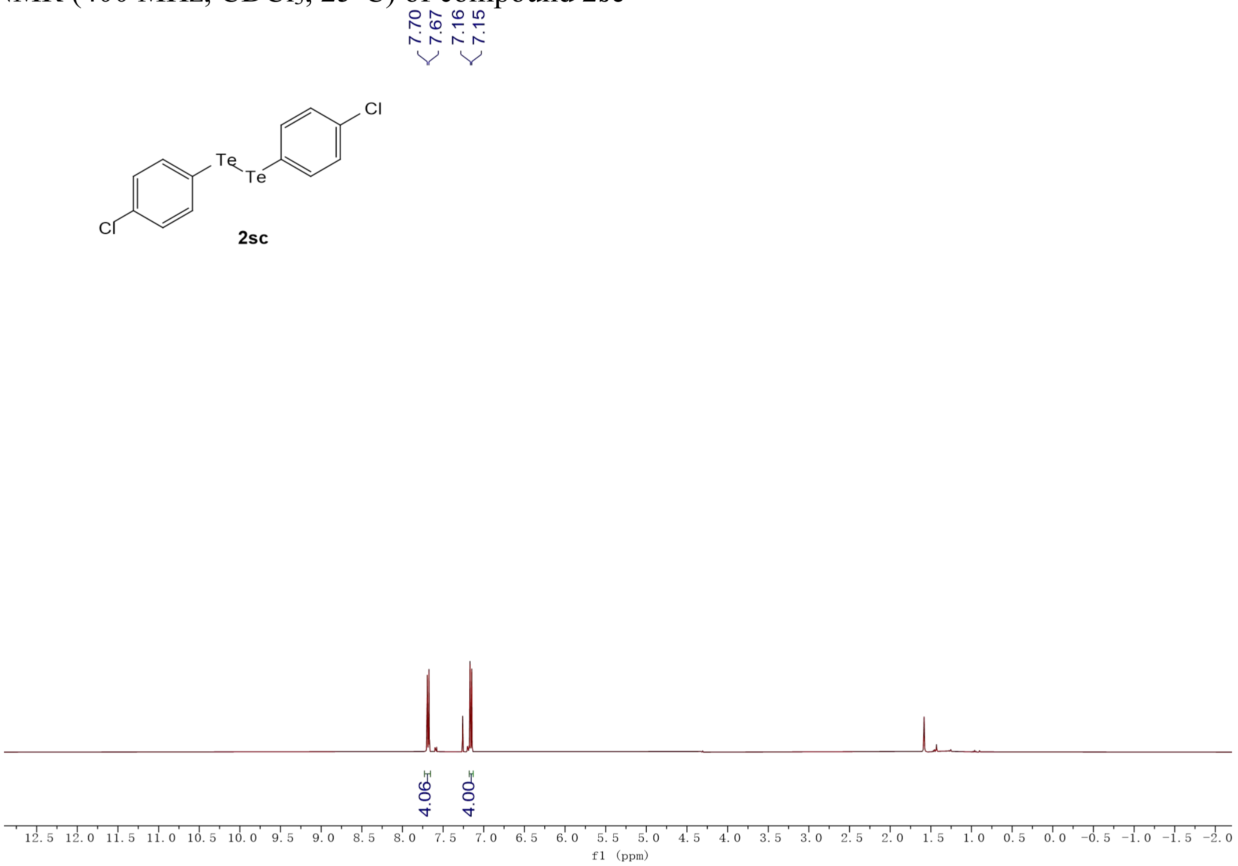

^{13}C NMR (101 MHz, $CDCl_3$, 25°C) of compound **2sc**

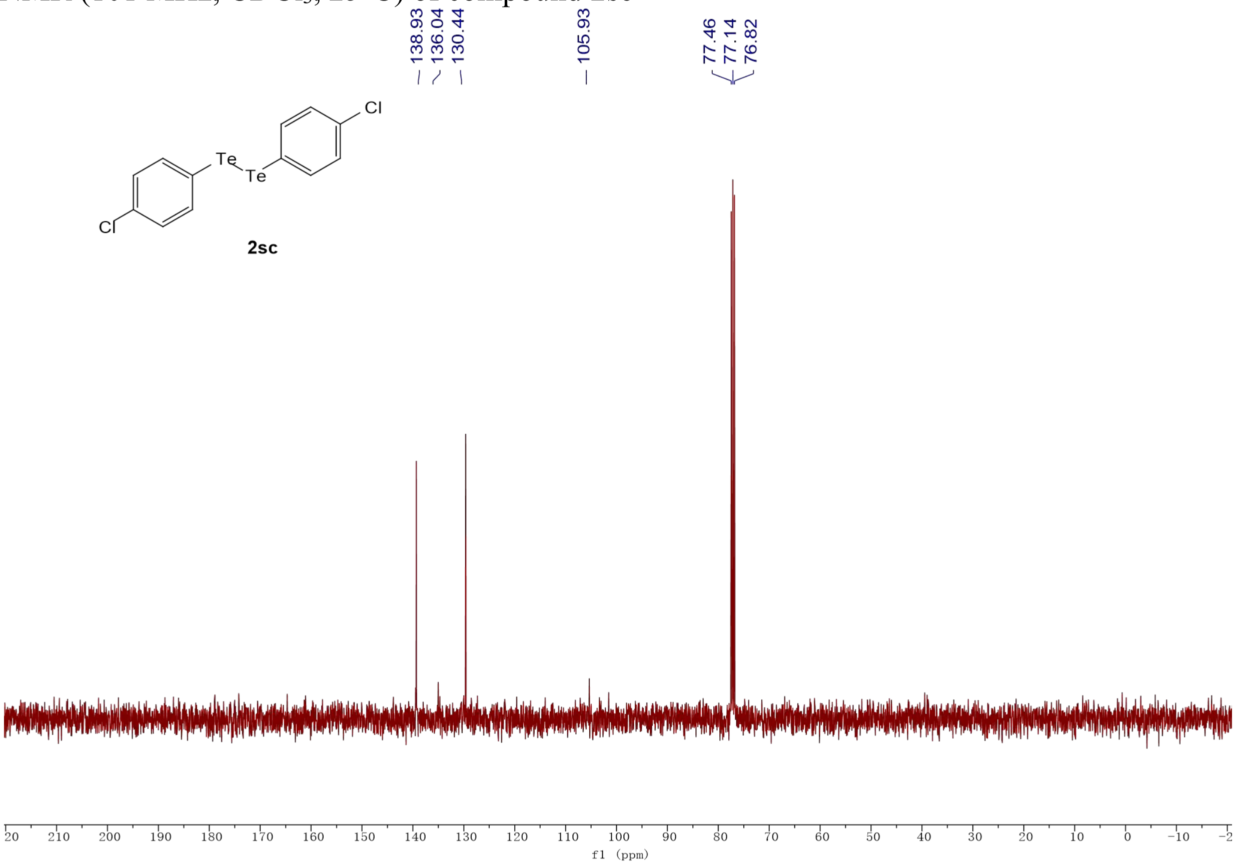

Supplementary Fig. 36. NMR spectra of compound **2sc**

^{1}H NMR (400 MHz, $CDCl_3$, 25°C) of compound **2sd**

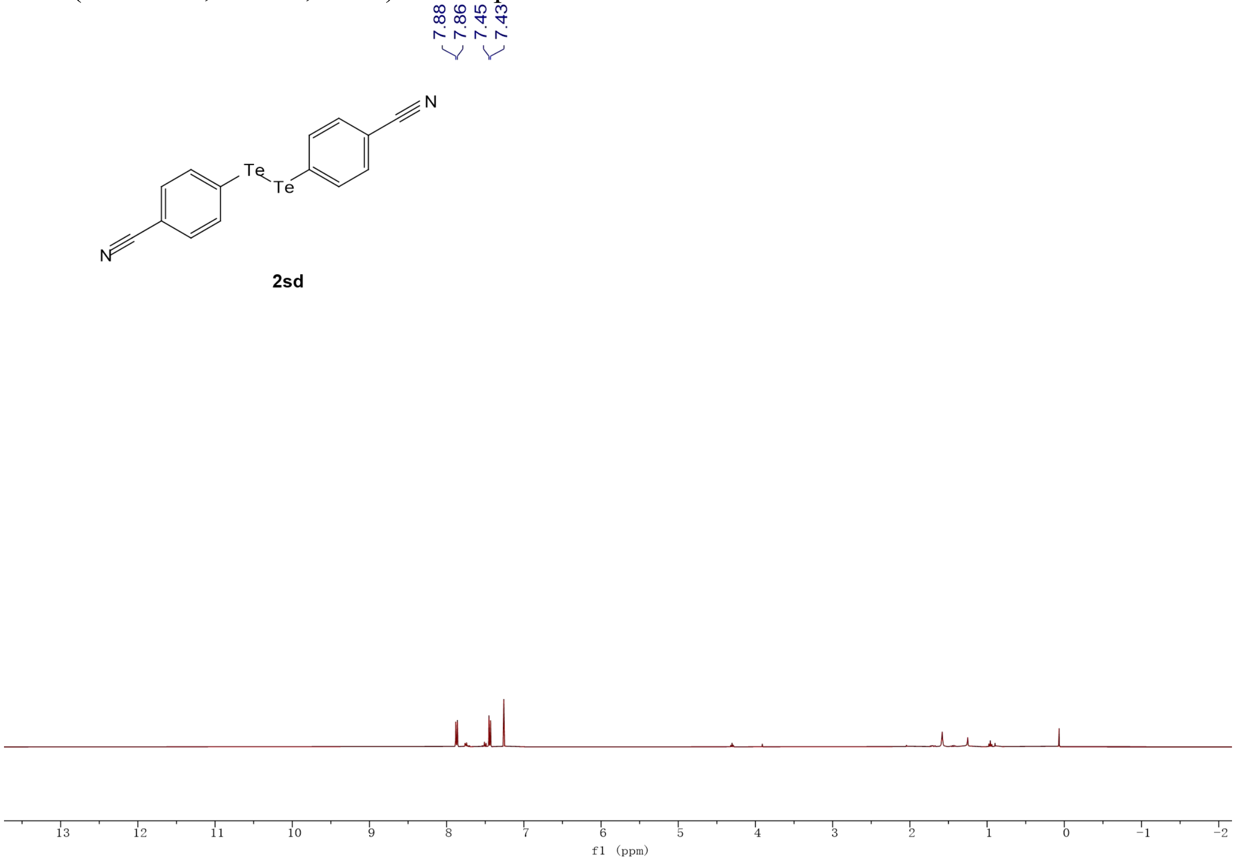

^{13}C NMR (101 MHz, $CDCl_3$, 25°C) of compound **2sd**

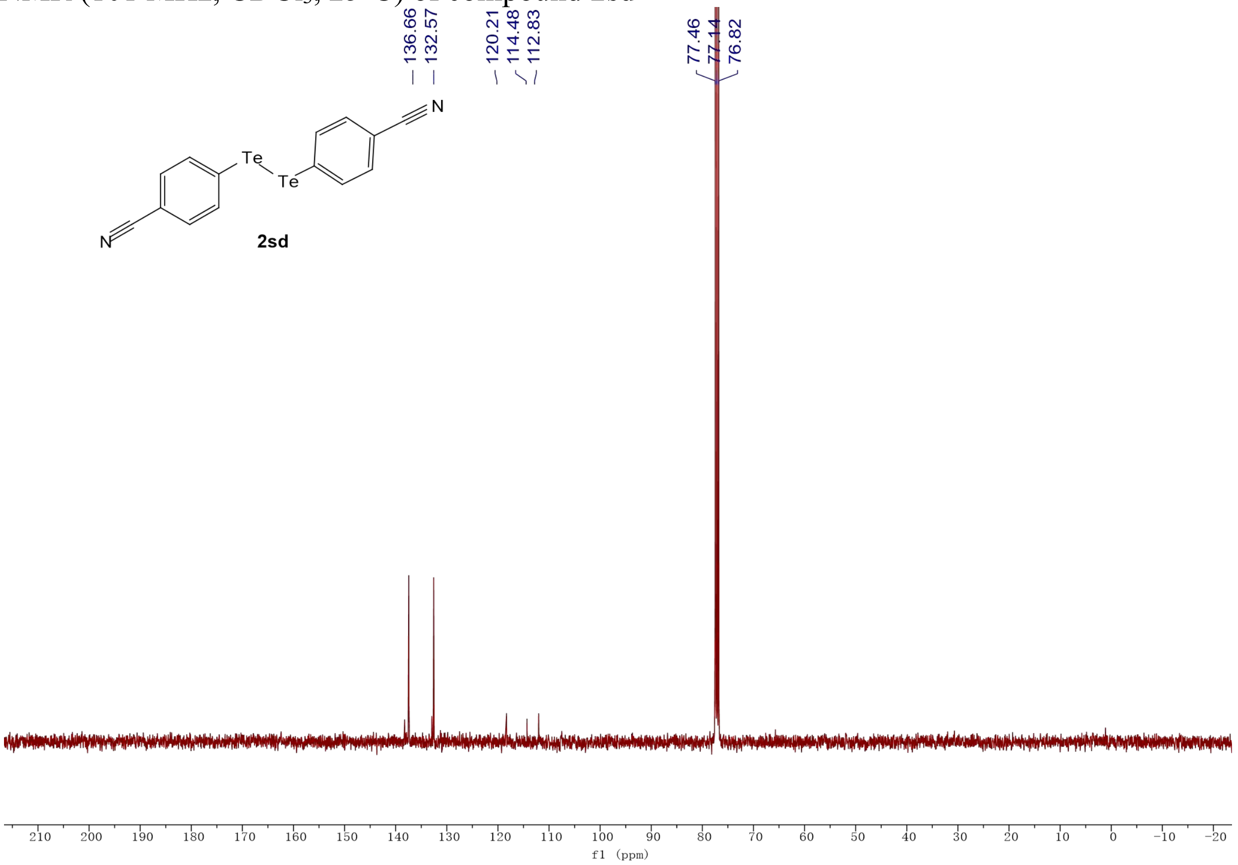

Supplementary Fig. 37. NMR spectra of compound **2sd**

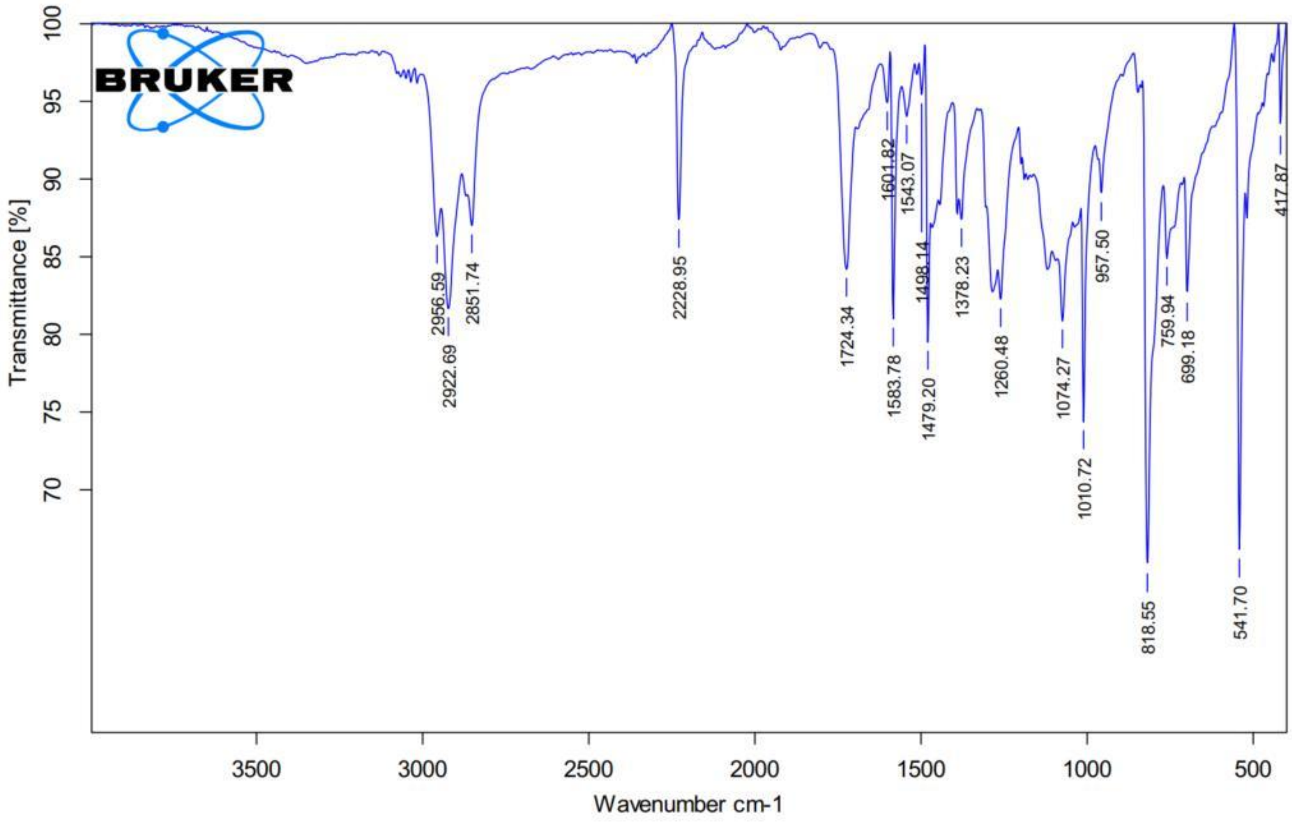

Supplementary Fig. 38. IR of compound **2sd**

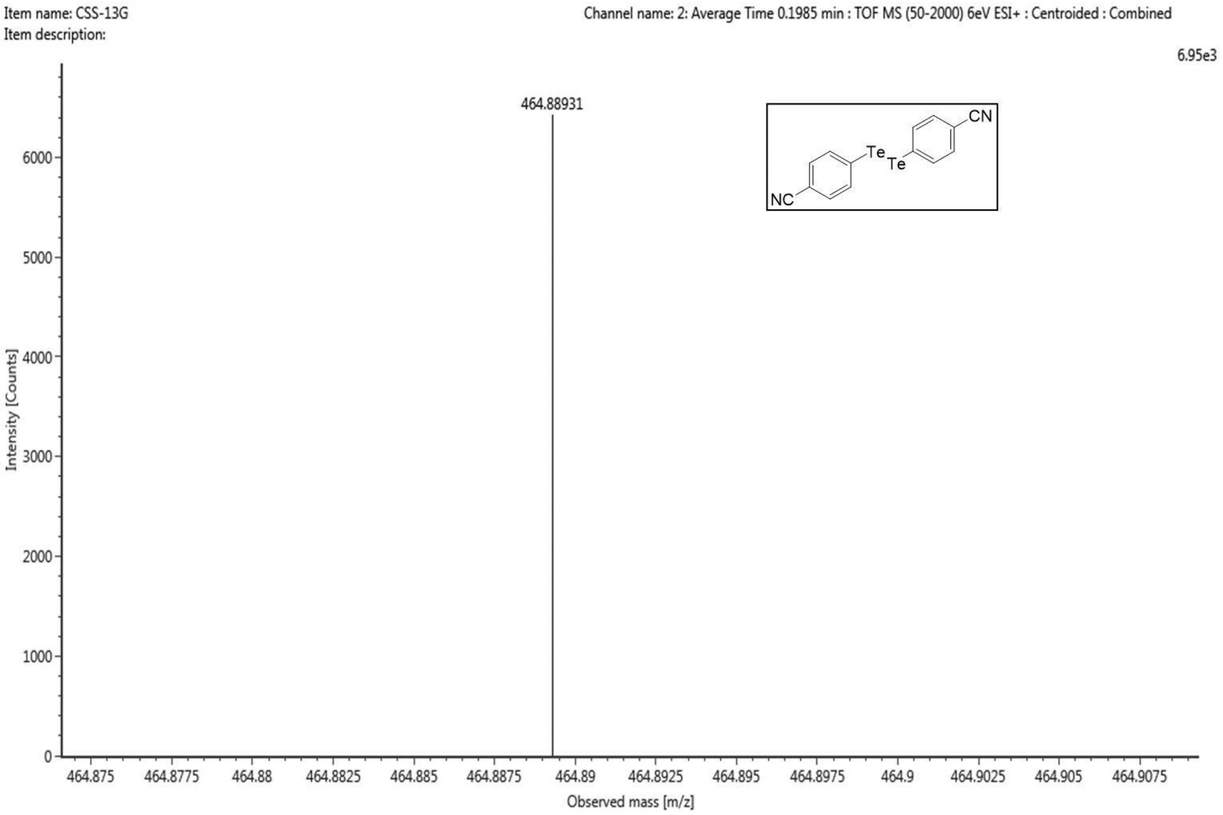

Supplementary Fig. 39. HR-MS of compound **2sd**

^{1}H NMR (400 MHz, $CDCl_3$, 25°C) of compound **2se**

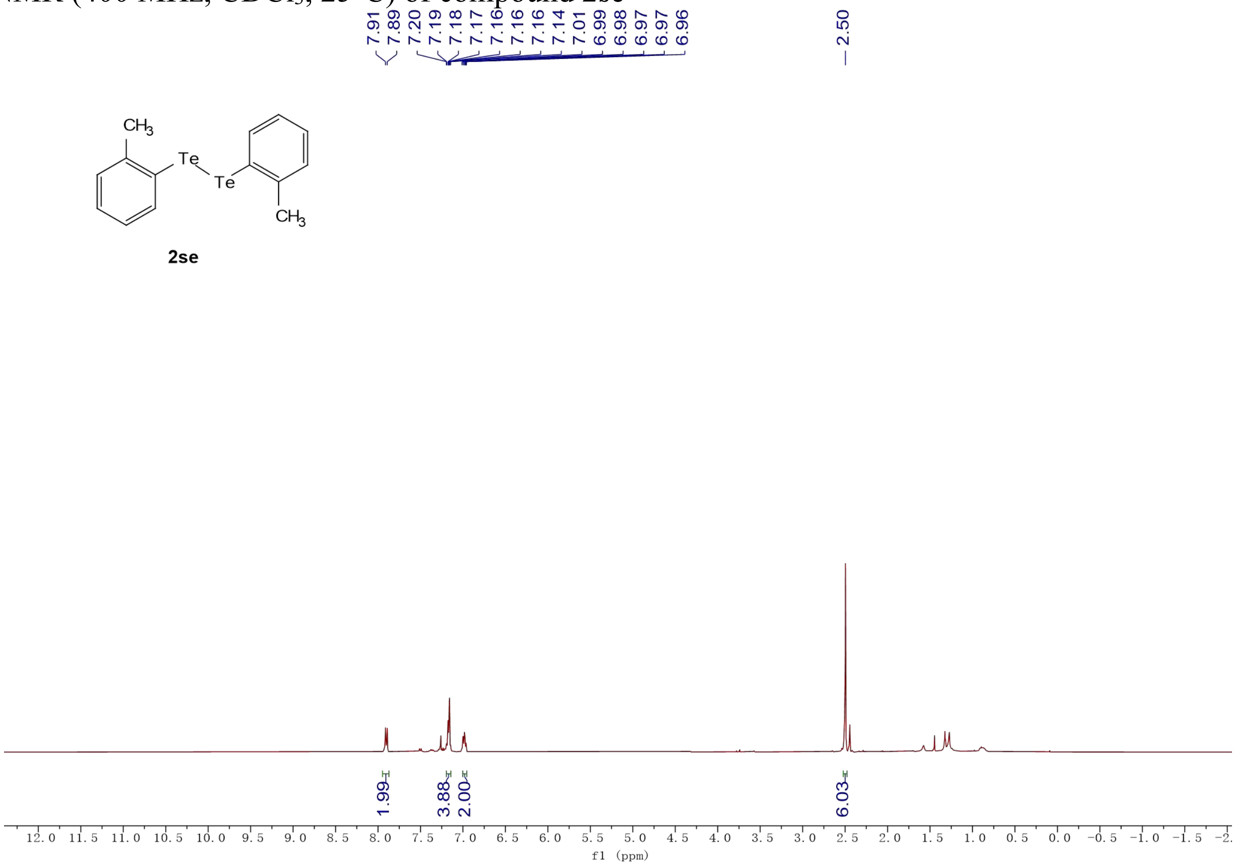

^{13}C NMR (101 MHz, $CDCl_3$, 25°C) of compound **2se**

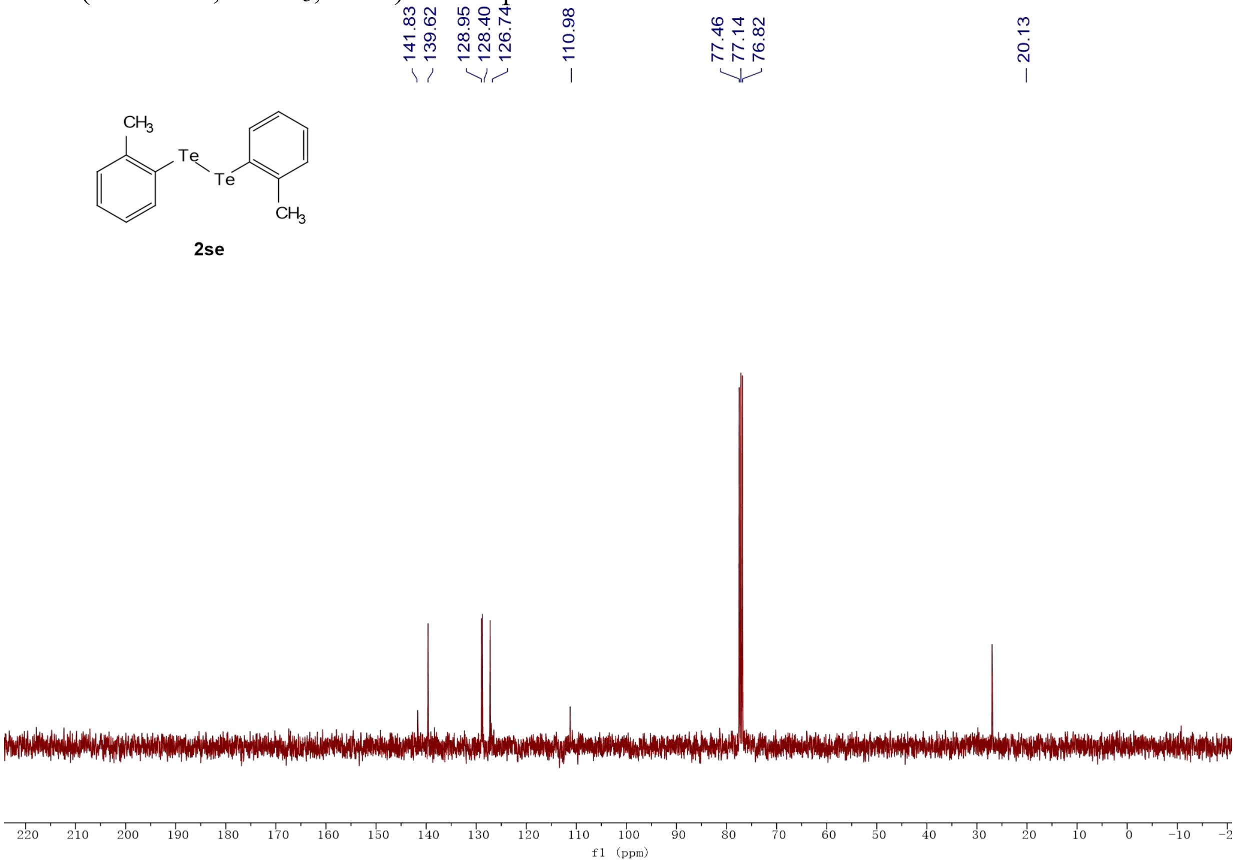

Supplementary Fig. 40. NMR spectra of compound **2se**

^{1}H NMR (400 MHz, $CDCl_3$, 25℃) of compound **2sf**

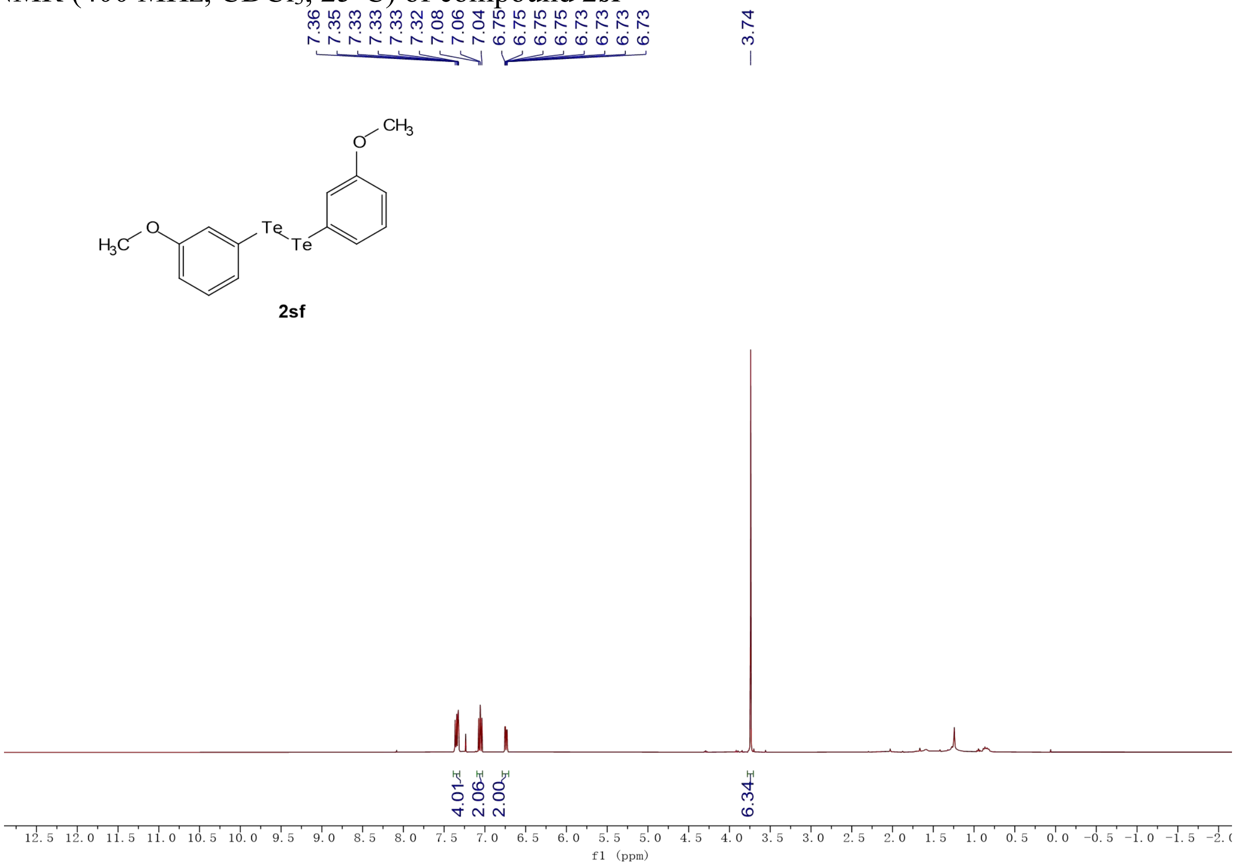

^{13}C NMR (101 MHz, $CDCl_3$, 25℃) of compound **2sf**

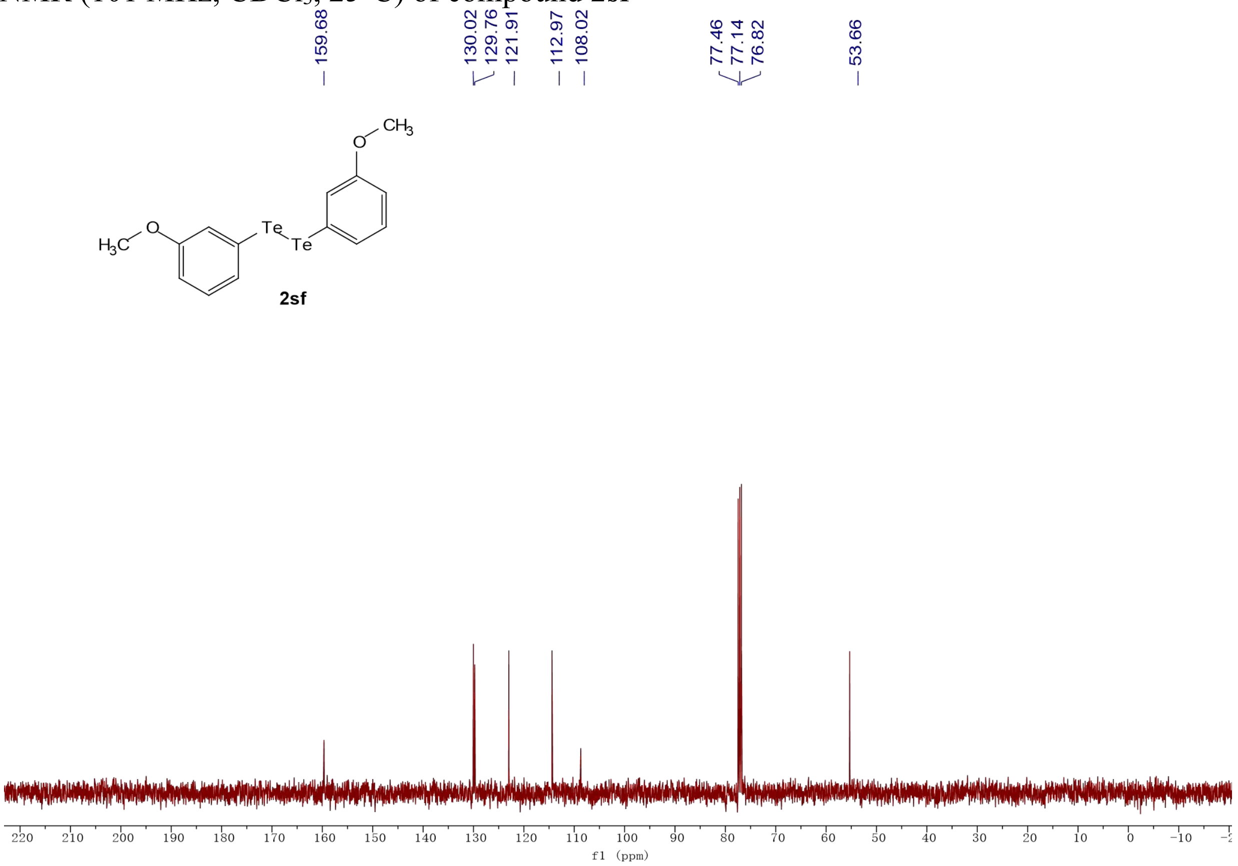

Supplementary Fig. 41. NMR spectra of compound **2sf**

^{1}H NMR (400 MHz, $CDCl_3$, 25°C) of compound **2sg**

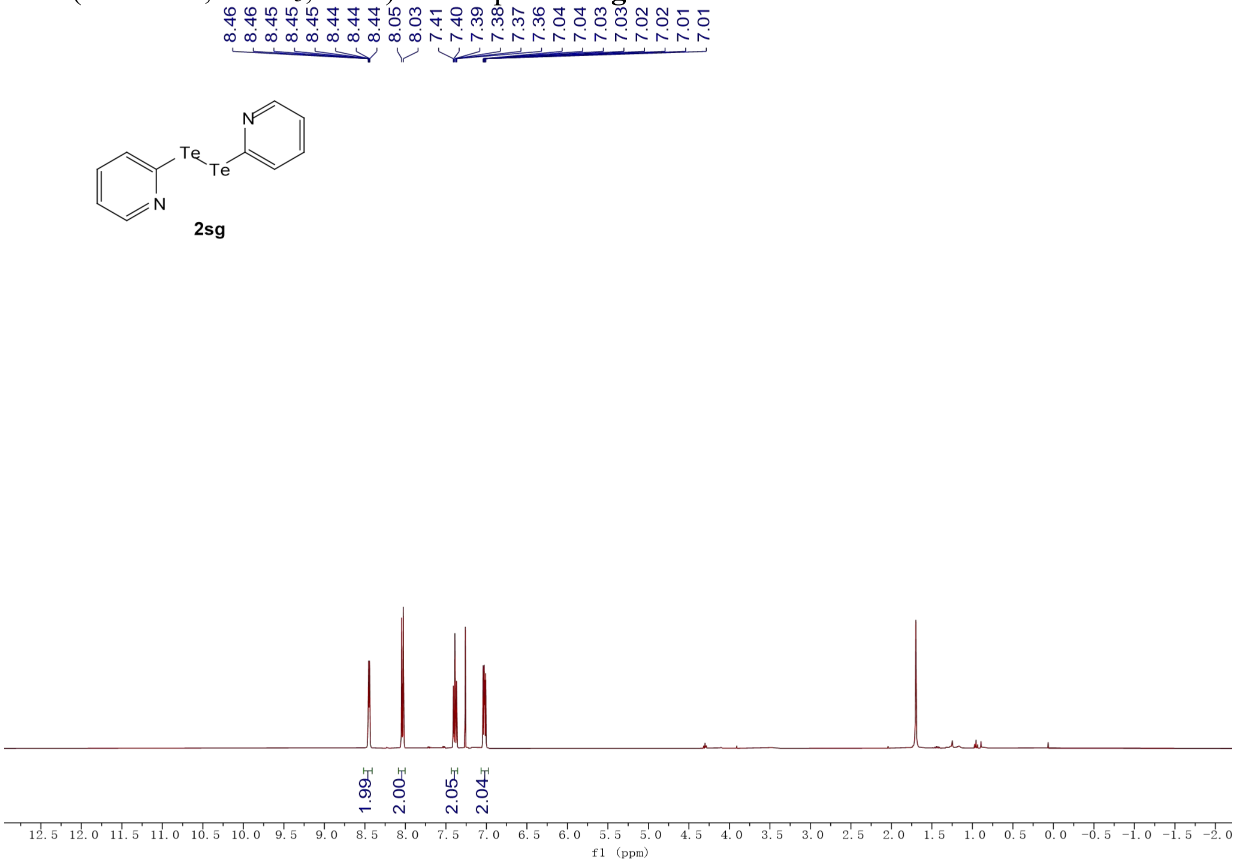

^{13}C NMR (101 MHz, $CDCl_3$, 25°C) of compound **2sg**

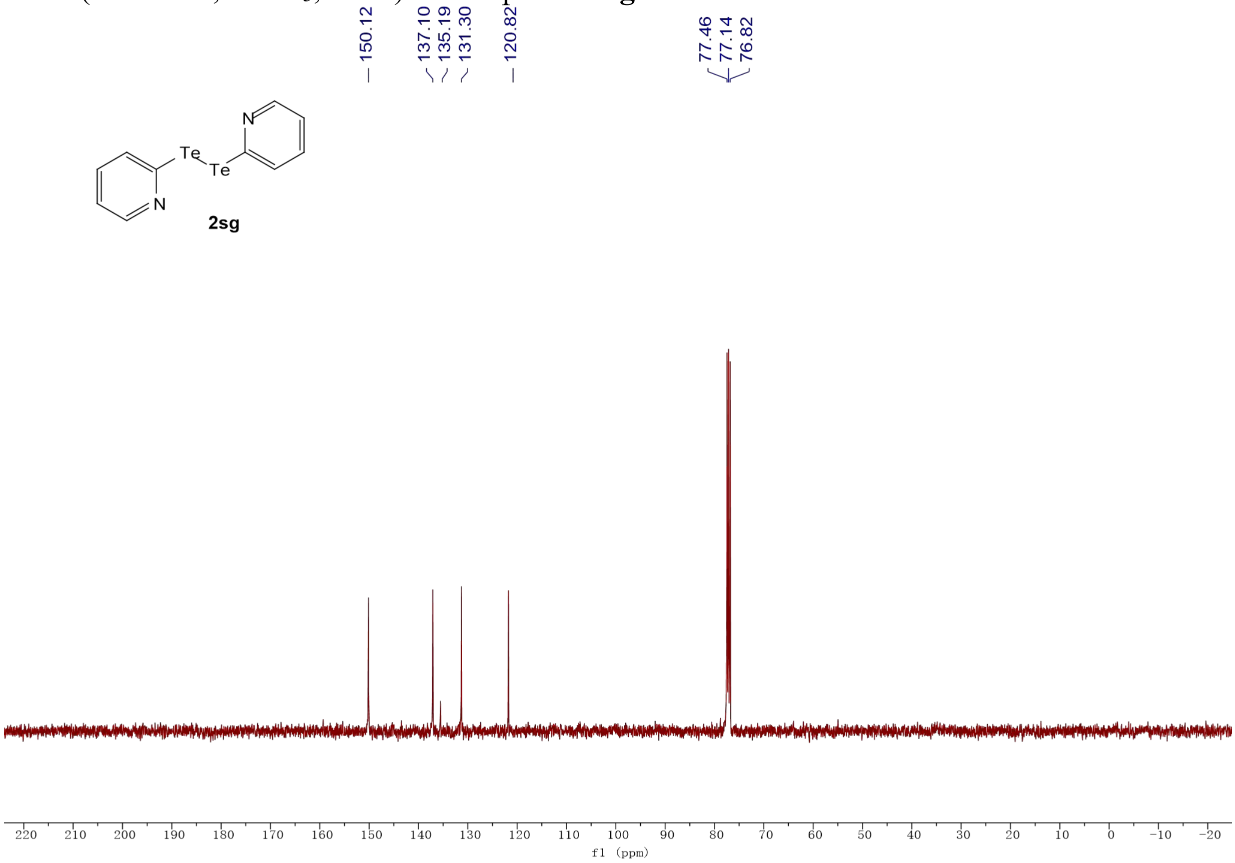

Supplementary Fig. 42. NMR spectra of compound **2sg**

^{1}H NMR (400 MHz, $CDCl_3$, 25°C) of compound **2sh**

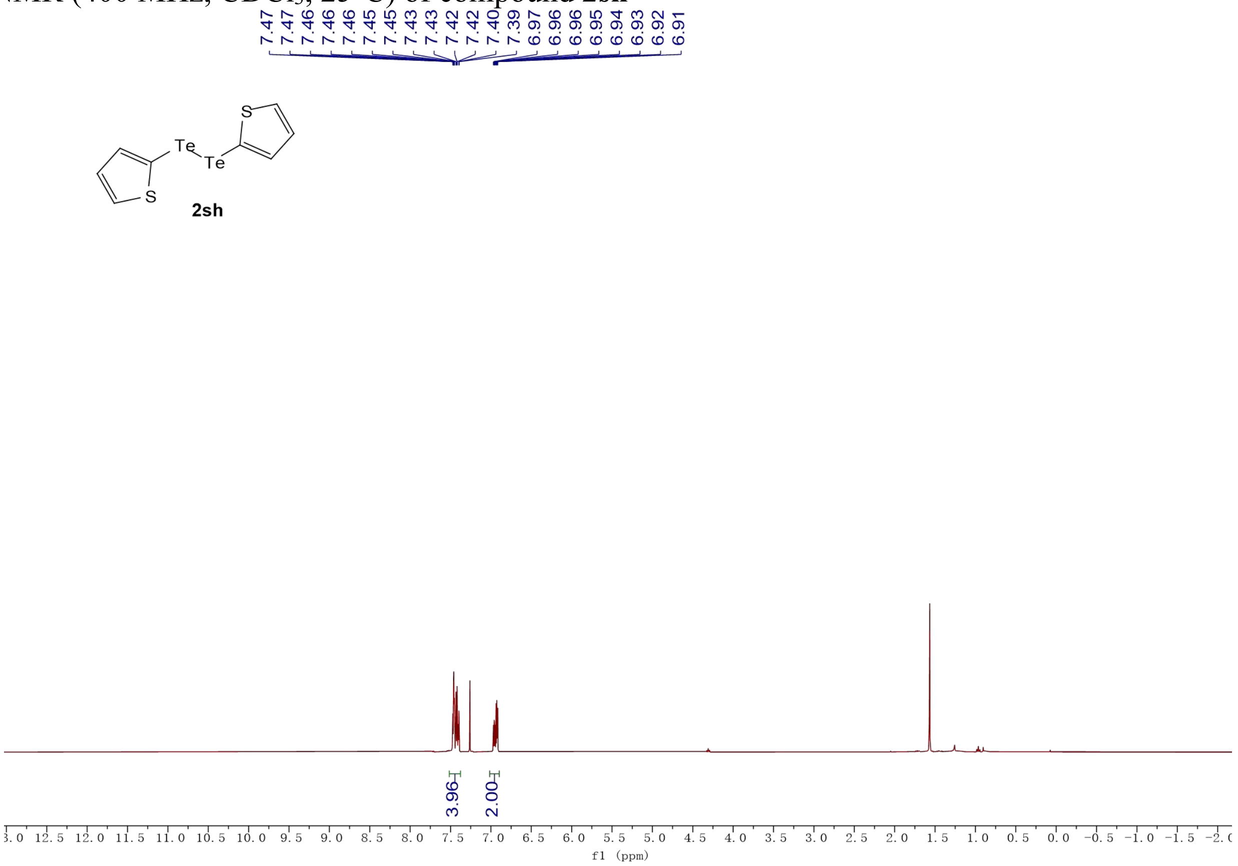

^{13}C NMR (101 MHz, $CDCl_3$, 25°C) of compound **2sh**

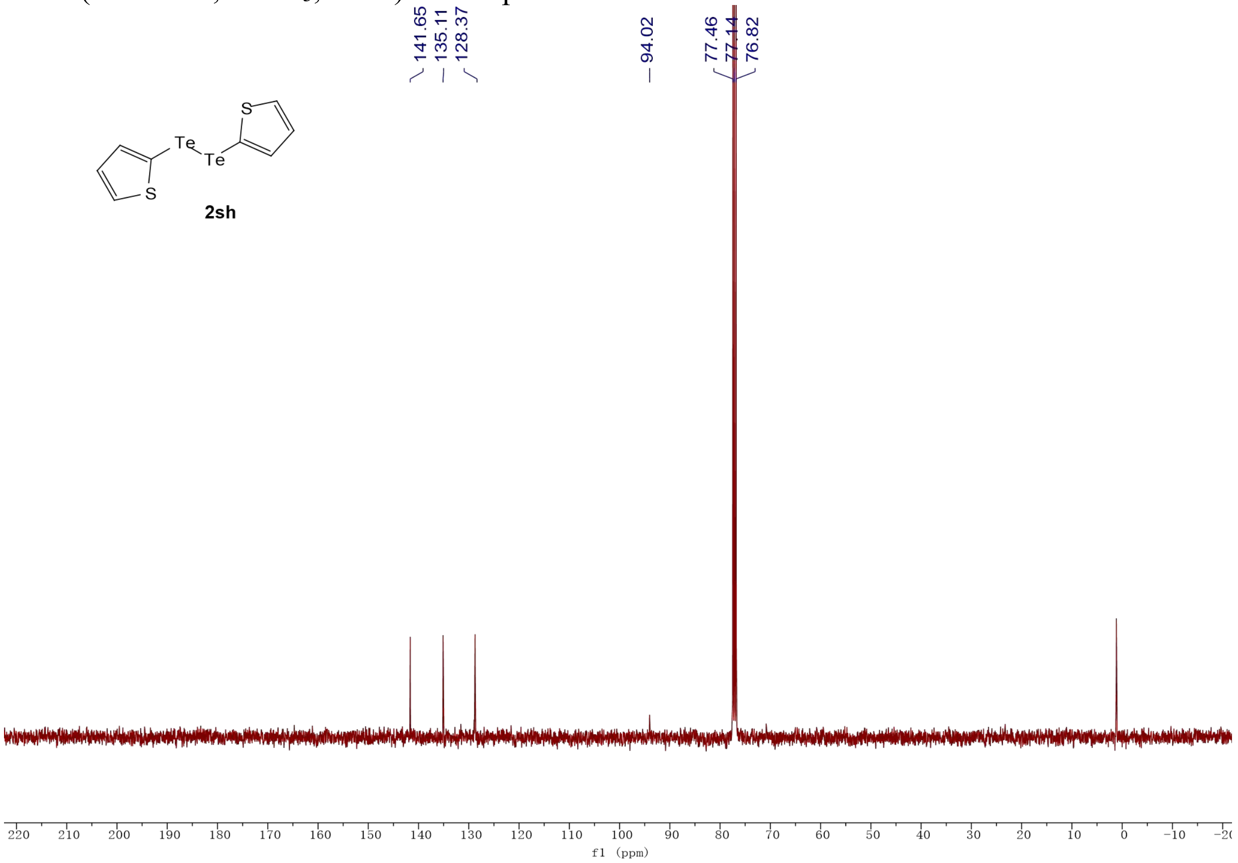

Supplementary Fig. 43. NMR spectra of compound **2sh**

^{1}H NMR (400 MHz, $CDCl_3$, 25°C) of compound **4a**

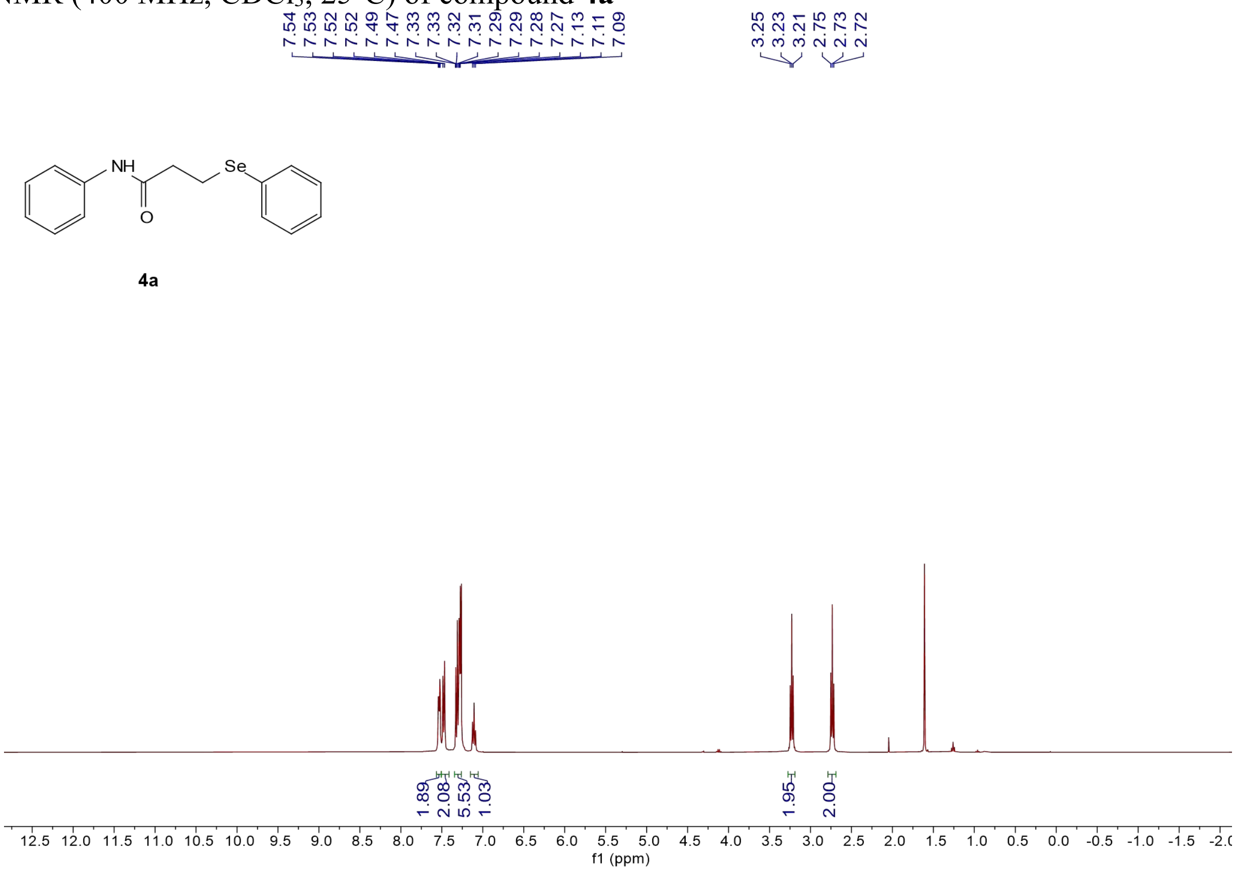

^{13}C NMR (101 MHz, $CDCl_3$, 25°C) of compound **4a**

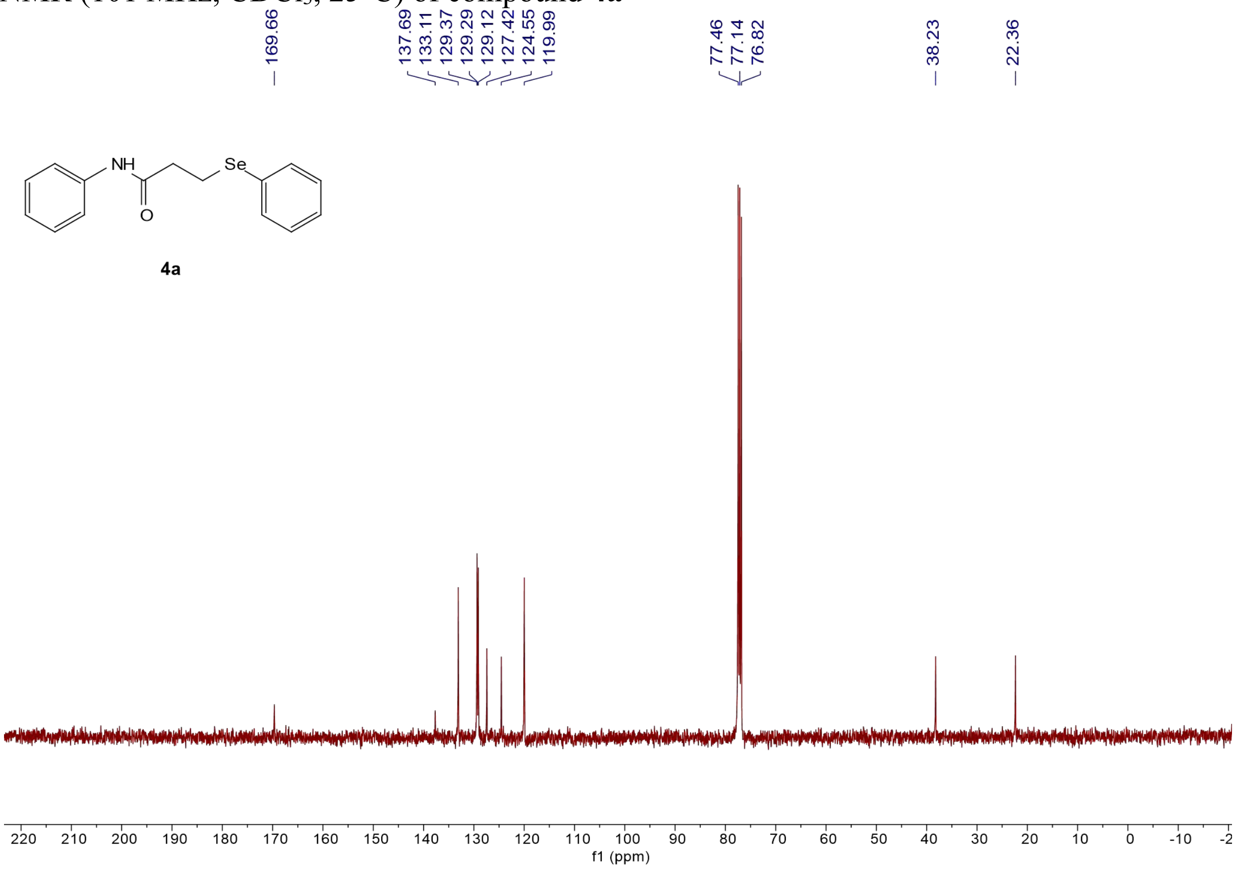

^{77}Se NMR (76 MHz, $CDCl_3$, 25°C) of compound **4a**

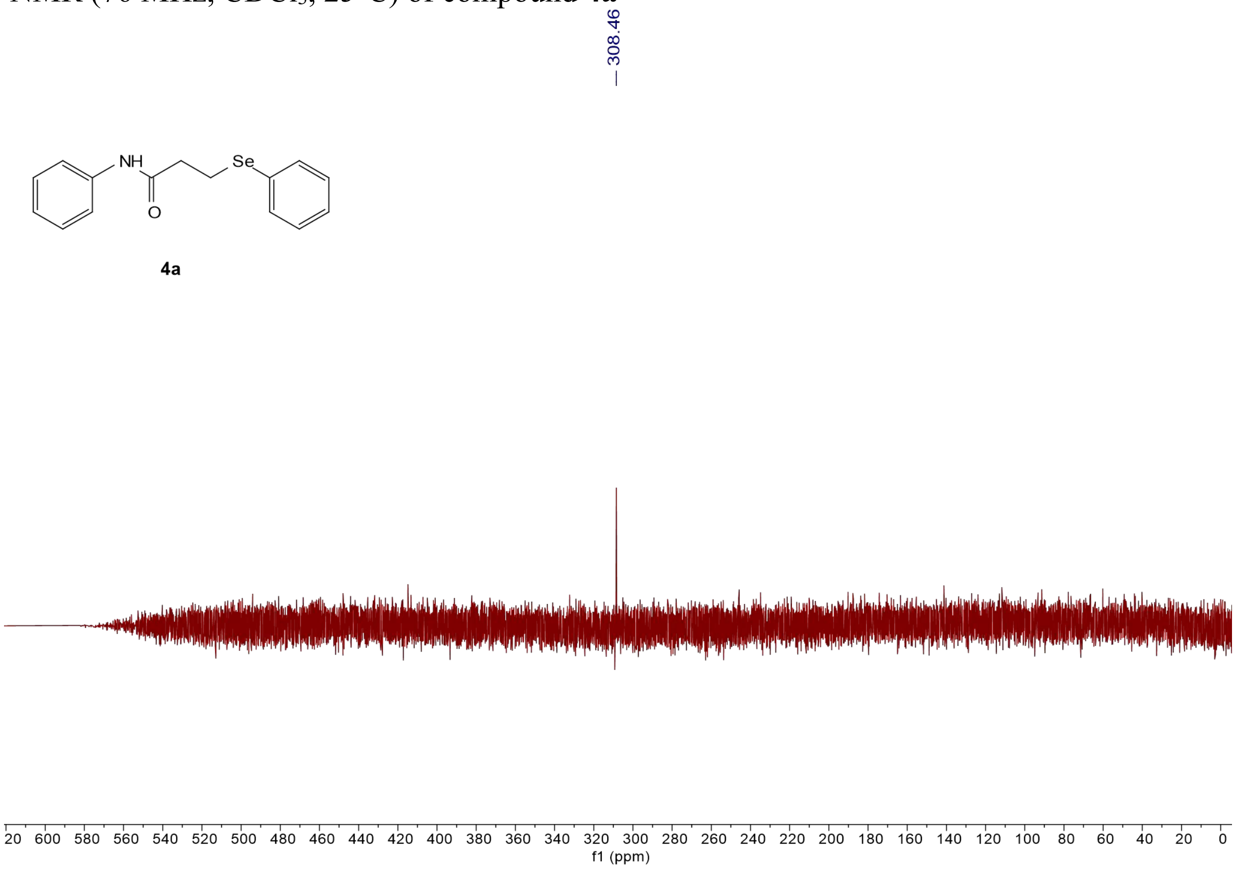

Supplementary Fig. 44. NMR spectra of compound **4a**

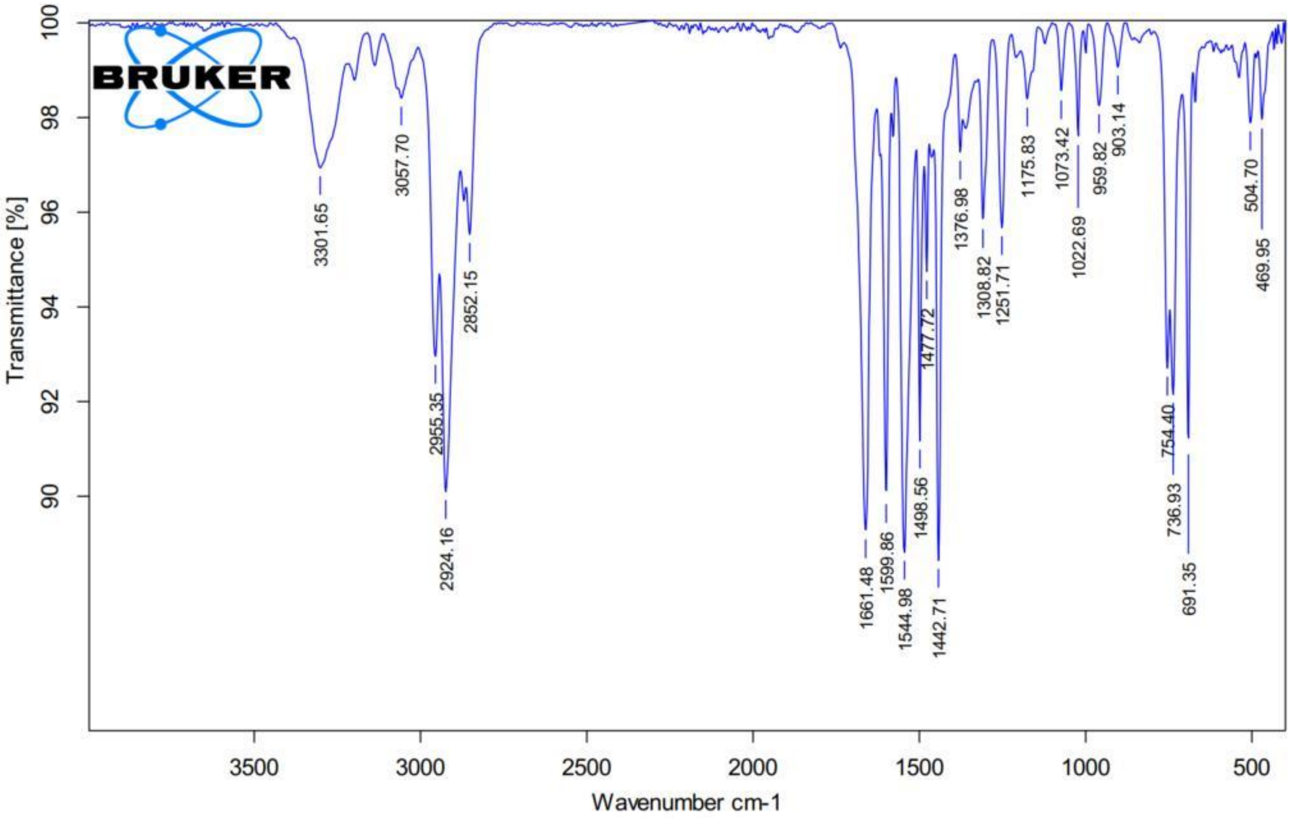

Supplementary Fig. 45. IR of compound **4a**

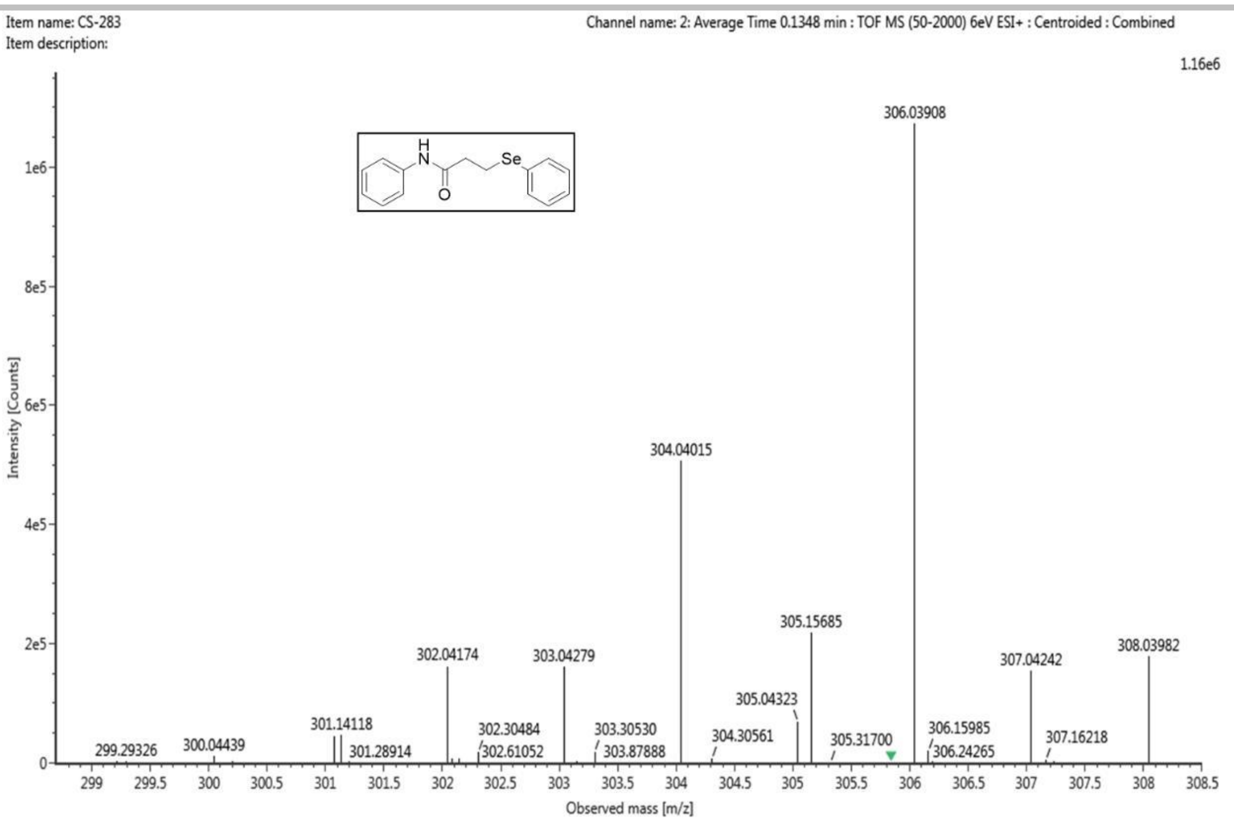

Supplementary Fig. 46. HR-MS of compound **4a**

^{1}H NMR (400 MHz, $CDCl_3$, 25℃) of compound **4b**

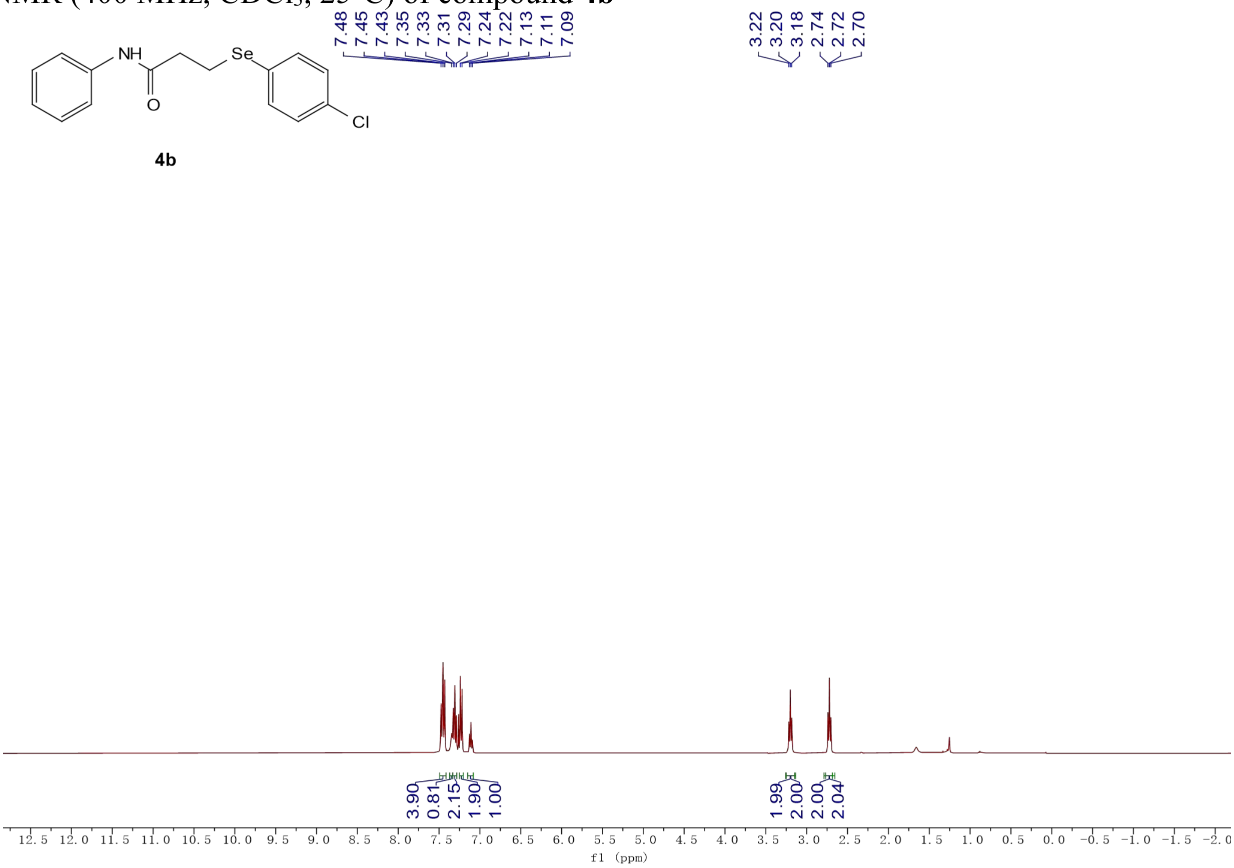

^{13}C NMR (101 MHz, $CDCl_3$, 25°C) of compound **4b**

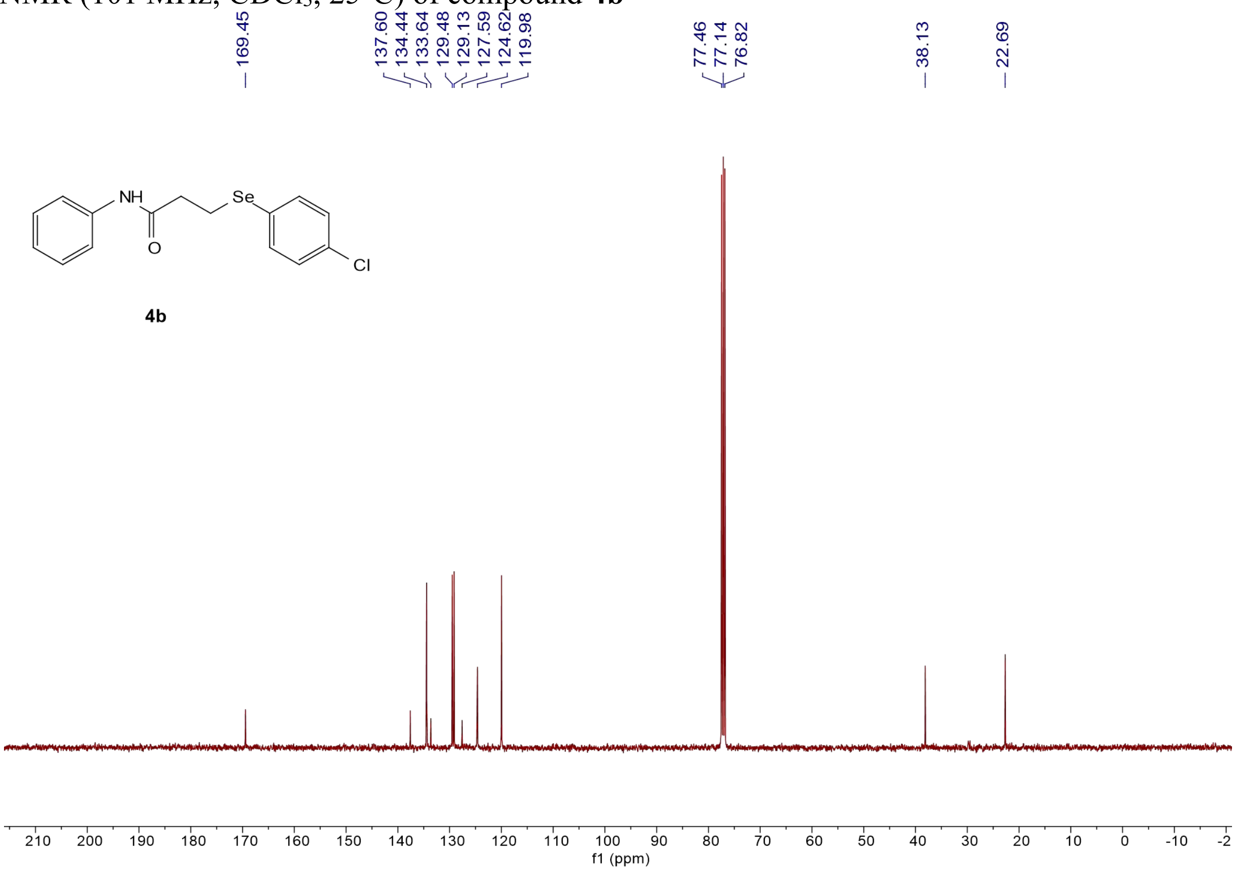

^{77}**Se NMR** (76 MHz, CDCl3, 25°C) of compound **4b**

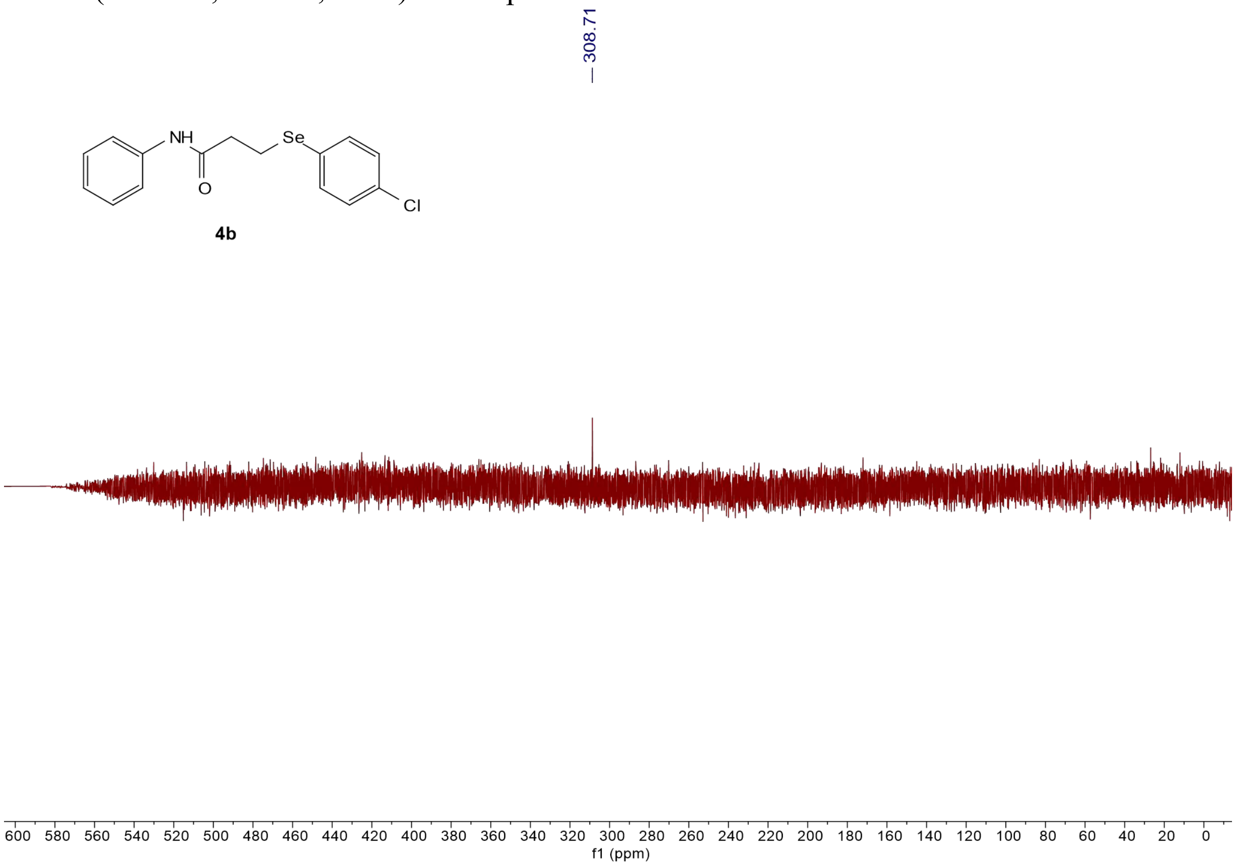

Supplementary Fig. 47. NMR spectra of compound **4b**

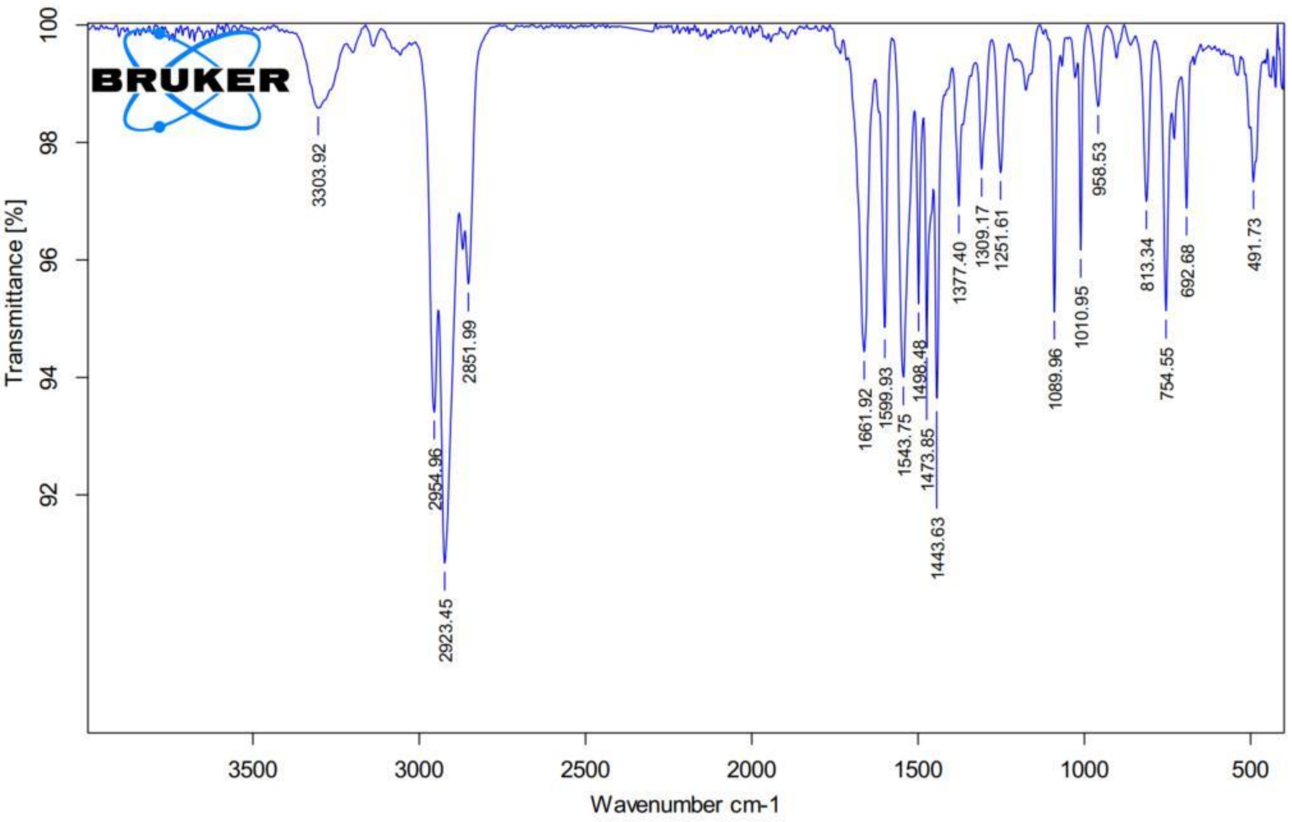

Supplementary Fig. 48. IR of compound **4b**

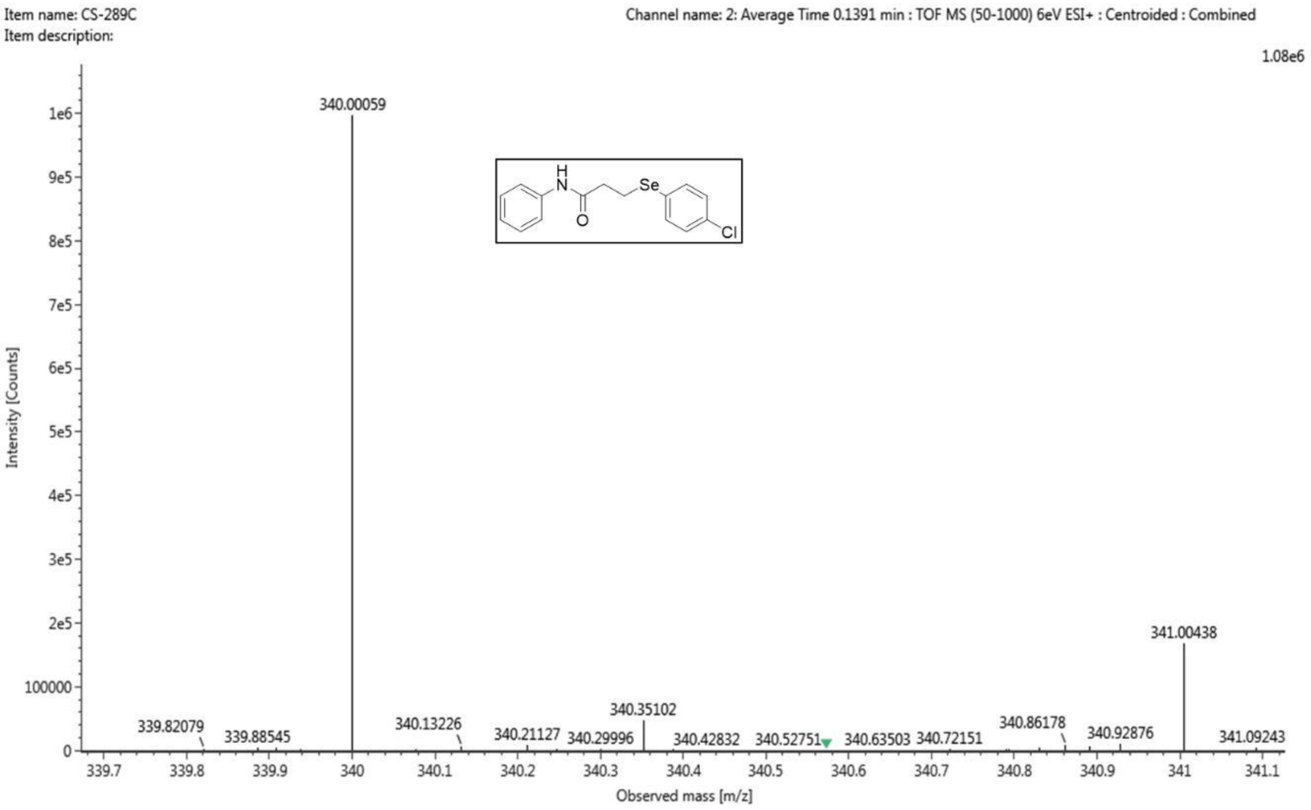

Supplementary Fig. 49. HR-MS of compound **4b**

^{1}H NMR (400 MHz, $CDCl_3$, 25°C) of compound **4c**

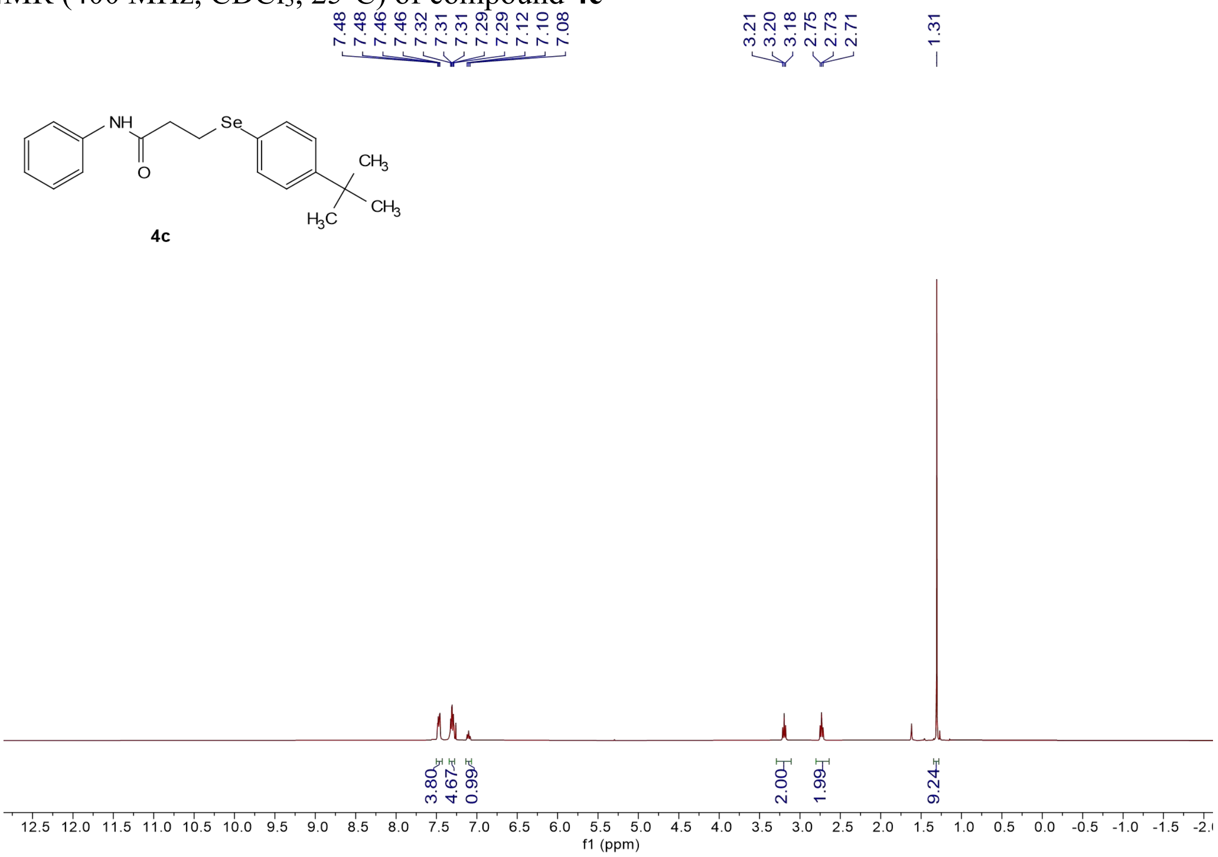

^{13}C NMR (101 MHz, $CDCl_3$, 25°C) of compound **4c**

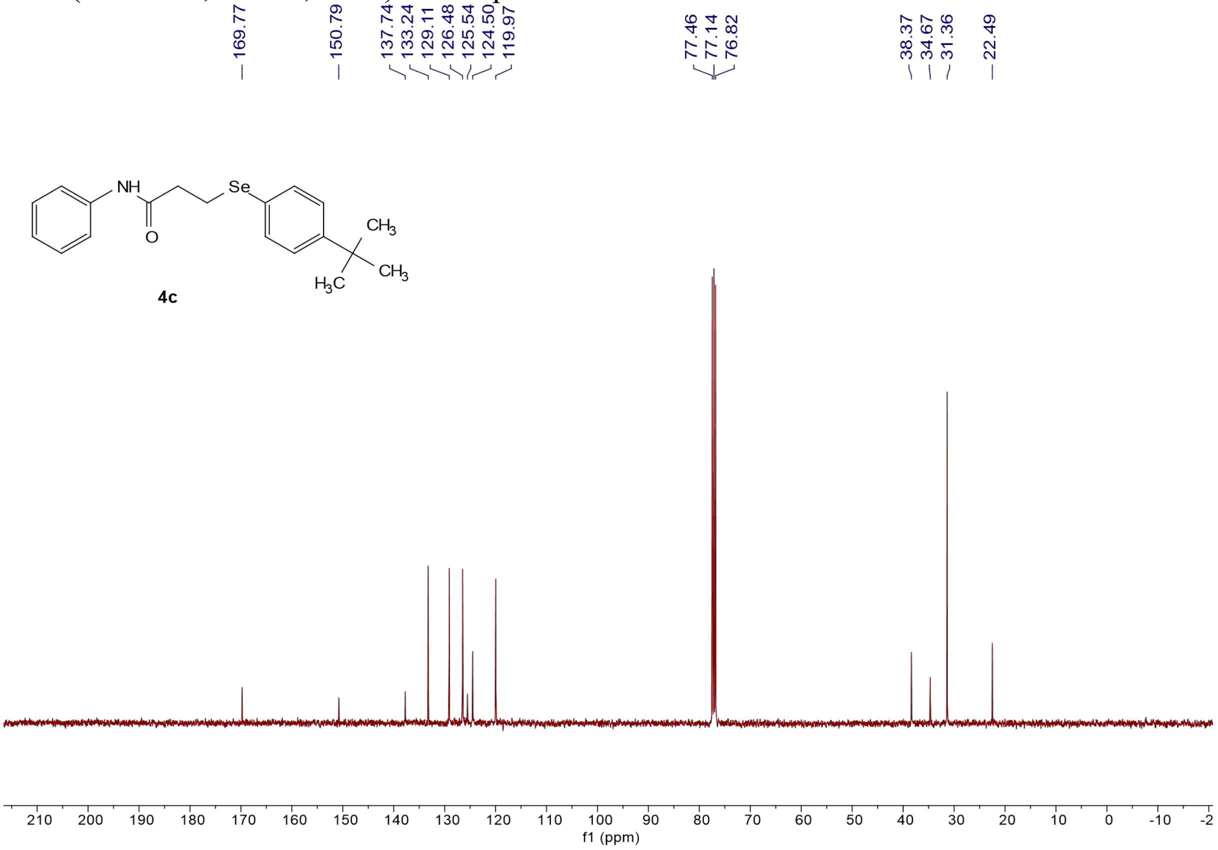

^{77}Se NMR (76 MHz, CDCl3, 25°C) of compound **4c**

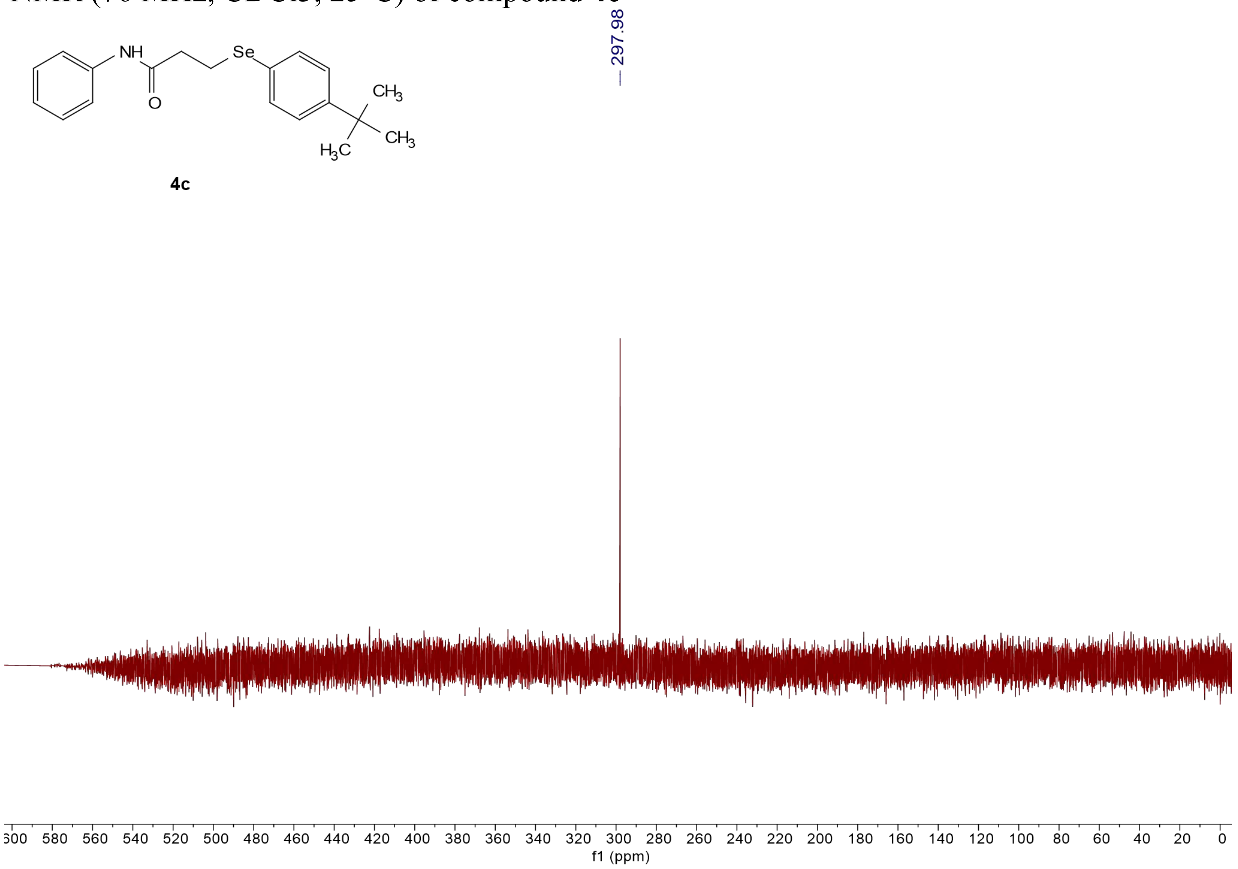

Supplementary Fig. 50. NMR spectra of compound **4c**

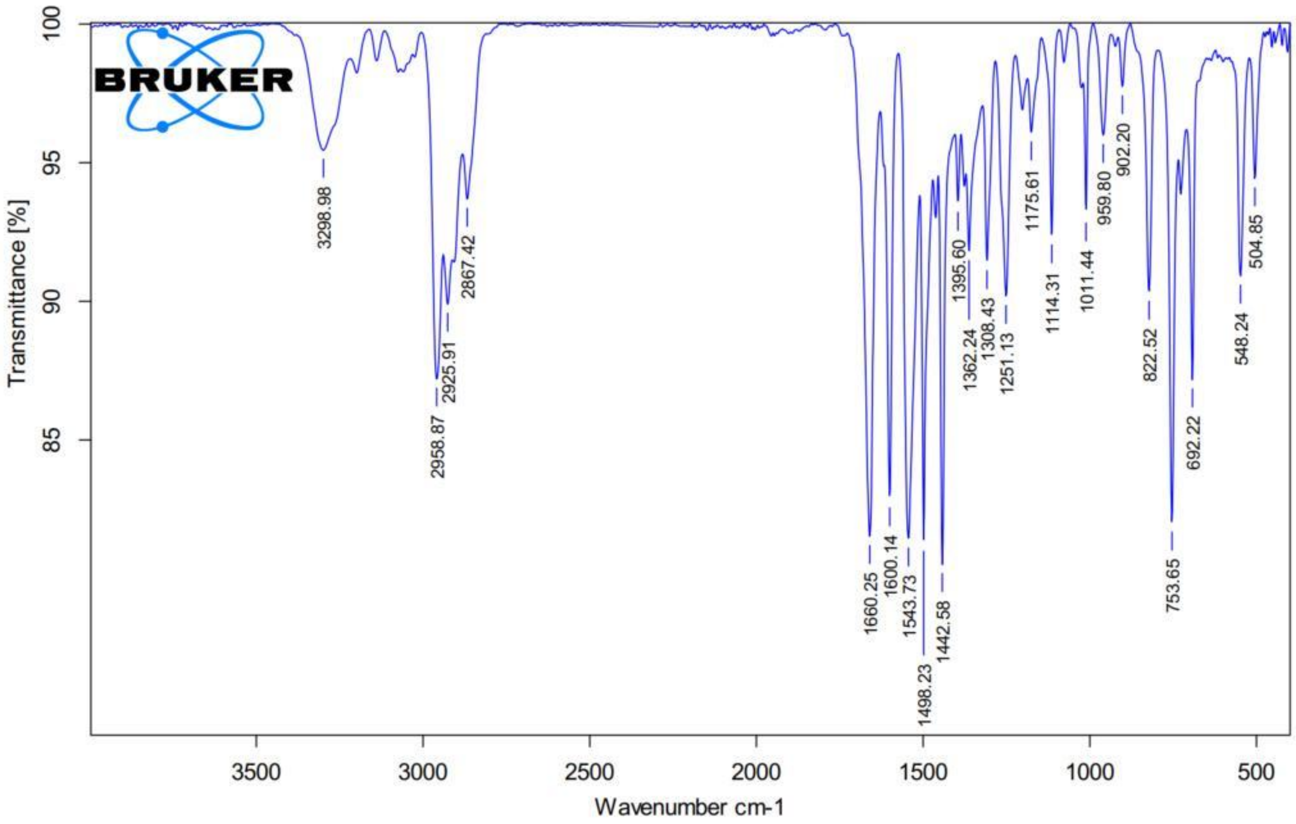

Supplementary Fig. 51. IR of compound **4c**

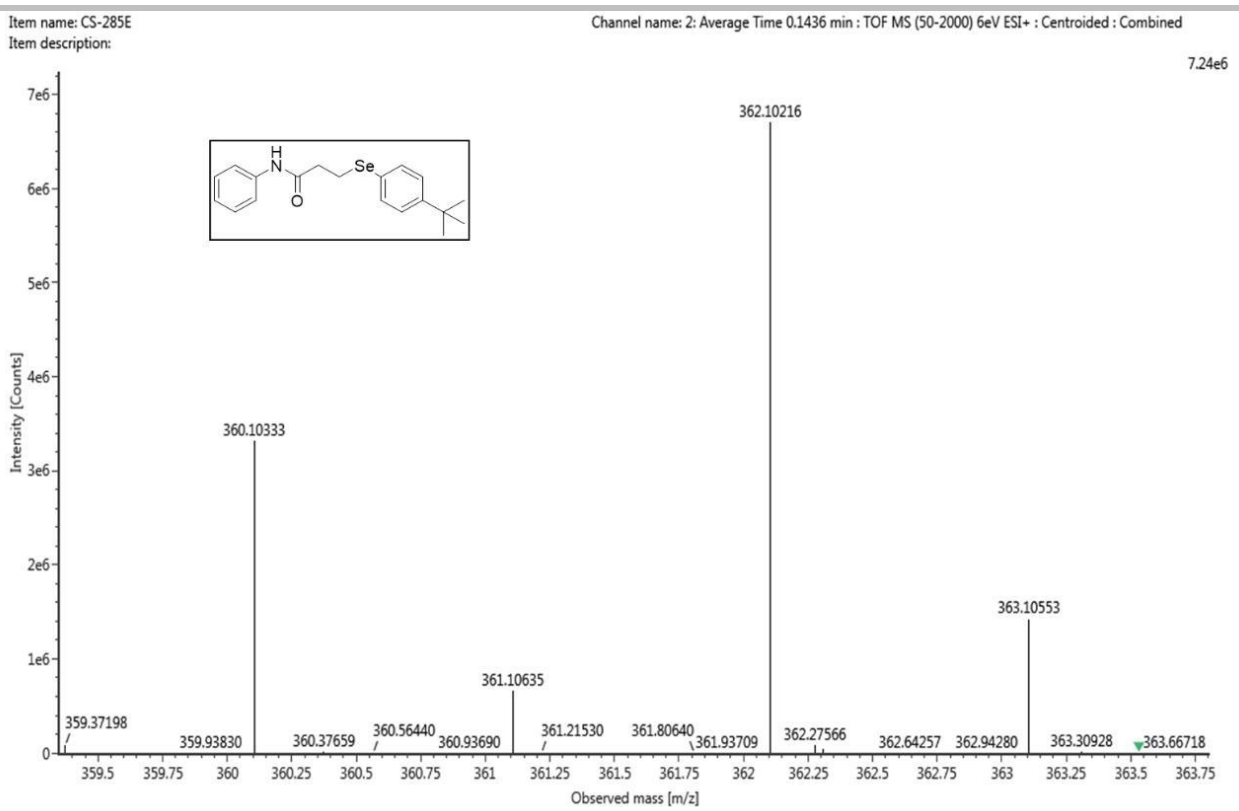

Supplementary Fig. 52. HR-MS of compound **4c**

^{1}H NMR (400 MHz, $CDCl_3$, 25°C) of compound **4d**

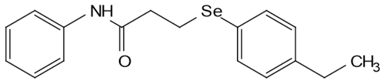

4d

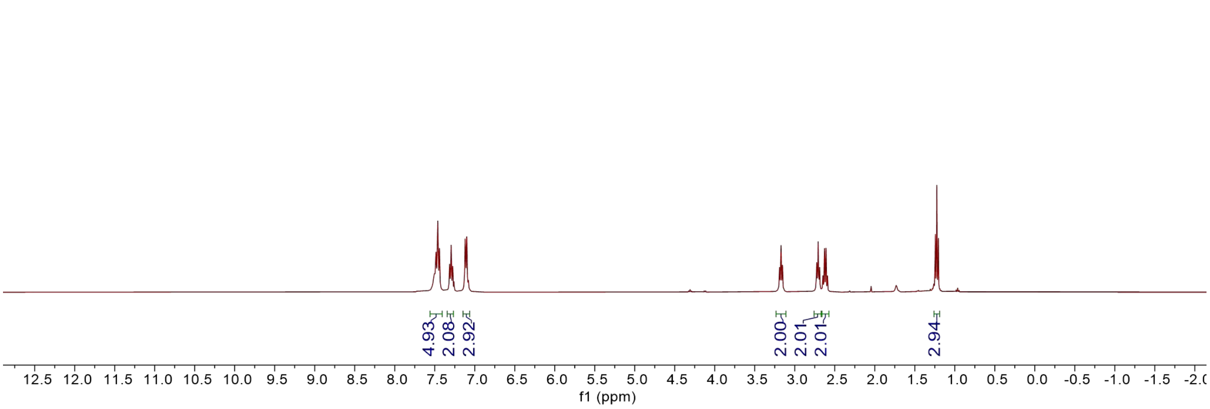

^{13}C NMR (101 MHz, $CDCl_3$, 25°C) of compound **4d**

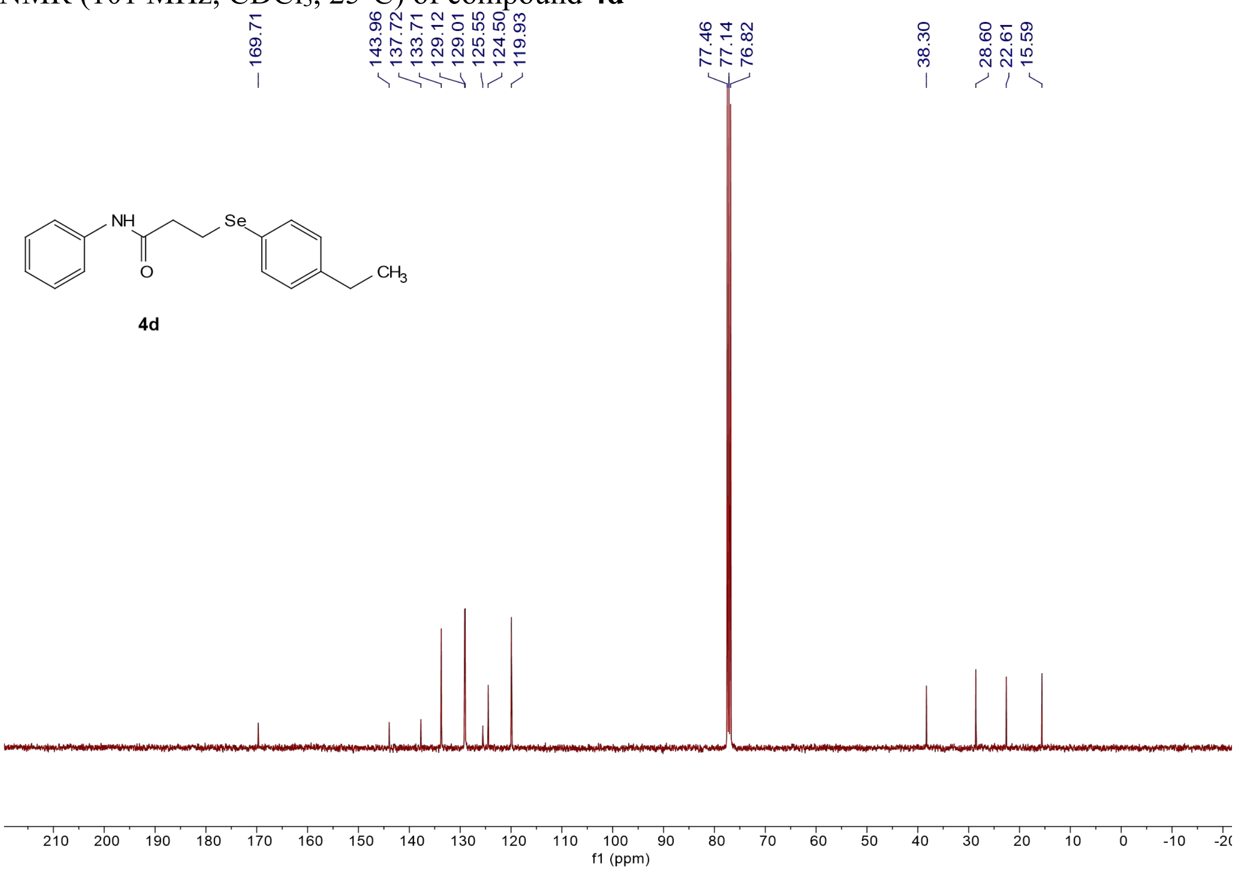

^{77}Se NMR (76 MHz, $CDCl_3$, 25°C) of compound **4d**

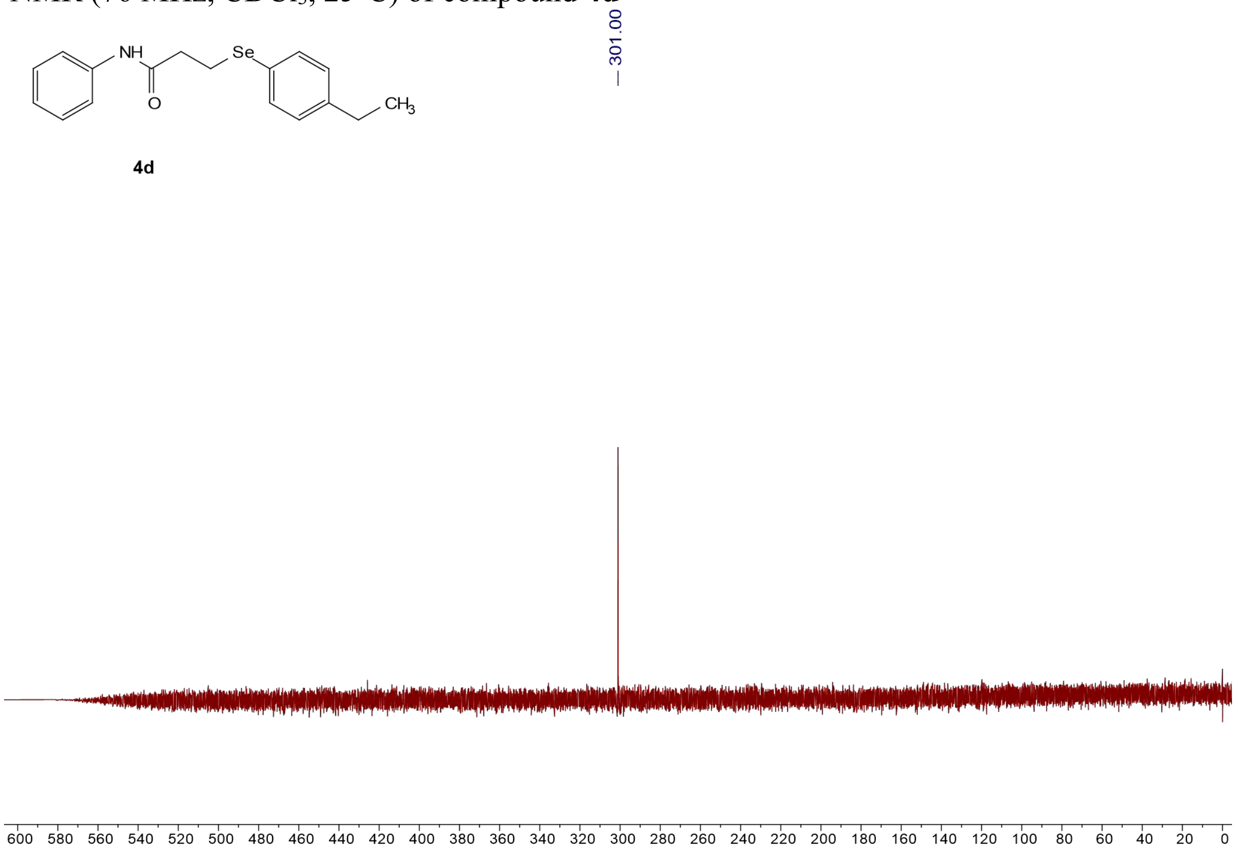

Supplementary Fig. 53. NMR spectra of compound **4d**

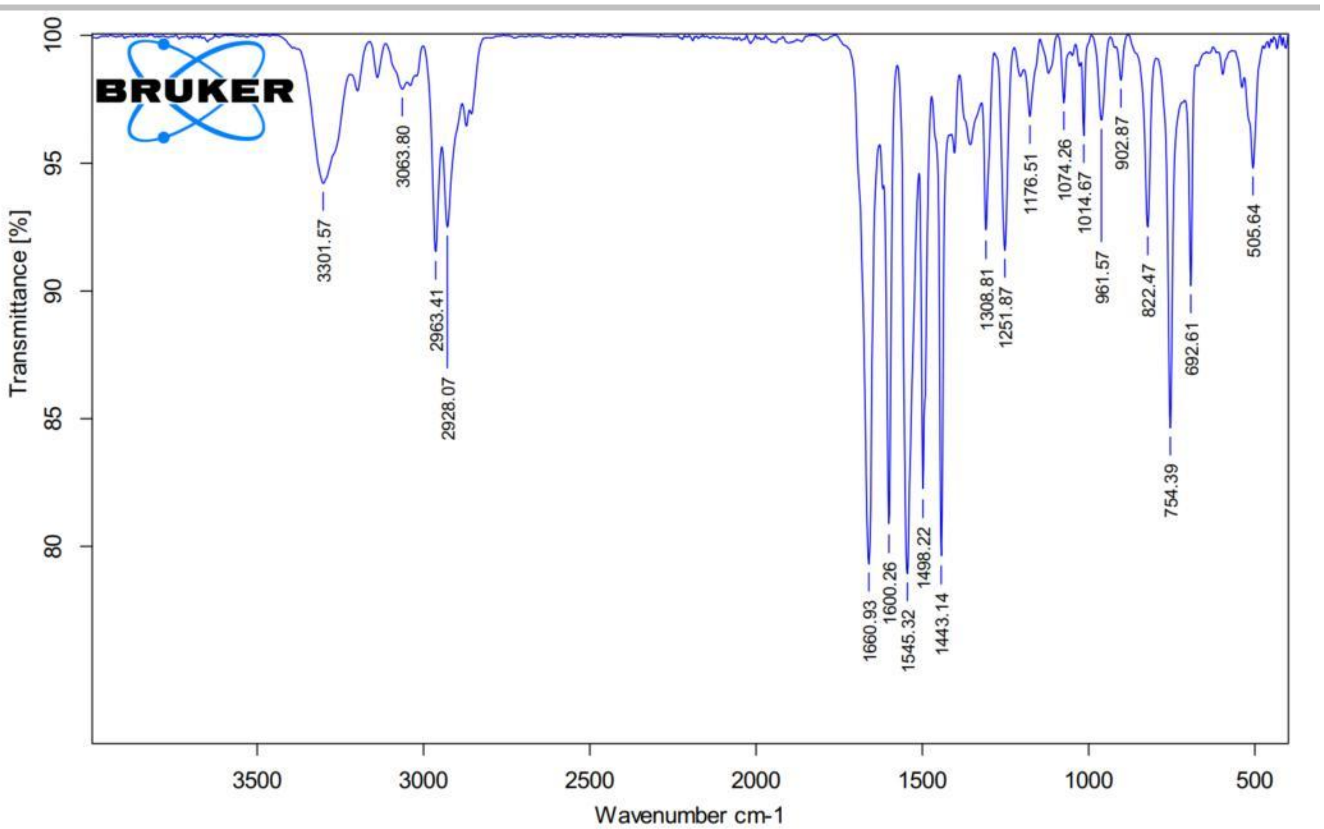

Supplementary Fig. 54. IR of compound **4d**

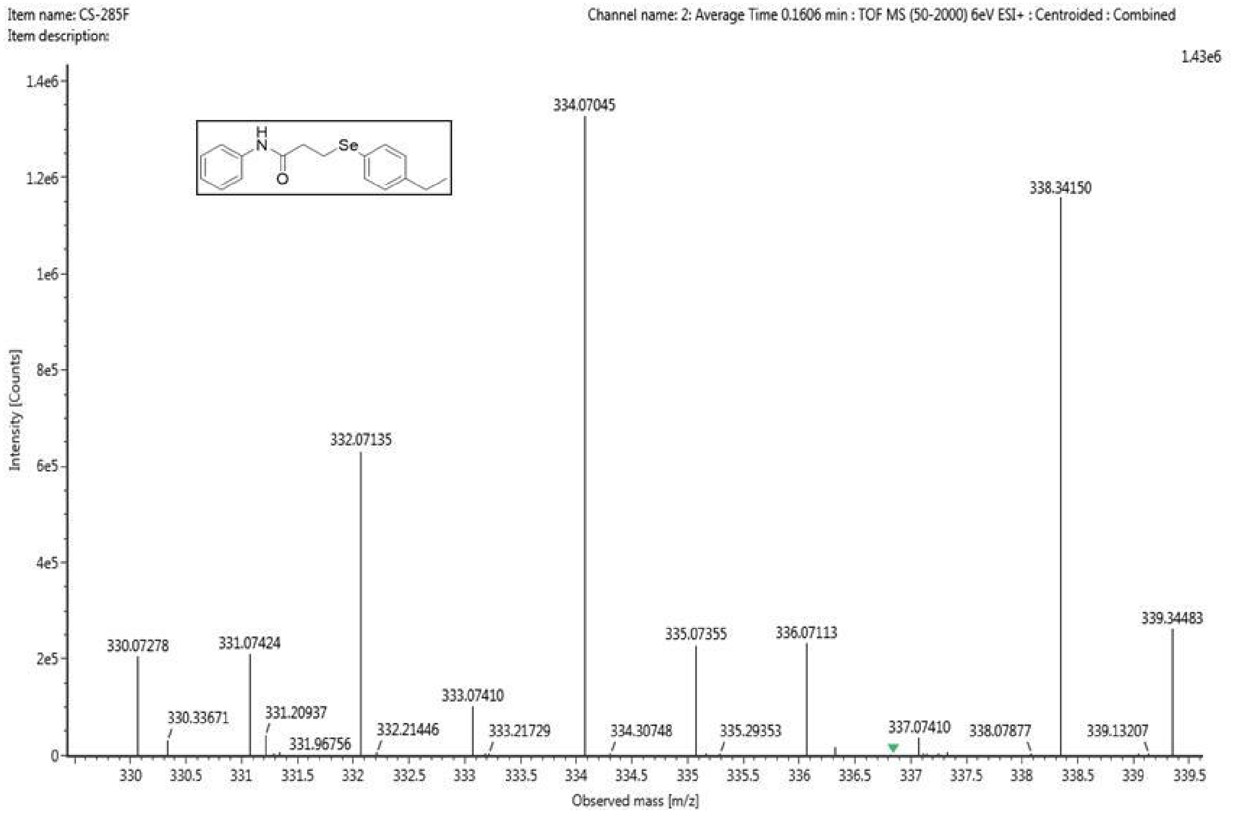

Supplementary Fig. 55. HR-MS of compound **4d**

^{1}H NMR (400 MHz, $CDCl_3$, 25°C) of compound **4e**

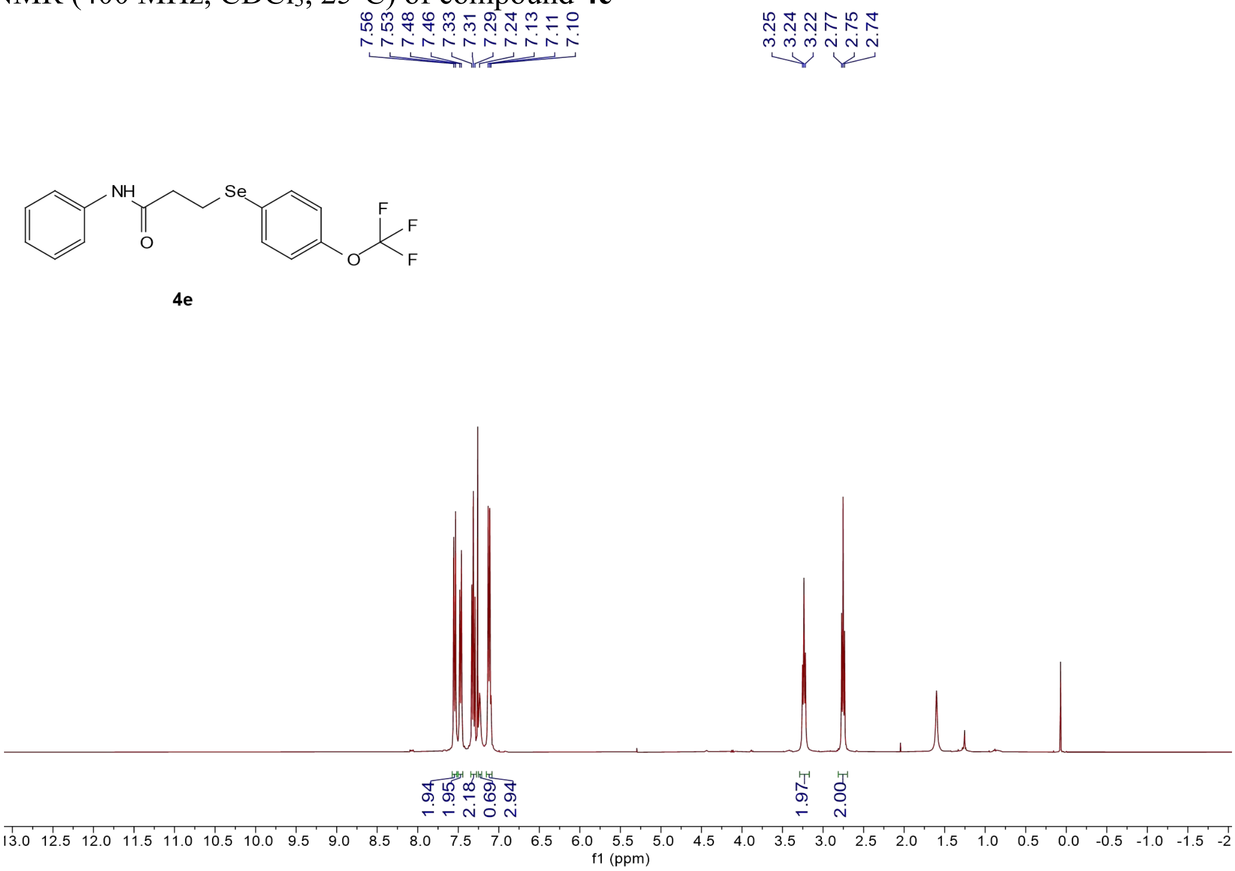

^{13}C NMR (101 MHz, $CDCl_3$, 25°C) of compound **4e**

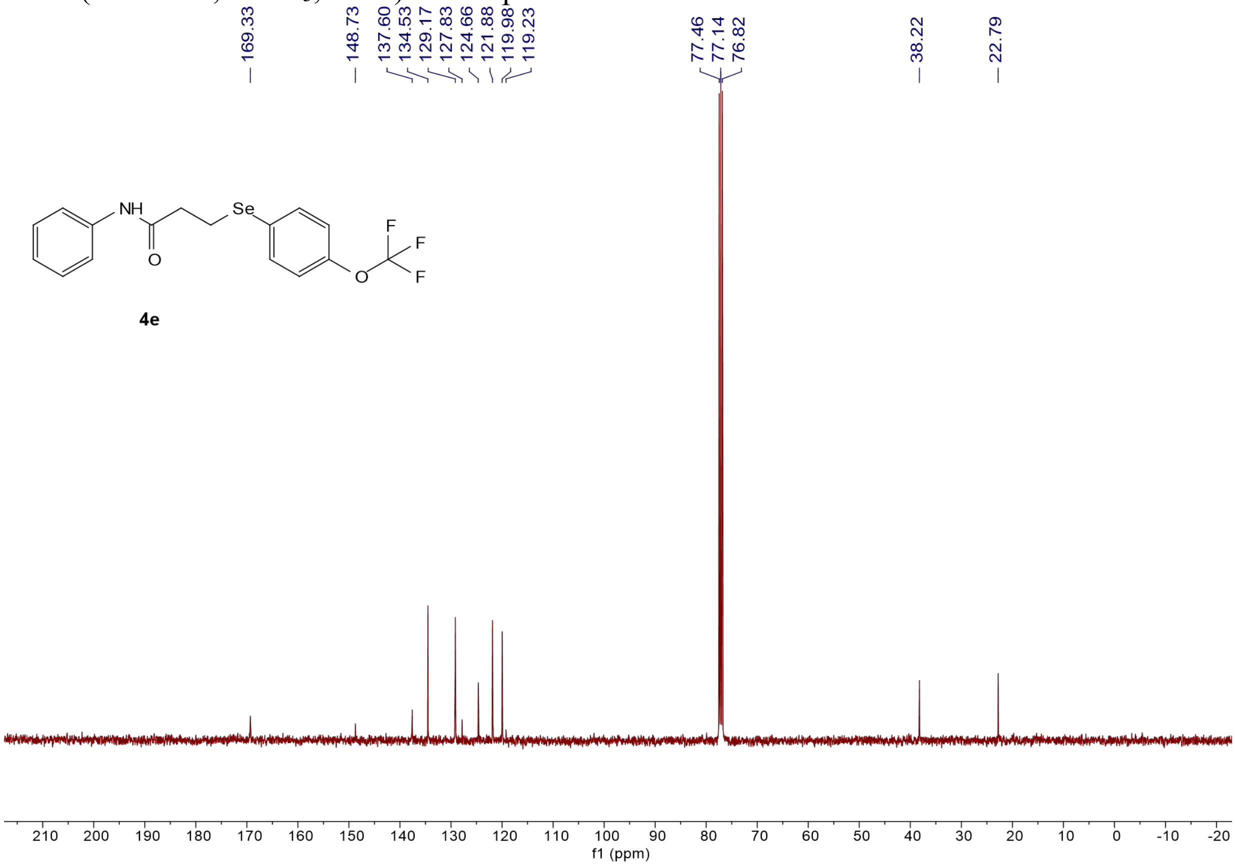

^{77}Se NMR (76 MHz, $CDCl_3$, 25°C) of compound **4e**

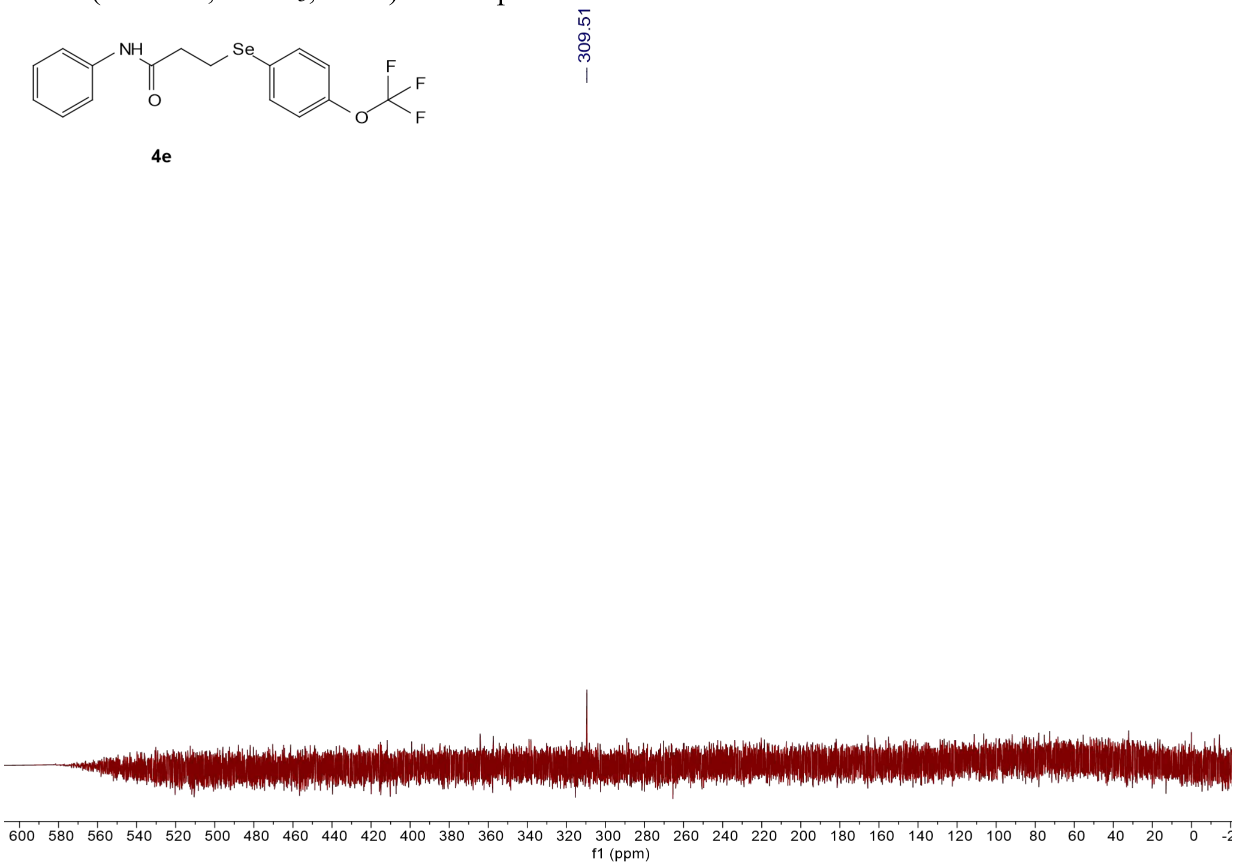

^{19}F NMR (376 MHz, $CDCl_3$, 25°C) of compound **4e**

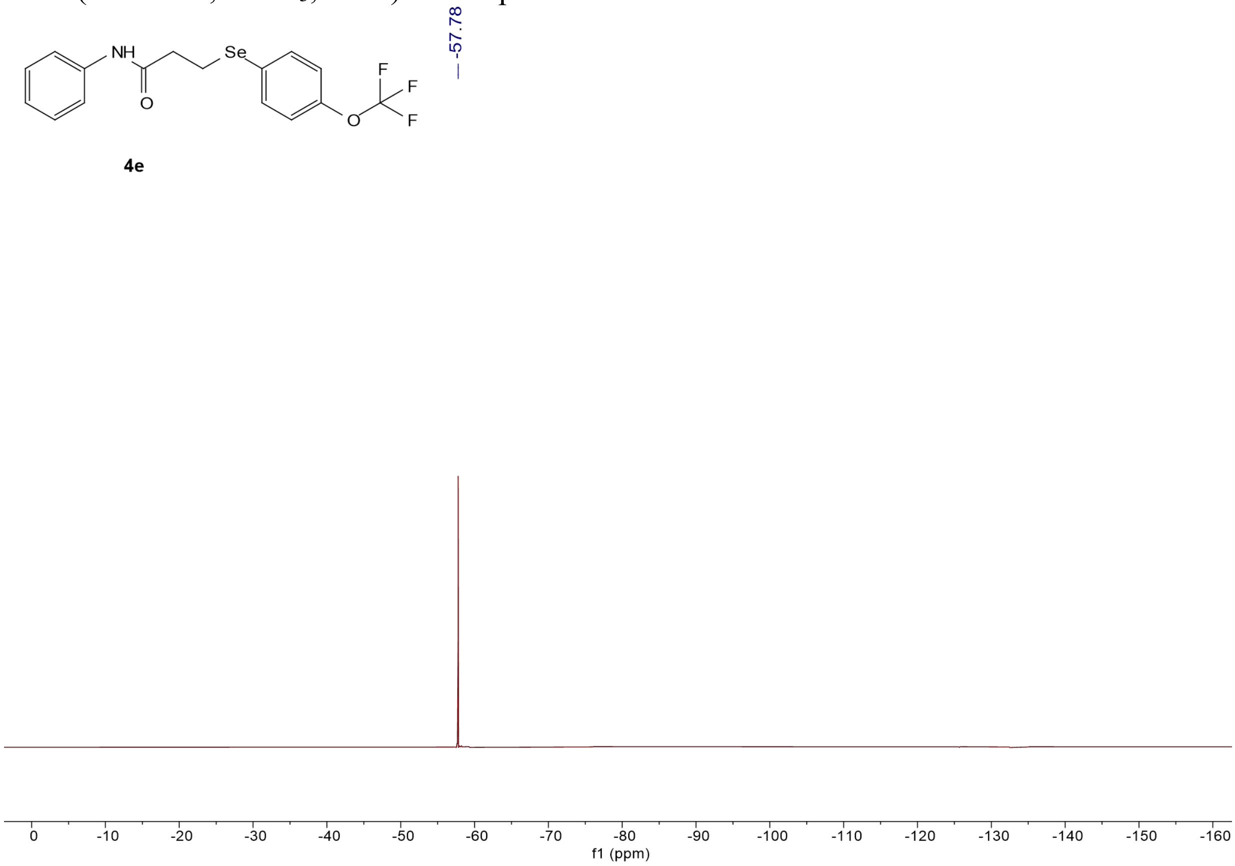

Supplementary Fig. 56. NMR spectra of compound **4e**

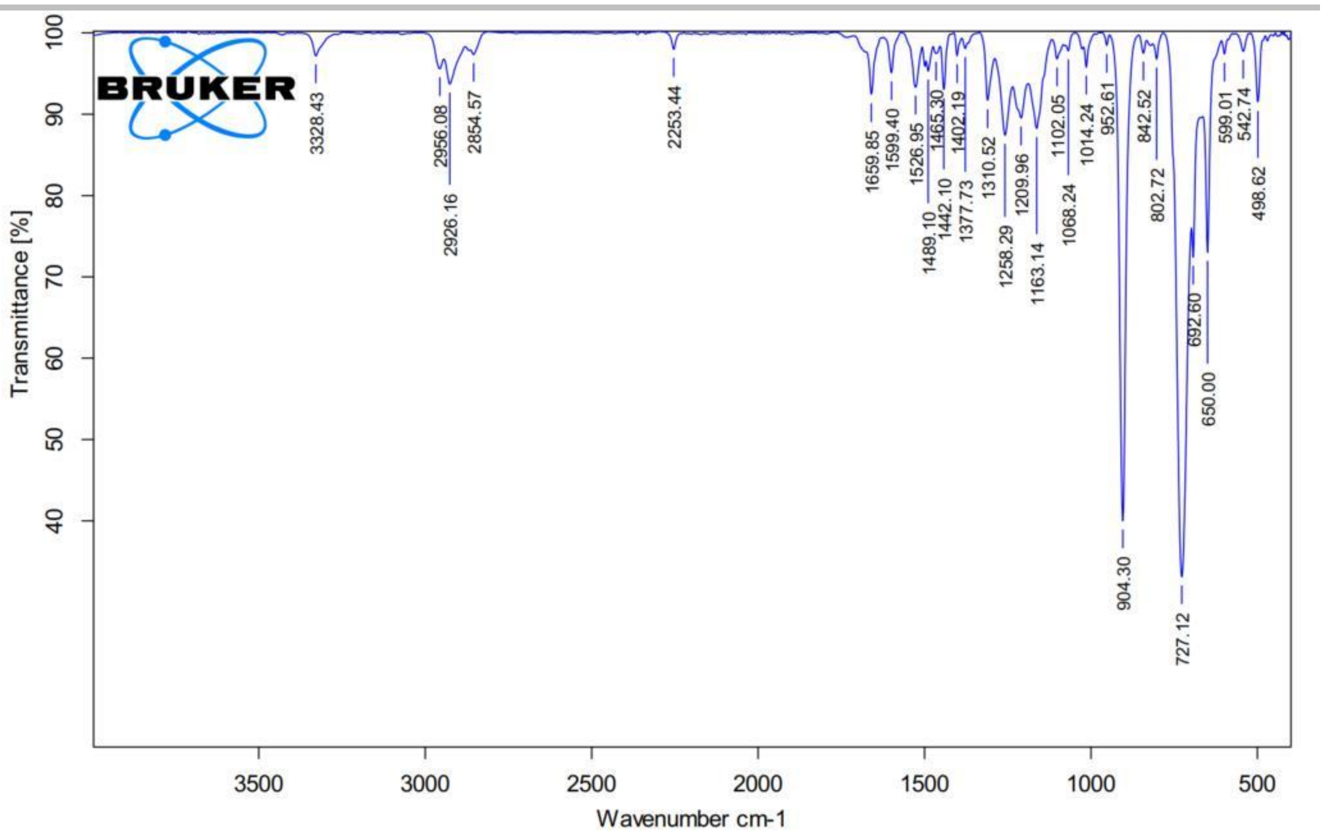

Supplementary Fig. 57. IR of compound **4e**

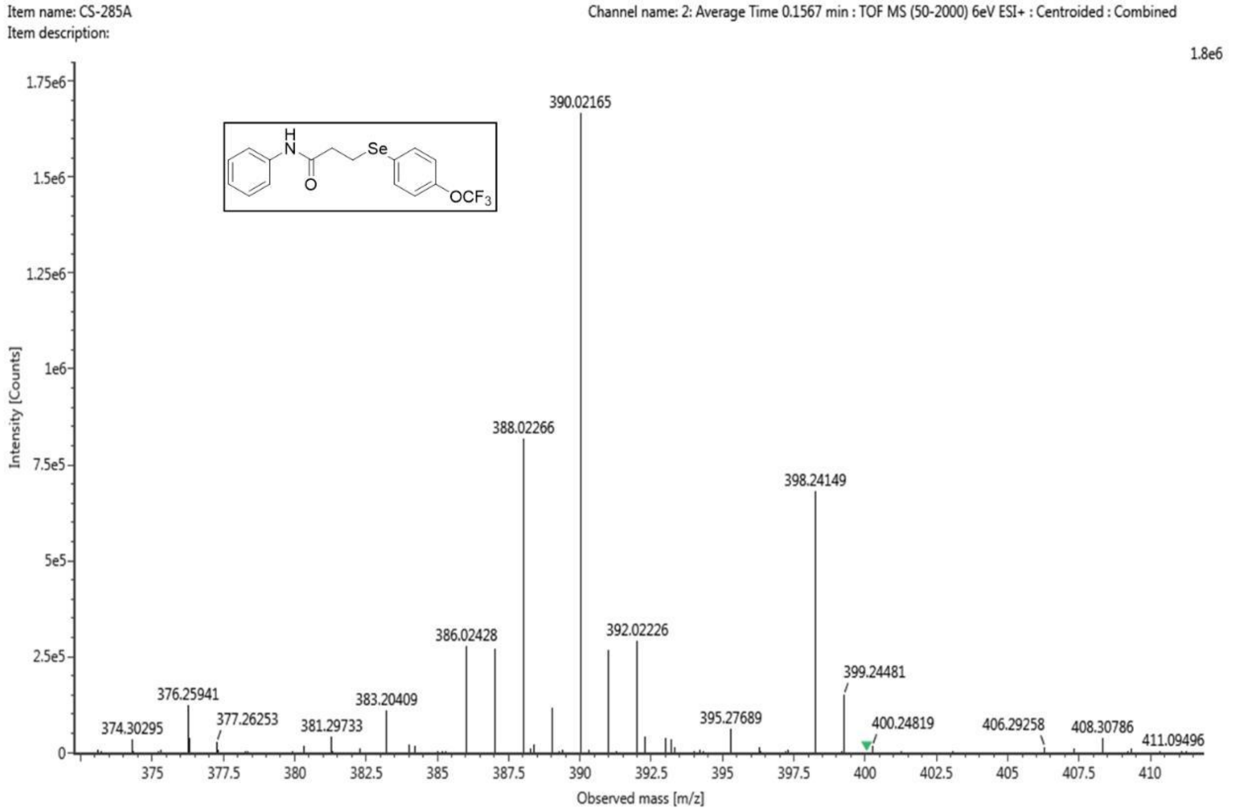

Supplementary Fig. 58. HR-MS of compound **4e**

^{1}H NMR (400 MHz, $CDCl_3$, 25°C) of compound **4f**

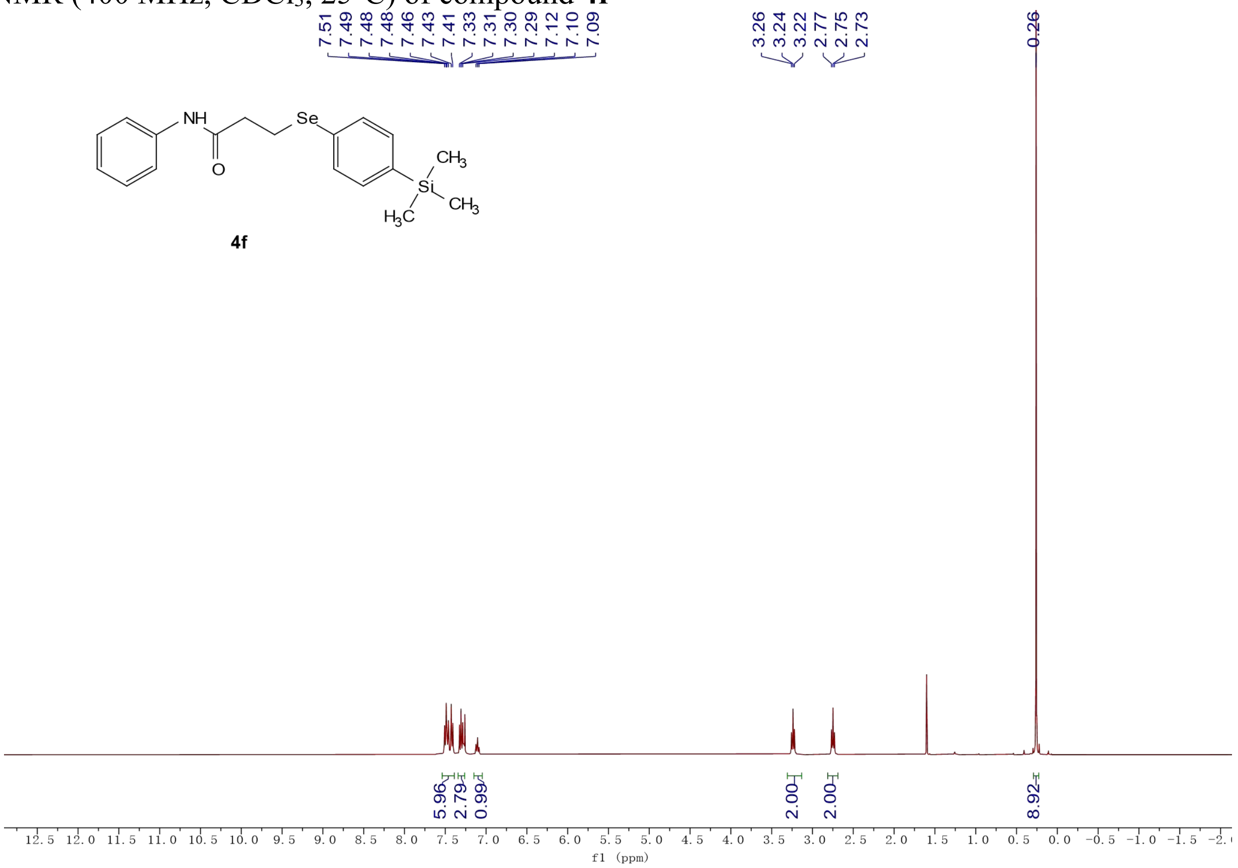

^{13}C NMR (101 MHz, $CDCl_3$, 25°C) of compound **4f**

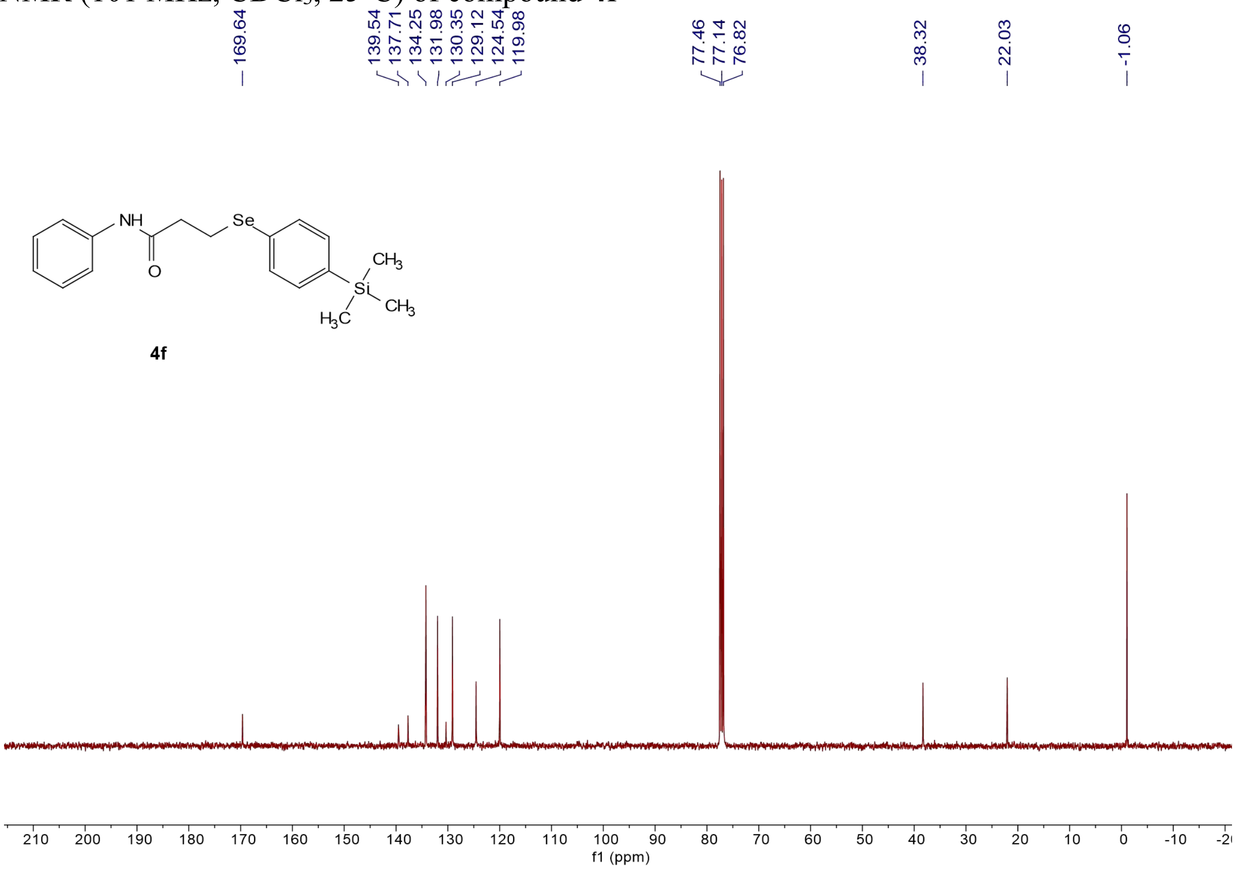

^{77}Se NMR (76 MHz, $CDCl_3$, 25°C) of compound **4f**

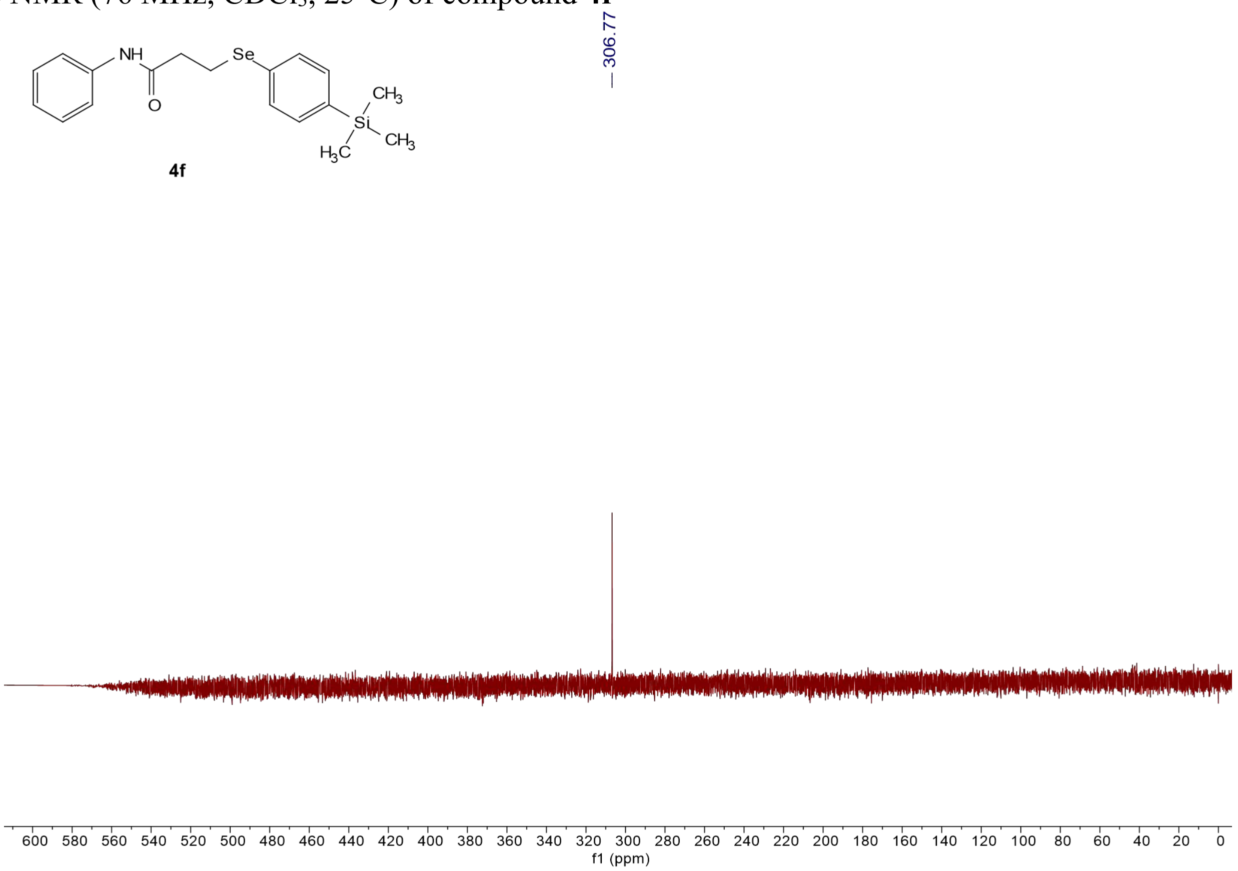

Supplementary Fig. 59. NMR spectra of compound **4f**

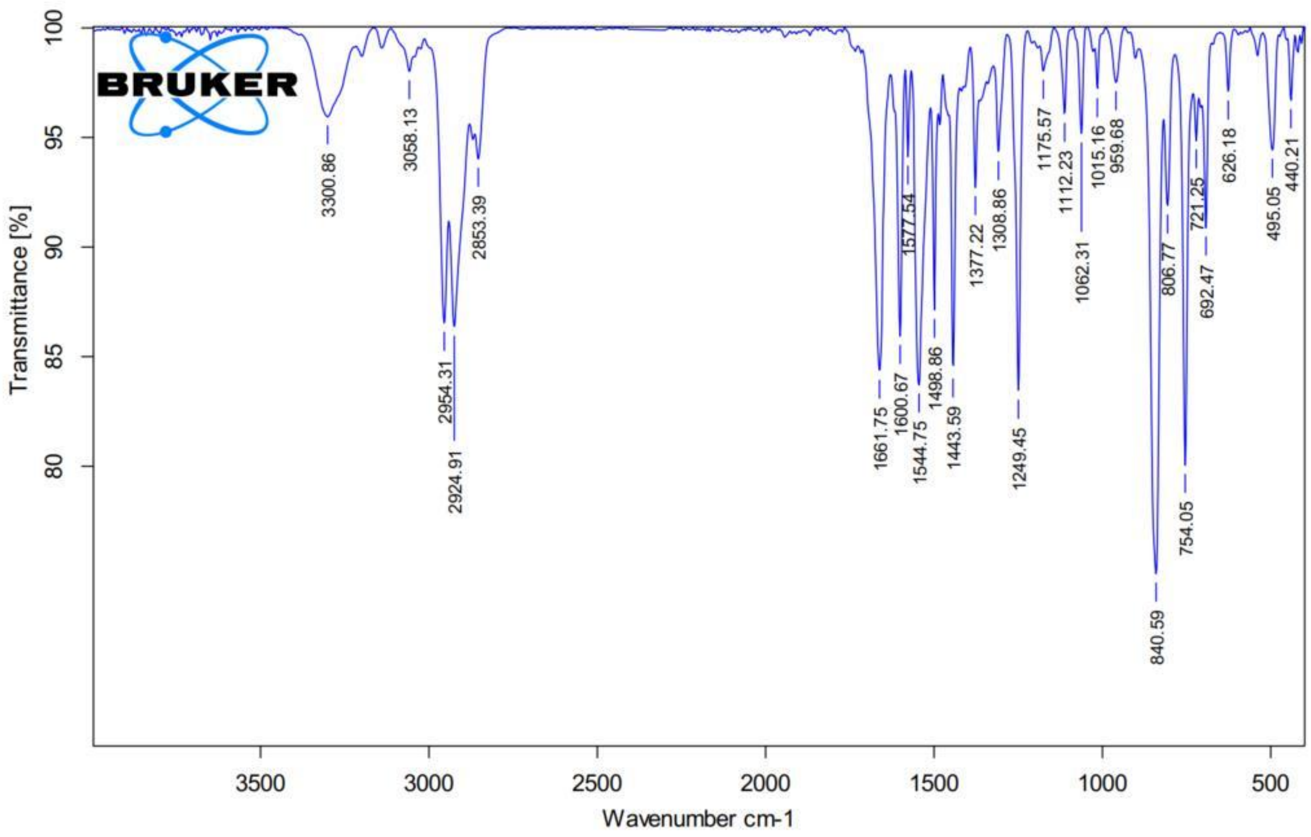

Supplementary Fig. 60. IR of compound **4f**

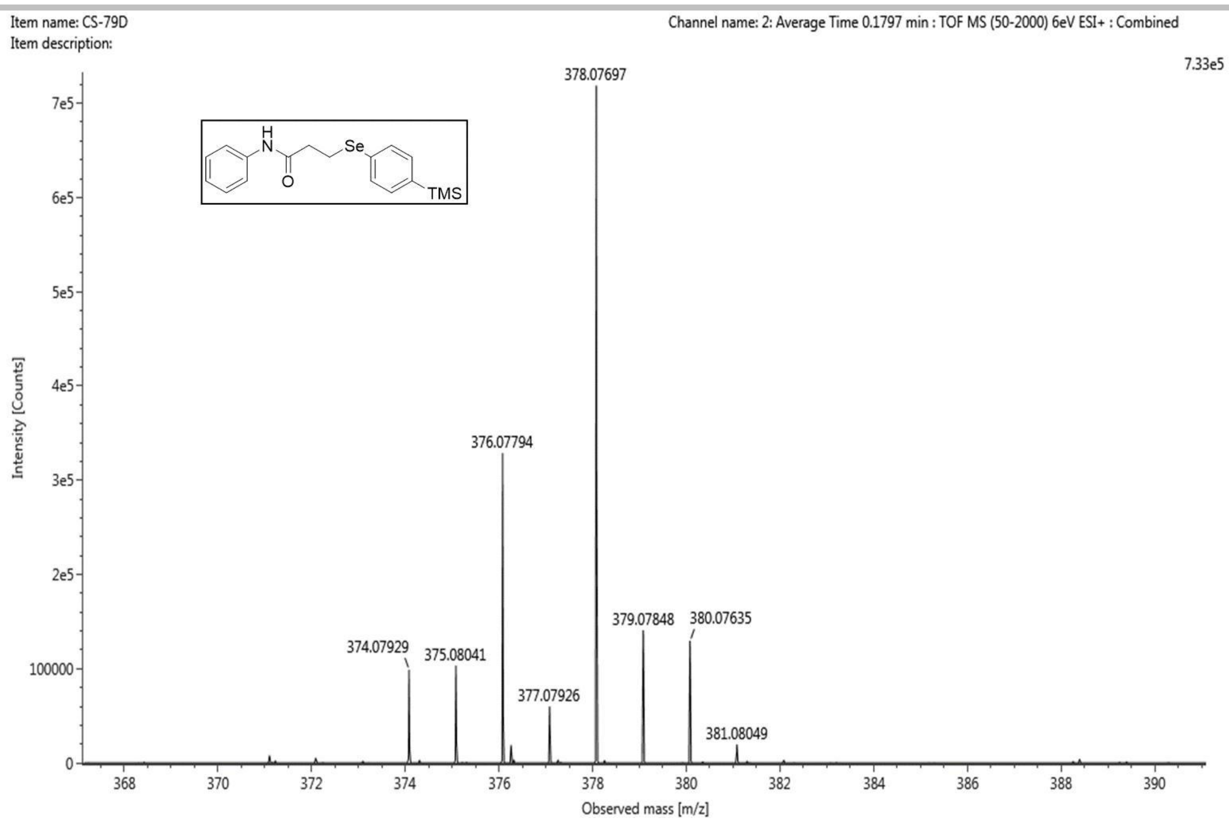

Supplementary Fig. 61. HR-MS of compound **4f**

^{1}H NMR (400 MHz, $CDCl_3$, 25°C) of compound **4g**

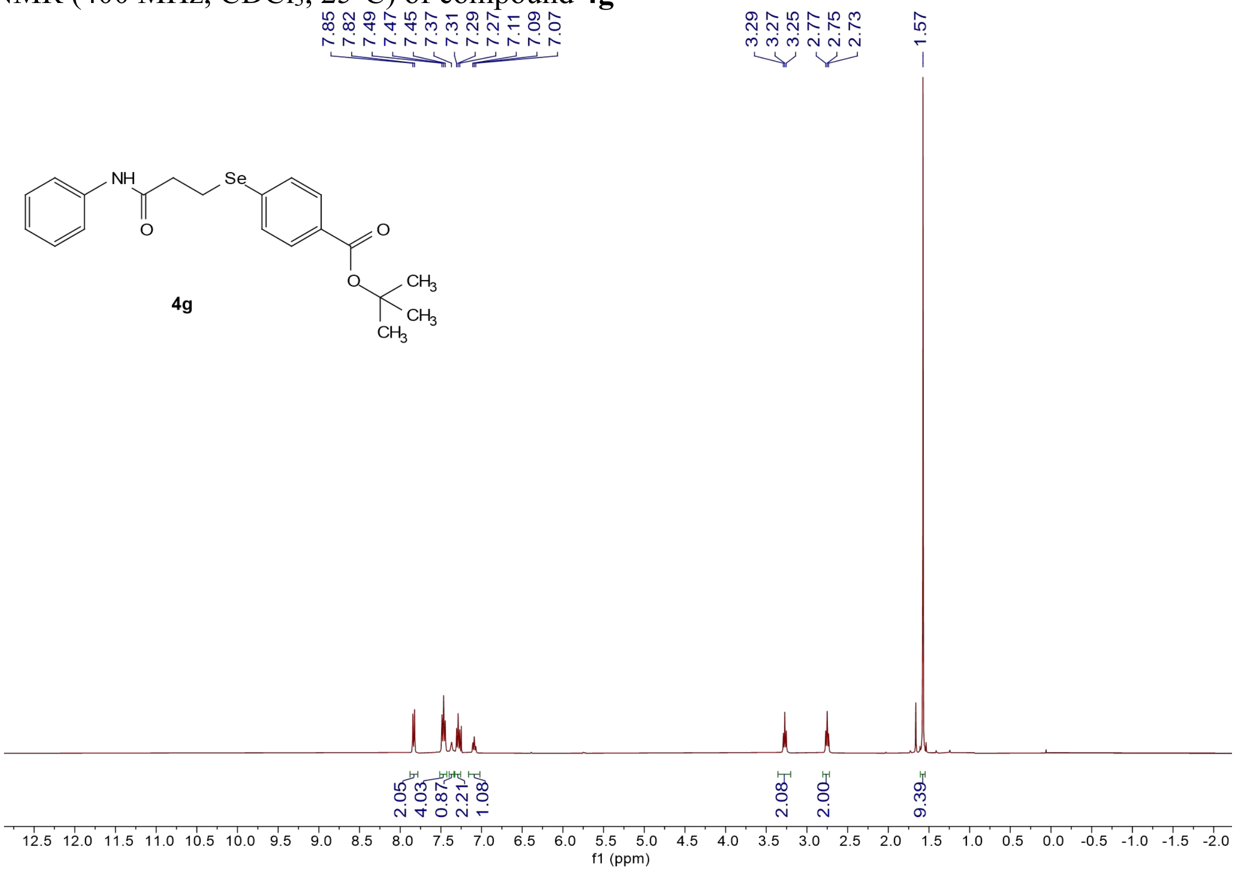

^{13}C NMR (101 MHz, $CDCl_3$, 25°C) of compound **4g**

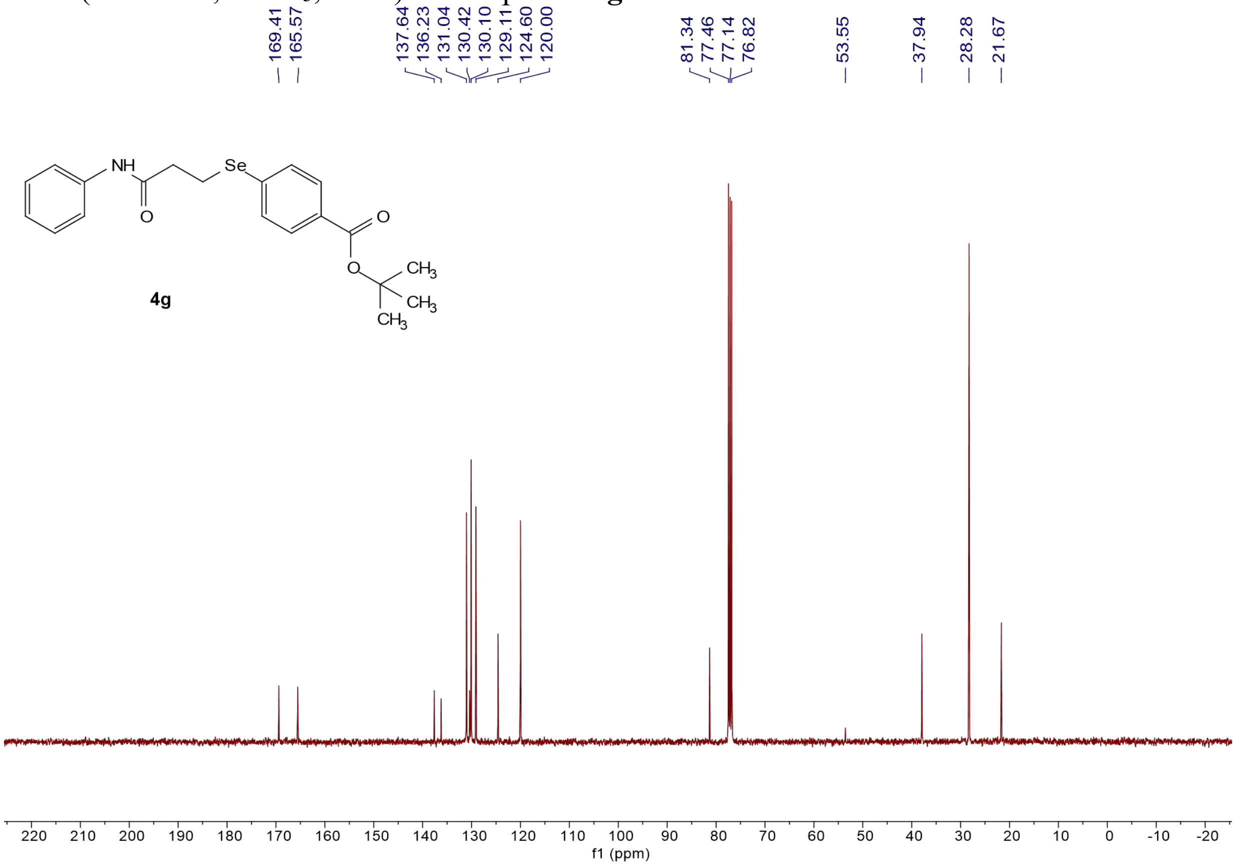

^{77}Se NMR (76 MHz, $CDCl_3$, 25°C) of compound **4g**

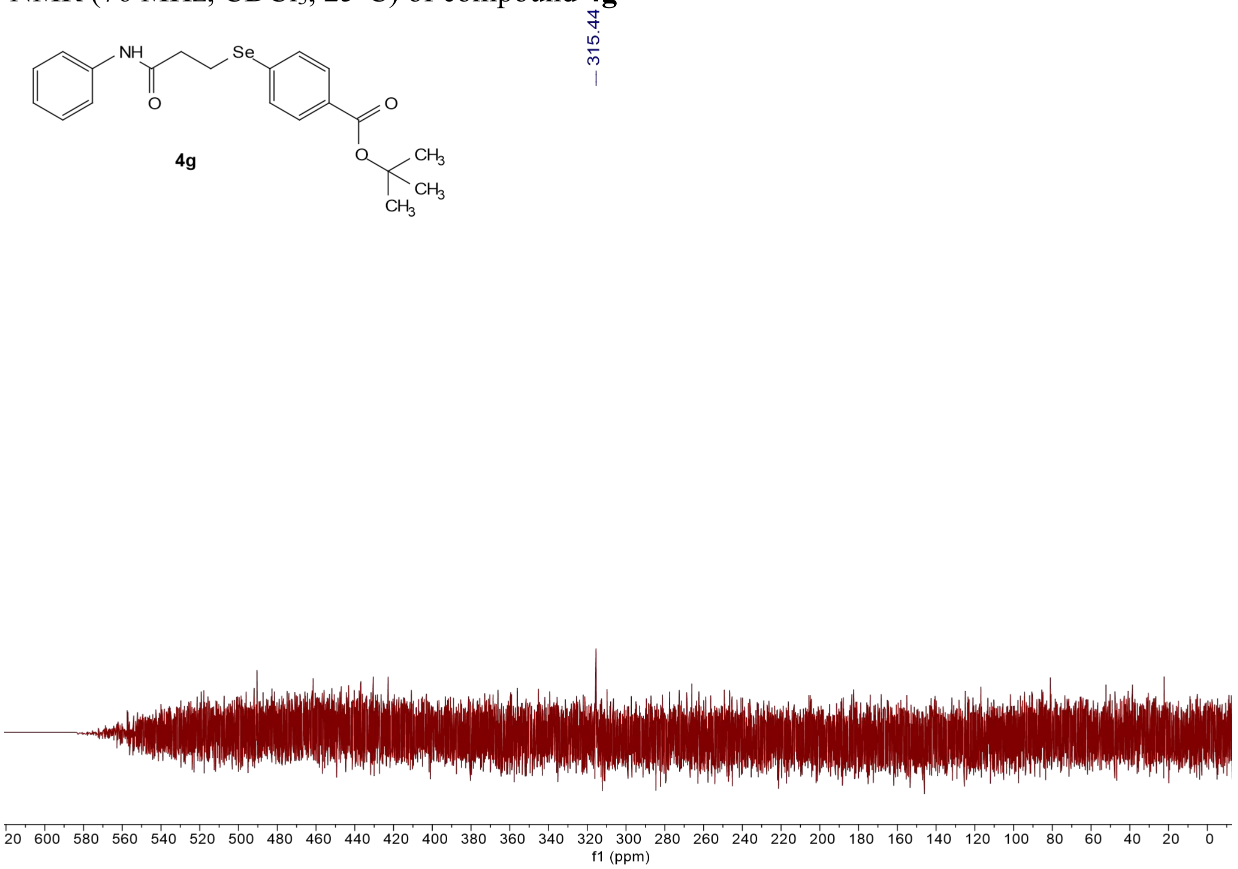

Supplementary Fig. 62. NMR spectra of compound **4g**

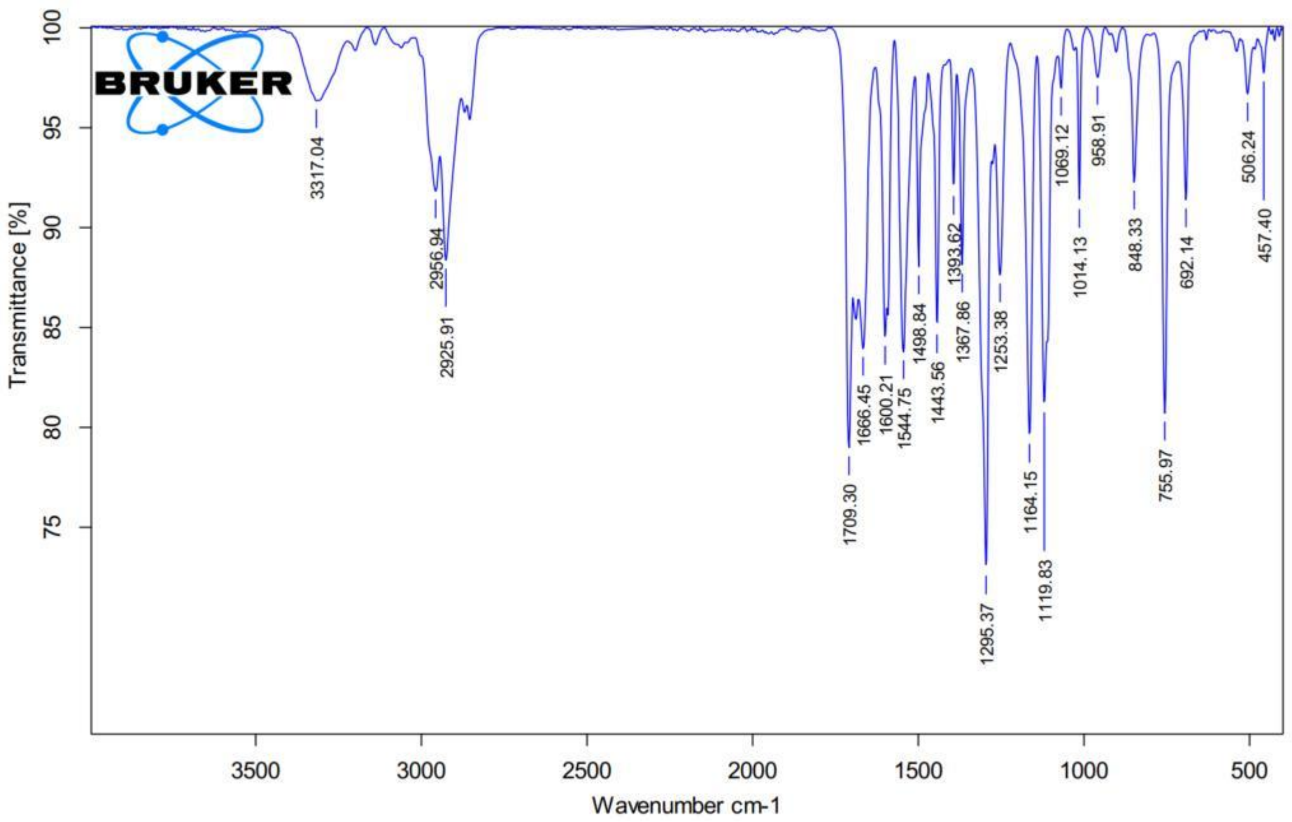

Supplementary Fig. 63. IR of compound **4g**

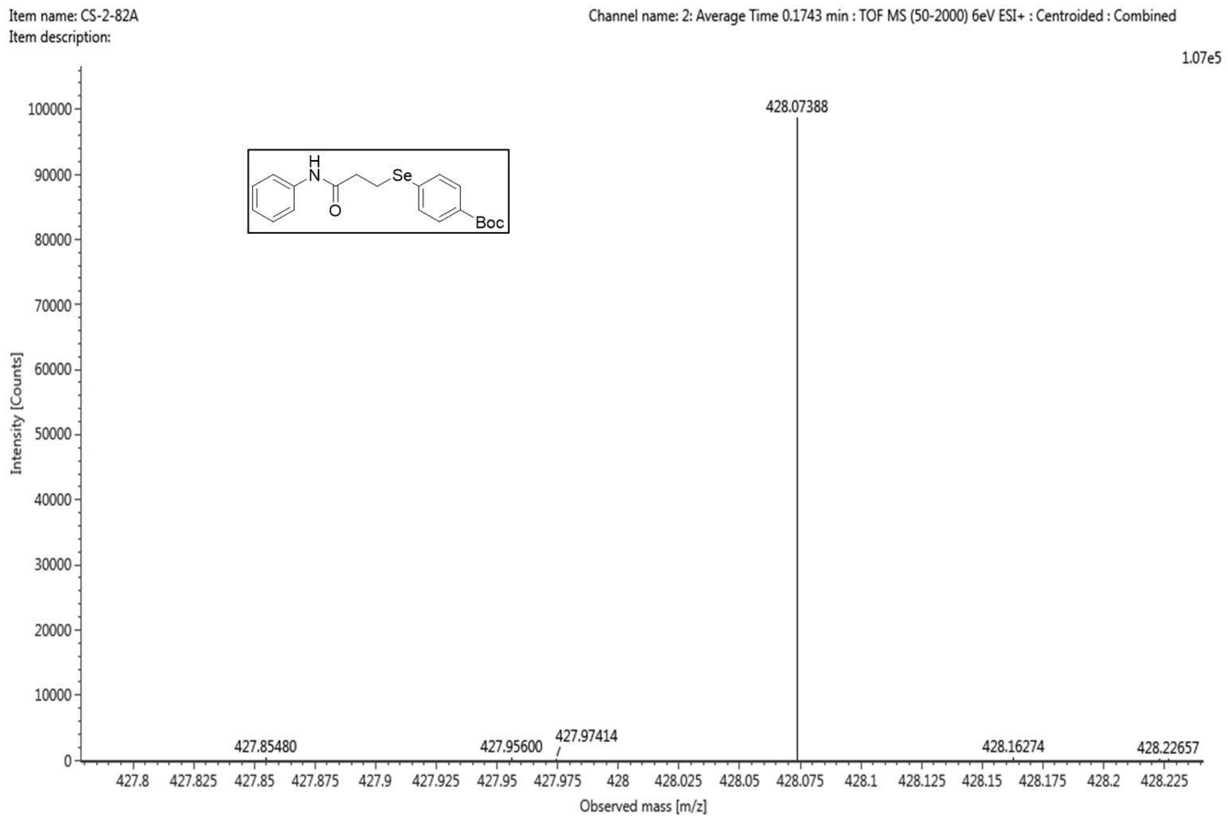

Supplementary Fig. 64. HR-MS of compound **4g**

^{1}H NMR (400 MHz, $CDCl_3$, 25°C) of compound **4h**

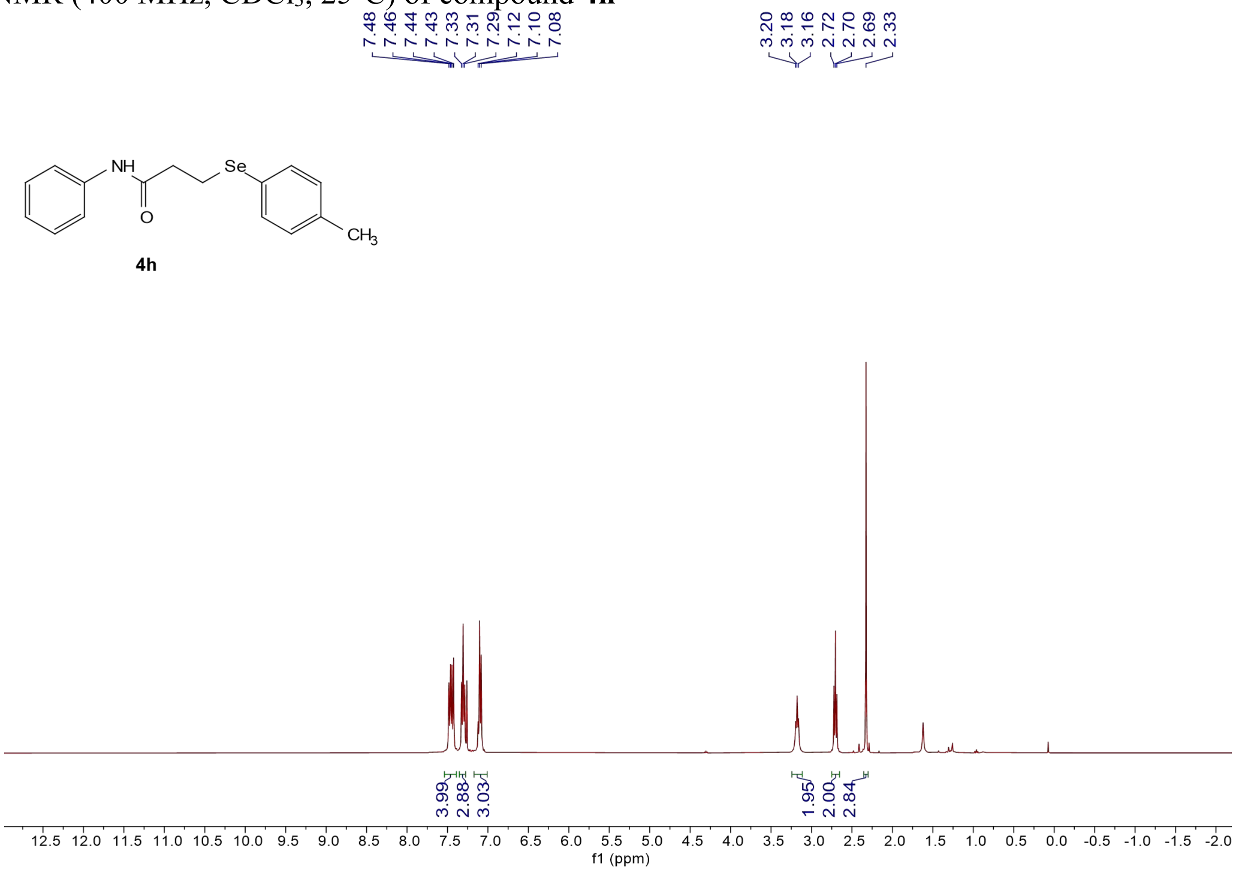

^{13}C NMR (101 MHz, $CDCl_3$, 25°C) of compound **4h**

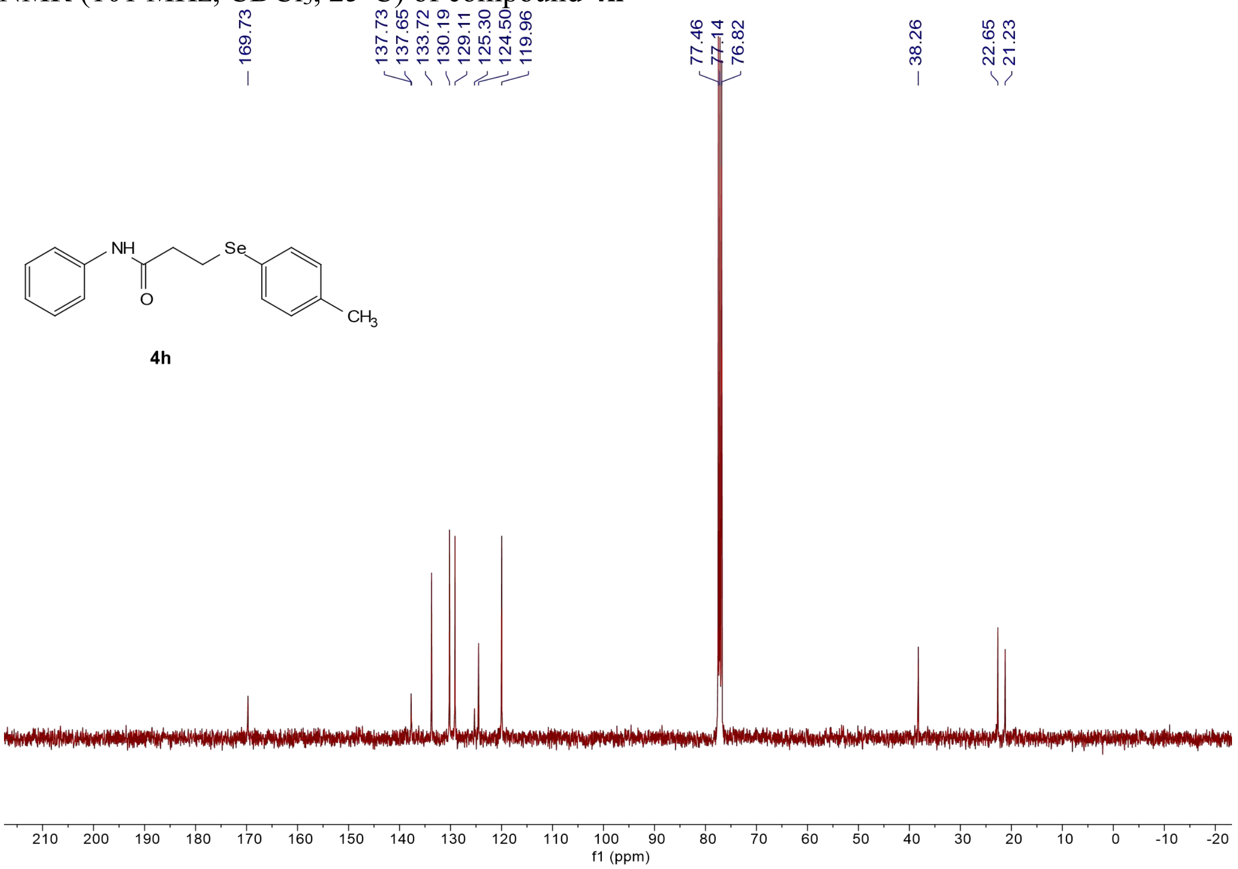

^{77}Se NMR (76 MHz, $CDCl_3$, 25°C) of compound **4h**

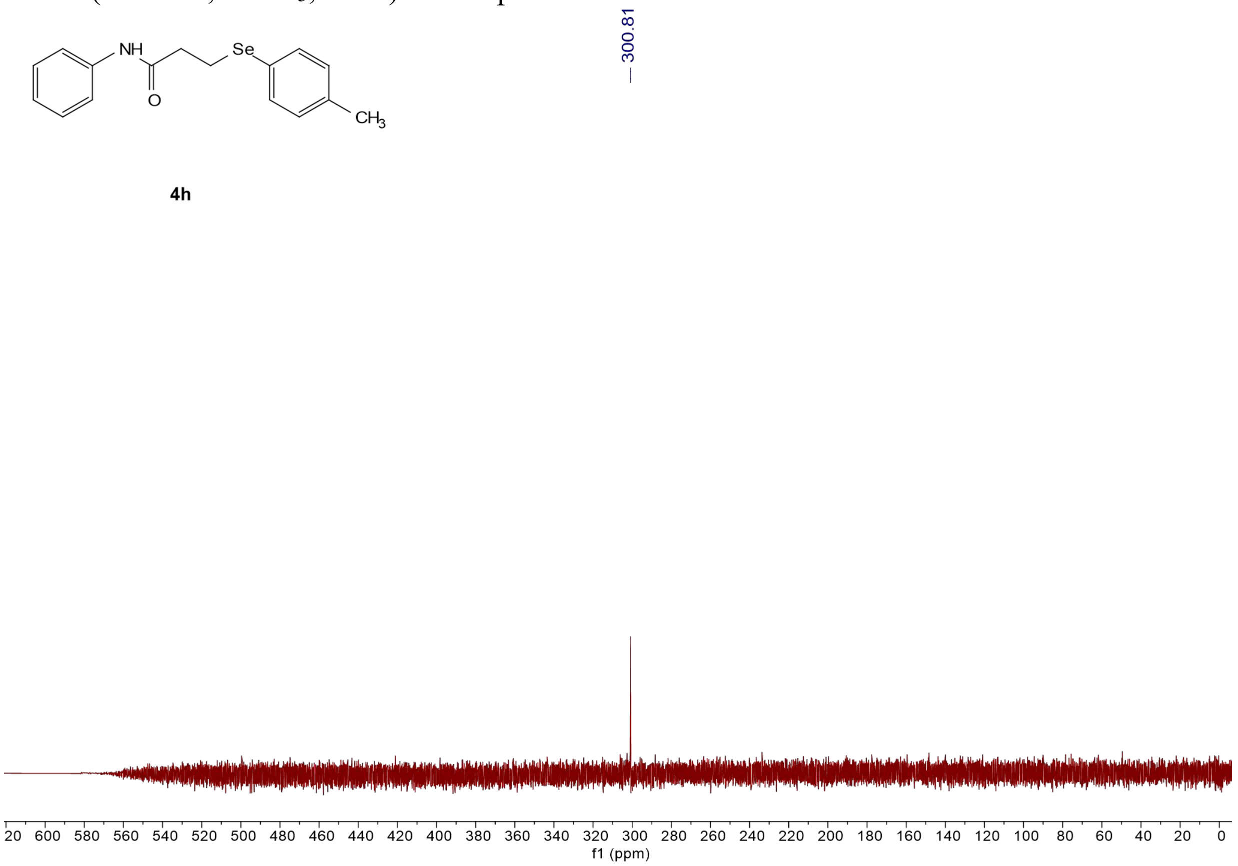

Supplementary Fig. 65. NMR spectra of compound **4h**

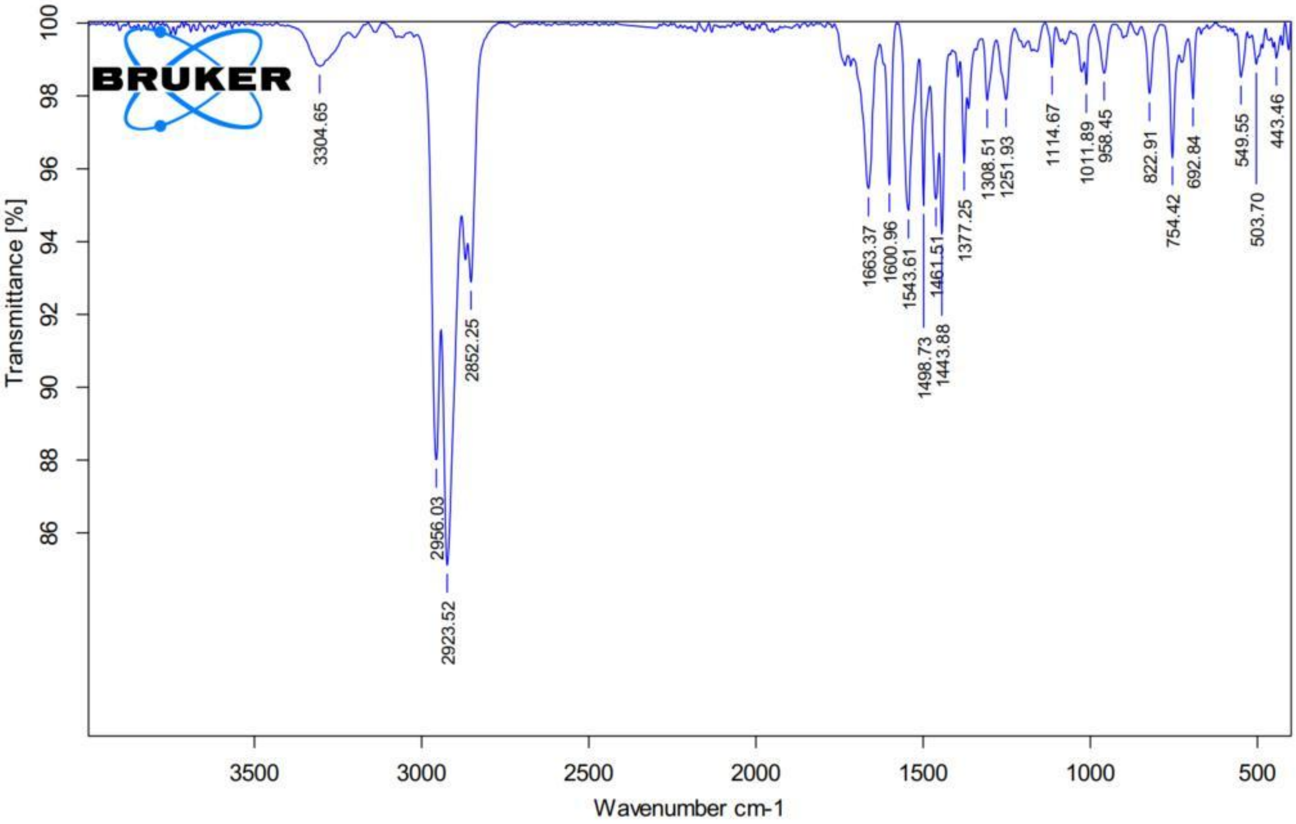

Supplementary Fig. 66. IR of compound **4h**

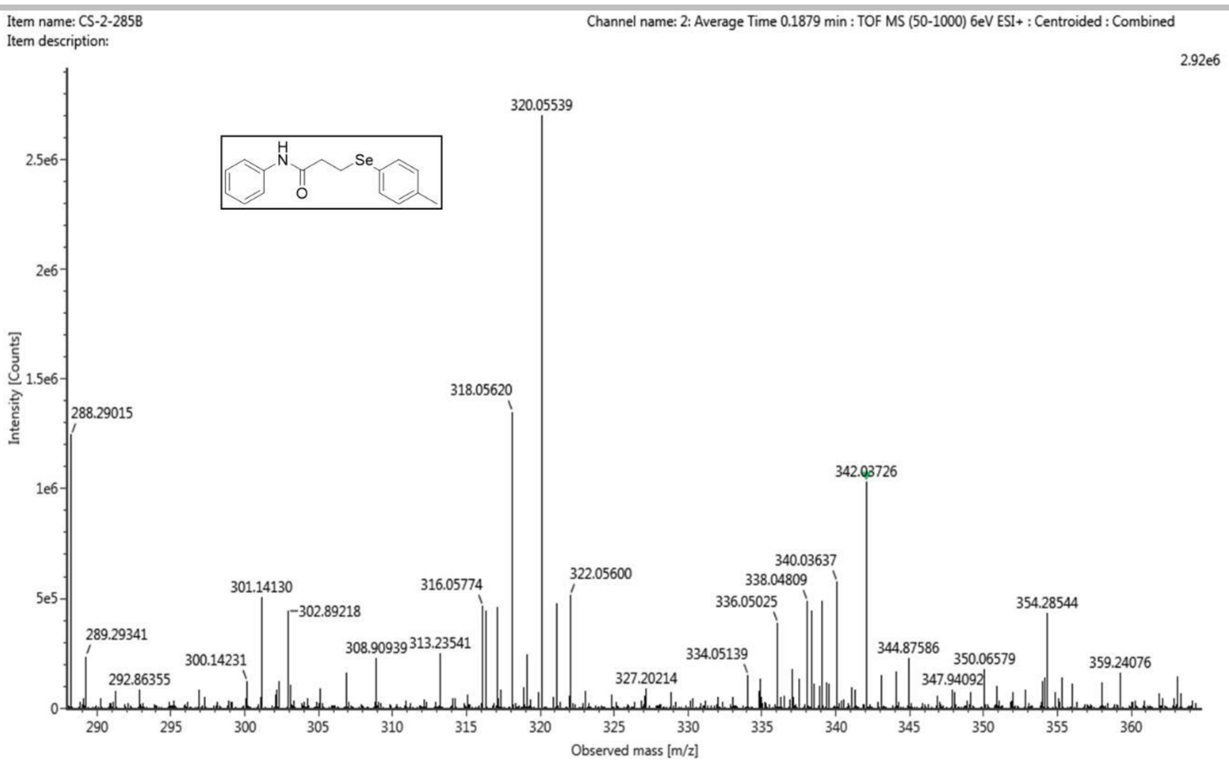

Supplementary Fig. 67. HR-MS of compound **4h**

^{1}H NMR (400 MHz, $CDCl_3$, 25°C) of compound **4i**

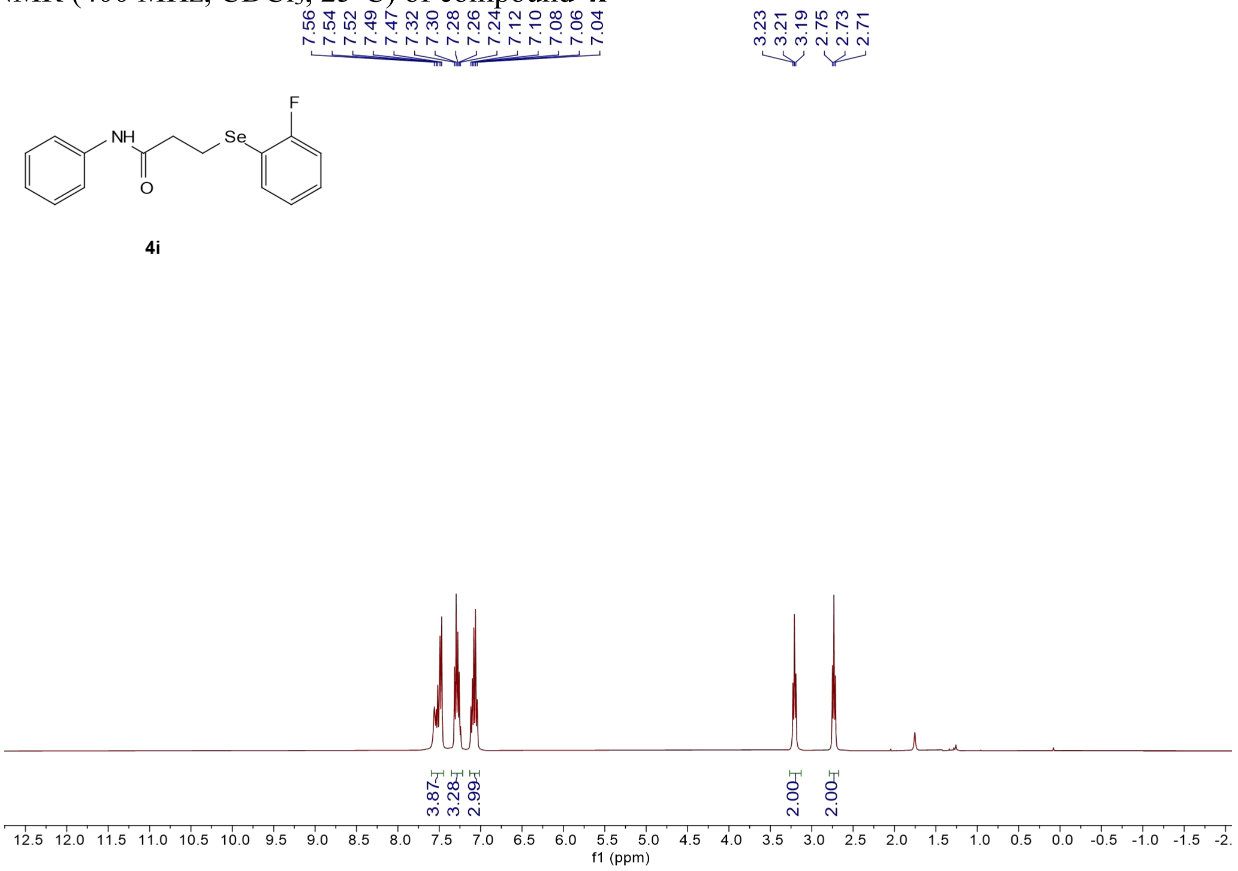

4i

^{13}C NMR (101 MHz, $CDCl_3$, 25°C) of compound **4i**

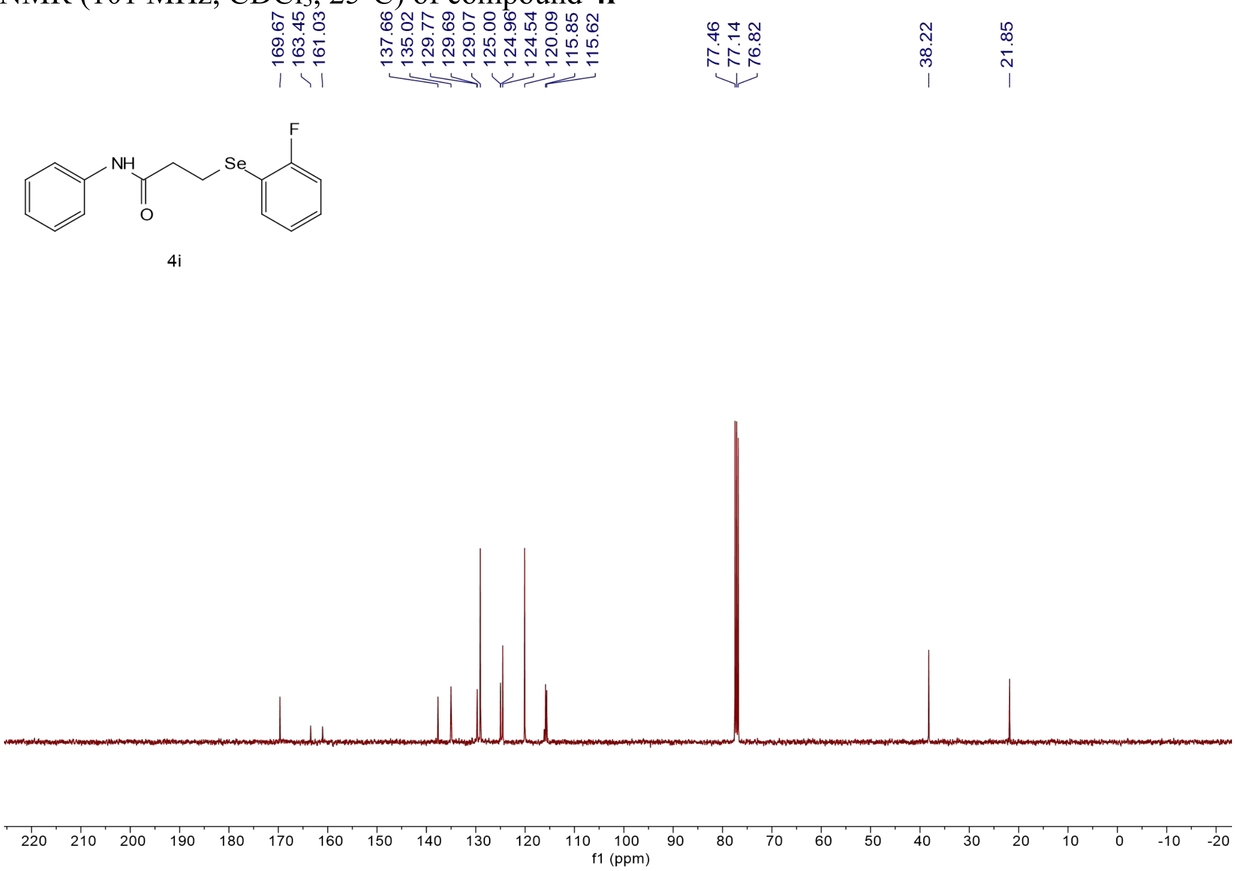

^{77}Se NMR (76 MHz, $CDCl_3$, 25°C) of compound **4i**

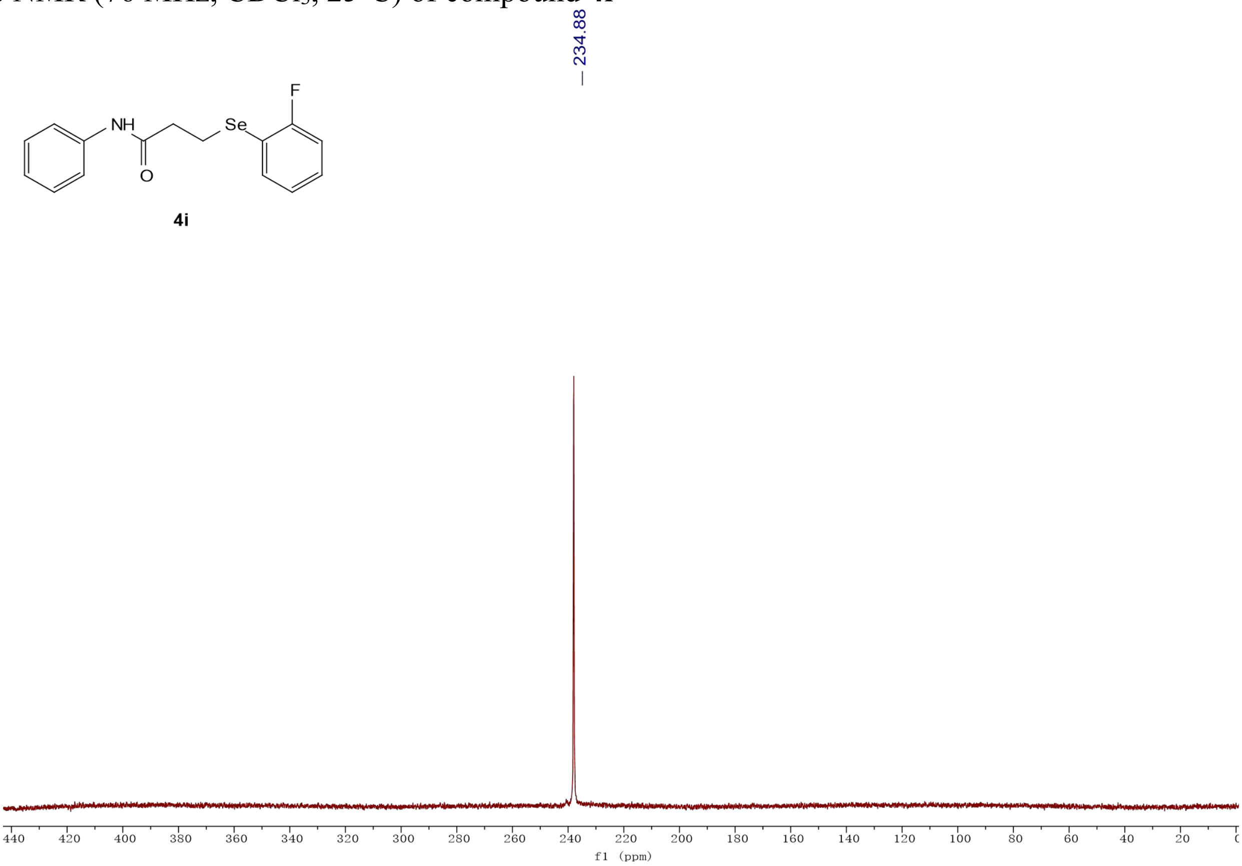

^{19}F NMR (376 MHz, $CDCl_3$, 25°C) of compound **4i**

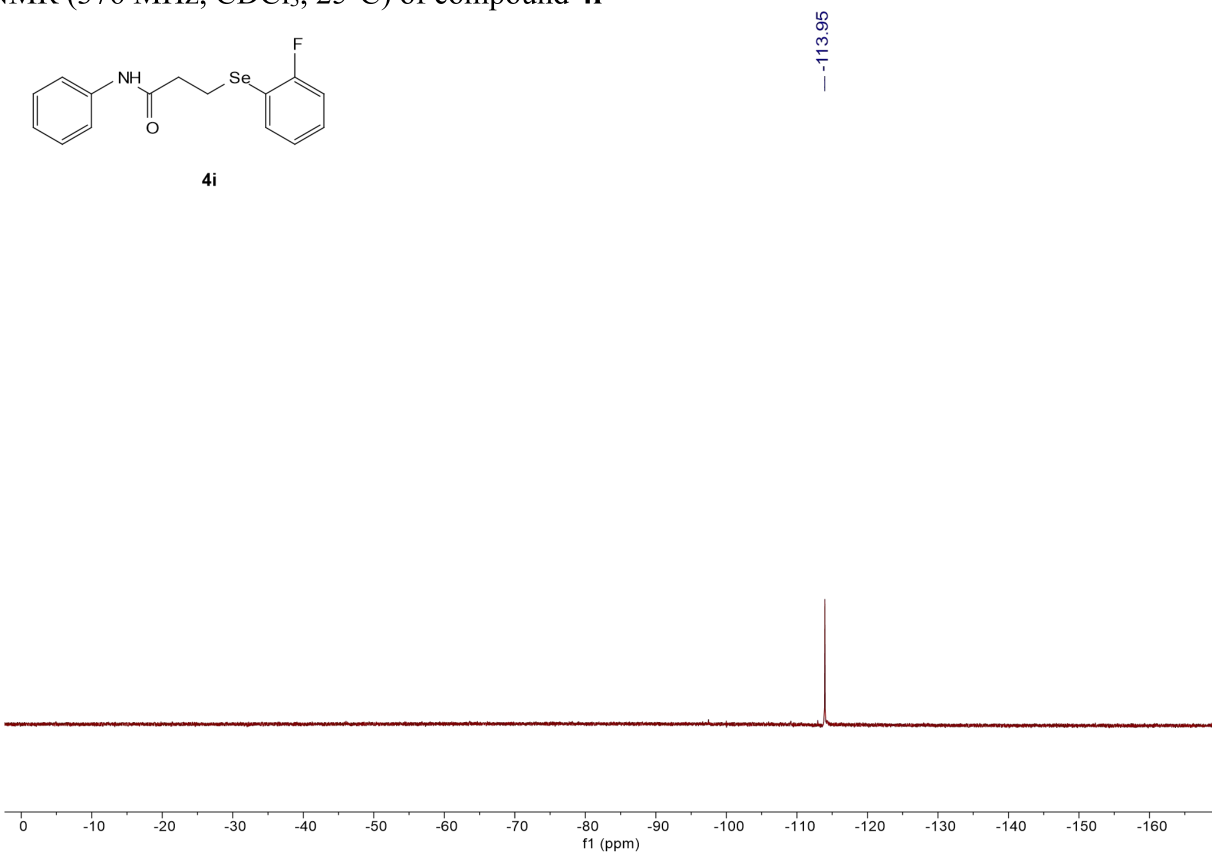

Supplementary Fig. 68. NMR spectra of compound **4i**

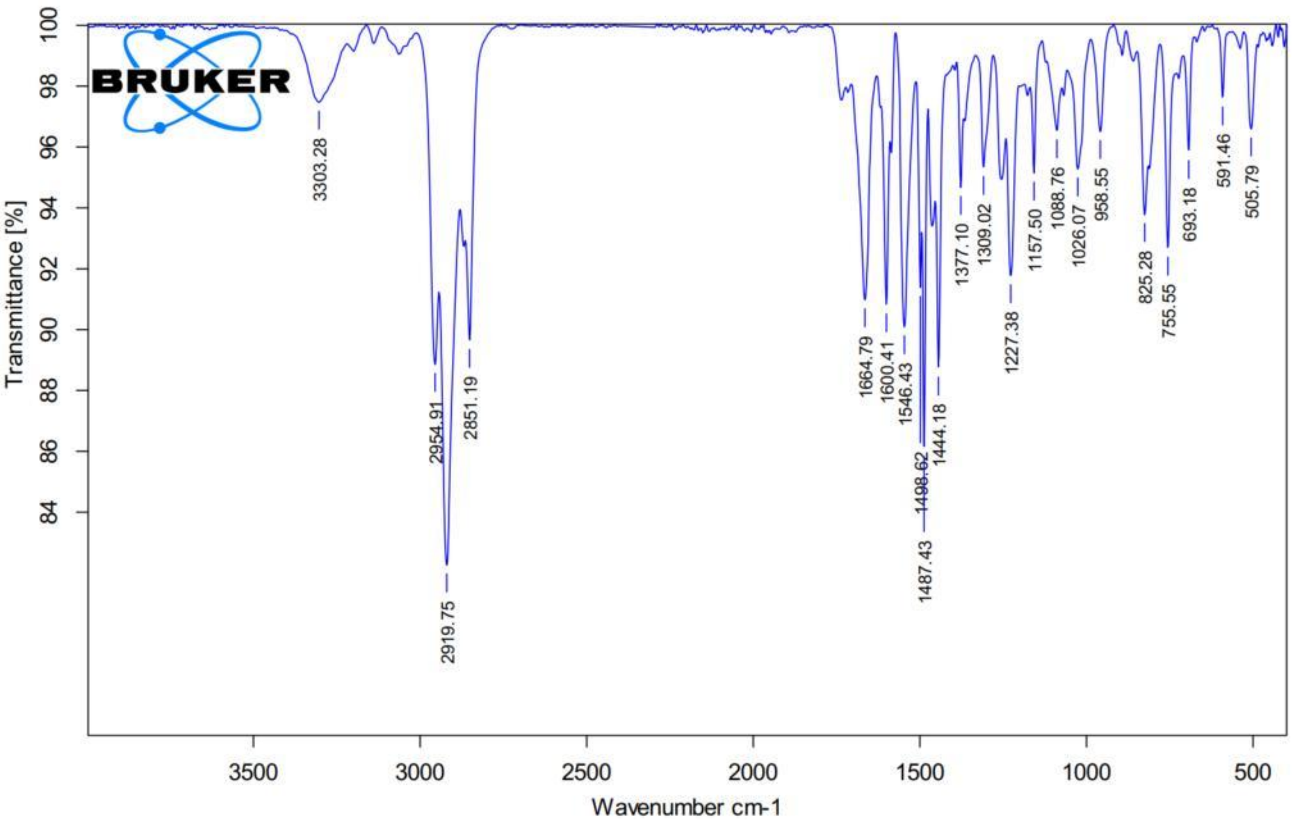

Supplementary Fig. 69. IR of compound **4i**

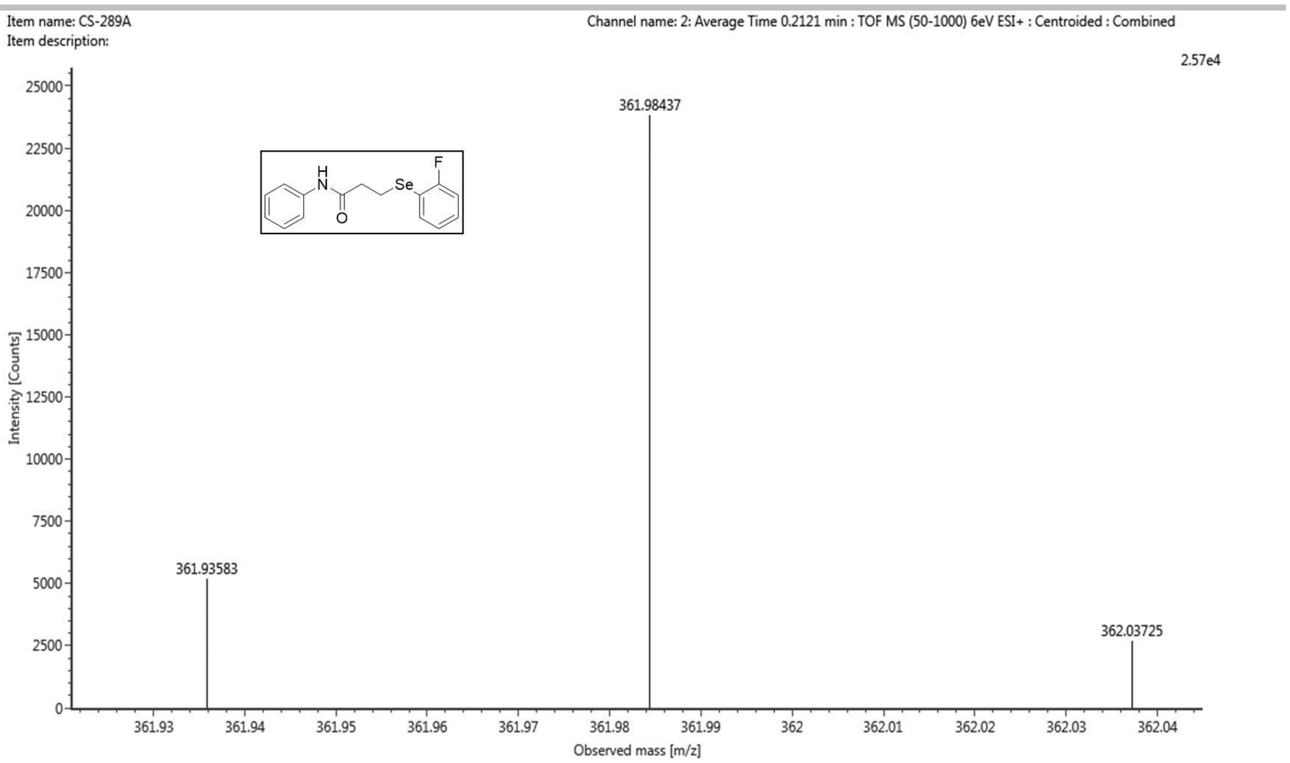

Supplementary Fig. 70. HR-MS of compound **4i**

^{1}H NMR (400 MHz, $CDCl_3$, 25°C) of compound **4j**

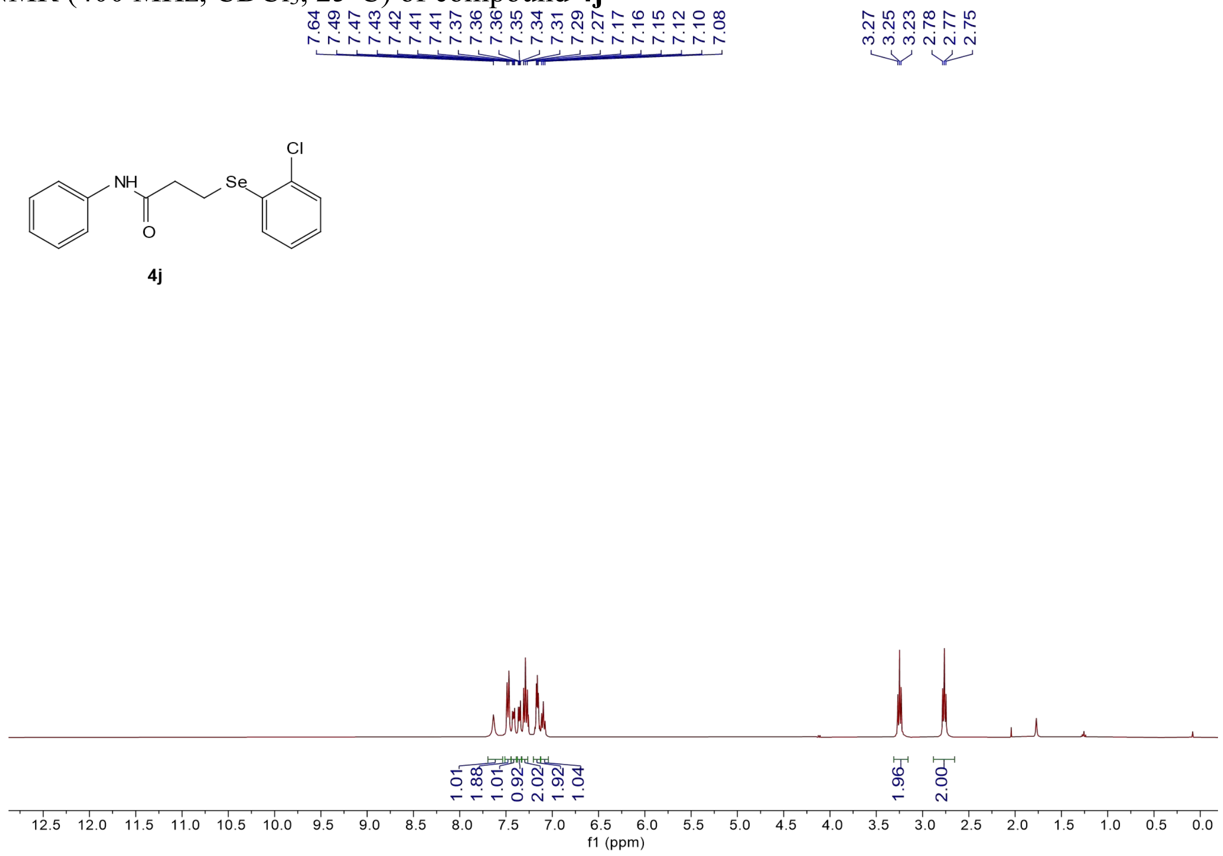

^{13}C NMR (101 MHz, $CDCl_3$, 25°C) of compound **4j**

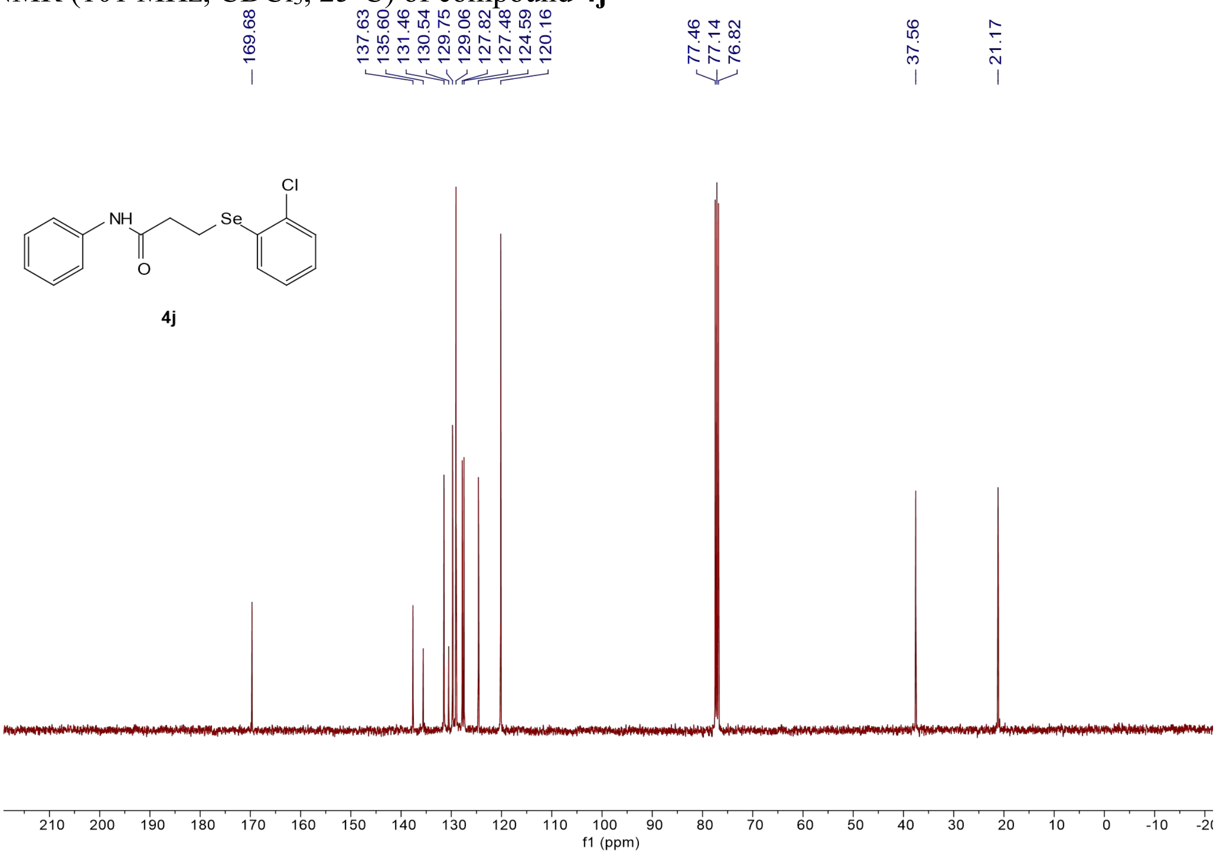

^{77}Se NMR (76 MHz, $CDCl_3$, 25°C) of compound **4j**

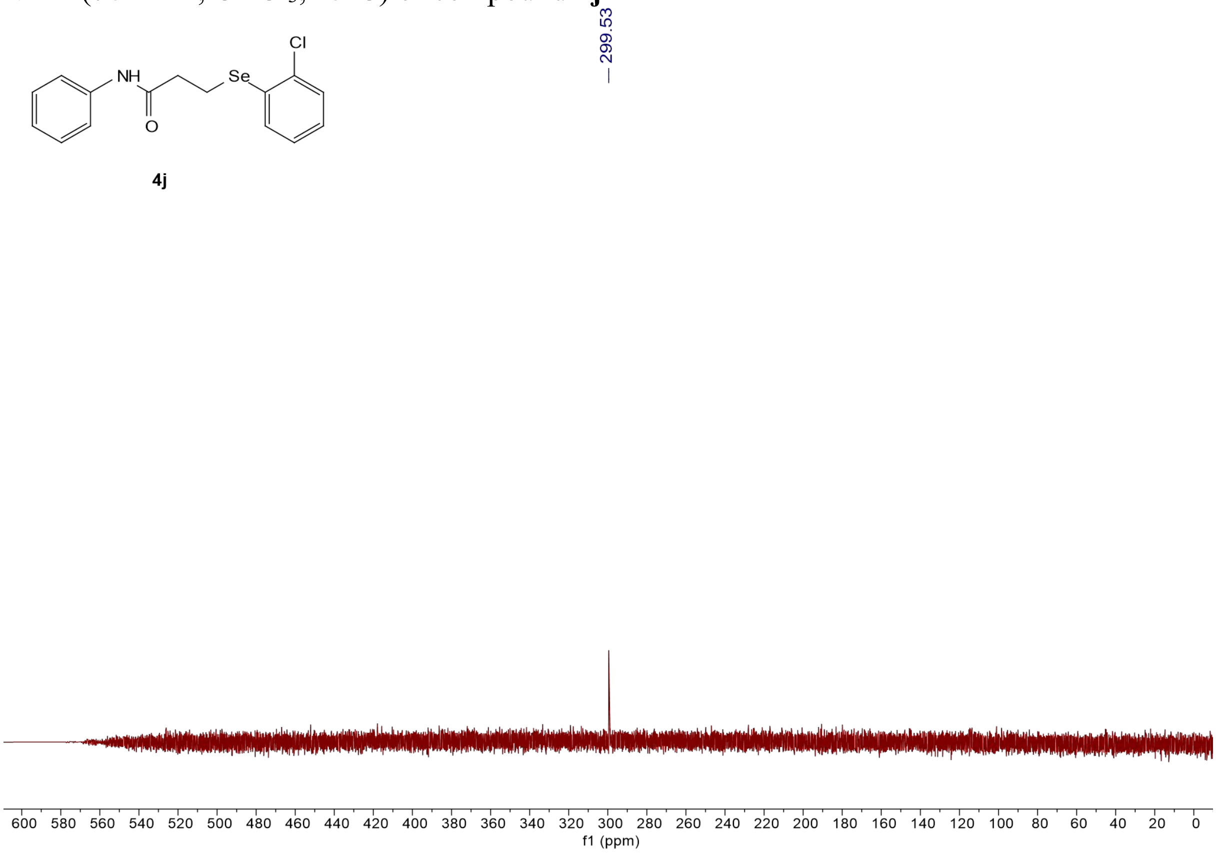

Supplementary Fig. 71. NMR spectra of compound 4j

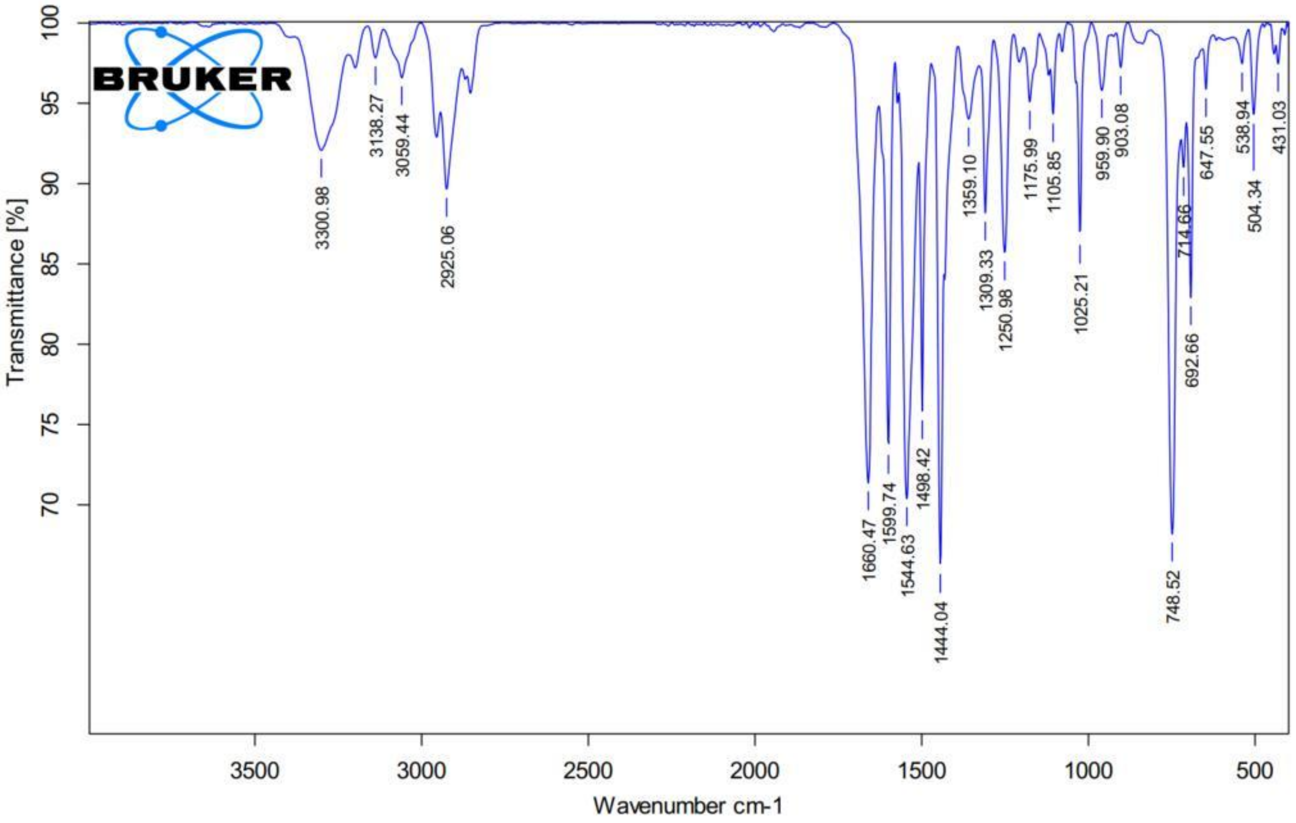

Supplementary Fig. 72. IR of compound **4j**

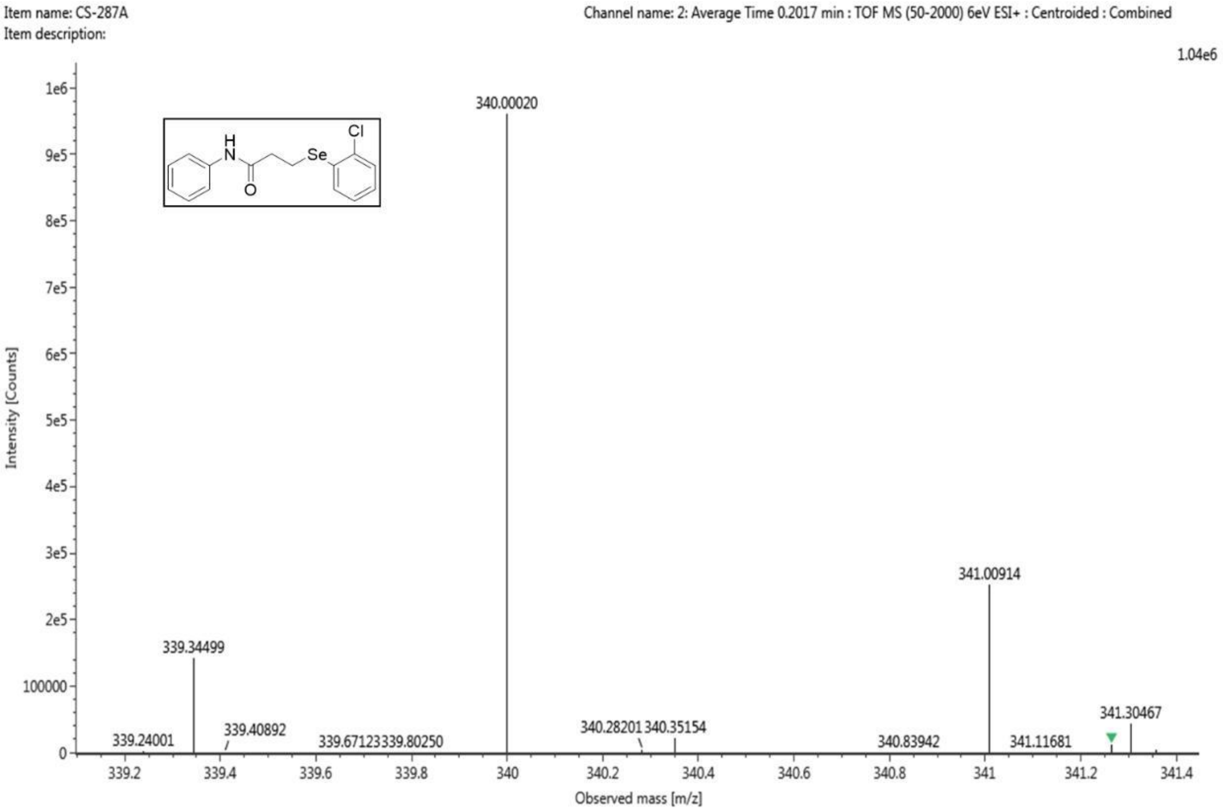

Supplementary Fig. 73. HR-MS of compound **4j**

^{1}H NMR (400 MHz, $CDCl_3$, 25°C) of compound **4k**

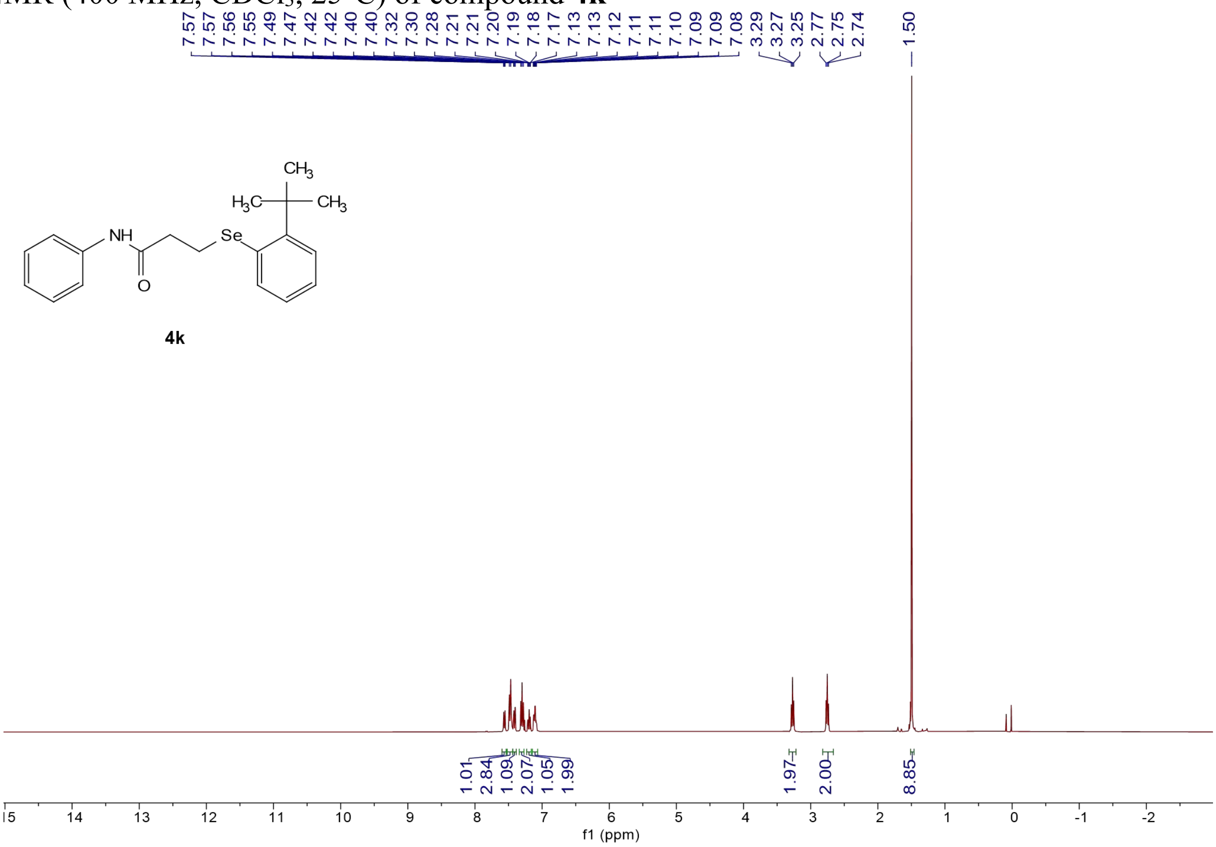

^{13}C NMR (101 MHz, $CDCl_3$, 25°C) of compound **4k**

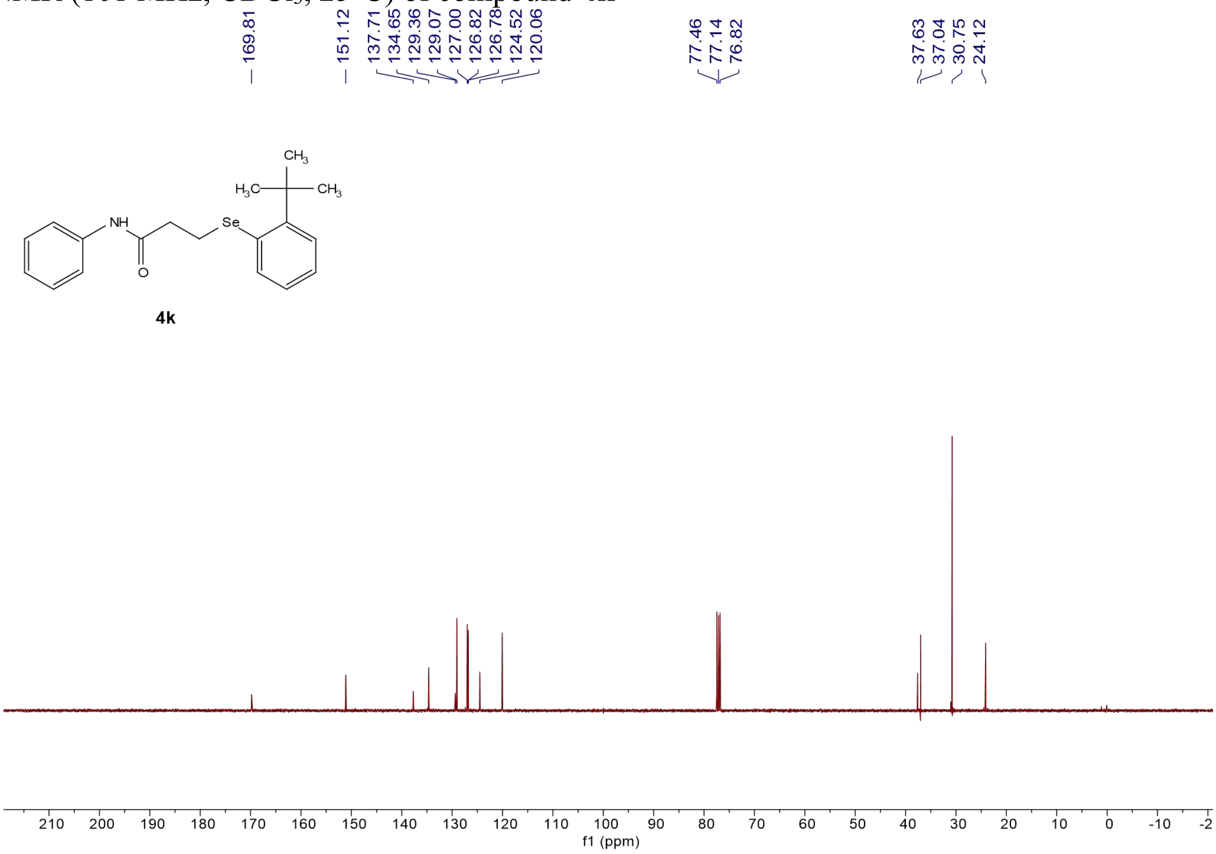

^{77}Se NMR (76 MHz, $CDCl_3$, 25°C) of compound **4k**

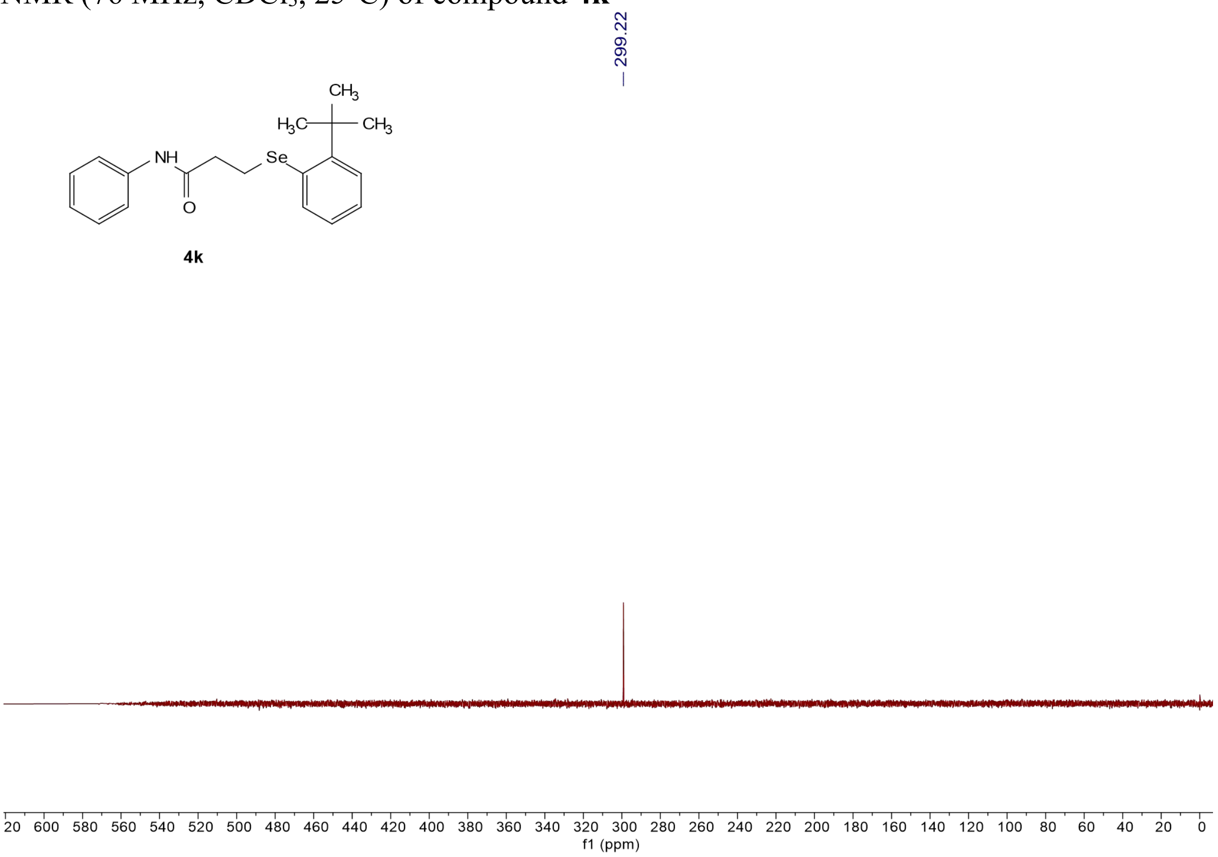

Supplementary Fig. 74. NMR spectra of compound 4k

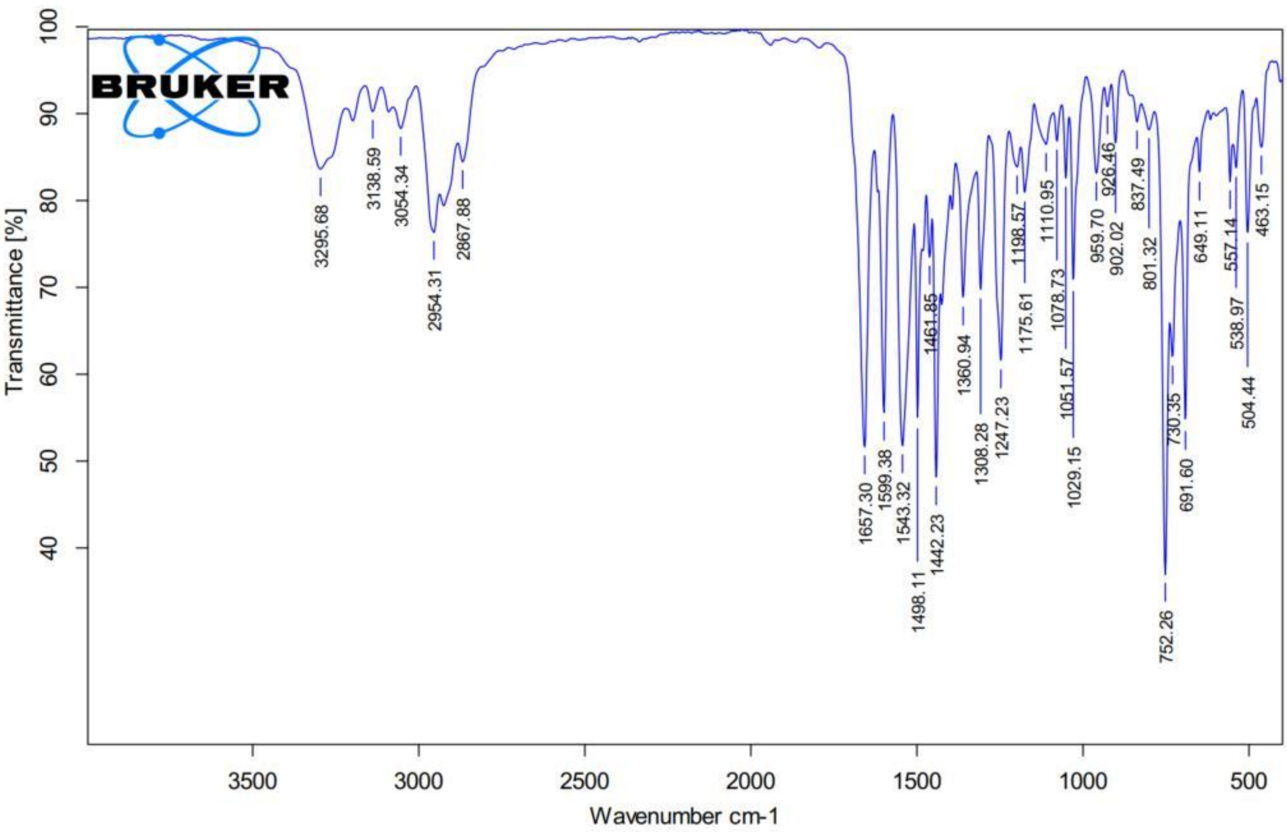

Supplementary Fig. 75. IR of compound **4k**

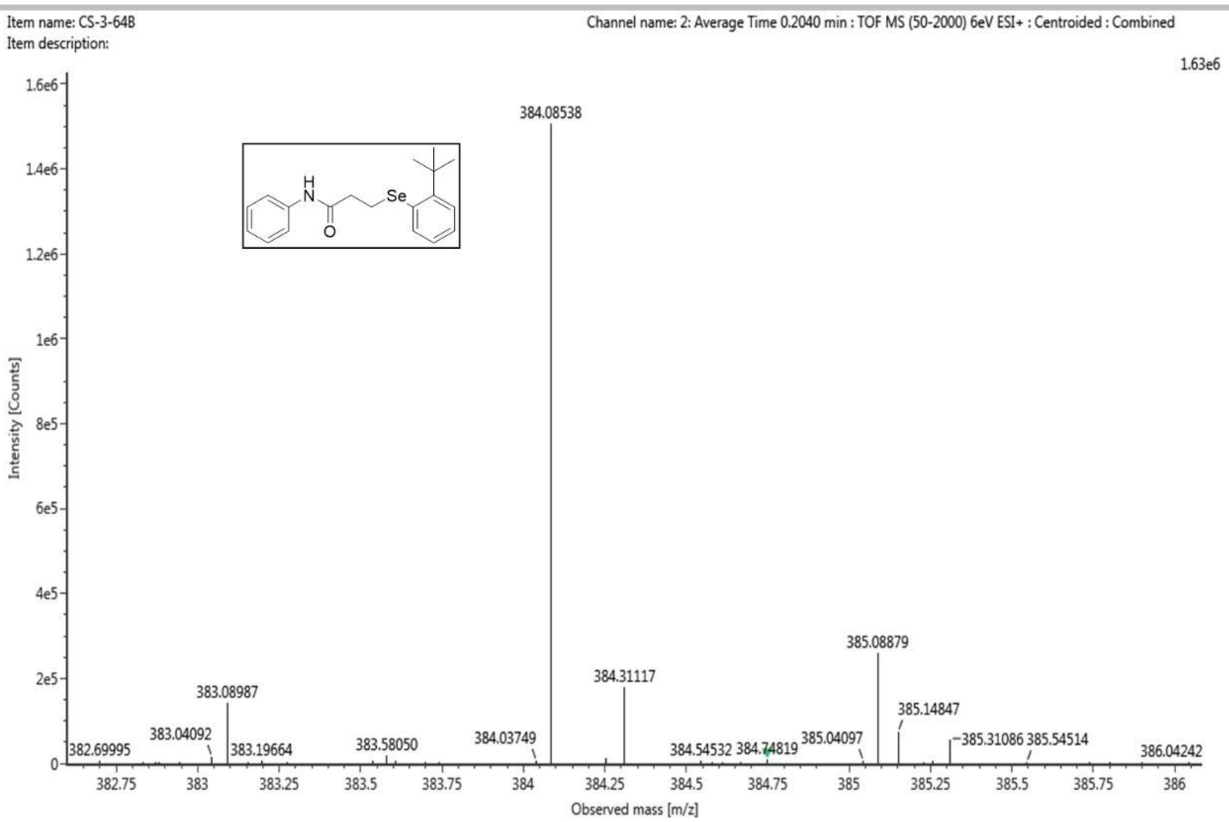

Supplementary Fig. 76. HR-MS of compound **4k**

^{1}H NMR (400 MHz, $CDCl_3$, 25°C) of compound **4l**

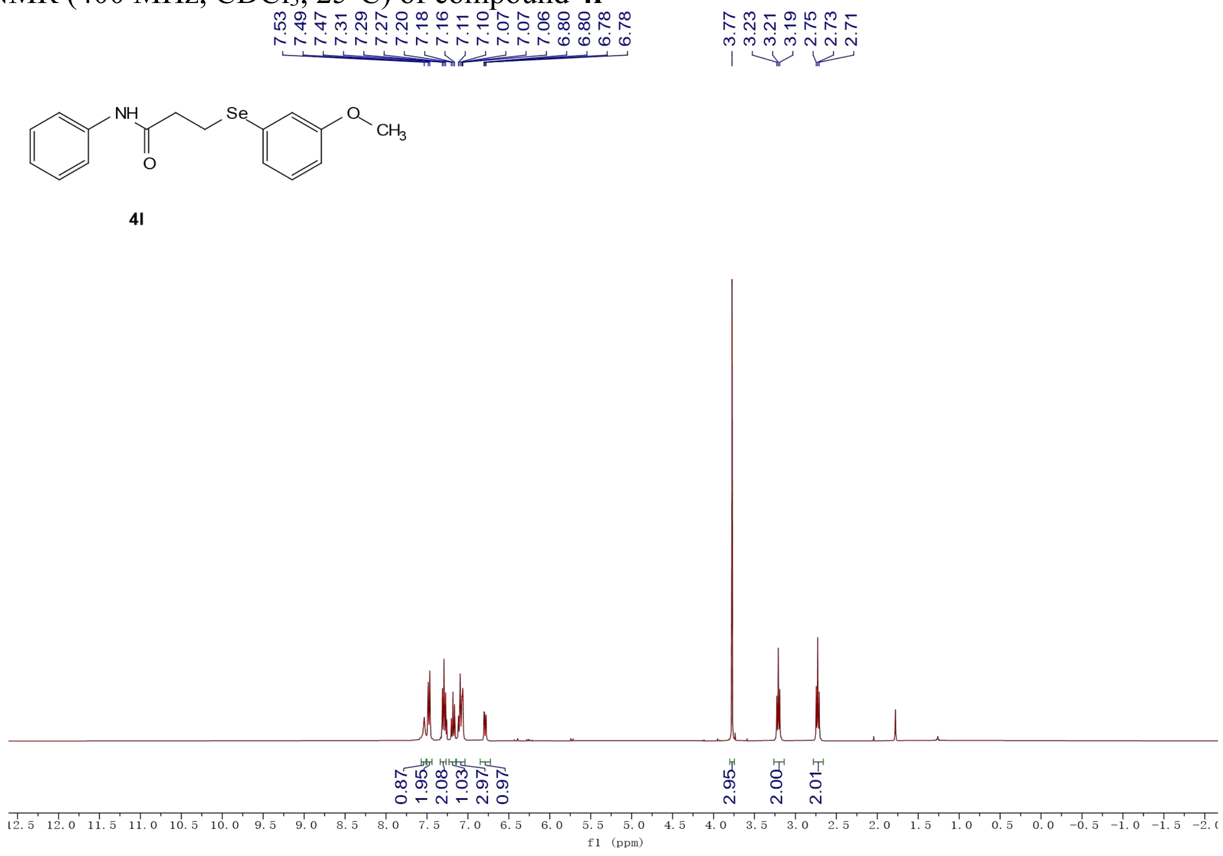

^{13}C NMR (101 MHz, $CDCl_3$, 25°C) of compound **4l**

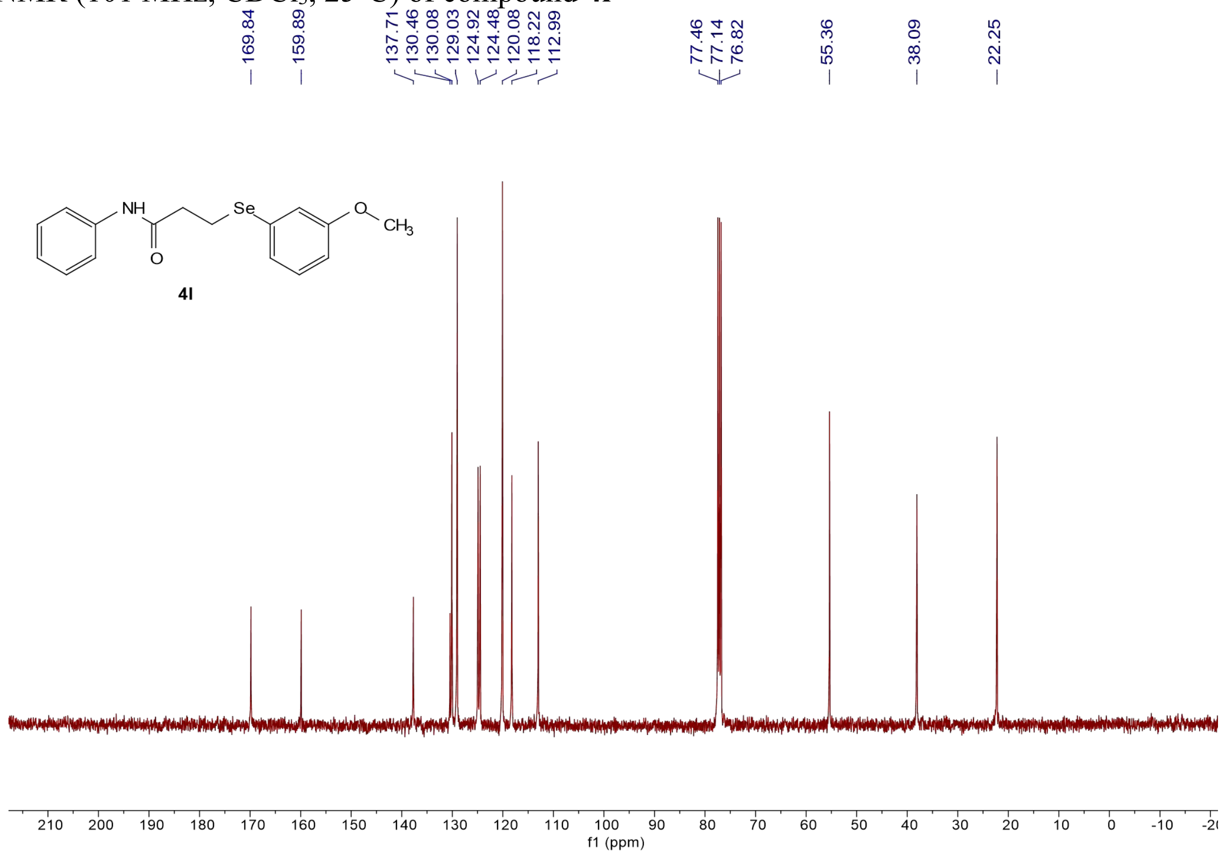

^{77}Se NMR (76 MHz, $CDCl_3$, 25°C) of compound **4l**

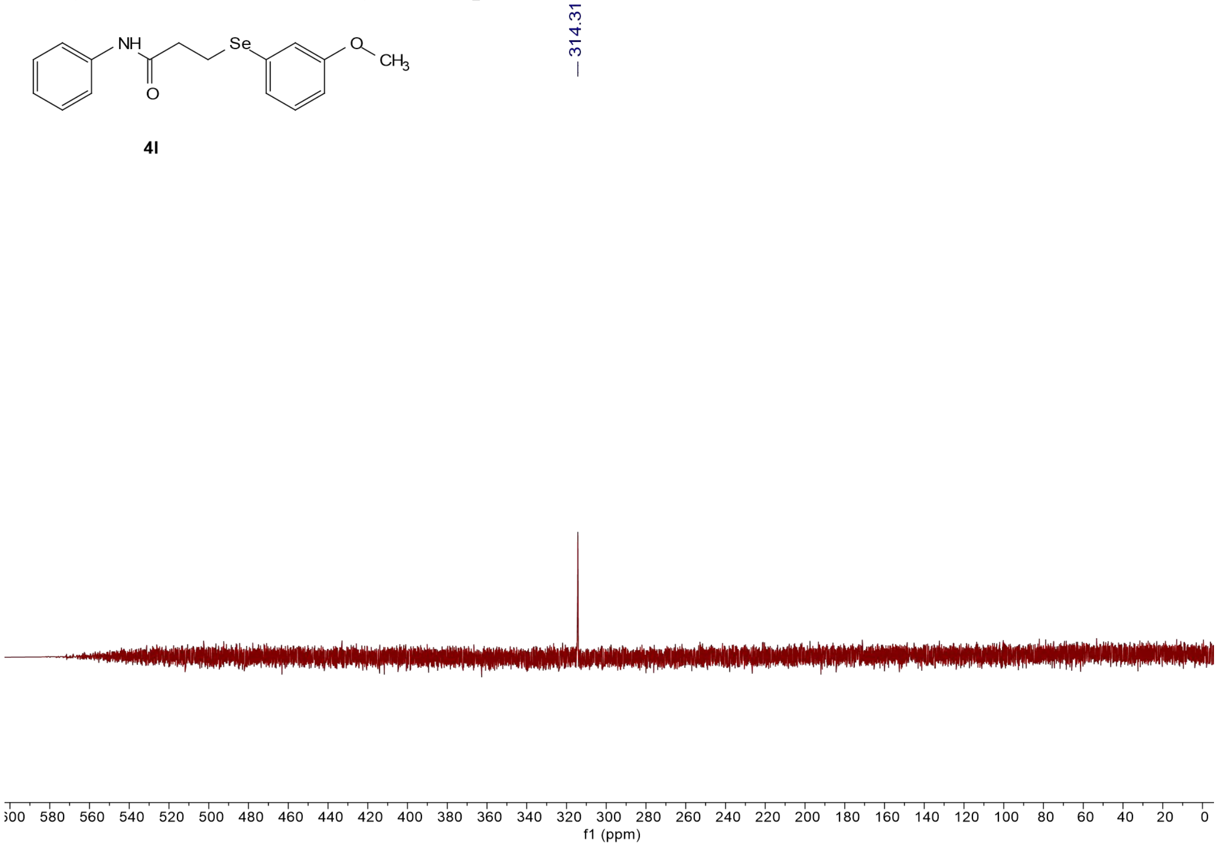

Supplementary Fig. 77. NMR spectra of compound **4l**

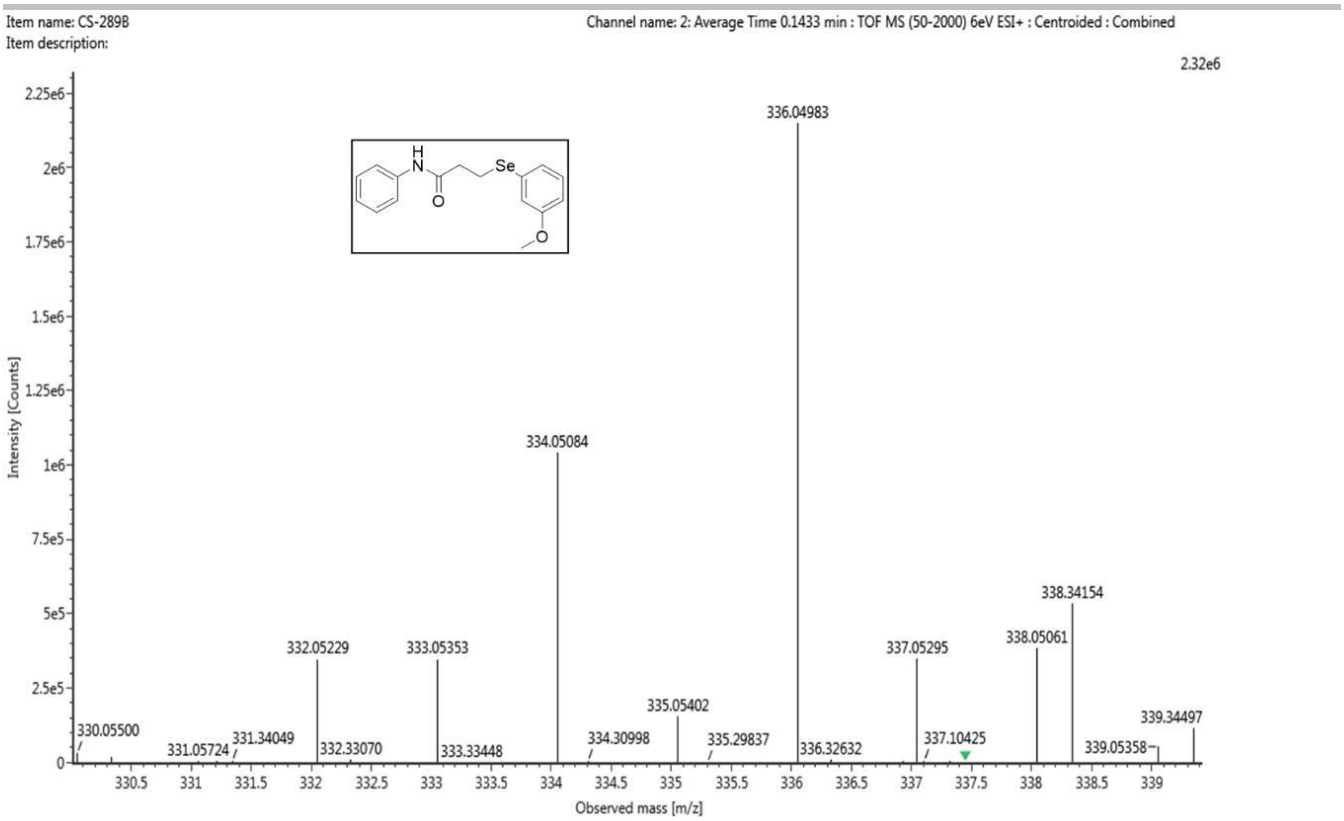

Supplementary Fig. 78. HR-MS of compound **4l**

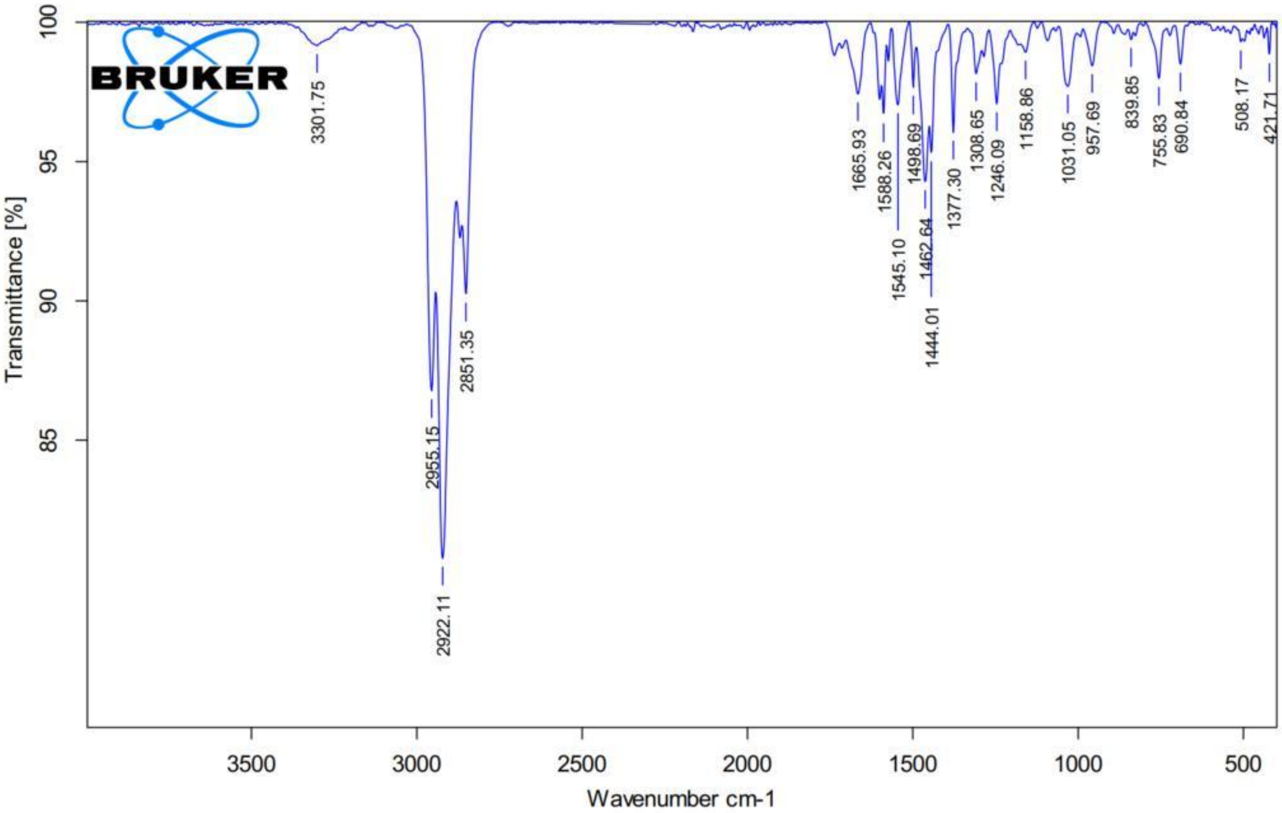

Supplementary Fig. 79. IR of compound **4l**

^{1}H NMR (400 MHz, $CDCl_3$, 25°C) of compound **4m**

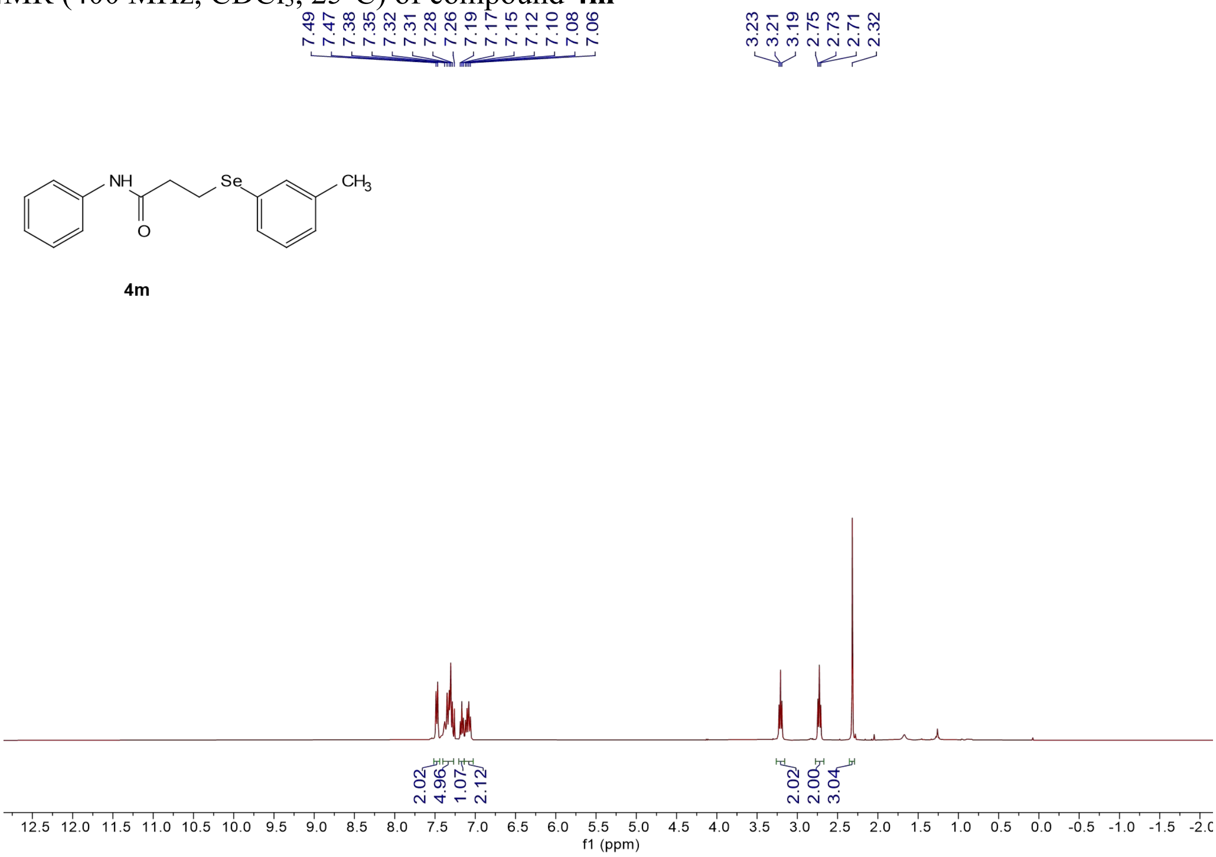

^{13}C NMR (101 MHz, $CDCl_3$, 25°C) of compound **4m**

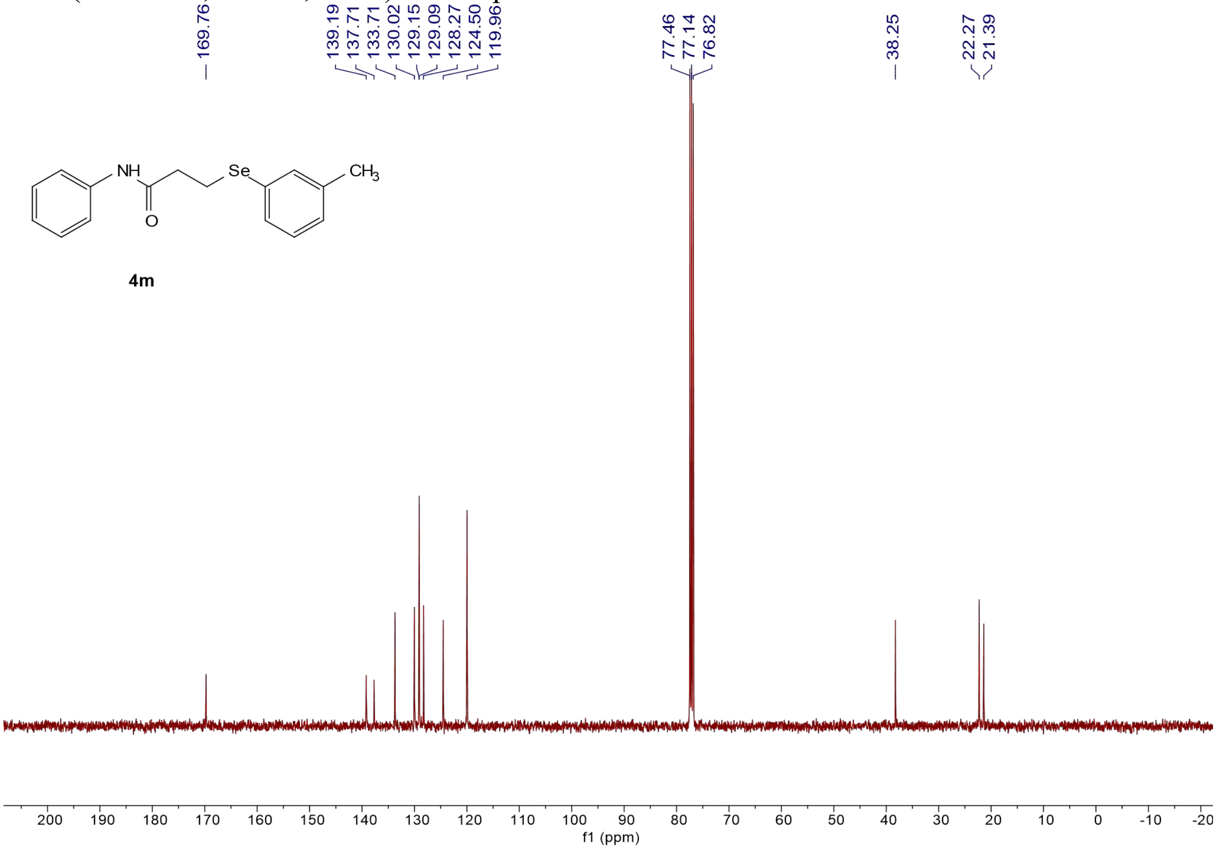

^{77}Se NMR (76 MHz, $CDCl_3$, 25°C) of compound **4m**

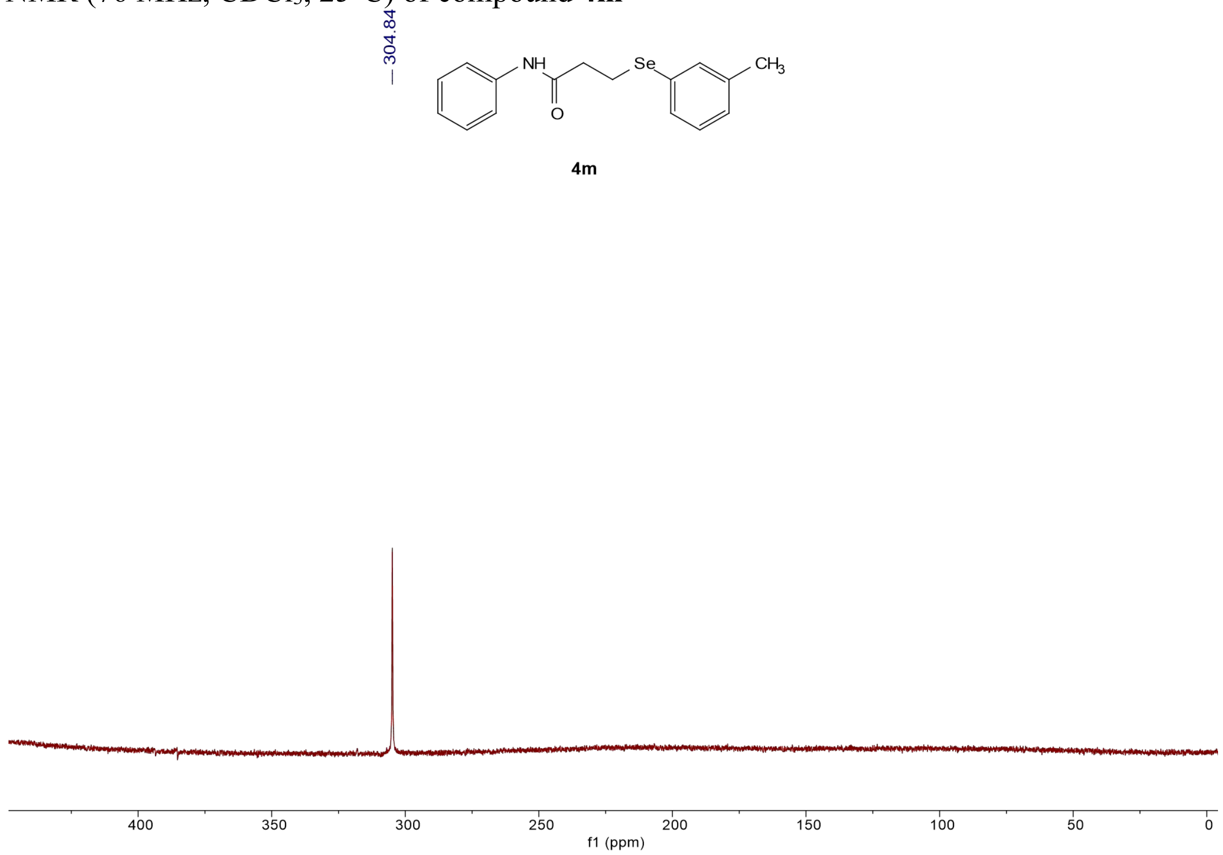

Supplementary Fig. 80. NMR spectra of compound **4m**

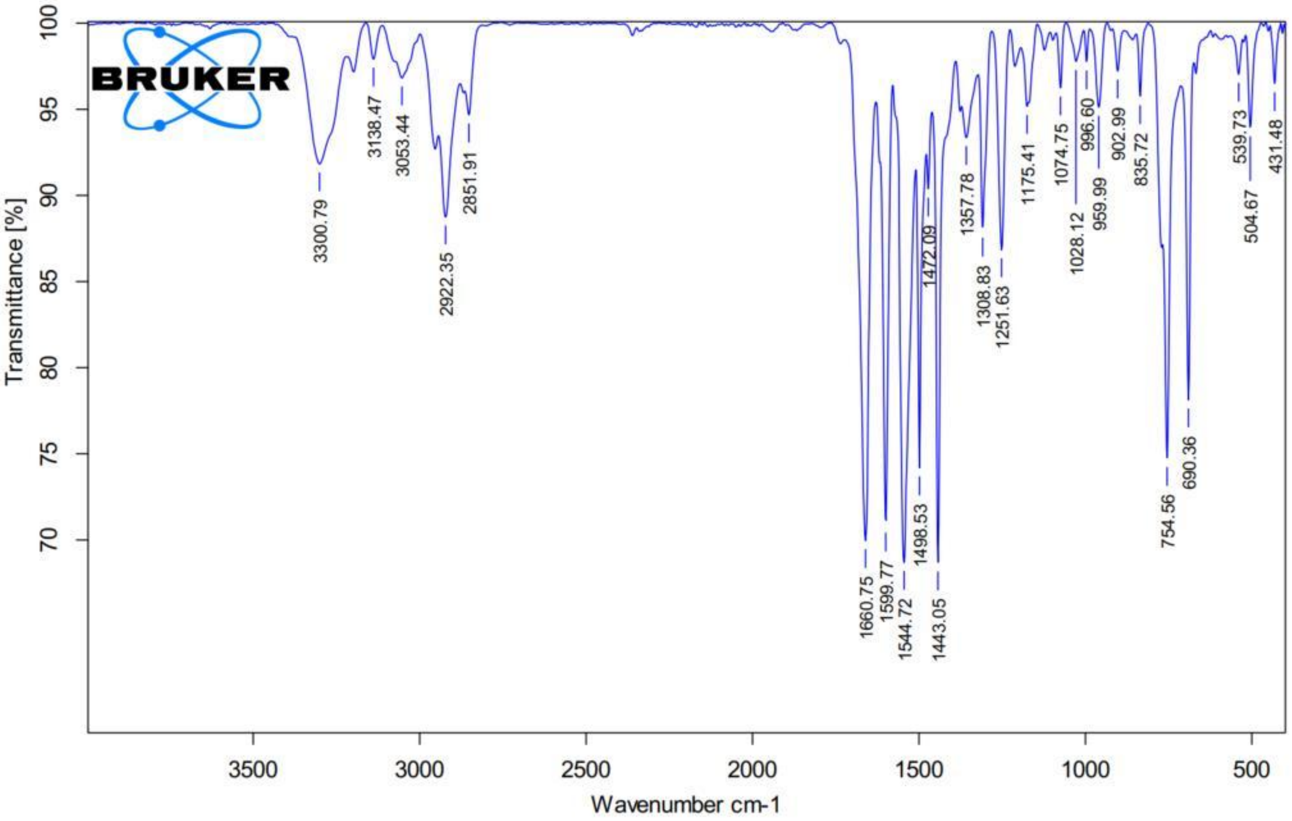

Supplementary Fig. 81. IR of compound **4m**

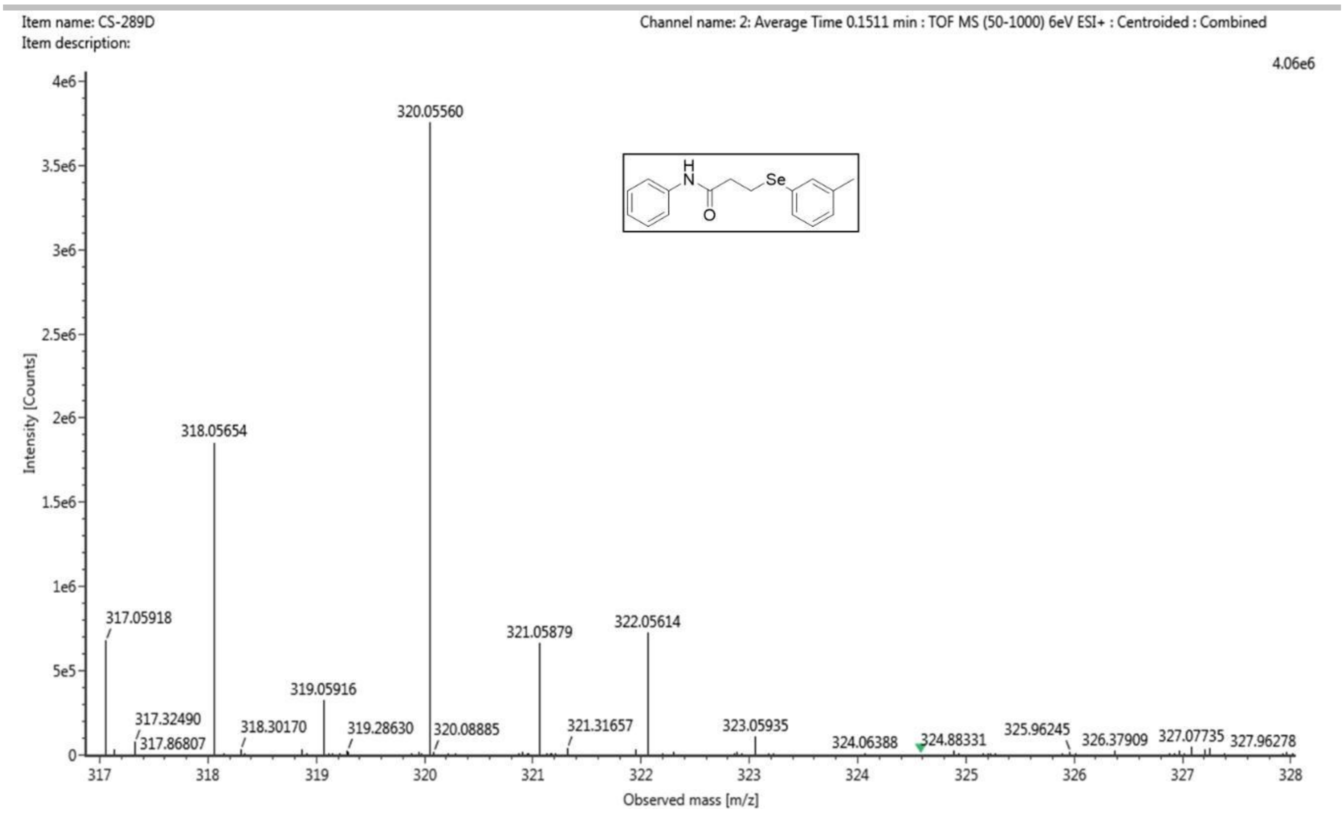

Supplementary Fig. 82. HR-MS of compound **4m**

^{1}H NMR (400 MHz, $CDCl_3$, 25°C) of compound **4n**

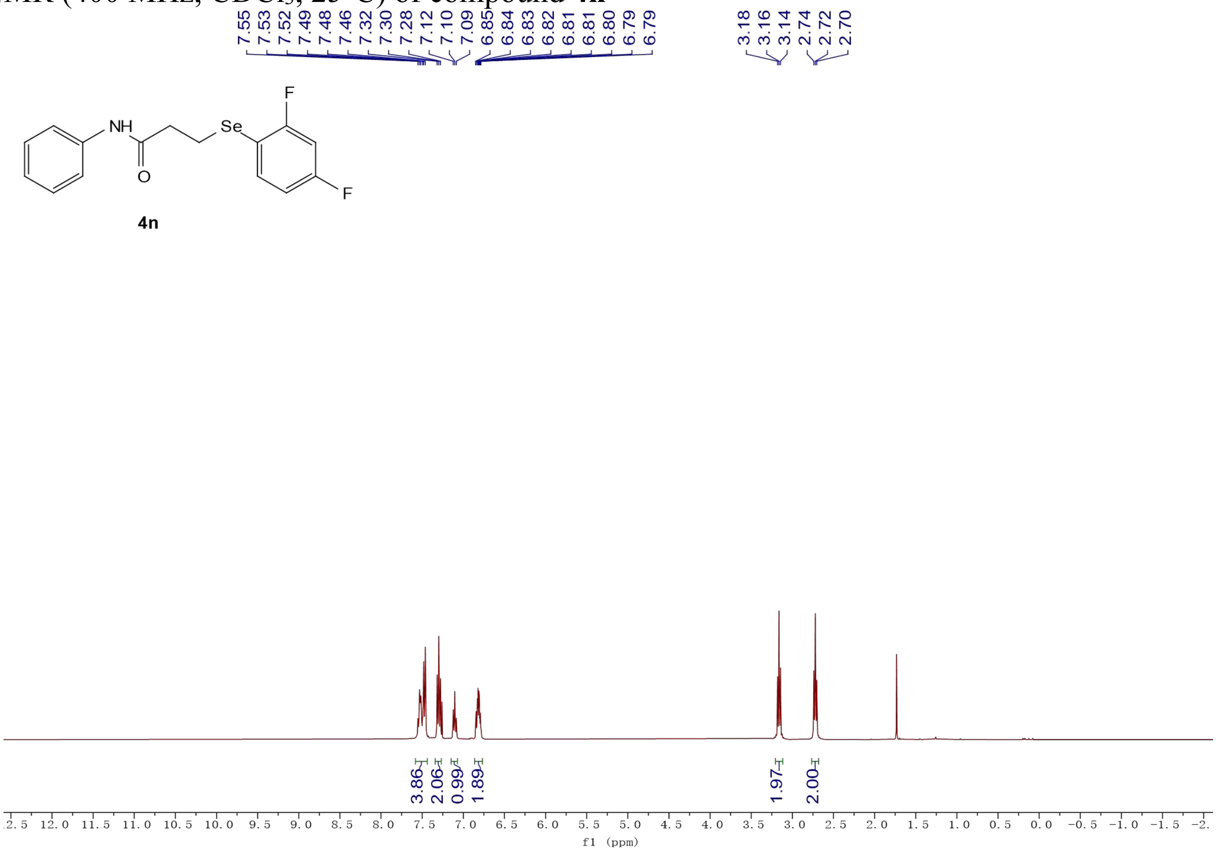

^{13}C NMR (101 MHz, $CDCl_3$, 25°C) of compound **4n**

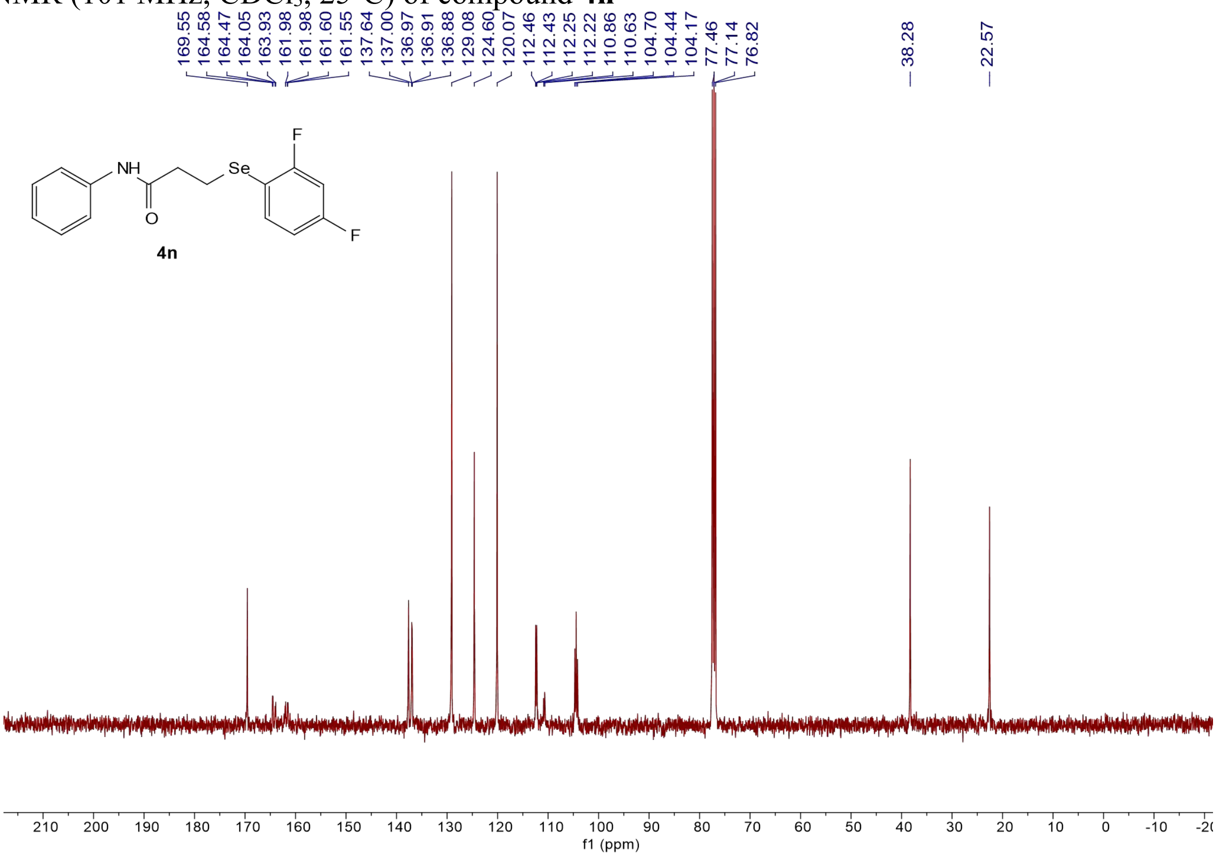

^{77}Se NMR (76 MHz, $CDCl_3$, 25°C) of compound **4n**

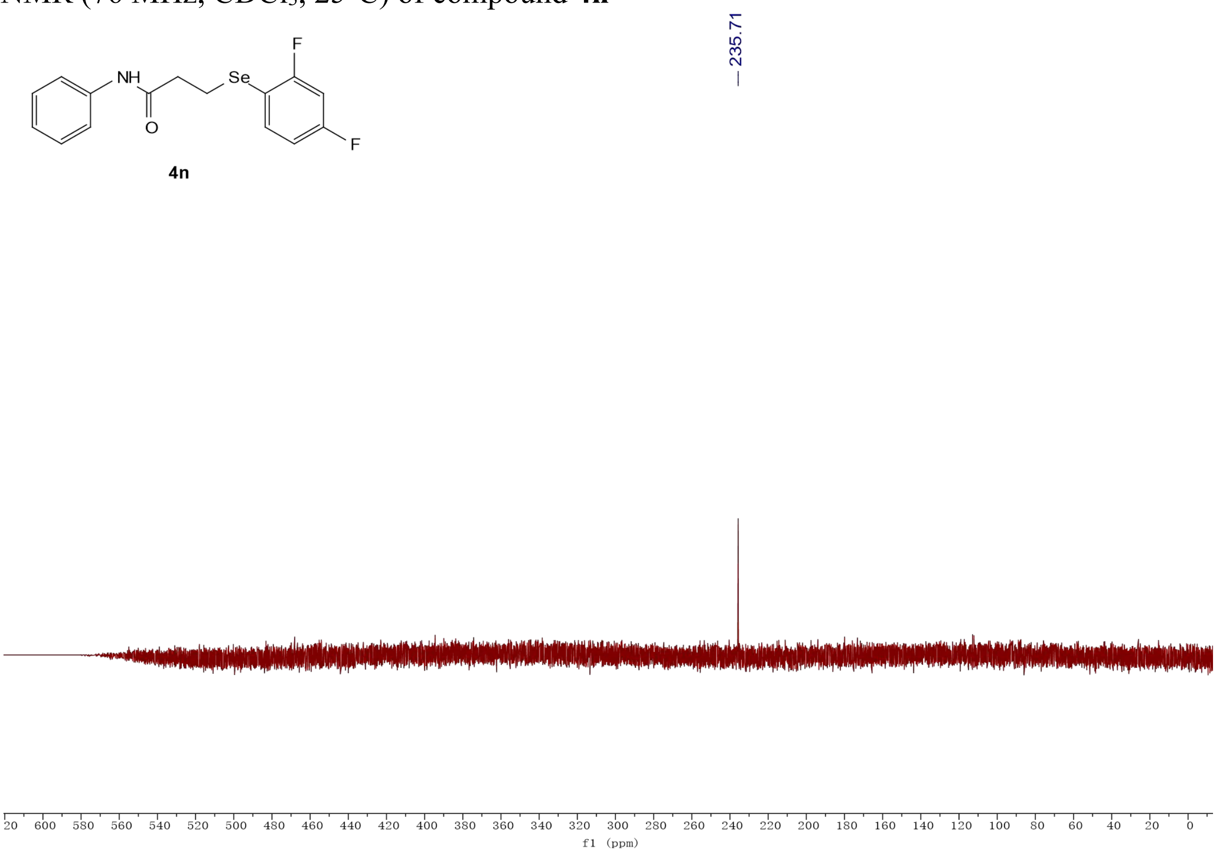

^{19}F NMR (376 MHz, $CDCl_3$, 25°C) of compound **4n**

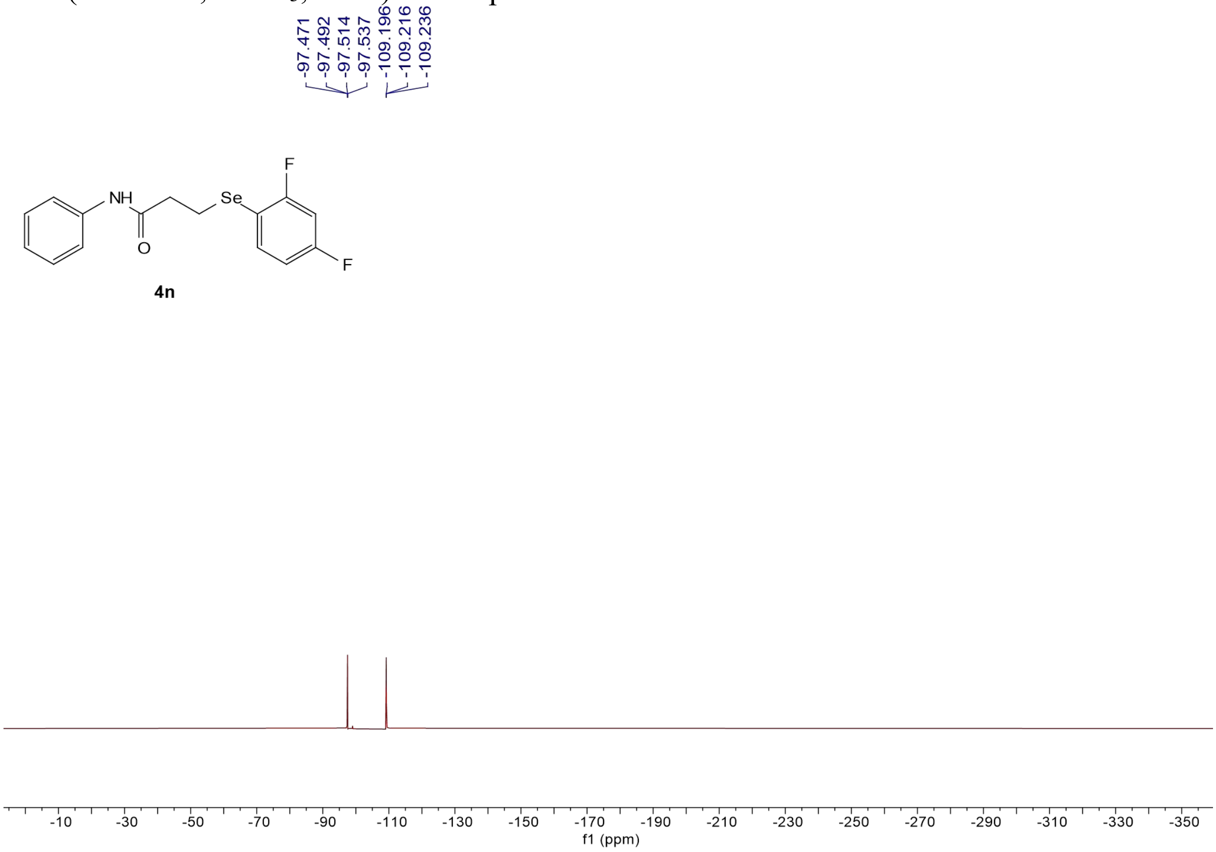

Supplementary Fig. 83. NMR spectra of compound **4n**

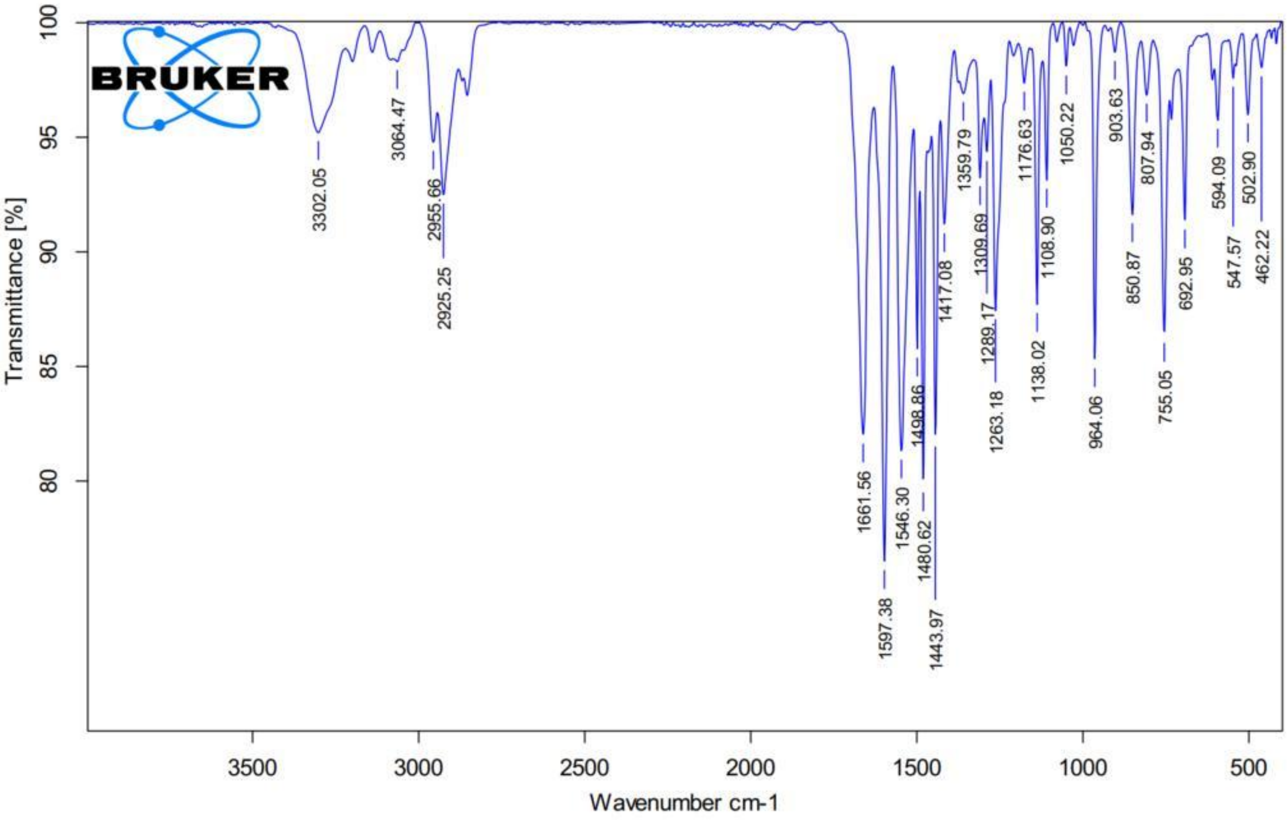

Supplementary Fig. 84. IR of compound **4n**

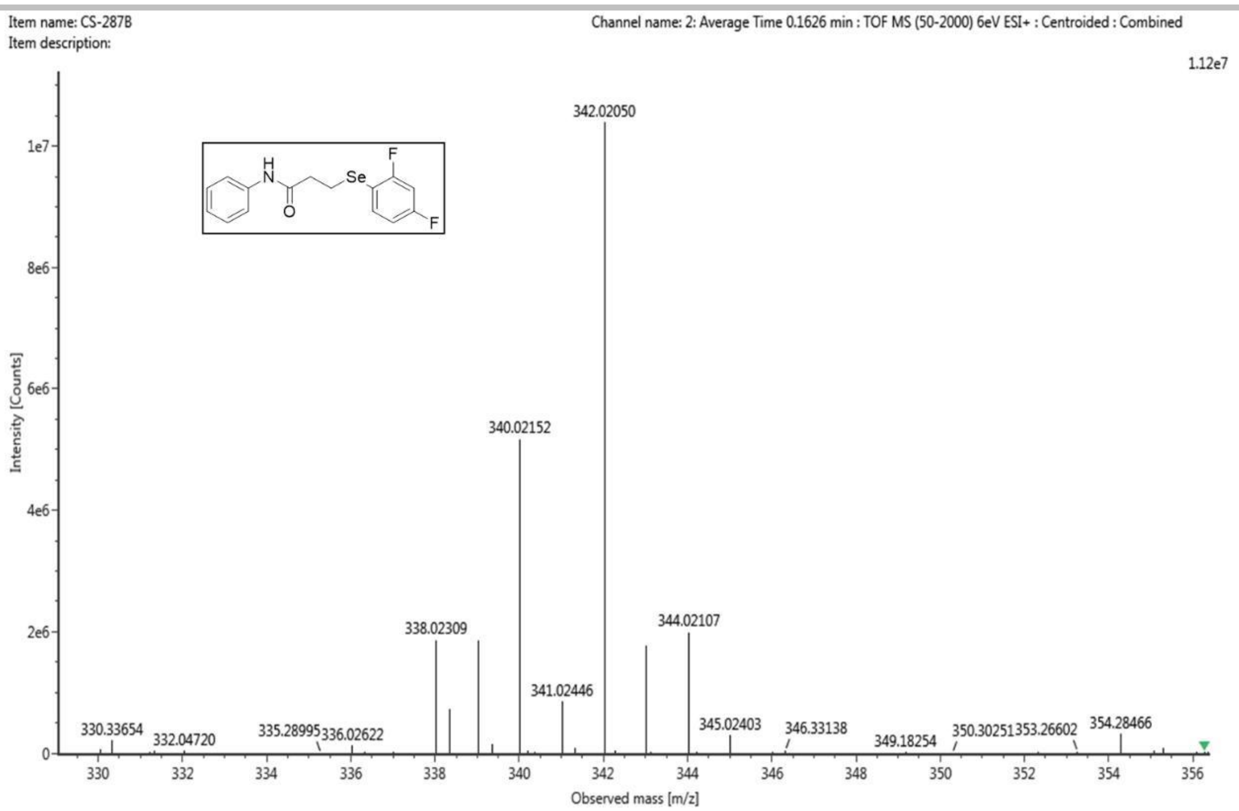

Supplementary Fig. 85. HR-MS of compound **4n**

^{1}H NMR (400 MHz, $CDCl_3$, 25°C) of compound **4o**

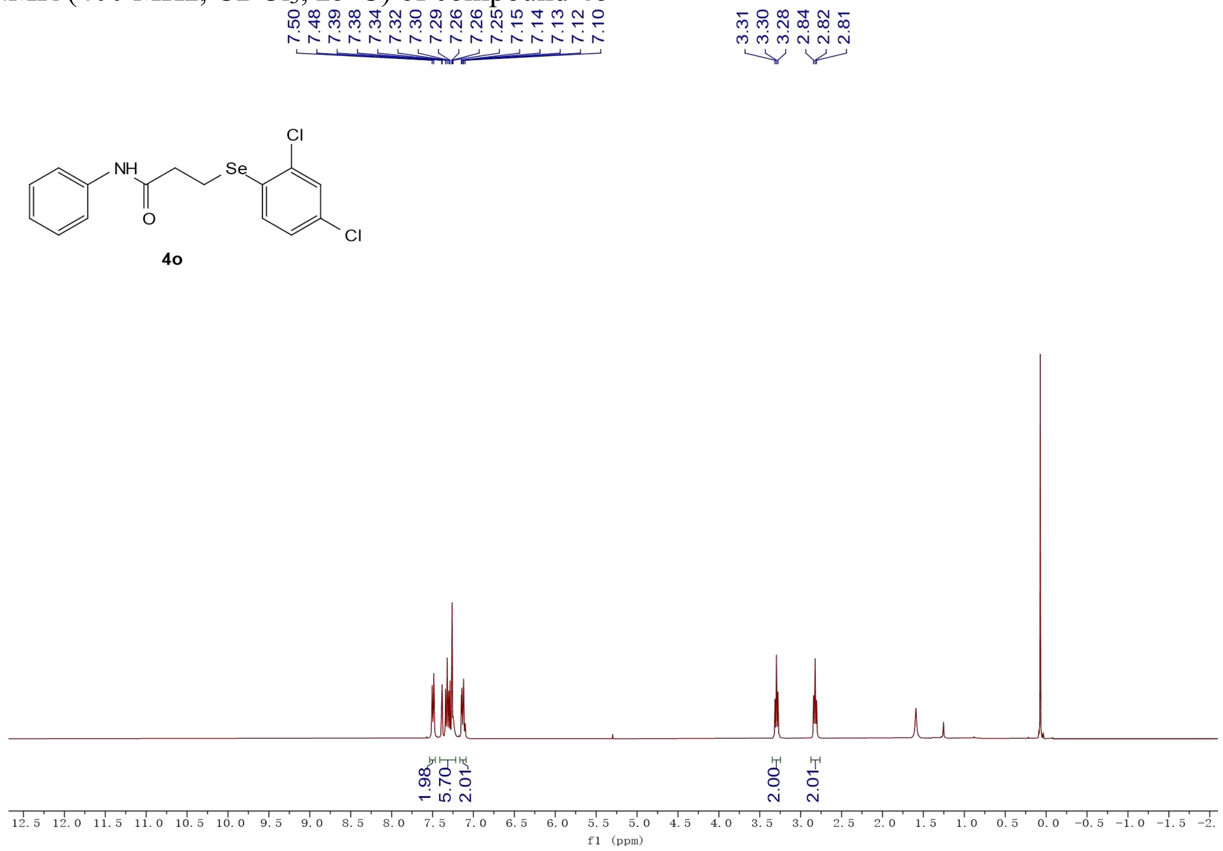

^{13}C NMR (101 MHz, $CDCl_3$, 25°C) of compound **4o**

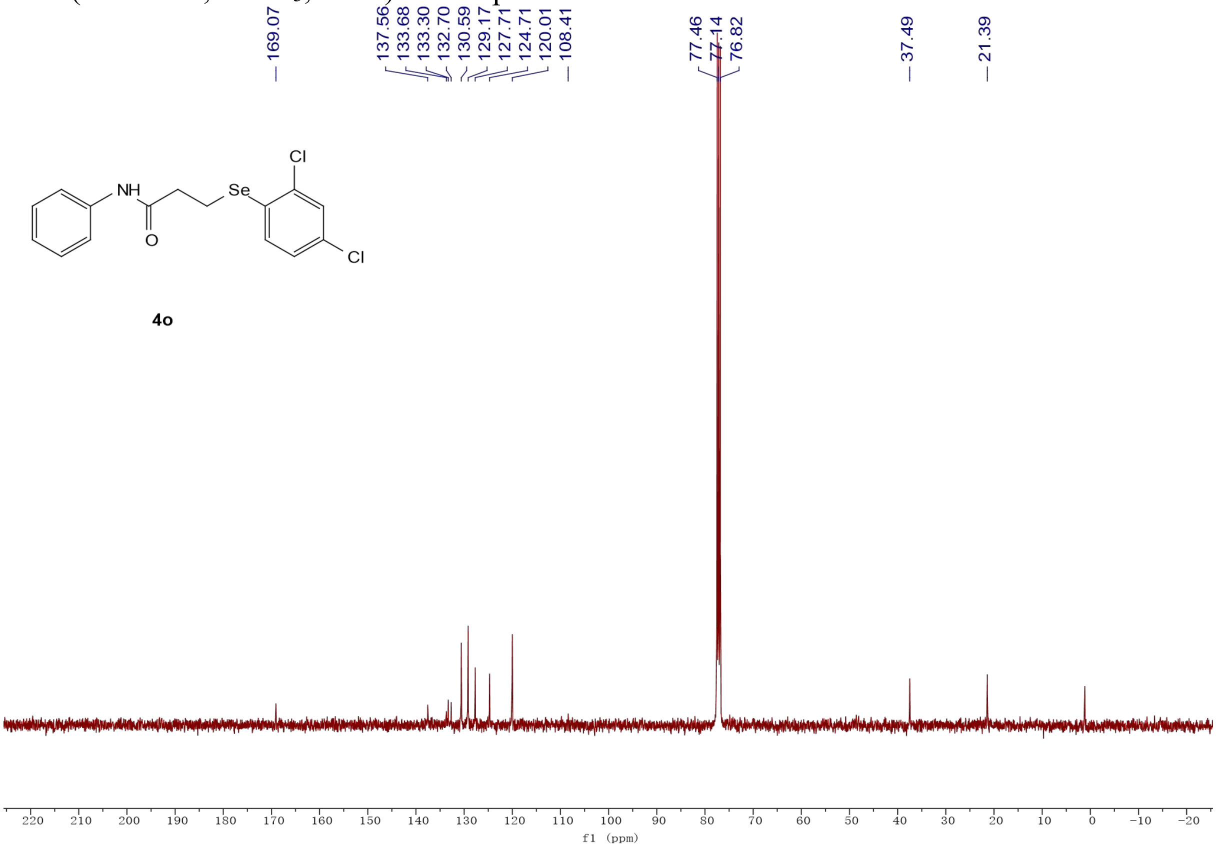

^{77}Se NMR (76 MHz, $CDCl_3$, 25°C) of compound **4o**

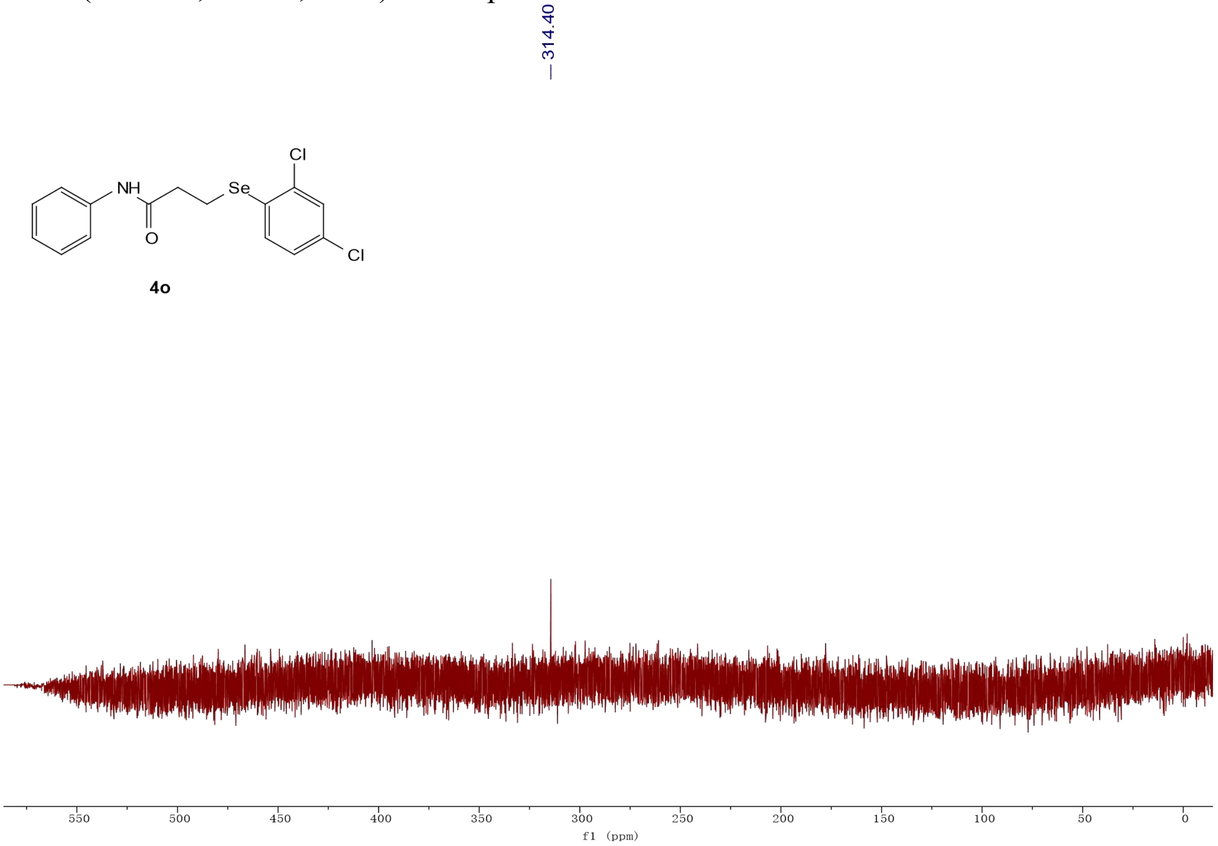

Supplementary Fig. 86. NMR spectra of compound **4o**

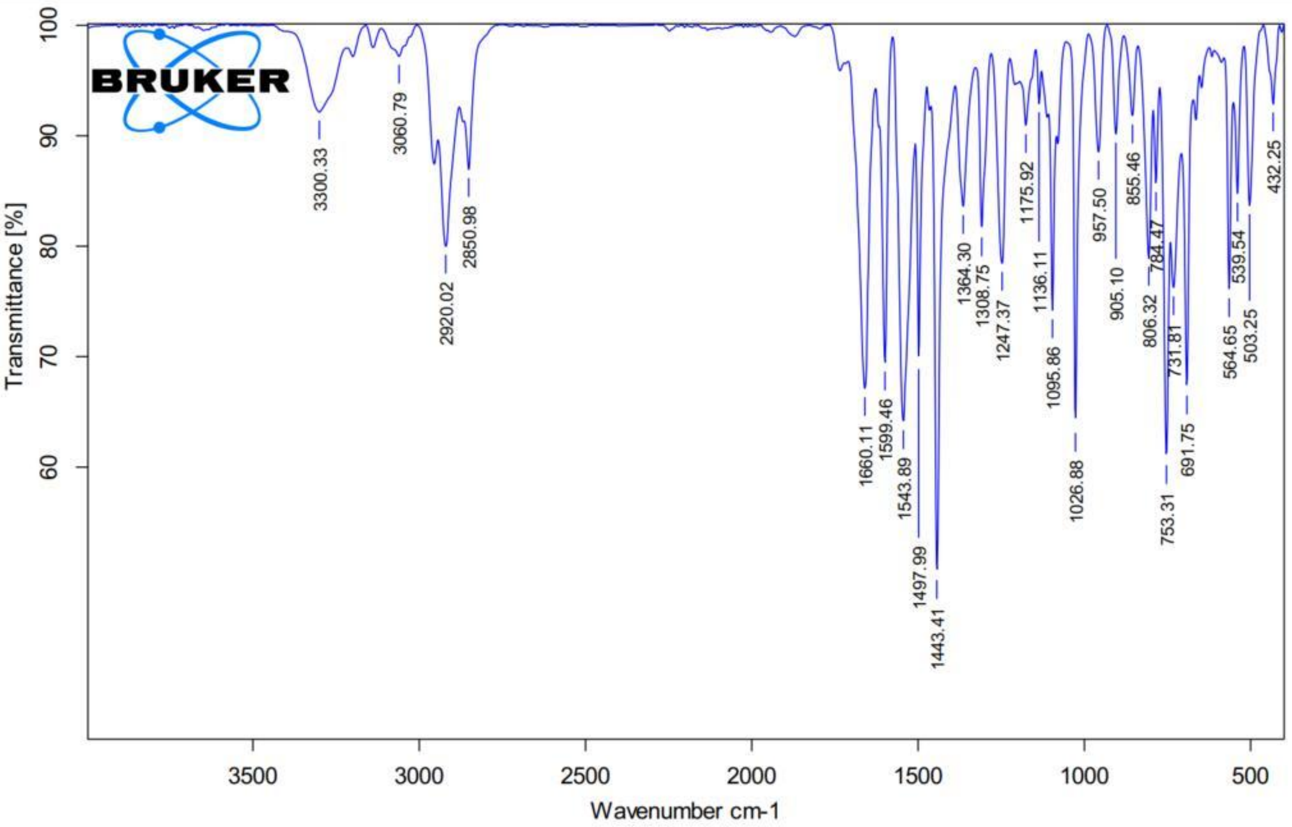

Supplementary Fig. 87. IR of compound **4o**

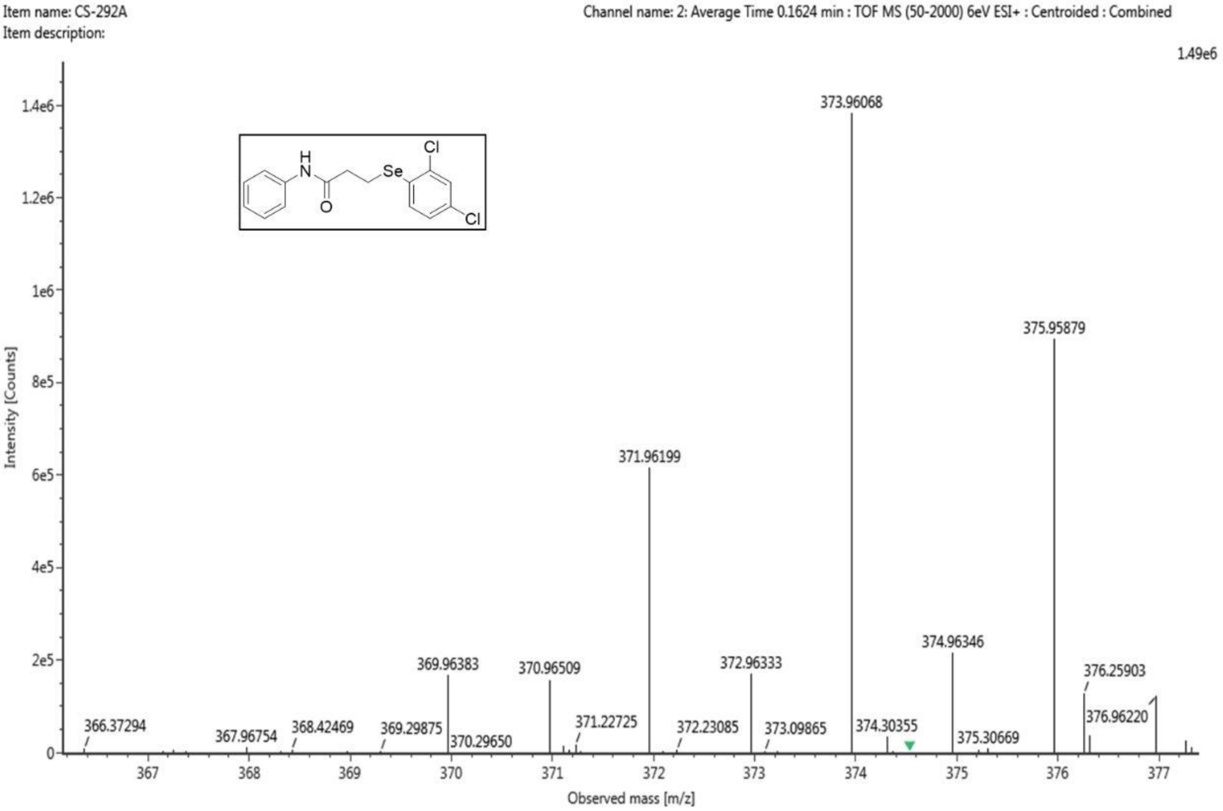

Supplementary Fig. 88. HR-MS of compound **4o**

^{1}H NMR (400 MHz, $CDCl_3$, 25°C) of compound **4p**

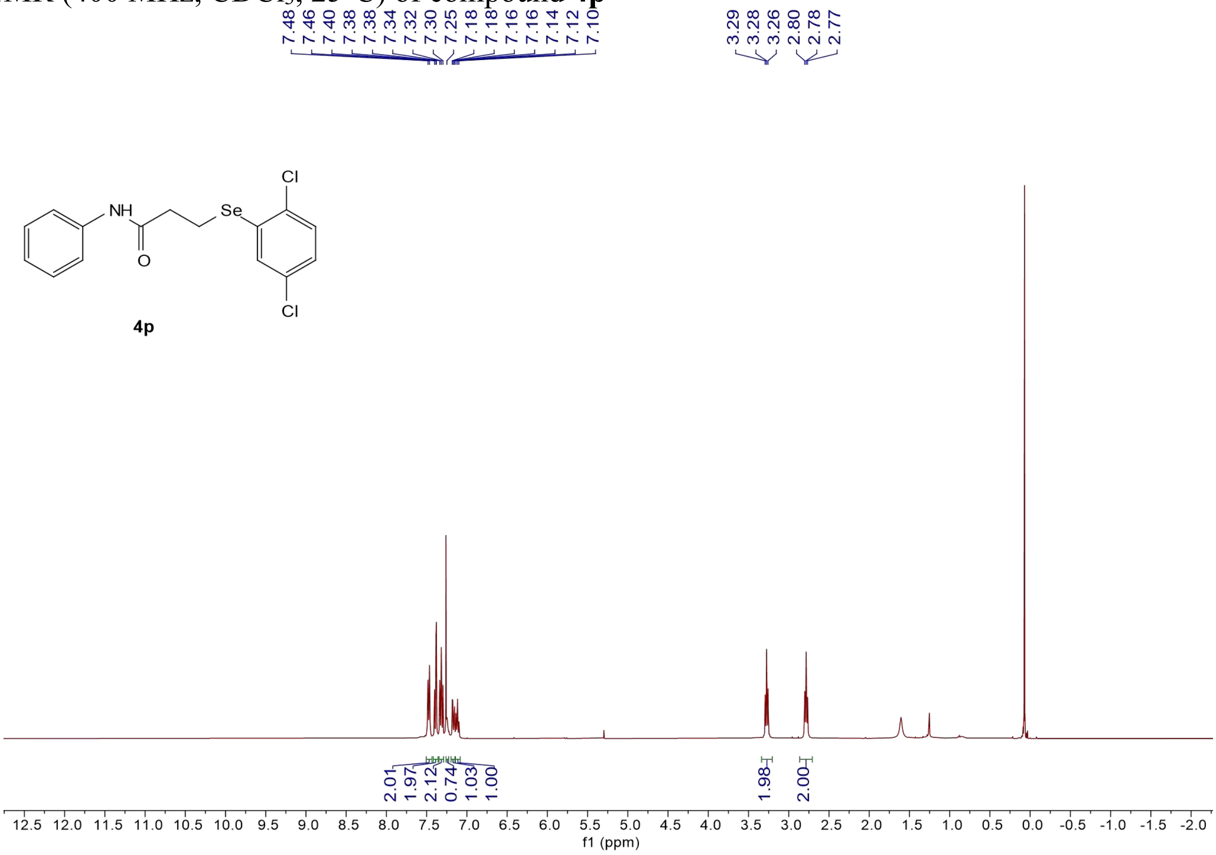

^{13}C NMR (101 MHz, $CDCl_3$, 25°C) of compound **4p**

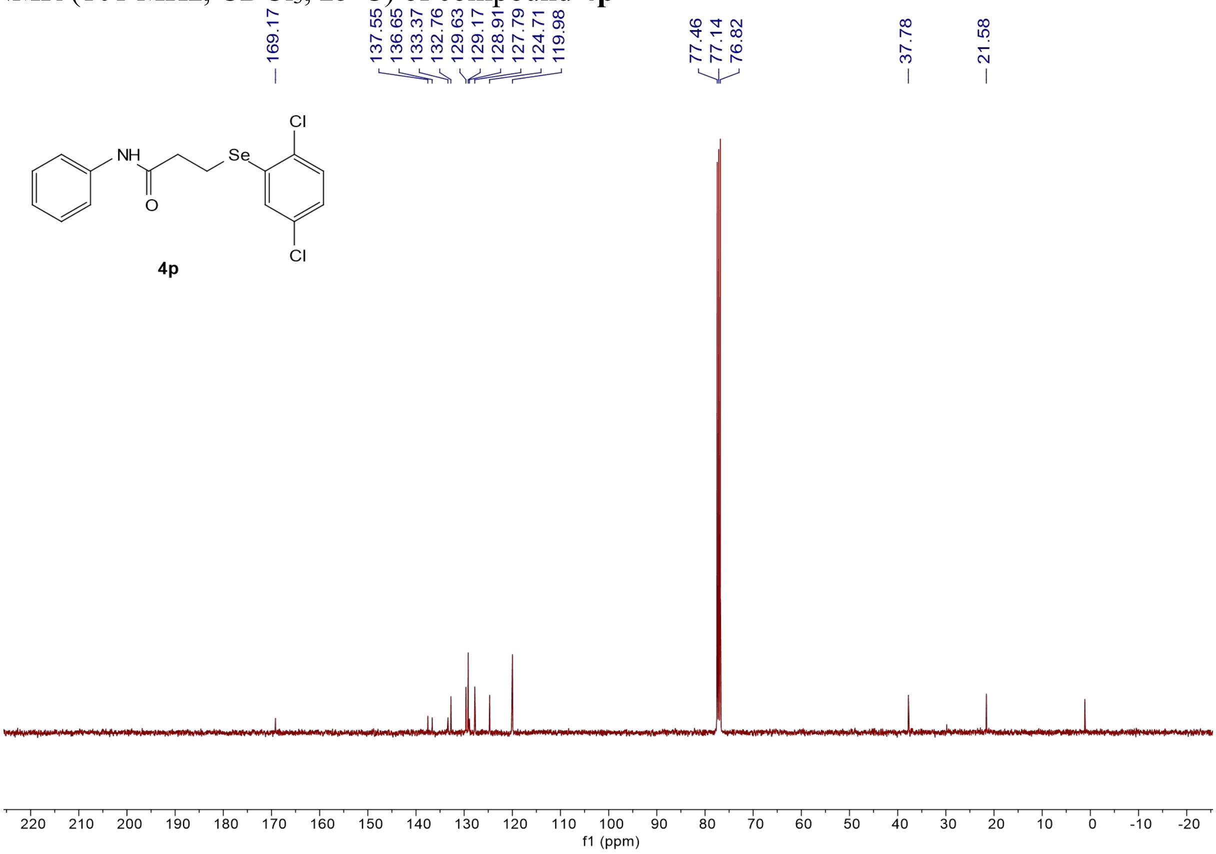

^{77}Se NMR (76 MHz, $CDCl_3$, 25°C) of compound **4p**

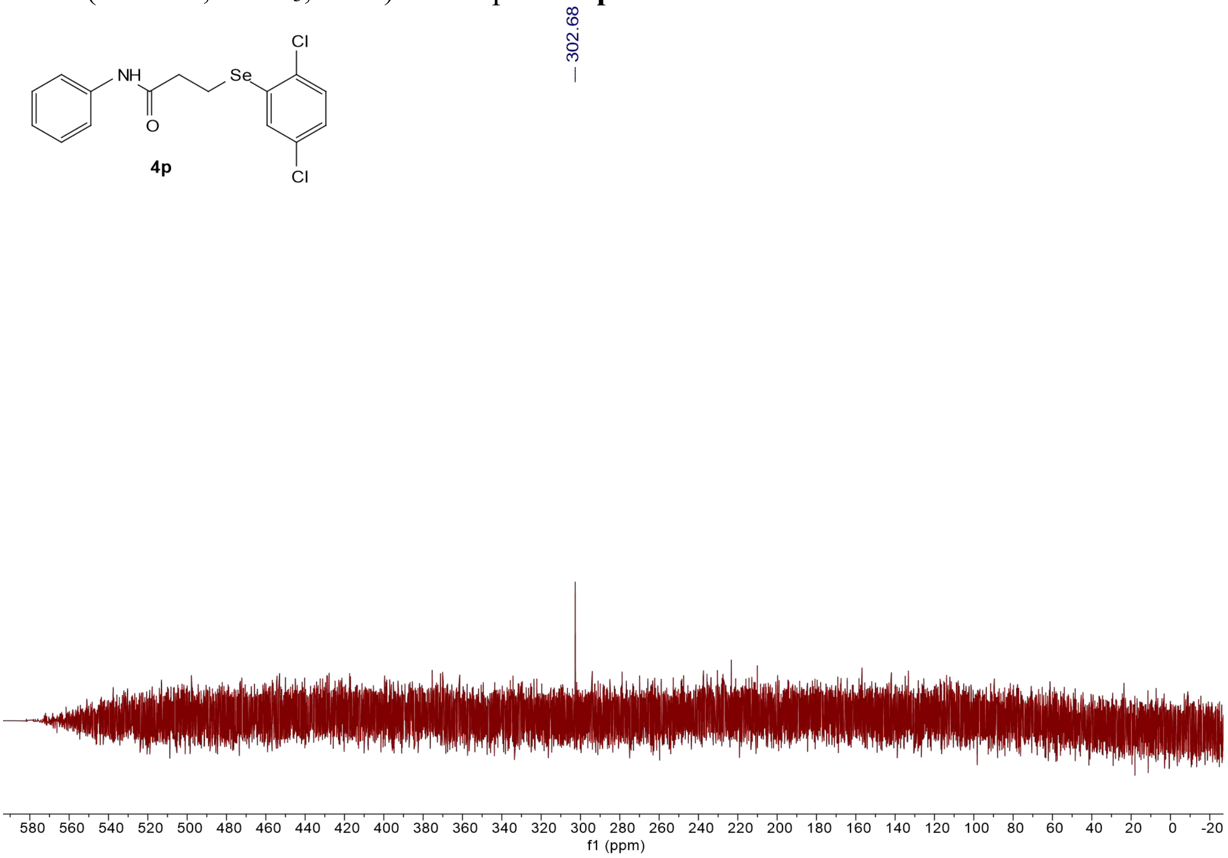

Supplementary Fig. 89. NMR spectra of compound **4p**

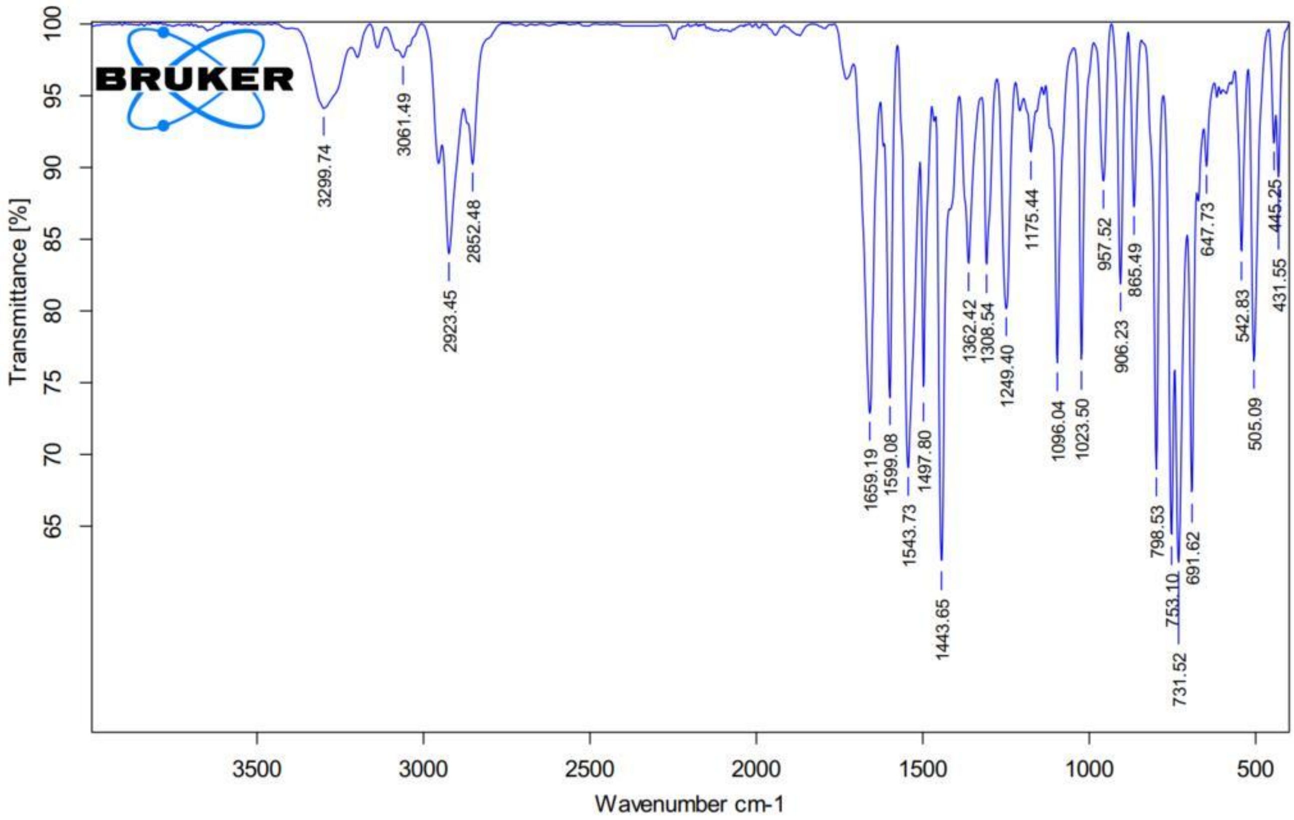

Supplementary Fig. 90. IR of compound **4p**

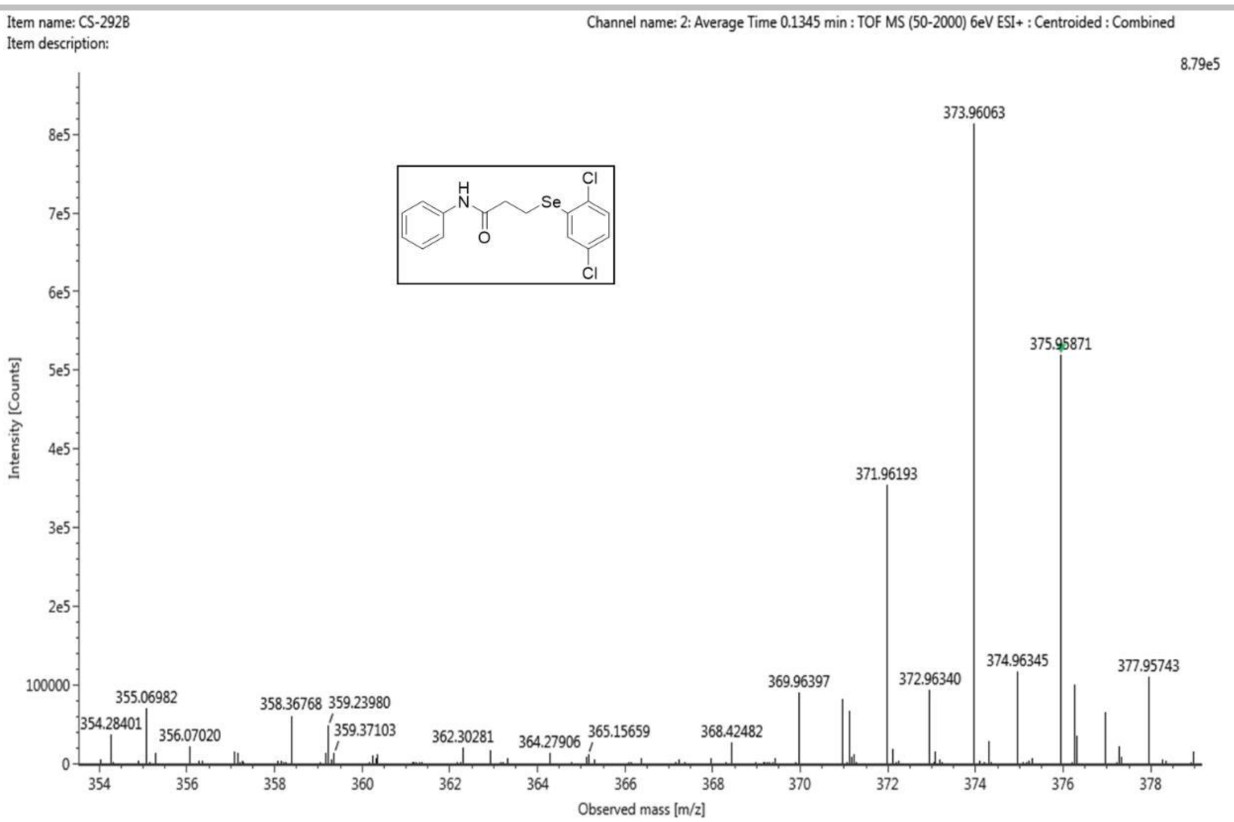

Supplementary Fig. 91. HR-MS of compound **4p**

^{1}H NMR (400 MHz, $CDCl_3$, 25°C) of compound **4q**

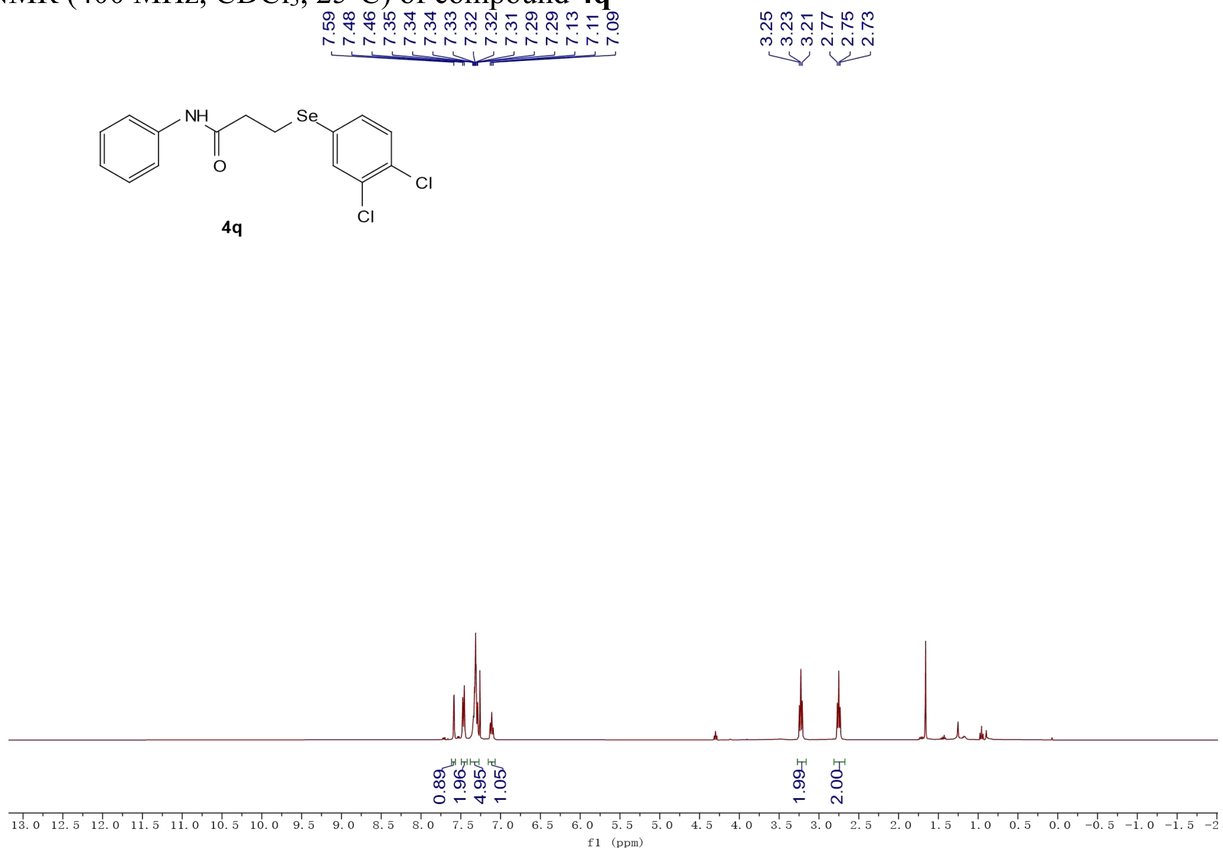

^{13}C NMR (101 MHz, $CDCl_3$, 25°C) of compound **4q**

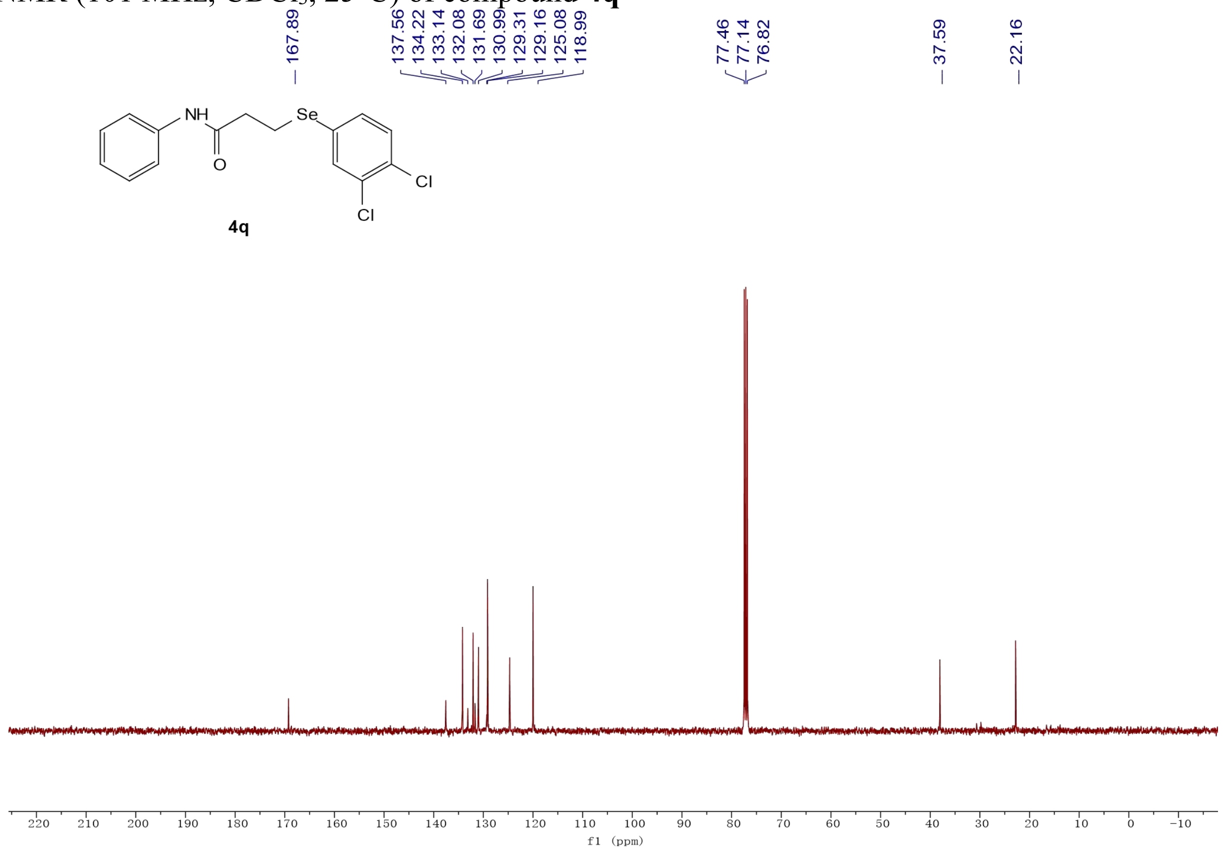

^{77}Se NMR (76 MHz, $CDCl_3$, 25°C) of compound **4q**

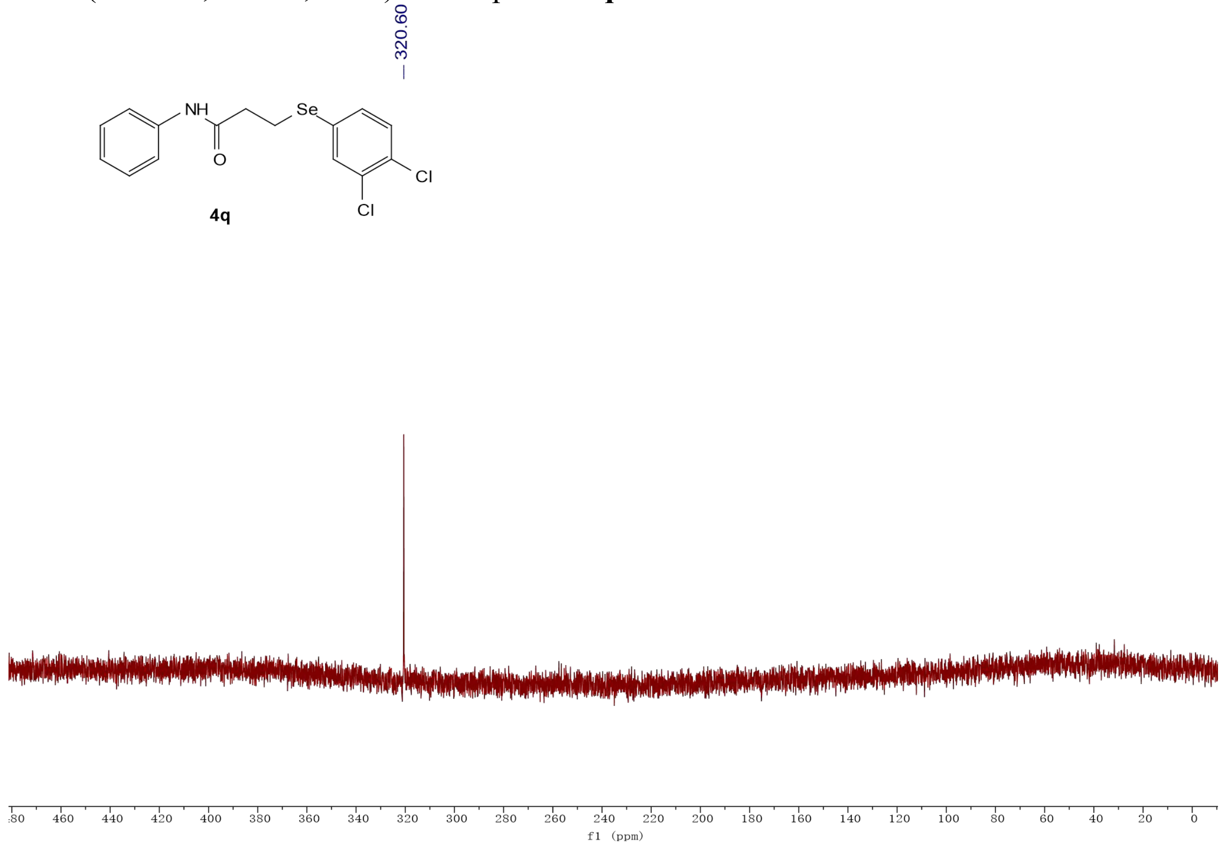

Supplementary Fig. 92. NMR spectra of compound **4q**

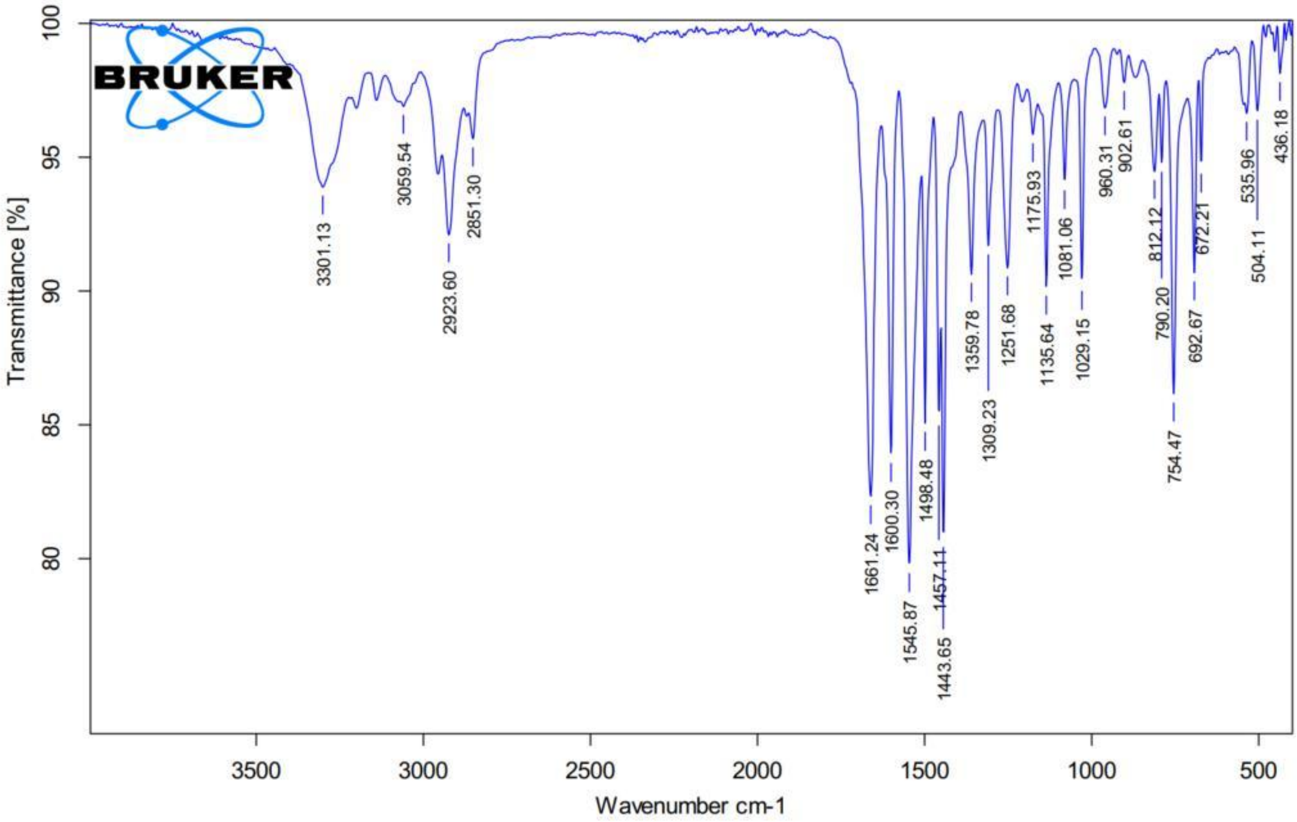

Supplementary Fig. 93. IR of compound **4q**

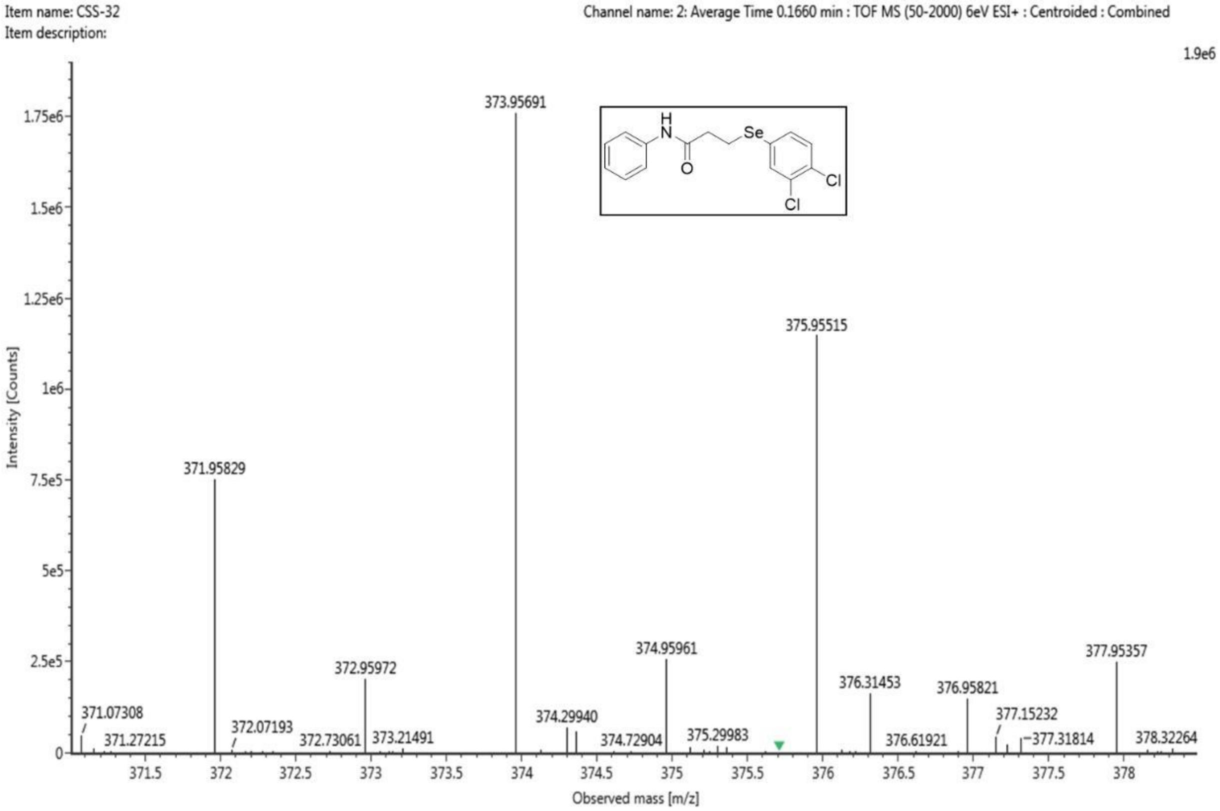

Supplementary Fig. 94. HR-MS of compound **4q**

^{1}H NMR (400 MHz, $CDCl_3$, 25°C) of compound **4r**

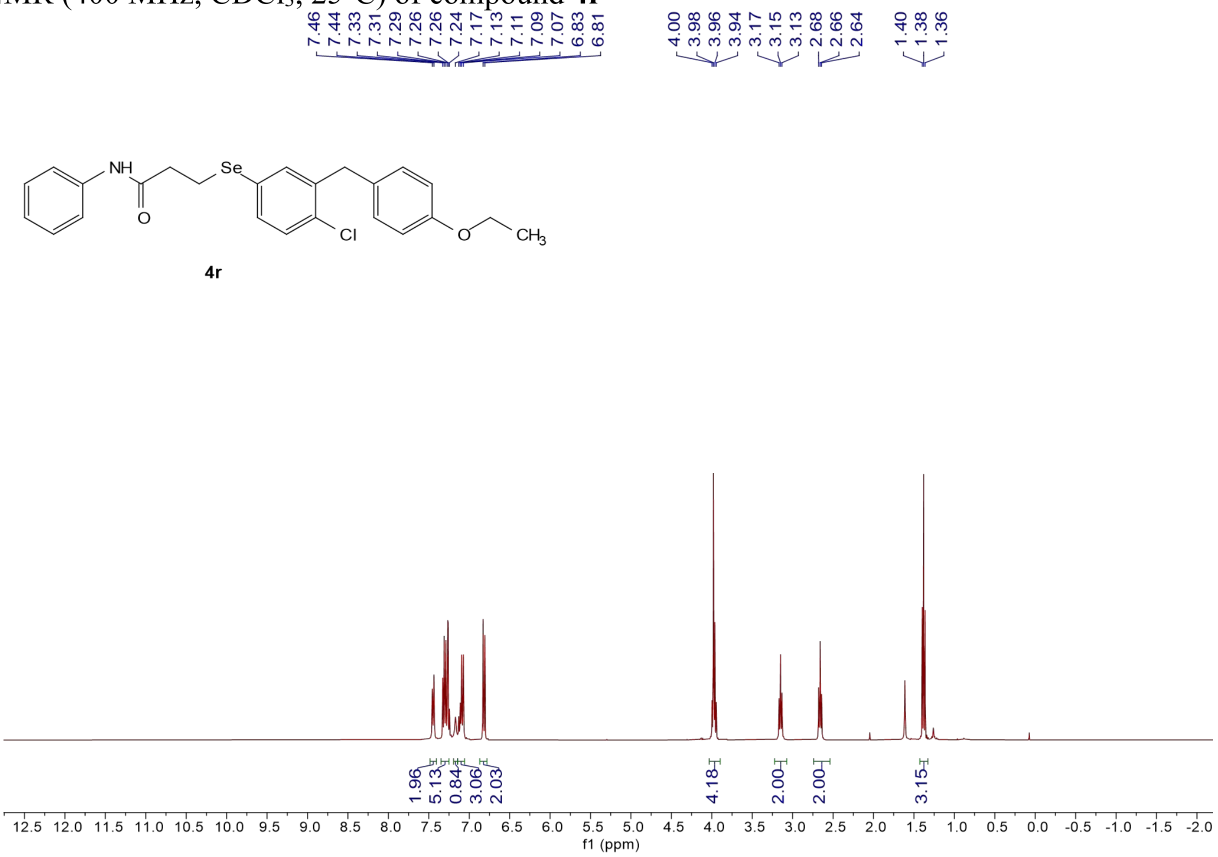

^{13}C NMR (101 MHz, $CDCl_3$, 25°C) of compound **4r**

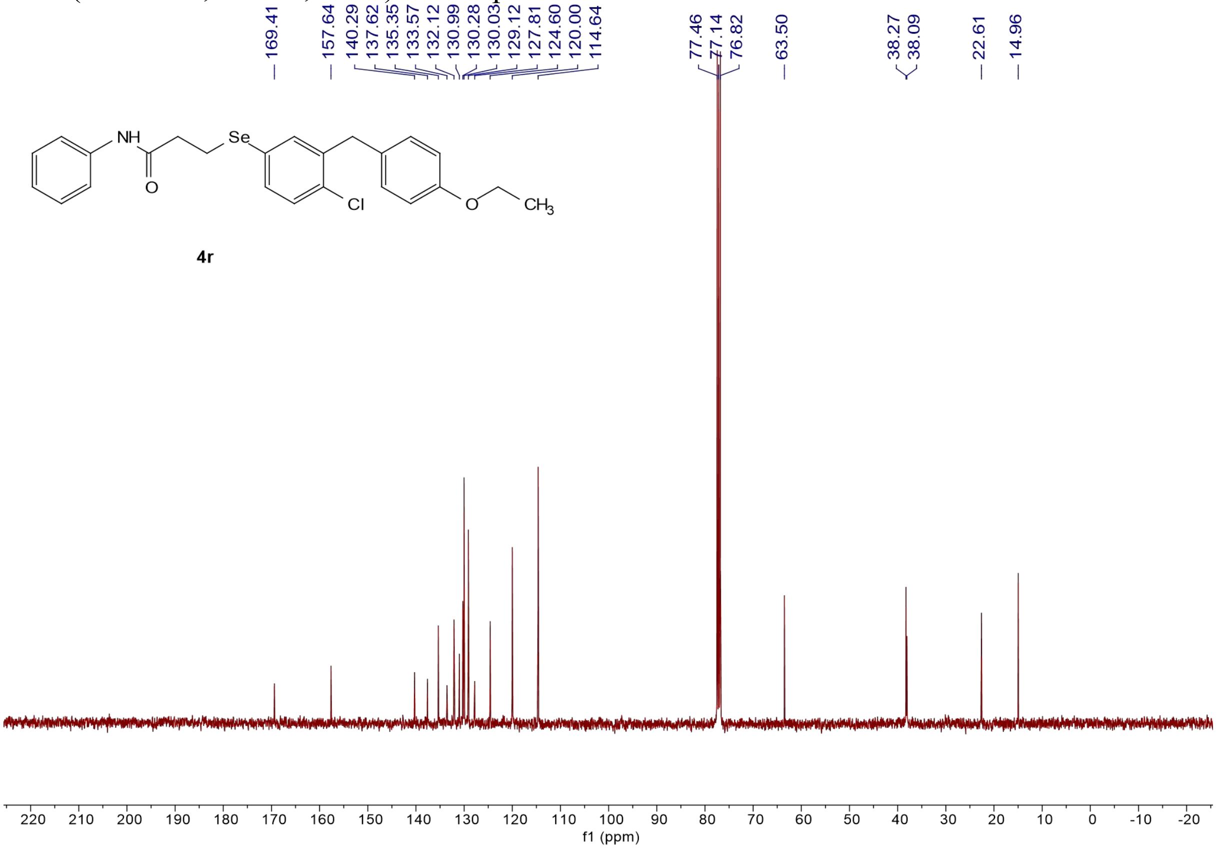

^{77}Se NMR (76 MHz, $CDCl_3$, 25°C) of compound **4r**

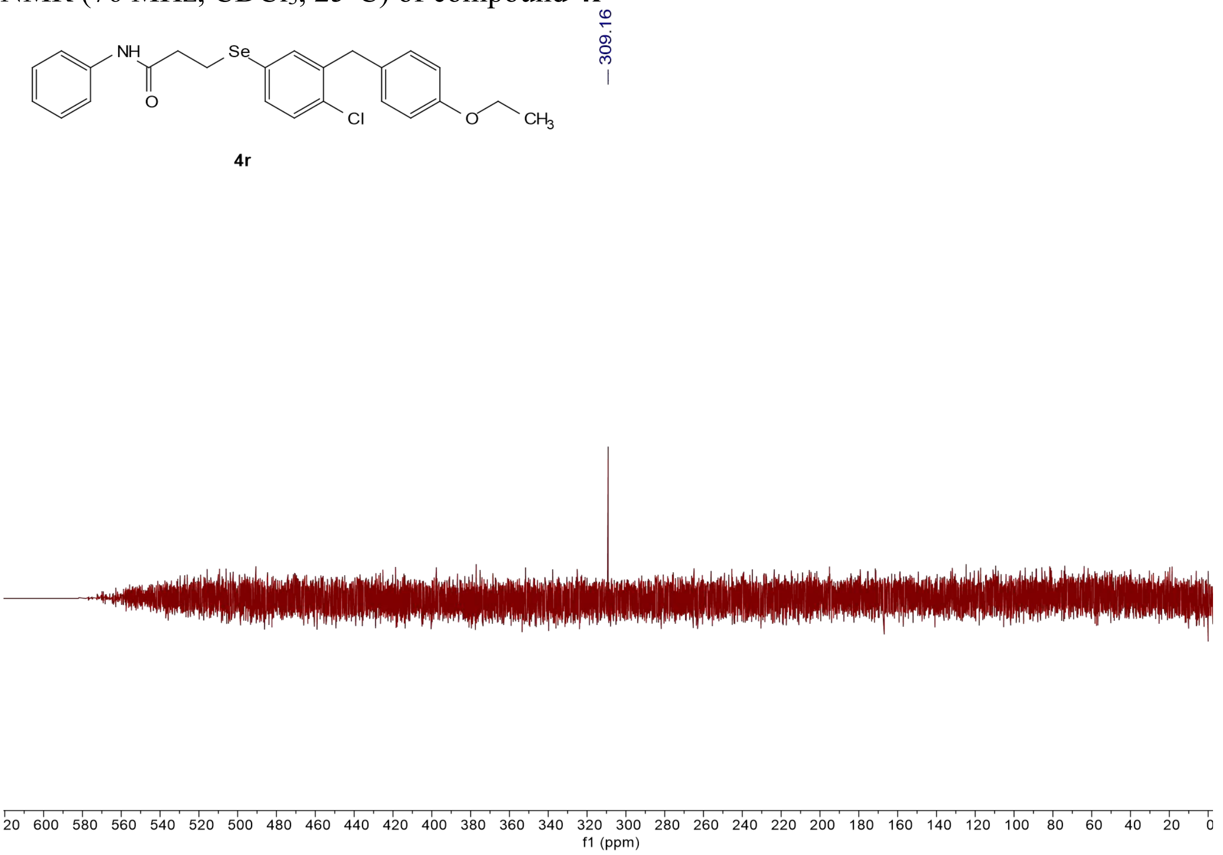

Supplementary Fig. 95. NMR spectra of compound **4r**

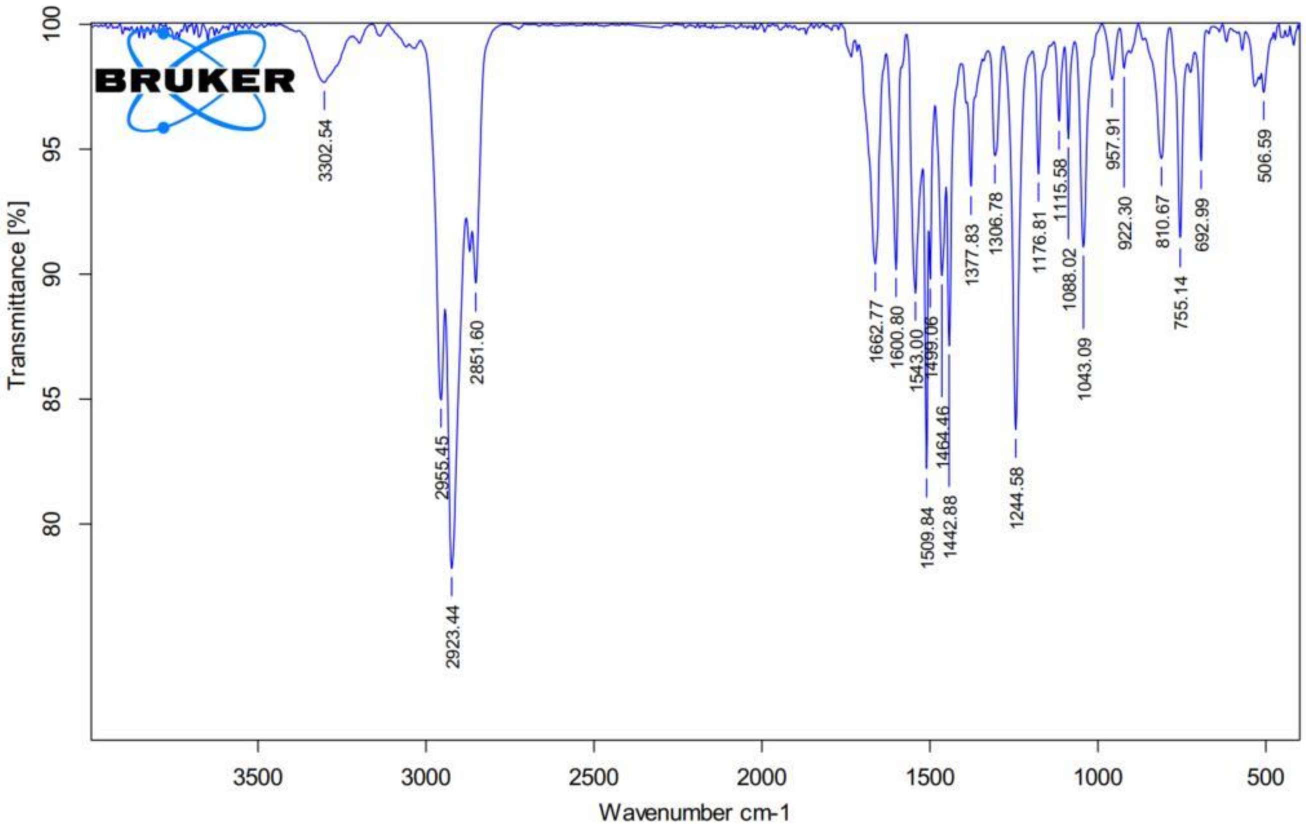

Supplementary Fig. 96. IR of compound **4r**

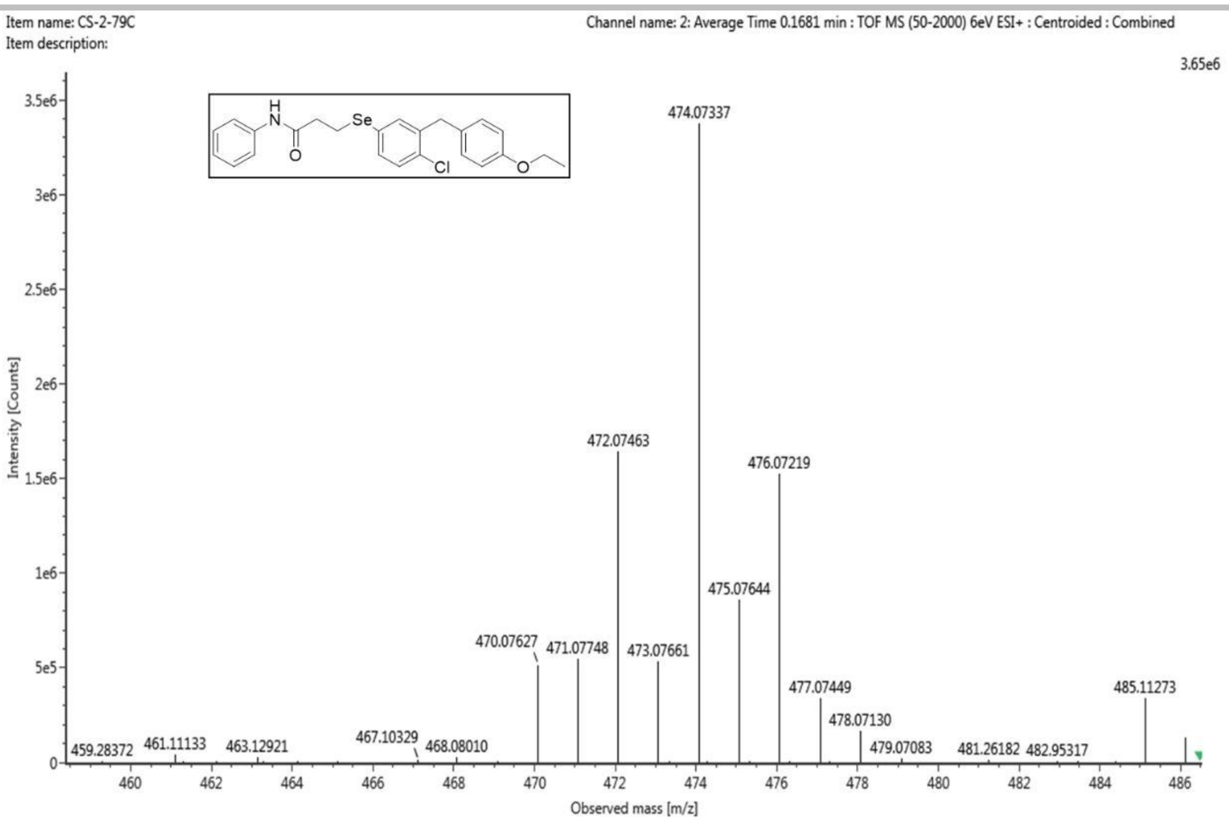

Supplementary Fig. 97. HR-MS of compound **4r**

^{1}H NMR (400 MHz, $CDCl_3$, 25°C) of compound **4s**

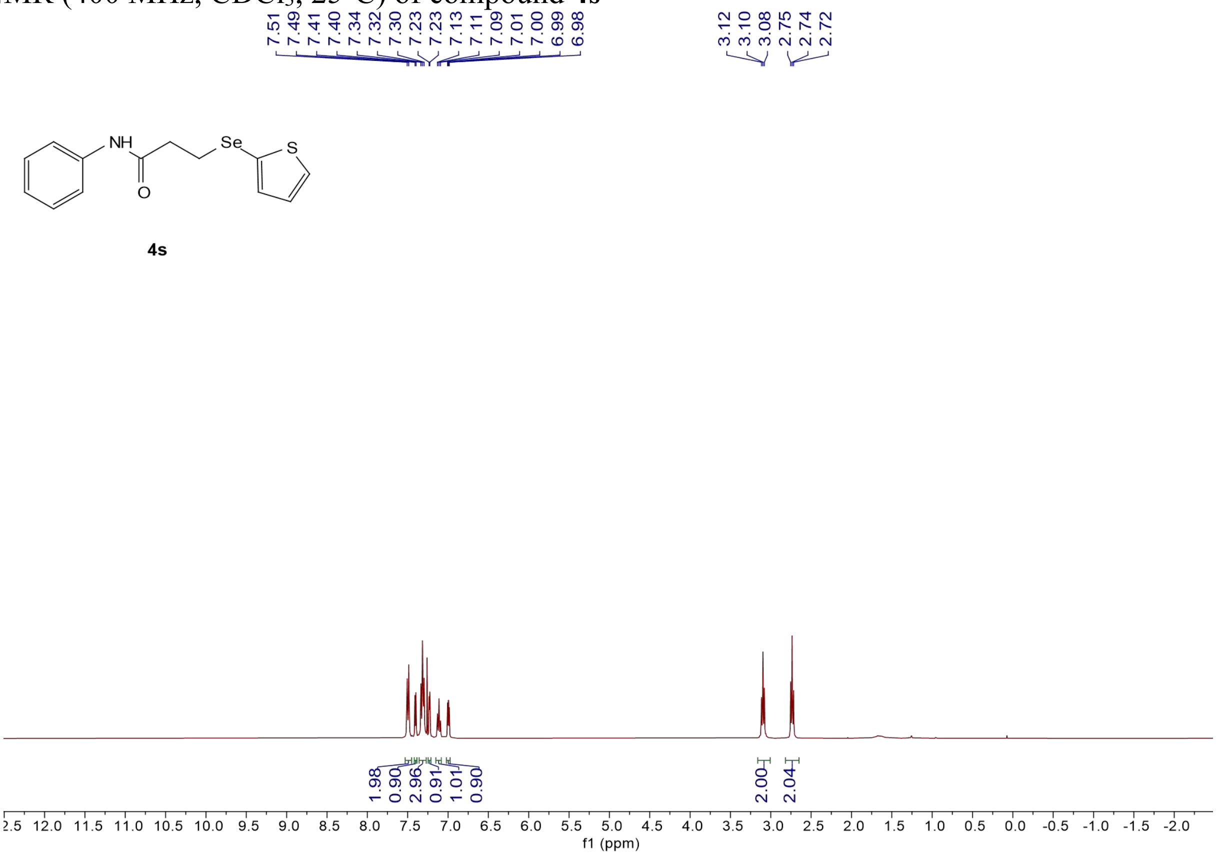

^{13}C NMR (101 MHz, $CDCl_3$, 25°C) of compound **4s**

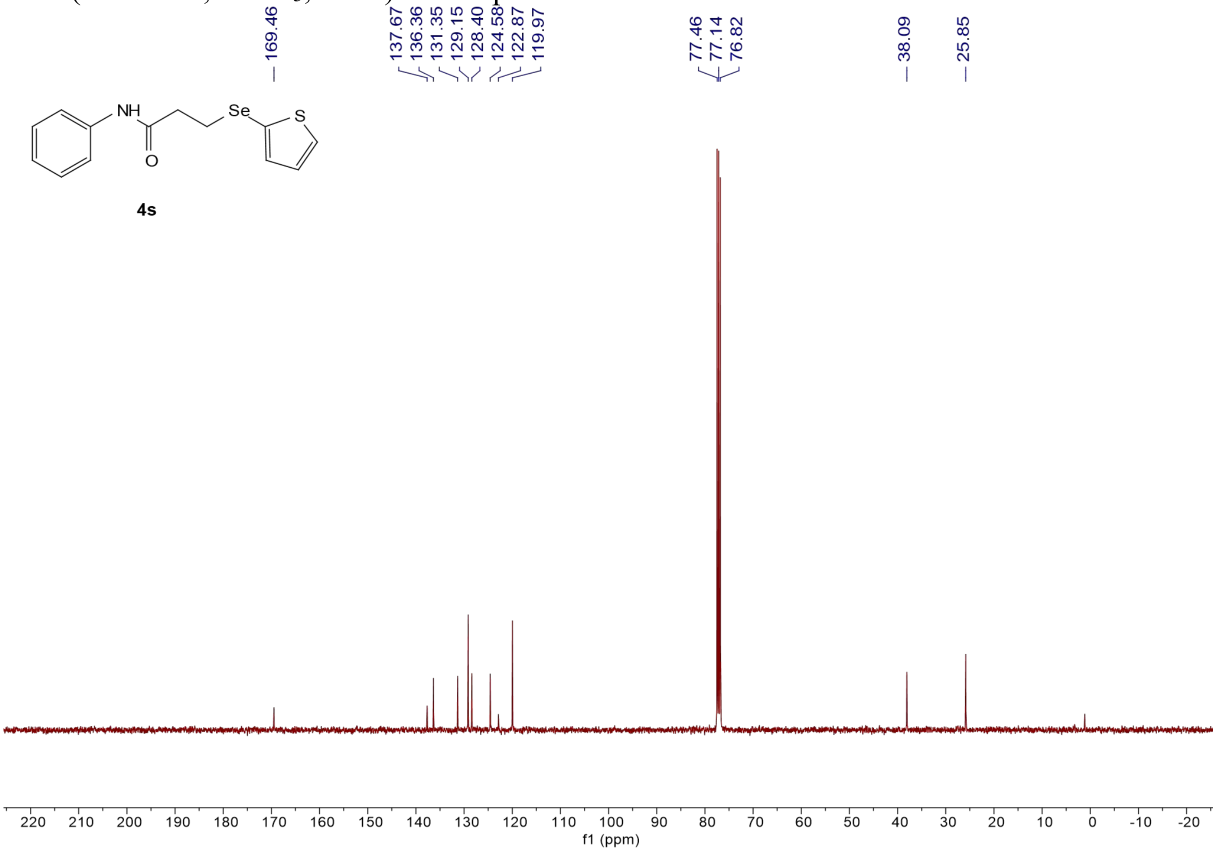

^{77}Se NMR (76 MHz, $CDCl_3$, 25°C) of compound **4s**

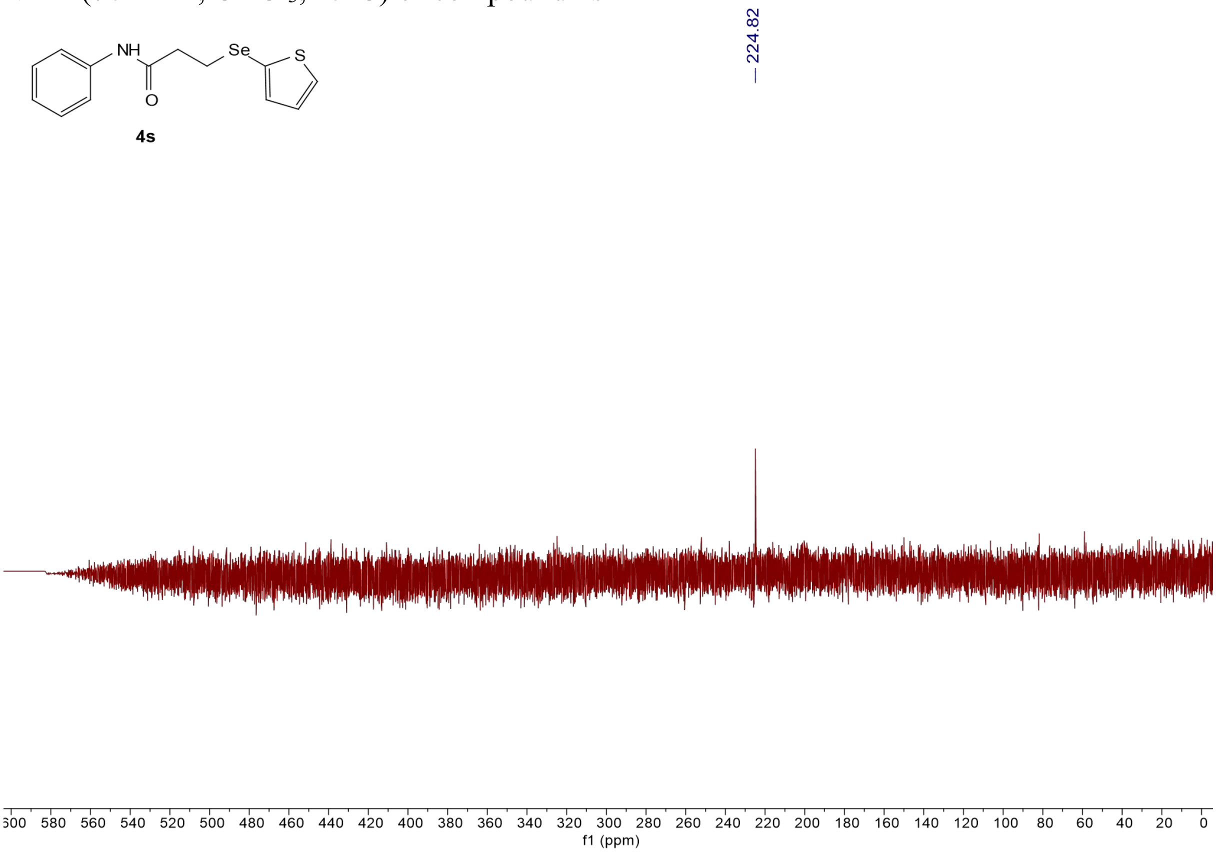

Supplementary Fig. 98. NMR spectra of compound **4s**

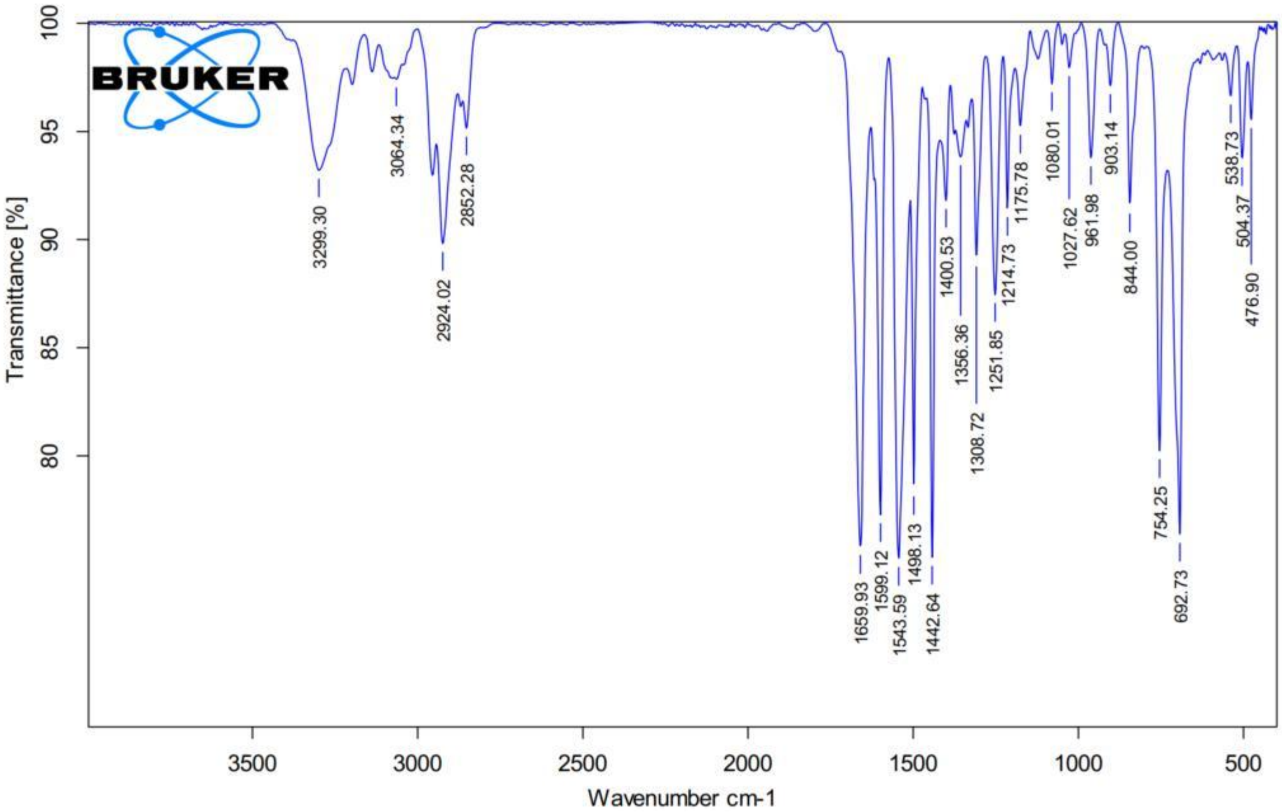

Supplementary Fig. 99. IR of compound **4s**

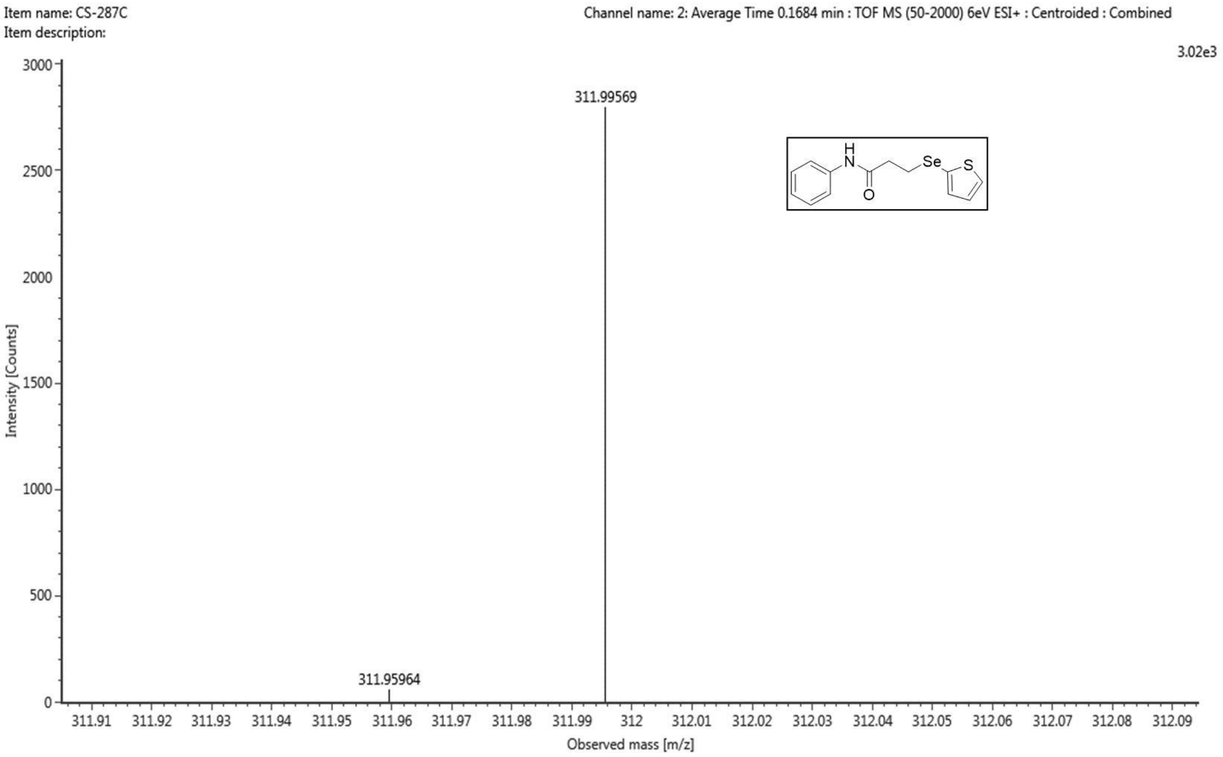

Supplementary Fig. 100. HR-MS of compound **4s**

^{1}H NMR (400 MHz, $CDCl_3$, 25℃) of compound **4t**

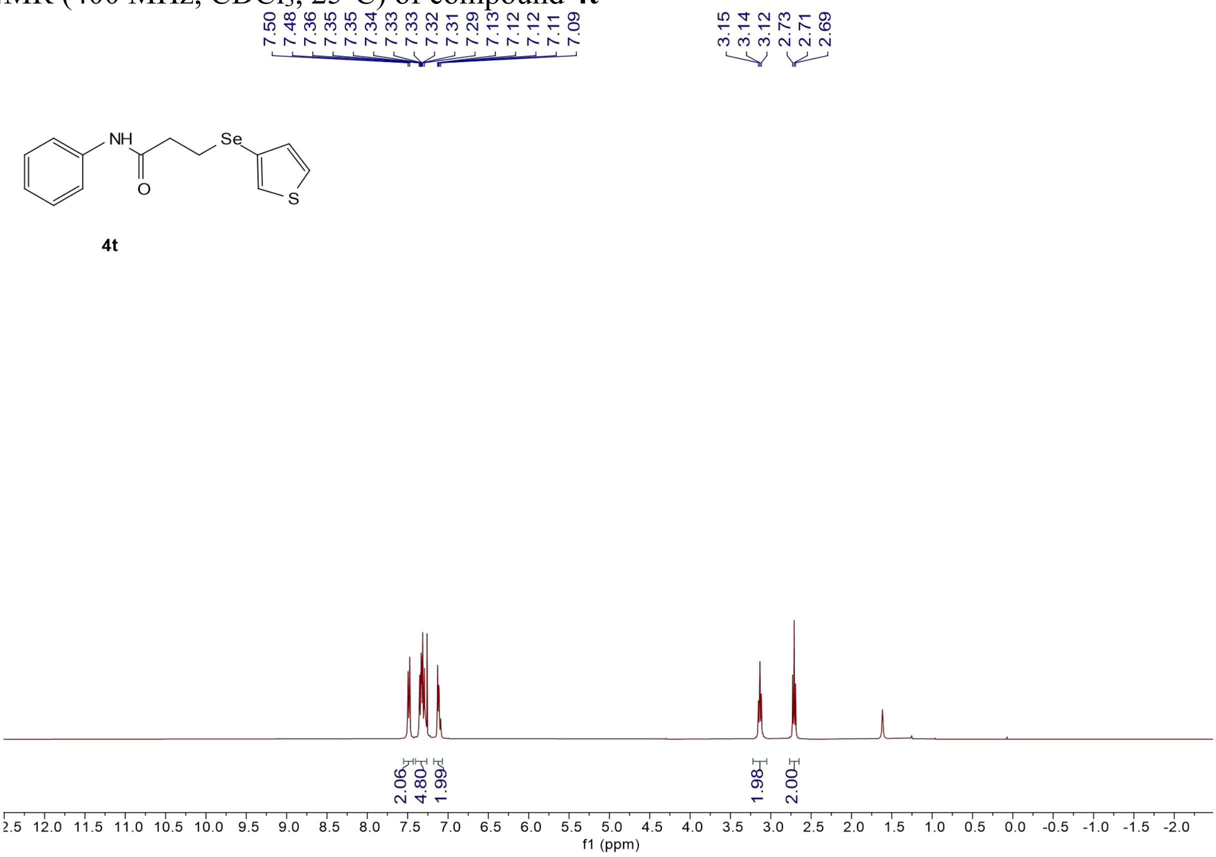

^{13}C NMR (101 MHz, $CDCl_3$, 25℃) of compound **4t**

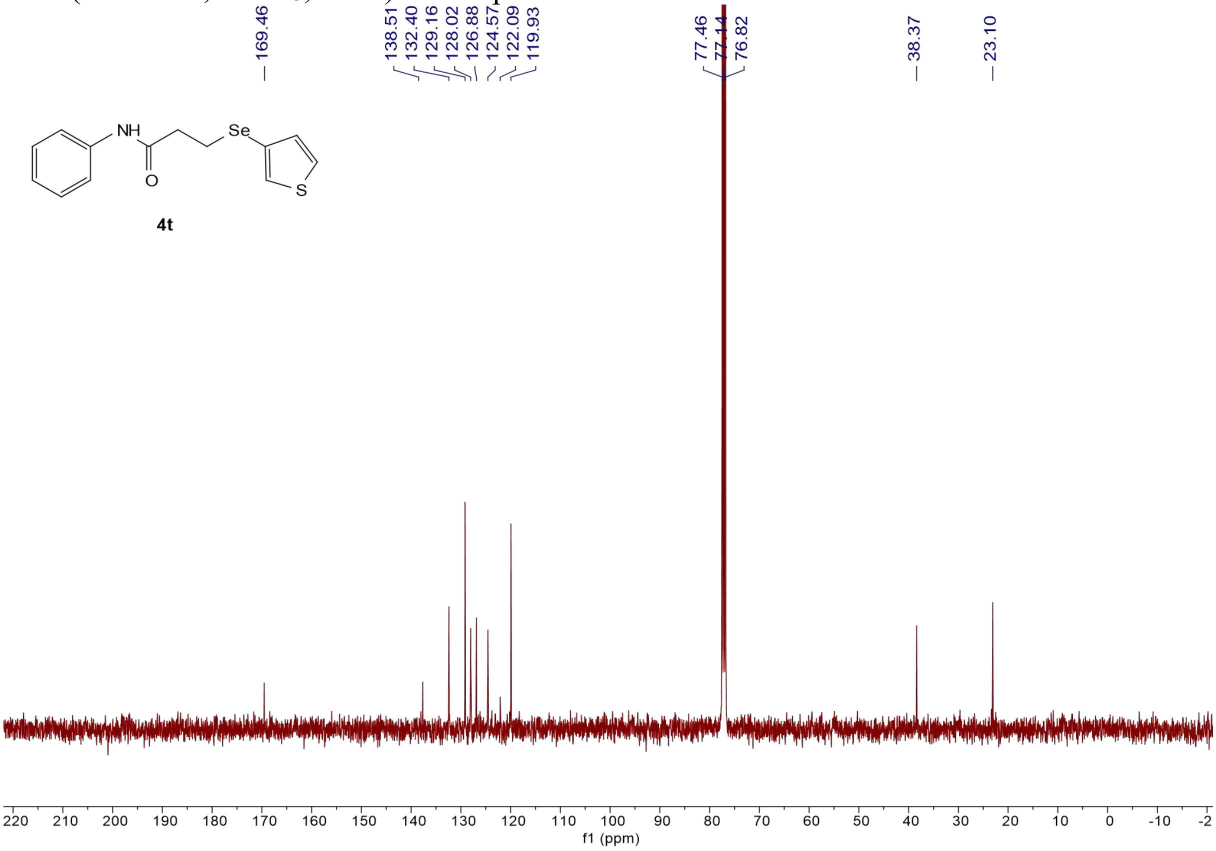

^{77}Se NMR (76 MHz, $CDCl_3$, 25°C) of compound **4t**

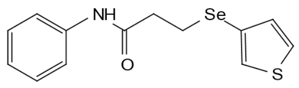

4t

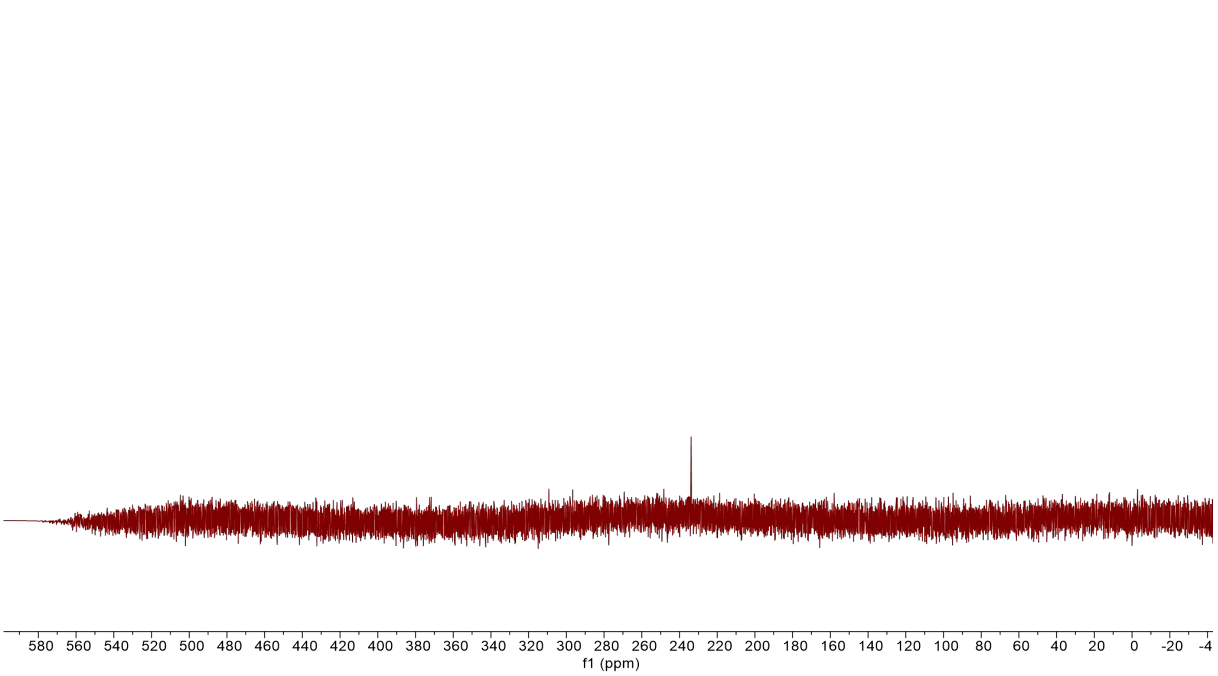

Supplementary Fig. 101. NMR spectra of compound **4t**

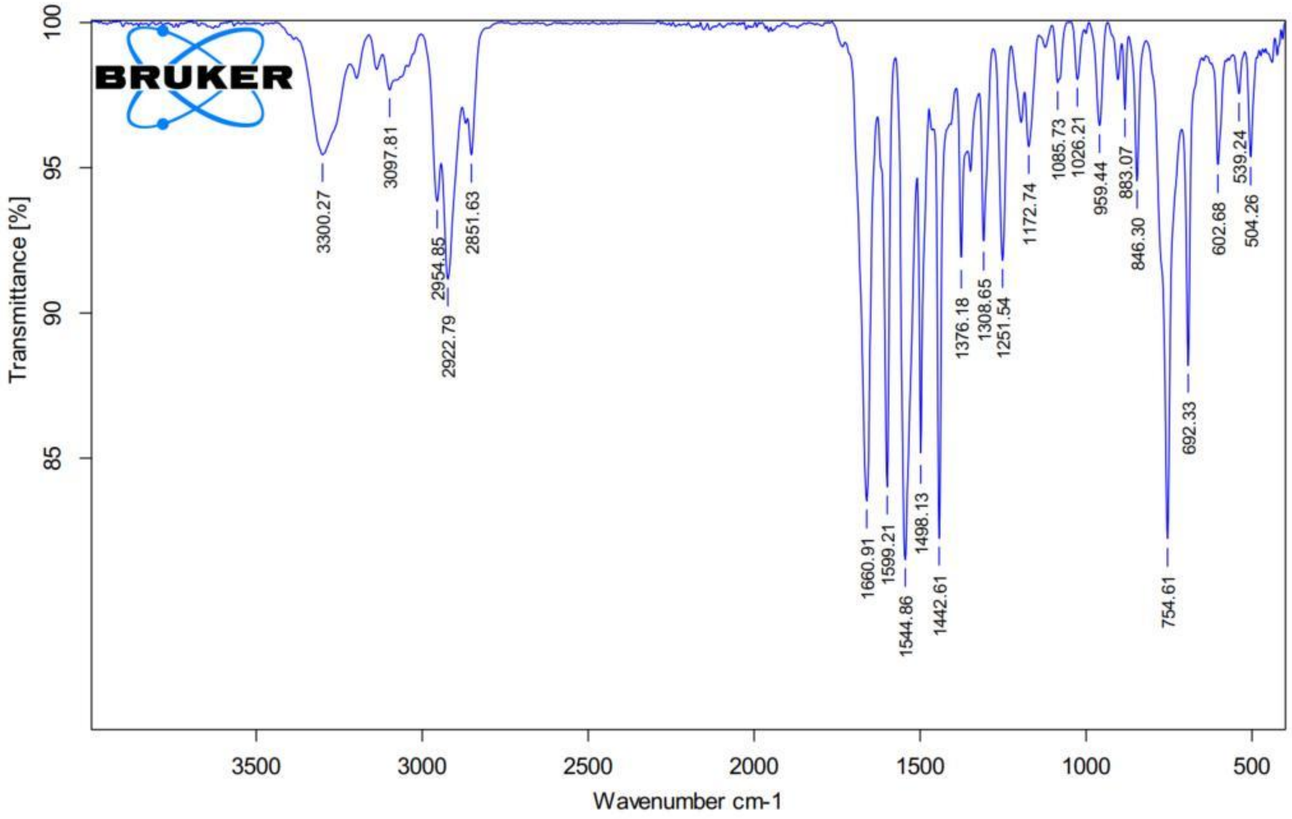

Supplementary Fig. 102. IR of compound **4t**

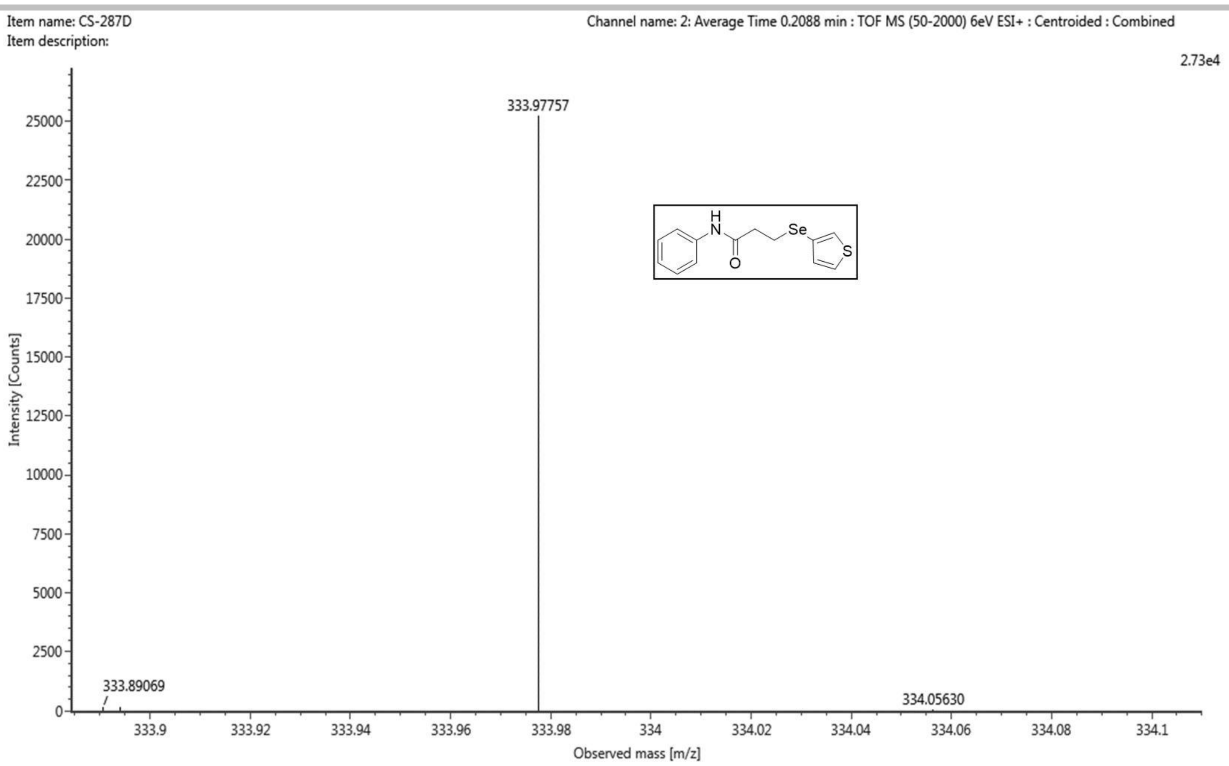

Supplementary Fig. 103. HR-MS of compound **4t**

^{1}H NMR (400 MHz, $CDCl_3$, 25°C) of compound **4u**

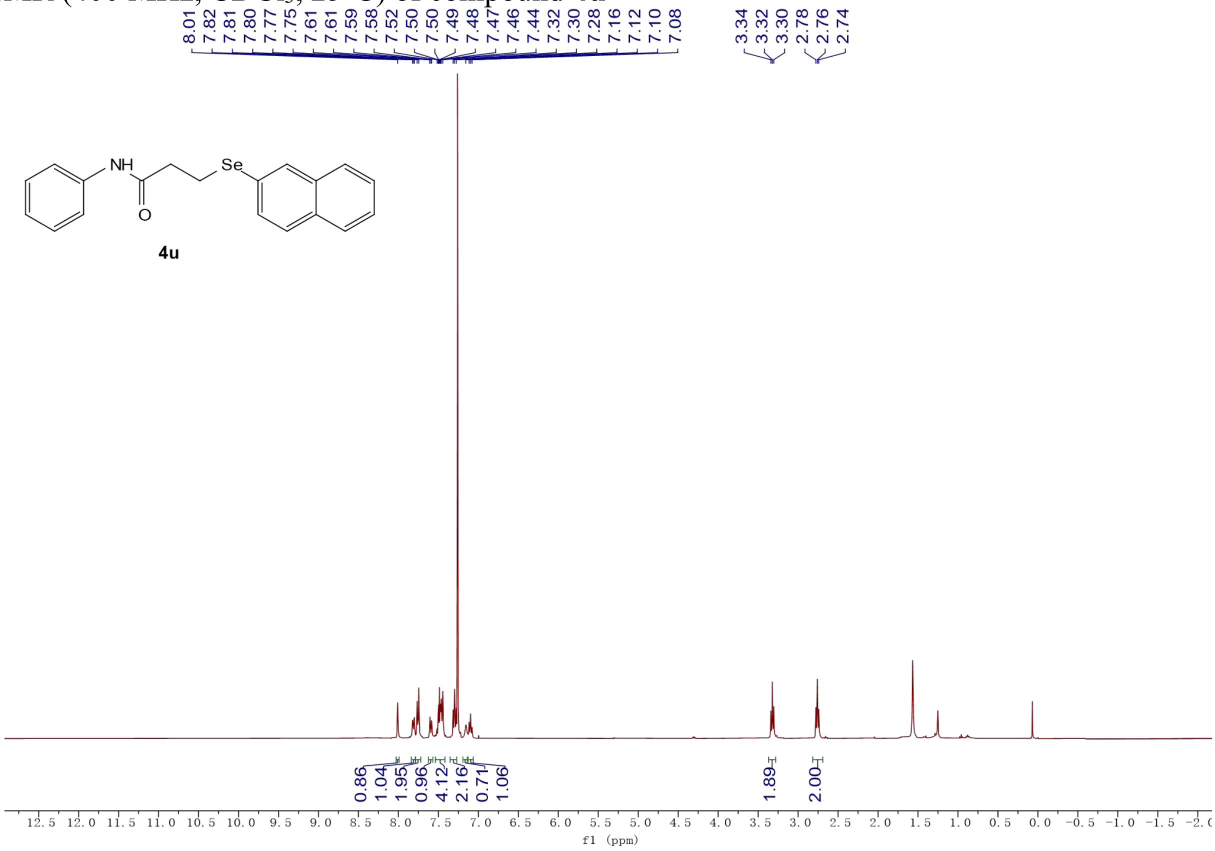

^{13}C NMR (101 MHz, $CDCl_3$, 25°C) of compound **4u**

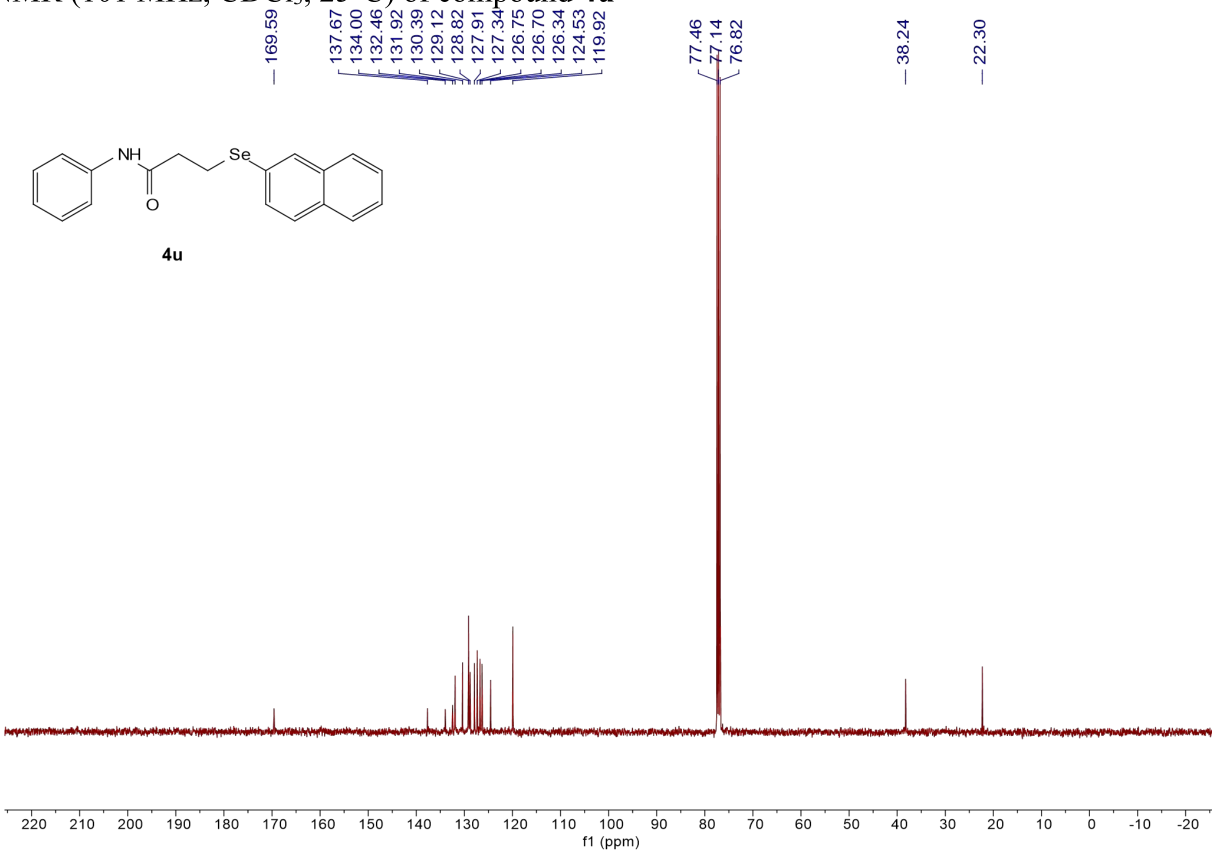

^{77}Se NMR (76 MHz, $CDCl_3$, 25°C) of compound **4u**

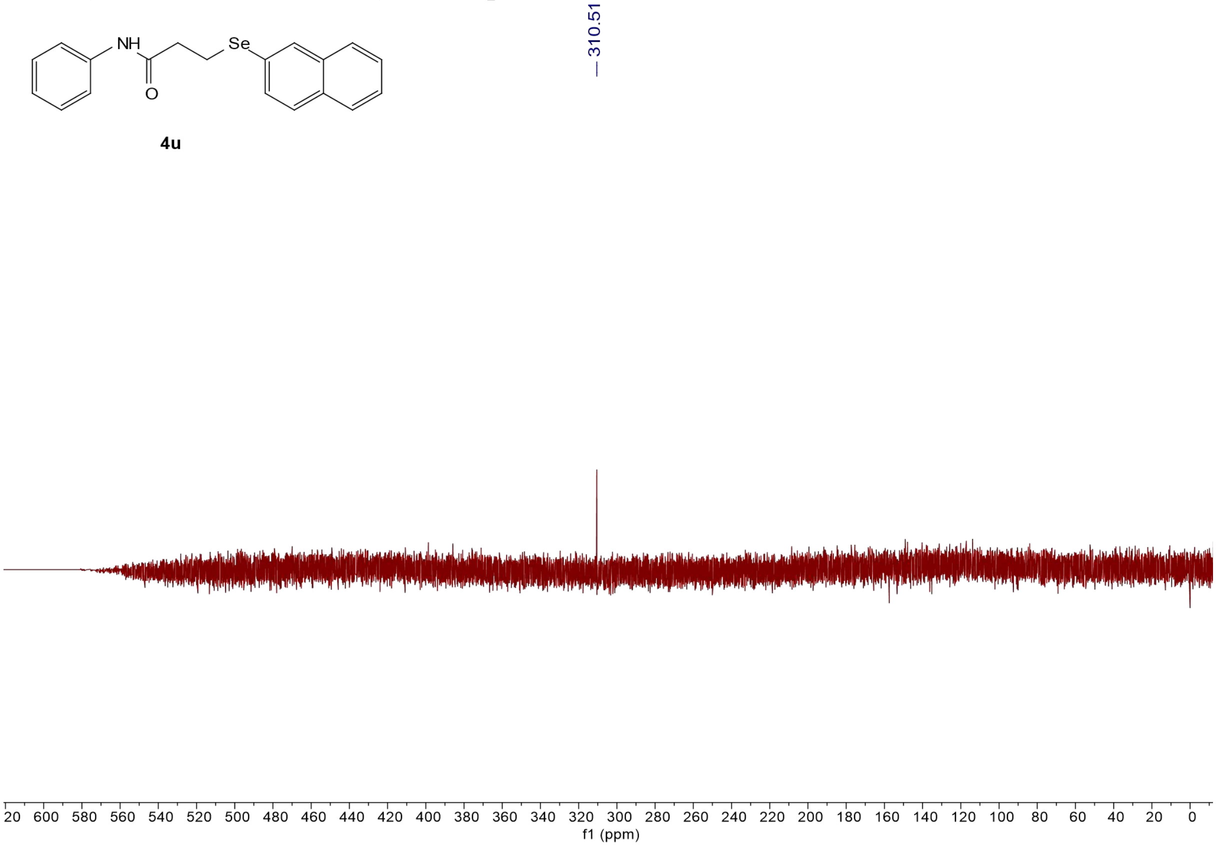

Supplementary Fig. 104. NMR spectra of compound **4u**

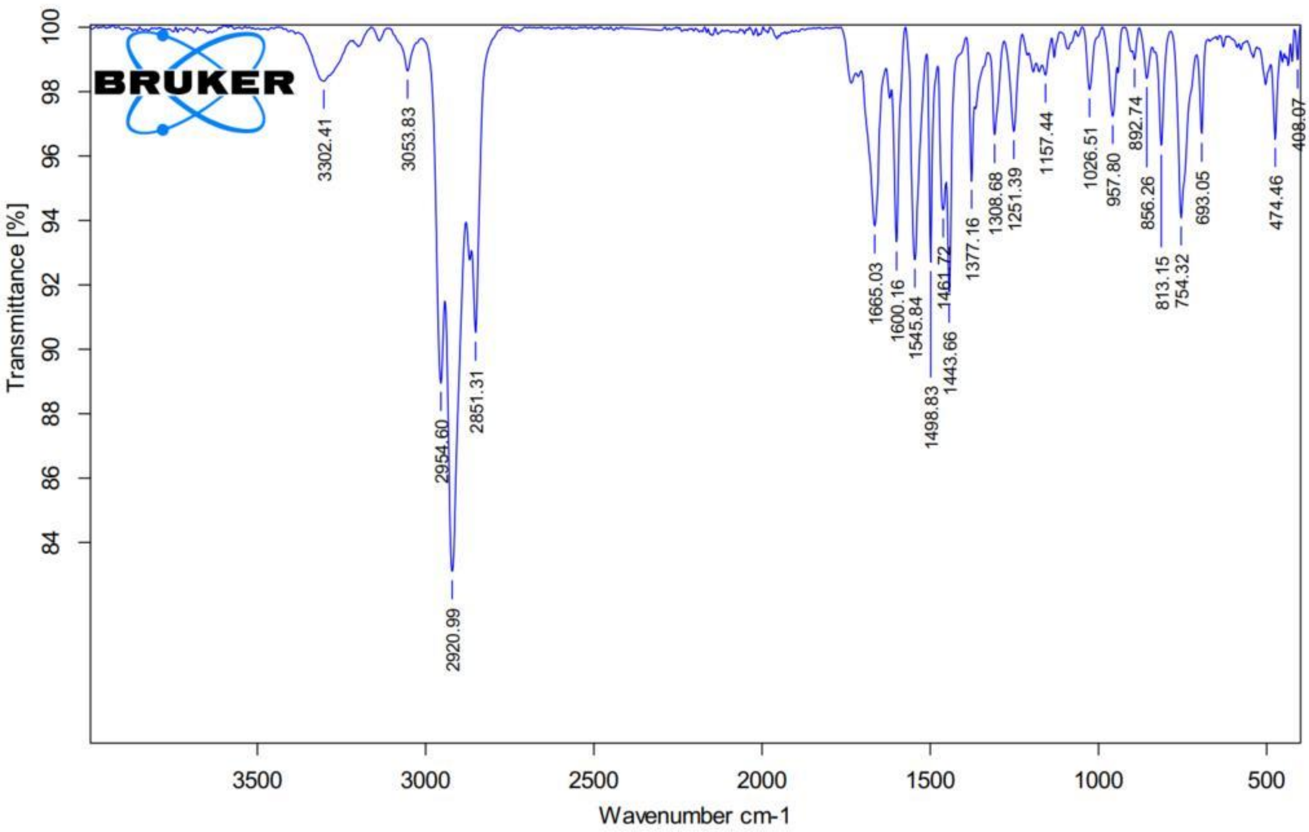

Supplementary Fig. 105. IR of compound **4u**

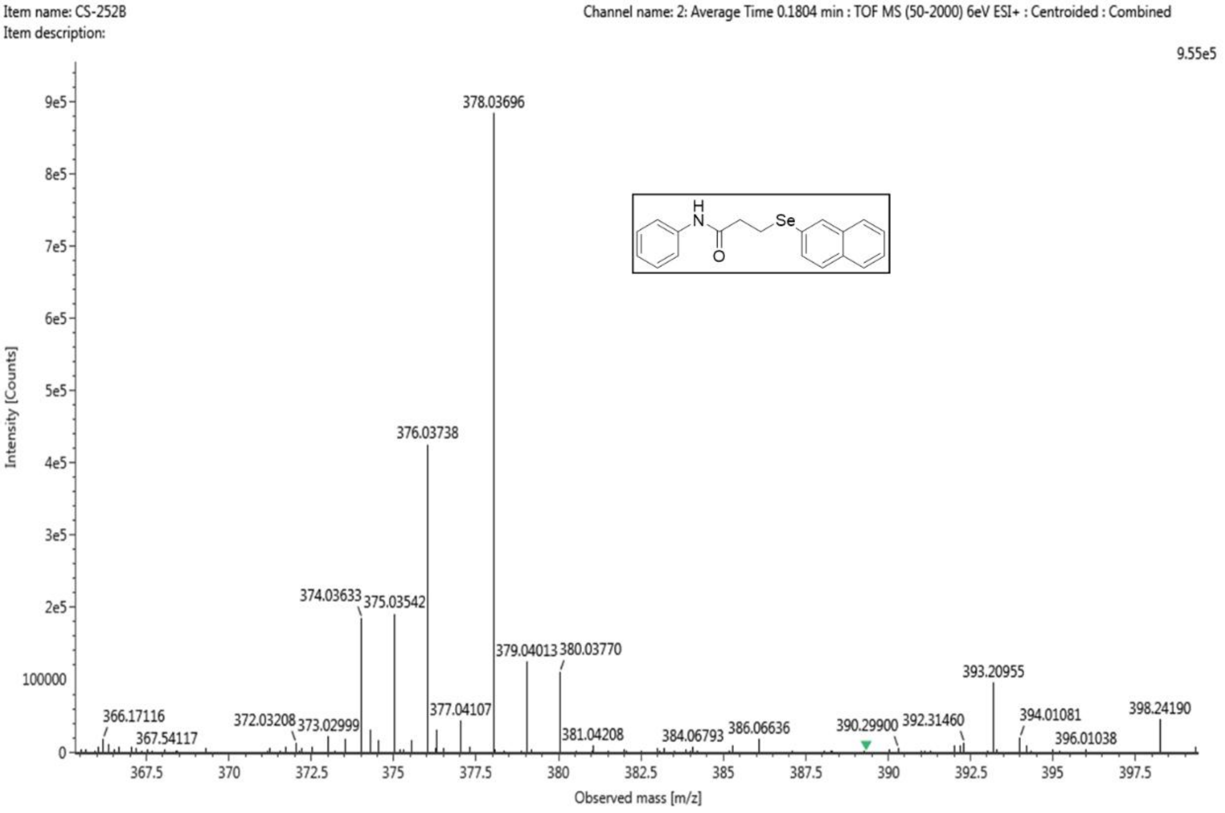

Supplementary Fig.106. HR-MS of compound **4u**

^{1}H NMR (400 MHz, $CDCl_3$, 25°C) of compound **4v**

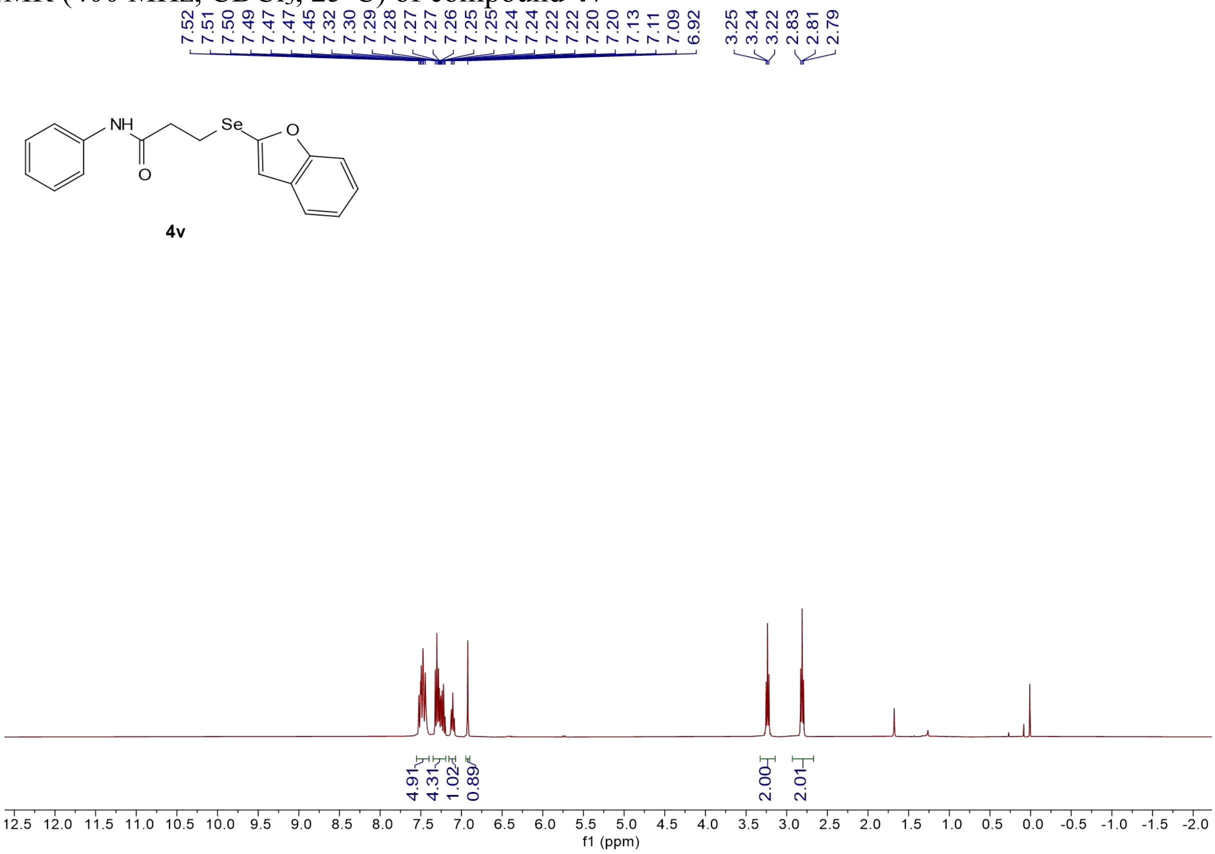

^{13}C NMR (101 MHz, $CDCl_3$, 25°C) of compound **4v**

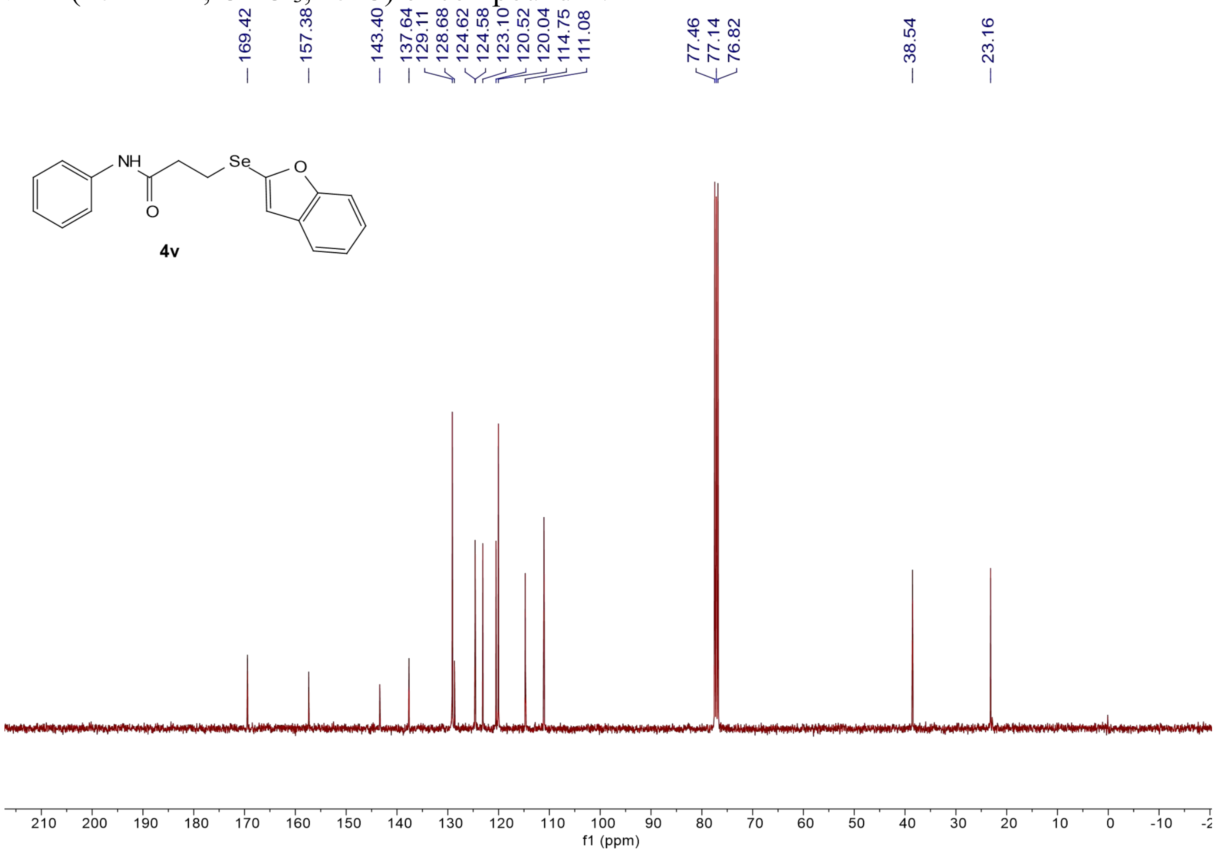

^{77}Se NMR (76 MHz, $CDCl_3$, 25°C) of compound **4v**

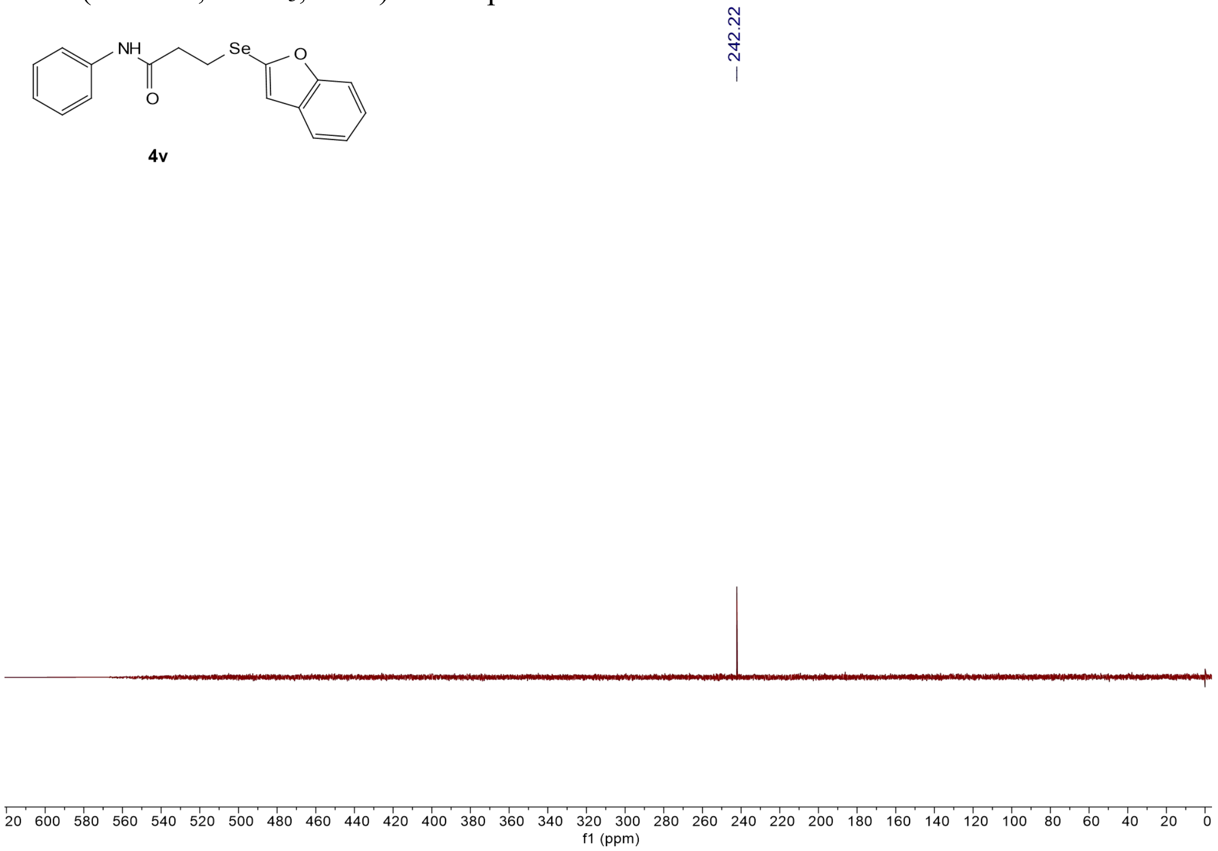

Supplementary Fig. 107. NMR spectra of compound **4v**

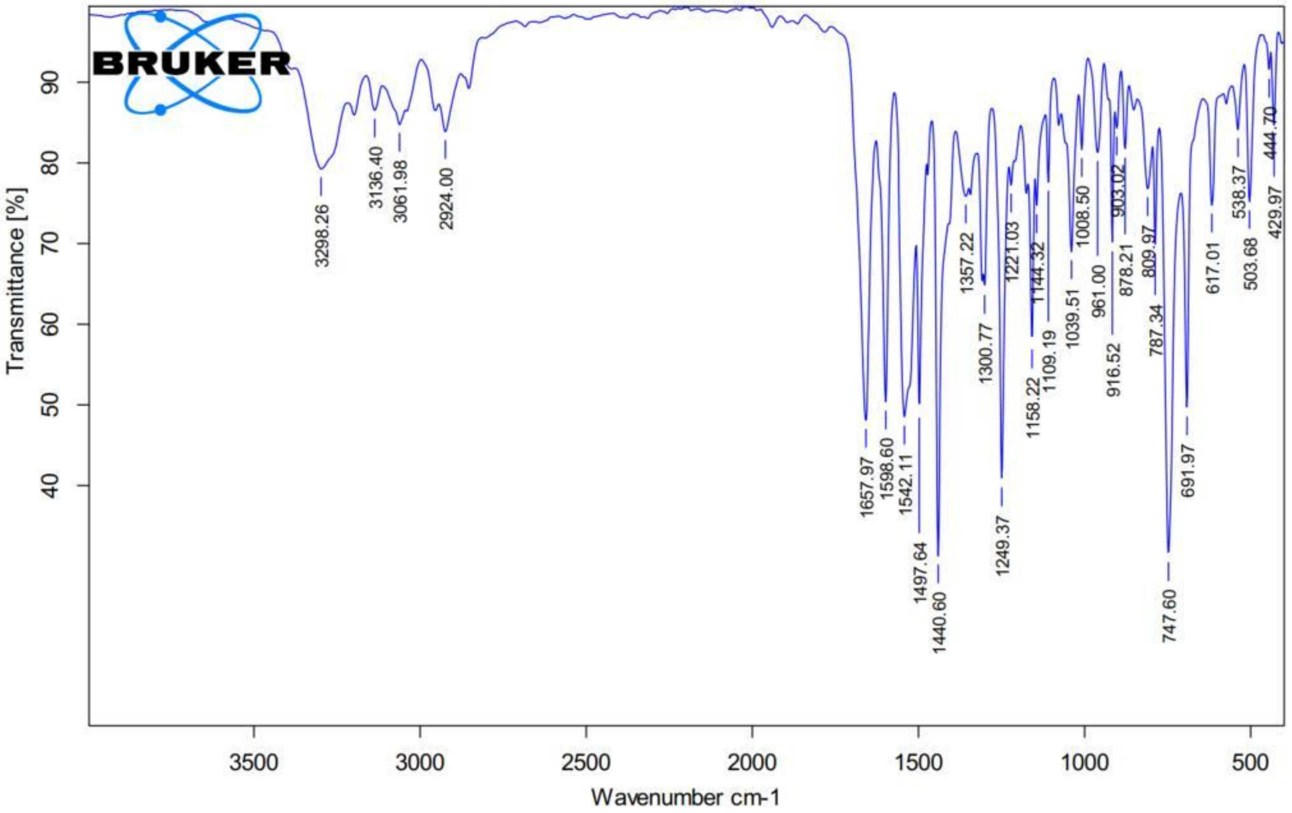

Supplementary Fig. 108. IR of compound **4v**

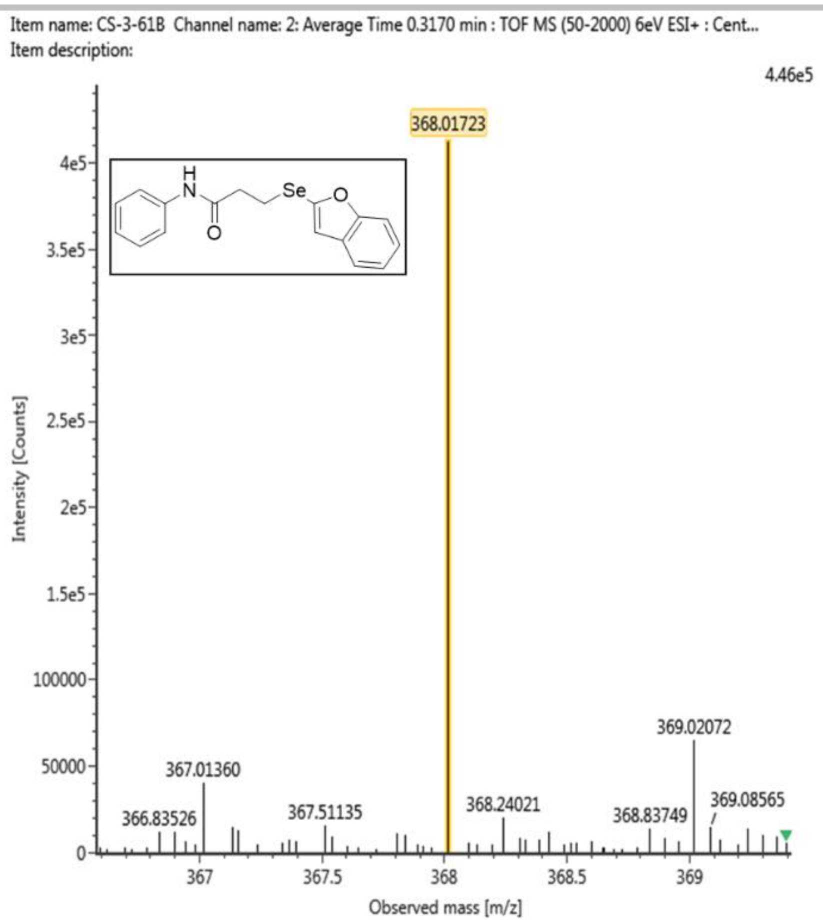

Supplementary Fig. 109. HR-MS of compound **4v**

^{1}H NMR (400 MHz, $CDCl_3$, 25°C) of compound **4w**

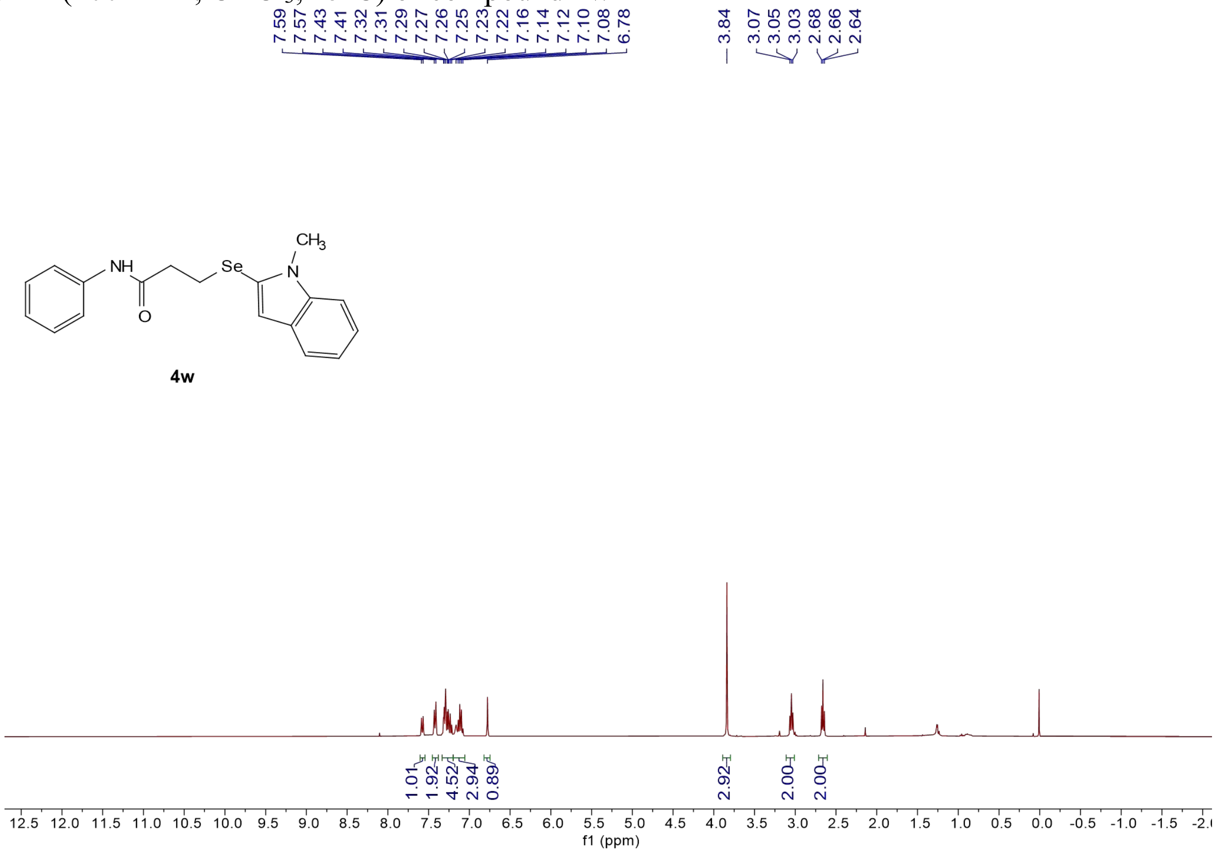

^{13}C NMR (101 MHz, $CDCl_3$, 25°C) of compound **4w**

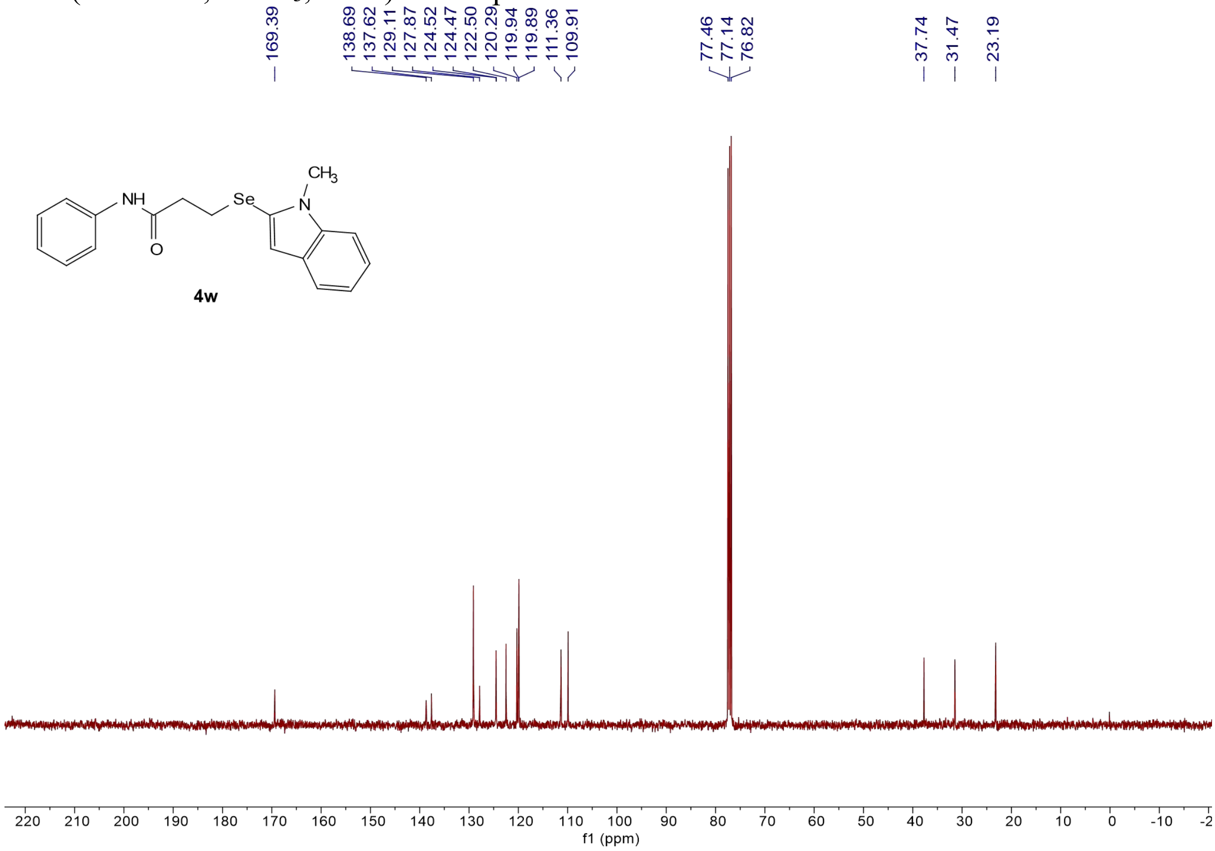

^{77}**Se NMR** (76 MHz, $CDCl_3$, 25°C) of compound **4w**

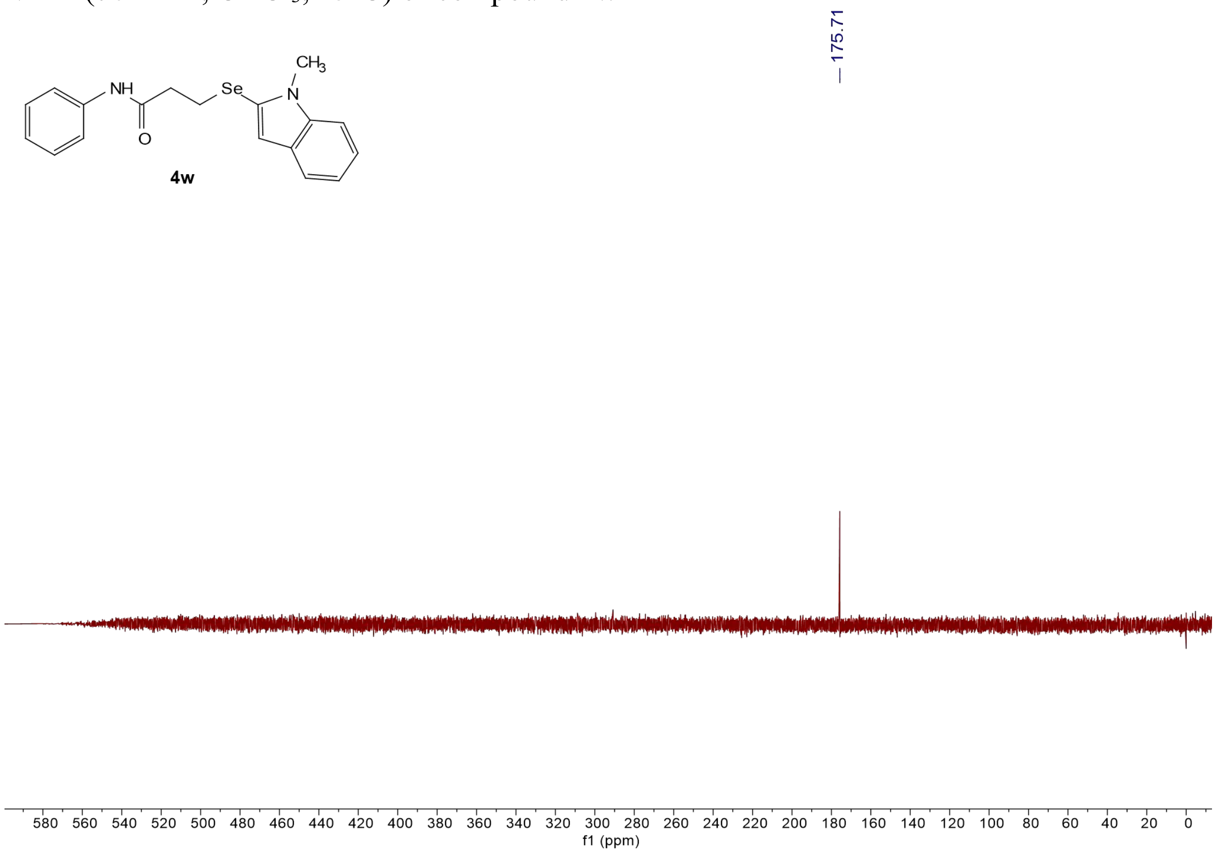

Supplementary Fig. 110. NMR spectra of compound **4w**

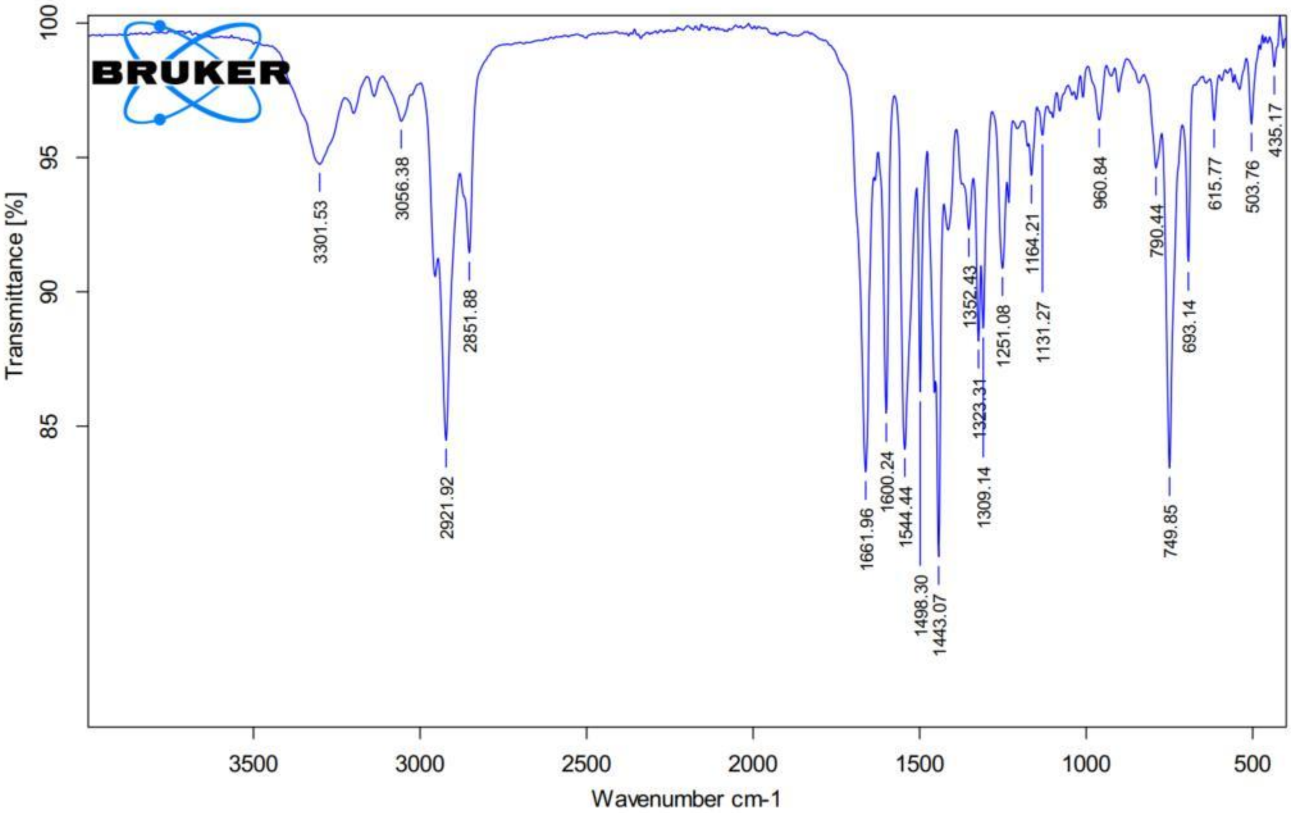

Supplementary Fig. 111. IR of compound **4w**

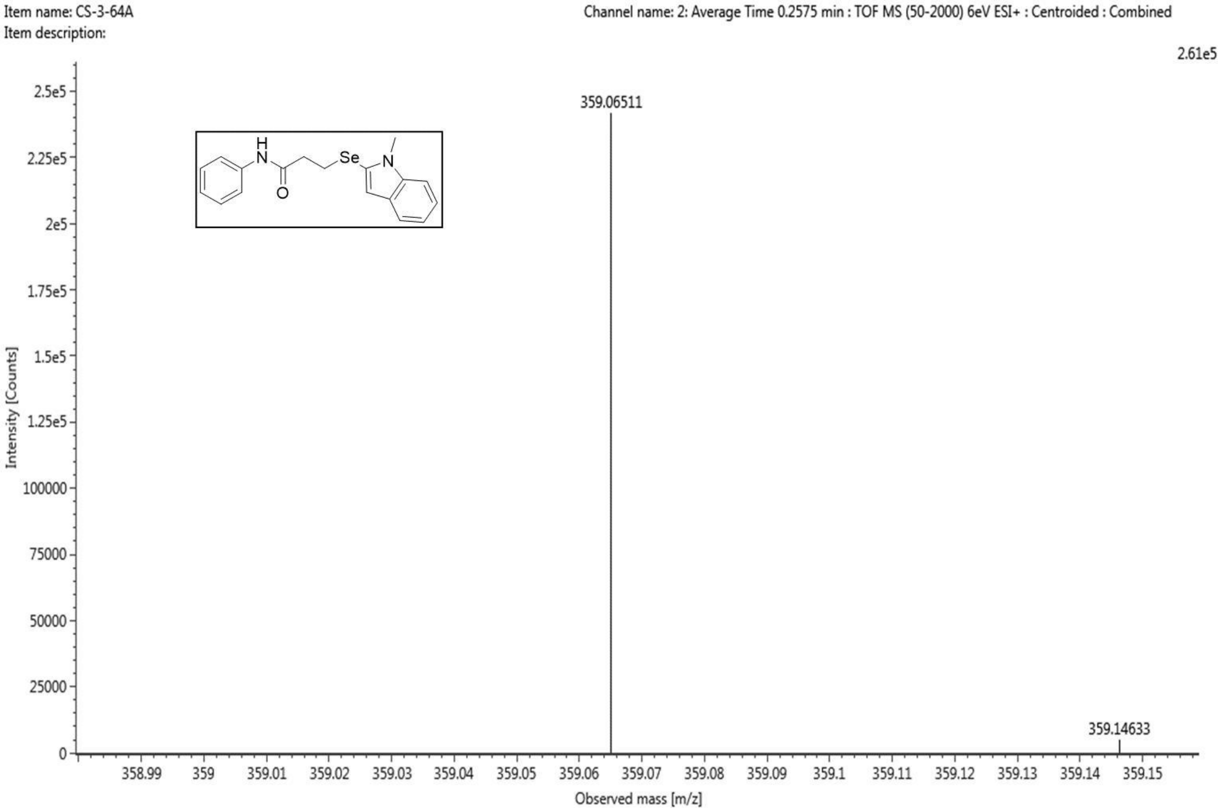

Supplementary Fig. 112. HR-MS of compound **4w**

^{1}H NMR (400 MHz, $CDCl_3$, 25°C) of compound **4x**

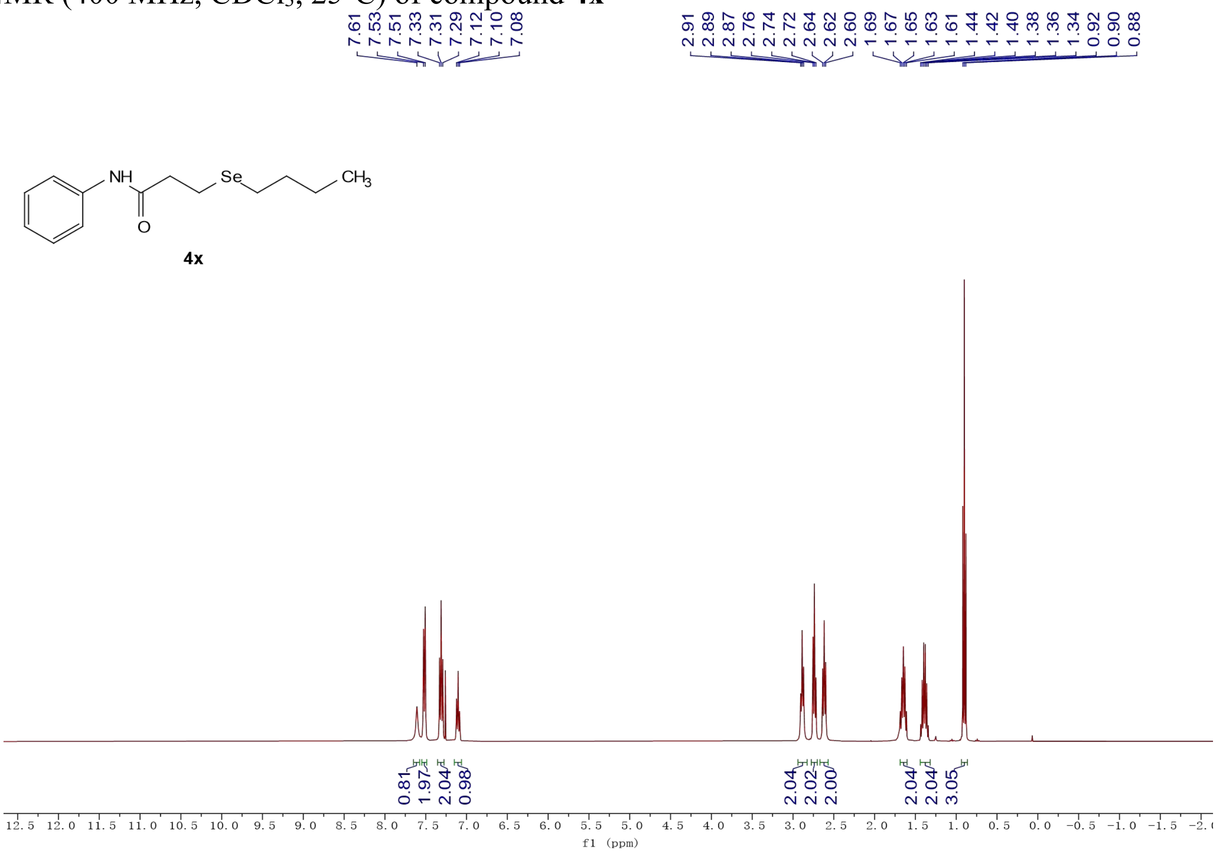

^{13}C NMR (101 MHz, $CDCl_3$, 25°C) of compound **4x**

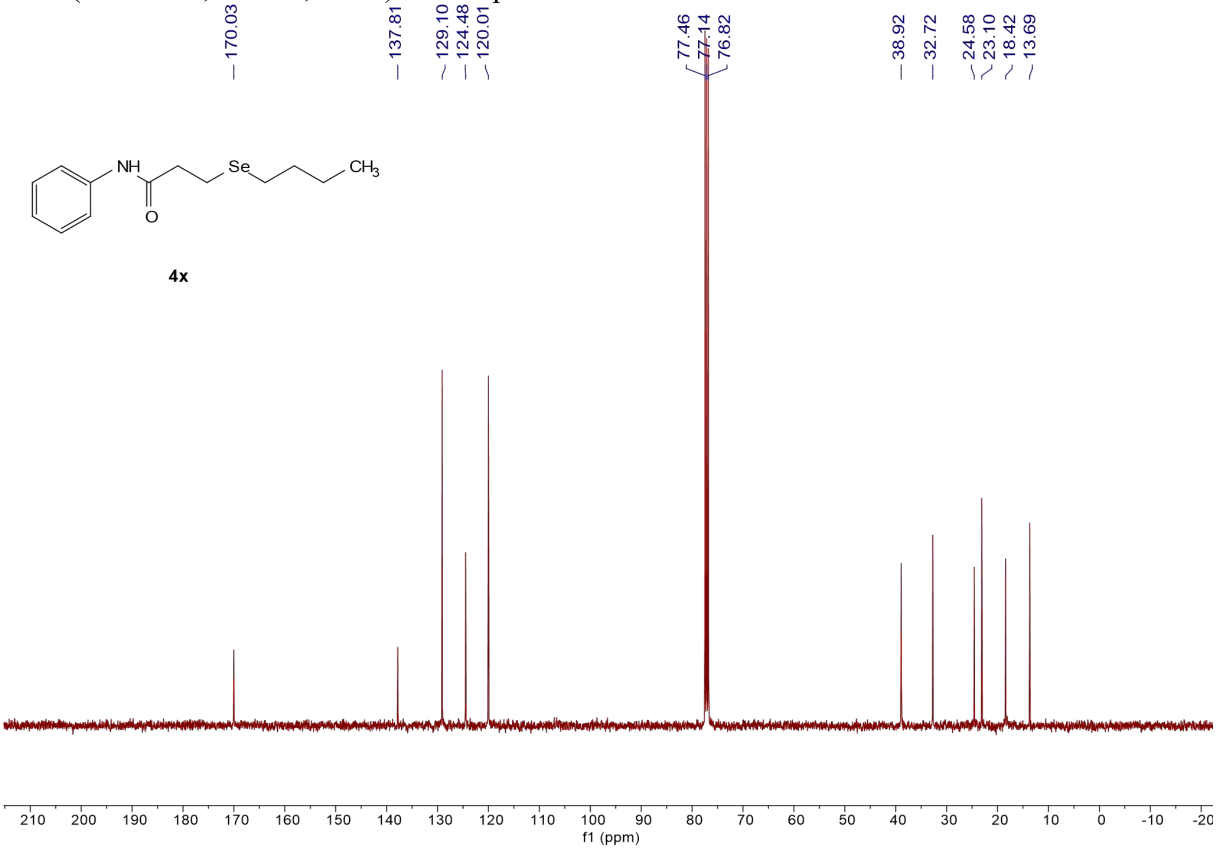

^{77}Se NMR (76 MHz, $CDCl_3$, 25°C) of compound **4x**

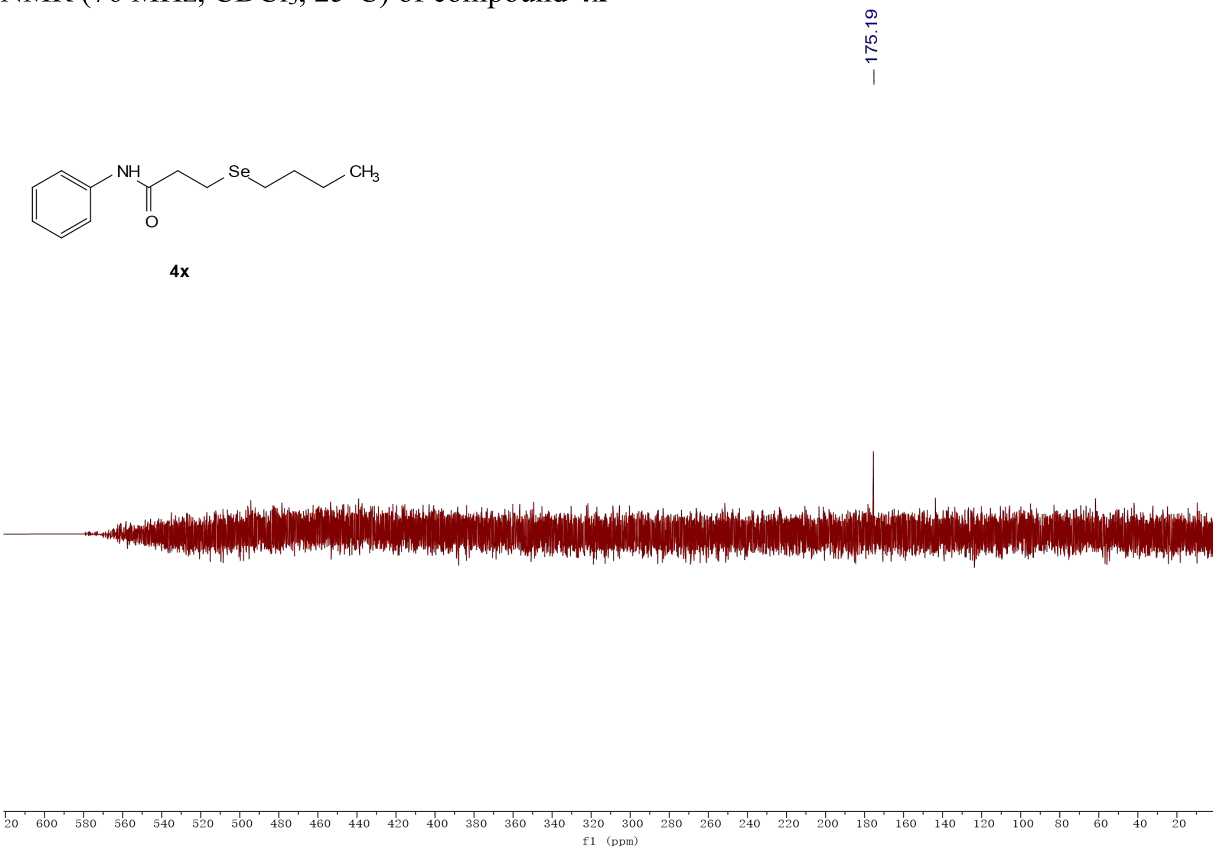

Supplementary Fig. 113. NMR spectra of compound **4x**

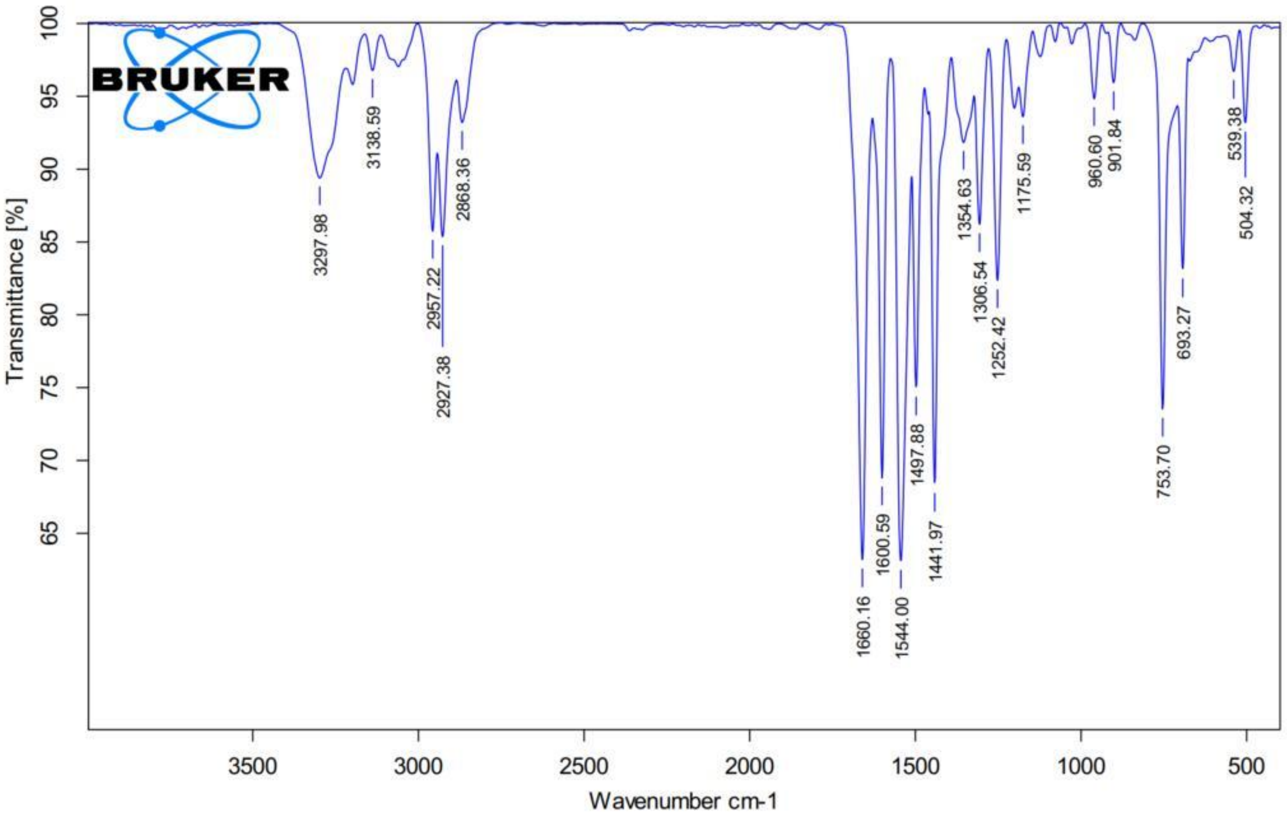

Supplementary Fig. 114. IR of compound **4x**

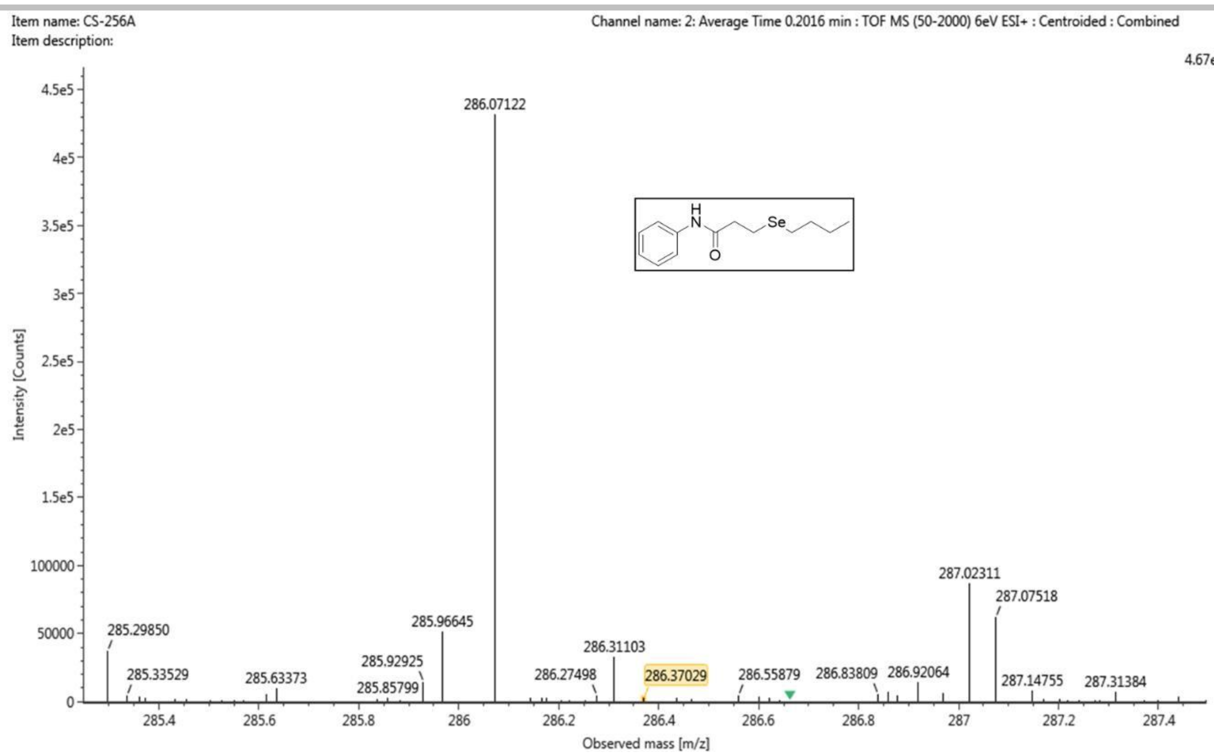

Supplementary Fig. 115. HR-MS of compound **4x**

^{1}H NMR (400 MHz, $CDCl_3$, 25°C) of compound **4y**

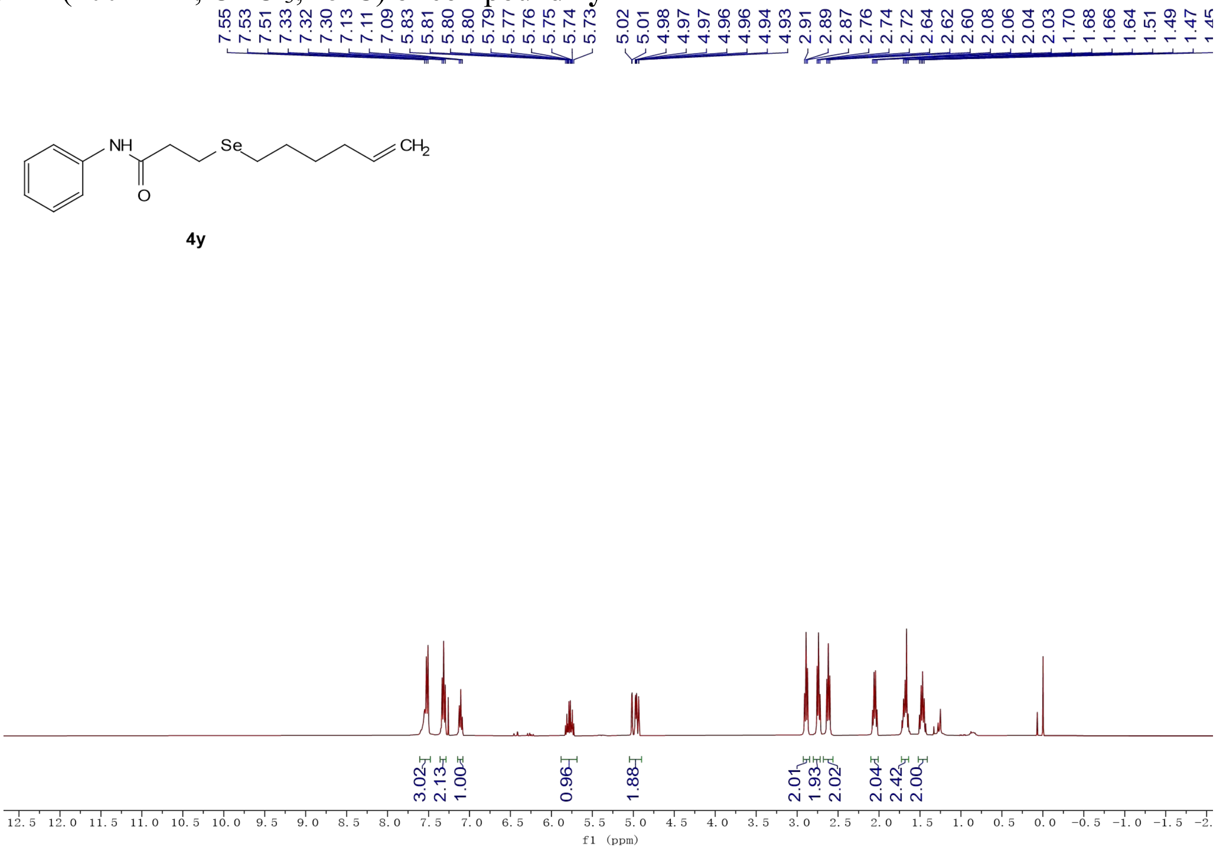

^{13}C NMR (101 MHz, $CDCl_3$, 25°C) of compound **4y**

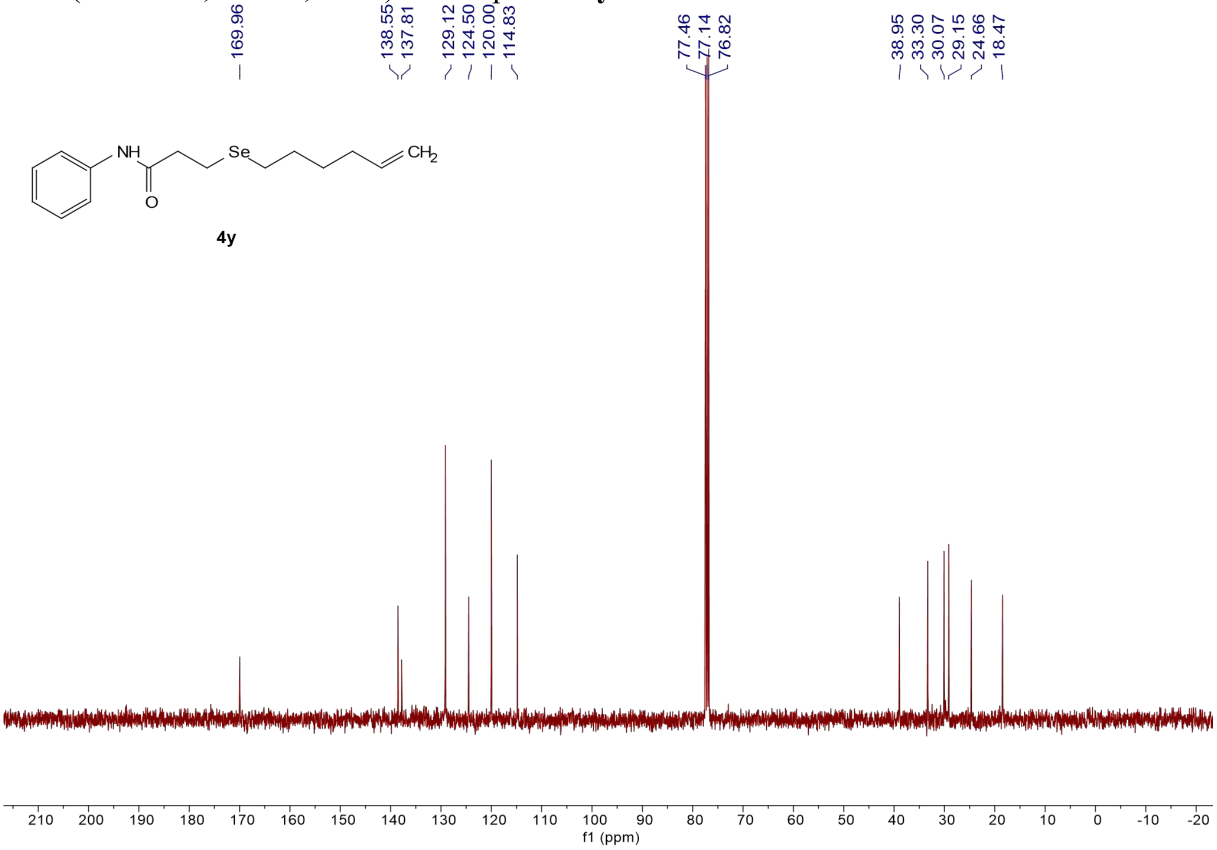

^{77}Se NMR (76 MHz, $CDCl_3$, 25°C) of compound **4y**

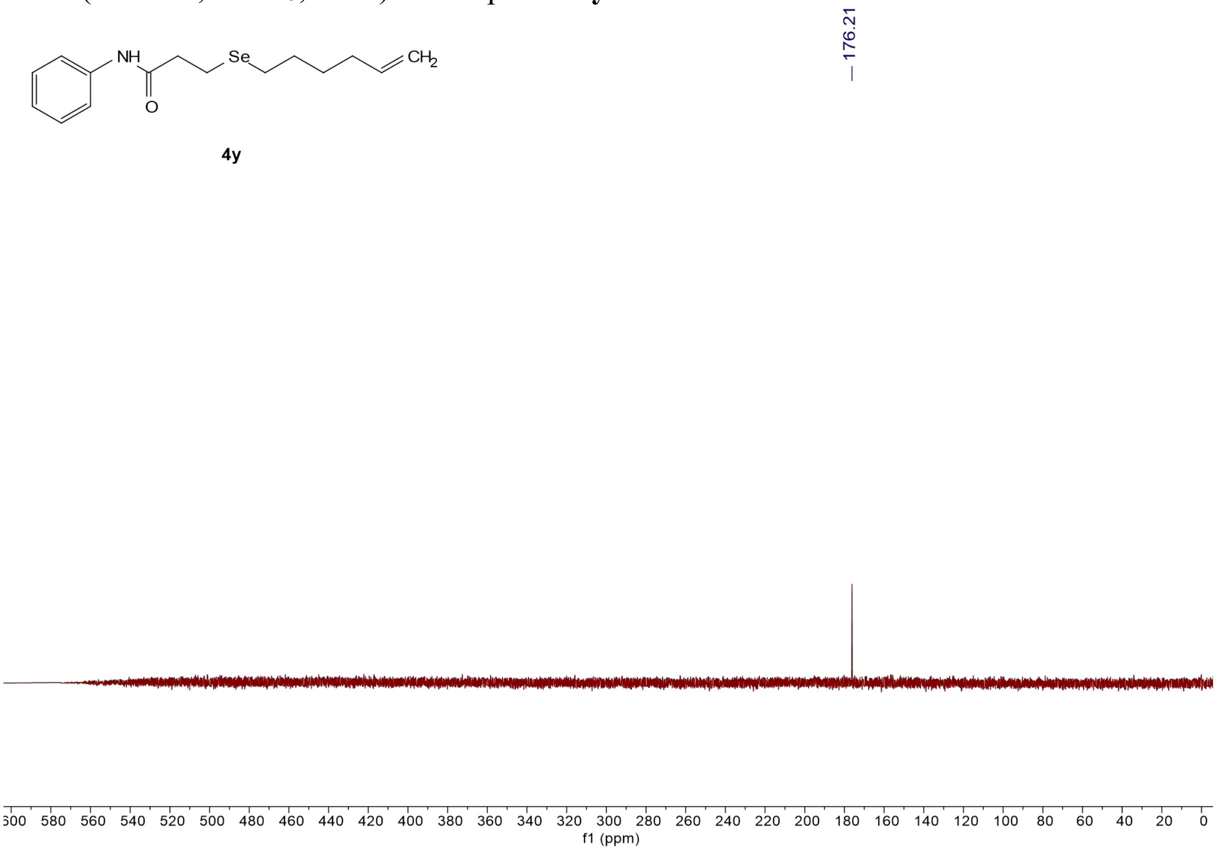

Supplementary Fig. 116. NMR spectra of compound **4y**

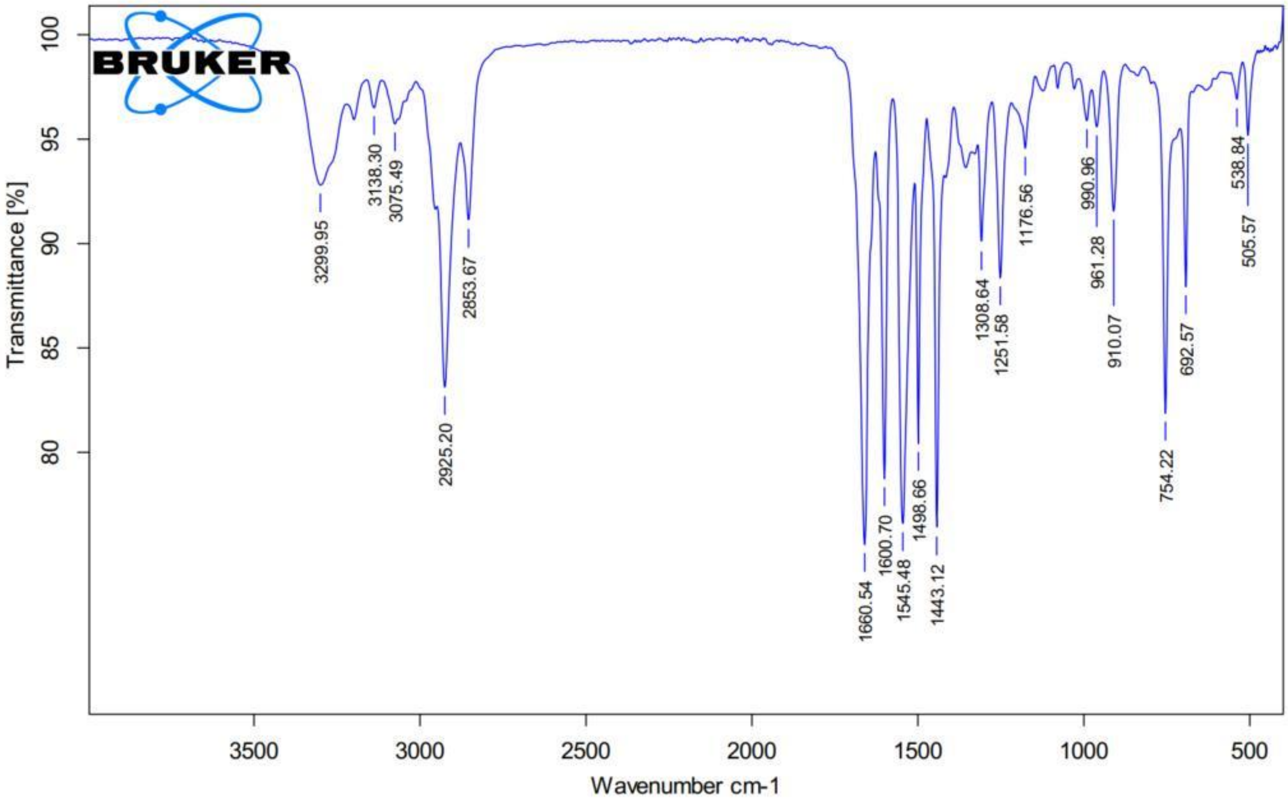

Supplementary Fig. 117. IR of compound **4y**

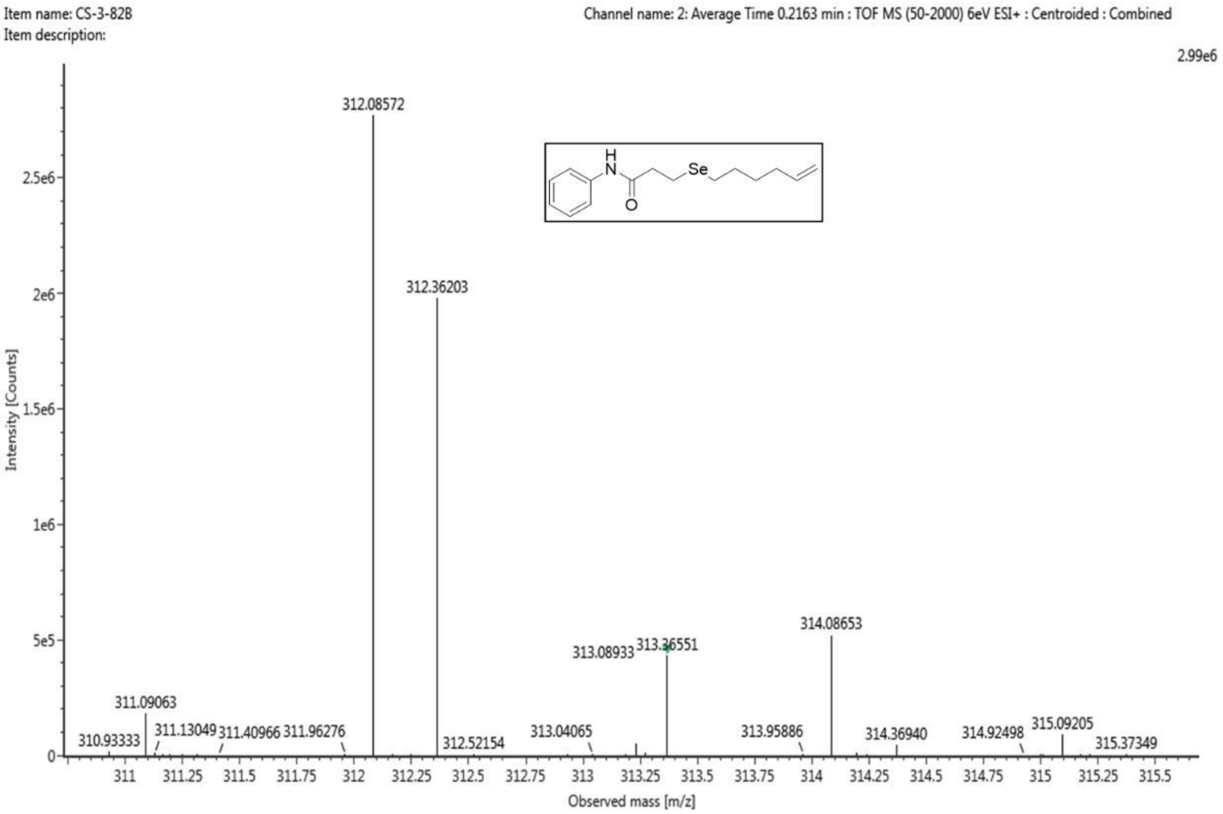

Supplementary Fig. 118. HR-MS of compound **4y**

^{1}H NMR (400 MHz, $CDCl_3$, 25℃) of compound **4z**

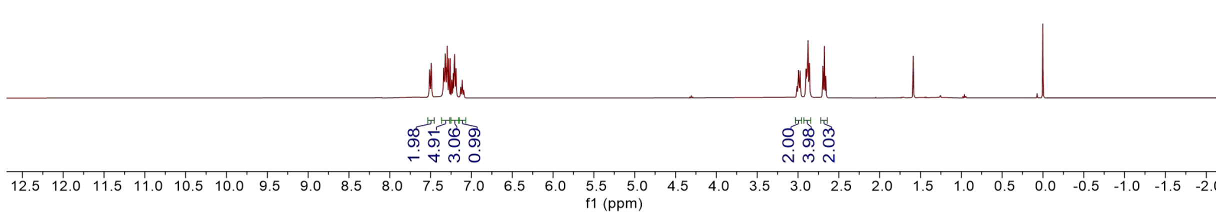

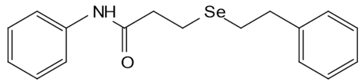

4z

^{13}C NMR (101 MHz, $CDCl_3$, 25℃) of compound **4z**

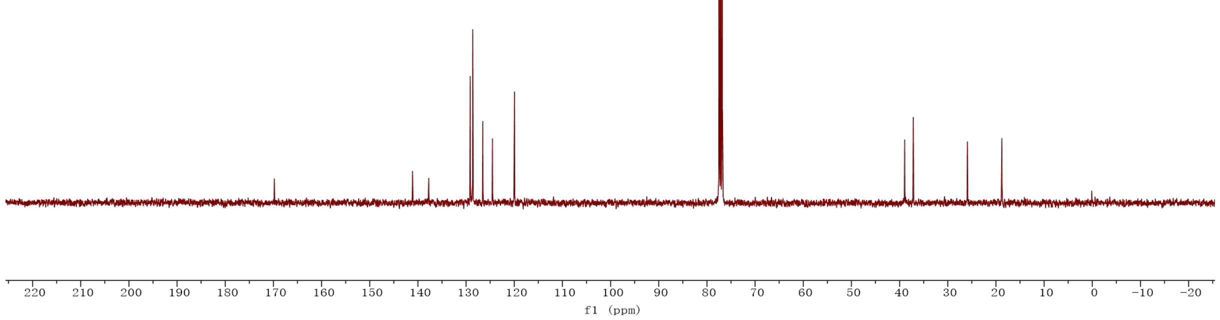

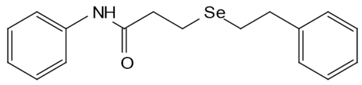

4z

^{77}Se NMR (76 MHz, $CDCl_3$, 25°C) of compound **4z**

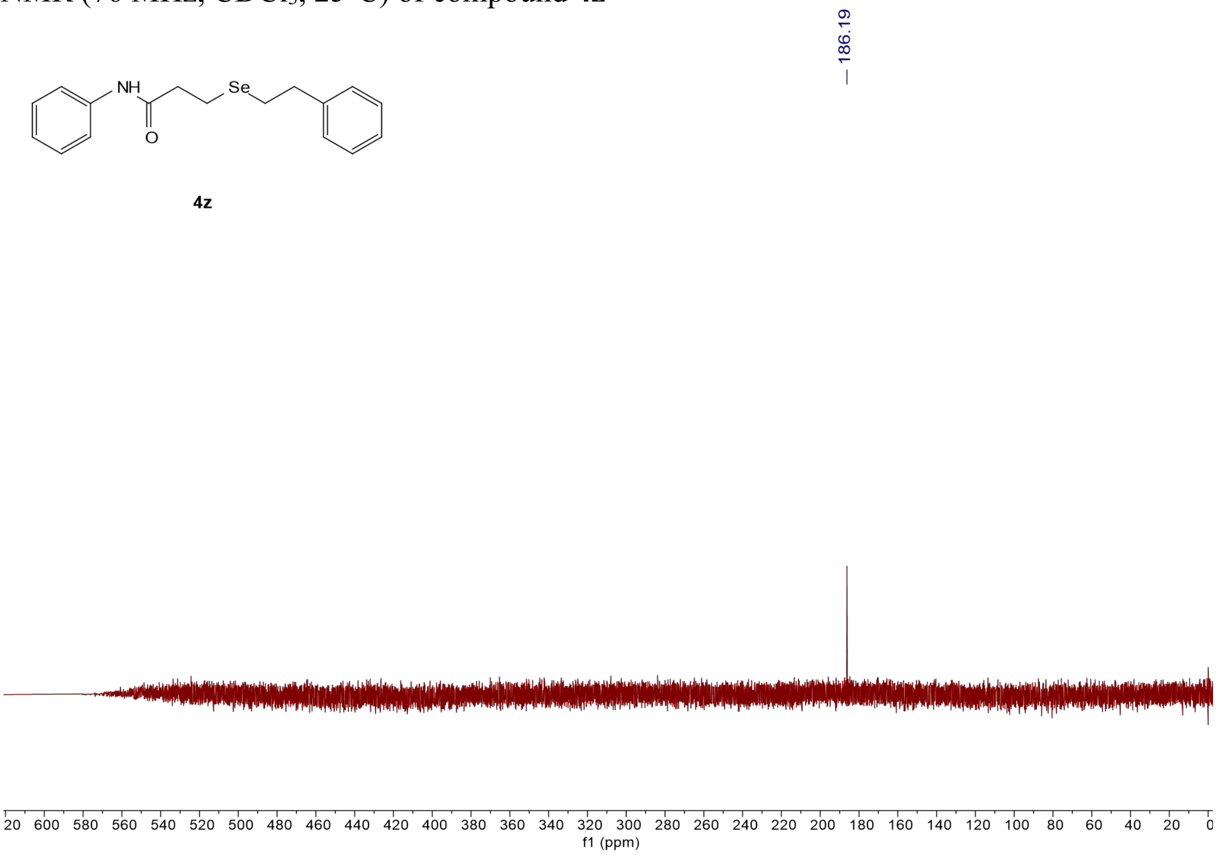

Supplementary Fig. 119. NMR spectra of compound **4z**

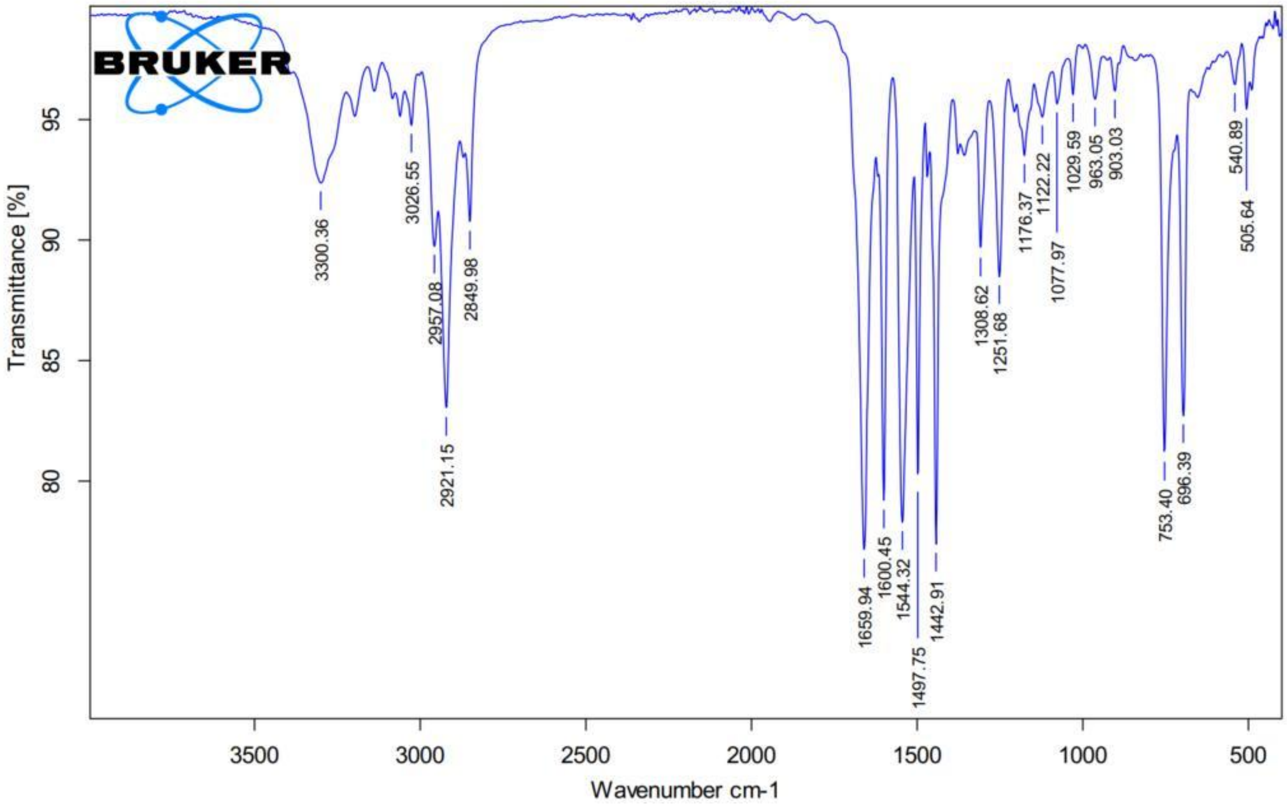

Supplementary Fig. 120. IR of compound **4z**

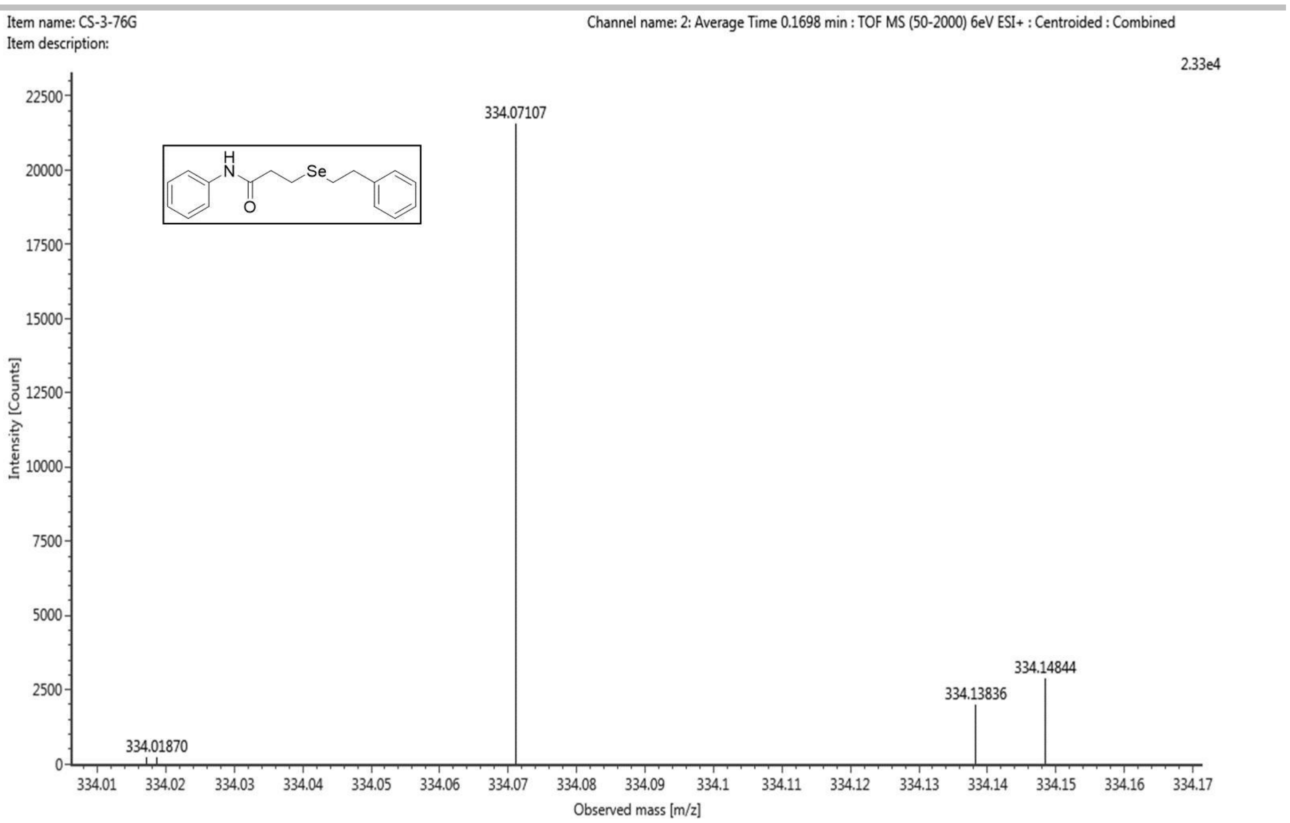

Supplementary Fig. 121. HR-MS of compound **4z**

^{1}H NMR (400 MHz, $CDCl_3$, 25°C) of compound **5**

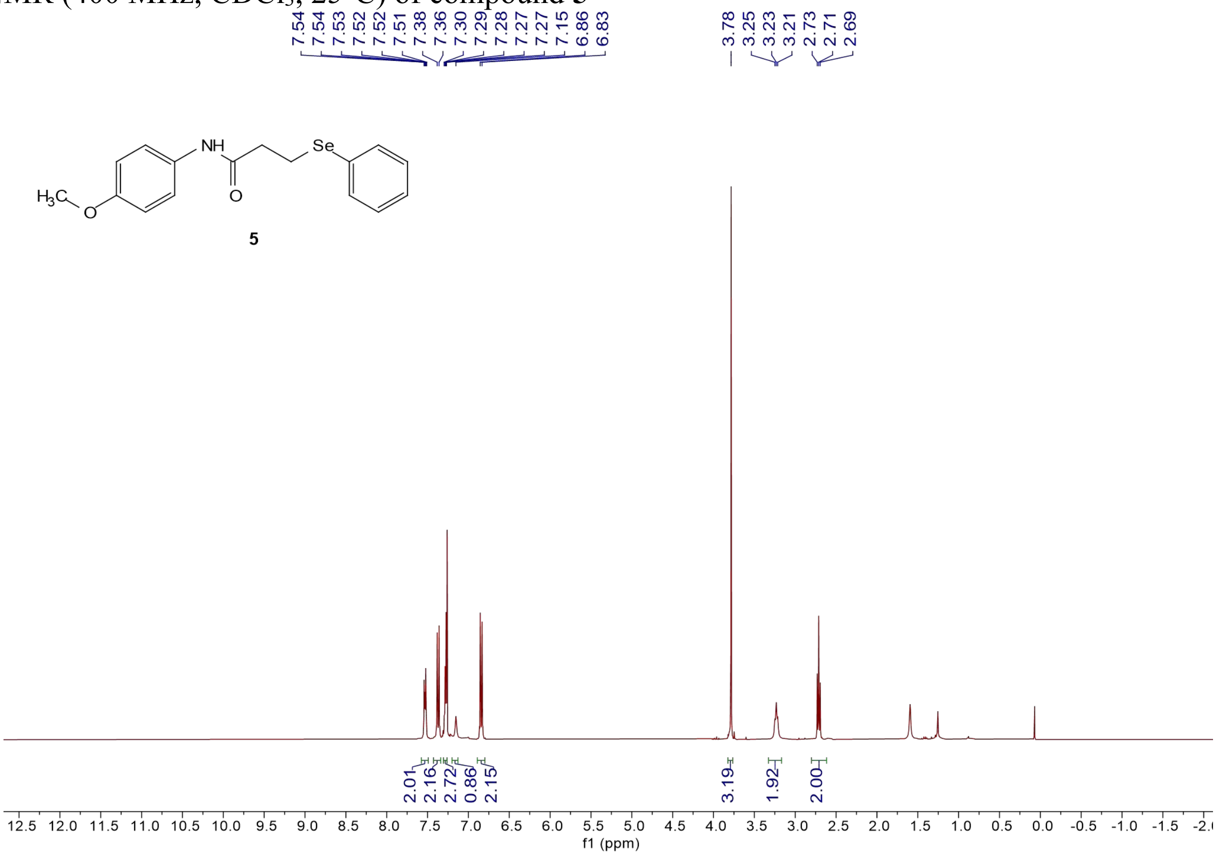

^{13}C NMR (101 MHz, $CDCl_3$, 25°C) of compound **5**

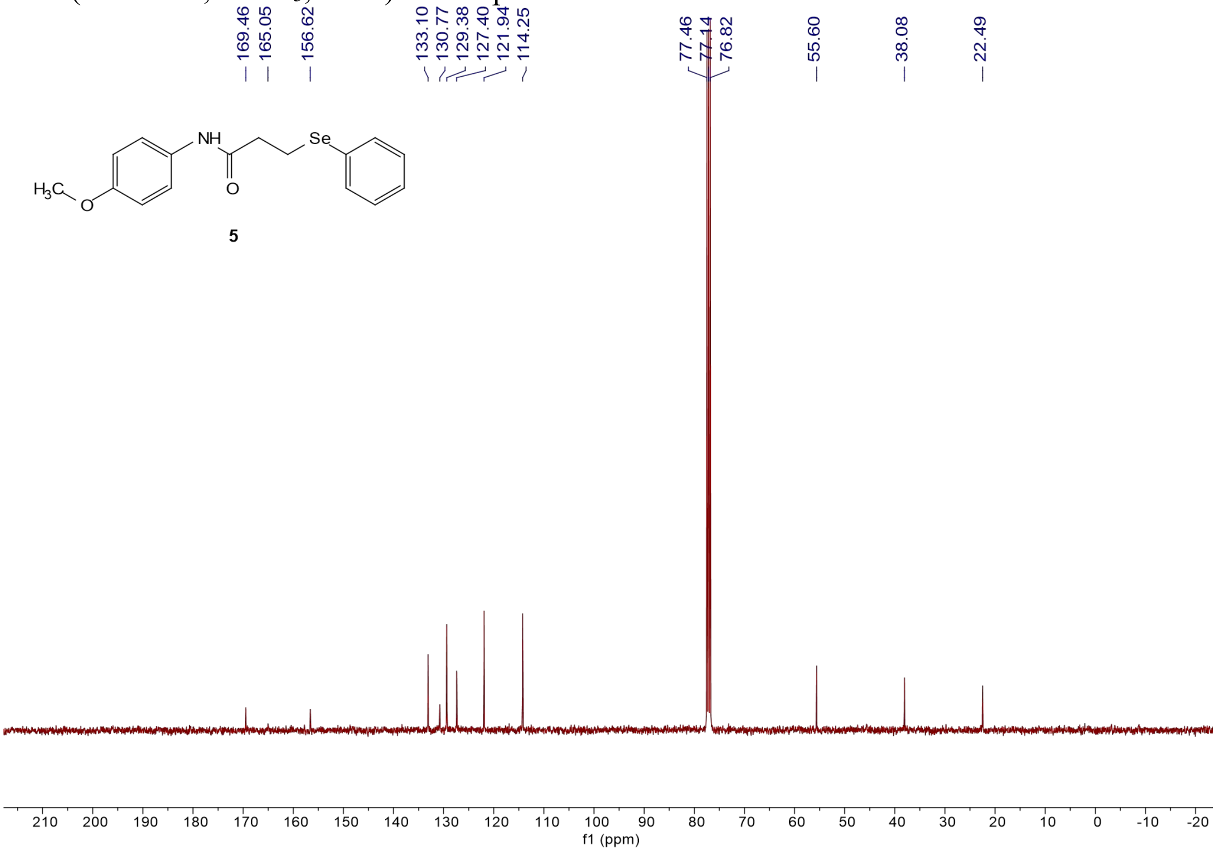

^{77}Se NMR (76 MHz, $CDCl_3$, 25°C) of compound **5**

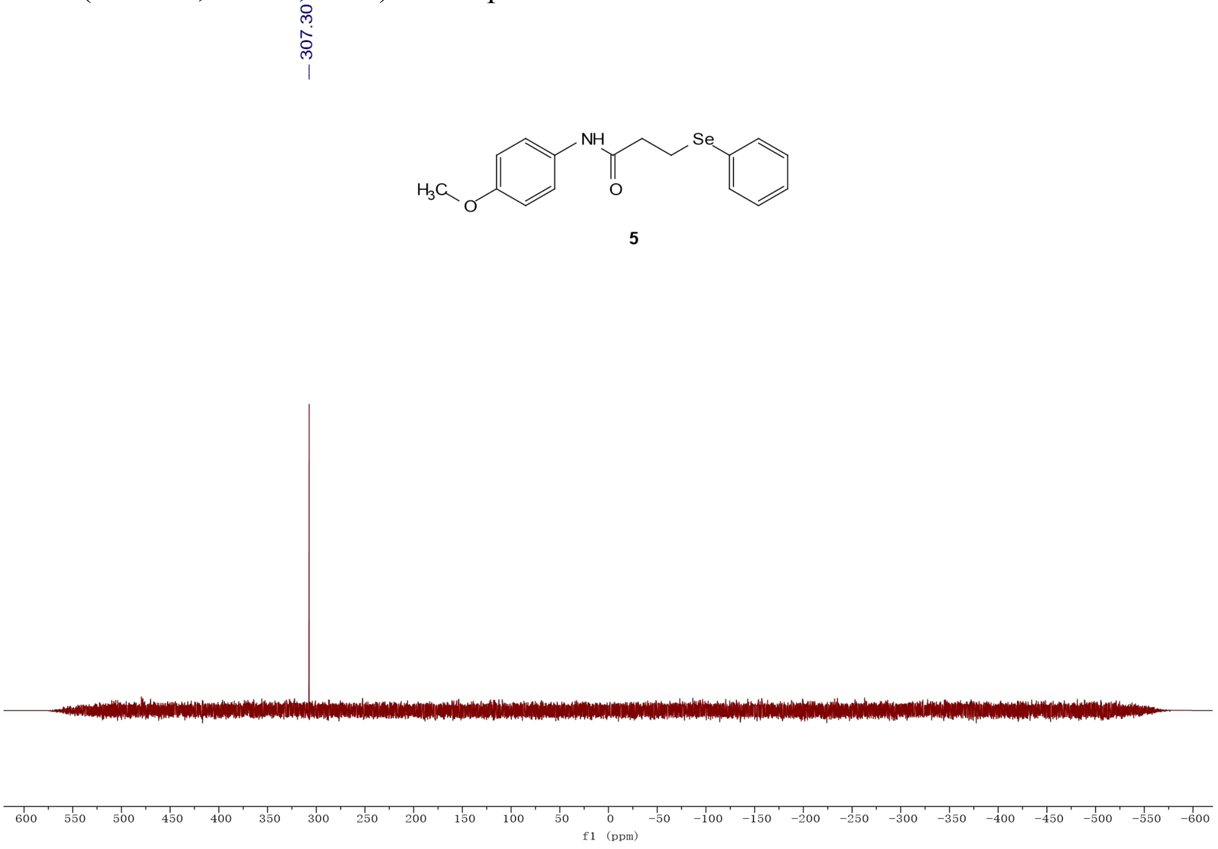

Supplementary Fig. 122. NMR spectra of compound **5**

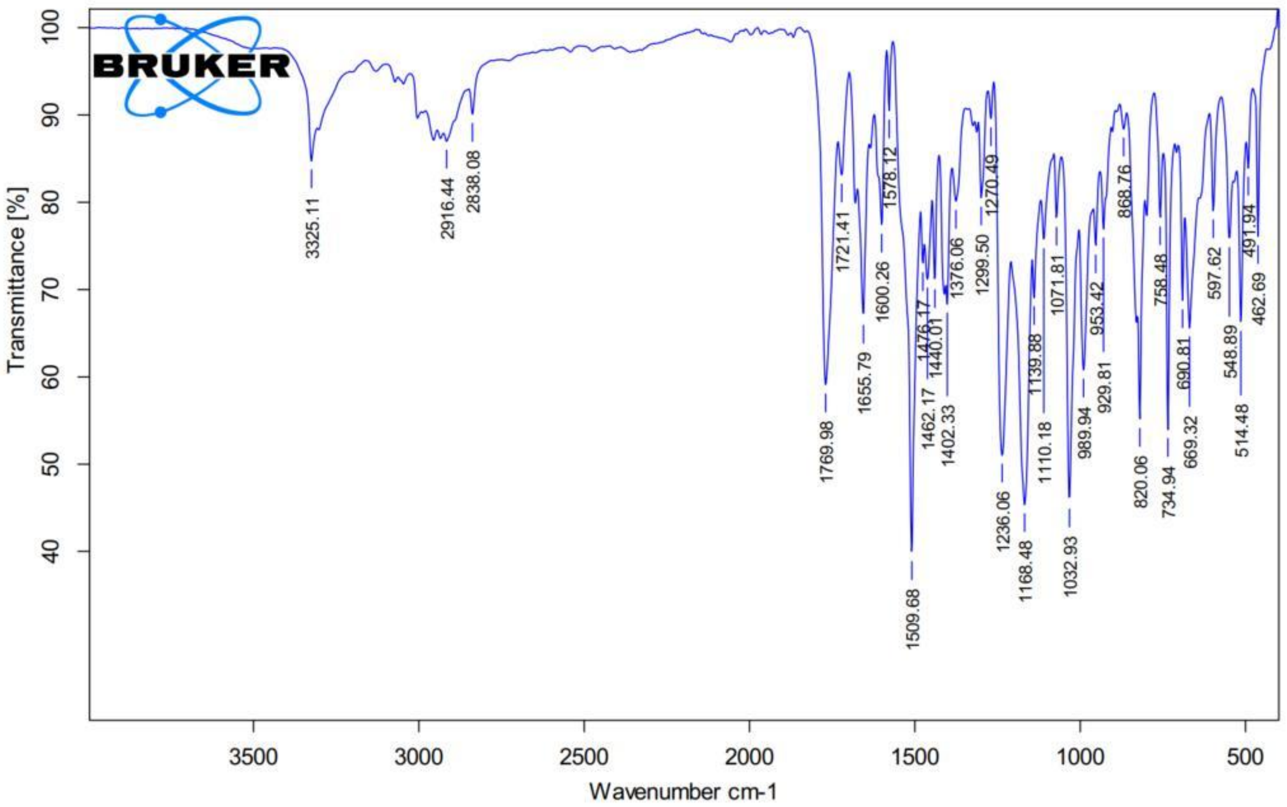

Supplementary Fig. 123. IR of compound **5**

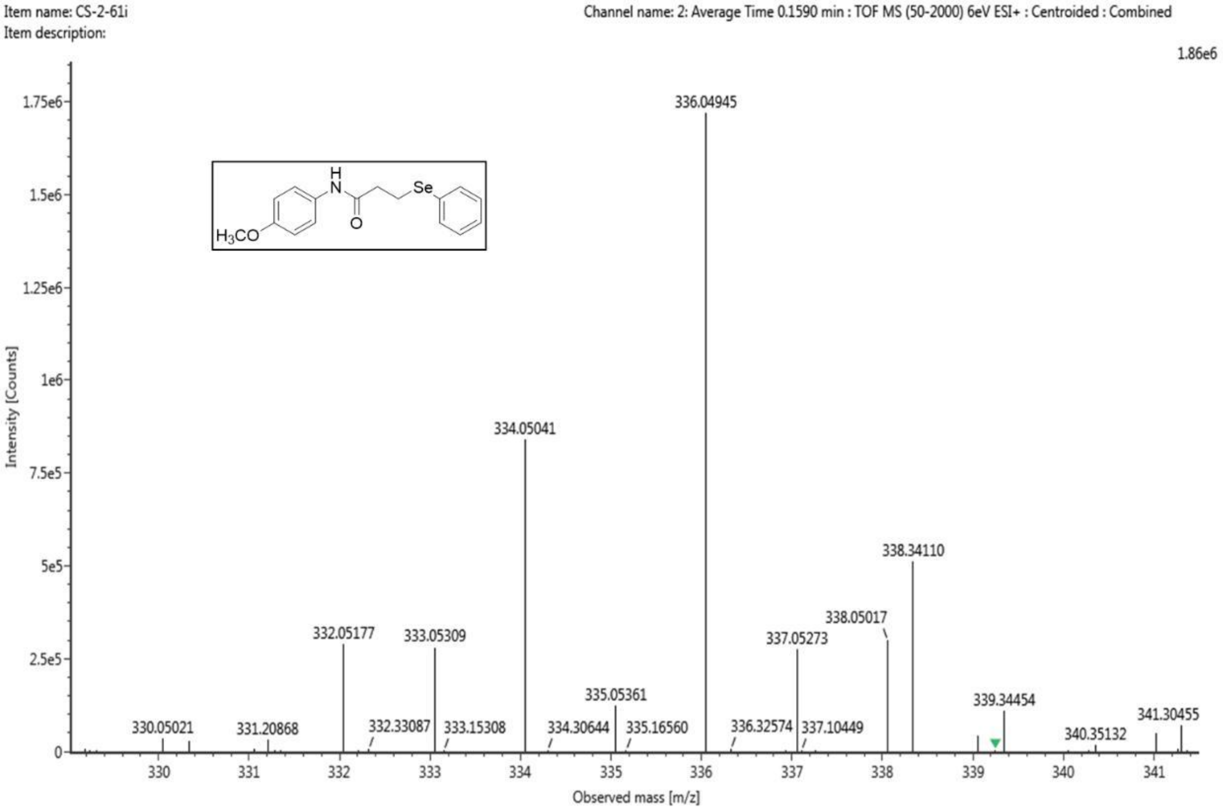

Supplementary Fig. 124. HR-MS of compound **5**

^{1}H NMR (400 MHz, $CDCl_3$, 25°C) of compound **6**

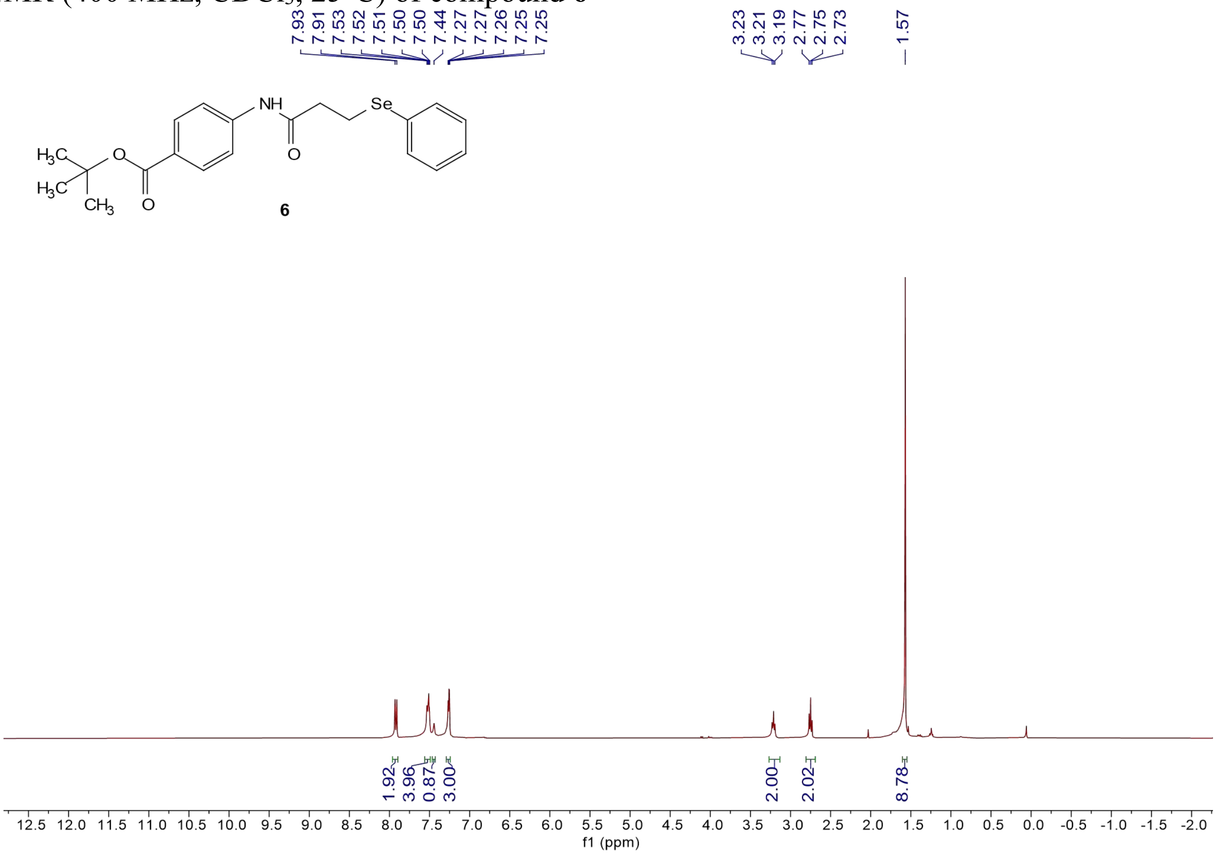

^{13}C NMR (101 MHz, $CDCl_3$, 25°C) of compound **6**

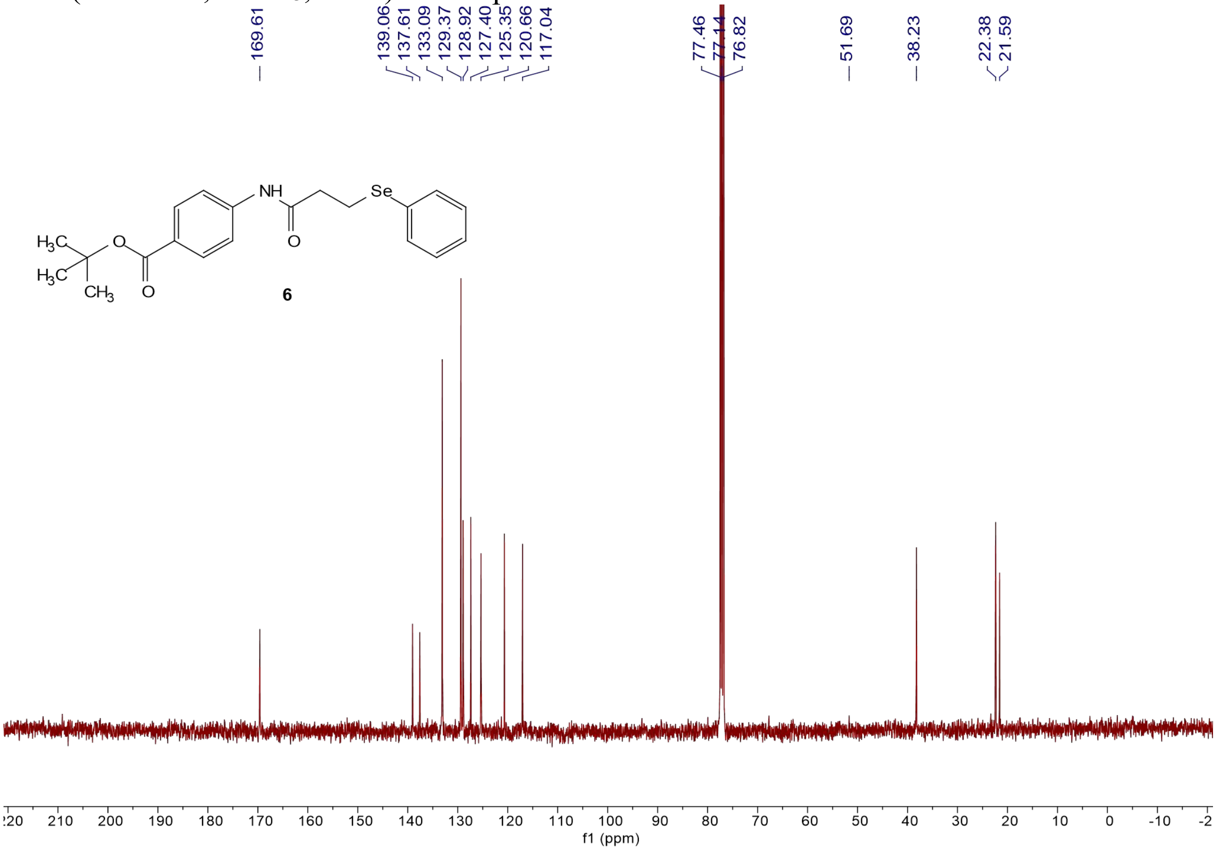

^{77}Se NMR (76 MHz, CDCl3, 25°C) of compound **6**

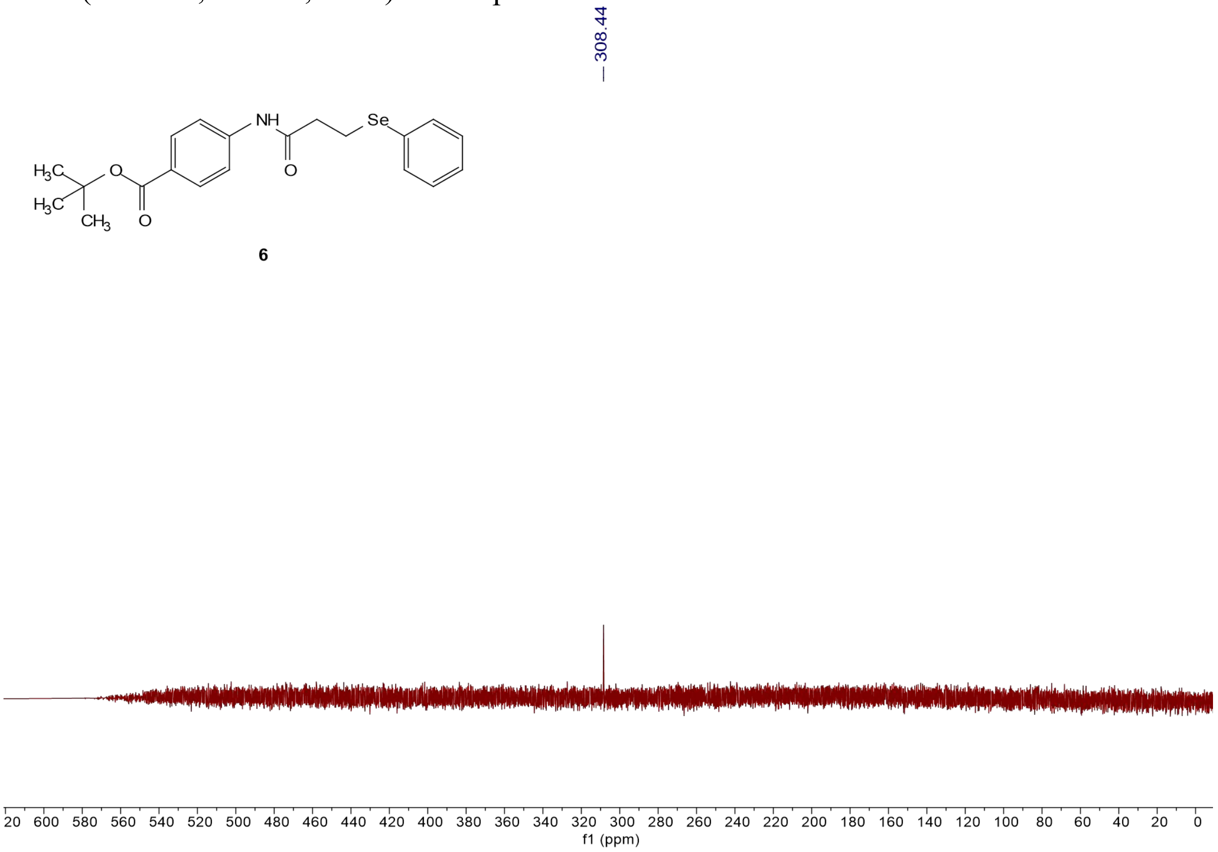

Supplementary Fig. 125. NMR spectra of compound **6**

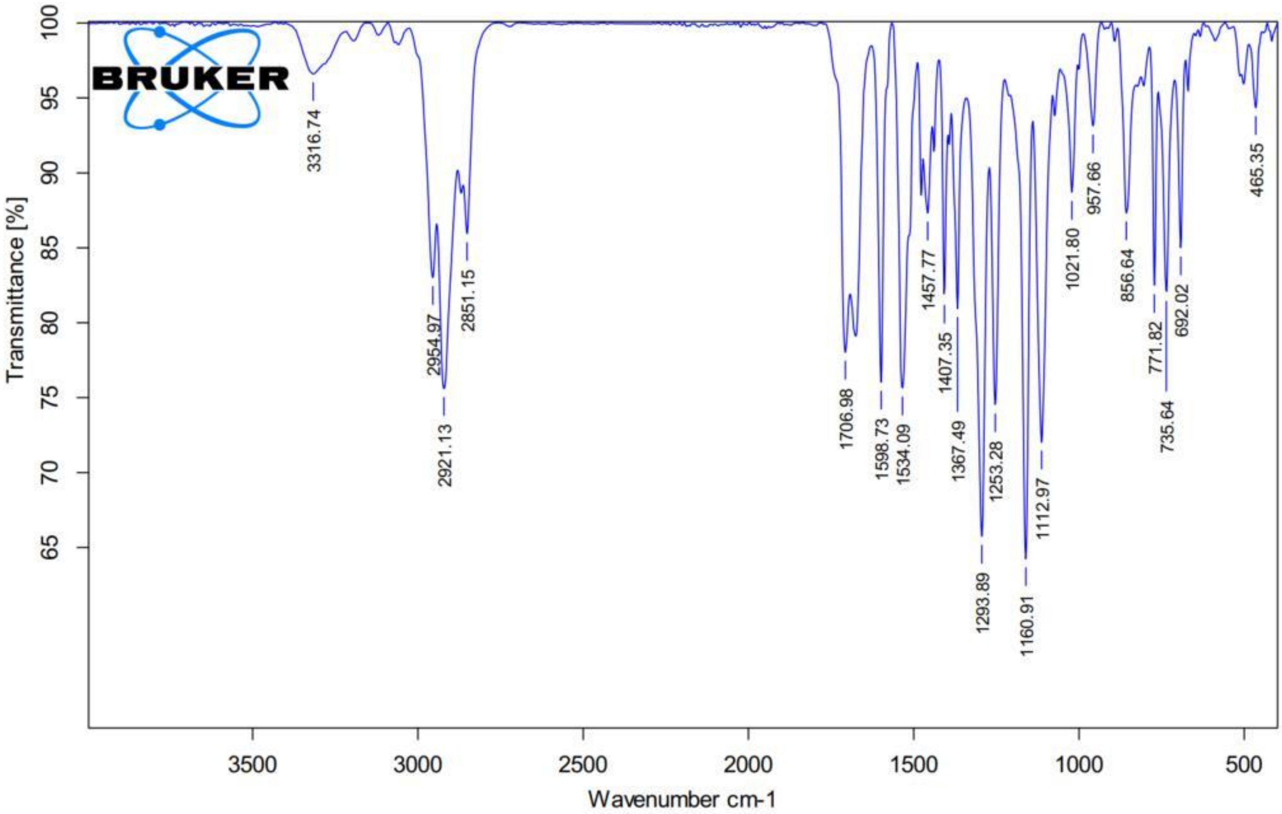

Supplementary Fig. 126. IR of compound **6**

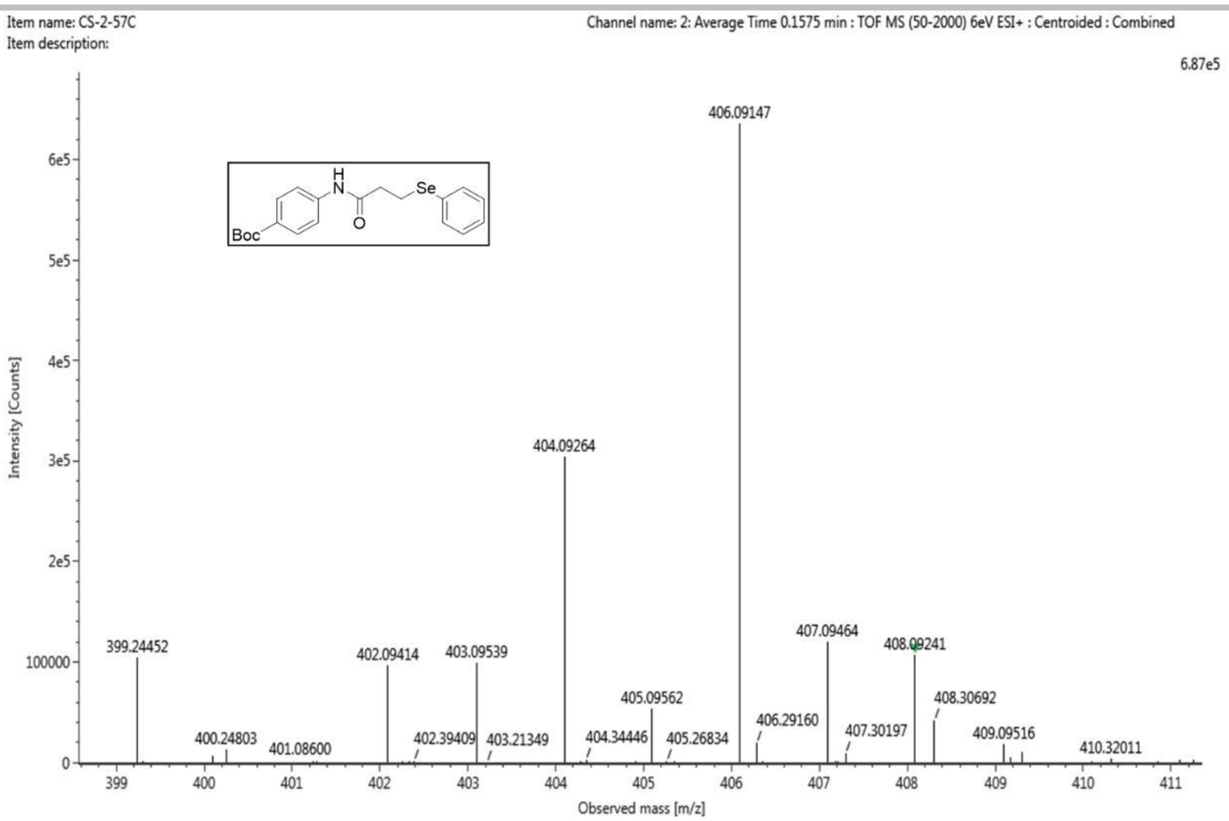

Supplementary Fig. 127. HR-MS of compound **6**

^{1}H NMR (400 MHz, $CDCl_3$, 25°C) of compound **7**

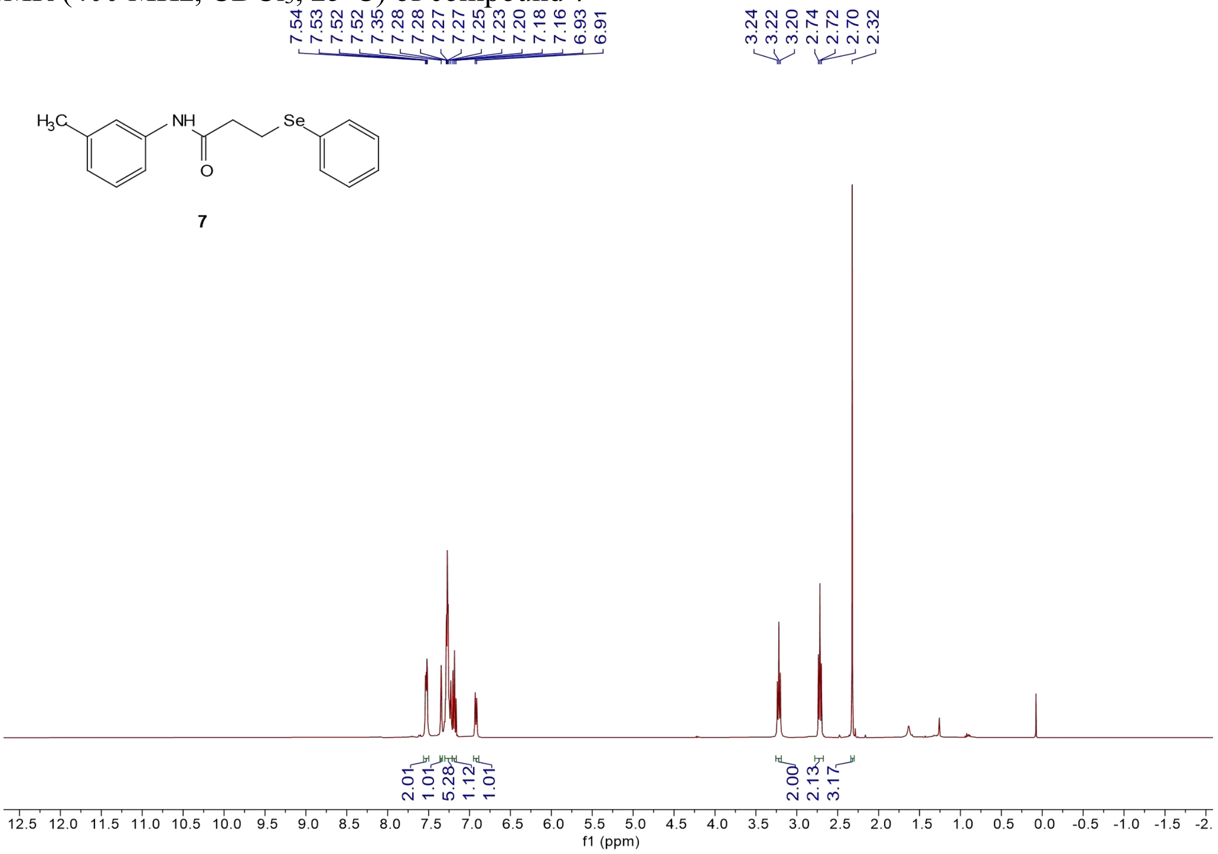

^{13}C NMR (101 MHz, $CDCl_3$, 25°C) of compound **7**

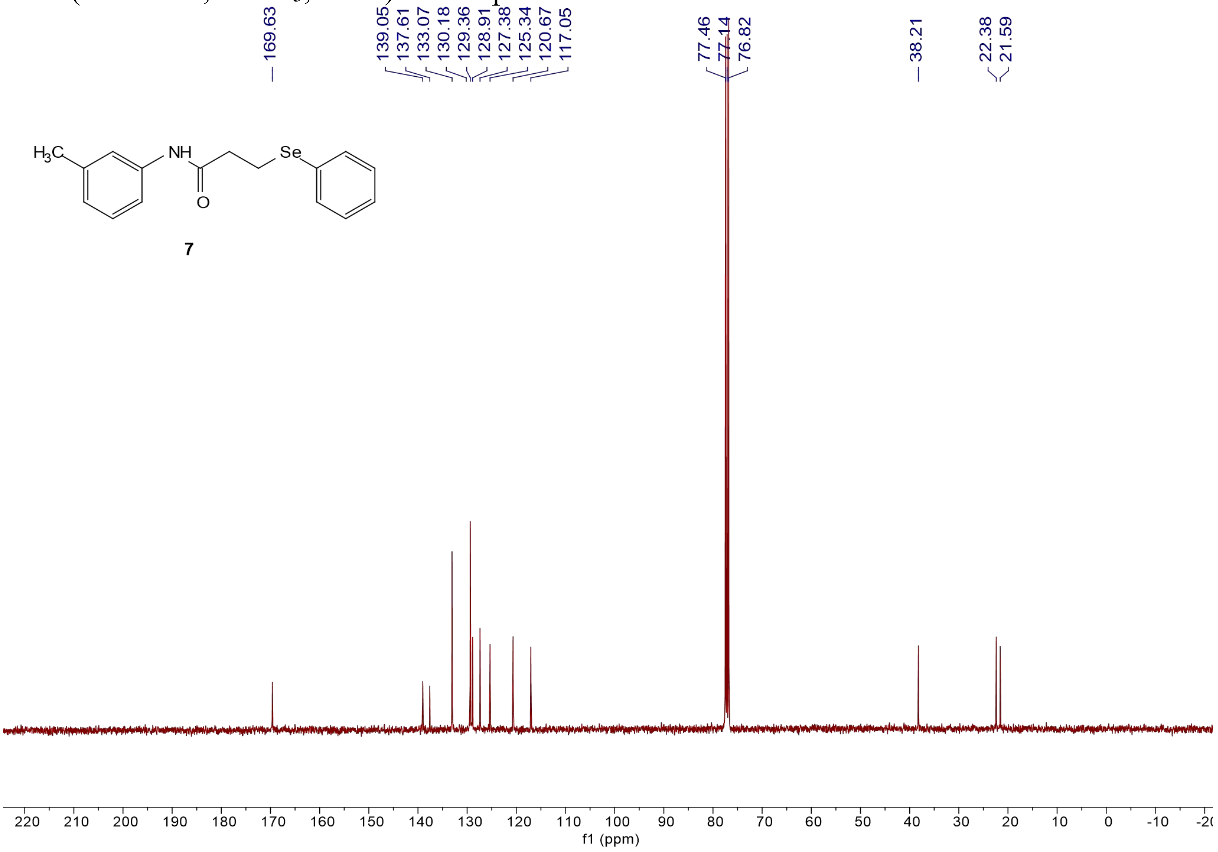

^{77}Se NMR (76 MHz, $CDCl_3$, 25°C) of compound **7**

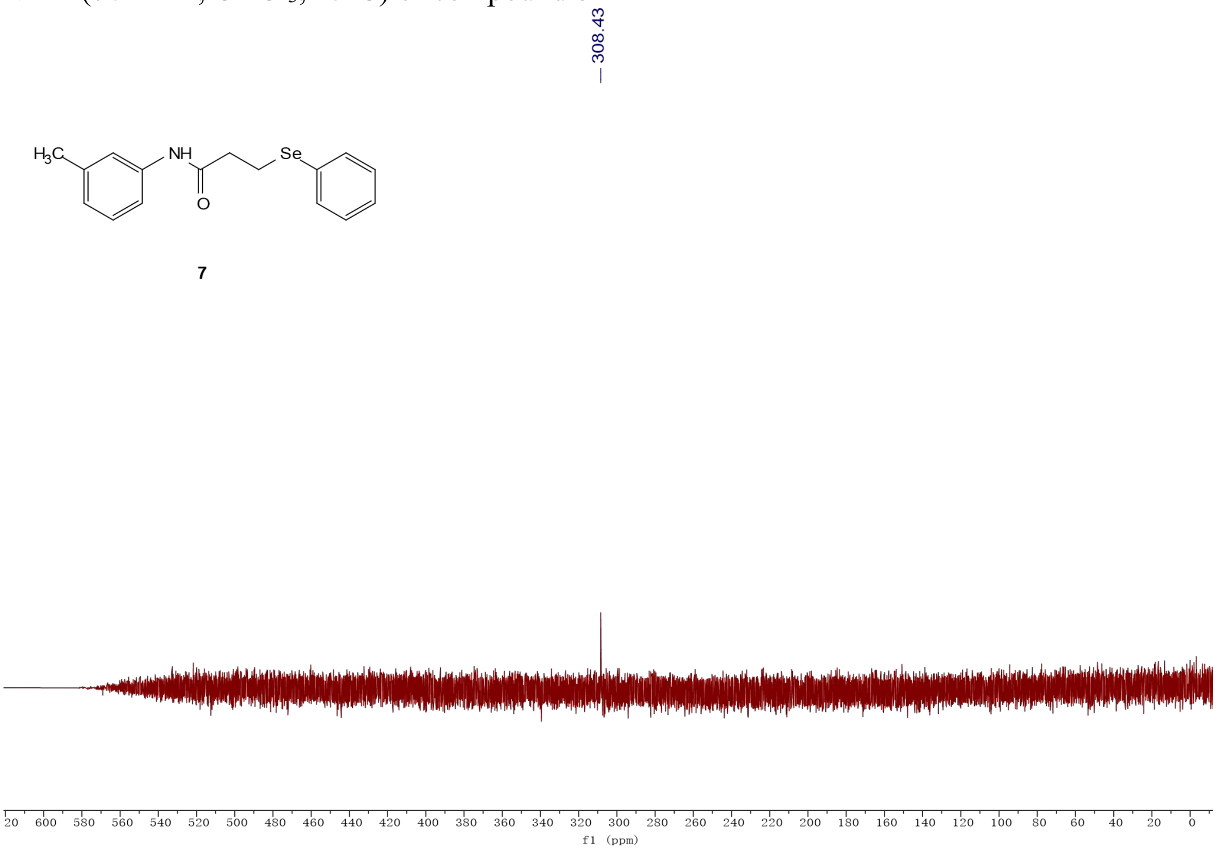

Supplementary Fig. 128. NMR spectra of compound **7**

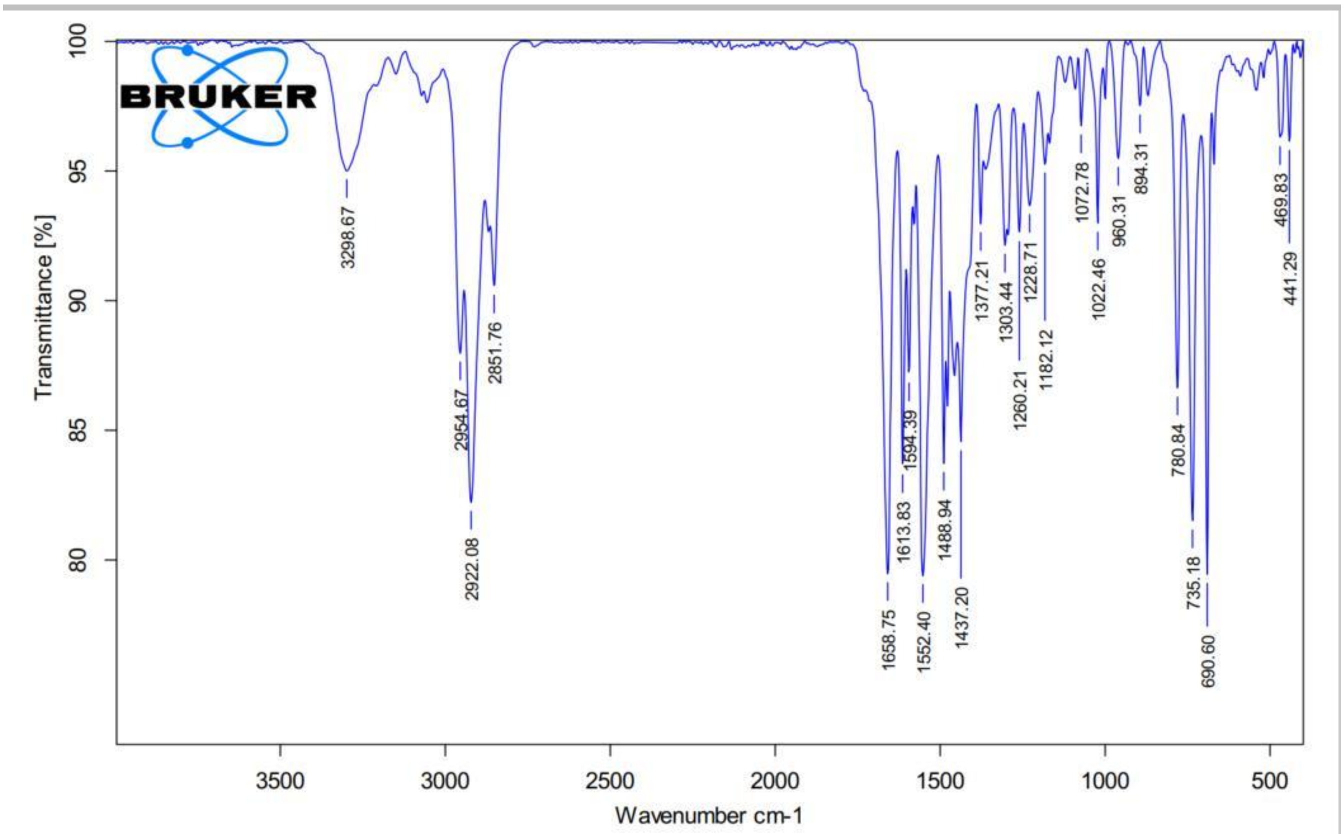

Supplementary Fig. 129. IR of compound **7**

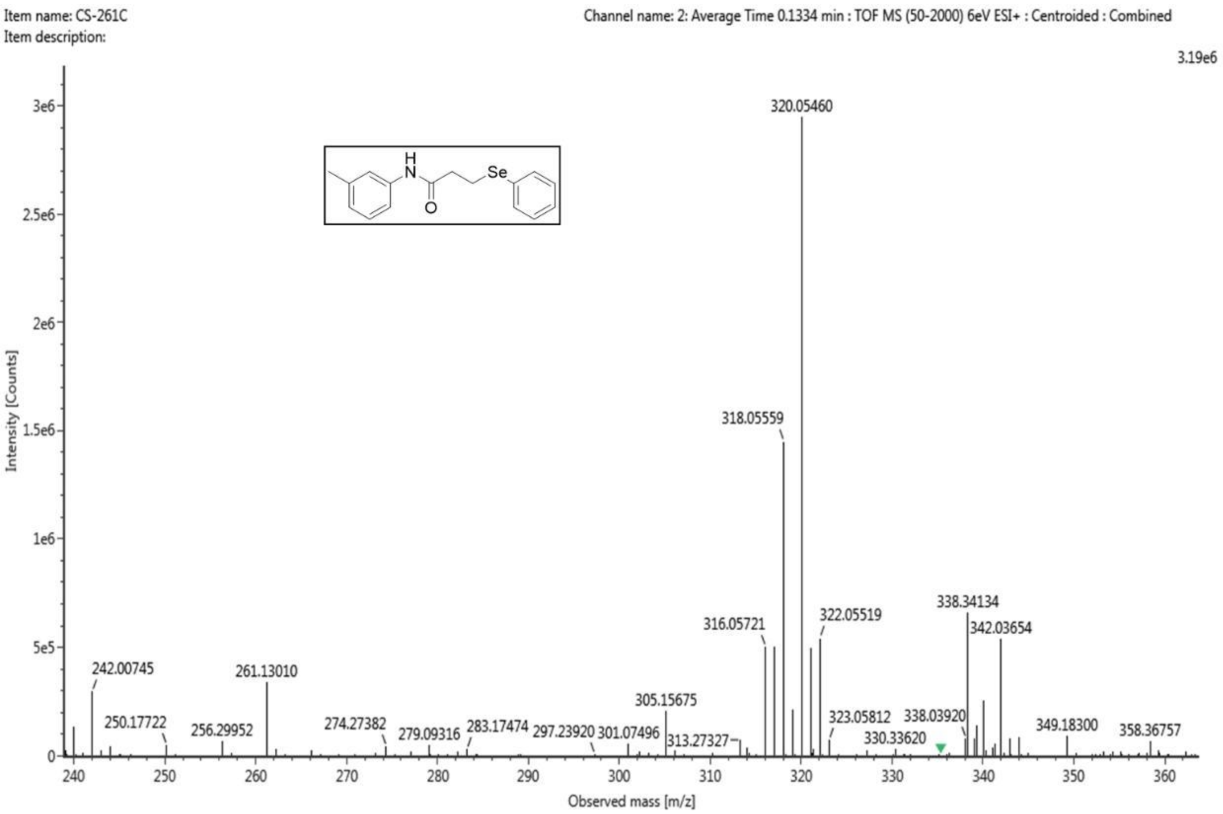

Supplementary Fig. 130. HR-MS of compound **7**

^{1}H NMR (400 MHz, $CDCl_3$, 25°C) of compound **8**

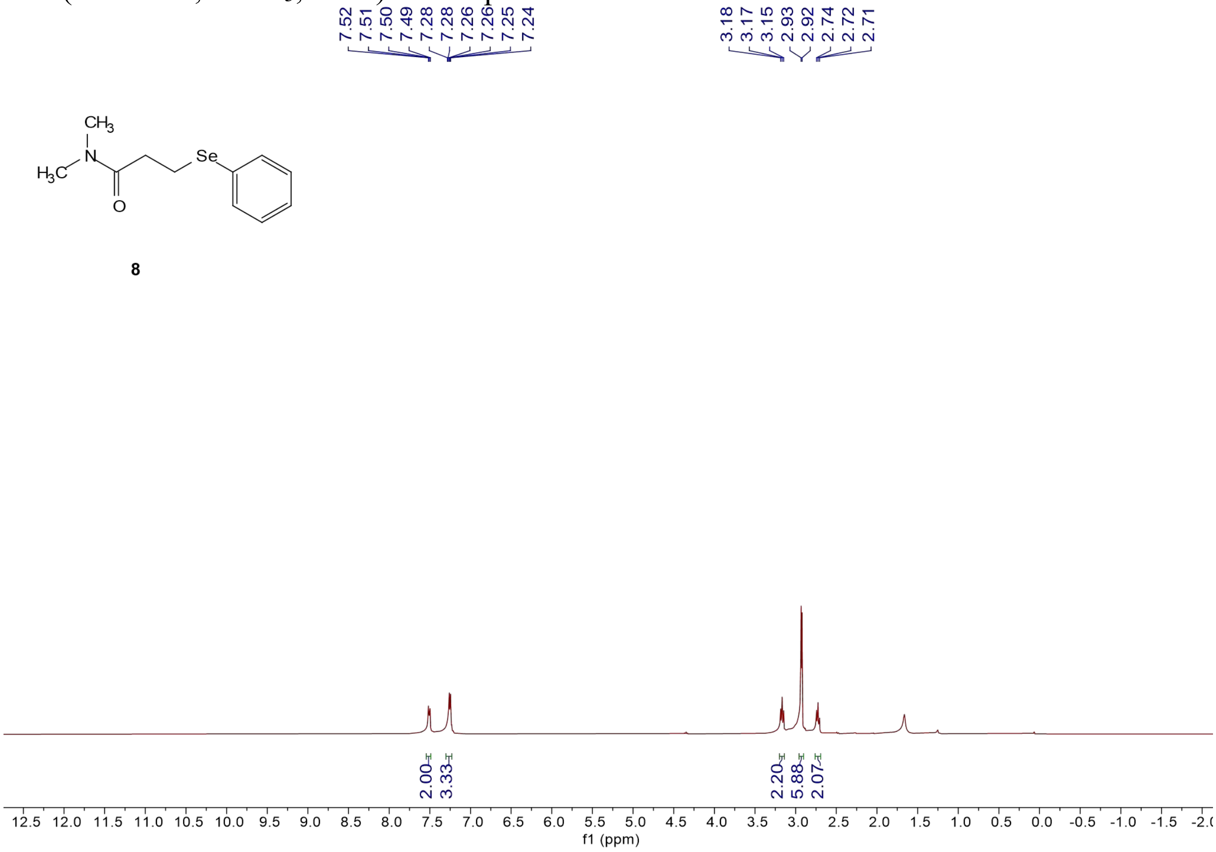

^{13}C NMR (101 MHz, $CDCl_3$, 25°C) of compound **8**

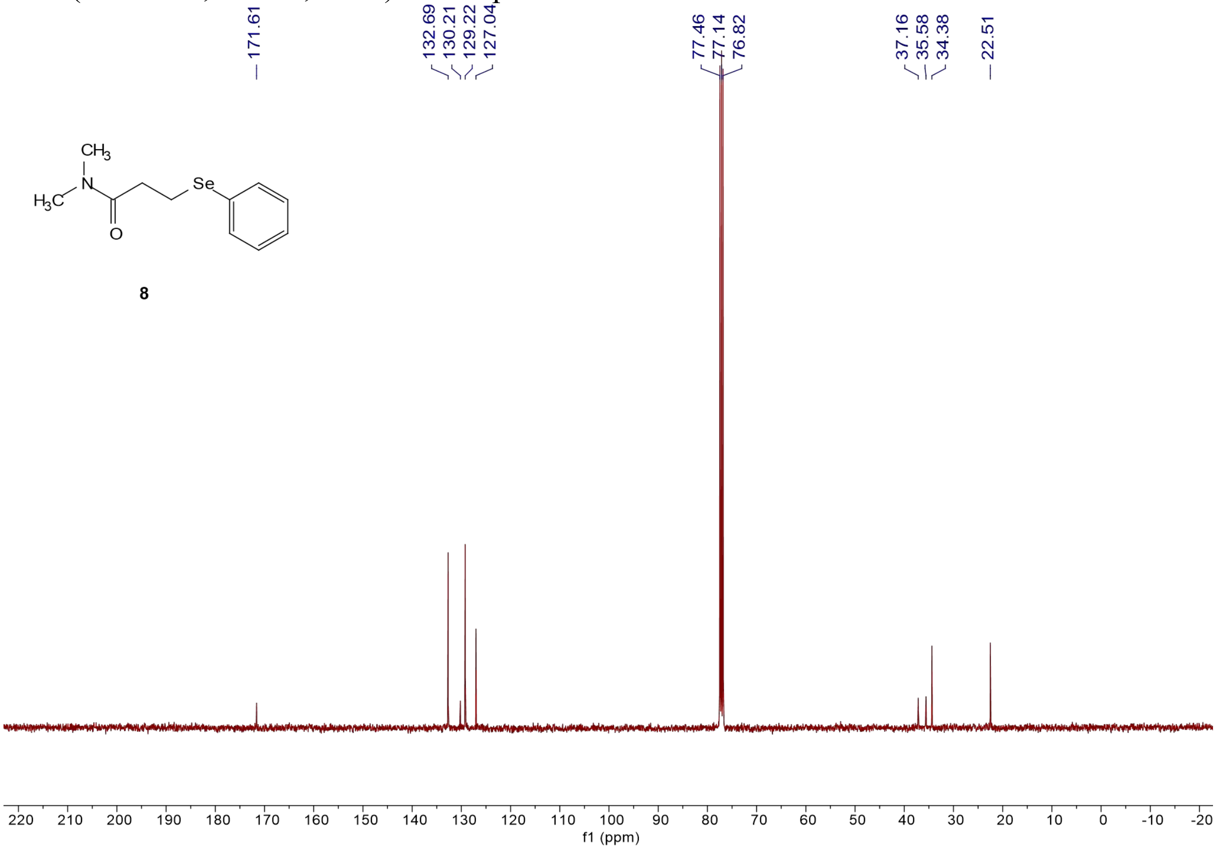

^{77}Se NMR (76 MHz, $CDCl_3$, 25°C) of compound **8**

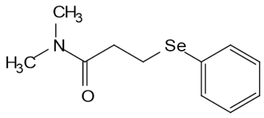

8

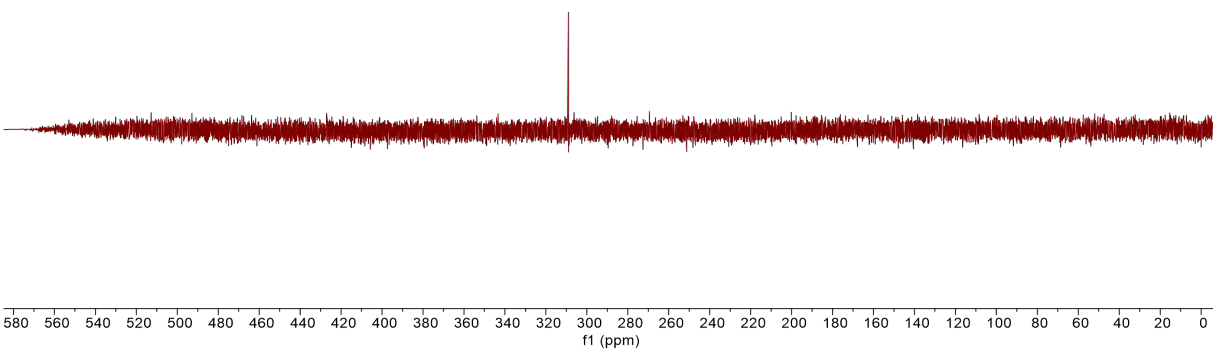

Supplementary Fig. 131. NMR spectra of compound **8**

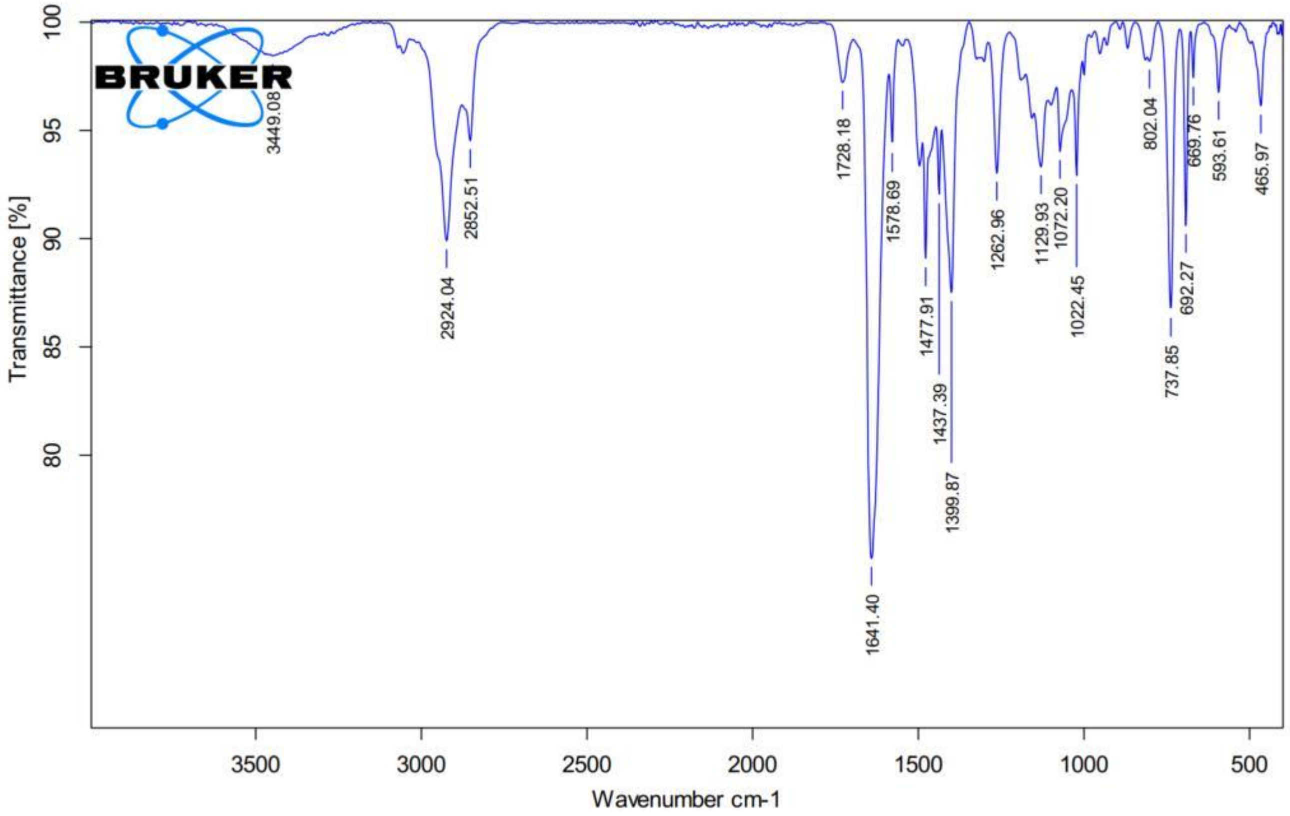

Supplementary Fig. 132. IR of compound **8**

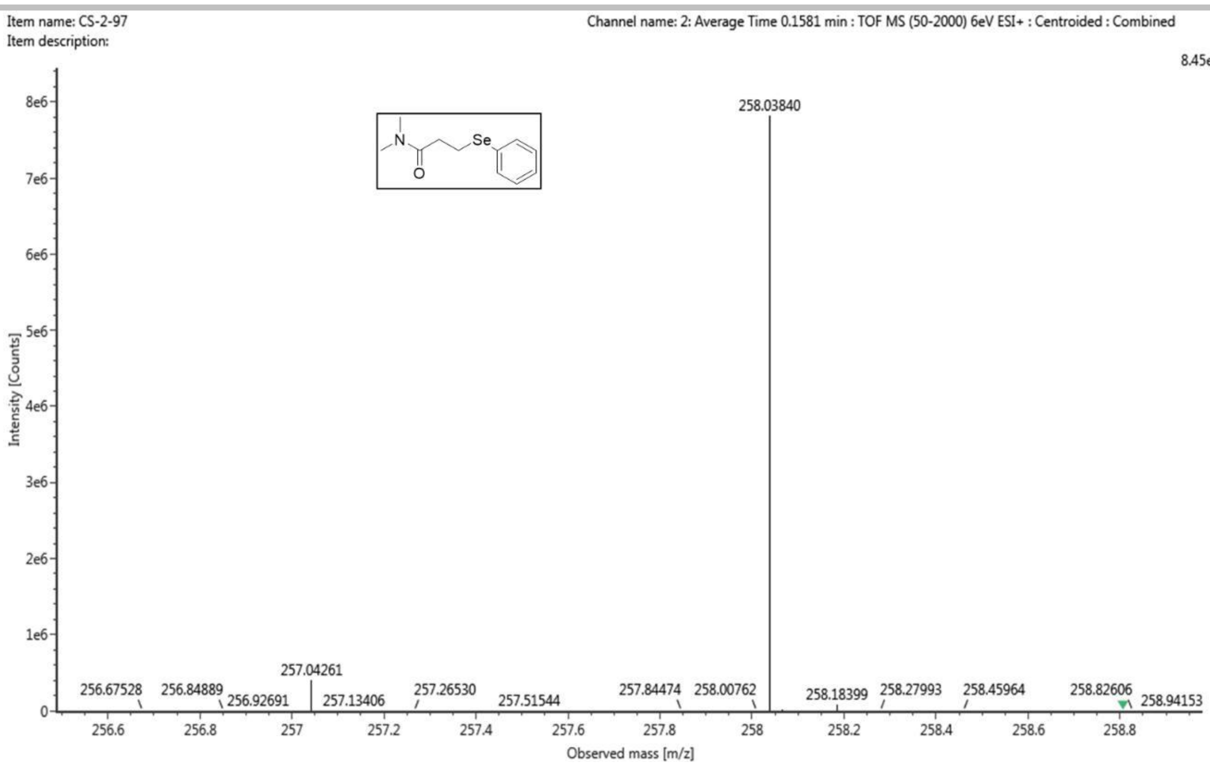

Supplementary Fig. 133. HR-MS of compound **8**

^{1}H NMR (400 MHz, $CDCl_3$, 25°C) of compound **9**

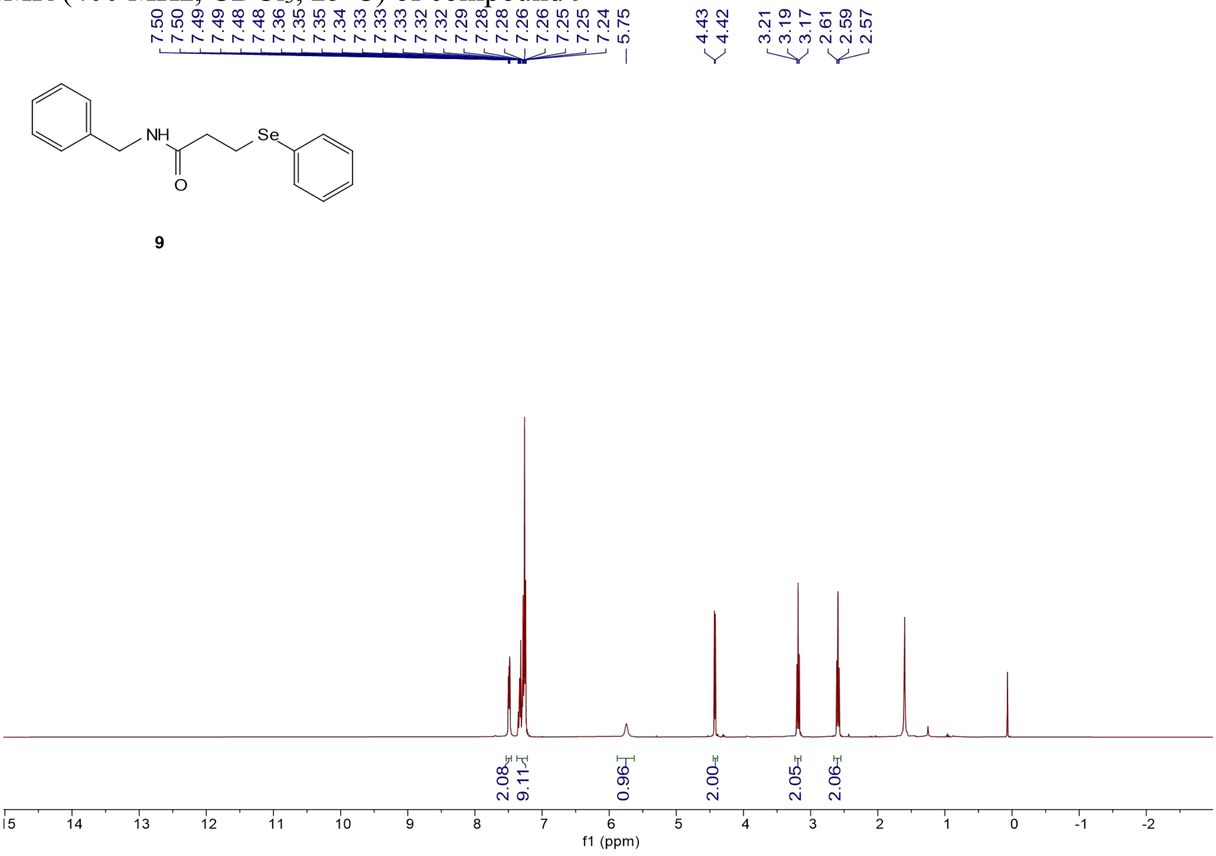

^{13}C NMR (101 MHz, $CDCl_3$, 25°C) of compound **9**

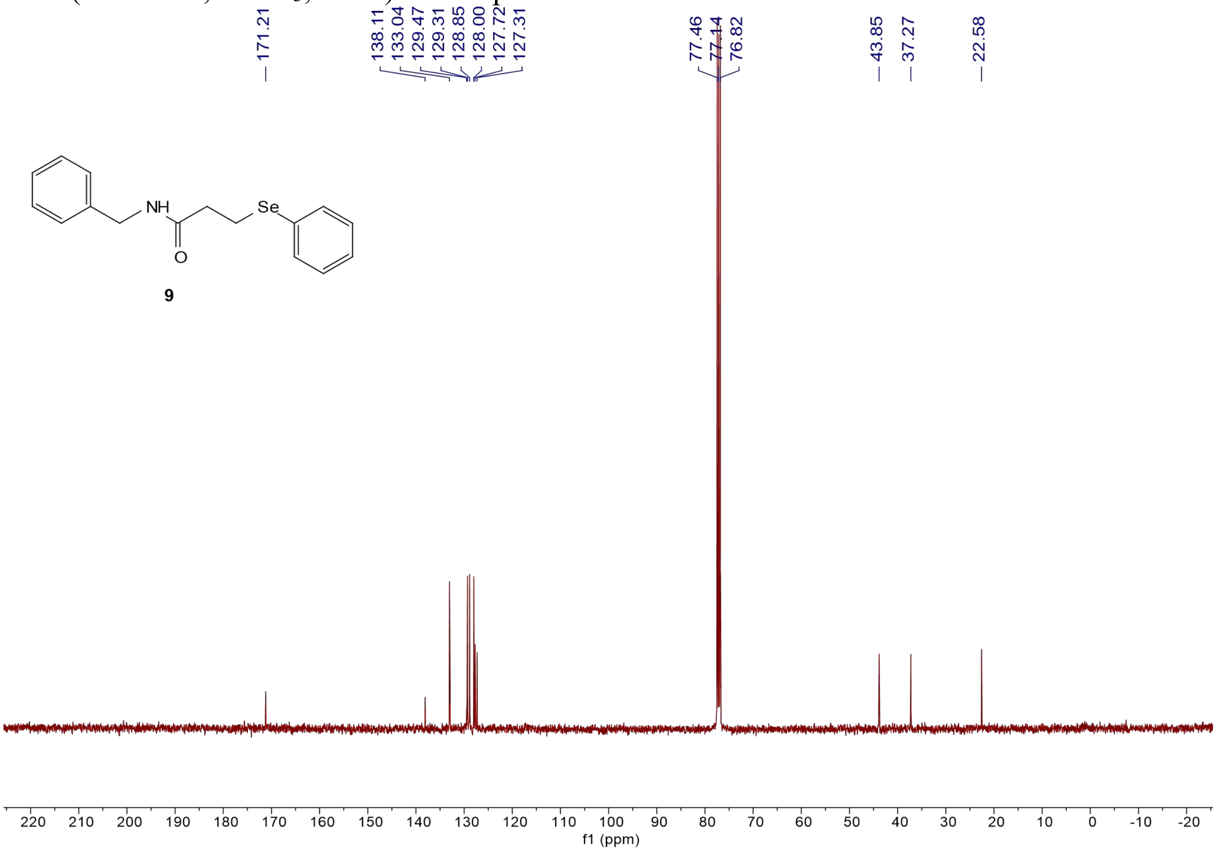

^{77}Se NMR (76 MHz, $CDCl_3$, 25°C) of compound **9**

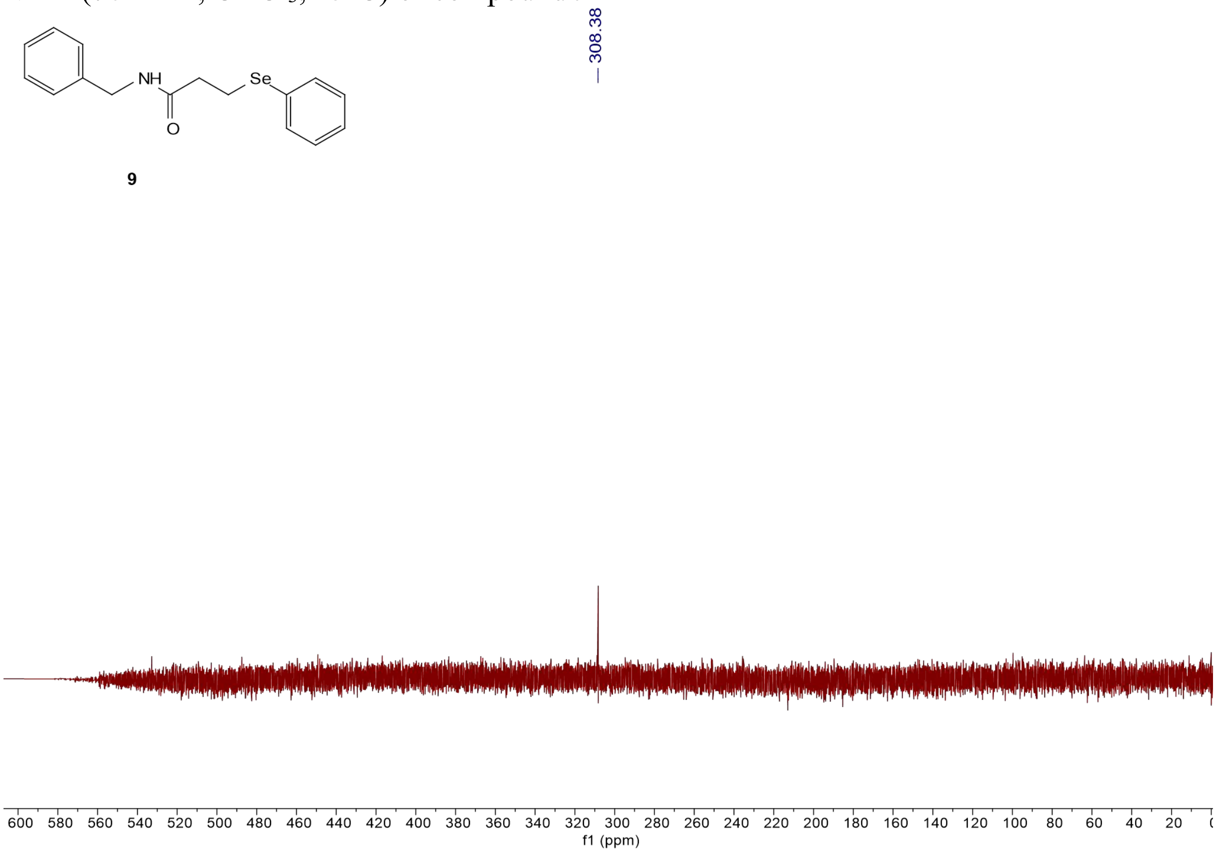

Supplementary Fig. 134. NMR spectra of compound **9**

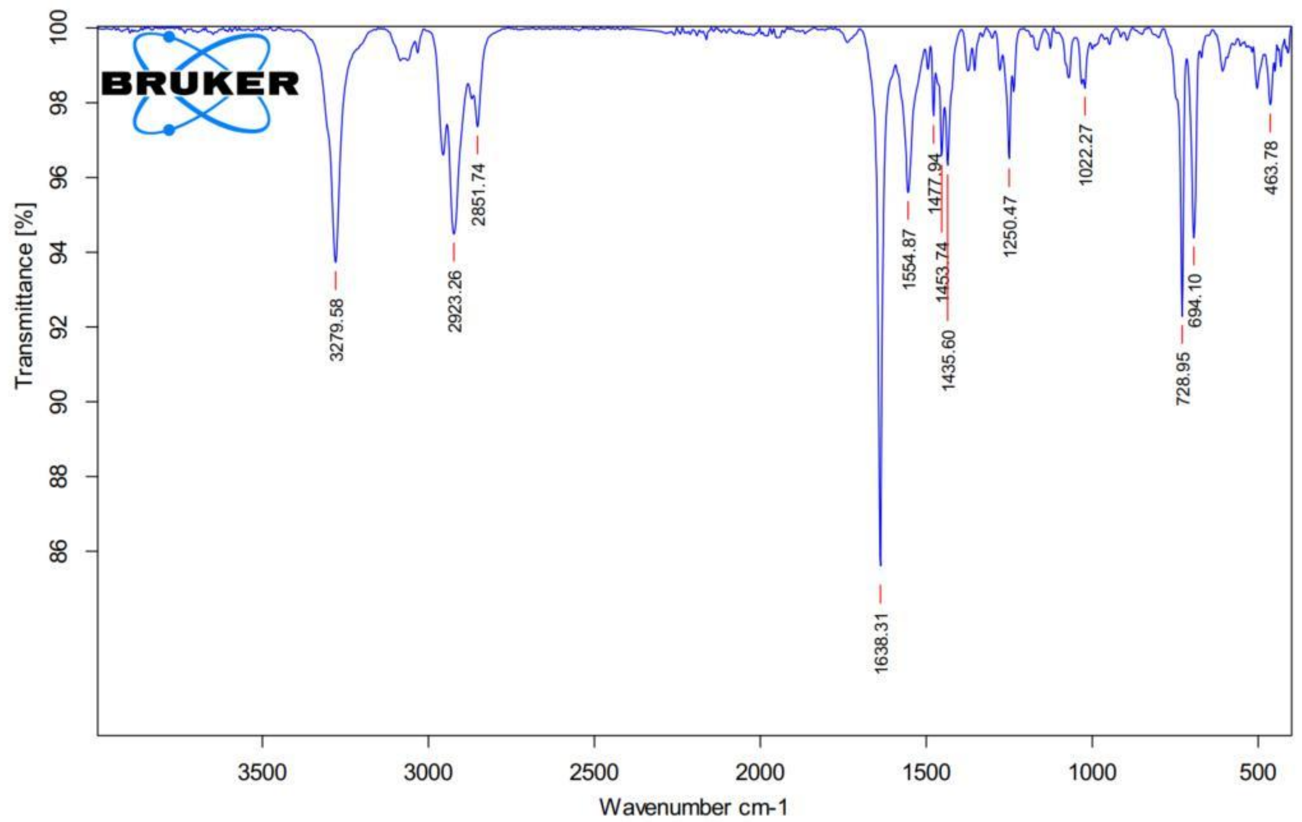

Supplementary Fig. 135. IR of compound **9**

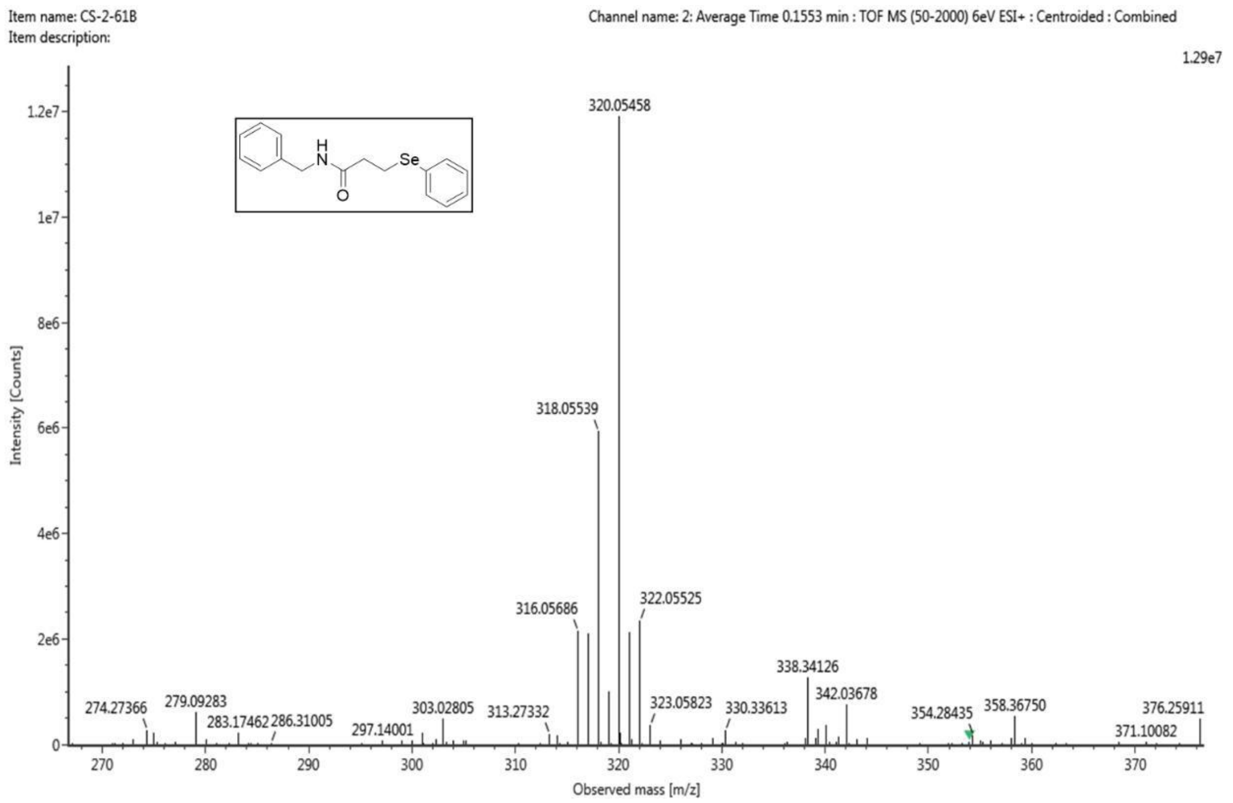

Supplementary Fig. 136. HR-MS of compound **9**

^{1}H NMR (400 MHz, $CDCl_3$, 25°C) of compound **10**

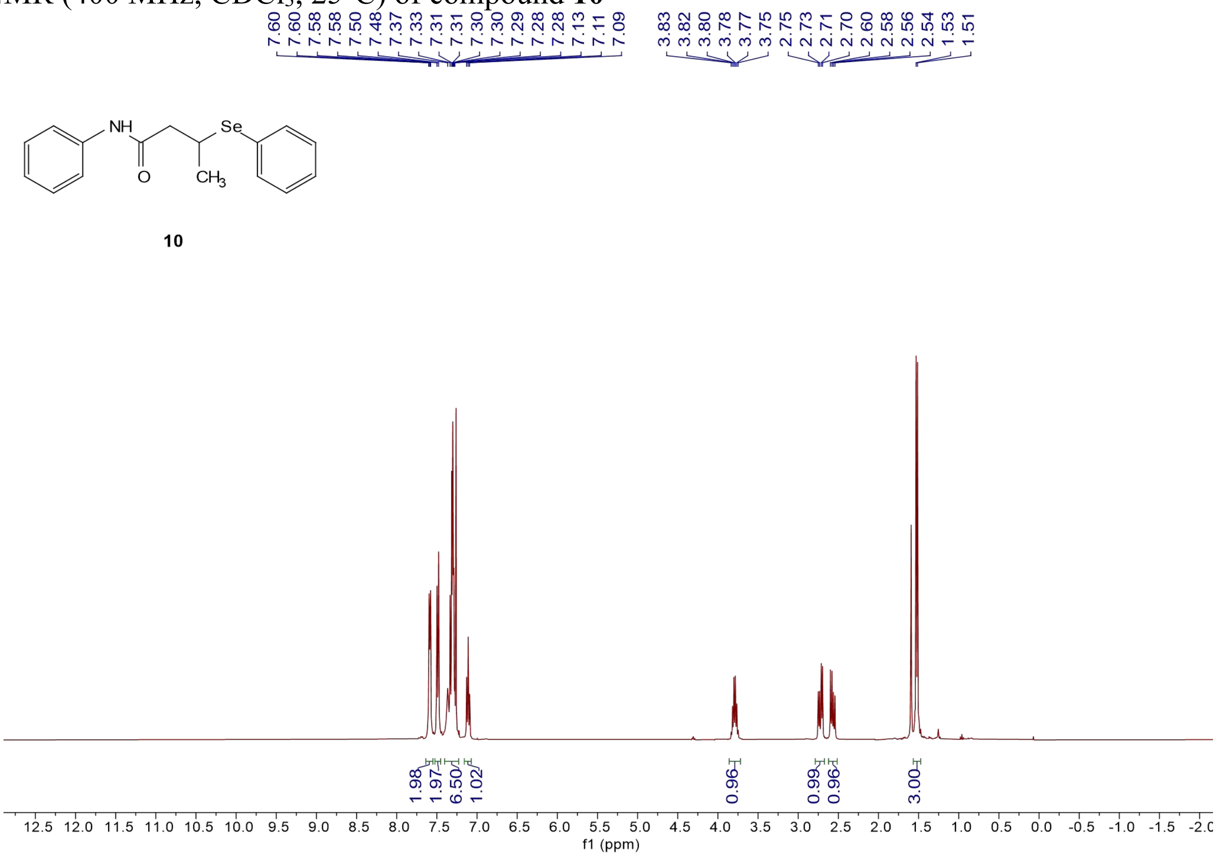

^{13}C NMR (101 MHz, $CDCl_3$, 25°C) of compound **10**

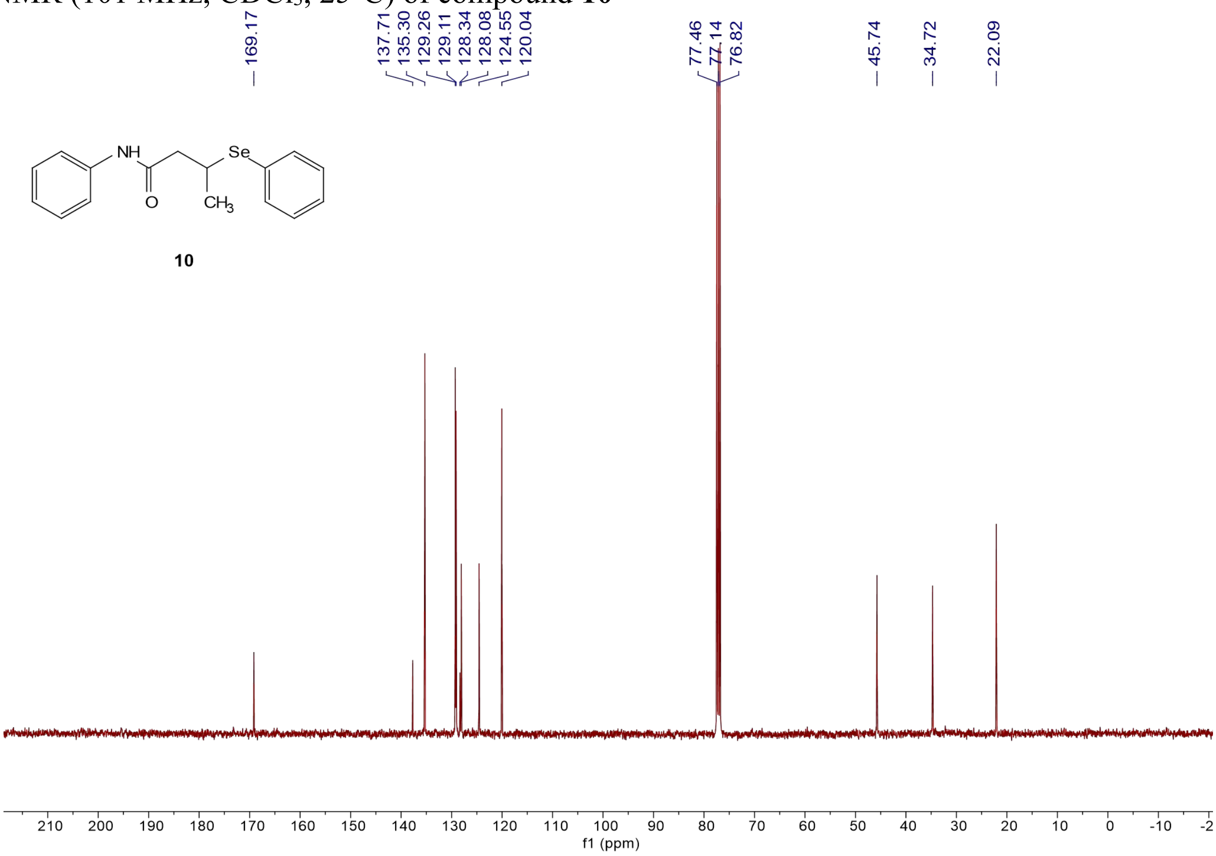

^{77}Se NMR (76 MHz, $CDCl_3$, 25°C) of compound **10**

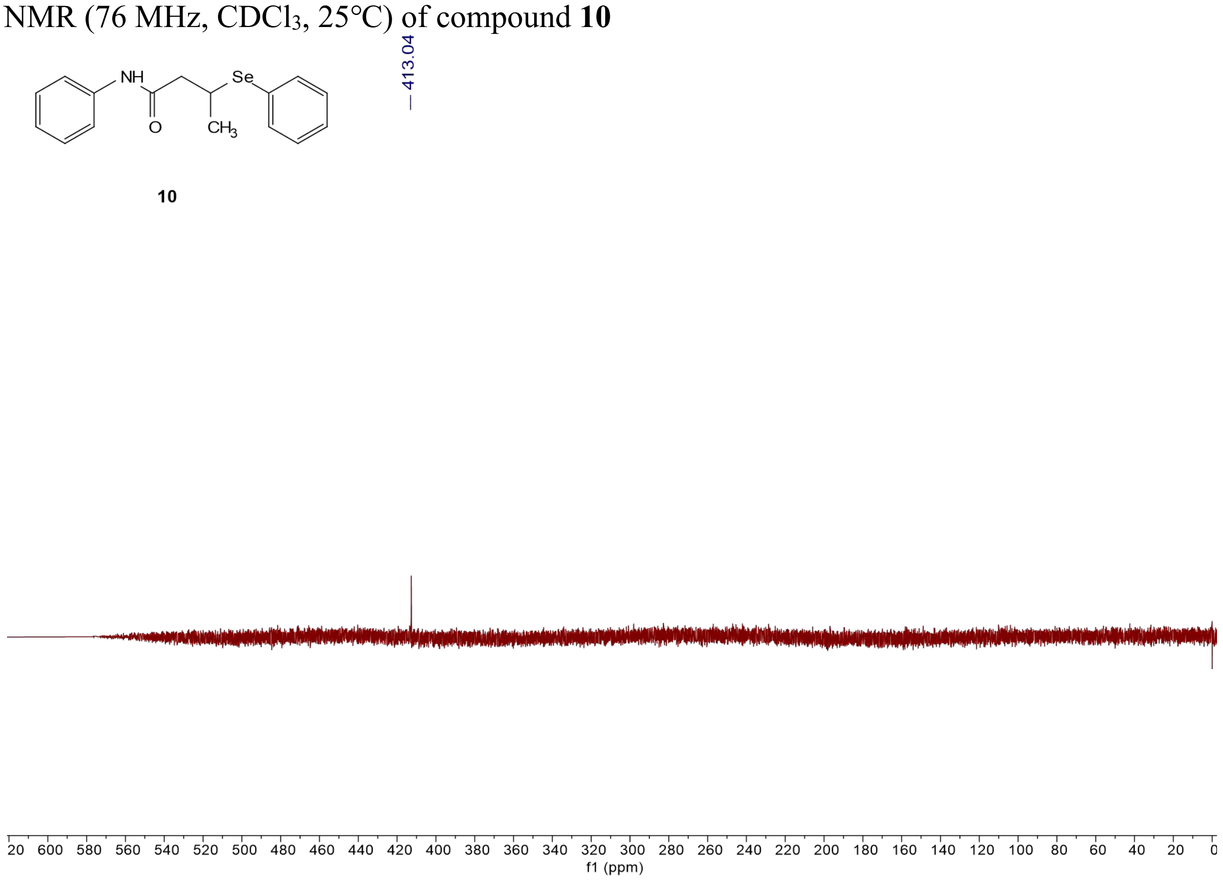

Supplementary Fig. 137. NMR spectra of compound **10**

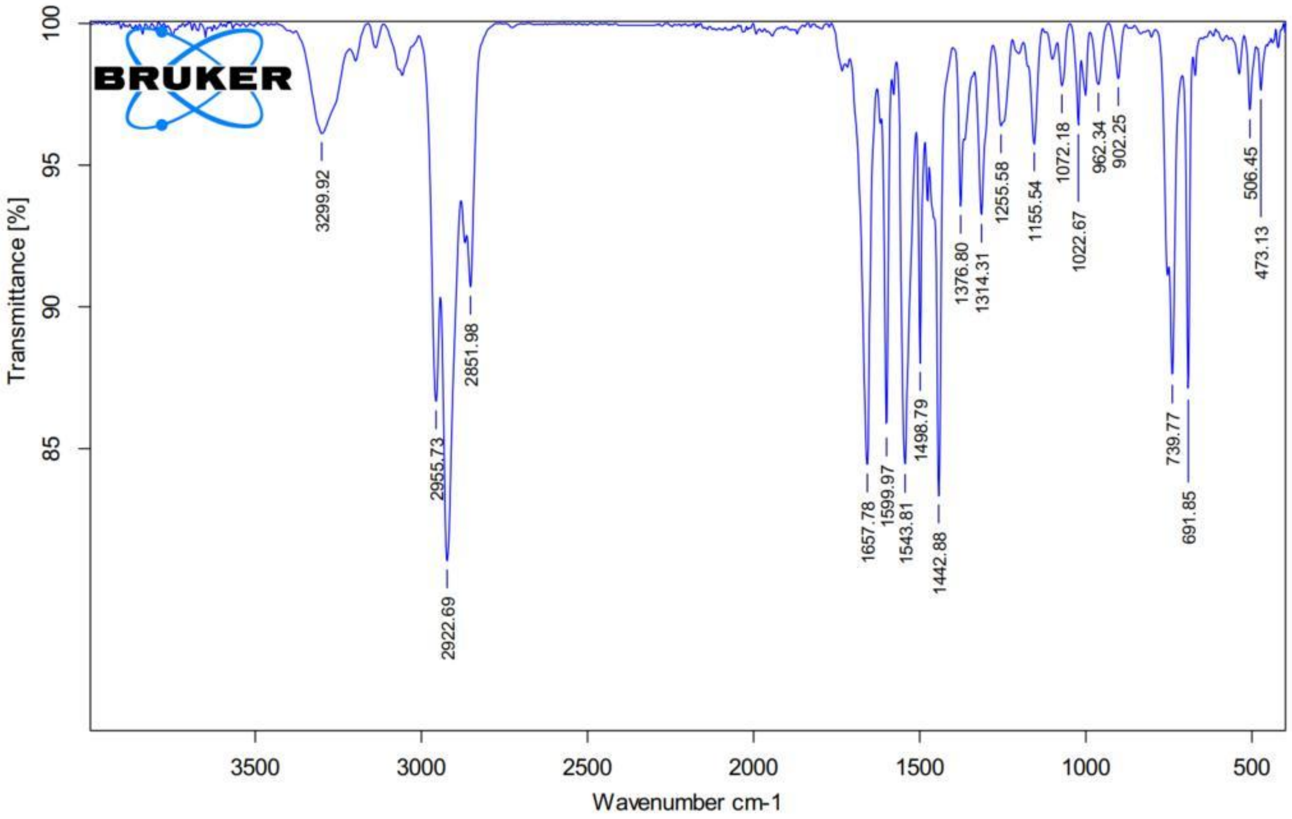

Supplementary Fig. 138. IR of compound **10**

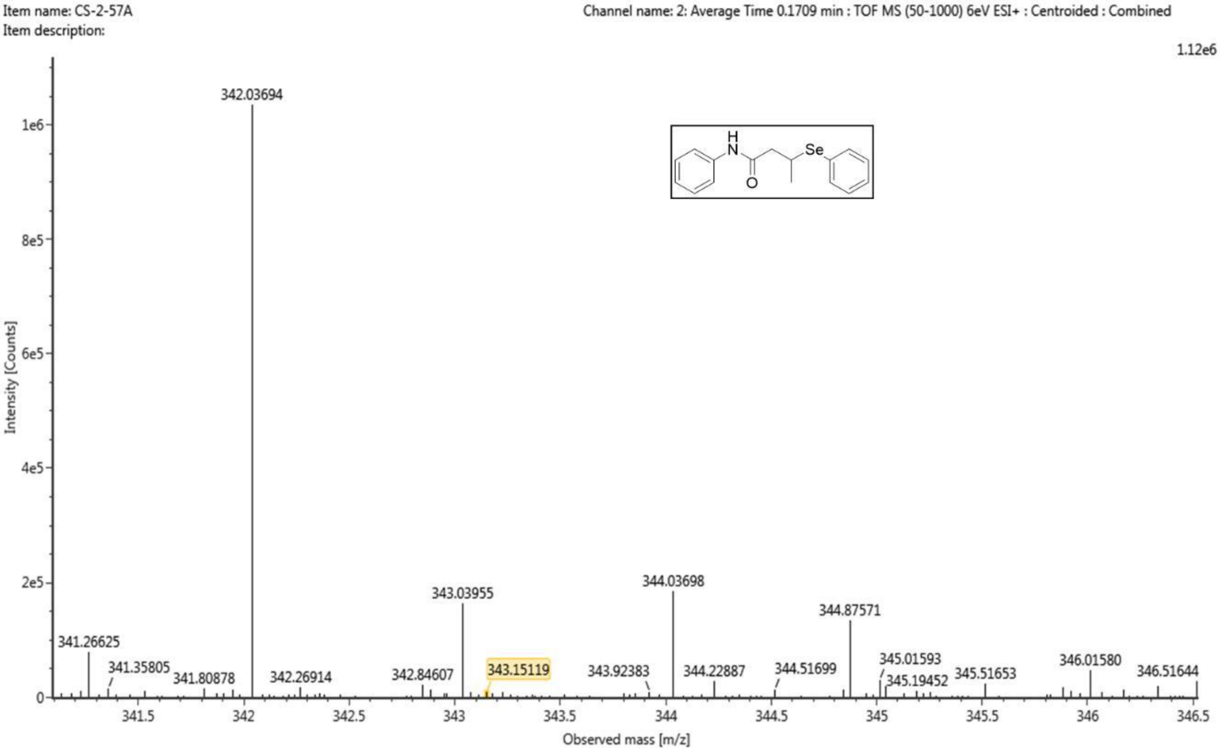

Supplementary Fig. 139. HR-MS of compound **10**

^{1}H NMR (400 MHz, $CDCl_3$, 25°C) of compound **11a**

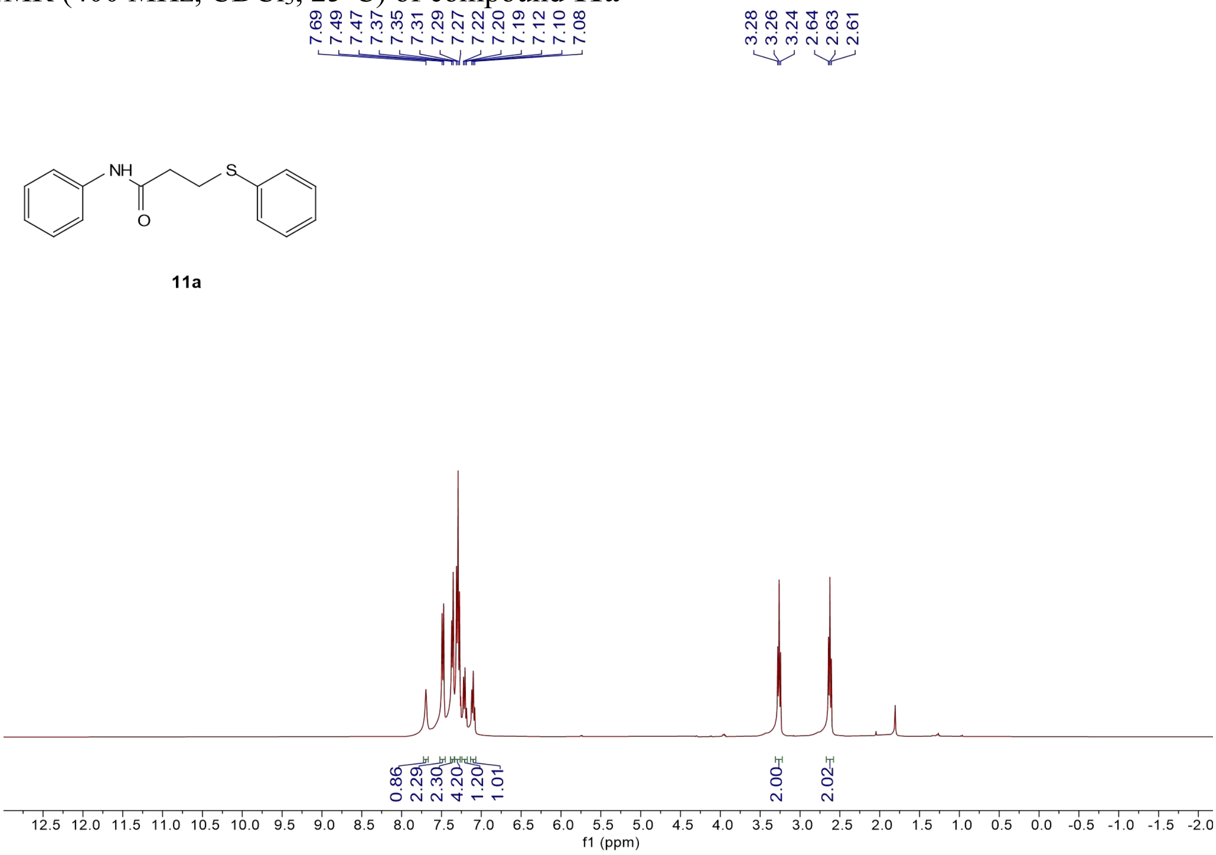

^{13}C NMR (101 MHz, $CDCl_3$, 25°C) of compound **11a**

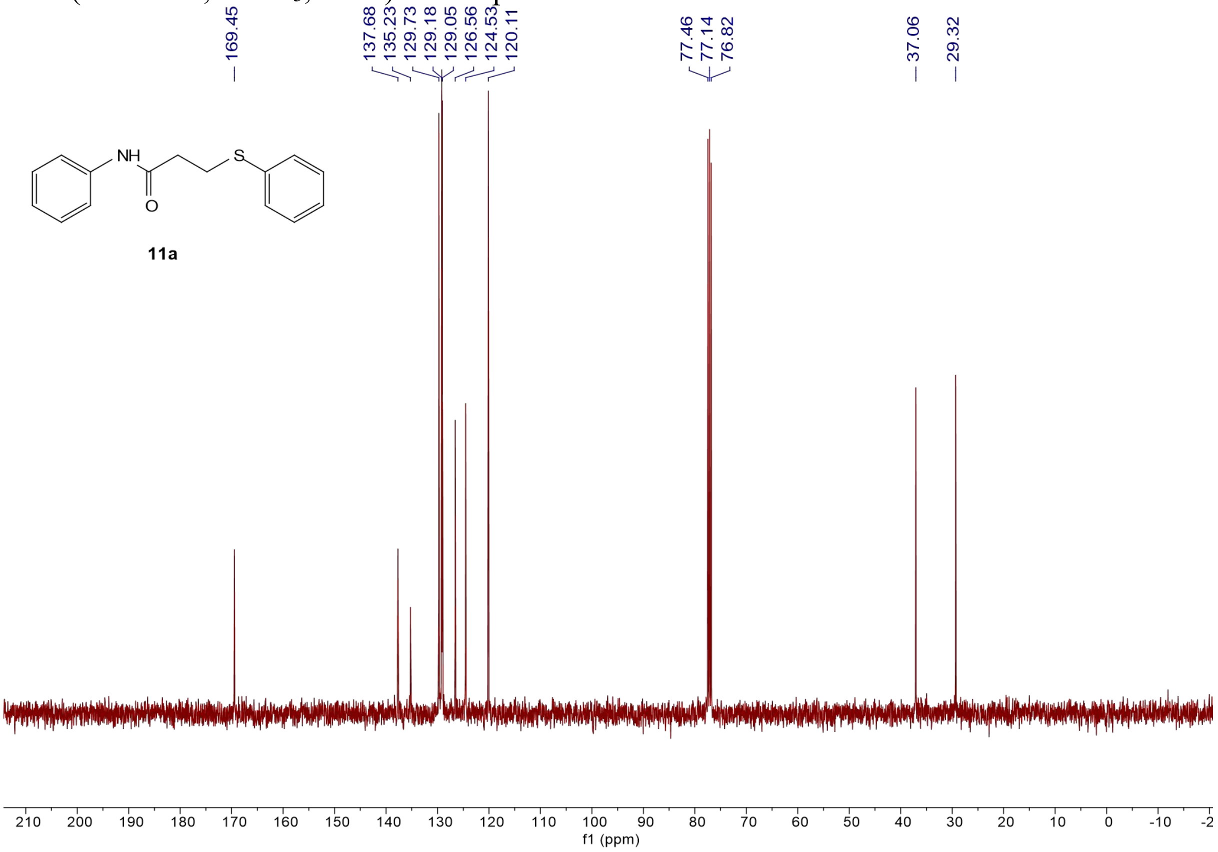

Supplementary Fig. 140. NMR spectra of compound **11a**

^{1}H NMR (400 MHz, $CDCl_3$, 25°C) of compound **11b**

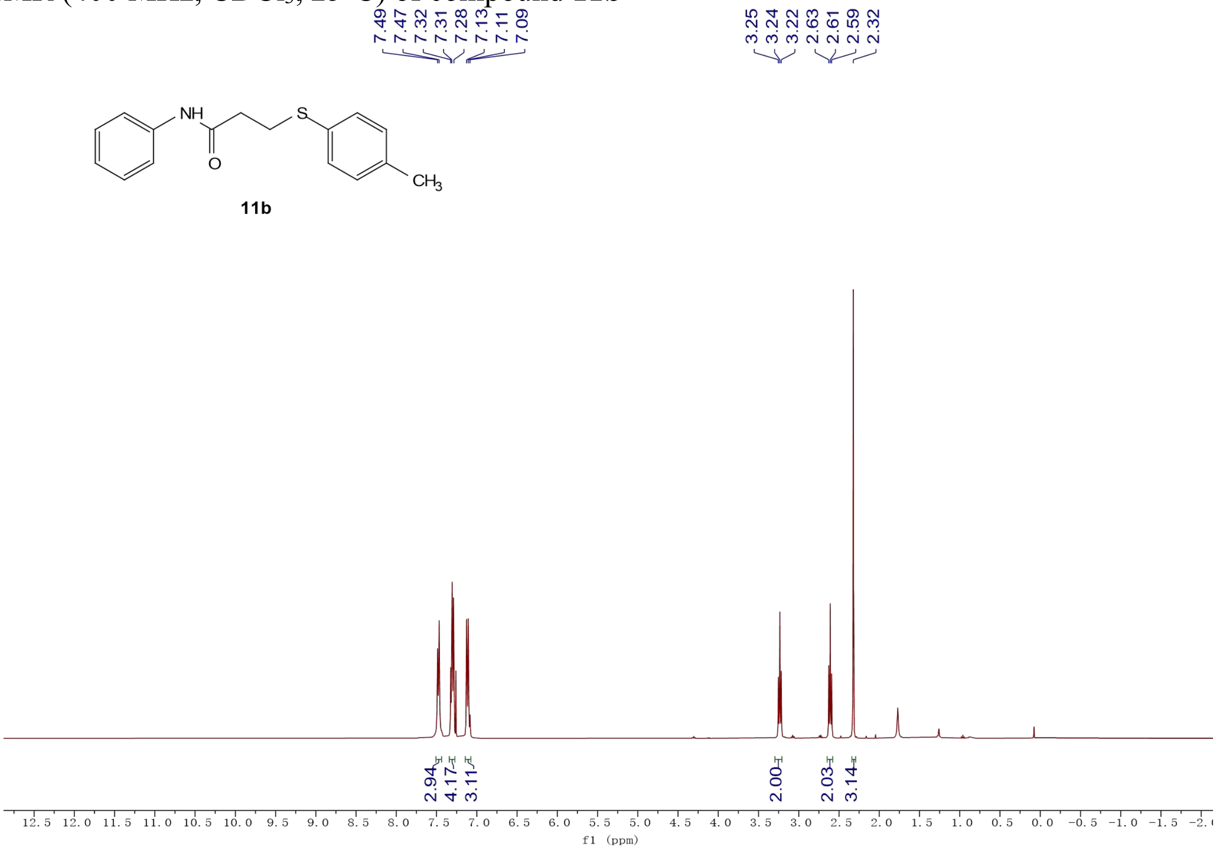

^{13}C NMR (101 MHz, $CDCl_3$, 25°C) of compound **11b**

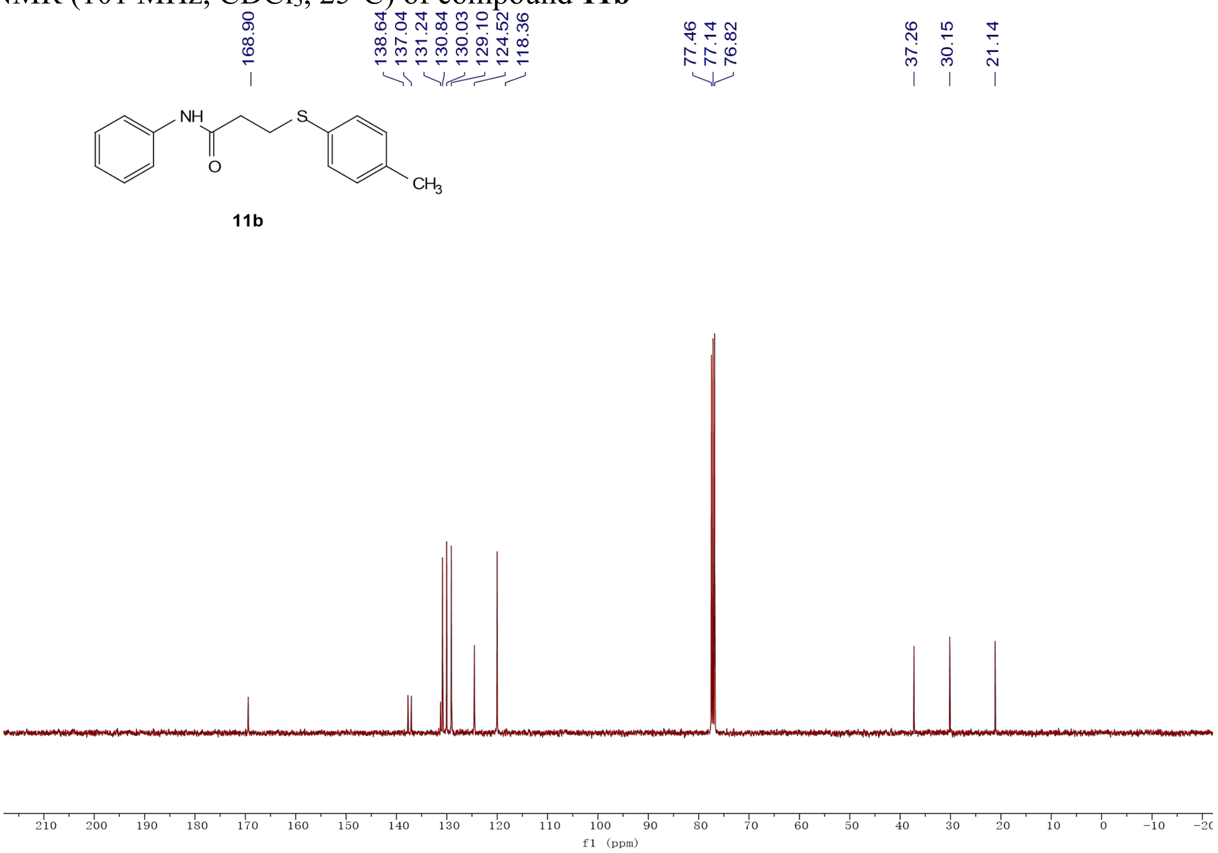

Supplementary Fig. 141. NMR spectra of compound **11b**

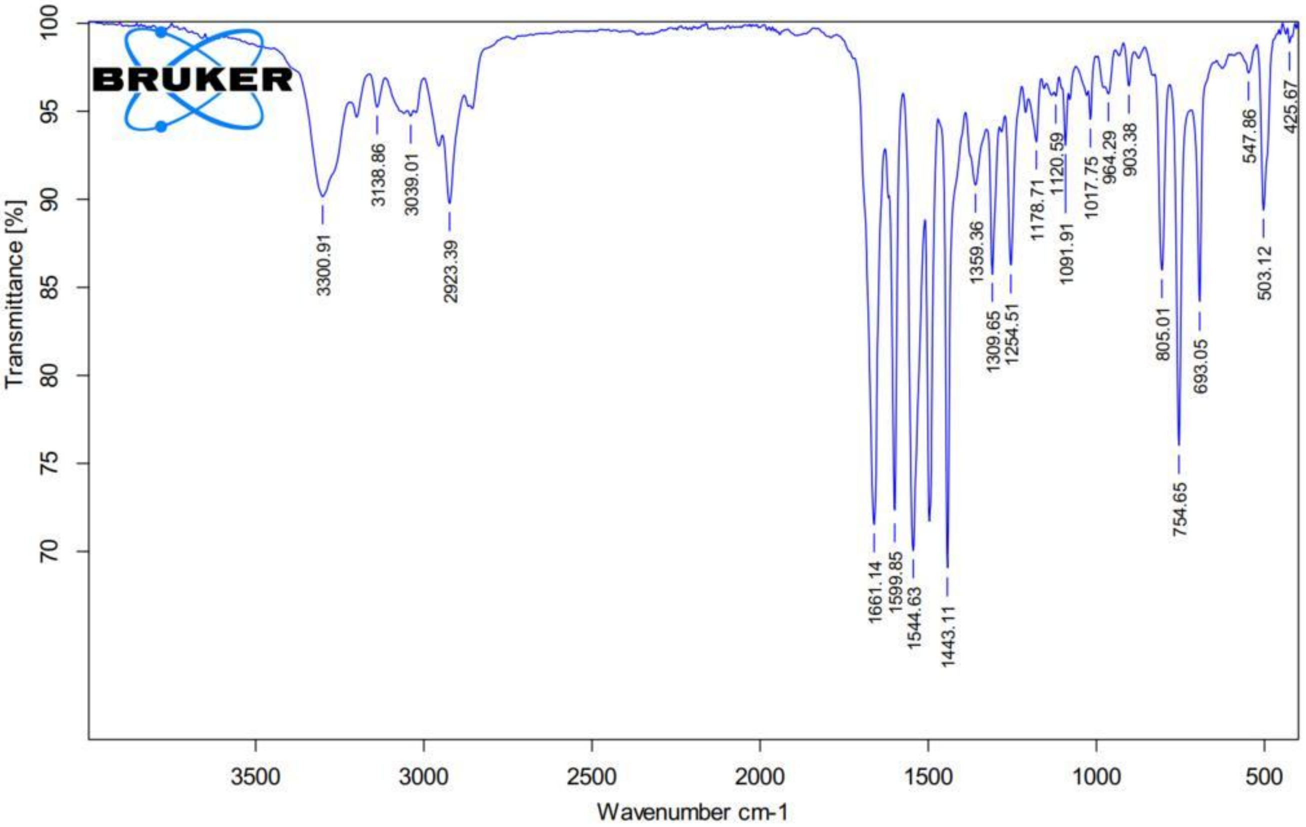

Supplementary Fig. 142. IR of compound **11b**

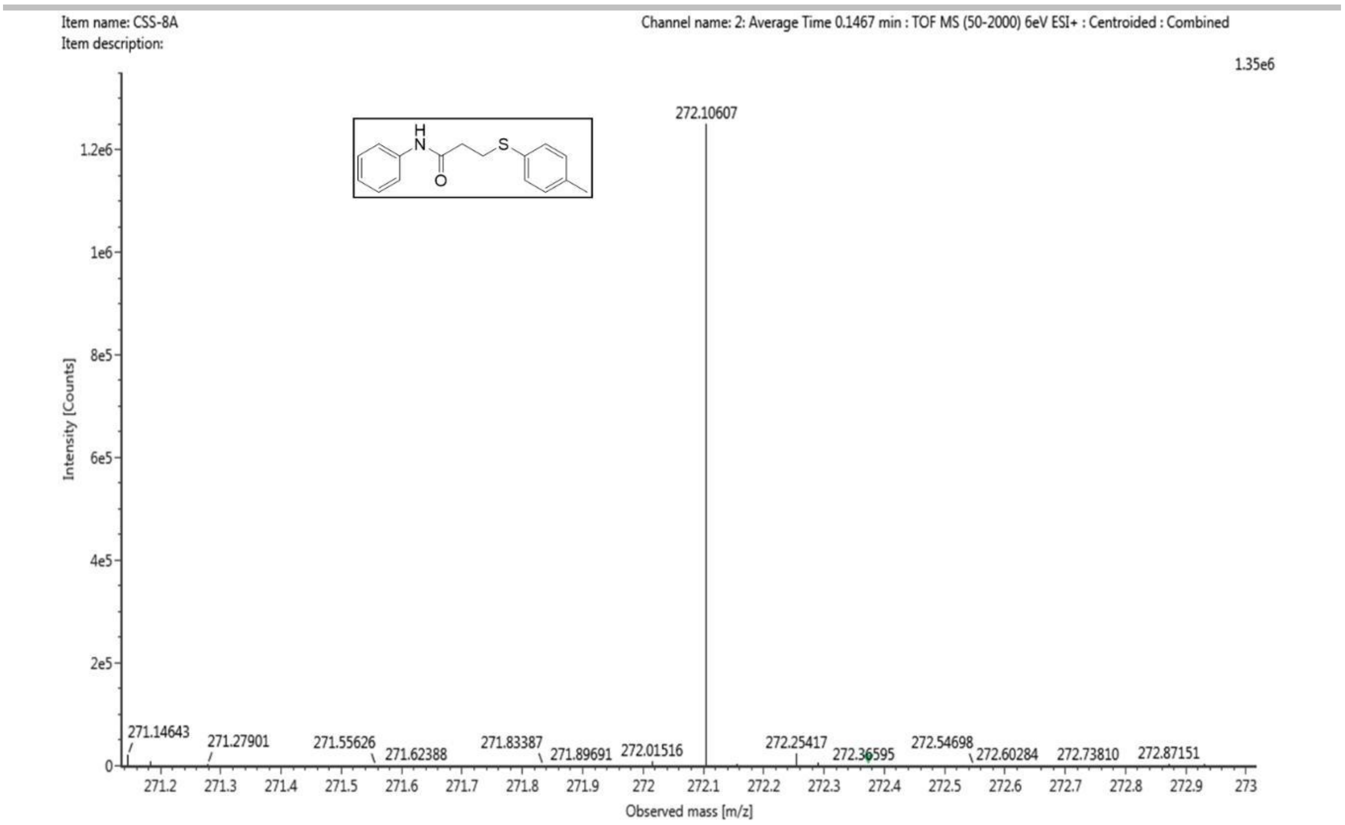

Supplementary Fig. 143. HR-MS of compound **11b**

^{1}H NMR (400 MHz, $CDCl_3$, 25°C) of compound **11c**

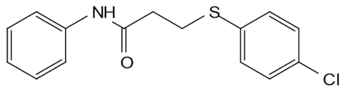

11c

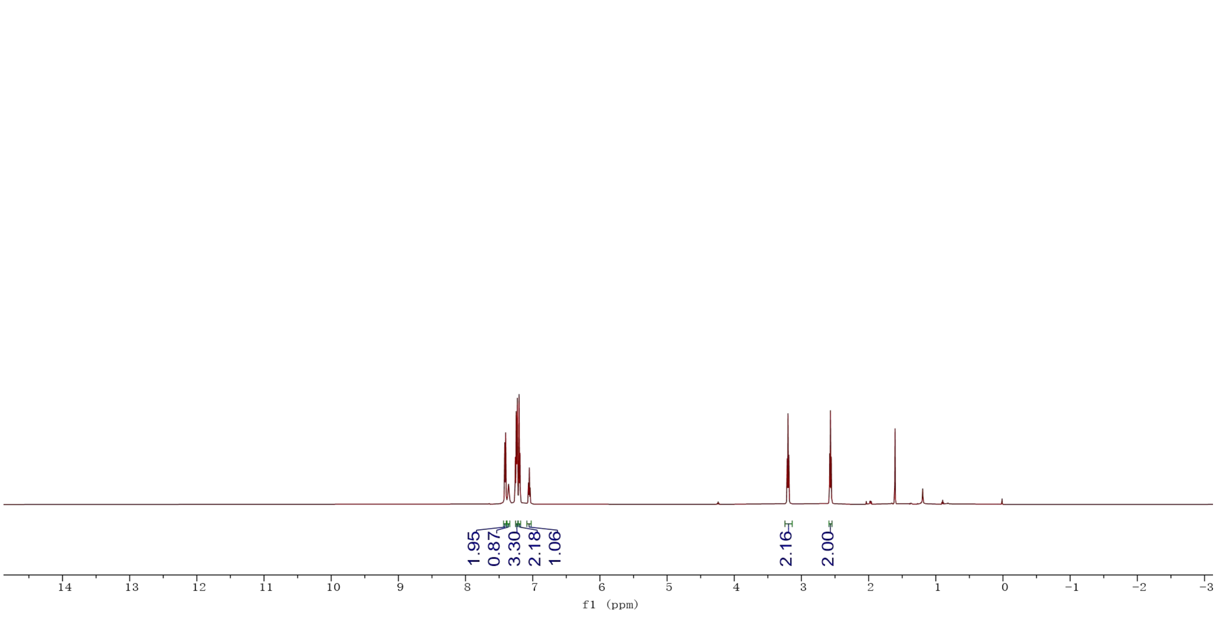

^{13}C NMR (150 MHz, $CDCl_3$, 25°C) of compound **11c**

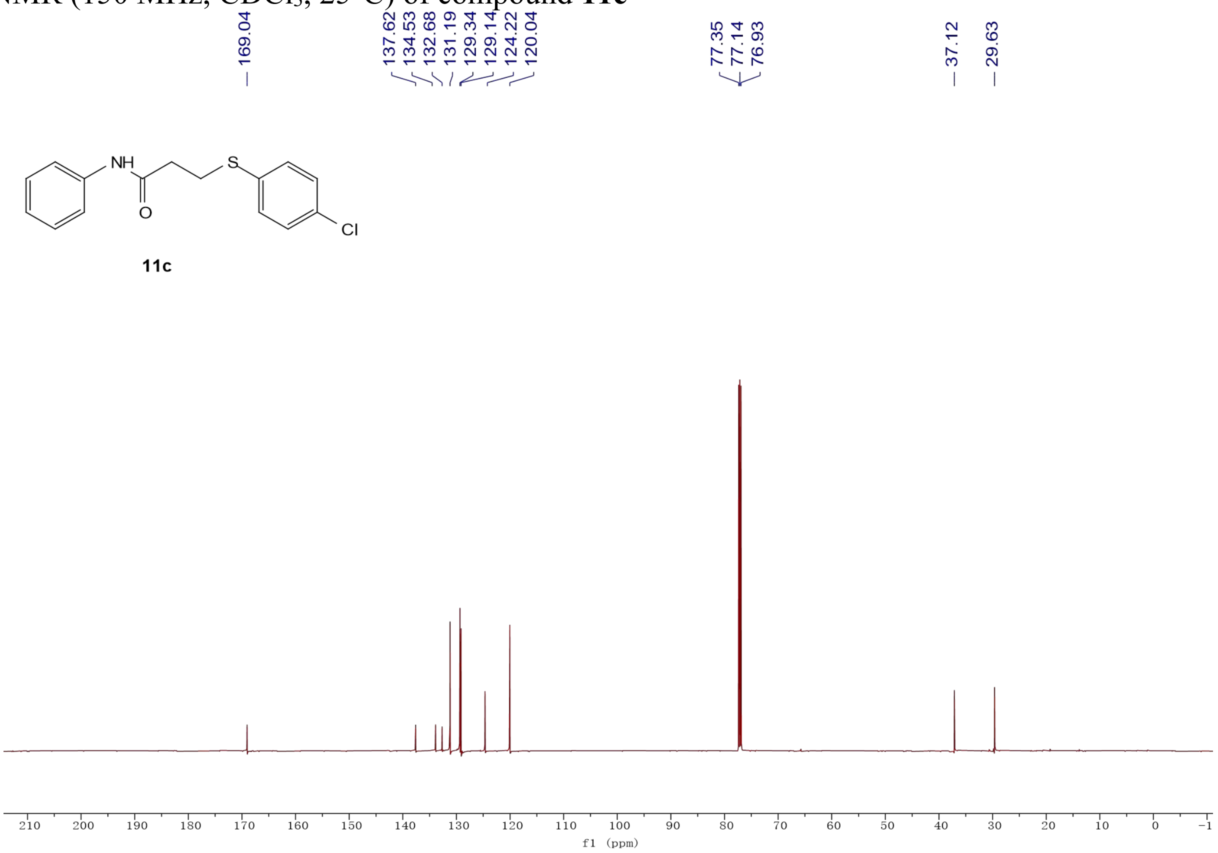

Supplementary Fig. 144. NMR of compound **11c**

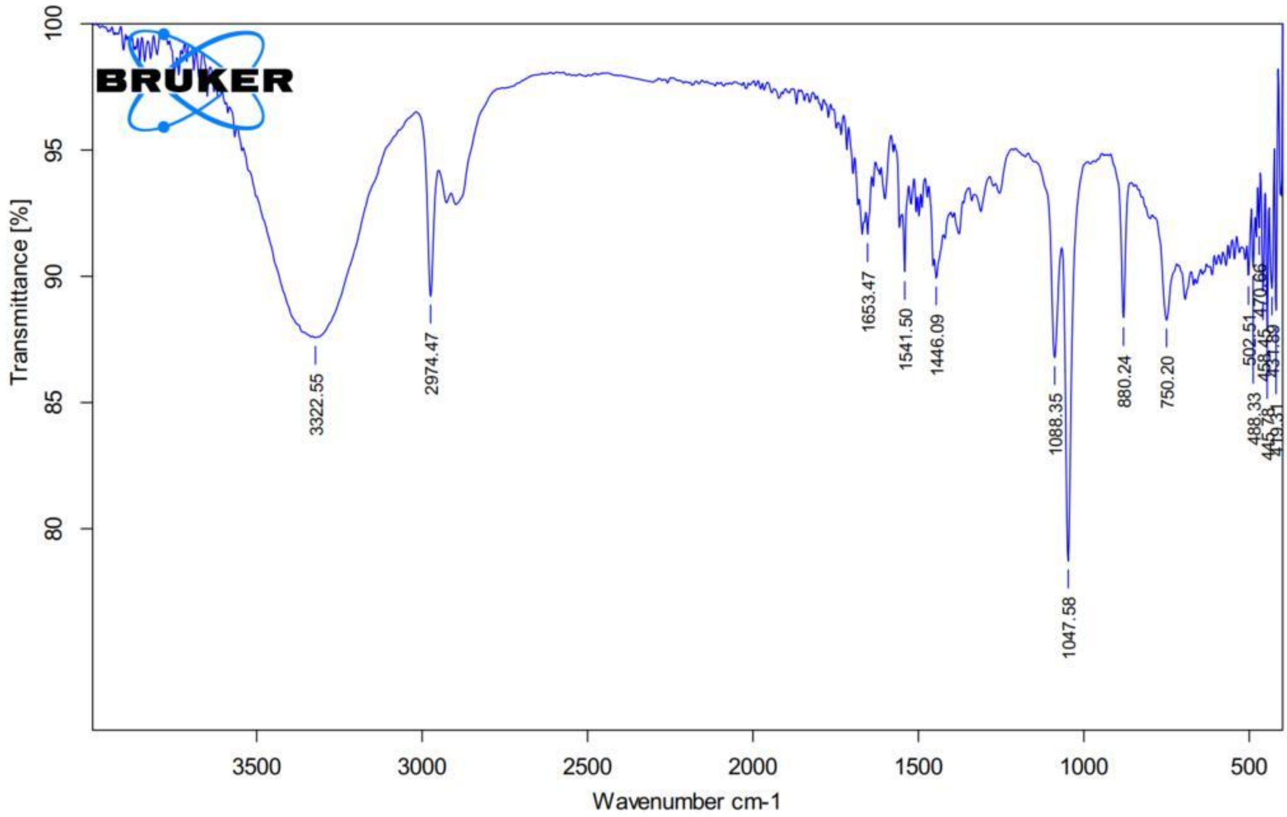

Supplementary Fig. 145. IR of compound **11c**

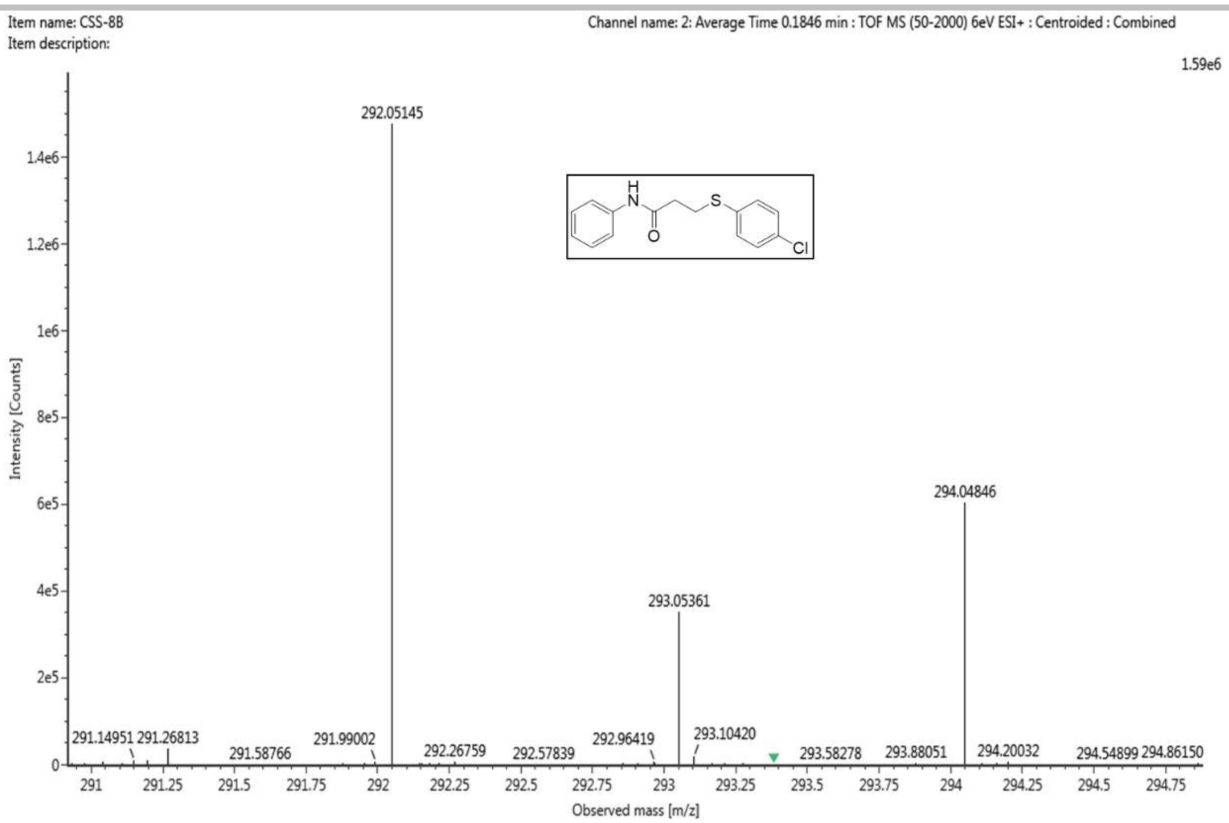

Supplementary Fig. 146. HR-MS of compound **11c**

^{1}H NMR (400 MHz, $CDCl_3$, 25°C) of compound **11d**

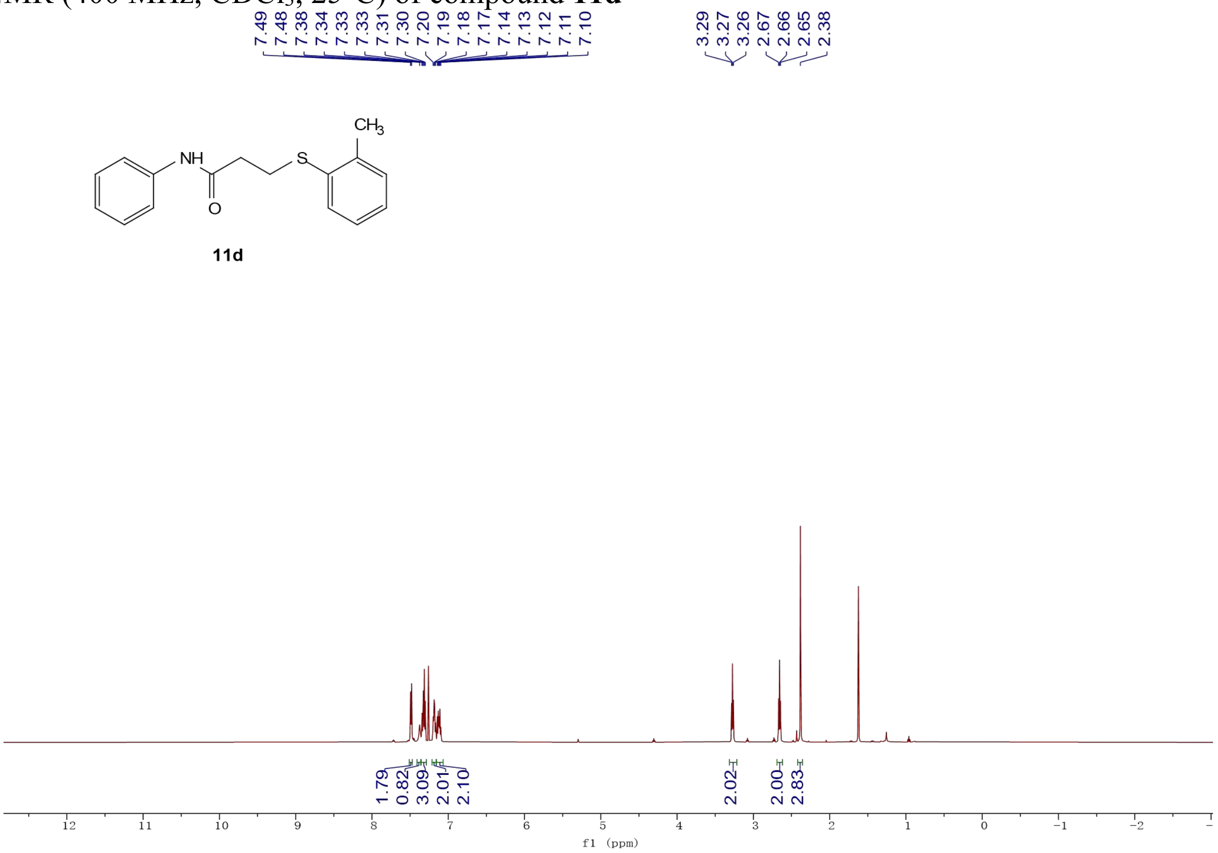

^{13}C NMR (150 MHz, $CDCl_3$, 25°C) of compound **11d**

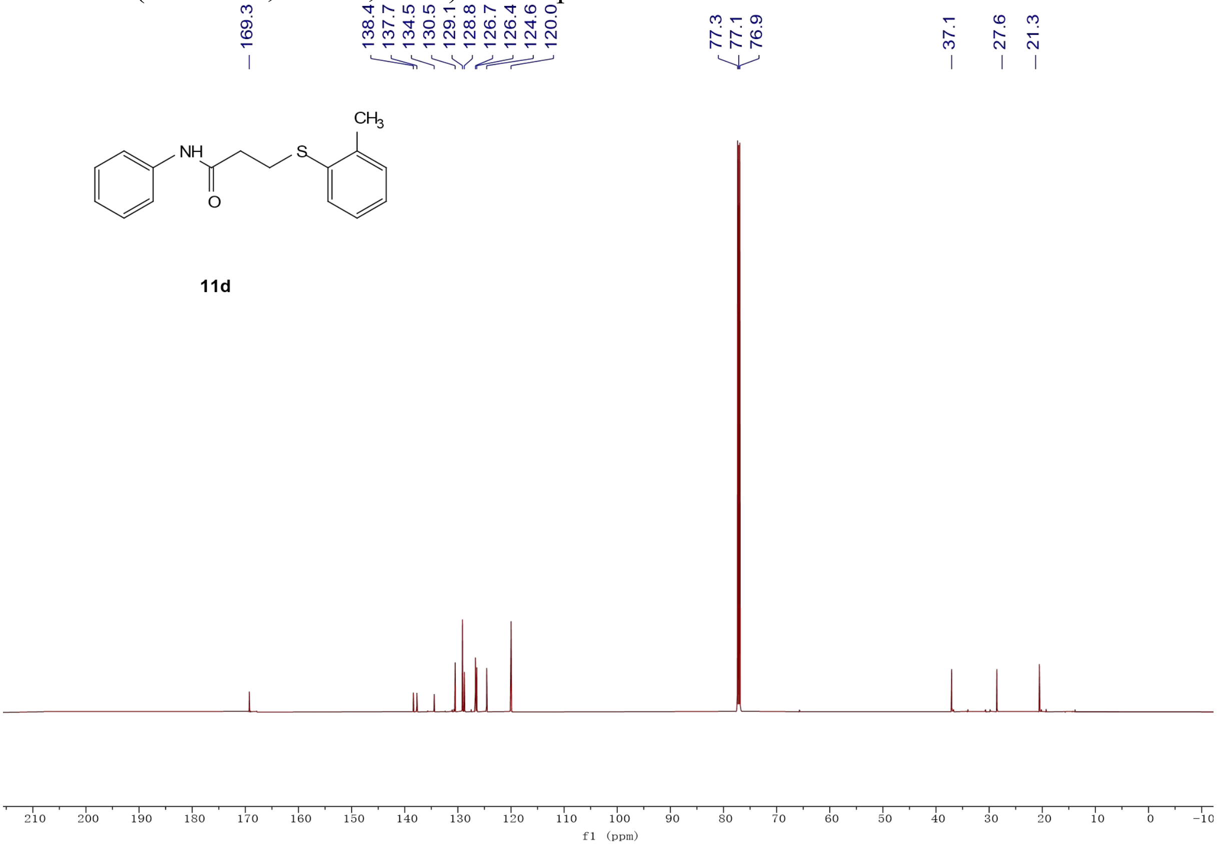

Supplementary Fig. 147. NMR of compound **11d**

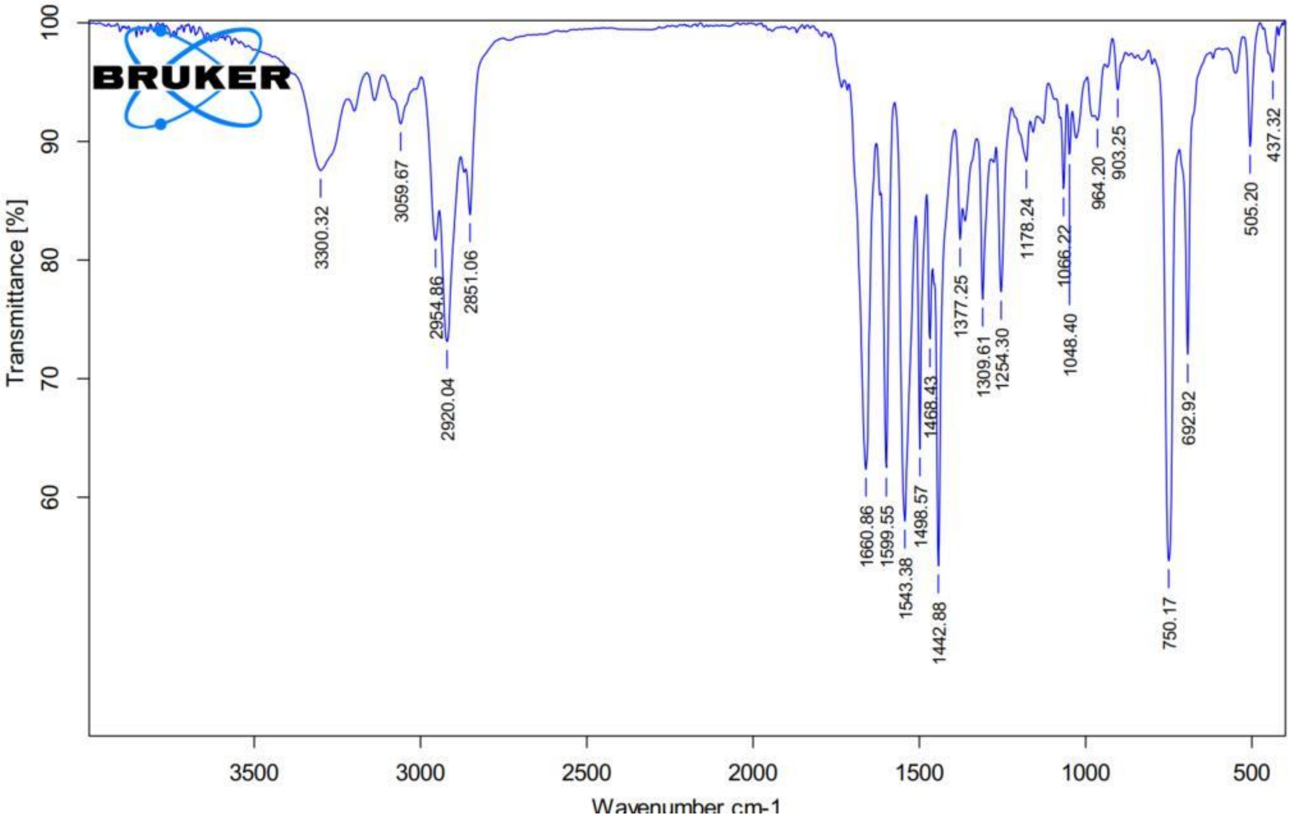

Supplementary Fig. 148. IR of compound **11d**

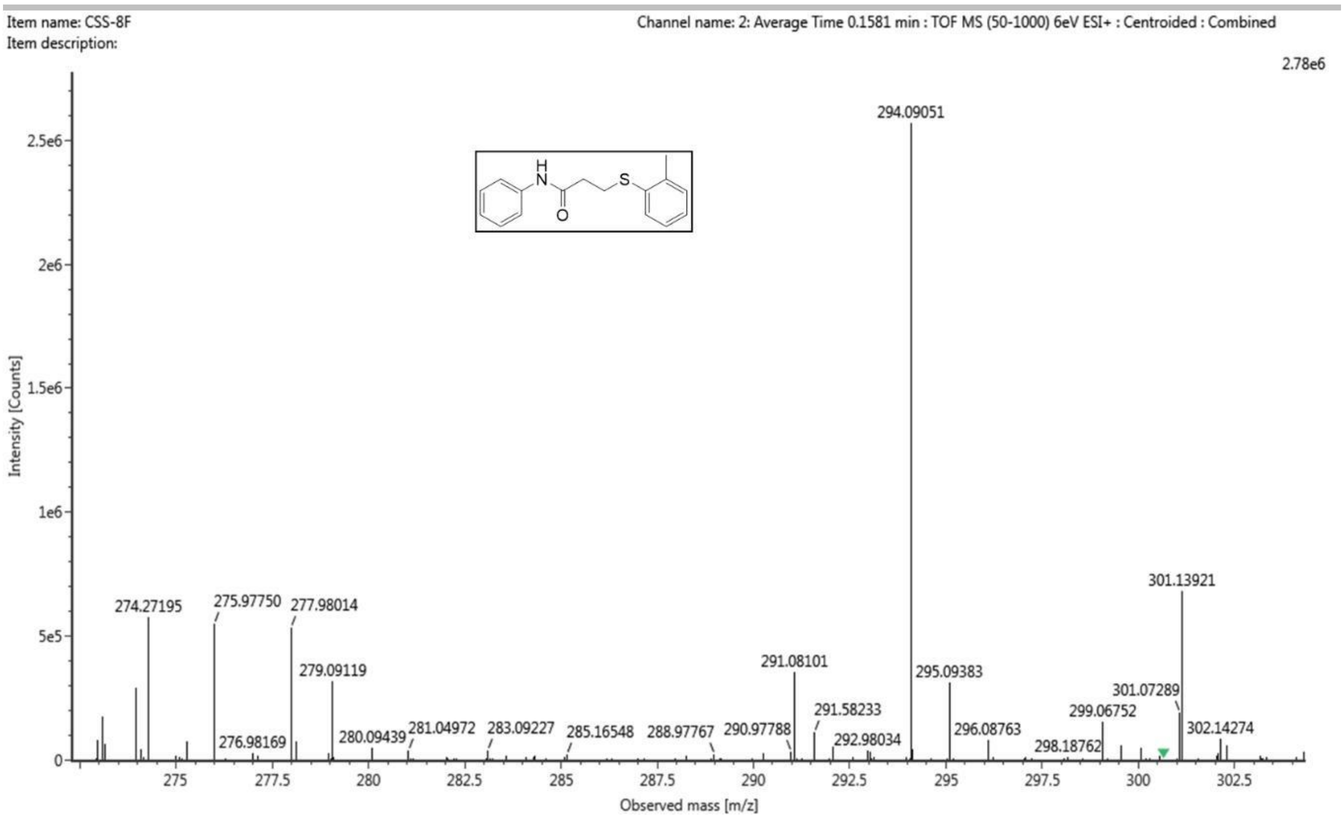

Supplementary Fig. 149. HR-MS of compound **11d**

^{1}H NMR (400 MHz, $CDCl_3$, 25°C) of compound **11e**

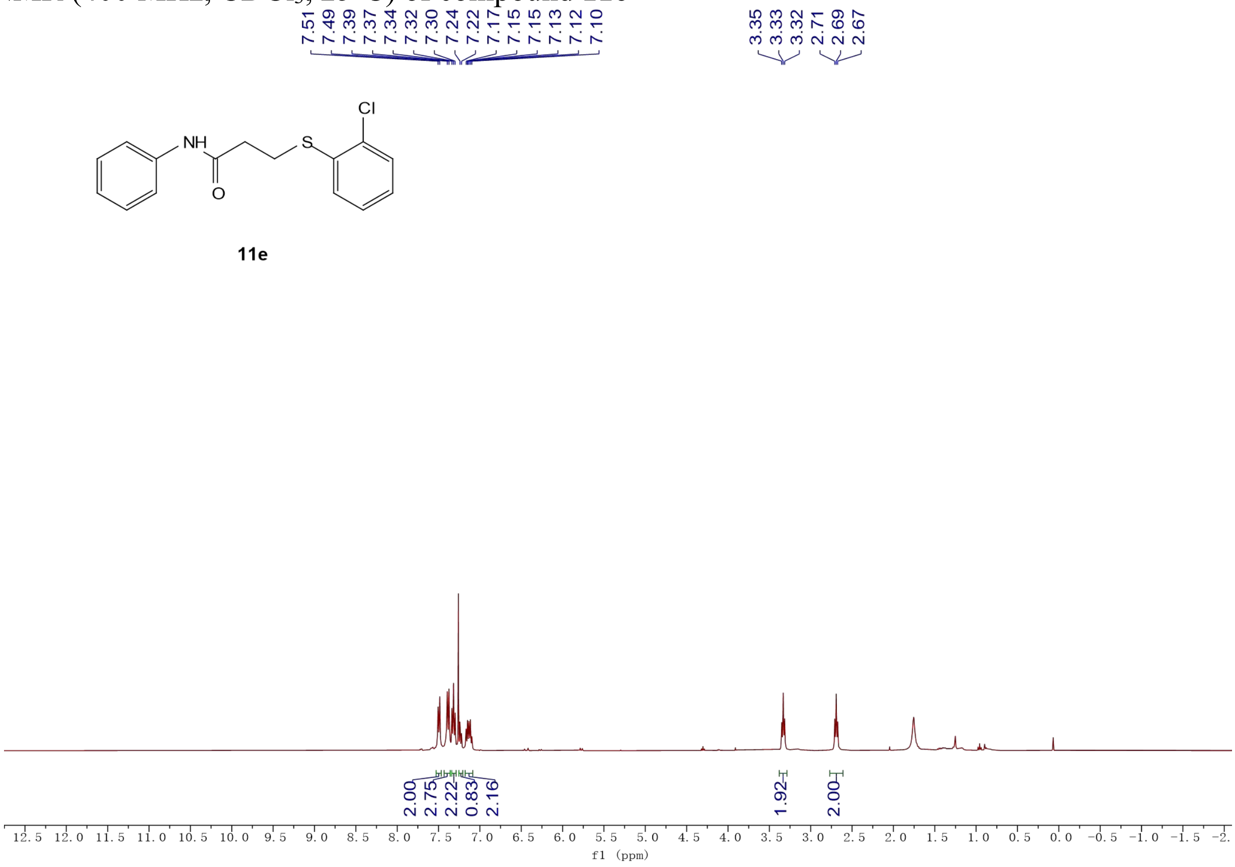

^{13}C NMR (101 MHz, $CDCl_3$, 25°C) of compound **11e**

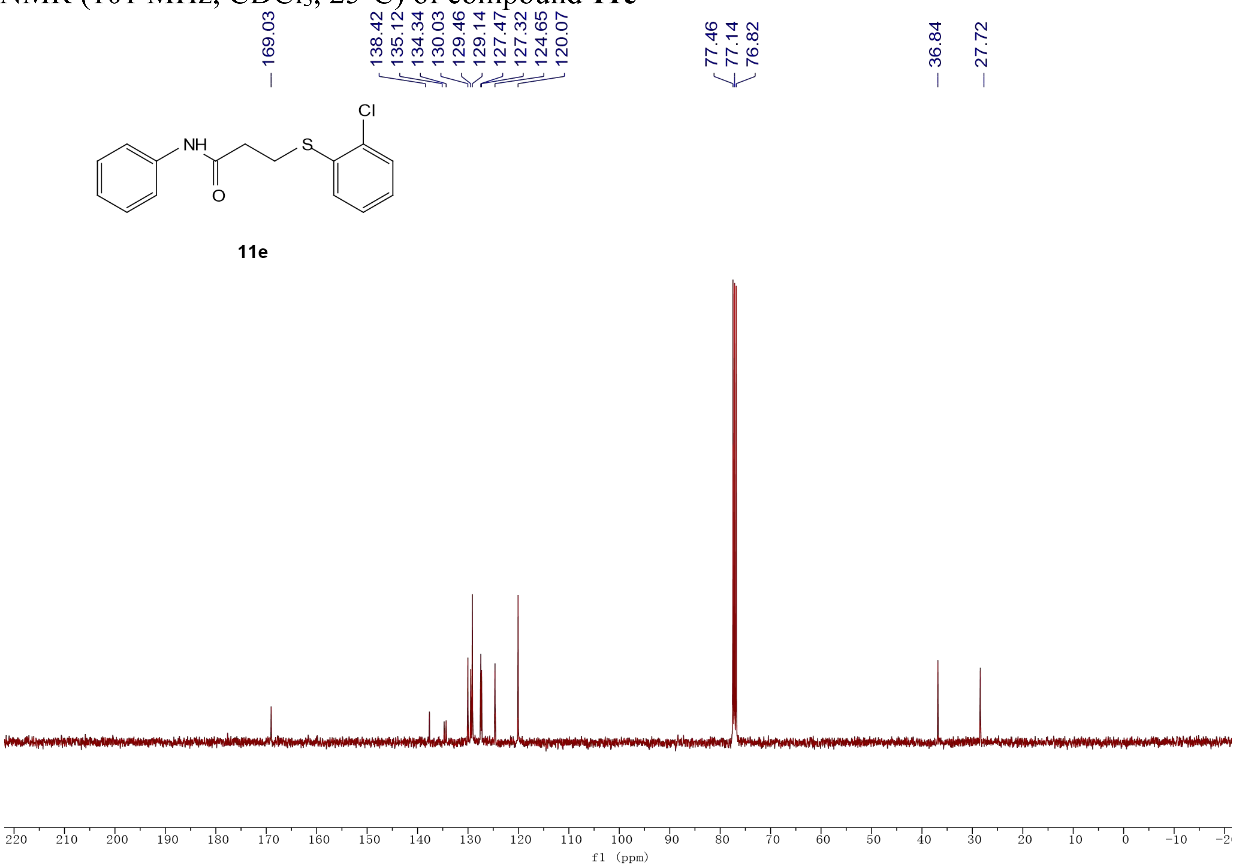

Supplementary Fig. 150. NMR of compound **11e**

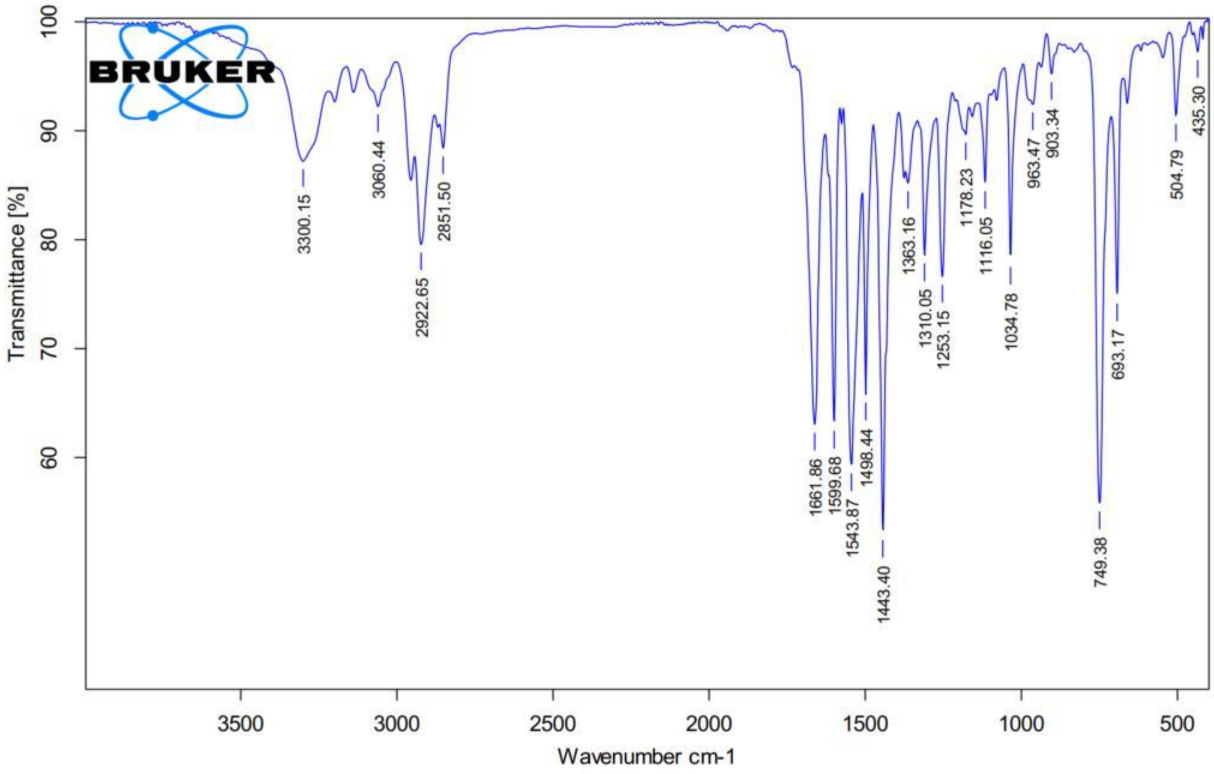

Supplementary Fig. 151. IR of compound **11e**

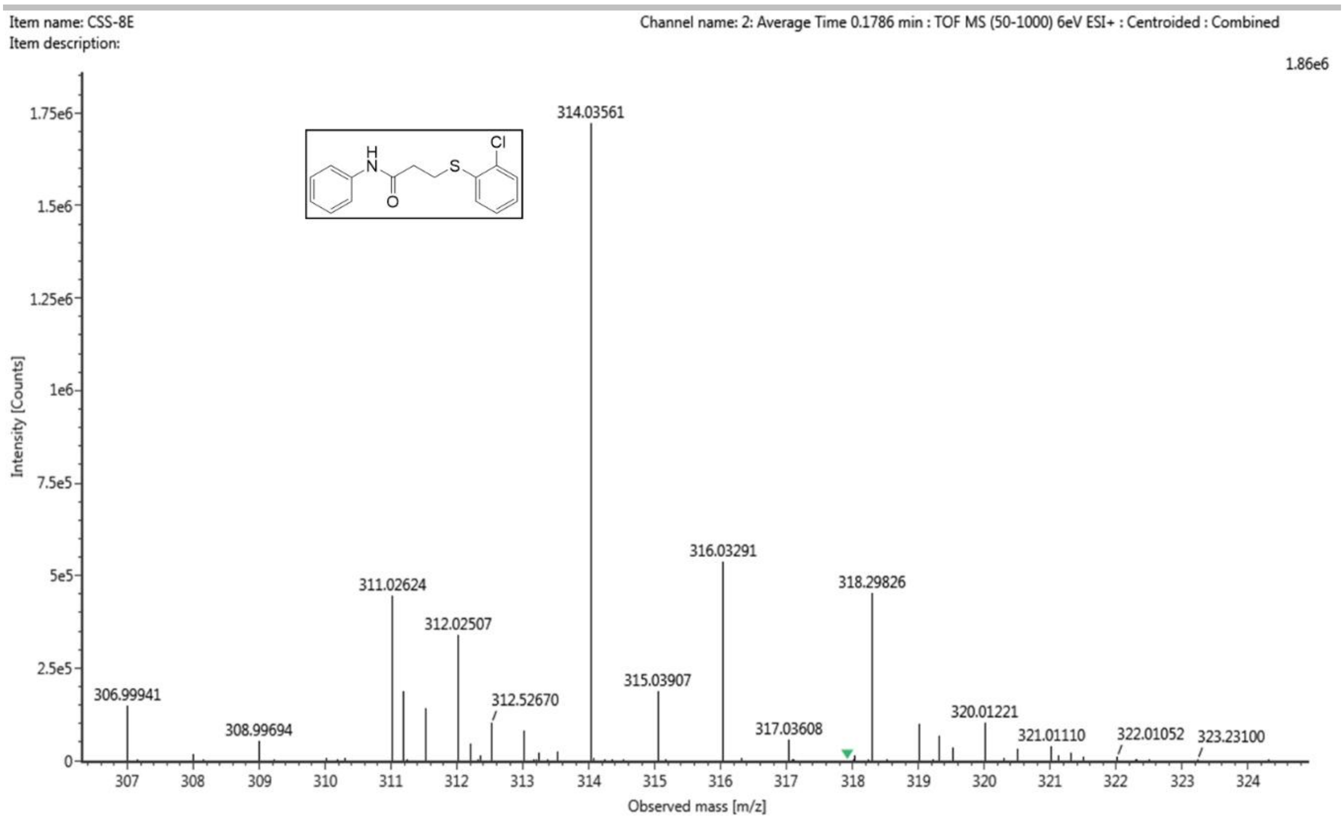

Supplementary Fig. 152. HR-MS of compound **11e**

^{1}H NMR (400 MHz, $CDCl_3$, 25°C) of compound **11f**

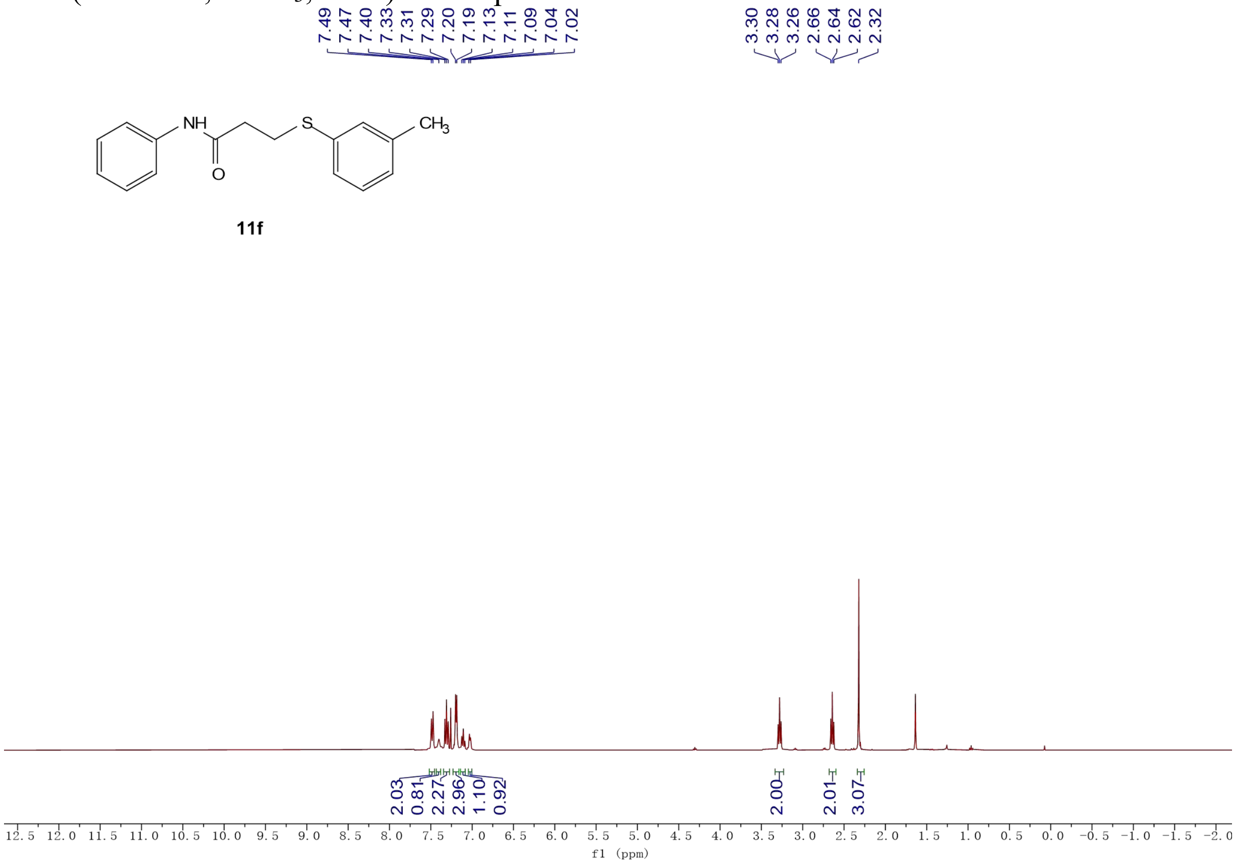

^{13}C NMR (101 MHz, $CDCl_3$, 25°C) of compound **11f**

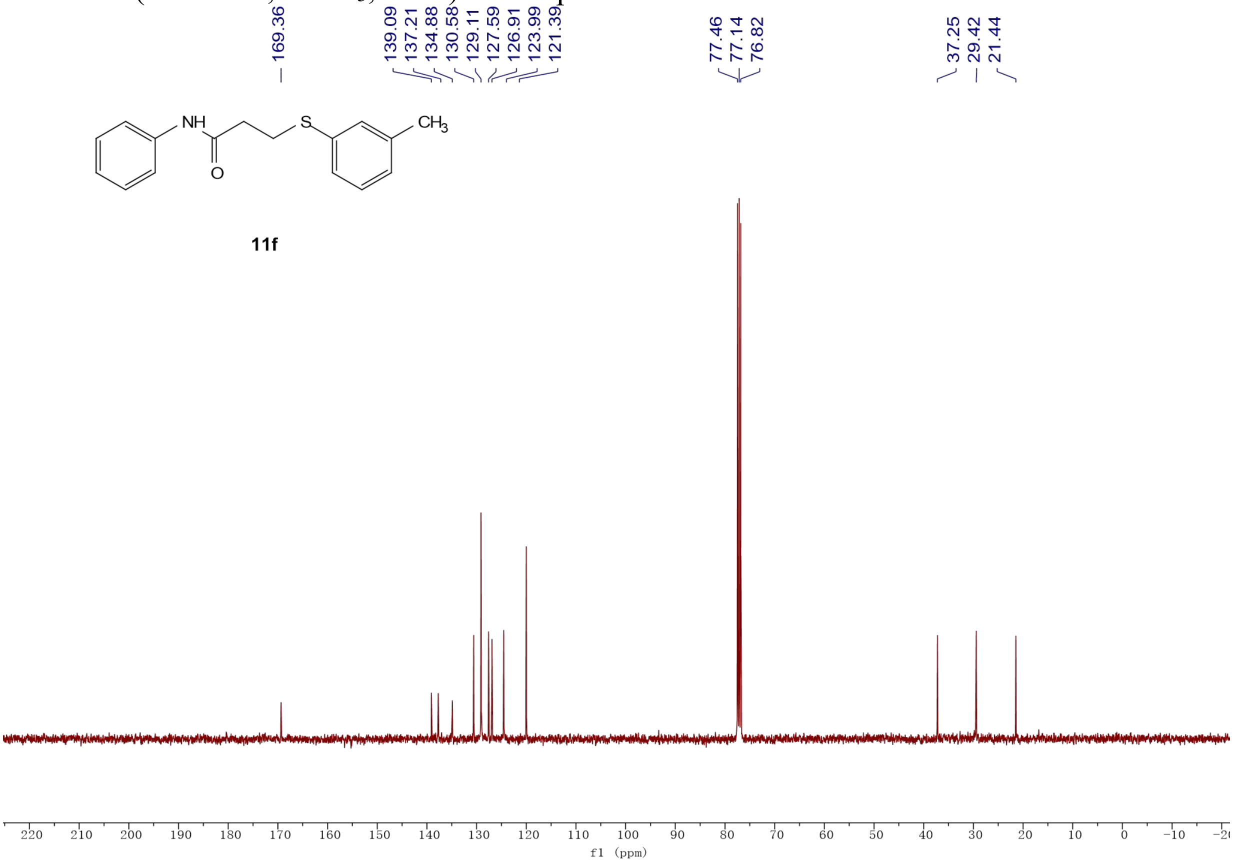

Supplementary Fig. 153. NMR of compound **11f**

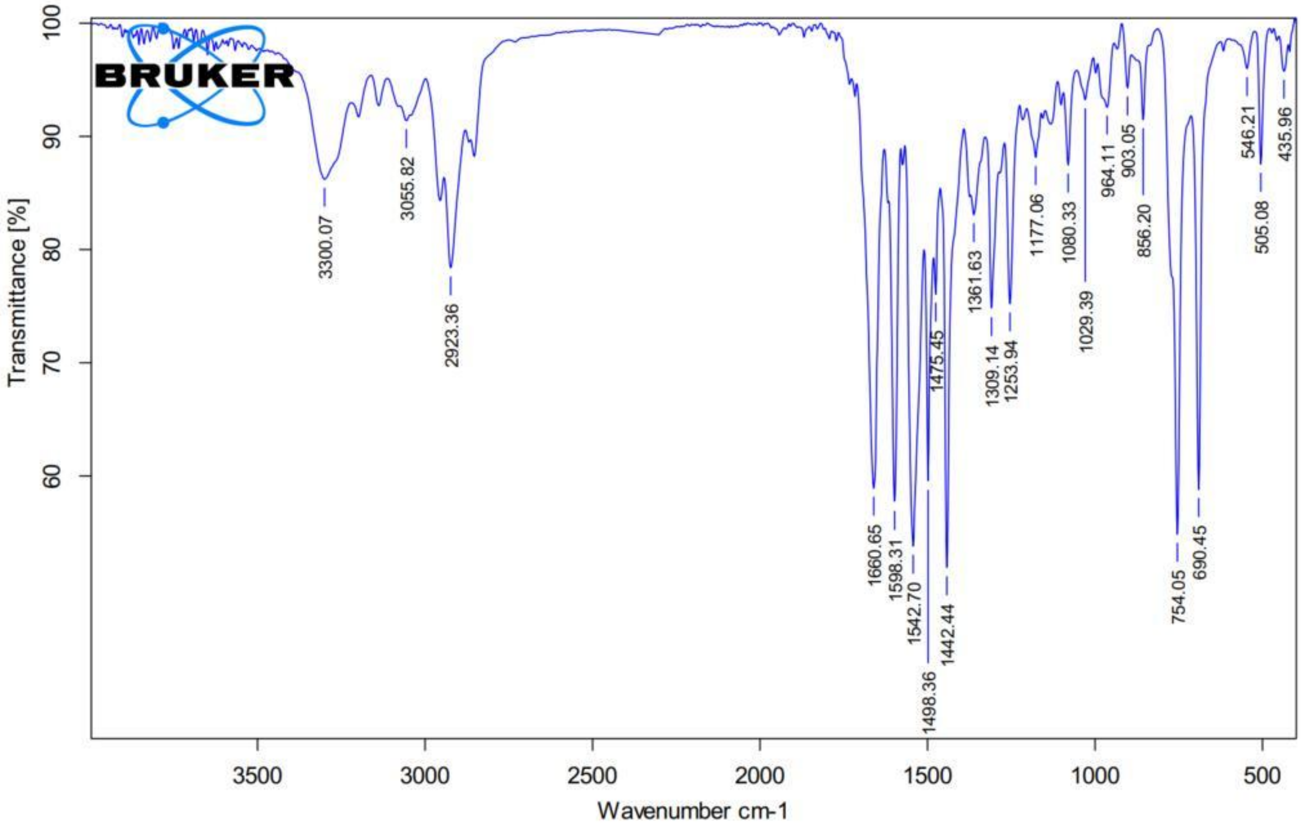

Supplementary Fig. 154. IR of compound **11f**

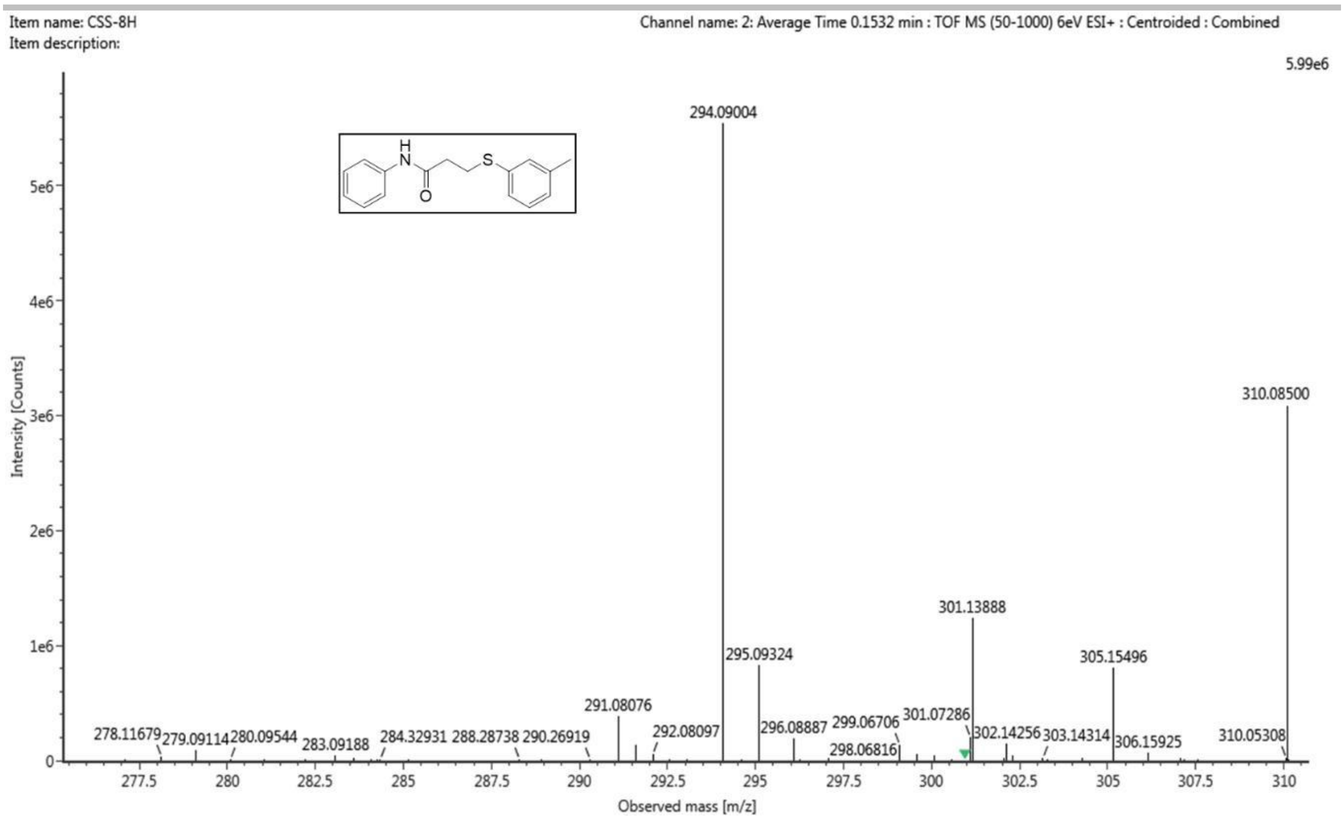

Supplementary Fig. 155. HR-MS of compound **11f**

^{1}**H NMR** (400 MHz, $CDCl_3$, 25°C) of compound **11g**

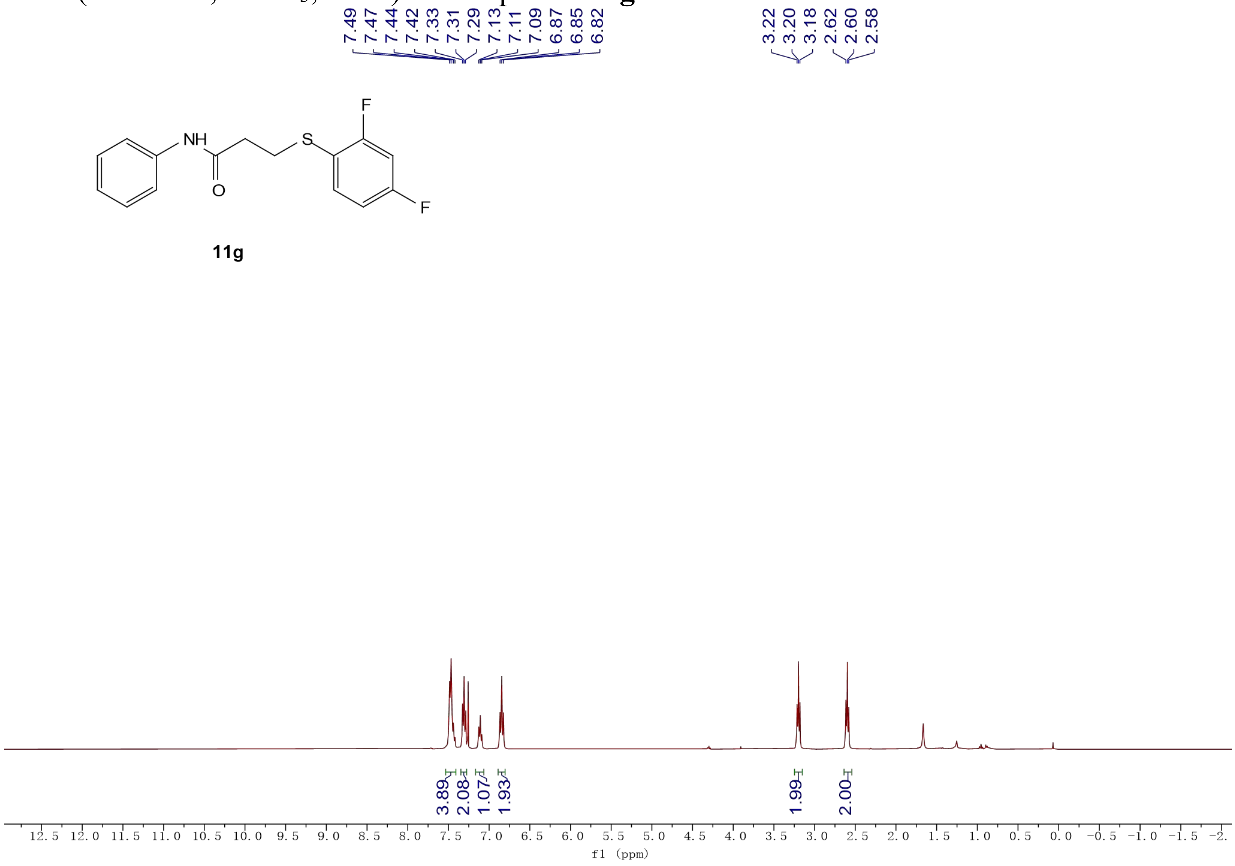

^{13}C NMR (101 MHz, $CDCl_3$, 25°C) of compound **11g**

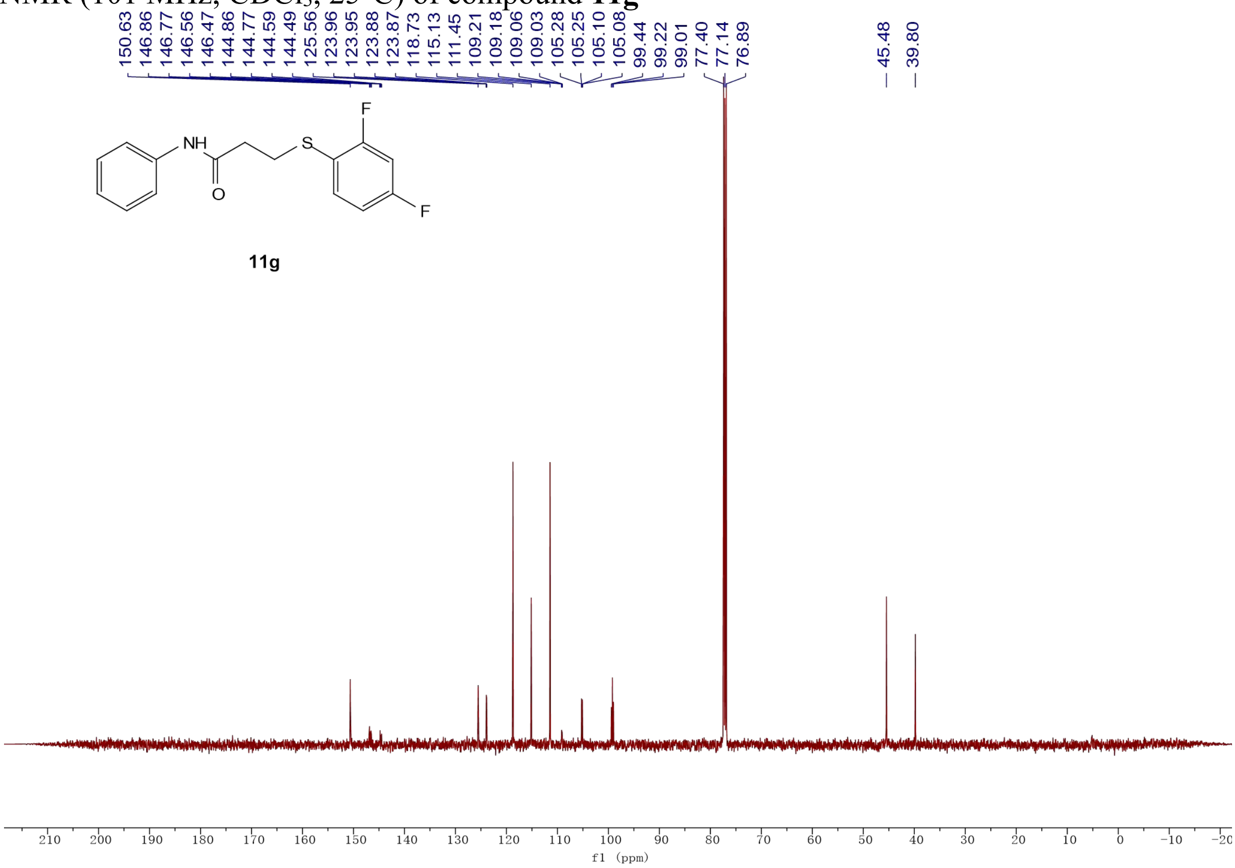

^{19}F NMR (376 MHz, $CDCl_3$, 25°C) of compound **11g**

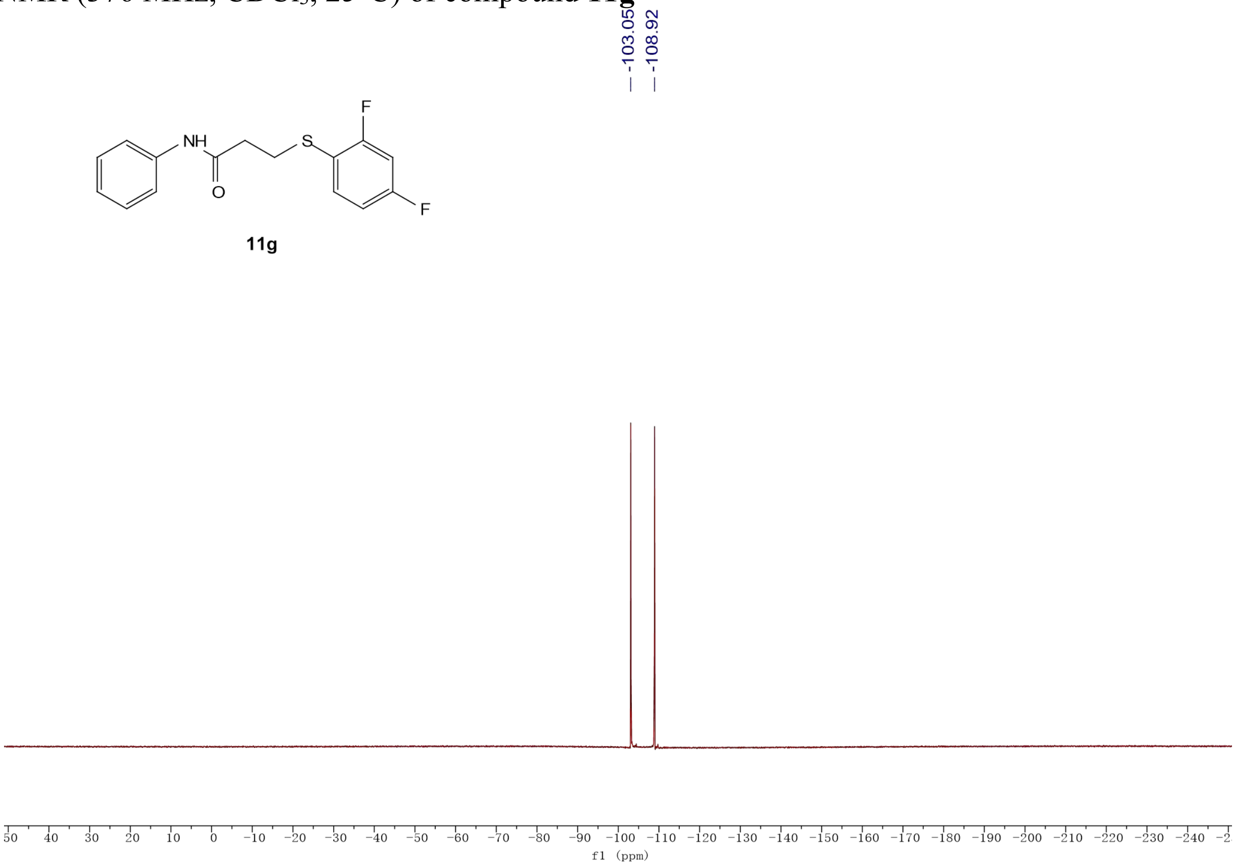

Supplementary Fig. 156. NMR of compound **11g**

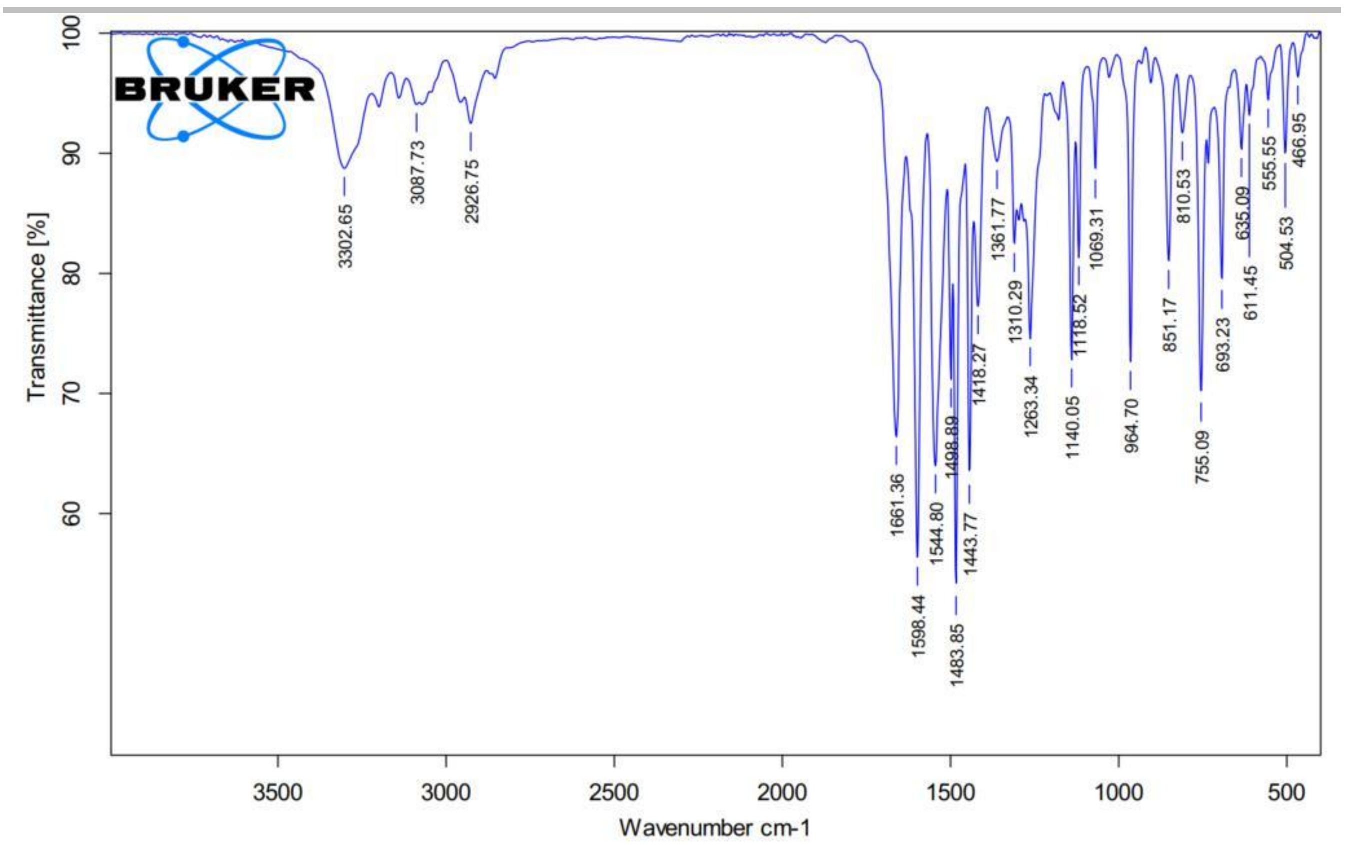

Supplementary Fig. 157. IR of compound **11g**

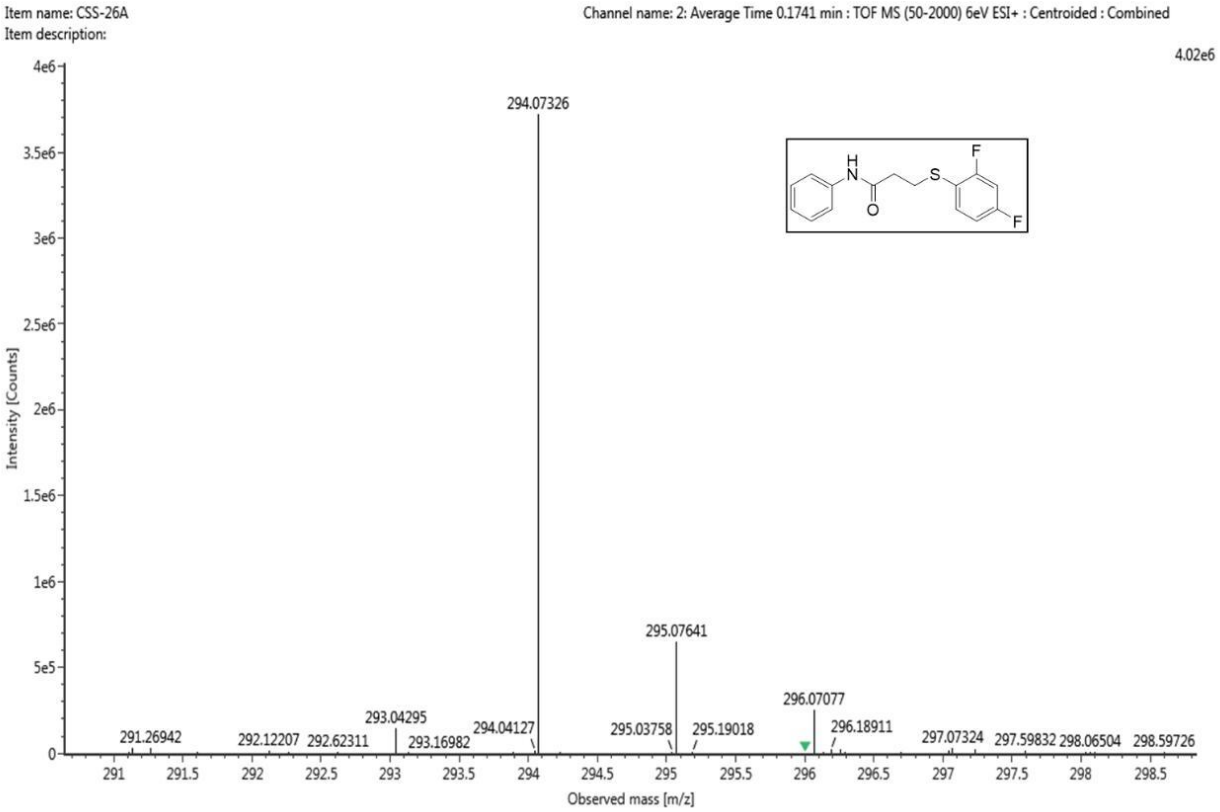

Supplementary Fig. 158. HR-MS of compound **11g**

^{1}H NMR (400 MHz, $CDCl_3$, 25°C) of compound **11h**

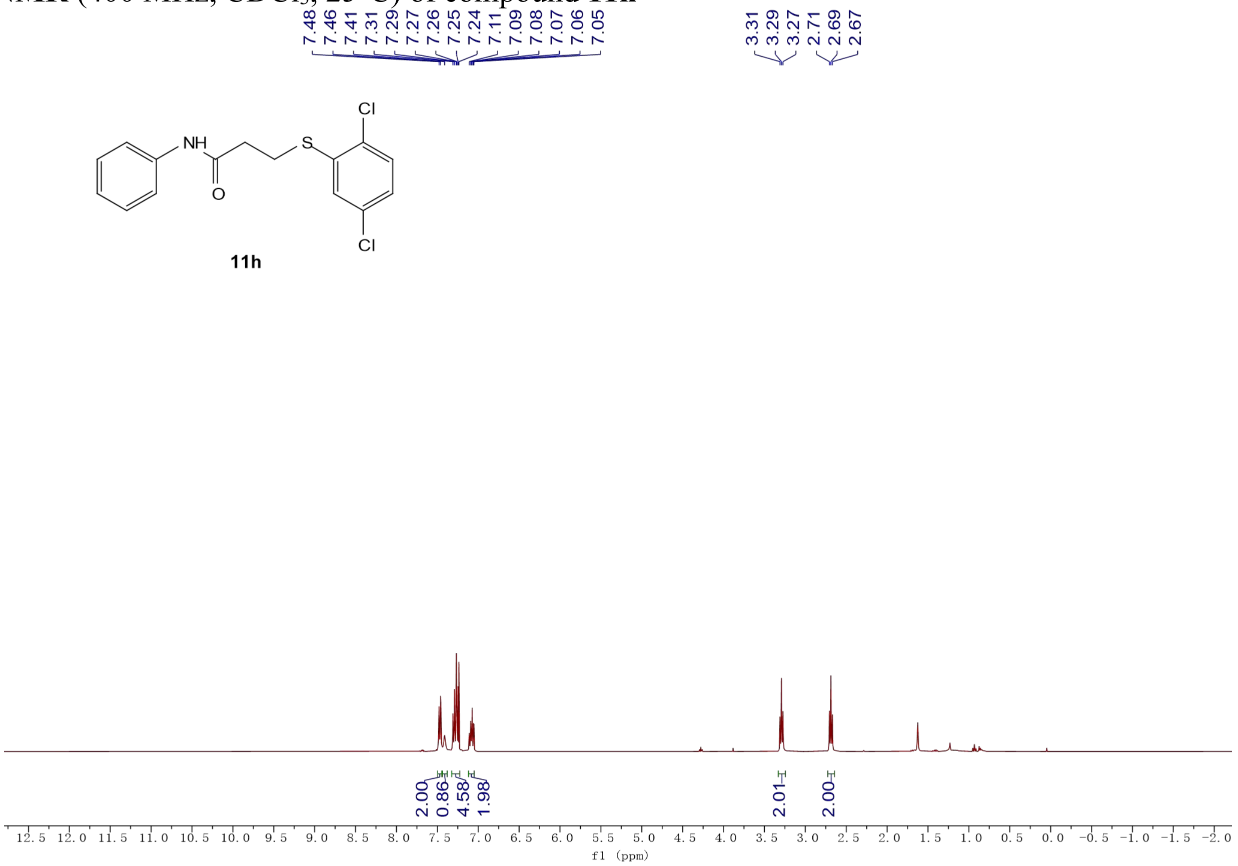

^{13}C NMR (101 MHz, $CDCl_3$, 25°C) of compound **11h**

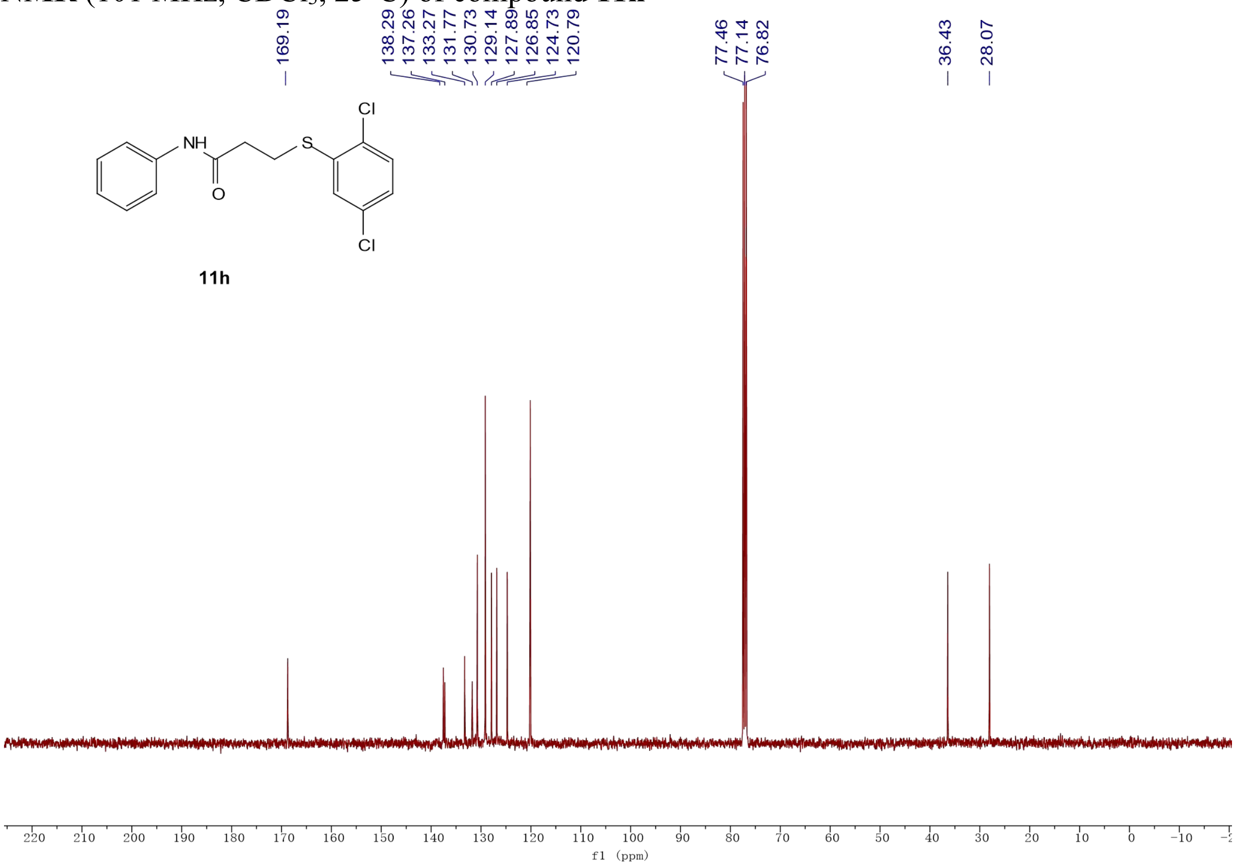

Supplementary Fig. 159. NMR of compound **11h**

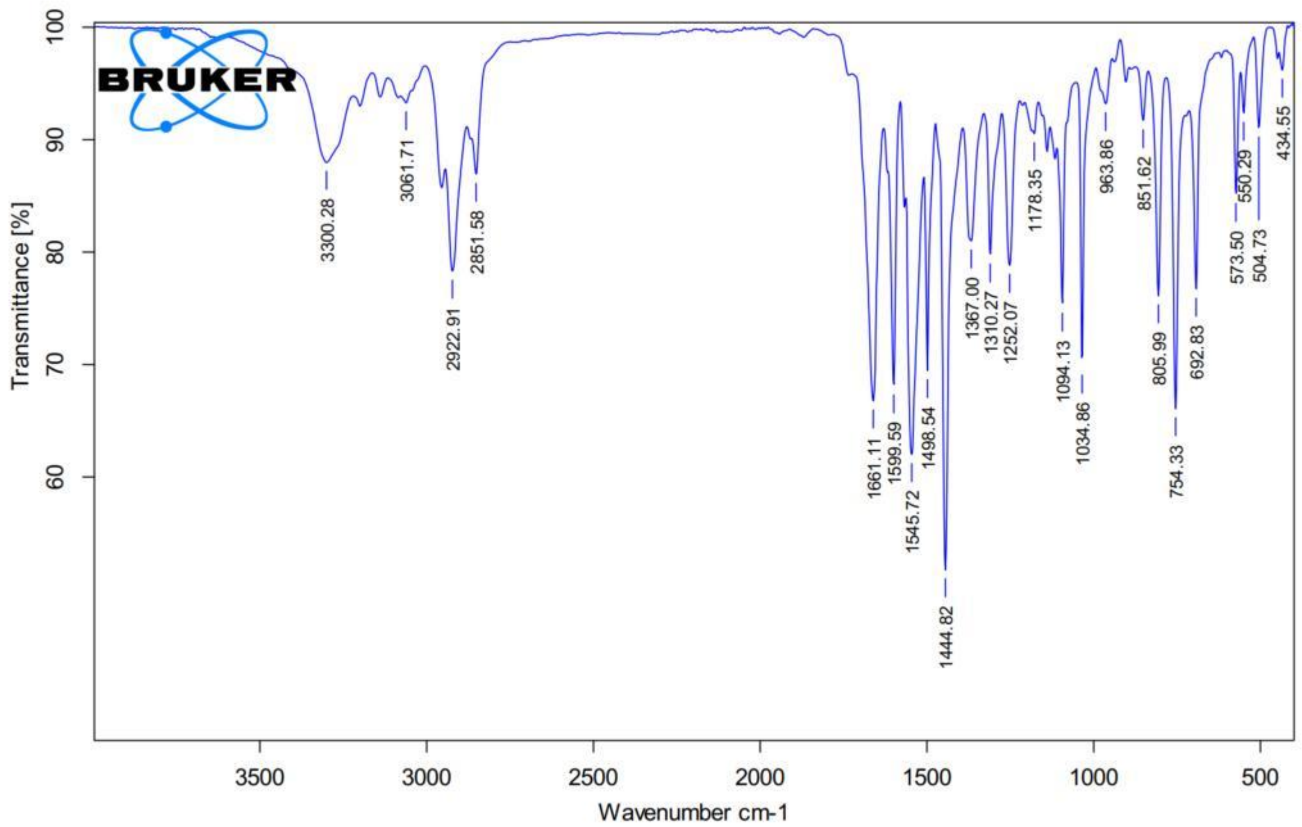

Supplementary Fig. 160. IR of compound **11h**

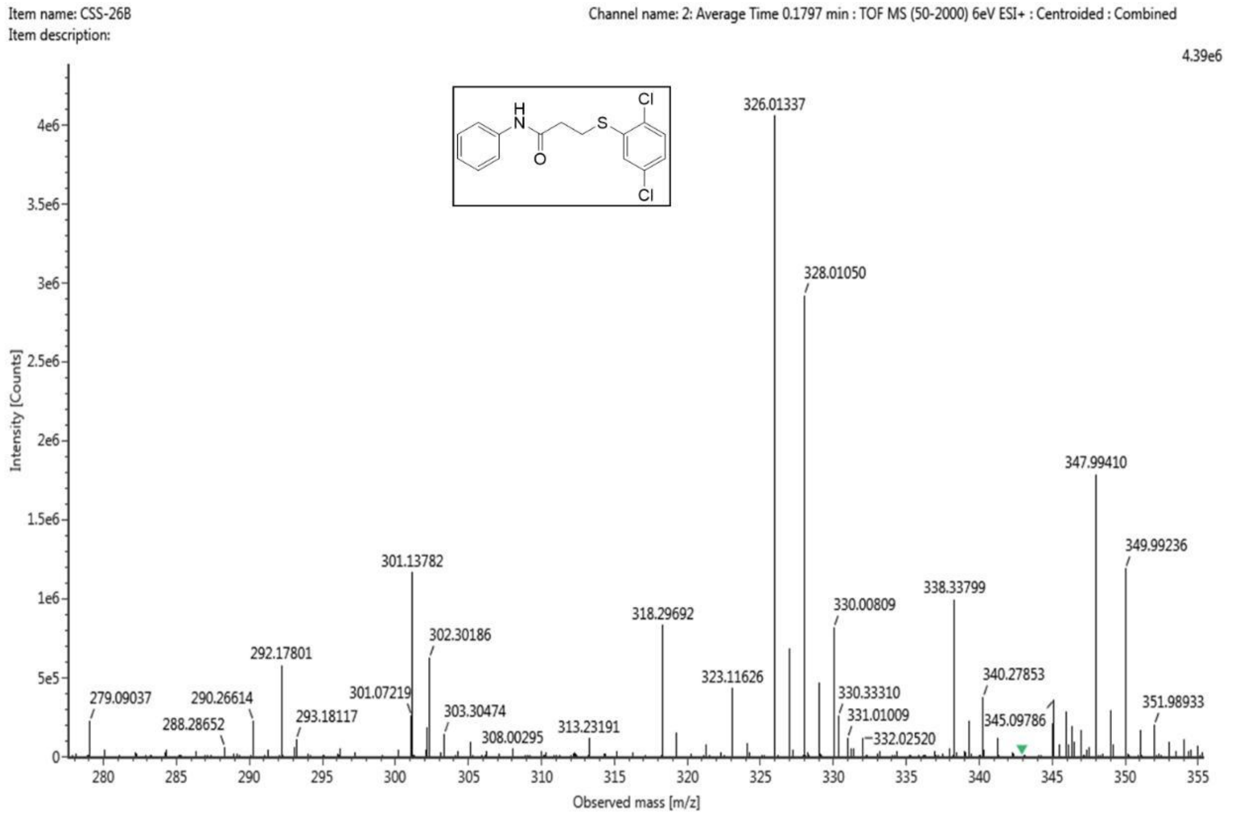

Supplementary Fig. 161. HR-MS of compound **11h**

^{1}H NMR (400 MHz, $CDCl_3$, 25°C) of compound **11i**

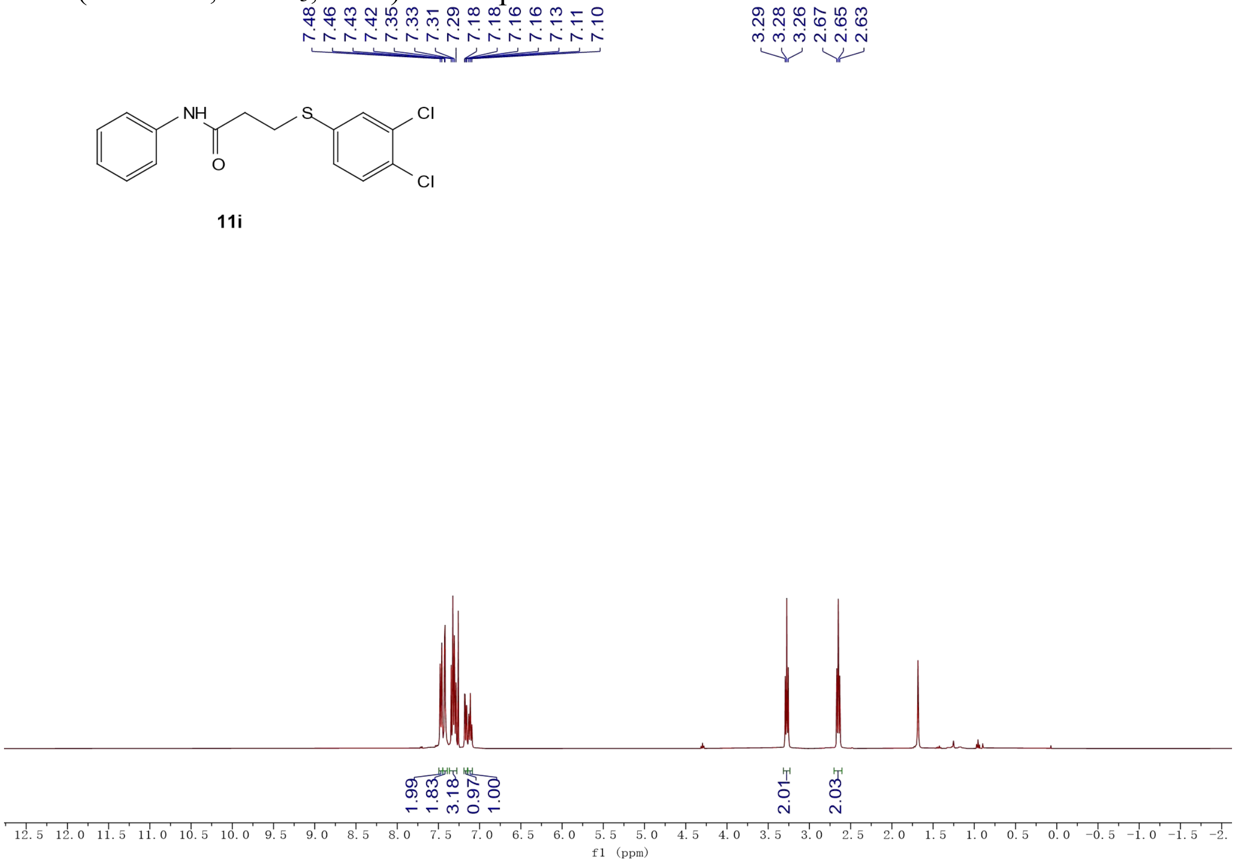

^{13}C NMR (101 MHz, $CDCl_3$, 25°C) of compound **11i**

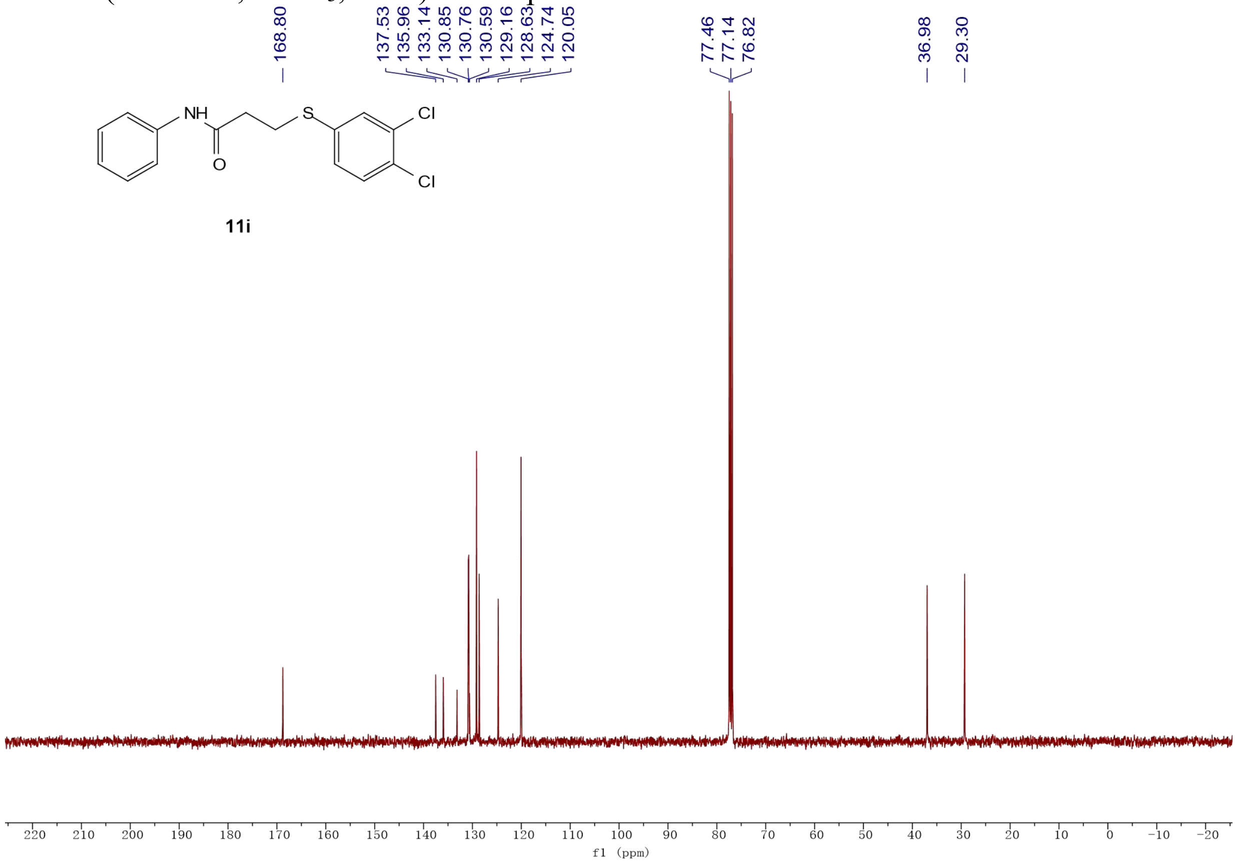

Supplementary Fig. 162. NMR of compound **11i**

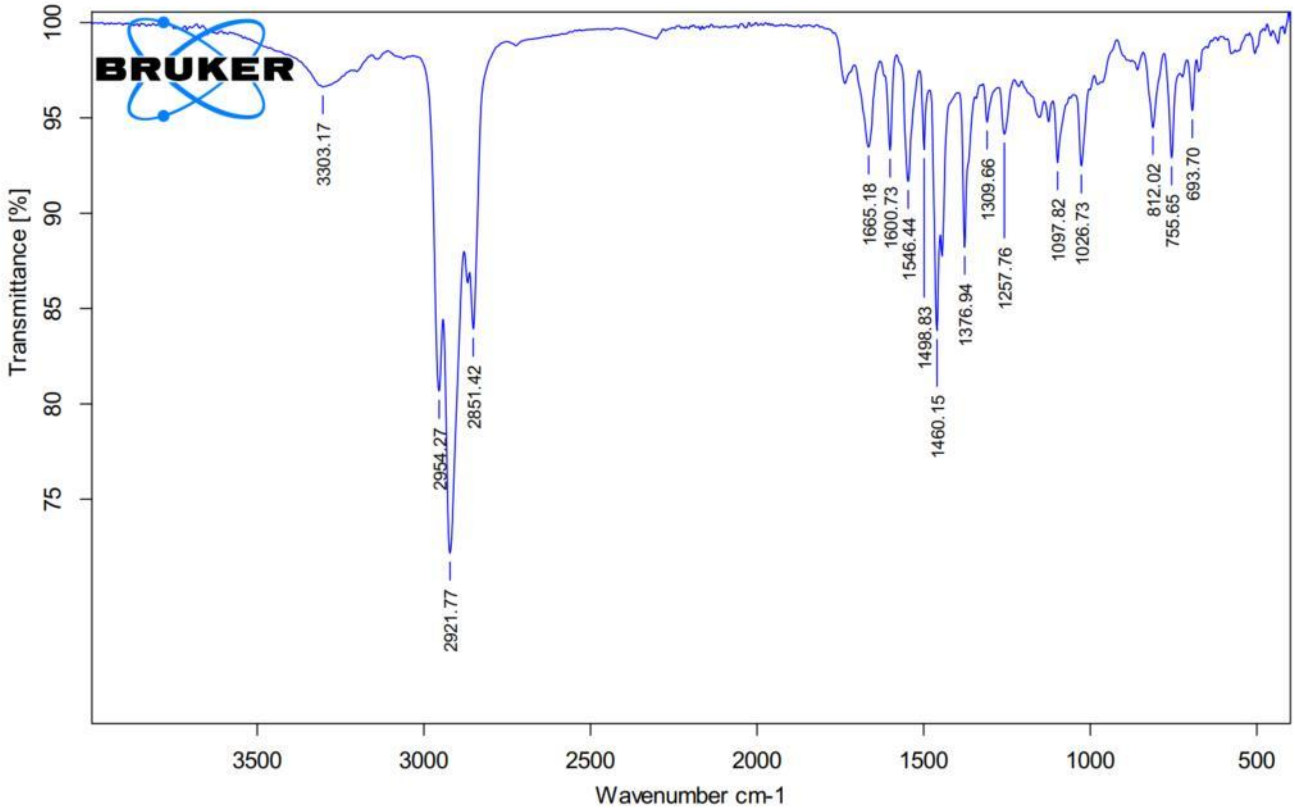

Supplementary Fig. 163. IR of compound **11i**

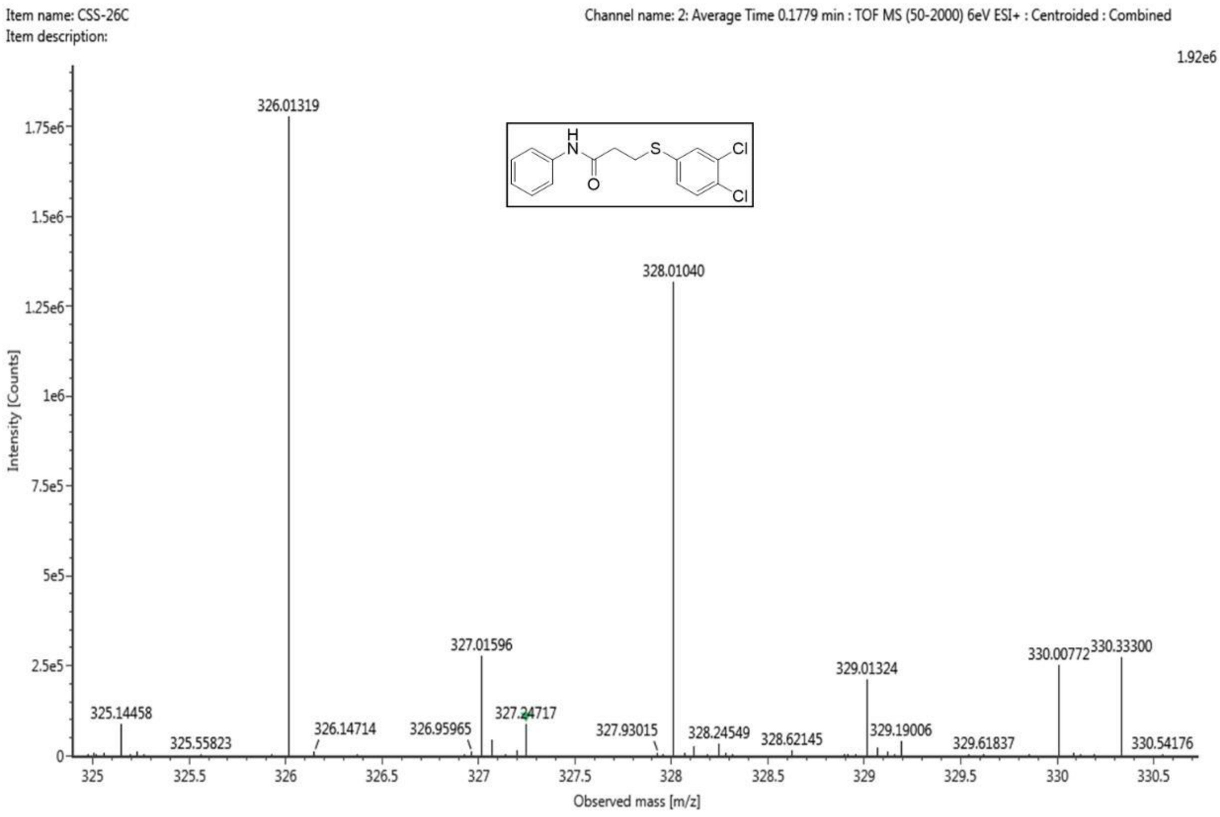

Supplementary Fig. 164. HR-MS of compound **11i**

^{1}H NMR (400 MHz, $CDCl_3$, 25℃) of compound **11j**

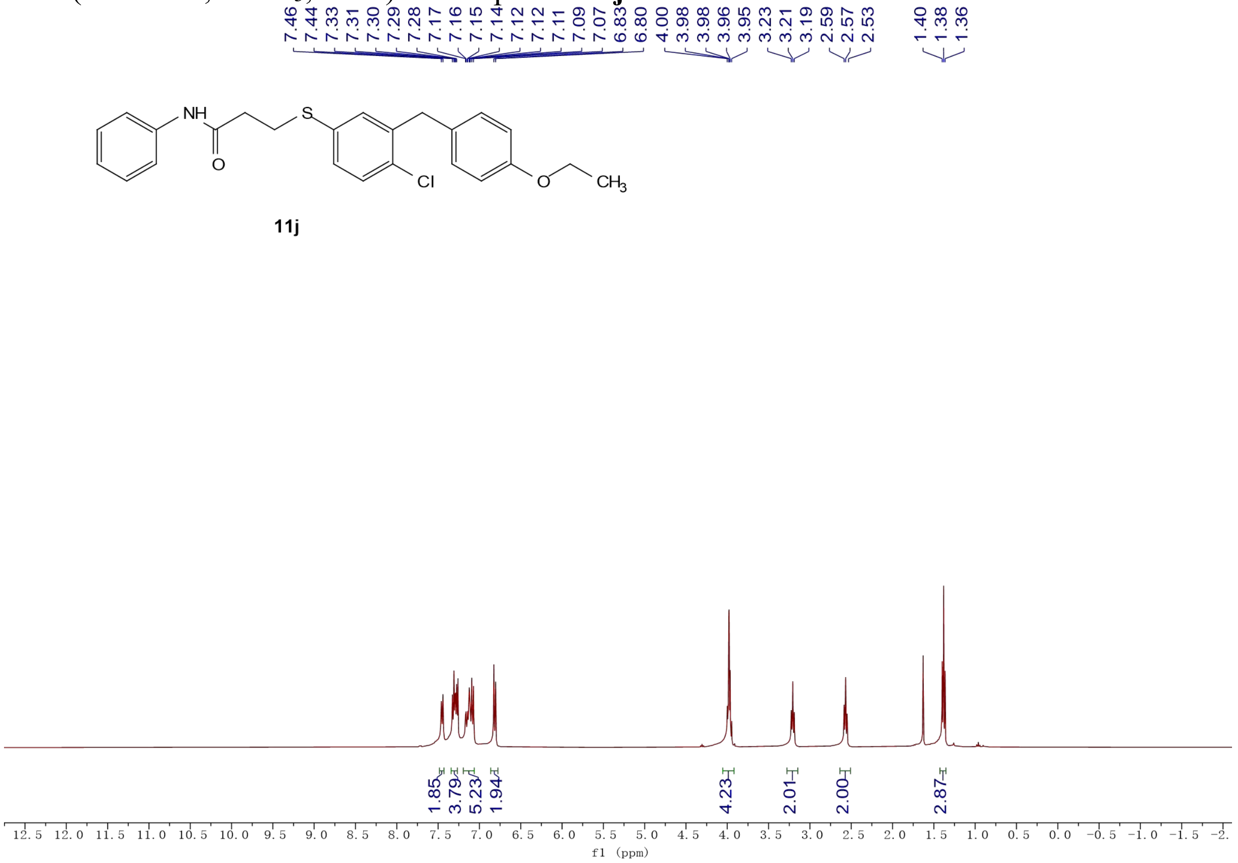

^{13}C NMR (150 MHz, $CDCl_3$, 25℃) of compound **11j**

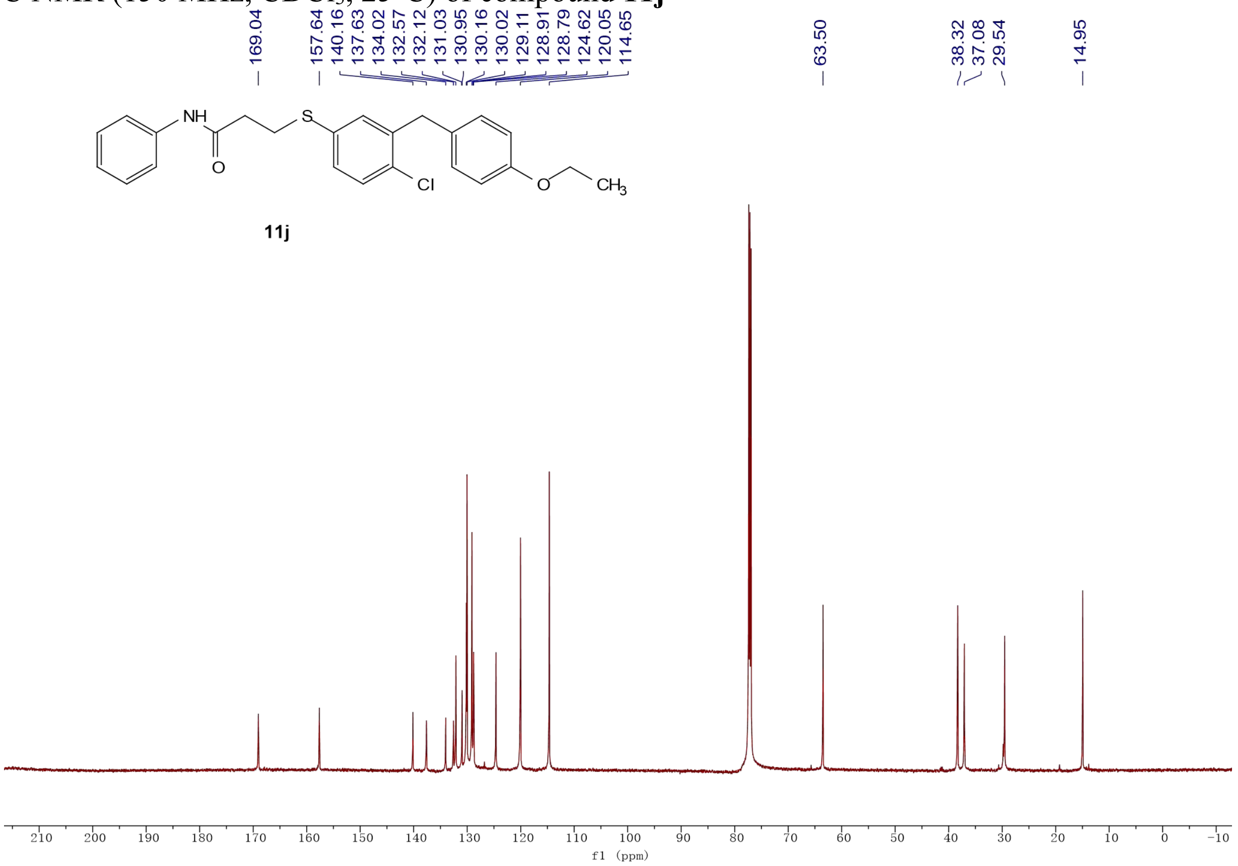

Supplementary Fig. 165. NMR of compound **11j**

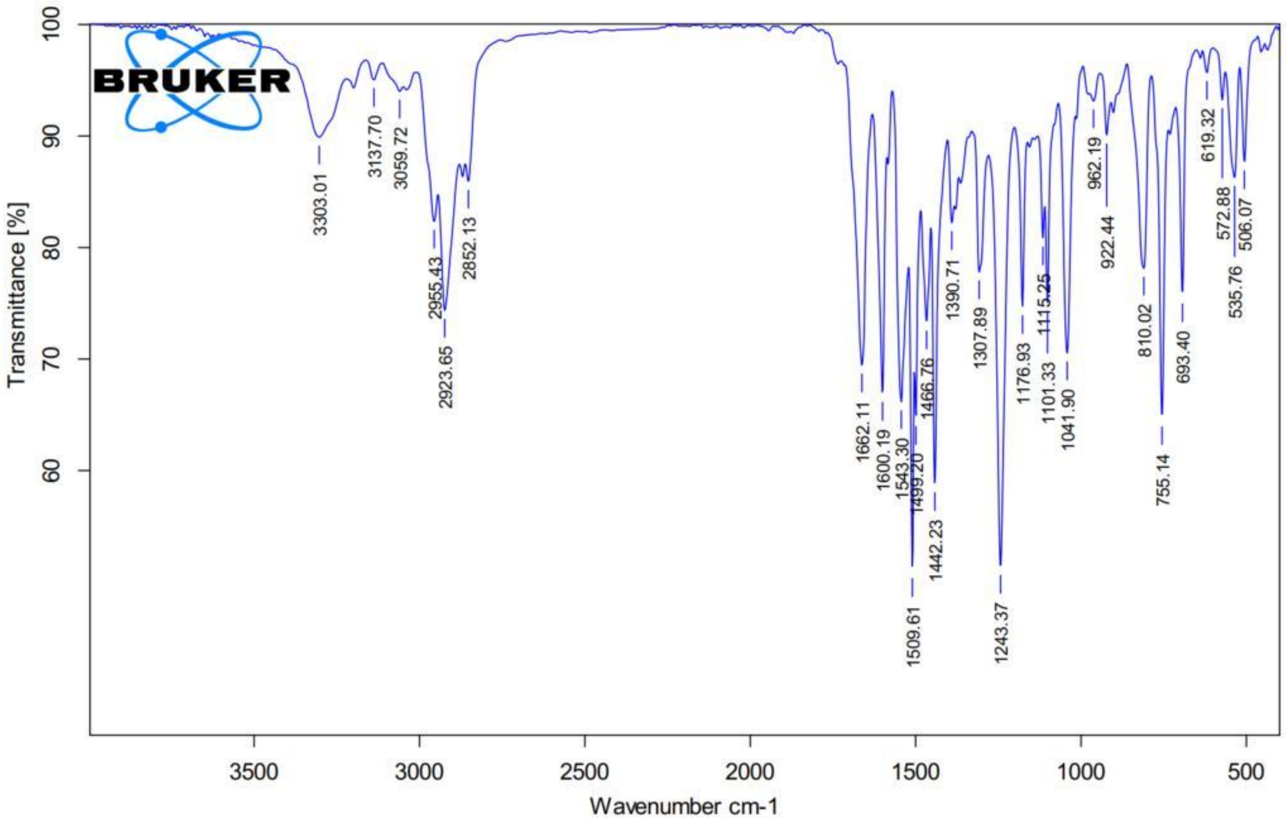

Supplementary Fig. 166. IR of compound **11j**

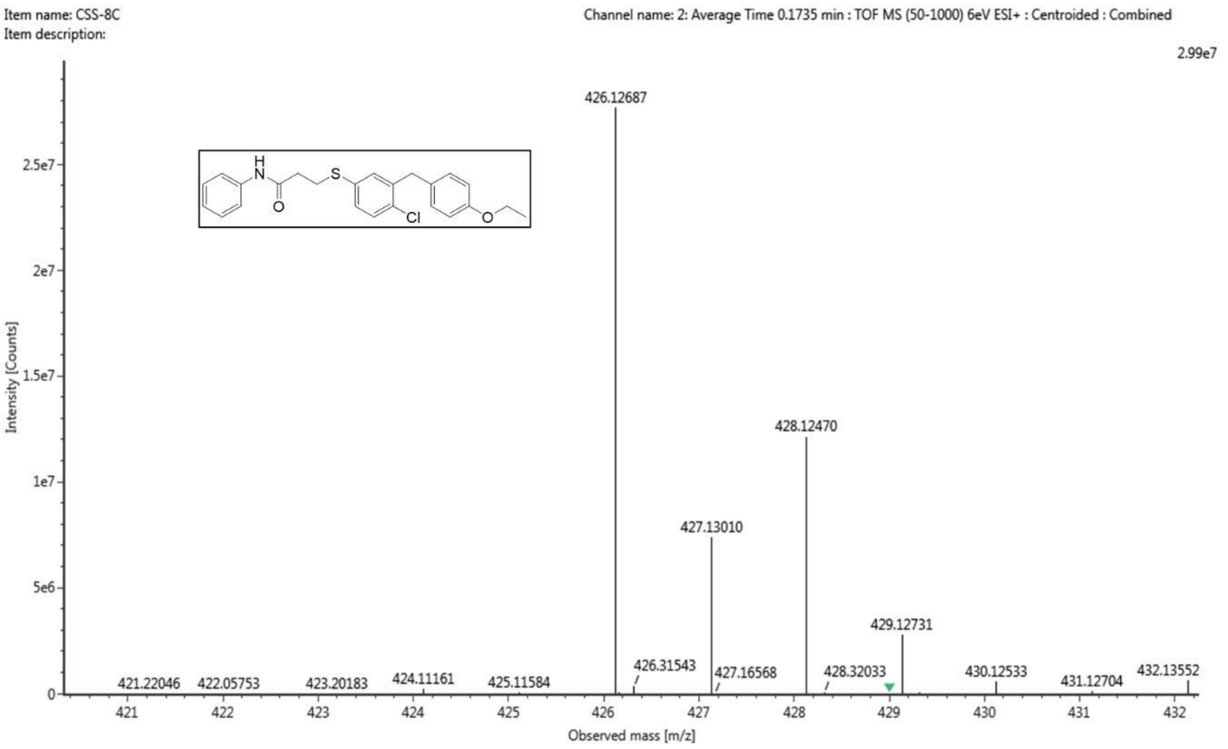

Supplementary Fig. 167. HR-MS of compound **11j**

^{1}H NMR (600 MHz, $CDCl_3$, 25℃) of compound **11k**

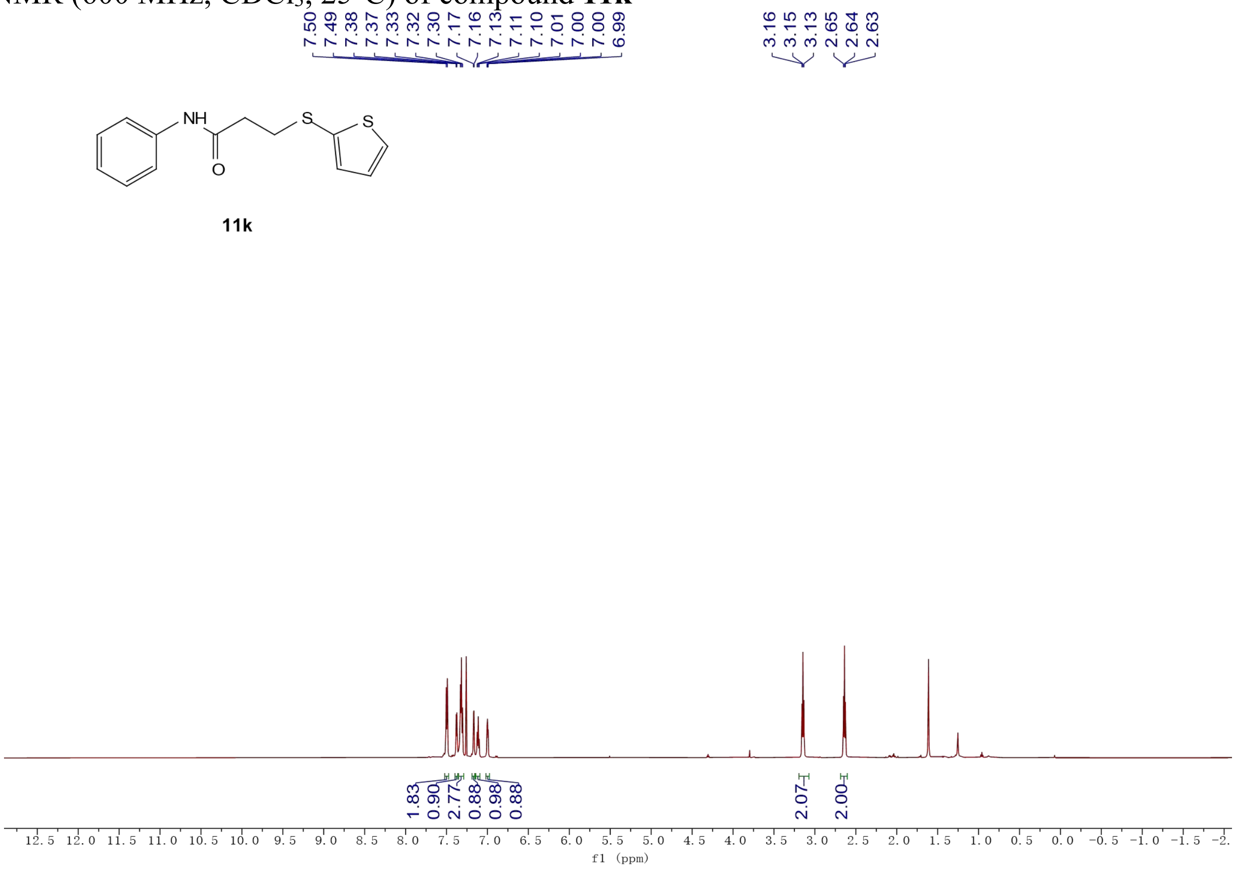

^{13}C NMR (150 MHz, $CDCl_3$, 25℃) of compound **11k**

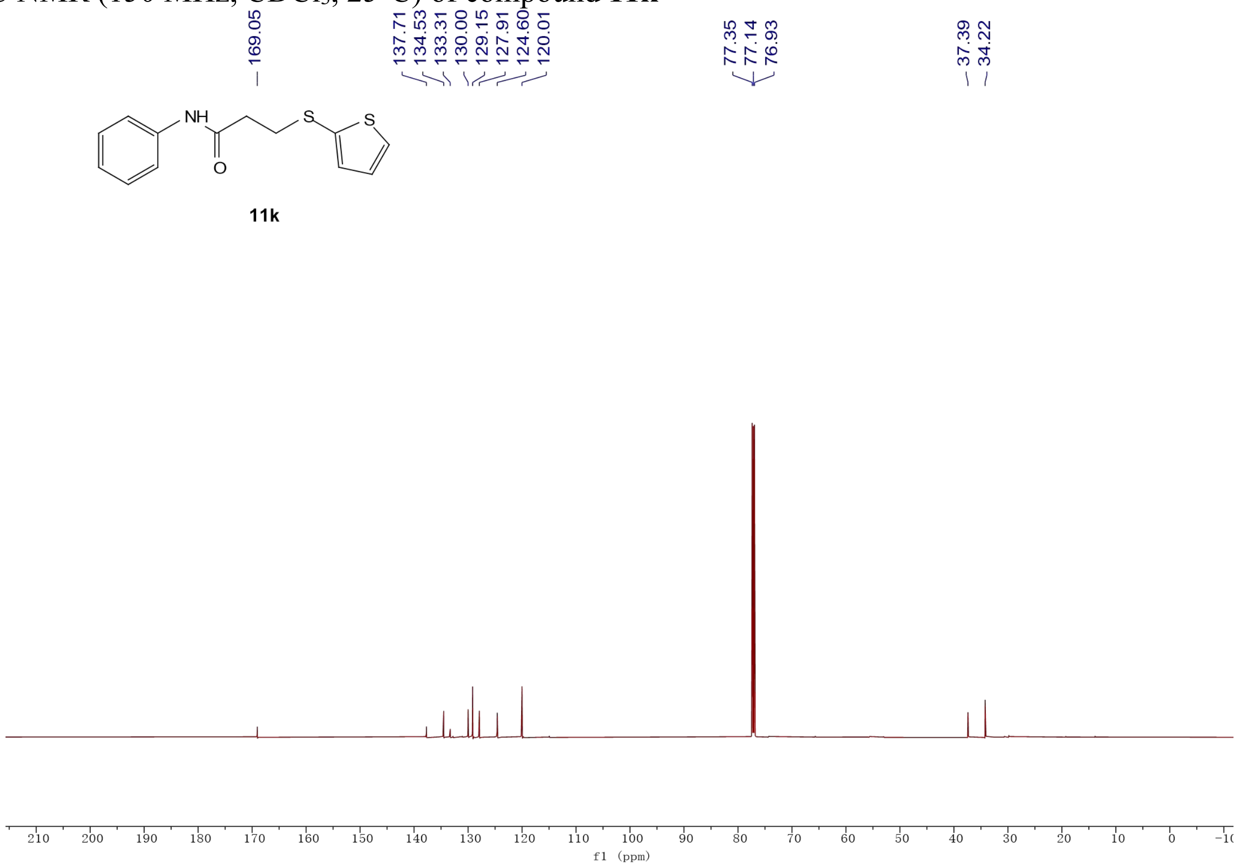

Supplementary Fig. 168. NMR of compound **11k**

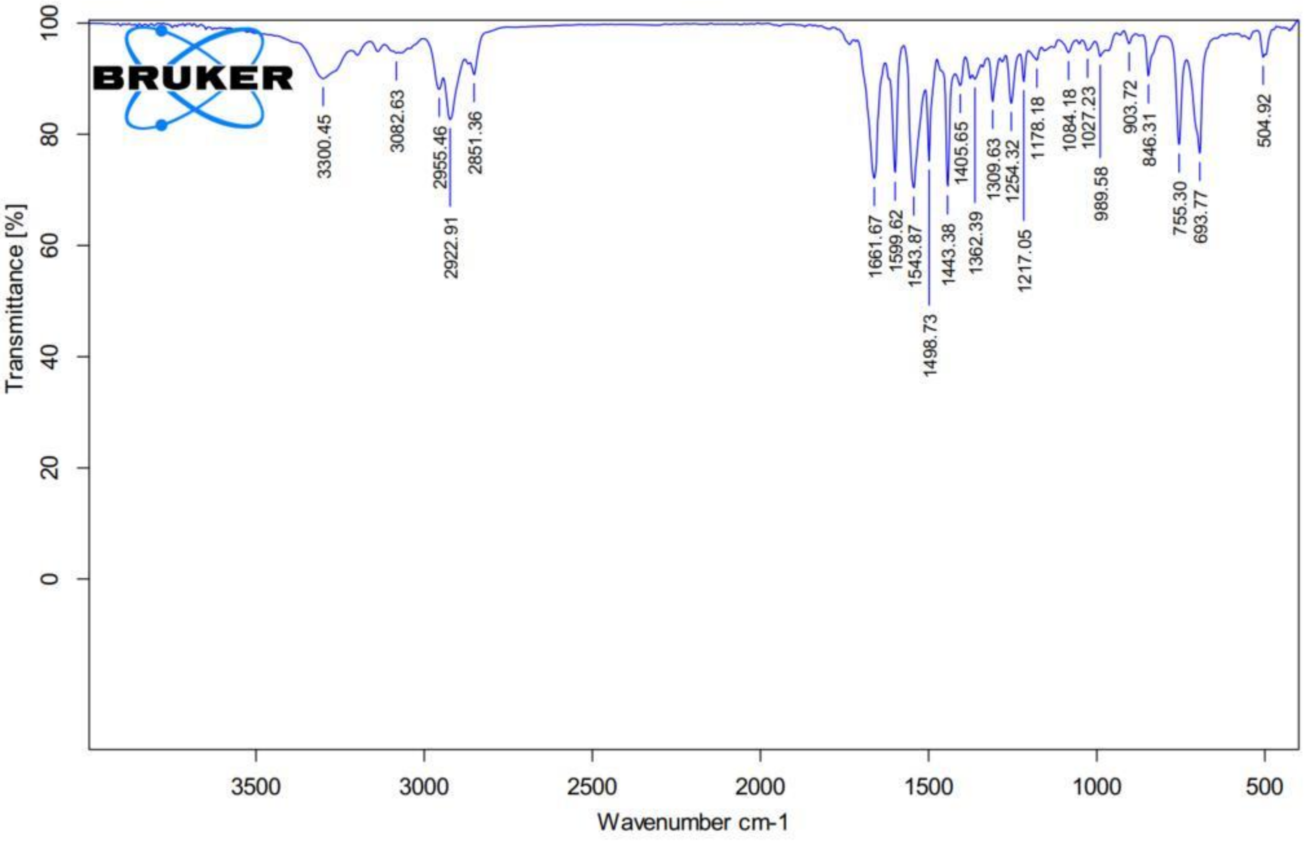

Supplementary Fig. 169. IR of compound **11k**

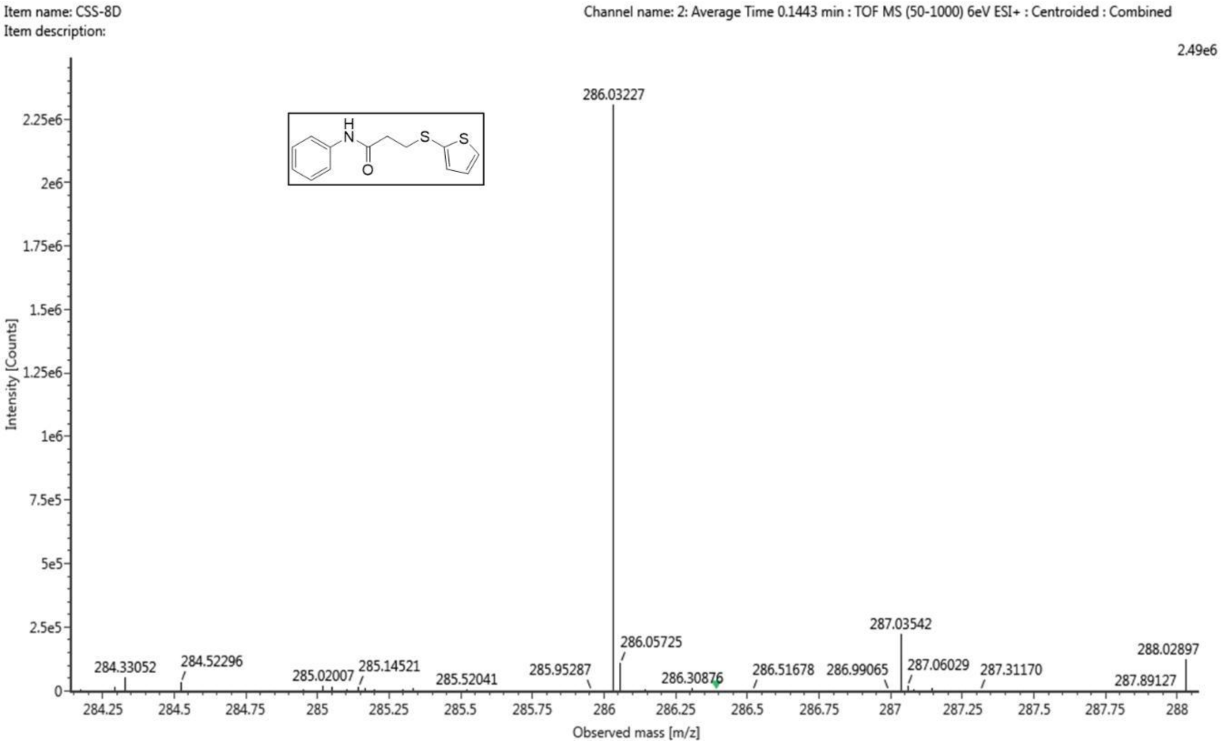

Supplementary Fig. 170. HR-MS of compound **11k**

^{1}H NMR (400 MHz, $CDCl_3$, 25℃) of compound **11l**

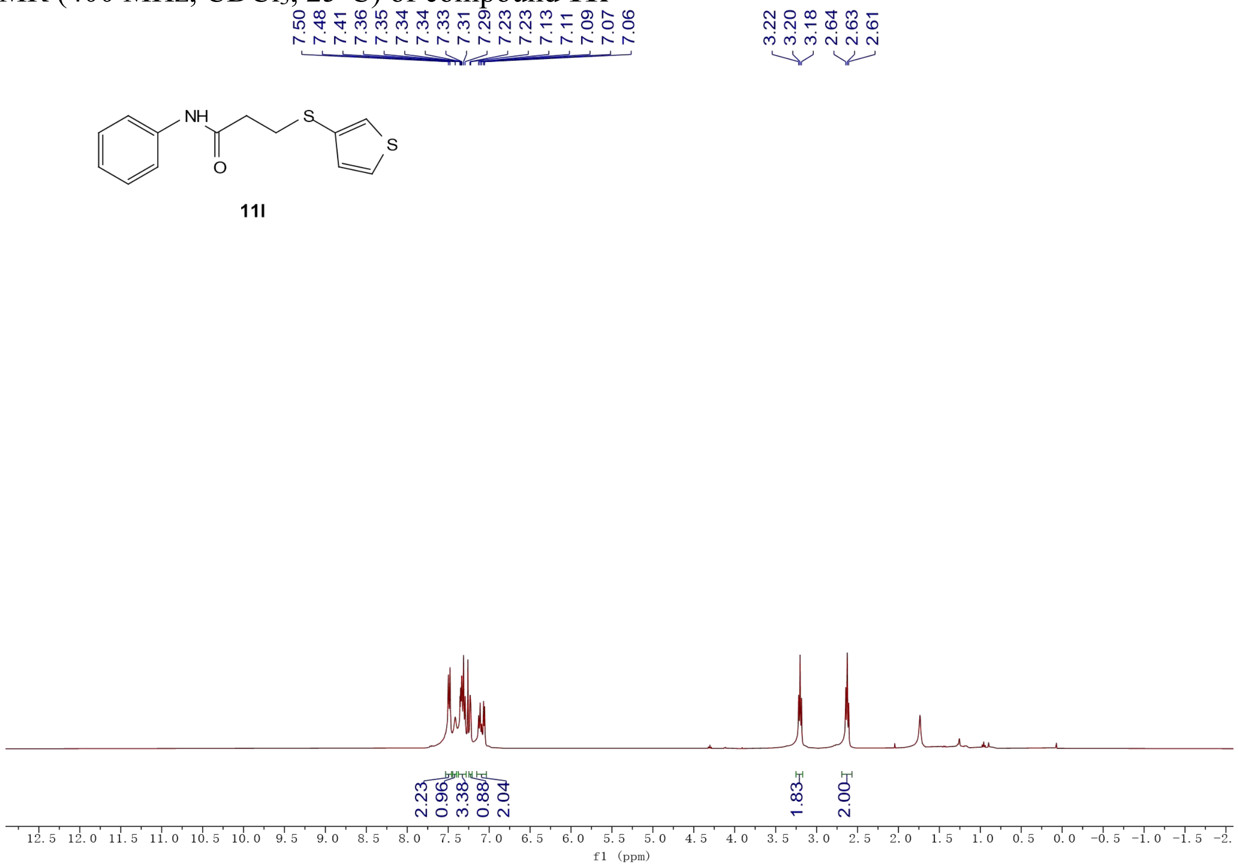

^{13}C NMR (101 MHz, $CDCl_3$, 25℃) of compound **11l**

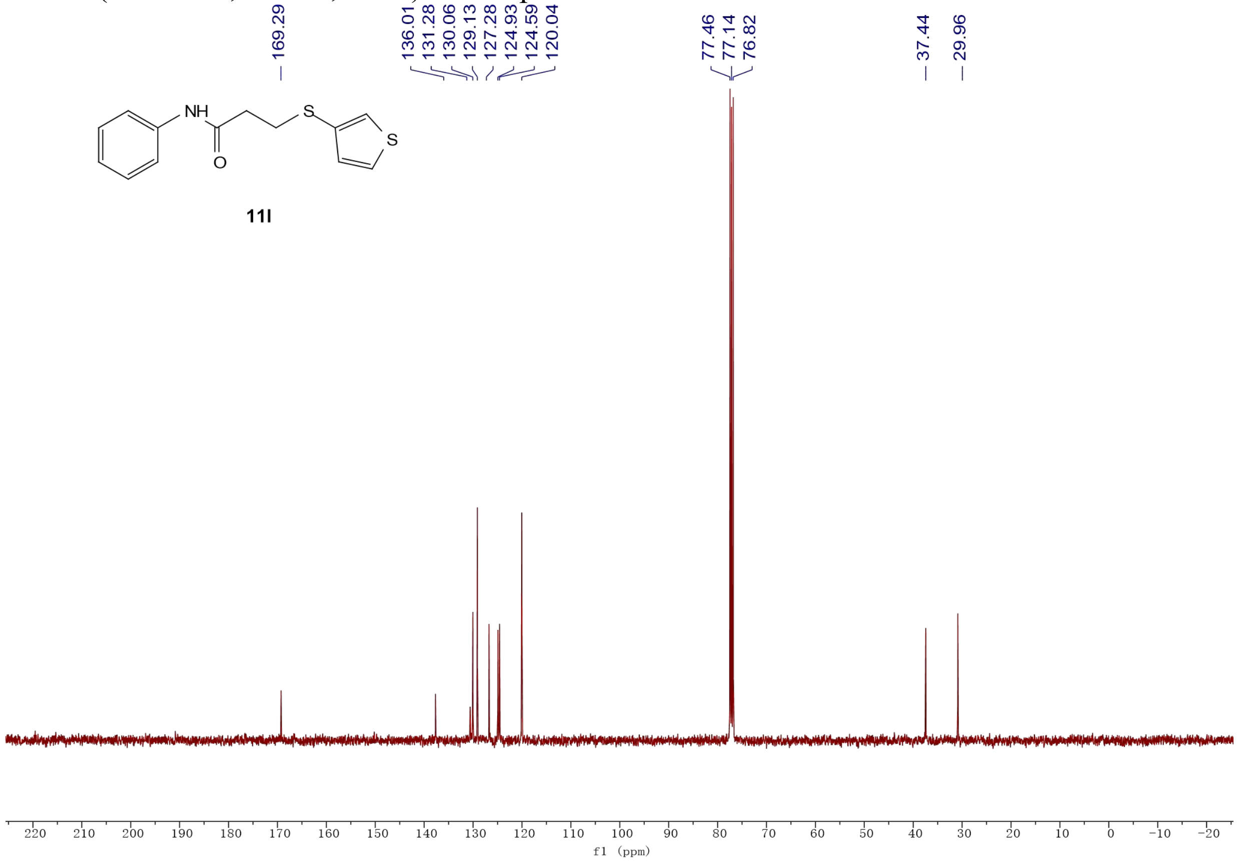

Supplementary Fig. 171. NMR of compound **11l**

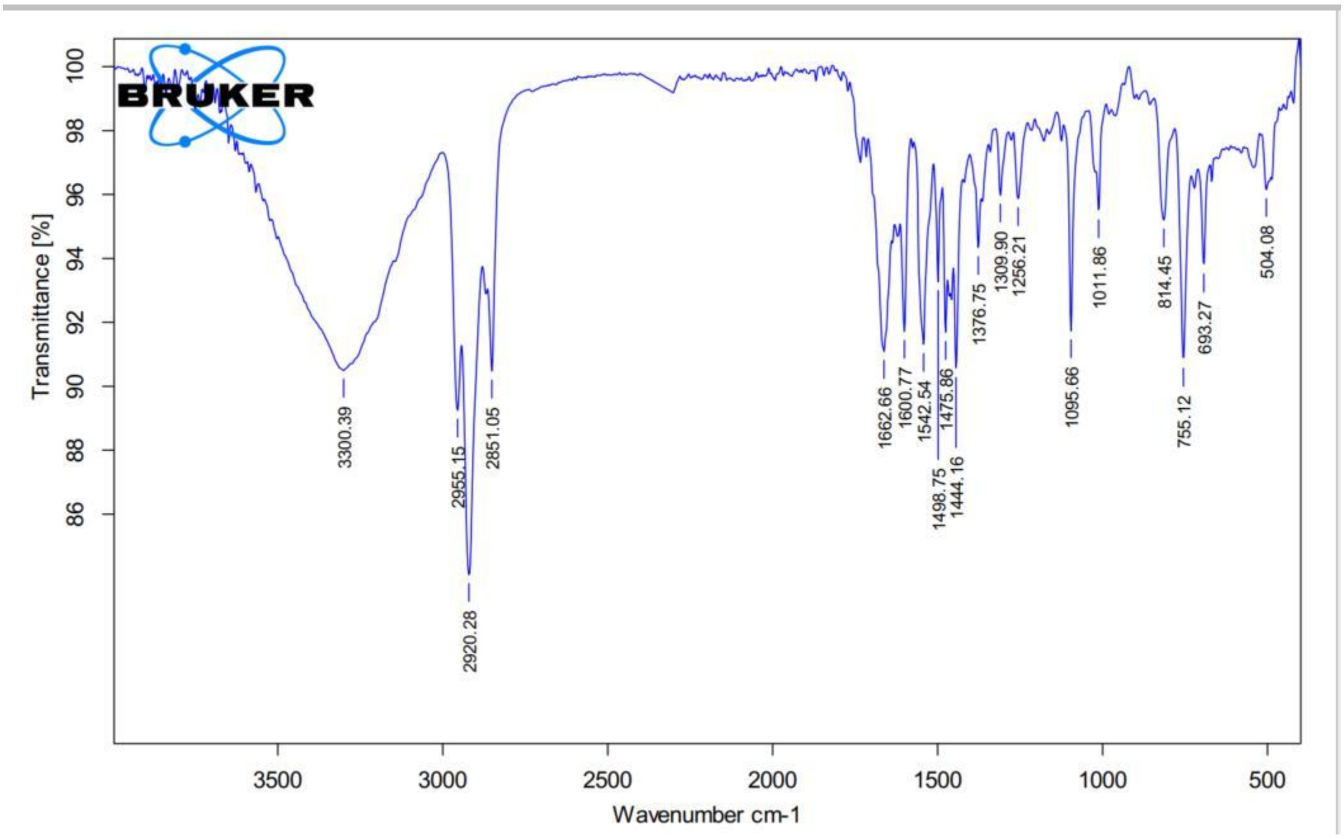

Supplementary Fig. 172. IR of compound **11l**

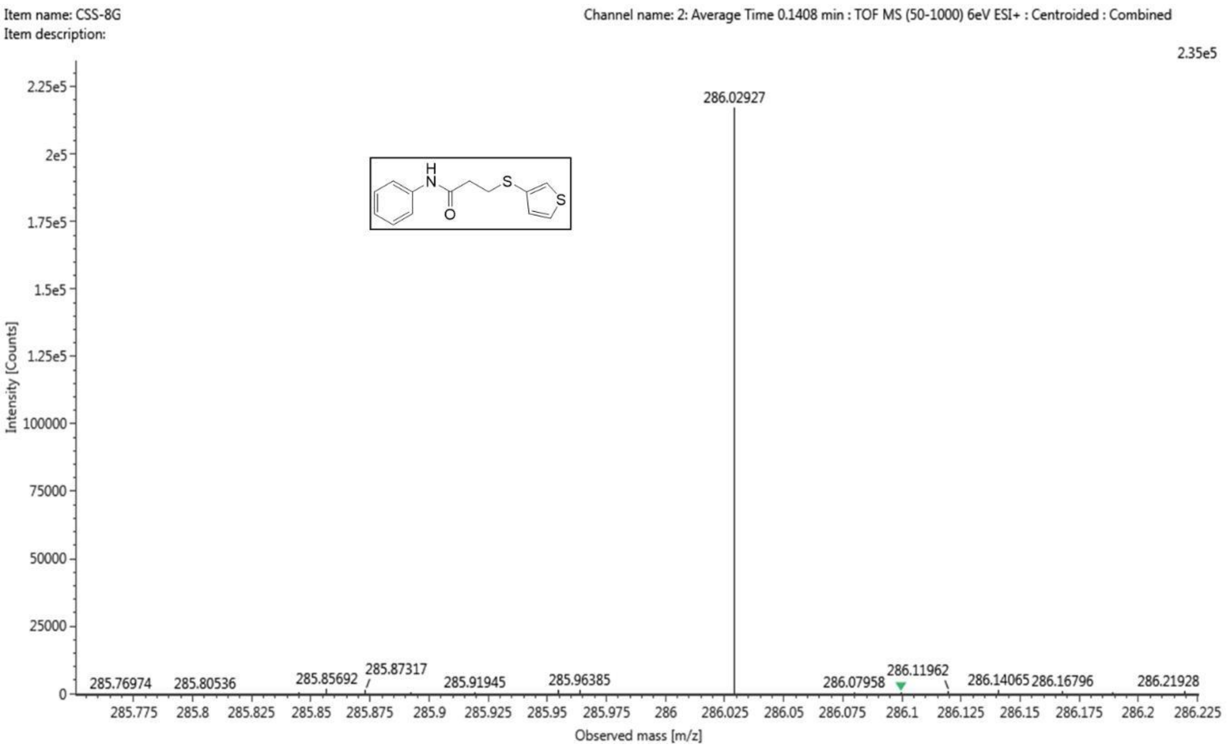

Supplementary Fig. 173. HR-MS of compound **11l**

^{1}H NMR (400 MHz, $CDCl_3$, 25°C) of compound **11m**

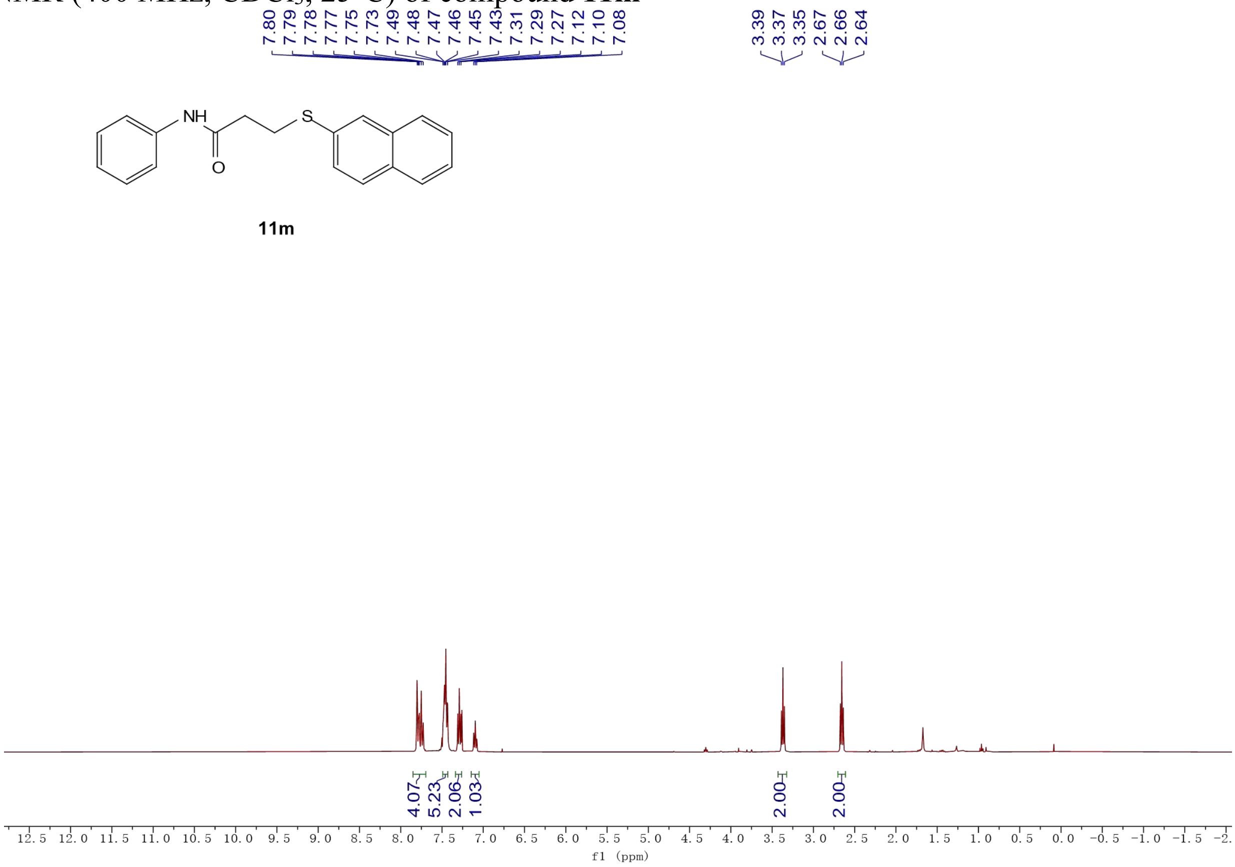

^{13}C NMR (101 MHz, $CDCl_3$, 25°C) of compound **11m**

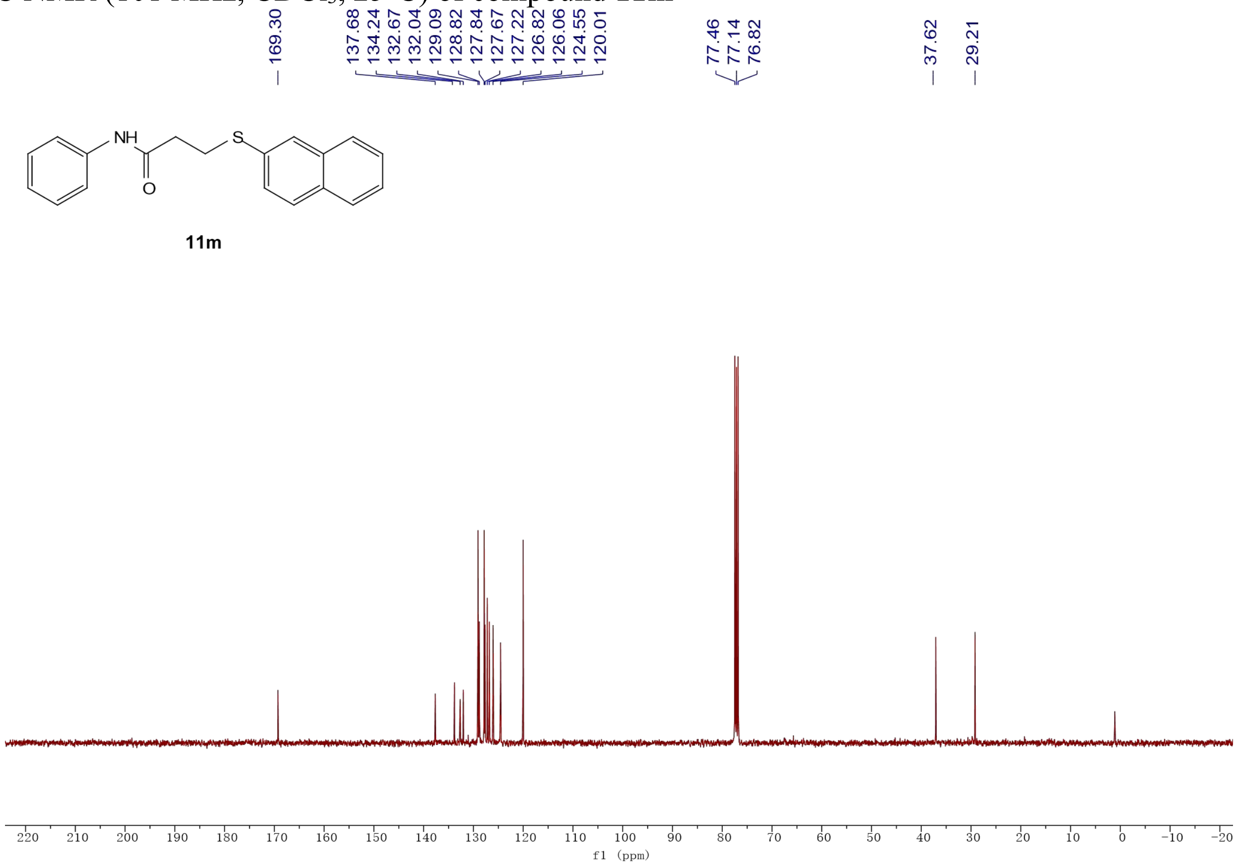

Supplementary Fig. 174. NMR of compound **11m**

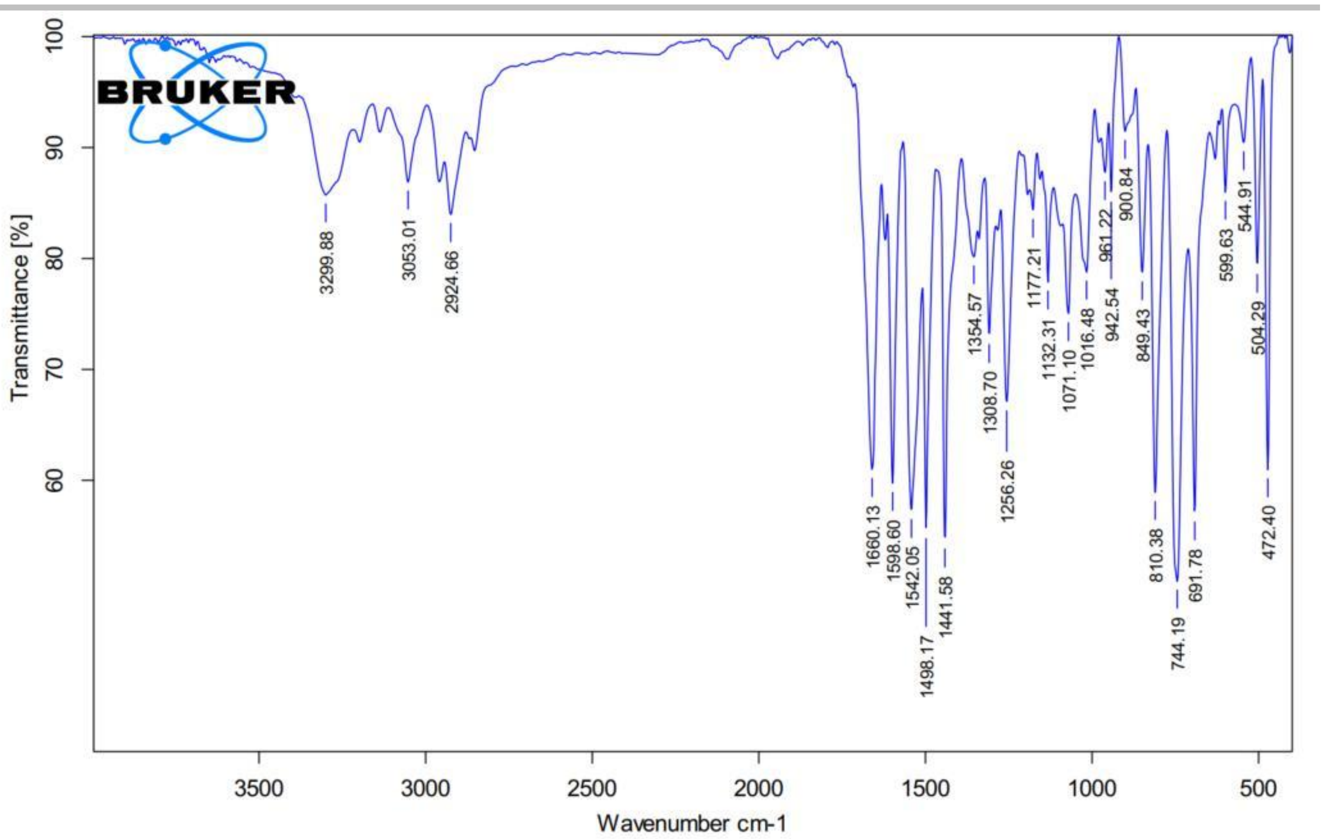

Supplementary Fig. 175. IR of compound **11m**

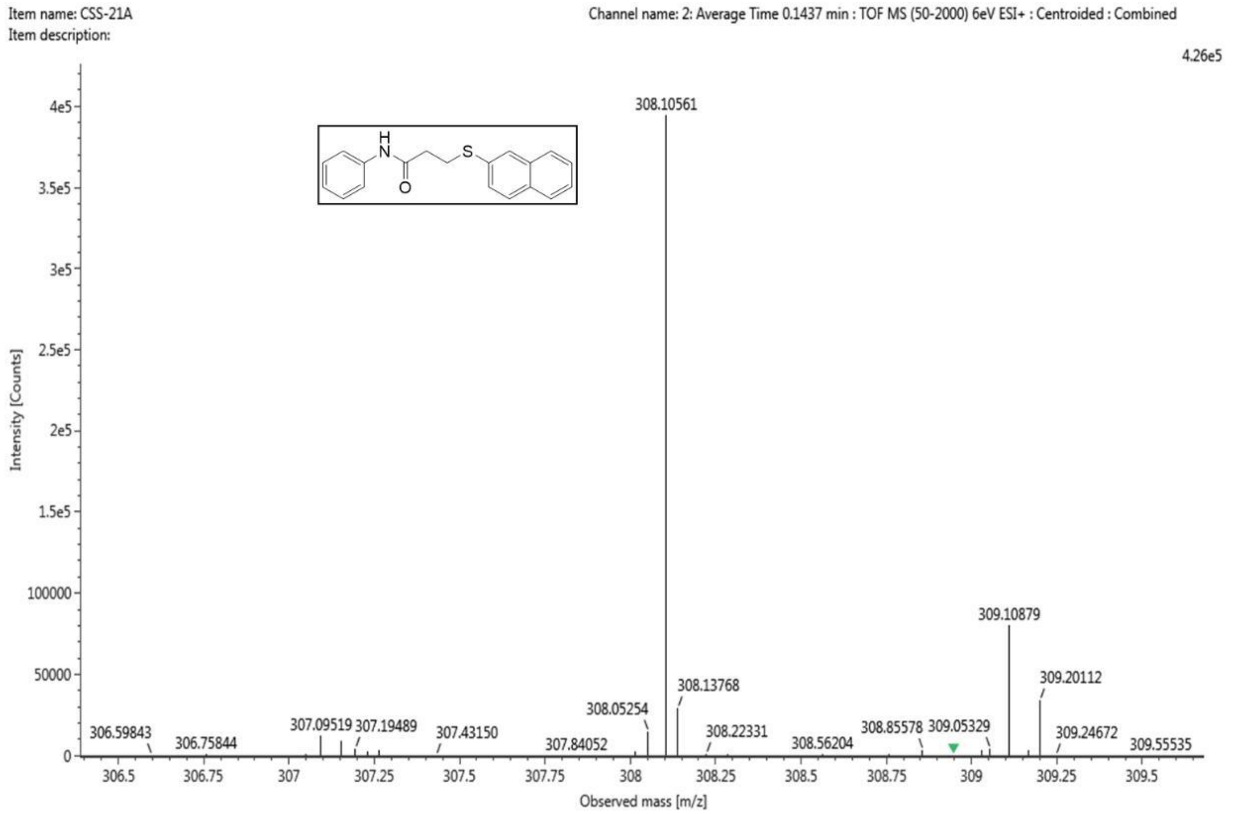

Supplementary Fig. 176. HR-MS of compound **11m**

^{1}H NMR (400 MHz, $CDCl_3$, 25°C) of compound **11n**

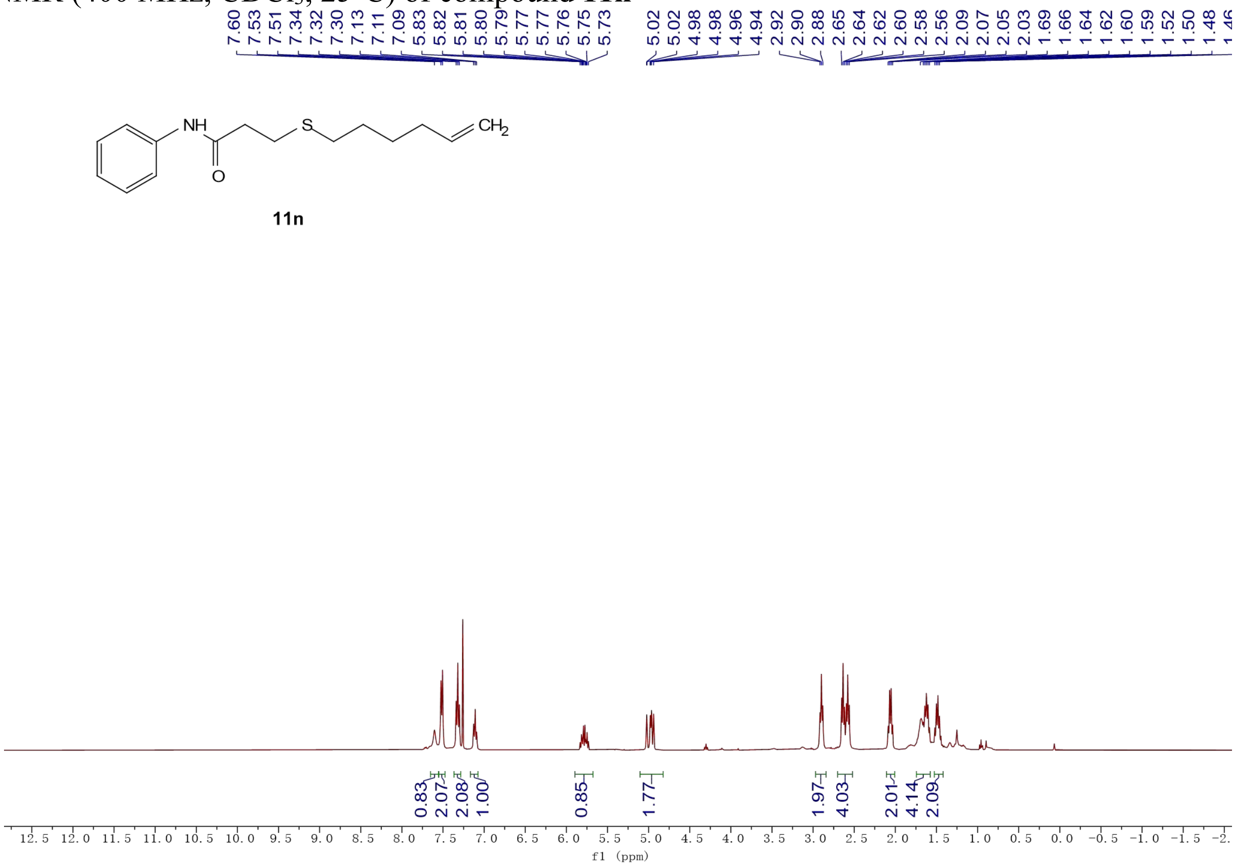

^{13}C NMR (101 MHz, $CDCl_3$, 25°C) of compound **11n**

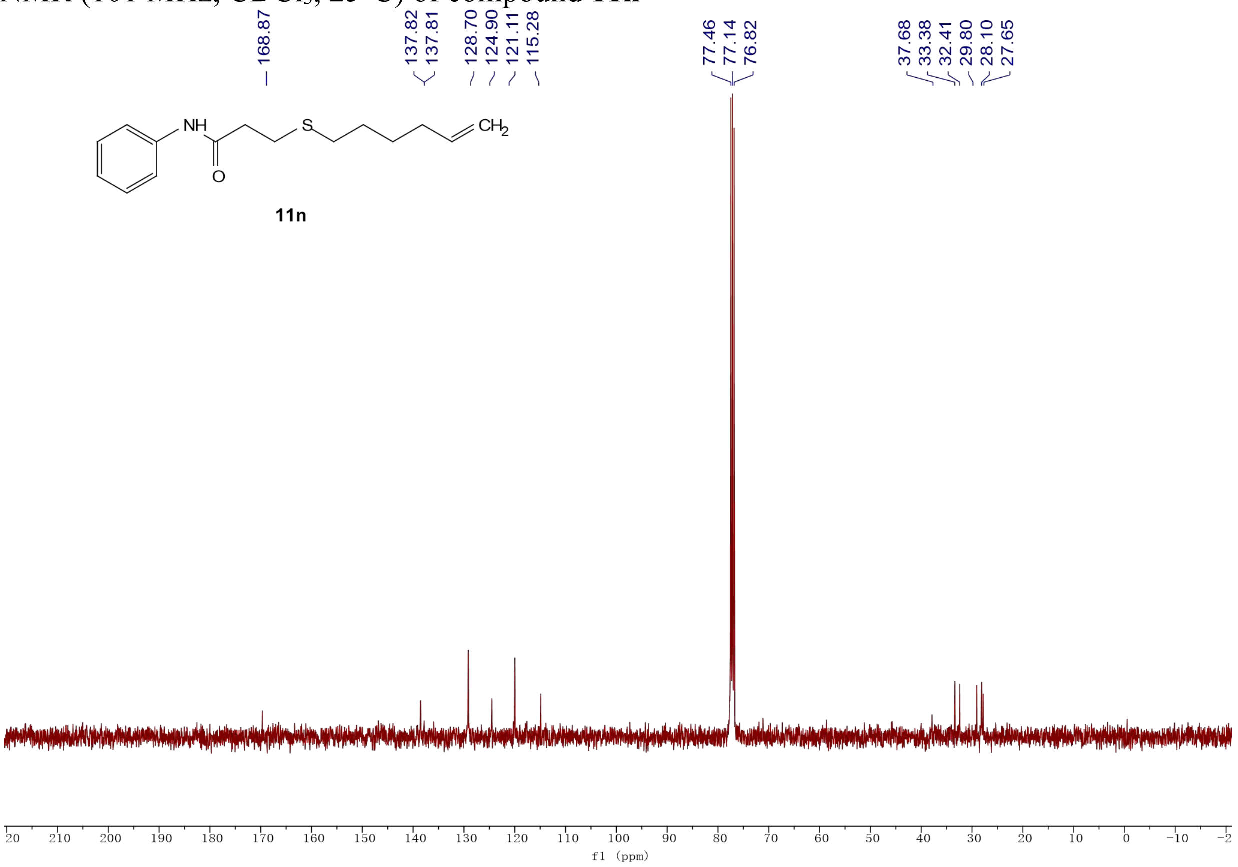

Supplementary Fig. 177. NMR of compound **11n**

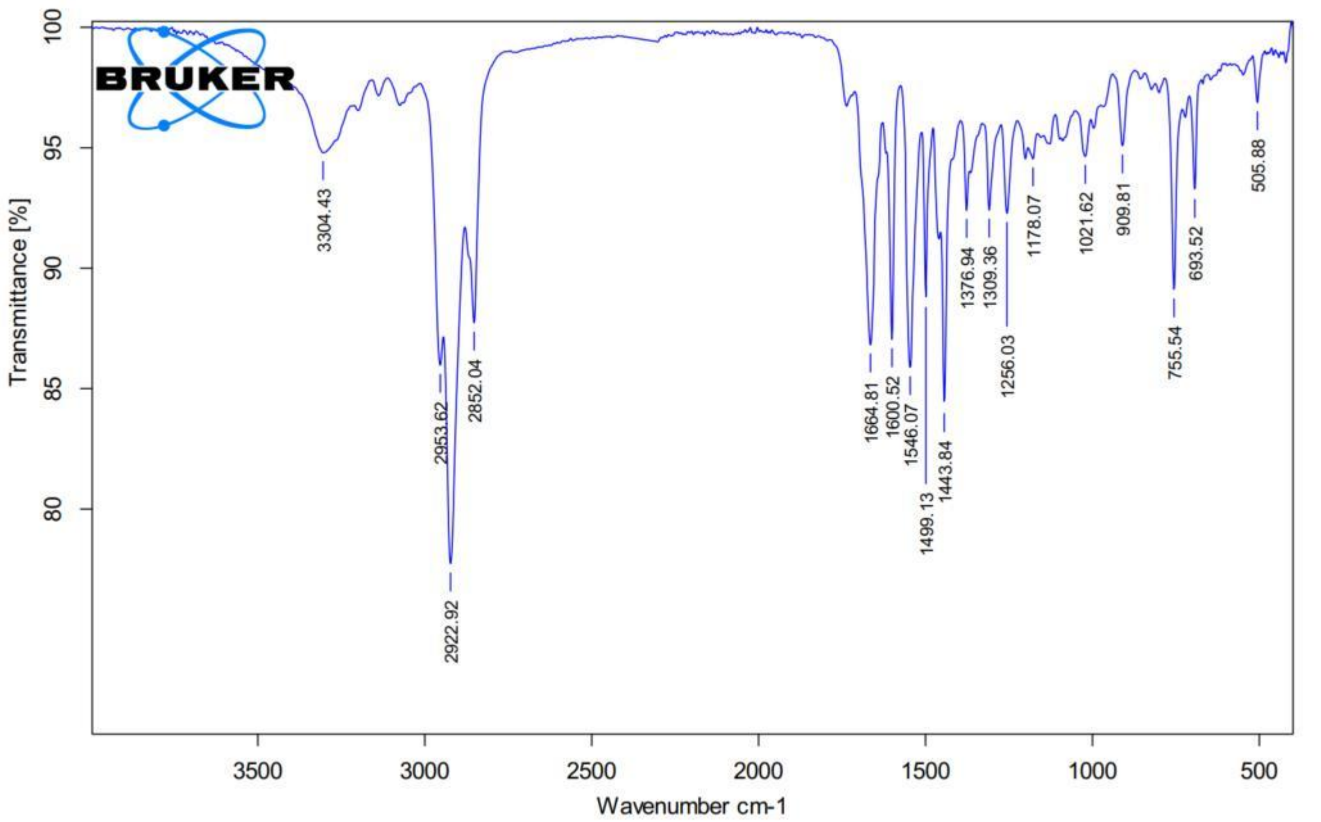

Supplementary Fig. 178. IR of compound **11n**

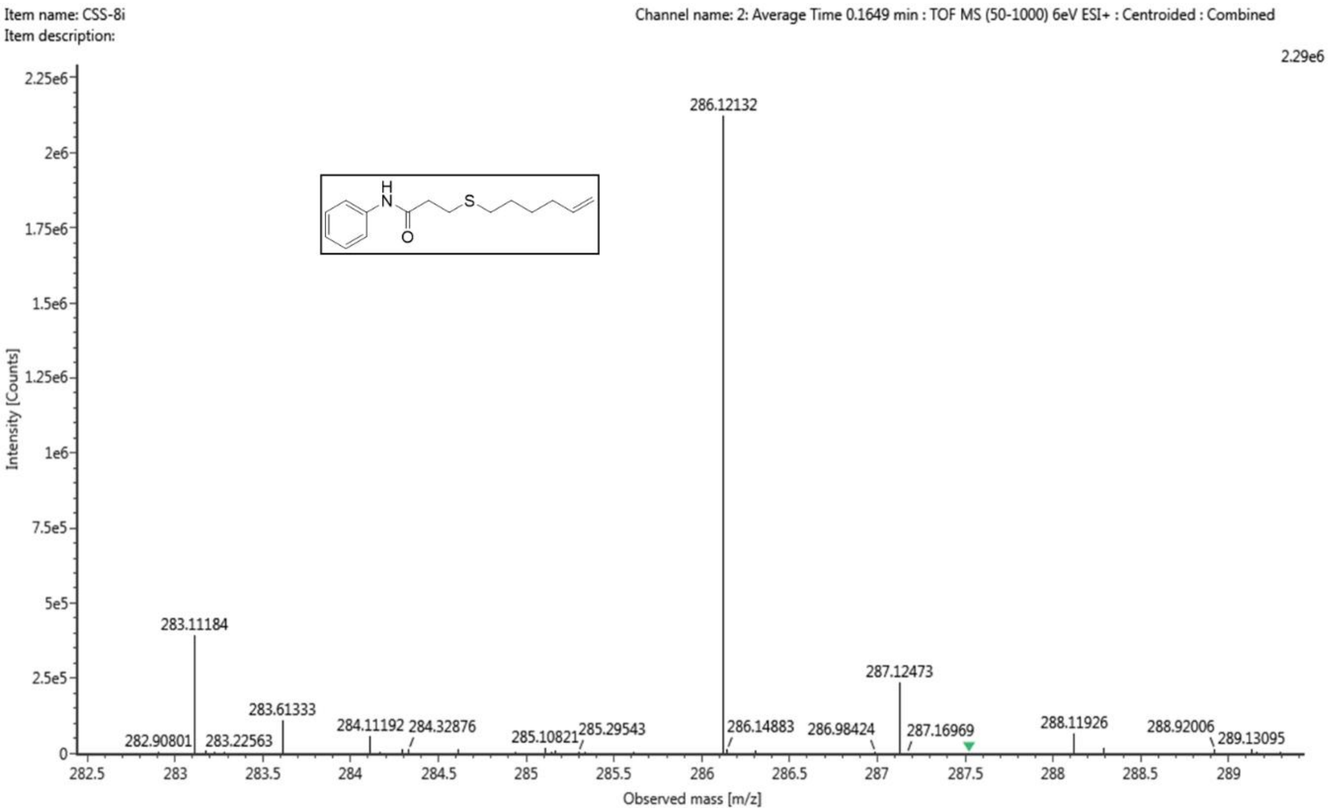

Supplementary Fig. 179. HR-MS of compound **11n**

^{1}H NMR (400 MHz, $CDCl_3$, 25°C) of compound **12a**

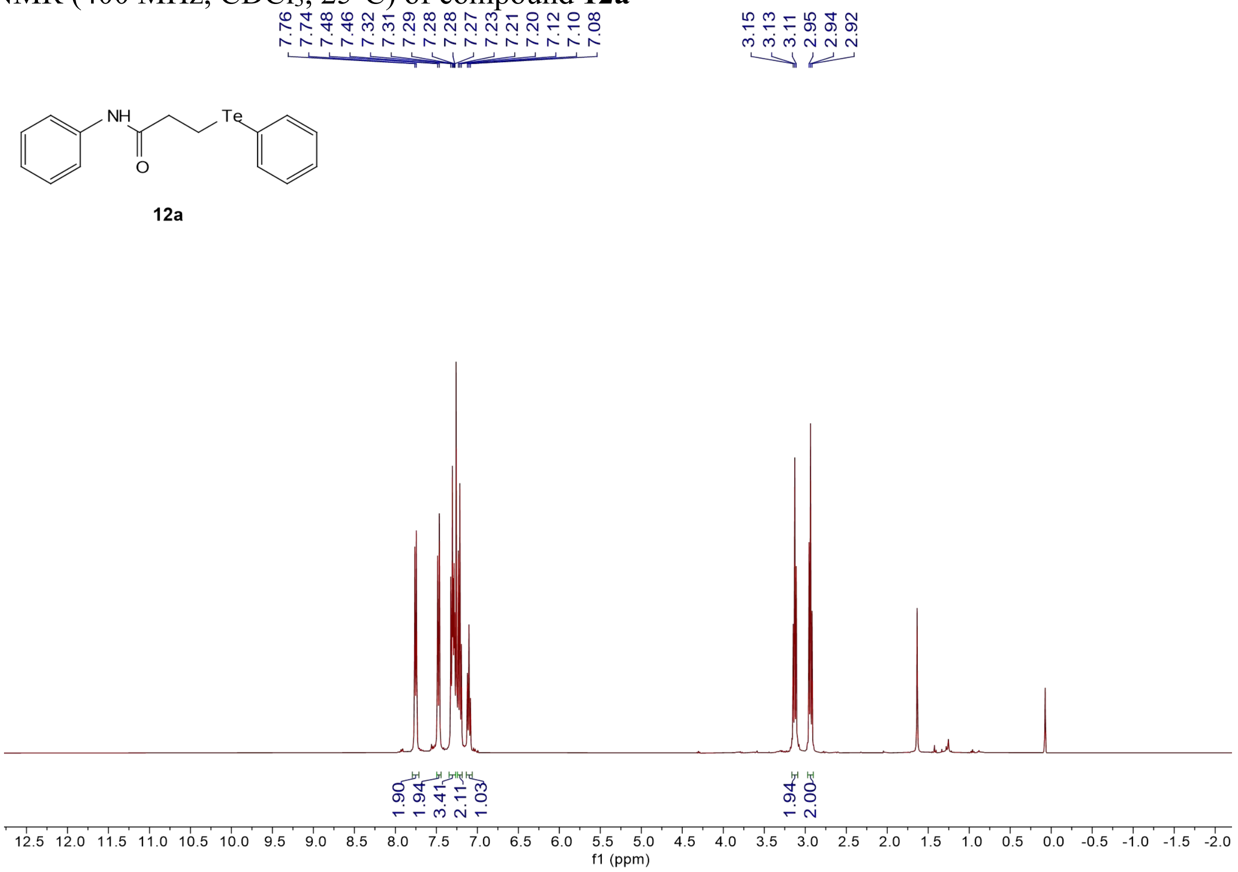

^{13}C NMR (101 MHz, $CDCl_3$, 25°C) of compound **12a**

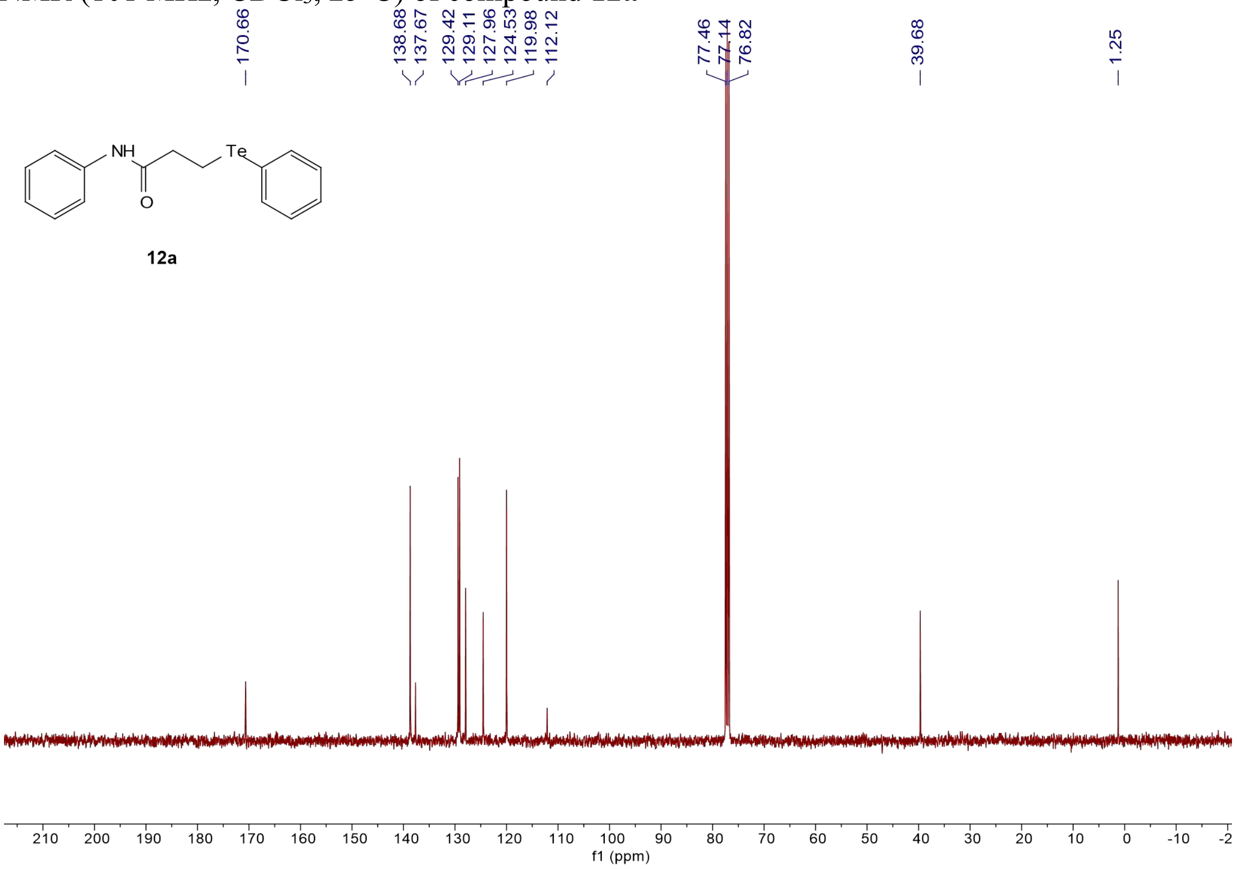

Supplementary Fig. 180. NMR of compound **12a**

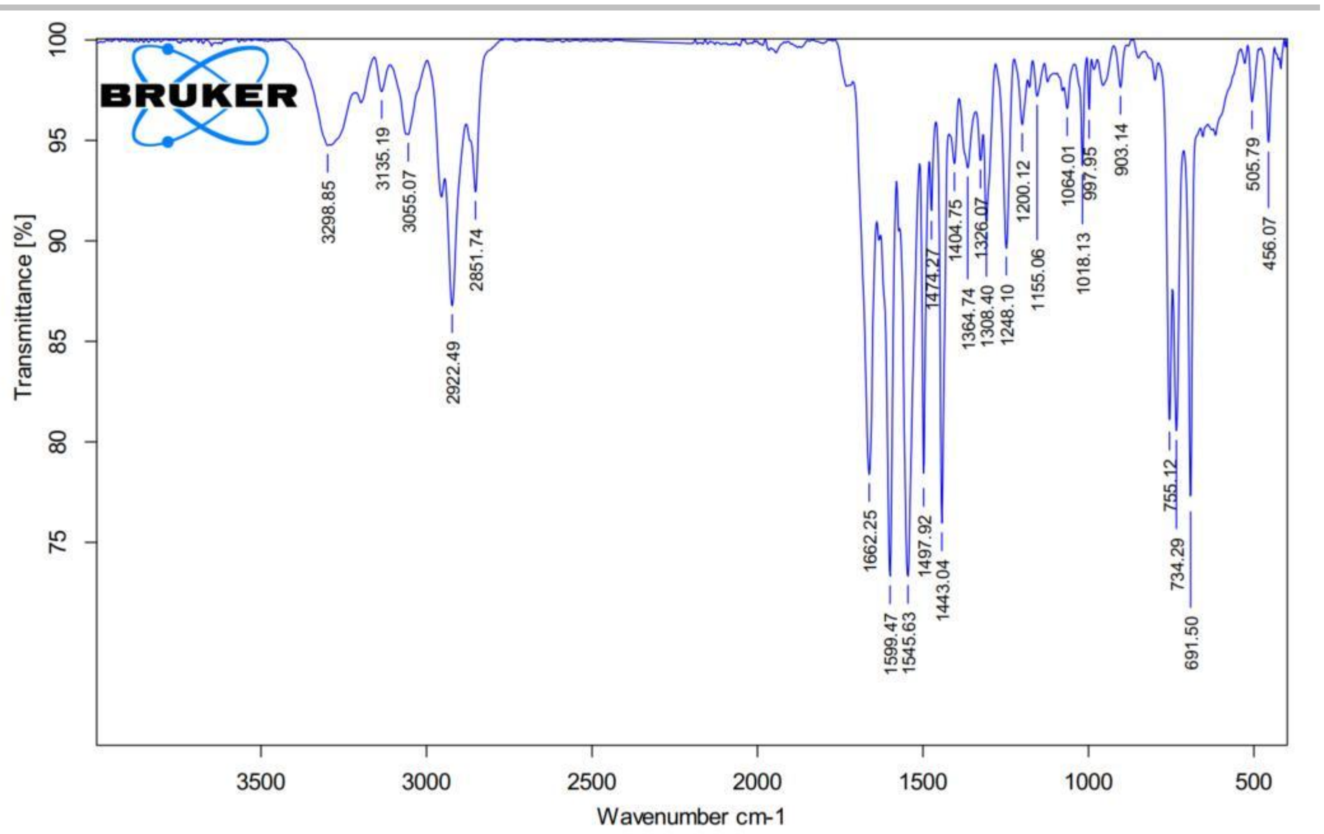

Supplementary Fig. 181. IR of compound **12a**

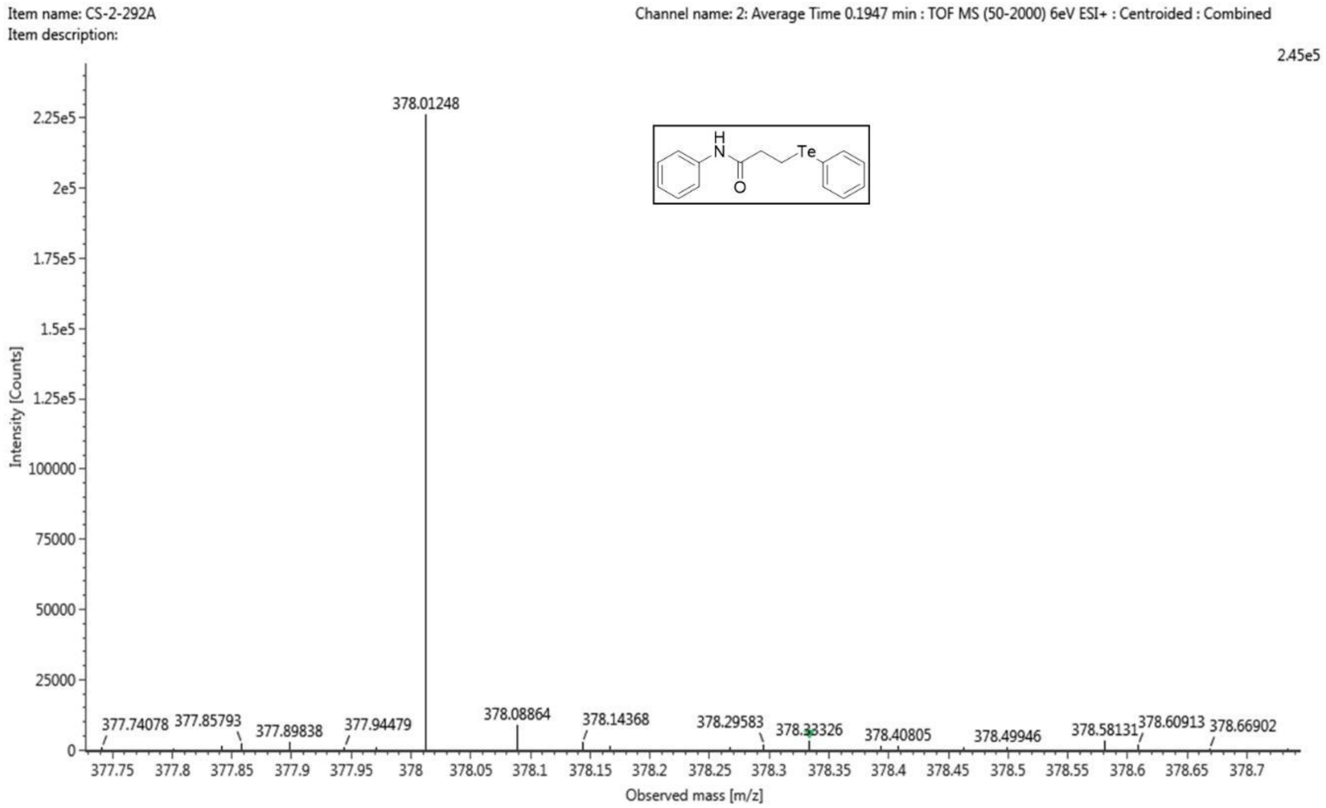

Supplementary Fig. 182. HR-MS of compound **12a**

^{1}H NMR (400 MHz, $CDCl_3$, 25°C) of compound **12b**

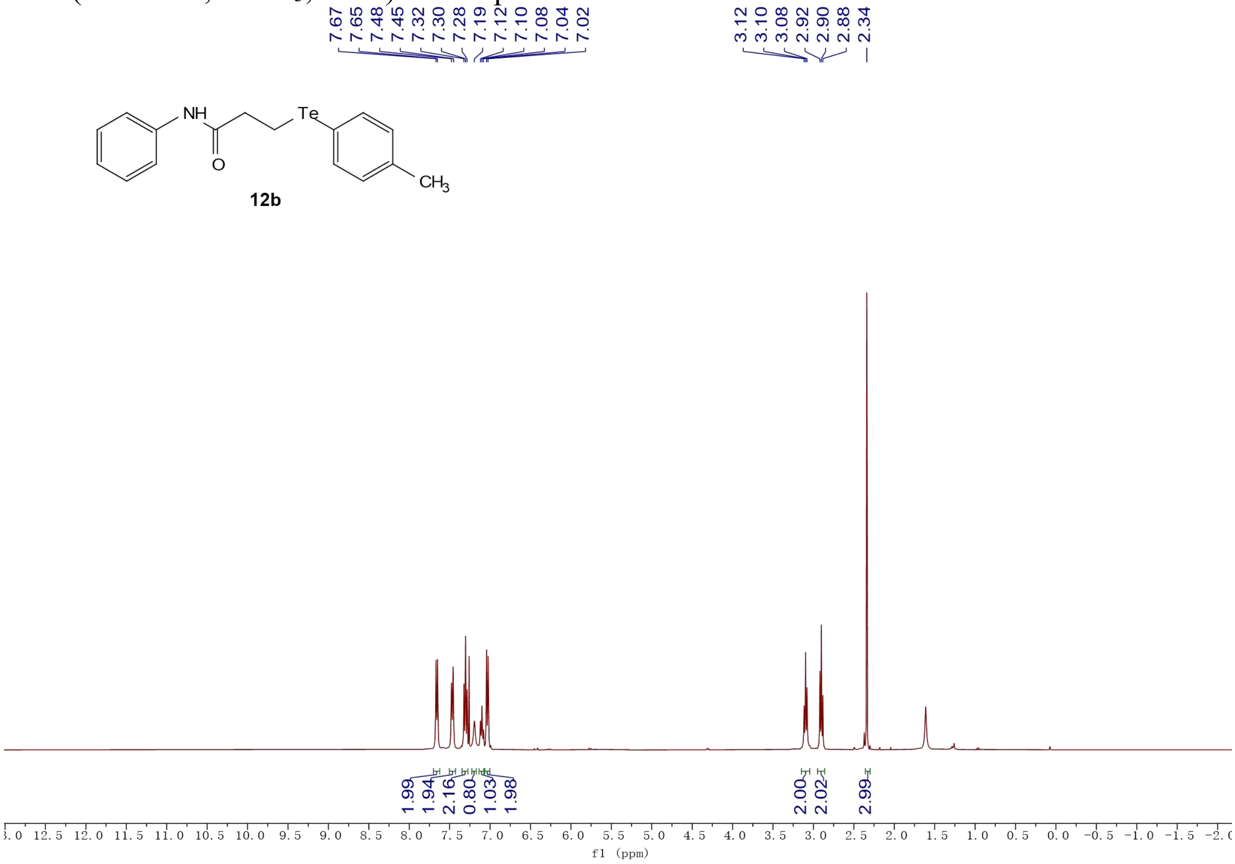

^{13}C NMR (150 MHz, $CDCl_3$, 25°C) of compound **12b**

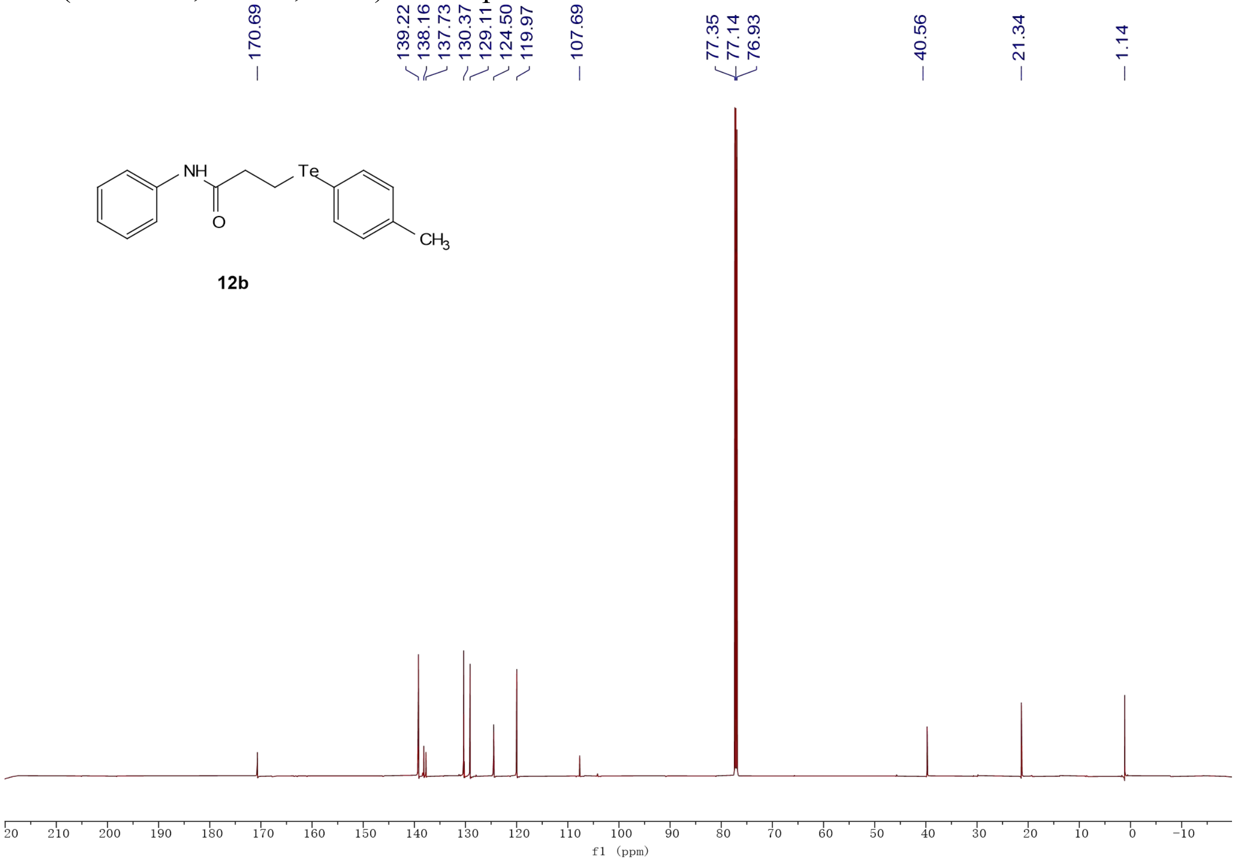

Supplementary Fig. 183. NMR of compound **12b**

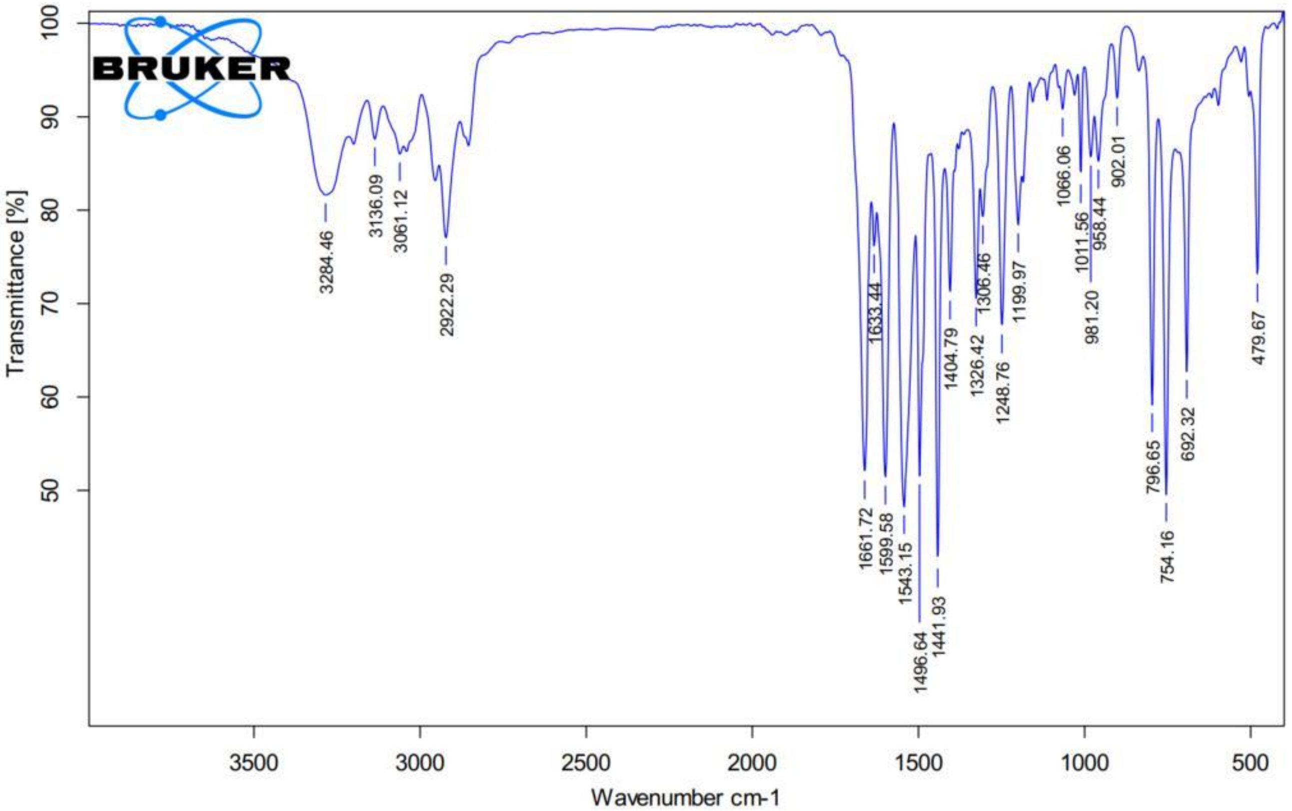

Supplementary Fig. 184. IR of compound **12b**

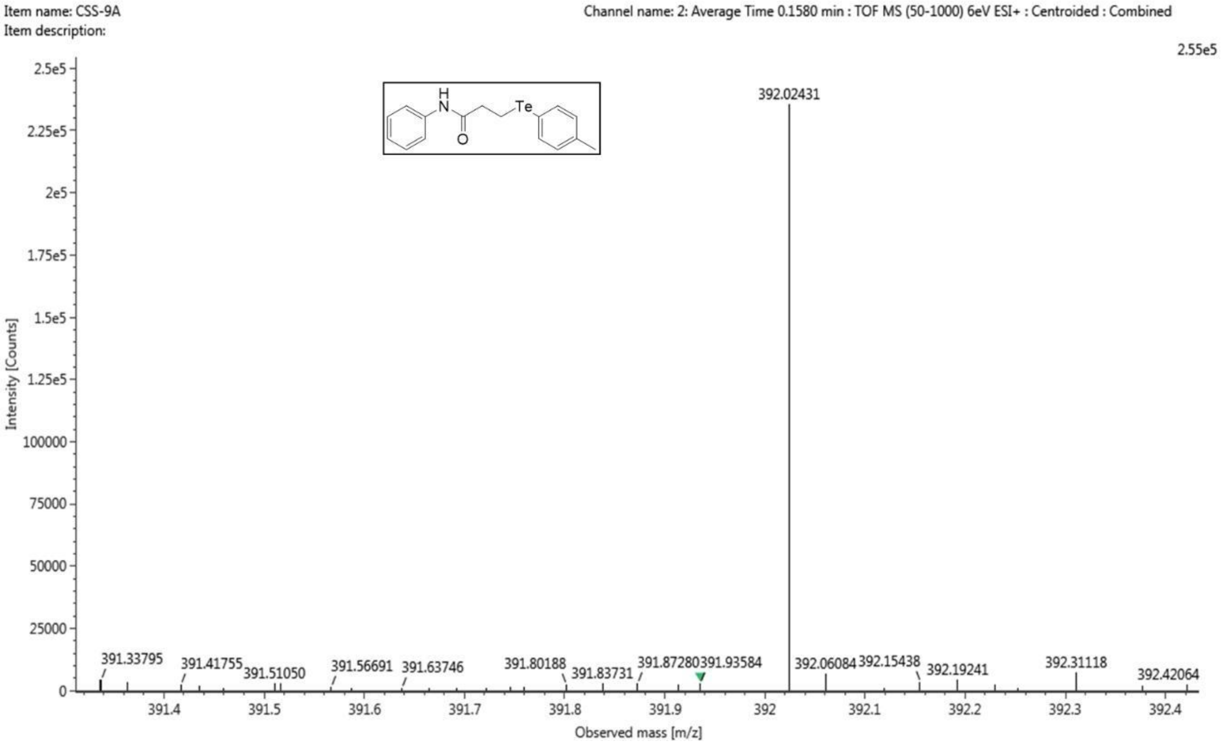

Supplementary Fig. 185. HR-MS of compound **12b**

^{1}H NMR (400 MHz, $CDCl_3$, 25℃) of compound **12c**

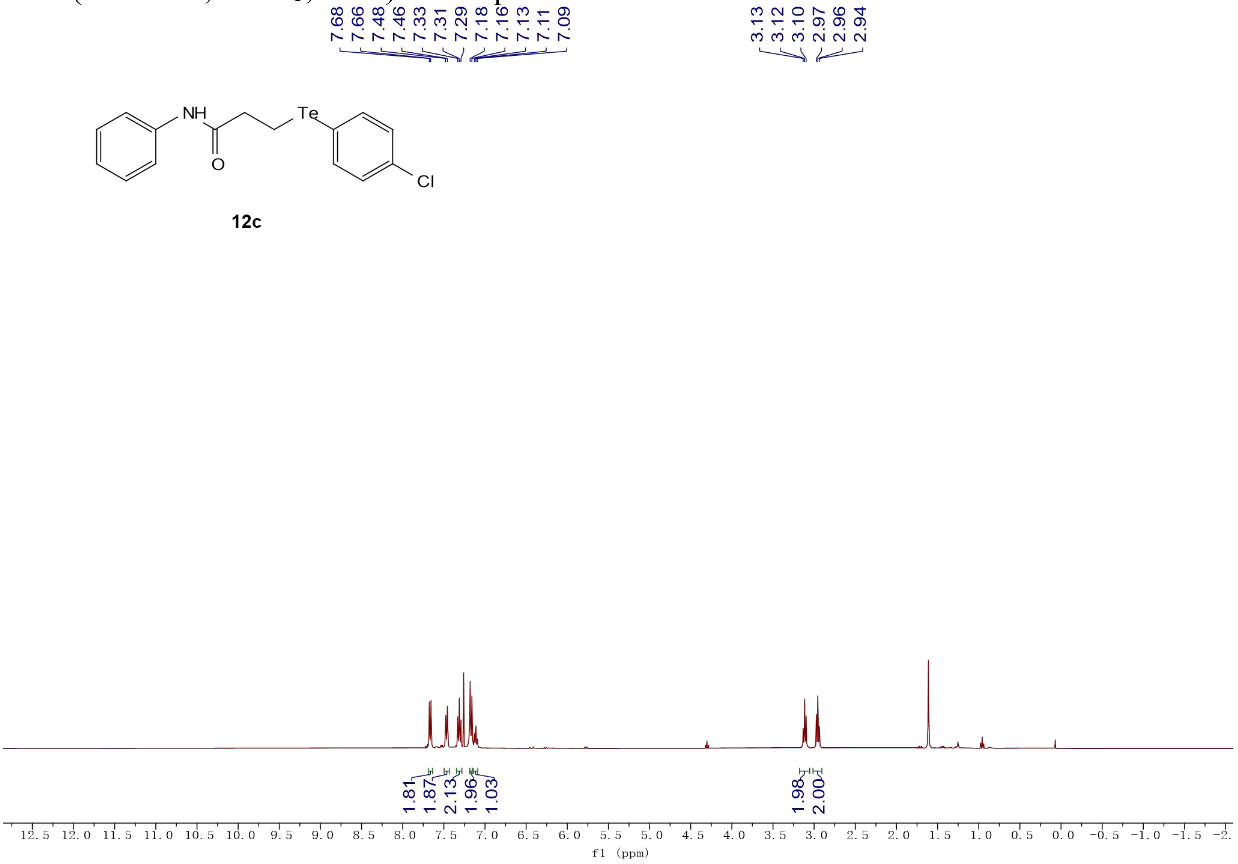

^{13}C NMR (101 MHz, $CDCl_3$, 25℃) of compound **12c**

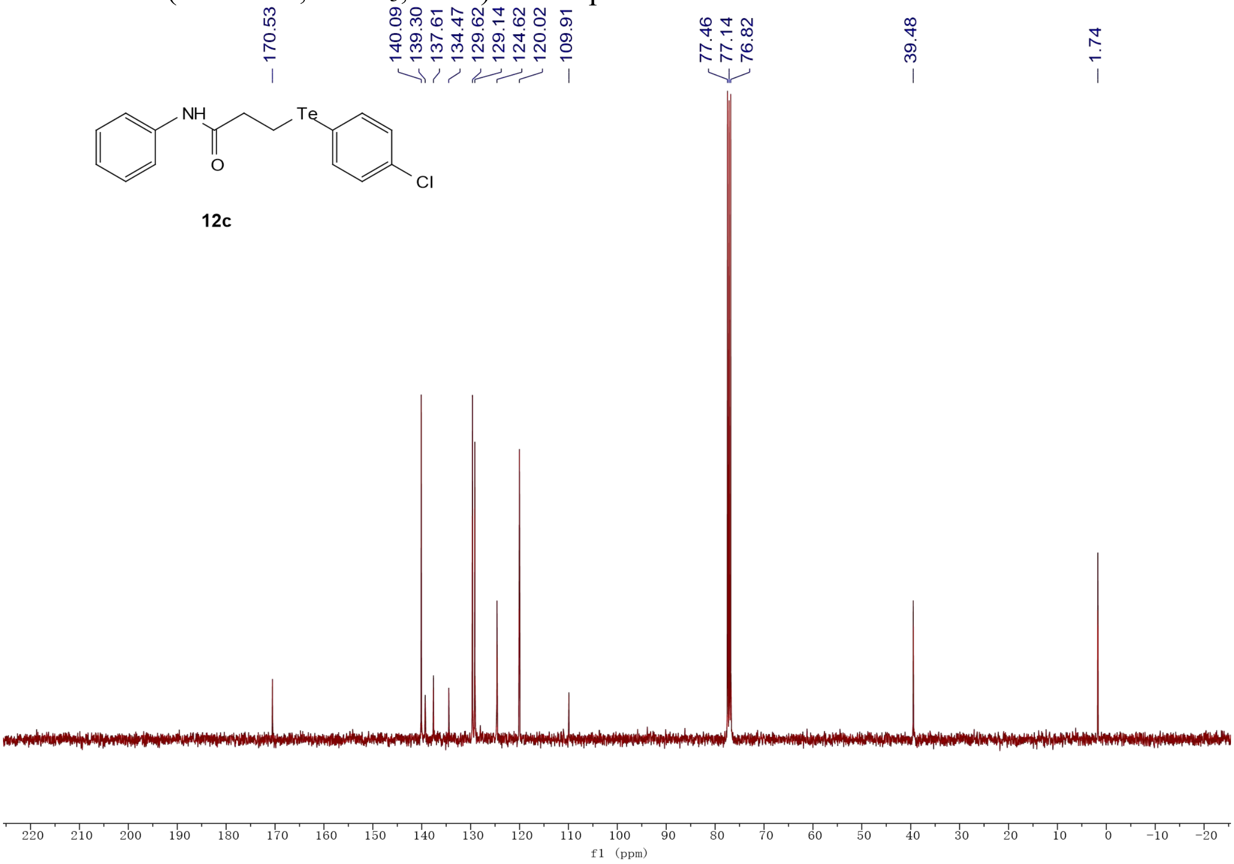

Supplementary Fig. 186. NMR of compound **12c**

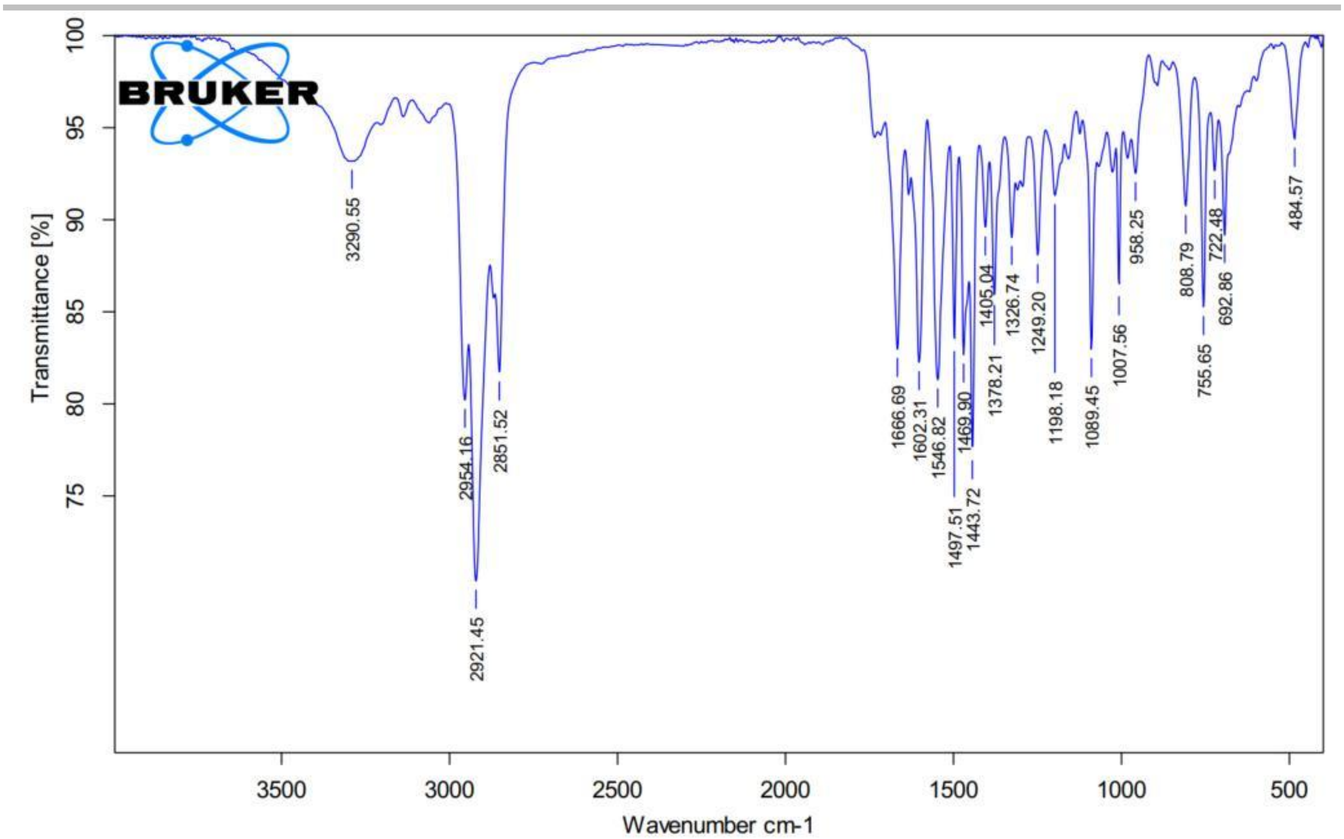

Supplementary Fig. 187. IR of compound **12c**

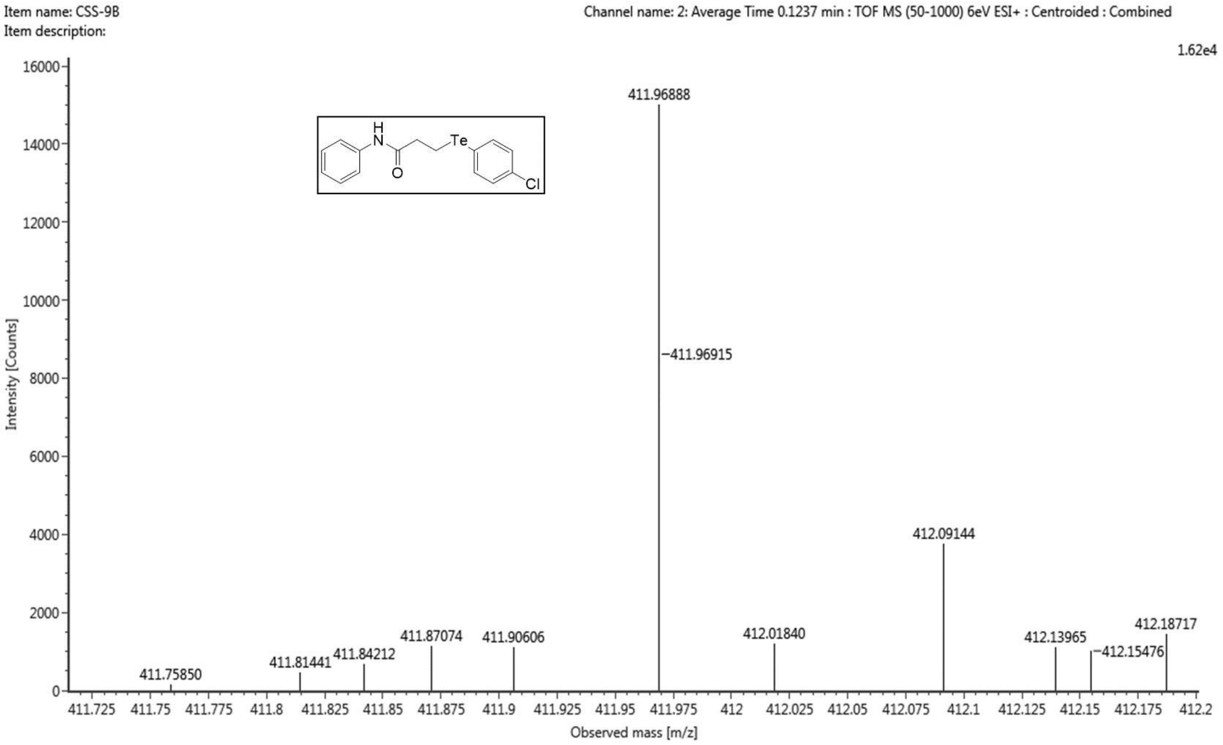

Supplementary Fig. 188. HR-MS of compound **12c**

^1H NMR (400 MHz, $CDCl_3$, 25°C) of compound **12d**

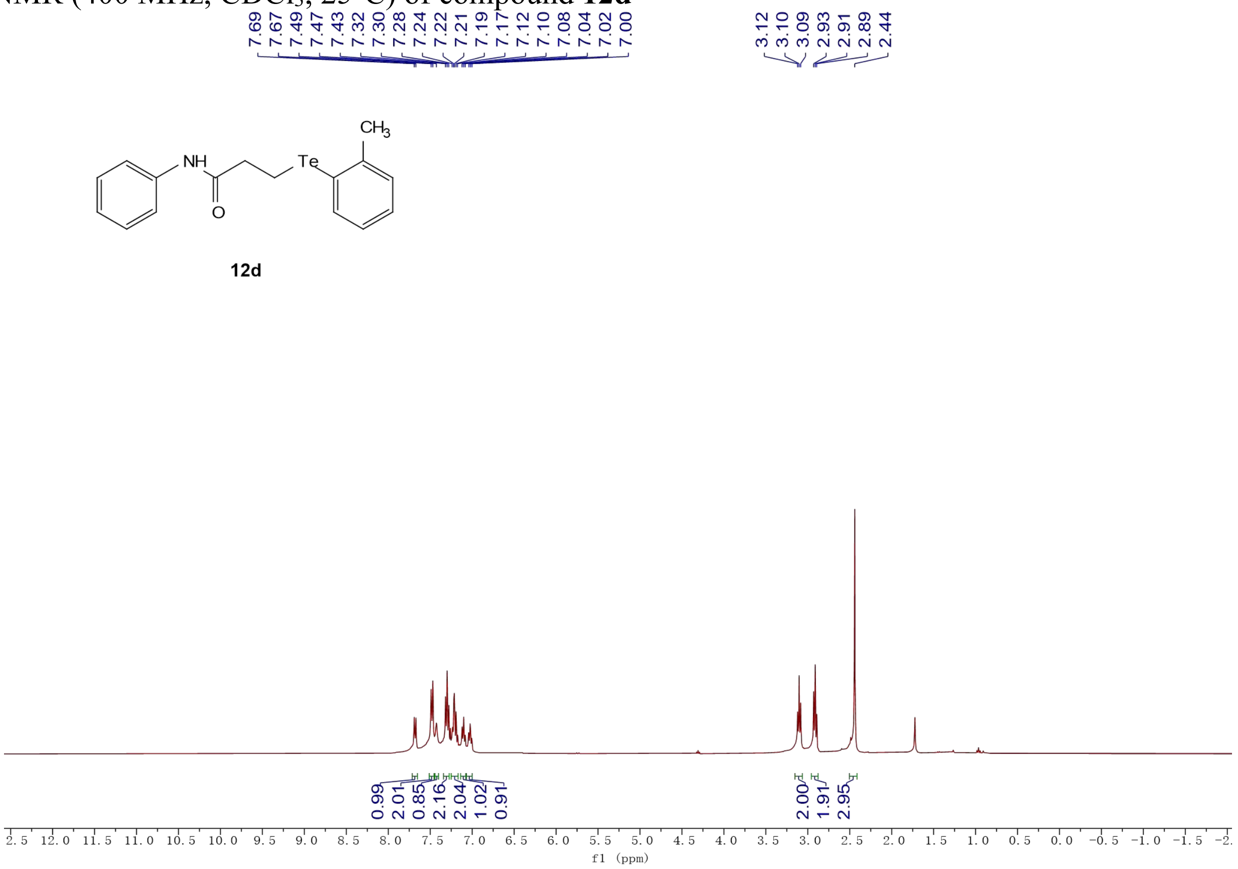

^{13}C NMR (101 MHz, $CDCl_3$, 25°C) of compound **12d**

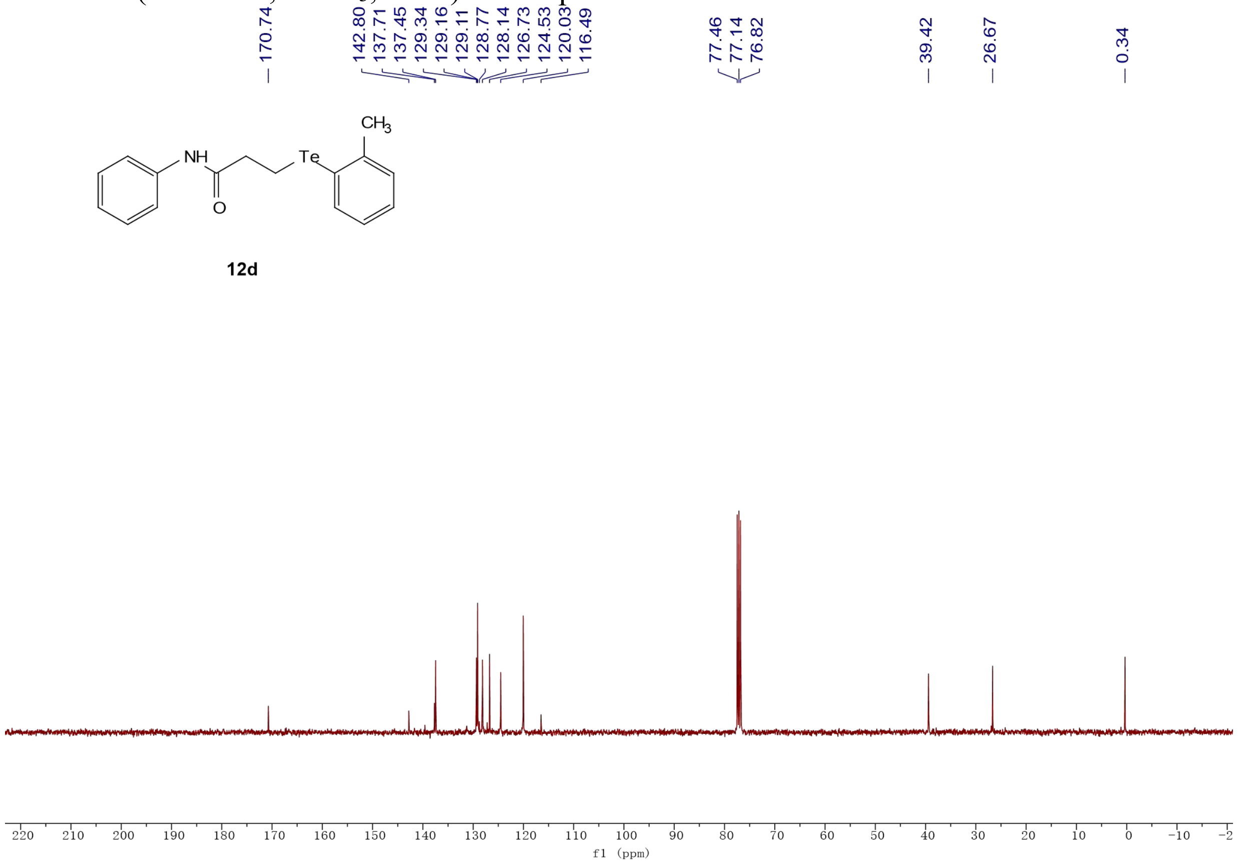

Supplementary Fig. 189. NMR of compound **12d**

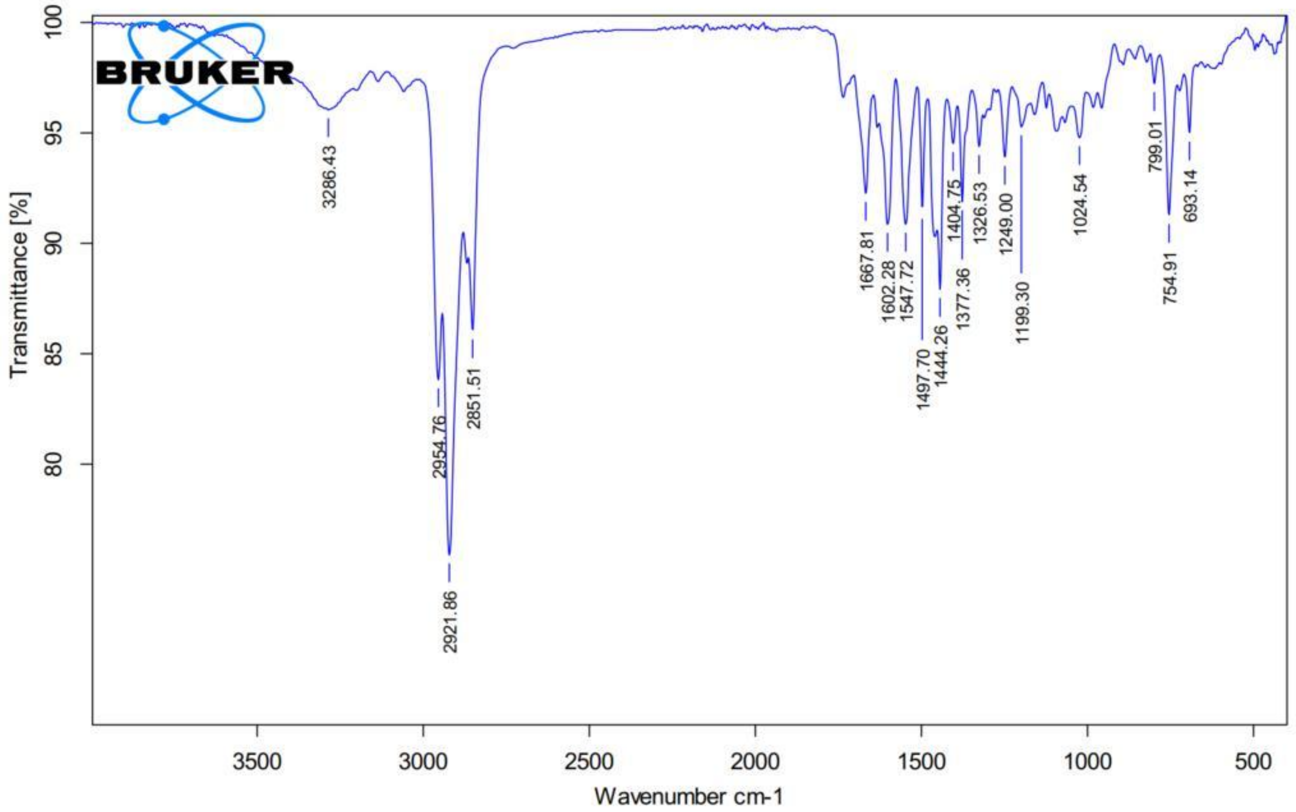

Supplementary Fig. 190. IR of compound **12d**

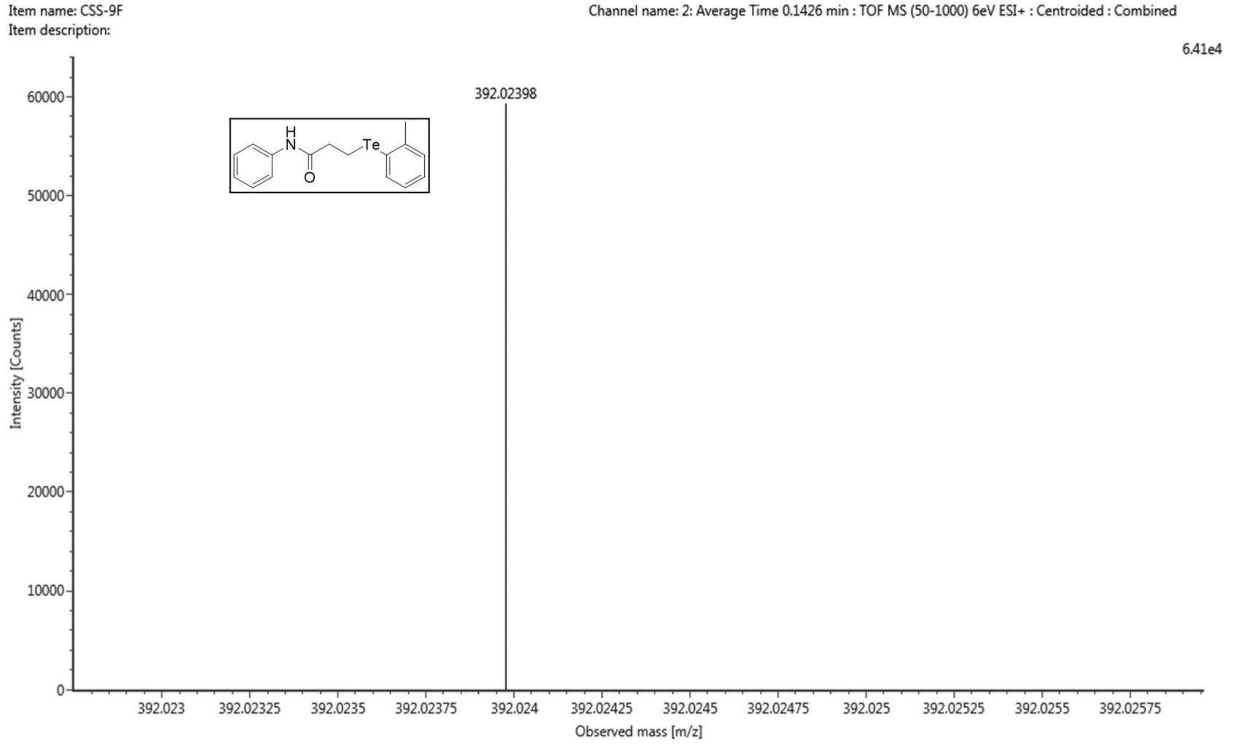

Supplementary Fig. 191. HR-MS of compound **12d**

^{1}H NMR (400 MHz, $CDCl_3$, 25°C) of compound **12e**

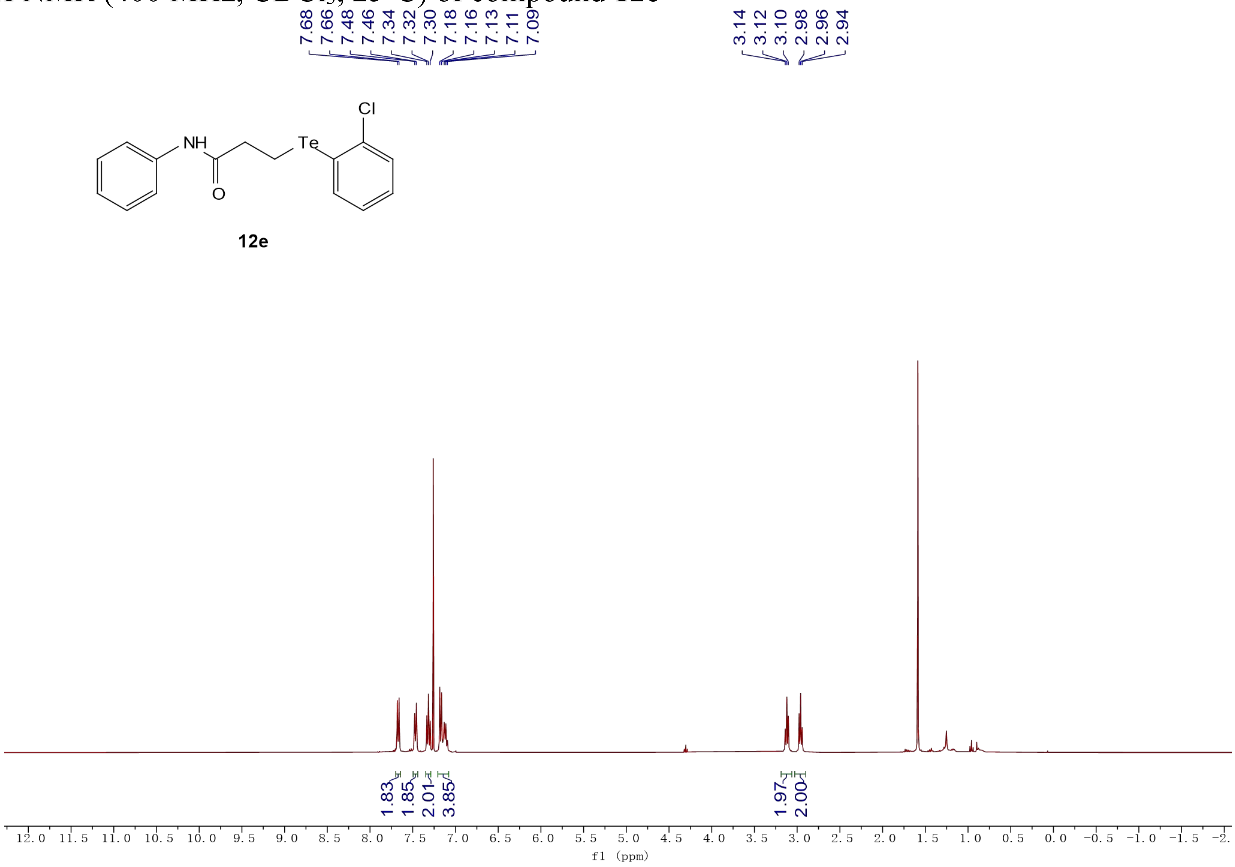

^{13}C NMR (101 MHz, $CDCl_3$, 25°C) of compound **12e**

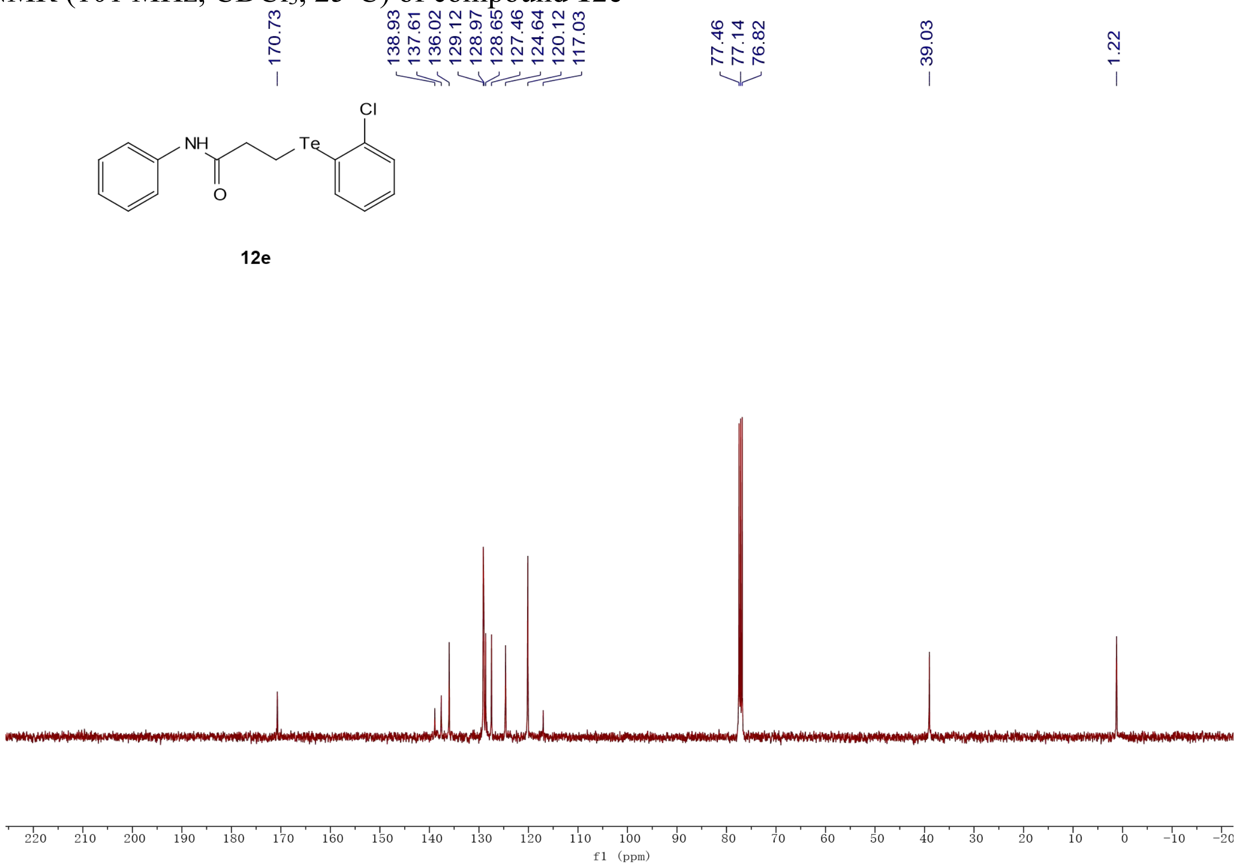

Supplementary Fig. 192. NMR of compound **12e**

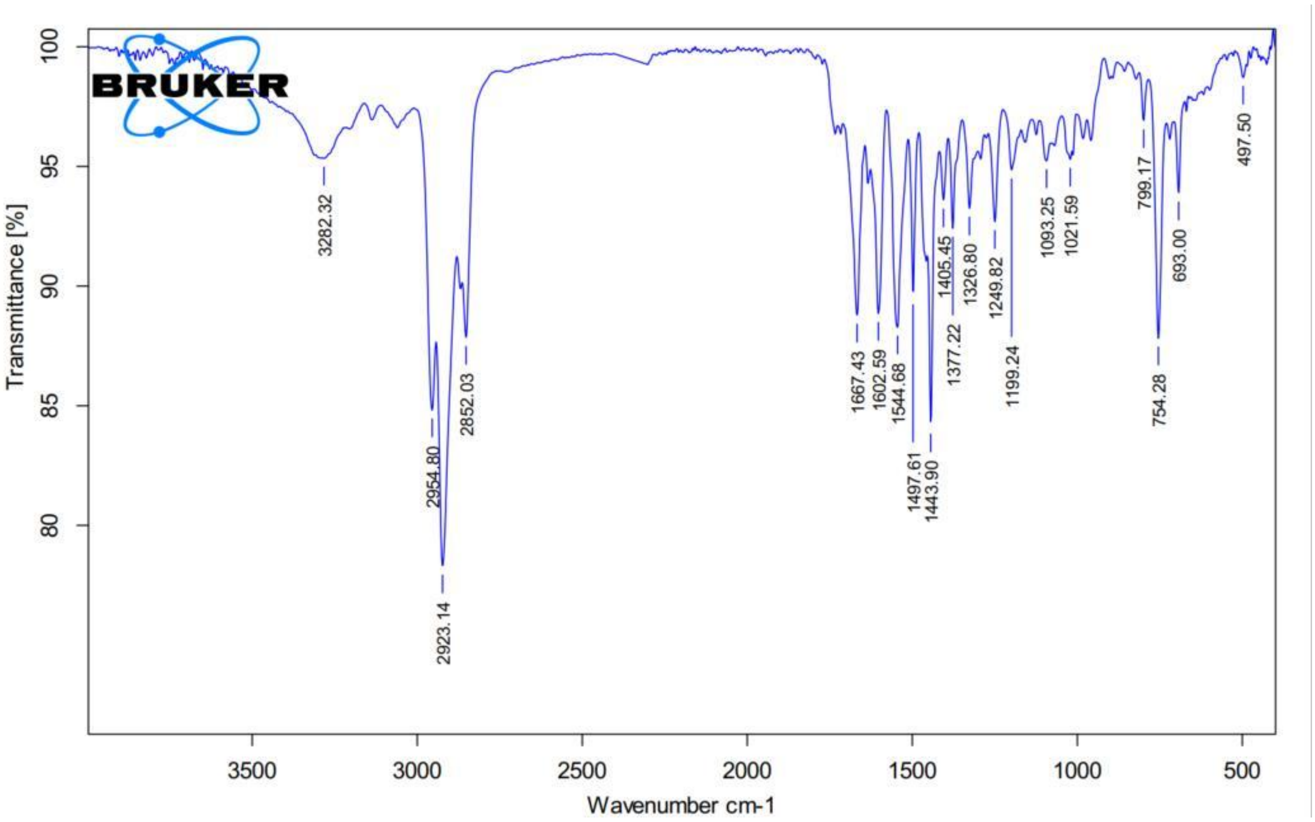

Supplementary Fig. 193. IR of compound **12e**

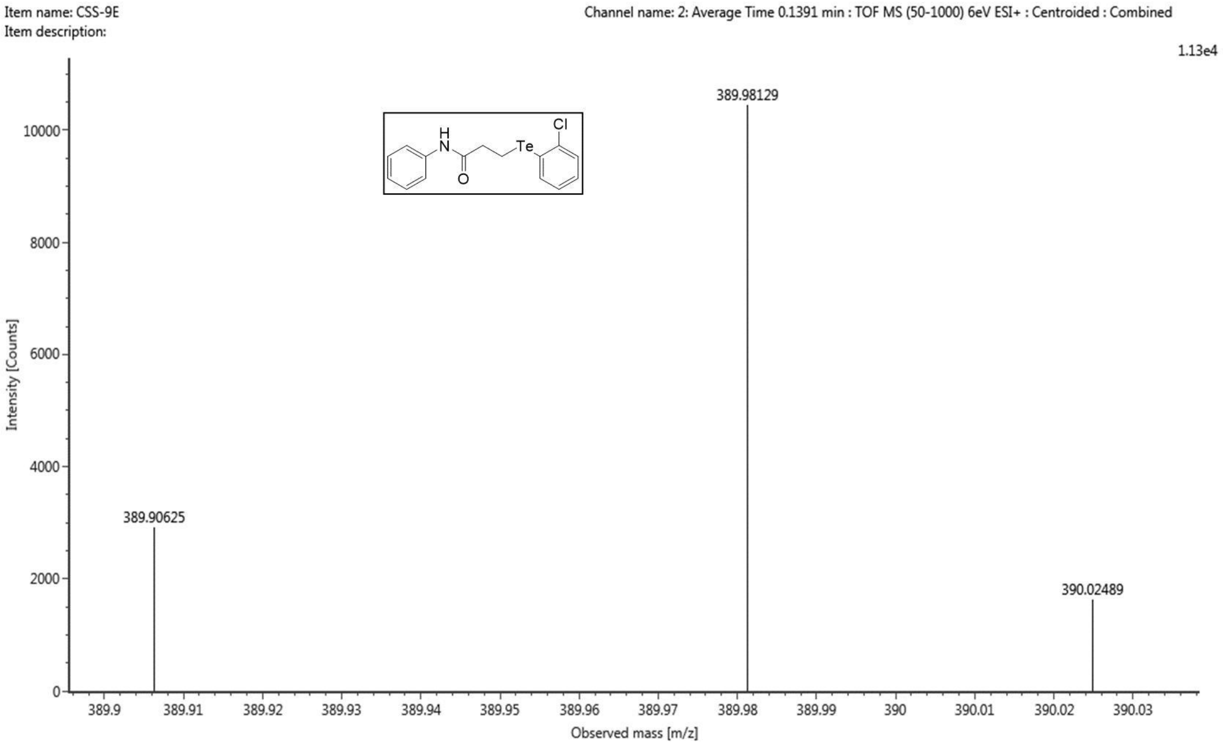

Supplementary Fig. 194. HR-MS of compound **12e**

^{1}H NMR (400 MHz, $CDCl_3$, 25°C) of compound **12f**

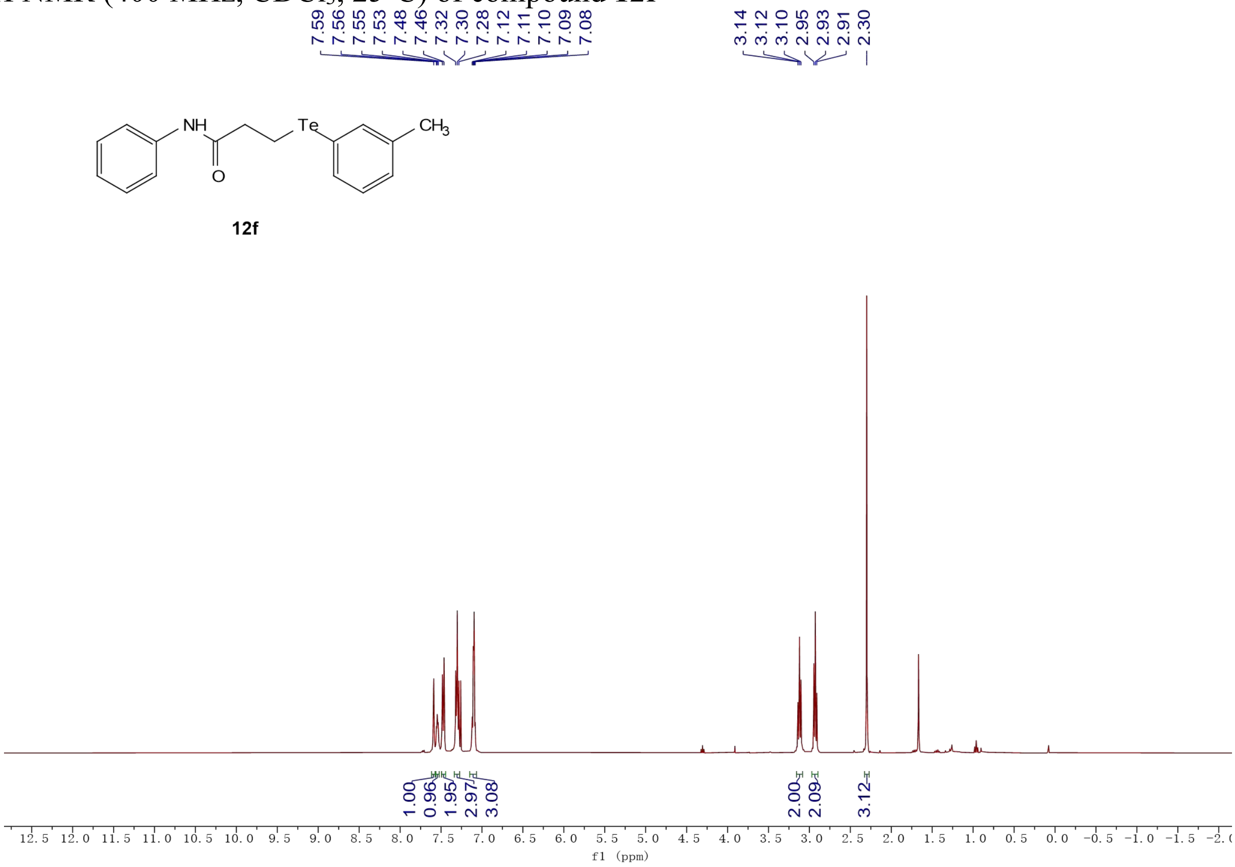

^{13}C NMR (101 MHz, $CDCl_3$, 25°C) of compound **12f**

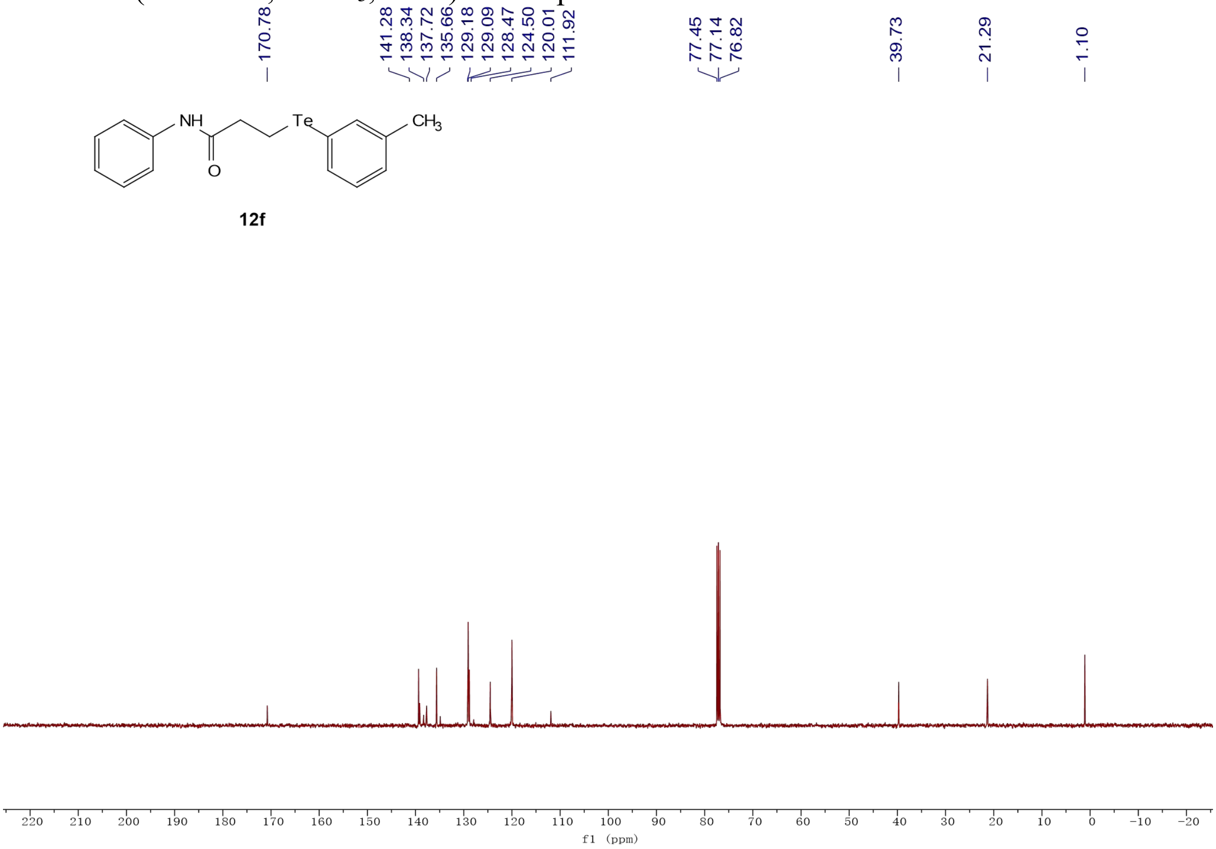

Supplementary Fig. 195. NMR of compound **12f**

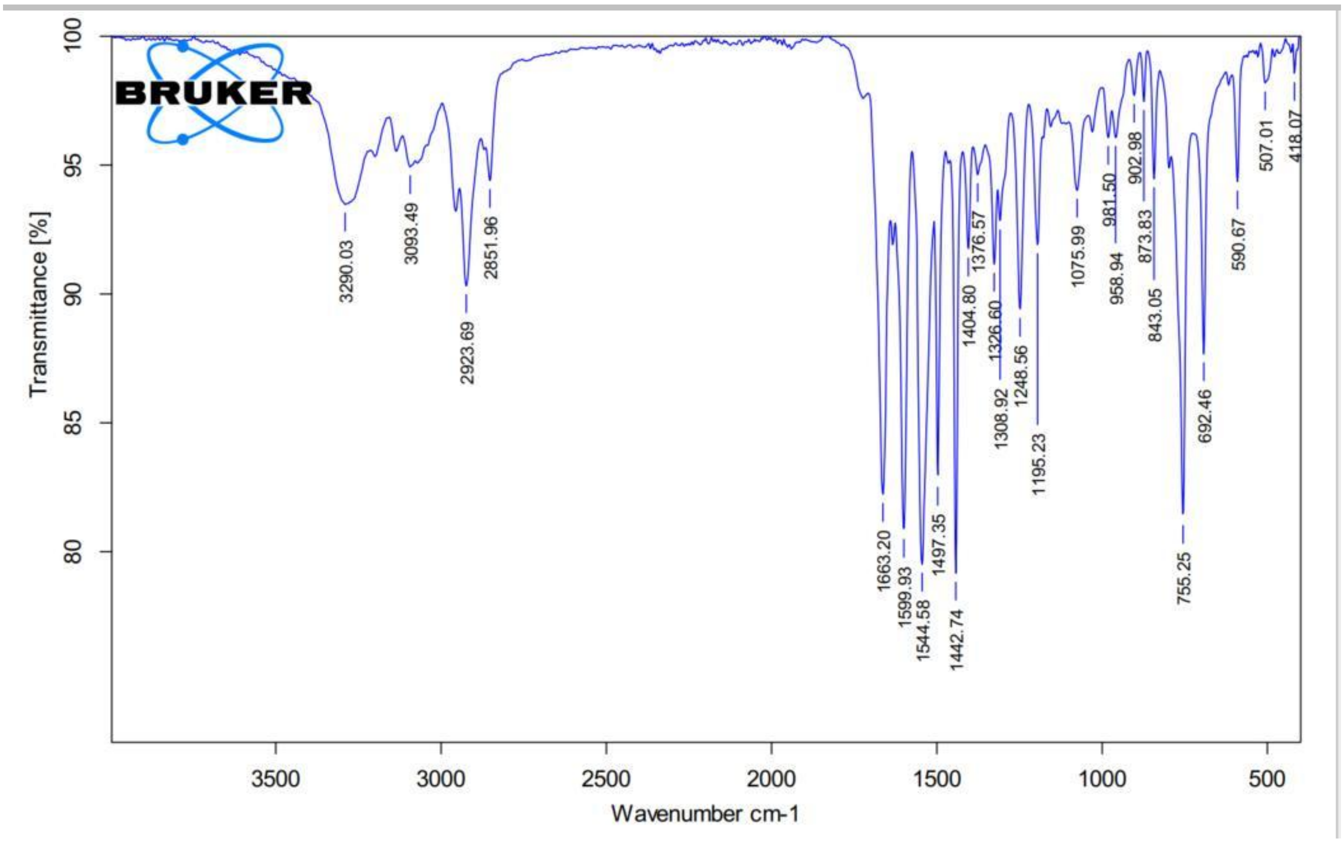

Supplementary Fig. 196. IR of compound **12f**

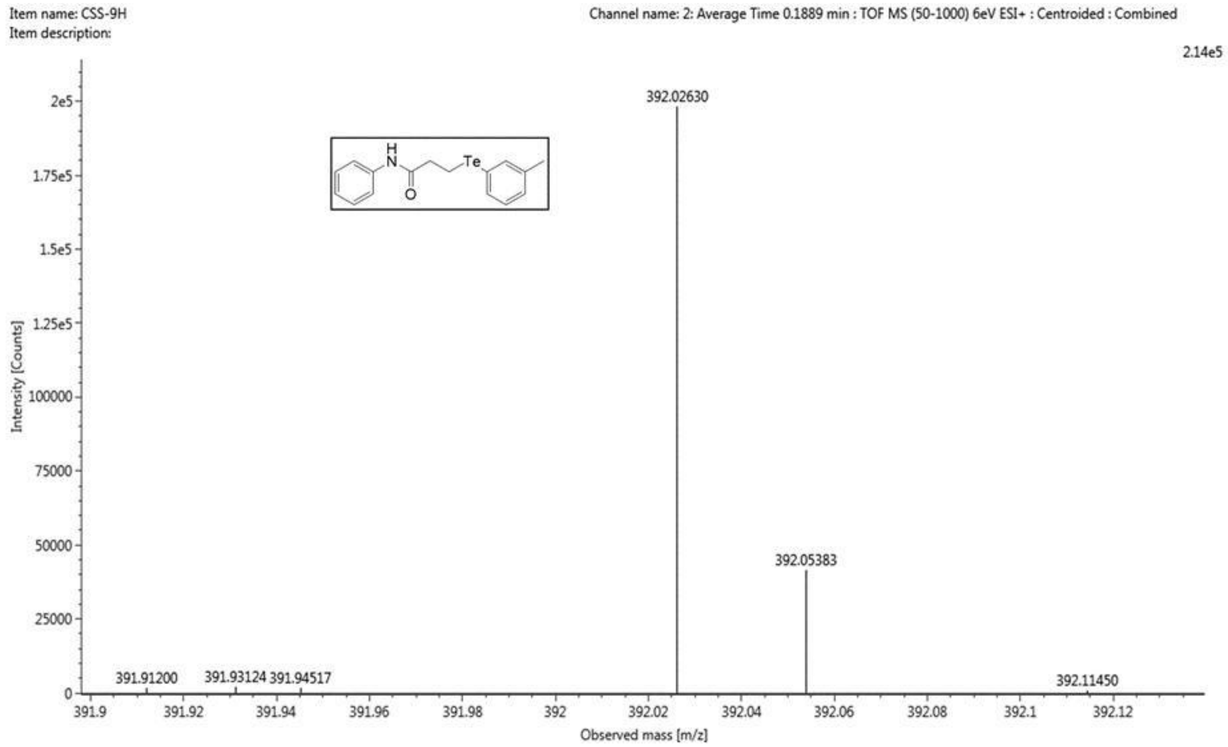

Supplementary Fig. 197. HR-MS of compound **12f**

^{1}H NMR (400 MHz, $CDCl_3$, 25°C) of compound **12g**

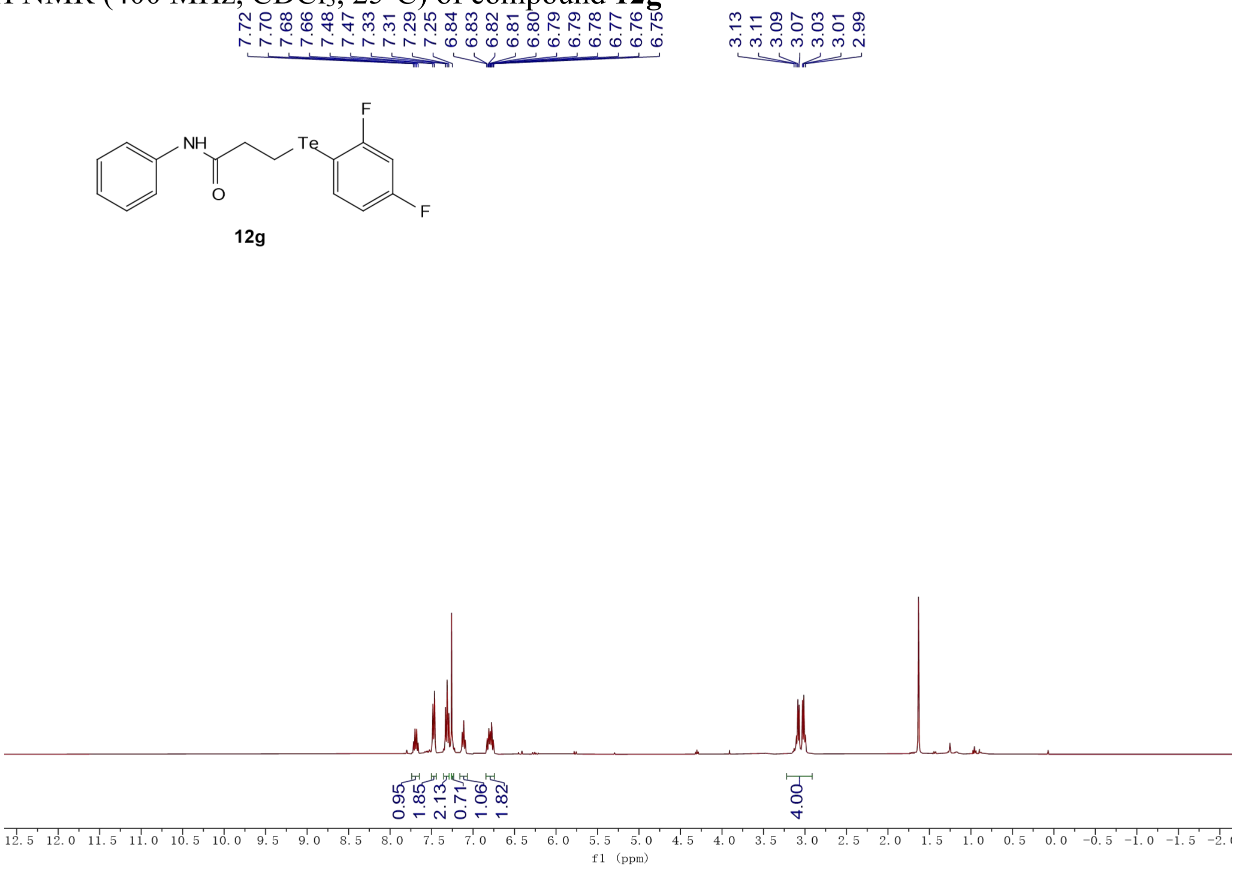

^{13}C NMR (101 MHz, $CDCl_3$, 25°C) of compound **12g**

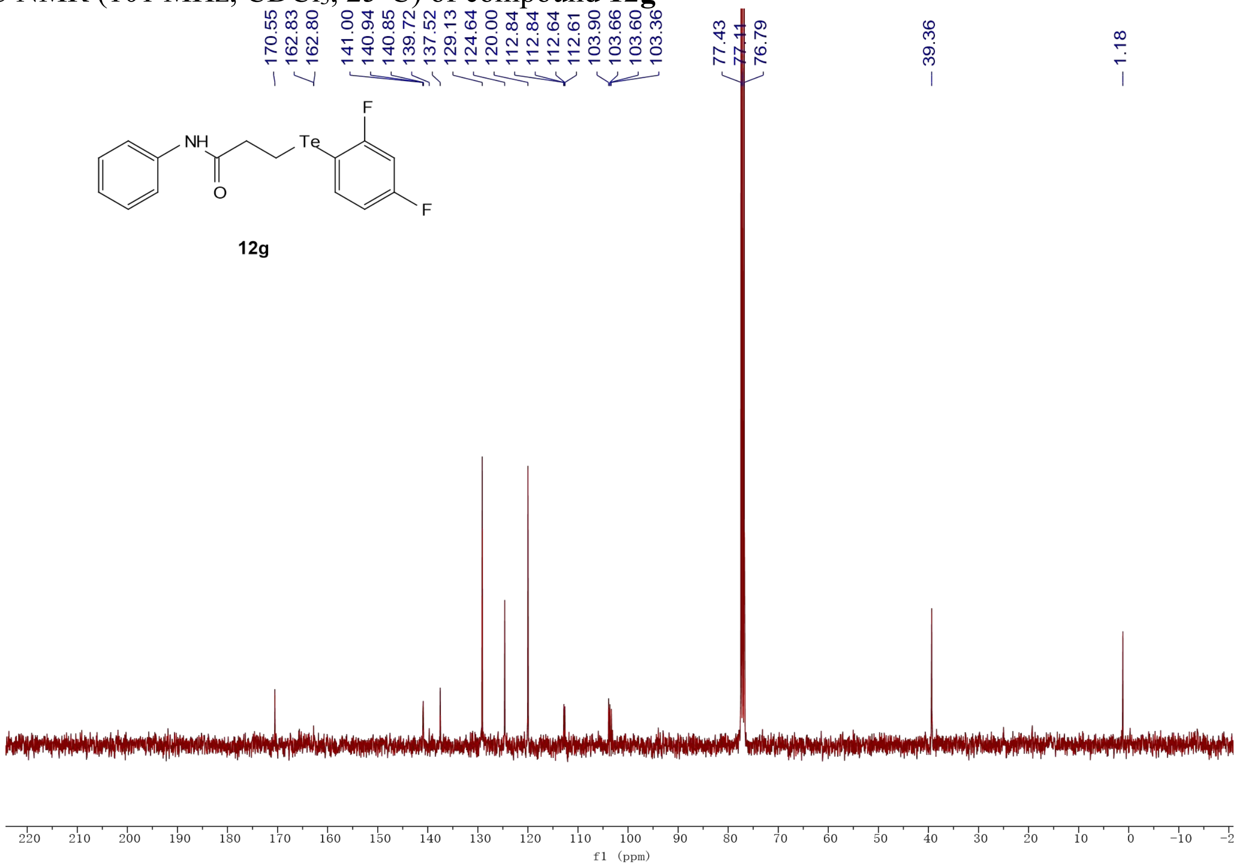

^{19}F NMR (376 MHz, $CDCl_3$, 25°C) of compound **12g**

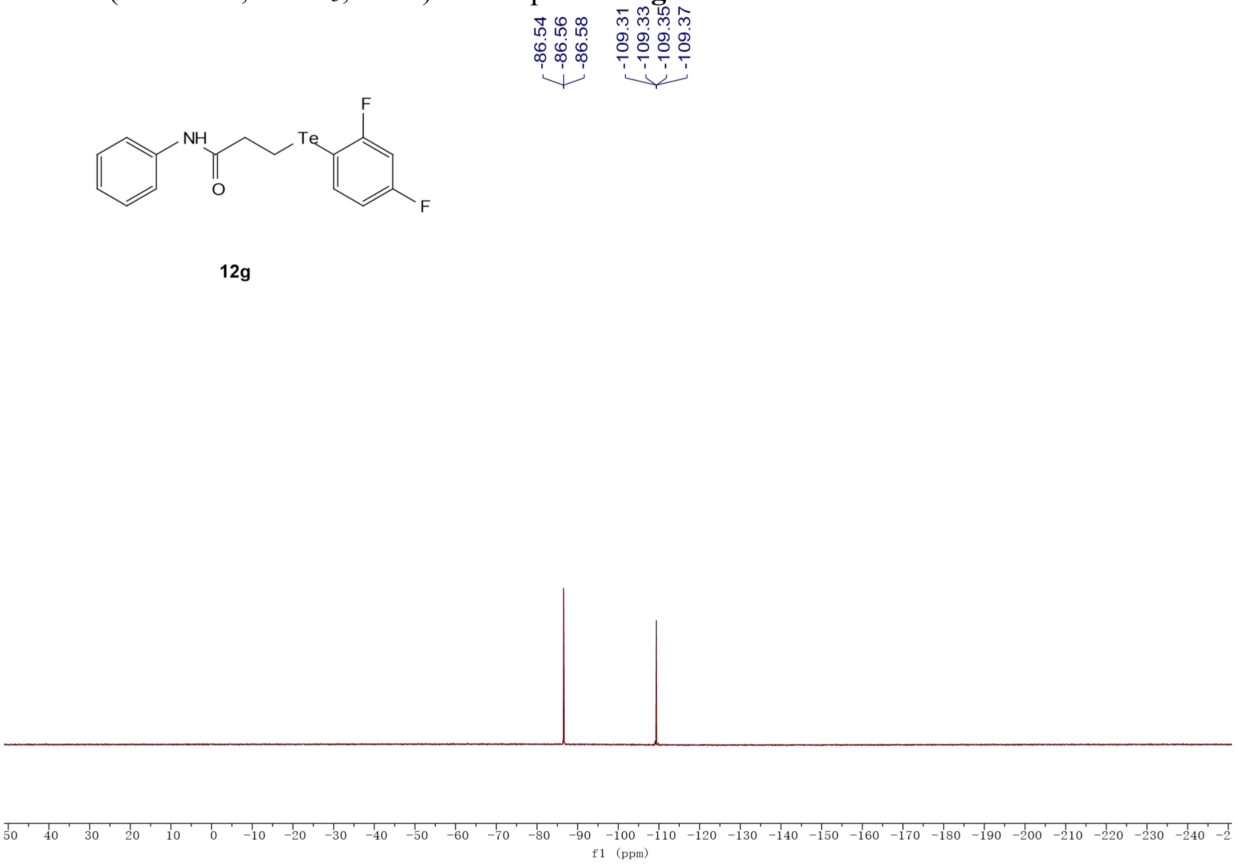

Supplementary Fig. 198. NMR of compound **12g**

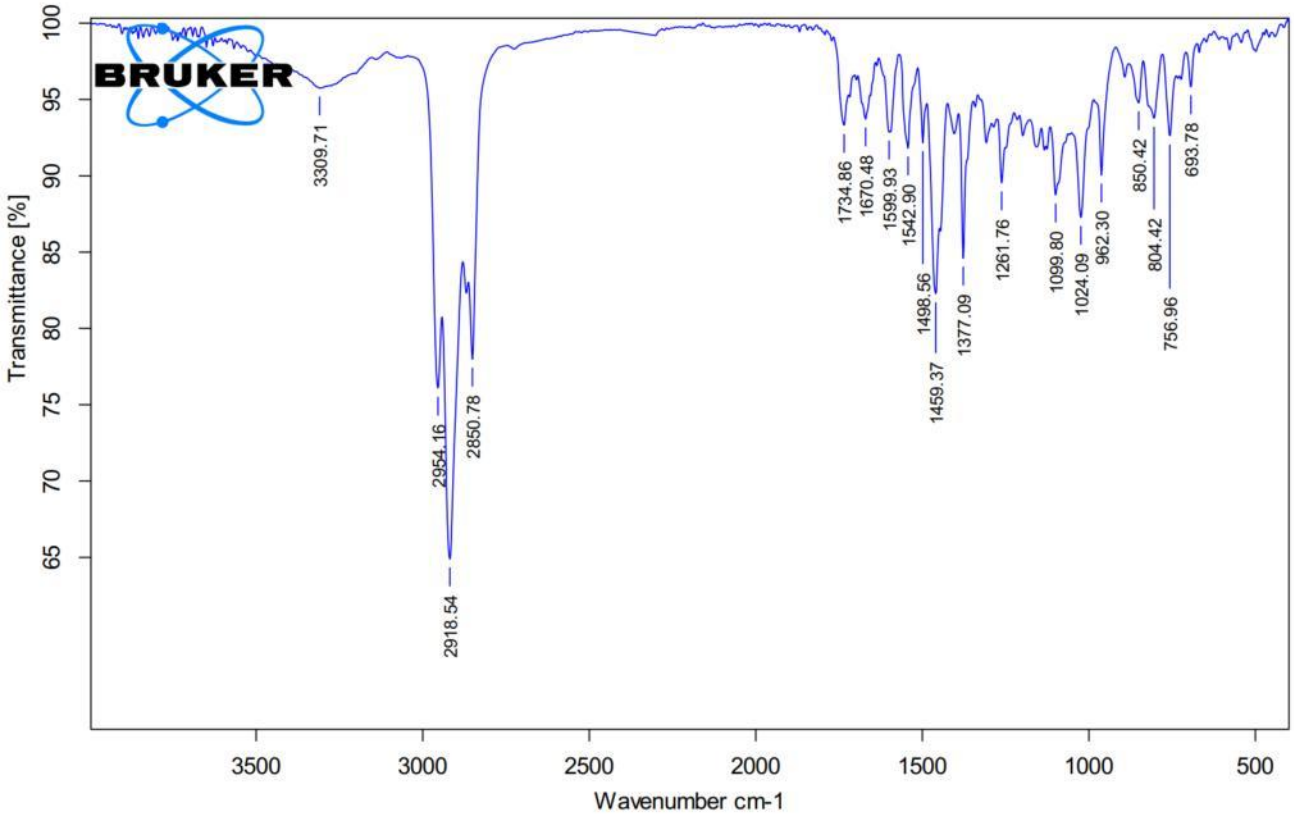

Supplementary Fig. 199. IR of compound **12g**

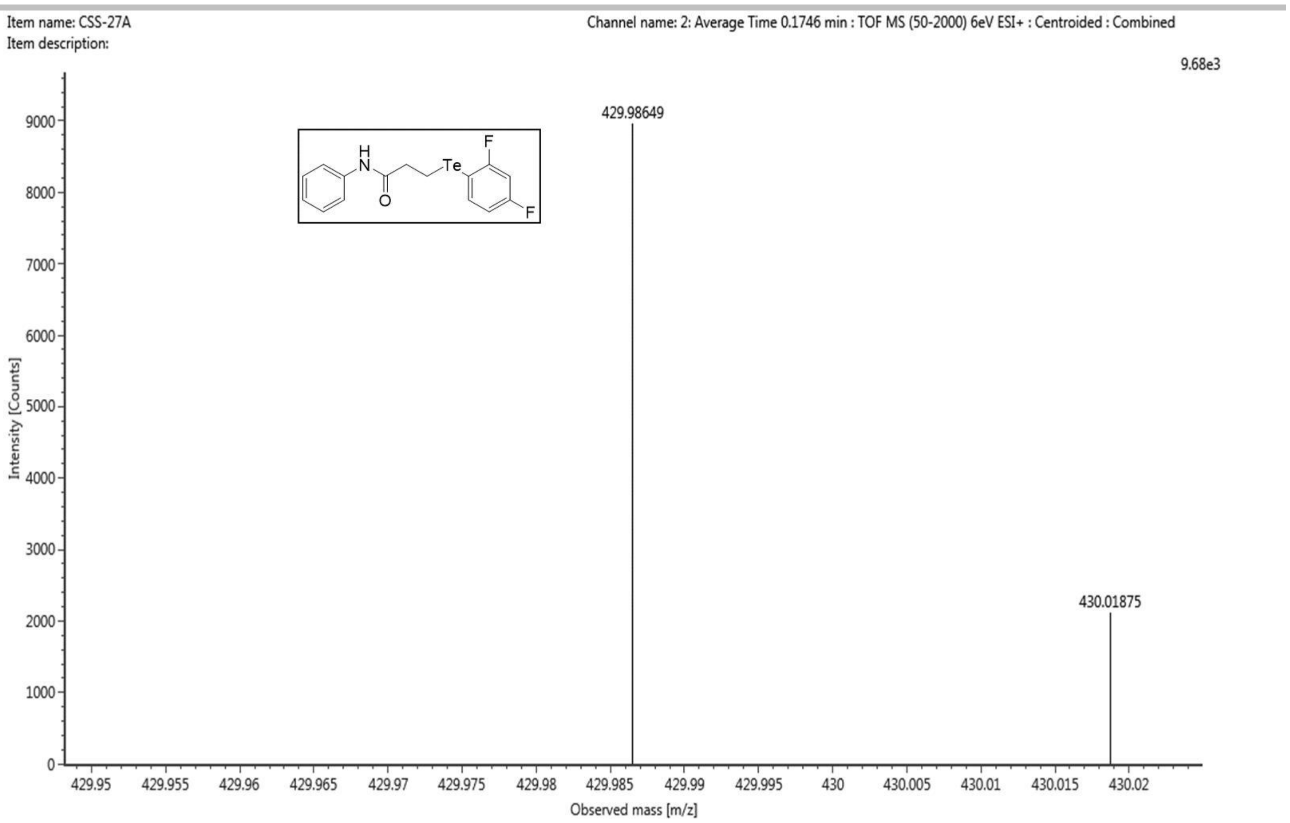

Supplementary Fig. 200. HR-MS of compound **12g**

^{1}H NMR (400 MHz, $CDCl_3$, 25°C) of compound **12h**

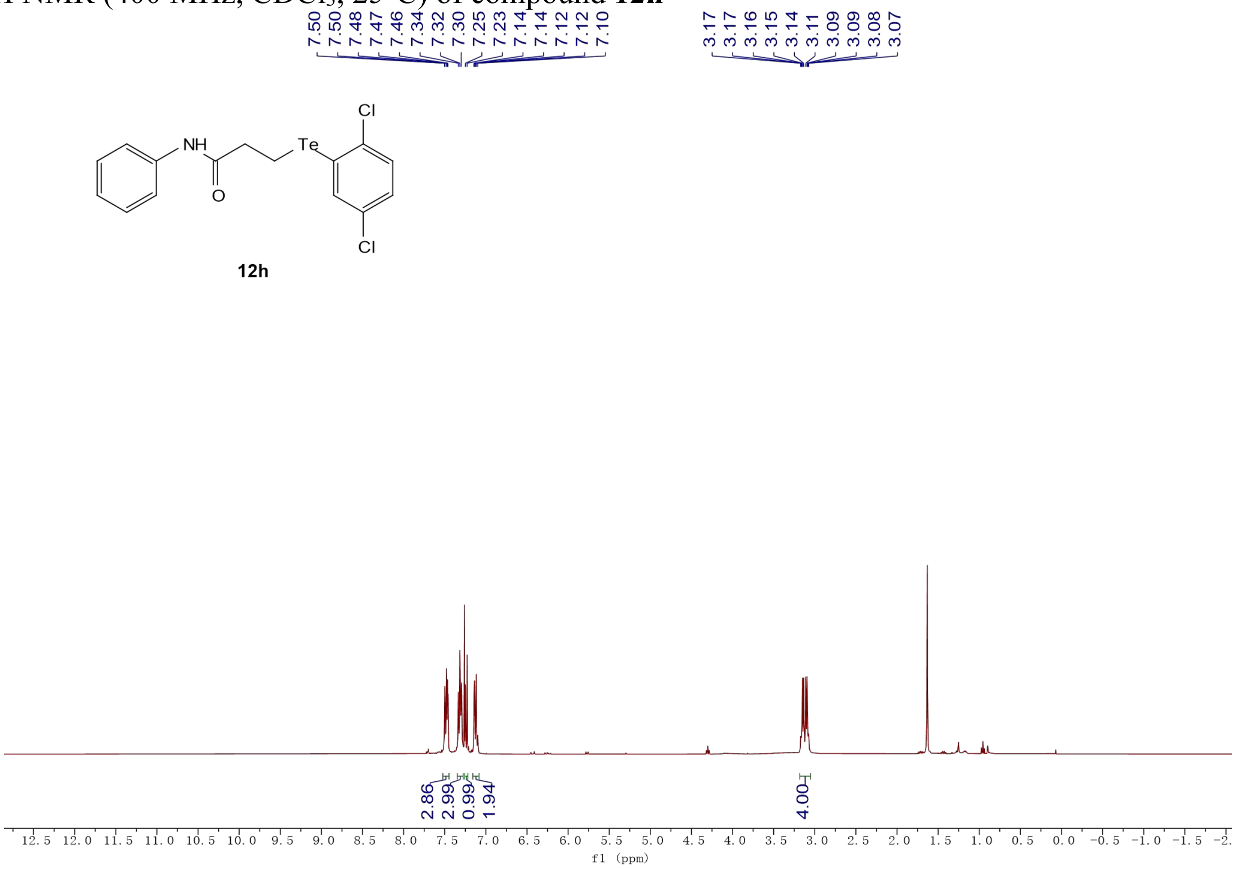

^{13}C NMR (101 MHz, $CDCl_3$, 25°C) of compound **12h**

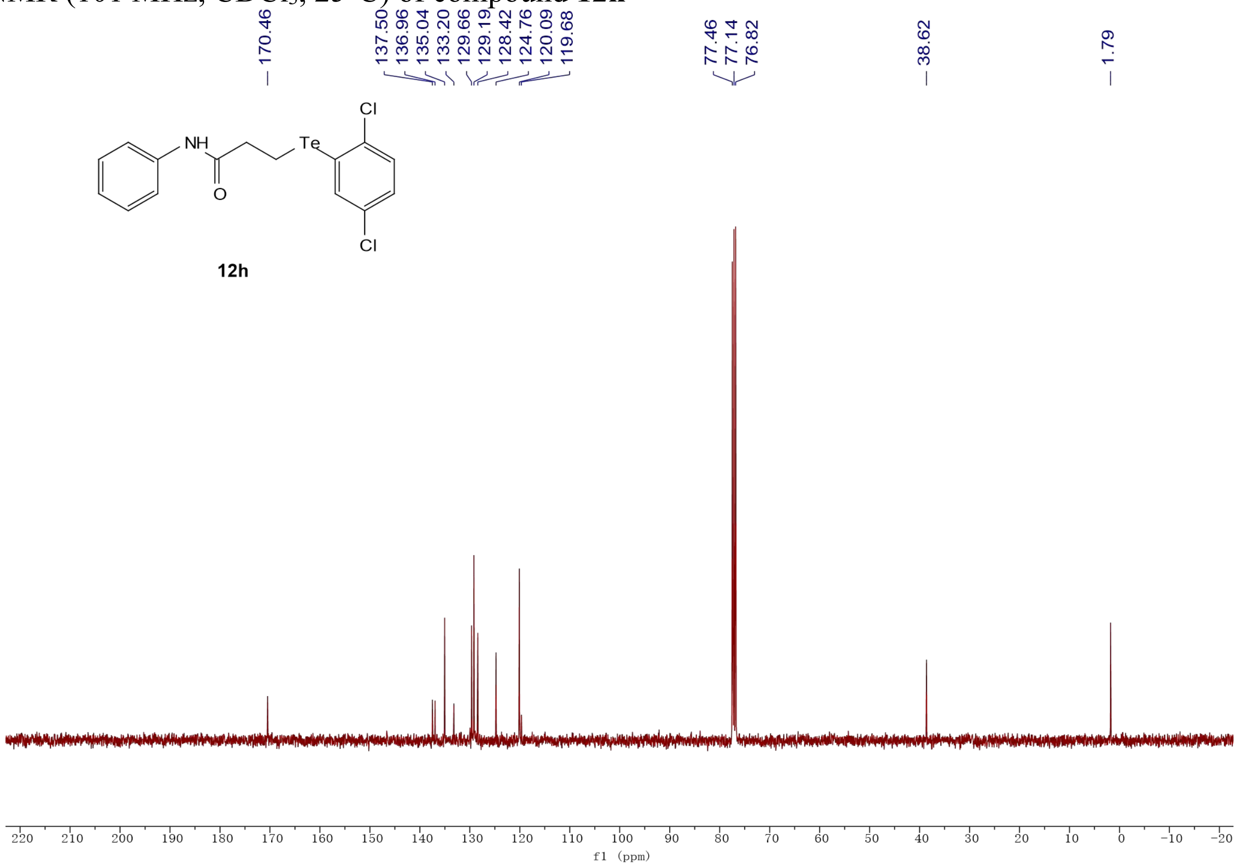

Supplementary Fig. 201. NMR of compound **12h**

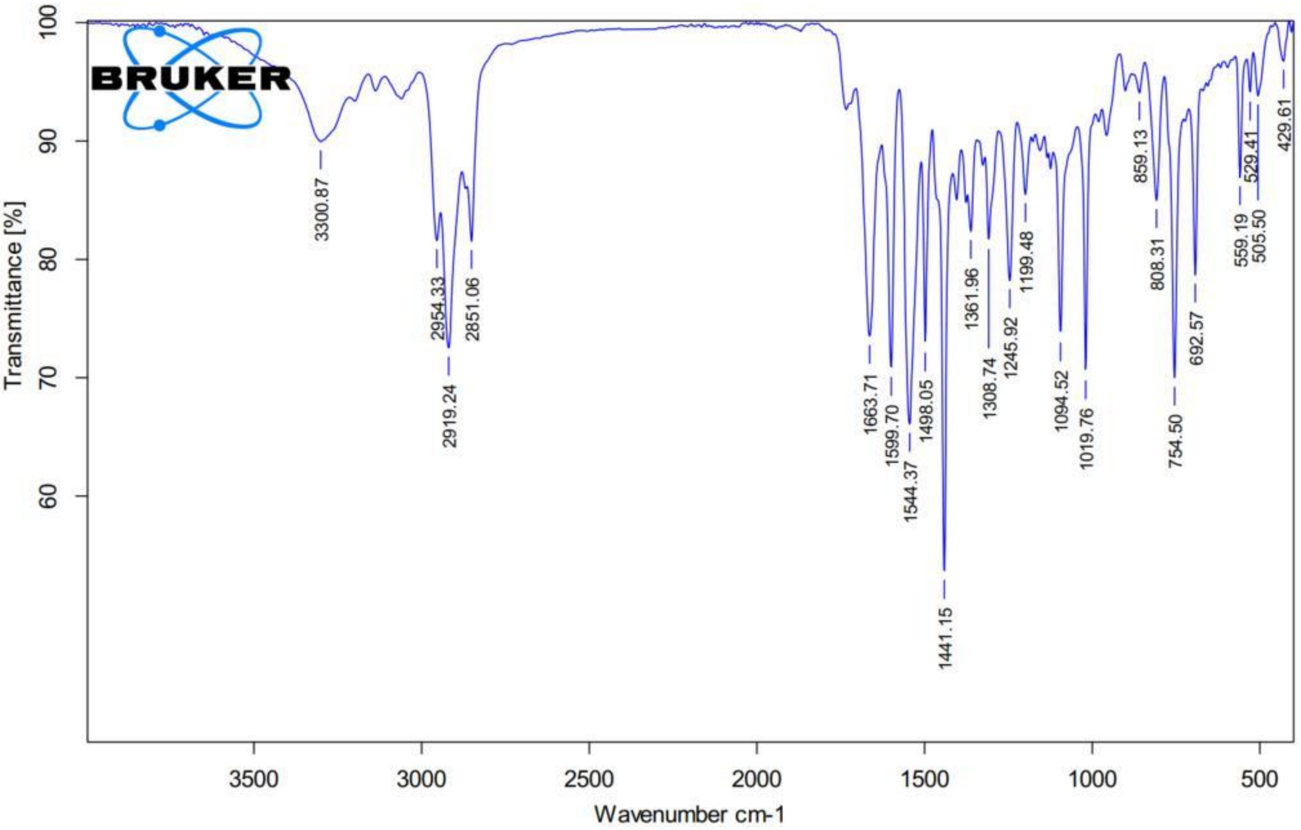

Supplementary Fig. 202. IR of compound **12h**

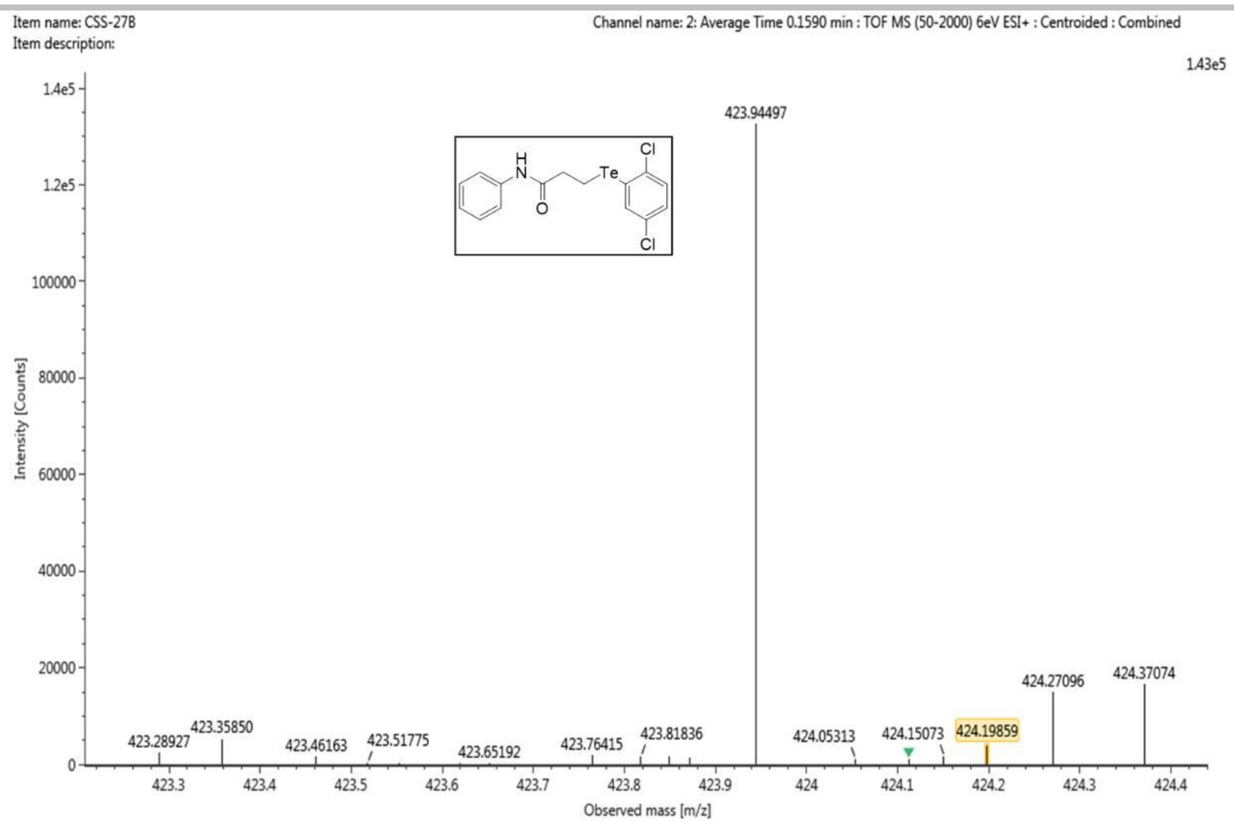

Supplementary Fig. 203. HR-MS of compound **12h**

^{1}H NMR (400 MHz, $CDCl_3$, 25°C) of compound **12i**

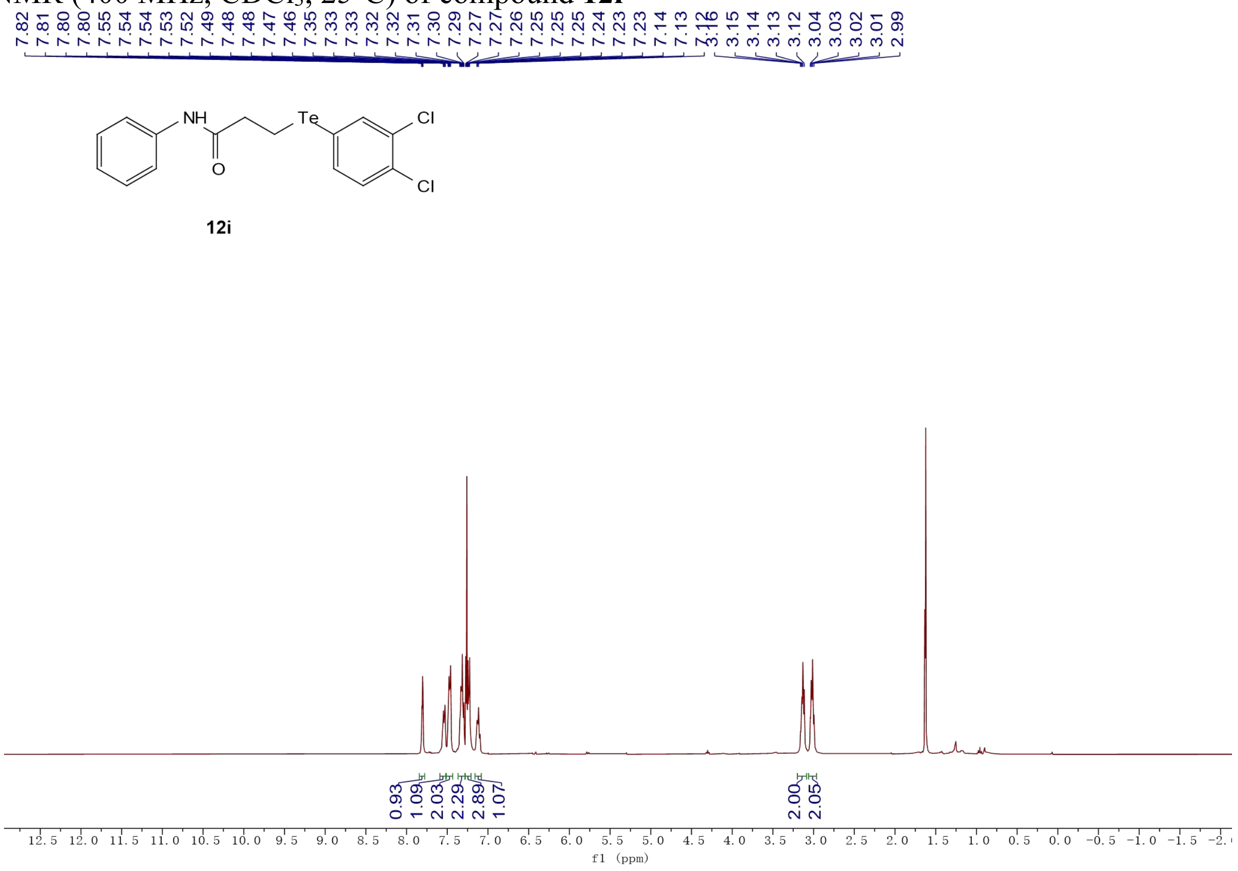

^{13}C NMR (101 MHz, $CDCl_3$, 25°C) of compound **12i**

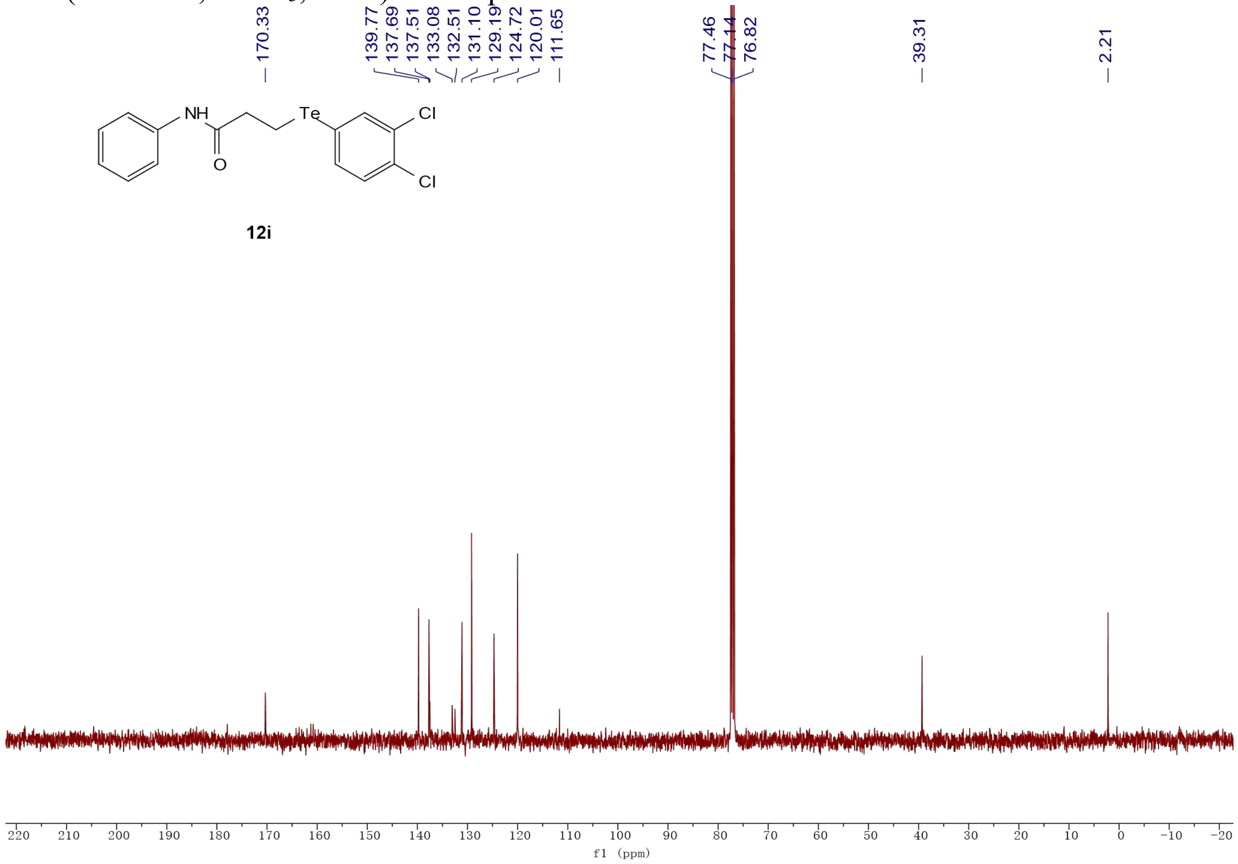

Supplementary Fig. 204. NMR of compound **12i**

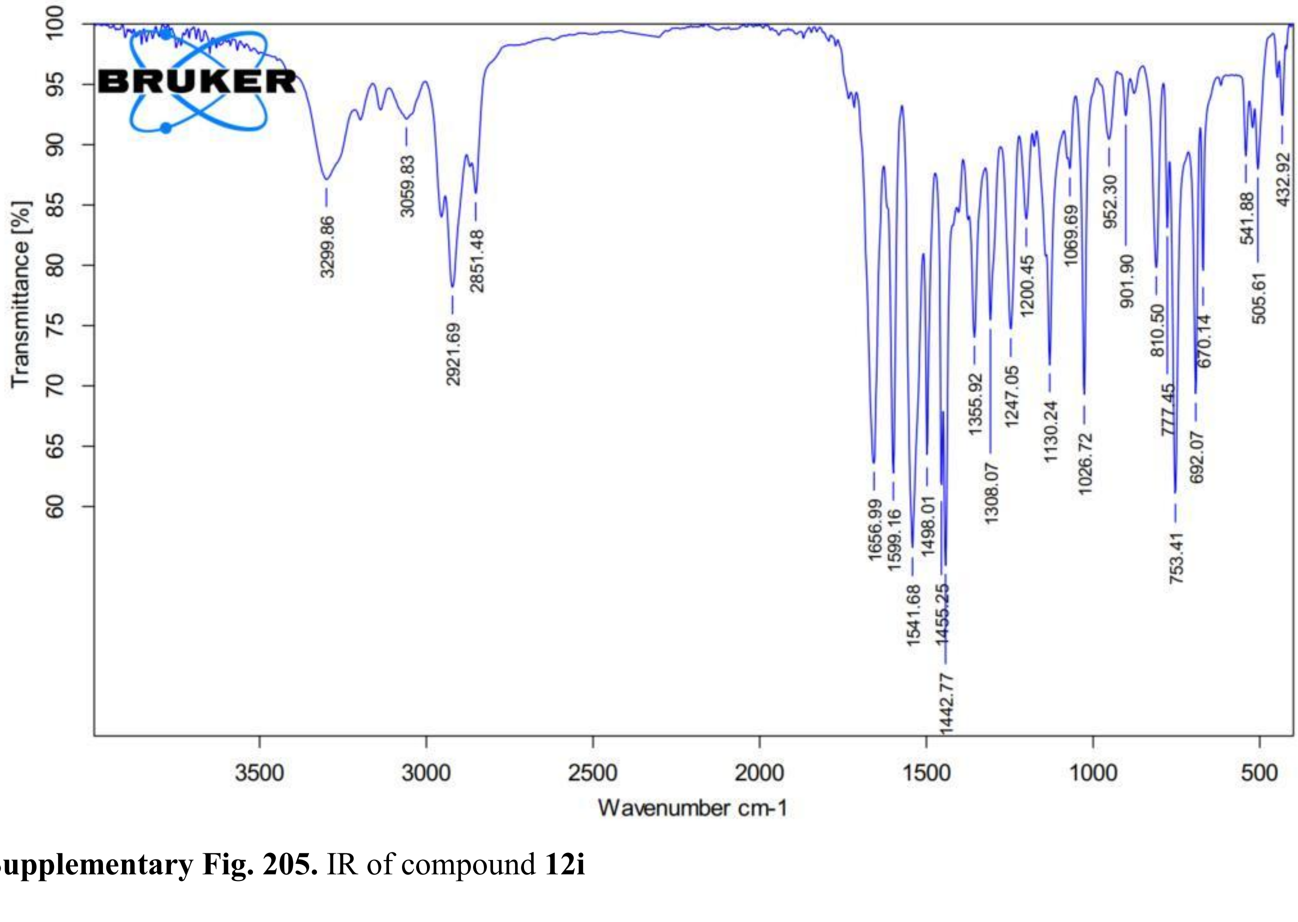

Supplementary Fig. 205. IR of compound **12i**

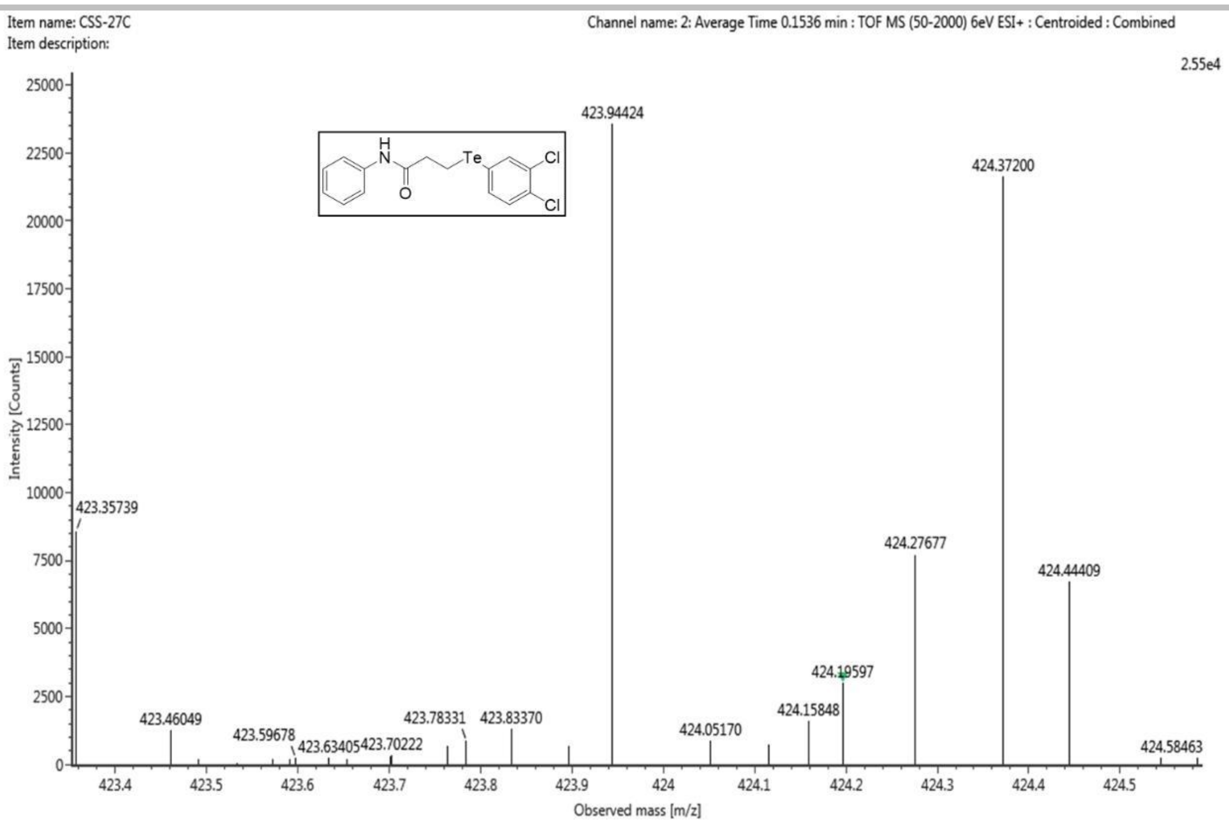

Supplementary Fig. 206. HR-MS of compound **12i**

^{1}H NMR (400 MHz, $CDCl_3$, 25°C) of compound **12j**

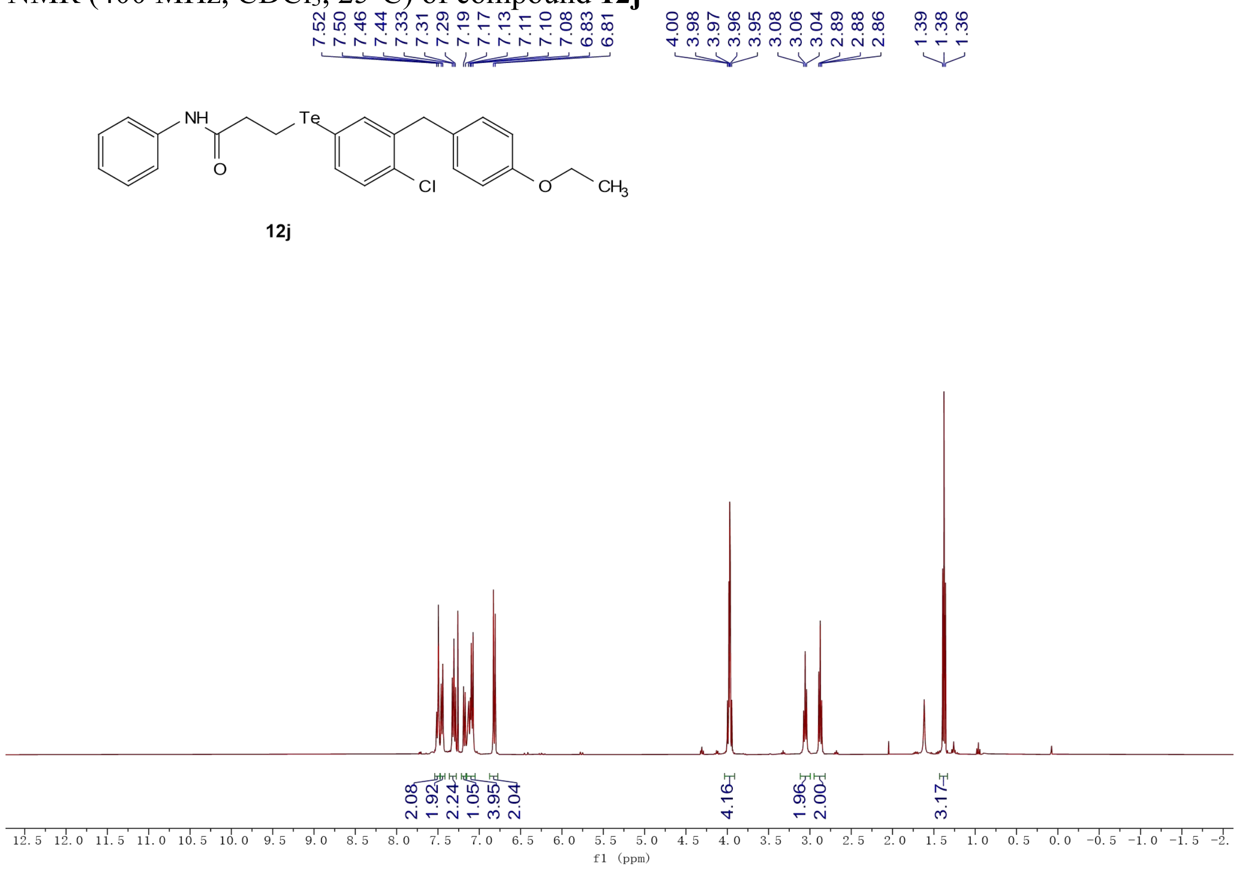

^{13}C NMR (101 MHz, $CDCl_3$, 25°C) of compound **12j**

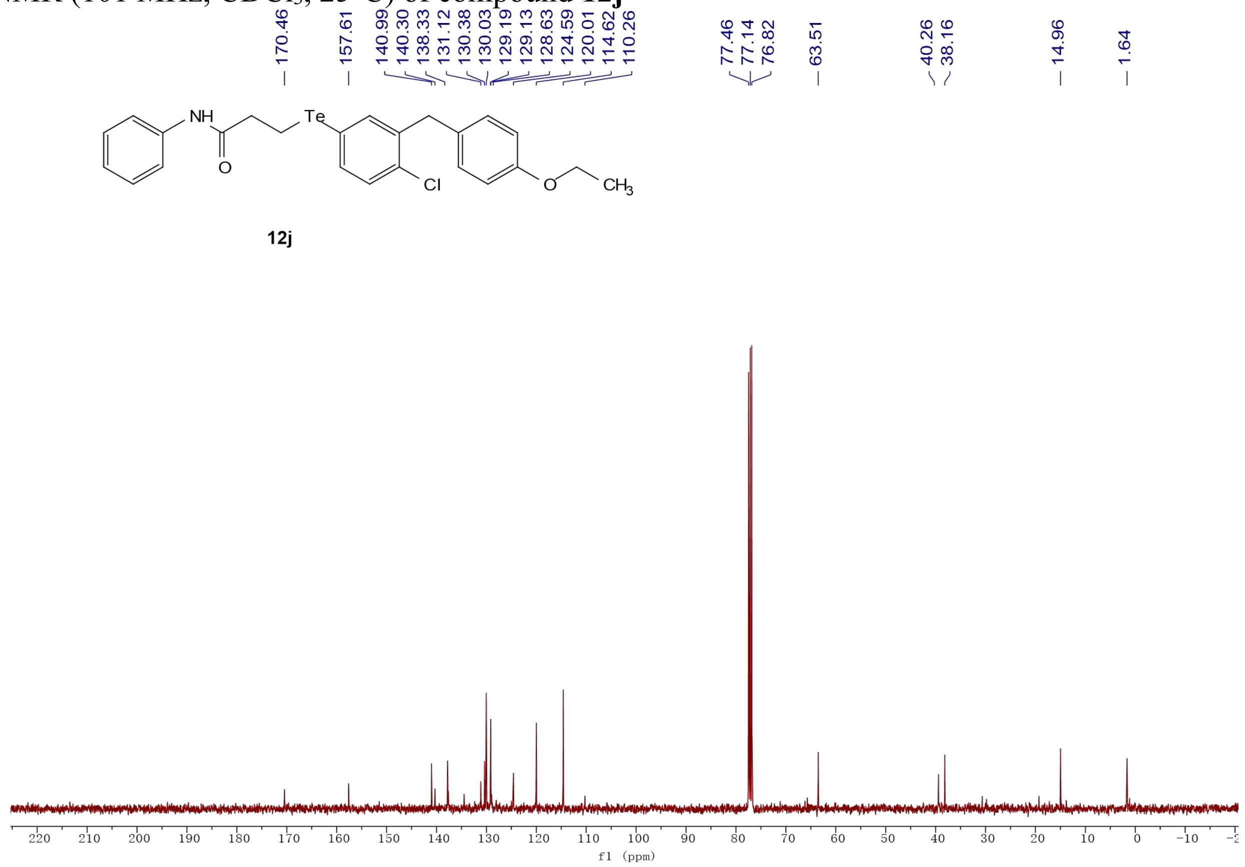

Supplementary Fig. 207. NMR of compound **12j**

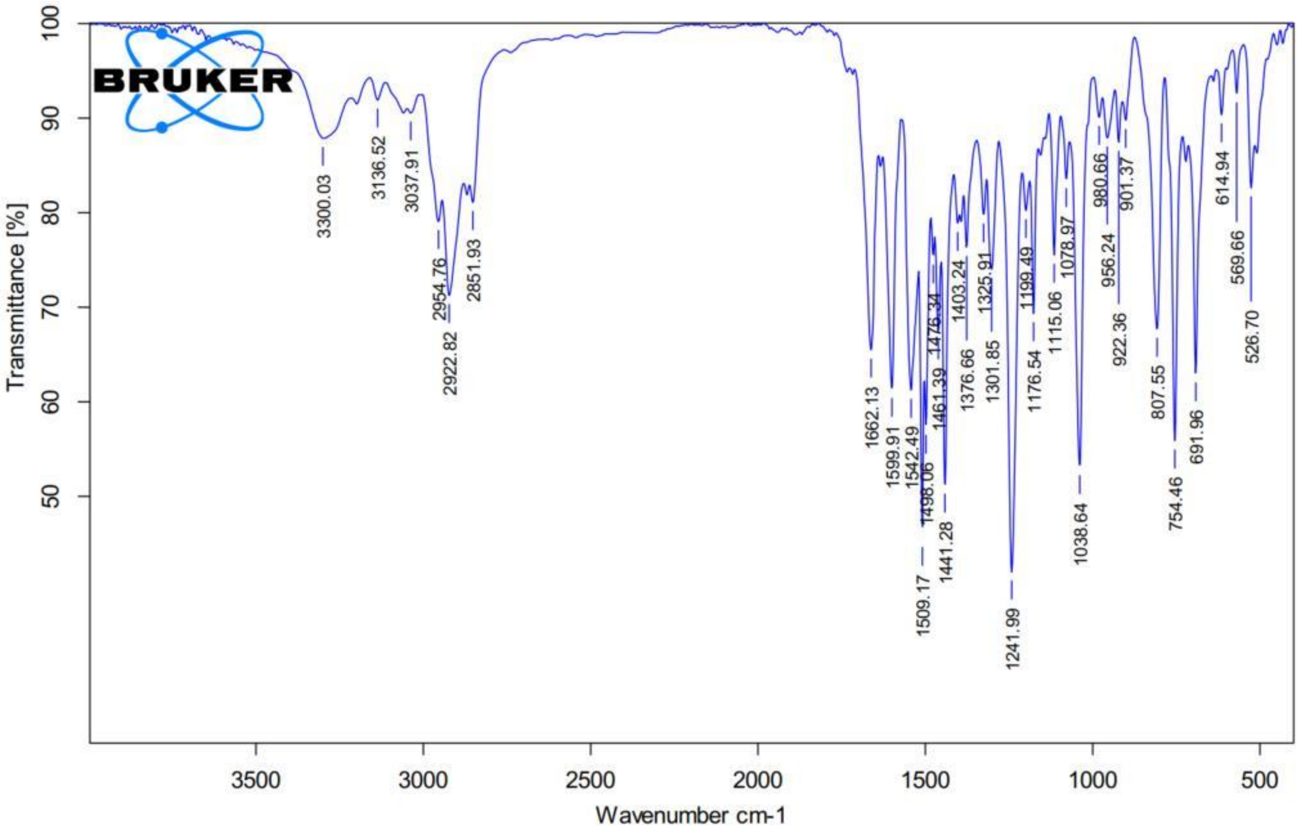

Supplementary Fig. 208. IR of compound **12j**

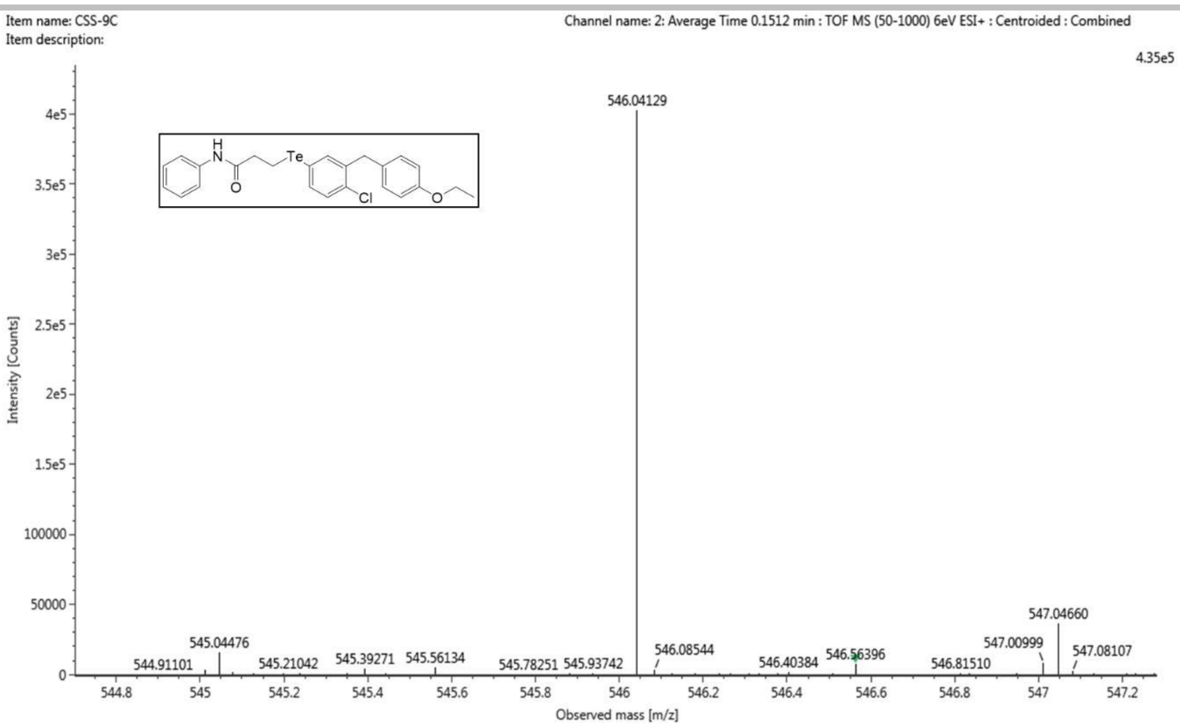

Supplementary Fig. 209. HR-MS of compound **12j**

^{1}H NMR (400 MHz, $CDCl_3$, 25°C) of compound **12k**

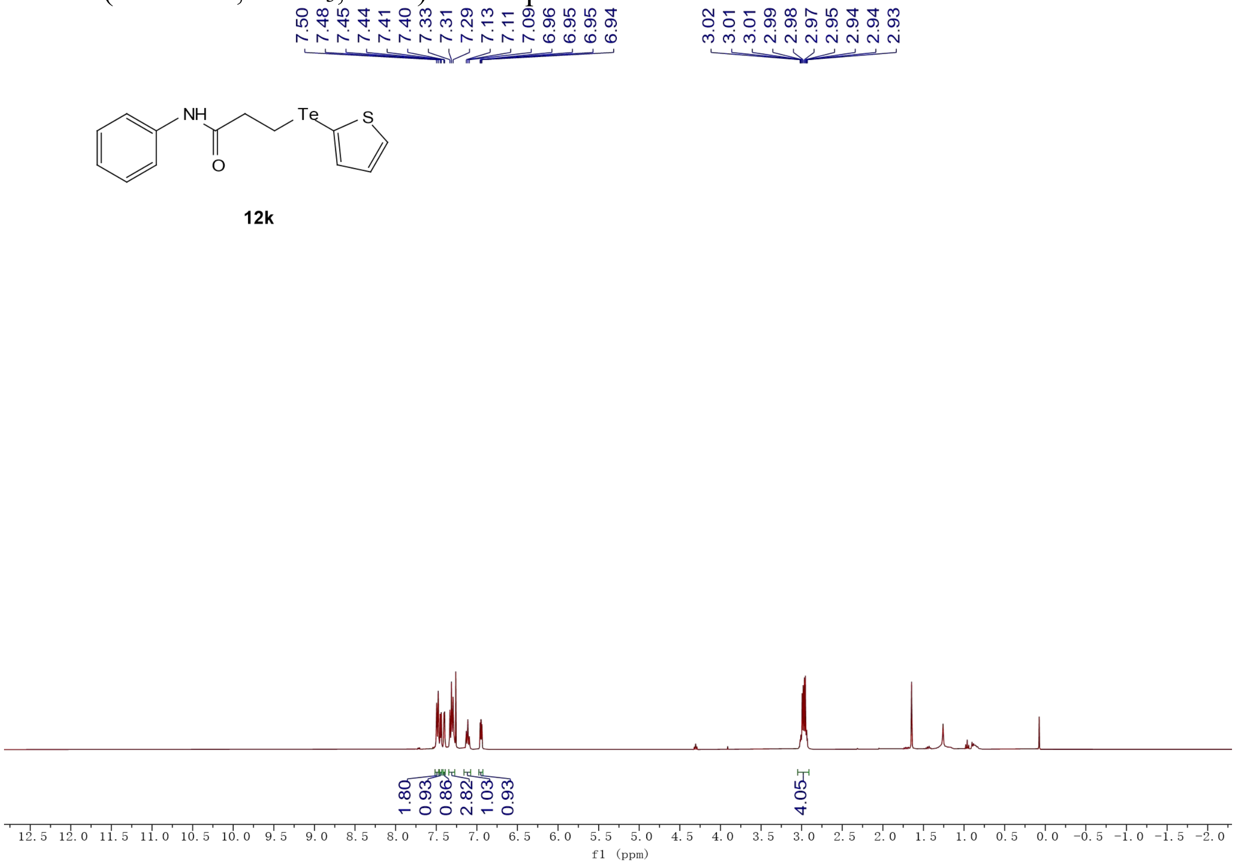

^{13}C NMR (101 MHz, $CDCl_3$, 25°C) of compound **12k**

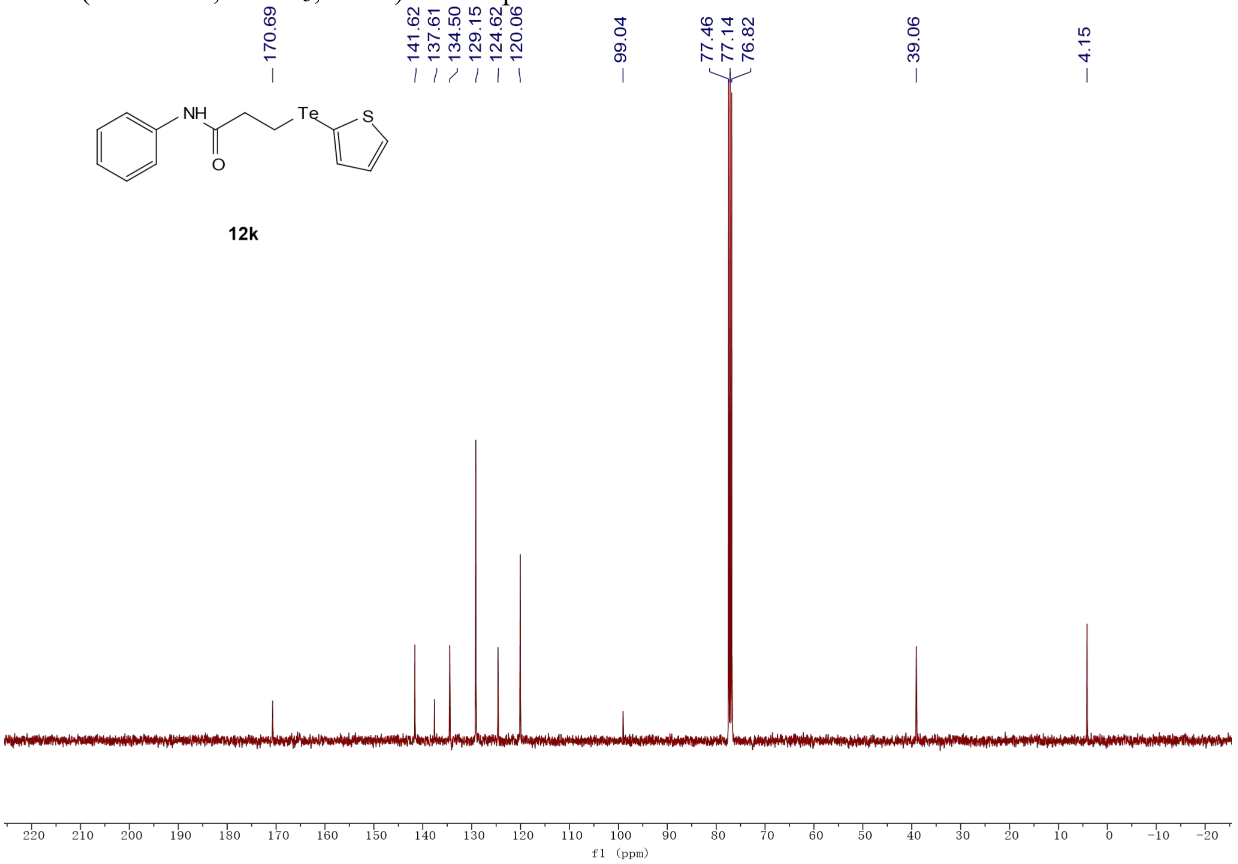

Supplementary Fig. 210. NMR of compound **12k**

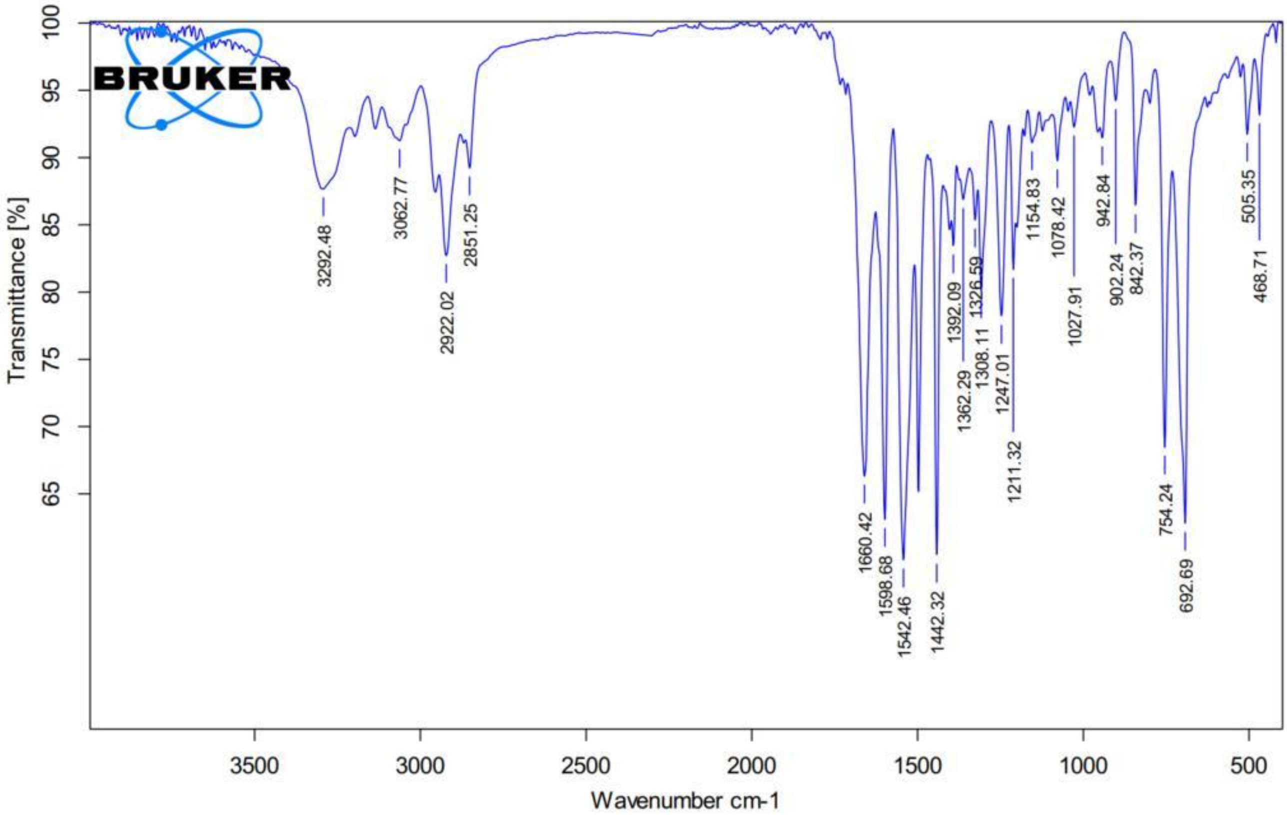

Supplementary Fig. 211. IR of compound **12k**

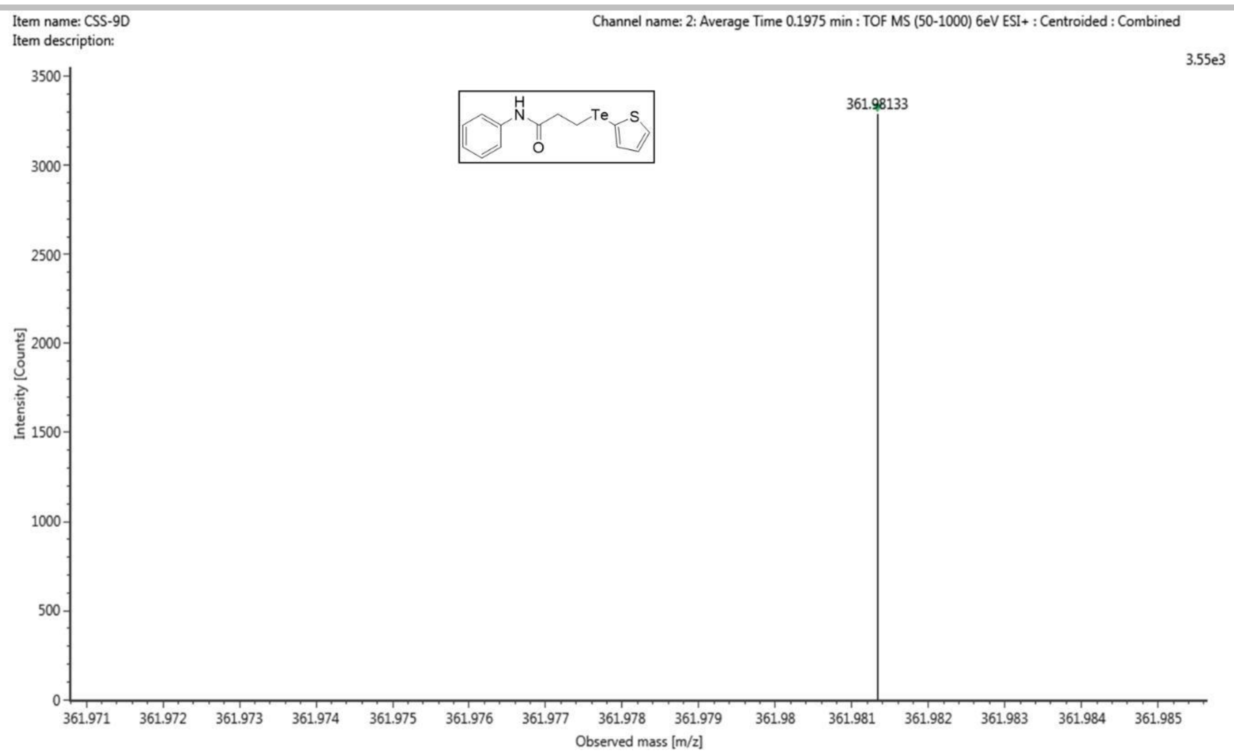

Supplementary Fig. 212. HR-MS of compound **12k**

^{1}H NMR (400 MHz, $CDCl_3$, 25°C) of compound **12l**

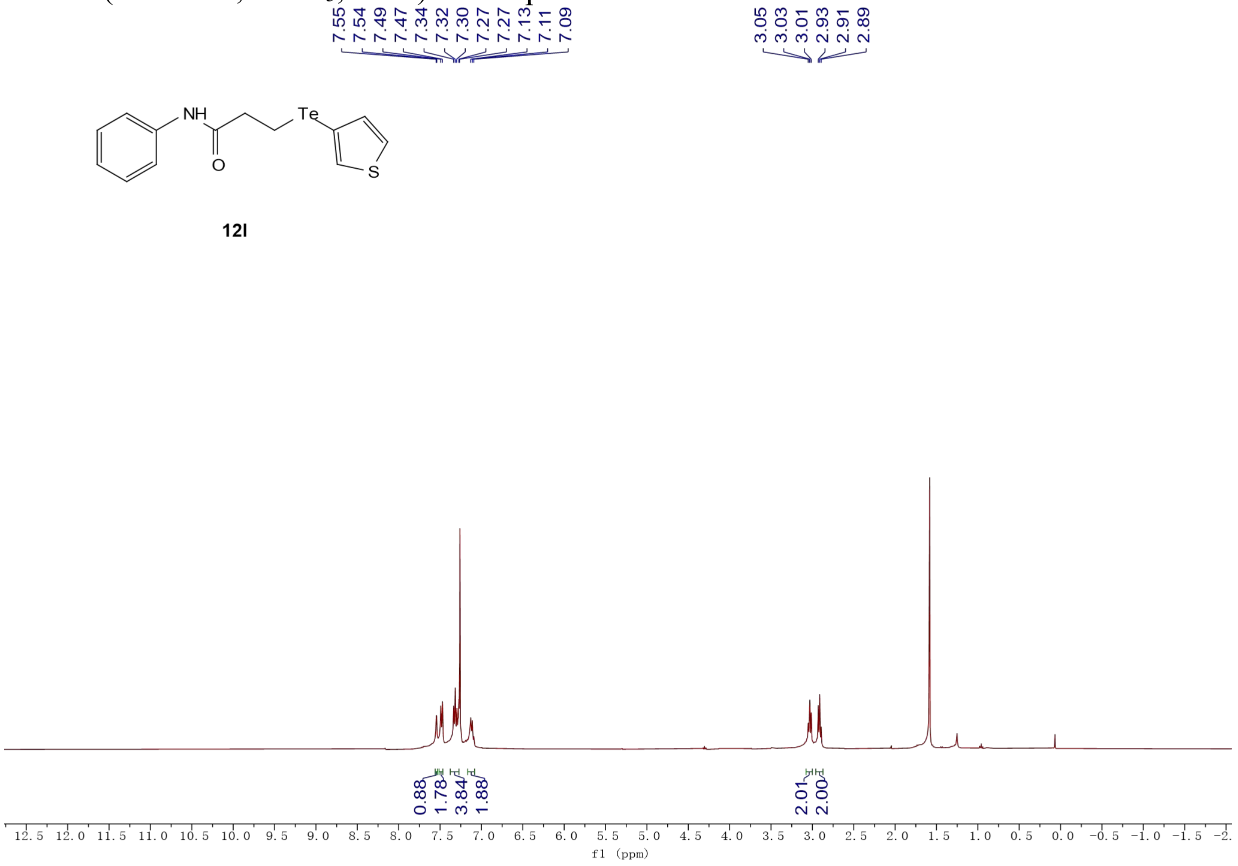

^{13}C NMR (101 MHz, $CDCl_3$, 25°C) of compound **12l**

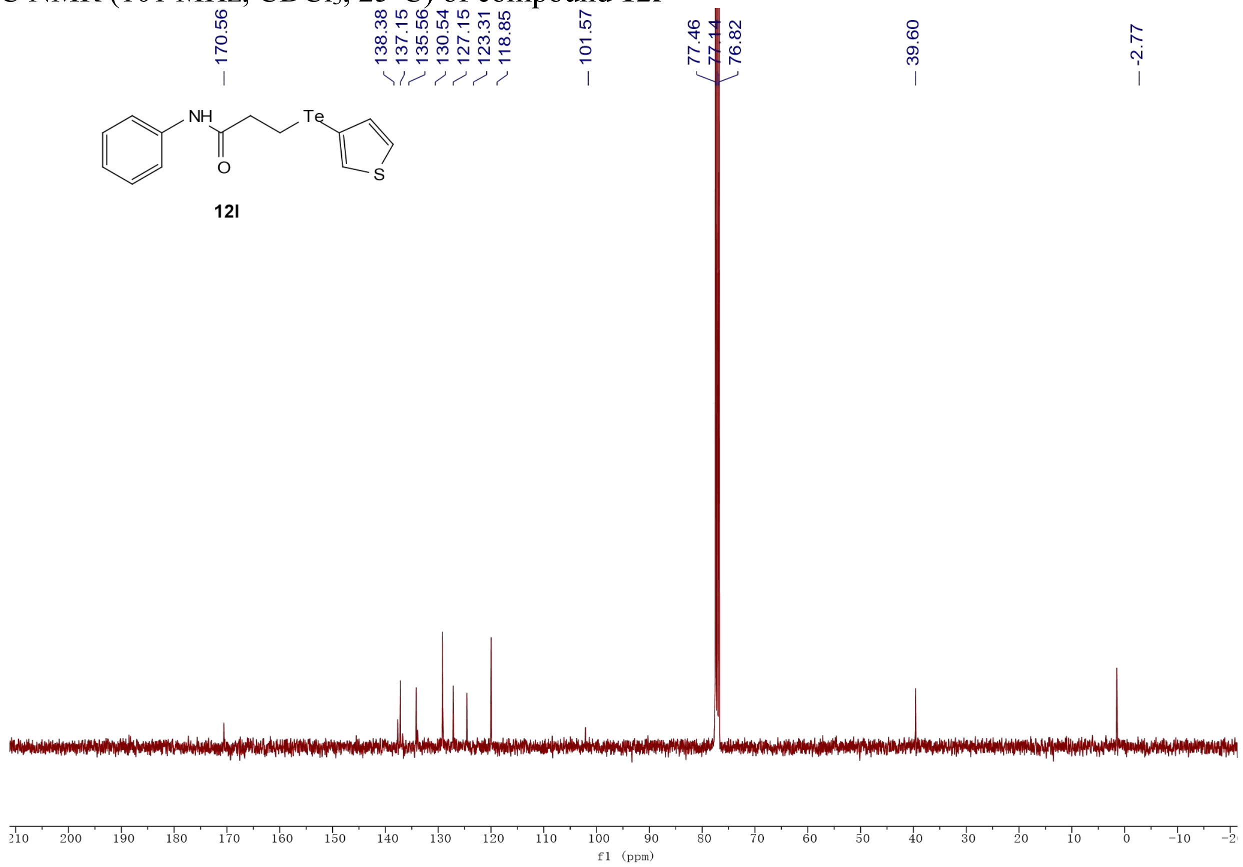

Supplementary Fig. 213. NMR of compound **12l**

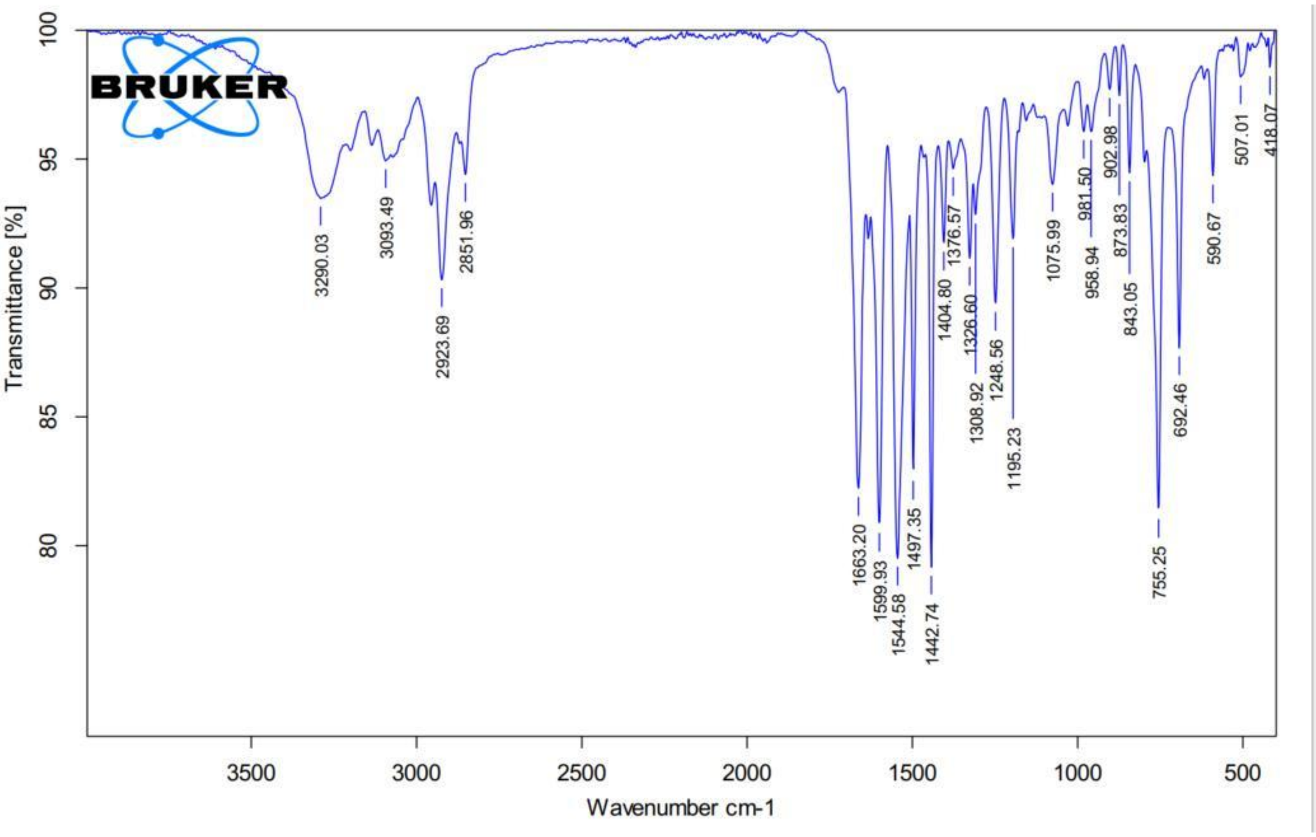

Supplementary Fig. 214. IR of compound **12l**

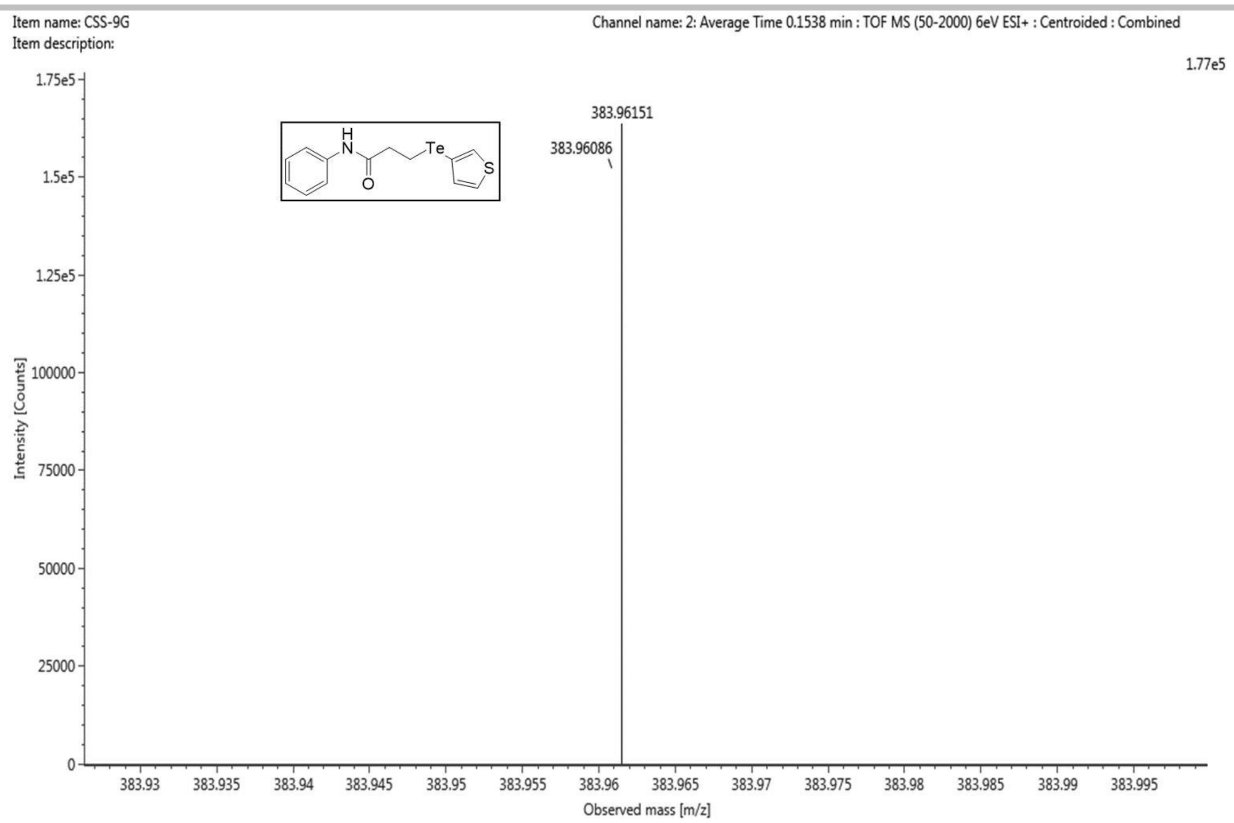

Supplementary Fig. 215. HR-MS of compound **12l**

^{1}H NMR (400 MHz, $CDCl_3$, 25°C) of compound **12m**

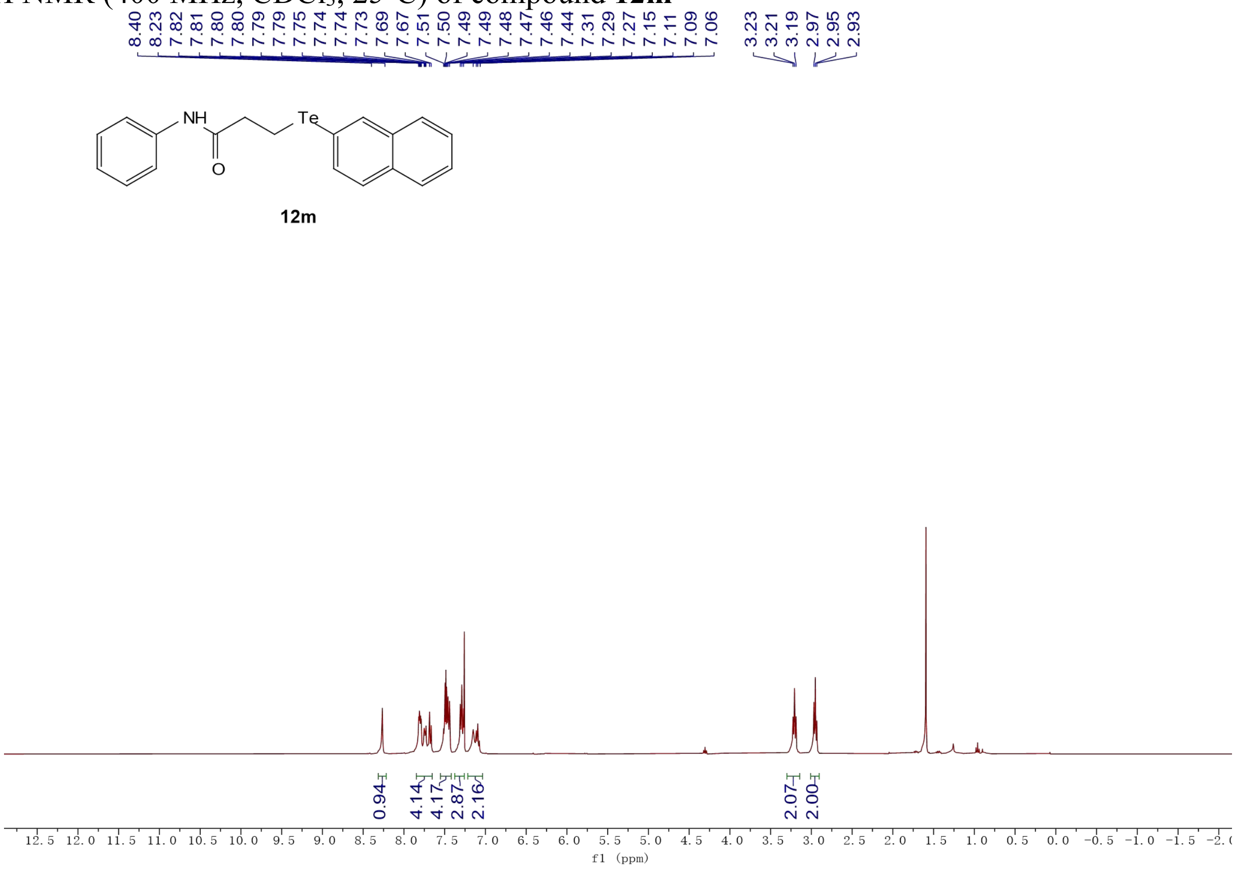

^{13}C NMR (101 MHz, $CDCl_3$, 25°C) of compound **12m**

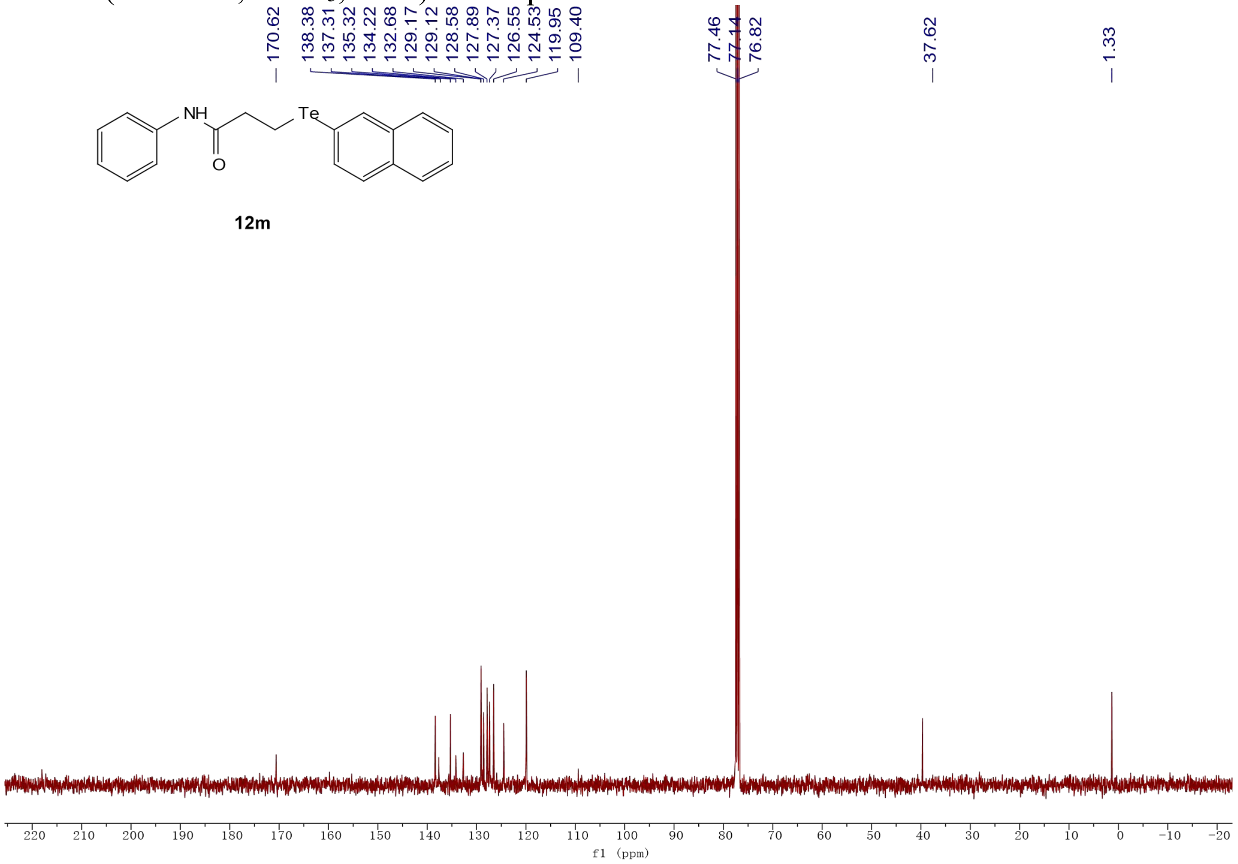

Supplementary Fig. 216. NMR of compound **12m**

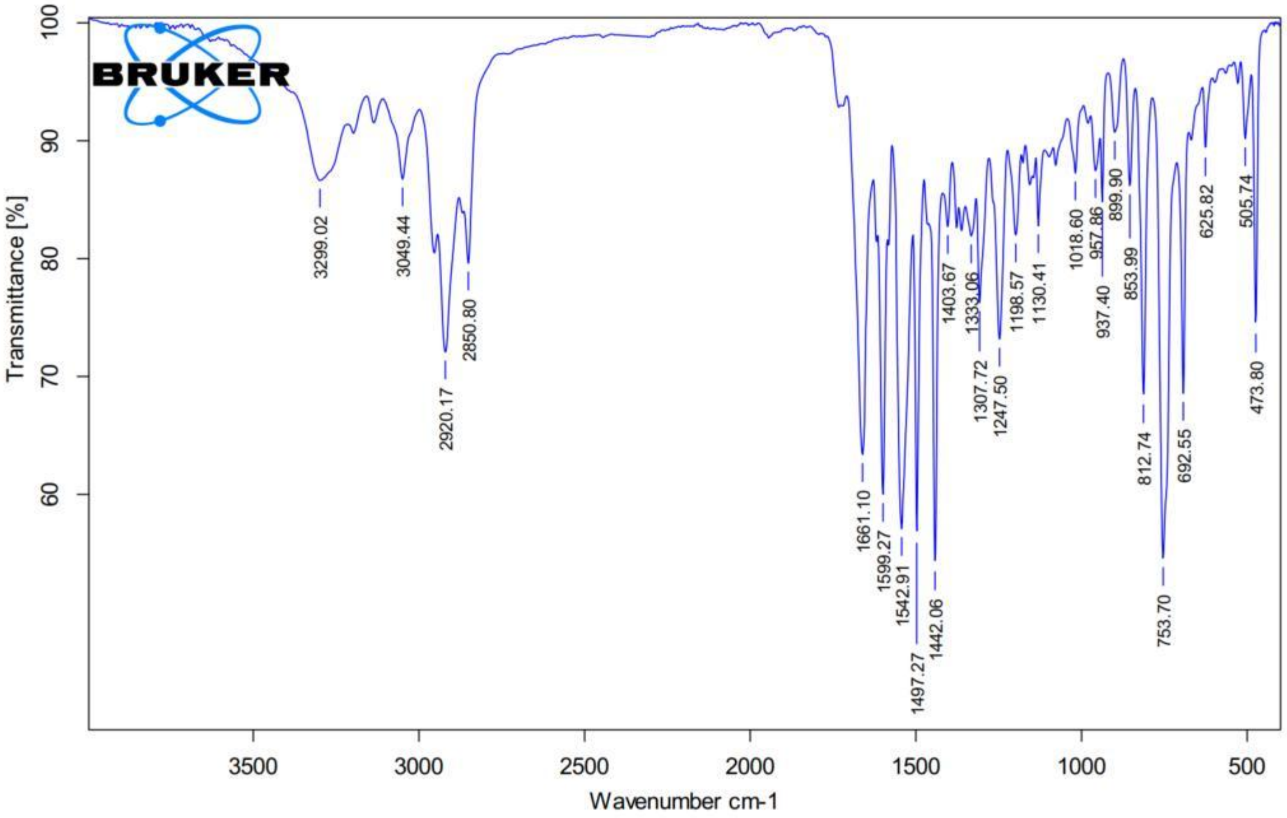

Supplementary Fig. 217. IR of compound **12m**

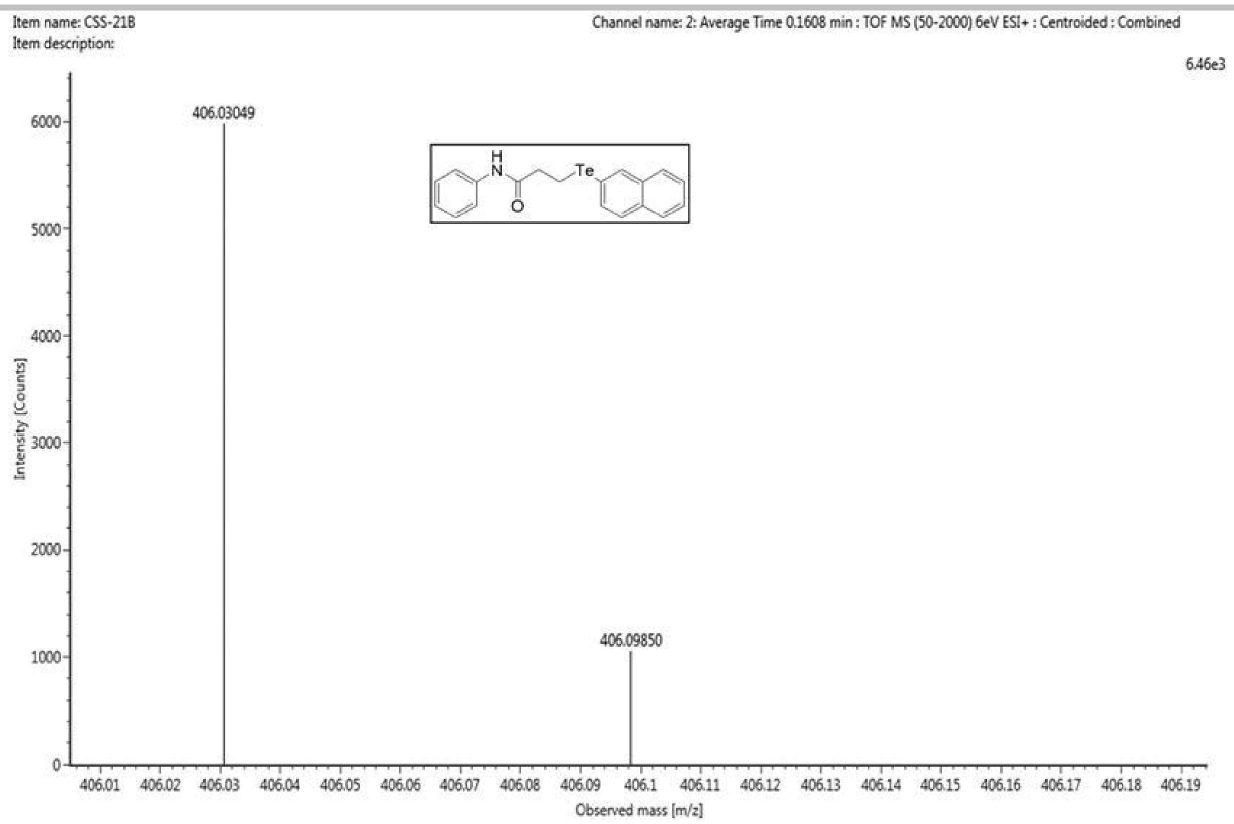

Supplementary Fig. 218. HR-MS of compound **12m**

^{1}H NMR (400 MHz, $CDCl_3$, 25°C) of compound **12n**

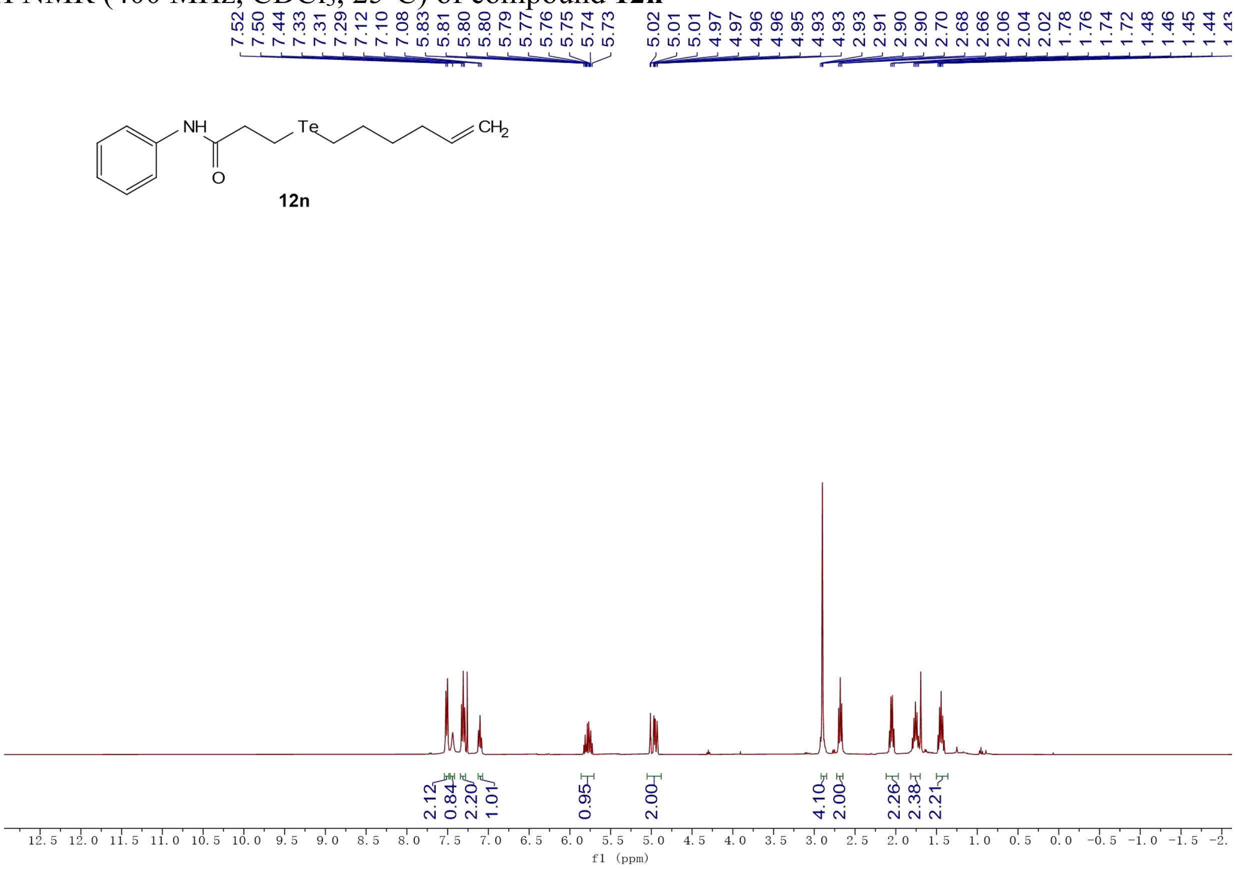

^{13}C NMR (101 MHz, $CDCl_3$, 25°C) of compound **12n**

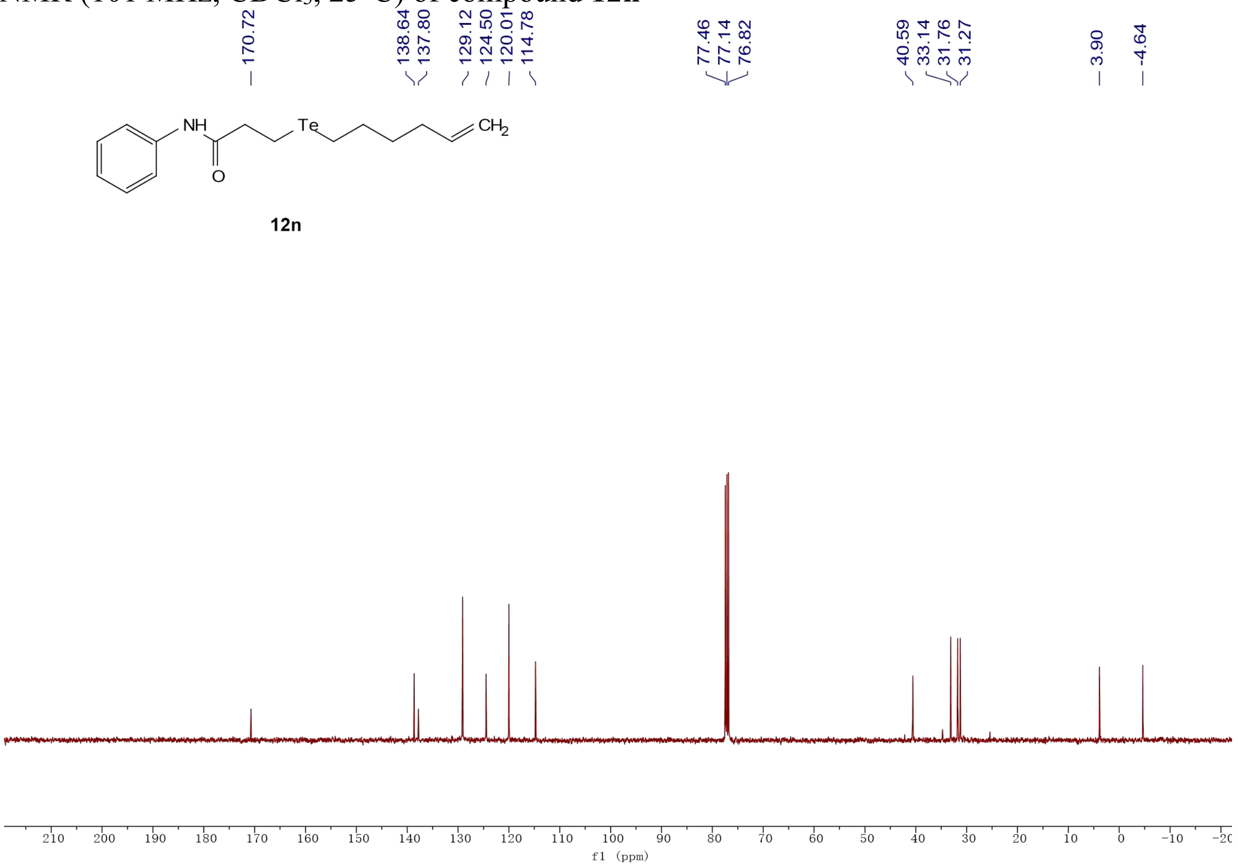

Supplementary Fig. 219. NMR of compound **12n**

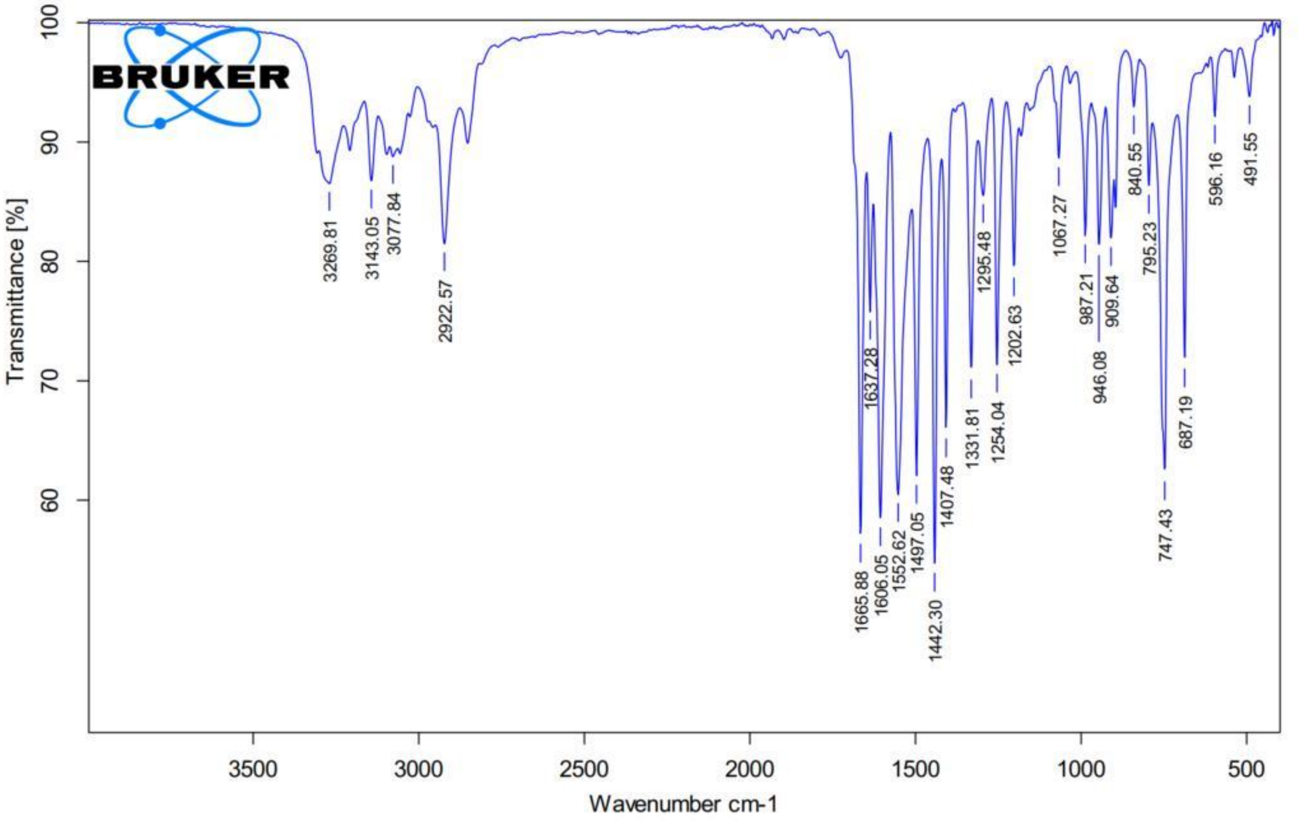

Supplementary Fig. 220. IR of compound **12n**

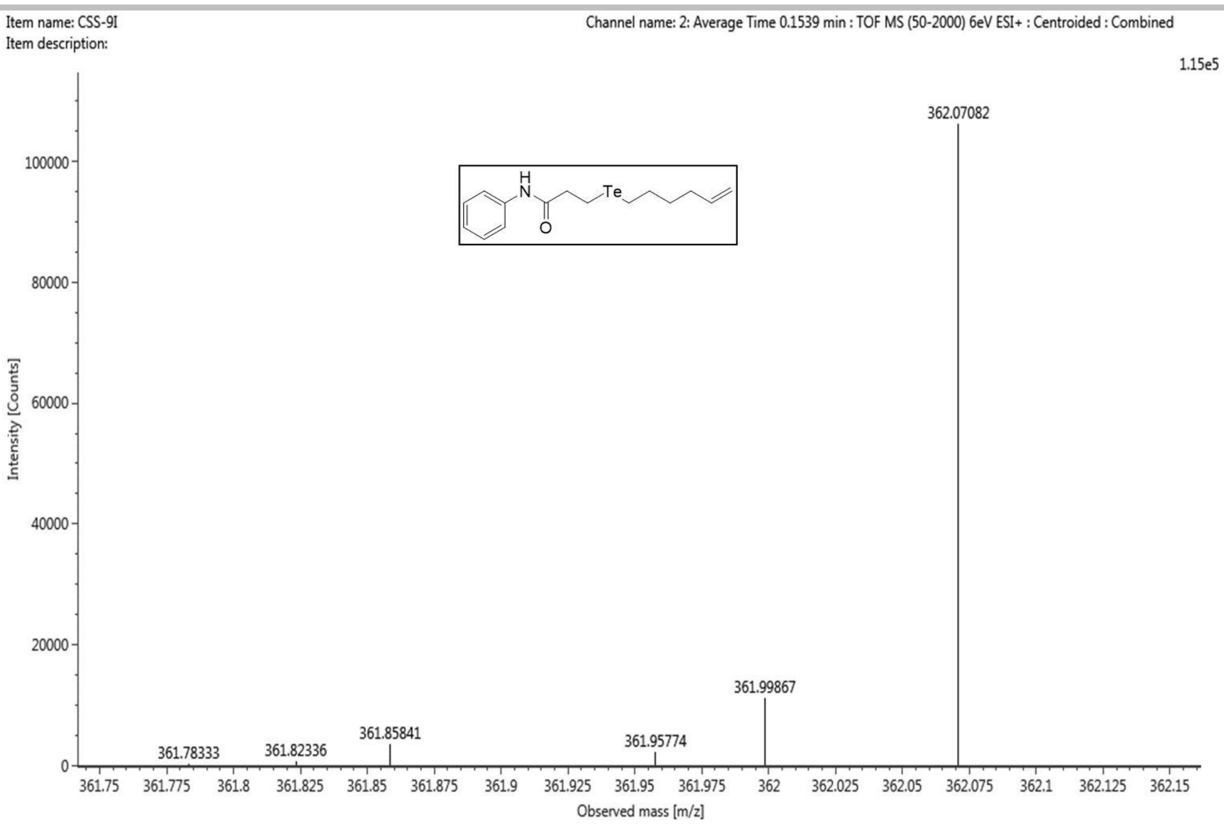

Supplementary Fig. 221. HR-MS of compound **12n**

^{1}H NMR (400 MHz, $CDCl_3$, 25°C) of compound **14**

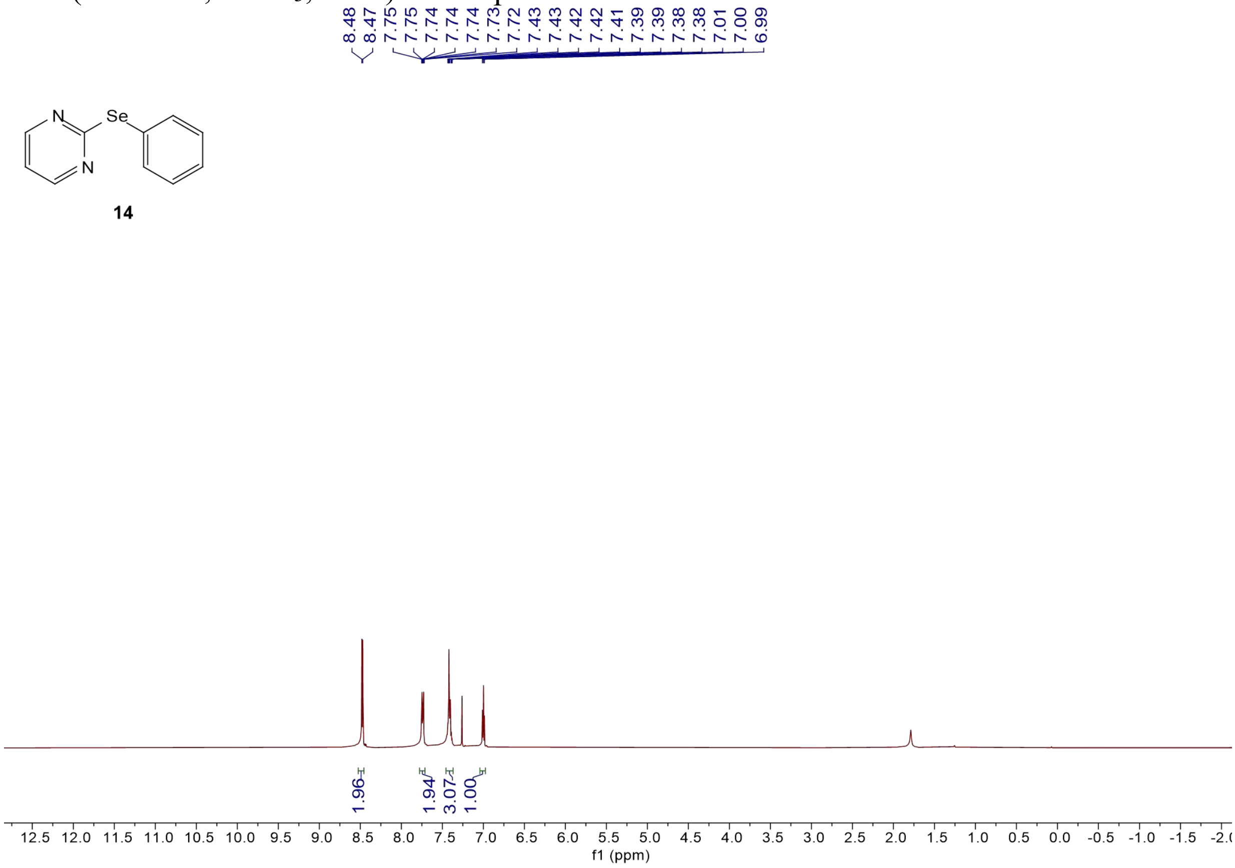

^{13}C NMR (101 MHz, $CDCl_3$, 25°C) of compound **14**

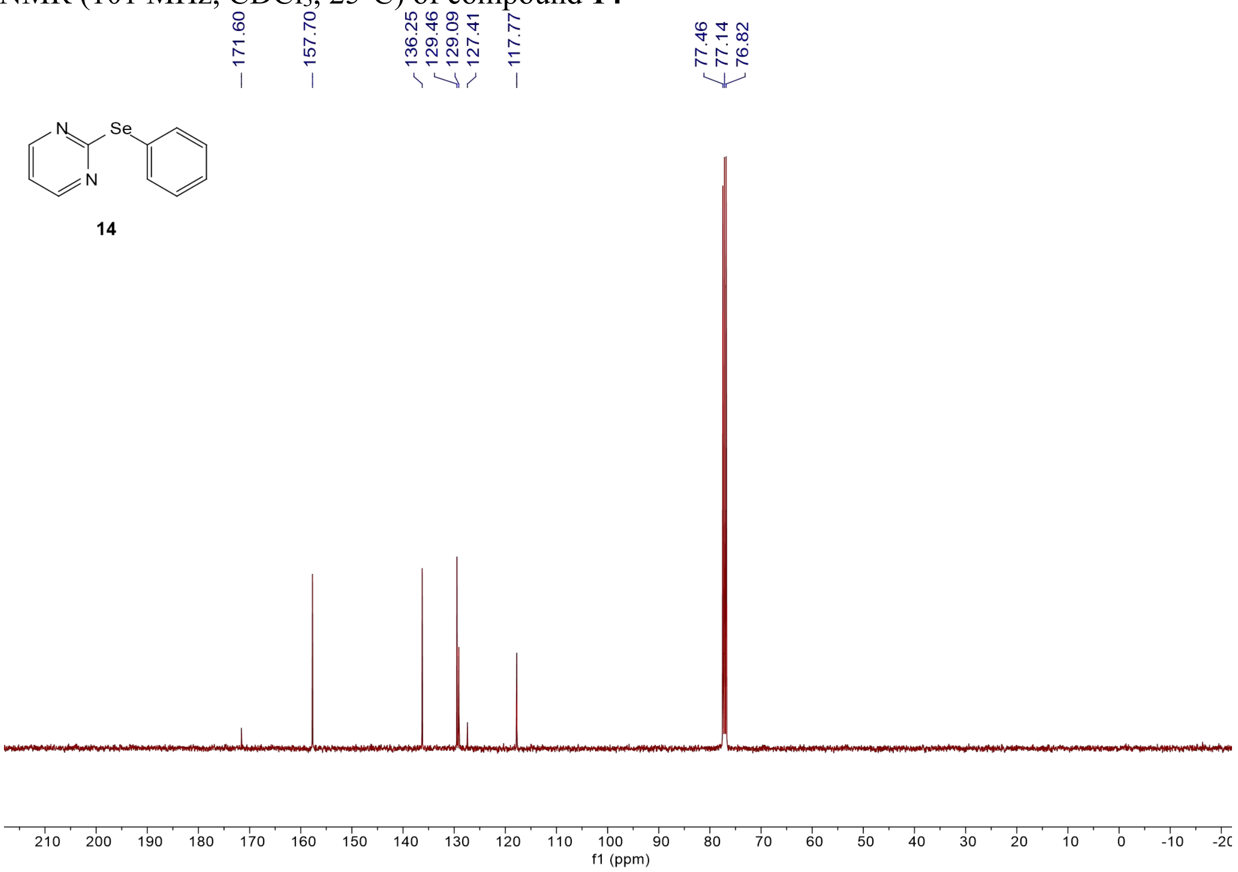

^{77}Se NMR (76 MHz, $CDCl_3$, 25°C) of compound **14**

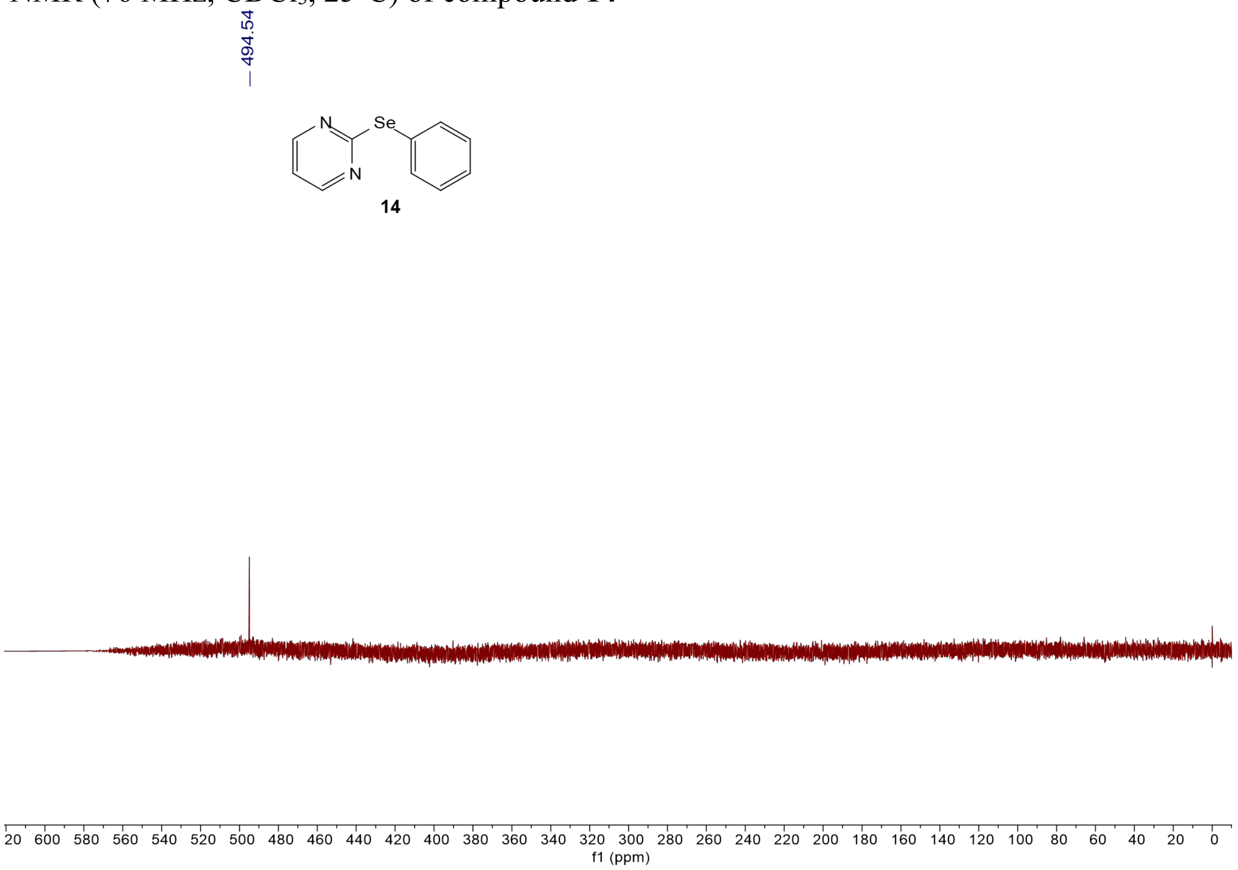

Supplementary Fig. 222. NMR of compound **14**

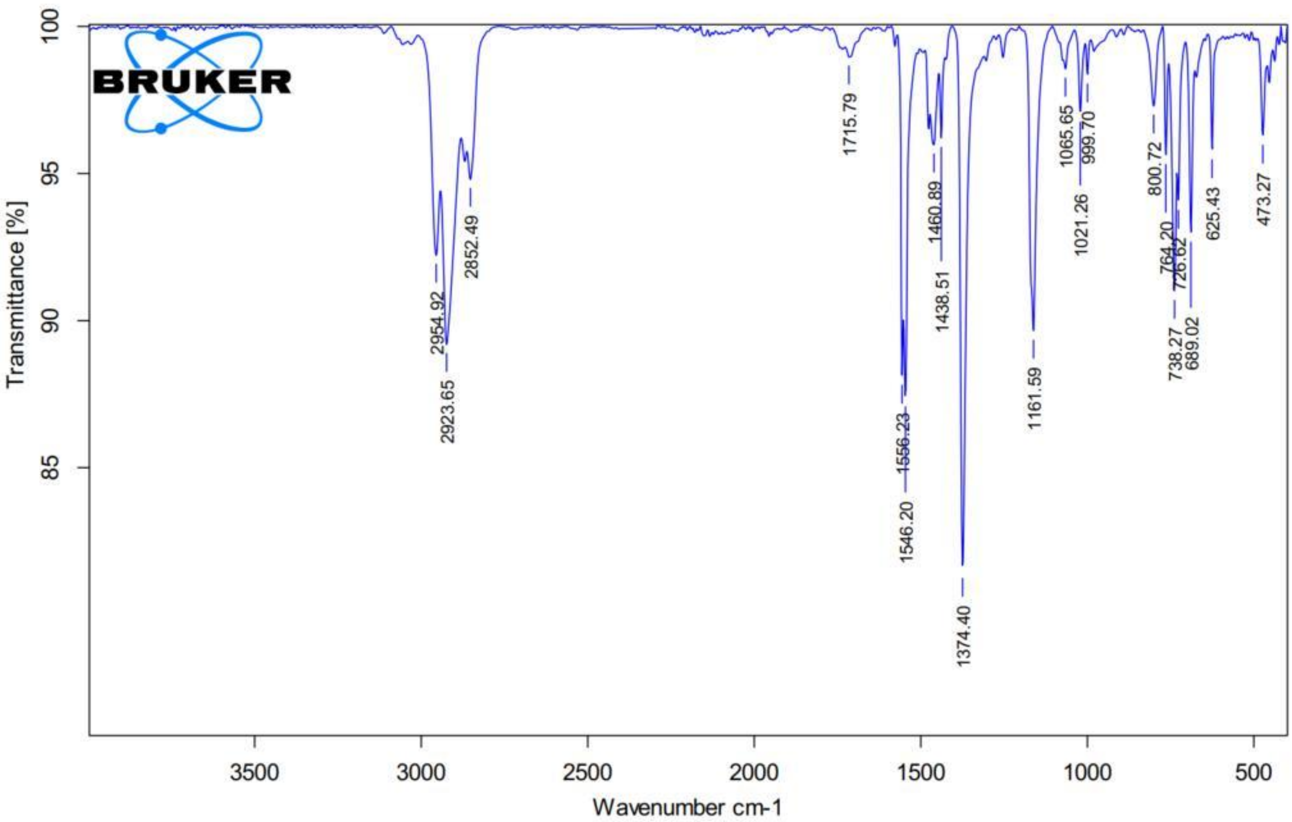

Supplementary Fig. 223. IR of compound **14**

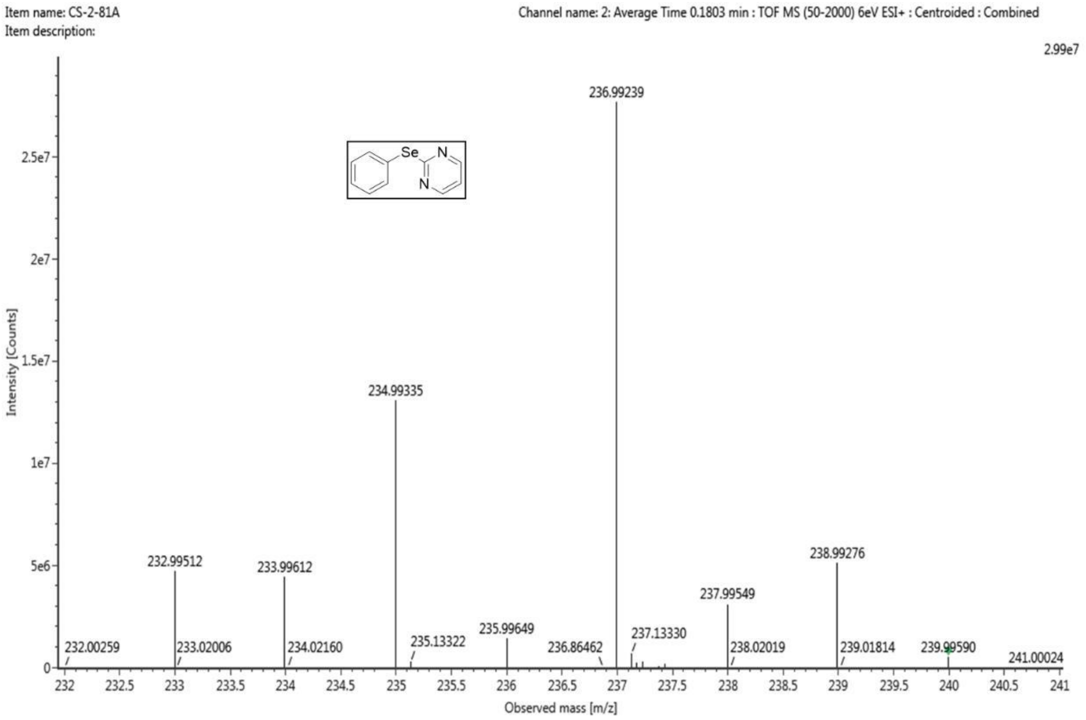

Supplementary Fig. 224. HR-MS of compound **14**

^{1}H NMR (400 MHz, $CDCl_3$, 25°C) of compound **15**

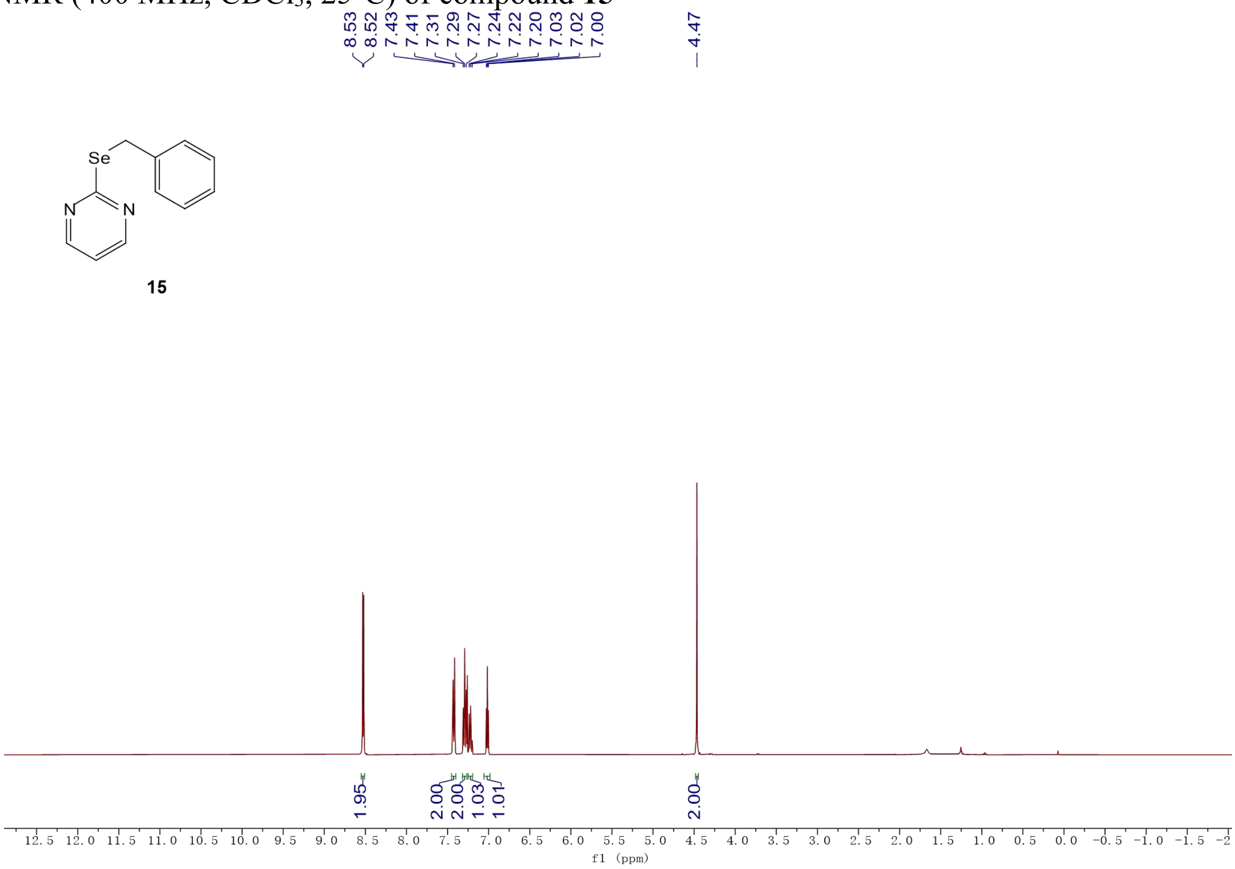

^{13}C NMR (101 MHz, $CDCl_3$, 25°C) of compound **15**

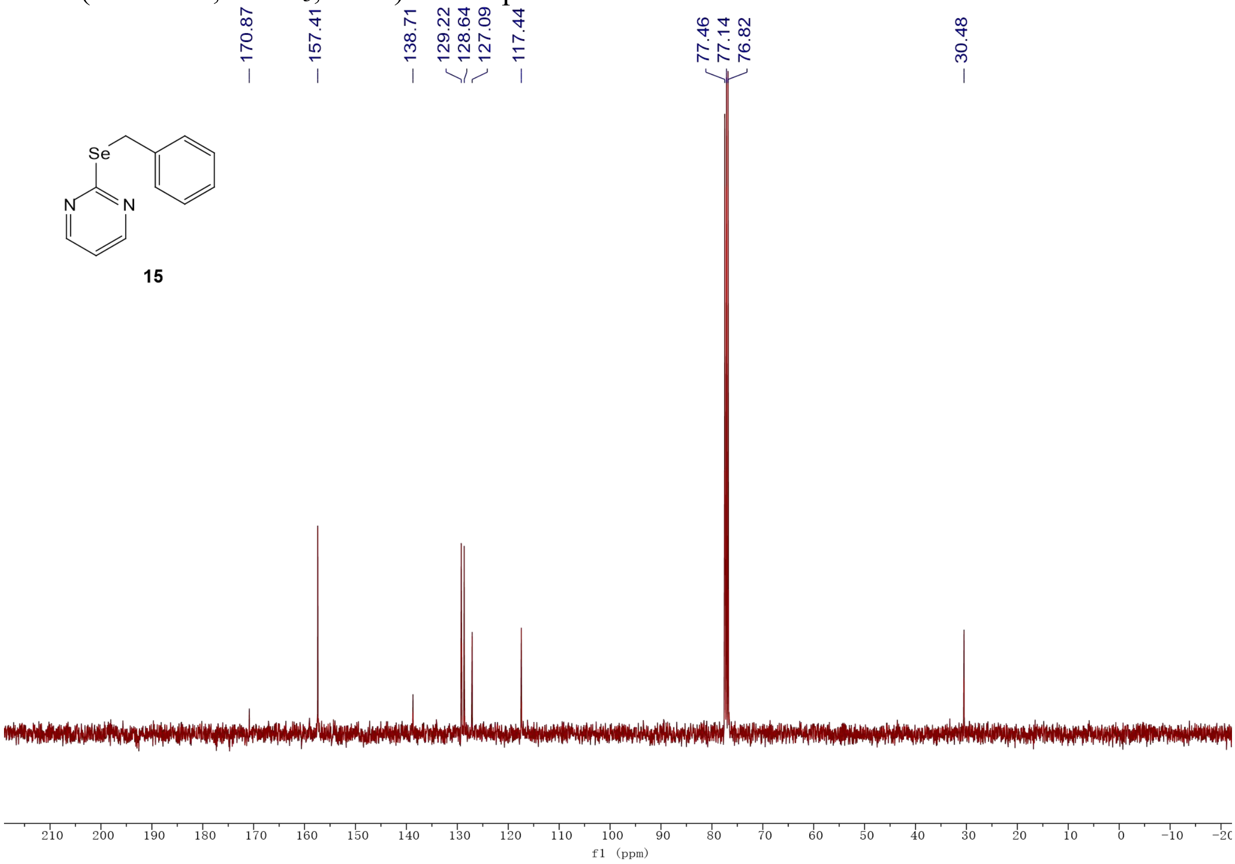

^{77}Se NMR (76 MHz, $CDCl_3$, 25°C) of compound **15**

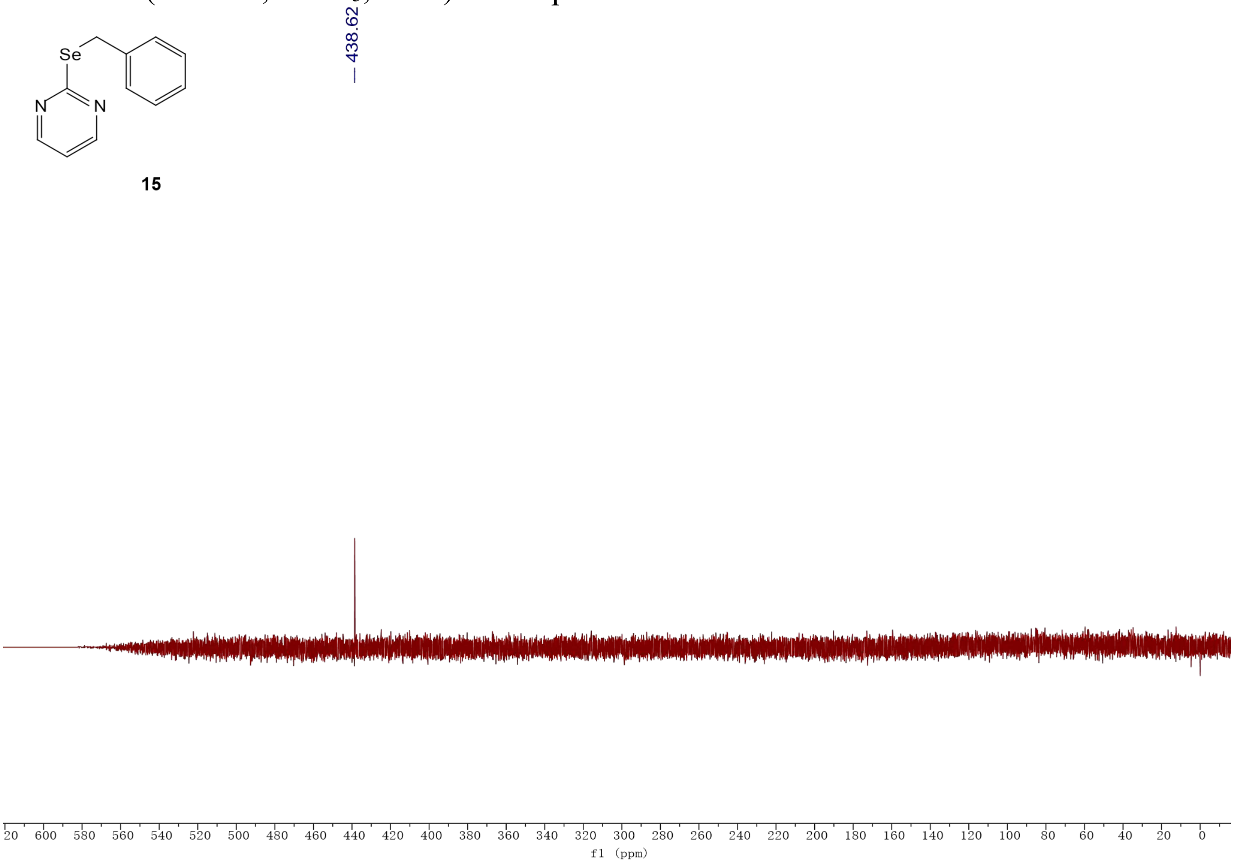

Supplementary Fig. 225. NMR of compound **15**

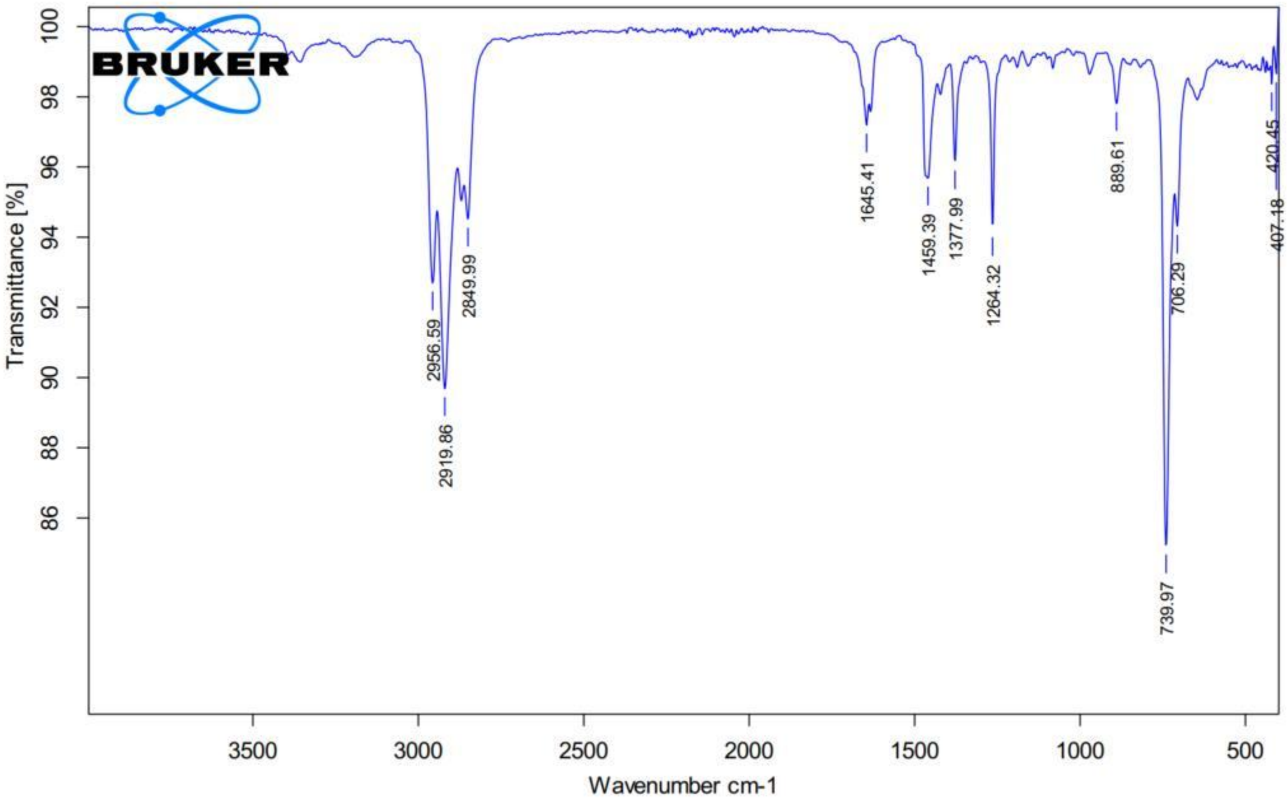

Supplementary Fig. 226. IR of compound **15**

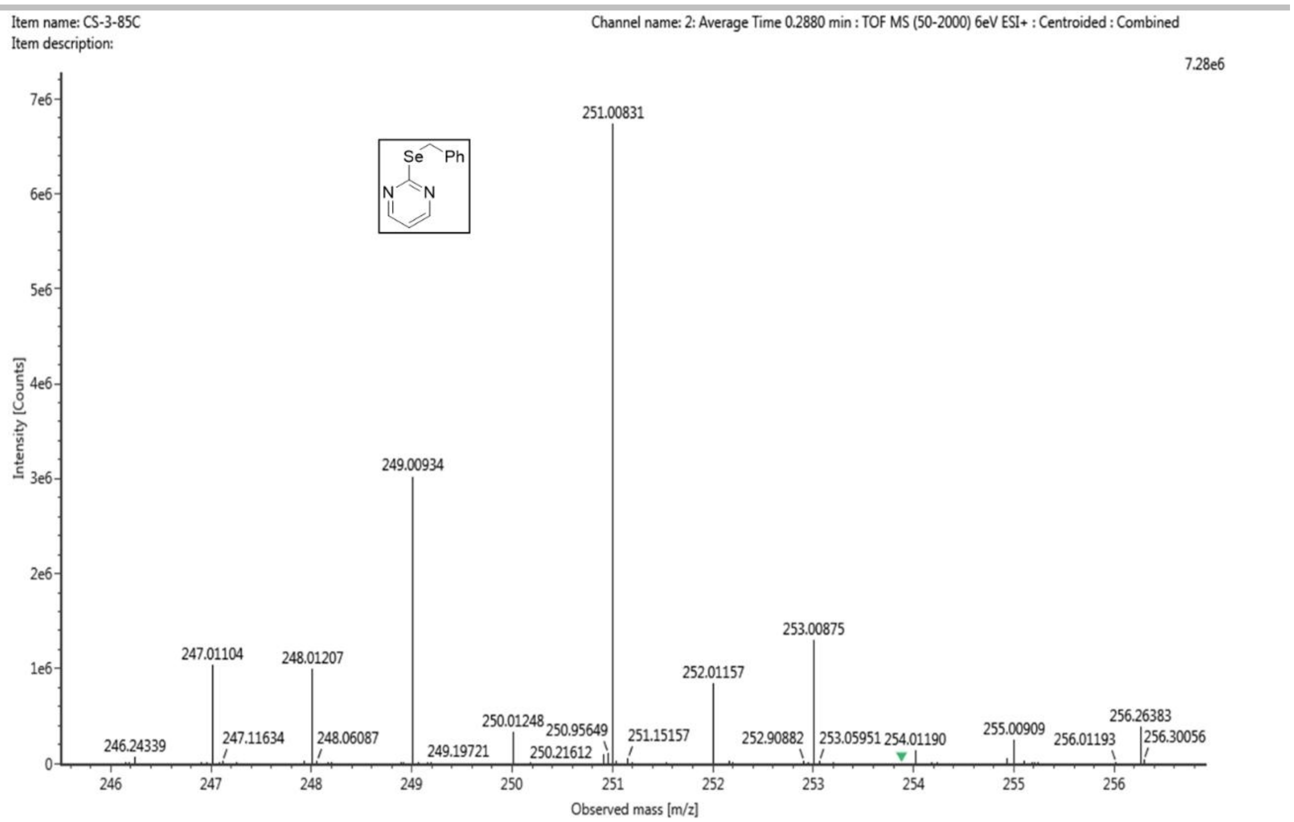

Supplementary Fig. 227. HR-MS of compound **15**

^{1}H NMR (400 MHz, $CDCl_3$, 25°C) of compound **16**

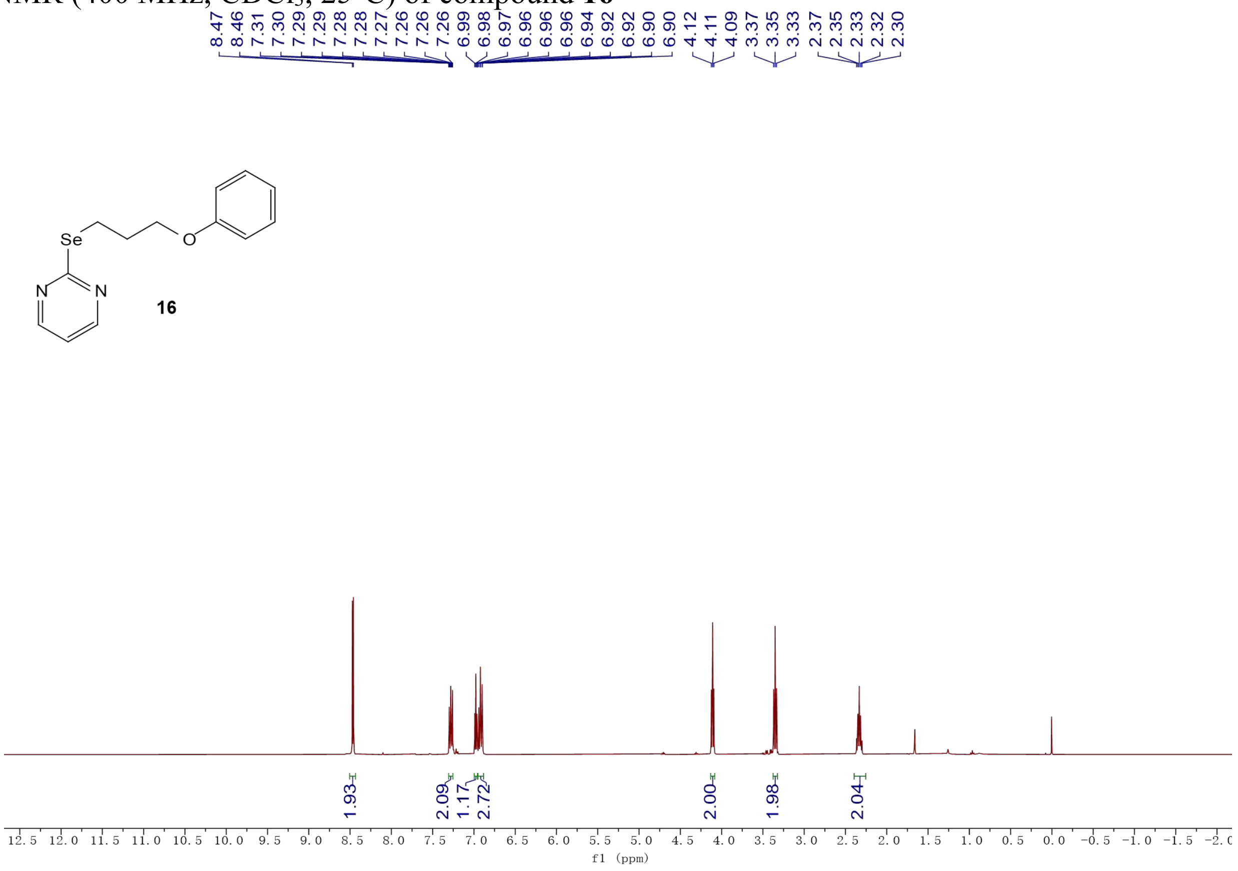

^{13}C NMR (101 MHz, $CDCl_3$, 25°C) of compound **16**

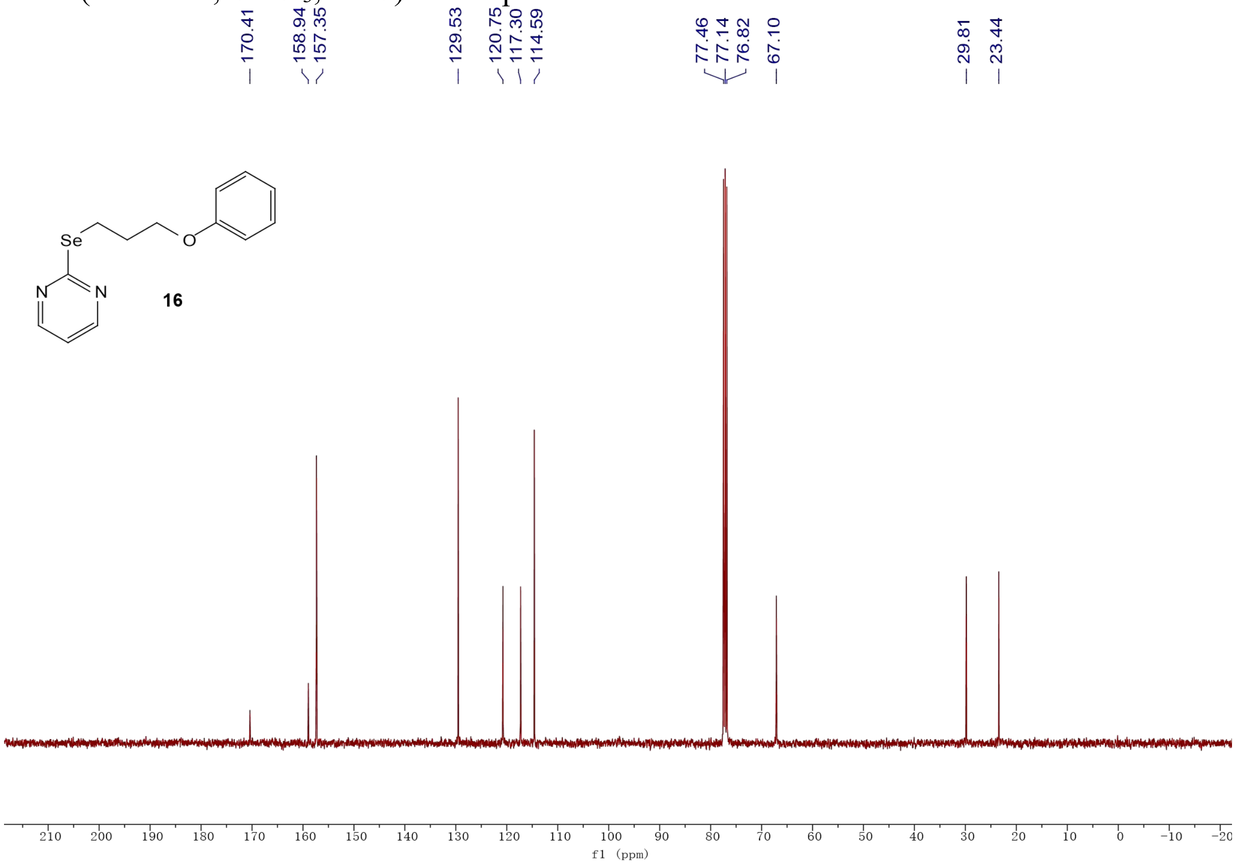

^{77}Se NMR (76 MHz, $CDCl_3$, 25°C) of compound **16**

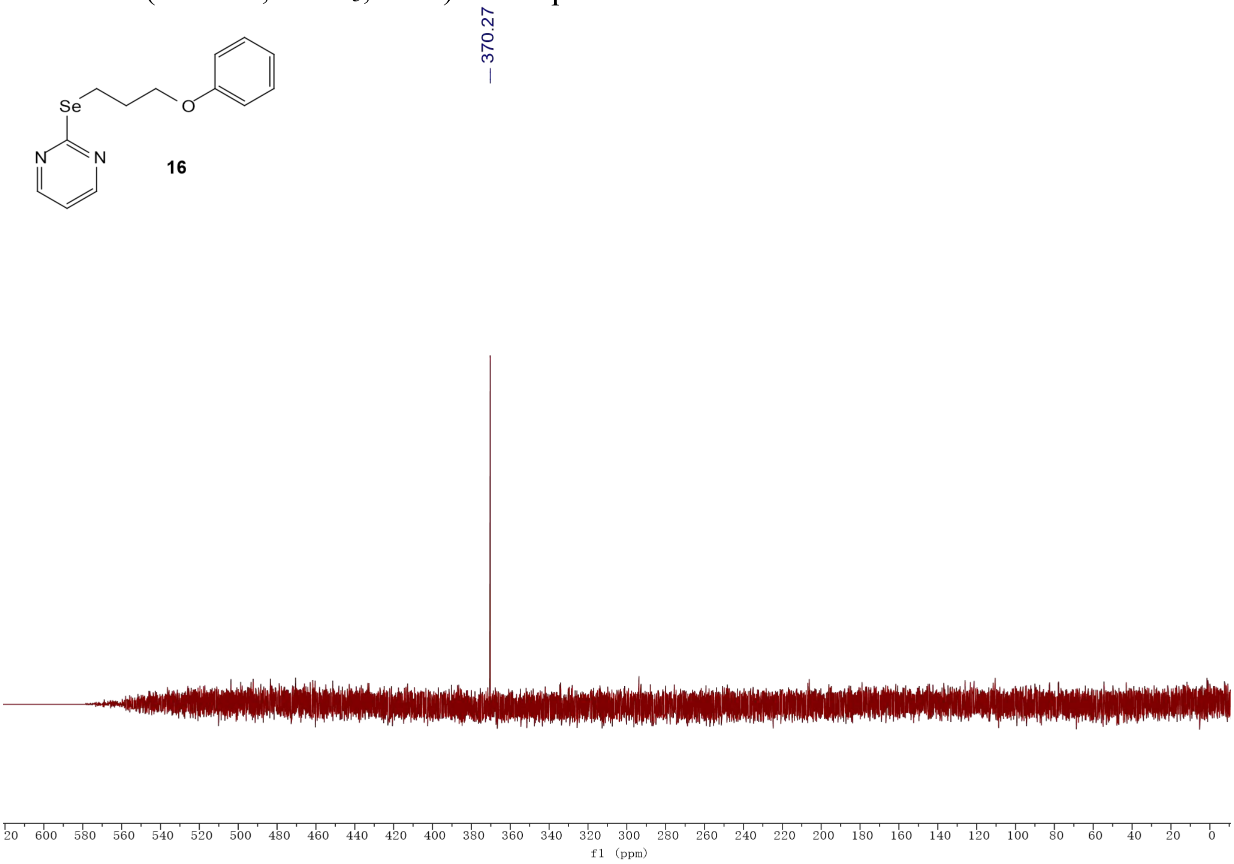

Supplementary Fig. 228. NMR of compound **16**

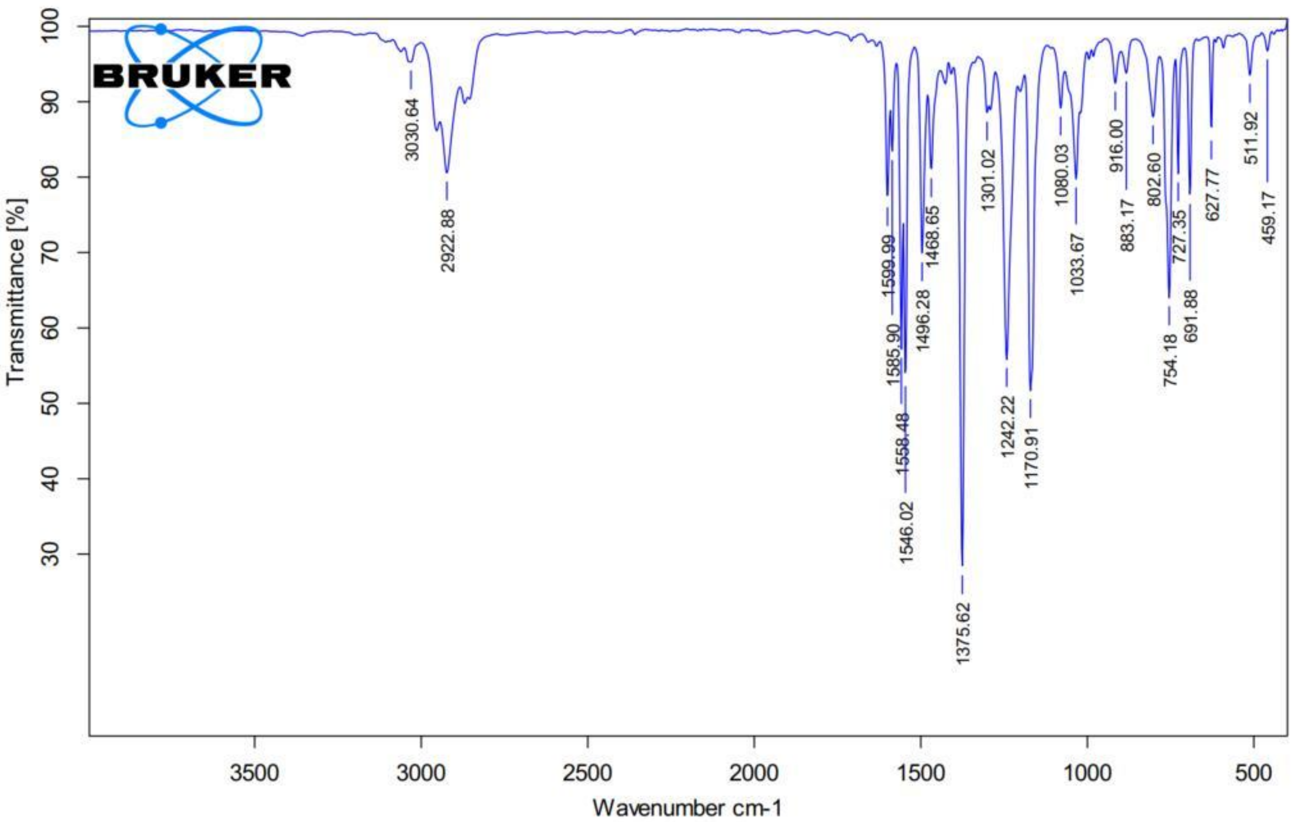

Supplementary Fig. 229. IR of compound **16**

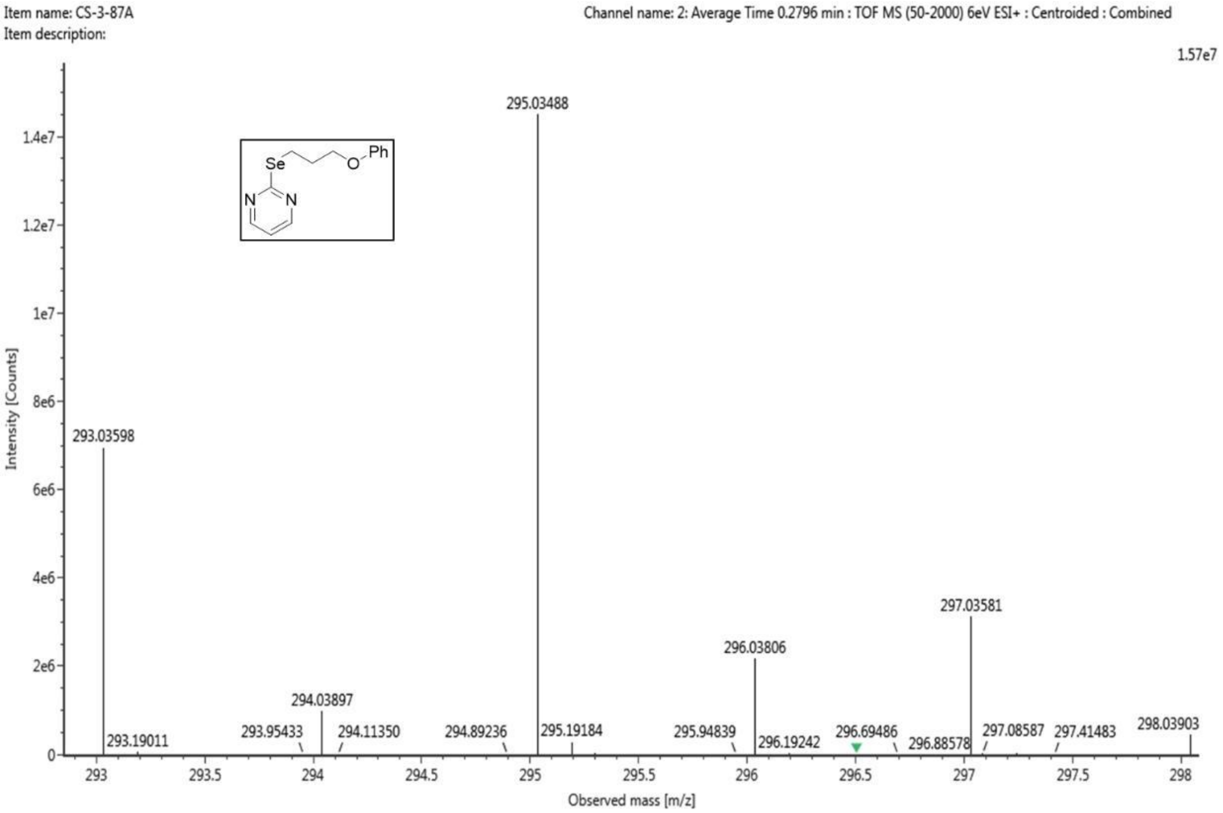

Supplementary Fig. 230. HR-MS of compound **16**

^{1}H NMR (400 MHz, $CDCl_3$, 25℃) of compound **17**

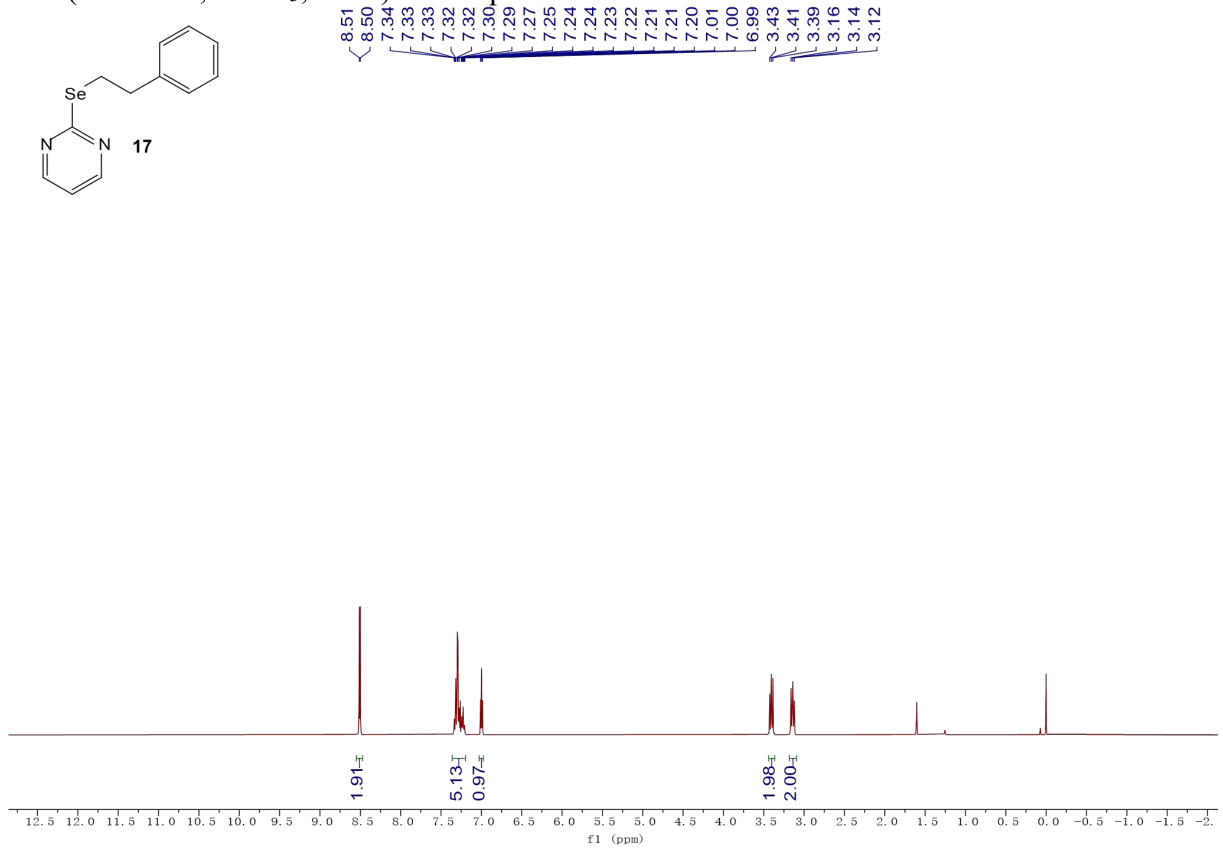

^{13}C NMR (101 MHz, $CDCl_3$, 25℃) of compound **17**

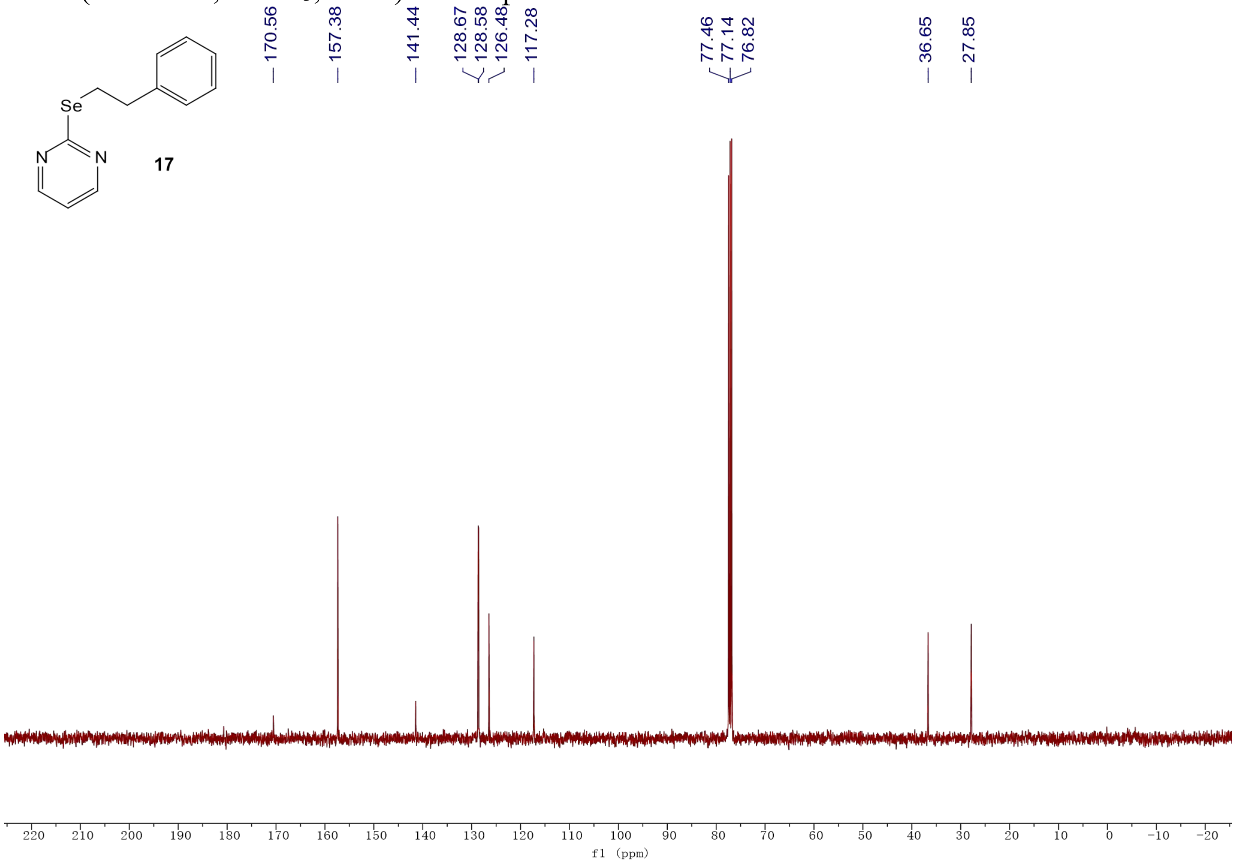

^{77}Se NMR (76 MHz, $CDCl_3$, 25°C) of compound **17**

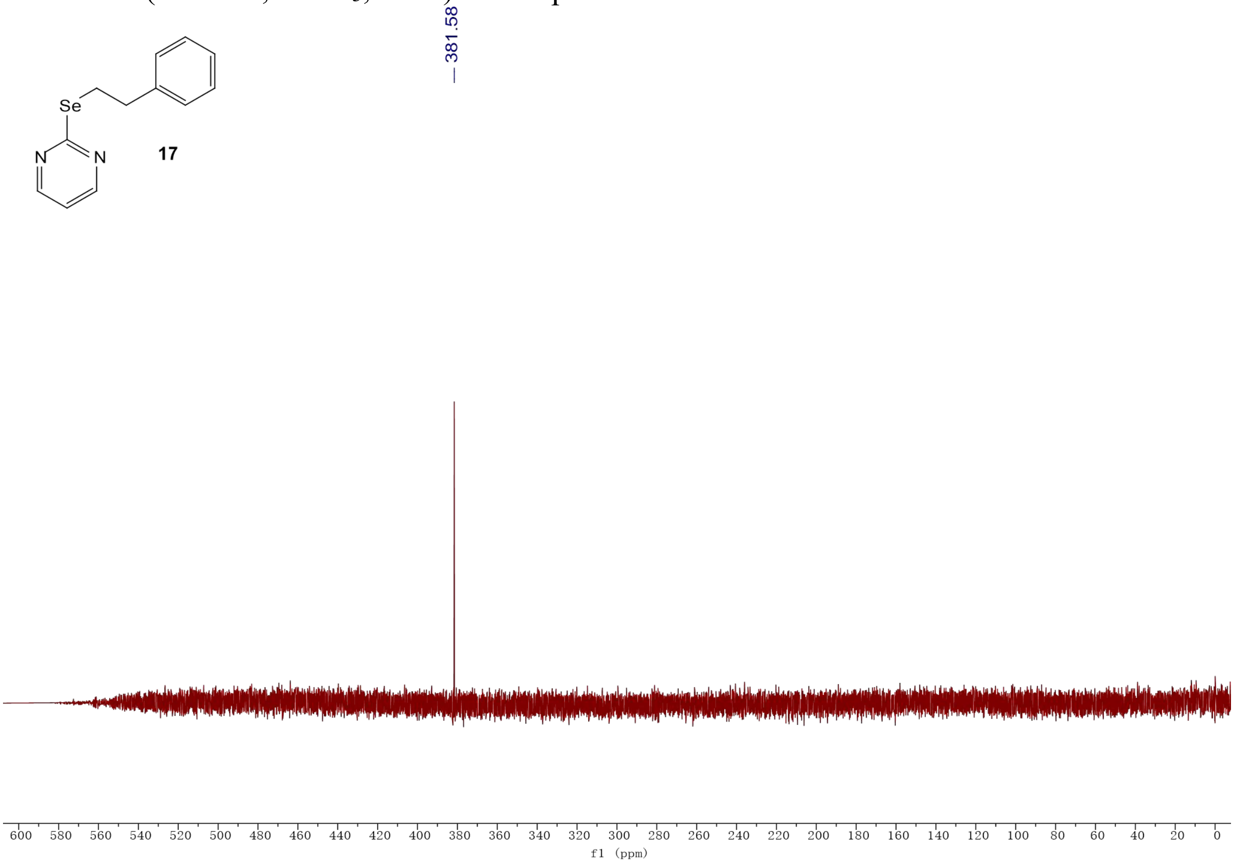

Supplementary Fig. 231. NMR of compound **17**

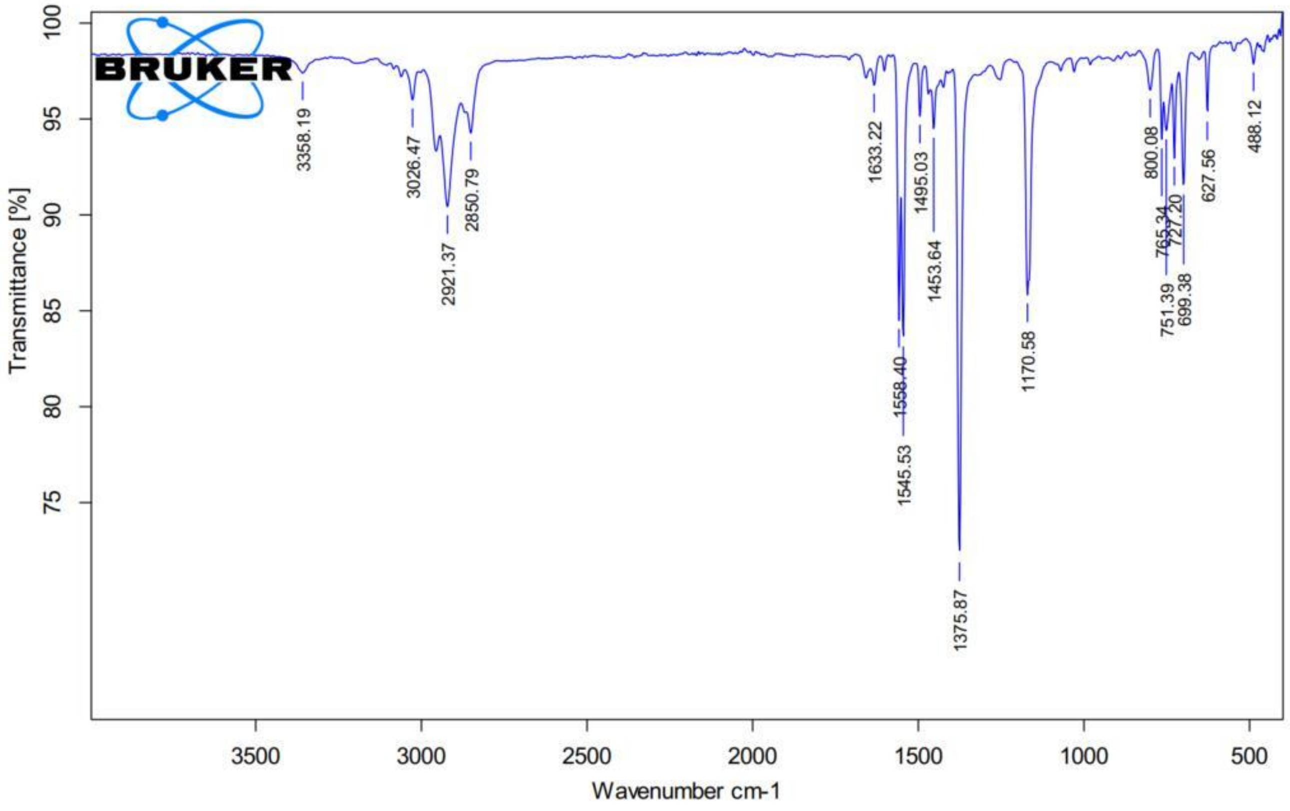

Supplementary Fig. 232. IR of compound **17**

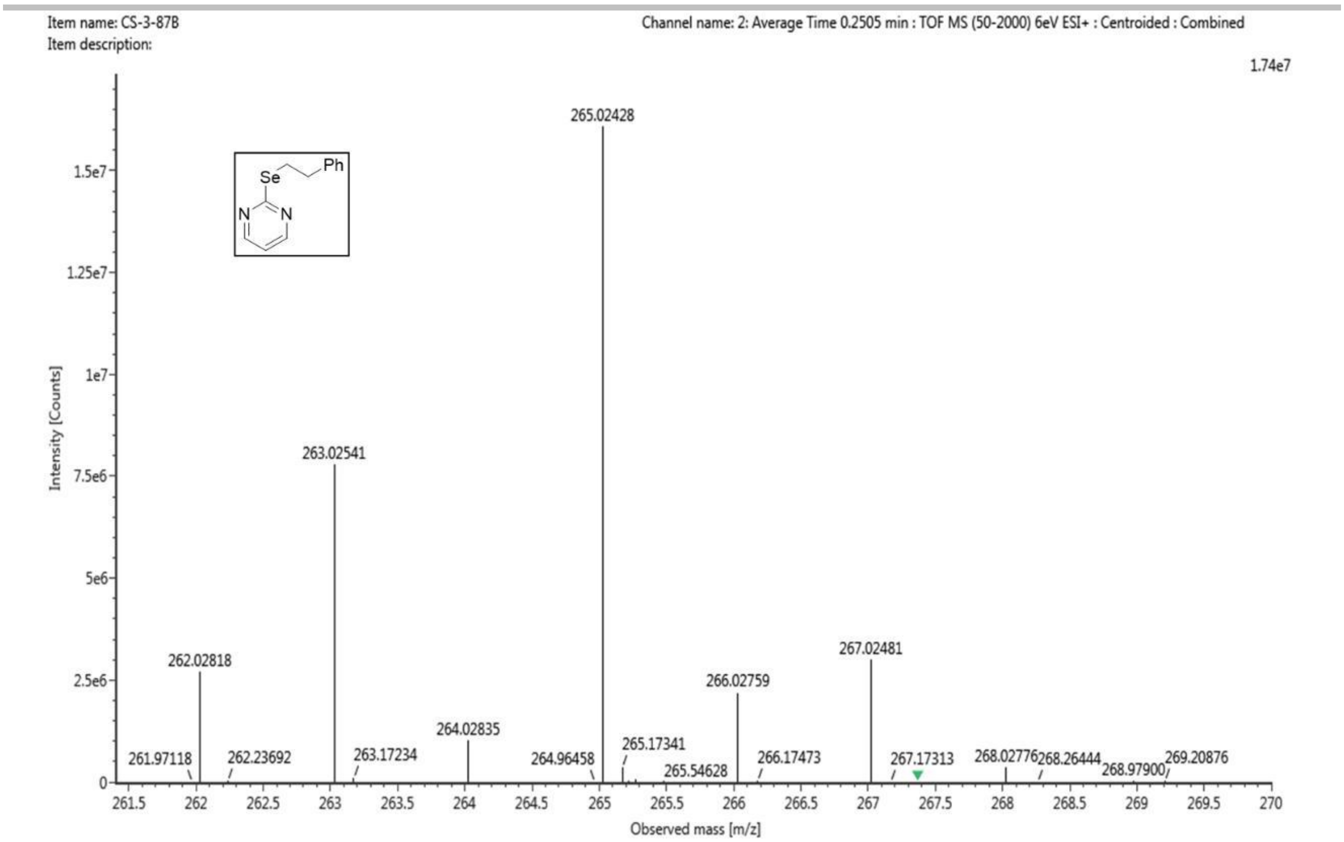

Supplementary Fig. 233. HR-MS of compound **17**

^{1}H NMR (400 MHz, $CDCl_3$, 25°C) of compound **18**

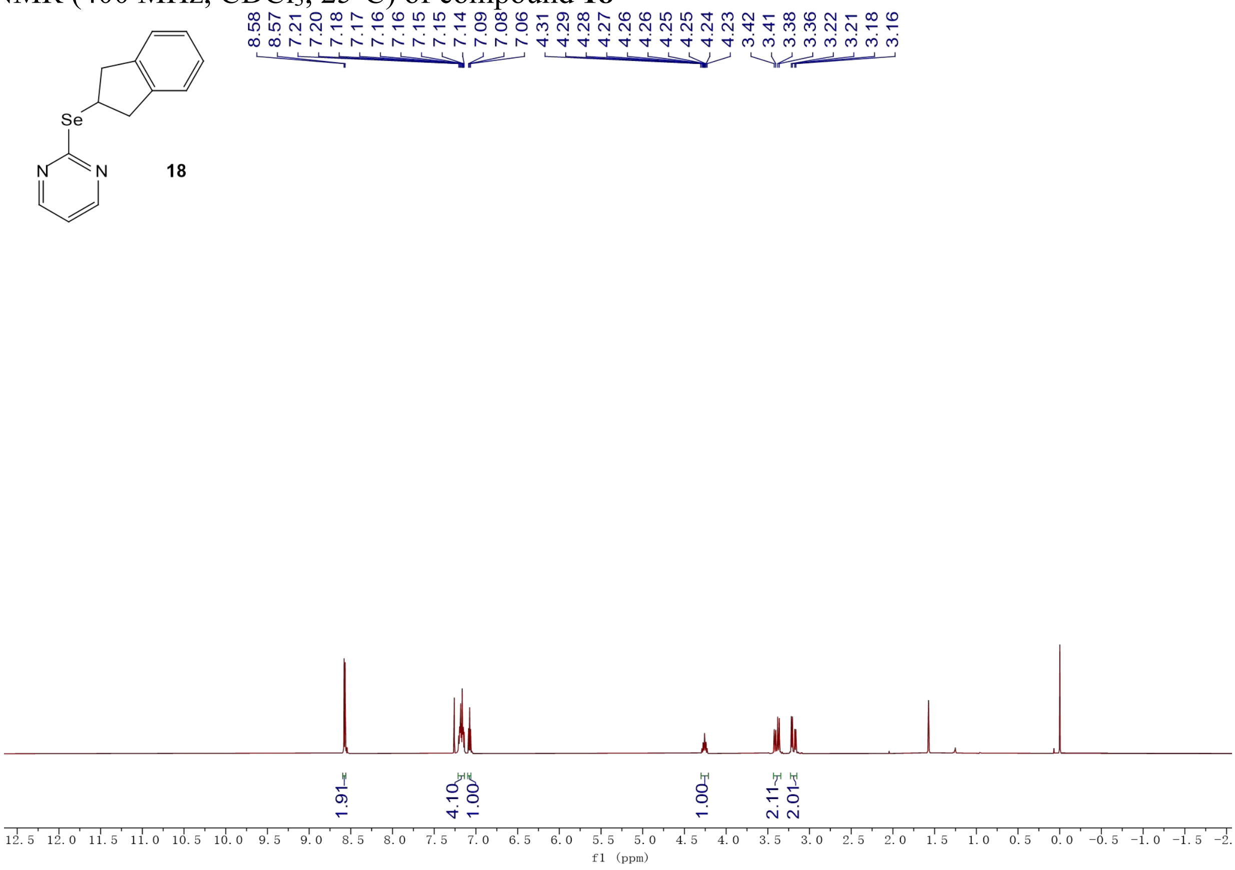

^{13}C NMR (101 MHz, $CDCl_3$, 25°C) of compound **18**

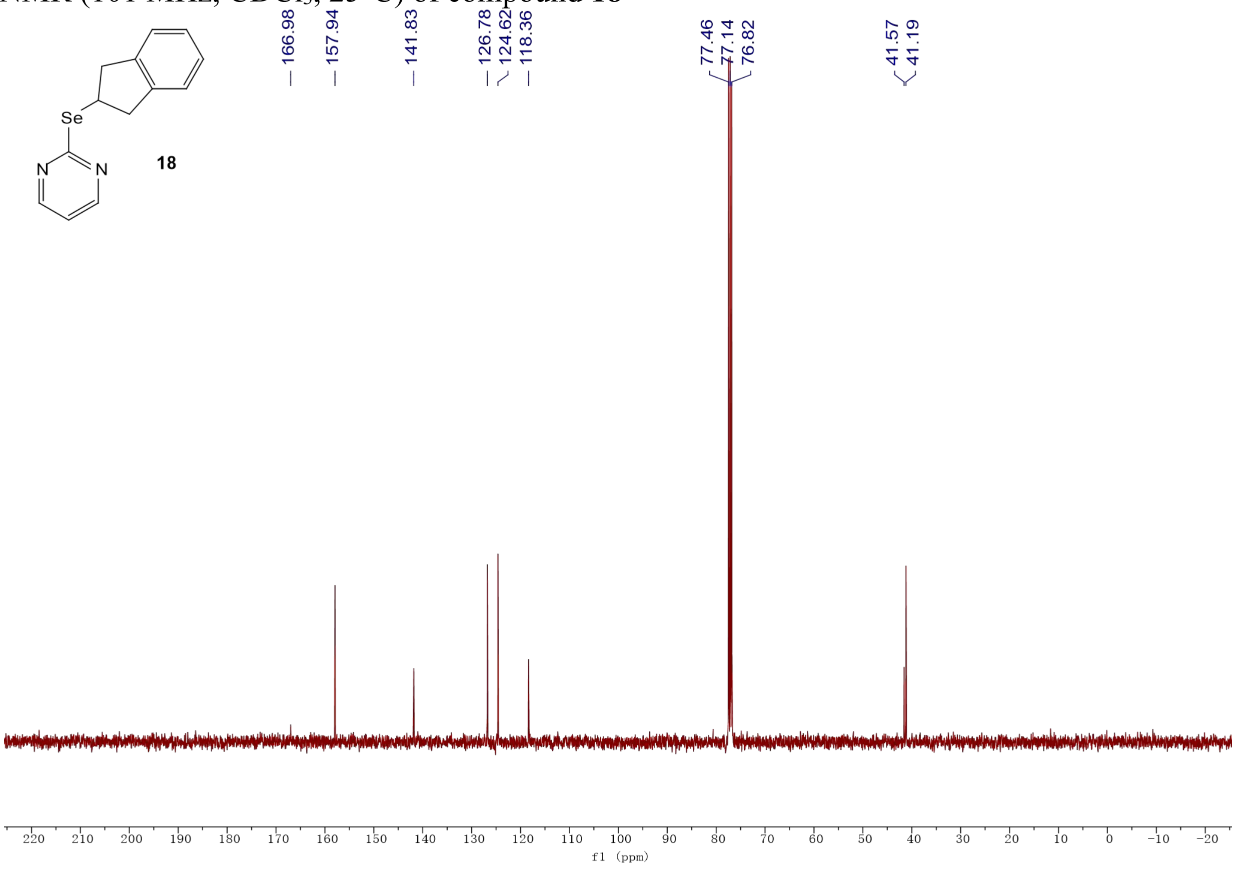

^{77}Se NMR (76 MHz, $CDCl_3$, 25°C) of compound **18**

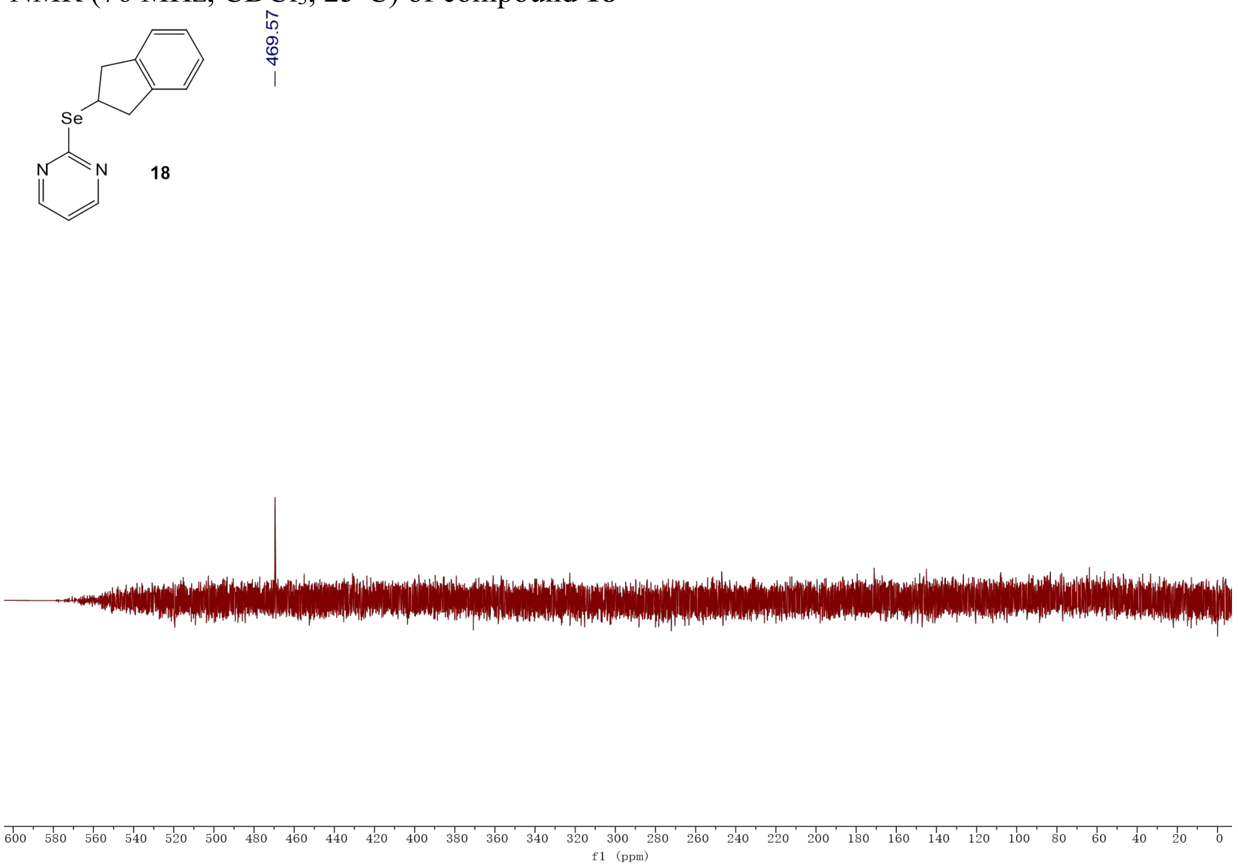

Supplementary Fig. 234. NMR of compound **18**

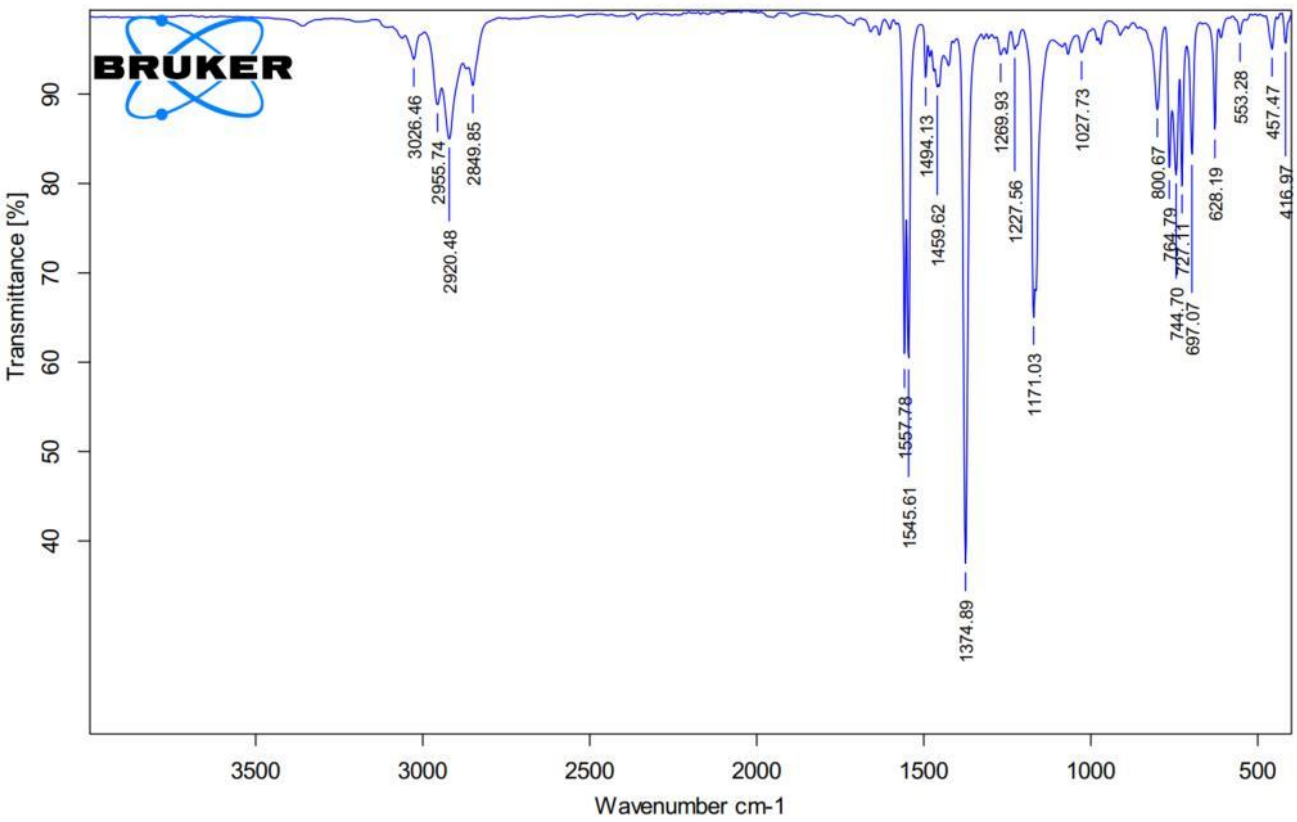

Supplementary Fig. 235. IR of compound **18**

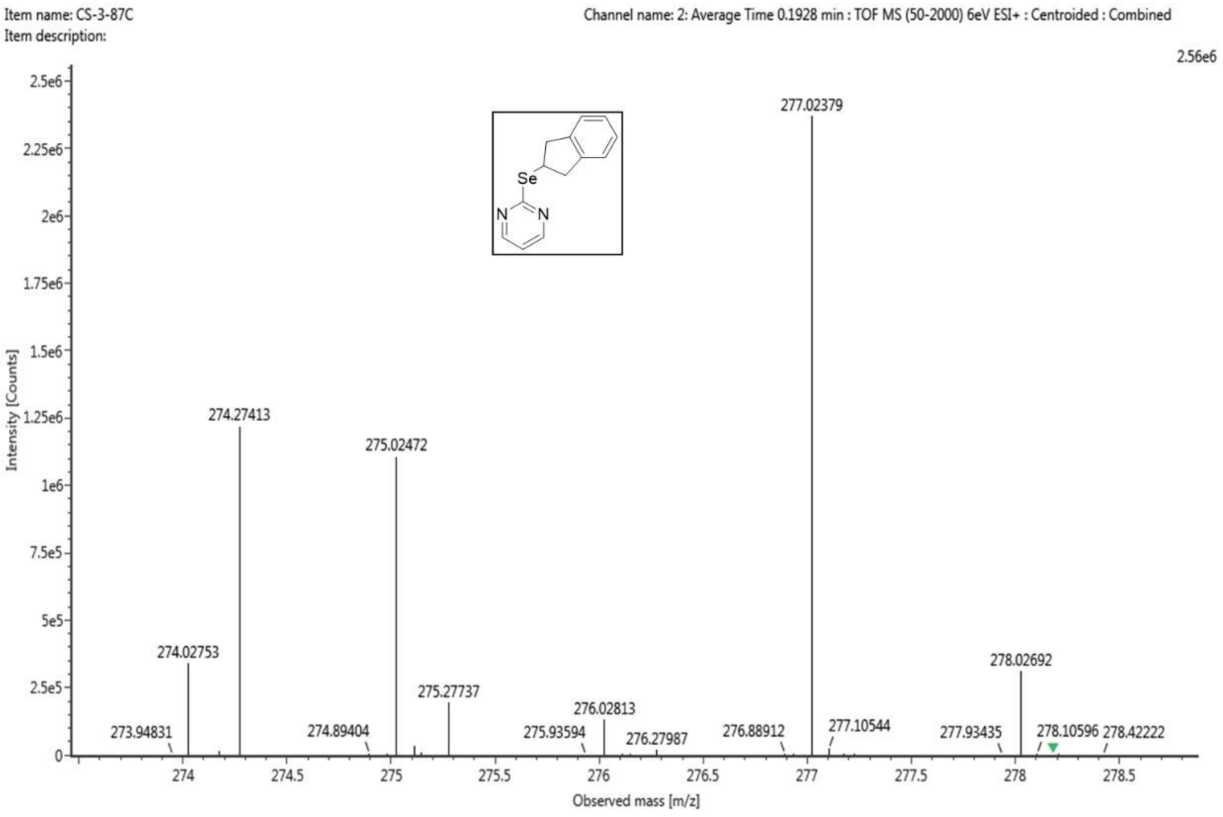

Supplementary Fig. 236. HR-MS of compound **18**

^{1}H NMR (400 MHz, $CDCl_3$, 25°C) of compound **19**

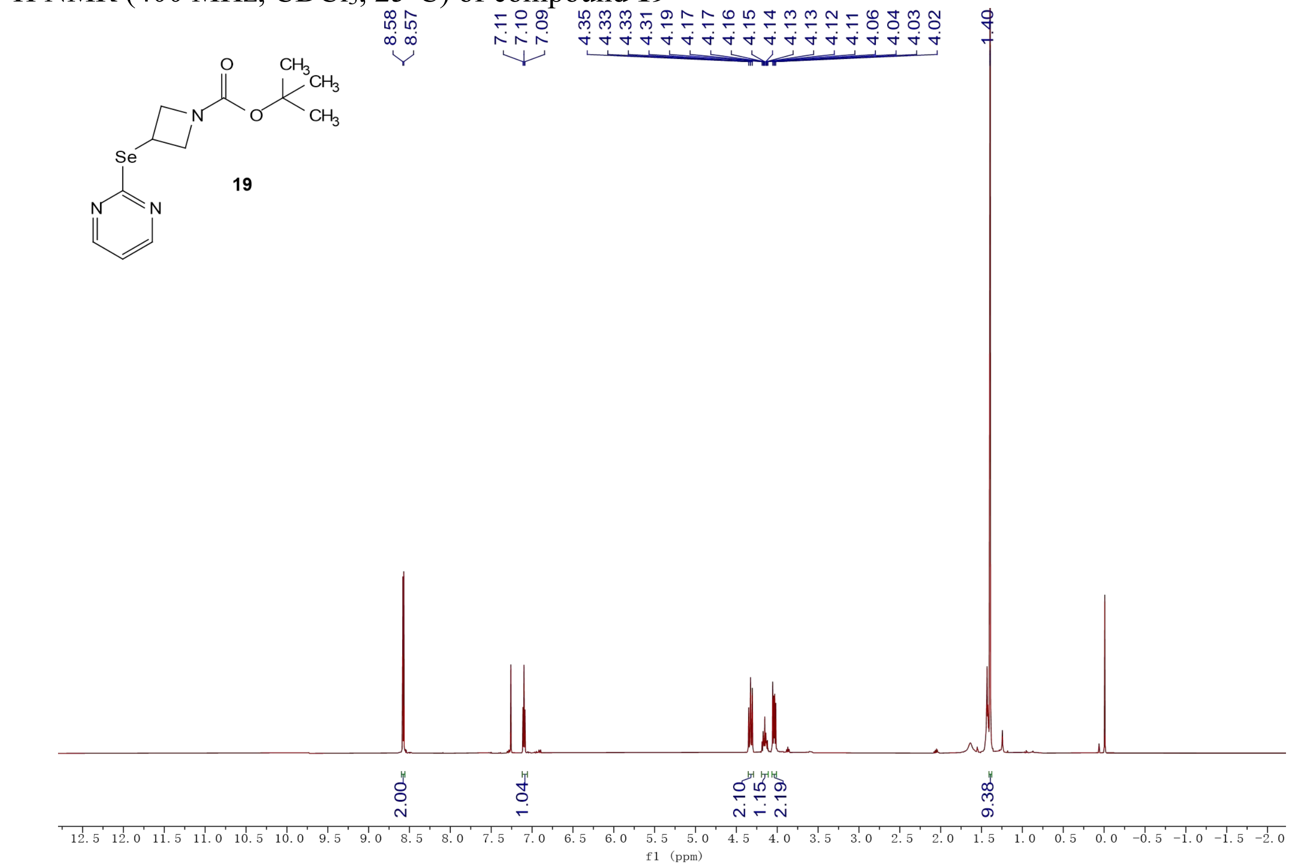

^{13}C NMR (101 MHz, $CDCl_3$, 25°C) of compound **19**

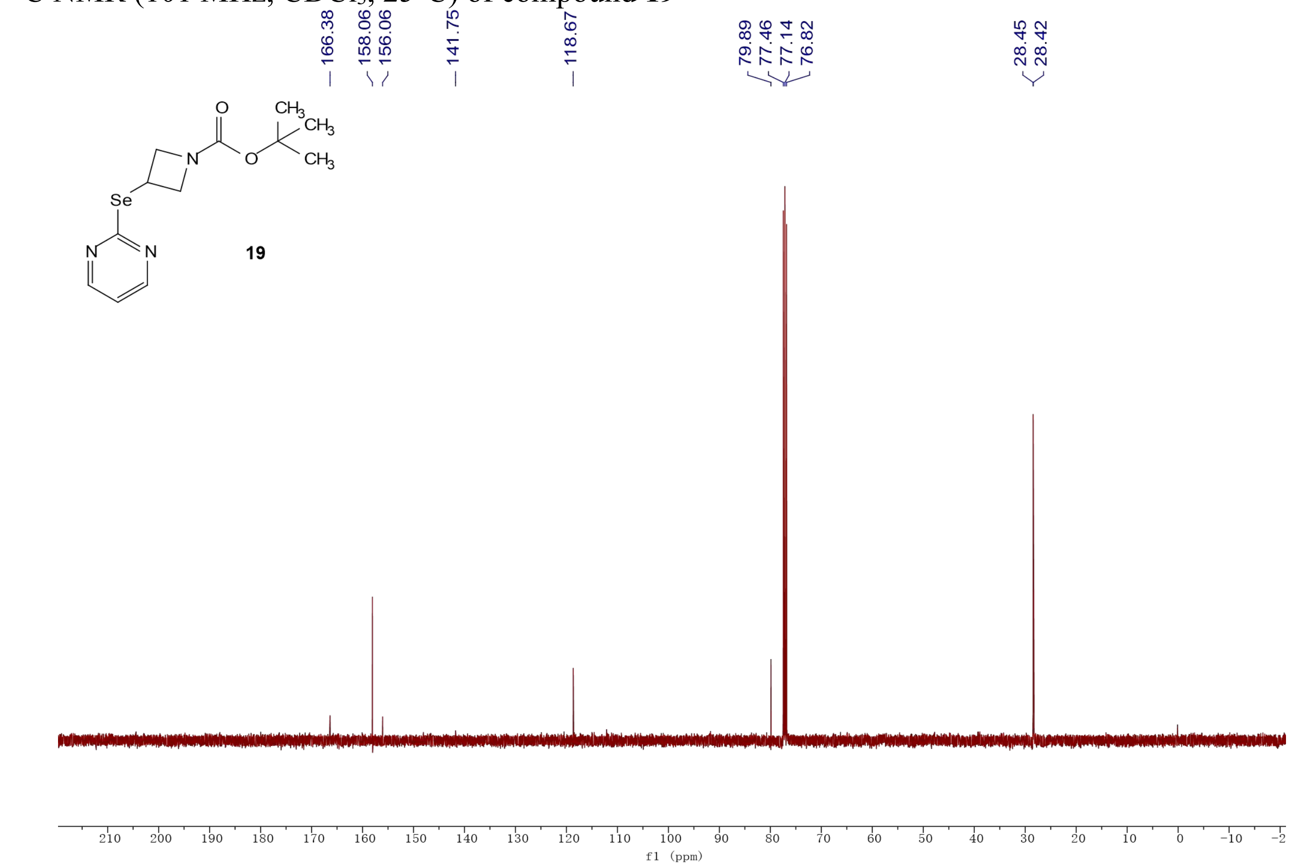

^{77}Se NMR (76 MHz, $CDCl_3$, 25°C) of compound **19**

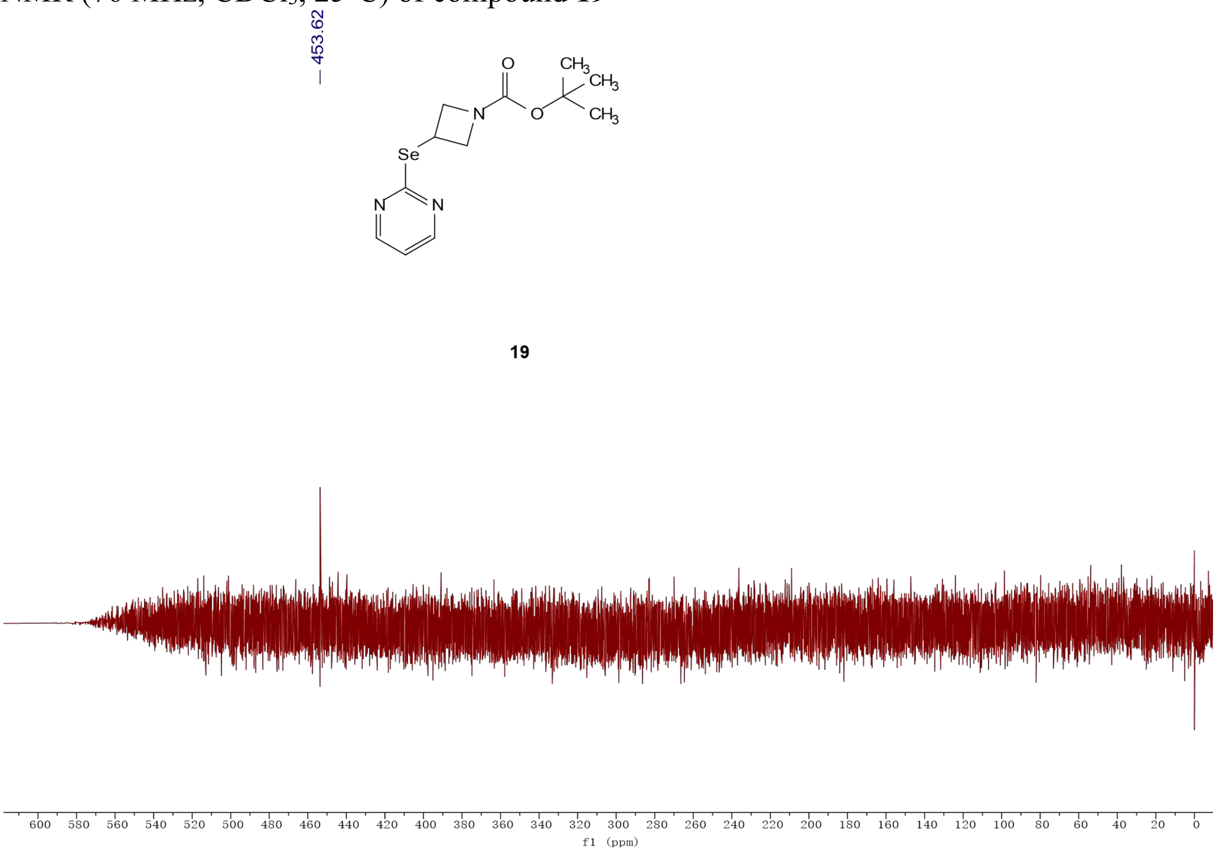

Supplementary Fig. 237. NMR of compound **19**

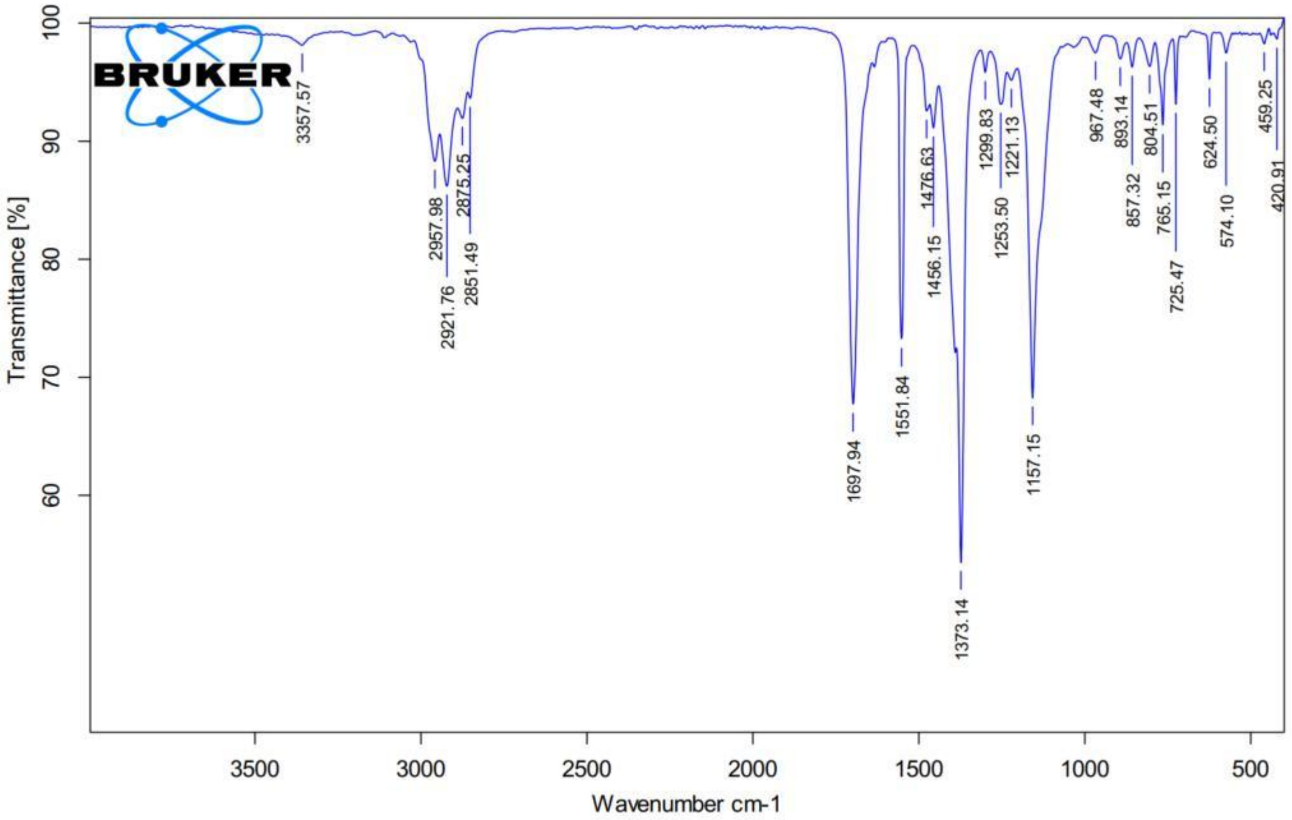

Supplementary Fig. 238. IR of compound **19**

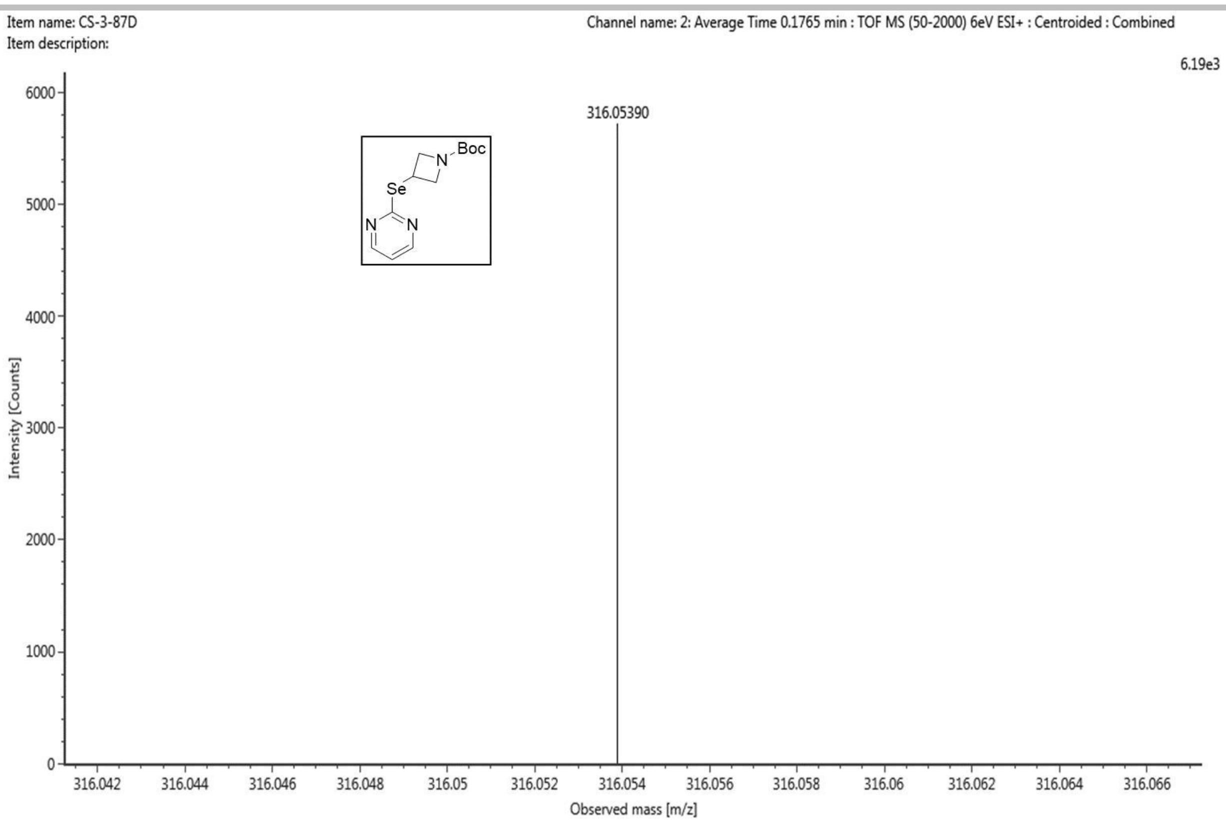

Supplementary Fig. 239. HR-MS of compound **19**

^{1}H NMR (400 MHz, $CDCl_3$, 25°C) of compound **21**

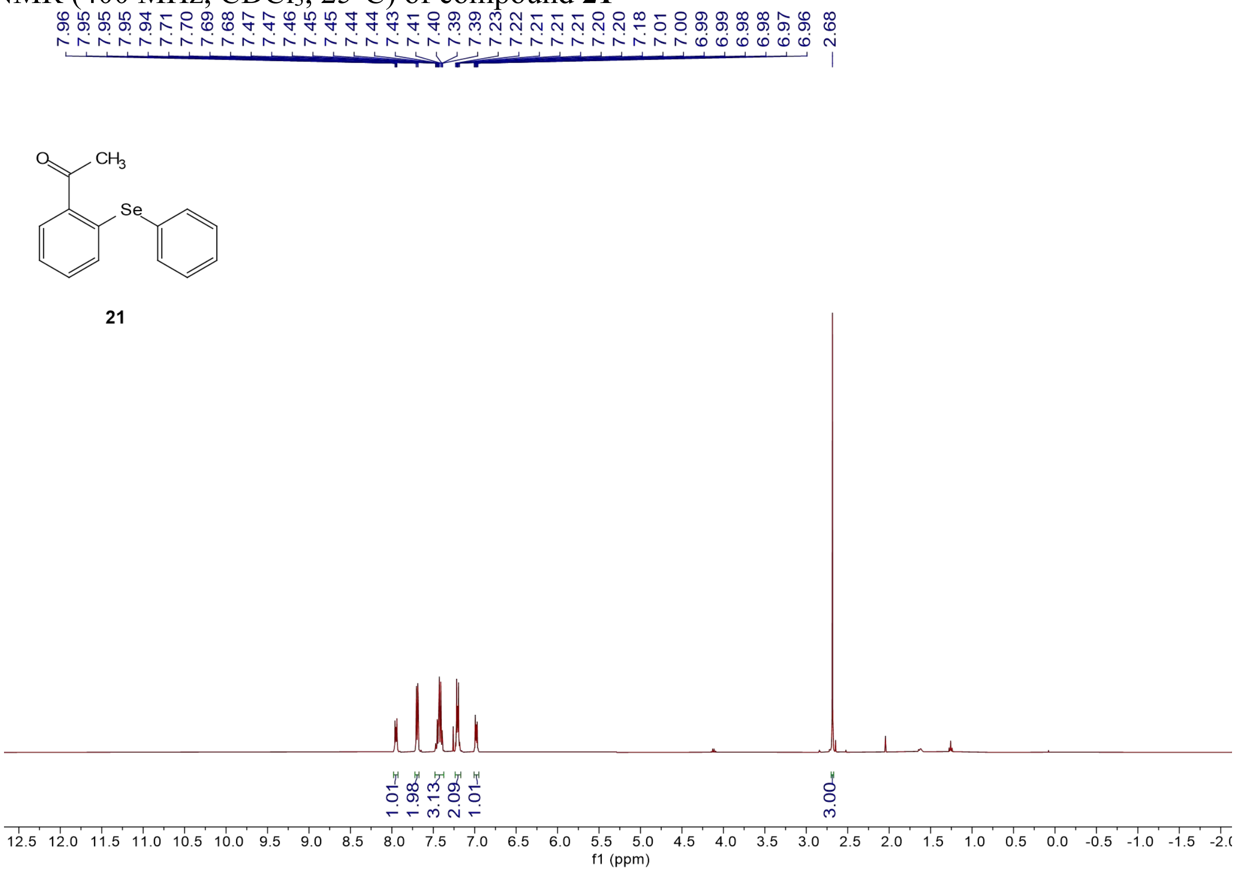

^{13}C NMR (101 MHz, $CDCl_3$, 25°C) of compound **21**

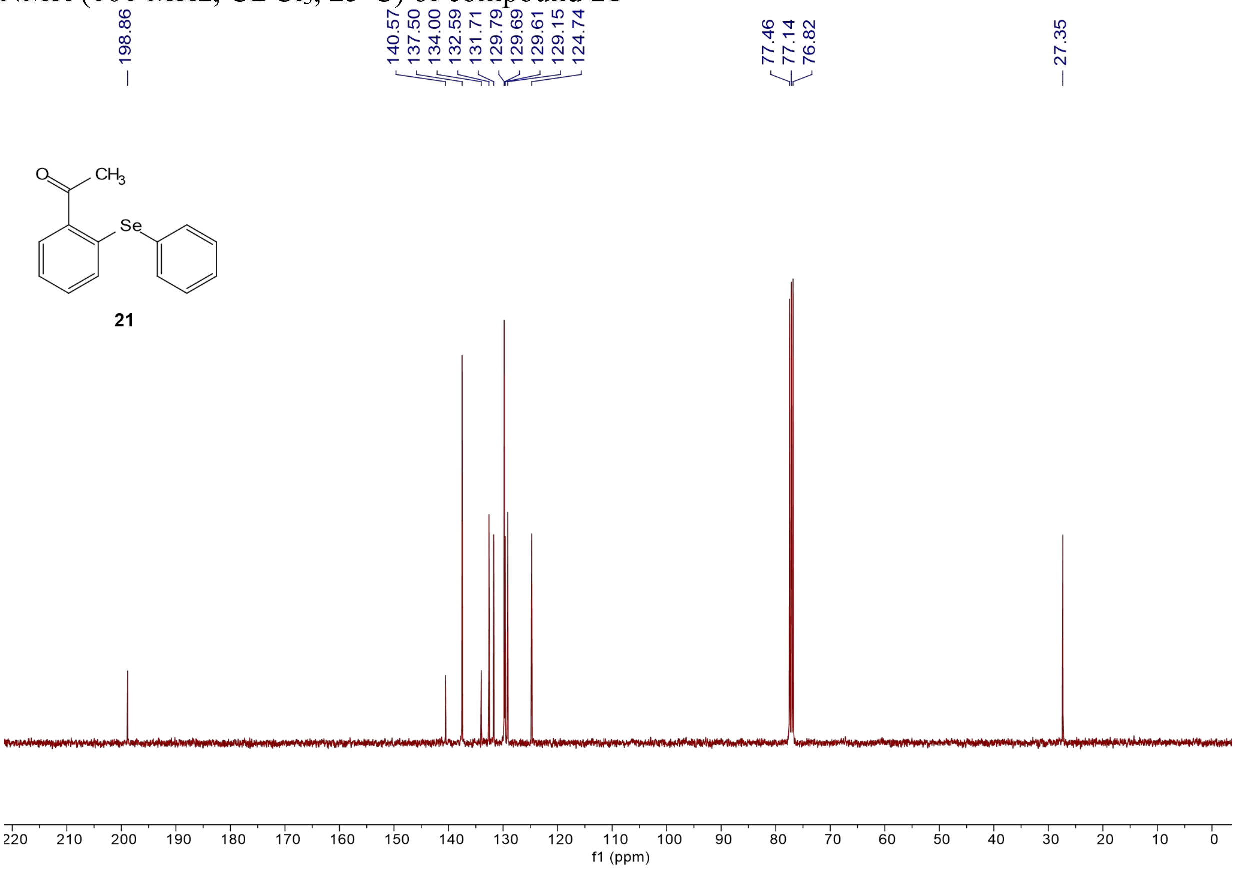

^{77}Se NMR (76 MHz, $CDCl_3$, 25°C) of compound **21**

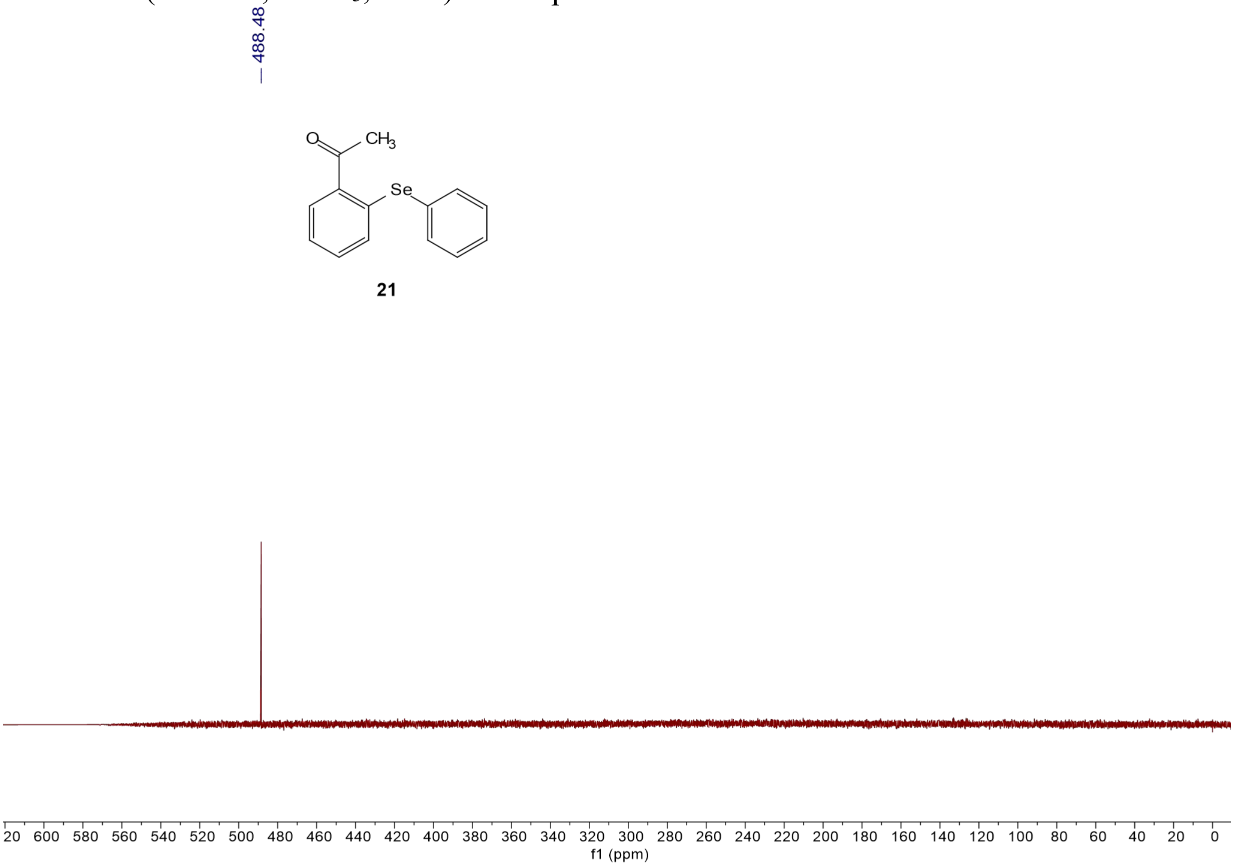

Supplementary Fig. 240. NMR of compound **21**

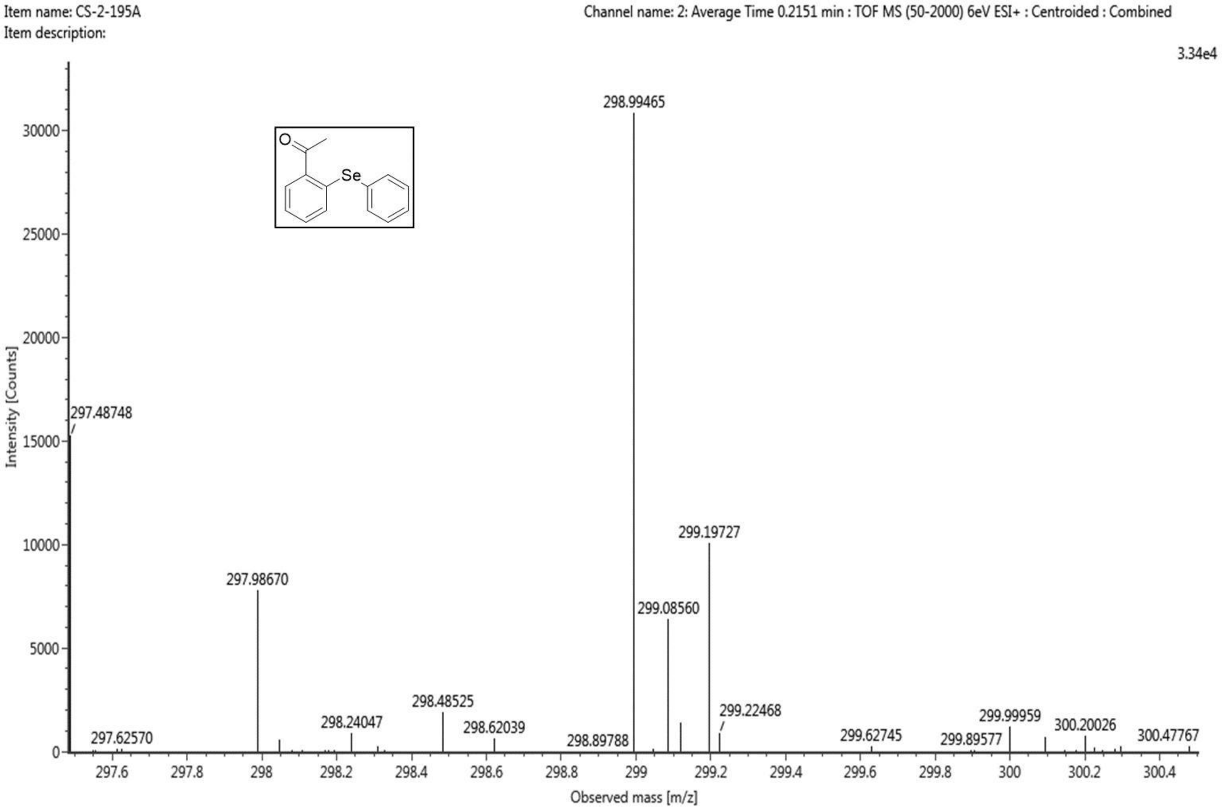

Supplementary Fig. 241. HR-MS of compound **21**

^{1}H NMR (400 MHz, $CDCl_3$, 25°C) of compound **23**

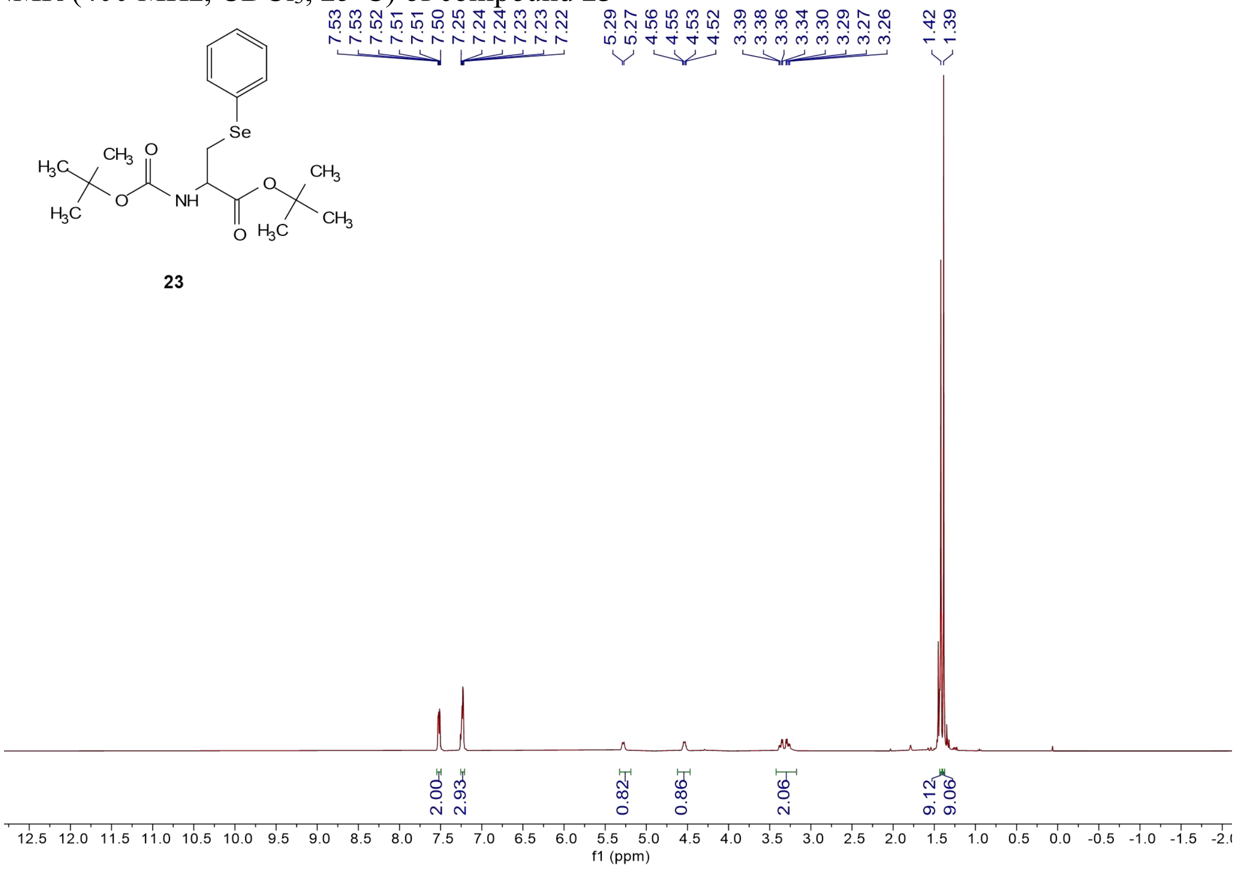

^{13}C NMR (101 MHz, $CDCl_3$, 25°C) of compound **23**

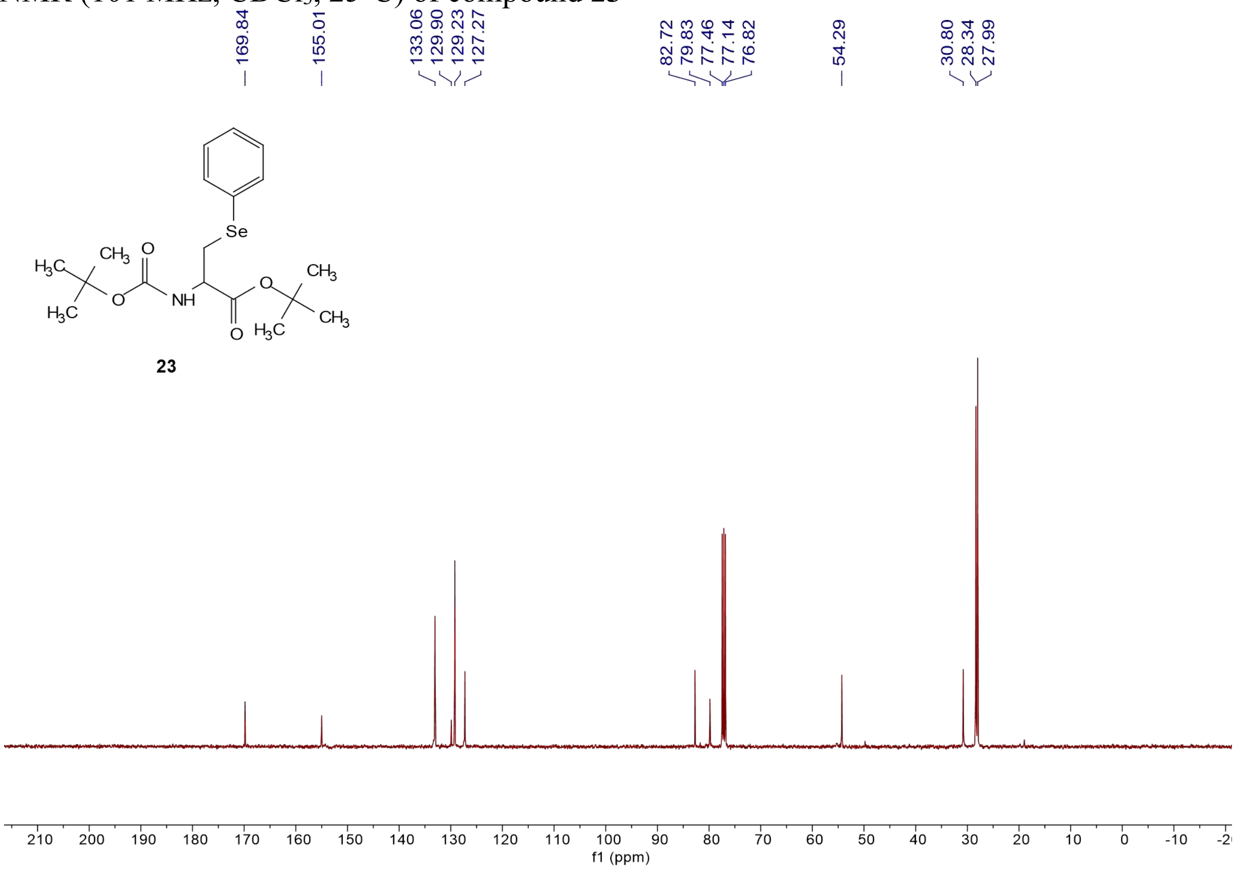

^{77}Se NMR (76 MHz, $CDCl_3$, 25°C) of compound **23**

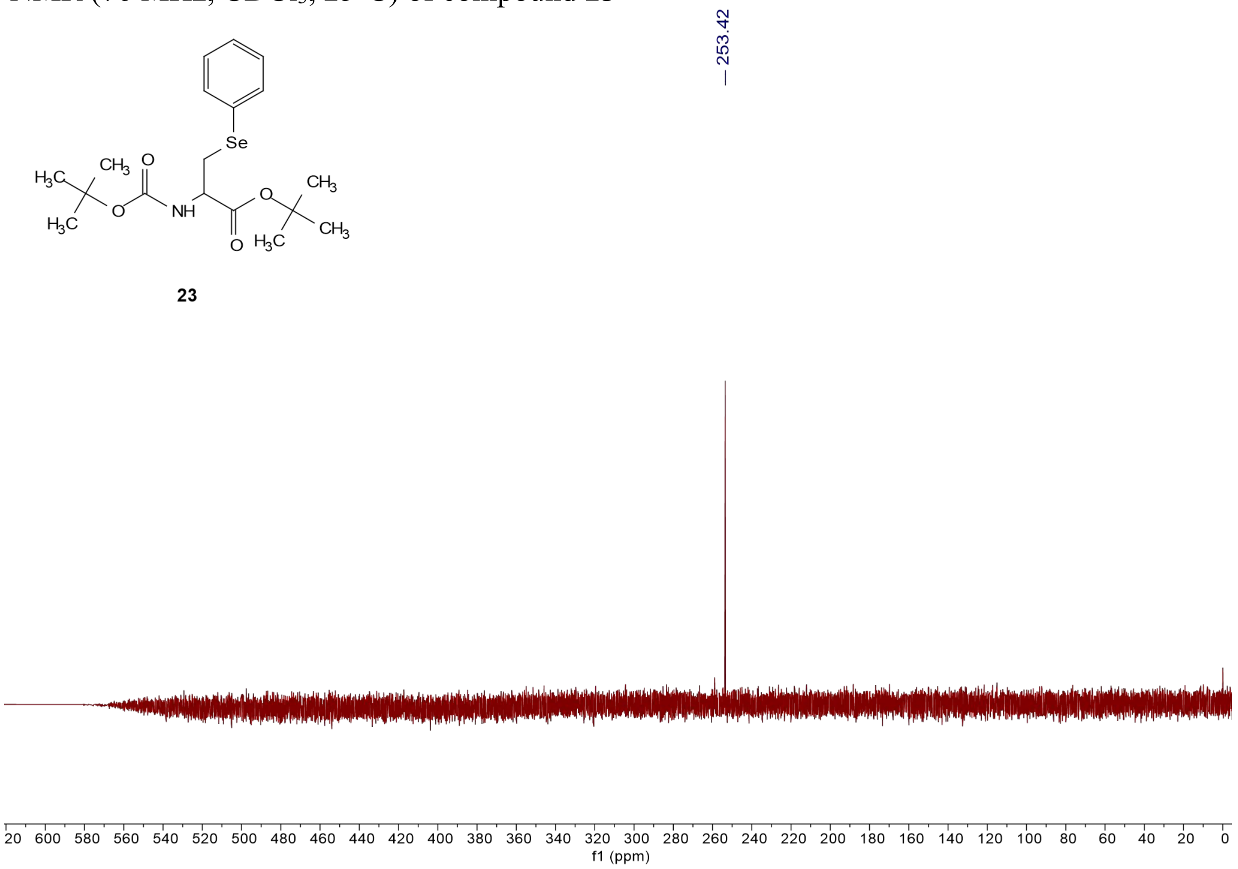

Supplementary Fig. 242. NMR of compound **23**

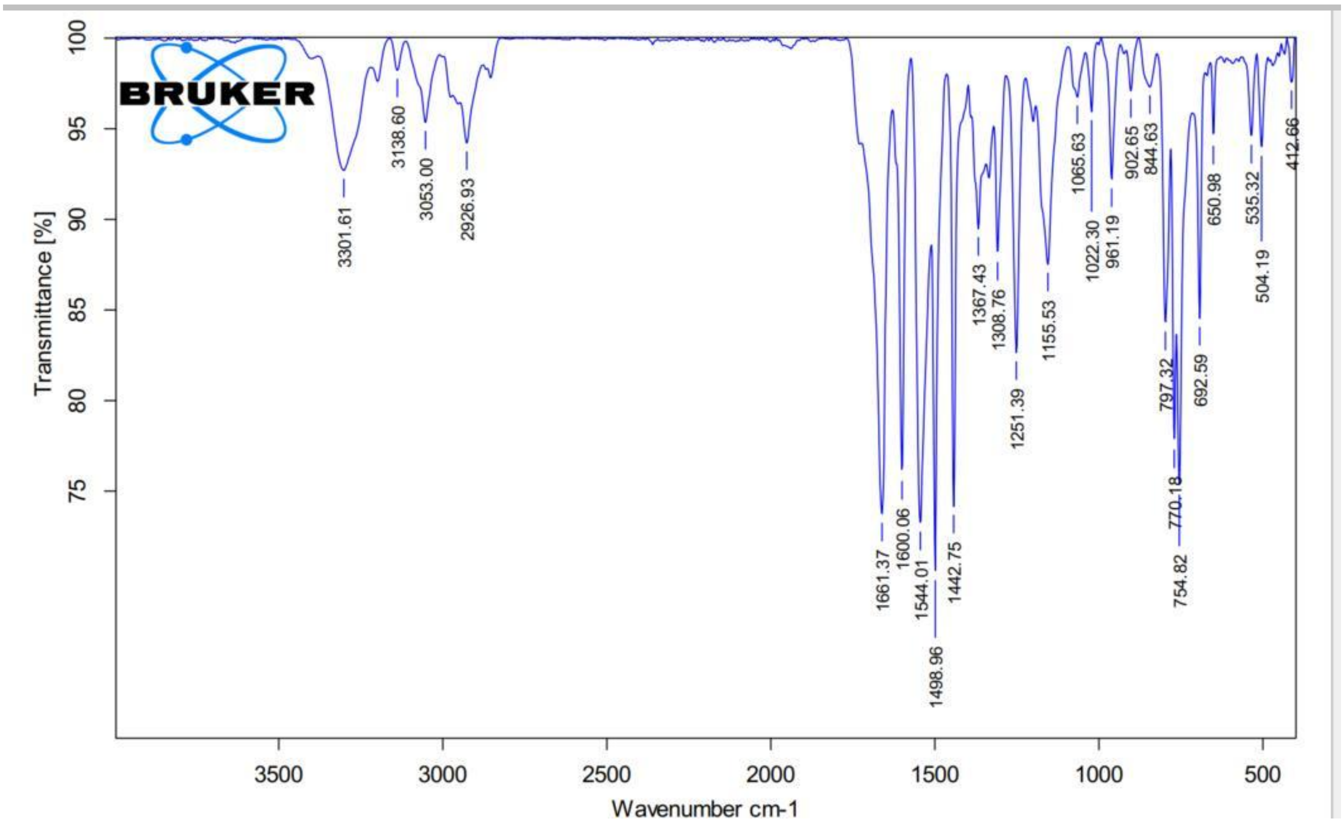

Supplementary Fig. 243. IR of compound **23**

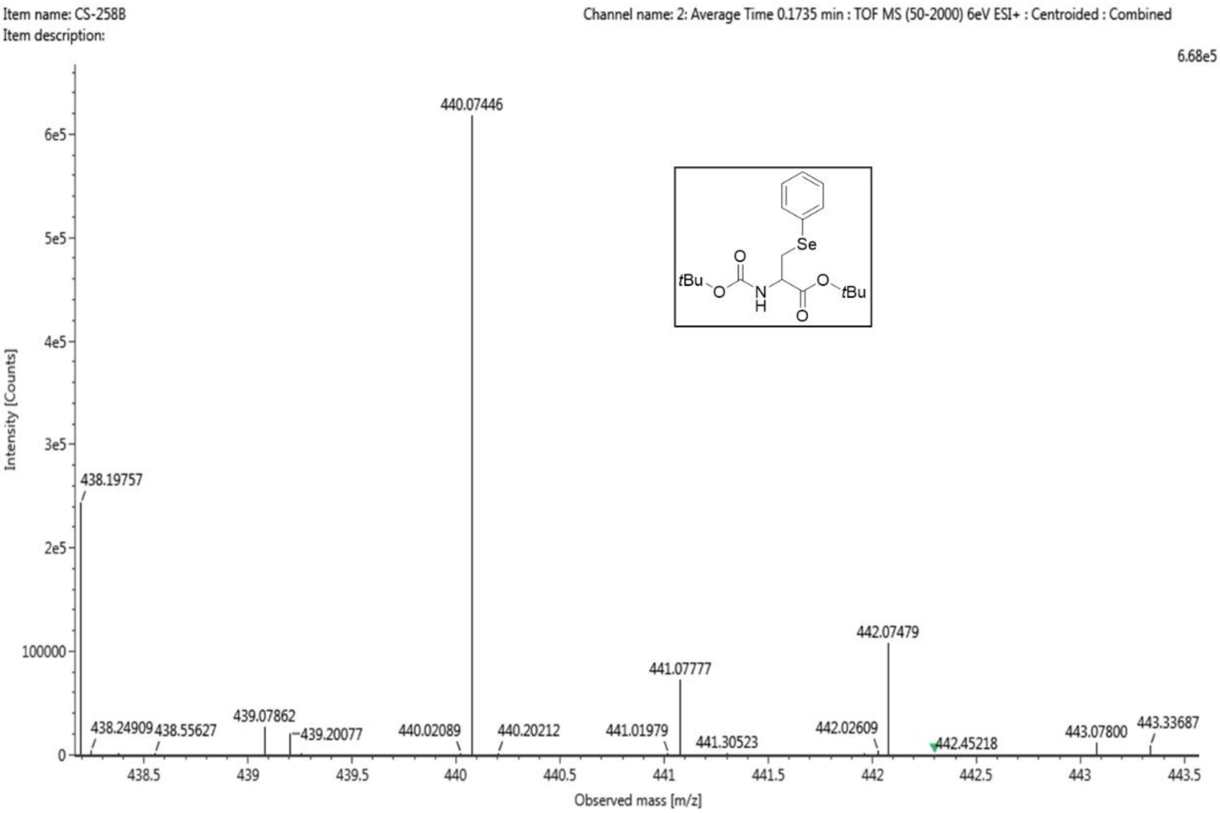

Supplementary Fig. 244. HR-MS of compound **23**

^{1}H NMR (400 MHz, $CDCl_3$, 25°C) of compound **25**

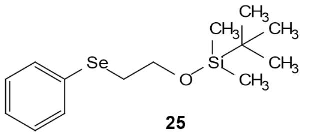

25

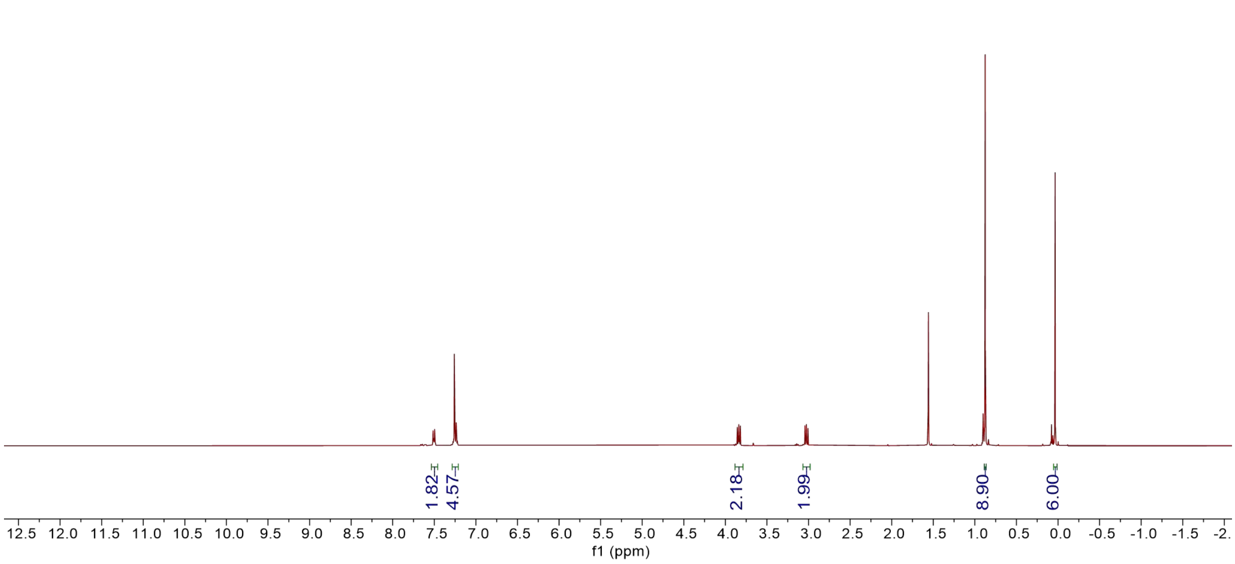

^{13}C NMR (101 MHz, $CDCl_3$, 25°C) of compound **25**

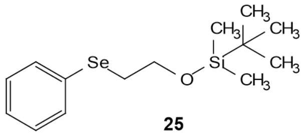

25

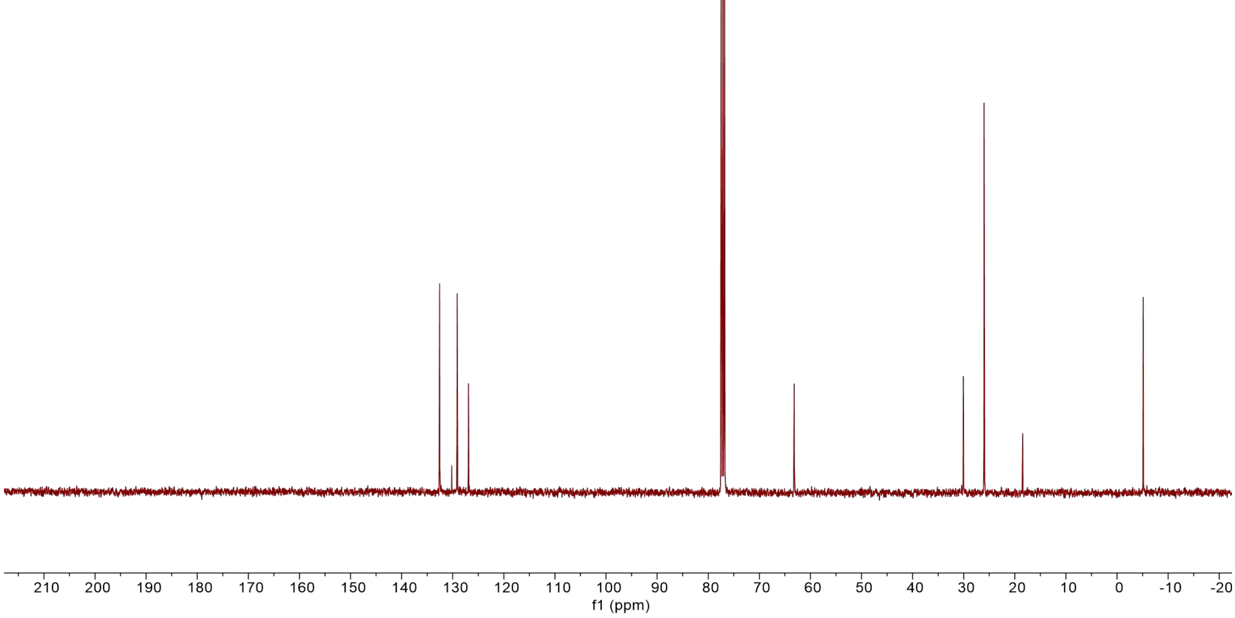

^{77}Se NMR (76 MHz, $CDCl_3$, 25°C) of compound **25**

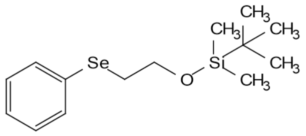

25

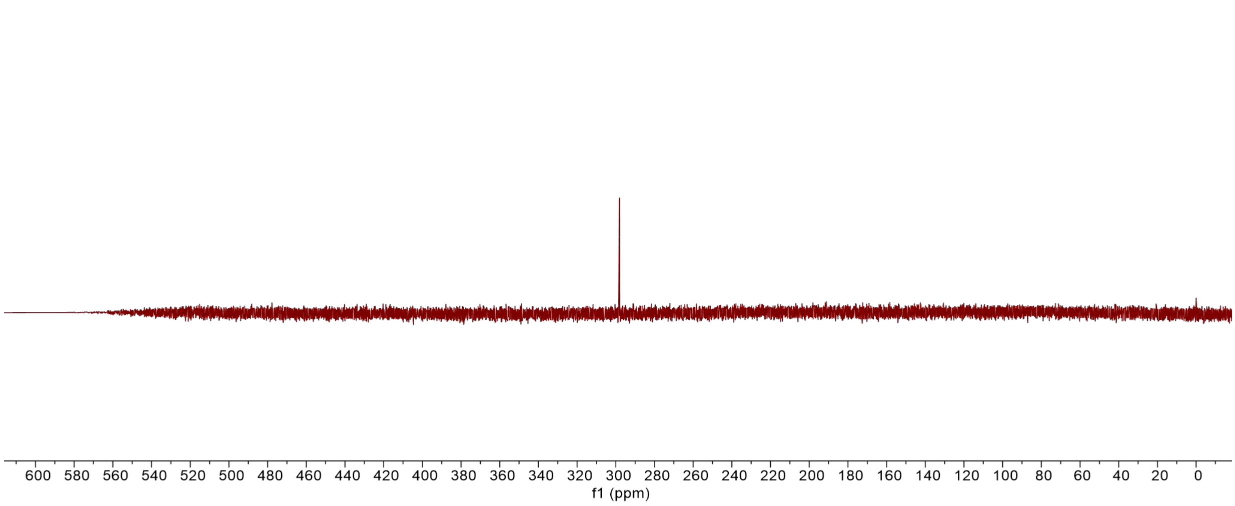

Supplementary Fig. 245. NMR of compound **25**

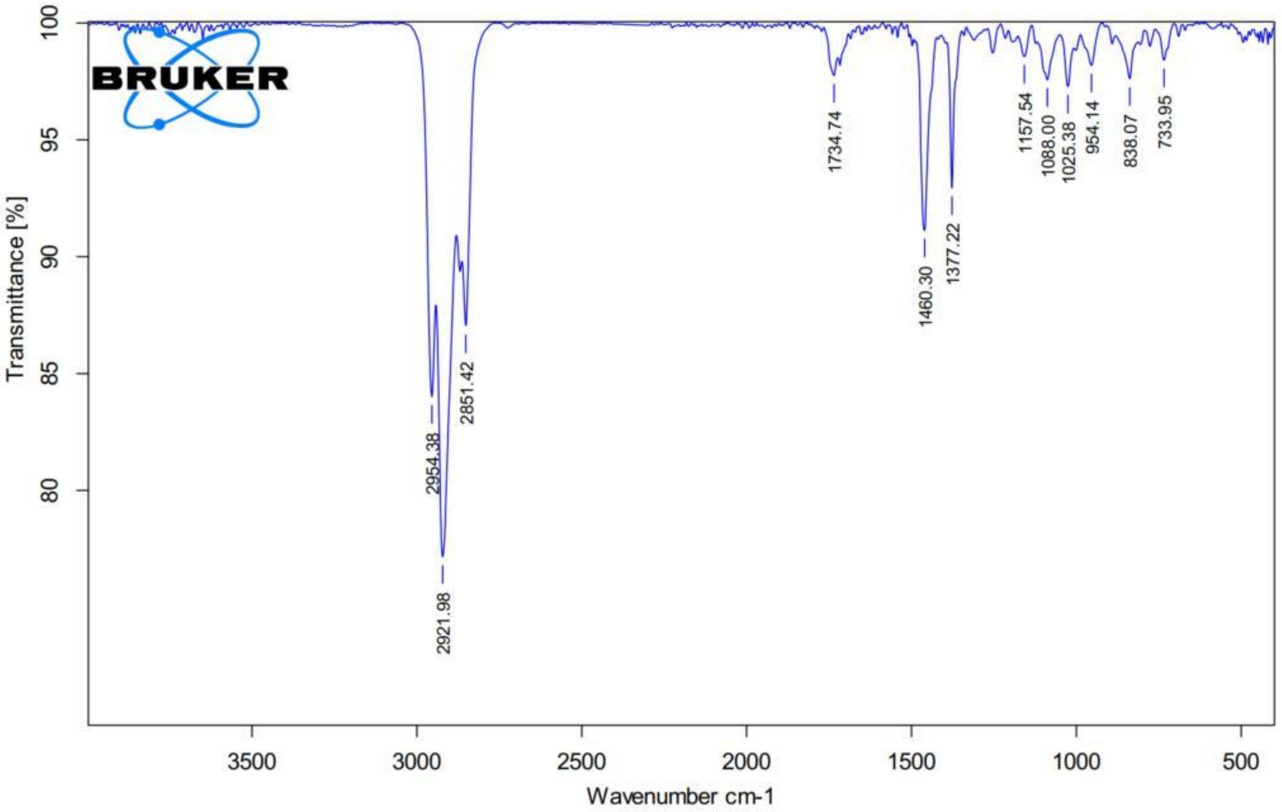

Supplementary Fig. 246. IR of compound **25**

^{1}H NMR (400 MHz, $CDCl_3$, 25°C) of compound **26**

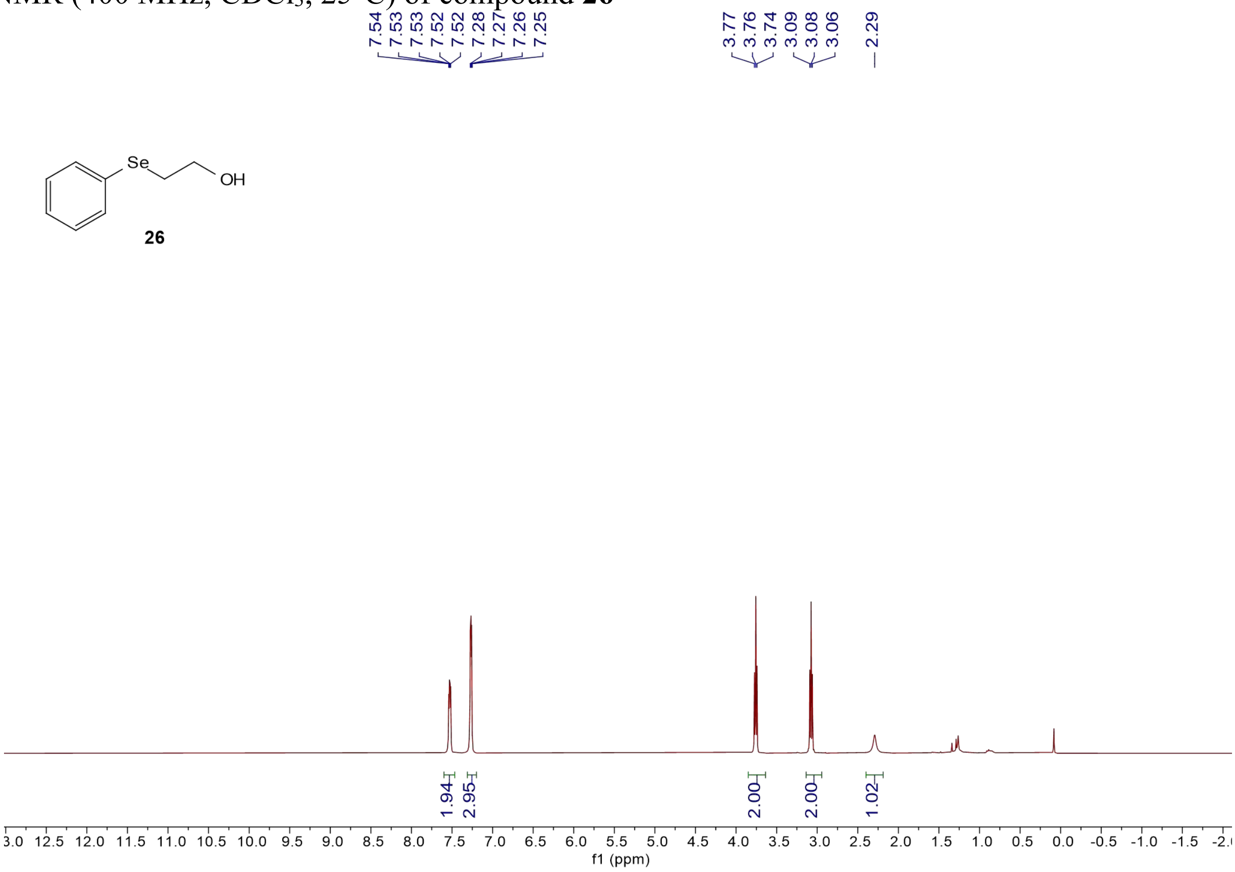

^{13}C NMR (101 MHz, $CDCl_3$, 25°C) of compound **26**

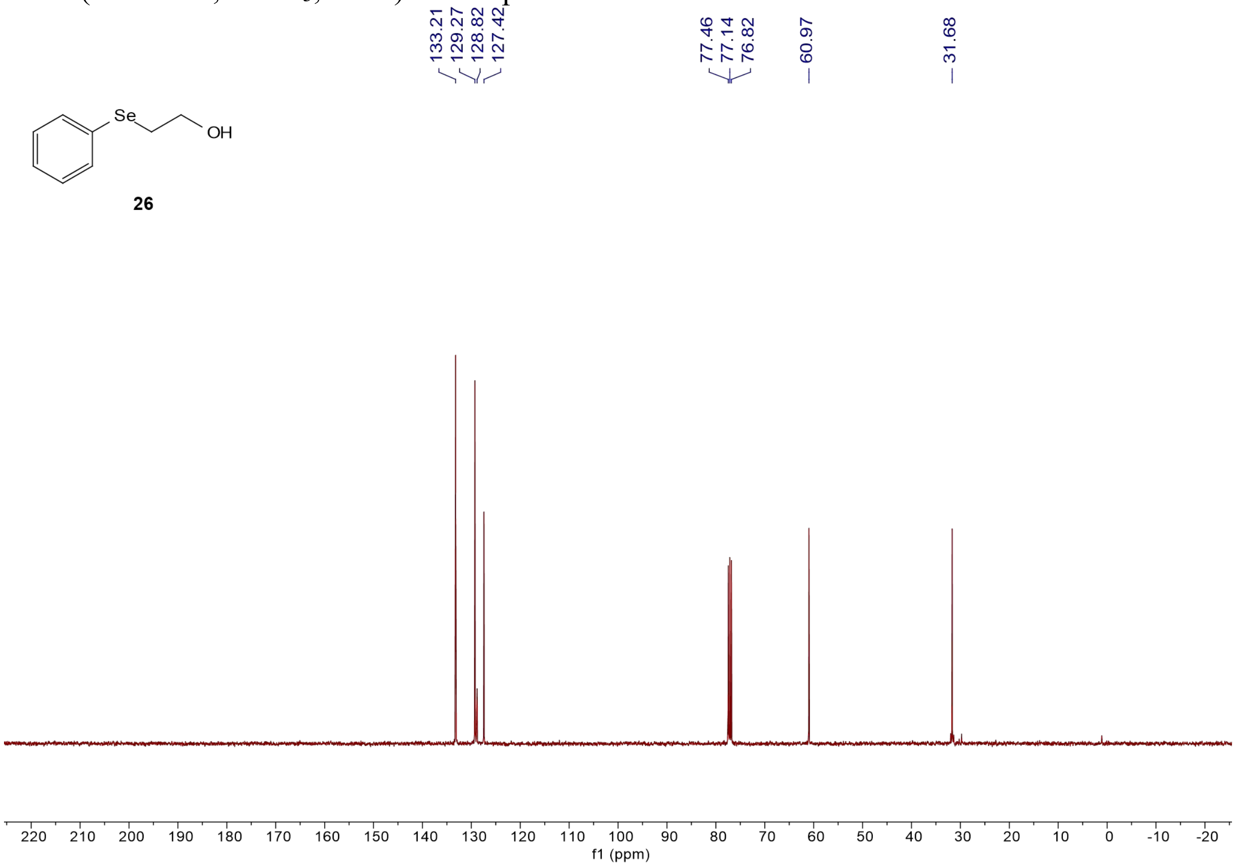

^{77}Se NMR (76 MHz, $CDCl_3$, 25°C) of compound **26**

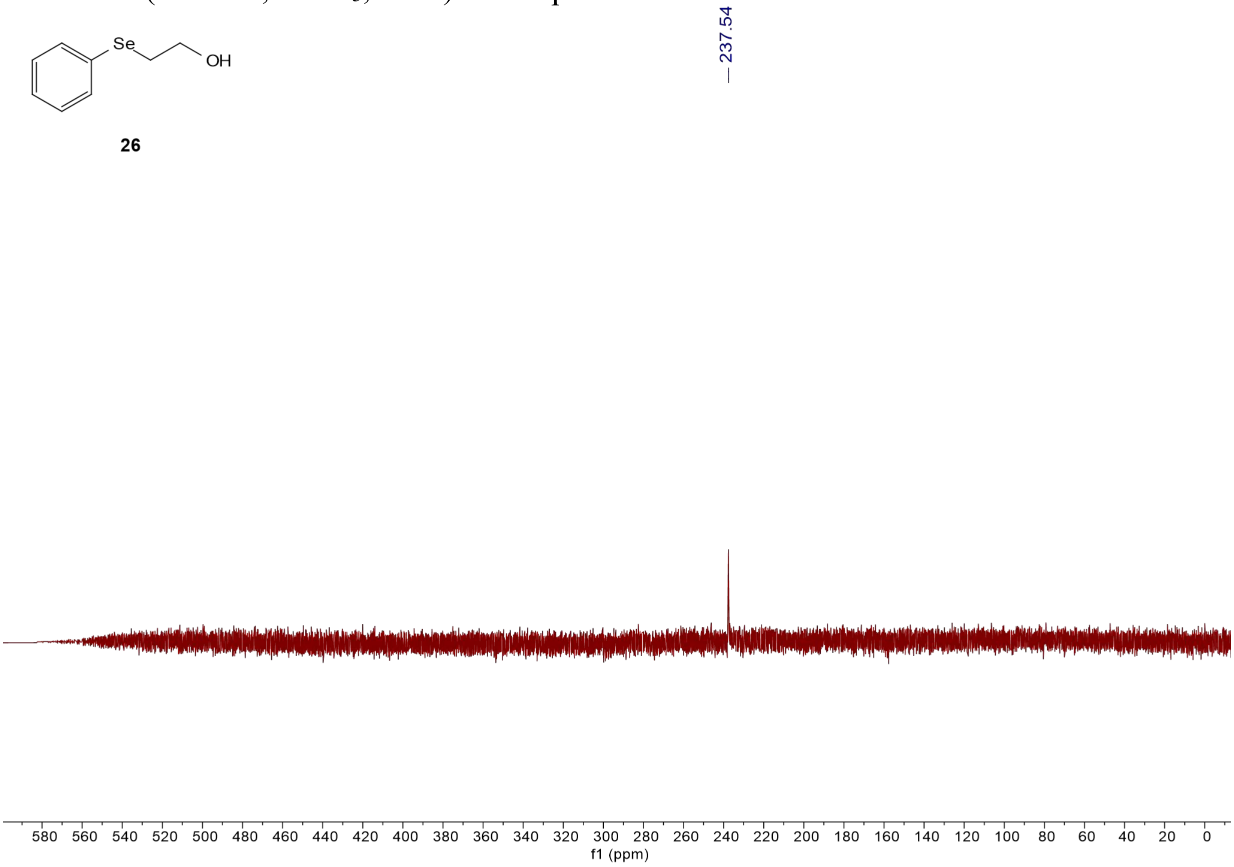

Supplementary Fig. 247. NMR of compound **26**

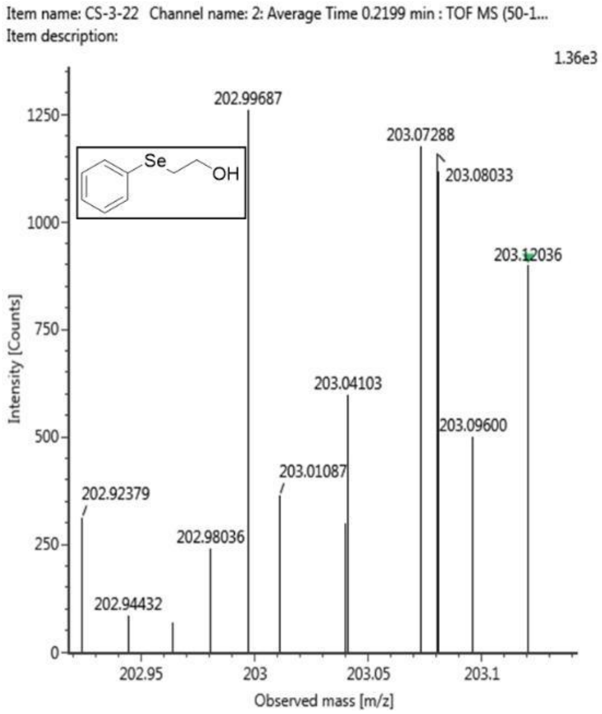

Supplementary Fig. 248. HR-MS of compound **26**

^{1}H NMR (400 MHz, $CDCl_3$, 25°C) of compound **28**

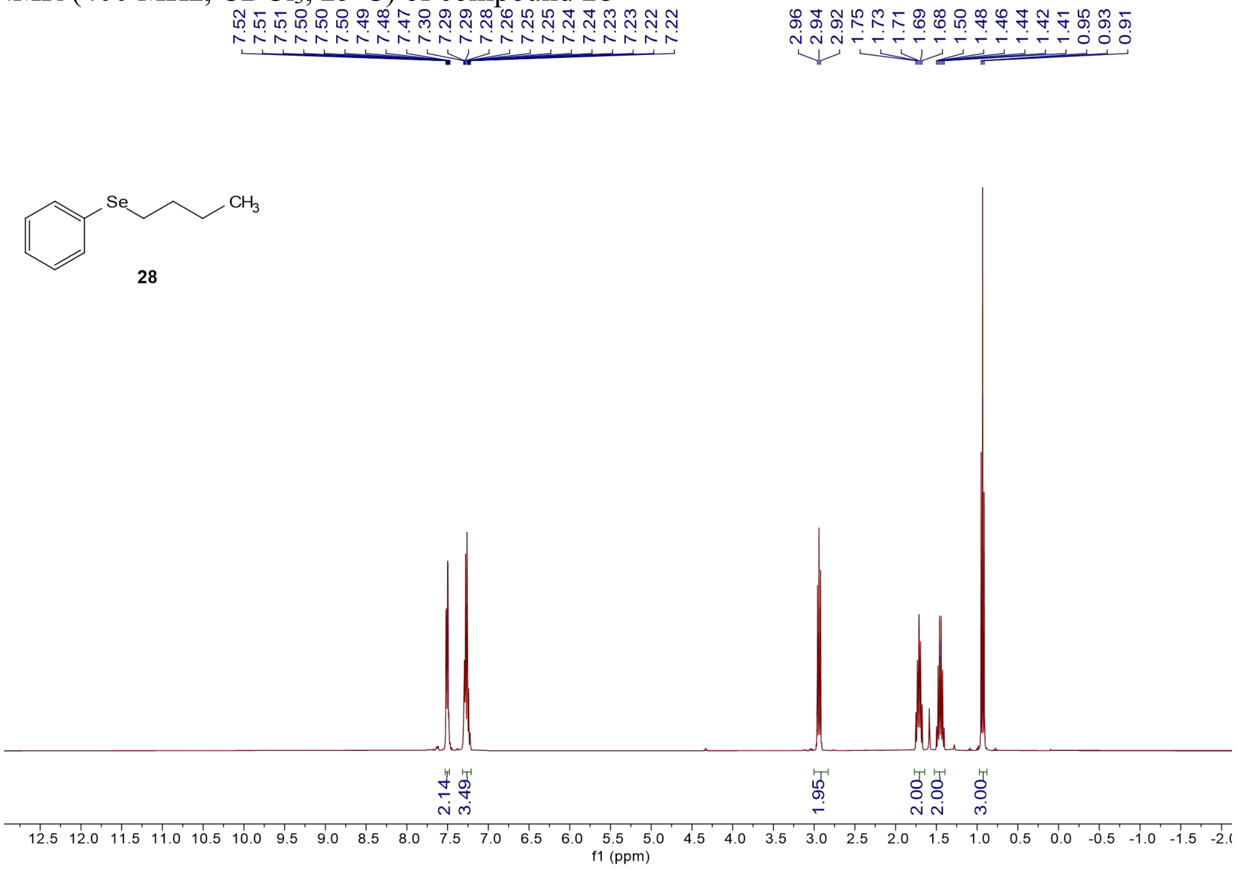

^{13}C NMR (101 MHz, $CDCl_3$, 25°C) of compound **28**

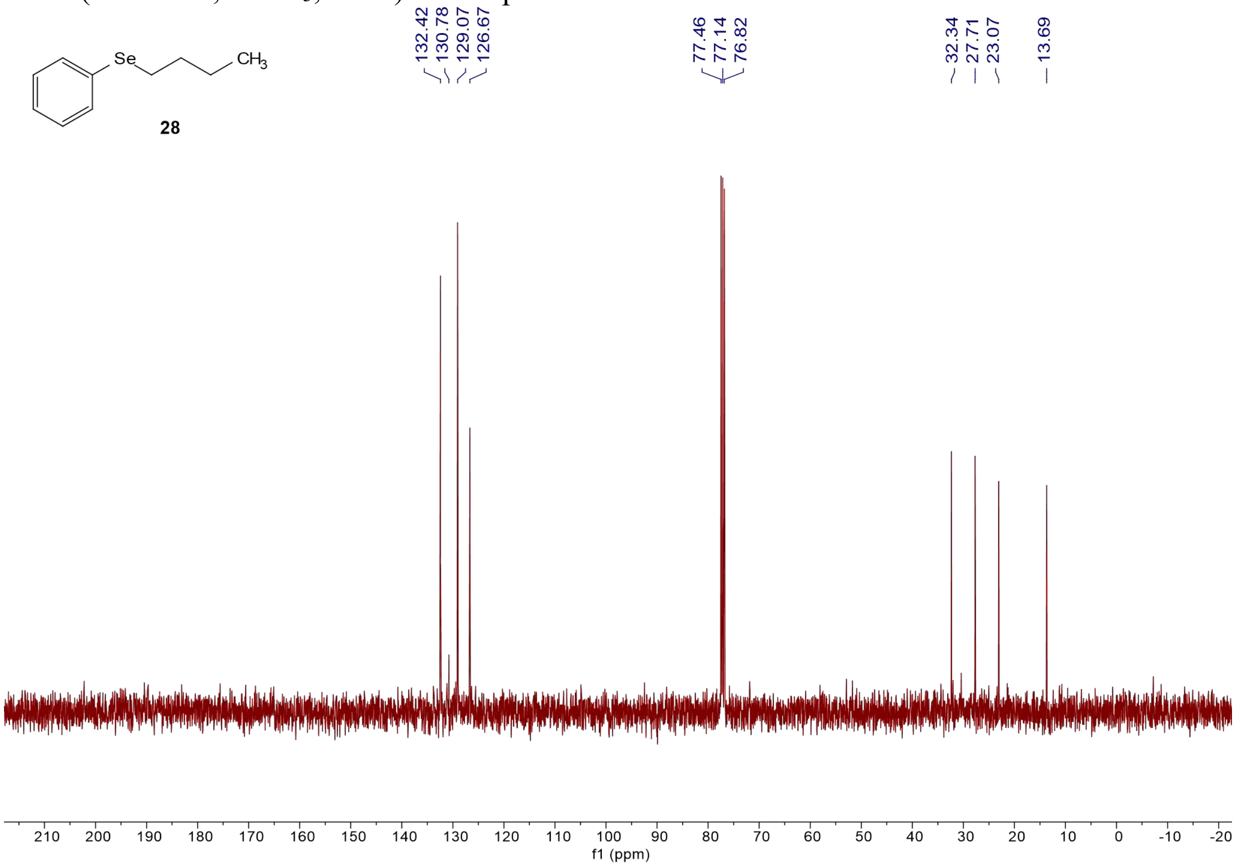

^{77}Se NMR (76 MHz, $CDCl_3$, 25°C) of compound **28**

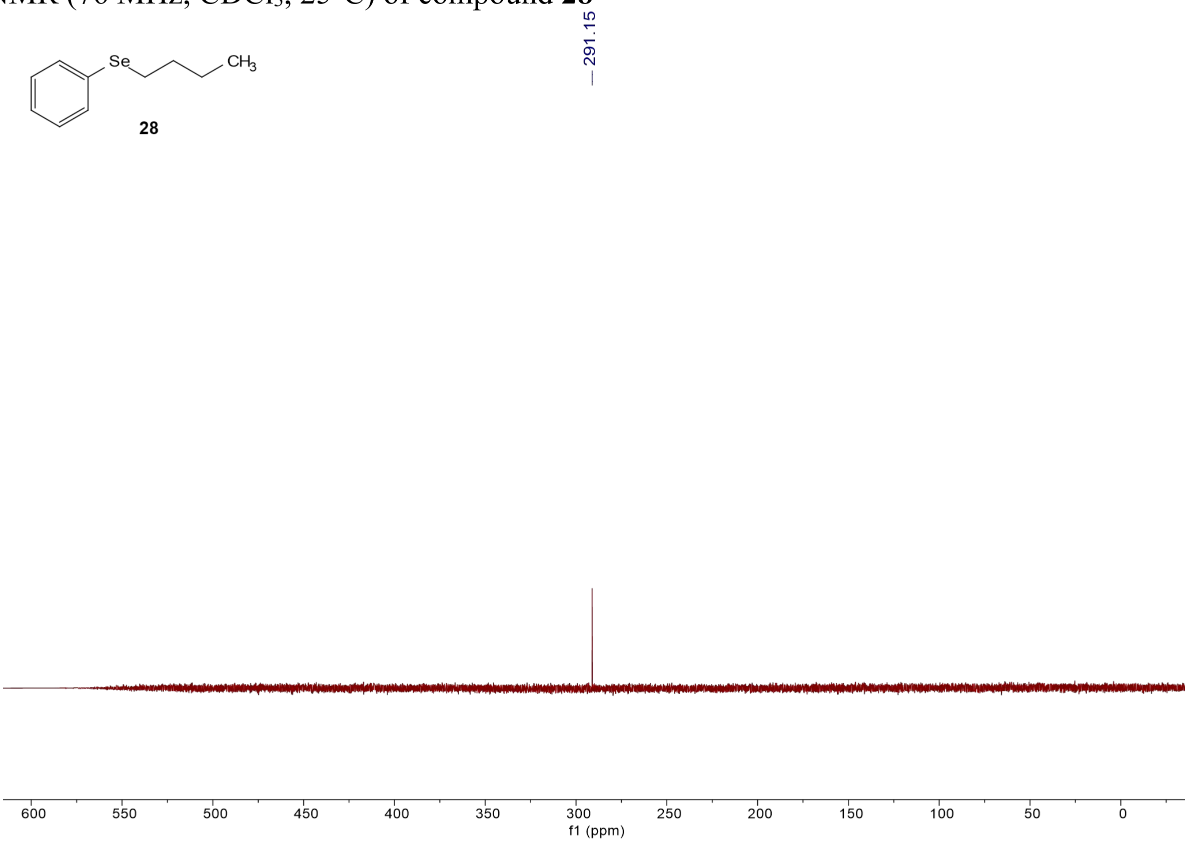

Supplementary Fig. 249. NMR of compound **28**

^{1}H NMR (400 MHz, $CDCl_3$, 25°C) of compound **30**

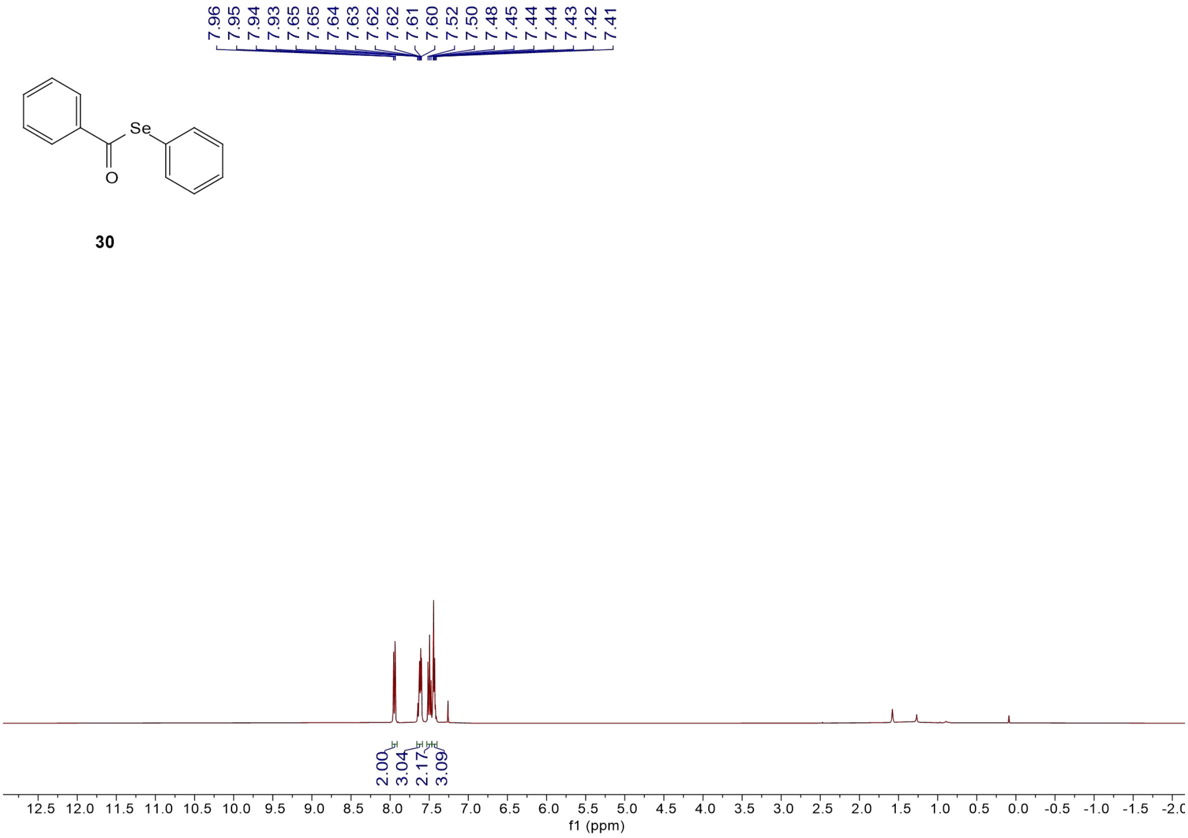

^{13}C NMR (101 MHz, $CDCl_3$, 25°C) of compound **30**

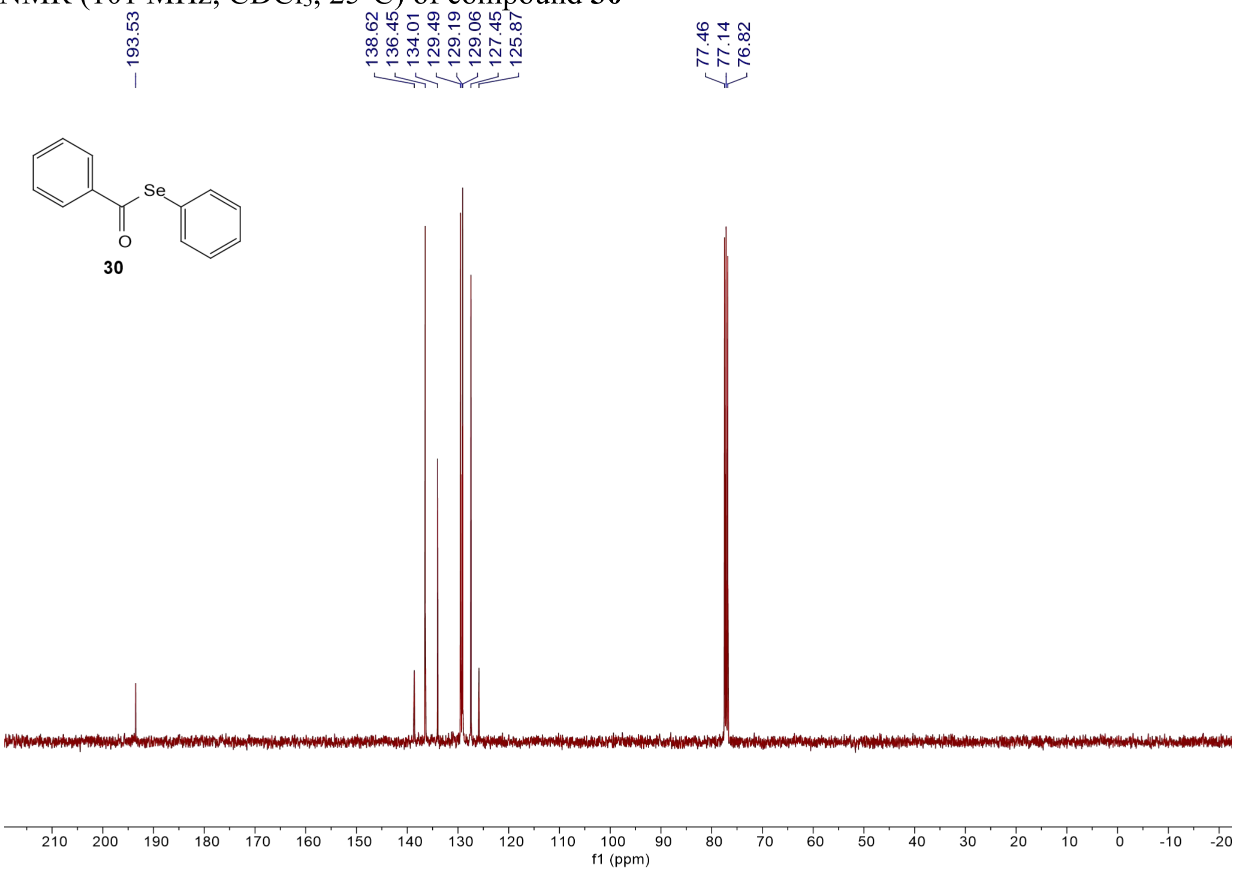

^{77}Se NMR (76 MHz, $CDCl_3$, 25°C) of compound **30**

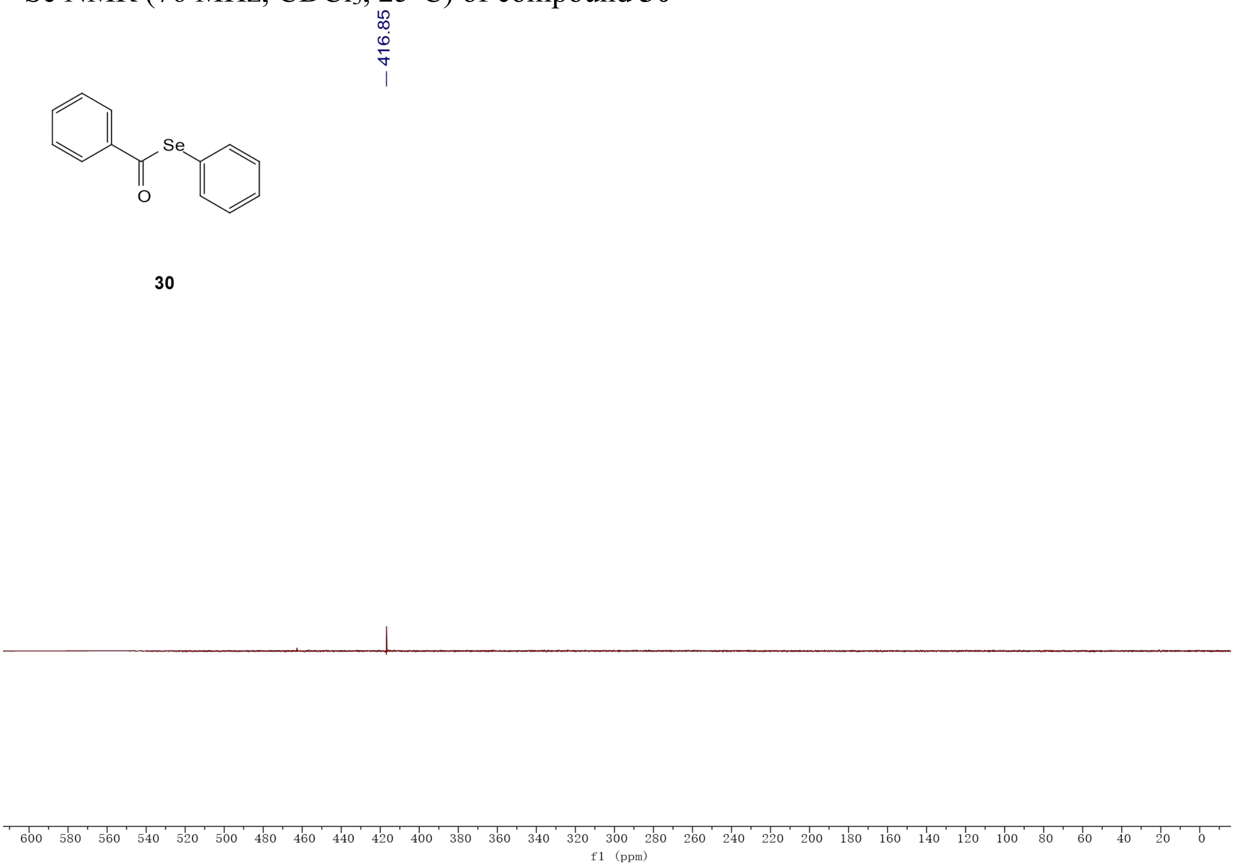

Supplementary Fig. 250. NMR of compound **30**

^1H NMR (400 MHz, $CDCl_3$, 25°C) of compound **32**

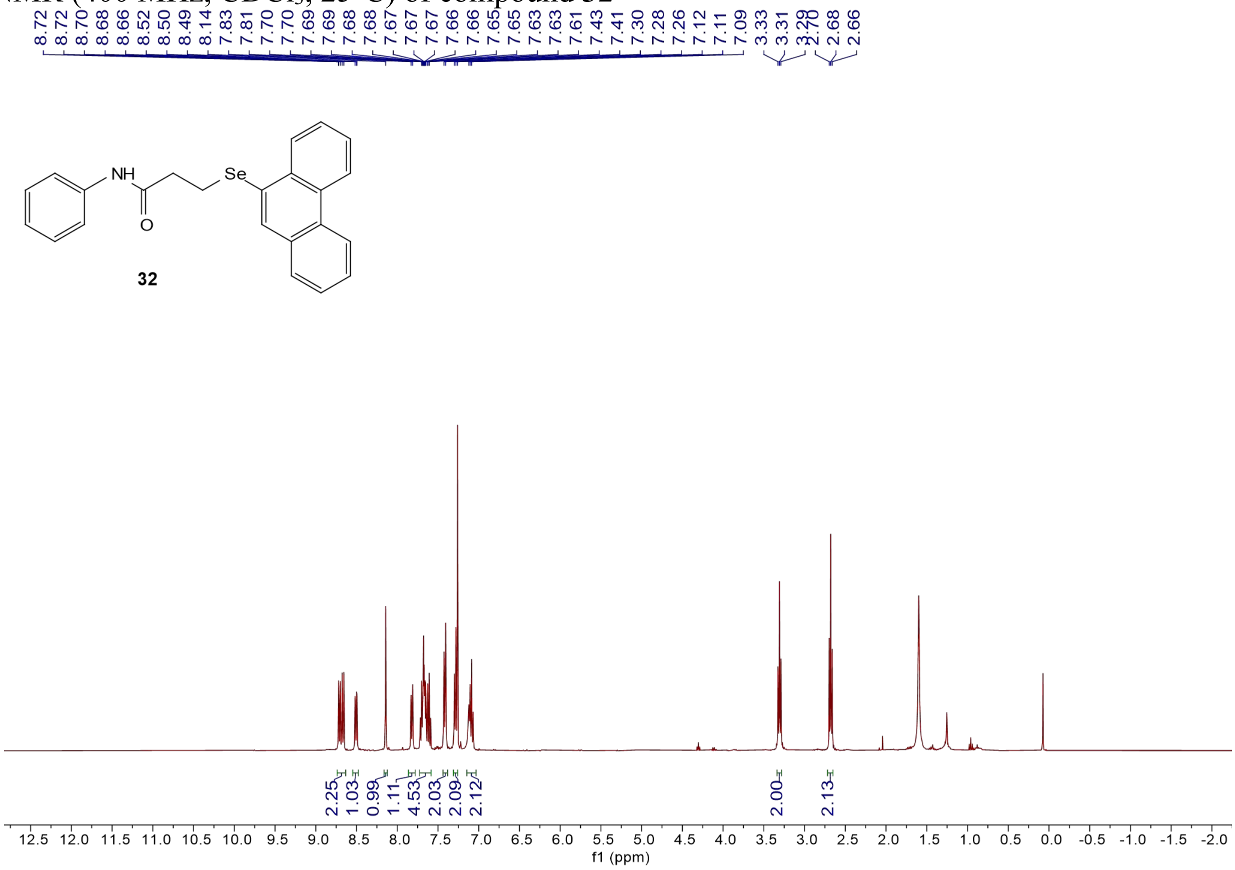

^{13}C NMR (101 MHz, $CDCl_3$, 25°C) of compound **32**

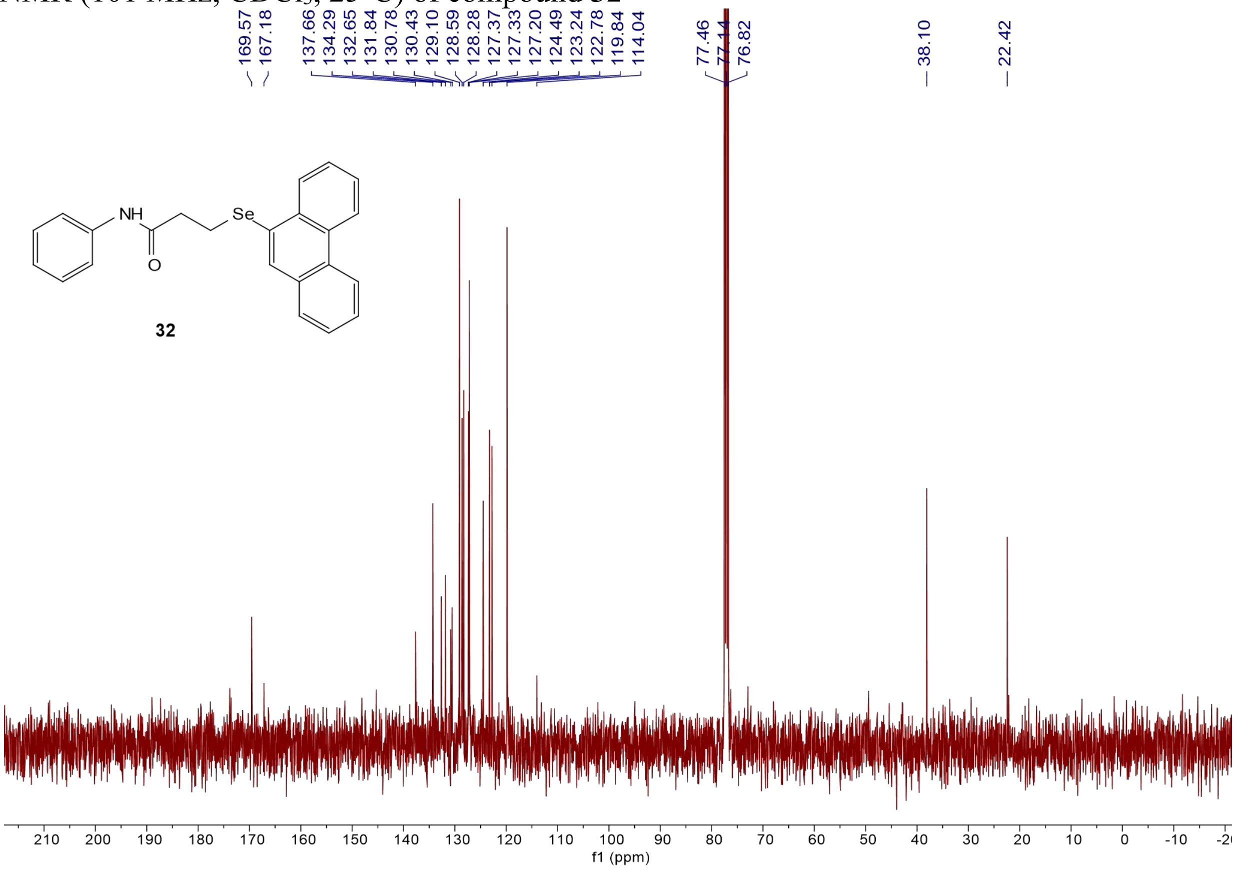

^{77}Se NMR (76 MHz, $CDCl_3$, 25°C) of compound **32**

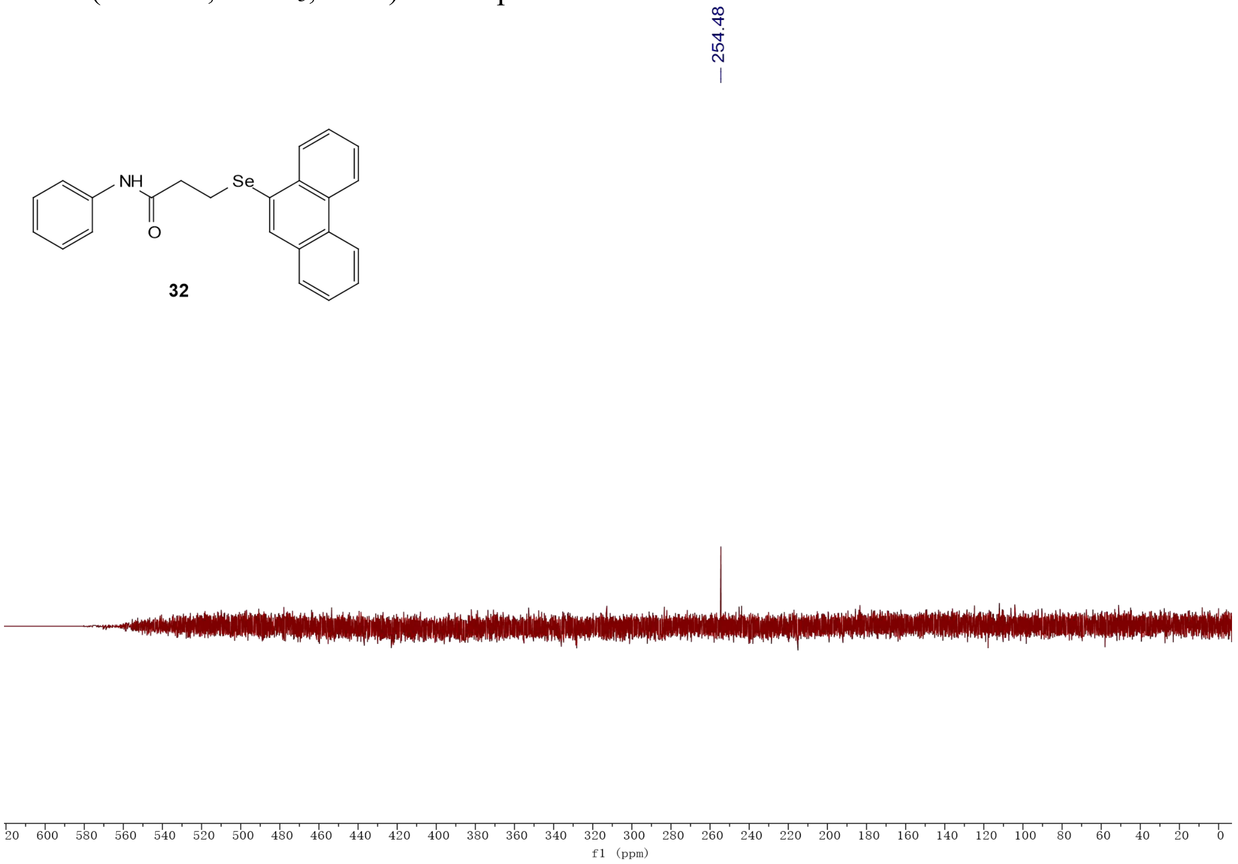

Supplementary Fig. 251. NMR of compound **32**

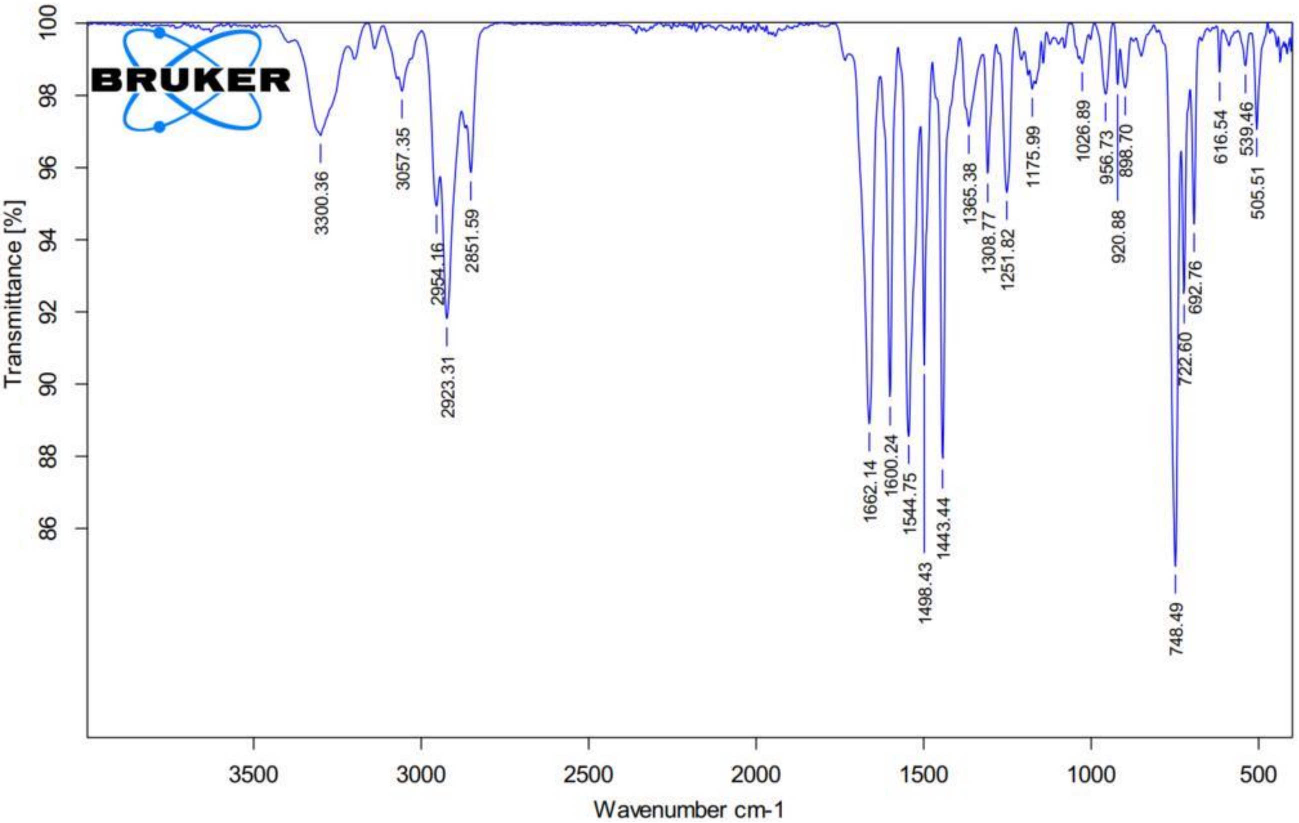

Supplementary Fig. 252. IR of compound **32**

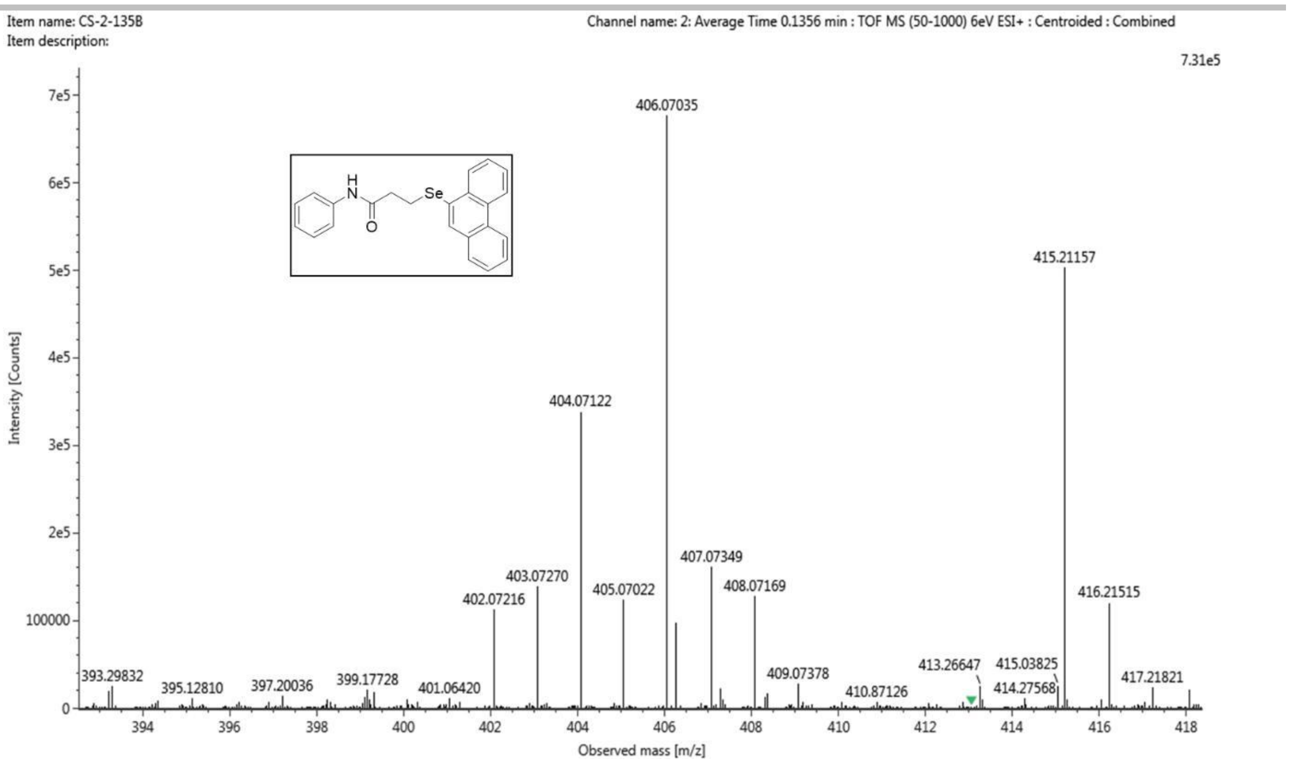

Supplementary Fig. 253. HR-MS of compound **32**

^{1}H NMR (400 MHz, DMSO-d$_6$, 25°C) of compound **34**

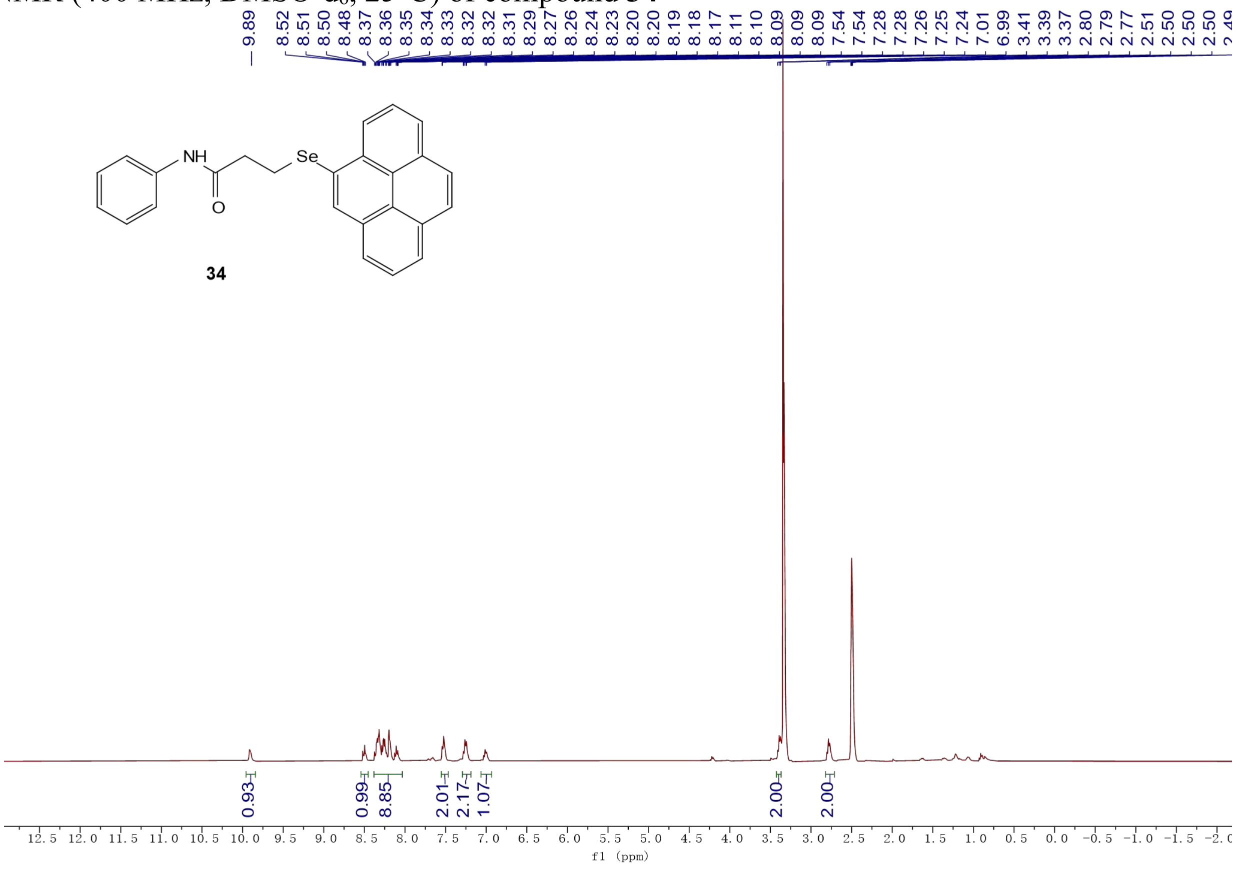

^{13}C NMR (101 MHz, DMSO-d_6, 25°C) of compound **34**

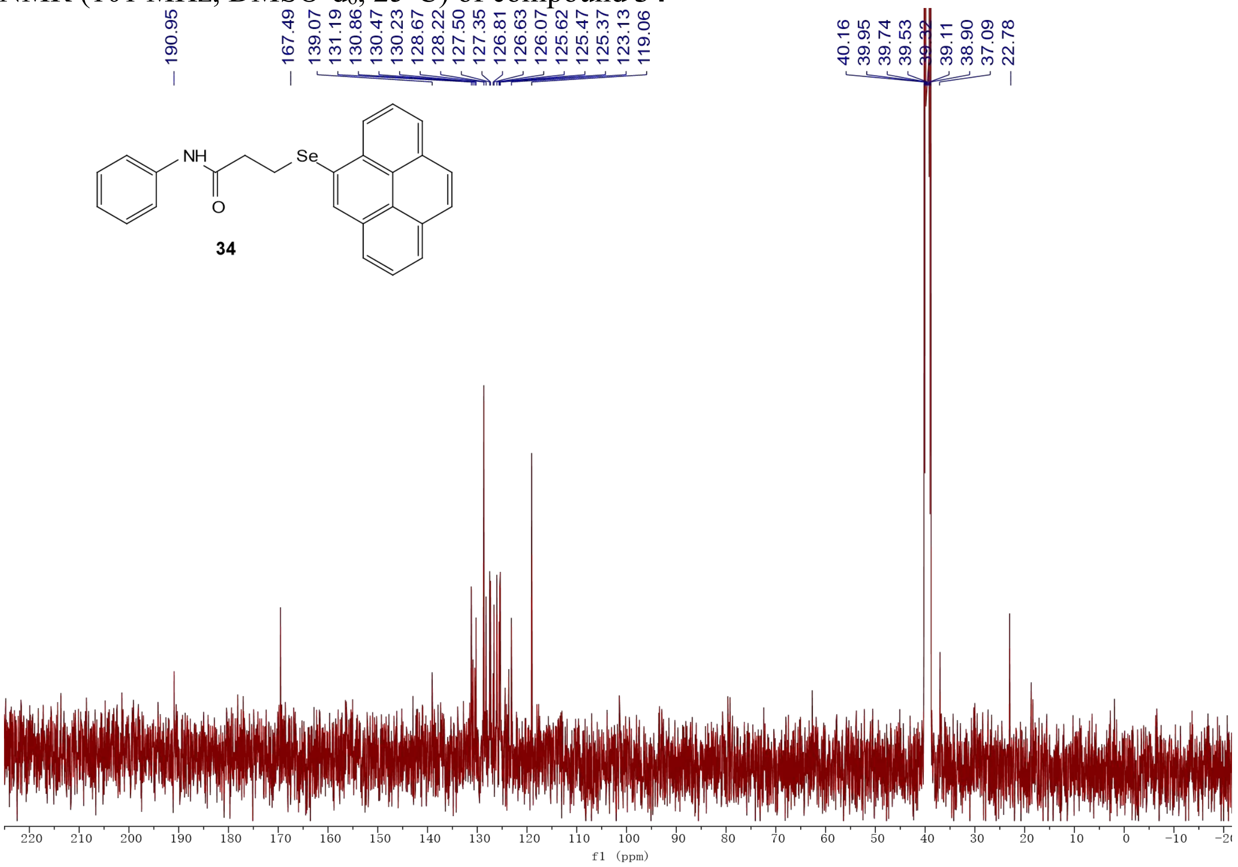

^{77}Se NMR (76 MHz, DMSO-d_6, 25°C) of compound **34**

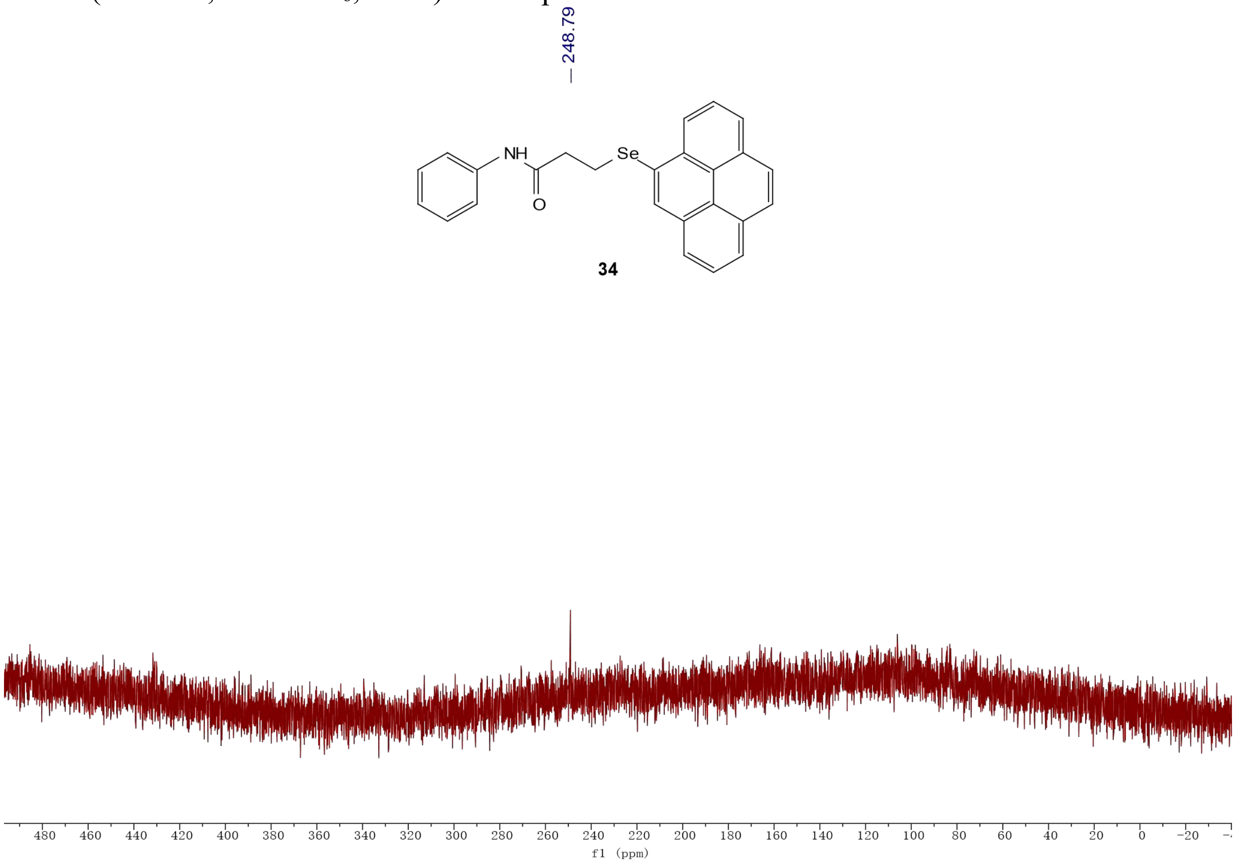

Supplementary Fig. 254. NMR of compound **34**

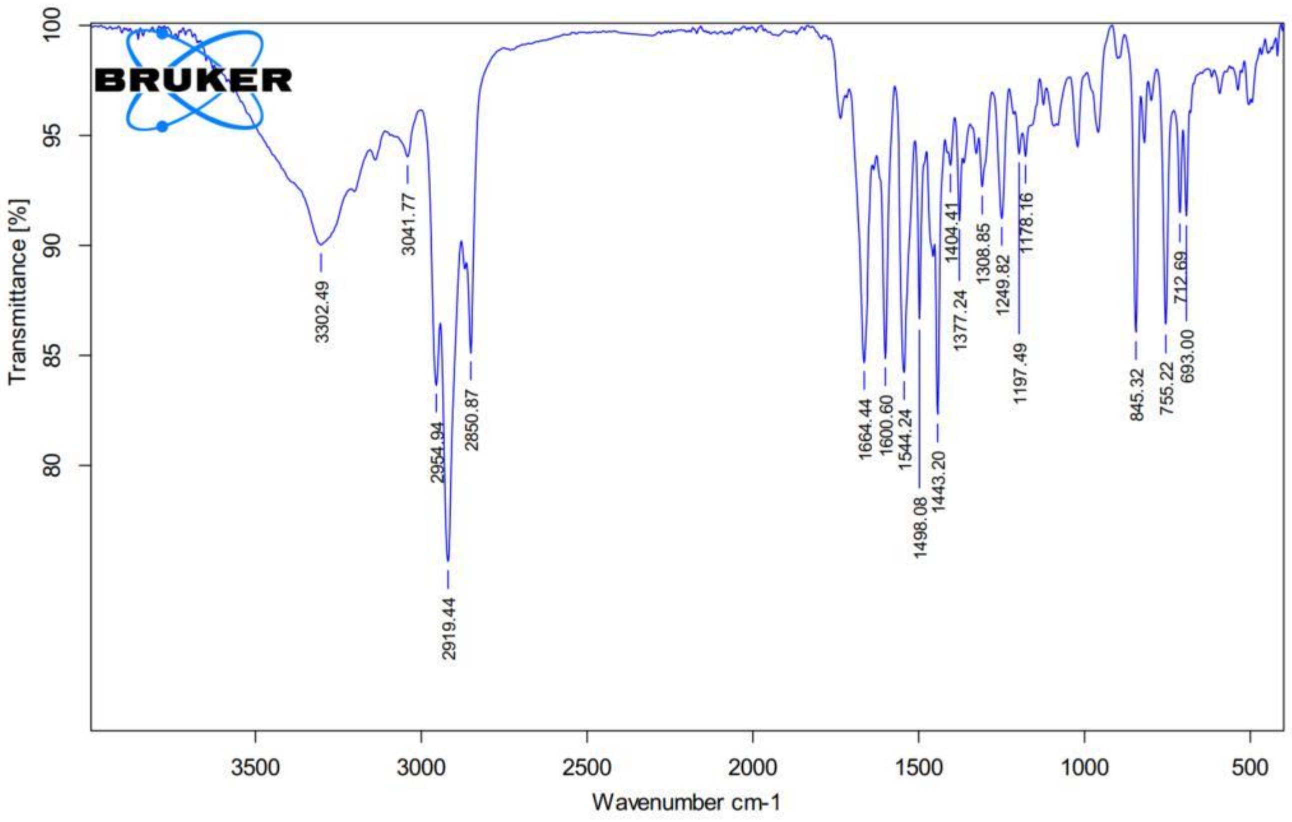

Supplementary Fig. 255. IR of compound **34**

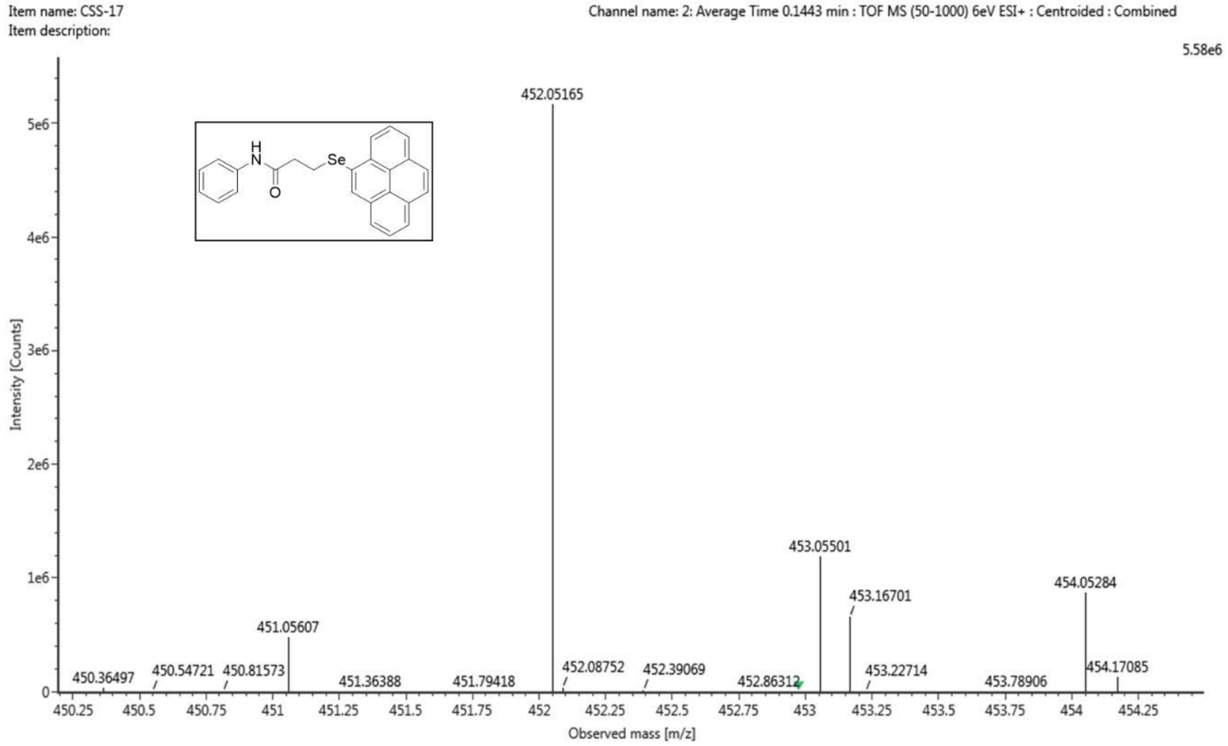

Supplementary Fig. 256. HR-MS of compound **34**

^{1}H NMR (400 MHz, $CDCl_3$, 25°C) of compound **36**

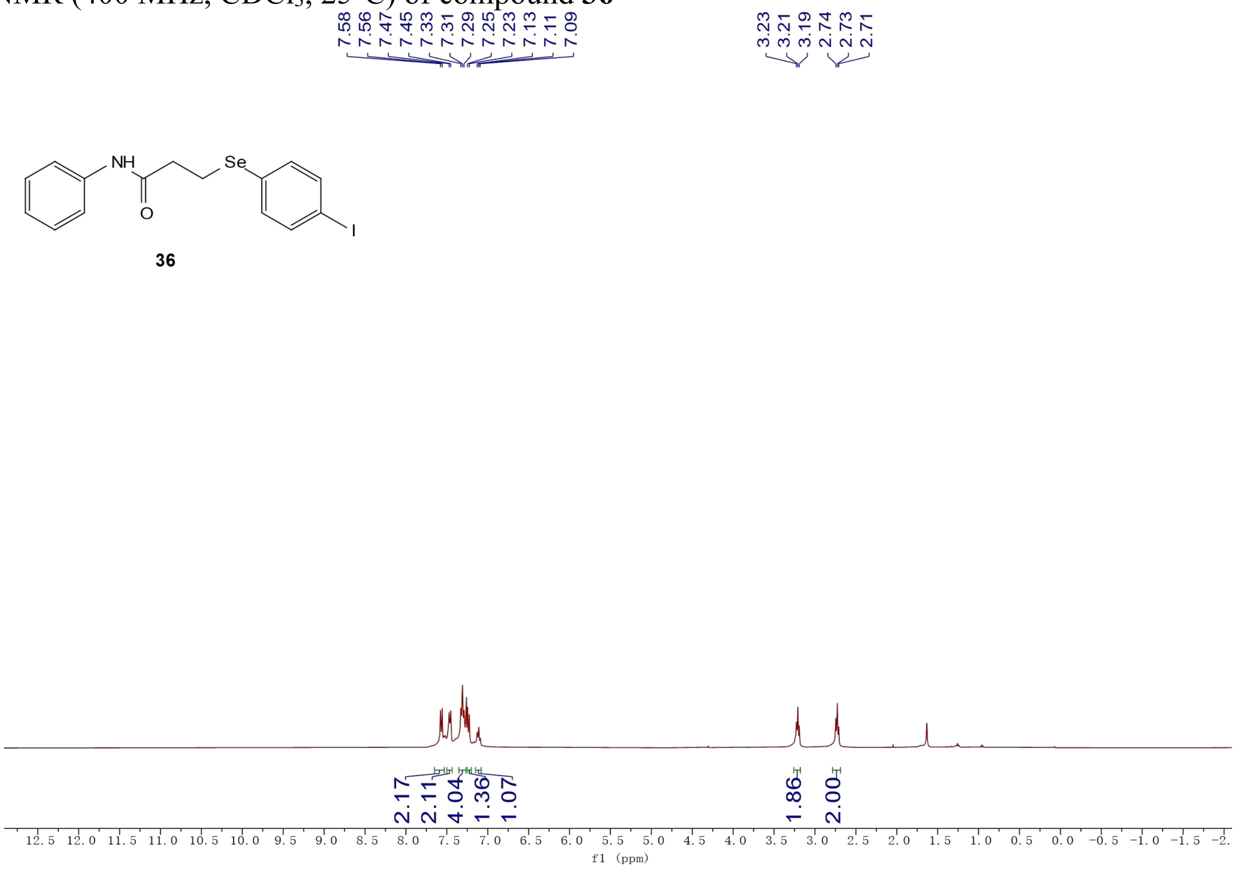

^{13}C NMR (101 MHz, $CDCl_3$, 25°C) of compound **36**

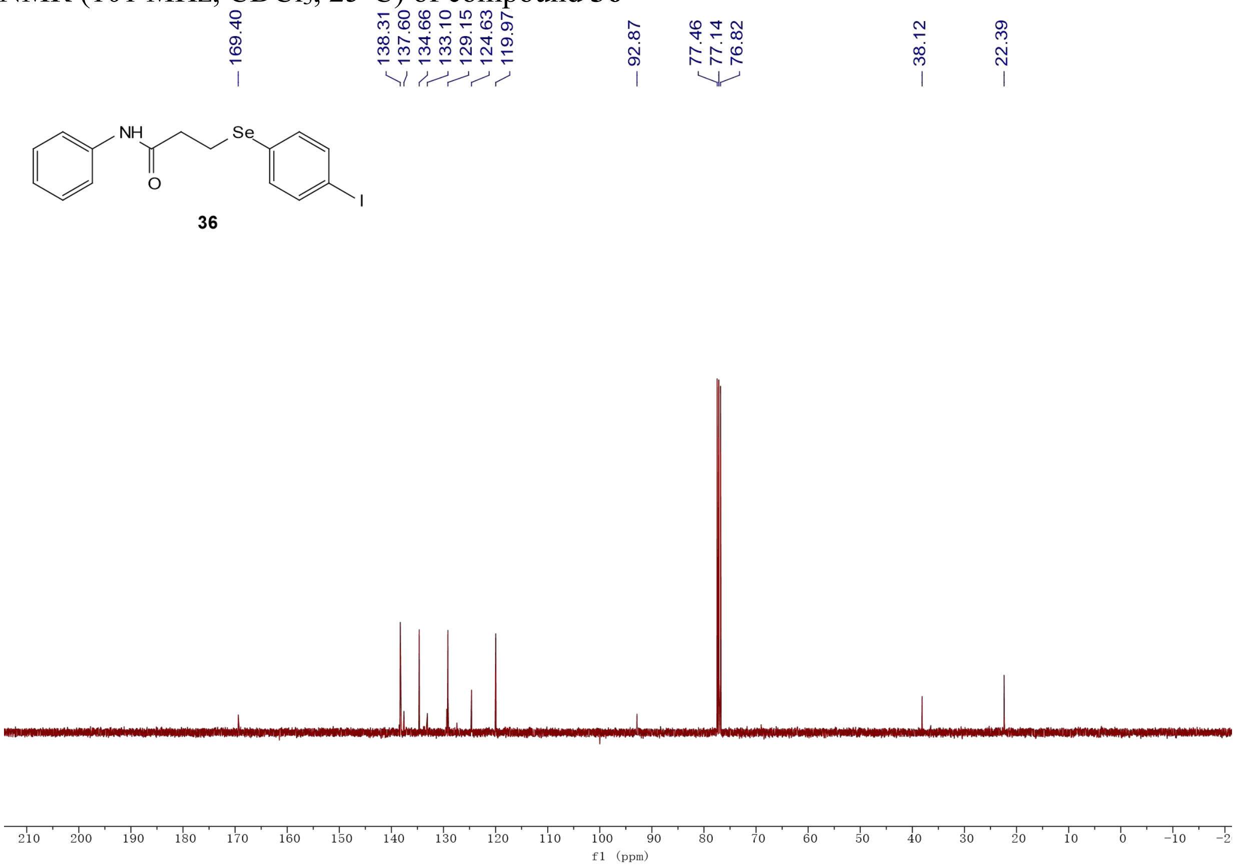

^{77}Se NMR (76 MHz, $CDCl_3$, 25°C) of compound **36**

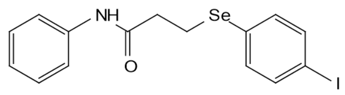

36

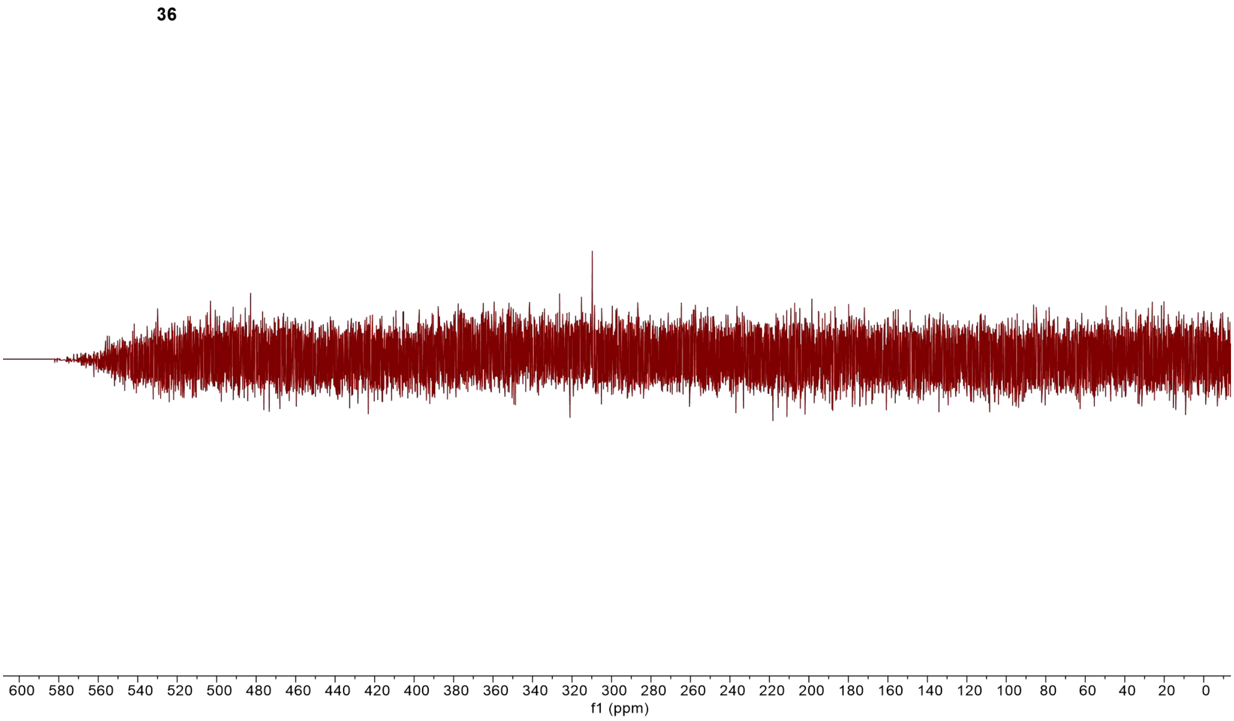

Supplementary Fig. 257. NMR of compound **36**

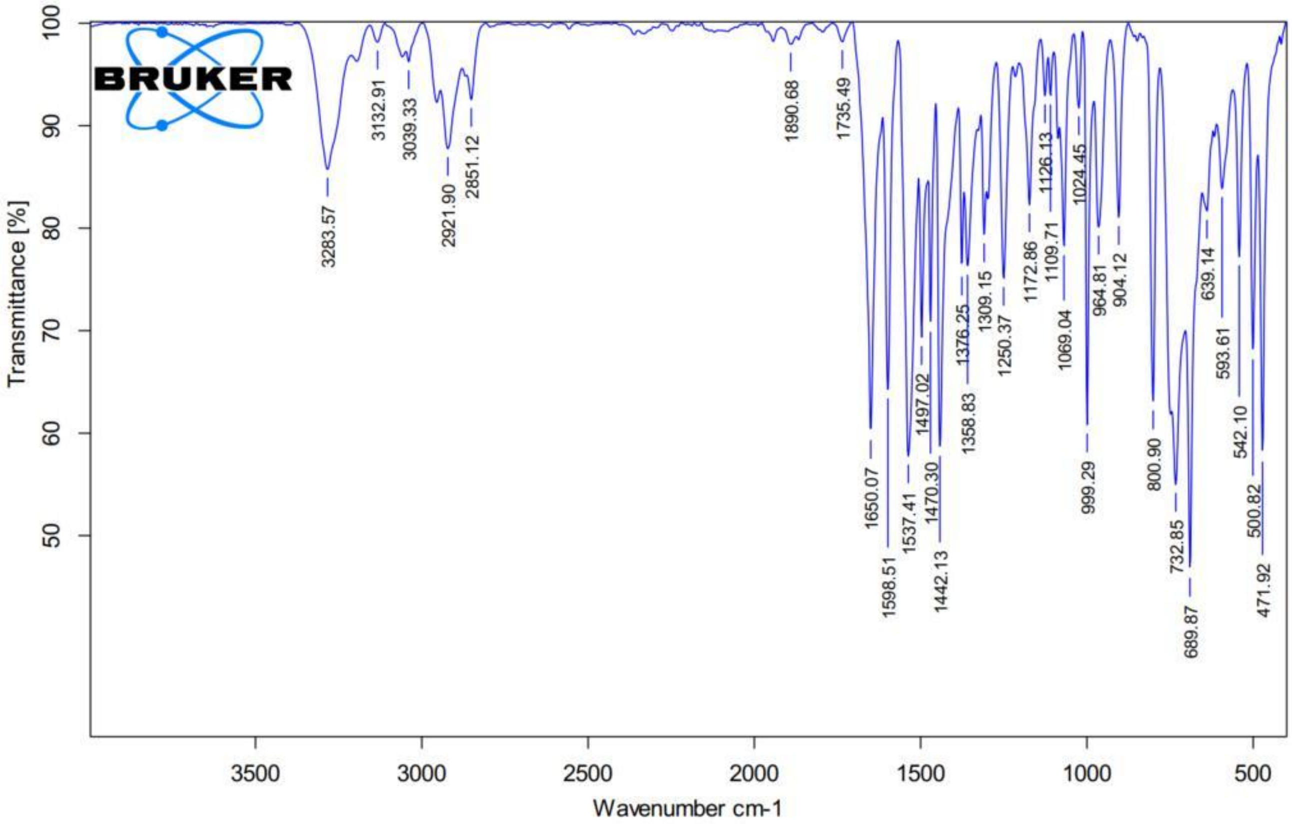

Supplementary Fig. 258. IR of compound **36**

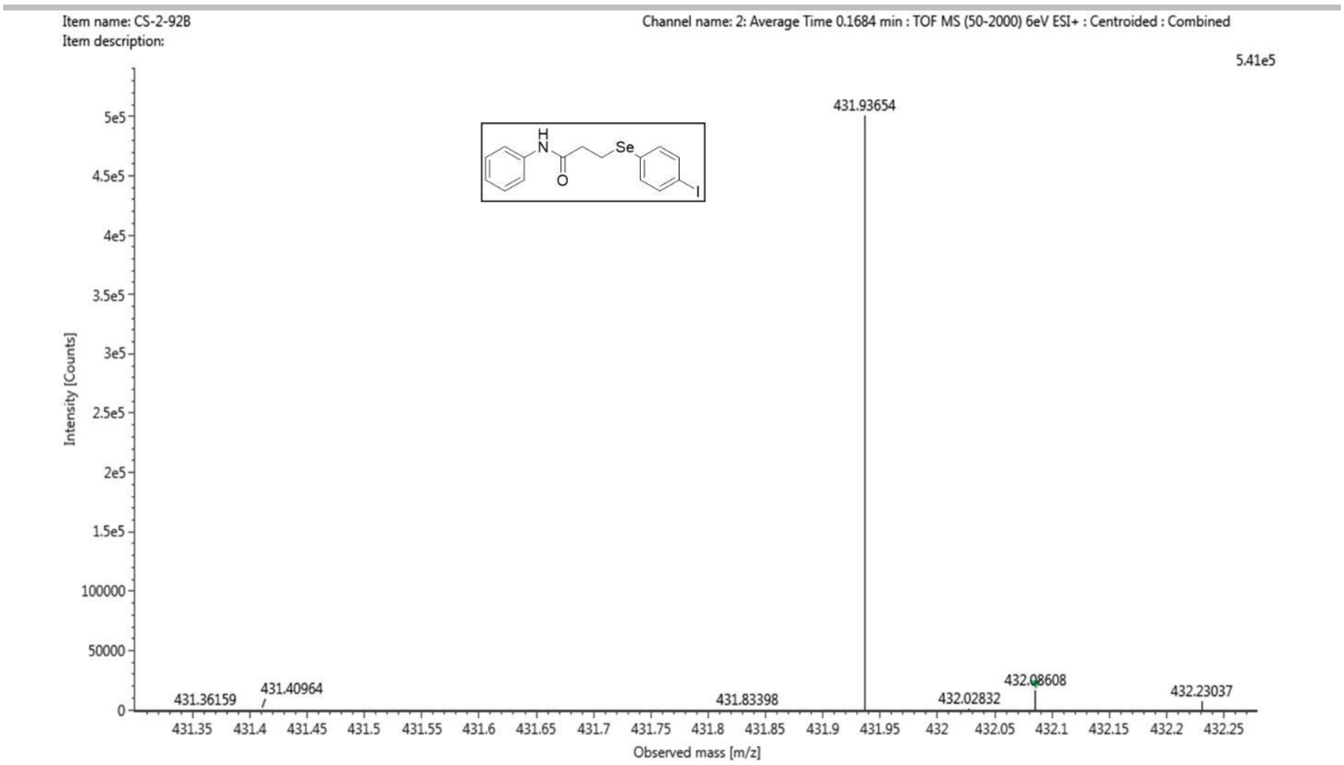

Supplementary Fig. 259. HR-MS of compound **36**

^{1}H NMR (400 MHz, $CDCl_3$, 25°C) of compound **37**

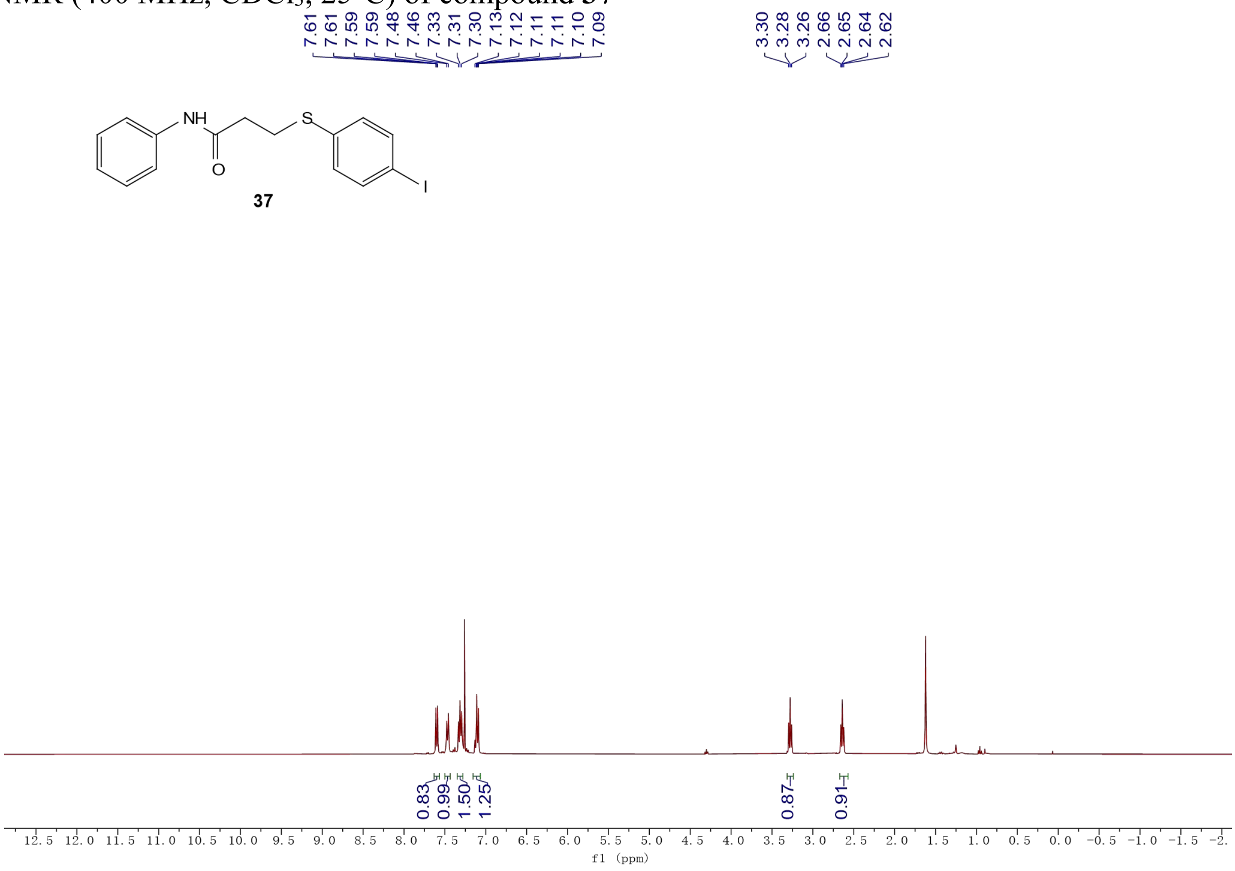

^{13}C NMR (101 MHz, $CDCl_3$, 25°C) of compound **37**

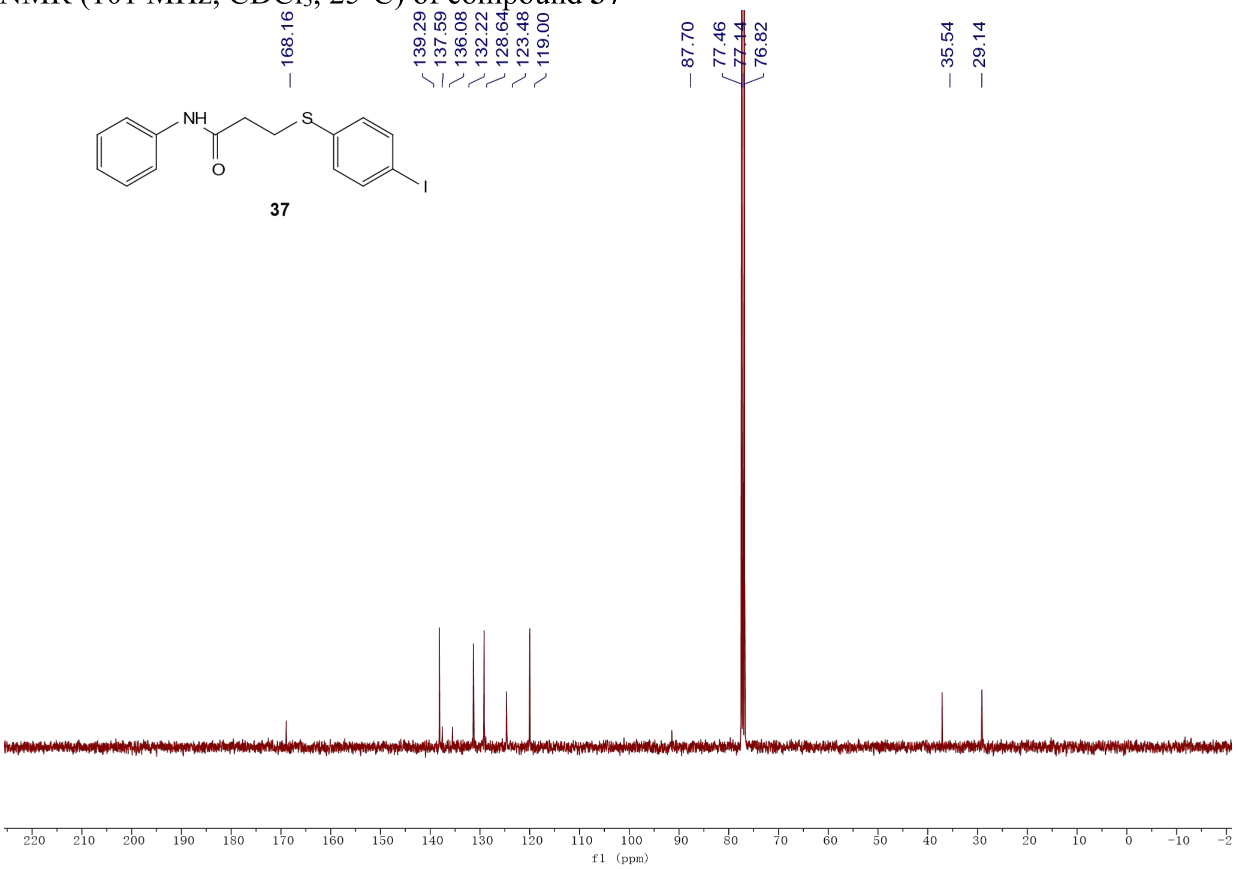

Supplementary Fig. 260. NMR of compound **37**

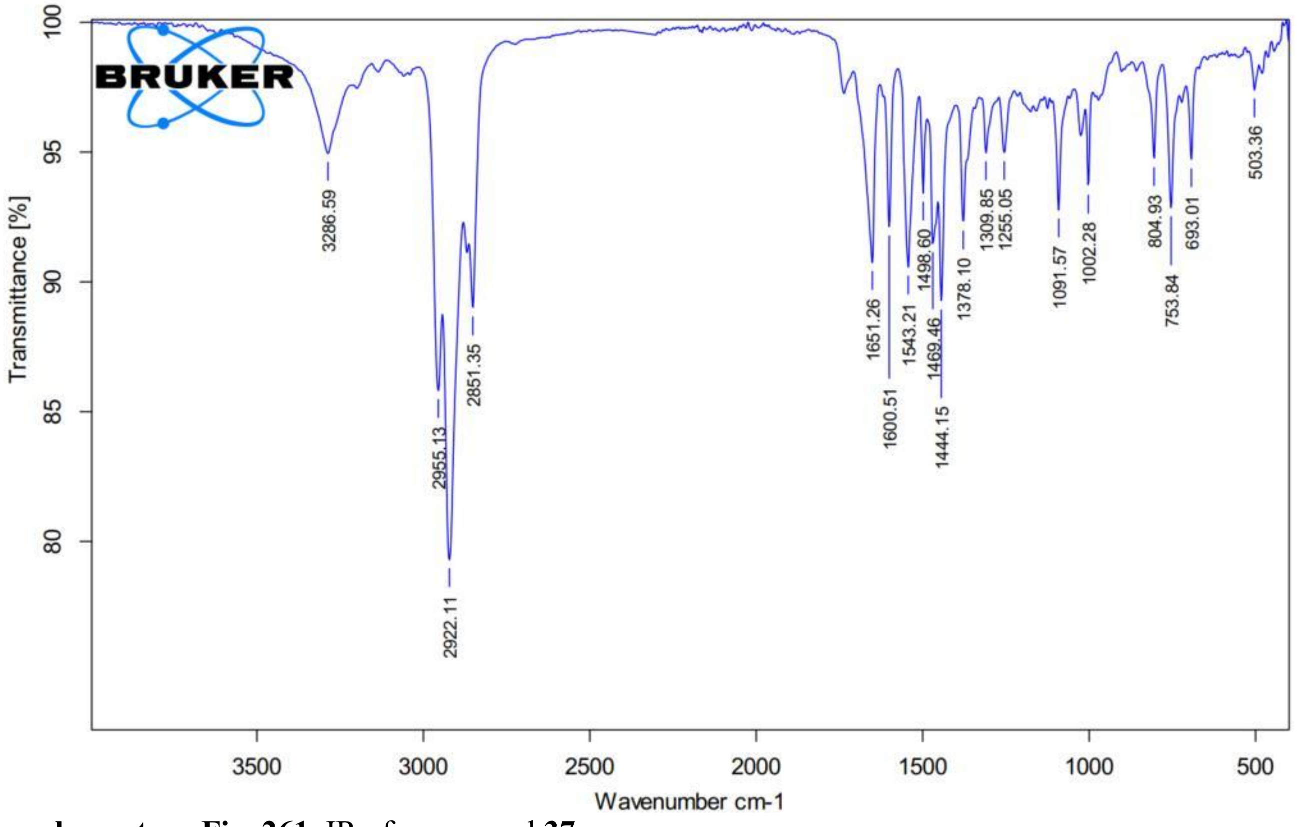

Supplementary Fig. 261. IR of compound **37**

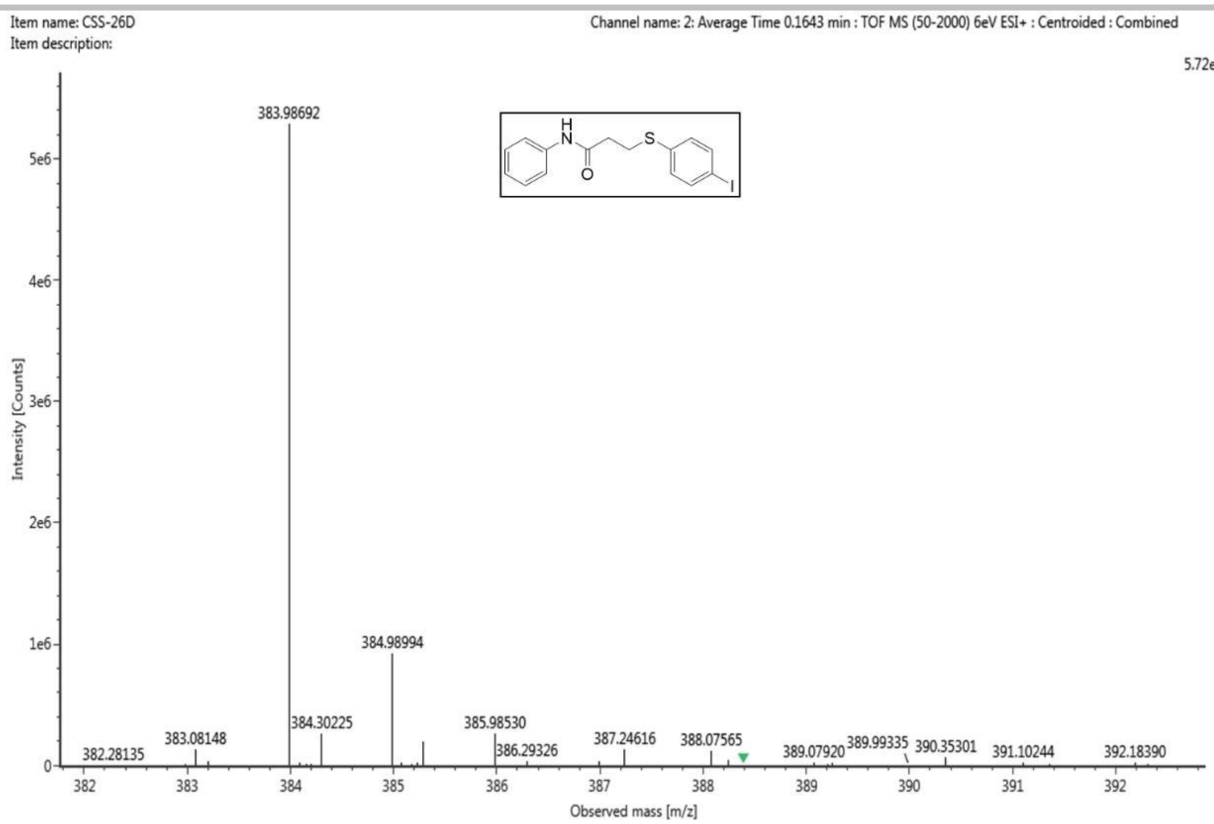

Supplementary Fig. 262. HR-MS of compound **37**

^{1}H NMR (400 MHz, $CDCl_3$, 25°C) of compound **38**

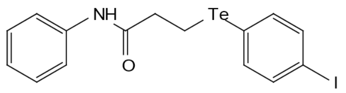

38

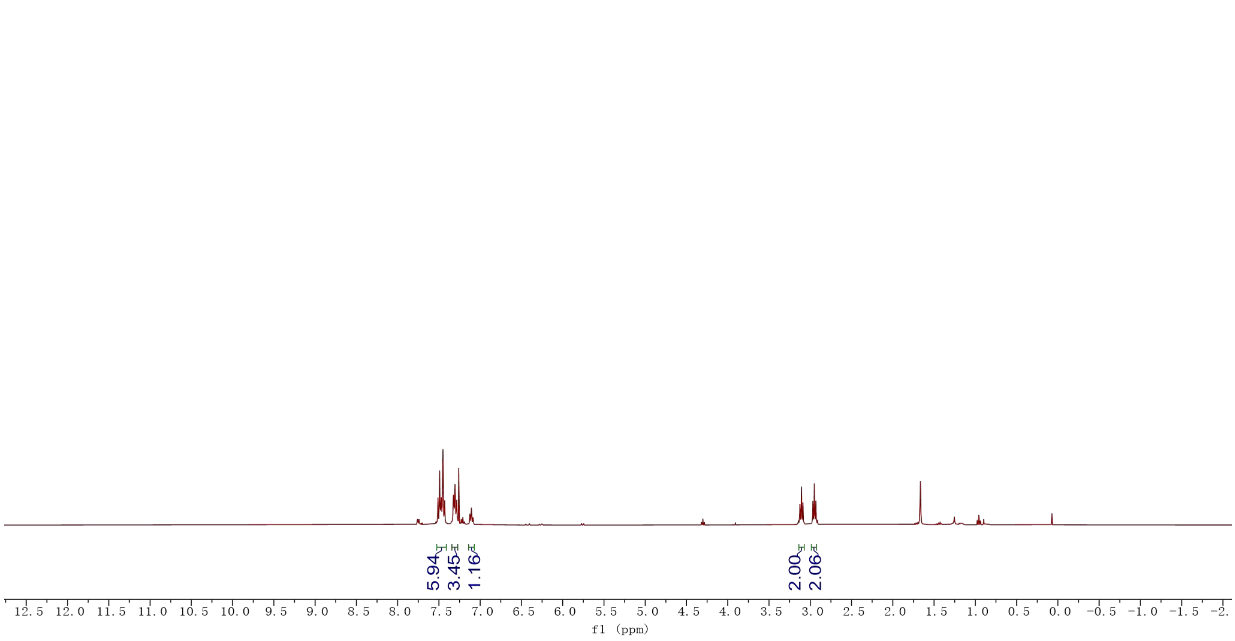

^{13}C NMR (101 MHz, $CDCl_3$, 25°C) of compound **38**

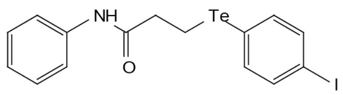

38

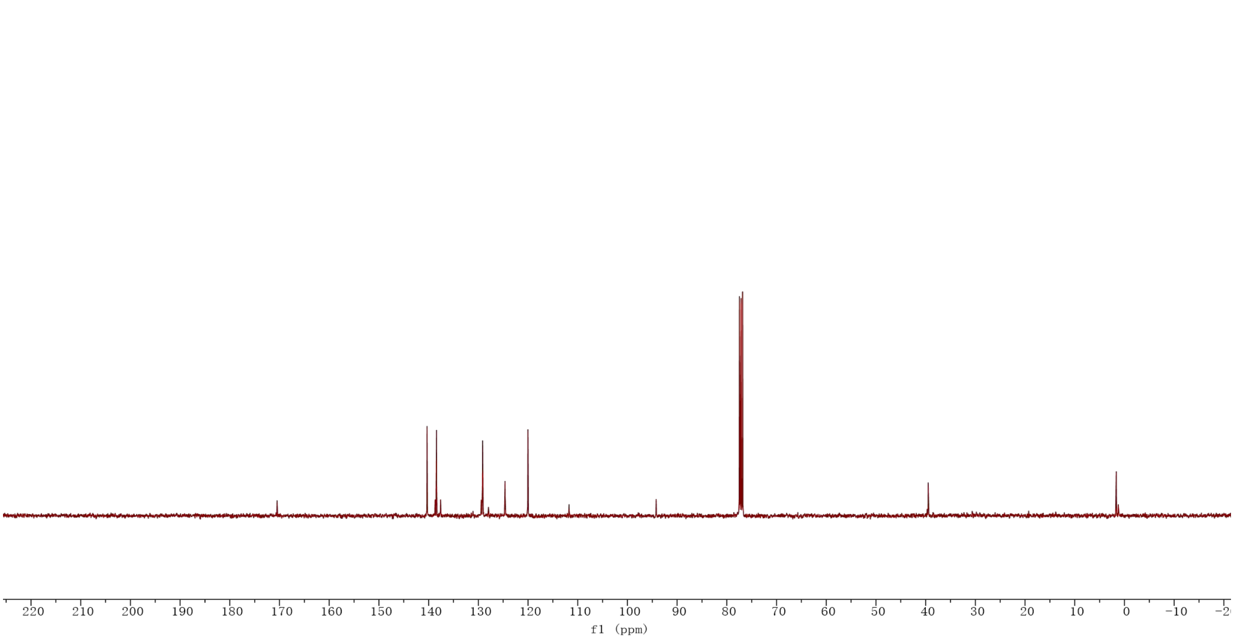

Supplementary Fig. 263. NMR of compound **38**

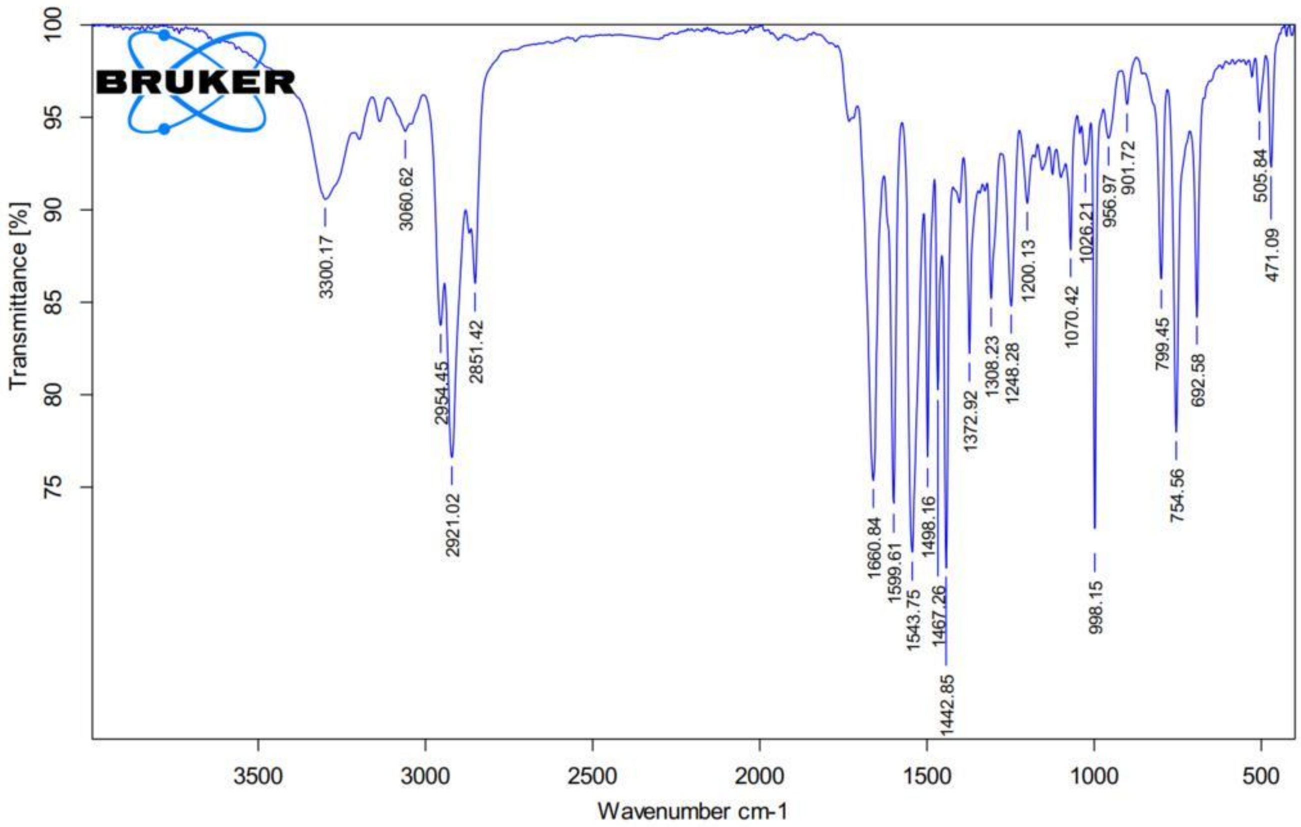

Supplementary Fig. 264. IR of compound **38**

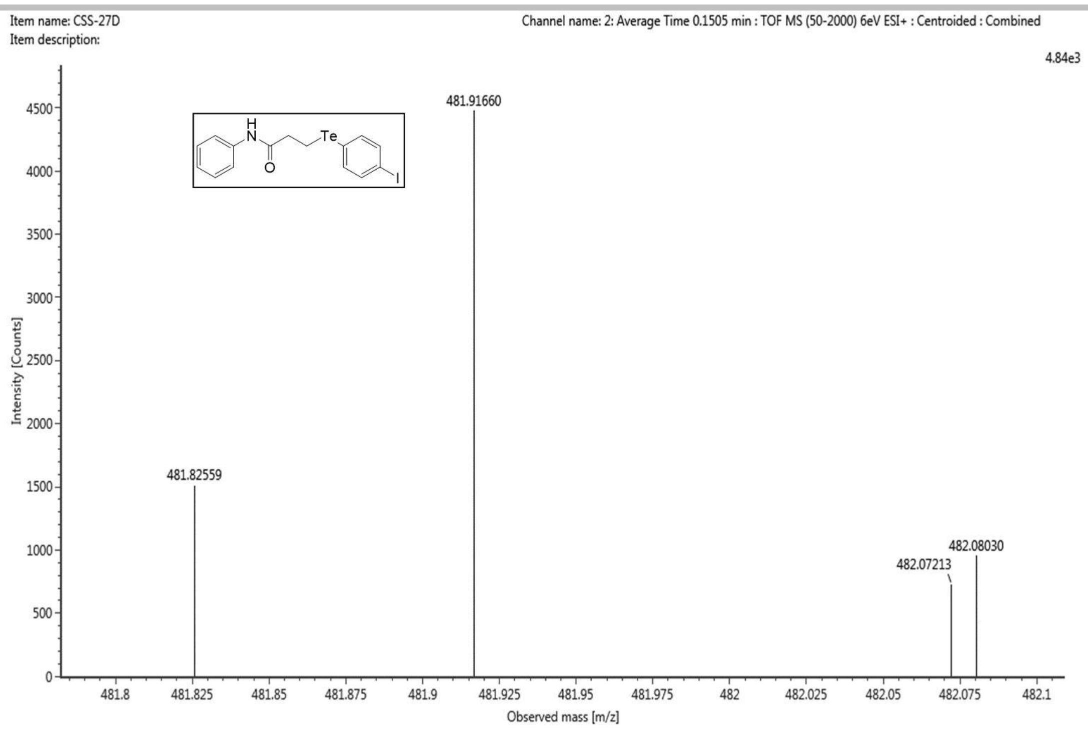

Supplementary Fig. 265. HR-MS of compound **38**

^{1}H NMR (400 MHz, $CDCl_3$, 25°C) of compound **40**

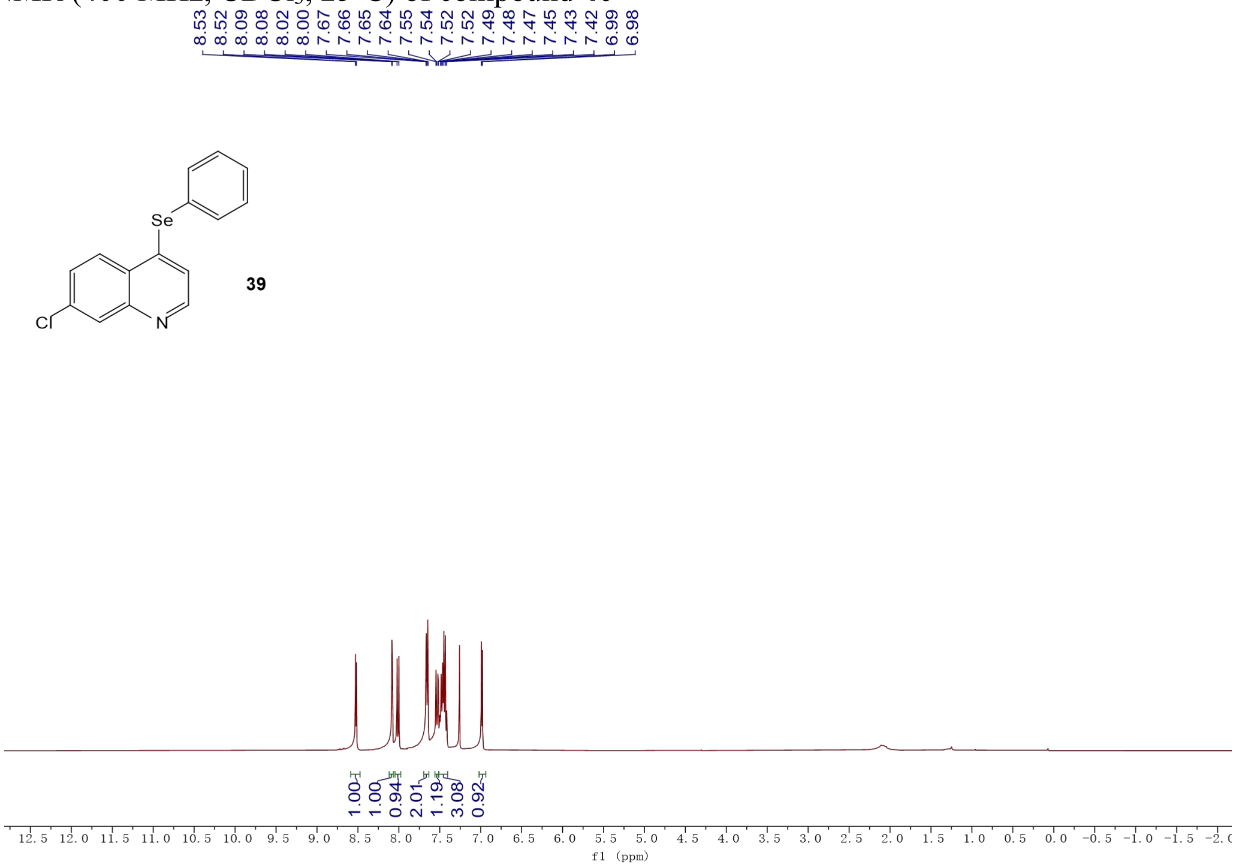

^{13}C NMR (101 MHz, $CDCl_3$, 25°C) of compound **40**

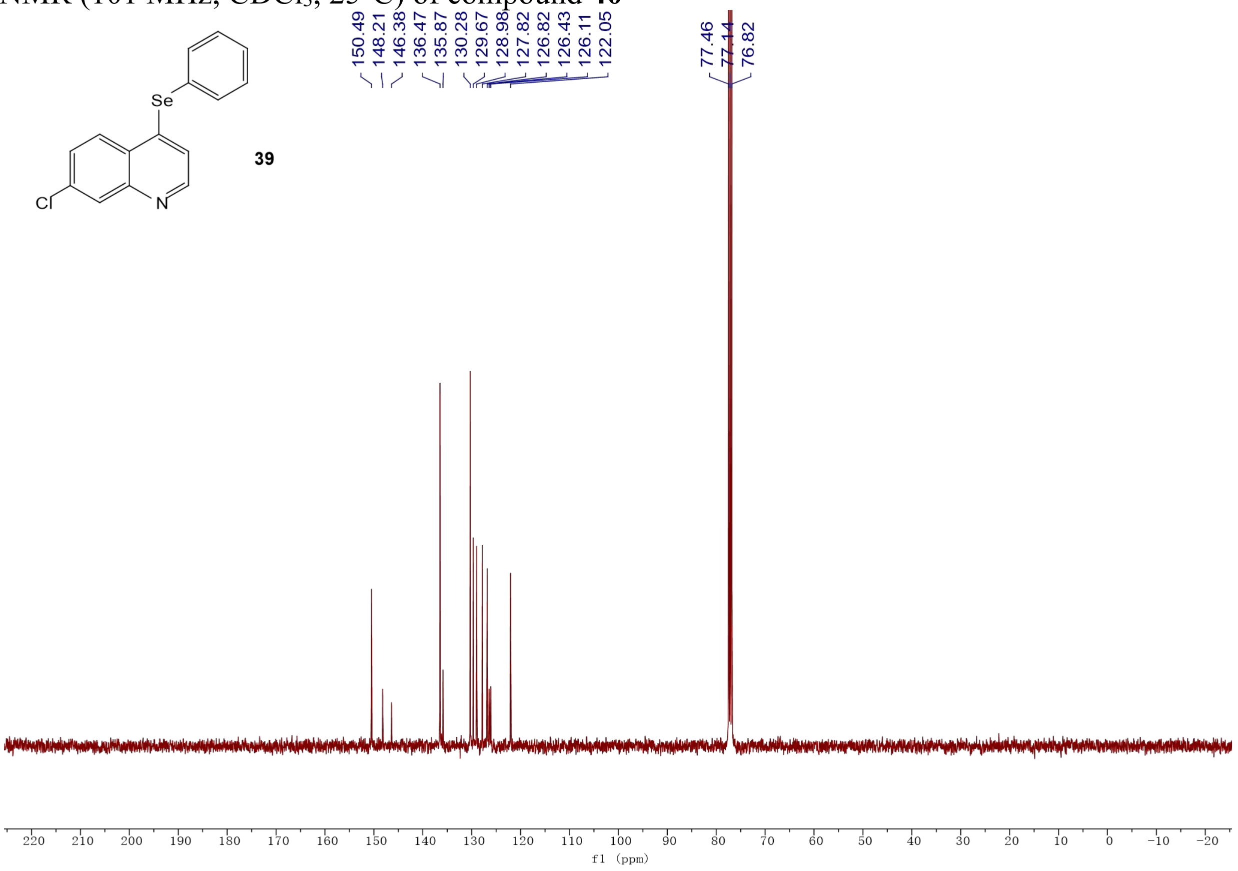

^{77}Se NMR (76 MHz, $CDCl_3$, 25°C) of compound **40**

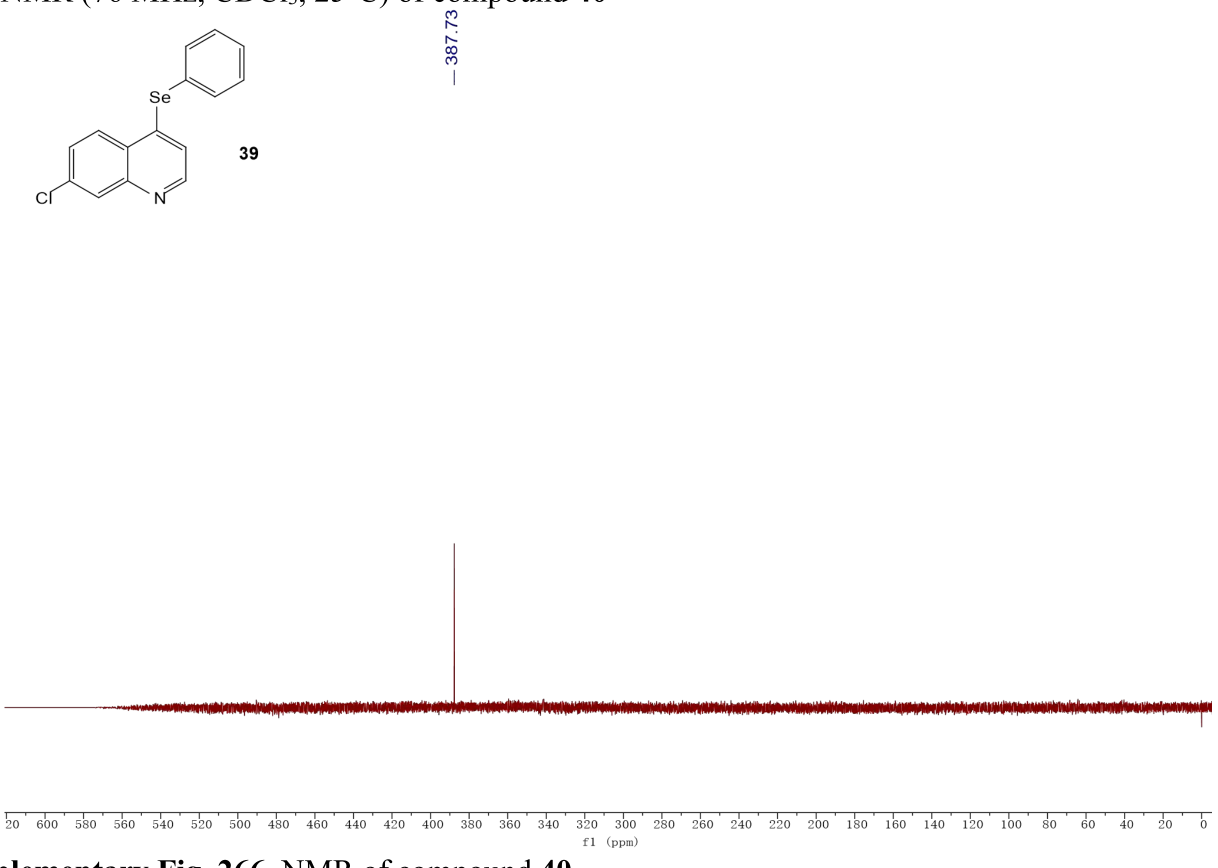

Supplementary Fig. 266. NMR of compound **40**

^1H NMR (400 MHz, $CDCl_3$, 25°C) of compound **41**

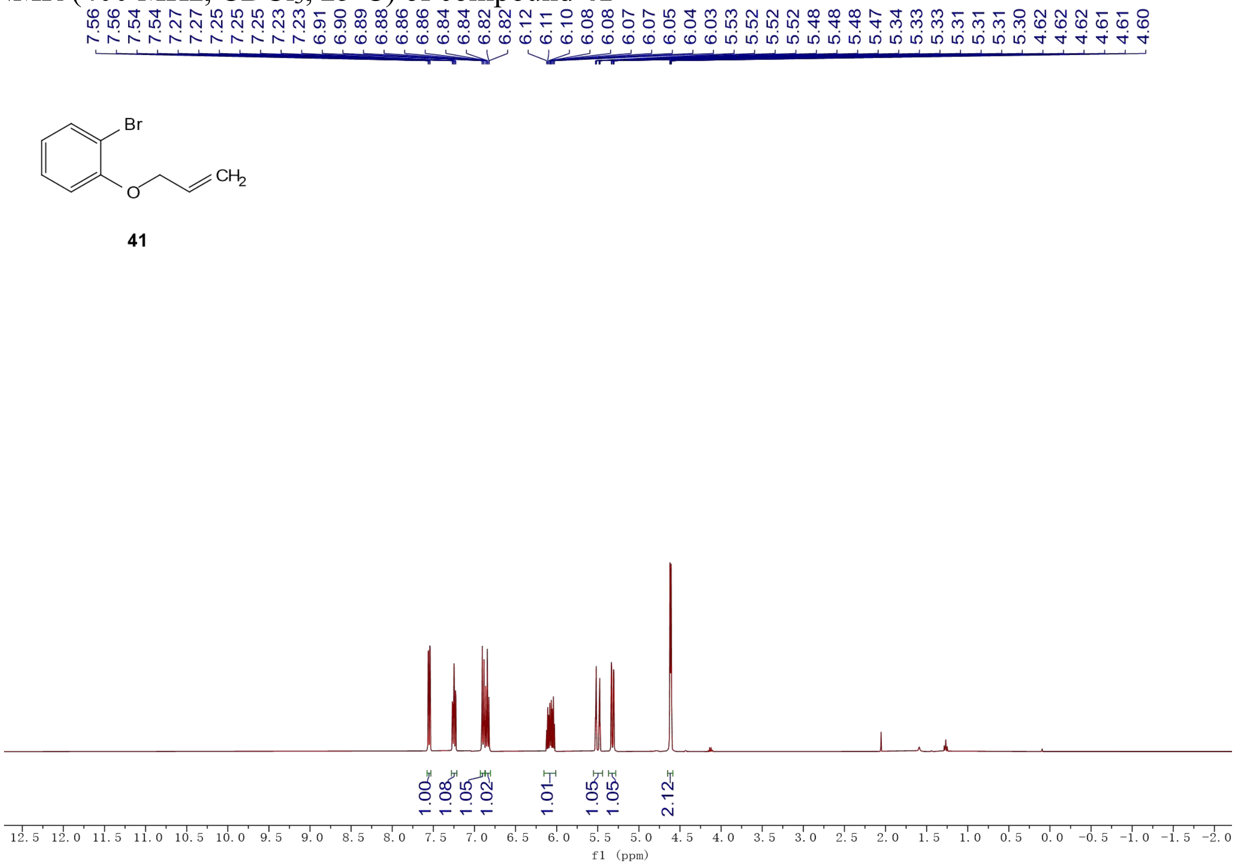

^{13}C NMR (101 MHz, $CDCl_3$, 25°C) of compound **41**

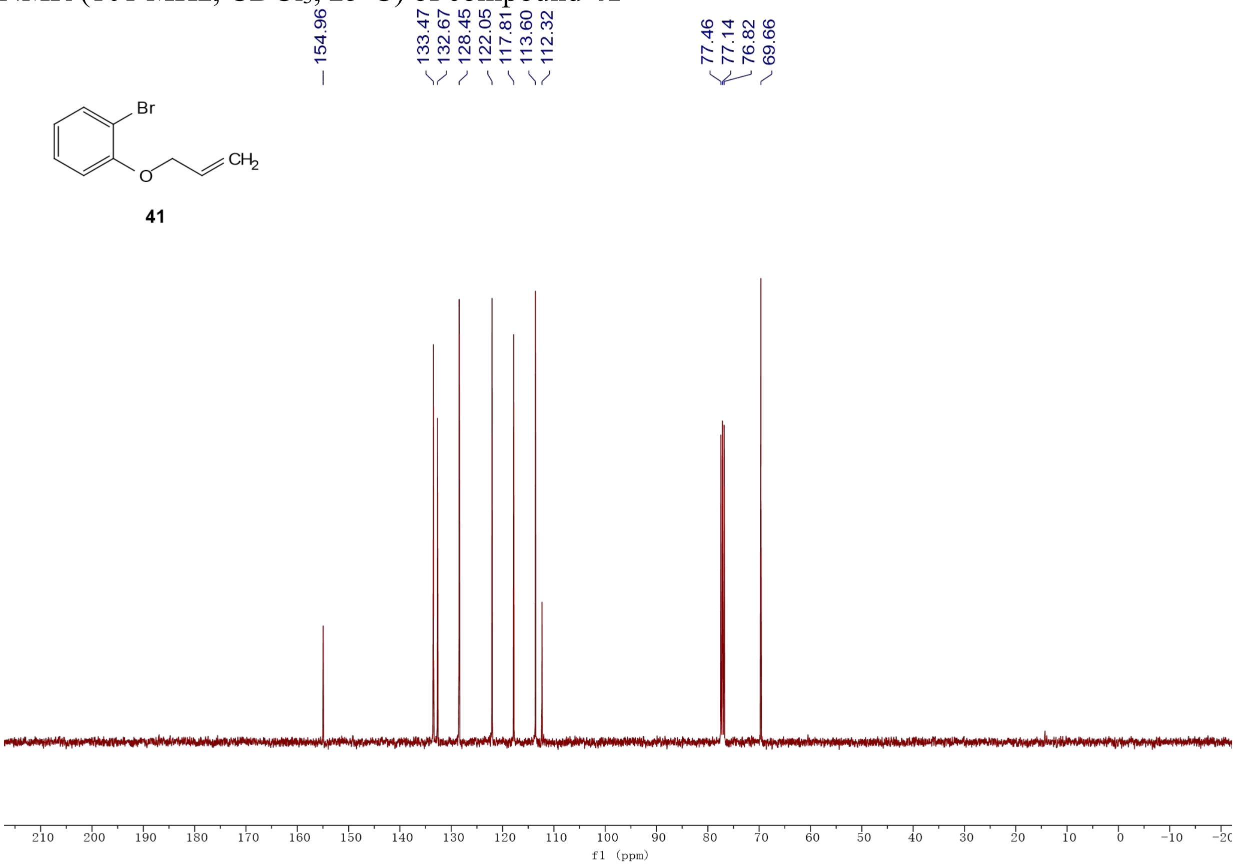

Supplementary Fig. 267. NMR of compound **41**

^{1}H NMR (400 MHz, $CDCl_3$, 25°C) of compound **45**

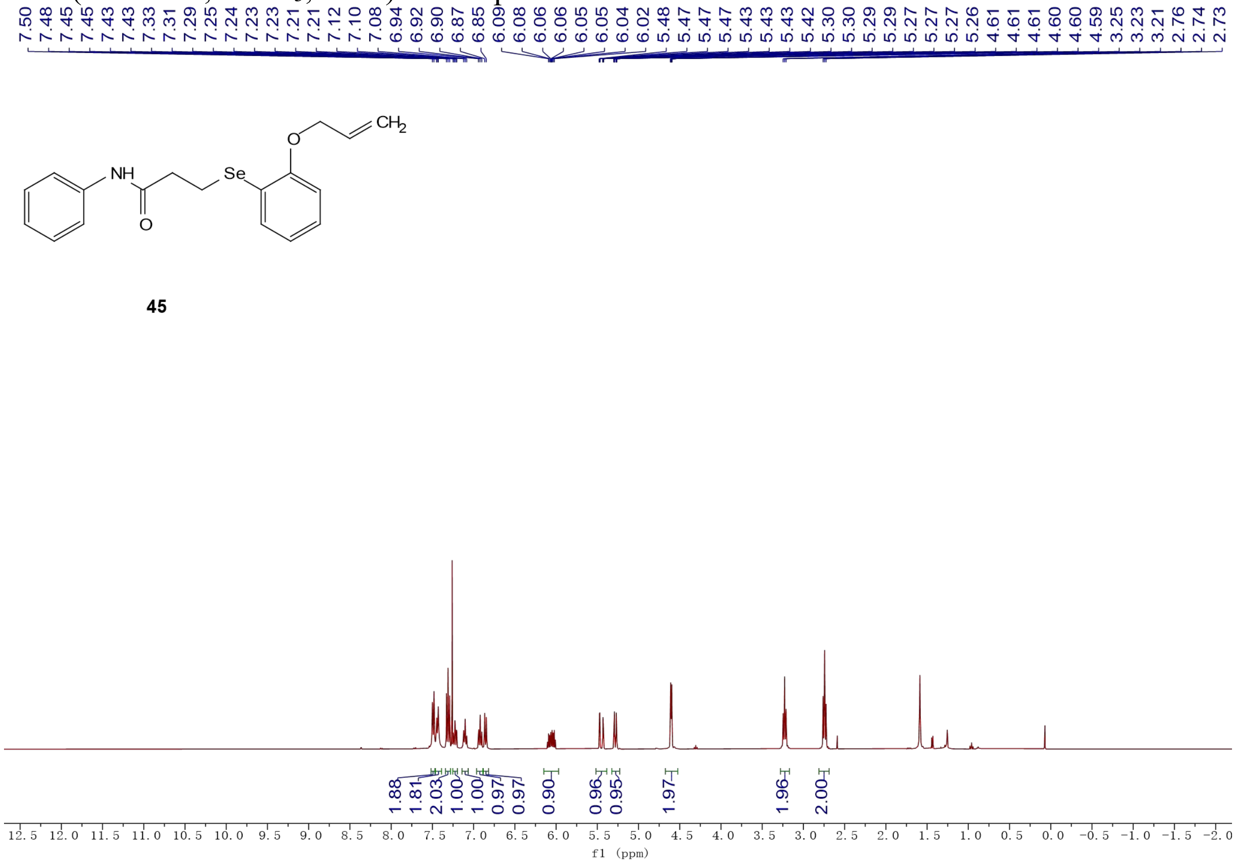

^{13}C NMR (101 MHz, $CDCl_3$, 25°C) of compound **45**

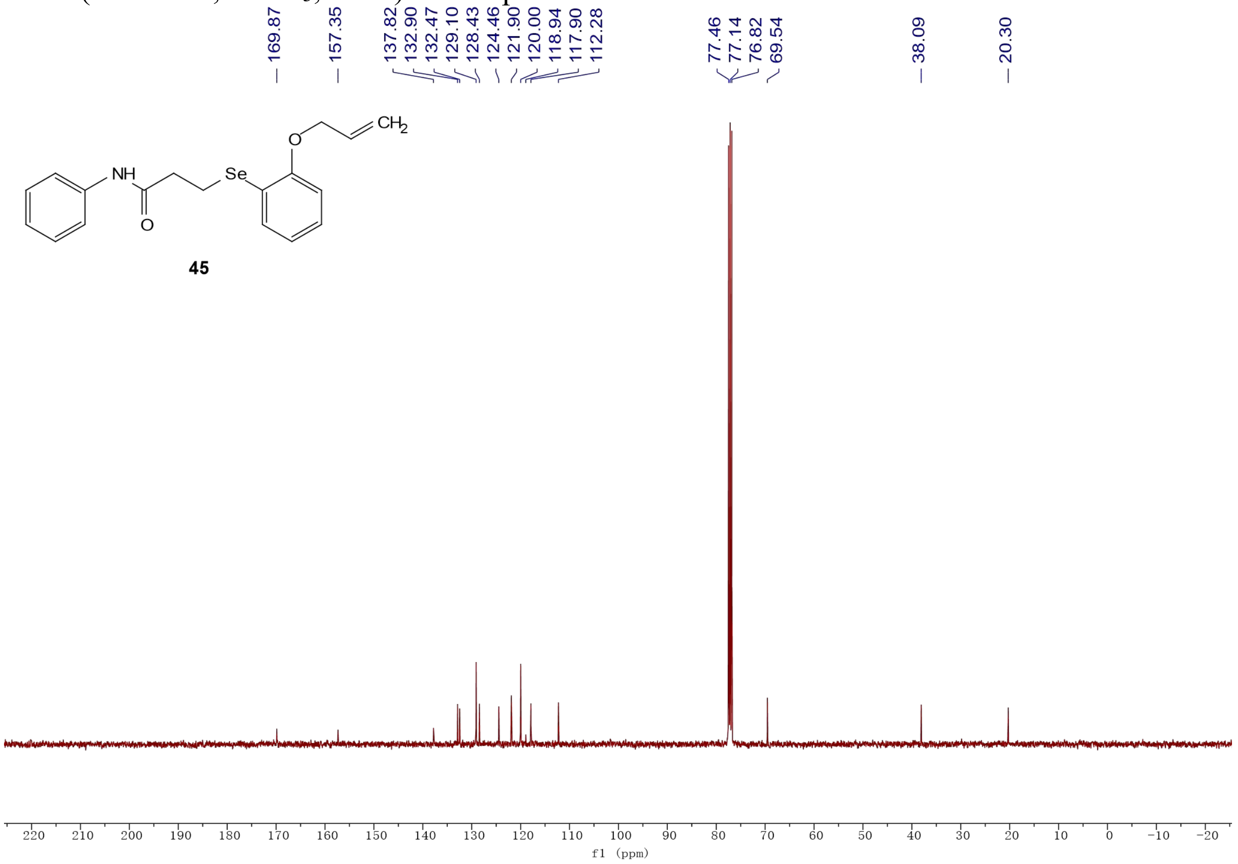

^{77}Se NMR (76 MHz, $CDCl_3$, 25°C) of compound **45**

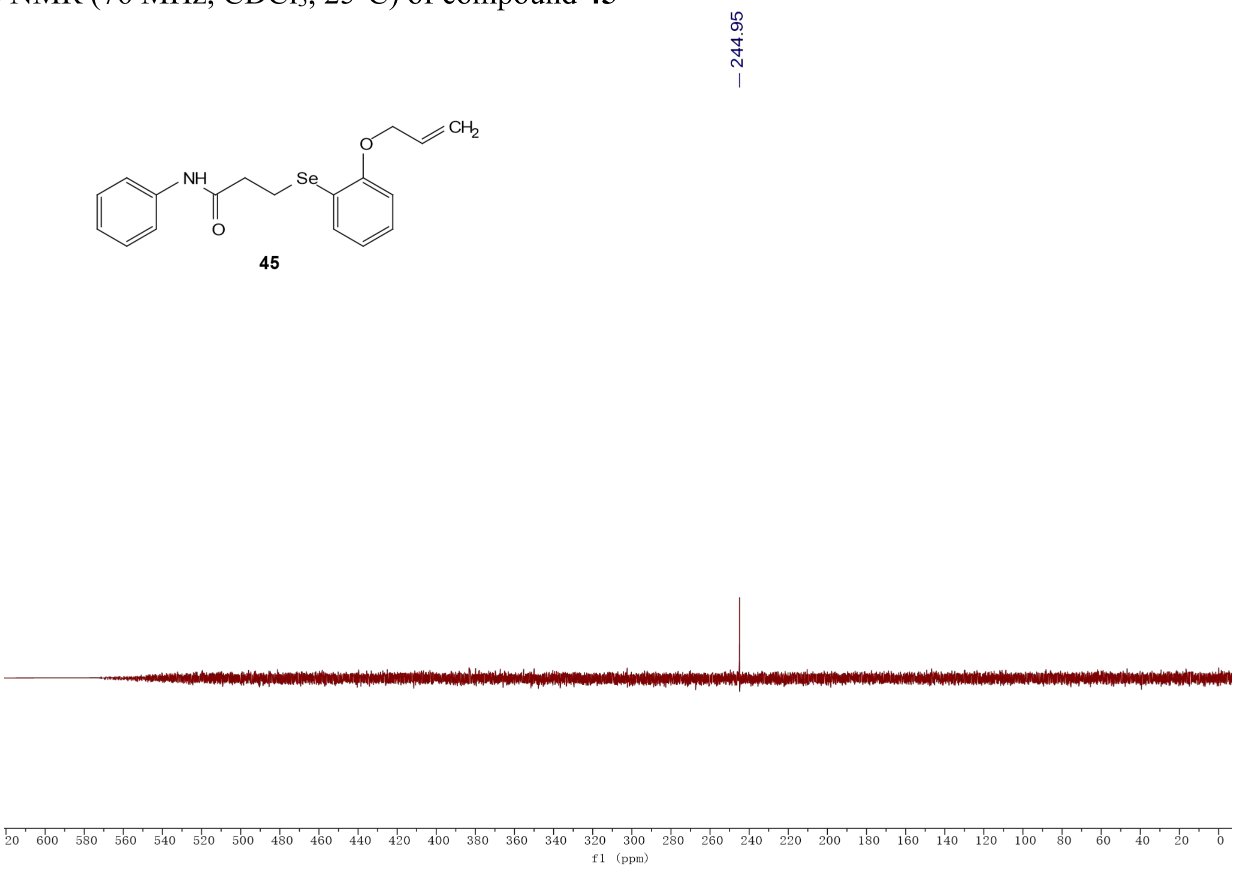

Supplementary Fig. 268. NMR of compound **45**

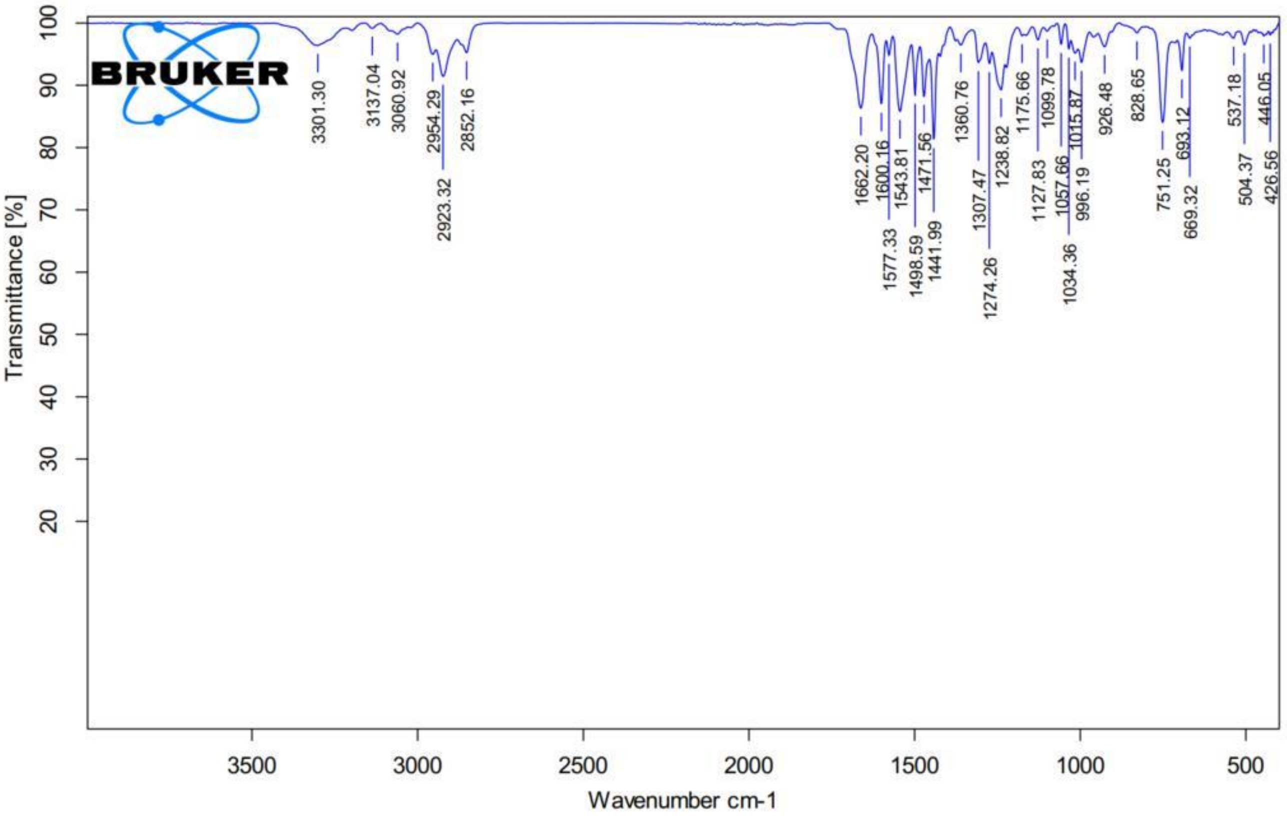

Supplementary Fig. 269. IR of compound **45**

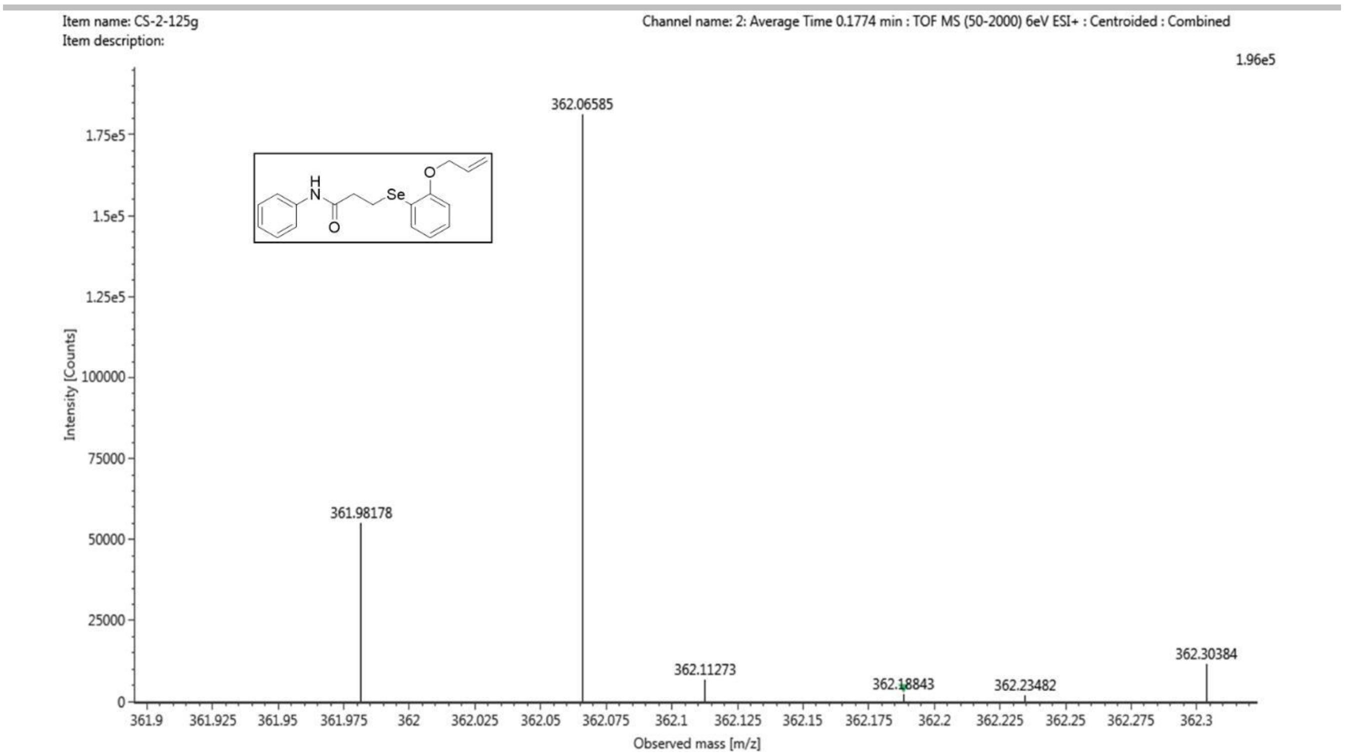

Supplementary Fig. 270. HR-MS of compound **45**

^{1}H NMR (400 MHz, $CDCl_3$, 25°C) of compound **3b**

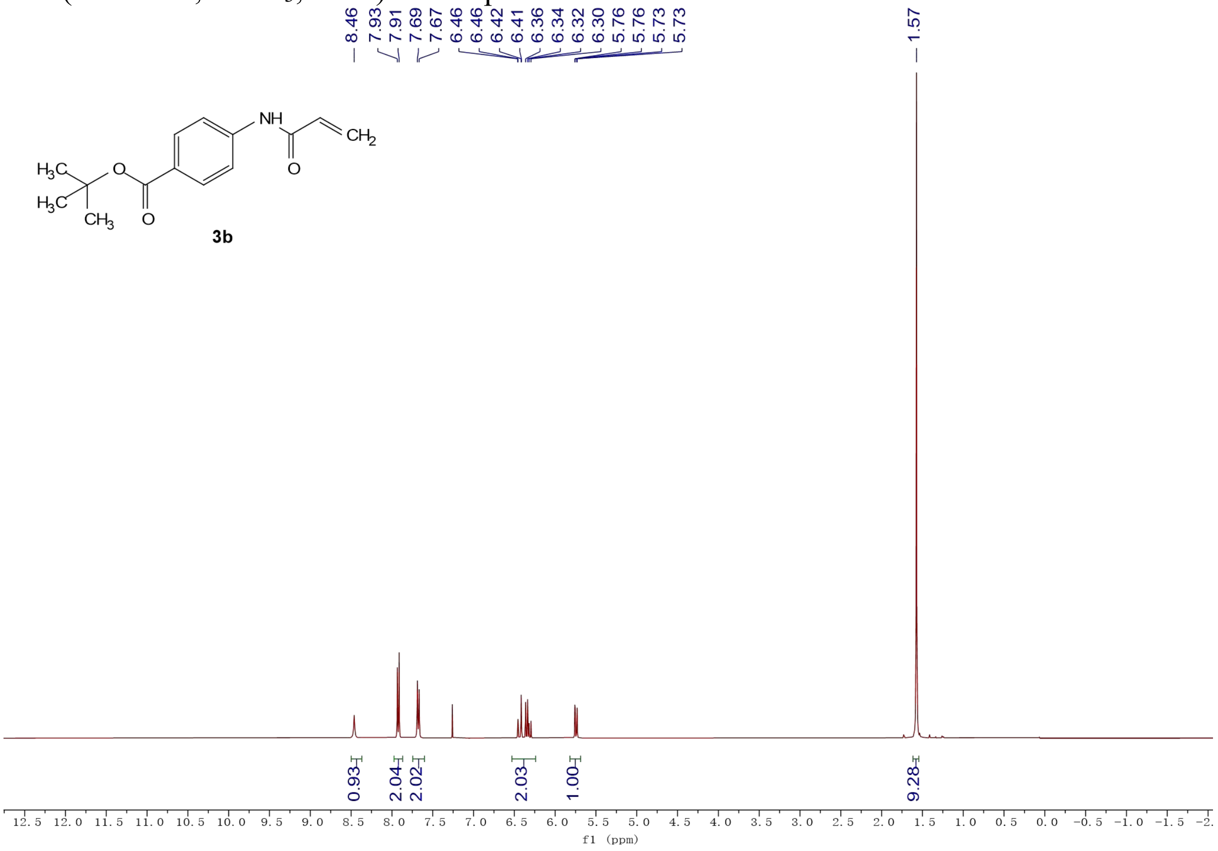

^{13}C NMR (101 MHz, $CDCl_3$, 25°C) of compound **3b**

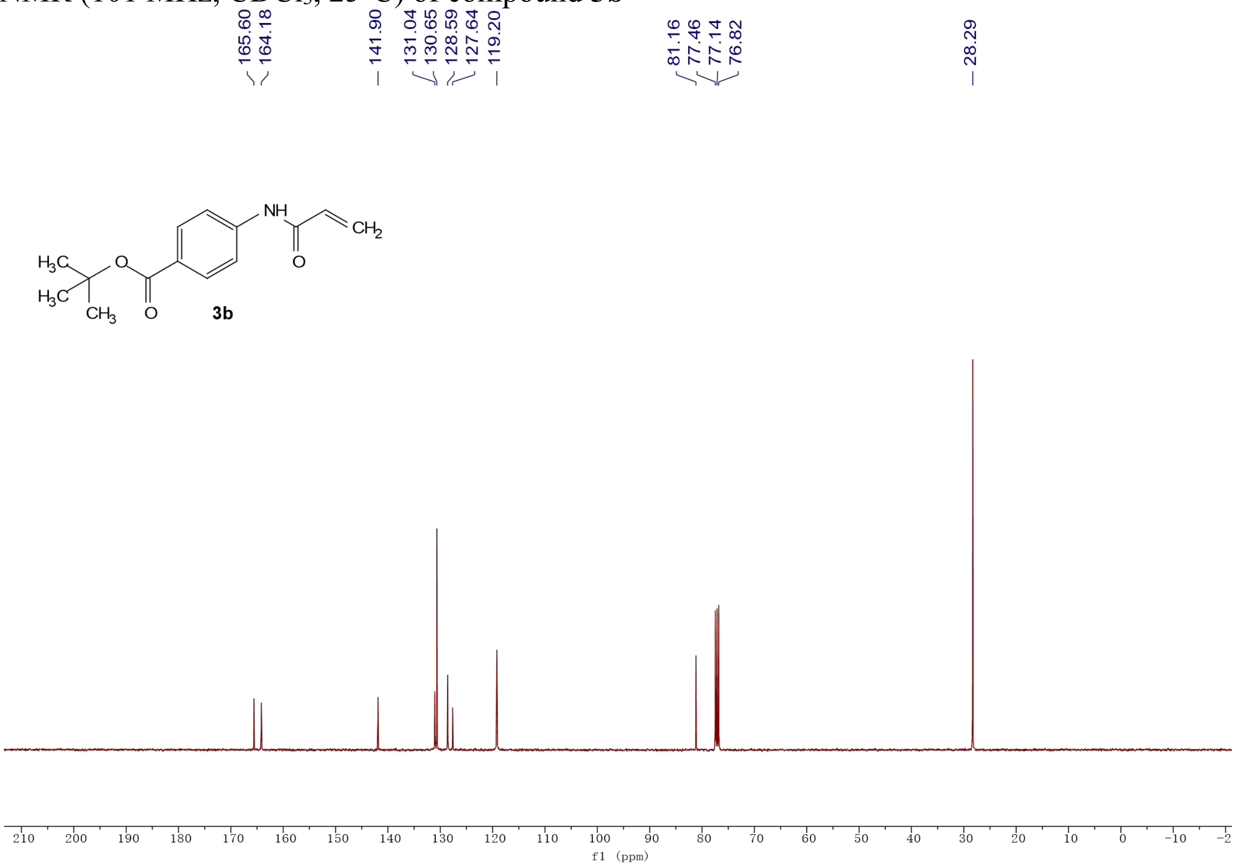

Supplementary Fig. 271. NMR of compound **3b**

^{1}H NMR (400 MHz, $CDCl_3$, 25°C) of compound **3c**

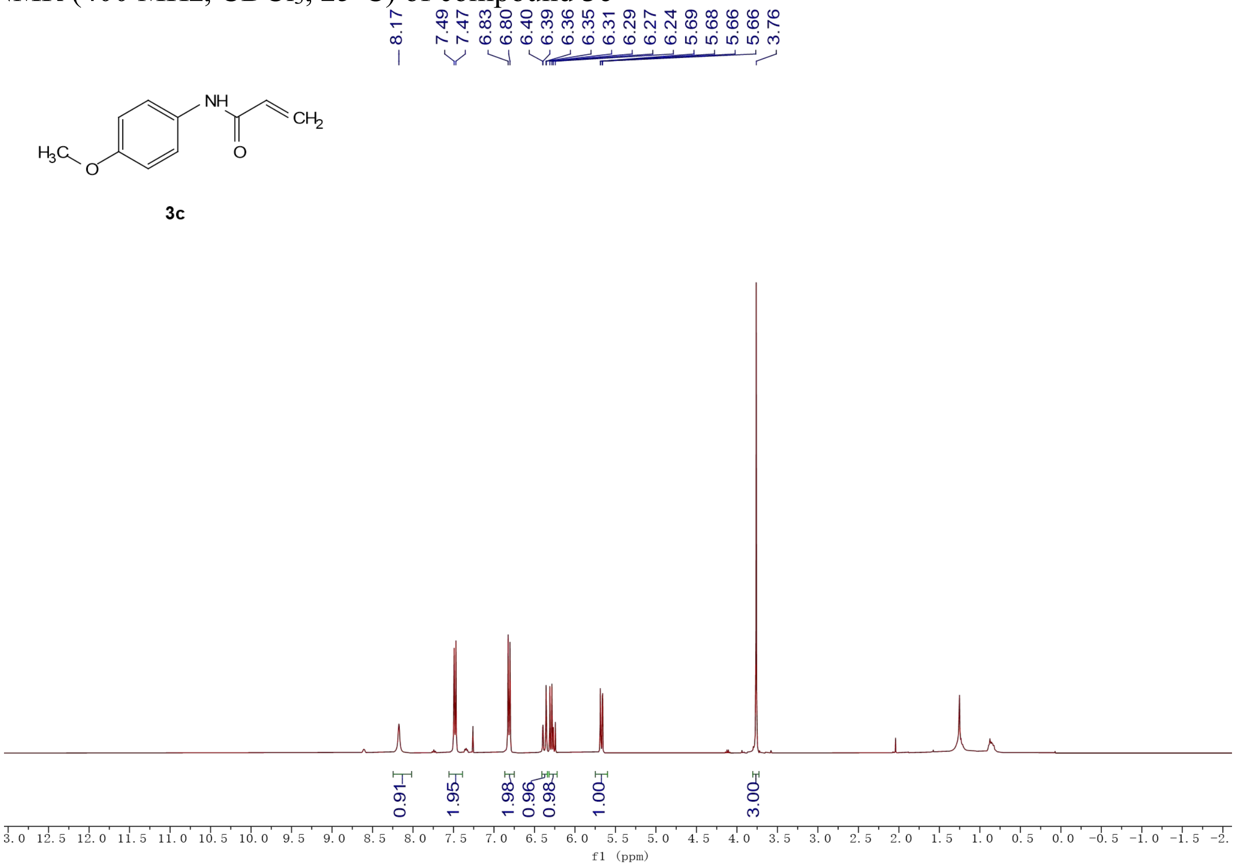

^{13}C NMR (101 MHz, $CDCl_3$, 25°C) of compound **3c**

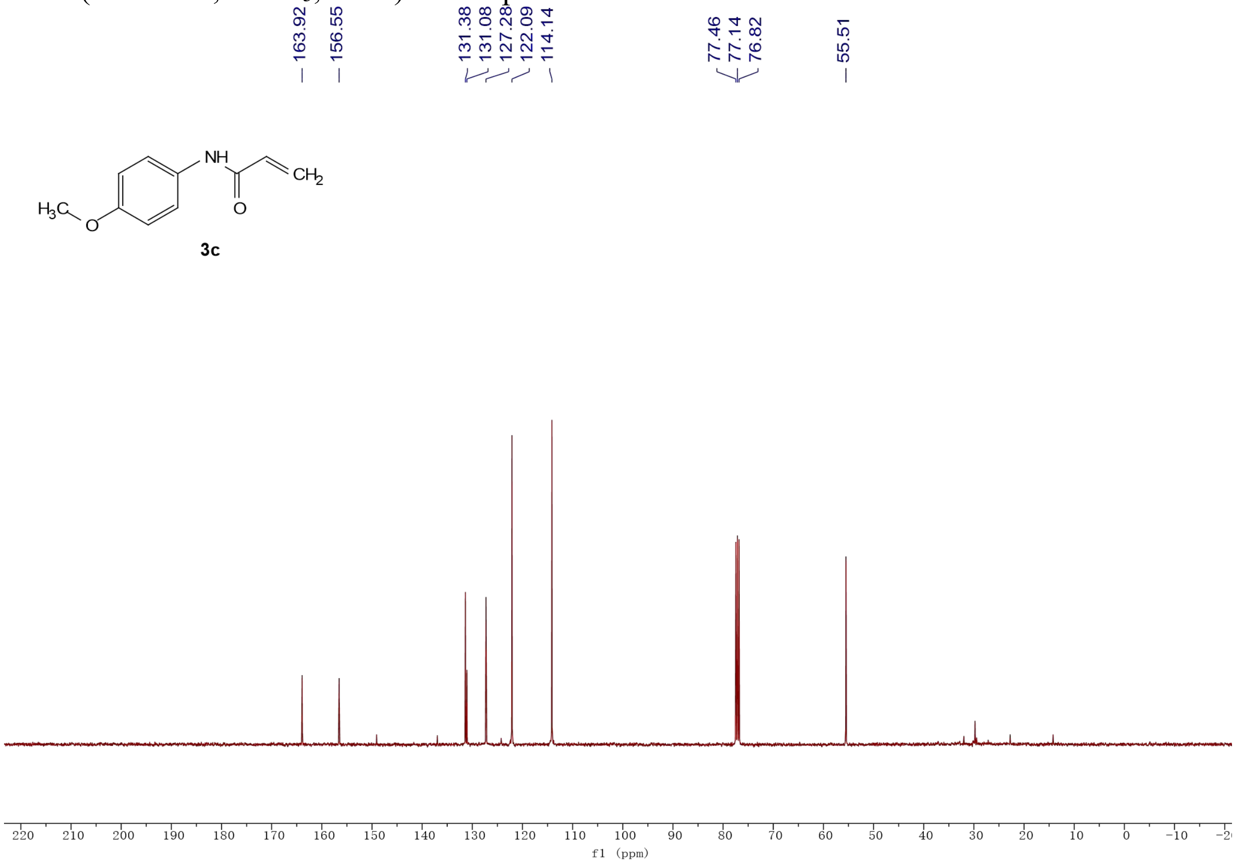

Supplementary Fig. 272. NMR of compound **3c**

^{1}H NMR (400 MHz, $CDCl_3$, 25°C) of compound **3d**

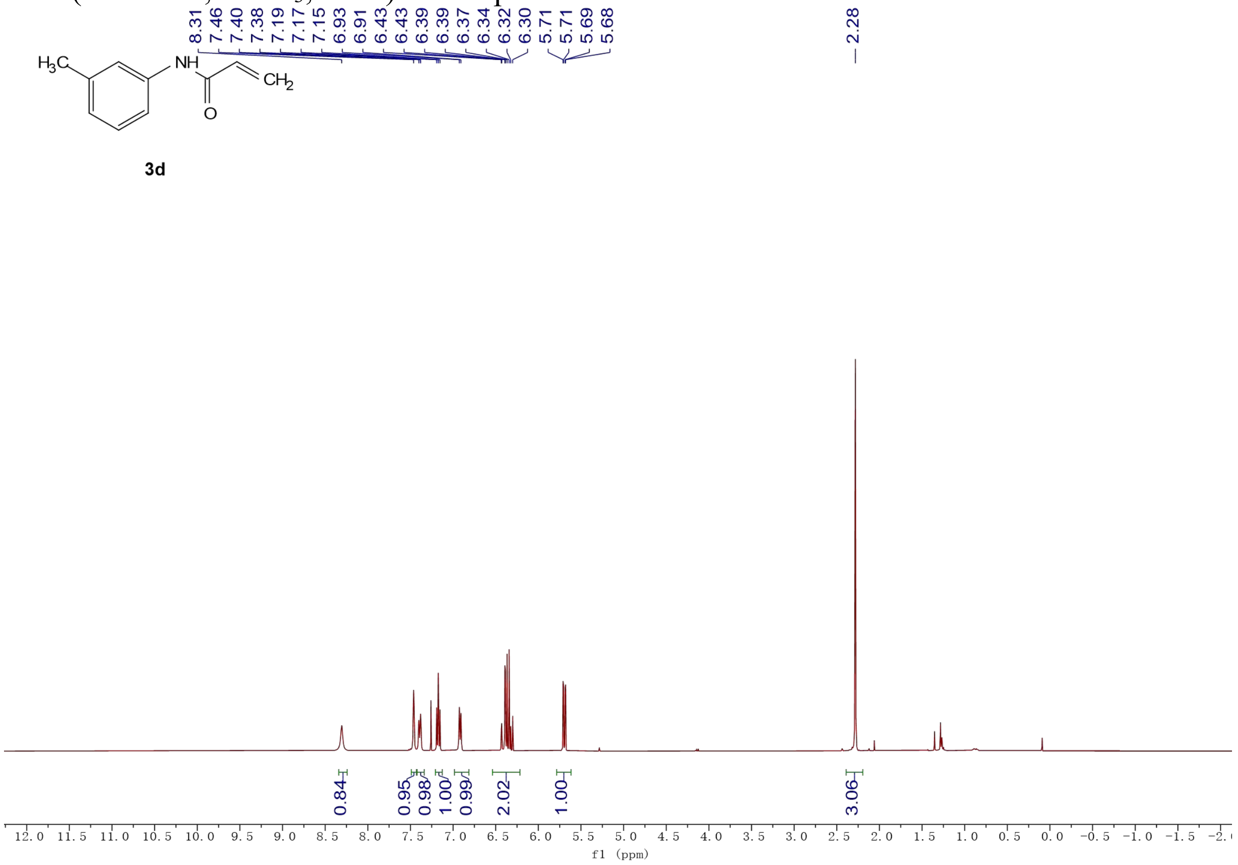

^{13}C NMR (101 MHz, $CDCl_3$, 25°C) of compound **3d**

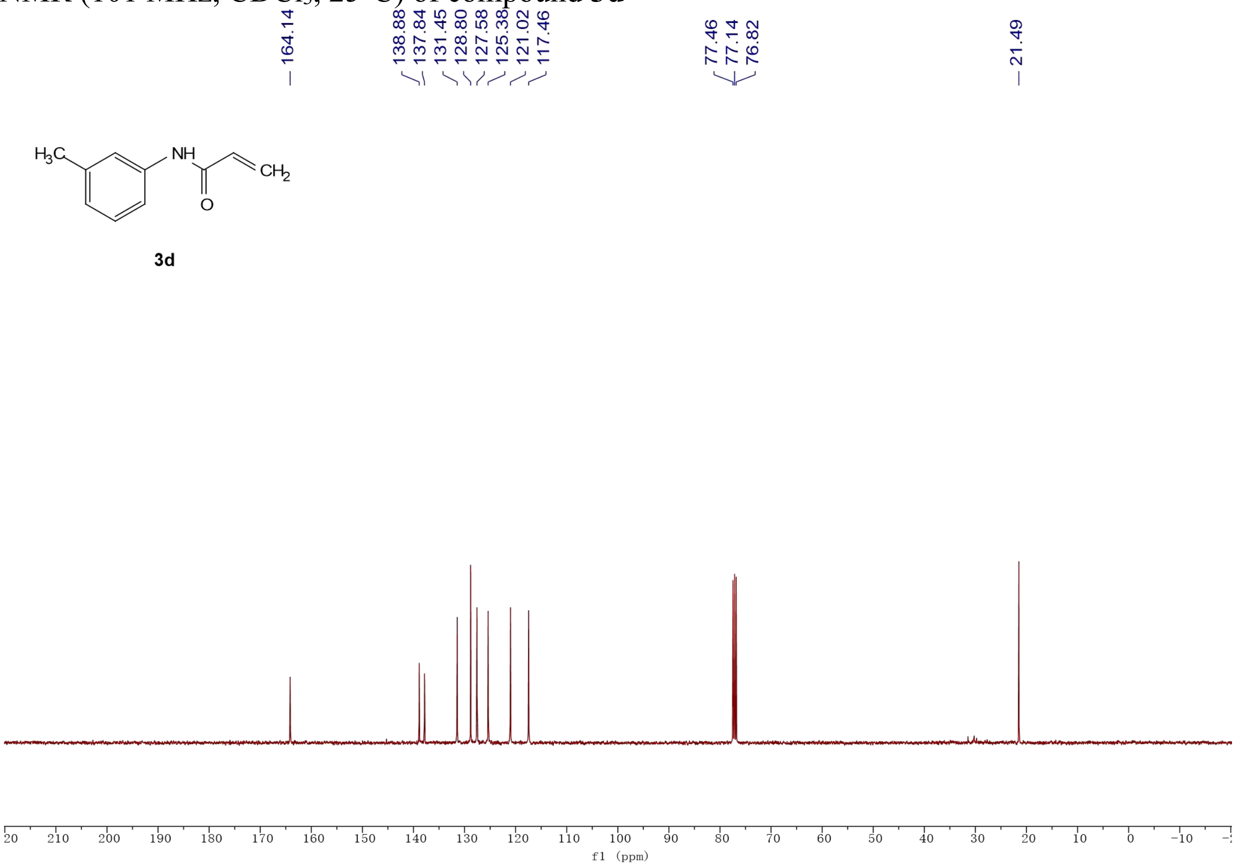

Supplementary Fig. 273. NMR of compound **3d**

^{1}H NMR (400 MHz, $CDCl_3$, 25°C) of compound **1s**

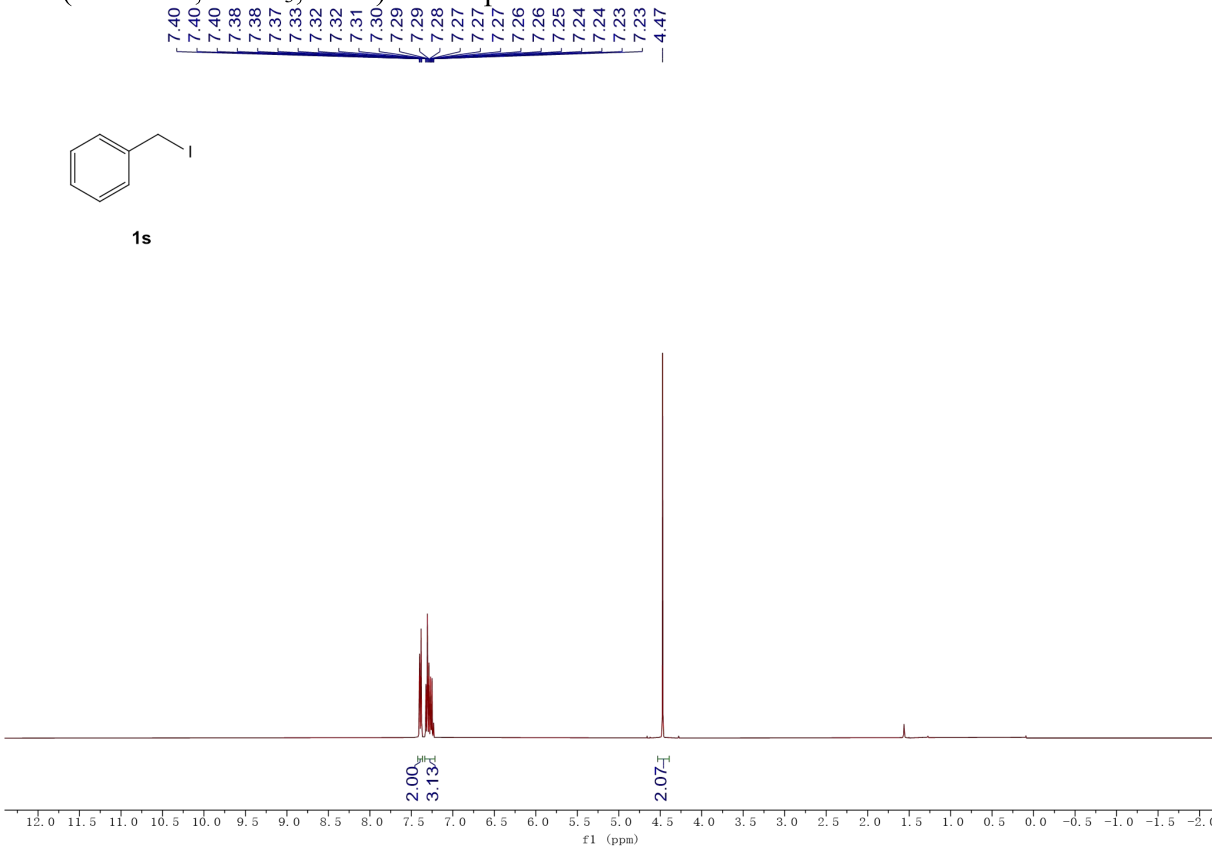

^{13}C NMR (101 MHz, $CDCl_3$, 25°C) of compound **1s**

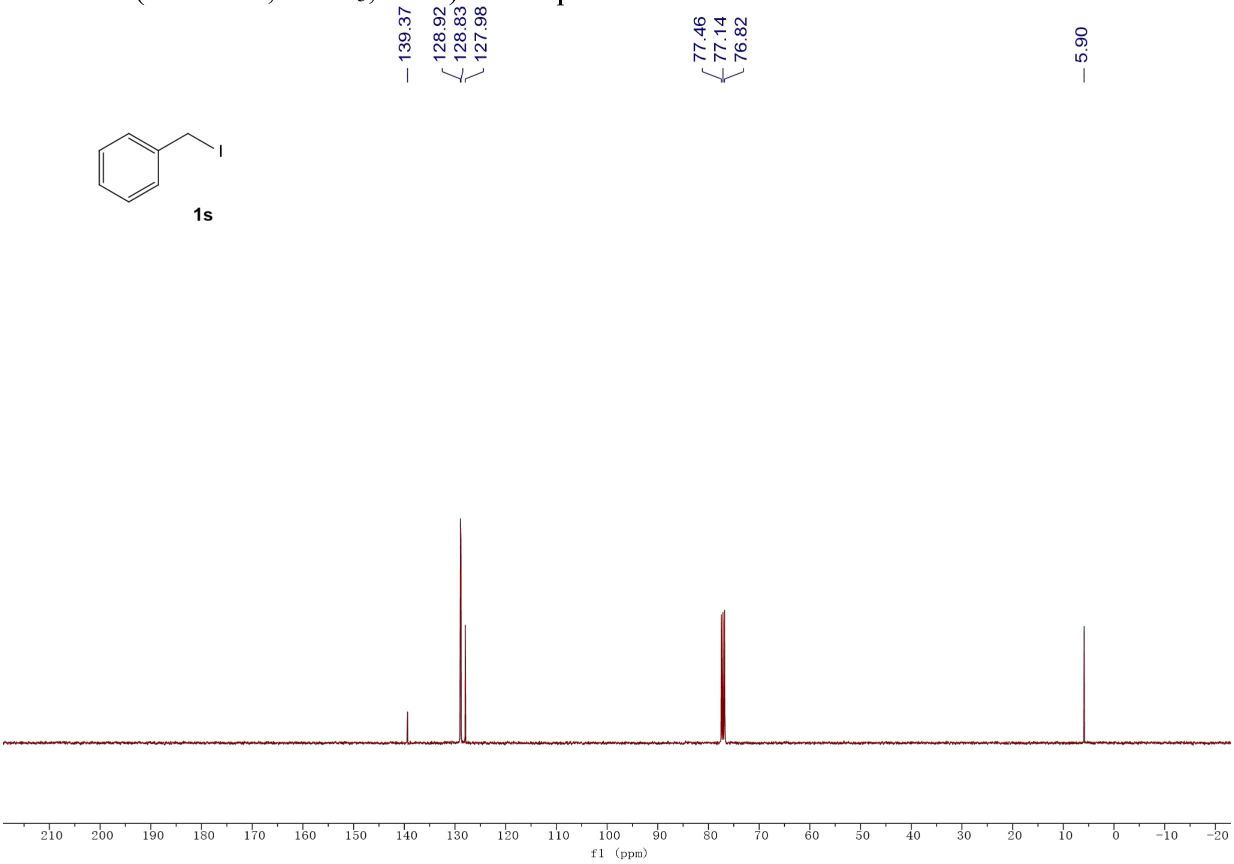

Supplementary Fig. 274. NMR of compound **1s**

^{1}H NMR (400 MHz, $CDCl_3$, 25°C) of compound **1t**

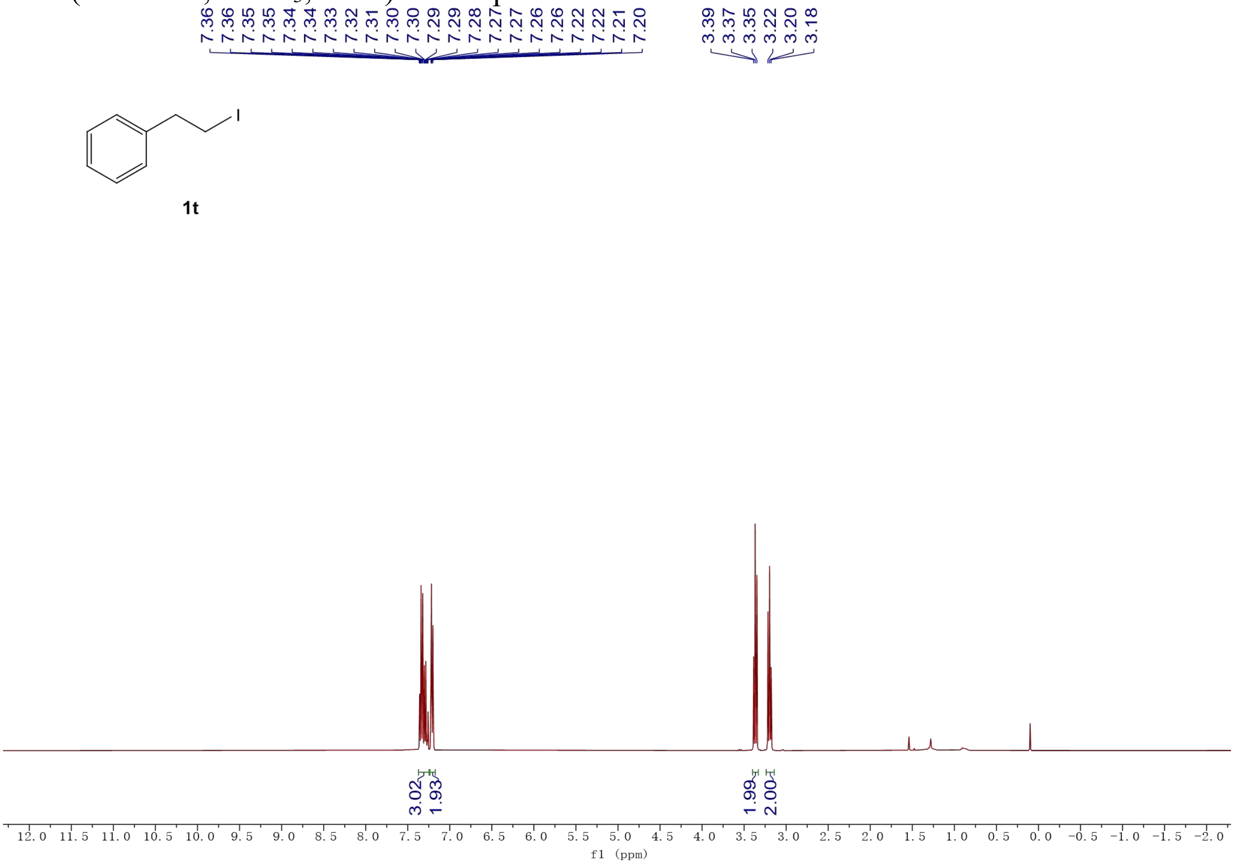

^{13}C NMR (101 MHz, $CDCl_3$, 25°C) of compound **1t**

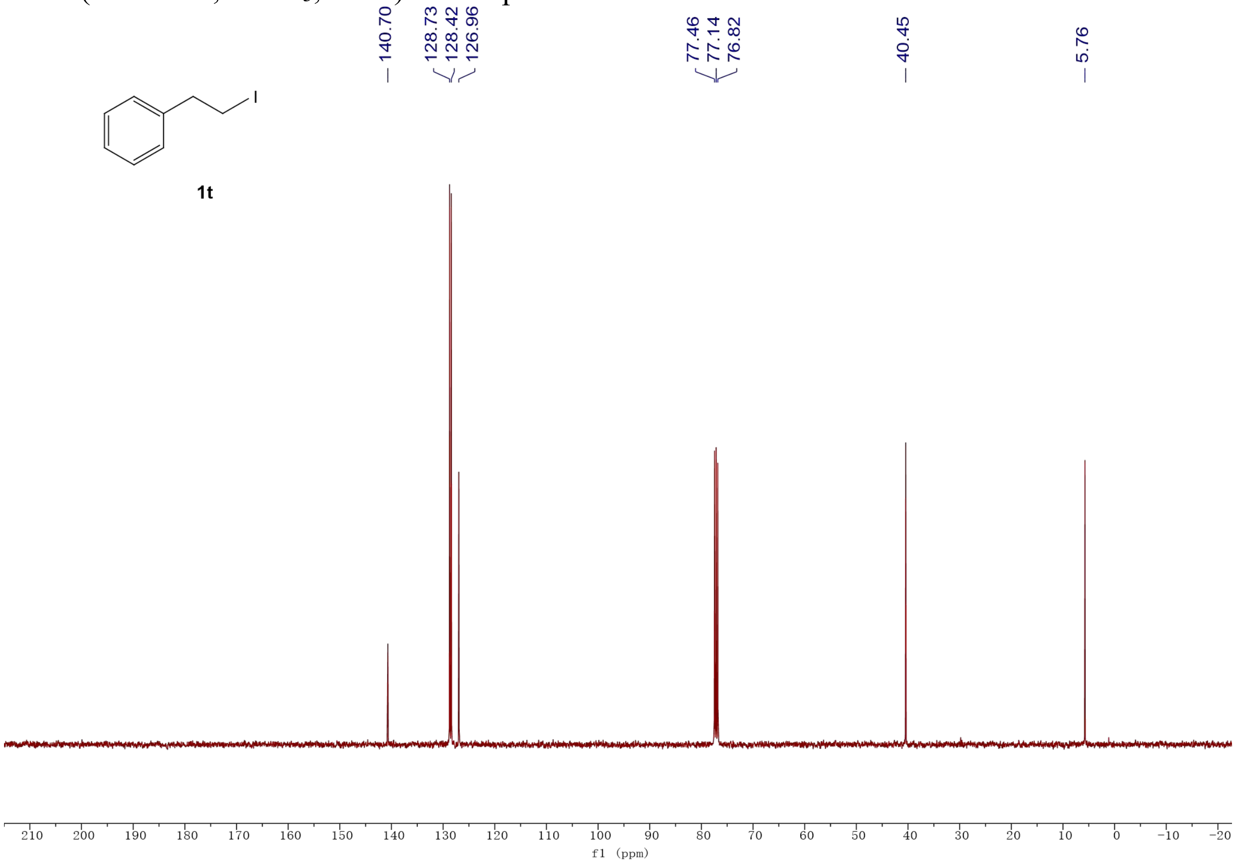

Supplementary Fig. 275. NMR of compound **1t**

^{1}H NMR (400 MHz, $CDCl_3$, 25°C) of compound **1u**

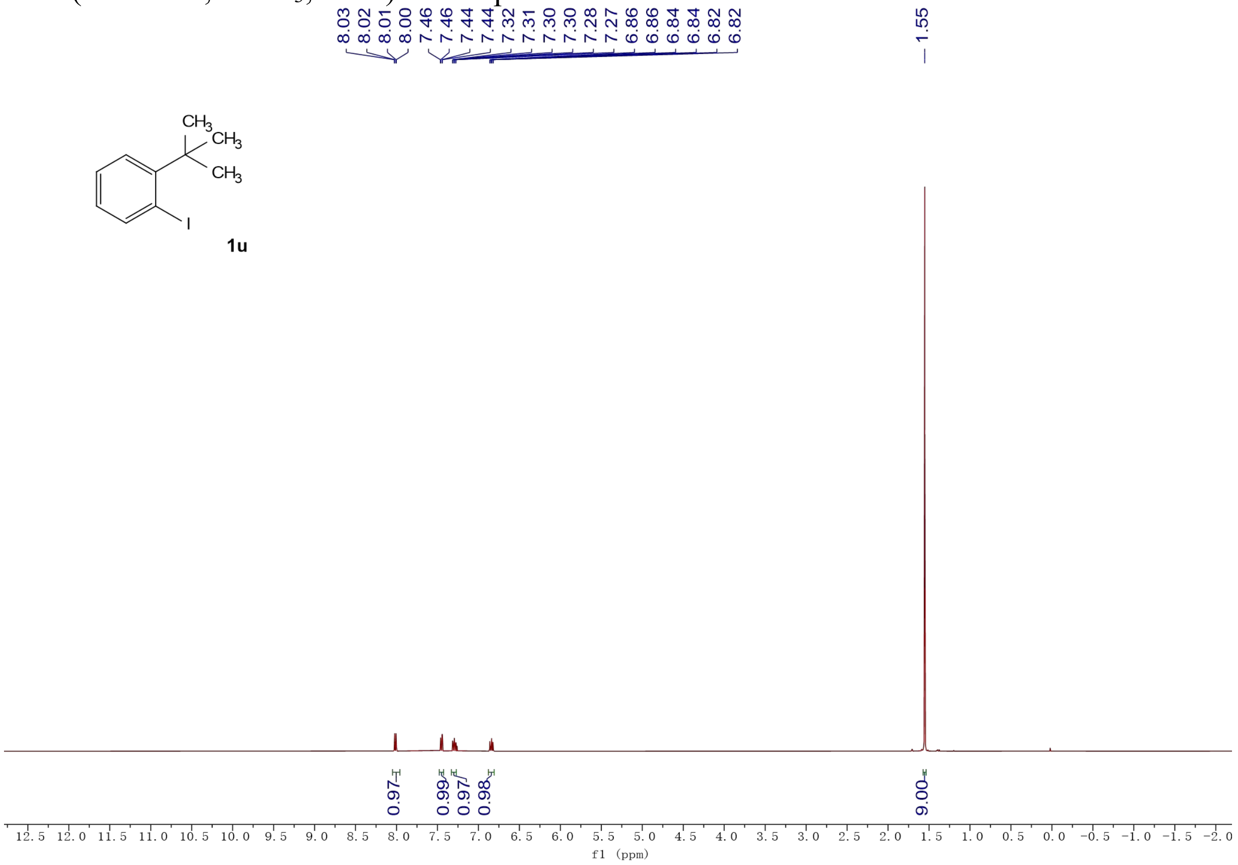

^{13}C NMR (101 MHz, $CDCl_3$, 25°C) of compound **1u**

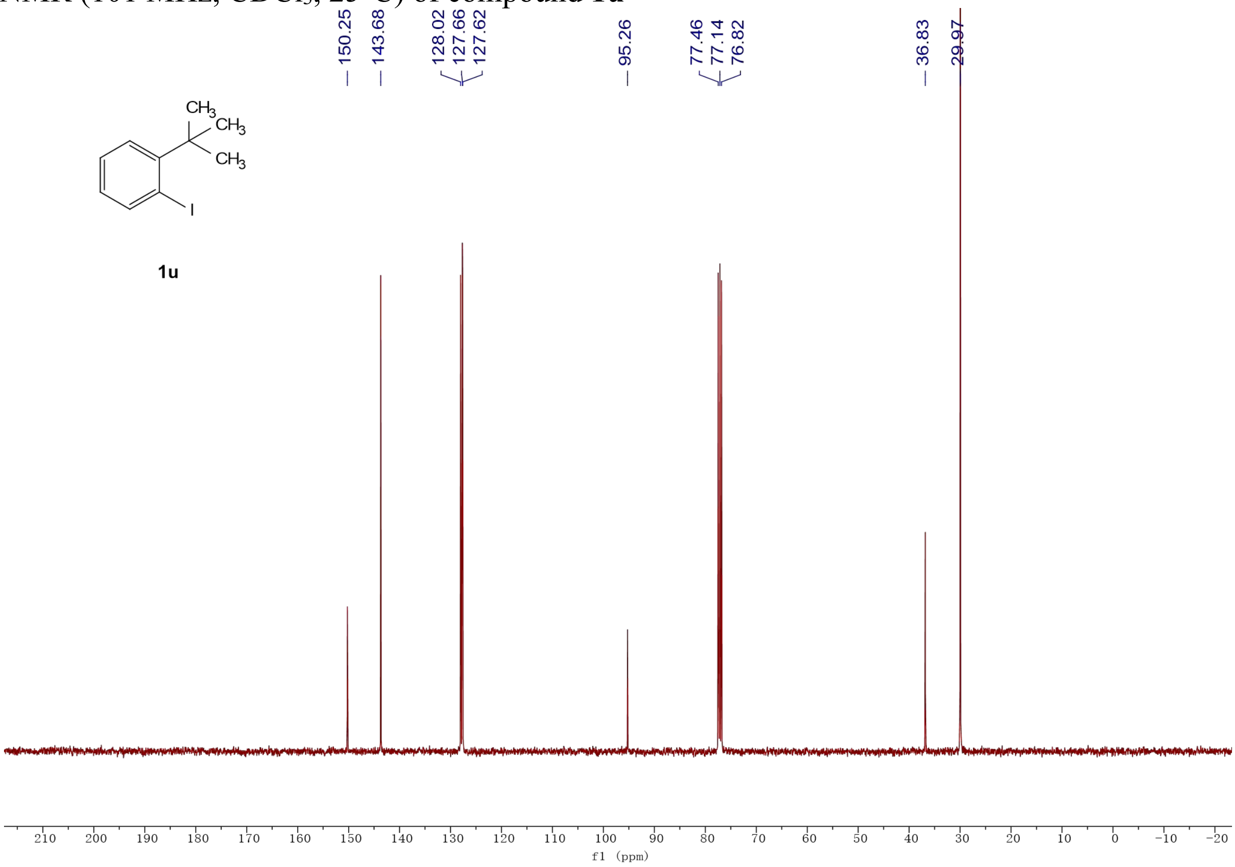

Supplementary Fig. 276. NMR of compound **1u**

^{1}H NMR (400 MHz, $CDCl_3$, 25°C) of compound **1v**

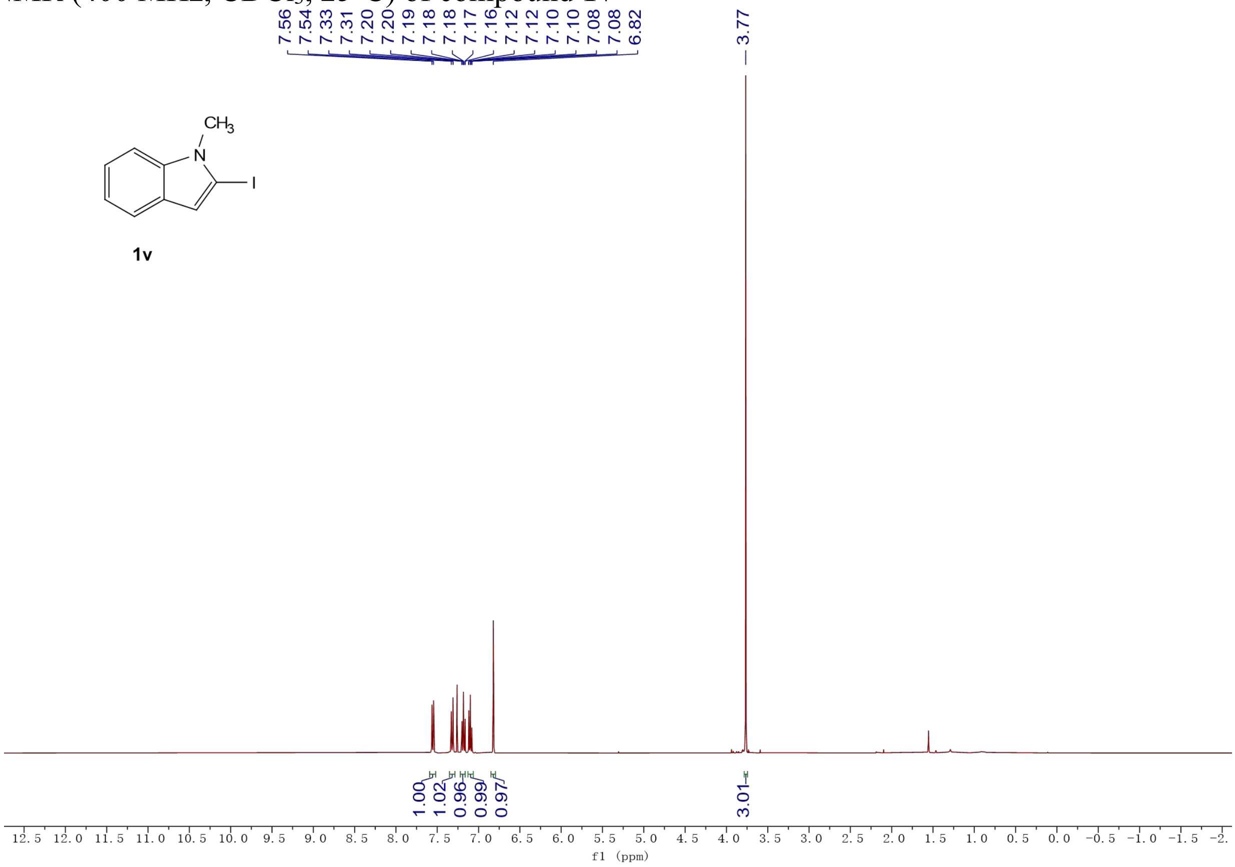

^{13}C NMR (101 MHz, $CDCl_3$, 25°C) of compound **1v**

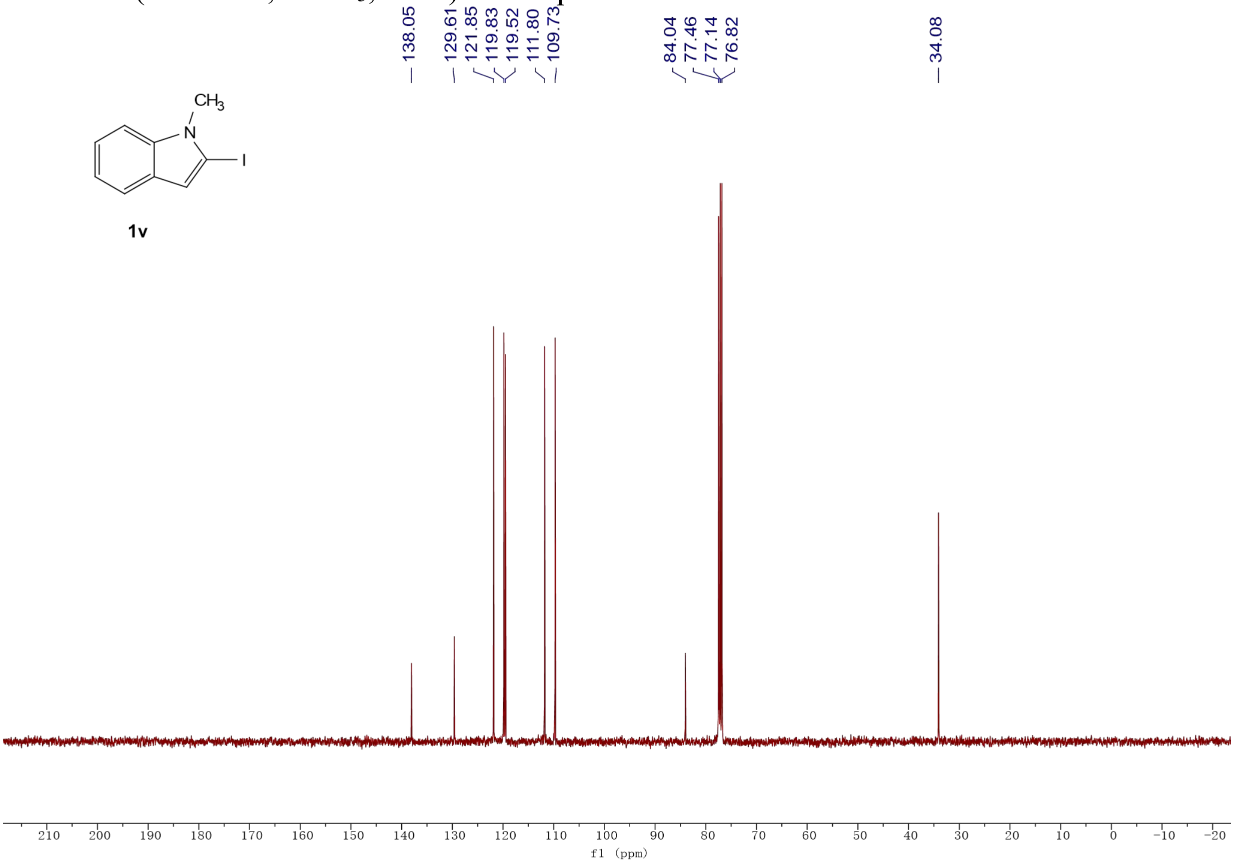

Supplementary Fig. 277. NMR of compound **1v**

^{1}H NMR (400 MHz, $CDCl_3$, 25°C) of compound **1w**

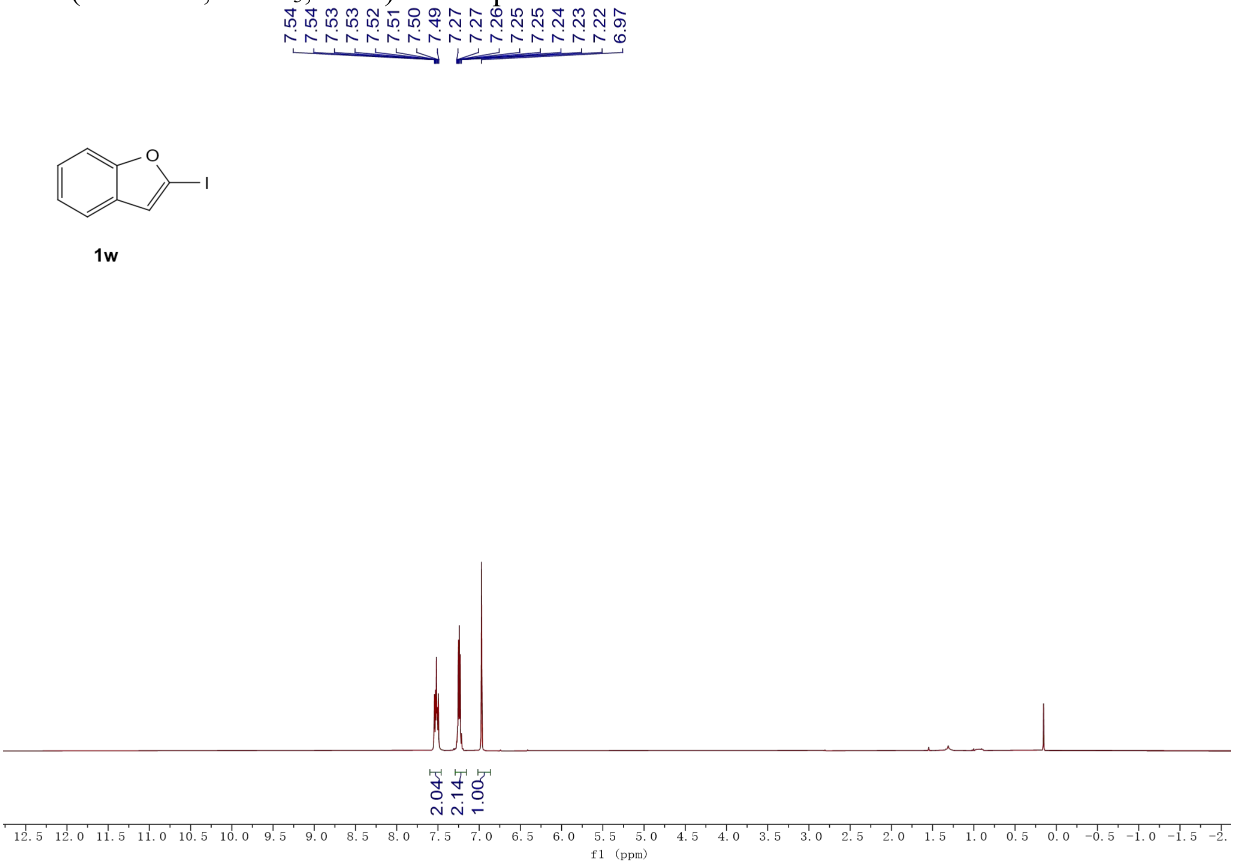

^{13}C NMR (101 MHz, $CDCl_3$, 25°C) of compound **1w**

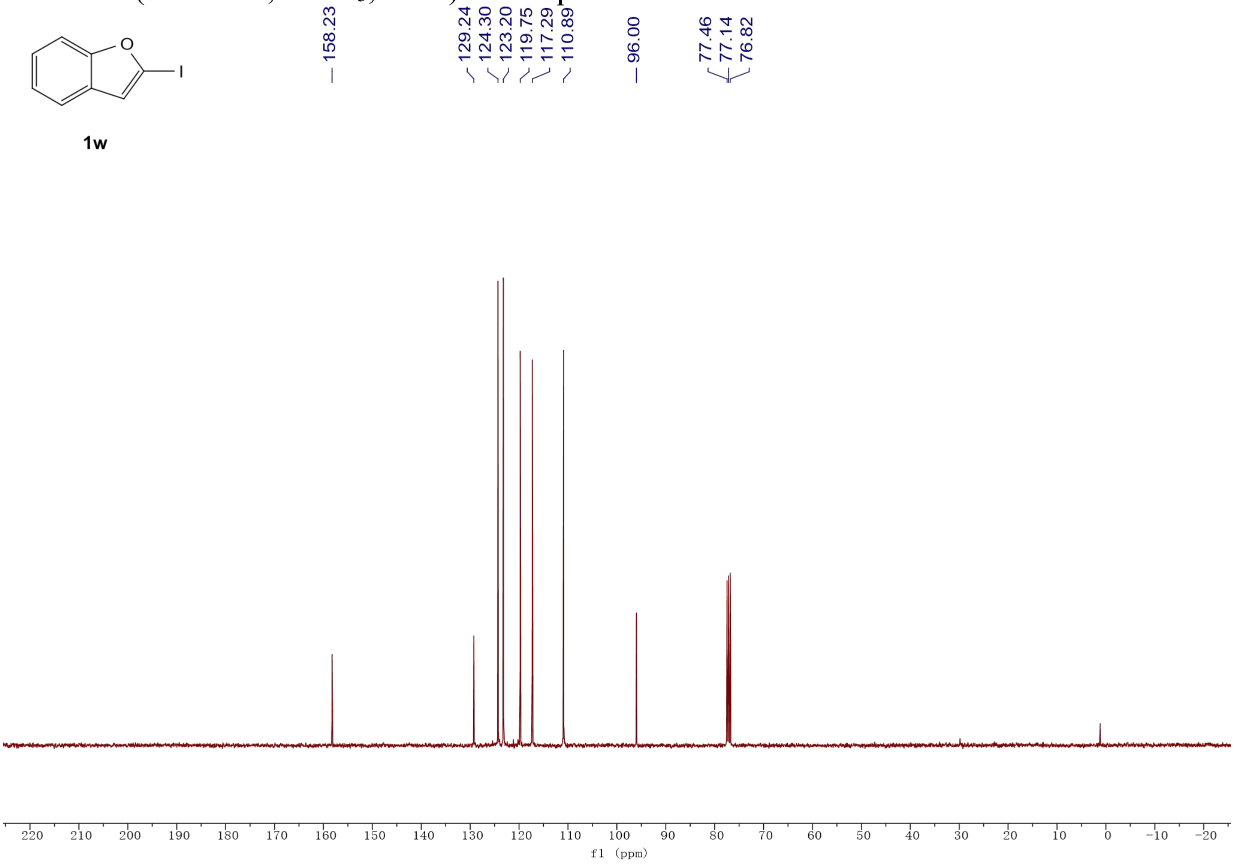

Supplementary Fig. 278. NMR of compound **1w**

^{1}H NMR (400 MHz, $CDCl_3$, 25°C) of compound **1x**

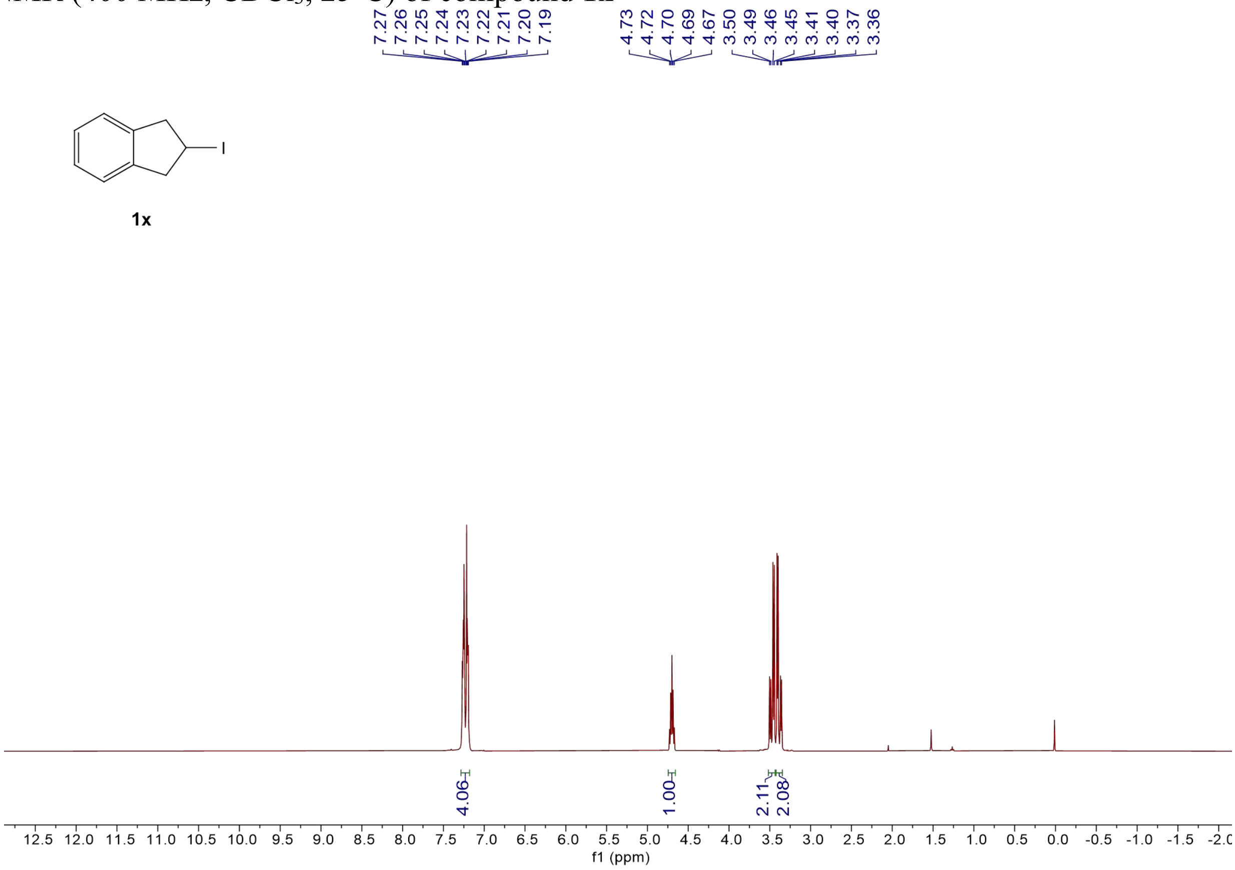

^{13}C NMR (101 MHz, $CDCl_3$, 25°C) of compound **1x**

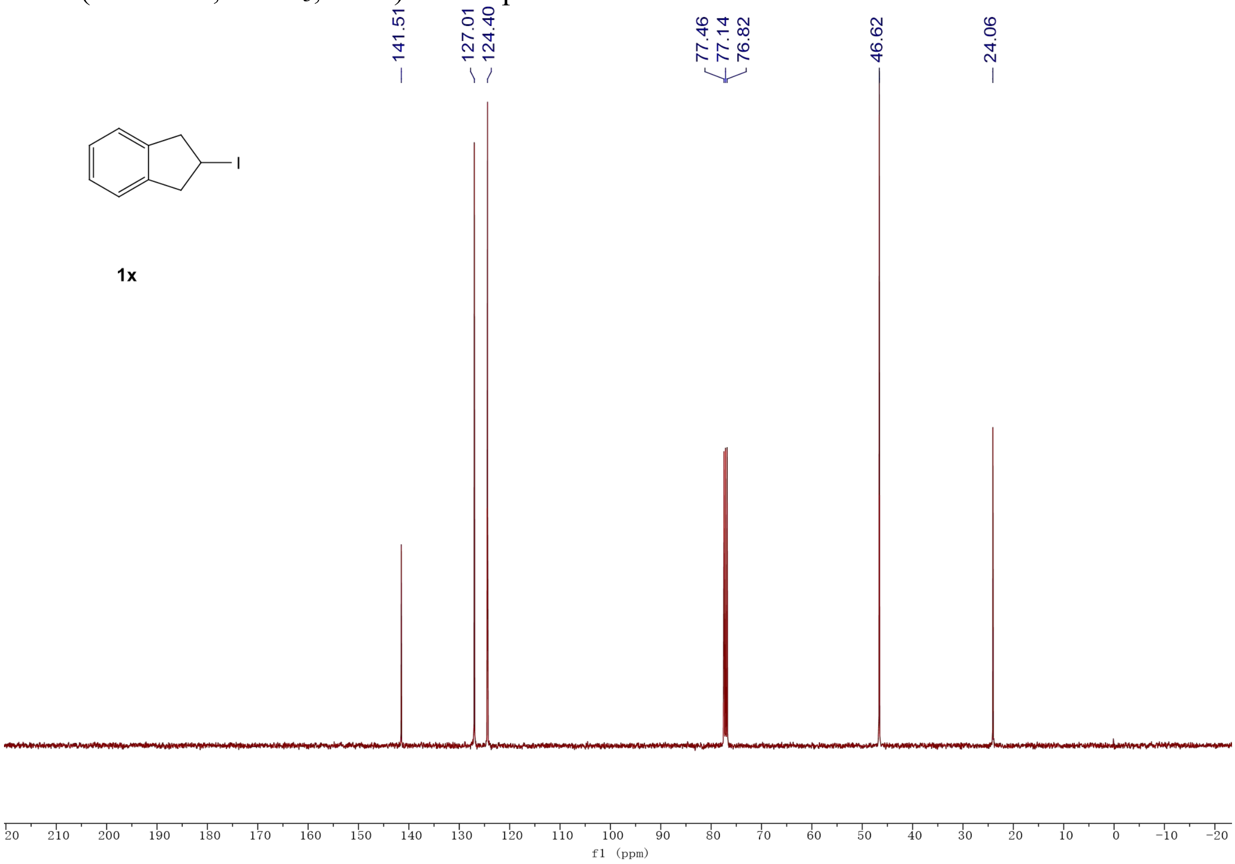

Supplementary Fig. 279. NMR of compound **1x**

^{1}H NMR (400 MHz, $CDCl_3$, 25°C) of compound **1y**

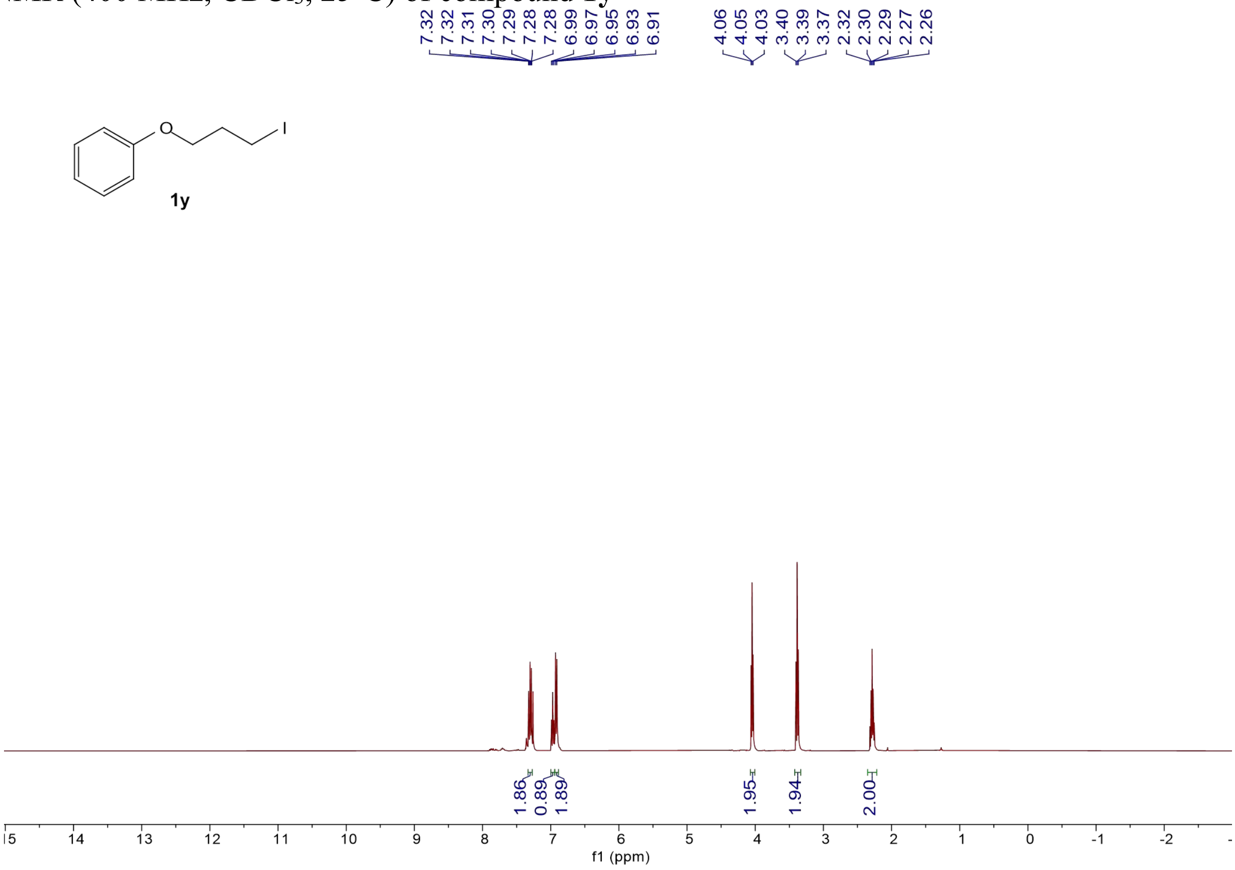

^{13}C NMR (101 MHz, $CDCl_3$, 25°C) of compound **1y**

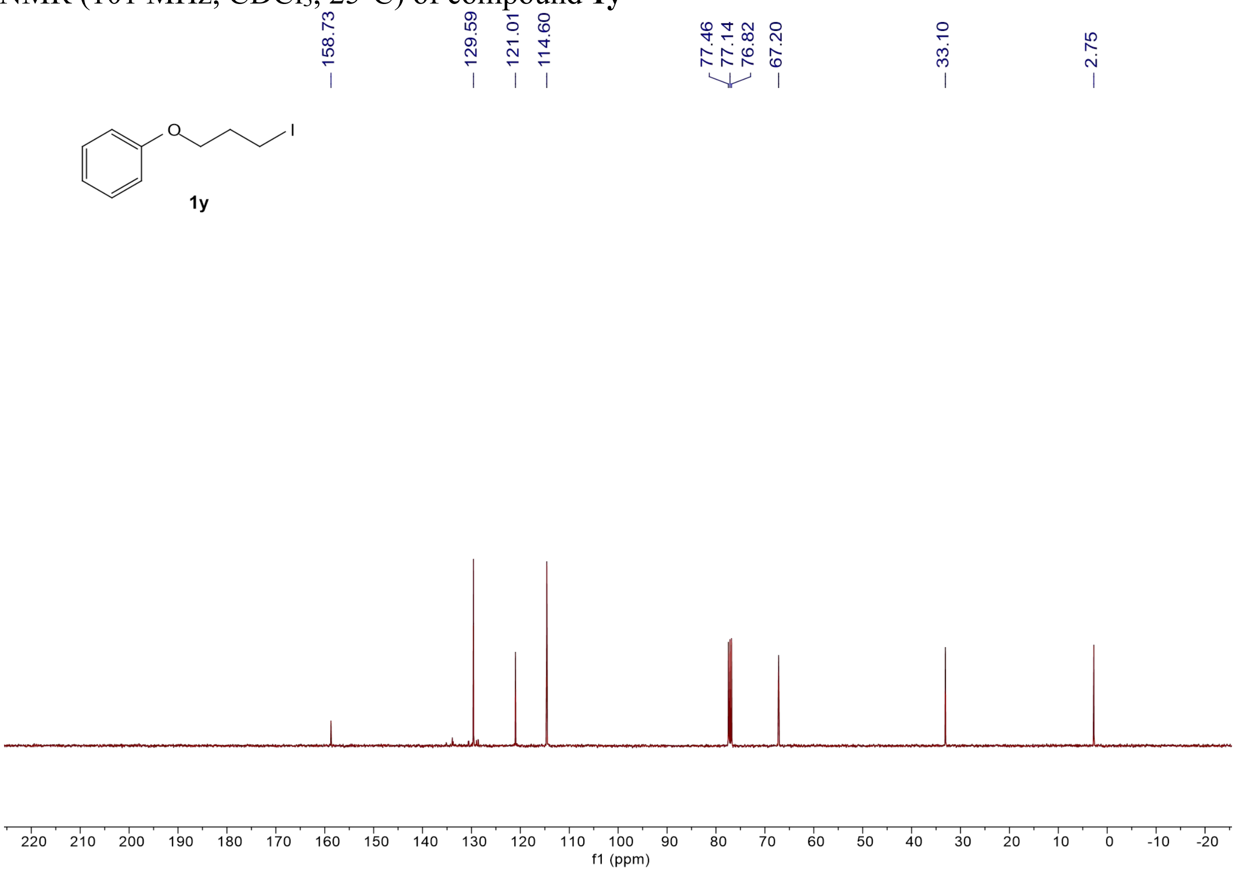

Supplementary Fig. 280. NMR of compound **1y**

References

1. Cai, L., Fu, L., Zhou, C.-L., Gao, Y.-Z., Li, S.-D. & Li, G. Rh(I)-catalyzed regioselective arylcarboxylation of acrylamides with arylboronic acids and CO_2. *Green Chem.* **22**, 7328-7332 (2020).
2. Das, D. H., Anal, J. M. & Rokhum, L. A mild and highly chemoselective iodination of alcohol using polymer supported DMAP. *J. Chem. Sci.* **128**, 1695-1701 (2016).
3. Edkins, R. M., Probert, M. R., Fucke, K., Robertson, C. M., Howard, J. A. K. & Beeby, A. The formation of peroxide degradation products of photochromic triphenylimidazolyl radical-dimers. *Phys. Chem. Chem. Phys.* **15**, 7848-7853 (2013).
4. Weingand, V., et al. Intermolecular desymmetrizing Gold-catalyzed yne-yne reaction of push-pull diarylalkynes. *Chem. -Eur. J.* **24**, 3725-3728 (2018).
5. Tong, Z.-X., Garry, O. L., Smith, P. J., Jiang, Y.-B., Mansfield, S. J. & Anderson, E. A., et al. Au(I)-catalyzed oxidative functionalization of yndiamides. *Org. Lett.* **23**, 4888-4892 (2021).
6. Wan, T., et al. Photoinduced halogen-atom transfer by *N*-Heterocyclic carbene-ligated boryl radicals for C(sp^3)-C(sp^3) bond formation. *J. Am. Chem. Soc.* **145**, 991-999 (2023).
7. Yoshimi, Y., et al. Electron transfer promoted photochemical reductive radical cyclization reactions of allyl 2-bromoaryl ethers. *Tetrahedron Lett.* **54**, 2419-2422 (2013).
8. Takahashi, R., et al. Mechanochemical synthesis of magnesium-based carbon nucleophiles in air and their use in organic synthesis. *Nat. Commun.* **12**, 6691 (2021).
9. Witte K, et al. Magnesium K-edge NEXAFS spectroscopy of chlorophyll a in solution. *J. Phys. Chem. B* . **120**, 11619-11627 (2016).
10. Cooney, R. R. & Urquhart, S. G. Chemical trends in the near-edge X-ray absorption fine structure of monosubstituted and para-bisubstituted benzenes. *J. Phys. Chem. B.* **108**, 18185-18191 (2004).
11. Derfus, A. M., Chan, W. C. W. & Bhatia, S. N. Probing the cytotoxicity of semiconductor quantum dots. *Nano Lett.* **4**, 11-18 (2004).
12. Li, Y.-Y., et al. Degradable selenium-containing polymers for low cytotoxic antibacterial materials. *ACS Macro Lett.* **11**, 1349-1354 (2022).
13. Deng, Y-Y., et al. Highly efficient synthesis of diselenides and ditellurides catalyzed by polyoxomolybdate-based copper. *New J. Chem.* **46**, 20078-20081 (2022).
14. Singh, D., Deobald, A. M., Camargo, L. R. S., Tabarelli, G., Rodrigues, O. E. D. & Braga, A.L. An efficient one-pot synthesis of symmetrical diselenides or ditellurides from halides with CuO nanopowder/Se^0 or Te^0/Base. *Org. Lett.* **12**, 3288-3291 (2010).
15. Node, M., Patra, P. K., Shanmugasundaram, K., Matoba, M., Nishide, K. & Kajimoto, T. Odorless diphenyl diselenide and disulfide: syntheses and applications. *Synthesis.* **2005**, 447-457 (2005).
16. Patil, D. V., Hong, Y. T., Kim, H. Y. & Oh, K. Visible-light-induced three-component selenofunctionalization of alkenes: an aerobic selenol oxidation approach. *Org. Lett.* **24**, 8465-8469 (2022).
17. Kommula, D., Li, Q., Ning, S.-Y., Liu, W.-J., Wang Q. & Zhao. Z. B. K. Iodine mediated synthesis of diaryl diselenides using SeO_2 as a selenium source. *Synthetic Commun.* **50**, 1026-1034 (2020).
18. Radzhabov, A. D., Soldatova, N. S., Ivanov, D. M., Yusubov, M. S., Kukushkin, V. Y. & Postnikov, P.S. Metal-free and atom-efficient protocol for diarylation of selenocyanate by diaryliodonium salts. *Org. Biomol. Chem.* **21**, 6743-6749 (2023).
19. Chen, C.-L., Li, J.-C., Liu, M.-C., Zhou, Y.-B. & Wu, H.-Y. Metal-free synthesis of diselenides and ditellurides by using TMSCN. *Tetrahedron Lett.* **113**, 154255 (2022).
20. Lin, B.-Z., et al. Enhancing the potential of miniature-scale DNA-compatible radical reactions via an electron donor–acceptor complex and a reversible adsorption to solid support strategy. *Org. Lett.* **23**, 7381-7385 (2021).
21. Zhu, F., O'Neill, S., Rodriguez, J. & Walczak, M. A. Stereoretentive reactions at the anomeric position: synthesis of selenoglycosides. *Angew. Chem. Int. Ed.* **57**, 7091-7095 (2018).
22. Sun, S., et al. Controllable synthesis of disulfides and thiosulfonates from sodium sulfinates mediated by hydroiodic acid using ethanol and H_2O as solvents. *Org. Biomol. Chem.* **20**, 8885-8892 (2022).
23. Das, A. & Thomas, K. R. J. Tuning selectivity in the visible-light-promoted coupling of thiols with alkenes by EDA vs TOCO complex formation. *ACS Omega*. **8**, 18275-18289 (2023).
24. Zheng, Y., Qing, F.-L., Huang, Y.-E. & Xu, X.-H. Tunable and practical synthesis of thiosulfonates and disulfides from sulfonyl chlorides in the presence of tetrabutylammonium iodide. *Adv. Synth. Catal.* **358**, 3477-3481 (2016).
25. Wang, G.-F., et al. Solid-state molecular oxygen activation using ball milling and a piezoelectric material for aerobic oxidation of thiols. *RSC Adv.* **12**, 18407-18411 (2022).
26. Abaee, M. S, Mojtahedi, M.M. & Navidipoor, S. Diethylamine-catalyzed dimerization of thiols: an inexpensive and green method for the synthesis of homodisulfides under aqueous conditions. *Synth Commun.* **41**, 170-176 (2010).

27. Zhu, C.-J., Wu, D.-F., Liu, H.-L., Meng, C.-W. & Tang, T.-D. Transformation of thiols to disulfides via an oxidant-free radical pathway on the zeolite ETS-10. *Green Chem.* **24**, 9033-9039 (2022).
28. Zha, J.-J., Wang, Z.-E., Liu, B.-X., Tan, Q.-T. & Xu, B. Multicomponent reaction of isocyanide, ditelluride, and Mn(III) carboxylate: synthesis of *N*-acyl tellurocarbamate. *Org. Lett.* **24**, 2863-2867 (2022).
29. Ren, X.-J., Liu, Q., Yang, Z.-S., Wang, Z.-X. & Chen, X.-Y. Aryldiazonium salts can serve as nitrogen-based lewis acid catalysts and their applications in the formation of photoactive charge transfer complexes. *Chin Chem Lett.* **34**, 107821 (2023).
30. Kassaee, M., Motamedi, E., Movassagh, B. & Poursadeghi, S. Iron-catalyzed formation of C-Se and C-Te bonds through cross coupling of aryl halides with Se(0) and Te(0)/nano-Fe_3O_4@GO. *Synthesis* **45**, 2337-2342 (2013).
31. Liu, Y.-W., et al. Thio-michael addition of α,β-unsaturated amides catalyzed by Nmm-based ionic liquids. *RSC Adv.* **7**, 43104-43113 (2017).
32. Duarte, L. F. B., et al. Organoselenium compounds from purines: synthesis of 6-arylselanylpurines with antioxidant and anticholinesterase activities and memory improvement effect. *Biorg. Med. Chem.* **25,** 6718-6723 (2017).
33. Wang, D.-Y., et al. Non-transition metal-mediated diverse aryl-heteroatom bond formation of arylammonium salts. *iScience.* **15,** 307-315 (2019).
34. Maity, P., Ahammed, S., Manna, R. N. & Ranu, B. C. Calcium mediated C-F bond substitution in fluoroarenes towards C-chalcogen bond formation. *Org. Chem. Front.* **4,** 69-76 (2017).
35. Husár, B., et al. Biomaterials based on low cytotoxic vinyl esters for bone replacement application. *J Polym Sci. A Polym Chem.* **49**, 4927-4934 (2011)
36. Chen, Q.-R., Wang, P.-P., Yan, T. & Cai, M.-Z. A highly efficient heterogeneous ruthenium(III)-catalyzed reaction of diaryl diselenides with alkyl halides leading to unsymmetrical diorganyl selenides. *J. Organomet. Chem.* **840**, 38-46 (2017).
37. Liu, X.-S., Li, M.-J., Dong, K.-X., Peng, S.-T. & Liu, L. Highly stereoselective synthesis of tetrasubstituted vinyl selenides via rhodium-catalyzed [1,4]-acyl migration of selenoesters and diazo compounds. *Org. Lett.* **24**, 2175-2180 (2022).
38. Murie, V. E., et al. Synthesis of 7-chloroquinoline derivatives using mixed lithium-magnesium reagents. *J. Org. Chem.* **86**, 13402-13419 (2021).
